# Development and Feasibility of an eHealth Diabetes Prevention Program Adapted for Older Adults—Results from a Randomized Control Pilot Study

**DOI:** 10.3390/nu16070930

**Published:** 2024-03-23

**Authors:** Suzannah Gerber, Rachel E. Silver, Sai Krupa Das, Savana S. Greene, Sadie R. Dix, Isabella Ramirez, Christina L. Morcos, Maria Carlota Dao, Lisa Ceglia, Susan B. Roberts

**Affiliations:** 1 Gerald J. and Dorothy R. Friedman School of Nutrition Science and Policy, Tufts University, 150 Harrison Avenue, Boston, MA 02111, USAsavana.greene@tufts.edu (S.S.G.); sadie.dix@tufts.edu (S.R.D.); christina.morcos@tufts.edu (C.L.M.); 2 Jean Mayer USDA Human Nutrition Research Center on Aging, Tufts University, 711 Washington Street, Boston, MA 02111, USA; 3 Department of Agriculture, Nutrition, and Food Systems, University of New Hampshire, Durham, NH 03824, USA; carlota.dao@unh.edu; 4 Division of Endocrinology, Diabetes and Metabolism, Tufts Medical Center, Boston, MA 02111, USA; 5 Geisel School of Medicine, Dartmouth College, 1 Rope Ferry Rd., Hanover, NH 03755, USA; susan.b.roberts@dartmouth.edu

**Keywords:** eHealth, lifestyle medicine, weight loss, older adults, remote monitoring, fitness, diet, intervention

## Abstract

Lifestyle programs that reduce health risks and support weight loss (WL) in older adults face adherence and attendance challenges due to reduced energy requirements, impaired mobility, lack of transportation, and low social support. Tailored lifestyle and weight management programs are needed to better support healthy aging for older adults. Here, we developed and piloted an age-adapted, remotely delivered modification of the Diabetes Prevention Program (DPP). The modification includes age-appropriate goals, visuals, and examples; flexible dietary composition; remote classroom and fitness-monitoring technology; and standardized online classroom materials employing pedagogical and behavior change theory. The modifications were designed to safeguard fidelity and to boost adherence, engagement, and knowledge integration, with the convenience of a fully remote WL program for diverse older adults. Six-month pilot data are presented from older adults (55–85 years, body mass index (BMI) 27–39.9 kg/m^2^, N = 20) randomly allocated to an online DPP intervention with weight, diet, and activity monitored remotely, or into a waitlisted control. The intervention achieved 100% attendance and adherence to self-monitoring. The intervention group mean (±SD) body weight change was −9.5% (±4.1); 90% lost ≥ 5%. By contrast, the control group gained 2.4% (±1.8). Once thought incompatible with older adults, remote interventions are feasible for older adults and can support fidelity, adherence, engagement, and clinically significant WL. Standardized materials are provided for future implementation.

## 1. Introduction

Forty percent of older adults in the United States have obesity, 49% have pre-diabetes, and 29% have type 2 diabetes [1,2,3]. The Diabetes Prevention Program (DPP) is a nationally recognized lifestyle intervention program designed to prevent diabetes and reduce risks for other cardiometabolic health outcomes with healthy lifestyle components including weight loss (WL), healthy diet, and exercise [4]. Higher attendance and regular participation in program sessions are associated with clinically impactful outcomes [5], but population compatibility, penetration, and program fidelity are known obstacles [6]. As a result, many interventions fail to attain clinically impactful weight loss (≥5%) or to support weight loss maintenance for six months or longer [7,8,9]. Thus, an adaptation with standardized presentation materials optimized for online delivery and targeted to the specific needs of older adults could potentially increase compatibility, convenience, and fidelity, resulting in greater reach, efficacy, and accessibility.

One strategy to increase the accessibility of programs is to deliver them remotely, or in hybrid formats. Remote DPP programs have been explored for over ten years with mixed results [10,11,12,13]. Systematic reviews of online DPP programs predominantly study college-educated, white adults, aged 50–60, and report a mean WL of only 3–5% of starting weight [9,14]. However, there is considerable heterogeneity in the delivery of online DPP programs; hybrid programs that offer variable in-person and/or partially remote delivery report an overall lower WL [9]. Nonetheless, in randomized controlled trials that compare online to in-person interventions, the online groups had higher participant retention and engagement rates [9,10,14]. Similarly, app-based adaptations and/or those utilizing smart fitness devices for remote monitoring reported the greatest WL and the highest improvements in anthropometrics and other metabolic biomarkers [14,15]. Irrespective of delivery, programs with higher levels of interventionist support reported the greatest success with WL outcomes [9]. Thus, well-crafted, remotely delivered programs with strong interventionist support may be able to join accessibility and convenience with the education and social support that these programs offer.

Advances in remote fitness technology including wearable fitness trackers, mobile apps, e-dashboards, network-enabled scales, and social fitness platforms have helped to maximize the success of remotely delivered programs [16,17,18]. These tools allow for robust and real-time self-monitoring, interventionist surveillance, group social interaction, interventionist support, increased goal salience, program adherence, and dynamic opportunities for self-improvement [19,20,21]. Remote classrooms have also experienced a growth in supportive technology including things like live whiteboards, interactive polls, and other virtual activities, as well as methods such as flipped classrooms, in-class social time, and collaborative instruction [22,23,24]. These advances track alongside contemporary remote classroom pedagogy which recognizes and emphasizes the need to foster increased cognitive presence and engagement, which can be mutually reinforced by these tools and techniques [22,23,24].

Pedagogical and behavior change theories are known to positively influence outcomes in educational intervention programs like the DPP [5,9,25]. The Community of Inquiry (COI) framework focuses on supporting cognitive, social, and teaching presence to drive engagement in online and remote education [22,23,24,26,27]. COI incorporates the four-part pedagogical structure of the Practical Inquiry Model (PIM): first, an evocative topic or problem is presented (triggering event); next, students iteratively explore the topic through reflection, collaboration, and discussion; this is followed by integration exercises like student-led instruction; and finally, it is finished with resolution tasks where the new knowledge is applied outside of the classroom [27,28,29]. In and outside of classroom settings, change-readiness models like the Transtheoretical Model (TTM) and collaborative motivation- and commitment-driving techniques like Motivational Interviewing (MI) are well-received by intervention participants and provide tools for interventionists to support progress [30,31,32,33,34,35,36,37,38]. Previously, these methods have been observed to increase the application of program skills outside of sessions [31], to increase healthy diet and physical activity in patients with pre-diabetes and type 2 diabetes [32], and to be particularly effective in remote classroom interventions [35,36]. Together, theory-informed methods and modern fitness and remote classroom technology join robust self-monitoring, interventionist surveillance, increased accessibility, convenience, and socialization opportunities with established behavior science to foster goal salience, program adherence, fidelity, and engagement [18,19,20,21].

The need to tailor interventions for at-risk populations to improve program reach and effectiveness is well established [39,40]. In prior studies, older adults requested modifications relevant to compatibility and functionality such as different approaches to physical exercise due to limited mobility, flexible scheduling due to transportation, and flexible dietary approaches due to a lack of social support [31,41]. Despite the known incompatibilities, no known published adaptations address these issues. While tailoring an online program to older adults could address known barriers to in-person attendance (e.g., mobility and transportation, as well as infection-avoidance), other modifications would be needed to address barriers to program adherence including nutrition literacy, self-efficacy, age-related exercise limitations, reduced social support and normativity of healthy diets, and to provide technology support for older adults [42,43]. Programs that rely on advanced technology have historically reported low compatibility with older adults, which largely limited their use [11]. However, internet use is now nearly ubiquitous—reported by over 95% of American adults, with nearly 90% reporting internet use ≥1 day [44]. Overall, technology fluency and adoption have increased among older adults with telehealth use becoming pervasive during the COVID-19 pandemic [11,16,17]. Nonetheless, remote DPP interventions for older adults are rare, but a recent pilot showed some success [41]. To our knowledge, previously published remote interventions for older adults pre-date the notable shift in eHealth and technology fluency among older adults that occurred during the COVID-19 pandemic [11,16,17]. Furthermore, there are no known adaptations that specifically seek to optimize the success of exclusively online programs for older adults while addressing the previously identified compatibility issues.

Remote programs could increase program accessibility for older adults, and paired with further modifications could help optimize acceptability, adherence, and engagement [18,19,20,21] and standardized presentation materials can help to safeguard program fidelity to support the scalability of implementation. Additionally, remote programs also offer greater ease of administration, data collection, and scheduling flexibility, along with a reduced need for physical meeting spaces which can reduce program costs and interventionist burden [21]. Overall, we identified fidelity, accessibility, convenience, engagement, support, and technology compatibility as elements that reduced adherence and overall efficacy in previous programs. To address these, we developed a theory-informed novel modification to the DPP to support a replicable eHealth program with standardized materials, tailored for WL success in diverse older adults. This report includes justifications for the modifications made, results of the pilot, and includes all presentation materials.

## 2. Materials and Methods

The Brain and Body Health Study (BB-Health) aims to look at how food and lifestyle influence multiple cardiometabolic outcomes. This report focuses on the implementation process of a novel modification to the Diabetes Prevention Program Group Lifestyle Balance (DPP-GLB) [4,45] and the corresponding weight loss outcomes. The modification described below was designed to facilitate standardized delivery, engagement, and high adherence to a remote lifestyle intervention for older adults. Here, we report the results from the six-month pilot randomized control feasibility trial (Clinical Trial Registration #NCT05542199). The Tufts Health Sciences Institutional Review Board approved this study. Participants were recruited between September 2022 and March 2023 and given a stipend for participation. Baseline population characteristics are shown in Table 1.

### 2.1. Adaptations to the DPP-GLB

The original DPP-GLB is designed to be delivered in person and centers around a written curriculum led by an interventionist during group sessions. Participants receive educational handouts in each session and the interventionist instructs based on a session-specific lesson plan which is intended to be secondary to group discussions [4,45]. In this modification, the core curriculum of the DPP-GLB was maintained including the use of participant handouts, goal targets such as 7% body weight reduction (accomplished through reduced energy intake and increased physical activity), group-meeting structure, and the temporal sequence of sessions (weekly: sessions 1–12; bi-weekly: sessions 13–16; and monthly through 22). Consistent with the DPP-GLB, participants were given a calorie counter, worksheets, and measuring cups. However, in this adaptation, lesson plans were restructured to include theory-informed prompts, individualized feedback, and formatting revisions to create dynamic standardized, online classroom presentations alongside flipped classroom lessons that adhere to the structured curriculum. Additionally, food and activity logs were primarily accomplished using the fitness device and corresponding application and dashboard, without relying on paper logs. 

Seven key areas (Table 2) of the program were adapted in this modification to facilitate remote delivery and to increase acceptability and adherence to an entirely remote intervention among older adults. Changes comparing this modification to the DPP-GLB are summarized in Table 2. All program presentation materials, including classroom polls and activities, were embedded into standardized presentation slides used in a videoconference-based, synchronous, remote classroom environment, to support fidelity across groups and for future implementations (Section S2).

#### 2.1.1. Supplemental Pedagogical and Behavior Change Theories for the Modification

We aimed to increase motivational salience and engagement using theory-informed approaches known to foster improved results. Session presentations included polling questions informed by Transtheoretical Model (TTM) constructs of Stage of Change (SOC) and Process of Change (POC) [46,47] modified from Gonzalez-Ramirez et al. [48] that recurred as intervention checkpoints to increase participant self-awareness and to enable stage-matched support and Motivational Interviewing (MI)-informed feedback, similar to the approaches described by Miller et al. [33], Van Der Veen et al. [49], Johnson et al. [50], and Prochaska et al. [51,52].

To foster group discussion and the internalization of key concepts, program topics, and the related behavior change goals were delivered as open-ended questions, polls, and discussion prompts, as well as communicated through participant-led and/or flipped classroom activities which have been previously observed to increase cognitive and social presence in remote classrooms. Methods for structuring questions and curricula for the remote classroom environment followed the Community of Inquiry (COI) framework [24]. Following the COI, a topic triggers a set of exploratory probing exercises, followed by group integration activities, and opportunities for applied and confirmatory resolution where participants vocalize subjective, first-person goals and intentions, consistent with MI concepts of “change talk” and “sustain talk”. The MI-informed evocation of motivational salience and goal planning guided the phrasing and frequency of discussion prompts and polls [33]. These prompts and polls offered participants various and recurring ways to examine and discuss progress, focus on obstacles that present challenges, and plan out intentions while engaging with peer and interventionist feedback employing the four processes of MI—experience validation, reframing, summarizing, and deployment of personal insights [30,33]—similar to methods described by Dineen et al. [31], in alignment with the logical framework presented in Hawkes et al. [25]

The flipped classroom structure was supported by emails with individualized feedback which were sent to each participant and included stage-matched and MI-informed affirmations of progress, identifications of recent behavioral choices that did/did not support success, and summaries of previously stated goals. These reports also included cues to review upcoming session materials and identified areas of specific success or challenges for them to present to the group in the upcoming session. Thus, sessions were able to foster large amounts of participant-led discussion time while addressing all major curriculum elements; sessions were a mixture of components delivered by the interventionist and components told through reflection and insights from the personal experiences of peer-participants. To ensure even participation and progression through curriculum goals within each session, every participant was called on directly to respond to each prompt and poll, and to share such reflections.

#### 2.1.2. Adaptation of Program Goals and Exercise, Diet, and Weight Recommendations for Older Adults

Daily activity and calorie targets, and successive weight loss goals, were re-evaluated throughout the program [53,54]. In contrast to the original DPP-GLB which sets uniform minimum activity targets and weight-category calorie limits irrespective of activity level or weight loss attainment, this adaptation set physical activity targets first at 5000 steps/day, which would then increase to 6000–10,000 steps per day depending on individual goals and ability. Moderate–Vigorous Physical Activity (MVPA) targets begin at 100 min/week and would then rise to 150–210+ min/week, similarly, depending on goals. Exercise recommendations focused on walking/jogging at various intensity levels as the main form of exercise, and although safe exercising practices from the original DPP-GLB were included (e.g., warm-ups/cool-downs and stretching), instruction to perform more strenuous physical activity was removed. As a result, session 13, which is centered around vigorous resistance exercise in the DPP-GLB, was reconfigured as a mid-program review, allowing concepts presented early in the program to be foregrounded, and providing practice and conceptual mastery opportunities for group members (Section S2). These changes addressed feedback from older adult participants discussed in Beasley et al. and Lee et al. [35,36,41] and also aimed to reduce the risk of injury in this vulnerable population during remote-only delivery. Although walking/jogging was the form of exercise directly recommended, participants were encouraged to continue or to undertake other exercise if deemed suitable by their doctor.

Concerning diet, calorie targets were initially determined by using the participant’s starting weight, as it is in the DPP-GLB. However, this modification employed energy intake ranges with the DPP-GLB target set as the minimum and the next higher level set as the maximum. Ranges were adjusted monthly based on weight change (e.g., reduced to support continued weight loss) and in response to activity levels (e.g., lower if less active, higher if very active). Dietary composition recommendations were modified from the low-fat and low-energy density focus of the DPP-GLB to emphasize flexibility in dietary patterns recognizing the well-known reduced willingness, physical, and financial ability to change dietary patterns among many older adults [43]. Participants were introduced to the healthy Mediterranean [55], DASH [56], MIND [57], and healthy plant-based [58] diet plans in the first session and were encouraged to follow preferred plans while strongly emphasizing three main targets: (1) dietary fiber > 25–35 g/day, by adding a daily serving of legumes, increasing whole fruits and vegetables, and eating most/all grains as whole grains [59]; (2) reducing saturated fat by emphasizing plant-based protein foods and smaller portions of lean animal-sourced foods, and limiting dairy to low-fat and/or fortified dairy-alternatives [60]; and (3) maintaining protein intake >50 g/day [61]. Reducing total dietary fat is a cornerstone of the diet recommendations in the original DPP-GLB, but in this modification, this was presented as one of several strategies for reducing total energy intake and as a strategy to prevent underestimating calories in food logs.

As in the DPP-GLB, the weight loss (WL) goal was 7% from baseline; however, in this modification, when this initial WL goal was achieved, participants were encouraged to self-determine either continued WL or to shift towards weight maintenance. Subsequent WL goals were set as an additional 5–7% of the new body weight, or less if a WL ≤ 5% would result in a BMI between 18.5 and 24.9 kg/m^2^.

**Figure 1:**
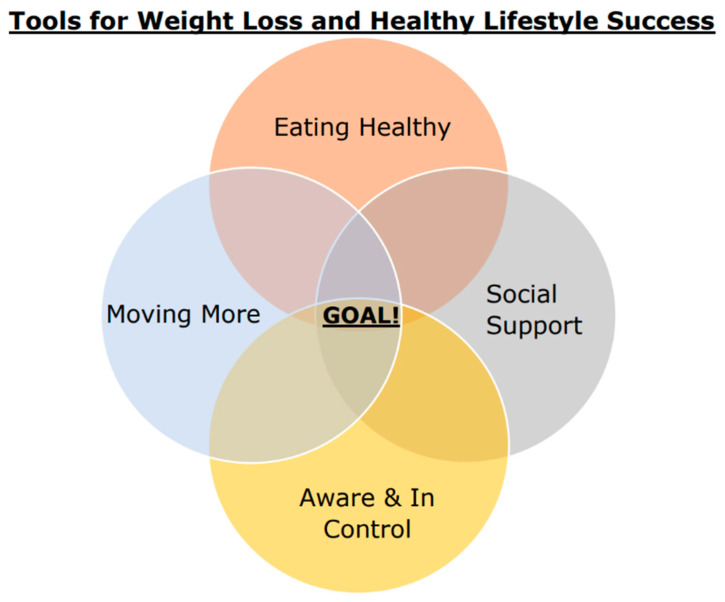
Main strategies of the adaptation, as presented to participants.

The practice of self-monitoring was a cornerstone of the intervention and factored heavily into the adaptation, supported by robust smart fitness tracking devices. Participants were encouraged to remain aware of their weight, intentional and spontaneous activity, foods consumed, sleep quality, water intake, sedentary time, and positive and negative social and environmental cues with daily logging and/or by reviewing values captured by their fitness devices. The concept of self-awareness (referred to as “Aware”; Figure 1) was repeated throughout the intervention, as a way to combat scale weight avoidance and to help develop self-monitoring behavioral routines. Awareness was also described as an essential component of self-control (“In Control”; Figure 1) which was a recurring empowerment cue to action contextualized as the components of personal health response to modifiable lifestyle choices. In other words, participants were encouraged to remain aware of their weight daily, even if the value was disappointing, in order to take immediate action and thus remain in control of their outcomes, and thereby remain in control of their overall health. Empowerment strategies have been identified as a critical component of success for health behavior change interventions that support healthy aging for older adults [62,63]. This adaptation presented the concepts of “Aware and In Control” for the above healthy lifestyle domains as equivalent in importance to eating healthy, moving more, and providing and receiving social support (Figure 1; see also Session 13, Section S2).

#### 2.1.3. Changes to Implementation and Self-Monitoring That Support Program Fidelity and Remote Delivery

The online group sessions were videoconference-based (Zoom, San Jose, CA, USA) including any make-up sessions. No sessions were offered in person, and the delivery was not hybrid, but exclusively remote. A key advantage to the remote format is the ability for participants to join from anywhere, the ability to accommodate rescheduled and make-up sessions, and/or the ability to allow group members to join other groups to make up a session they missed [13].

To support the remote monitoring of physical activity, weight, and dietary intake, participants were given a wearable fitness tracker (Fitbit Inspire 2; Fitbit, San Francisco, CA, USA) and a Wi-Fi-enabled scale (Fitbit Aria Air), which were networked with the corresponding web-based dashboard and smartphone application (Fitbit, 2022–2023). The dashboard enabled both self and interventionist monitoring, in addition to logging, automatic heart rate and activity intensity measurement, behavioral cues and reminders, messaging, and other supportive resources for adherence. Participants were asked to use these every day, to weigh daily upon waking after voiding in the morning, and to log all daily foods.

Standardized presentation materials were developed to present curriculum materials during group sessions and to ensure program fidelity (PowerPoint, Microsoft, Redmond, WA, USA). Each session was led by an interventionist using these materials which feature interactive elements to support engagement, rather than a passive lecture format or an unstructured group discussion. The same materials are utilized across groups ensuring no material is omitted and that different groups receive the same information [6,18,64]. Presentations include the use of modern videoconference tools such as live whiteboards and interactive polls, and also provide structured discussion prompts, solicit flipped classrooms with participant-led lessons and personal testimonies, and also deploy theory-informed checkpoints to monitor progress [13,18,65] (Section S2). Each session was structured using the COI framework with a critical topic for health, followed by interactive exploration that centered around personal examples from each of the group members, integration opportunities where participants offered advice to each other based on feedback or their own experiences, and planning to apply the knowledge gained in the time in between sessions structured using Motivational Interviewing techniques to elicit change and sustain talk. This resolution-driving exercise was presented at the end of each session and again at the beginning of the subsequent session for reinforcement using callbacks and personalized examples of how the knowledge was applied outside of the session. This callback and examination of resolution exercises provided both an opportunity for commitment and goal salience and an opportunity for peers to witness healthy social norms developing within the group. Group-specific examples were gathered during sessions and able to be used as personalized examples in future discussions to enable group cohesion and cognitive presence with the material [39]. Standardized presentation materials also included rich visuals, such as images depicting diverse older adults.

#### 2.1.4. Participant and Interventionist Interactions and Communication in the Adaptation

Social support has been identified as an important lever of successful health behavior change [47,66,67]. Social isolation and social-norm-based change-aversion have been especially indicated as significant barriers to healthy lifestyle change among older adults [42,62,68]. To address this, every session began with socialization and reconnection time. These periods afforded participants time to get to know one another, to celebrate life events, and to strengthen social bonds between each other and with the interventionist. This socialization time segued into the resolution discussion of successes and challenges during the period between sessions and allowed for organic social support and problem-solving before the structured educational session began.

As described above, detailed progress reports were privately sent to each participant prior to each session. Reports included affirmations, recommendations about how to improve outcomes, and feedback based on remotely collected data. Reports typically included a reflection on recent progress towards goal attainment, device management assistance, reminders relevant to that participant’s action plan, an honest assessment of observed adherence, and prompting in preparation for discussions relevant to the upcoming session for the flipped classroom time. Reports also offered an open line of communication for participants enabling them to request additional support and to respond and ask questions privately, if desired.

Participants were linked to each other and to the interventionist through the online fitness dashboard and the corresponding app, synchronized with the fitness devices. The dashboard and app visibly share goals and activity levels with other group members, allowing for social comparison among participants; it also provides the interventionist with a venue for role-modeling healthy behaviors. The dashboard and app also had an internal messaging system that supported affirmations, questions, and direct peer-to-peer support outside of scheduled sessions. Additionally, the dashboard itself sent automatic, programmatic nudges that increase the salience of these social comparison pieces (e.g., step-count rank compared to the group; awards for top placement), and within-person progress compared to the prior week (e.g., accolades for increasing steps, reducing weight, or reaching logging or performance targets) [21,66].

Consistent with MI and prior MI-based DPP modifications [30,31,33,69], we utilized routine affirmations of participant experiences and choices during discussions, in response to prompts and polls, based on dashboard-indicated progress, and in private reports. These exercises provided opportunities to engage by both giving and receiving support and to iteratively refine action plans based on peer and interventionist responses. Self-affirmations were also elicited during sessions and part of resolution exercises practiced independently.

### 2.2. Pilot Randomized Controlled Study Using the Adapted Intervention

The pilot randomized controlled trial was pre-registered (#NCT05542199) and the protocol for the full trial was previously published [70,71,72]. In brief, the Brain and Body Health Study (BB-Health) is a food and lifestyle-based clinical trial designed to evaluate changes in cognitive function, weight status, brain blood flow, and accompanying biomarkers of obesity (e.g., blood lipids). The feasibility pilot trial was conducted at the Jean Mayer USDA Human Nutrition Research Center on Aging (HNRCA) at Tufts University. Participants were recruited by the visitor services team of the HNRCA and through local print and electronic media (e.g., advertisements, featured in newspapers, and social media posts) as well as through the posting of flyers in public places, such as local YMCAs, senior centers, supermarkets, and libraries. Additionally, HNRCA Volunteer Services recruits from an existing roster of contacts who have previously indicated a willingness to participate in nutrition research. This report describes participants randomized either into a healthy lifestyle program (DPP) or into a control group with no instructions regarding diet and lifestyle. CONSORT guidelines for the reporting of pilot randomized clinical control trials were used and can be found in Section S3. The sample size was determined to detect ≥ 80% power for WL among randomly allocated intervention and control groups of approximately equal size. Randomization occurred after baseline assessment, stratified by age (55–69; 70–85) and BMI (27–32.9; 33–39.9). Participants were randomized by a statistician using the Research Electronic Data Capture (REDCap) randomization module in a 1:1 allocation (intervention or waitlisted control), which remained the same throughout this study. All participants, regardless of allocation, received equal monetary compensation for their time spent in this study and during outcome assessments—no additional stipend was given for participation in the lifestyle intervention. Stipends were distributed at multiple points throughout this study; at 6 months, pilot participants would have received approximately USD 1000 each. The principal investigator, physician, interventionist, and participants were unblinded to the randomized group assignment. Staff conducting outcome measurements were blinded throughout the study. Free-living adults, aged 55–85 years old, with a BMI of 27–39.9 kg/m^2^, who have access to a computer and/or smartphone, and who are medically cleared to participate were eligible (Table 1).

To allow for rolling admissions, initial sessions up to the fourth were conducted one-on-one or in small groups, after which participants regularly met in groups of 4–10. Make-up sessions were usually accomplished by joining another group. Intervention sessions were recorded and transcribed, and poll responses were extracted. This report includes data from the first 20 enrolled participants, n = 10 randomized into the intervention (representing two total intervention groups) and n = 10 randomized into the control group, and followed during a six-month pilot of the novel modification. Participant characteristics at baseline can be seen in Table 1.

Session attendance and engagement were documented. Engagement was defined as the percentage of sessions where each individual actively contributed verbal comments during the meeting and responded to all session prompts and polls. Fidelity was defined as the complete presentation of all program curricula and materials to all participants [64].

Remote monitoring matched to the intervention sequence was utilized for intervention participant weight, physical activity, and food records. The following data summaries were downloaded from the commercial dashboard (Fitbit, 2022–2023): the ending weight for each week, average daily step count, Moderate–Vigorous Physical Activity (MVPA) minutes, and food logs. The percentage at/above predefined targets was used to categorize adherence: days with step count above 5000; days with MVPA above 22; weeks with ≥3 days of food log; and weeks with ≥3 days of scale weights. MVPAs were determined using heart rate measured as beats per minute (BPM), with moderate BPM 110–132 and vigorous BPM ≥ 133 BPM. On-site fasted weight was collected in duplicate at baseline, and at two subsequent on-site visits (at approximately three and six months) post-enrollment for both intervention and control groups (See Section S1).

### 2.3. Statistical Analysis

Paired two-sample *t*-tests were used to assess new weight status in the intervention group after six months of intervention exposure (17 sessions) compared to baseline weight, using the at-home scale. Unpaired two-sample *t*-tests compared between-group differences (intervention versus control) in mean weight change percent between baseline and the third on-site visit using the research center scale (approximately six months post-enrollment, agnostic to intervention exposure; Section S1). At-home and on-site scale measurements were validated with repeat measures at three different time points (Section S1) using Pearson correlation coefficients (Rho) with means assessed for statistically significant differences using paired *t*-tests. Paired two-sample *t*-tests assuming unequal variances were used to compare low- and high-weight-loss groups. Analyses were conducted in Microsoft Excel with the Data Analytics extension (v.2103 Excel, Microsoft, Redmond, WA, USA). Data from the current study are available upon reasonable request.

## 3. Results

After six months of the program pilot, participants reported a high degree of enjoyment and acceptability of the program, indicating good program compatibility. Additionally, participants indicated that the remote format supported their success (a sampling of questions and responses can be found in Supplemental Table S1). No harm relevant to the intervention was reported. Meeting attendance was 100% for intervention participants, and session engagement was high (Table 3). A total of 4 out of 120 participant sessions were make-up sessions. Program fidelity was high—both intervention groups received the same presentation material, and no curriculum materials were omitted for either group. The standardized presentations and perfect attendance ensured that the dose was the same across groups and in make-up sessions. Self-monitoring adherence was also high: all intervention participants wore their fitness trackers daily, weighed themselves >3 x/week, and logged food >3 x/week. The reported calorie adherence to the set calorie range was 74% for all six months (Table 3). The starting step count target of 5000 steps/day had 92% adherence over six months. Adherence to the MVPA target of 150+ min/week was lower (Table 3).

**Figure 2:**
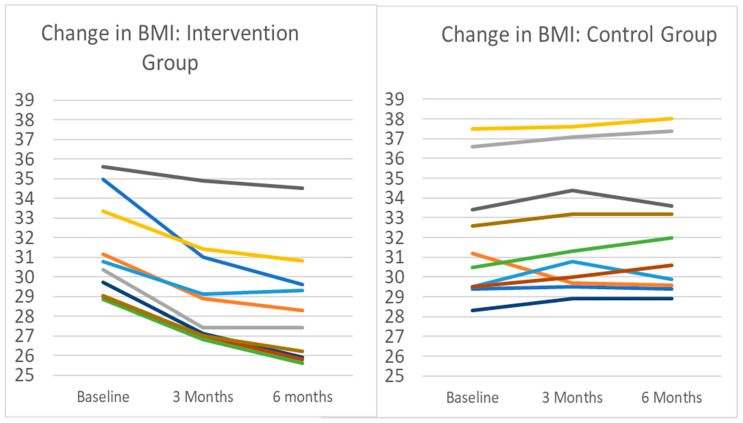
Change in BMI for intervention and control groups. Note: Each line represents an individual participant in the pilot. Intervention participants (n = 10) completed 3 months of remotely delivered intervention, including self-monitoring tasks, healthy lifestyle sessions, and interventionist support communication. Control participants (n = 10) were instructed to maintain current lifestyle behaviors. Mean (SD) change in body weight in the intervention group after 3 months was −7.1% (3.0%), from a baseline mean (SD) BMI of 31.3 (2.63) to BMI 29.06 (2.80). All intervention participants lost weight during the period. During the same time period, the BMI change in controls was a mean increase of 0.9 kg/m^2^ from 31.3 to 32.2, representing a 1.2% increase in mean body weight. Nine of the ten control group participants gained weight during the period.

After 12 intervention sessions (approximately three months post-enrollment; Section S1), the mean (SD) weight change for intervention participants compared to baseline was −7.0% (±3.0), which was significantly different from baseline (*p* < 0.0001) (Table 3). After 17 sessions (approximately six months), the mean weight change from baseline for the intervention group was −9.5% (±4.1) (*p* < 0.0001). By contrast, at three months post-enrollment, the mean weight change in the control group was +1.2% (±2.5), and was +2.4% (±1.8) at six months. The change in BMI for both groups is shown in Figure 2. Agnostic to intervention session progression, research center scales showed a weight change of −5.3% (±3.1) at three months post-enrollment in the intervention group, and −8.4% (±4.1) at six months. The between-group difference in mean weight was significant at both time points (intervention vs. control at 3 months, *p* < 0.004; at 6 months, *p* < 0.001). There was a small but statistically significant difference in date-matched WL comparing the home scale to the research center scale (−0.5 ± 0.6 kg; *p* < 0.004) (more details about comparative scale validation can be found in Section S1).

**Figure 3:**
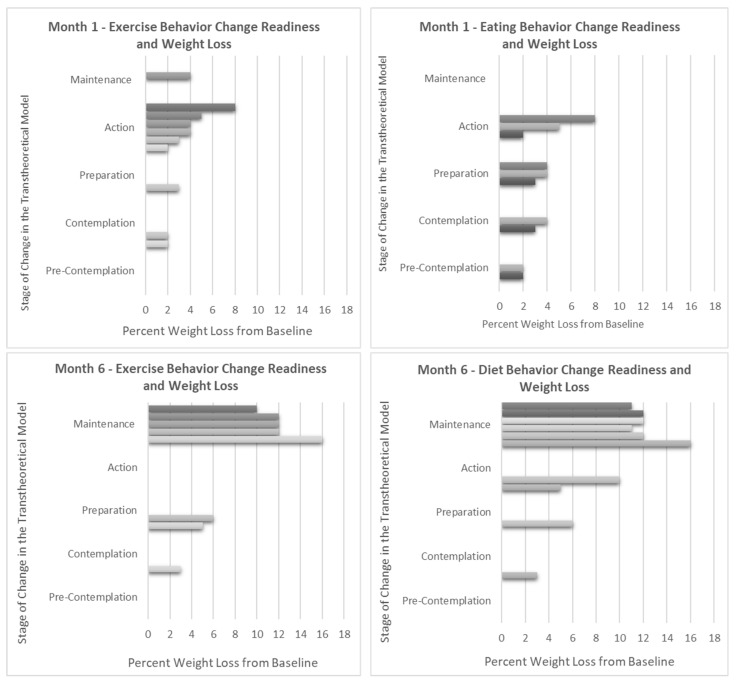
Weight change compared to Stage of Change readiness for exercise and diet at 1 and 6 months. Note: Each bar represents an individual in the pilot. Information on the Stage of Change (SOC) is gathered throughout the intervention during in-session polling. In the Transtheoretical Model, the SOC is an indication of the level of change readiness for a given health behavior and is known to change over time, sometimes progressing towards increasing levels of action and sometimes backsliding towards non-adherence. As expected, greater percentages of weight loss correspond with progression towards action and maintenance for both diet and exercise behaviors.

Weight loss percentage and Stage of Change (SOC) progression were closely tied. No participants slid backwards in their SOC; all but two participants moved forward towards action and maintenance (Figure 3). These two participants also had the lowest overall weight loss percentage from one to six months-- one participant remained in the preparation stage for both diet and exercise achieving 6% weight loss, and the other remained in the contemplation stage for diet achieving 3% weight loss. Interestingly, those in session one who indicated an exclusive focus on increasing exercise had a much lower mean (SD) weight loss at six months compared with participants who intended to change their eating behaviors (−5.0 ± 1.4% versus −12 ± 1.8%, *p* < 0.005) (Supplemental Table S1).

## 4. Discussion

Previous eHealth weight loss (WL) programs for older adults have reported mixed success [16,17]. The original DPP achieved 7% WL with intensive intervention components including individual and group meetings, and provided food and other services; however, the results from scaled-up implementations of DPP and other similar programs have typically produced WL results of only 3–5% [16,17]. The DPP adaptation described here builds upon the strength of the original DPP, the DPP-GLB, as well as other remotely delivered programs and interventions tailored for older adults [6,10,41,73] to achieve a mean WL of 9.5% after approximately six months in a diverse group of older adults. The mean WL was significant in intervention participants; furthermore, 90% achieved and sustained WL ≥ 5%—the level widely recognized to be clinically impactful [74]. During a similar period, the control group gained an average of 2.4% above their baseline body weight, with only one control group participant losing weight (−5.2%).

Main behavioral targets for WL, healthy eating, and daily exercise as well as strategies like logging were mostly unchanged from the original DPP. However, instruction of the curriculum, recommendations for achieving those targets, and the format in which they were delivered and logged were augmented by additional theory-informed components and reformatted into standardized presentations to maximize engagement and fidelity in the remote classroom setting. High fidelity, engagement, and interventionist and group support likely fostered increased acceptance and adherence to the program, which likely contributed to the greater weight loss outcomes achieved here. Stage-matched interventions informed by the Stage of Change (SOC) and Transtheoretical Model (TTM) are known to positively influence health outcomes in lifestyle programs, including the DPP-GLB [31,33,37,38], and for older adults [34]. Prior studies have also found MI to be effective in diet and exercise interventions, complementary to TTM [69], compatible with DPP, and effective for older adults [69,75]. These techniques are widely considered so effective because they support self-efficacy and skill-building [31] and have been observed to increase the adoption of program skills outside of sessions in remote programs [31]. Furthermore, Motivational Interviewing (MI) is known to integrate well with flipped classrooms where open discussions can elicit “change talk” and “sustain talk”, and in activities designed to encourage change motivation, drive the salience of personal values and self-directed goals, and to reinforce commitment [33]. The addition of these theory-informed approaches gave our participants the chance to trial and practice change while routinely reflecting and revisiting these experiences with peers.

As remote classroom education becomes more common, pedagogical theory aimed at optimizing online education has rapidly expanded. However, prior online health behavior change programs mostly preceded advances in cultural acceptance and strategy advancement for online education. As such, current and future eHealth programs can benefit from employing strategies known to increase the success of online education. In our remotely delivered sessions, members were invited to openly examine goals, share subjective experiences, identify and work through obstacles collaboratively, and offer each other support and advice. The Community of Inquiry (COI) framework provided an efficient, theory-based outline for restructuring the curriculum for an online classroom approach. Additional SOC and MI components provided tested theoretical strategies to improve the efficacy of discussions and feedback. While the theoretical underpinnings of the DPP are often discussed as unknown [25,76], this adaptation explicitly and consistently applies theory-based methods throughout all sessions to drive engagement, self-awareness, and commitment. The original designers of the DPP indicated that support and feedback from interventionists during programs were a critical element, predictive of greater weight loss success [9,67,77]. We built upon that important finding and applied the COI framework, the TTM, and MI to guide and enhance interventionist–participant interactions. Supporting the utility of these adaptations, our pilot had very high meeting attendance rates, fitness tracker usage, regular weight monitoring, and food logging relative to previous reports [6,7,9]. Participants also reported their appreciation for the convenience of the remote format and the ability to attend while at home, work, commuting, on vacation, and while caring for grandchildren.

Fidelity has previously been identified as a major issue for lifestyle program efficacy [6]. To address this, we developed standardized presentations to support program fidelity (Section S2) which, along with remote monitoring technology, allowed for an entirely online intervention. Sessions were structured around visual slides presented in a videoconference and outlined all session points. This allowed all participants to receive the same intervention information and to join other groups for make-up sessions without continuity issues. Session polling and structured conversation prompts resulted in high participation and engagement with learning objectives and offered the interventionist the ability to evaluate knowledge integration [78,79]. The use of the flipped classroom approach allowed concepts to be explored among peers and affirmed by both peers and the interventionist [78]. Additionally, operating the program remotely improved the ease of administration, required few research center resources, increased scheduling flexibility when, and enabled robust data collection, consistent with prior reports [21].

Technology utilization has historically been considered an obstacle for tech-based and/or remote programs for older adults [65,80]. The utilization of fitness tracking devices is associated with improved health outcomes for older adults, and some offer important added benefits including alerts for atrial fibrillation and for monitoring fall risks [16,17,20,80,81,82,83,84]. However, older adults are less likely than other adults to adopt these devices independently [81,82,83,84,85]. The perceived complexity and learning burden have been identified as key barriers to the adoption of fitness devices, but prior research suggests that when interventionist support is available, older adults indicate acceptance, enjoyment, and increased use of these self-monitoring tools [81,82,83,84,85]. Indeed, when adopted, these technologies seem to support greater WL success for older adults compared to younger adults [82,83,84]. Nevertheless, online, and tech-based interventions have historically largely been considered incompatible with older adults, and thus infrequently attempted, and at times even discouraged [65,80]. However, the recent uptick in telehealth and smart technology utilization among adults > 55 years of age presents a unique opportunity [16,17]. We found that the modifications made in this adaptation offered ample opportunity to support adoption, high utilization, and participant mastery of these modern tools. In previous studies, mixed doses of remoteness (i.e., hybrid formats) were suspected to negatively impact intervention programs, compared with exclusively in-person program formats [9]. Based on our pilot, we think that exclusively remote delivery may provide the opportunity for participants to try, practice, and adjust to technologies with exposure to more savvy peers. Unlike in a shifting format, or in one where participants can elect to go in-person, enrollment in a fully remote program provides opportunity for both vicarious and mastery experiences known to be key drivers for self-efficacy, alongside the persuasive and affective messaging of the program materials and interventionist communications [86].

Another minor, but interesting, observation was that participants who intended at baseline to focus primarily on exercise reached an average of 5% WL by six months, whereas those intending to change diet achieved an average of 12%. This supports findings by us and others that exercise may not play a central role in weight loss [87,88].

The proportion of adults in the United States over 55 years of age continues to increase, and along with it, the rise in prevalence of diet- and age-related chronic illness. Intervention programs that address lifestyle factors known to improve healthy aging and to reduce the prevalence, severity, and progression of these chronic illnesses are an urgent public health priority [1,2,3]. The DPP was developed to address major components of the disease progression from overweight/obesity and pre-diabetes to type 2 diabetes [4]; given that these components share considerable overlap with strategies to address other major diseases including cardiovascular disease, liver disease, kidney disease, and cognitive decline, strategies that optimize the efficacy of such interventions may have a substantial impact on the development and progression of several, co-ocurring and discrete major non-communicable diseases [74,89]. Our pilot was able to produce clinically impactful results among participants, whereas the otherwise similar participants randomized to the control group had a marked increase in their weight during the same period. Full-scale trials are needed to demonstrate the efficacy of this approach; however, if successful, further implementation in community and healthcare settings could help reduce the burden of cardiometabolic disease for this high-risk population.

### Strengths and Limitations

A major strength of our study was the development of materials to support future remote delivery of the DPP (Section S2). In addition, we used a randomized trial design to pilot-test the new modification. Our study did find a small, one-time variation in weight between at-home and research center scales. However, this one-time observation only occurred among three participants (Section S1) and was most likely the result of participants neglecting to fast before their on-site visit, or because the amount of variation was very small, it may be due to fluid intake/retention. The small difference in values was reported but did not change the conclusion that the intervention achieved substantial weight loss for all participants and clinically impactful weight loss (>5%) in 90%.

This report includes results from a six-month pilot feasibility trial, and, as such, has a small sample size (n = 20). Future research will evaluate longer-term results and within larger samples, including investigations within sub-groups and identified strata to support greater generalizability and scalability. Additionally, a larger study will be able to compare results to other biomarkers and comprehensive dietary intake assessment data.

## 5. Conclusions

This adaptation of the DPP was tailored to older adults and delivered as an entirely online, remote-classroom, eHealth intervention. Tailoring approaches for compatibility and program fidelity are likely to drive greater engagement and adherence to programs, with corresponding results in WL outcomes. Through a combination of personalized feedback, facilitated conversations, stage-matched prompts, dynamic classroom sessions, and remote-monitoring technology, we implemented a lifestyle program that fostered high adherence, attendance, and engagement (100%). Improved adherence and engagement were accompanied by clinically significant WL (9.5%), which is known to be a vital component of healthy aging among older adults with overweight and obesity.

Once thought incompatible with older adults, remote-education-based healthy lifestyle programs can be feasible and effective for this priority group. Remote technology and videoconferencing enable interventionist support, the development of health-promoting self-monitoring habits, and an environment of peer support that enables robust health behavior change. The use of standardized classroom materials developed for this pilot helped to safeguard fidelity to the program curriculum and can be utilized in future implementations with the potential to increase the efficacy of lifestyle interventions in older adult populations.

## Figures and Tables

**Table 1:** Study baseline characteristics.

Baseline Characteristics	Intervention n = 10	Control n = 10
Age, years mean(SD)	65.5(4.6)	65.3(6.3)
BMI, kg/m^2^ mean(SD)	31.3(2.63)	31.3(2.98)
Female, n(%)	7(70)	2(20)
Race n(%)		
Black/African American ^ƚ^	4(40)	1(10)
Hispanic/Latine	1(10)	1(10)
White ^ 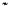 ^	5(50)	8(80)
		
Educational Attainment n(%)		
High School Diploma/Equivalent	2(20)	3(30)
Associate’s Degree	2(20)	0(0)
Bachelor’s Degree	6(60)	7(70)
		
Weight Category n(%)		
25 < BMI < 30	4(40)	4(40)
BMI ≥ 30	6(60)	6(60)

^ƚ^ Non-Hispanic Black/African American. ^
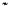
^ Non-Hispanic White.

**Table 2:** Adaptation versus Diabetes Prevention Program Group Lifestyle Balance.

BB-Health Adaptation	DPP-GLB
Technology: Wearable fitness tracker for remote data capture. ○ Automatic step counting; ○ Automatic exercise duration and intensity; ○ Automatic sedentary time; ○ Automatic sleep quality; ○ Optional Mindfulness. Wi-Fi Scale for remote data capture. Mobile Application and Web Dashboard. ○ Self-monitoring trends and reports. ○ Logging food and water. ○ Goal setting. ○ Reminders. ○ Social fitness platform. ▪ Messaging group and interventionist; ▪ Comparing progress; ▪ Recipes and exercise ideas; ▪ Achievement affirmations.	Optional-pedometer. Reference material and instruction for oral delivery without slide presentation; self-monitored logging by hand.
Online classroom materials: Session presentation slides. ○ Motivational Interviewing prompts; ○ Transtheoretical Model assessments, including Stage of Change; ○ Salience tasks with visual prompts; ○ Group goal setting and problem solving; ○ Polling, multi-media. Flipped classroom. ○ Peer-led segments; ○ Resonant examples carried forward. Age- and diversity-relevant images and examples. ○ Includes discussion of both cognitive and cardiometabolic health; ○ Stage of life and healthy aging focus; ○ Cultural and family focus; ○ High-definition photography and modern graphics for visual aids. Presenter guide embedded in slide notes to support quality control in delivery.	In person, sometimes remote. Discussion-based, with handouts (presenter and participant guide). General audience examples. Fewer images and simplistic graphics.
Affirmations and reports: Affirmations (cheers and progress) sent > 3× weekly. Trend alert messages. ○ Uptrend in body weight > 3 days; ○ No log/weight > 2 days. Detailed progress report. ○ Diet composition; ○ Goal attainment; ○ Diet and exercise recommendations; ○ Preparation for contribution to the group discussion.	Optional: In-person private weighing and review of logs prior to sessions.
Flexible diet composition: Supports range of fat and carbohydrate intakes and popular diet patterns. Emphasized age-appropriate protein targets and includes plant-based sources of protein. Emphasize high-fiber targets. ○ Legumes, fruit, and vegetables; ○ Reduced total grains, and recommends only whole grains. Emphasize reduced saturated fats. ○ Recommend healthy fats (unsaturated fats); ○ Total fat reduction only recommended when calorie budget exceeded. Supported healthy dietary patterns such as plant-based, MIND, Mediterranean, and DASH diet in session one with encouraged meal planning. Includes calorie counter guide and electronic food database to facilitate faster food logging, and food measuring tools.	Low-calorie, fixed. Low-fat and/or high-volume. My Plate. DASH diet was introduced during the second half of the program. Includes calorie counter guide, food-measuring tools.
Meetings: Same schedule, meeting time, and group format as DPP. Videoconferencing using any computer or smartphone. Discussion aloud or using the text-based chat. Make-up sessions. ○ Joining another group or one-on-one with the interventionist. Review progress data simultaneously with sessions and curriculum examples. Adult partner exposure to session discussions is allowed if requested.	In-person, infrequently by videoconference (primarily in COVID-19 adaptations).
Goal setting successively rather than fixed: Calorie range: ○ Supported exercise-adjusted targets adapted for older adults; ○ Weight progress-adjusted calorie targets. Activity level: ○ Focus on walking, or usual exercise with doctor approval; ○ Increase over time based on improved fitness. Weight loss: ○ Pace and attainment self-guided; ○ Shorter-term and longer-term individualized, initially 7% long-term; ○ Choice to focus on additional weight loss or weight loss maintenance after 7%; secondary goals based on new weight, not starting weight.	Calorie ranges are set based on starting weight with the corresponding fat (g) target. Activity levels are the same for all participants and include aerobic and resistance exercise including sports suitable for general audience. Weight loss goal is set to 7% for all participants, based on starting weight. Continued weight loss is only minimally discussed. Other health goals are secondary to weight loss and behavioral targets.
Theory-informed changes Session concepts implemented as didactic polling to the following: ○ Drive participation and evocative vocalizing; ○ Encourage personal insights, self-reflection, and peer-based reflection. Theory checkpoints such as Stage and Process of Change checkpoints: ○ Group and individual awareness raising; ○ Normativity of change; ○ Stage assessment; ○ Stage-matched recommendations. Open discussion for experience validation, the opportunity for reframing and summarizing challenges and successes with input from group members and interventionist.	Presenter guide encourages group discussion, which may eclipse key session concepts. Workbook-oriented examples and education.

**Table 3:** Adherence and outcomes for intervention participants.

Intervention (n = 10)						
	1 Month	2 Months	3 Months	4 Months	5 Months	6 Months
Adherence						
Daily kcal, mean(SD)	1284(305)	1289(352)	1289(387)	1407(742)	1252(466)	1326(490)
Calorie target, %(SD)	75(25)	77(27)	75(32)	70(28)	74(25)	73(29)
Steps/day, mean(SD)	10,918(6020)	10,765(5871)	10,872(5738)	10,932(5790)	11,453(5709)	11,608(5946)
Step target, %(SD)	95(11)	93(17)	93(17)	90(24)	85(27)	88(21)
MVPA/week, mean(SD)	455(436)	474(459)	492(430)	498(418)	530(395)	533(395)
Exercise target, %(SD)	70(42)	68(44)	73(34)	63(41)	65(43)	70(44)
Attendance, %(SD)	100(0)	100(0)	100(0)	100(0)	100(0)	100(0)
Scale weight, %(SD)	100(0)	100(0)	100(0)	100(0)	100(0)	100(0)
Food logging, %(SD)	100(0)	100(0)	100(0)	100(0)	98(3)	100(0)
Engagement, %(SD)	100(0)	100(0)	100(0)	100(0)	100(0)	100(0)
Outcomes	1 month	2 months	3 months	4 months	5 months	6 months
Weight change, %(SD)	−3.8(1.8)	−5.5(3.4)	−7.0(3.0)	−7.5(3.6)	−9.0 (4.4)	−9.5(4.1)
BMI, kg/m^2^ mean(SD)	29.9(2.6)	29.4(2.6)	29.1(2.7)	28.73(2.8)	28.40(2.8)	28.3(2.8)

Weight (Wi-Fi home scale) and Food log (self-report) adherence are defined as the percentage of weeks with these self-monitoring tasks completed ≥ 3 days/week. Engagement is defined as the percentage of sessions where each individual actively contributed verbal comments to the meeting and responded to all session prompts and polls [64]. Calorie target is the mean percentage of weeks where the average daily caloric intake was at or below the individual calorie intake target. Step target is the mean percentage of weeks with a daily step count average ≥ 5000. Moderate–Vigorous Physical Activity (MVPA) is exercise minutes with a heart rate above 110 beats per minute (BPM). Exercise target is the mean percentage of weeks with a weekly average of MVPA minutes ≥ 150. Weight outcomes are derived from home scales, with the group mean at each time point; changes are compared to baseline. The baseline BMI for the intervention group was 31.3 kg/m^2^ (±2.63) (see Supplemental Section S1).

## Data Availability

Pending IRB approval, pilot study data may be available to research institutions upon reasonable request.

## Supplementary Materials

The following supporting information can be downloaded at: https://www.mdpi.com/2072-6643/16/7/930/s1, Table S1: Example Participant Responses to Session Prompts and Poll Questions; Section S1: Comparisons Between the Research Center Scale and the Home Wi-Fi Scale; Section S2: All Standardized Presentation Materials; Section S3: CONSORT 2010 checklist of information to include when reporting a pilot or feasibility trial.
