# Supplementary material for: Development and Feasibility of an eHealth Diabetes Prevention Program Adapted for Older Adults—Results from a Randomized Control Pilot Study
Source: Nutrients. 2024 Mar 23;16(7):930. doi: 10.3390/nu16070930 (PMC11154527; doi:10.3390/nu16070930)
Supplement: Supplementary file 1 [file nutrients-16-00930-s001.zip › nutrients-2902367-supplementary.pdf]

SUPPLEMENTAL MATERIALS

Supplemental Table S1. Example Participant Responses to Session Prompts and Poll Questions.

	Session 4 prompt: *"How do you know it is working? What keeps you coming back?"*	Session 2 prompt: *"Where can you improve the most?"*	Session 1 prompt: *"What does success look like to you?"*
#5, Female	*"I am able to fit it into my schedule because I can zoom in from anywhere. In the car, at home, or with my grandkids I can still participate—I never have to miss a session."*	*"I need to make more time for exercise"*	*"I'd like to lose more weight."*
#1, Female	***"I lost weight right away. I'm not going to say its been difficult, its been both difficult and not difficult. Its kind of exciting and the program definitely helps me with the motivation to keep going."***	*"I need to have a plan for each meal, and plan to increase my fiber and protein the most"*	*"The scale keeps going down, I have more energy, and I feel happy."*
#6, Male	*"This program is great because it is making me more aware of what I'm eating and why I'm eating it and what I should be eating. I am becoming more aware of what a healthier way of living is and how to get going on it."*	*"I need to try to eat my meals at the same time every day to keep from snacking and overeating"*	*"Having at least one healthy meal per day, trying new foods and skipping the junk food."*
#3, Female	*"You know I always thought 'I eat well', and, thanks to {the program}, I now realize changing is going to help me lose the weight quicker. I realized that because I'm older it takes longer to get into shape."*	*"I'm still looking for things to improve, you'll tell me what I need to change as we go."*	*"My head is still in the game, I'm proud of my progress and I feel motivated to keep going."*
#2, Male	*"I wouldn't miss a session for the world. I love coming to meet with you guys every week."*	*" I don't get enough water throughout the day."*	*"When I see changes in my body."*
#7, Female	*"I've realized that I've got to take control of my life to be able to control my weight. I am learning that that means making my diet healthier. I am understanding how to keep myself physically healthy and how to monitor myself."*	*"I need to eat more slowly and choose healthier food options."*	*"Control impulses to eat when stressed and enjoy a well-prepared meal and avoiding snacking"*
#4, Female	*"Weight loss isn't always perfectly predictable. That's not how it works. If I keep on going and coming to meetings I am going to feel better, and my clothes are [already] much looser no matter what the scale says."*	*"I need to get back to my routine of drinking more water and doing yoga."*	*"I feel like I've accomplished what I set out to when I get 5,000 steps a day, and once I lose 10 pounds."*

#8, Male	*"I enjoy the approach of this program because I want to lose weight but I also want to have a diet I can enjoy."*	*"I want to improve controlling my portions, making healthy food choices, and not losing sight of my goals."*	*"Losing weight and also having a diet I can enjoy"*
#9, Female	*"Higher energy levels and physical flexibility due to all the walking."*	*"Being accurate about measuring my food and making more time for exercise."*	*"Early weight loss and keeping the weight off"*
#10, Female	*"The scale has dropped and my clothes don't fit the same and also how I feel, having more energy."*	*"I could improve my sleep. And portion control of what I eat."*	*"Losing weight on the scale or [when] your clothes are feeling a little bit looser. Remembering the words that seem to disappear from my head when trying to have a conversation with somebody."*

Note: Each session contains multiple polls and discussion prompts. All prompts and polls are included in the standardized presentation materials included in Appendix 2.

Section S1. Comparisons Between the Research Center Scale and the Home Wi-Fi Scale

At baseline, and at two additional time points a fasting duplicate measurement of weight was taken on-site for all participants, with shoes and outer clothing removed, using a calibrated scale at the research center.

At baseline (on-site visit 1), study participants were weighed while fasted at the beginning of the visit, using the center scale, prior to randomization. Individuals randomized into the intervention were then each assigned a wi-fi scale and weighed with the wi-fi scale during intervention onboarding, commonly in the afternoon of their baseline visit. For some participants, intervention onboarding was completed within four days of on-site baseline (mean 1.6 days). Participants in the control group were not assigned a wi-fi scale. The mean(SD) difference in weight between the wi-fi scales and the center scale at baseline was 0.3 kgs. ($\pm$0.47) and with a Pearson Correlation of Rho >0.99. The t-test for differences in paired means was not significant at baseline ($p<0.06$).

To allow for scheduling flexibility, the protocol allowed for the second on-site study visit to occur within a window up to 28 days before or after the 12 week point of enrollment. The difference in time between three months of intervention exposure and the second on-site visit ranged from 21 days before to 6 days after. All intervention group participants weighed themselves using their wi-fi scale at home in the morning prior to arriving at the study center for their second on-site visit. The mean(SD) difference in weight between date-matched wi-fi scale and center scale was -0.5 kgs (±0.9) with a Pearson Correlation of Rho >0.99. The t-test for differences in paired means was not significant at the second on-site visit (p<0.13). Mean(SD) weight loss according to the center scale was 5.3% (±3.1) from baseline to visit two, compared to 6.1% (±3.6).

The visit window for the third on-site visit was the same as the second, and visits ranged from 1 day before to 12 days after 24 weeks of enrollment. All intervention group participants weighed themselves using their wi-fi scale at home in the morning prior to arriving at the study center for their scheduled third on-site visit. The mean(SD) difference in date-matched weight between wi-fi scale and center scale was -0.5 kgs (±0.6) with a Pearson Correlation of Rho >0.99. The t-test for differences in paired means was significant for the third on-site visit (p<0.04), apparently due to discrepancies in scale weights for three participants. Mean(SD) weight loss according to the center scale was 8.4% (±4.1) from baseline to third visit, compared to 9.3% (±4.1) on the wi-fi scale on the same day. The t-test of difference in mean weight loss from baseline to the third site visit was significant with a small difference in average weight loss between the home scale and the clinic scale (9.5% ±4.5 vs 9.0% ±4.4, p<0.004). Given that three participants appeared to account for the discrepancy, with lower home weights on the day of the clinic visit relative to prior home weights, we speculate that the small difference in weight between first morning scale weight at home and later morning scale weight on the center scale in those individuals was due to fluid intake and/or from non-compliance with the requirement to fast prior to clinic measurements. Further research is needed to investigate the cause of the small (0.5 kg) discrepancy noted here.

Section S2. All Standardized Presentation Materials (PDF Files ATTACHED; Complete presentation files (PPTX) available)

Section S3

CONSORT 2010 checklist of information to include when reporting a pilot or feasibility trial*

Section/Topic	Item No	Checklist item	Reported on page No
**Title and abstract**			
	1a	Identification as a pilot or feasibility randomised trial in the title	1, Title
	1b	Structured summary of pilot trial design, methods, results, and conclusions (for specific guidance see CONSORT abstract extension for pilot trials)	1
**Introduction**			
Background and objectives	2a	Scientific background and explanation of rationale for future definitive trial, and reasons for randomised pilot trial	1-3
	2b	Specific objectives or research questions for pilot trial	2-3
**Methods**			
Trial design	3a	Description of pilot trial design (such as parallel, factorial) including allocation ratio	10
	3b	Important changes to methods after pilot trial commencement (such as eligibility criteria), with reasons	n/a
Participants	4a	Eligibility criteria for participants	10
	4b	Settings and locations where the data were collected	3, 10
	4c	How participants were identified and consented	10

Interventions	5	The interventions for each group with sufficient details to allow replication, including how and when they were actually administered	3-11
Outcomes	6a	Completely defined prespecified assessments or measurements to address each pilot trial objective specified in 2b, including how and when they were assessed	10-11
	6b	Any changes to pilot trial assessments or measurements after the pilot trial commenced, with reasons	n/a
	6c	If applicable, prespecified criteria used to judge whether, or how, to proceed with future definitive trial	n/a
Sample size	7a	Rationale for numbers in the pilot trial	10
	7b	When applicable, explanation of any interim analyses and stopping guidelines	n/a
Randomisation:			
Sequence generation	8a	Method used to generate the random allocation sequence	10
	8b	Type of randomisation(s); details of any restriction (such as blocking and block size)	10
Allocation concealment mechanism	9	Mechanism used to implement the random allocation sequence (such as sequentially numbered containers), describing any steps taken to conceal the sequence until interventions were assigned	10
Implementation	10	Who generated the random allocation sequence, who enrolled participants, and who assigned participants to interventions	10
Blinding	11a	If done, who was blinded after assignment to interventions (for example, participants, care providers, those assessing outcomes) and how	10
	11b	If relevant, description of the similarity of interventions	n/a

Statistical methods	12	Methods used to address each pilot trial objective whether qualitative or quantitative	10
**Results**			
Participant flow (a diagram is strongly recommended)	13a	For each group, the numbers of participants who were randomly assigned, received intended treatment, and were assessed for each objective	10-11, Table 1
	13b	For each group, losses and exclusions after randomisation, together with reasons	n/a
Recruitment	14a	Dates defining the periods of recruitment and follow-up	3, 10
	14b	Why the pilot trial ended or was stopped	n/a
Baseline data	15	A table showing baseline demographic and clinical characteristics for each group	Table 1
Numbers analysed	16	For each objective, number of participants (denominator) included in each analysis. If relevant, these numbers should be by randomised group	11-16
Outcomes and estimation	17	For each objective, results including expressions of uncertainty (such as 95% confidence interval) for any estimates. If relevant, these results should be by randomised group	11-16
Ancillary analyses	18	Results of any other analyses performed that could be used to inform the future definitive trial	11-16
Harms	19	All important harms or unintended effects in each group (for specific guidance see CONSORT for harms)	11
	19a	If relevant, other important unintended consequences	n/a
**Discussion**			

Limitations	20	Pilot trial limitations, addressing sources of potential bias and remaining uncertainty about feasibility	16
Generalisability	21	Generalisability (applicability) of pilot trial methods and findings to future definitive trial and other studies	15-16
Interpretation	22	Interpretation consistent with pilot trial objectives and findings, balancing potential benefits and harms, and<br>considering other relevant evidence	14-16
	22a	Implications for progression from pilot to future definitive trial, including any proposed amendments	16
**Other information**			
Registration	23	Registration number for pilot trial and name of trial registry	3
Protocol	24	Where the pilot trial protocol can be accessed, if available	3, 10
Funding	25	Sources of funding and other support (such as supply of drugs), role of funders	18
	26	Ethical approval or approval by research review committee, confirmed with reference number	18

Citation: Eldridge SM, Chan CL, Campbell MJ, Bond CM, Hopewell S, Thabane L, et al. CONSORT 2010 statement: extension to randomised pilot and feasibility trials. BMJ. 2016;355. This is an Open Access article distributed in accordance with the terms of the Creative Commons Attribution (CC BY 3.0) license (http://creativecommons.org/licenses/by/3.0/), which permits others to distribute, remix, adapt and build upon this work, for commercial use, provided the original work is properly cited.

*We strongly recommend reading this statement in conjunction with the CONSORT 2010, extension to randomised pilot and feasibility trials, Explanation and Elaboration for important clarifications on all the items. If relevant, we also recommend reading CONSORT extensions for cluster randomised trials, non-inferiority and equivalence trials, non-pharmacological treatments, herbal interventions, and pragmatic trials. Additional extensions are forthcoming: for those and for up-to-date references relevant to this checklist, see www.consort-statement.org.
