# Supplementary material for: Development and Feasibility of an eHealth Diabetes Prevention Program Adapted for Older Adults—Results from a Randomized Control Pilot Study
Source: Nutrients. 2024 Mar 23;16(7):930. doi: 10.3390/nu16070930 (PMC11154527; doi:10.3390/nu16070930)
Supplement: Supplementary file 1 [file nutrients-16-00930-s001.zip › Session 15 _SUPP.pdf]

BB-HEALTH

e-Healthy Older Adults Modification

Diabetes Prevention Program

Session #15
MINDFULNESS!

Group Opening Discussion

BB-HEALTH

WELCOME BACK! **HOW WAS THE LAST TWO WEEKS**?

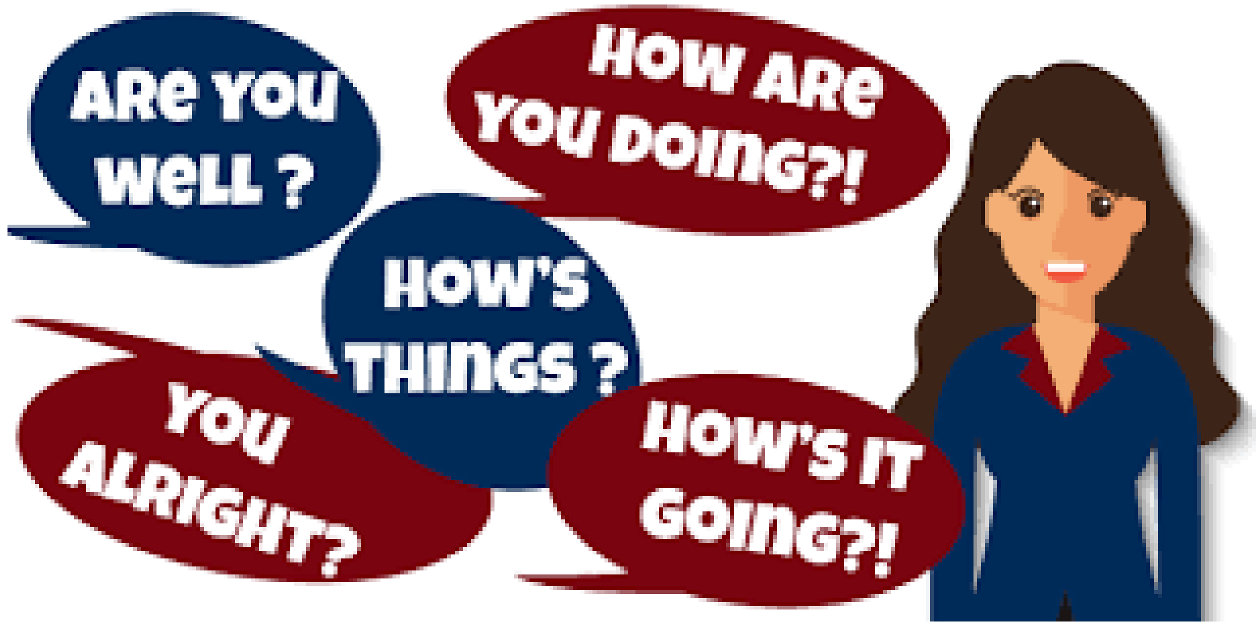

1. Did you track every meal?
2. Did you weigh every day, & record your health today?
3. Did you stay Aware and In Control?
4. Did you increase your moderate and vigorous exercise?
5. Did you email the Intervention Team and review the response?
6. What were your unique challenges and successes?

REMINDER: CHARGE YOUR FITBIT & SCALE DURING THIS MEETING!

Putting it into practice

Where did you buy healthy food in the last month? (select all)

- Grocery store
- At work
- While traveling or commuting
- At a café
- At a restaurant
- At a street food vendor
- At a convenience or corner store?
- Other (discuss)

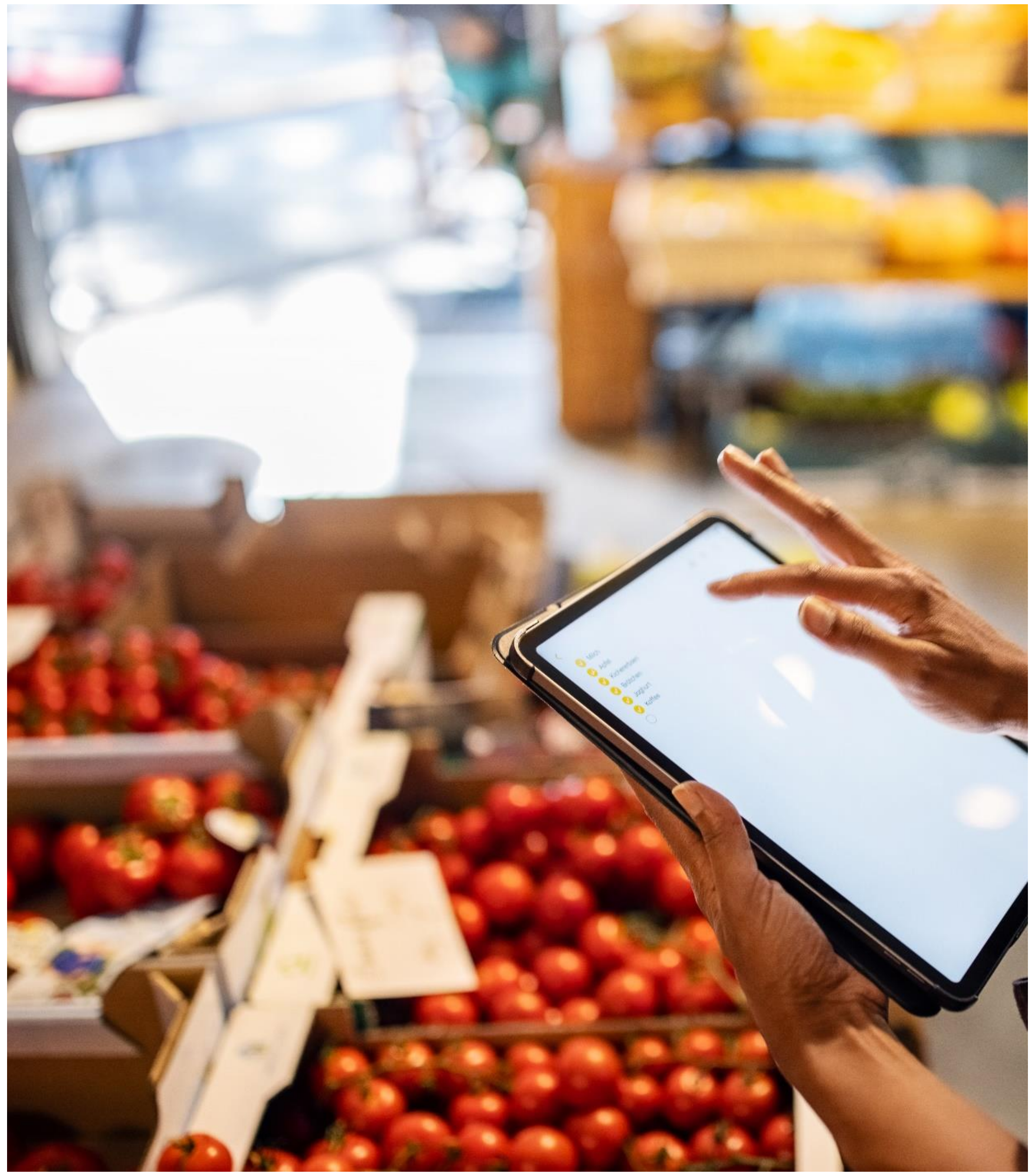

What are some healthy foods you will choose in the coming weeks?

- *Respond in the chat!*

Different Settings

BB-HEALTH

What are some healthy foods will you buy at the grocery store?

What are some healthy foods you will order at restaurants and cafes?

What are some healthy foods you will choose at work or spending the day out of your home?

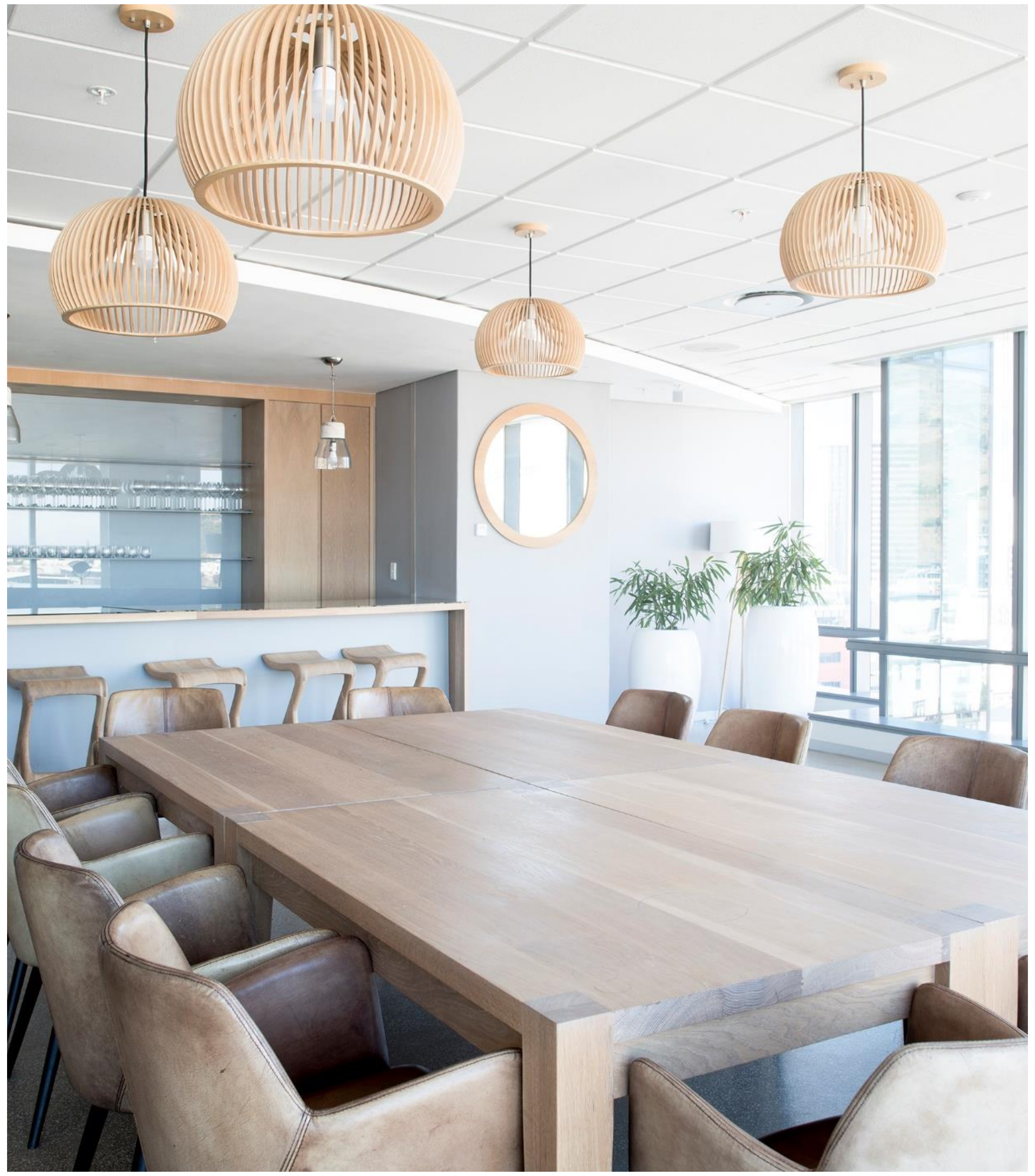

OPEN DISCUSSION- and feel free to add your ideas and questions into the chat!

Mindful Eating -- Aware

Pausing to focus on the experience of eating

- Eating slowly
- Taking time to SAVOR the taste, texture, and feeling of eating
- Removing distractions, like TV or reading
- Able to feel physical sensation of "fullness" and satisfaction

The opposite is mindless eating

- Eating without feeling satisfied
- Eating more than you really want
- Eating without enjoying

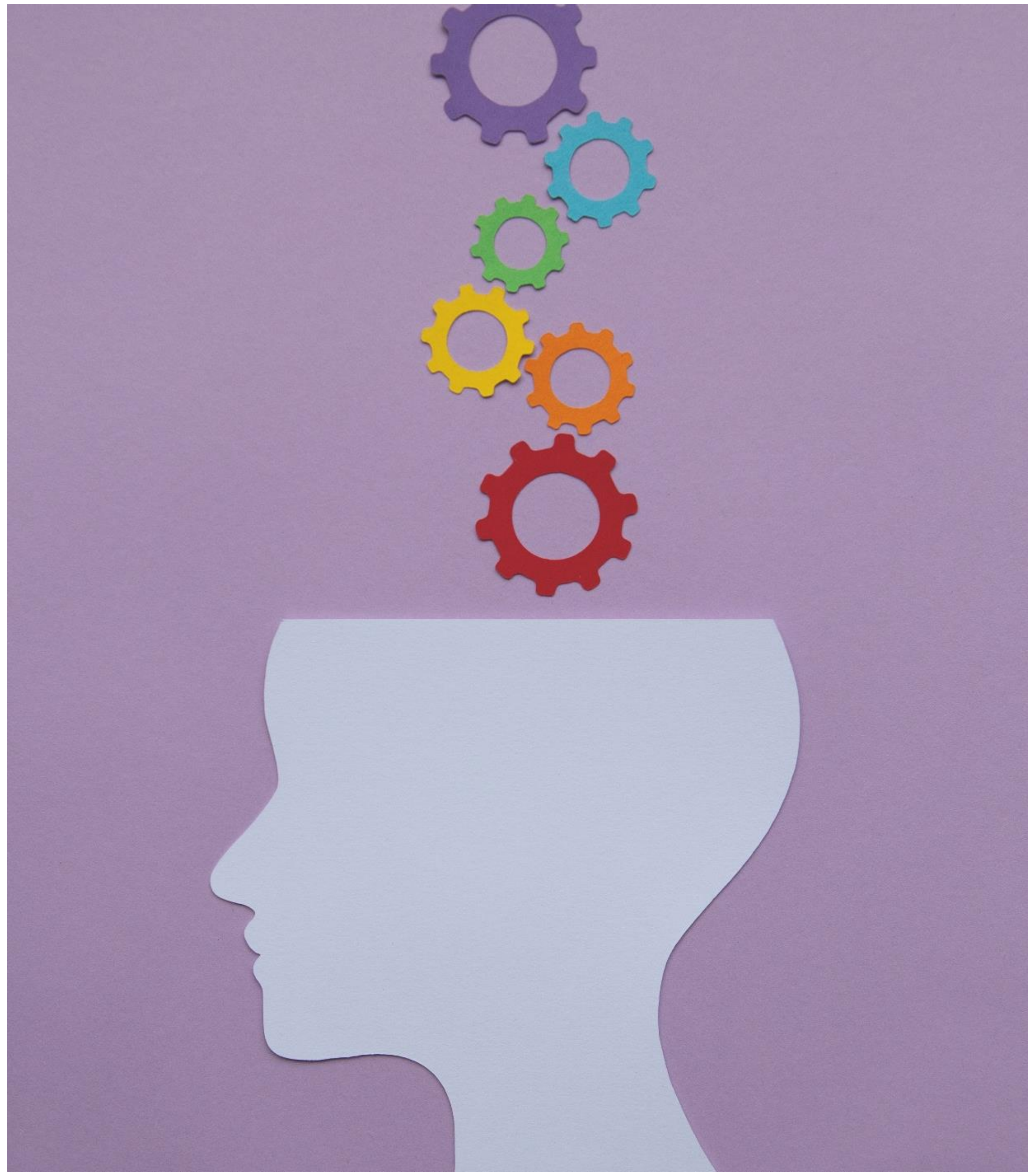

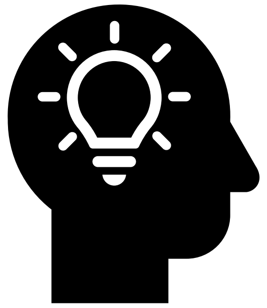

Aim to incorporate mindful eating:

1. **make meals last 20+ minutes**.
2. **Pause to check hunger/ fullness.**

You can save leftovers for later!

Mindful Movement -- Aware

BB-HEALTH

Being aware and *intentional* about how we move

- Taking the stairs instead of the elevator
- Standing during meetings instead of sitting
- Being aware of feeling better after moving
- Increasing the speed or duration to get heart rate up

The opposite is:

- Staying too sedentary
- Ignoring body cues and discomforts
- Only exercising because you have to

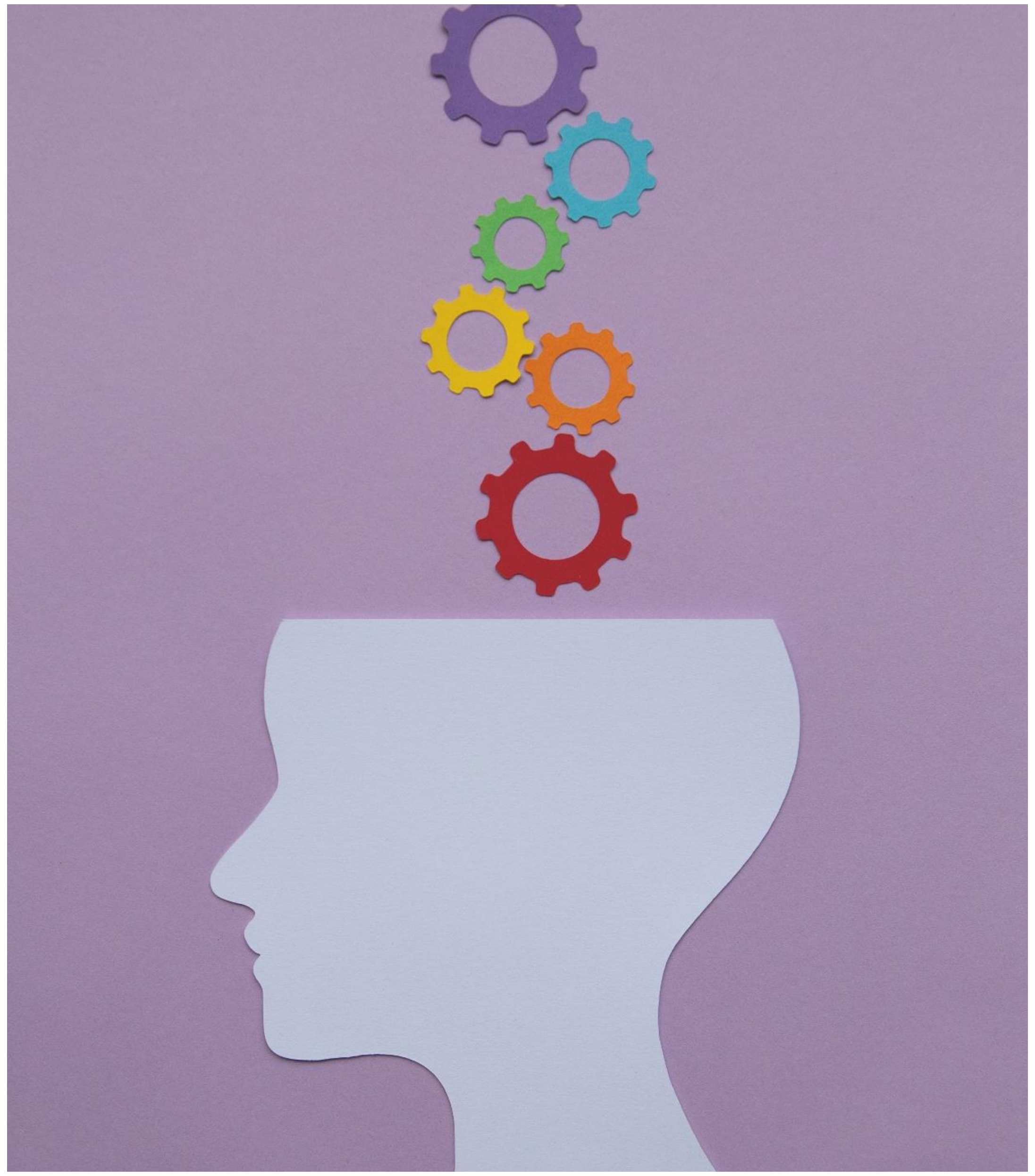

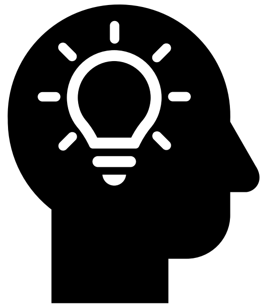

Movement is a way to feel better, think better, and to enjoy the world around you. Show appreciation to your body for being able to move you through your life!

Mindfulness

What are you typically doing while eating (select all)

- Watching TV
- Driving
- Working
- Reading
- Talking with friends and family
- Focused on the meal, eating the food
- Experiencing gratitude for a good meal
- Examining the flavors and textures
- Other (discuss)

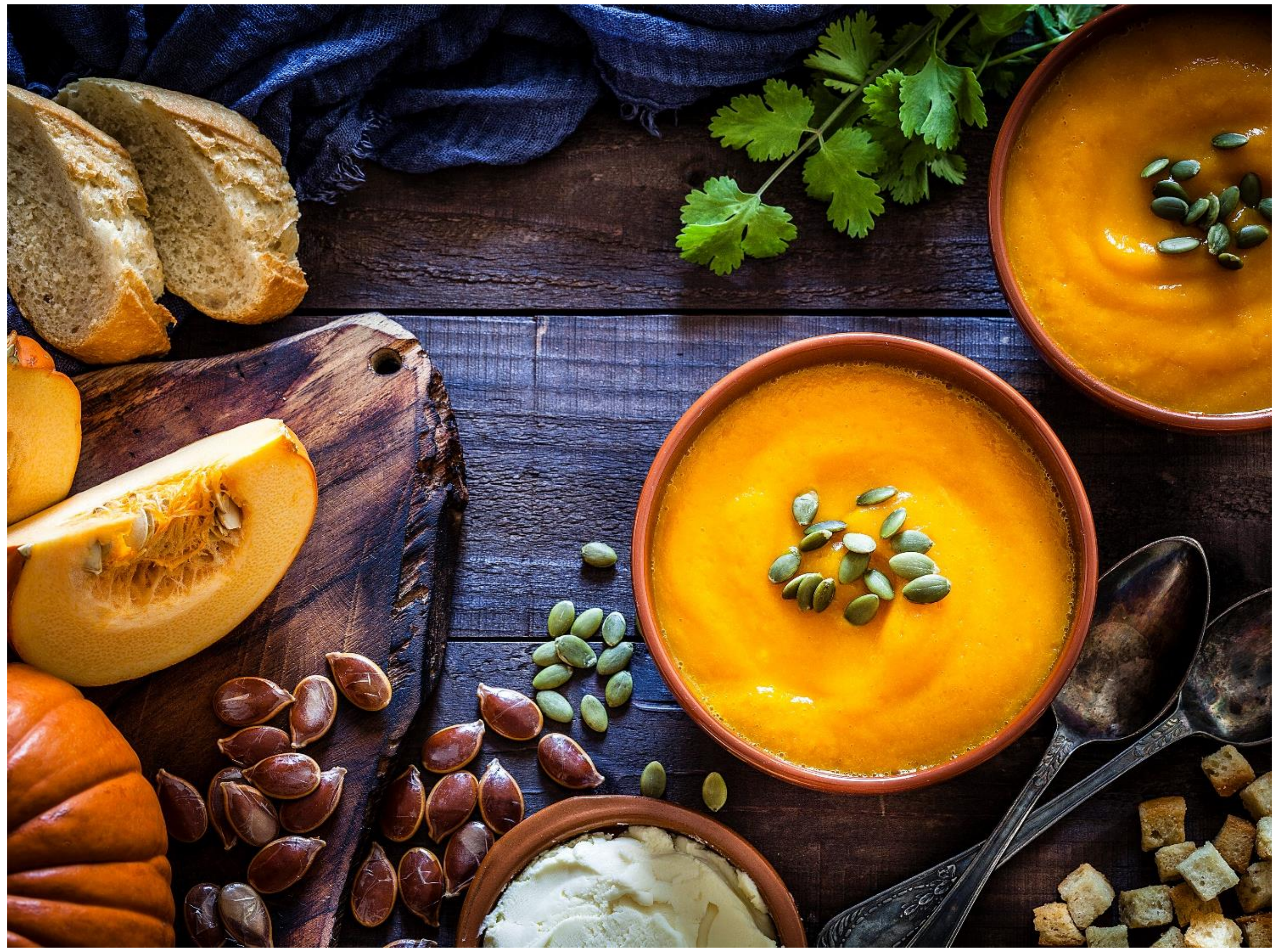

BB-HEALTH

Mindfulness

How long does it usually take you to eat a meal?
(select one)

- I eat as quickly as I can (less than 5 min)
- I don't spend much time eating (5-10 min)
- I take my time but don't take pauses (10-15min)
- I try to eat slowly and enjoy (15-20 min)
- I savor every bite and take pauses (20-25 min)
- I eat slowly (25-30 minutes)
- I take a long time to eat a meal (30-35 minutes)

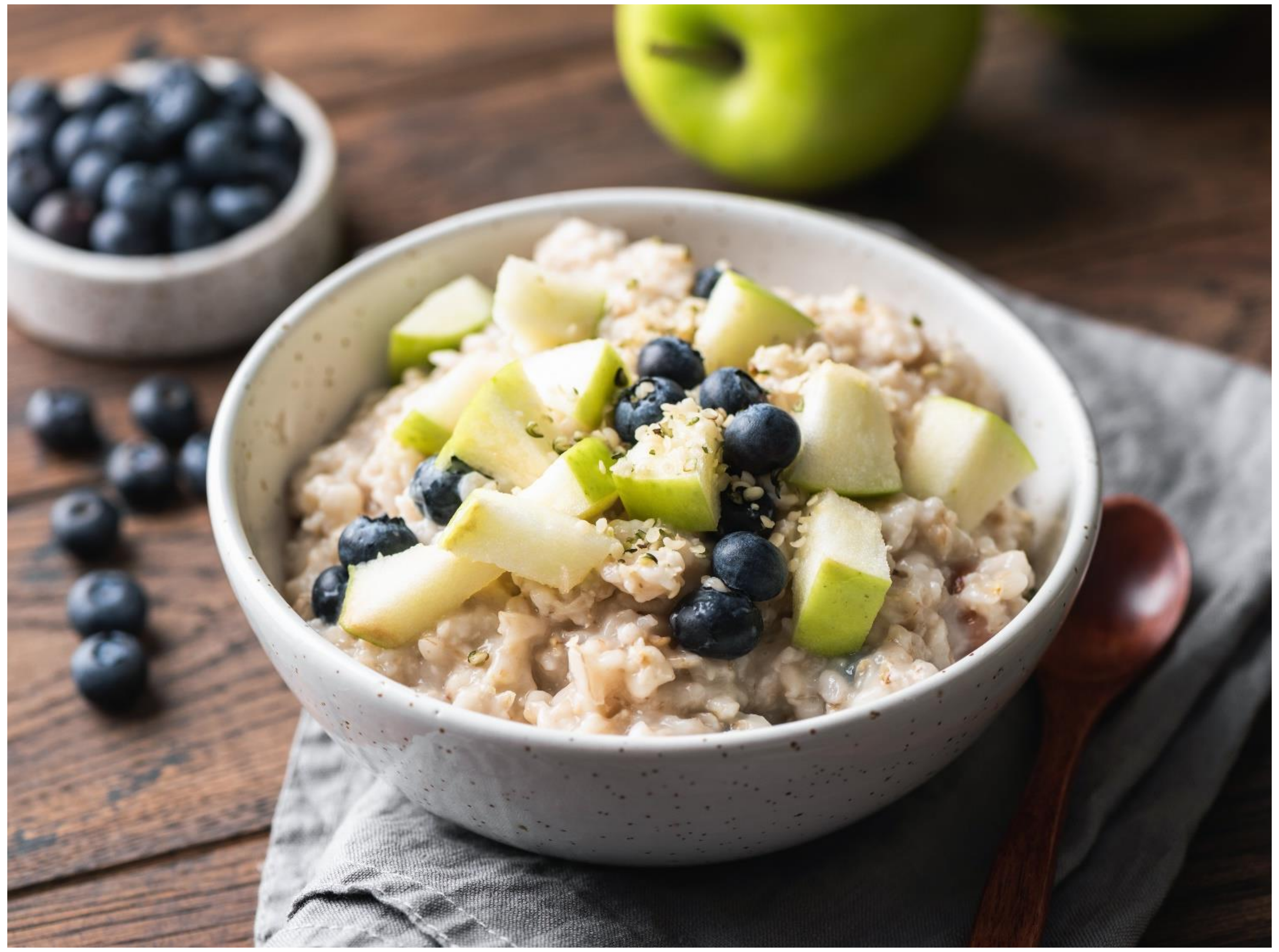

Mindfulness

My typical movement and activity is: (select all)

- Pre-planned walking or activities
- Moving around the house/ Chores
- Spontaneous, but often
- Spontaneous and infrequent
- Walking the dog
- Only when someone invites me/reminds me
- How I break up my day
- How I start my day
- Other (discuss)

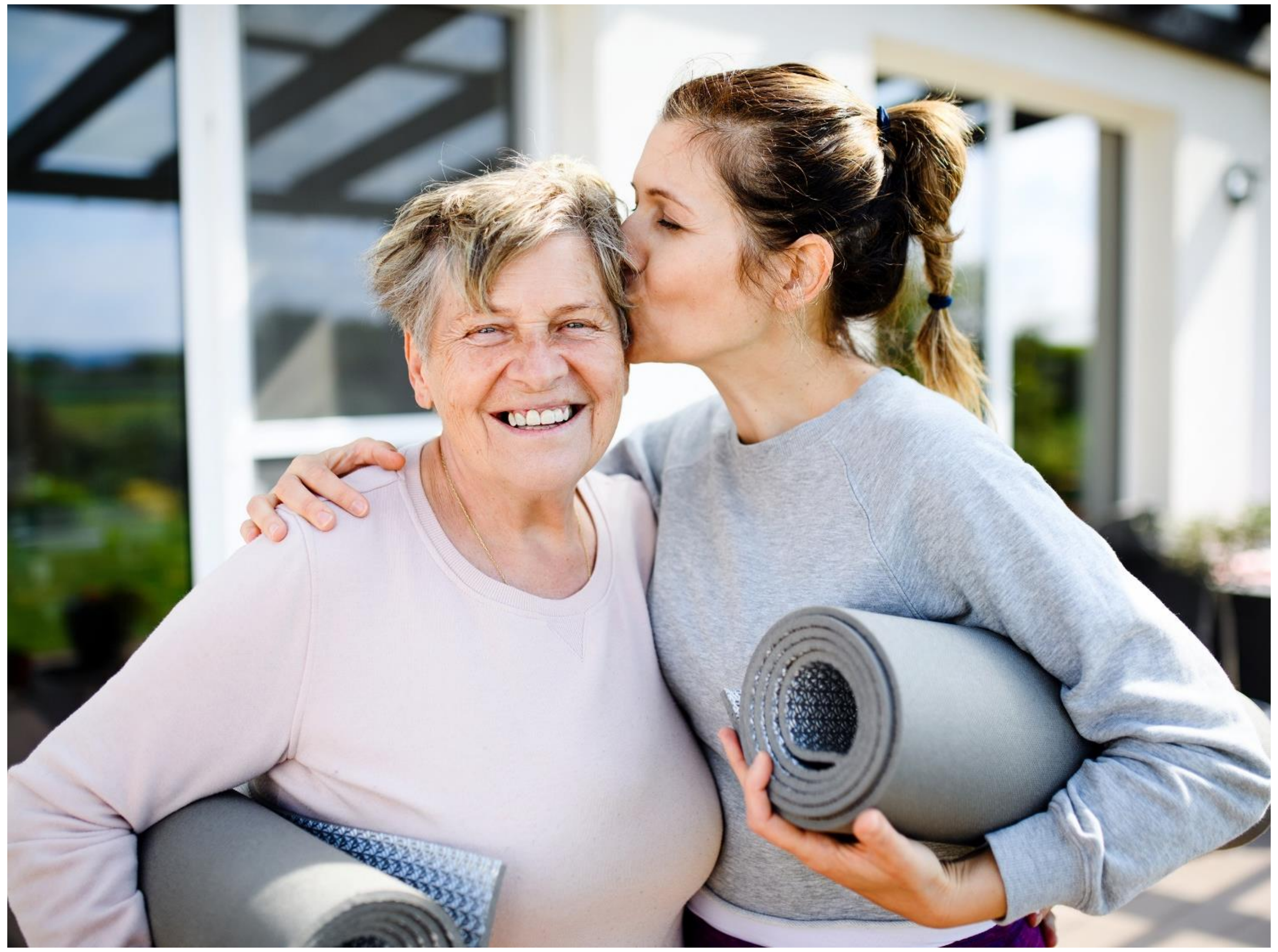

Mindfulness

BB-HEALTH

How much do you enjoy physical activity? (select one)

- I love it, crave it, and always want it
- It makes me feel better and I do it frequently
- I enjoy it, but don't always want to do it
- I like it sometimes
- Its ok, but I don't really want to do it
- I don't like regular physical activity
- I strongly dislike it, and I actively avoid it

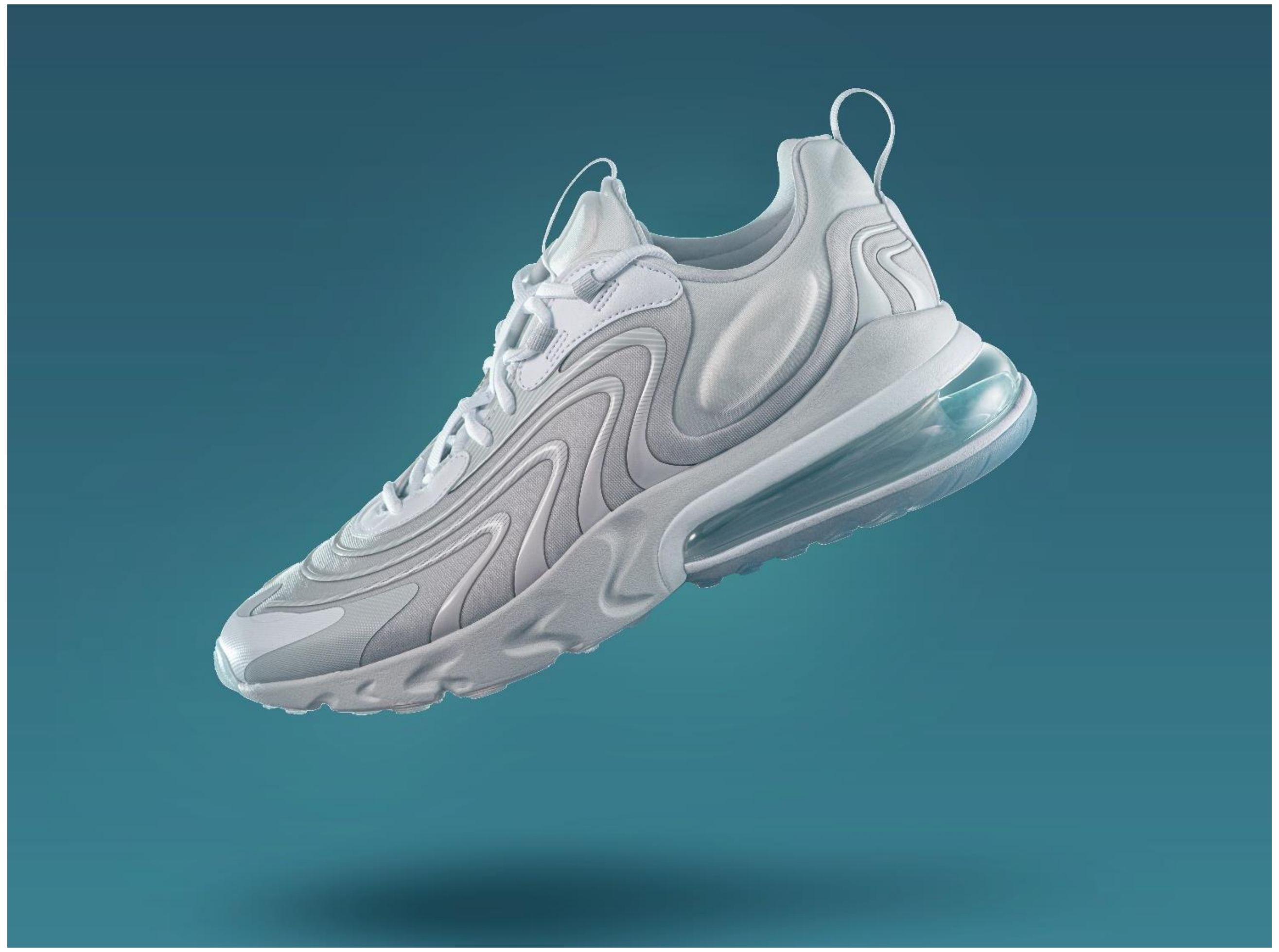

Put it into Practice

Your Lifestyle, in Control!

1. Track those meals and take your health EVERYDAY!
   - Monitor your progress and identify areas for improvement towards YOUR GOALS

2. Aim to get in 90 min of moderate or vigorous exercise ***a week*** by our next session!
   - Make your total active time over 200 min this week!
   - Keep up your steps, reduce sedentary time– get all 9!

3. Practice Mindful Eating – write a journal entry about the flavor and experience of eating slowly
   - Focus on breathing and take frequent pauses
   - Don't rush to the next bite without fully enjoying your current bite

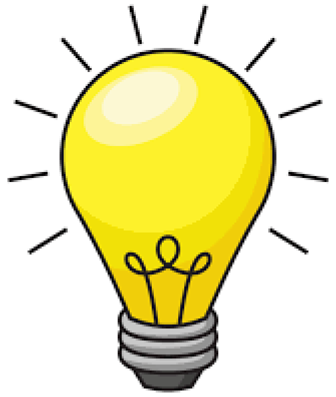

THIS WEEK'S TIP: ***Mindfulness is a tool to unlock enjoyment***
Healthy eating and physical activity can be chores or pleasures– focus on what you like about them and your experience will become more joyful!

Questions?

B B - H E A L T H

Over the next two weeks:

1. Increase your physical activity!

2. Track your food and exercise and **<u>Weigh Every Day</u>**

3. Remember your calorie, fat, and activity targets

4. Email your logs and progress to the Intervention Team and Review the response!

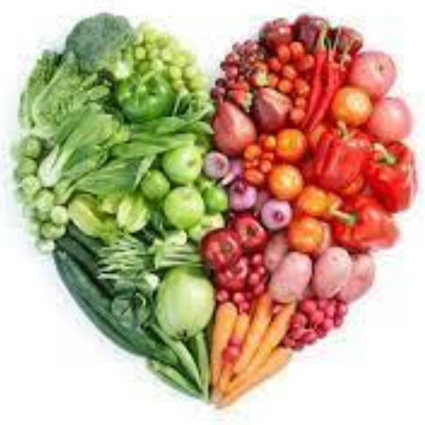

FOOD FOR THOUGHT

- Slowing down is great for mood too because it helps you enjoy life more!
- Success is best supported when you lean in to joy, not increase restriction
