# Supplementary material for: Development and Feasibility of an eHealth Diabetes Prevention Program Adapted for Older Adults—Results from a Randomized Control Pilot Study
Source: Nutrients. 2024 Mar 23;16(7):930. doi: 10.3390/nu16070930 (PMC11154527; doi:10.3390/nu16070930)
Supplement: Supplementary file 1 [file nutrients-16-00930-s001.zip › session 17_SUPP.pdf]

BB-HEALTH

e-Healthy Older Adults Modification

Diabetes Prevention Program

Session #17
SIT LESS, MOVE MORE!

BB-HEALTH

Group Opening Discussion

WELCOME BACK! <u>**HOW WAS LAST MONTH**</u>?

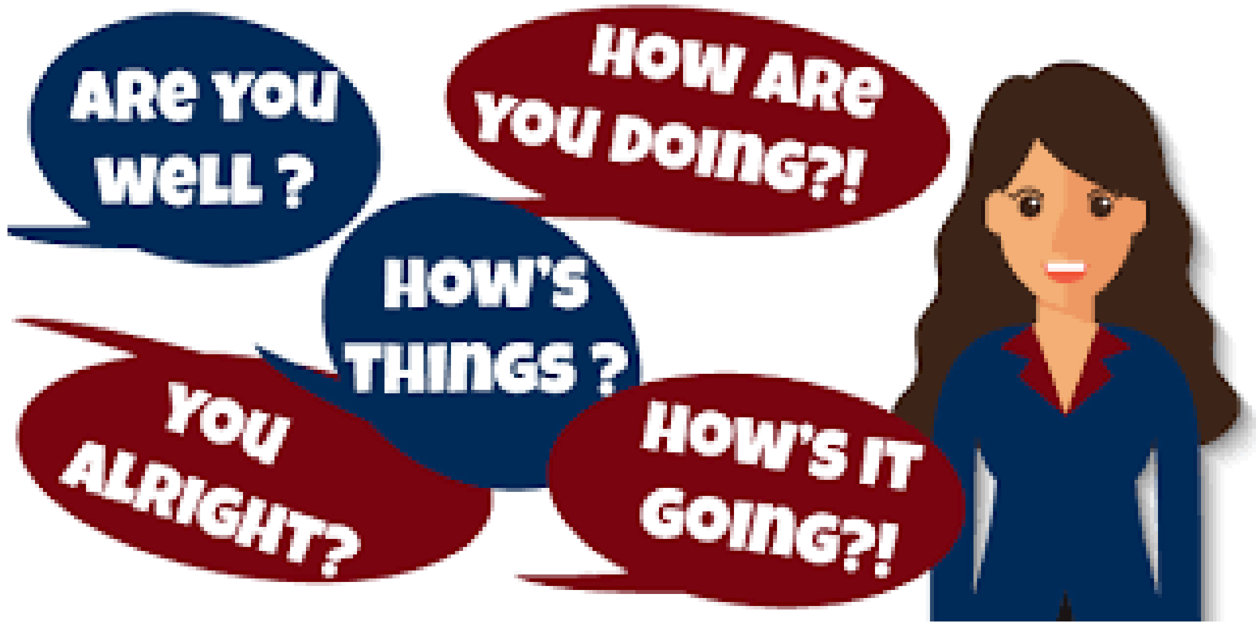

1. Did you track every meal?
2. Did you weigh every day, & record your health today?
3. Did you have good Sleep Hygiene?
4. Did you manage your stress and increase resilience?
5. Did you email the Intervention Team and review the response?
6. What were your unique challenges and successes?

REMINDER: CHARGE YOUR FITBIT & SCALE DURING THIS MEETING!

Join the movement movement

How does physical activity make you feel? (select all)

- Physically good (more flexible, stronger, less achy)
- More energy
- More connected to the world around me
- Tired
- Achy
- More checked out
- Other, discuss

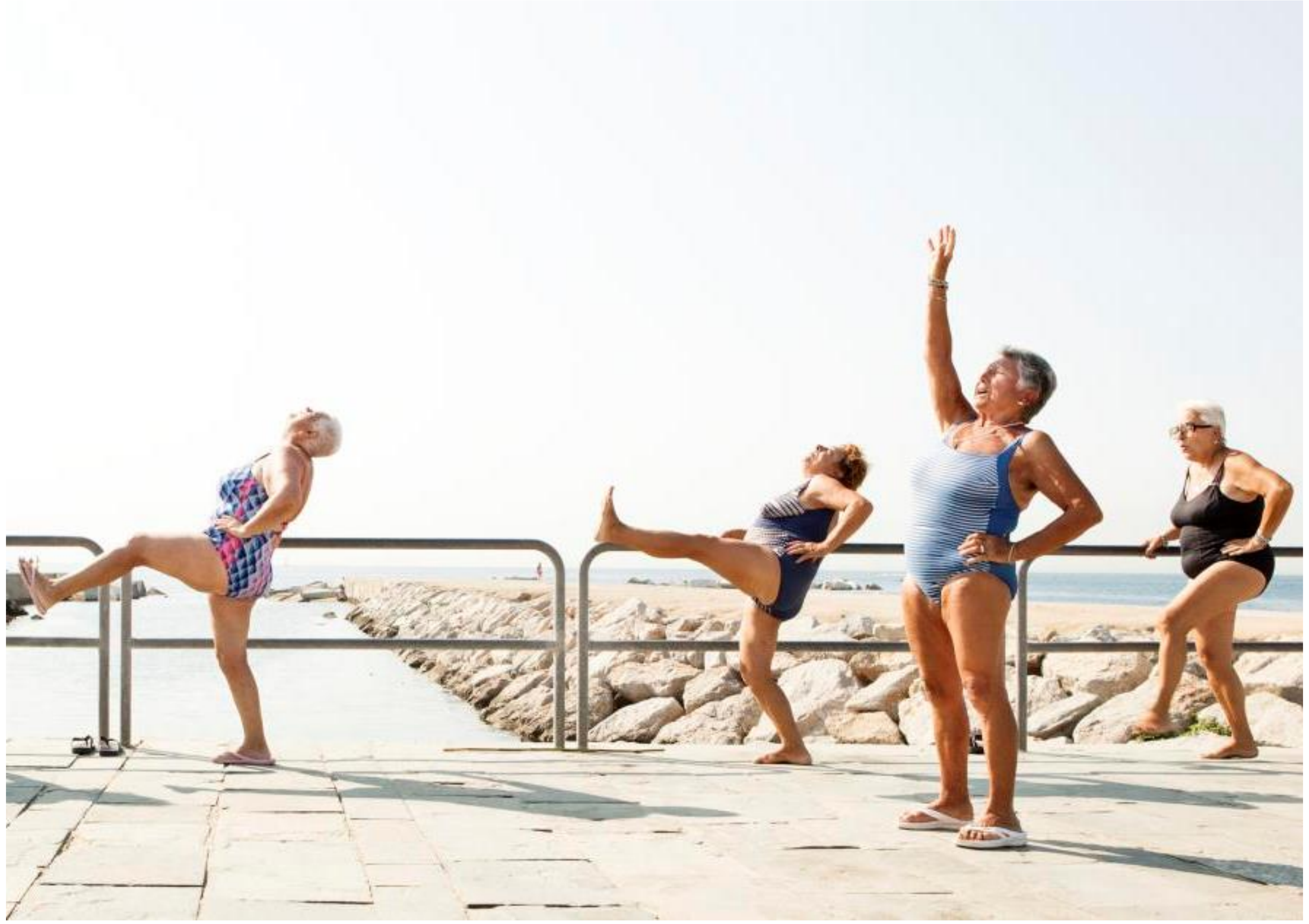

Bringing activity levels up

BB-HEALTH

What daily activities can I add activity or standing to instead of sitting and being sedentary? (select all)

- Phone calls
- Meetings
- Housework
- Desk work
- Watching TV
- Running errands
- Playing with kids and grandkids
- Playing with family pets
- Other (discuss)

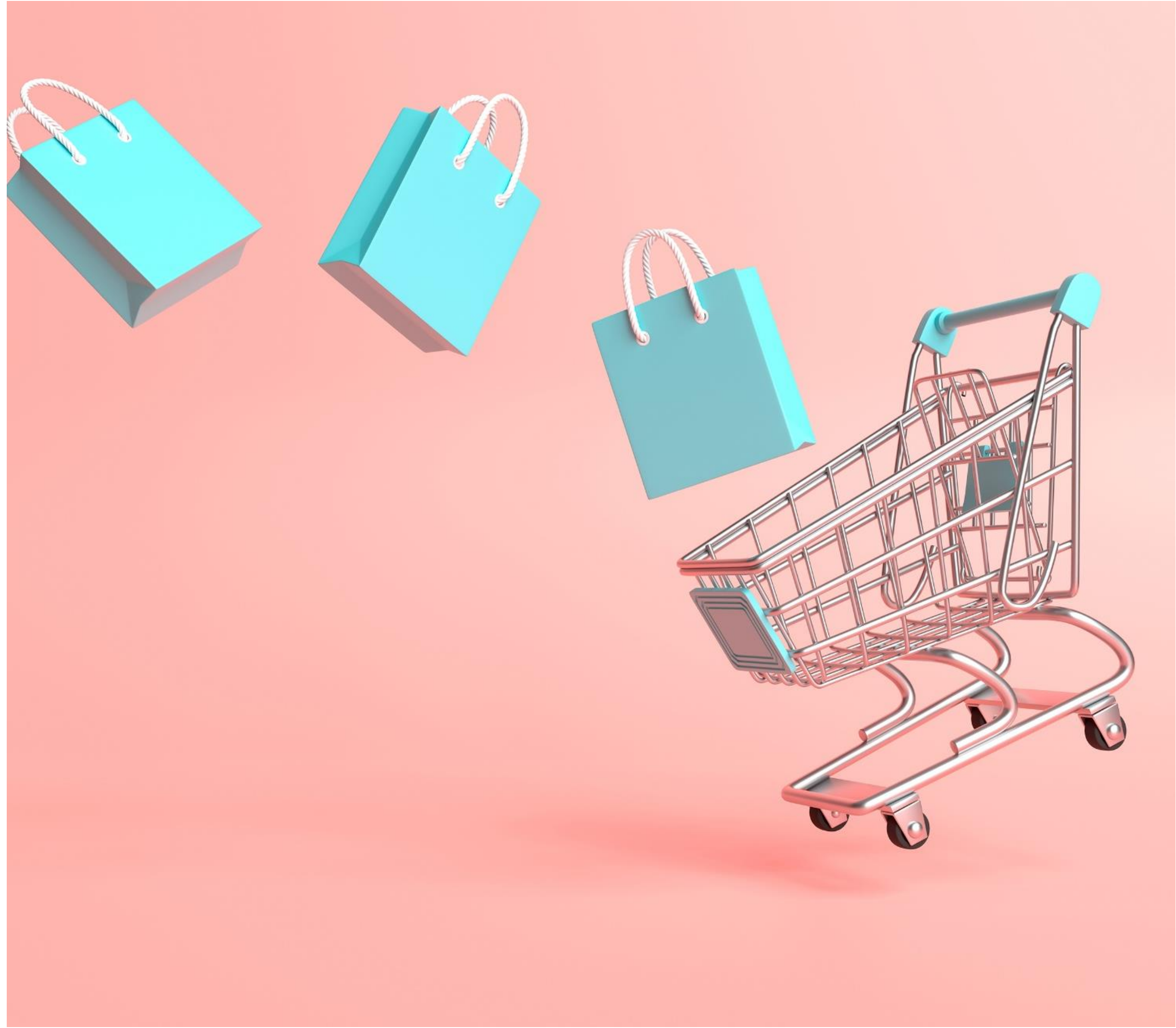

Tell us in the chat how you would add or increase physical activity into these daily tasks

Sit Less

BB-HEALTH

Standing more makes a difference!

- Getting your steps in is good
- Getting your moderate and vigorous activity is good
- Standing instead of sitting can take you even further

(See page 1– Sadie and Sam)

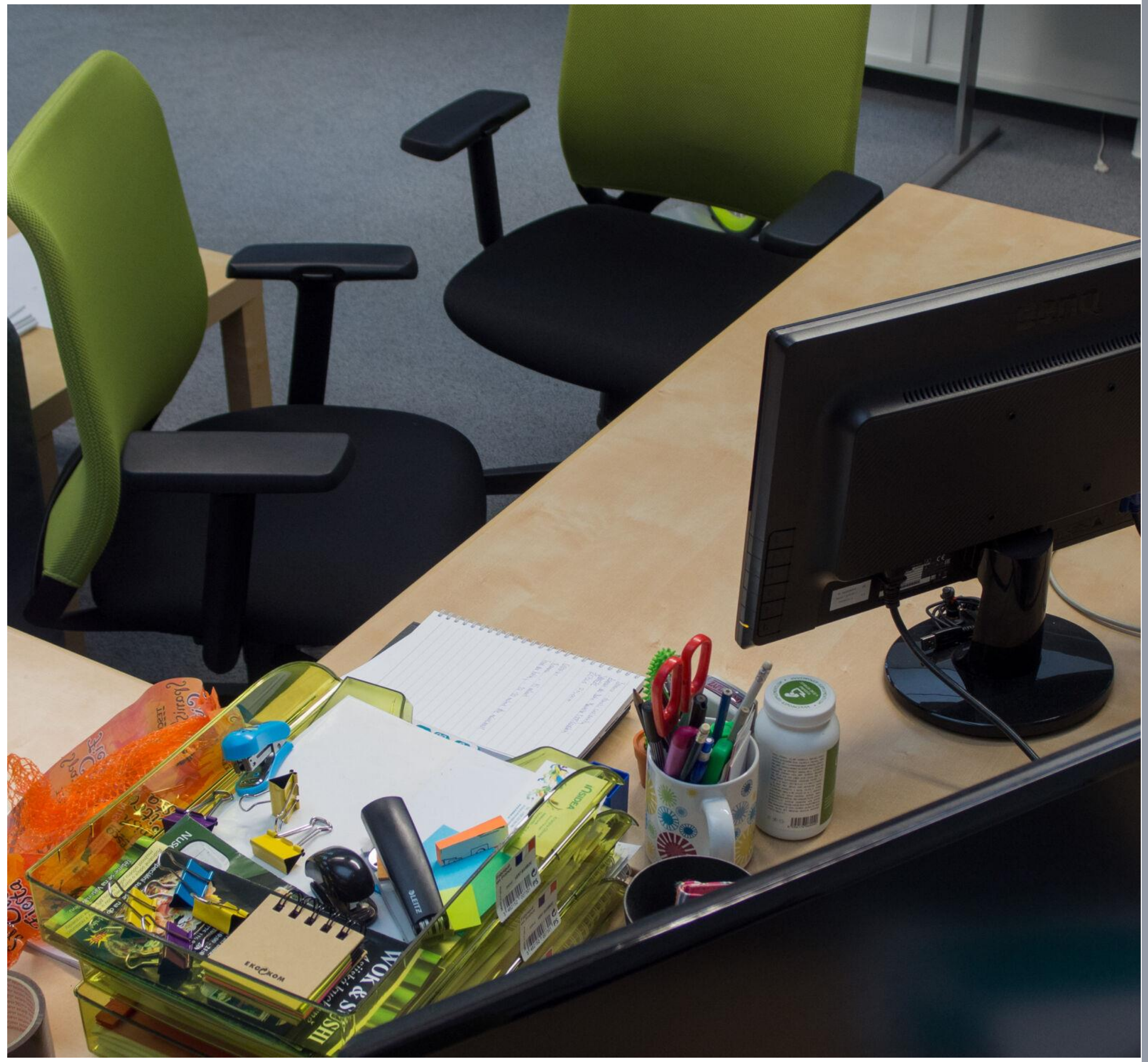

Is your day more like Sadie or more like Sam?

- ➢ Sitting is becoming the new normal (page 3)
- ➢ this month we review sedentary time from Fitbit

What would you change sit less and move more?

How to Sit Less

BB-HEALTH

Ways to help reduce sedentary time:

1. Set your Fitbit activity reminders
2. Hit those 250 steps an hour at least 9 times
3. Limit screen time to 2-3 hours a day
4. Stand or jog in place during commercials
5. Read and answer all emails standing up
6. **<u>Other (discuss, and add ideas in the chat)</u>**

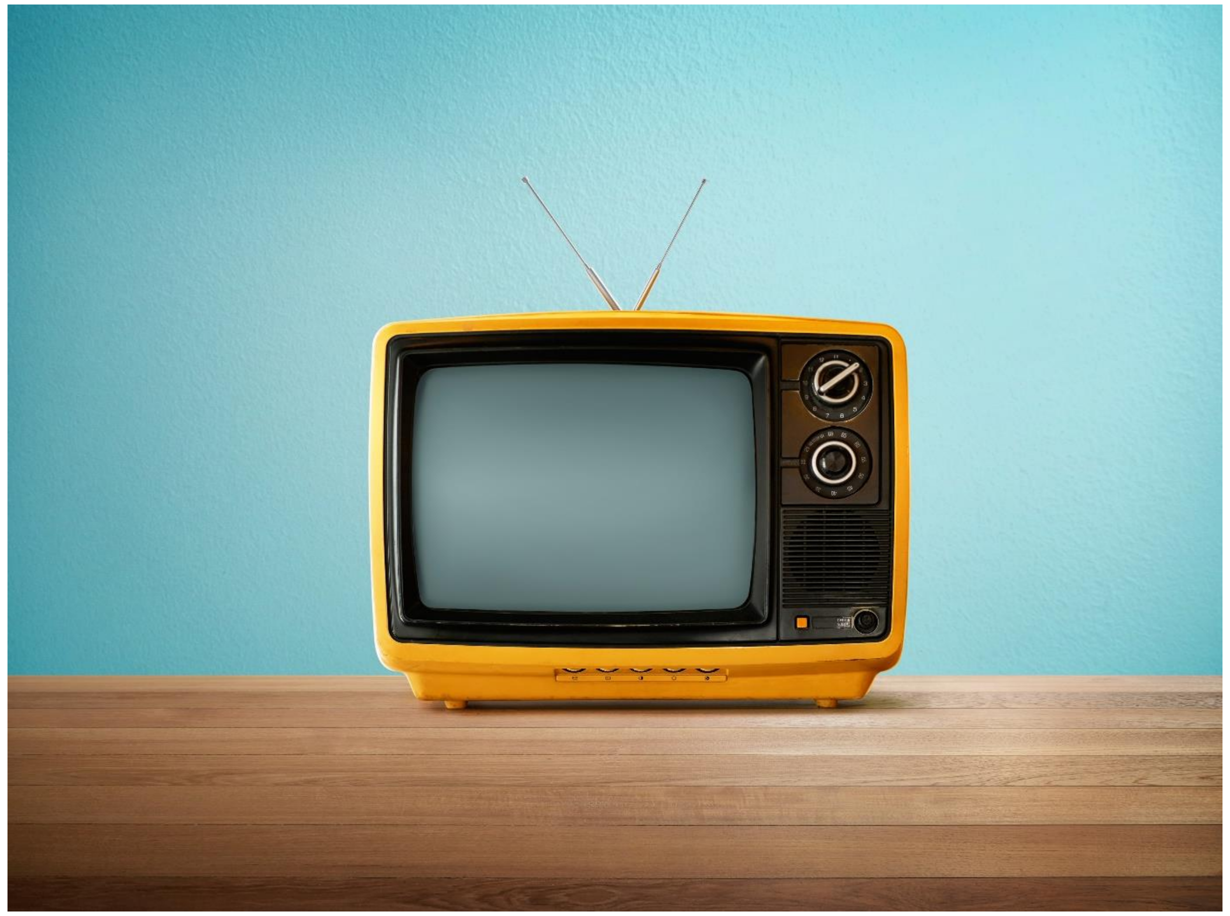

TABS & SUPER TABS

B B - H E A L T H

TABS = **T**ake **A B**reak from **S**itting

SUPER TABS= Sitting Breaks of 10 min+

1. Break up sitting with intentional standing
   - Track all 1 min TABS
   - Track all SUPER TABS in units of 10 mins
2. Be AWARE of when you are sitting a long time
3. Take CONTROL: stand up and move around
4. Identify times and places when you sit more
5. Make a plan for adding TABS and SUPER TABS

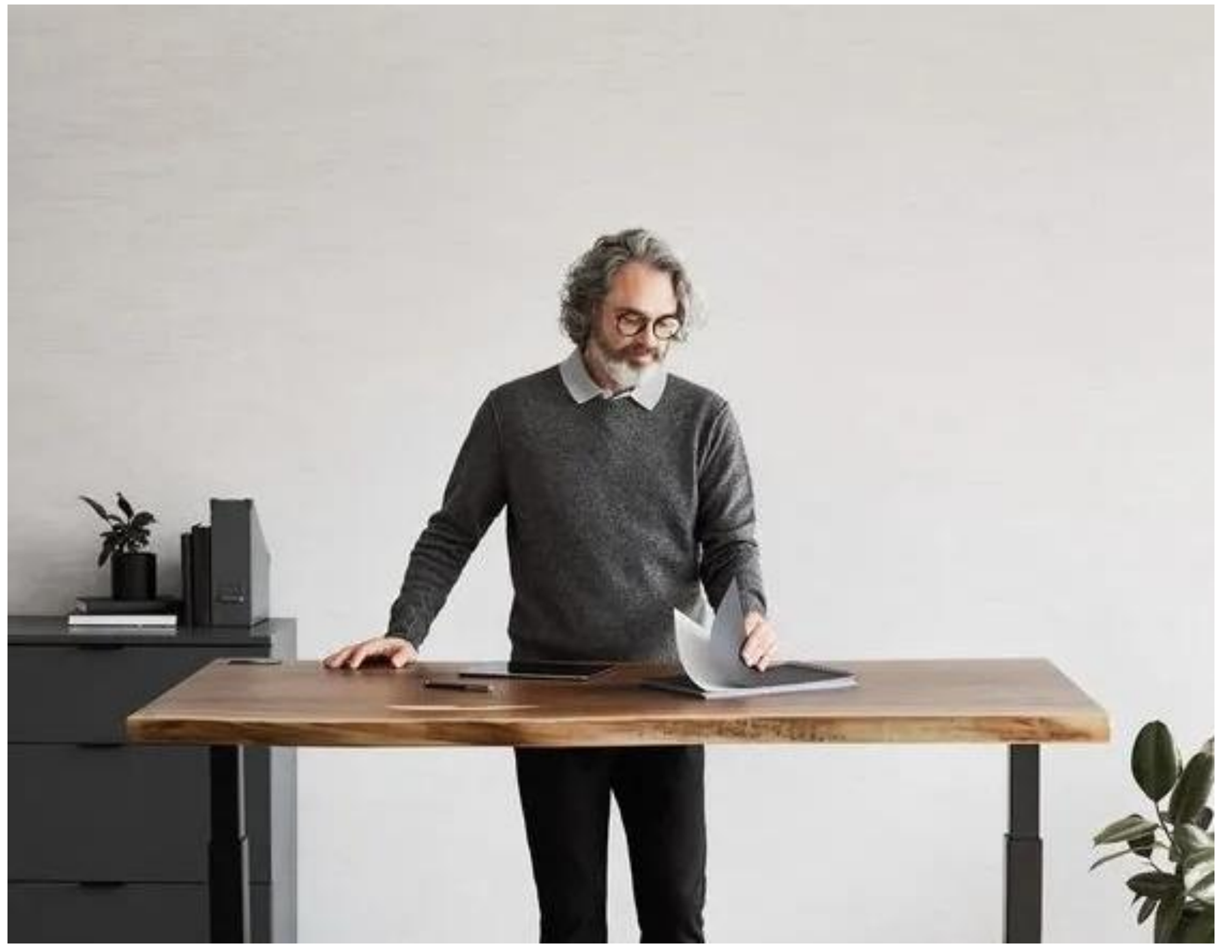

See page 6 for ideas on how to stand or move more.
See page 8 to set some TABS and SUPER TABS GOALS!
Use the Sit Less Tracker Session 16 page 9

Put it into Practice

Your Sit Less and MOVE MORE!

1. Track those meals and take your health EVERYDAY!
   - Monitor your progress and identify areas for improvement towards YOUR GOALS

2. Aim to get in 100 min of moderate or vigorous exercise ***a week*** by our next session!
   - Make your total active time over 200 min this week!
   - Keep up your steps, reduce sedentary time– get all 9!

3. Track your sitting time
   - Add standing or light walking into at least one daily routine
   - Plan out some TABS and SUPERTABS

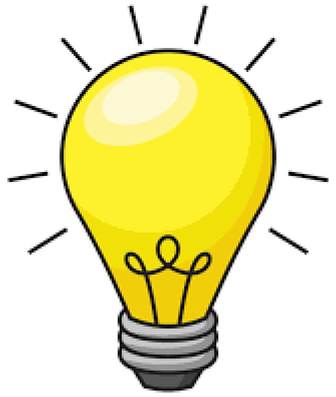

THIS MONTH'S TIP: *Make movement the new normal*
Can you dance while doing chores? Can you walk around the block while waiting on friends to show up? Why sit when you can stand, and stand when you can walk?

Questions?

BB-HEALTH

For next month:

1. Decrease your sedentary time!
2. Track your food and exercise and **Weigh Every Day**
3. Remember your calorie, fat, and activity targets
4. Email your logs and progress to the Intervention Team and Review the response!

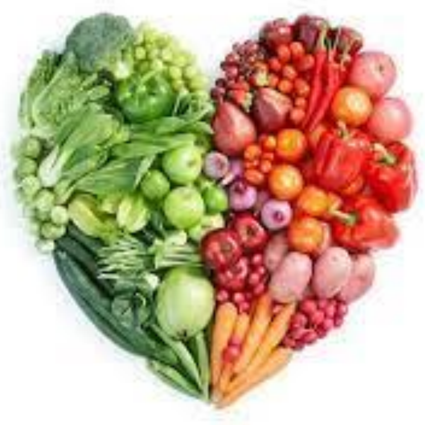

FOOD FOR THOUGHT

Let people in your life know that you are the one to keep up with now!
