# Supplementary material for: Development and Feasibility of an eHealth Diabetes Prevention Program Adapted for Older Adults—Results from a Randomized Control Pilot Study
Source: Nutrients. 2024 Mar 23;16(7):930. doi: 10.3390/nu16070930 (PMC11154527; doi:10.3390/nu16070930)
Supplement: Supplementary file 1 [file nutrients-16-00930-s001.zip › Session 21.pptx]

## Slide 1
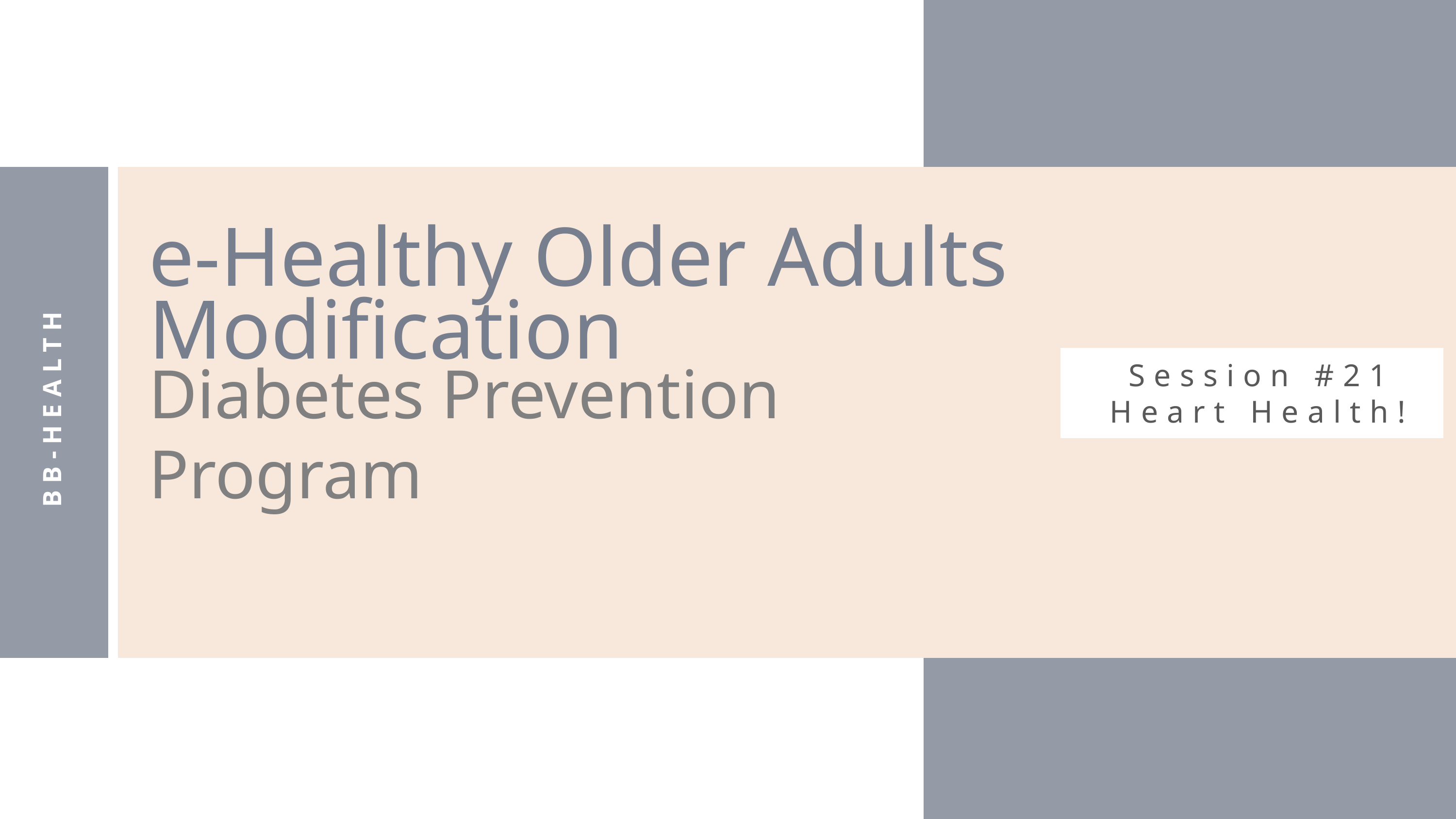

OPEN REPORTS
e-Healthy Older Adults Modification
Session #21
Heart Health!
Diabetes Prevention Program
BB-HEALTH

## Slide 2
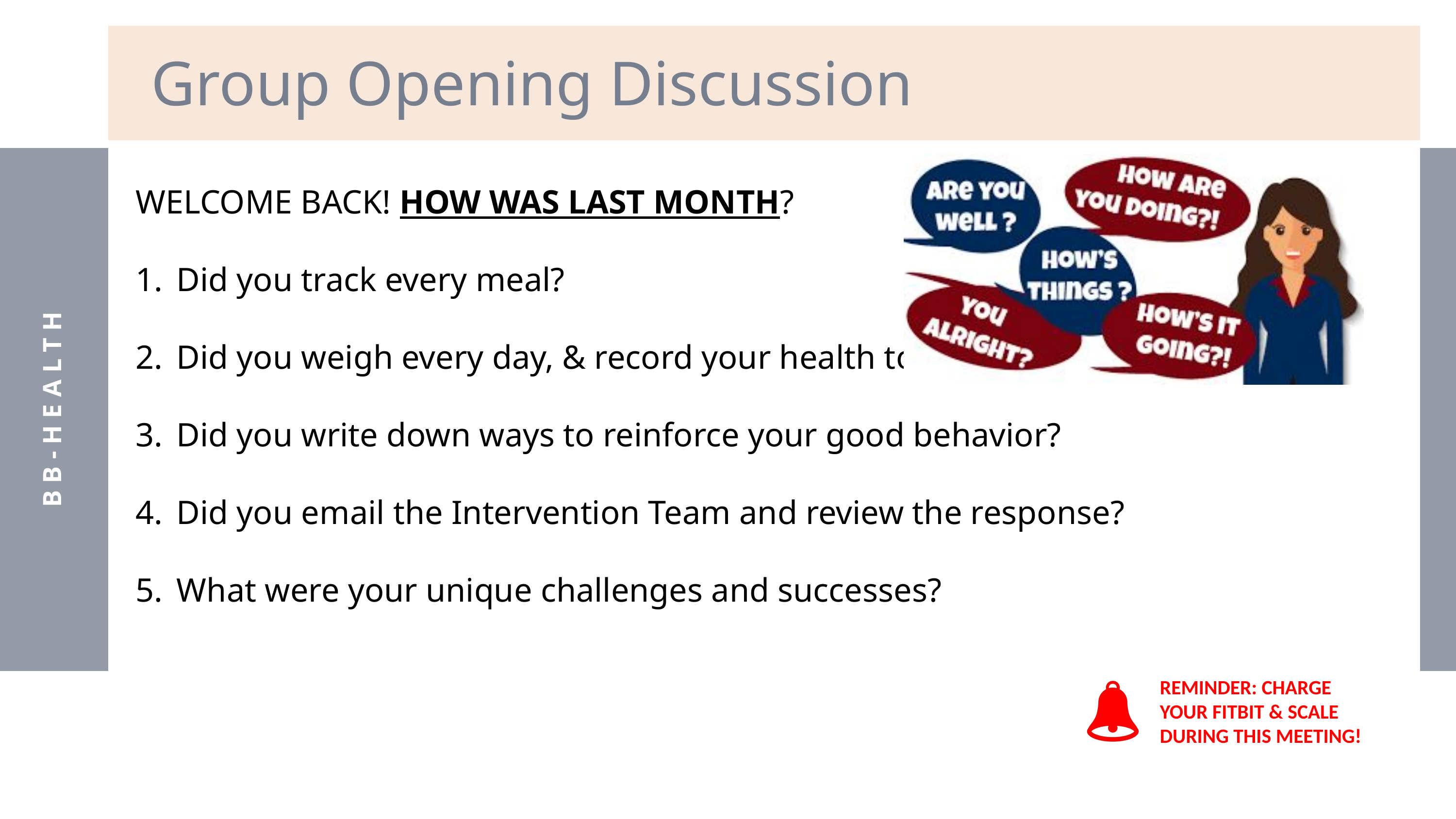

Group Opening Discussion
WELCOME BACK! HOW WAS LAST MONTH?
Did you track every meal?
Did you weigh every day, & record your health today?
Did you write down ways to reinforce your good behavior?
Did you email the Intervention Team and review the response?
What were your unique challenges and successes?
BB-HEALTH
REMINDER: CHARGE YOUR FITBIT & SCALE DURING THIS MEETING!

## Slide 3
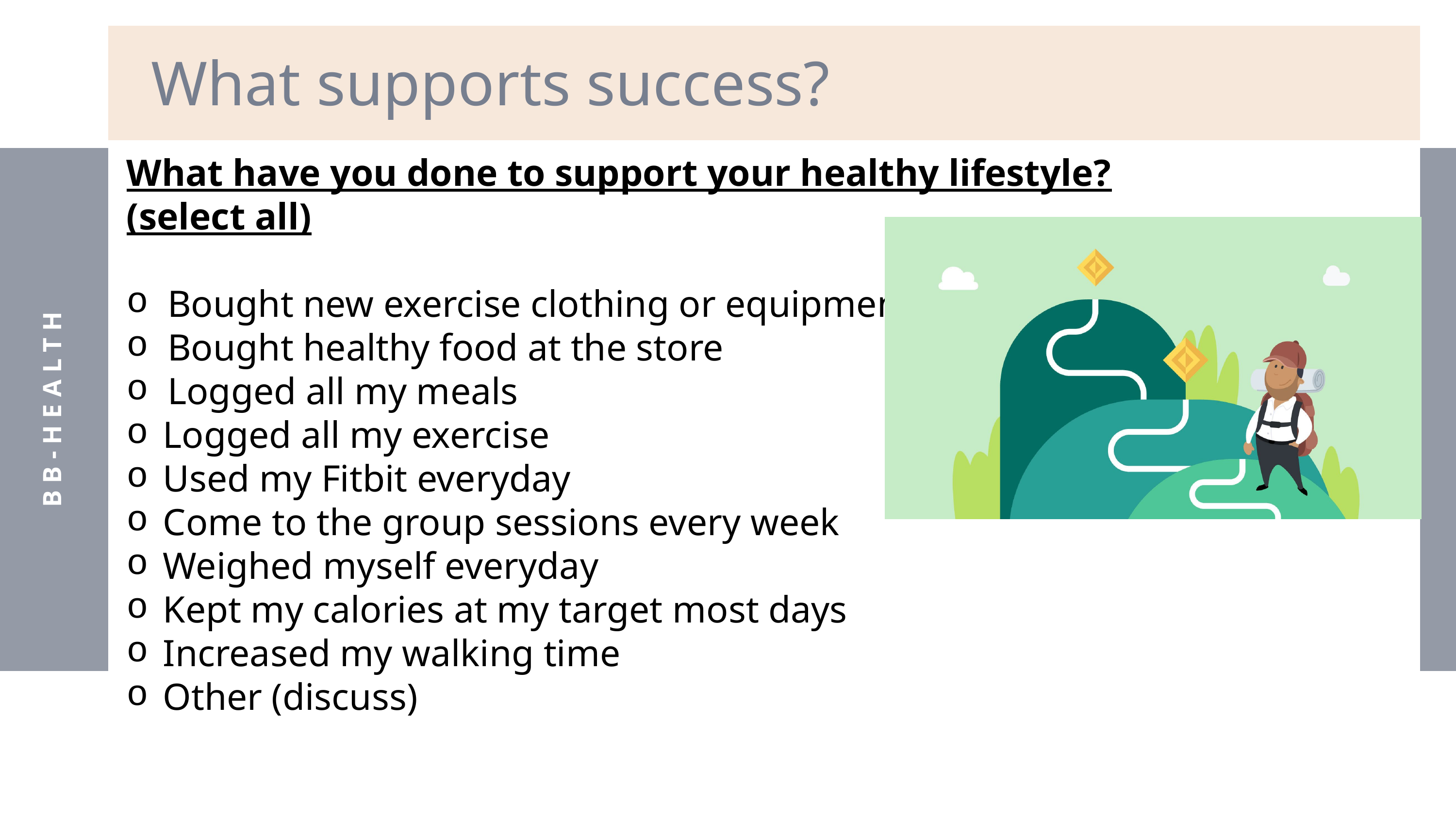

What supports success?
What have you done to support your healthy lifestyle? (select all)
Bought new exercise clothing or equipment
Bought healthy food at the store
Logged all my meals
Logged all my exercise
Used my Fitbit everyday
Come to the group sessions every week
Weighed myself everyday
Kept my calories at my target most days
Increased my walking time
Other (discuss)
BB-HEALTH

## Slide 4
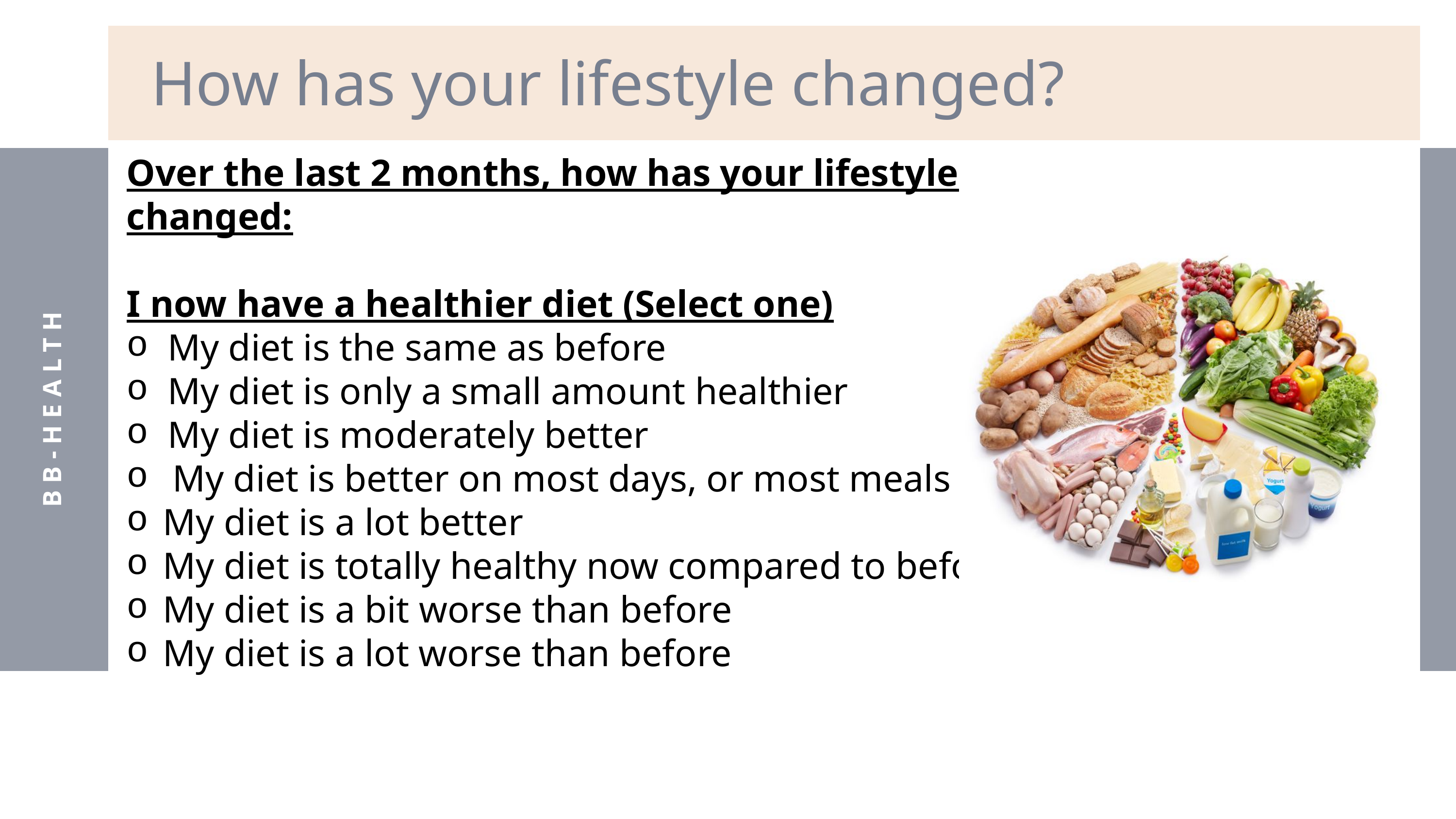

How has your lifestyle changed?
Over the last 2 months, how has your lifestyle changed:
I now have a healthier diet (Select one)
My diet is the same as before
My diet is only a small amount healthier
My diet is moderately better
 My diet is better on most days, or most meals
My diet is a lot better
My diet is totally healthy now compared to before
My diet is a bit worse than before
My diet is a lot worse than before
BB-HEALTH

## Slide 5
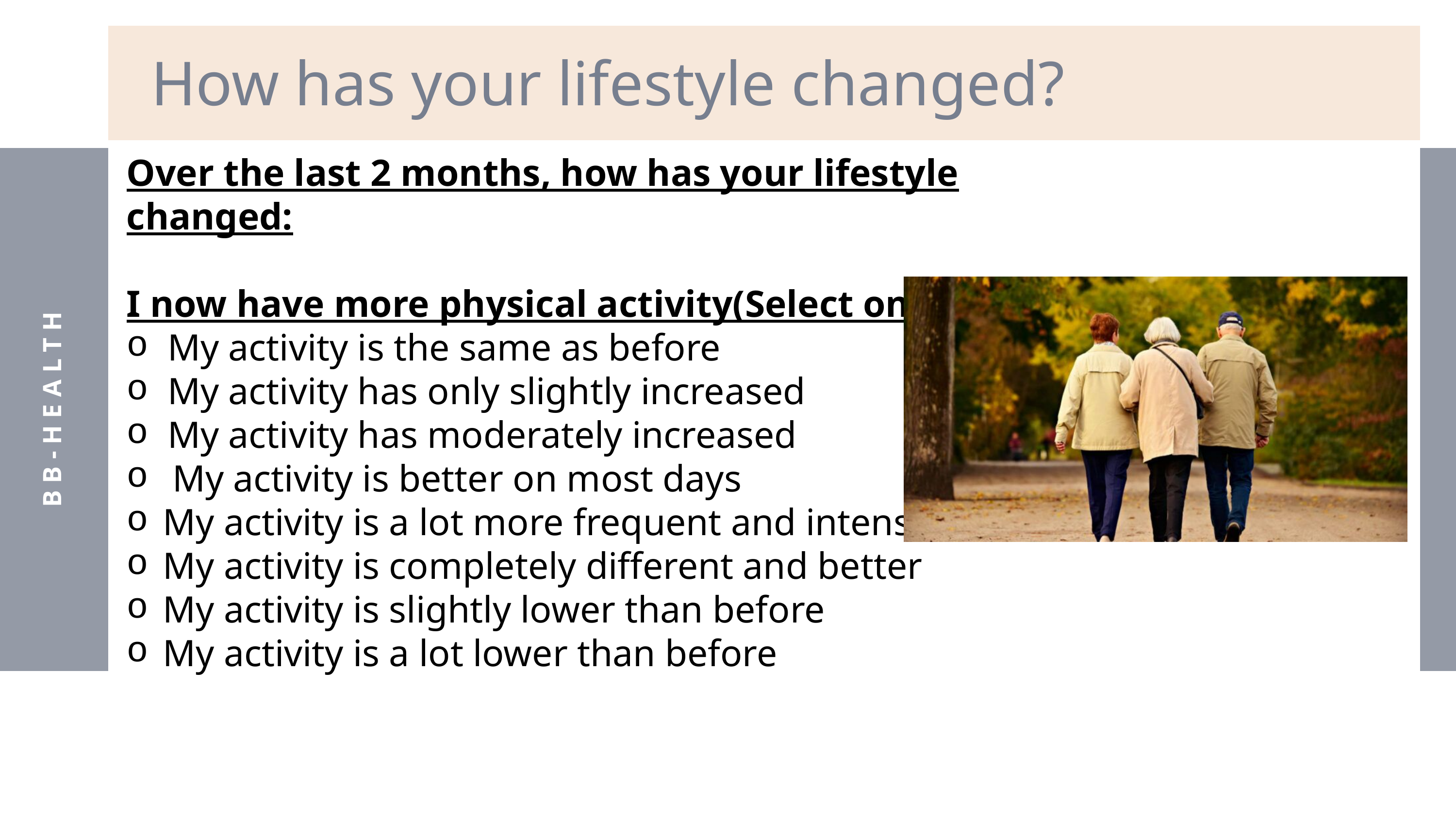

How has your lifestyle changed?
Over the last 2 months, how has your lifestyle changed:
I now have more physical activity(Select one)
My activity is the same as before
My activity has only slightly increased
My activity has moderately increased
 My activity is better on most days
My activity is a lot more frequent and intensive
My activity is completely different and better
My activity is slightly lower than before
My activity is a lot lower than before
BB-HEALTH

## Slide 6
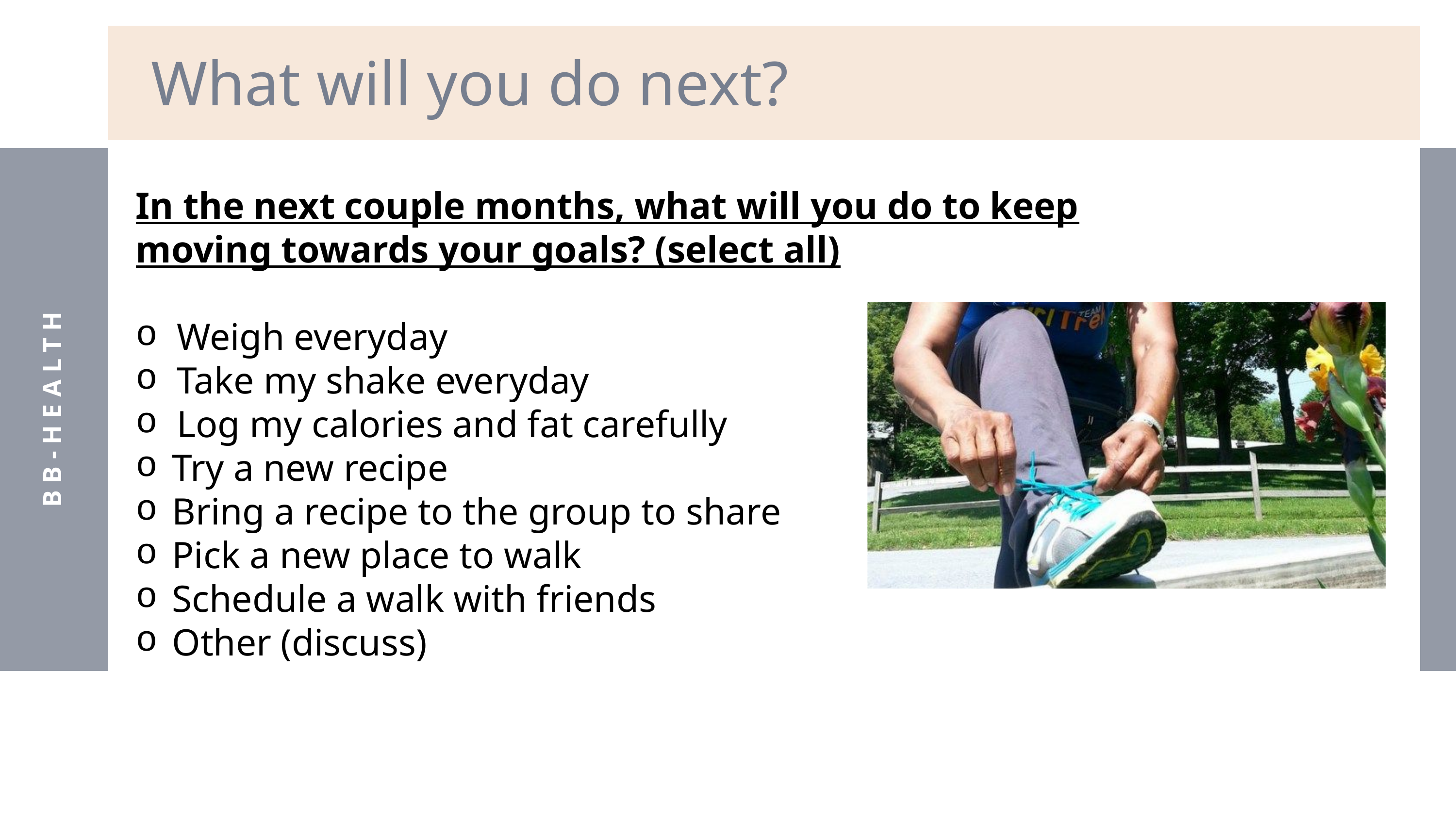

What will you do next?
In the next couple months, what will you do to keep moving towards your goals? (select all)
Weigh everyday
Take my shake everyday
Log my calories and fat carefully
Try a new recipe
Bring a recipe to the group to share
Pick a new place to walk
Schedule a walk with friends
Other (discuss)
BB-HEALTH

## Slide 7
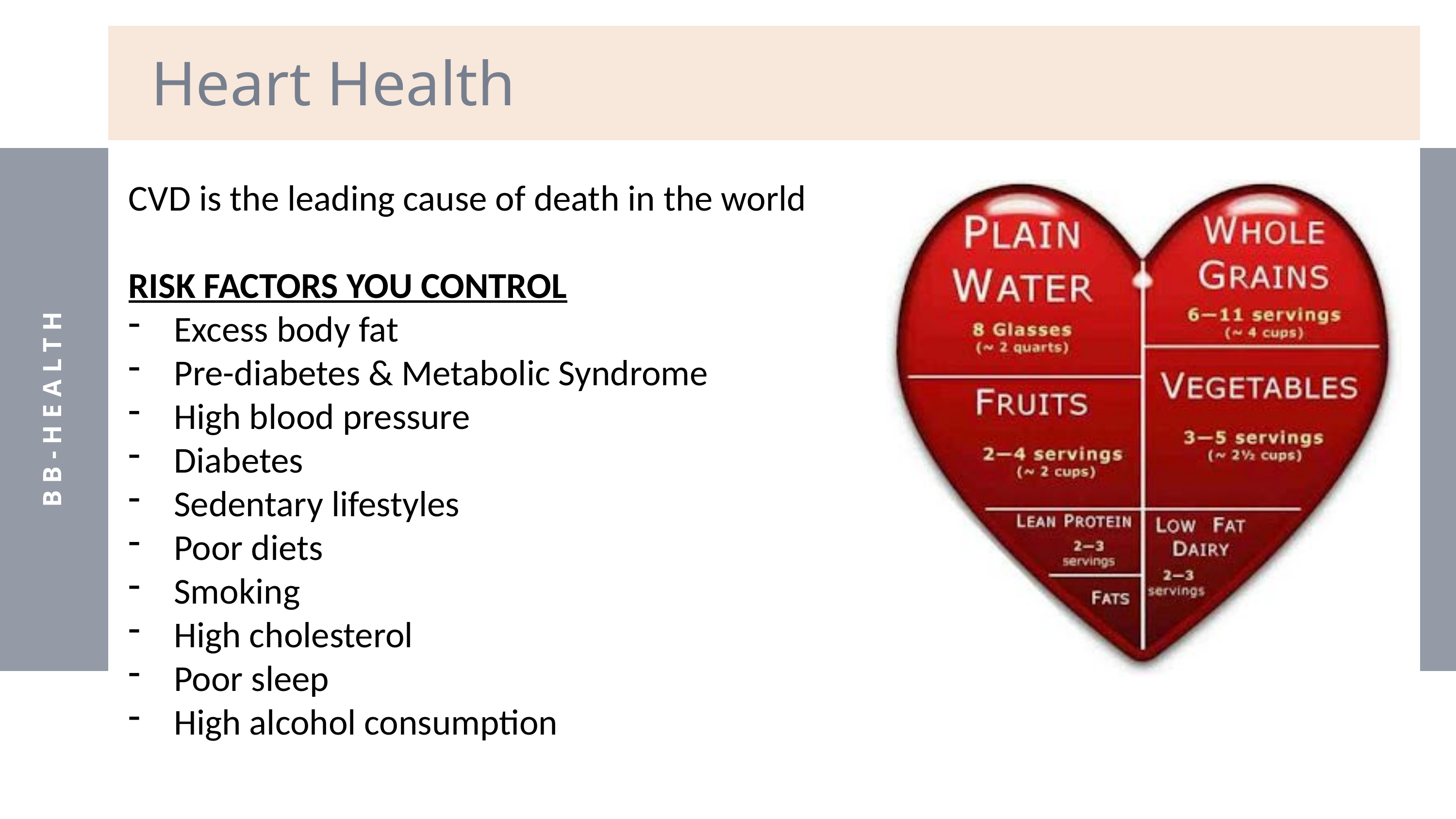

Heart Health
CVD is the leading cause of death in the world
RISK FACTORS YOU CONTROL
Excess body fat
Pre-diabetes & Metabolic Syndrome
High blood pressure
Diabetes
Sedentary lifestyles
Poor diets
Smoking
High cholesterol
Poor sleep
High alcohol consumption
BB-HEALTH

## Slide 8
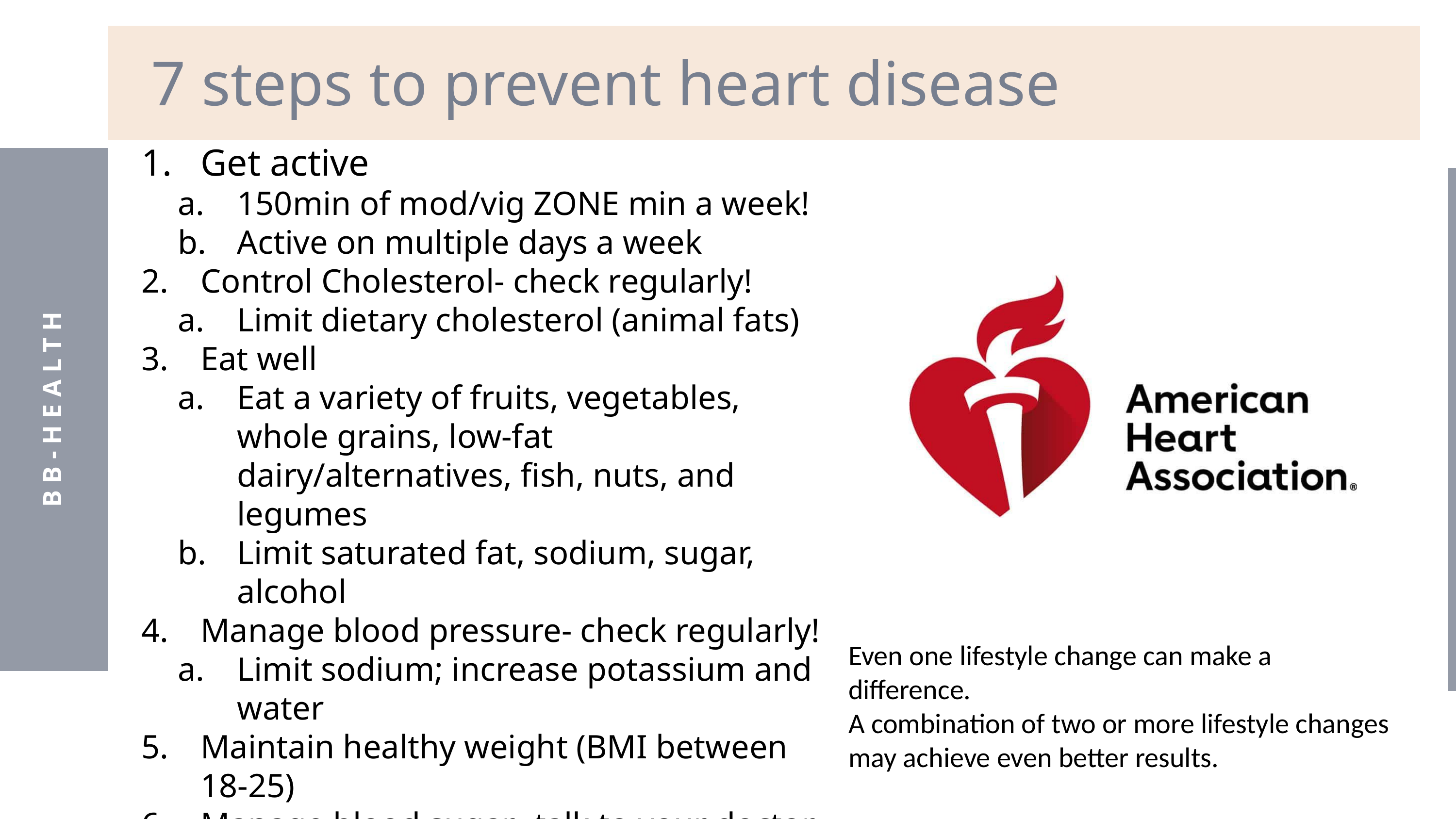

7 steps to prevent heart disease
Get active
150min of mod/vig ZONE min a week!
Active on multiple days a week
Control Cholesterol- check regularly!
Limit dietary cholesterol (animal fats)
Eat well
Eat a variety of fruits, vegetables, whole grains, low-fat dairy/alternatives, fish, nuts, and legumes
Limit saturated fat, sodium, sugar, alcohol
Manage blood pressure- check regularly!
Limit sodium; increase potassium and water
Maintain healthy weight (BMI between 18-25)
Manage blood sugar- talk to your doctor
Don’t smoke, avoid secondhand smoke
BB-HEALTH
Even one lifestyle change can make a difference.
A combination of two or more lifestyle changes may achieve even better results.

## Slide 9
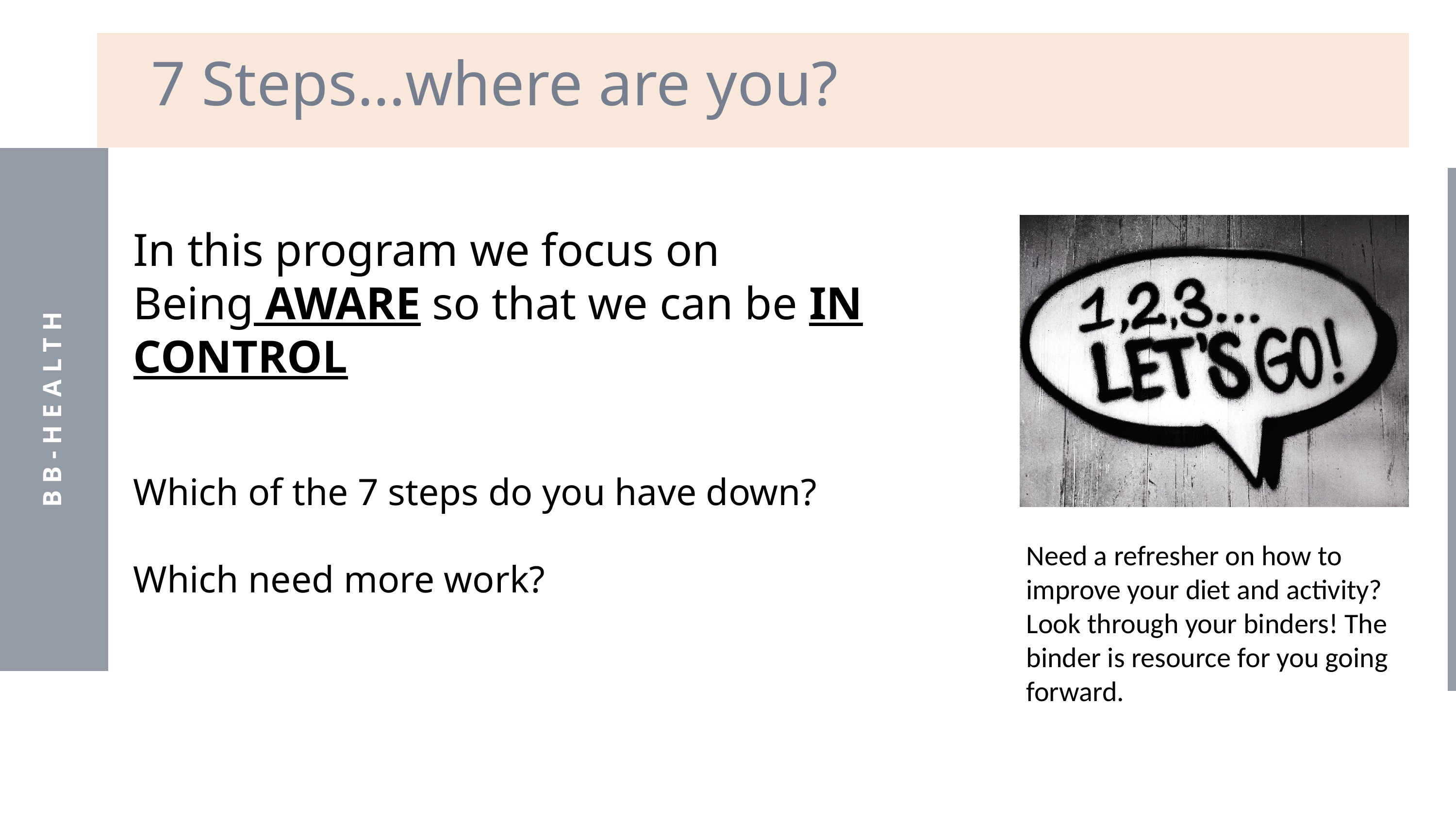

7 Steps…where are you?
In this program we focus on
Being AWARE so that we can be IN CONTROL
Which of the 7 steps do you have down?
Which need more work?
BB-HEALTH
Need a refresher on how to improve your diet and activity? Look through your binders! The binder is resource for you going forward.

## Slide 10
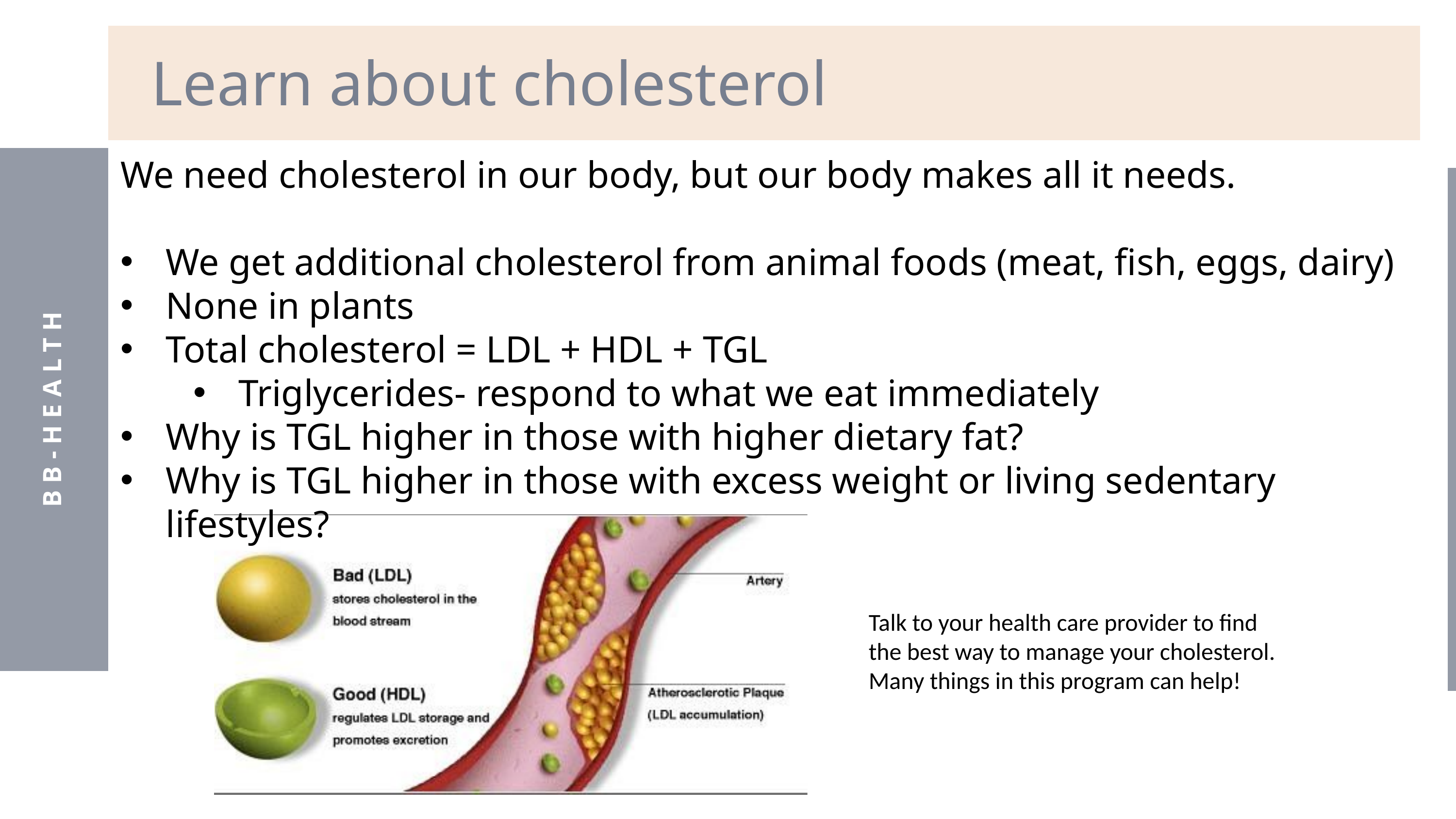

Learn about cholesterol
We need cholesterol in our body, but our body makes all it needs.
We get additional cholesterol from animal foods (meat, fish, eggs, dairy)
None in plants
Total cholesterol = LDL + HDL + TGL
Triglycerides- respond to what we eat immediately
Why is TGL higher in those with higher dietary fat?
Why is TGL higher in those with excess weight or living sedentary lifestyles?
BB-HEALTH
Talk to your health care provider to find the best way to manage your cholesterol. Many things in this program can help!

## Slide 11
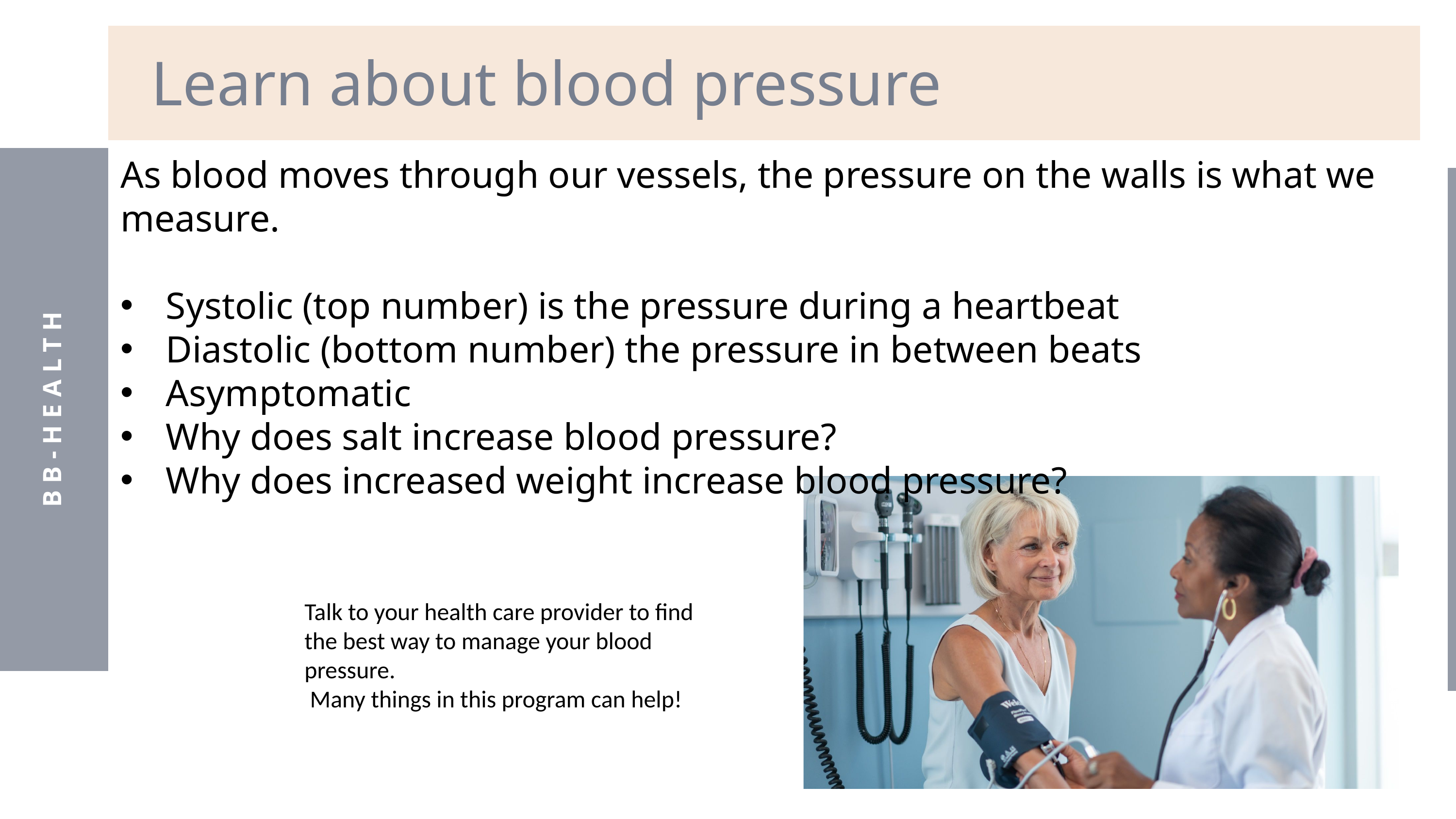

Learn about blood pressure
As blood moves through our vessels, the pressure on the walls is what we measure.
Systolic (top number) is the pressure during a heartbeat
Diastolic (bottom number) the pressure in between beats
Asymptomatic
Why does salt increase blood pressure?
Why does increased weight increase blood pressure?
BB-HEALTH
Talk to your health care provider to find the best way to manage your blood pressure.
 Many things in this program can help!

## Slide 12
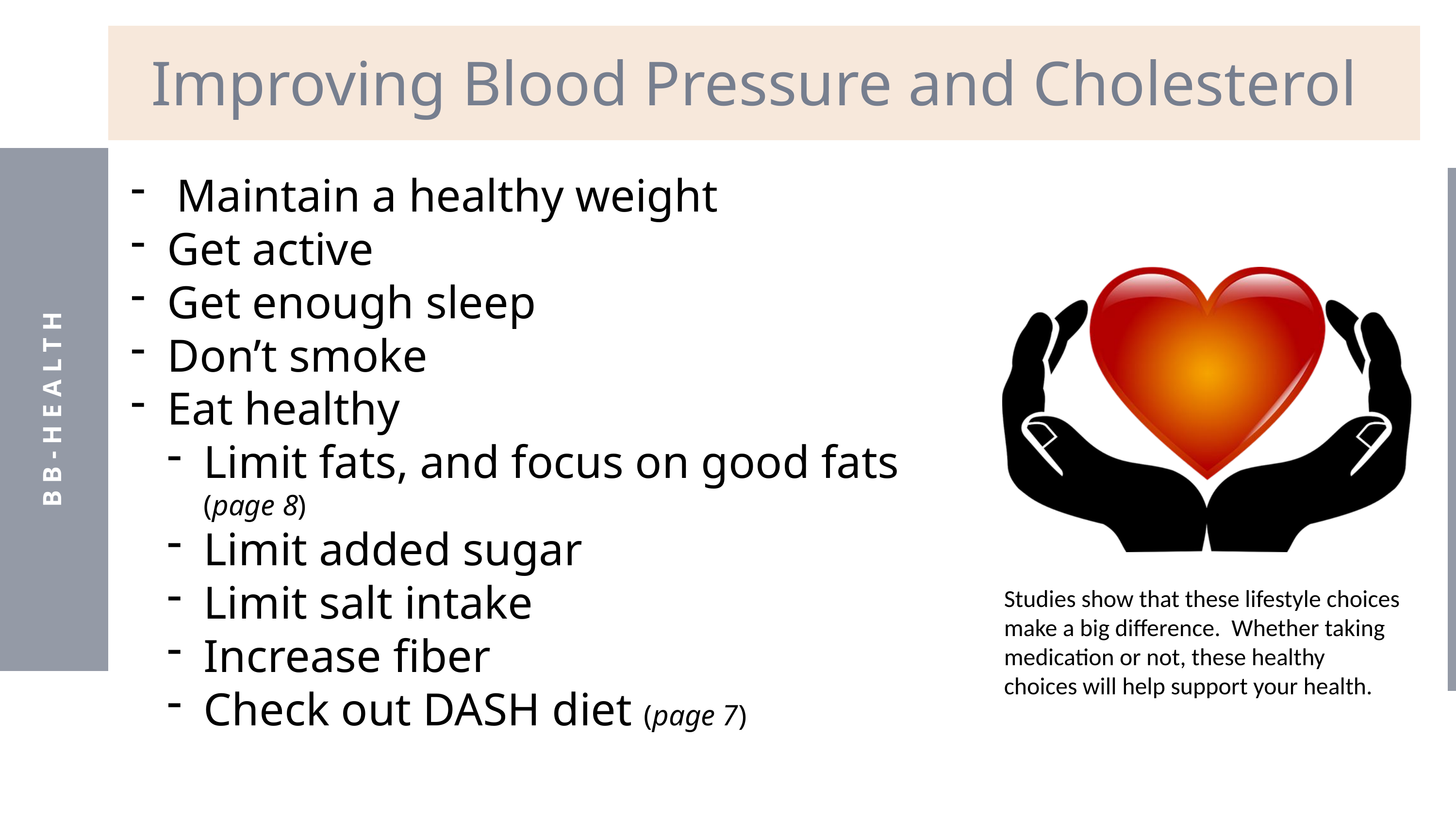

Improving Blood Pressure and Cholesterol
Maintain a healthy weight
Get active
Get enough sleep
Don’t smoke
Eat healthy
Limit fats, and focus on good fats (page 8)
Limit added sugar
Limit salt intake
Increase fiber
Check out DASH diet (page 7)
BB-HEALTH
Studies show that these lifestyle choices make a big difference. Whether taking medication or not, these healthy choices will help support your health.

## Slide 13
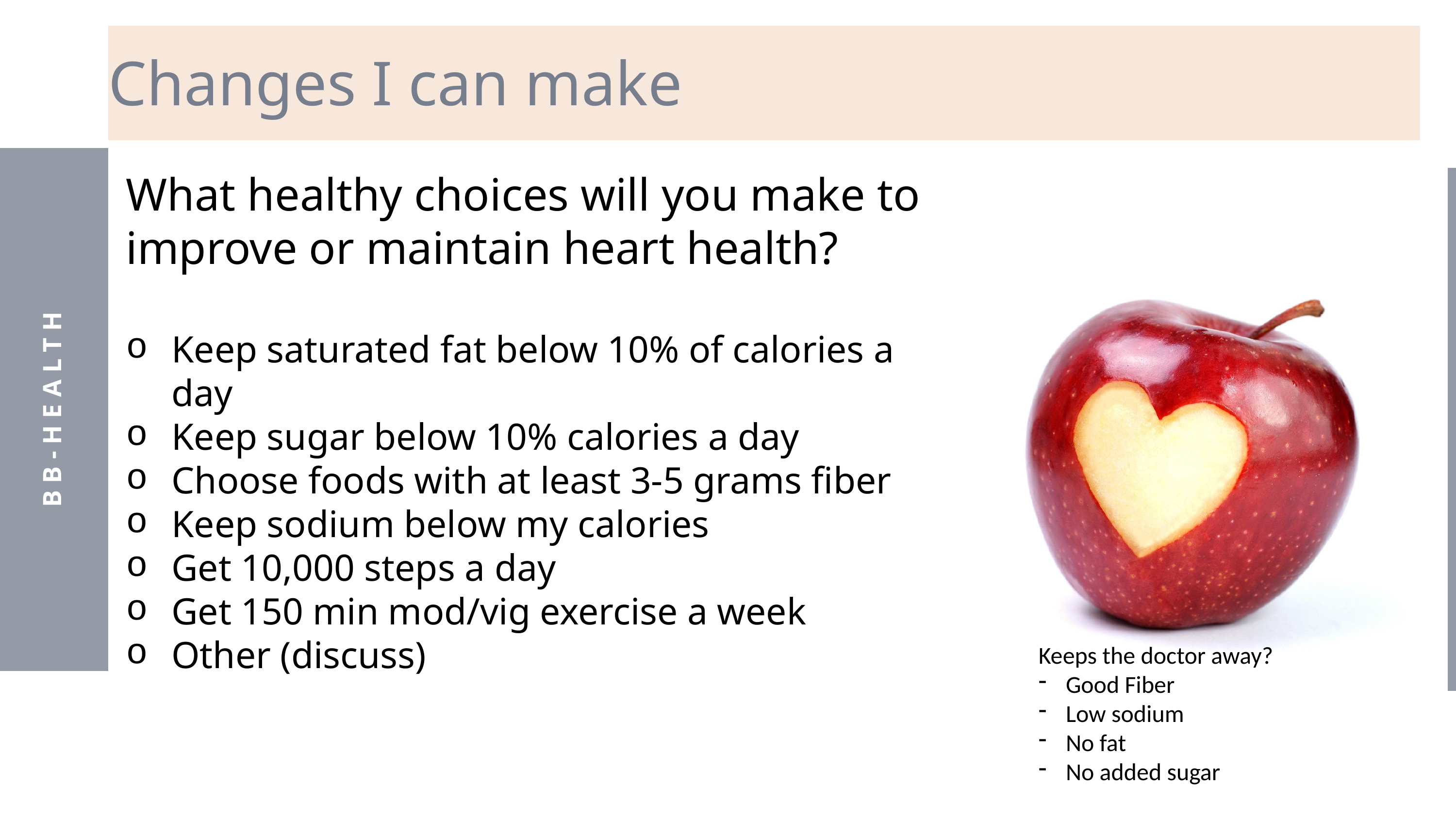

Changes I can make
What healthy choices will you make to improve or maintain heart health?
Keep saturated fat below 10% of calories a day
Keep sugar below 10% calories a day
Choose foods with at least 3-5 grams fiber
Keep sodium below my calories
Get 10,000 steps a day
Get 150 min mod/vig exercise a week
Other (discuss)
BB-HEALTH
Keeps the doctor away?
Good Fiber
Low sodium
No fat
No added sugar

## Slide 14
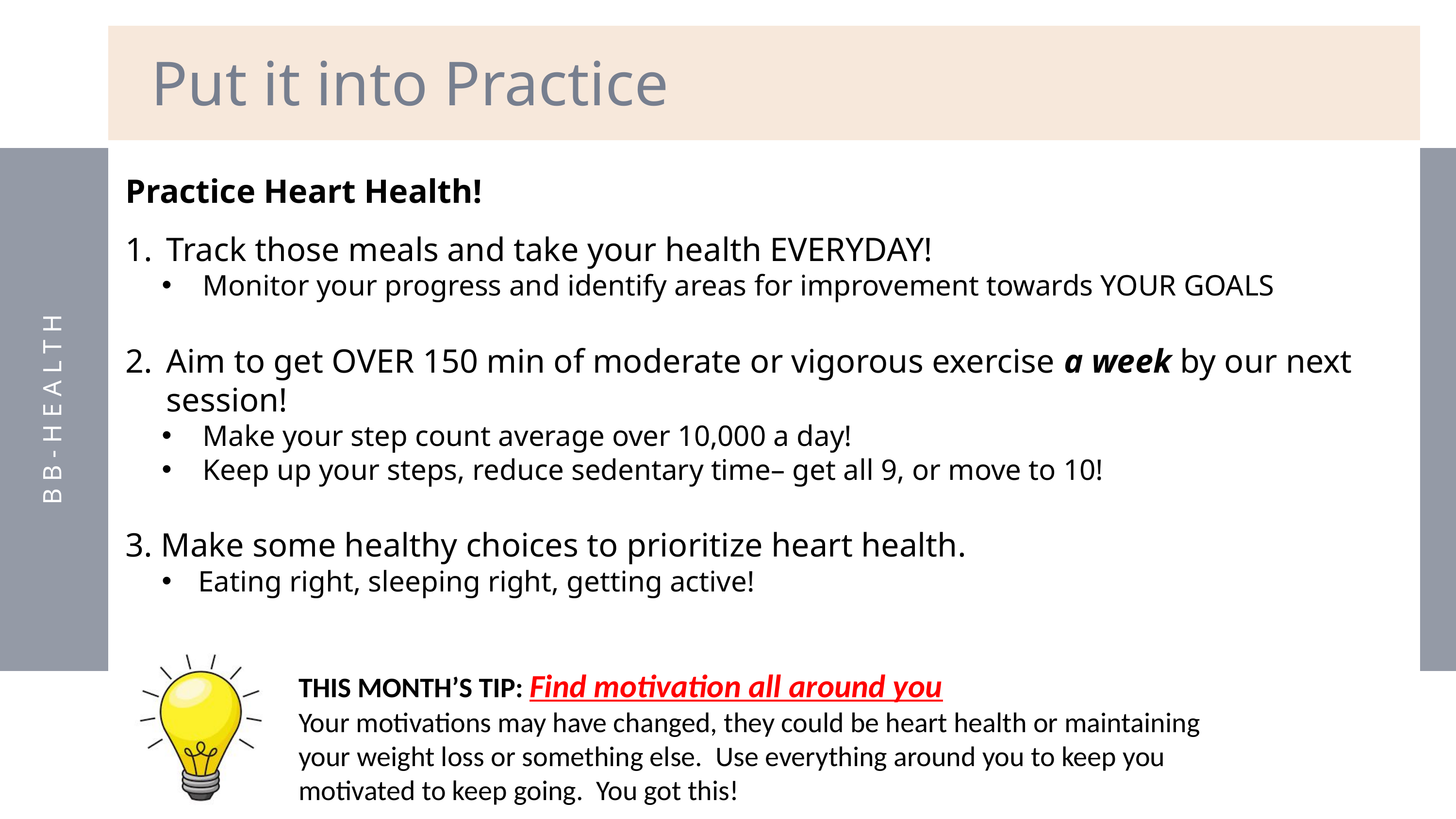

Put it into Practice
Practice Heart Health!
Track those meals and take your health EVERYDAY!
Monitor your progress and identify areas for improvement towards YOUR GOALS
Aim to get OVER 150 min of moderate or vigorous exercise a week by our next session!
Make your step count average over 10,000 a day!
Keep up your steps, reduce sedentary time– get all 9, or move to 10!
3. Make some healthy choices to prioritize heart health.
Eating right, sleeping right, getting active!
BB-HEALTH
THIS MONTH’S TIP: Find motivation all around you
Your motivations may have changed, they could be heart health or maintaining your weight loss or something else. Use everything around you to keep you motivated to keep going. You got this!

## Slide 15
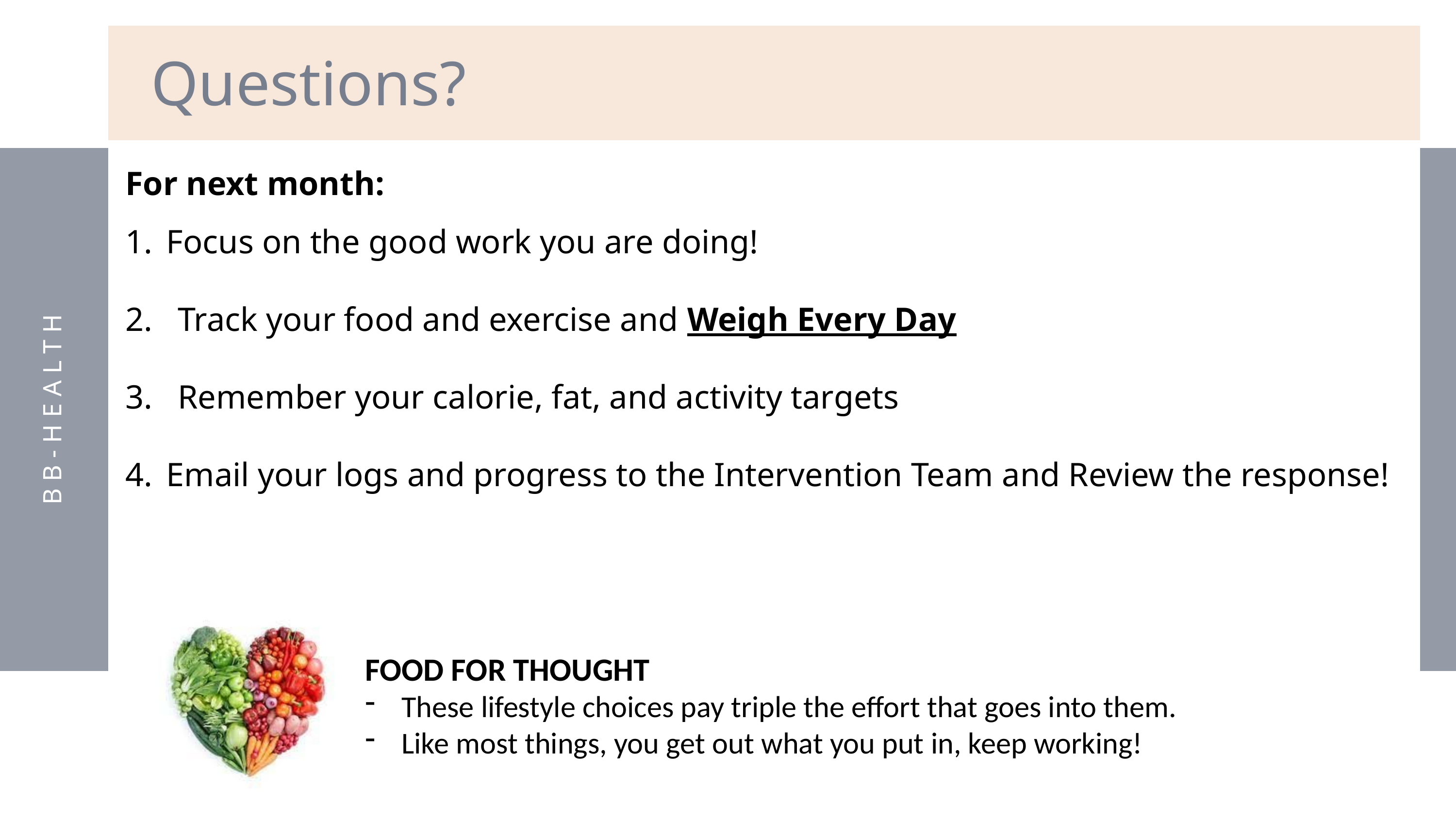

Questions?
For next month:
Focus on the good work you are doing!
2. Track your food and exercise and Weigh Every Day
3. Remember your calorie, fat, and activity targets
Email your logs and progress to the Intervention Team and Review the response!
BB-HEALTH
FOOD FOR THOUGHT
These lifestyle choices pay triple the effort that goes into them.
Like most things, you get out what you put in, keep working!
