# Supplementary material for: Development and Feasibility of an eHealth Diabetes Prevention Program Adapted for Older Adults—Results from a Randomized Control Pilot Study
Source: Nutrients. 2024 Mar 23;16(7):930. doi: 10.3390/nu16070930 (PMC11154527; doi:10.3390/nu16070930)
Supplement: Supplementary file 1 [file nutrients-16-00930-s001.zip › Session 21_SUPP.pdf]

BB-HEALTH

# e-Healthy Older Adults Modification

## Diabetes Prevention Program

**Session #21**
Heart Health!

# Group Opening Discussion

BB-HEALTH

WELCOME BACK! <u>**HOW WAS LAST MONTH**</u>?

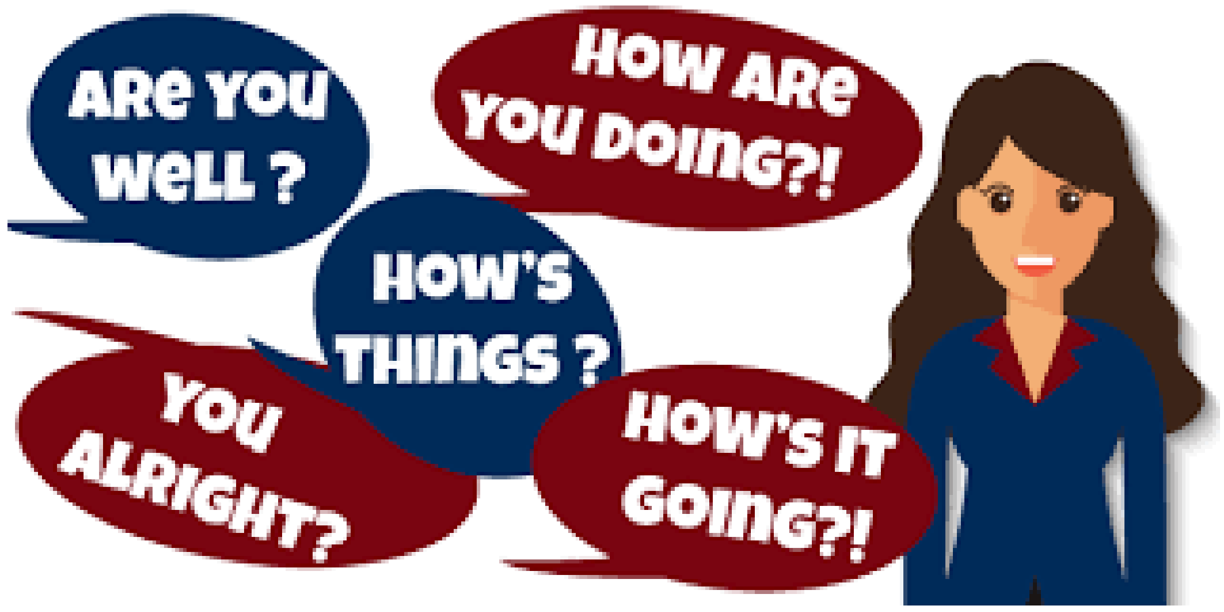


1. Did you track every meal?
2. Did you weigh every day, & record your health today?
3. Did you write down ways to reinforce your good behavior?
4. Did you email the Intervention Team and review the response?
5. What were your unique challenges and successes?

**REMINDER: CHARGE YOUR FITBIT & SCALE DURING THIS MEETING!**

# What supports success?

BB-HEALTH

**What have you done to support your healthy lifestyle? (select all)**

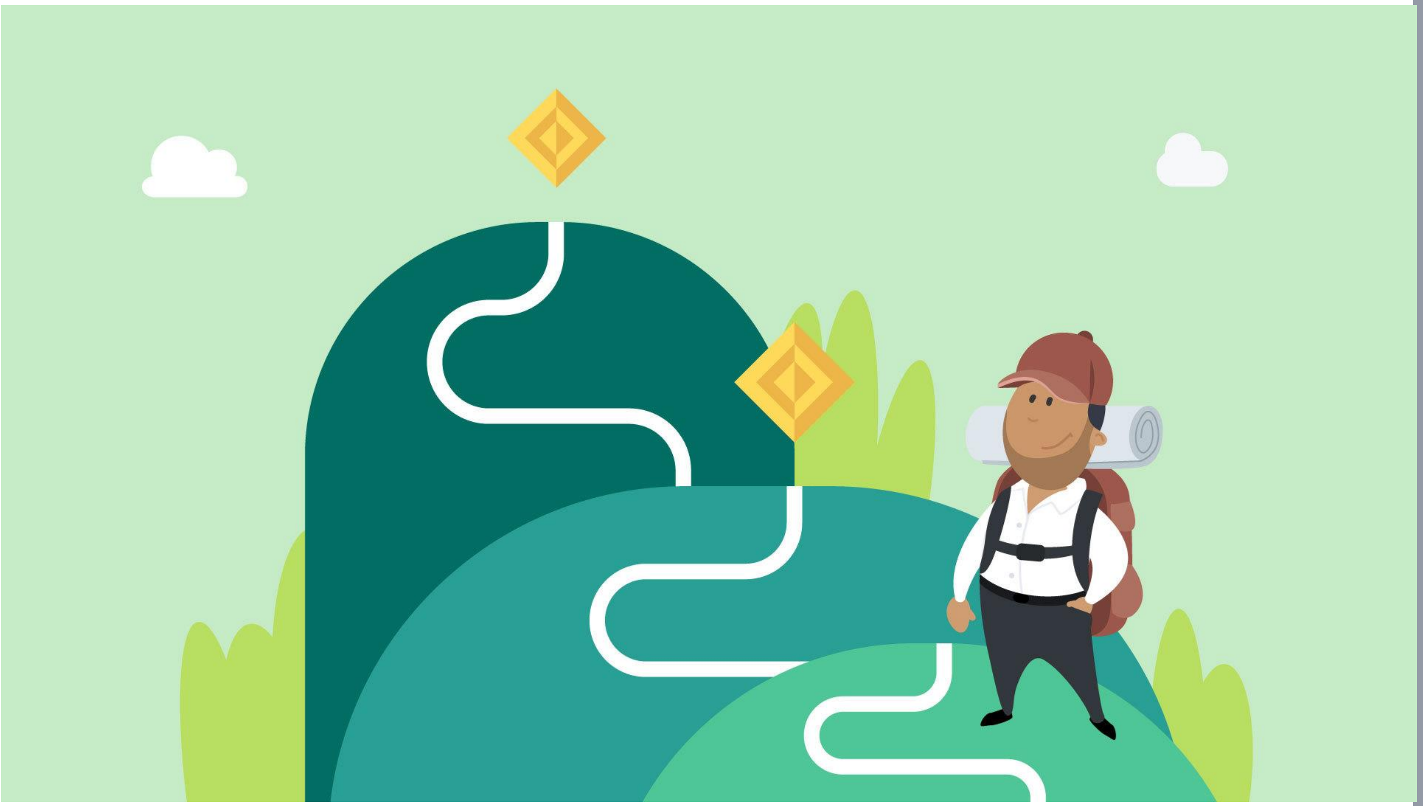

- Bought new exercise clothing or equipment
- Bought healthy food at the store
- Logged all my meals
- Logged all my exercise
- Used my Fitbit everyday
- Come to the group sessions every week
- Weighed myself everyday
- Kept my calories at my target most days
- Increased my walking time
- Other (discuss)

# How has your lifestyle changed?



**Over the last 2 months, how has your lifestyle changed:**

**I now have a healthier diet (Select one)**

- My diet is the same as before
- My diet is only a small amount healthier
- My diet is moderately better
- My diet is better on most days, or most meals
- My diet is a lot better
- My diet is totally healthy now compared to before
- My diet is a bit worse than before
- My diet is a lot worse than before

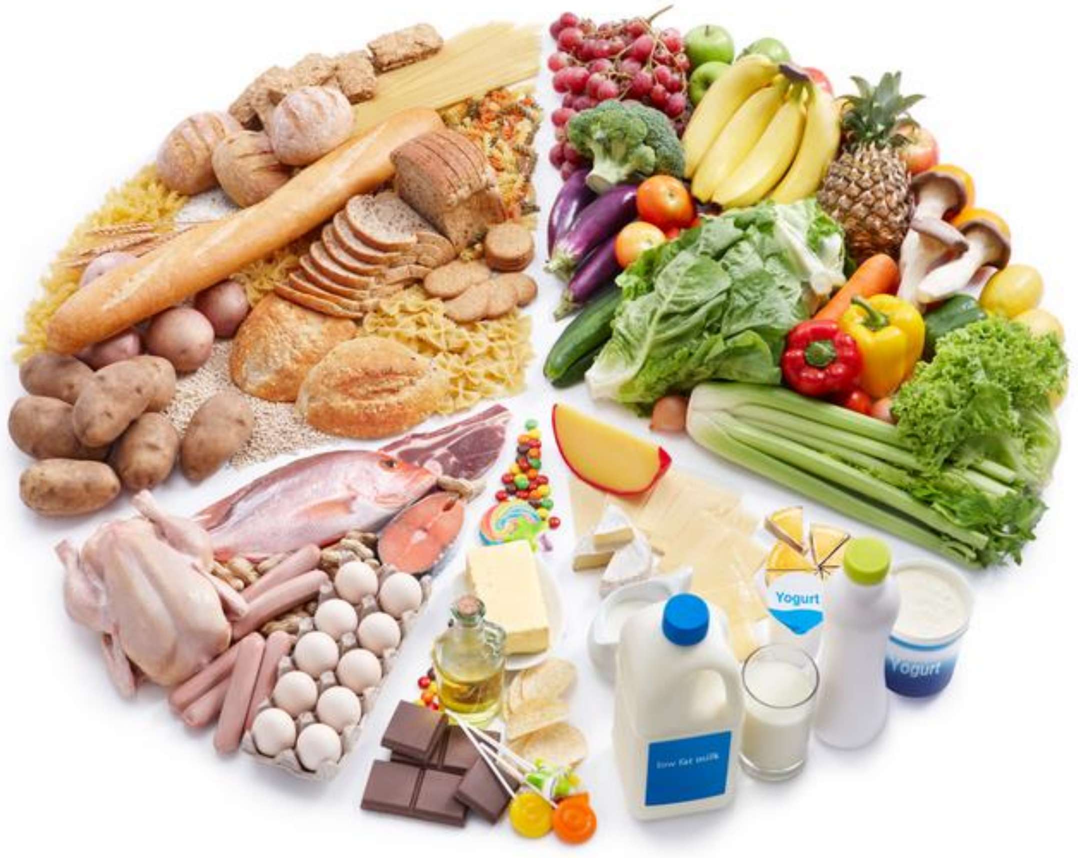

# How has your lifestyle changed?



**Over the last 2 months, how has your lifestyle changed:**

**I now have more physical activity(Select one)**

- o My activity is the same as before
- o My activity has only slightly increased
- o My activity has moderately increased
- o My activity is better on most days
- o My activity is a lot more frequent and intensive
- o My activity is completely different and better
- o My activity is slightly lower than before
- o My activity is a lot lower than before

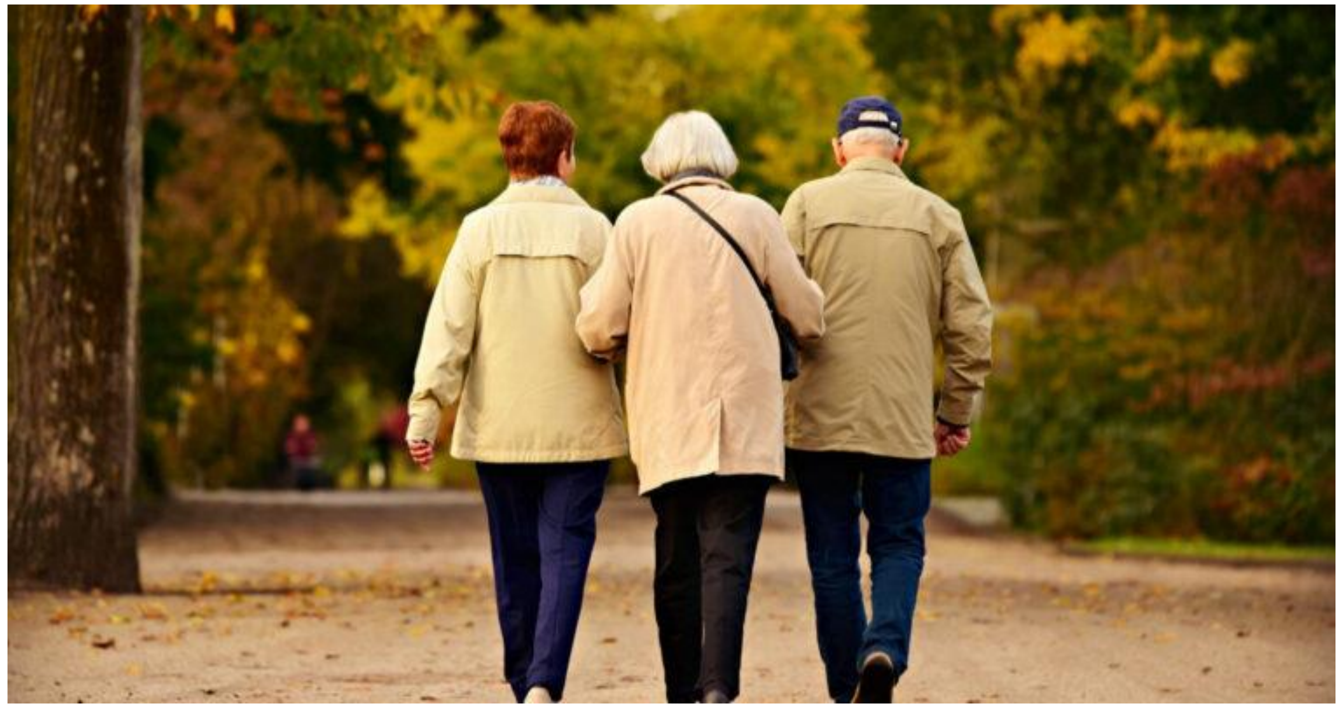

# What will you do next?

BB-HEALTH

<u>In the next couple months, what will you do to keep moving towards your goals? (select all)</u>

- Weigh everyday
- Take my shake everyday
- Log my calories and fat carefully
- Try a new recipe
- Bring a recipe to the group to share
- Pick a new place to walk
- Schedule a walk with friends
- Other (discuss)

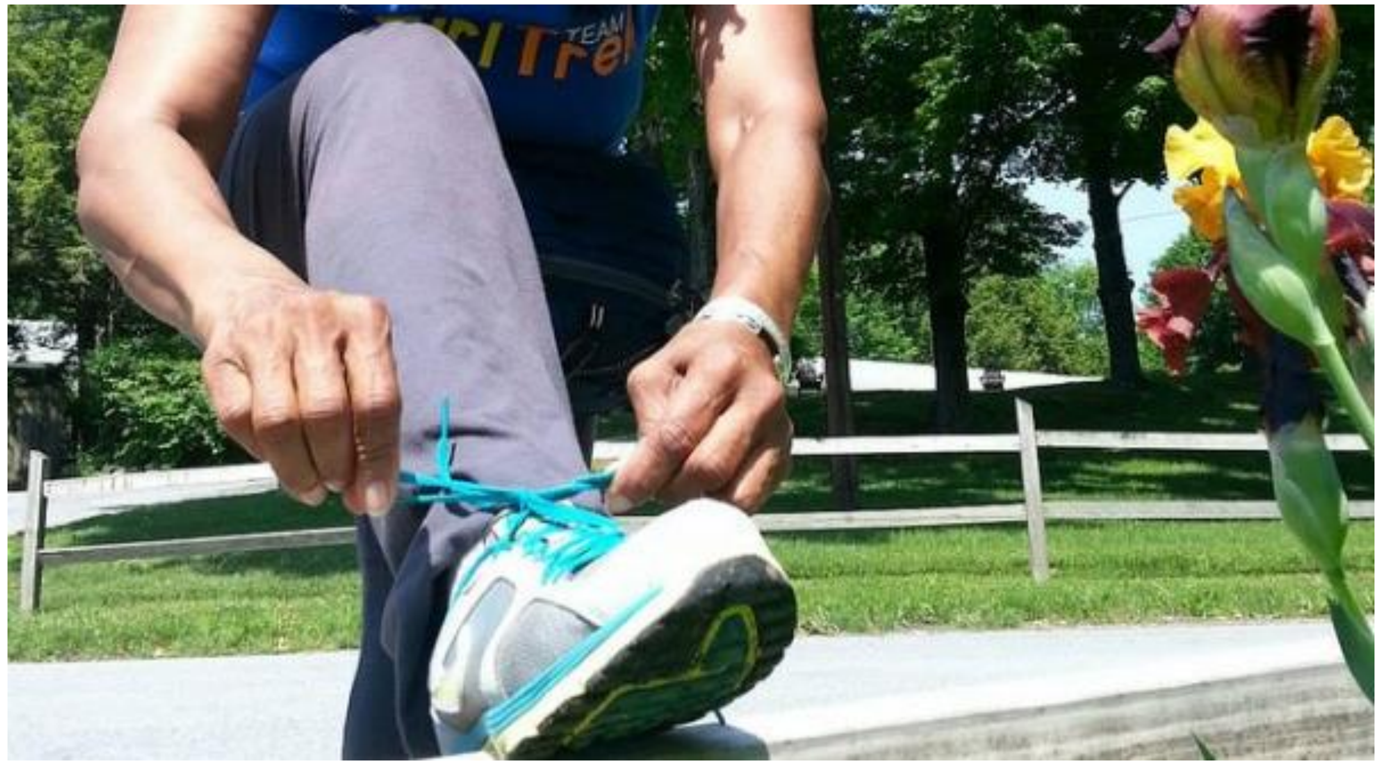

# Heart Health



CVD is the leading cause of death in the world

**RISK FACTORS YOU CONTROL**

- Excess body fat
- Pre-diabetes & Metabolic Syndrome
- High blood pressure
- Diabetes
- Sedentary lifestyles
- Poor diets
- Smoking
- High cholesterol
- Poor sleep
- High alcohol consumption

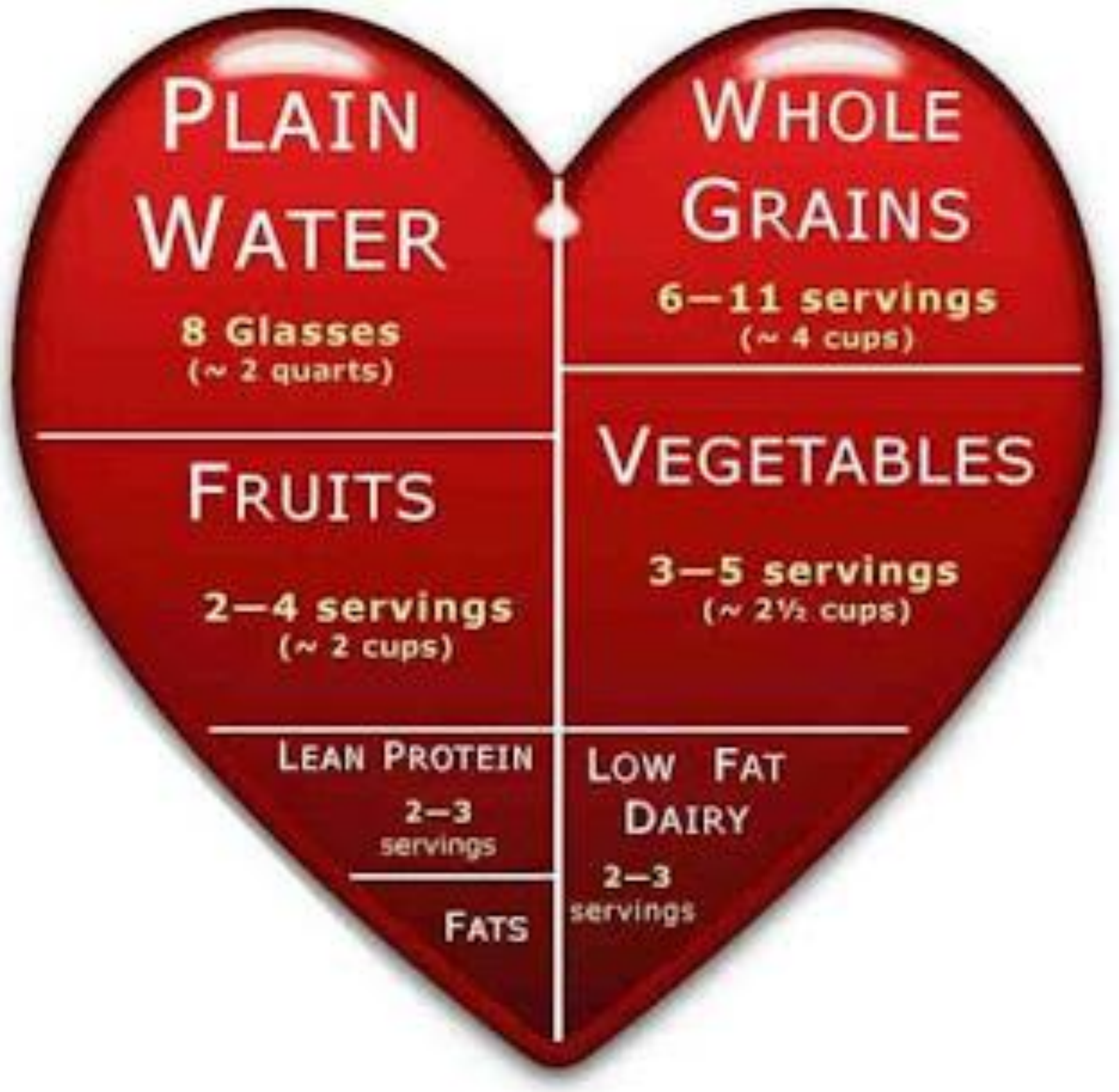

# 7 steps to prevent heart disease



1. Get active
   a. 150min of mod/vig ZONE min a week!
   b. Active on multiple days a week
2. Control Cholesterol- check regularly!
   a. Limit dietary cholesterol (animal fats)
3. Eat well
   a. Eat a variety of fruits, vegetables, whole grains, low-fat dairy/alternatives, fish, nuts, and legumes
   b. Limit saturated fat, sodium, sugar, alcohol
4. Manage blood pressure- check regularly!
   a. Limit sodium; increase potassium and water
5. Maintain healthy weight (BMI between 18-25)
6. Manage blood sugar- talk to your doctor
7. Don't smoke, avoid secondhand smoke

American Heart Association.

Even one lifestyle change can make a difference.
A combination of two or more lifestyle changes may achieve even better results.

# 7 Steps…where are you?

BB-HEALTH

In this program we focus on
Being **AWARE** so that we can be **IN CONTROL**

Which of the 7 steps do you have down?

Which need more work?

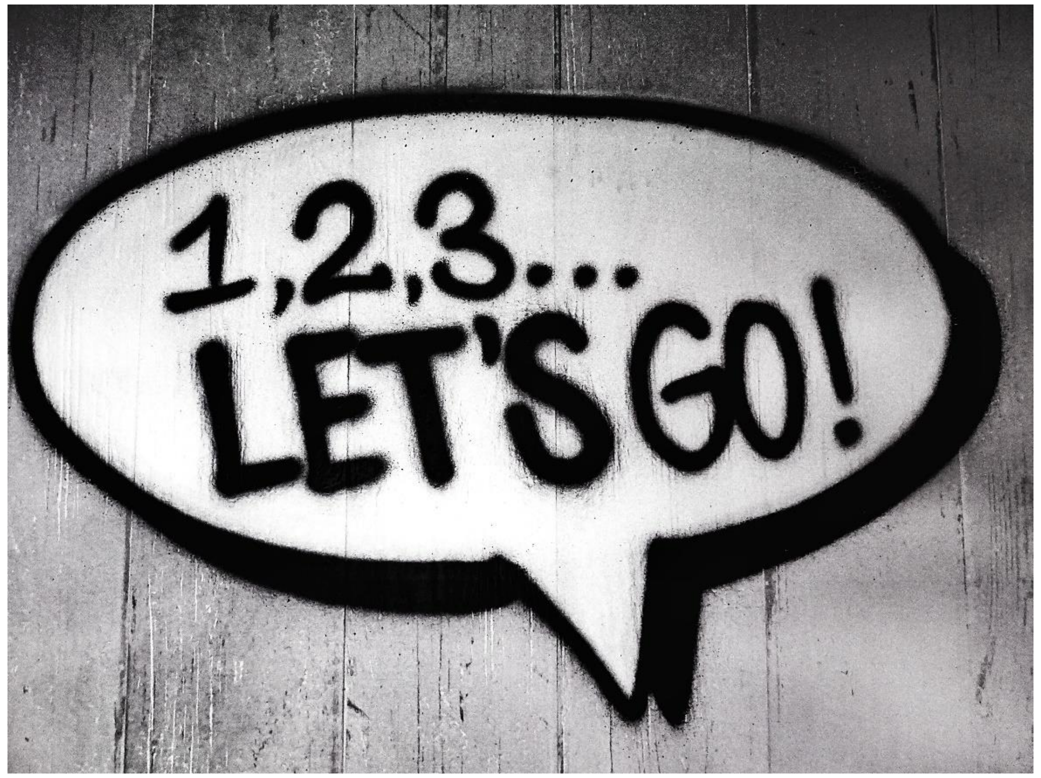


Need a refresher on how to improve your diet and activity? Look through your binders! The binder is resource for you going forward.

# Learn about cholesterol

B B - H E A L T H

We need cholesterol in our body, but our body makes all it needs.

- We get additional cholesterol from animal foods (meat, fish, eggs, dairy)
- None in plants
- Total cholesterol = LDL + HDL + TGL
    - Triglycerides- respond to what we eat immediately
- Why is TGL higher in those with higher dietary fat?
- Why is TGL higher in those with excess weight or living sedentary lifestyles?

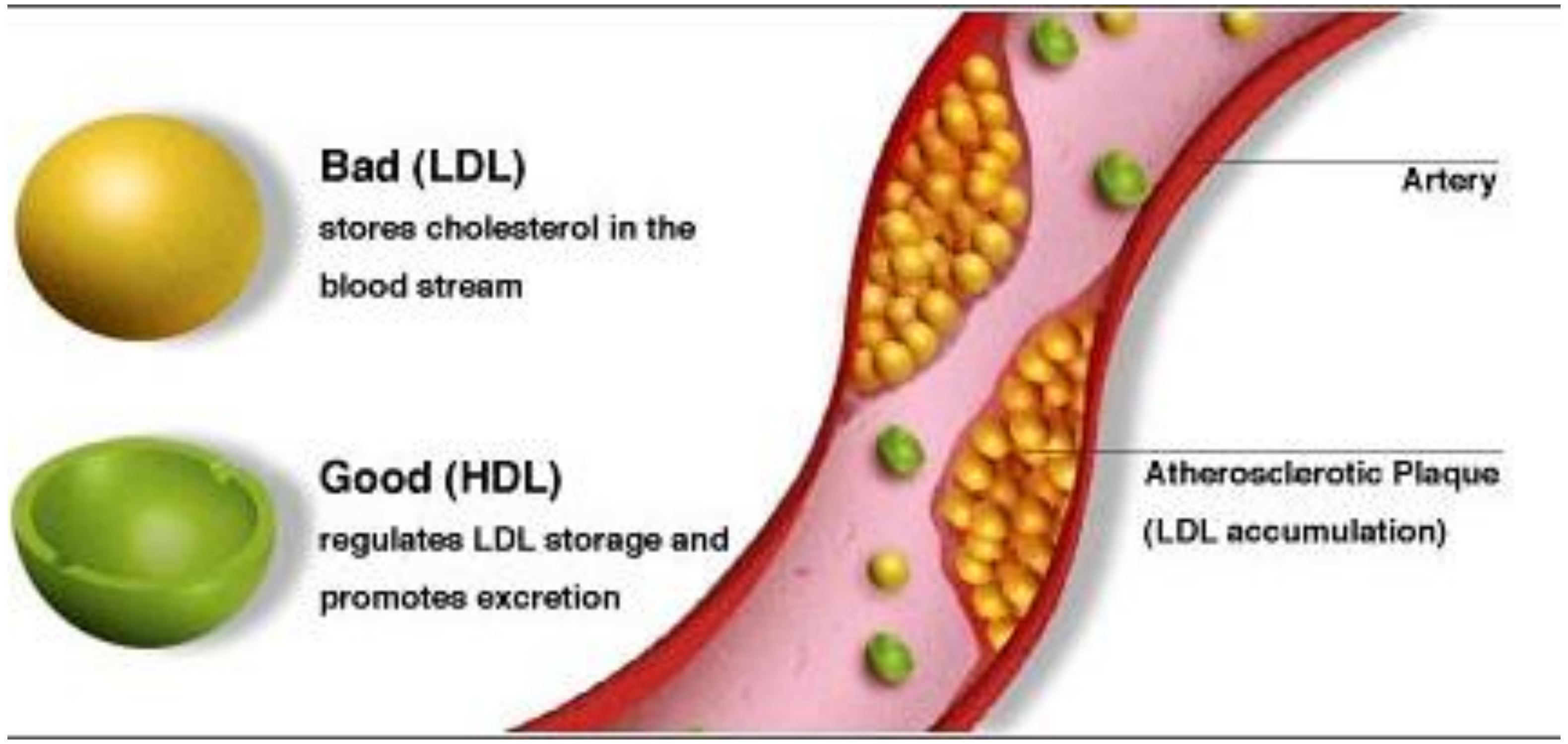


Talk to your health care provider to find the best way to manage your cholesterol. Many things in this program can help!

# Learn about blood pressure

BB-HEALTH

As blood moves through our vessels, the pressure on the walls is what we measure.

- Systolic (top number) is the pressure during a heartbeat
- Diastolic (bottom number) the pressure in between beats
- Asymptomatic
- Why does salt increase blood pressure?
- Why does increased weight increase blood pressure?

Talk to your health care provider to find the best way to manage your blood pressure.
Many things in this program can help!

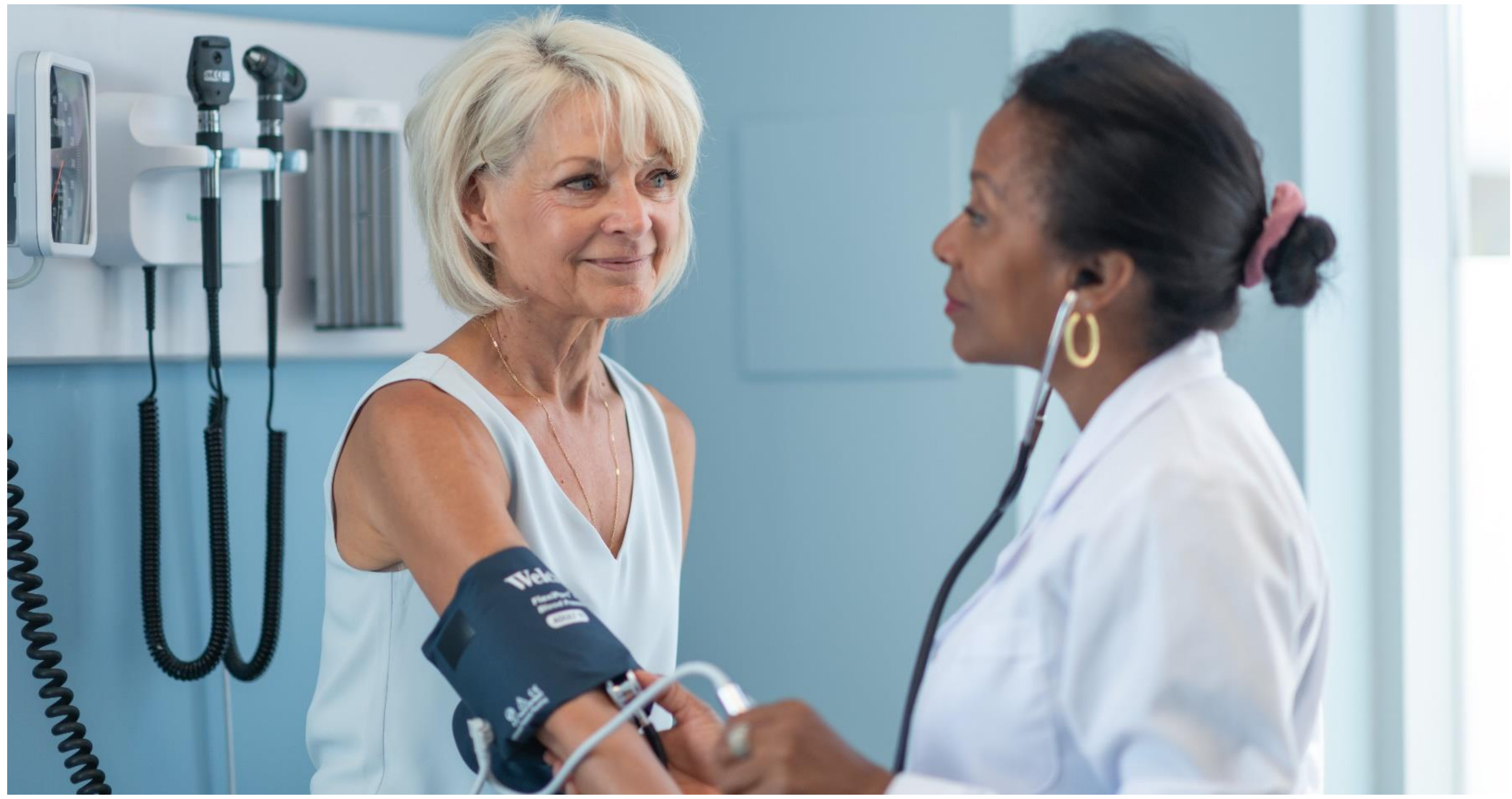

# Improving Blood Pressure and Cholesterol

- Maintain a healthy weight
- Get active
- Get enough sleep
- Don’t smoke
- Eat healthy
  - Limit fats, and focus on good fats (*page 8*)
  - Limit added sugar
  - Limit salt intake
  - Increase fiber
  - Check out DASH diet (*page 7*)

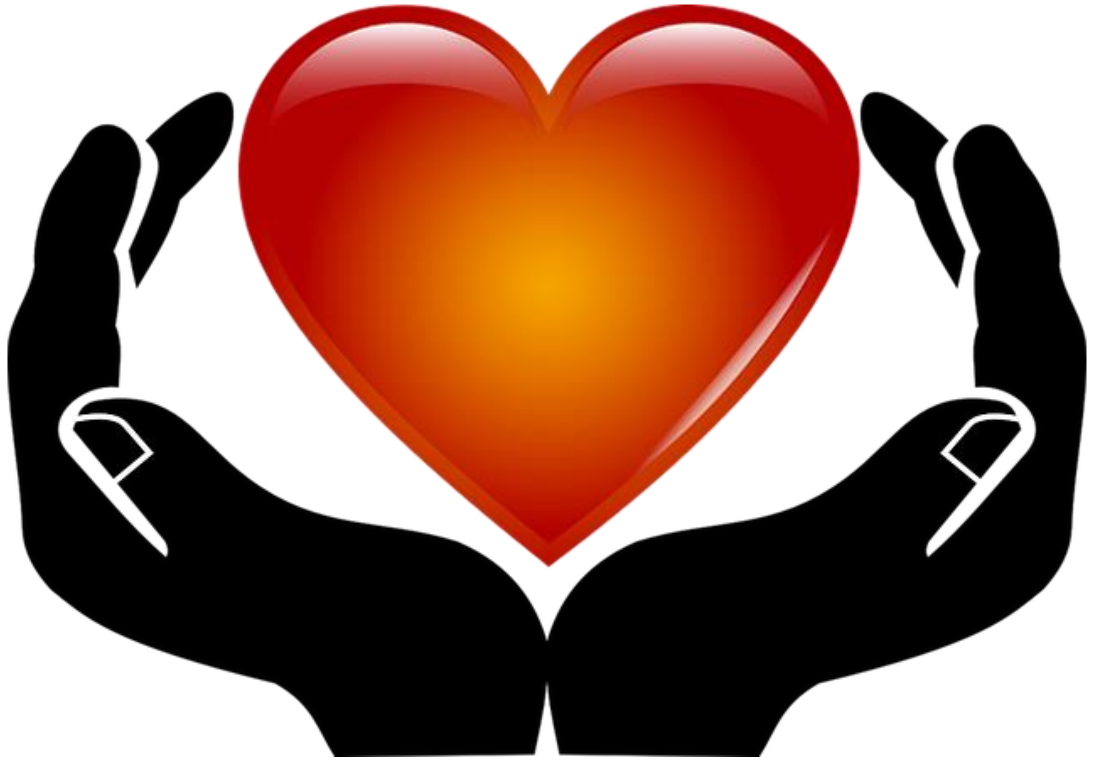

Studies show that these lifestyle choices make a big difference. Whether taking medication or not, these healthy choices will help support your health.

# Changes I can make

BB-HEALTH

What healthy choices will you make to improve or maintain heart health?

- Keep saturated fat below 10% of calories a day
- Keep sugar below 10% calories a day
- Choose foods with at least 3-5 grams fiber
- Keep sodium below my calories
- Get 10,000 steps a day
- Get 150 min mod/vig exercise a week
- Other (discuss)

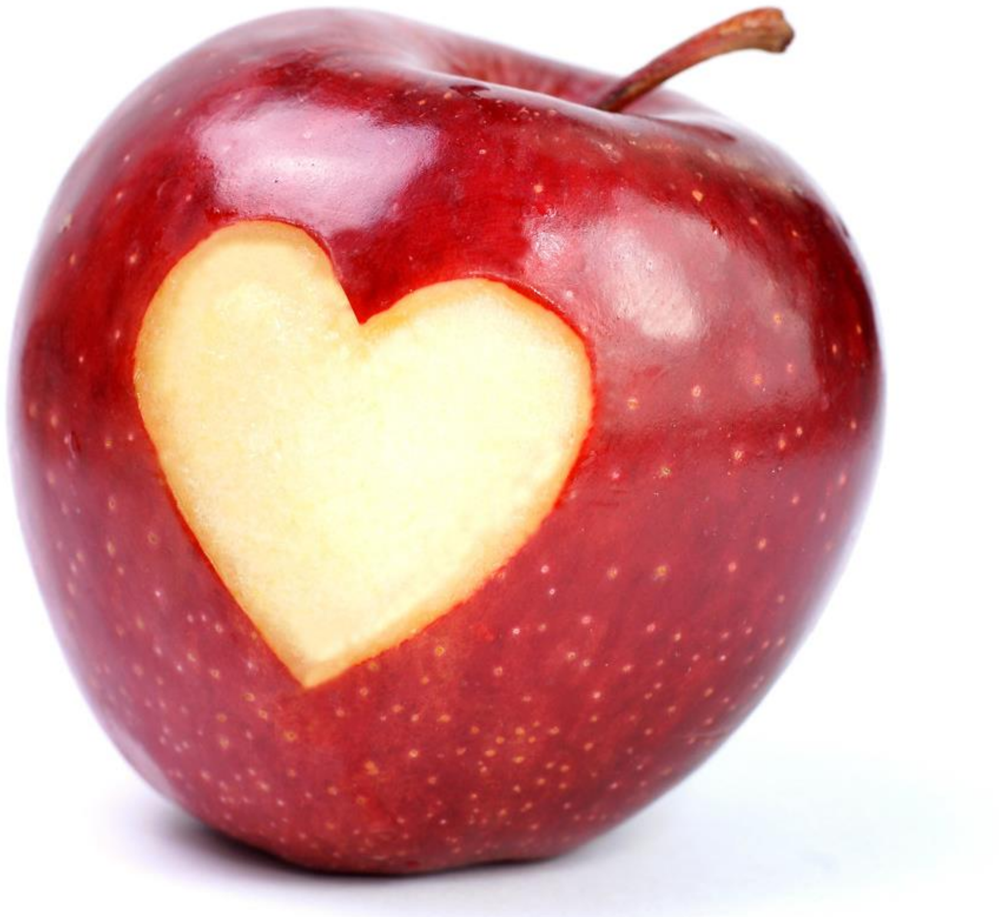

Keeps the doctor away?
- Good Fiber
- Low sodium
- No fat
- No added sugar

# Put it into Practice



**Practice Heart Health!**

1. Track those meals and take your health EVERYDAY!
   - Monitor your progress and identify areas for improvement towards YOUR GOALS

2. Aim to get OVER 150 min of moderate or vigorous exercise ***a week*** by our next session!
   - Make your step count average over 10,000 a day!
   - Keep up your steps, reduce sedentary time– get all 9, or move to 10!

3. Make some healthy choices to prioritize heart health.
   - Eating right, sleeping right, getting active!

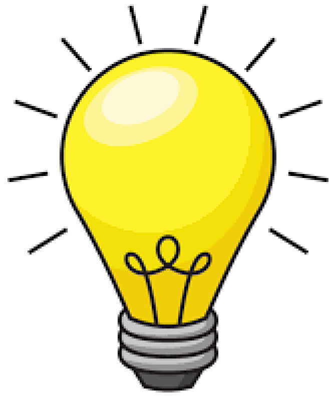

**THIS MONTH'S TIP:** ***Find motivation all around you***
Your motivations may have changed, they could be heart health or maintaining your weight loss or something else. Use everything around you to keep you motivated to keep going. You got this!

# Questions?

B B - H E A L T H

**For next month:**

1. Focus on the good work you are doing!

2. Track your food and exercise and **<u>Weigh Every Day</u>**

3. Remember your calorie, fat, and activity targets

4. Email your logs and progress to the Intervention Team and Review the response!

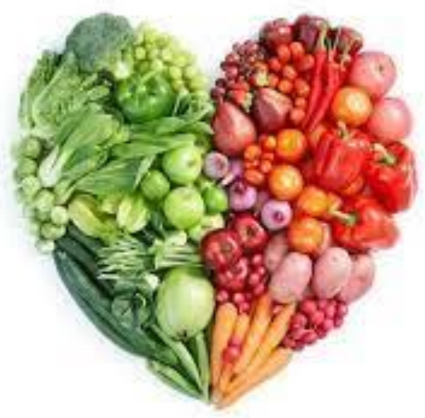

**FOOD FOR THOUGHT**

- These lifestyle choices pay triple the effort that goes into them.
- Like most things, you get out what you put in, keep working!
