# Supplementary material for: Development and Feasibility of an eHealth Diabetes Prevention Program Adapted for Older Adults—Results from a Randomized Control Pilot Study
Source: Nutrients. 2024 Mar 23;16(7):930. doi: 10.3390/nu16070930 (PMC11154527; doi:10.3390/nu16070930)
Supplement: Supplementary file 1 [file nutrients-16-00930-s001.zip › Session 22.pptx]

## Slide 1
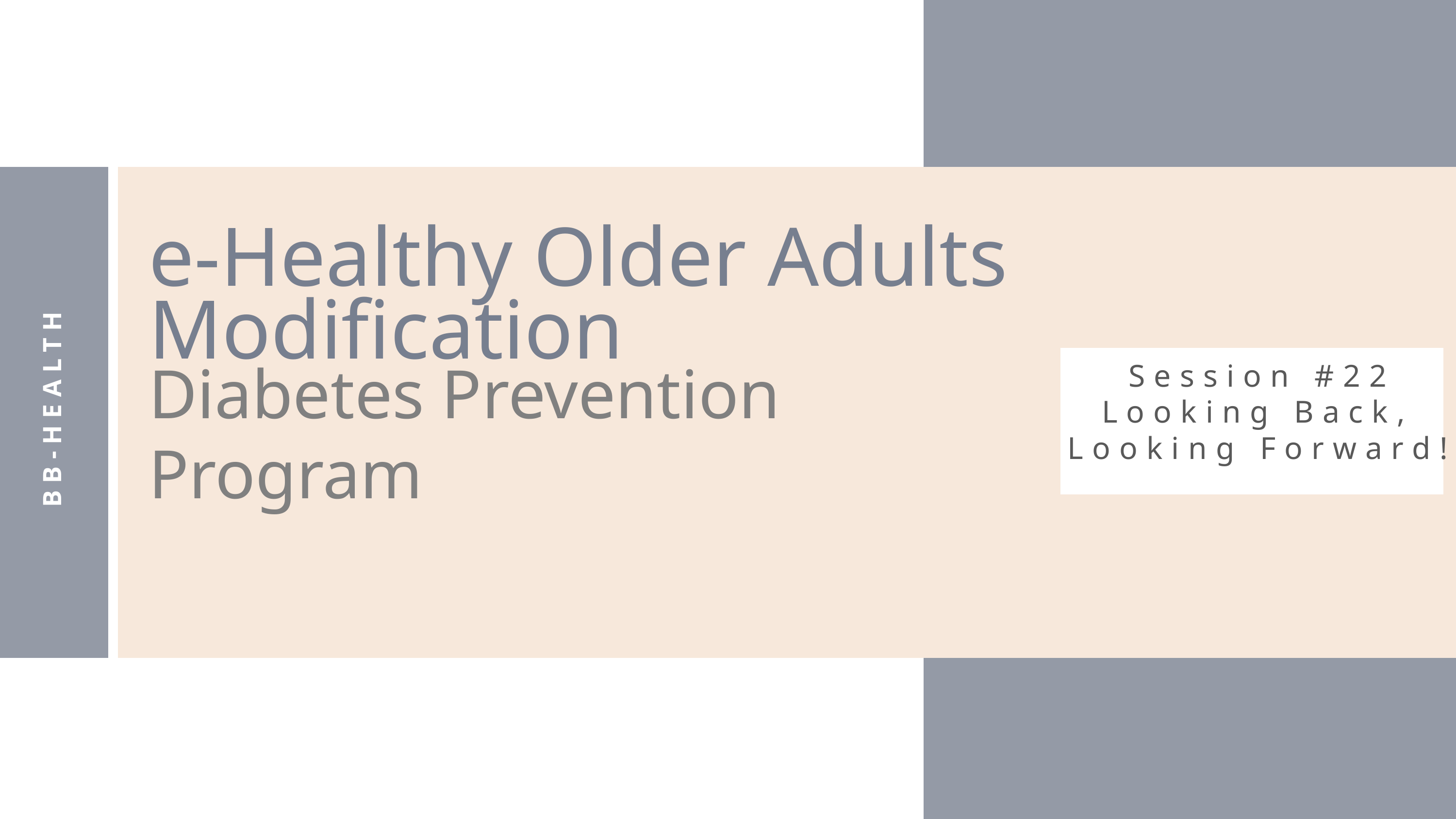

OPEN REPORTS
e-Healthy Older Adults Modification
Session #22
Looking Back, Looking Forward!
Diabetes Prevention Program
BB-HEALTH

## Slide 2
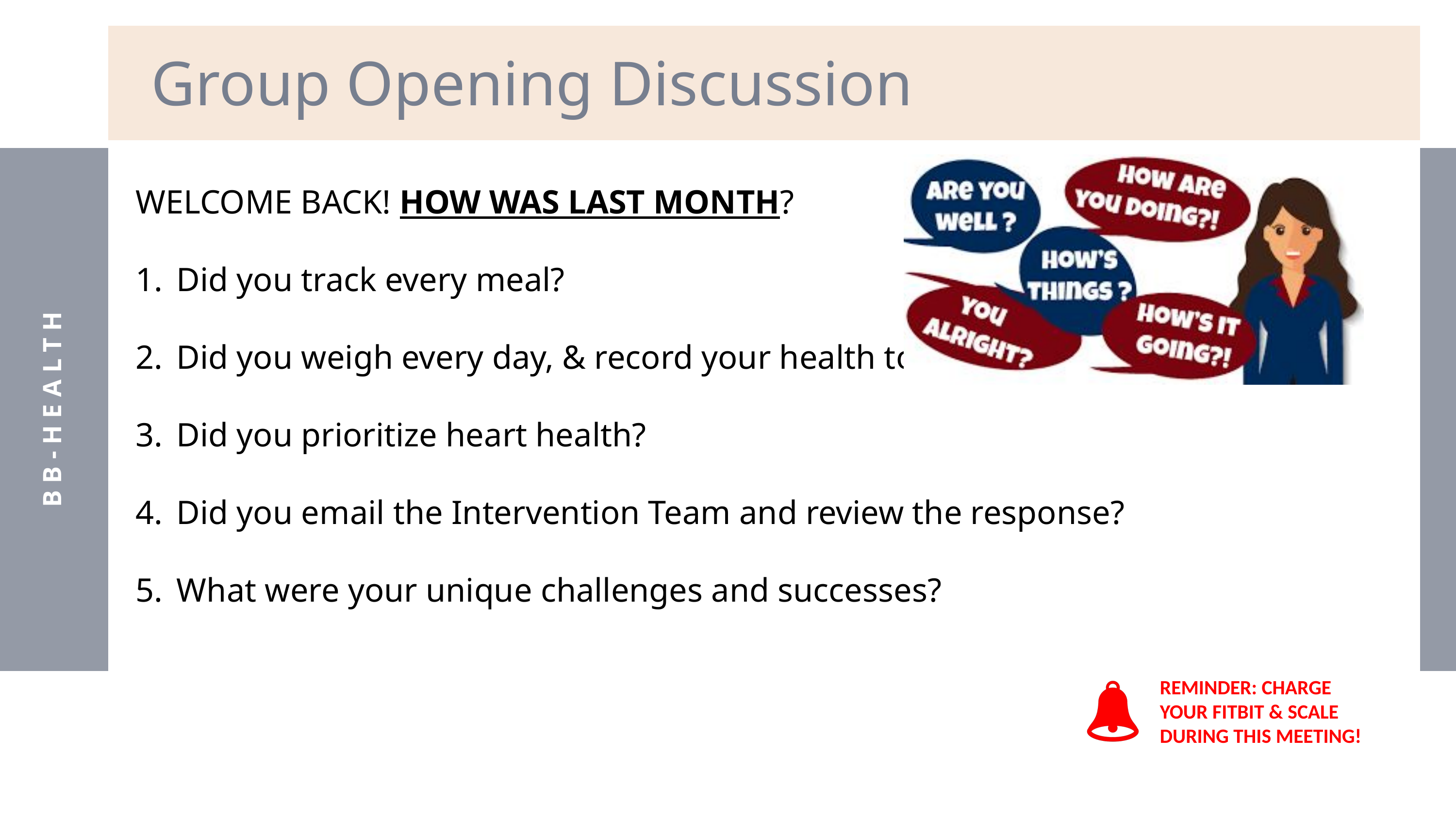

Group Opening Discussion
WELCOME BACK! HOW WAS LAST MONTH?
Did you track every meal?
Did you weigh every day, & record your health today?
Did you prioritize heart health?
Did you email the Intervention Team and review the response?
What were your unique challenges and successes?
BB-HEALTH
REMINDER: CHARGE YOUR FITBIT & SCALE DURING THIS MEETING!

## Slide 3
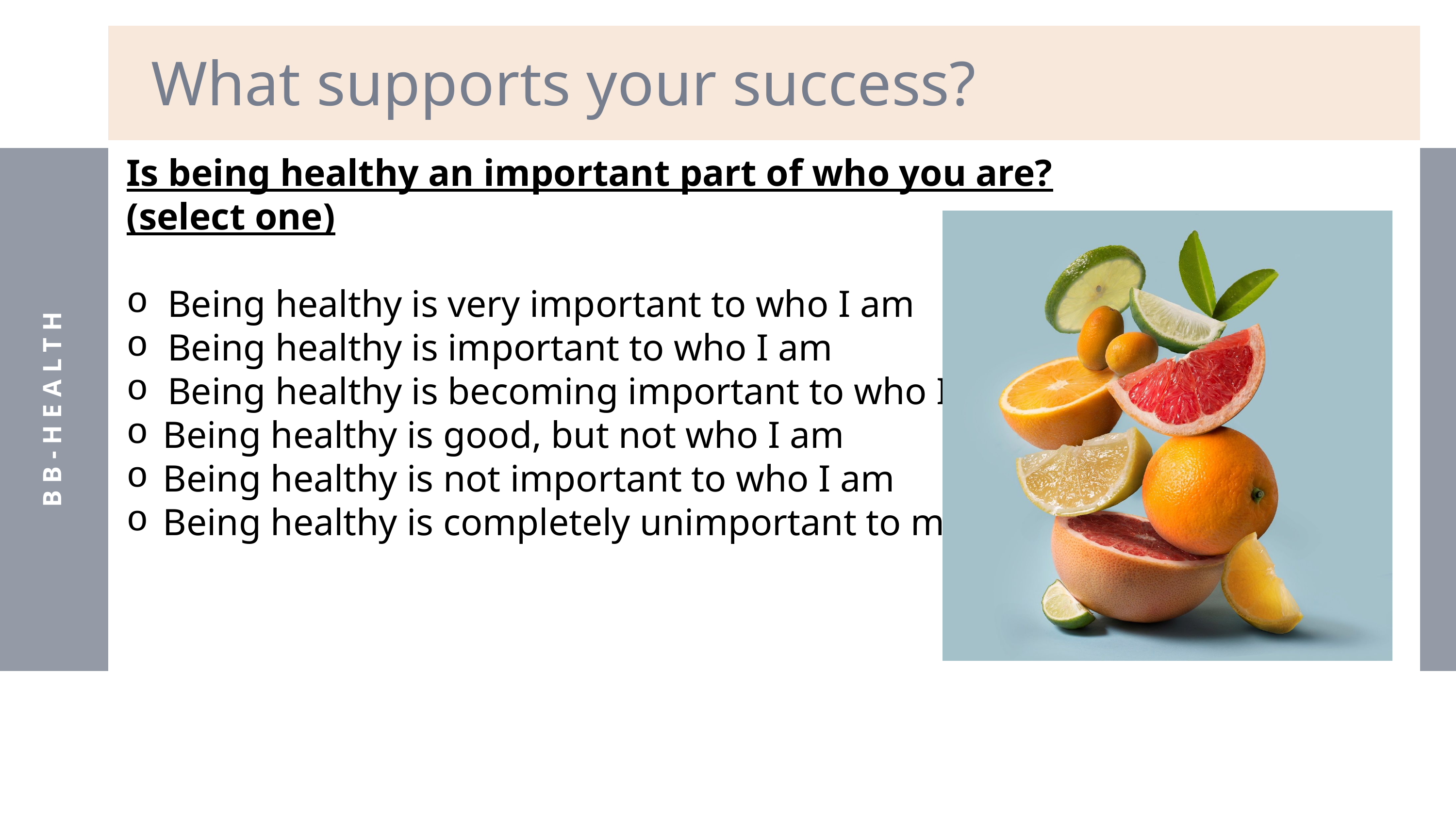

What supports your success?
Is being healthy an important part of who you are? (select one)
Being healthy is very important to who I am
Being healthy is important to who I am
Being healthy is becoming important to who I am
Being healthy is good, but not who I am
Being healthy is not important to who I am
Being healthy is completely unimportant to me
BB-HEALTH

## Slide 4
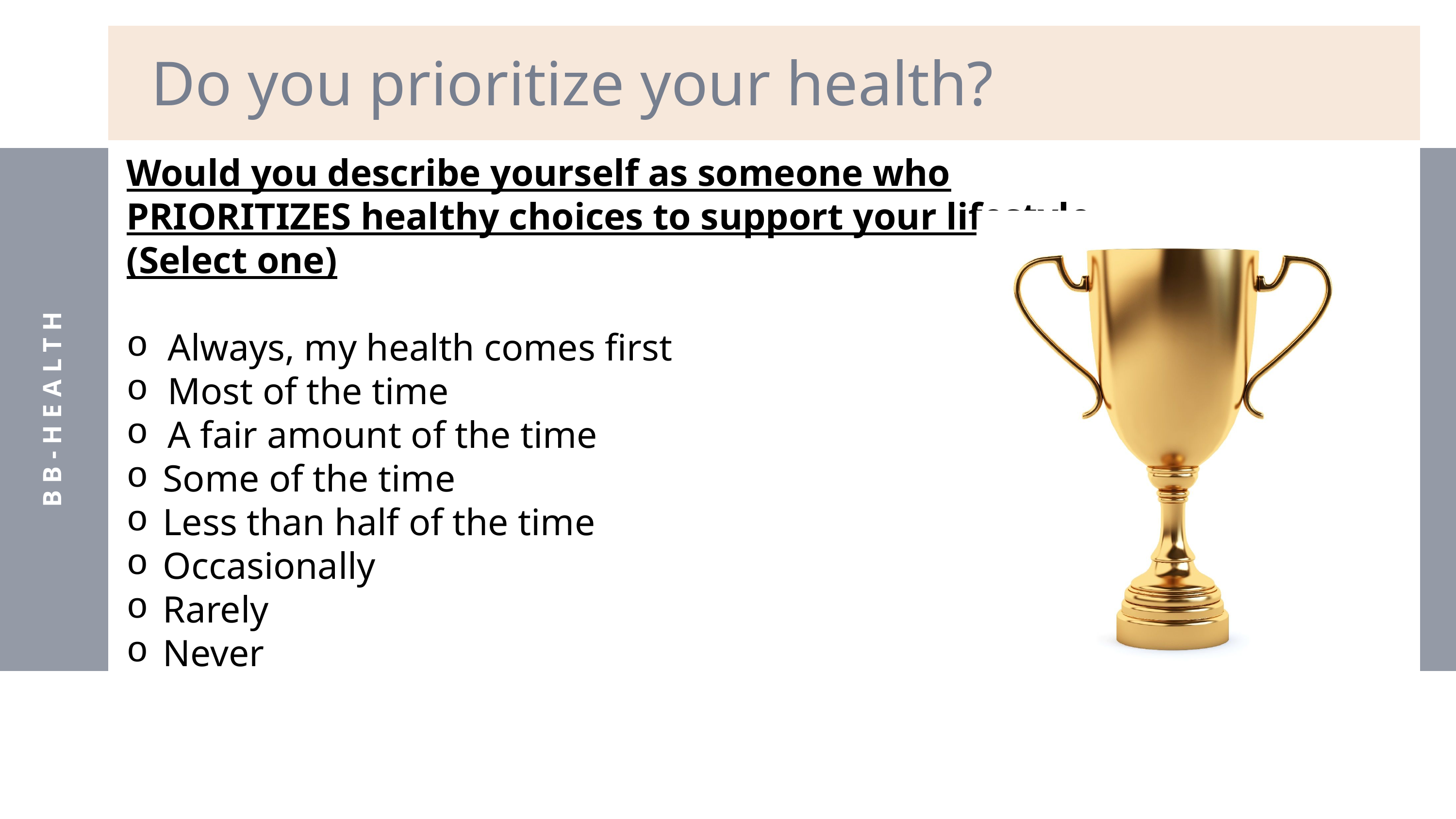

Do you prioritize your health?
Would you describe yourself as someone who PRIORITIZES healthy choices to support your lifestyle (Select one)
Always, my health comes first
Most of the time
A fair amount of the time
Some of the time
Less than half of the time
Occasionally
Rarely
Never
BB-HEALTH

## Slide 5
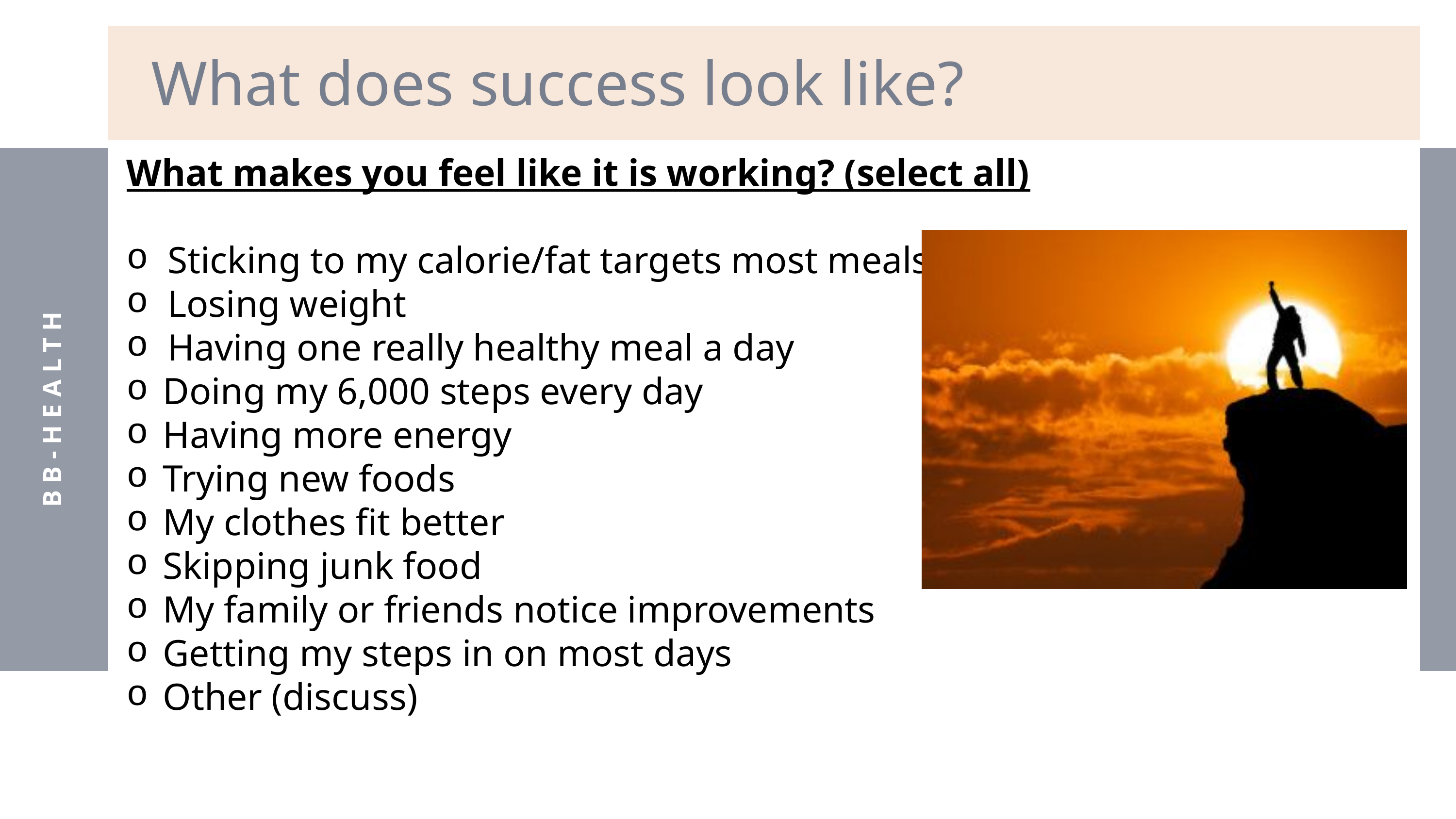

What does success look like?
What makes you feel like it is working? (select all)
Sticking to my calorie/fat targets most meals
Losing weight
Having one really healthy meal a day
Doing my 6,000 steps every day
Having more energy
Trying new foods
My clothes fit better
Skipping junk food
My family or friends notice improvements
Getting my steps in on most days
Other (discuss)
BB-HEALTH

## Slide 6
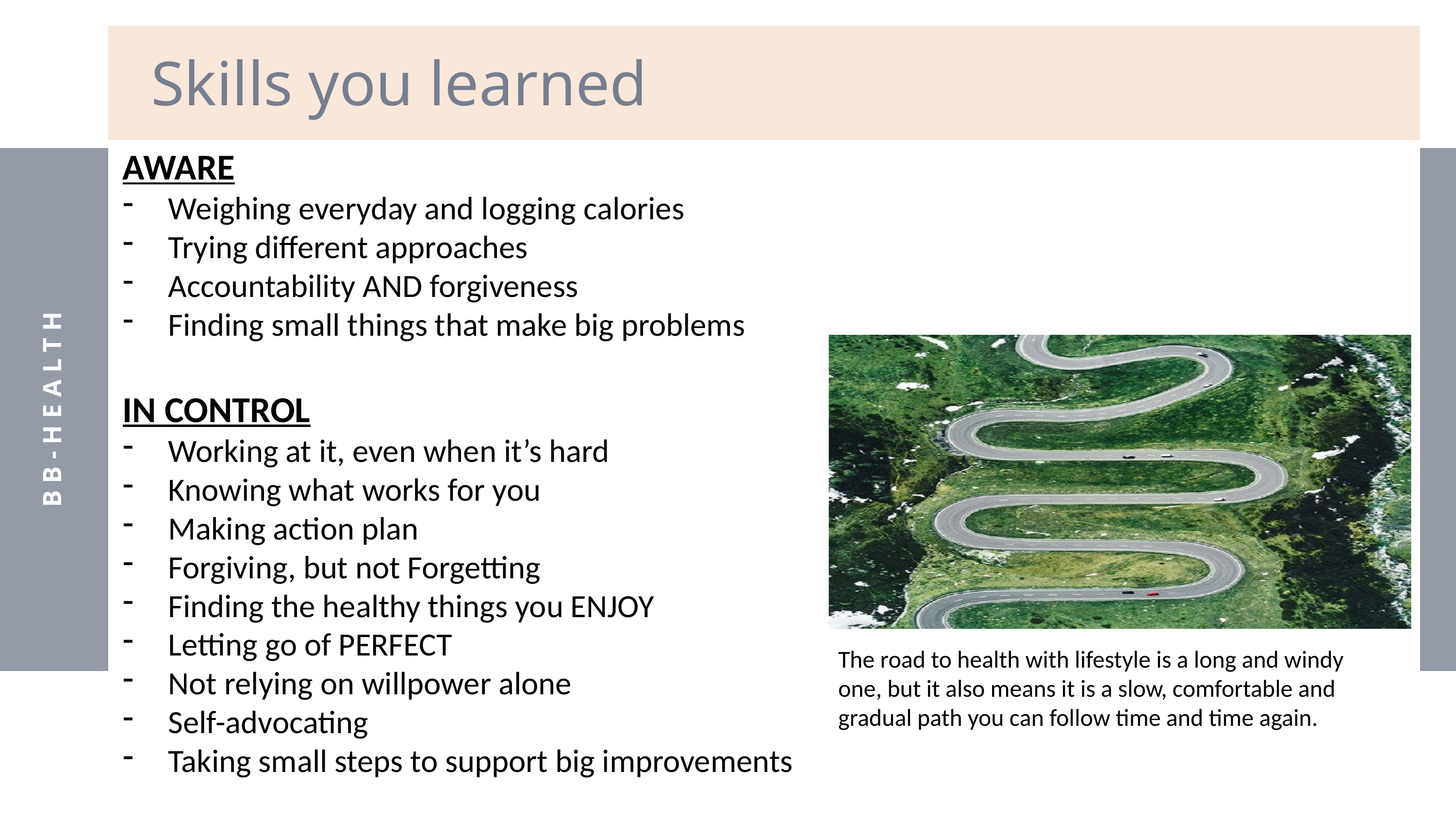

Skills you learned
AWARE
Weighing everyday and logging calories
Trying different approaches
Accountability AND forgiveness
Finding small things that make big problems
IN CONTROL
Working at it, even when it’s hard
Knowing what works for you
Making action plan
Forgiving, but not Forgetting
Finding the healthy things you ENJOY
Letting go of PERFECT
Not relying on willpower alone
Self-advocating
Taking small steps to support big improvements
BB-HEALTH
The road to health with lifestyle is a long and windy one, but it also means it is a slow, comfortable and gradual path you can follow time and time again.

## Slide 7
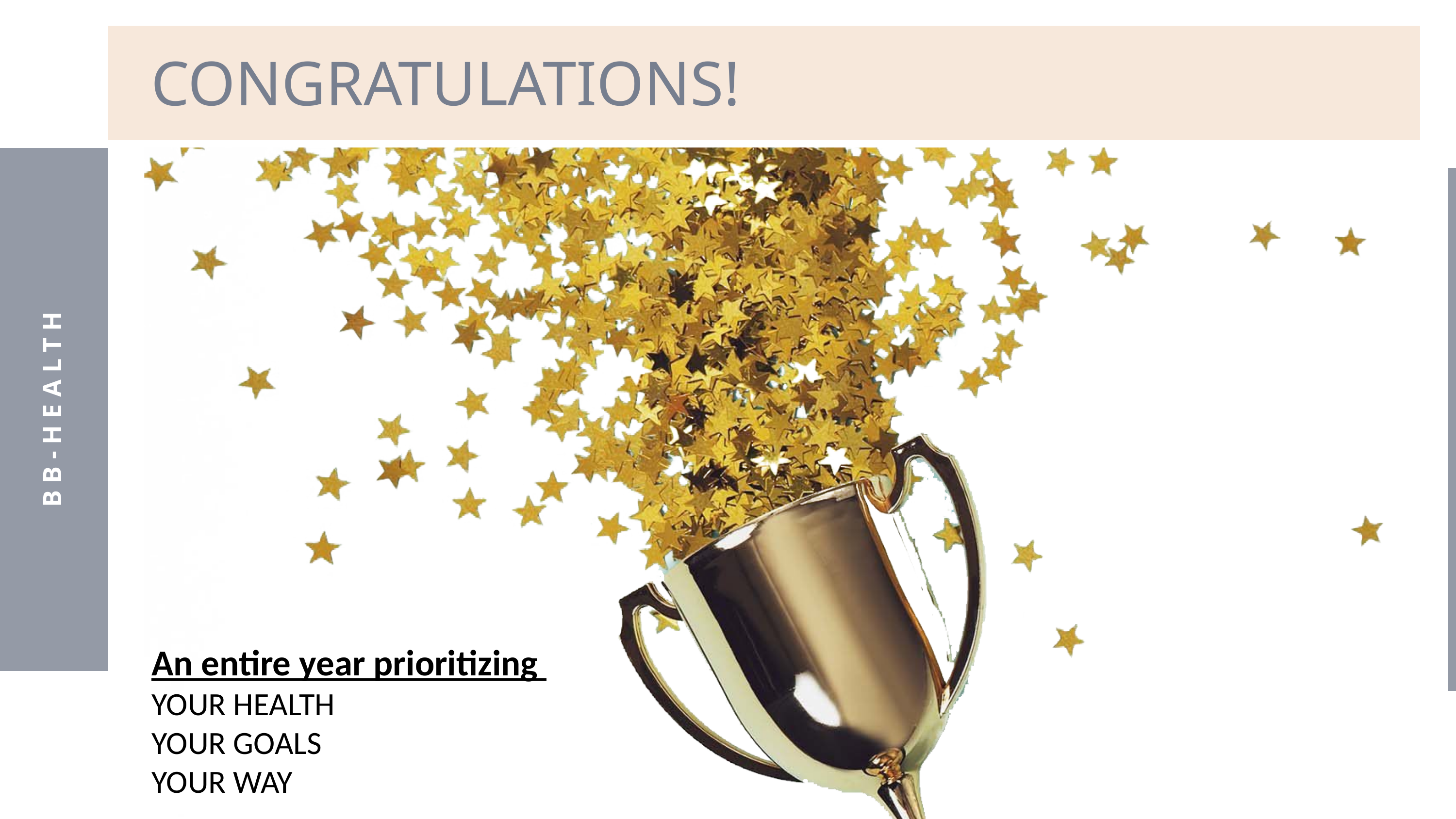

CONGRATULATIONS!
BB-HEALTH
An entire year prioritizing
YOUR HEALTH
YOUR GOALS
YOUR WAY

## Slide 8
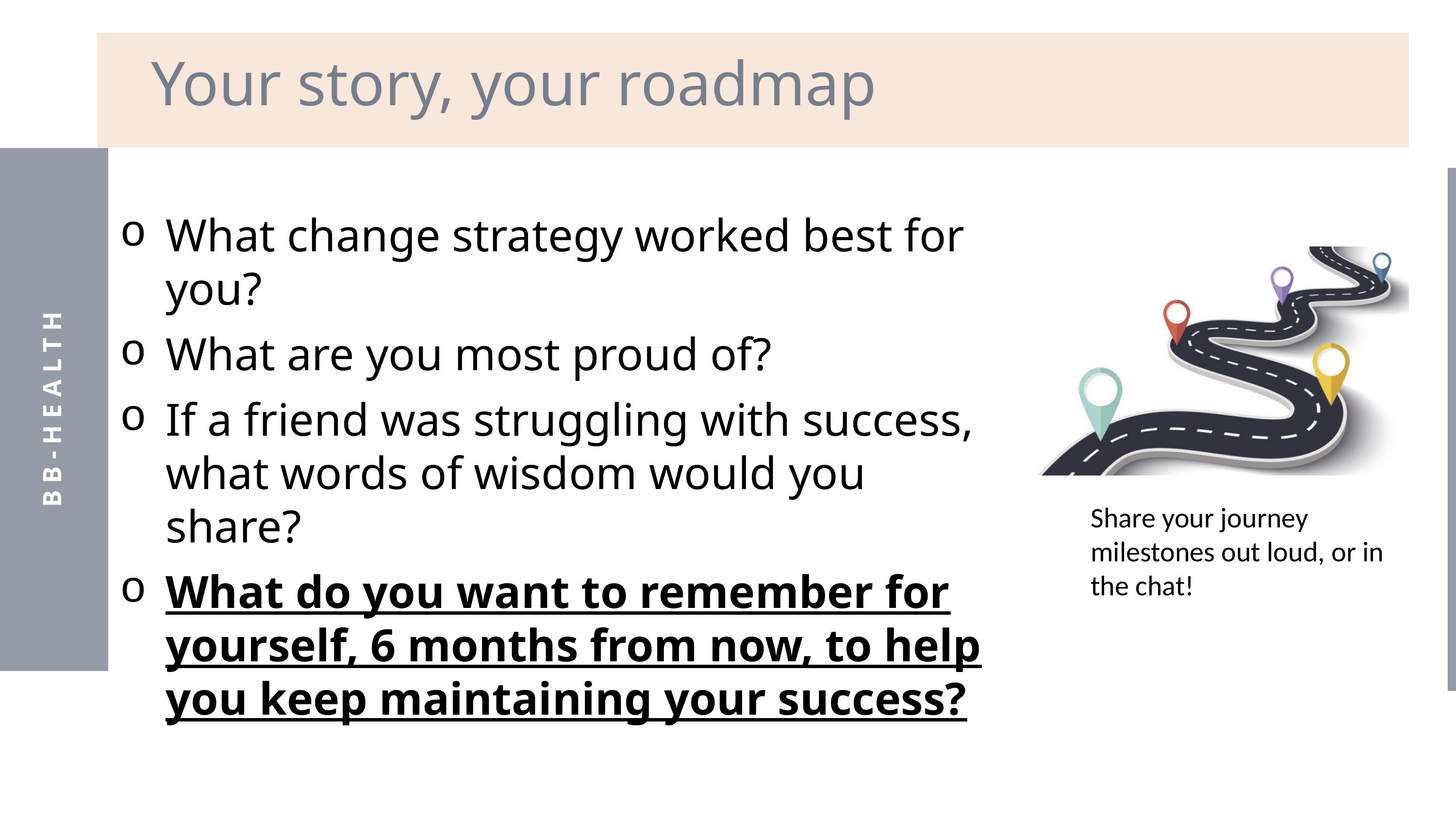

Your story, your roadmap
What change strategy worked best for you?
What are you most proud of?
If a friend was struggling with success, what words of wisdom would you share?
What do you want to remember for yourself, 6 months from now, to help you keep maintaining your success?
BB-HEALTH
Share your journey milestones out loud, or in the chat!

## Slide 9
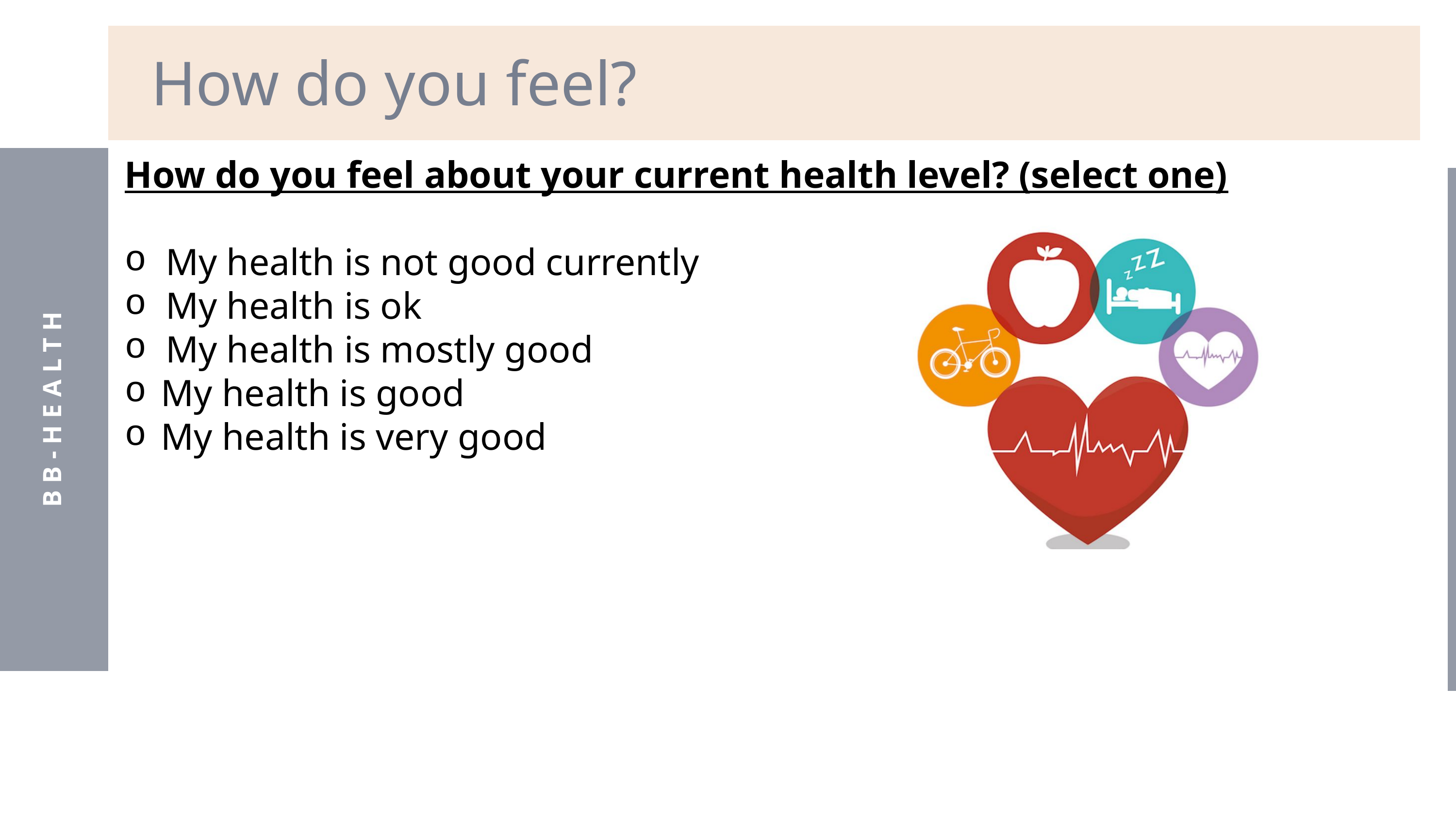

How do you feel?
How do you feel about your current health level? (select one)
My health is not good currently
My health is ok
My health is mostly good
My health is good
My health is very good
BB-HEALTH

## Slide 10
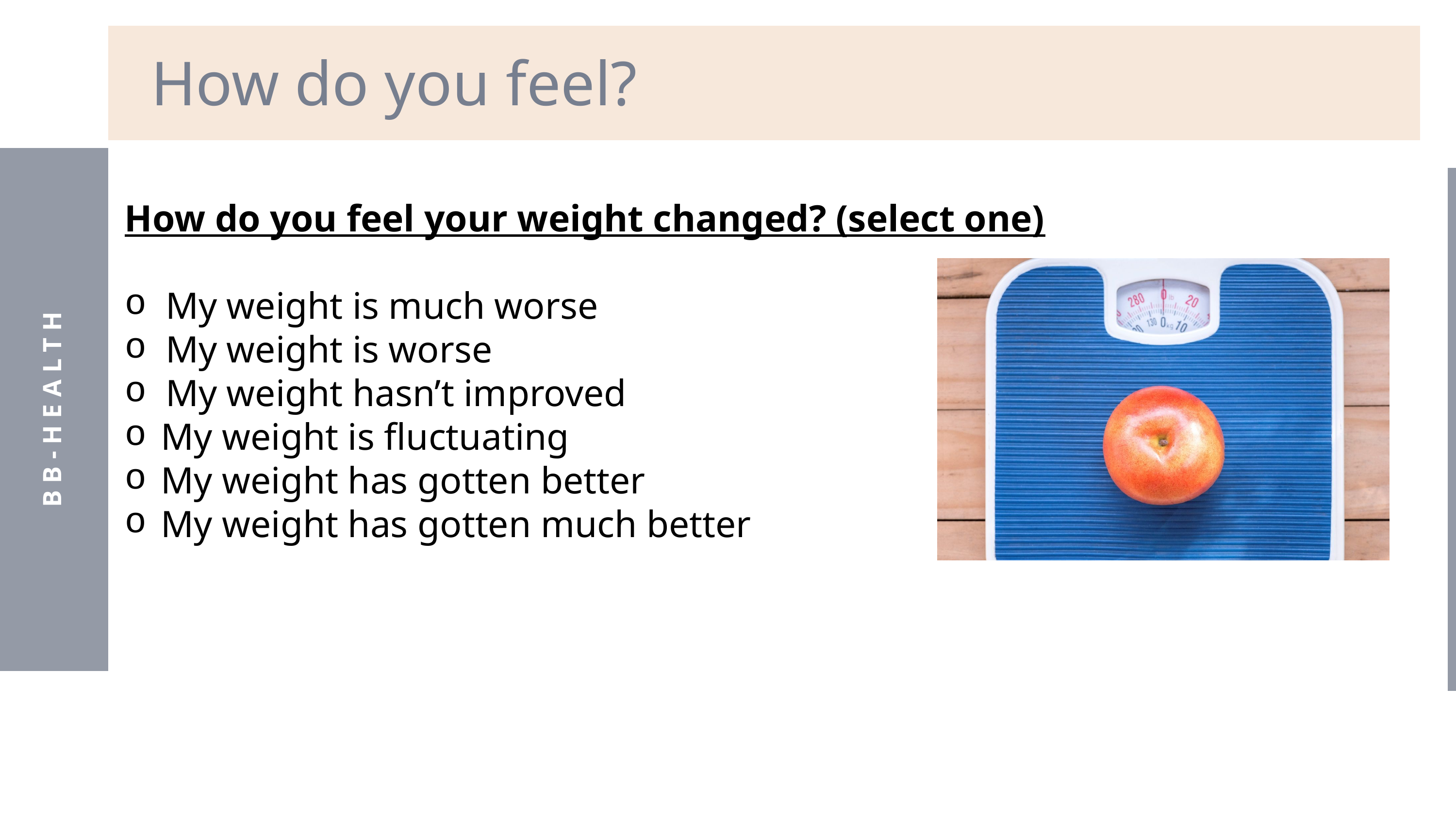

How do you feel?
How do you feel your weight changed? (select one)
My weight is much worse
My weight is worse
My weight hasn’t improved
My weight is fluctuating
My weight has gotten better
My weight has gotten much better
BB-HEALTH

## Slide 11
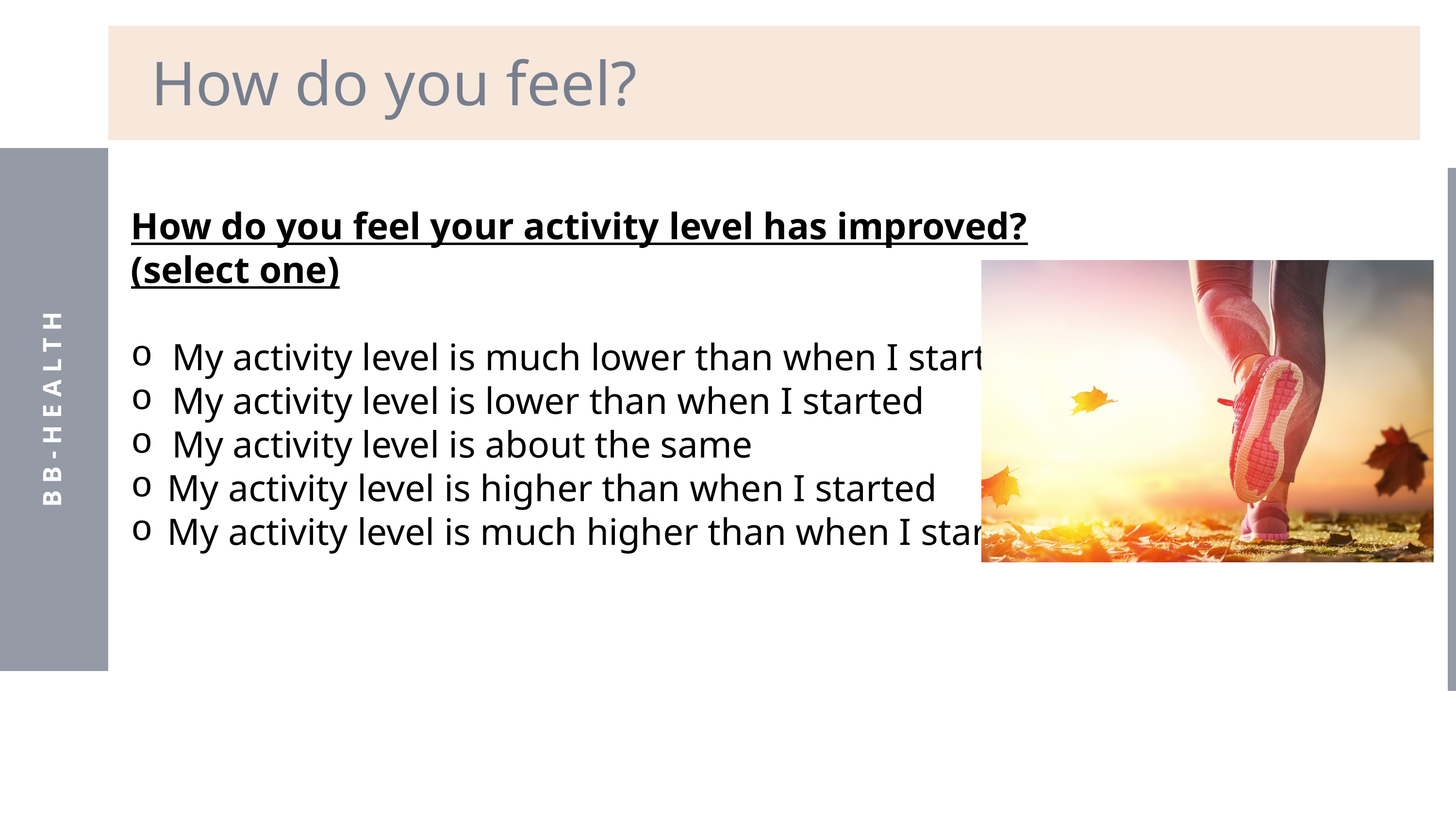

How do you feel?
How do you feel your activity level has improved? (select one)
My activity level is much lower than when I started
My activity level is lower than when I started
My activity level is about the same
My activity level is higher than when I started
My activity level is much higher than when I started
BB-HEALTH

## Slide 12
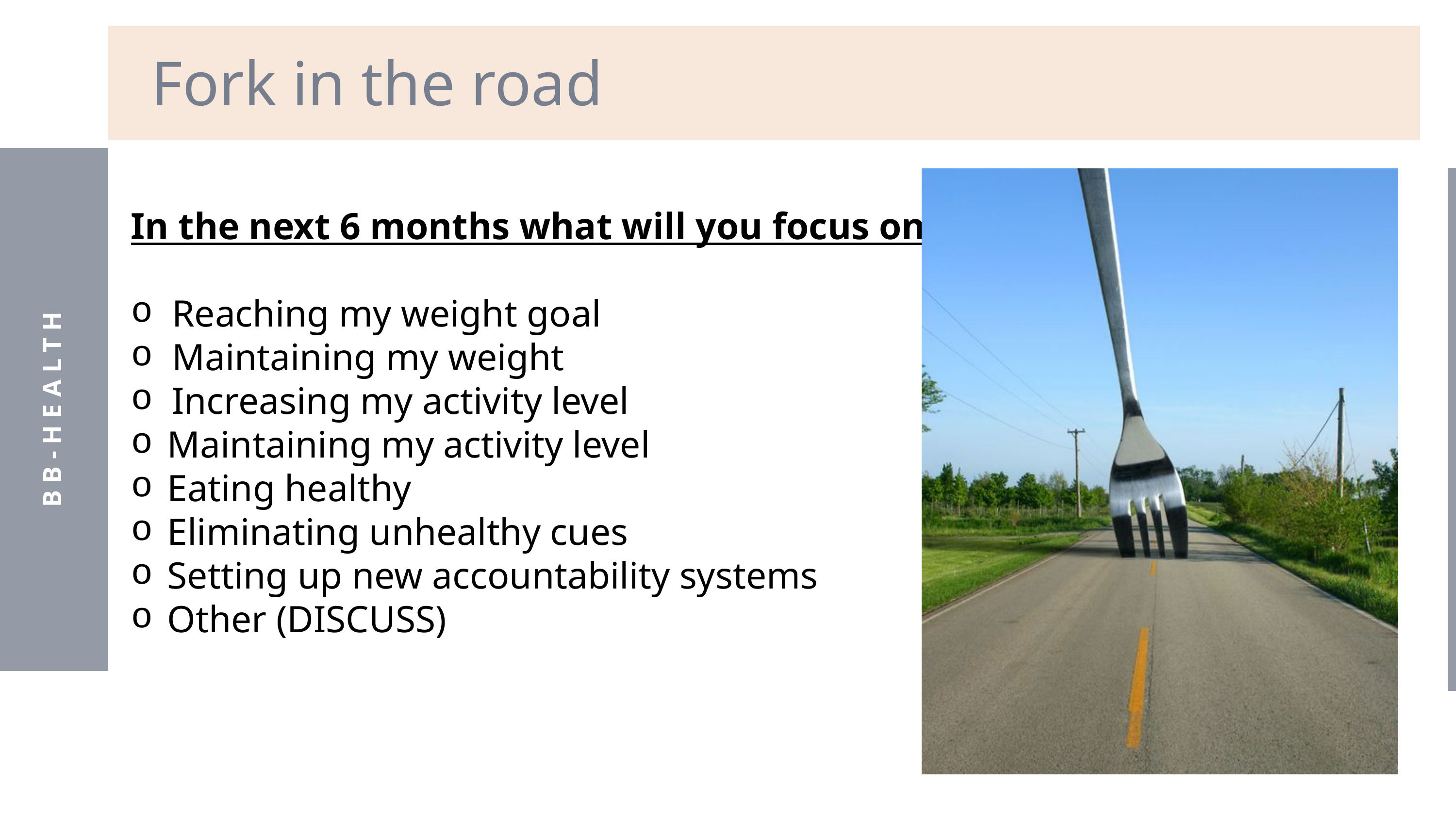

Fork in the road
In the next 6 months what will you focus on?
Reaching my weight goal
Maintaining my weight
Increasing my activity level
Maintaining my activity level
Eating healthy
Eliminating unhealthy cues
Setting up new accountability systems
Other (DISCUSS)
BB-HEALTH

## Slide 13
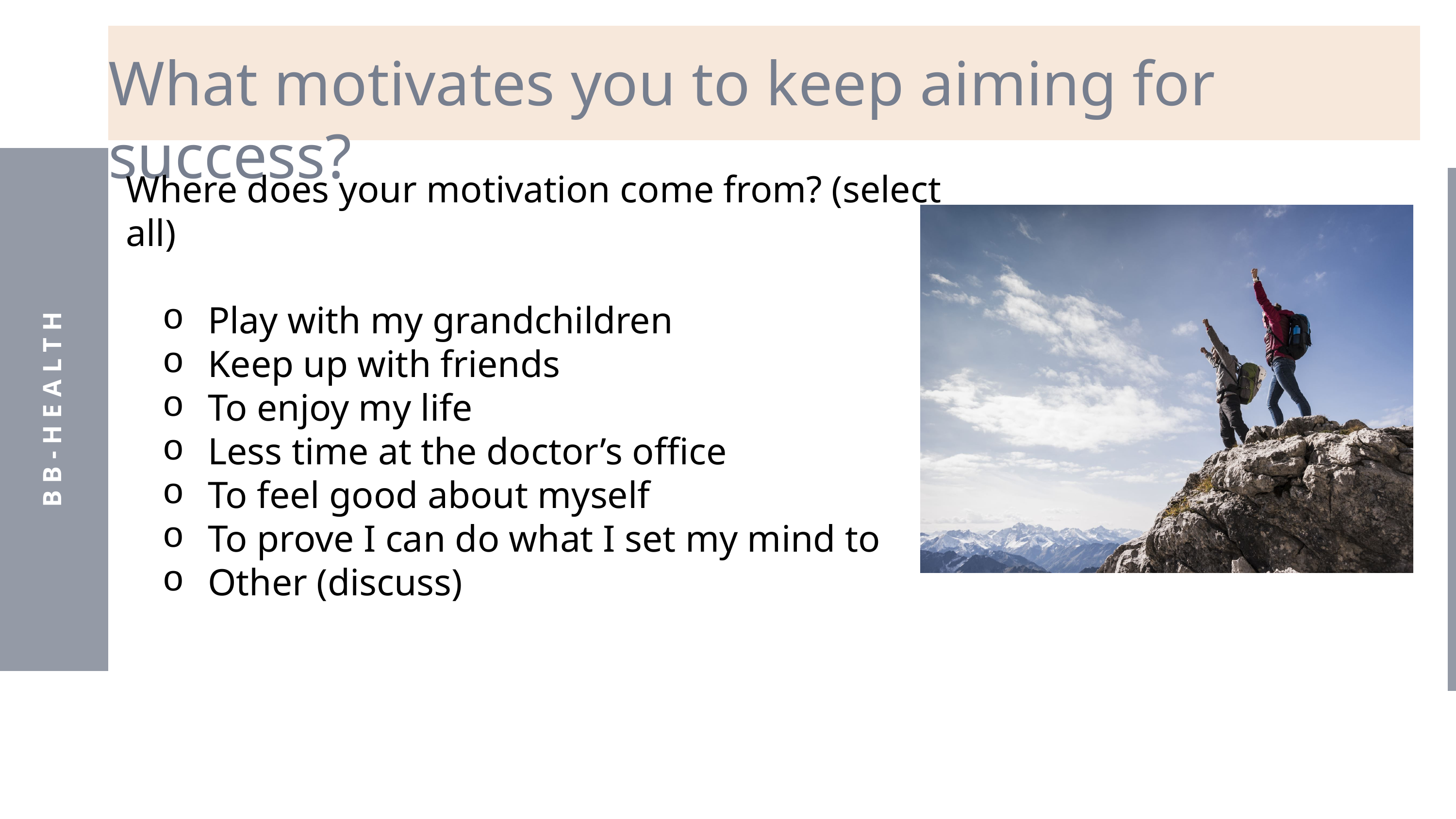

What motivates you to keep aiming for success?
Where does your motivation come from? (select all)
Play with my grandchildren
Keep up with friends
To enjoy my life
Less time at the doctor’s office
To feel good about myself
To prove I can do what I set my mind to
Other (discuss)
BB-HEALTH

## Slide 14
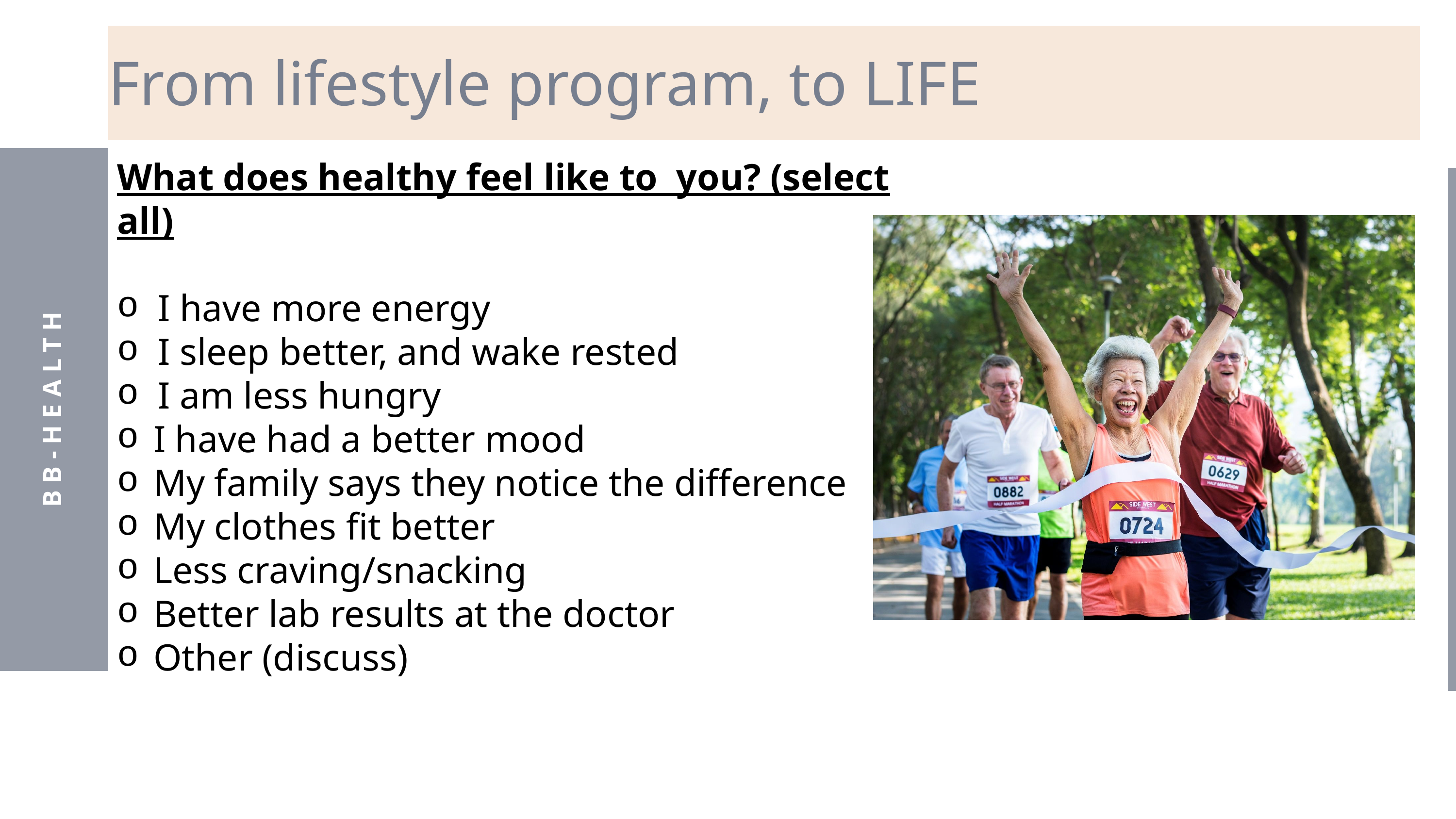

From lifestyle program, to LIFE
What does healthy feel like to you? (select all)
I have more energy
I sleep better, and wake rested
I am less hungry
I have had a better mood
My family says they notice the difference
My clothes fit better
Less craving/snacking
Better lab results at the doctor
Other (discuss)
BB-HEALTH

## Slide 15
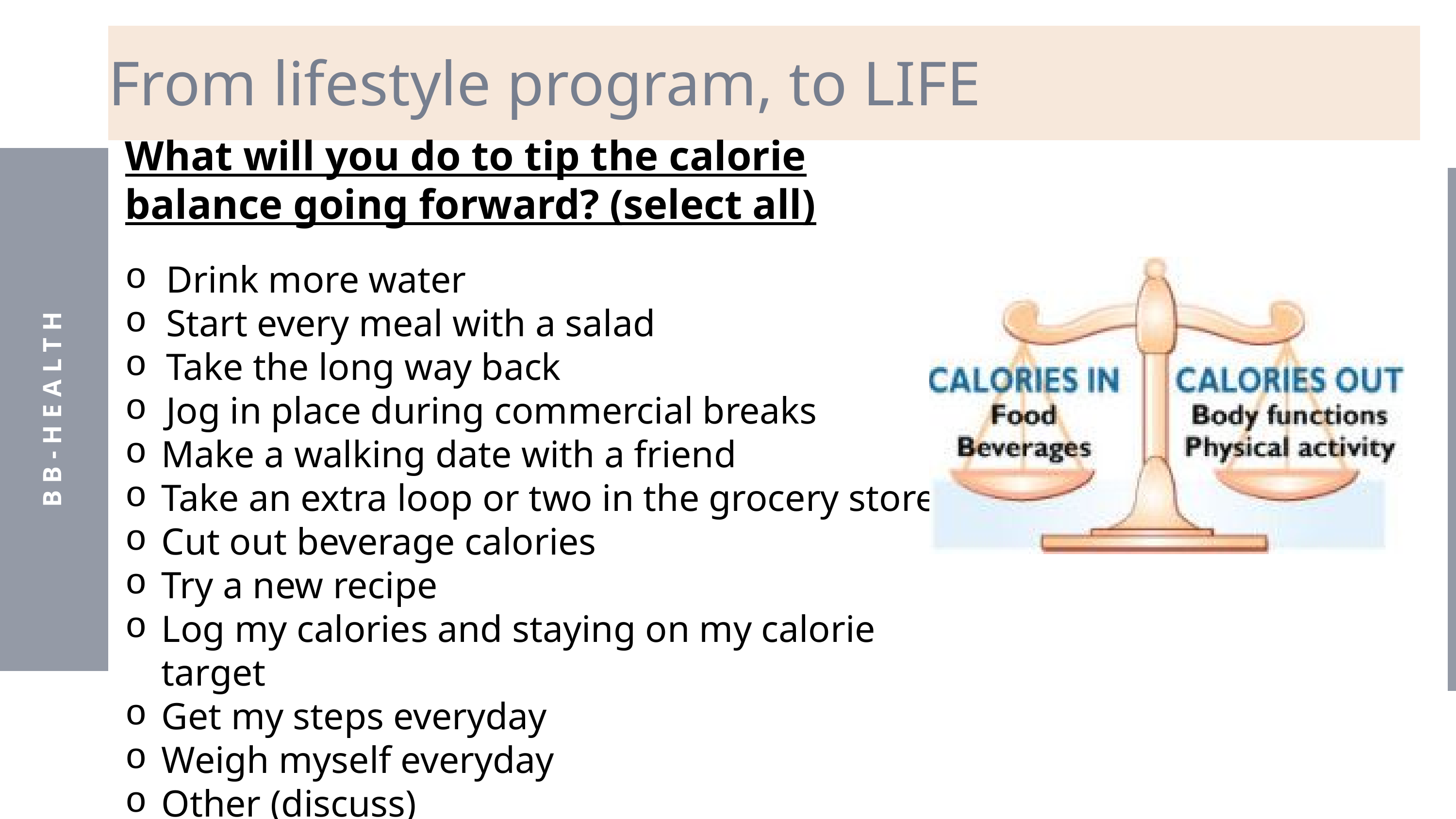

From lifestyle program, to LIFE
What will you do to tip the calorie balance going forward? (select all)
Drink more water
Start every meal with a salad
Take the long way back
Jog in place during commercial breaks
Make a walking date with a friend
Take an extra loop or two in the grocery store
Cut out beverage calories
Try a new recipe
Log my calories and staying on my calorie target
Get my steps everyday
Weigh myself everyday
Other (discuss)
BB-HEALTH

## Slide 16
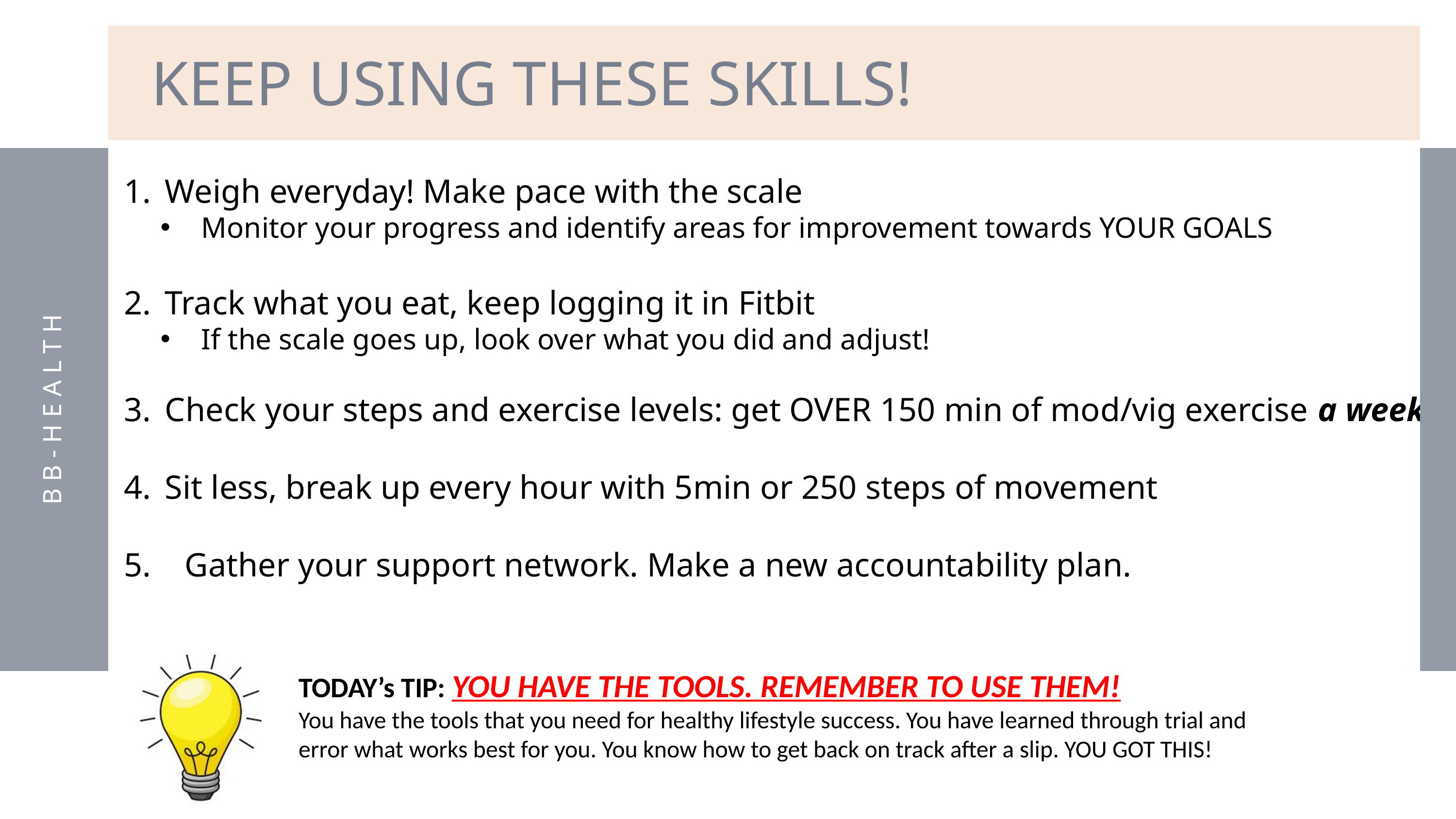

KEEP USING THESE SKILLS!
Weigh everyday! Make pace with the scale
Monitor your progress and identify areas for improvement towards YOUR GOALS
Track what you eat, keep logging it in Fitbit
If the scale goes up, look over what you did and adjust!
Check your steps and exercise levels: get OVER 150 min of mod/vig exercise a week
Sit less, break up every hour with 5min or 250 steps of movement
5. Gather your support network. Make a new accountability plan.
BB-HEALTH
TODAY’s TIP: YOU HAVE THE TOOLS. REMEMBER TO USE THEM!
You have the tools that you need for healthy lifestyle success. You have learned through trial and error what works best for you. You know how to get back on track after a slip. YOU GOT THIS!

## Slide 17
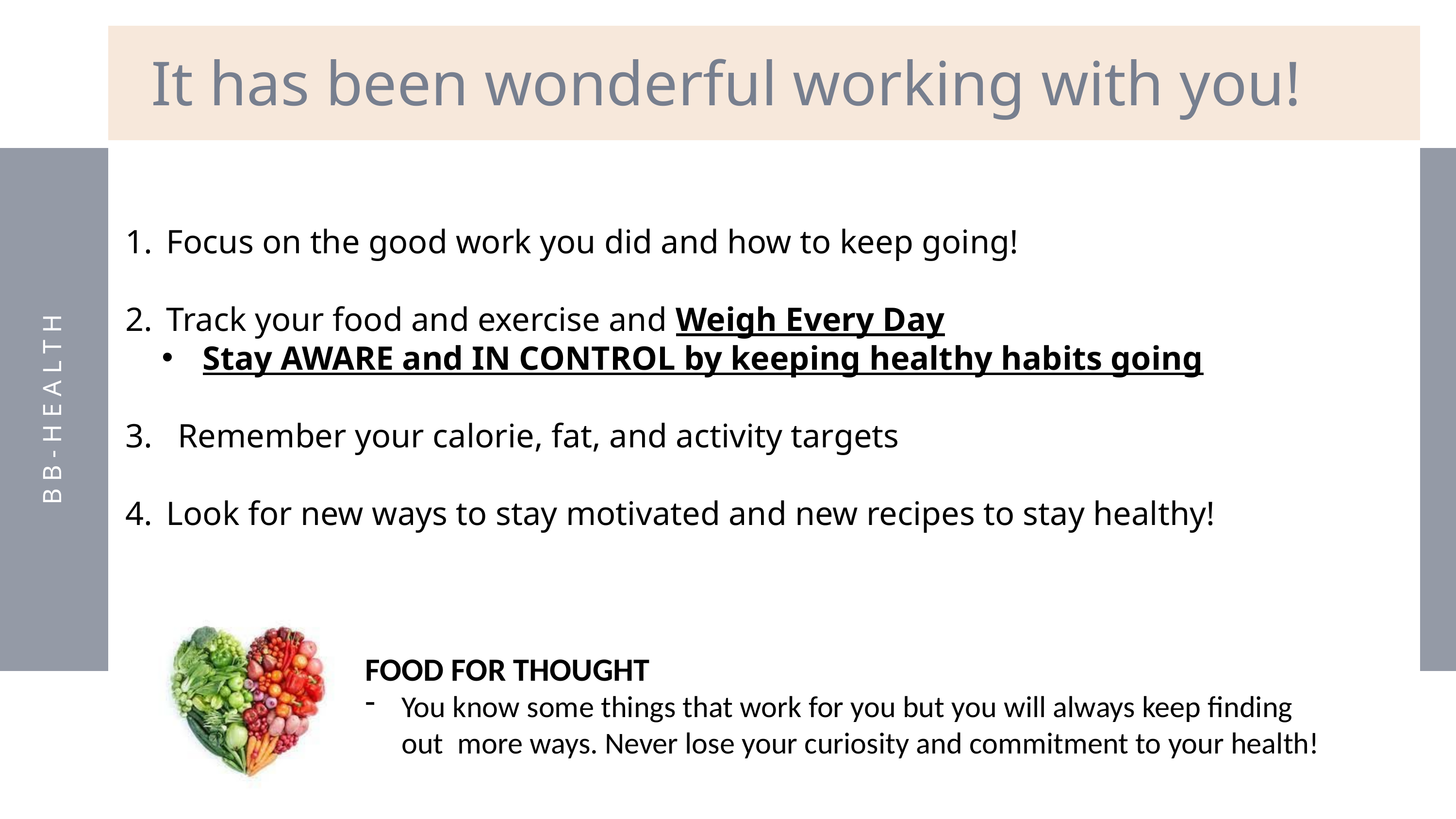

It has been wonderful working with you!
Focus on the good work you did and how to keep going!
Track your food and exercise and Weigh Every Day
Stay AWARE and IN CONTROL by keeping healthy habits going
3. Remember your calorie, fat, and activity targets
Look for new ways to stay motivated and new recipes to stay healthy!
BB-HEALTH
FOOD FOR THOUGHT
You know some things that work for you but you will always keep finding out more ways. Never lose your curiosity and commitment to your health!

## Slide 18
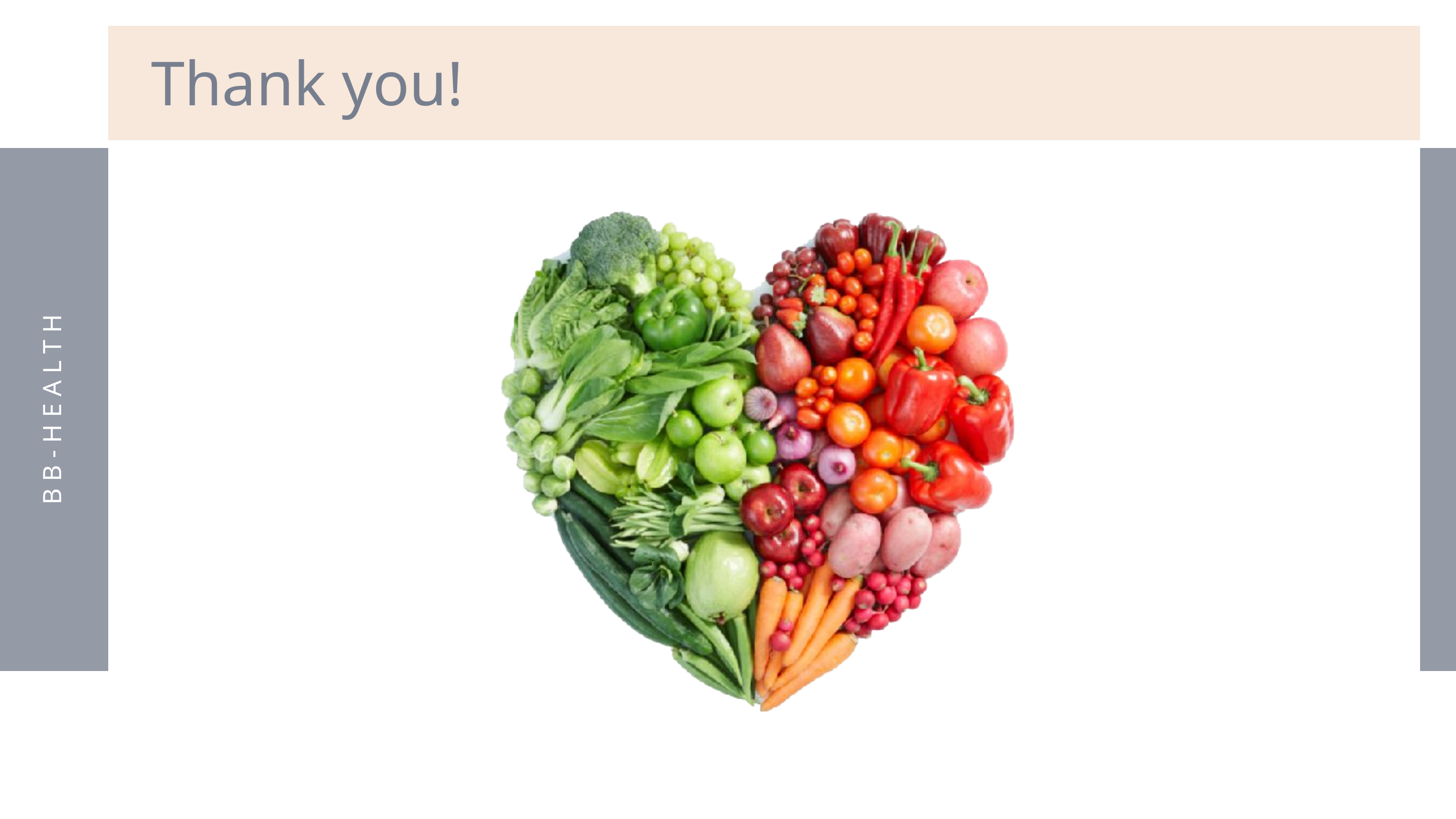

Thank you!
BB-HEALTH
