# Supplementary material for: Development and Feasibility of an eHealth Diabetes Prevention Program Adapted for Older Adults—Results from a Randomized Control Pilot Study
Source: Nutrients. 2024 Mar 23;16(7):930. doi: 10.3390/nu16070930 (PMC11154527; doi:10.3390/nu16070930)

BB-HEALTH

e-Healthy Older Adults Modification

Diabetes Prevention Program

Session #22
Looking Back,
Looking Forward!

Group Opening Discussion

BB-HEALTH

WELCOME BACK! <u>HOW WAS LAST MONTH</u>?

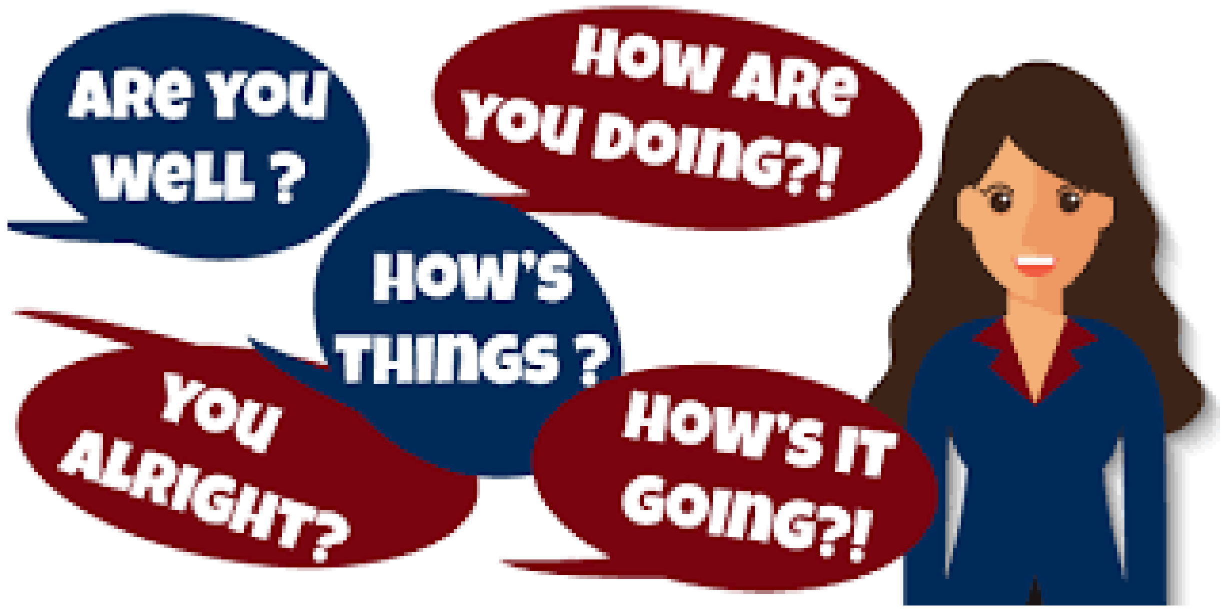

1. Did you track every meal?
2. Did you weigh every day, & record your health today?
3. Did you prioritize heart health?
4. Did you email the Intervention Team and review the response?
5. What were your unique challenges and successes?

REMINDER: CHARGE YOUR FITBIT & SCALE DURING THIS MEETING!

What supports your success?

BB-HEALTH

<u>Is being healthy an important part of who you are? (select one)</u>

- Being healthy is very important to who I am
- Being healthy is important to who I am
- Being healthy is becoming important to who I am
- Being healthy is good, but not who I am
- Being healthy is not important to who I am
- Being healthy is completely unimportant to me

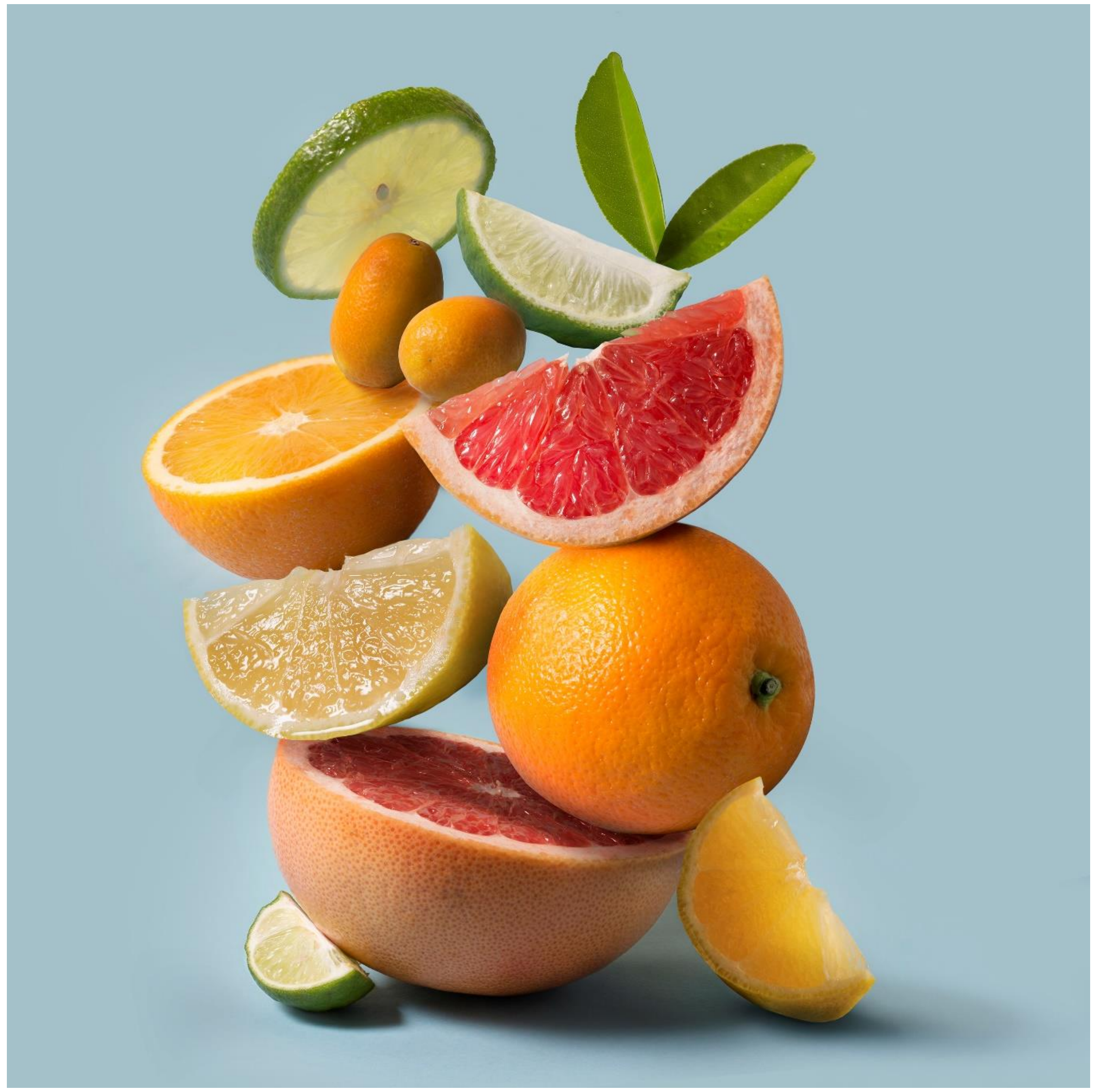

Do you prioritize your health?

BB-HEALTH

Would you describe yourself as someone who PRIORITIZES healthy choices to support your lifestyle (Select one)

- Always, my health comes first
- Most of the time
- A fair amount of the time
- Some of the time
- Less than half of the time
- Occasionally
- Rarely
- Never

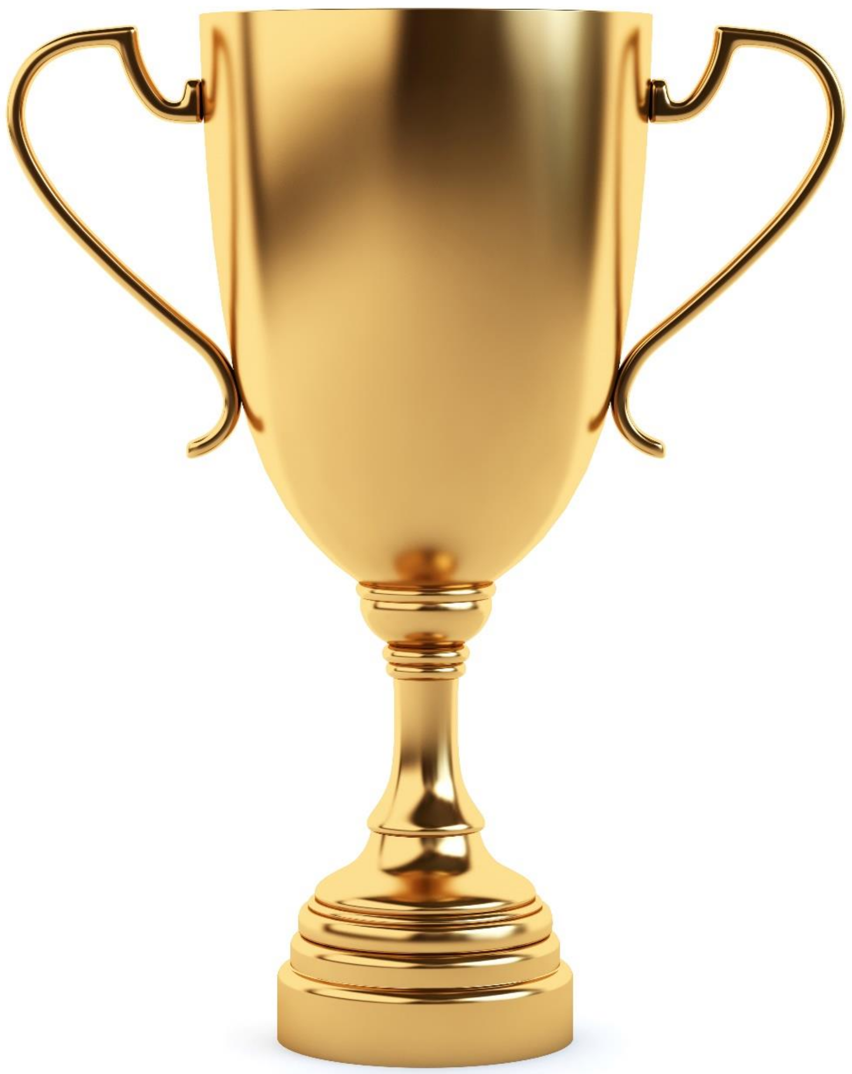

What does success look like?

BB-HEALTH

<u>What makes you feel like it is working? (select all)</u>

- ○ Sticking to my calorie/fat targets most meals
- ○ Losing weight
- ○ Having one really healthy meal a day
- ○ Doing my 6,000 steps every day
- ○ Having more energy
- ○ Trying new foods
- ○ My clothes fit better
- ○ Skipping junk food
- ○ My family or friends notice improvements
- ○ Getting my steps in on most days
- ○ Other (discuss)

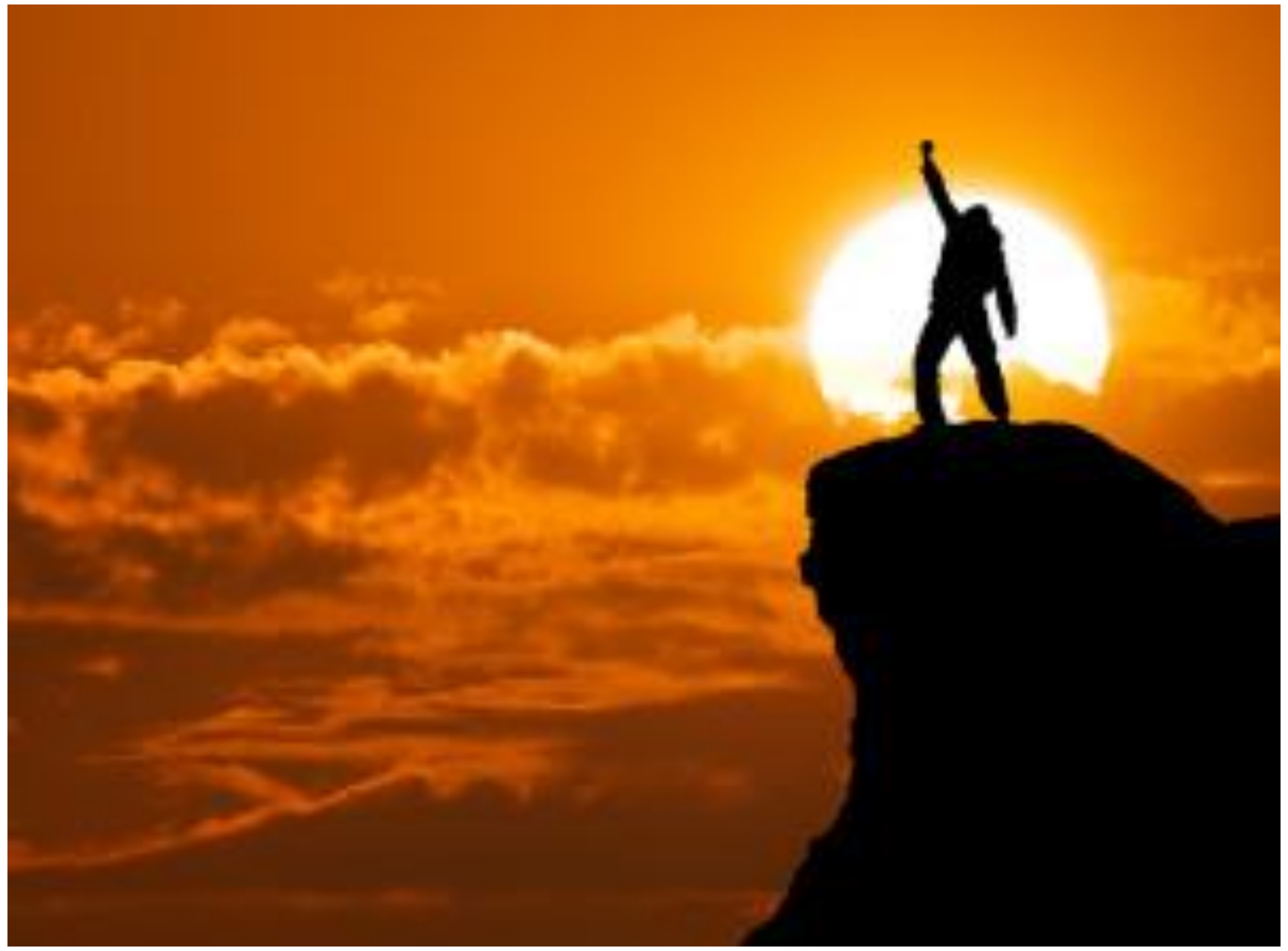

Skills you learned

B B - H E A L T H

AWARE

- Weighing everyday and logging calories
- Trying different approaches
- Accountability AND forgiveness
- Finding small things that make big problems

IN CONTROL

- Working at it, even when it’s hard
- Knowing what works for you
- Making action plan
- Forgiving, but not Forgetting
- Finding the healthy things you ENJOY
- Letting go of PERFECT
- Not relying on willpower alone
- Self-advocating
- Taking small steps to support big improvements

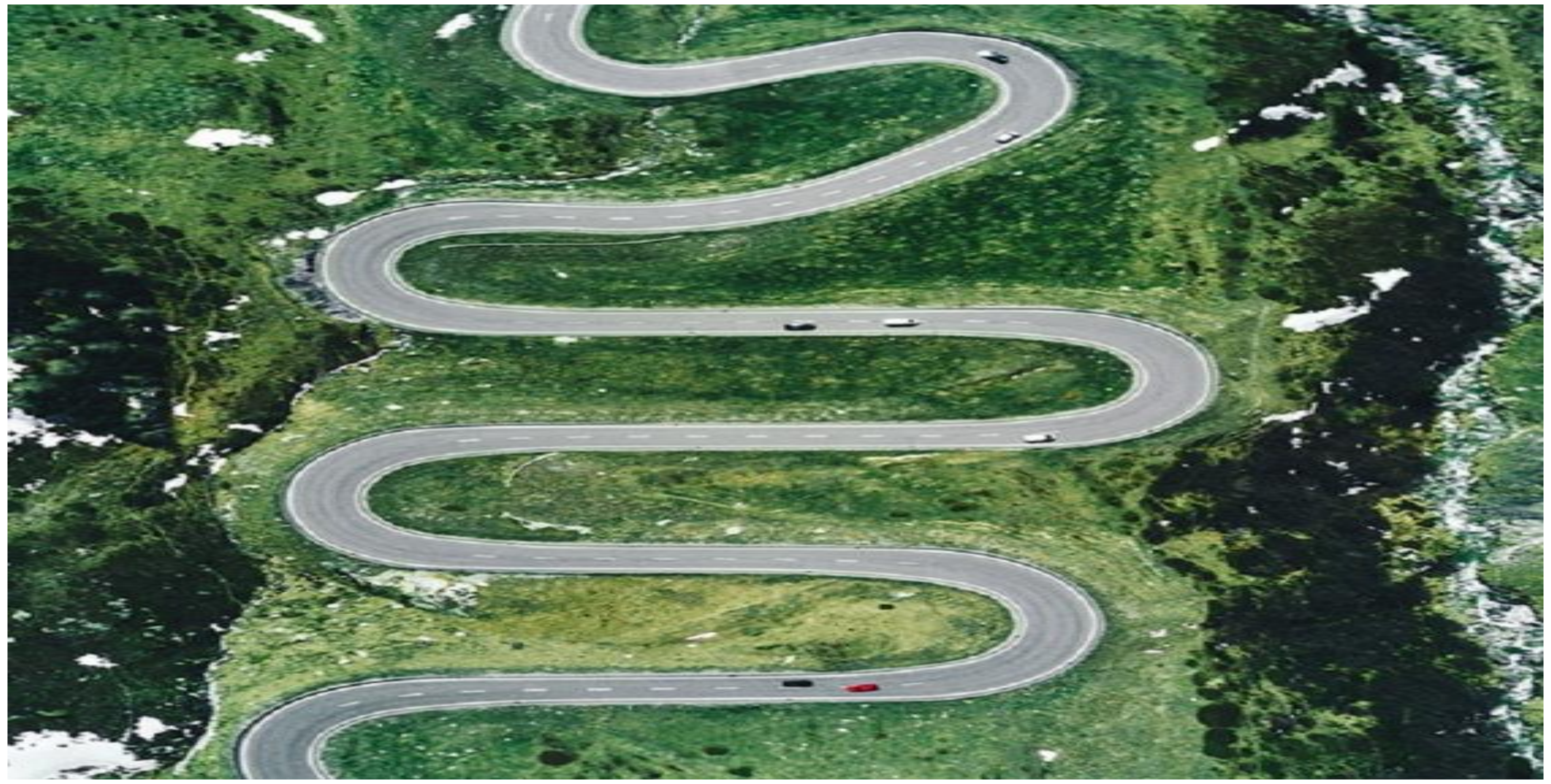

The road to health with lifestyle is a long and windy one, but it also means it is a slow, comfortable and gradual path you can follow time and time again.

CONGRATULATIONS!

B B - H E A L T H

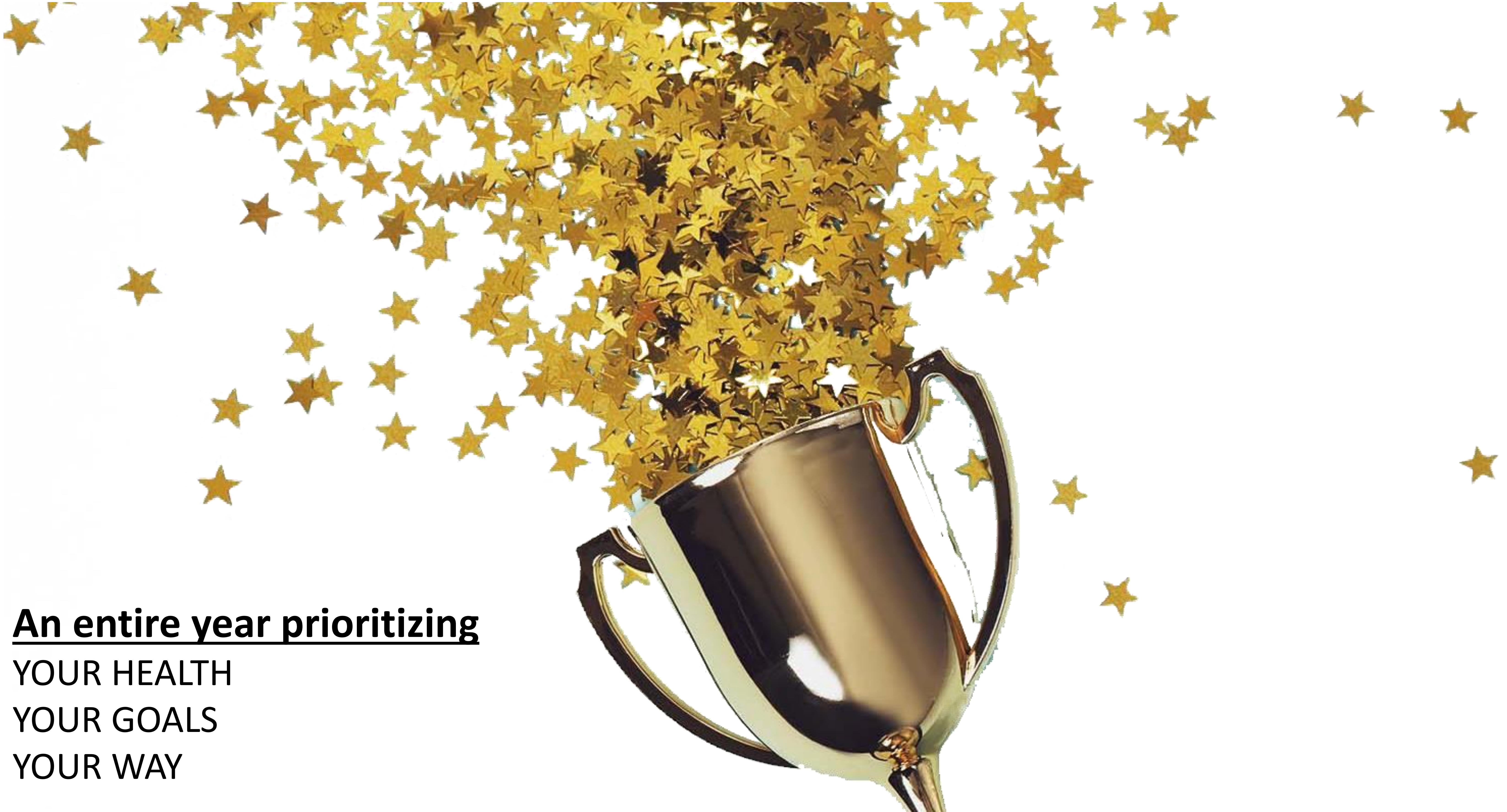

<u>An entire year prioritizing</u>

YOUR HEALTH
YOUR GOALS
YOUR WAY

Your story, your roadmap

- What change strategy worked best for you?
- What are you most proud of?
- If a friend was struggling with success, what words of wisdom would you share?
- **What do you want to remember for yourself, 6 months from now, to help you keep maintaining your success?**

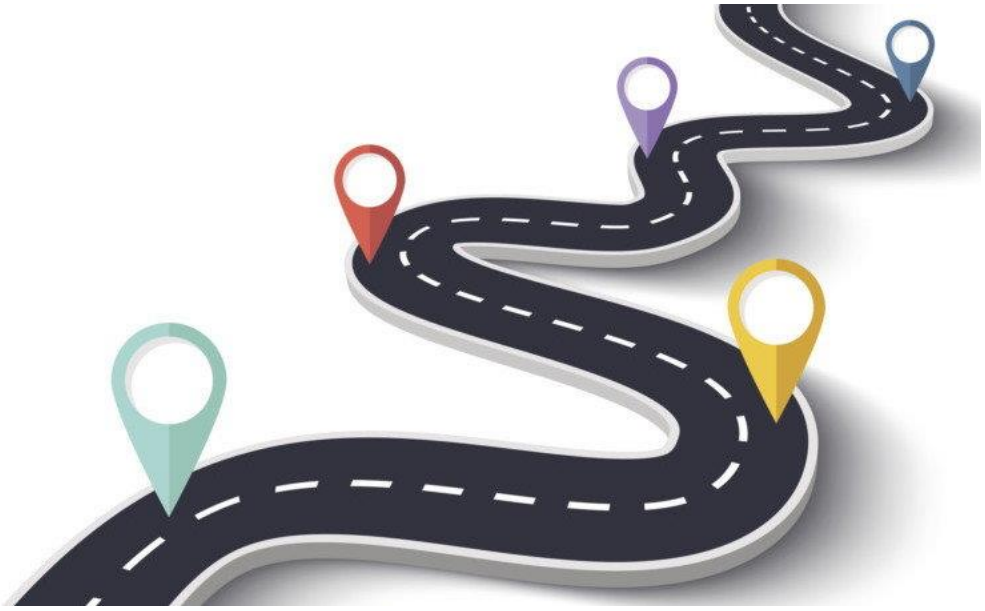

Share your journey milestones out loud, or in the chat!

How do you feel?

<u>**How do you feel about your current health level? (select one)**</u>

- My health is not good currently
- My health is ok
- My health is mostly good
- My health is good
- My health is very good

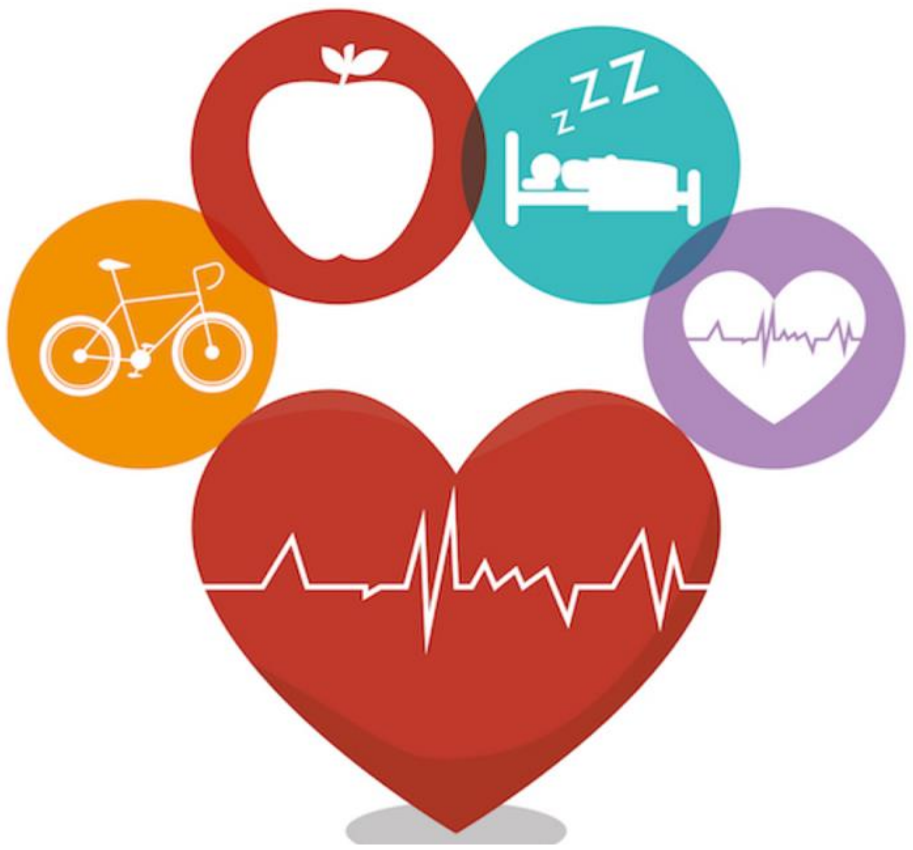

How do you feel?

BB-HEALTH

How do you feel your weight changed? (select one)

- o My weight is much worse
- o My weight is worse
- o My weight hasn't improved
- o My weight is fluctuating
- o My weight has gotten better
- o My weight has gotten much better

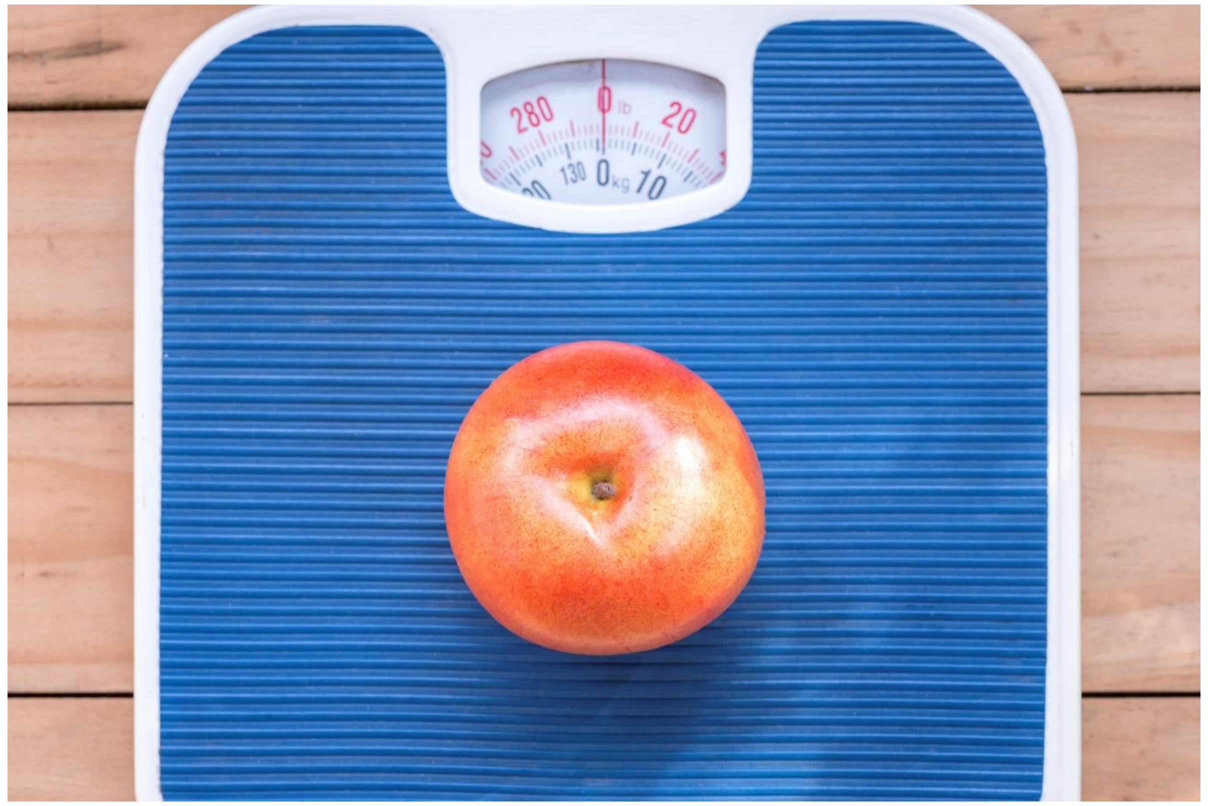

How do you feel?

How do you feel your activity level has improved? (select one)

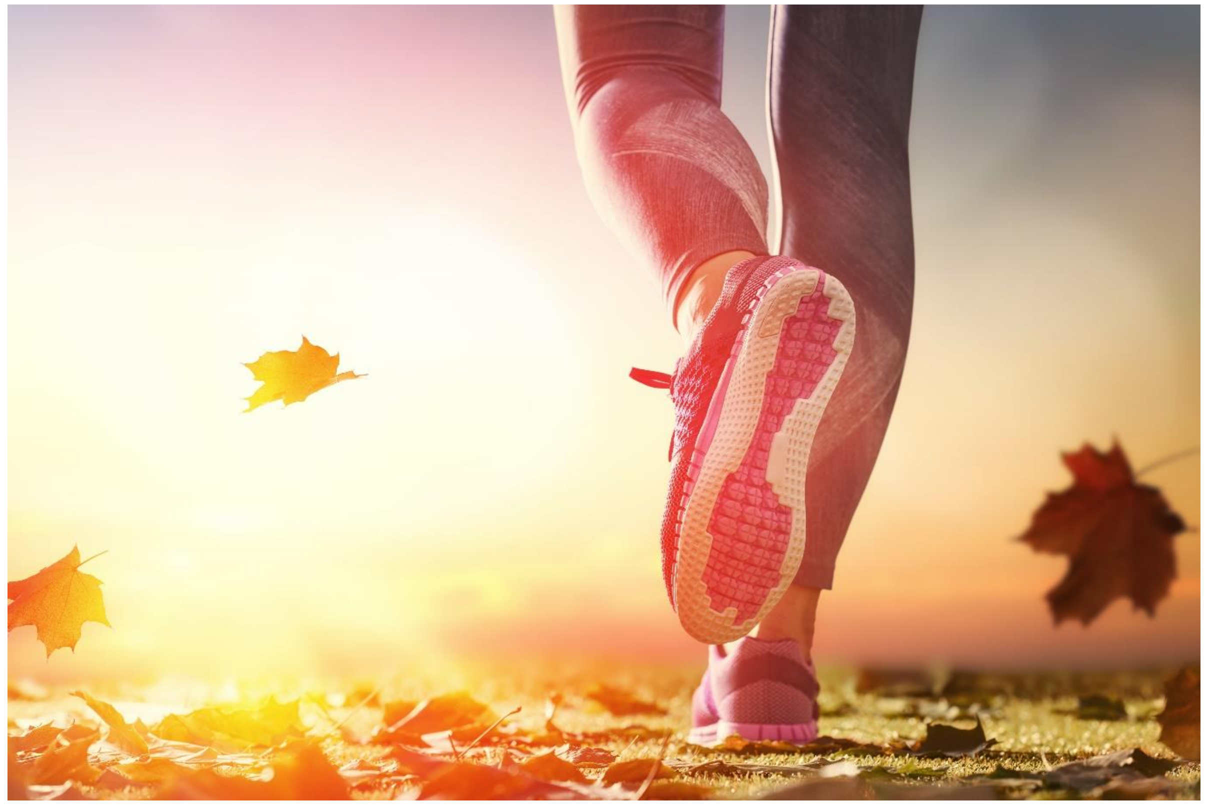

- ○ My activity level is much lower than when I started
- ○ My activity level is lower than when I started
- ○ My activity level is about the same
- ○ My activity level is higher than when I started
- ○ My activity level is much higher than when I started

Fork in the road

BB-HEALTH

<u>In the next 6 months what will you focus on?</u>

- Reaching my weight goal
- Maintaining my weight
- Increasing my activity level
- Maintaining my activity level
- Eating healthy
- Eliminating unhealthy cues
- Setting up new accountability systems
- Other (DISCUSS)

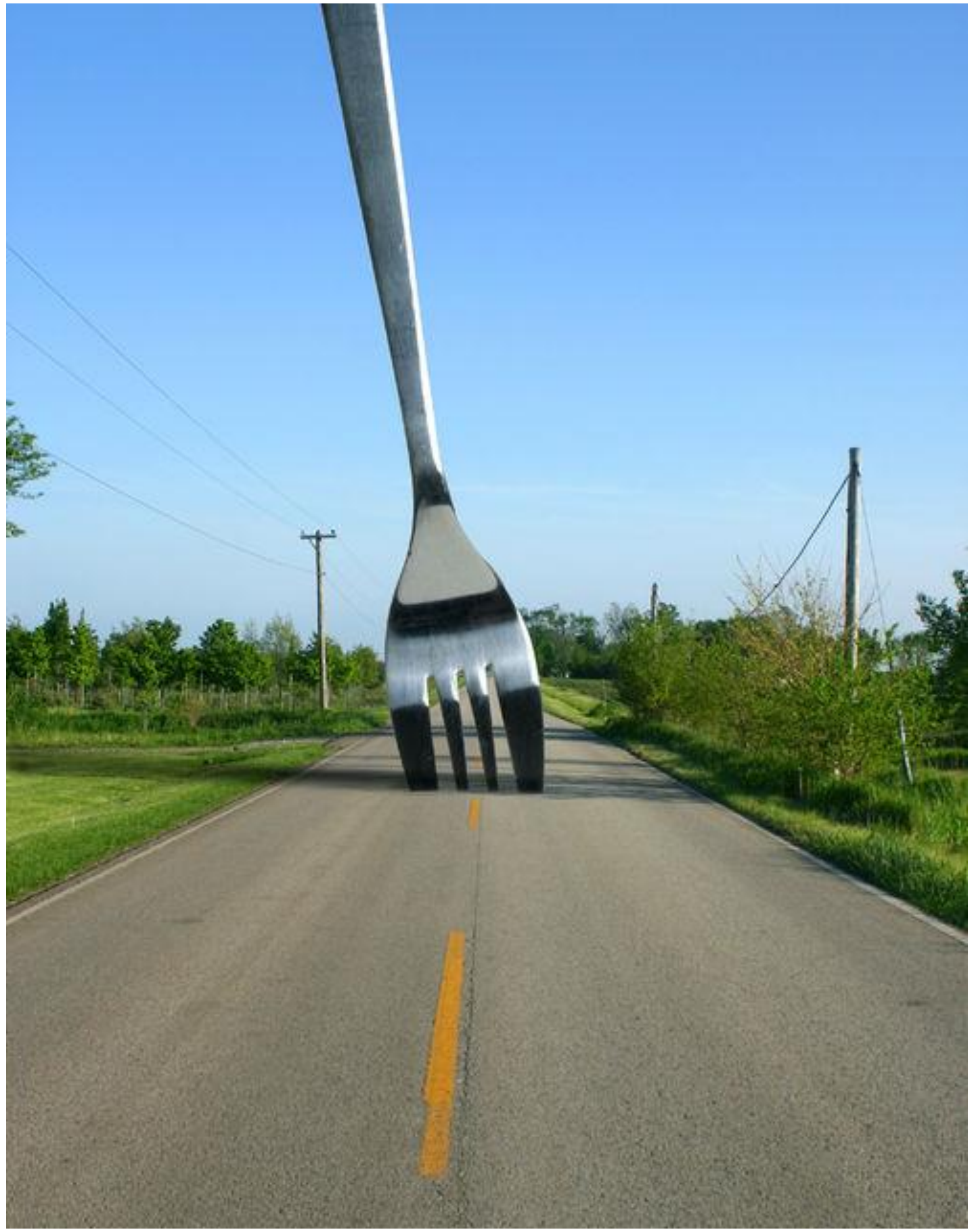

What motivates you to keep aiming for success?

Where does your motivation come from? (select all)

- Play with my grandchildren
- Keep up with friends
- To enjoy my life
- Less time at the doctor's office
- To feel good about myself
- To prove I can do what I set my mind to
- Other (discuss)

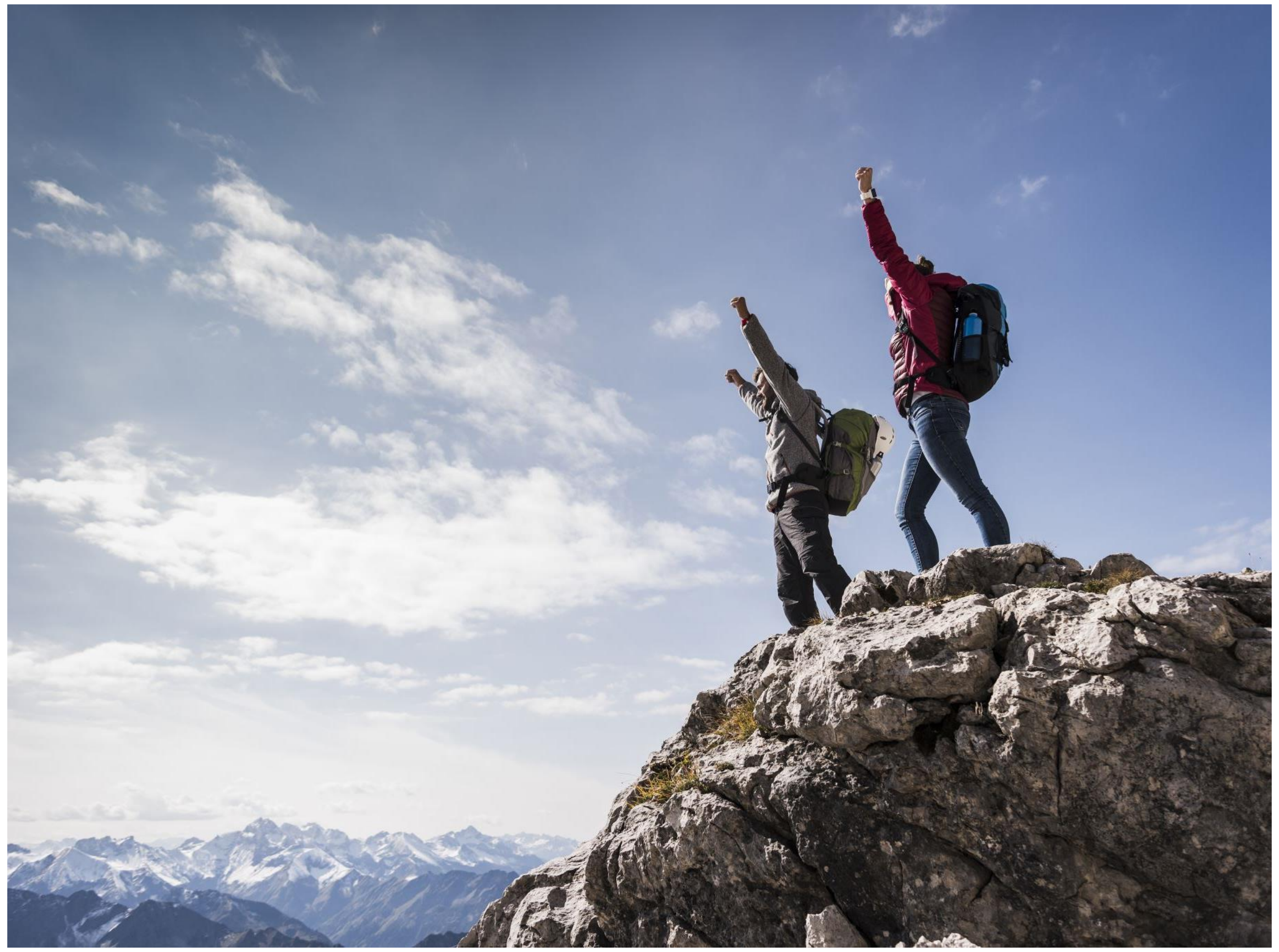

From lifestyle program, to LIFE

BB-HEALTH

What does healthy feel like to you? (select all)

- I have more energy
- I sleep better, and wake rested
- I am less hungry
- I have had a better mood
- My family says they notice the difference
- My clothes fit better
- Less craving/snacking
- Better lab results at the doctor
- Other (discuss)

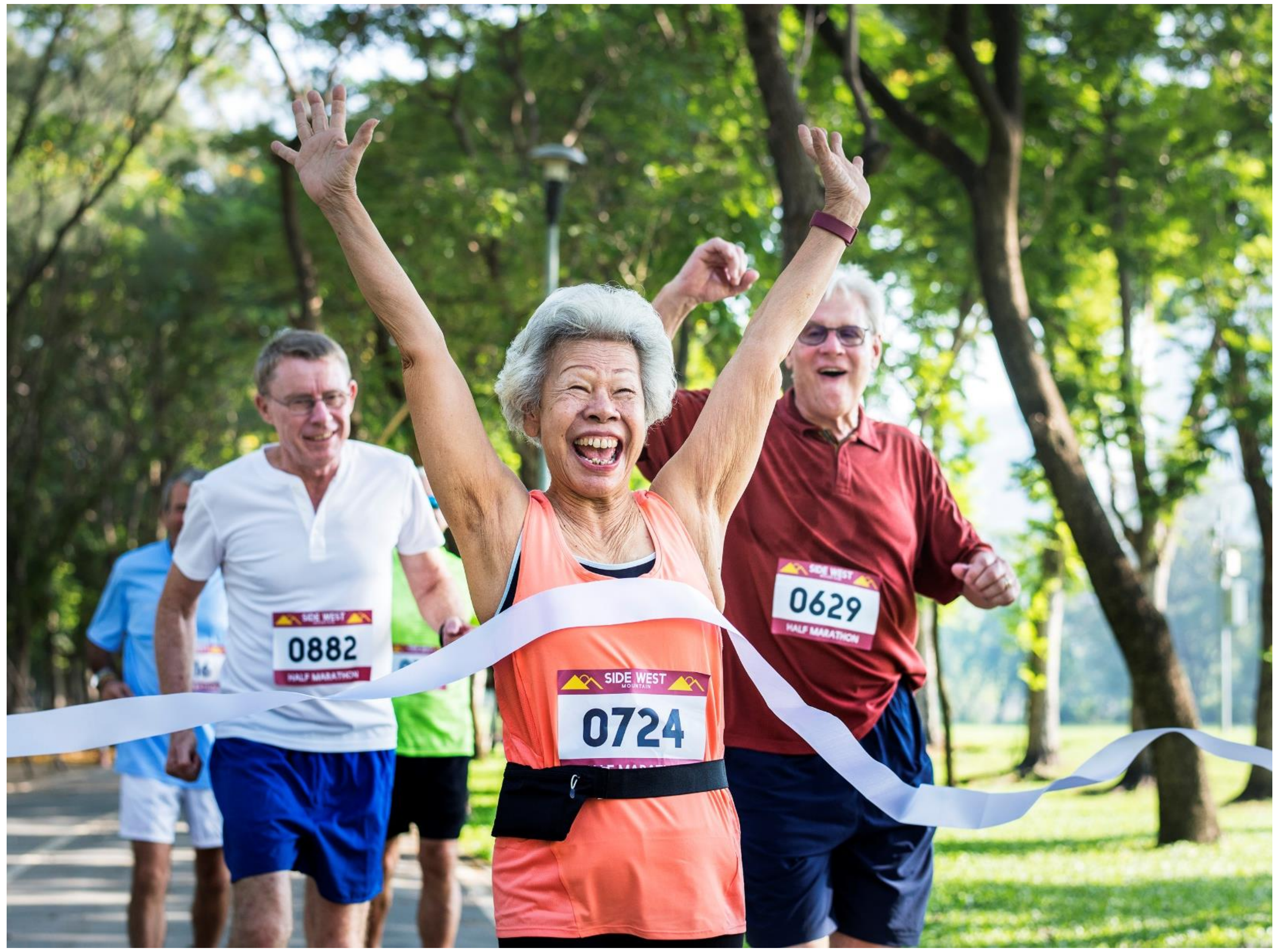

From lifestyle program, to LIFE

What will you do to tip the calorie balance going forward? (select all)

- Drink more water
- Start every meal with a salad
- Take the long way back
- Jog in place during commercial breaks
- Make a walking date with a friend
- Take an extra loop or two in the grocery store
- Cut out beverage calories
- Try a new recipe
- Log my calories and staying on my calorie target
- Get my steps everyday
- Weigh myself everyday
- Other (discuss)

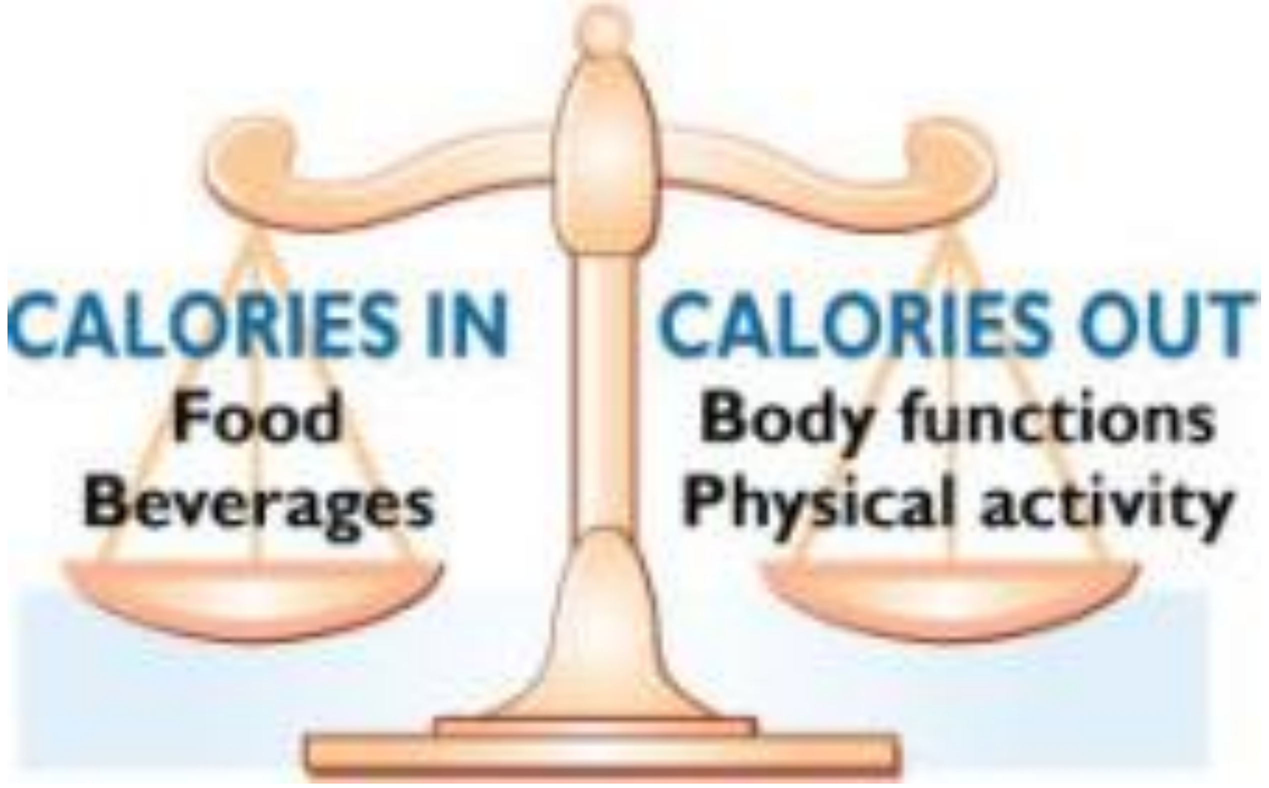

KEEP USING THESE SKILLS!

BB-HEALTH

1. Weigh everyday! Make pace with the scale
   - Monitor your progress and identify areas for improvement towards YOUR GOALS

2. Track what you eat, keep logging it in Fitbit
   - If the scale goes up, look over what you did and adjust!

3. Check your steps and exercise levels: get OVER 150 min of mod/vig exercise ***a week***

4. Sit less, break up every hour with 5min or 250 steps of movement

5. Gather your support network. Make a new accountability plan.

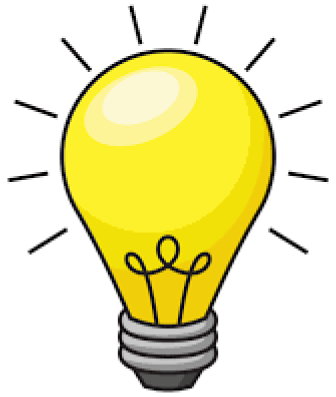

TODAY's TIP: ***YOU HAVE THE TOOLS. REMEMBER TO USE THEM!***
You have the tools that you need for healthy lifestyle success. You have learned through trial and error what works best for you. You know how to get back on track after a slip. YOU GOT THIS!

It has been wonderful working with you!

B B - H E A L T H

1. Focus on the good work you did and how to keep going!

2. Track your food and exercise and **<u>Weigh Every Day</u>**
   - **<u>Stay AWARE and IN CONTROL by keeping healthy habits going</u>**

3. Remember your calorie, fat, and activity targets

4. Look for new ways to stay motivated and new recipes to stay healthy!

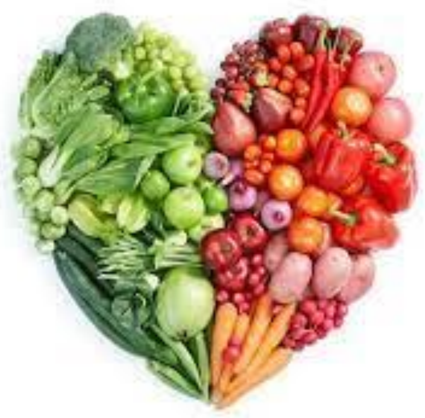

FOOD FOR THOUGHT

- You know some things that work for you but you will always keep finding out more ways. Never lose your curiosity and commitment to your health!

Thank you!

B B - H E A L T H

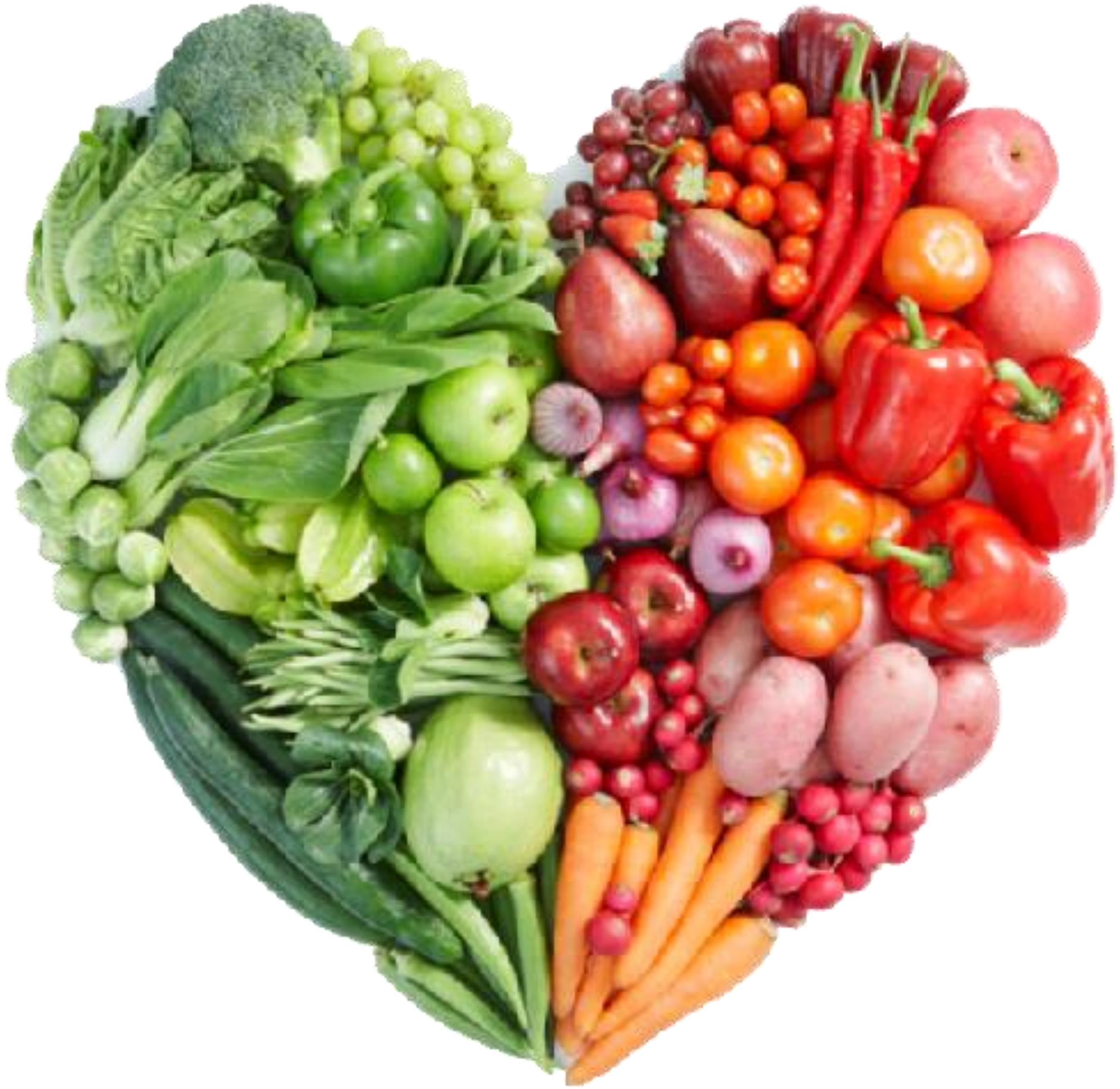

Supplement: Supplementary file 1 [file nutrients-16-00930-s001.zip › Session 22_SUPP.pdf]
