# Supplementary material for: Development and Feasibility of an eHealth Diabetes Prevention Program Adapted for Older Adults—Results from a Randomized Control Pilot Study
Source: Nutrients. 2024 Mar 23;16(7):930. doi: 10.3390/nu16070930 (PMC11154527; doi:10.3390/nu16070930)
Supplement: Supplementary file 1 [file nutrients-16-00930-s001.zip › Session13.pptx]

## Slide 1
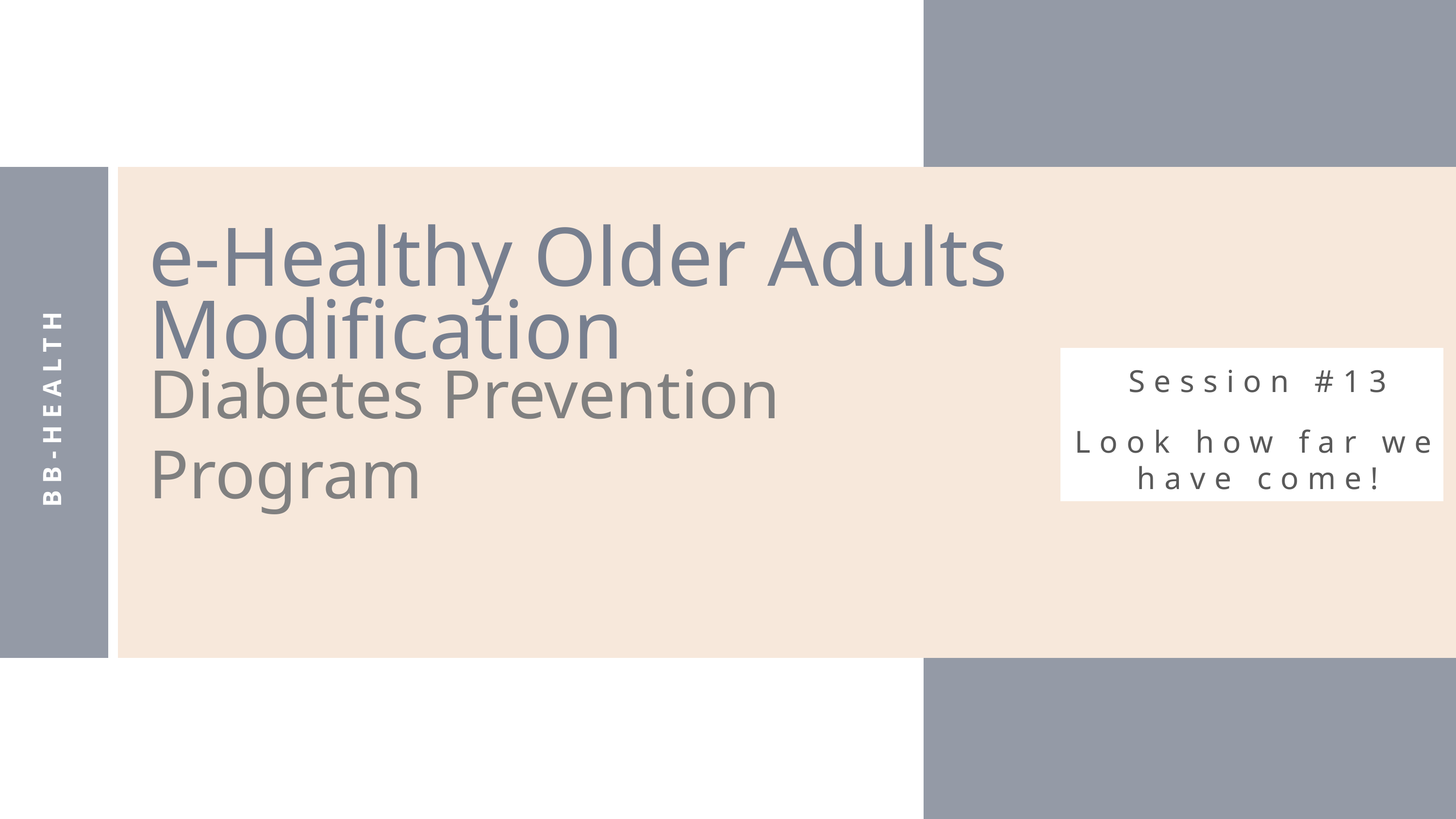

OPEN REPORTS
e-Healthy Older Adults Modification
Session #13
Look how far we have come!
Diabetes Prevention Program
BB-HEALTH

## Slide 2
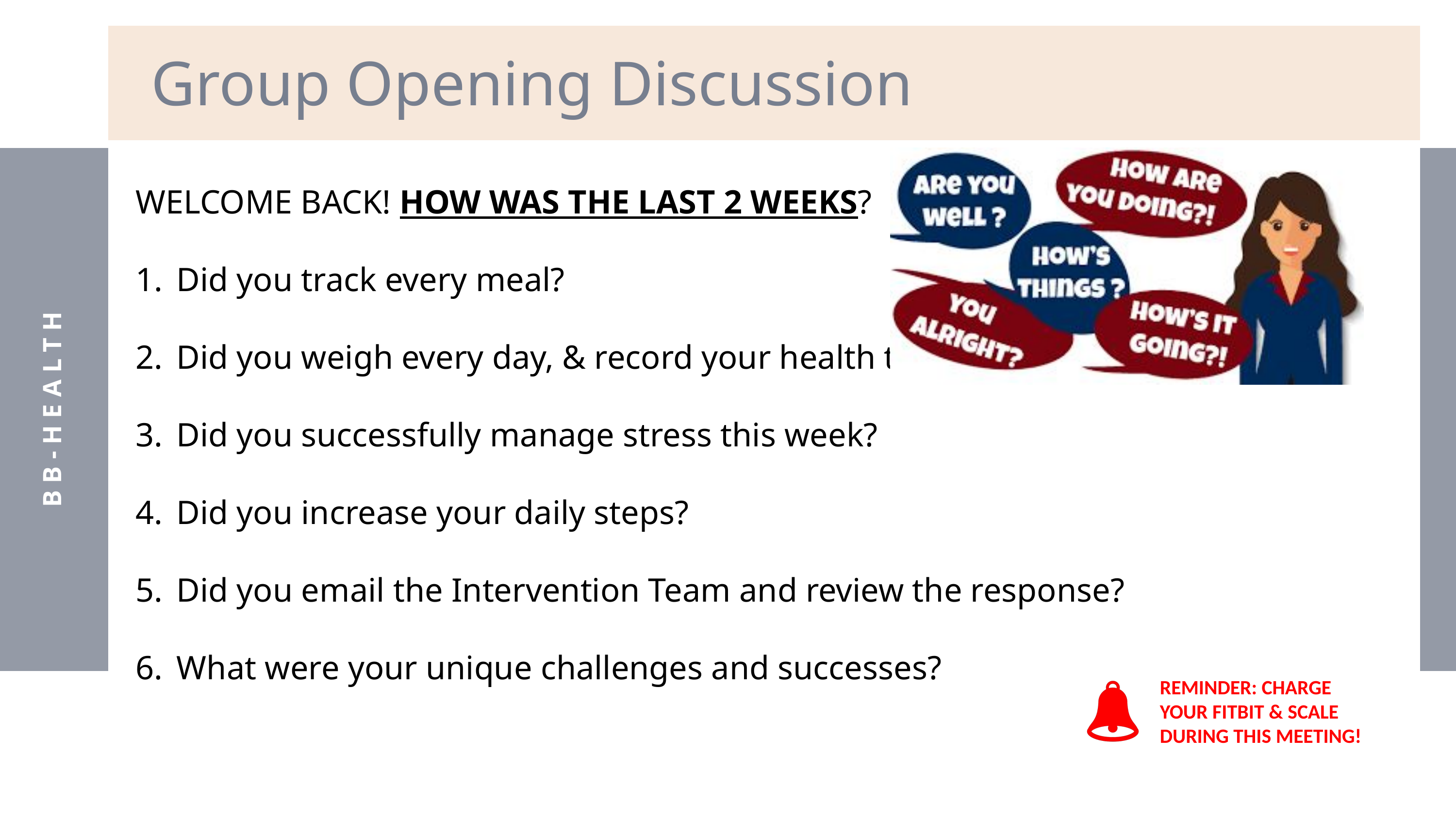

Group Opening Discussion
WELCOME BACK! HOW WAS THE LAST 2 WEEKS?
Did you track every meal?
Did you weigh every day, & record your health today?
Did you successfully manage stress this week?
Did you increase your daily steps?
Did you email the Intervention Team and review the response?
What were your unique challenges and successes?
BB-HEALTH
REMINDER: CHARGE YOUR FITBIT & SCALE DURING THIS MEETING!

## Slide 3
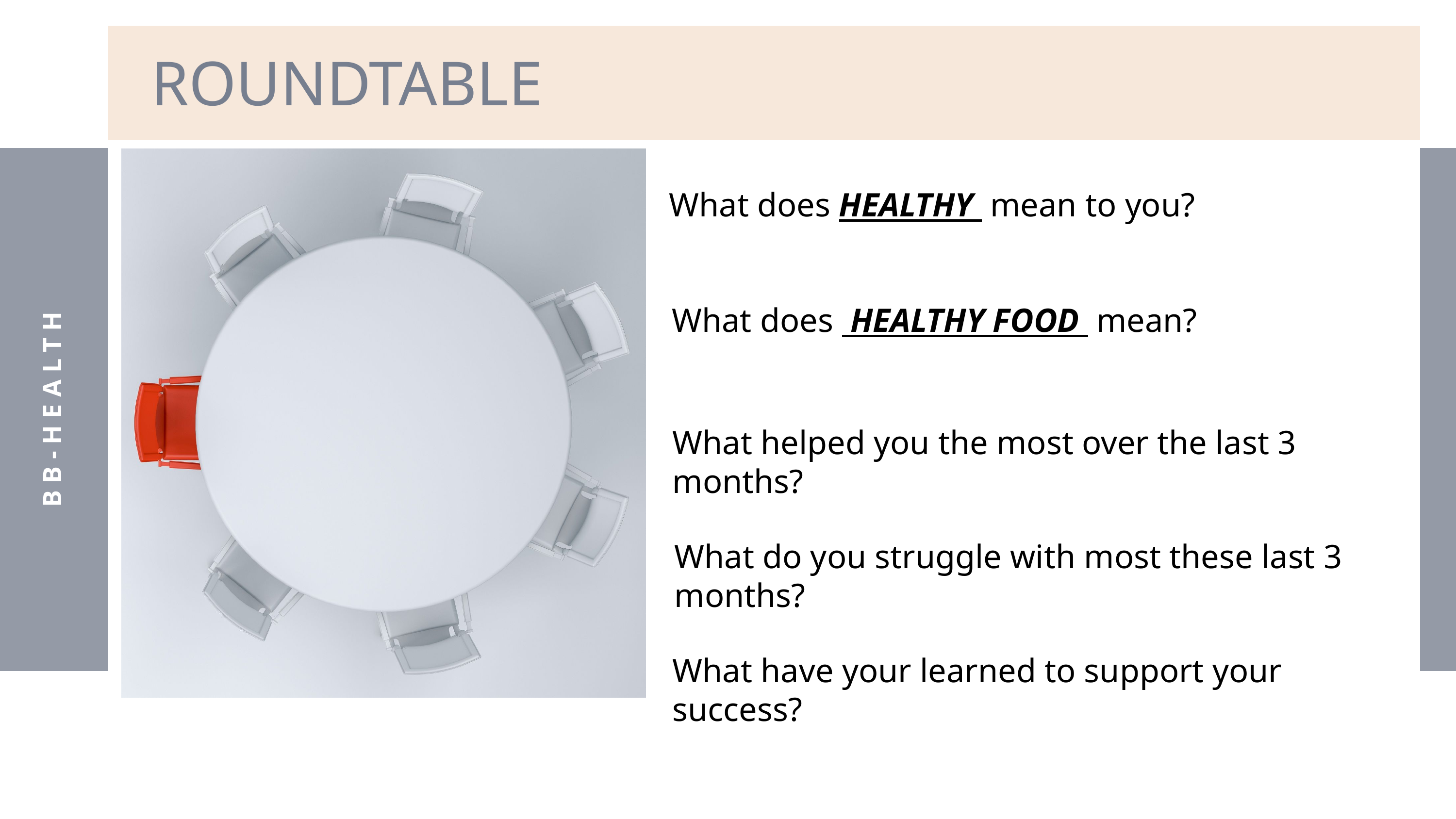

ROUNDTABLE
What does HEALTHY mean to you?
What does HEALTHY FOOD mean?
BB-HEALTH
What helped you the most over the last 3 months?
What do you struggle with most these last 3 months?
What have your learned to support your success?

## Slide 4
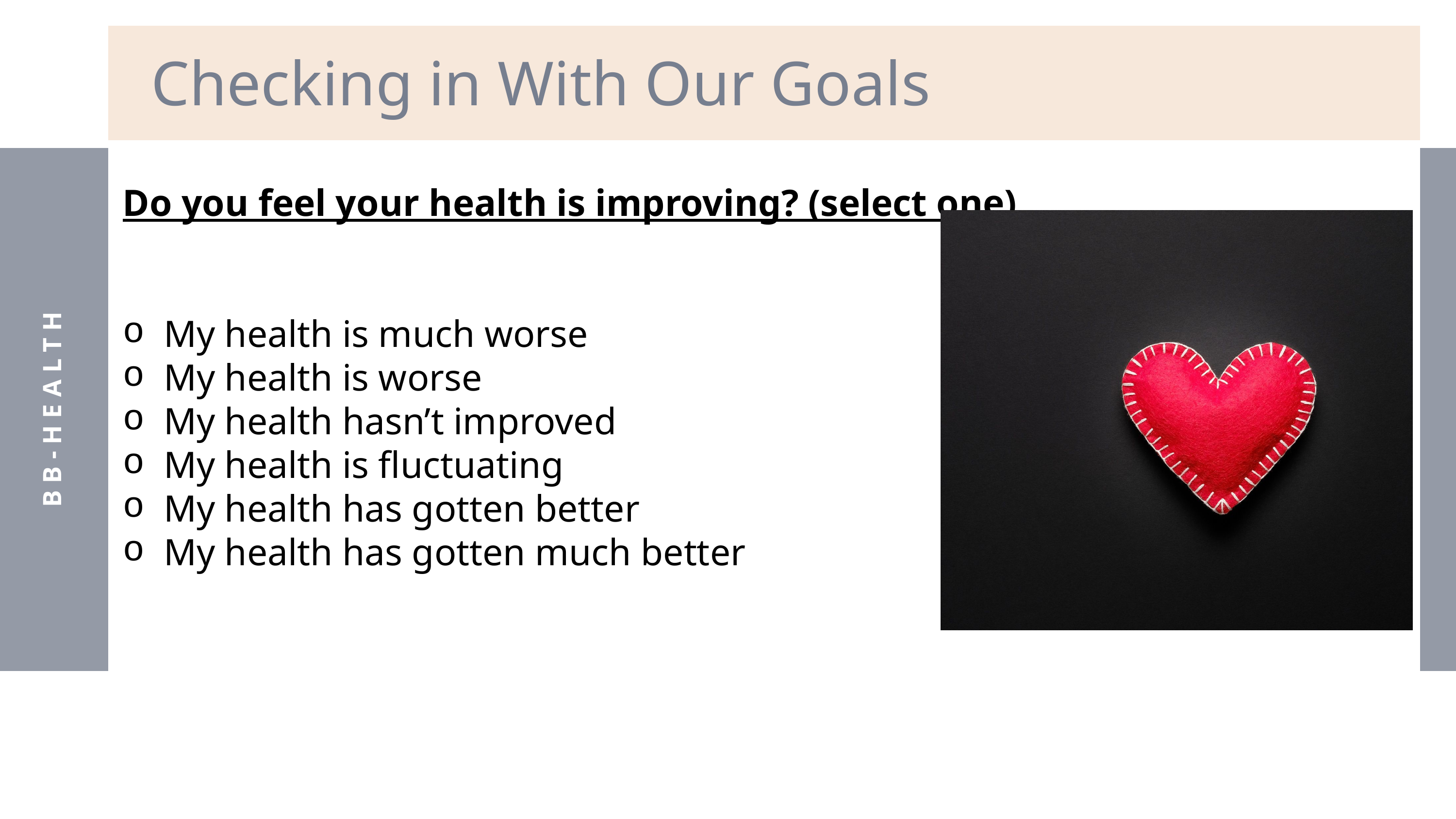

Checking in With Our Goals
Do you feel your health is improving? (select one)
My health is much worse
My health is worse
My health hasn’t improved
My health is fluctuating
My health has gotten better
My health has gotten much better
BB-HEALTH

## Slide 5
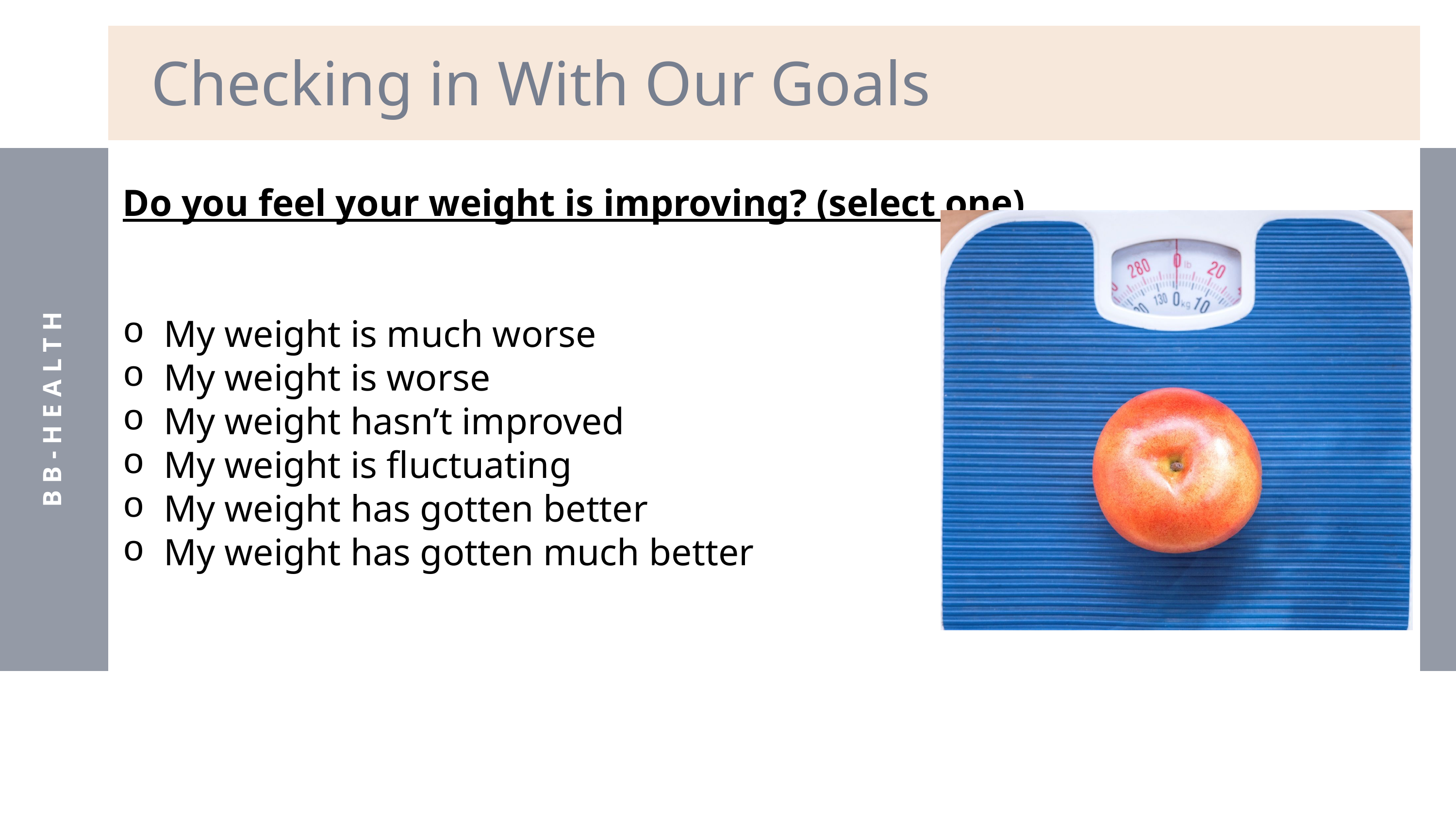

Checking in With Our Goals
Do you feel your weight is improving? (select one)
My weight is much worse
My weight is worse
My weight hasn’t improved
My weight is fluctuating
My weight has gotten better
My weight has gotten much better
BB-HEALTH

## Slide 6
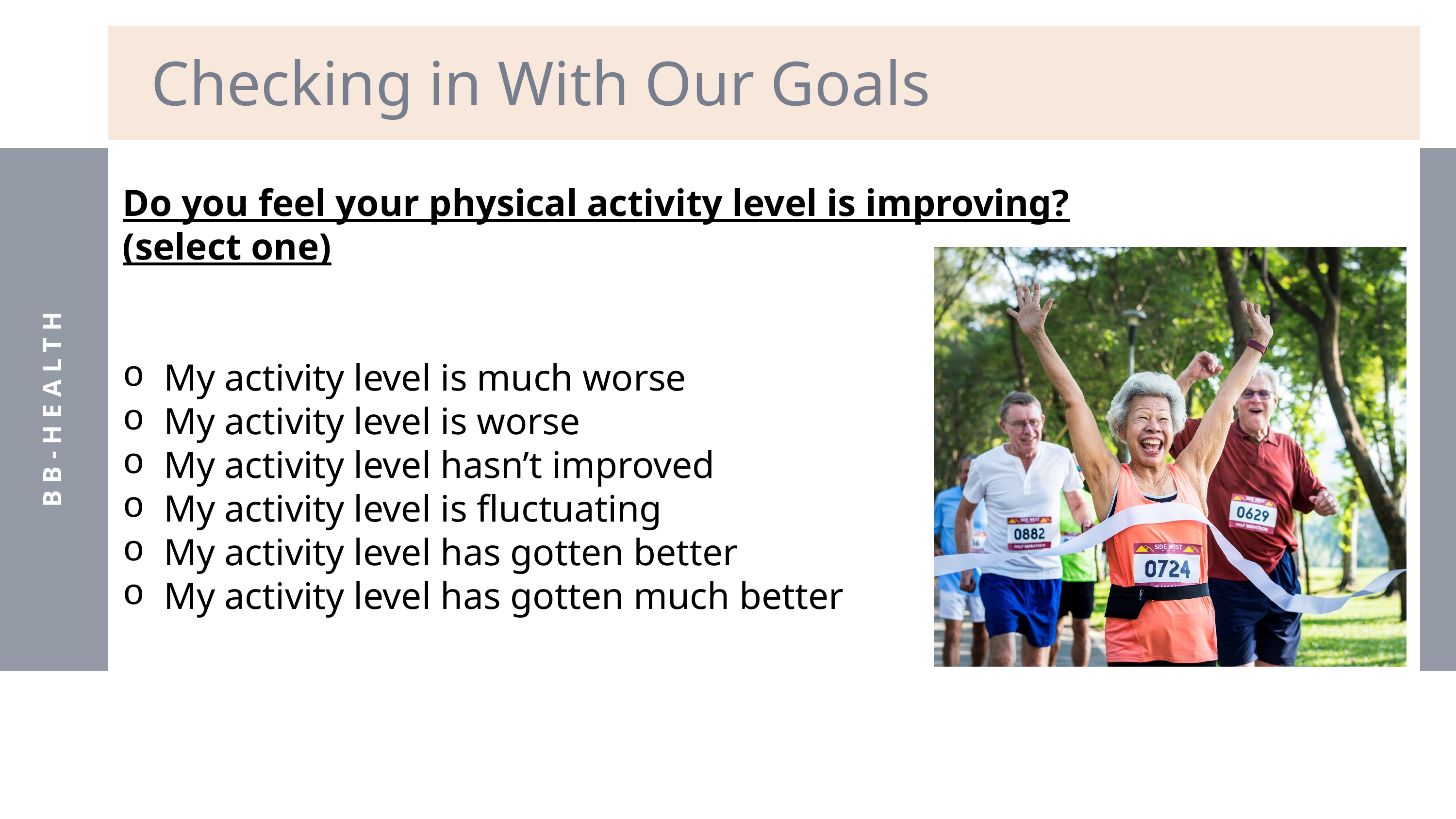

Checking in With Our Goals
Do you feel your physical activity level is improving? (select one)
My activity level is much worse
My activity level is worse
My activity level hasn’t improved
My activity level is fluctuating
My activity level has gotten better
My activity level has gotten much better
BB-HEALTH

## Slide 7
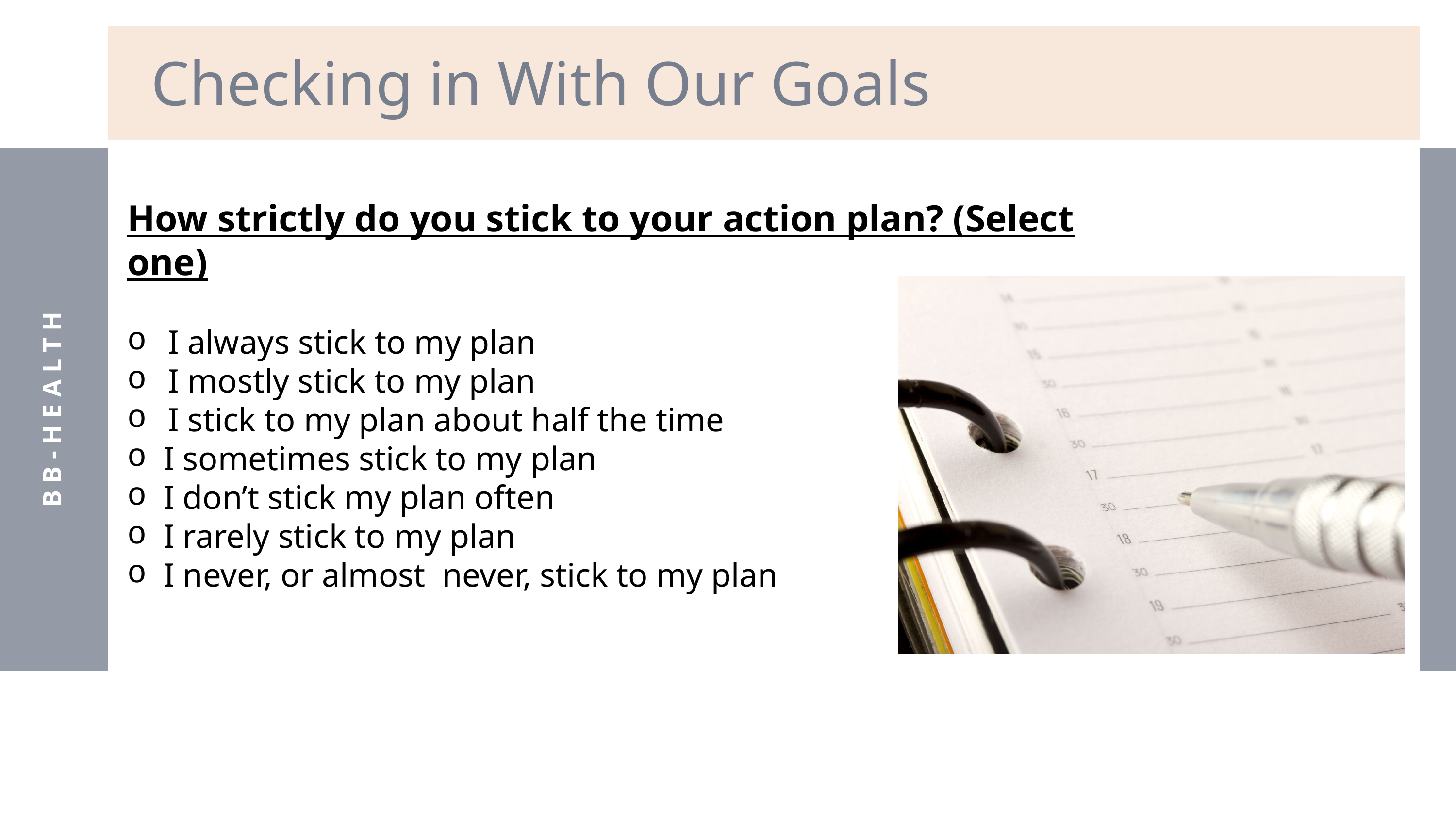

Checking in With Our Goals
How strictly do you stick to your action plan? (Select one)
I always stick to my plan
I mostly stick to my plan
I stick to my plan about half the time
I sometimes stick to my plan
I don’t stick my plan often
I rarely stick to my plan
I never, or almost never, stick to my plan
BB-HEALTH

## Slide 8
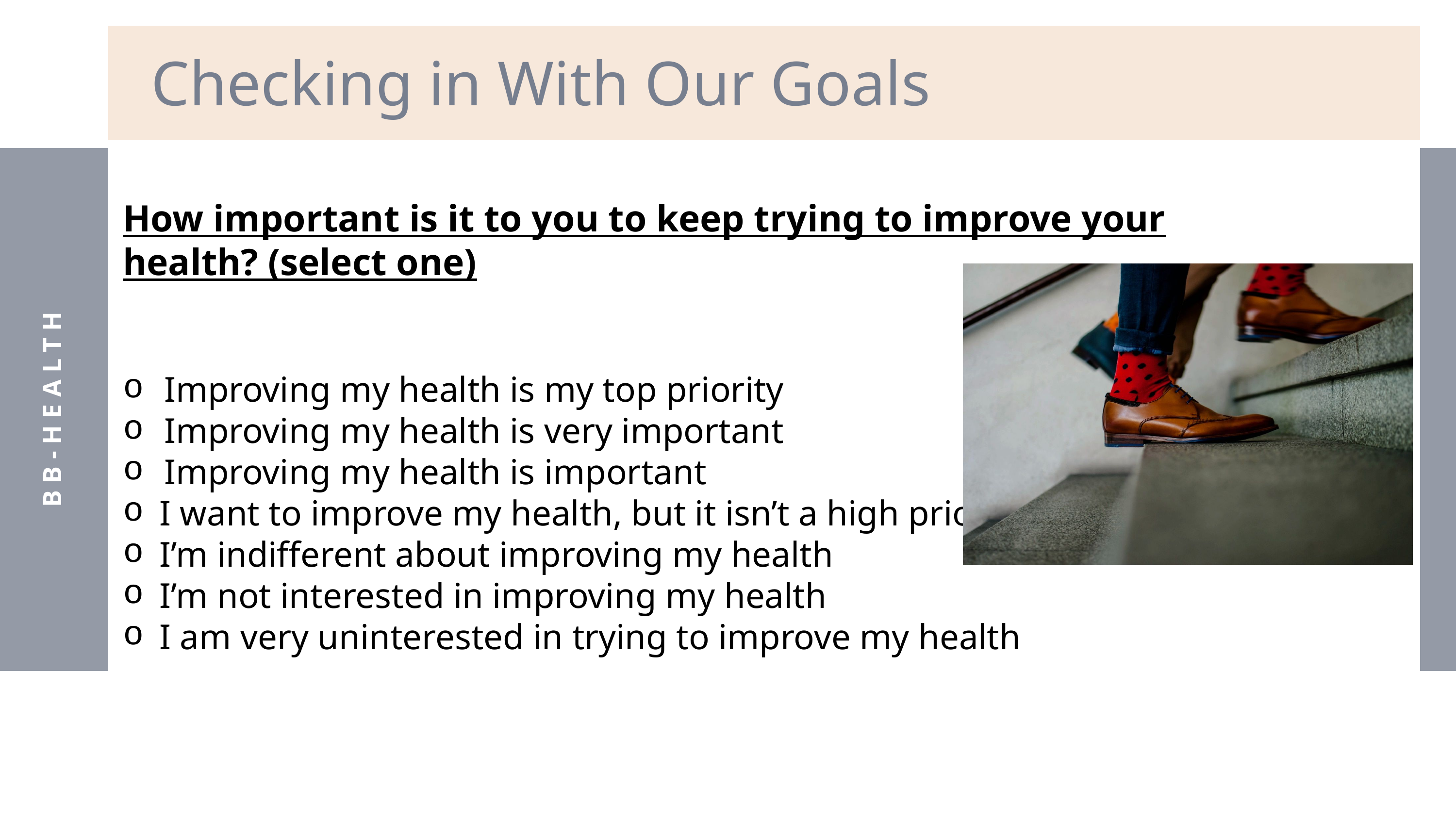

Checking in With Our Goals
How important is it to you to keep trying to improve your health? (select one)
Improving my health is my top priority
Improving my health is very important
Improving my health is important
I want to improve my health, but it isn’t a high priority
I’m indifferent about improving my health
I’m not interested in improving my health
I am very uninterested in trying to improve my health
BB-HEALTH

## Slide 9
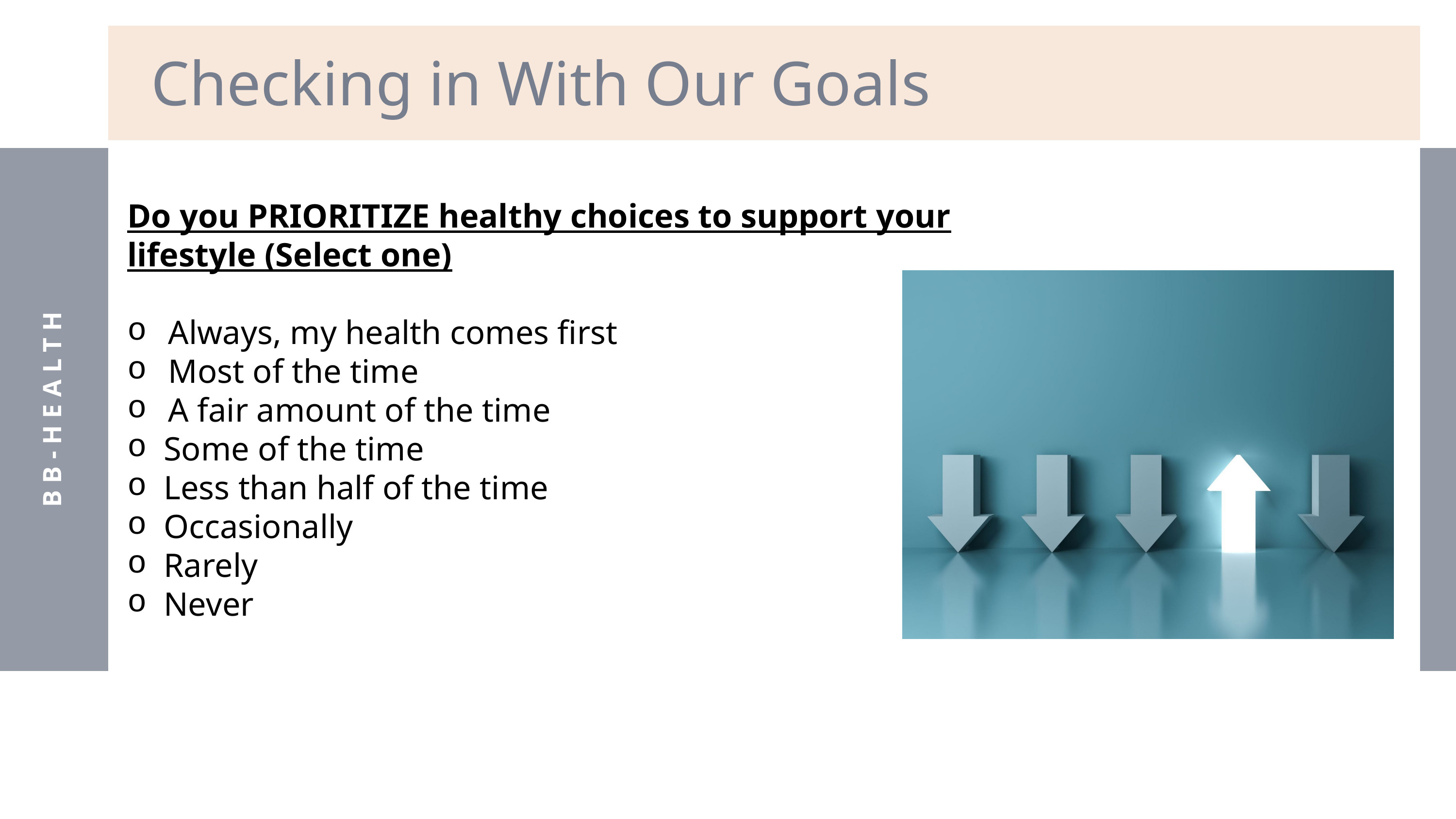

Checking in With Our Goals
Do you PRIORITIZE healthy choices to support your lifestyle (Select one)
Always, my health comes first
Most of the time
A fair amount of the time
Some of the time
Less than half of the time
Occasionally
Rarely
Never
BB-HEALTH

## Slide 10
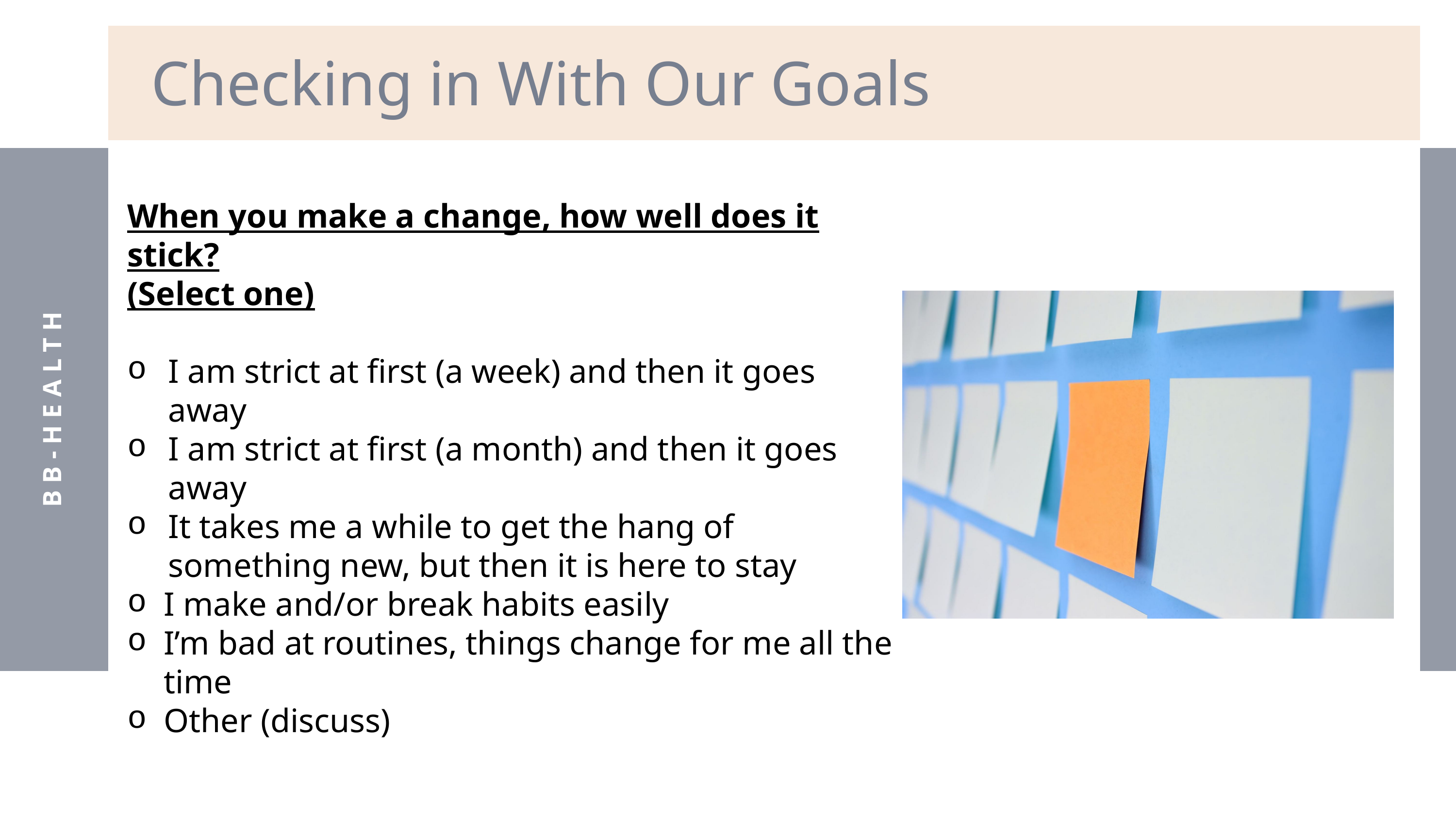

Checking in With Our Goals
When you make a change, how well does it stick?
(Select one)
I am strict at first (a week) and then it goes away
I am strict at first (a month) and then it goes away
It takes me a while to get the hang of something new, but then it is here to stay
I make and/or break habits easily
I’m bad at routines, things change for me all the time
Other (discuss)
BB-HEALTH

## Slide 11
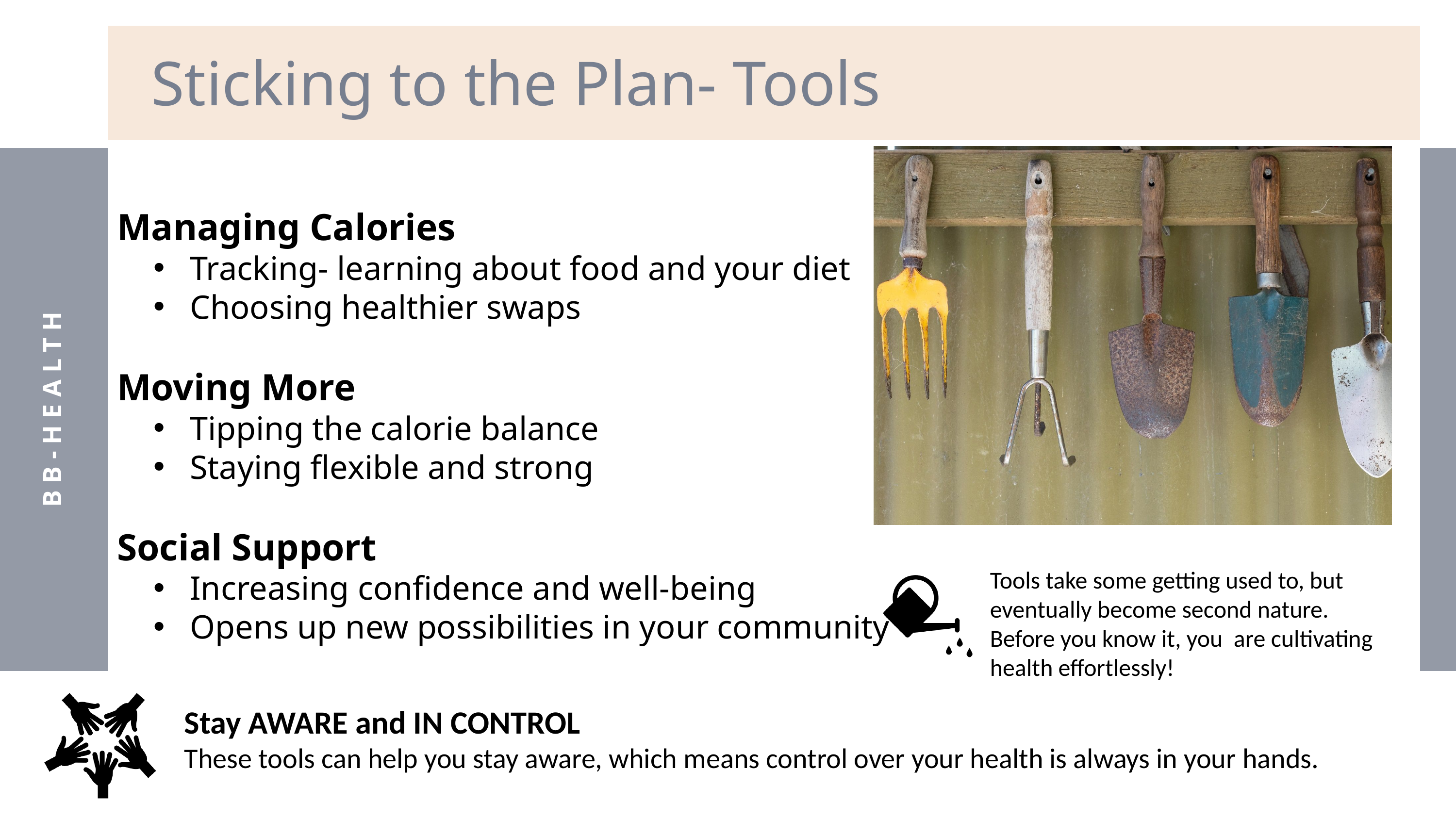

Sticking to the Plan- Tools
Managing Calories
Tracking- learning about food and your diet
Choosing healthier swaps
Moving More
Tipping the calorie balance
Staying flexible and strong
Social Support
Increasing confidence and well-being
Opens up new possibilities in your community
BB-HEALTH
Tools take some getting used to, but eventually become second nature. Before you know it, you are cultivating health effortlessly!
Stay AWARE and IN CONTROL
These tools can help you stay aware, which means control over your health is always in your hands.

## Slide 12
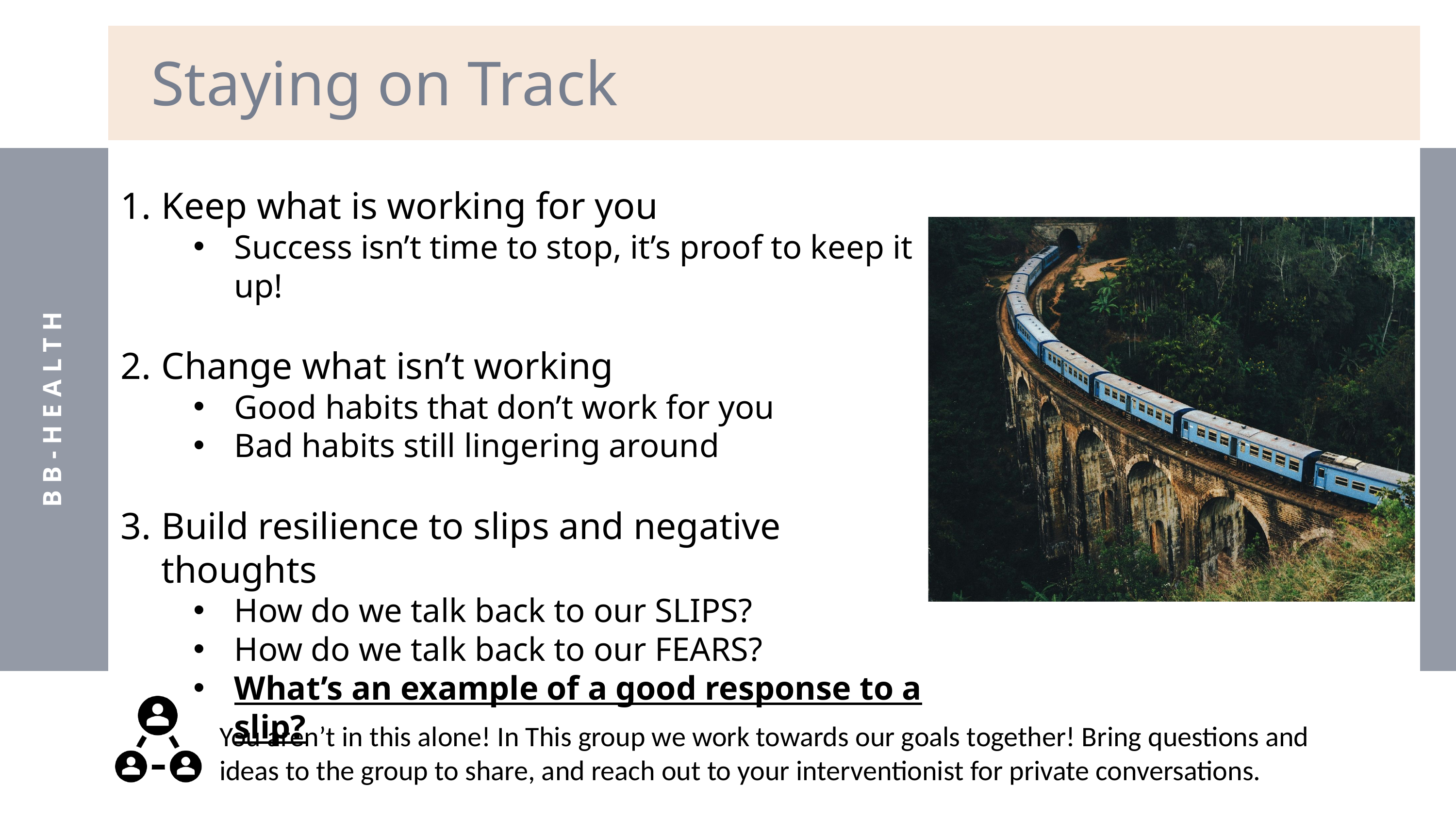

Staying on Track
Keep what is working for you
Success isn’t time to stop, it’s proof to keep it up!
Change what isn’t working
Good habits that don’t work for you
Bad habits still lingering around
Build resilience to slips and negative thoughts
How do we talk back to our SLIPS?
How do we talk back to our FEARS?
What’s an example of a good response to a slip?
BB-HEALTH
You aren’t in this alone! In This group we work towards our goals together! Bring questions and ideas to the group to share, and reach out to your interventionist for private conversations.

## Slide 13
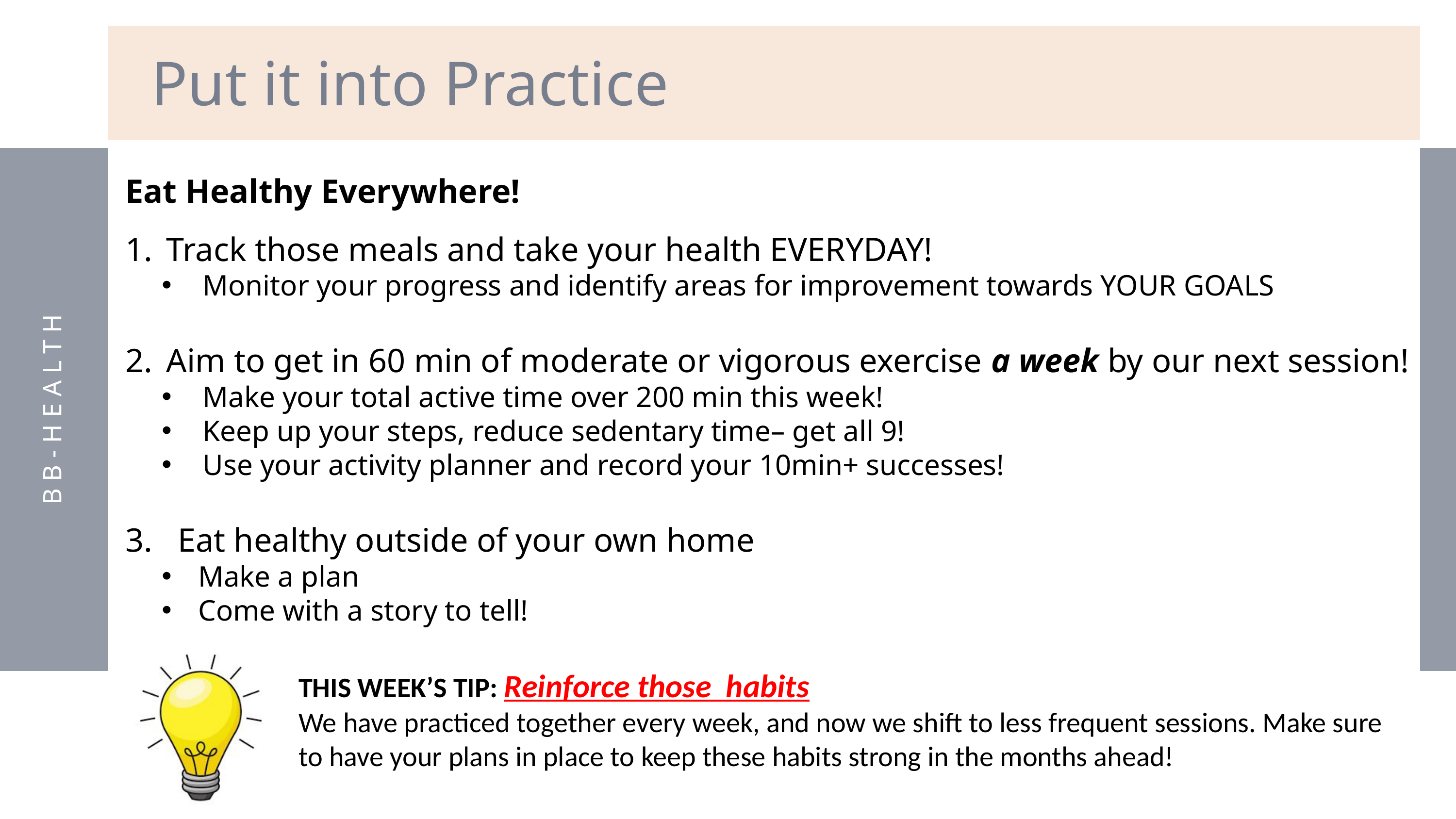

Put it into Practice
Eat Healthy Everywhere!
Track those meals and take your health EVERYDAY!
Monitor your progress and identify areas for improvement towards YOUR GOALS
Aim to get in 60 min of moderate or vigorous exercise a week by our next session!
Make your total active time over 200 min this week!
Keep up your steps, reduce sedentary time– get all 9!
Use your activity planner and record your 10min+ successes!
3. Eat healthy outside of your own home
Make a plan
Come with a story to tell!
BB-HEALTH
THIS WEEK’S TIP: Reinforce those habits
We have practiced together every week, and now we shift to less frequent sessions. Make sure to have your plans in place to keep these habits strong in the months ahead!

## Slide 14
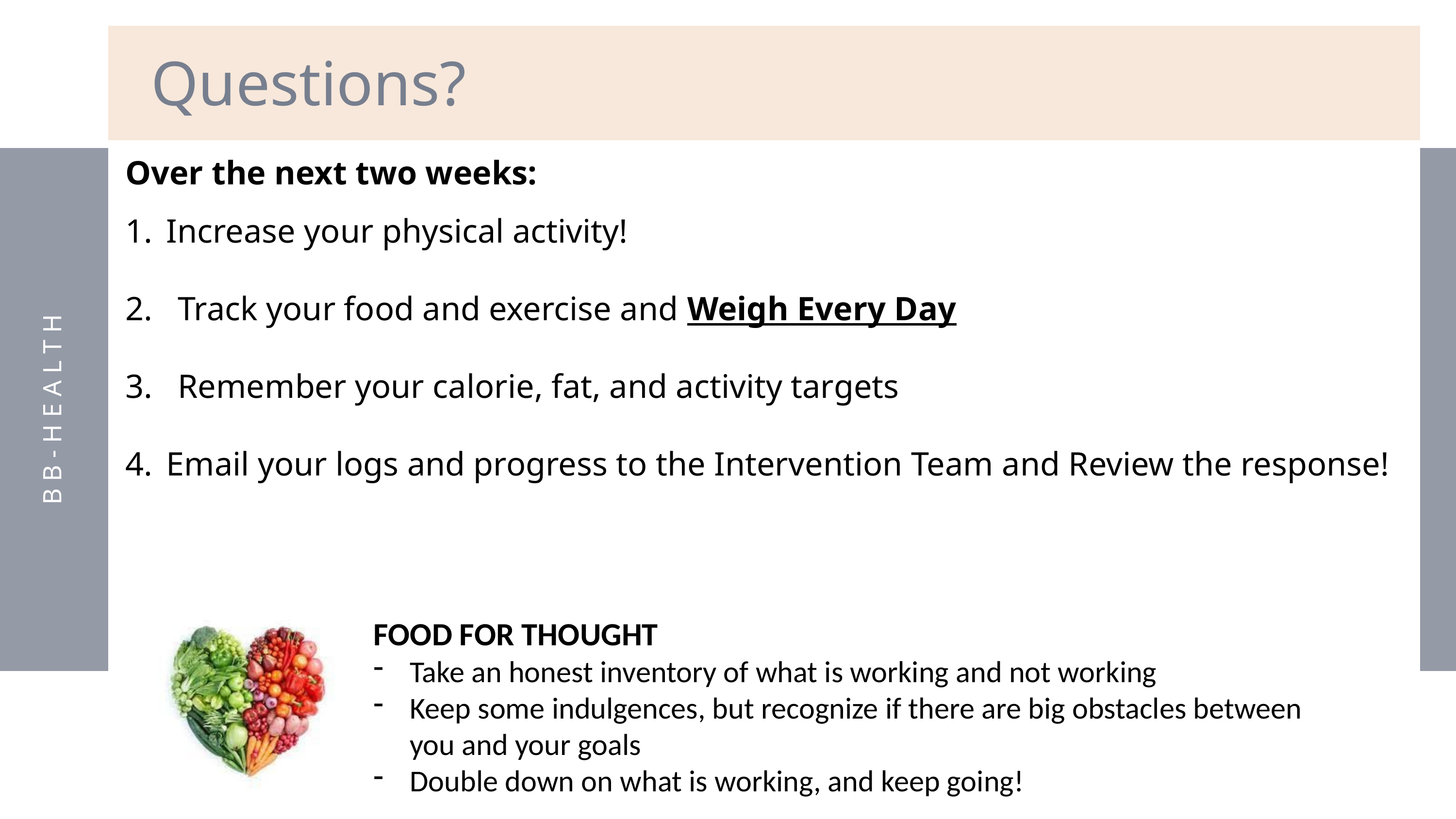

Questions?
Over the next two weeks:
Increase your physical activity!
2. Track your food and exercise and Weigh Every Day
3. Remember your calorie, fat, and activity targets
Email your logs and progress to the Intervention Team and Review the response!
BB-HEALTH
FOOD FOR THOUGHT
Take an honest inventory of what is working and not working
Keep some indulgences, but recognize if there are big obstacles between you and your goals
Double down on what is working, and keep going!
