# Supplementary material for: Development and Feasibility of an eHealth Diabetes Prevention Program Adapted for Older Adults—Results from a Randomized Control Pilot Study
Source: Nutrients. 2024 Mar 23;16(7):930. doi: 10.3390/nu16070930 (PMC11154527; doi:10.3390/nu16070930)
Supplement: Supplementary file 1 [file nutrients-16-00930-s001.zip › Session13_Handout_SUPP.pdf]

Session 13:
Mid-Program Review and Sneak Preview

We have covered a lot of tools to help you achieve your health goals. Let's review them and discuss what has helped and where we can go from here.

<u>Tools for Weight Loss and Healthy Lifestyle Success</u>

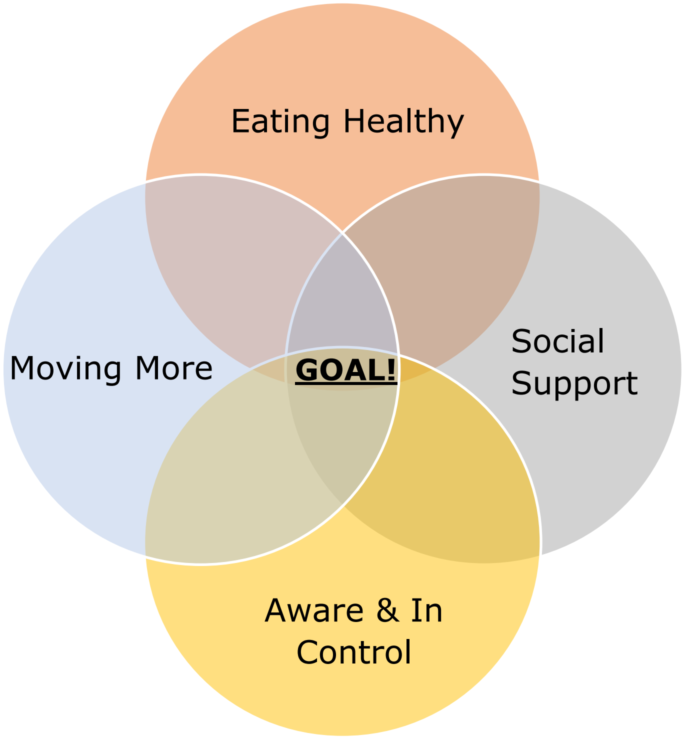

Up until now we have focused on setting goals, like weight loss, and the tools to achieve those goals like counting calories, being aware of fat in our food, managing social cues, asking for support, sitting less and moving more, and more.

After 3 months, you are probably aware that there is a difference between setting goals, learning how to use new tools, planning for success, and actually achieving or maintaining that success.

Now we will reflect upon the last 3 months, review tools, and what worked and didn't work while sharing with each other for support and to learn from each other.

Revisiting Our Goals

What was your Day 1 Goal?

____

____

What do you feel has helped you the most during these 3 months?

____

____

What obstacles are still in your way?

____

____

What is your Mid-Program Goal?

____

____

What is one thing you will do between this session and next to help move you towards your Mid-Program goal?

____

____

Habits for Success

Learning tools requires change, but establishing new habits helps you use those tools better. Once a chance becomes a habit it can make healthy living feel like less of a chore and makes healthy choices more automatic. This is how we can make healthy choices into a healthy lifestyle.

We build on early successes to help those healthy habits form, and we can review areas for improvement to help those early successes turn into long term wins. Together, new skills, new habits, and strong recovery from slip ups can help you achieve *and maintain* your goals.

Tool and Their Habits

- <u>Tracking Calories</u>
    - You can get the hang of it and:
        - Make food choices more predictable
        - Make calorie budgets that still include the foods you love
        - If you slip up, you know how to get back on track
    - When you find recipes and meal plans you like it can:
        - Shopping faster and easier
        - Help you adjust to lower calories with less hunger
        - Give you go-to options and take the guesswork out

- <u>Moving More</u>
    - Planning walks can help:
        - Break up your day
        - You connect and make plans with loved ones
        - Build up your energy
        - Give you something to look forward to
    - Adding more spontaneous activity can:
        - Shake things up
        - Make activity feel like an adventure
        - Space out time in between meals

- Social Support
  - Sticking to your plan can:
    - Inspire those around you to improve their help
    - Get people to support you to improve your life
    - Make it easier to find people, places and things in your environment that fit into your new healthy lifestyle
  - Progressing toward goals can:
    - Show the people in your that living healthy is possible
    - Help you advocate for your goals and needs
    - Make you feel confident

- Being Aware & In Control
  - Staying aware of progress can:
    - Let you see small and big successes
    - Help identify obstacles or places for improvement
    - Keep you in control of your health
  - Staying in control can:
    - Keep you moving towards your goals
    - Show you that slip ups aren't a big deal
    - Empower you to keep your health plan working
    - Help you catch problems early, and recover easily

Staying on track

Nobody is perfect and no one has all the answers. In our group environment we have talked about a lot of ideas, and some work for you and some don't.

What is one idea you learned from a group member that helped you?

____

____

What is something you tried that didn't work for you?

____

____

What was something that was hard when you first tried it and now is like second nature to you?

____

____

What is something you didn't try before but would be ready to try now?

____

____

Describe one way that you recovered from a healthy lifestyle slip?

____

How has committing to your health improved your life in other ways?

____

____

Have friends or family commented on your success? If so, how?

____

____

If not, how have you made them aware of your goals and successes?

____

____

GLB- a group working towards our goals together.

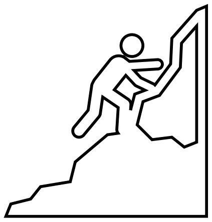

Building on Success: Preview of What's to Come

Think about all that you have learned and accomplished in these 3 months. We have 8+ months left in our program, how far can we go?

Group Lifestyle Balance Session 14-22	
**#14**<br>Two weeks away!	Take Charge of Your Lifestyle<br>• Your goals, your progress<br>• Empowering your success
**#15**<br>One month away!	Mindful Eating, Mindful Movement<br>• Aware and In Control<br>• Being in the moment<br>• Enjoying Healthy Living
**#16**<br>Two months away!	Manage Your Stress<br>• Overcoming Obstacles<br>• Self-Care
**#17**<br>Three months Away!	Sit Less for Your Health<br>• Awareness of bad habits<br>• New movement techniques
**#18**<br>Four months away!	More Volume, Fewer Calories<br>• New tips to curb hunger<br>• Eat more food but eat less calories
**#19**<br>Five months away!	Stay Active<br>• Motivation<br>• Setting new goals
**#20**<br>Six months away!	Balance Your Thoughts<br>• Focus on the positive<br>• Self-Care
**#21**<br>Seven months away!	Heart Health<br>• How healthy choices to help your heart
**#22**<br>Eight months away!	Look Back and Look Forward<br>• Keep going on your own<br>• You got this!

To Do:

The Basics:

☐ Keep track of your weight.

☐ Record what you eat and drink using your preferred method. Come as close as you can to your calorie and fat gram goals.

☐ Record your physical activity using your preferred method. Come as close as you can to your activity goals.

- Be physically active for at least 150 minutes per week.
- Aim for an average of at least 50,000 steps per week.

☐ Keep moving throughout the day.

What's New: Review Successes and Strengthen New Goals

☐ Check in with your progress and keep moving forward.

☐ Congratulate yourself for making it this far and gear up for the next half of the program!
