# Supplementary material for: Development and Feasibility of an eHealth Diabetes Prevention Program Adapted for Older Adults—Results from a Randomized Control Pilot Study
Source: Nutrients. 2024 Mar 23;16(7):930. doi: 10.3390/nu16070930 (PMC11154527; doi:10.3390/nu16070930)
Supplement: Supplementary file 1 [file nutrients-16-00930-s001.zip › Session13_SUPP.pdf]

BB-HEALTH

e-Healthy Older Adults Modification

Diabetes Prevention Program

Session #13

Look how far we have come!

Group Opening Discussion

BB-HEALTH

WELCOME BACK! <u>HOW WAS THE LAST 2 WEEKS</u>?

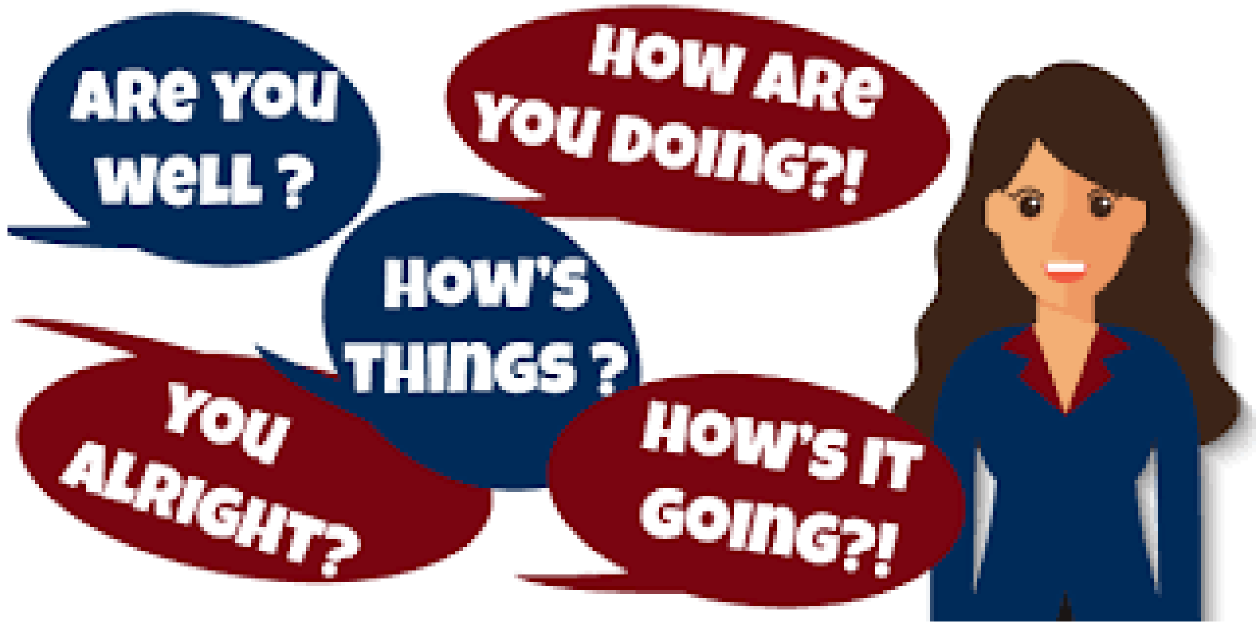

1. Did you track every meal?

2. Did you weigh every day, & record your health today?

3. Did you successfully manage stress this week?

4. Did you increase your daily steps?

5. Did you email the Intervention Team and review the response?

6. What were your unique challenges and successes?

REMINDER: CHARGE YOUR FITBIT & SCALE DURING THIS MEETING!

ROUNDTABLE

BB-HEALTH

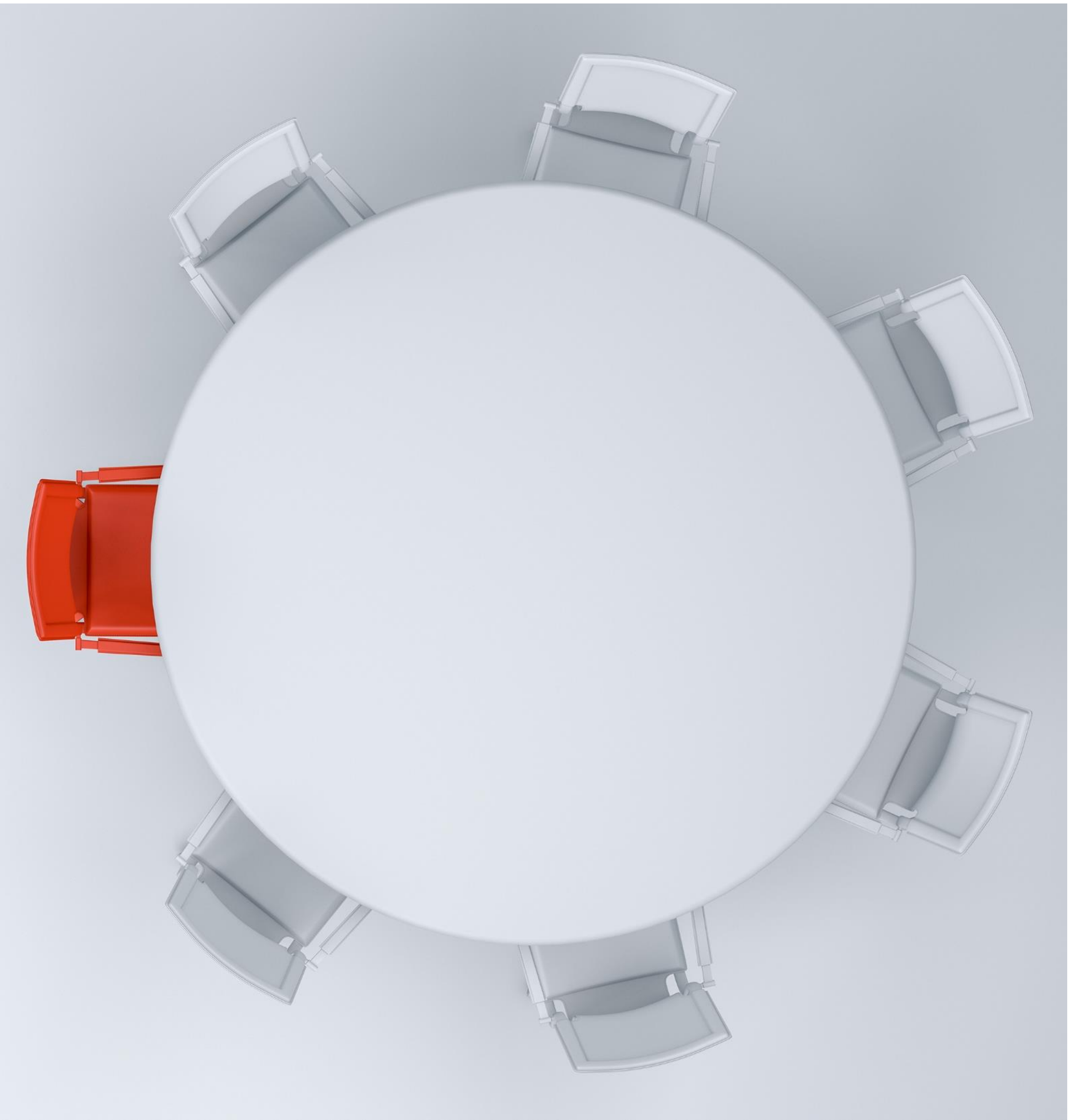

What does ***HEALTHY*** mean to you?

What does ***HEALTHY FOOD*** mean?

What helped you the most over the last 3 months?

What do you struggle with most these last 3 months?

What have your learned to support your success?

Checking in With Our Goals

<u>Do you feel your health is improving? (select one)</u>

- My health is much worse
- My health is worse
- My health hasn’t improved
- My health is fluctuating
- My health has gotten better
- My health has gotten much better

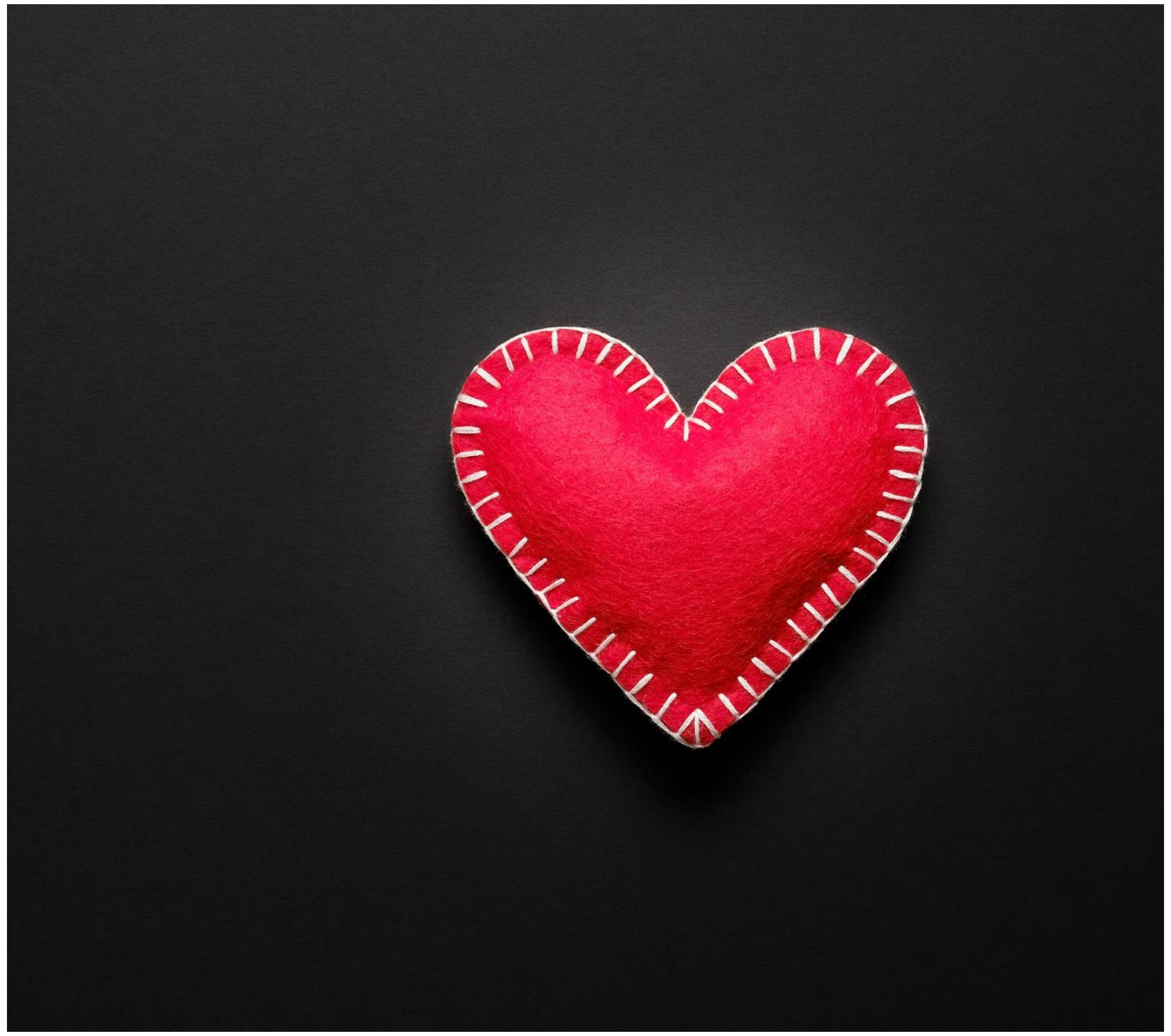

Checking in With Our Goals

<u>Do you feel your weight is improving? (select one)</u>

- My weight is much worse
- My weight is worse
- My weight hasn't improved
- My weight is fluctuating
- My weight has gotten better
- My weight has gotten much better

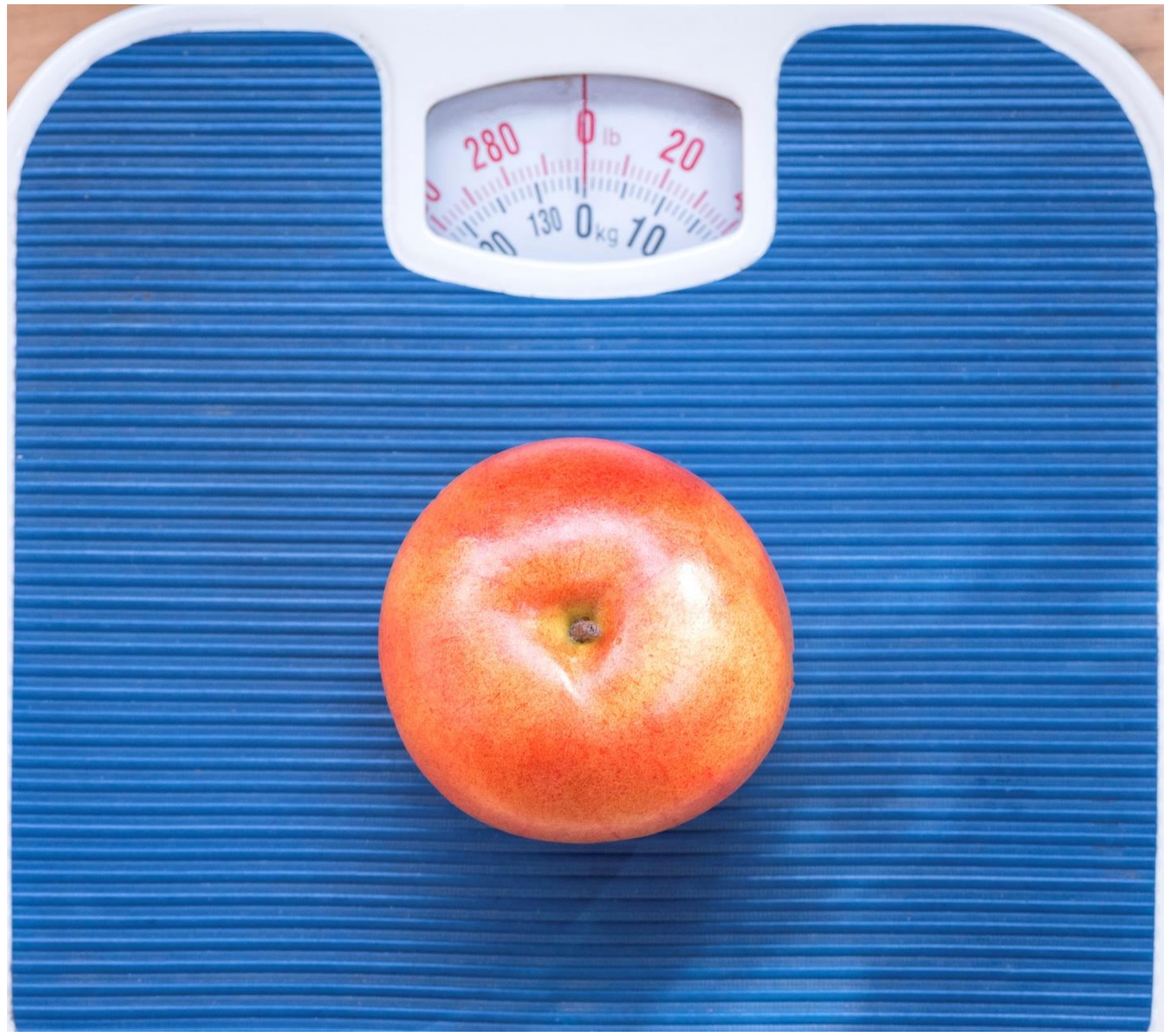

Checking in With Our Goals

Do you feel your physical activity level is improving? (select one)

- ○ My activity level is much worse
- ○ My activity level is worse
- ○ My activity level hasn't improved
- ○ My activity level is fluctuating
- ○ My activity level has gotten better
- ○ My activity level has gotten much better

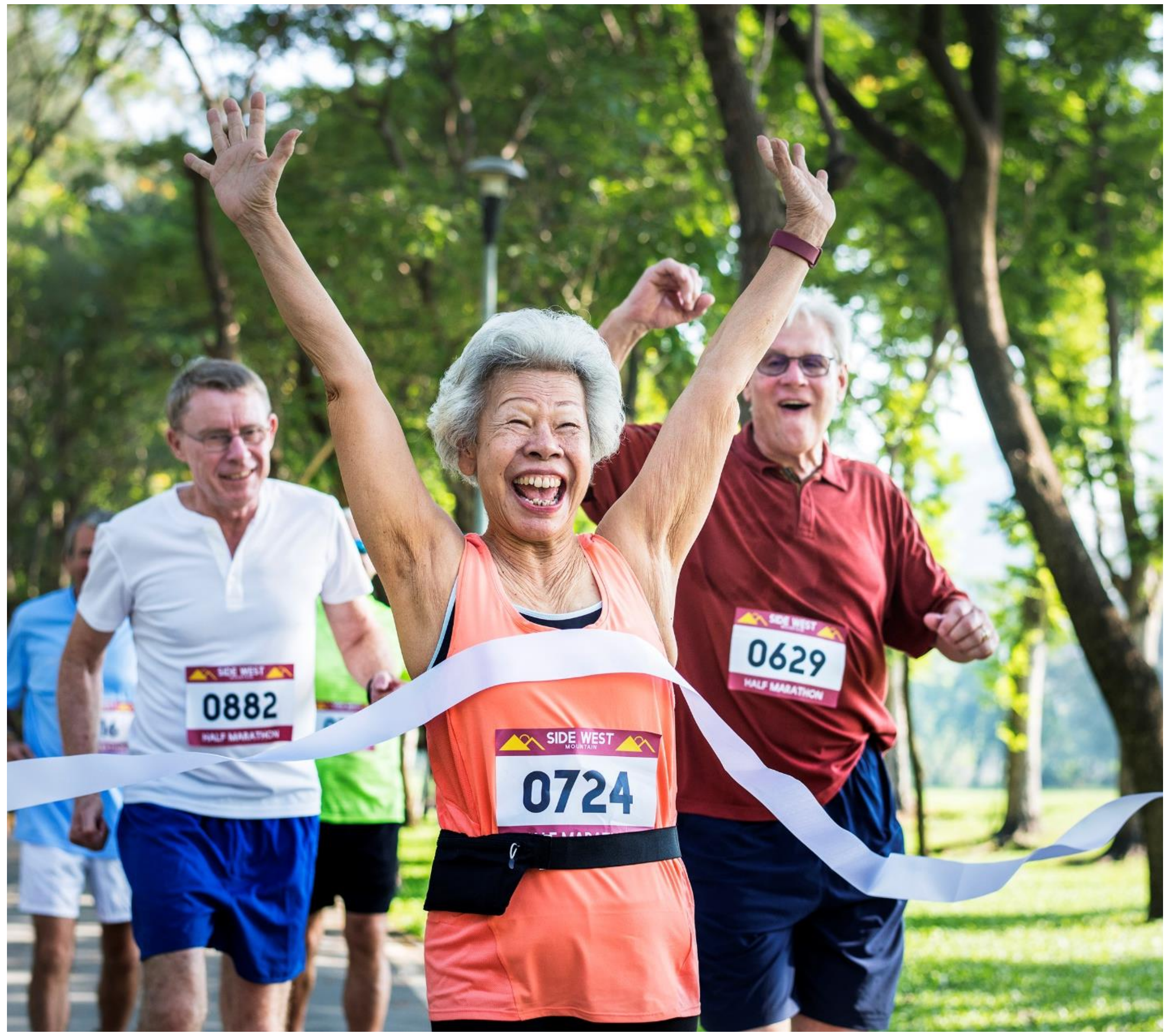

Checking in With Our Goals

<u>How strictly do you stick to your action plan? (Select one)</u>

- I always stick to my plan
- I mostly stick to my plan
- I stick to my plan about half the time
- I sometimes stick to my plan
- I don’t stick my plan often
- I rarely stick to my plan
- I never, or almost never, stick to my plan

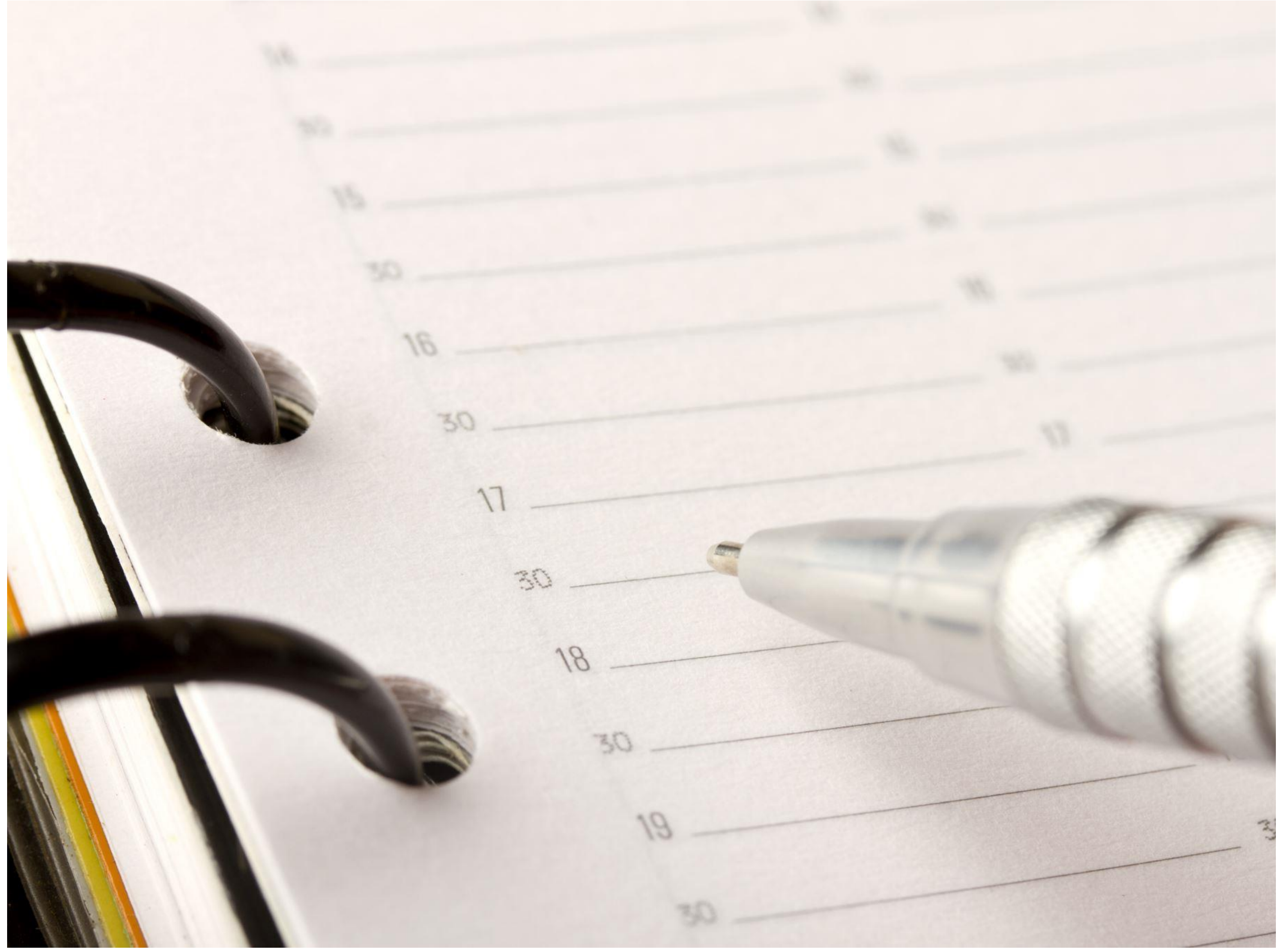

Checking in With Our Goals

<u>How important is it to you to keep trying to improve your health? (select one)</u>

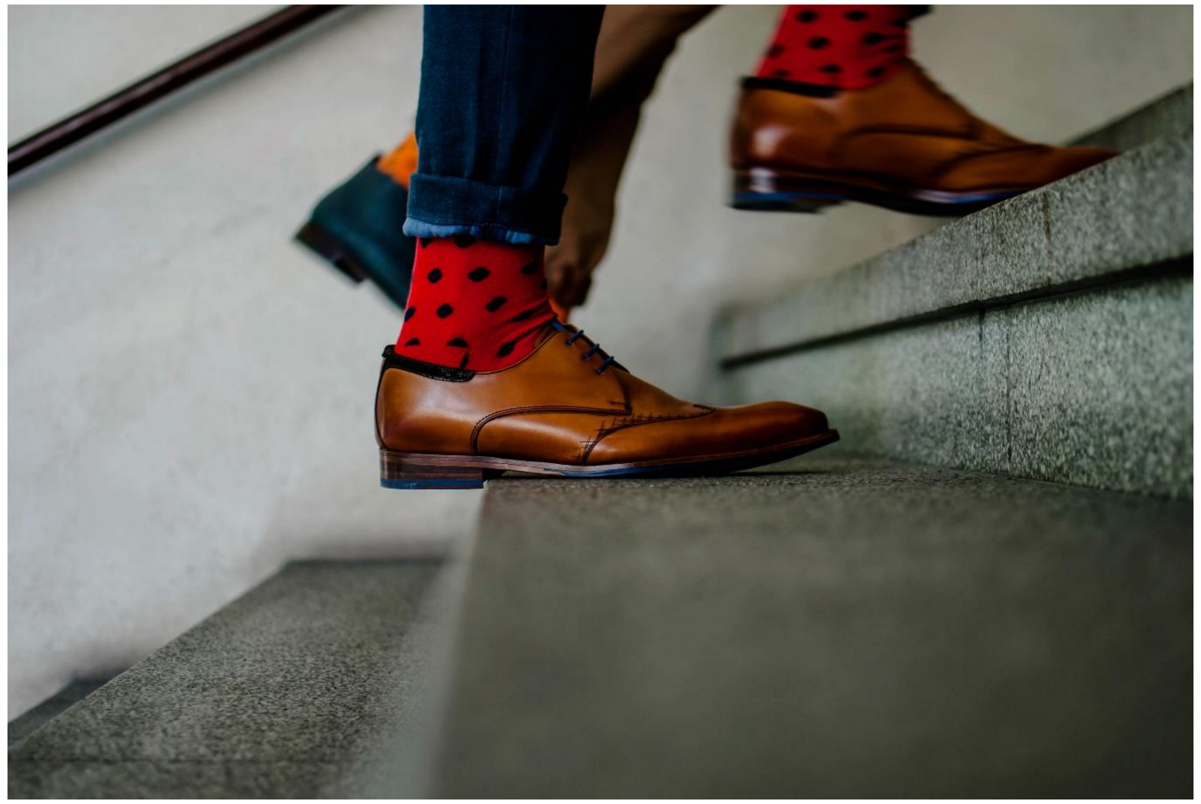

- Improving my health is my top priority
- Improving my health is very important
- Improving my health is important
- I want to improve my health, but it isn't a high priority
- I'm indifferent about improving my health
- I'm not interested in improving my health
- I am very uninterested in trying to improve my health

Checking in With Our Goals

B B - H E A L T H

Do you PRIORITIZE healthy choices to support your lifestyle (Select one)

- Always, my health comes first
- Most of the time
- A fair amount of the time
- Some of the time
- Less than half of the time
- Occasionally
- Rarely
- Never

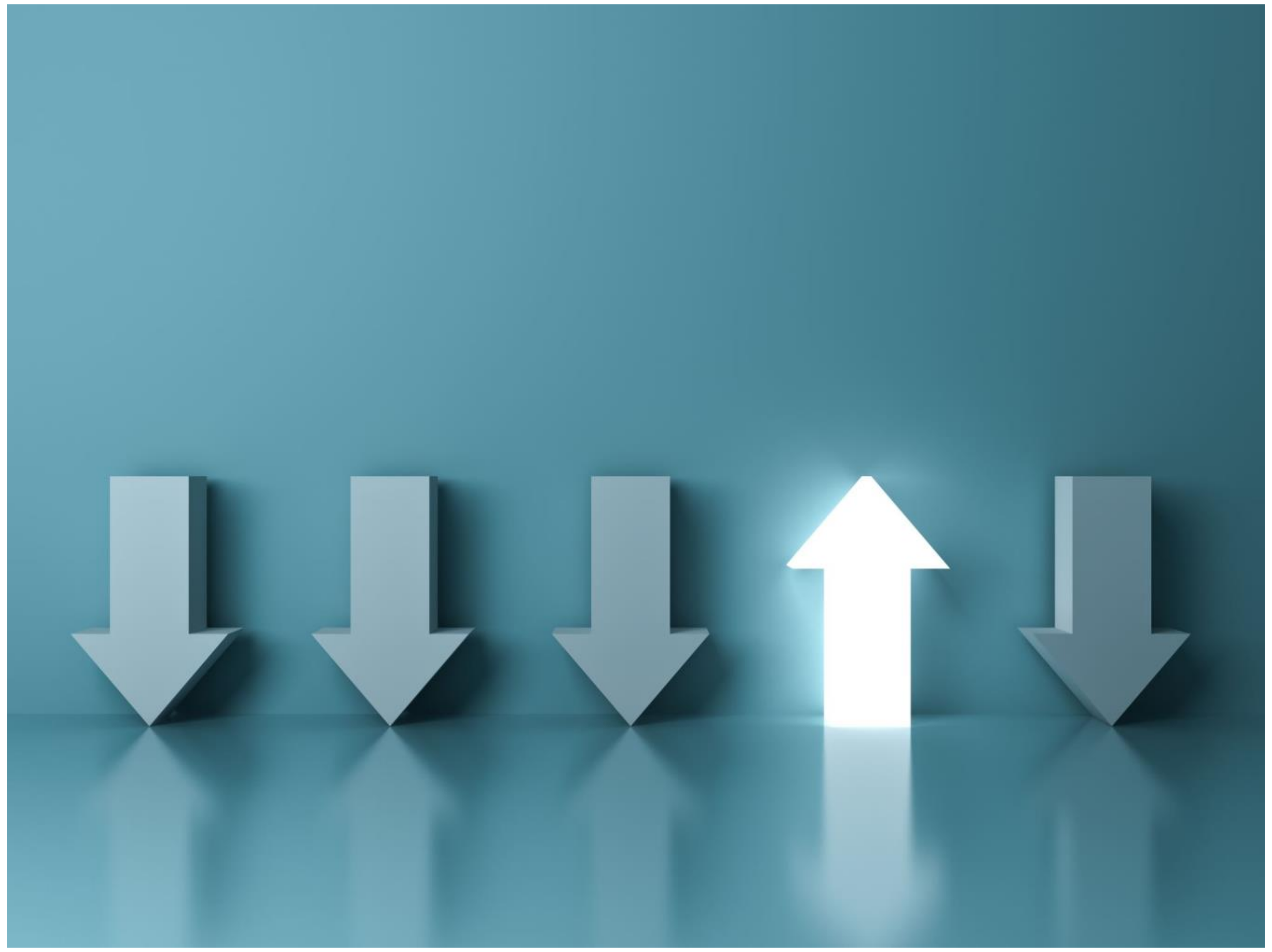

Checking in With Our Goals

BB-HEALTH

When you make a change, how well does it stick? (Select one)

- I am strict at first (a week) and then it goes away
- I am strict at first (a month) and then it goes away
- It takes me a while to get the hang of something new, but then it is here to stay
- I make and/or break habits easily
- I'm bad at routines, things change for me all the time
- Other (discuss)

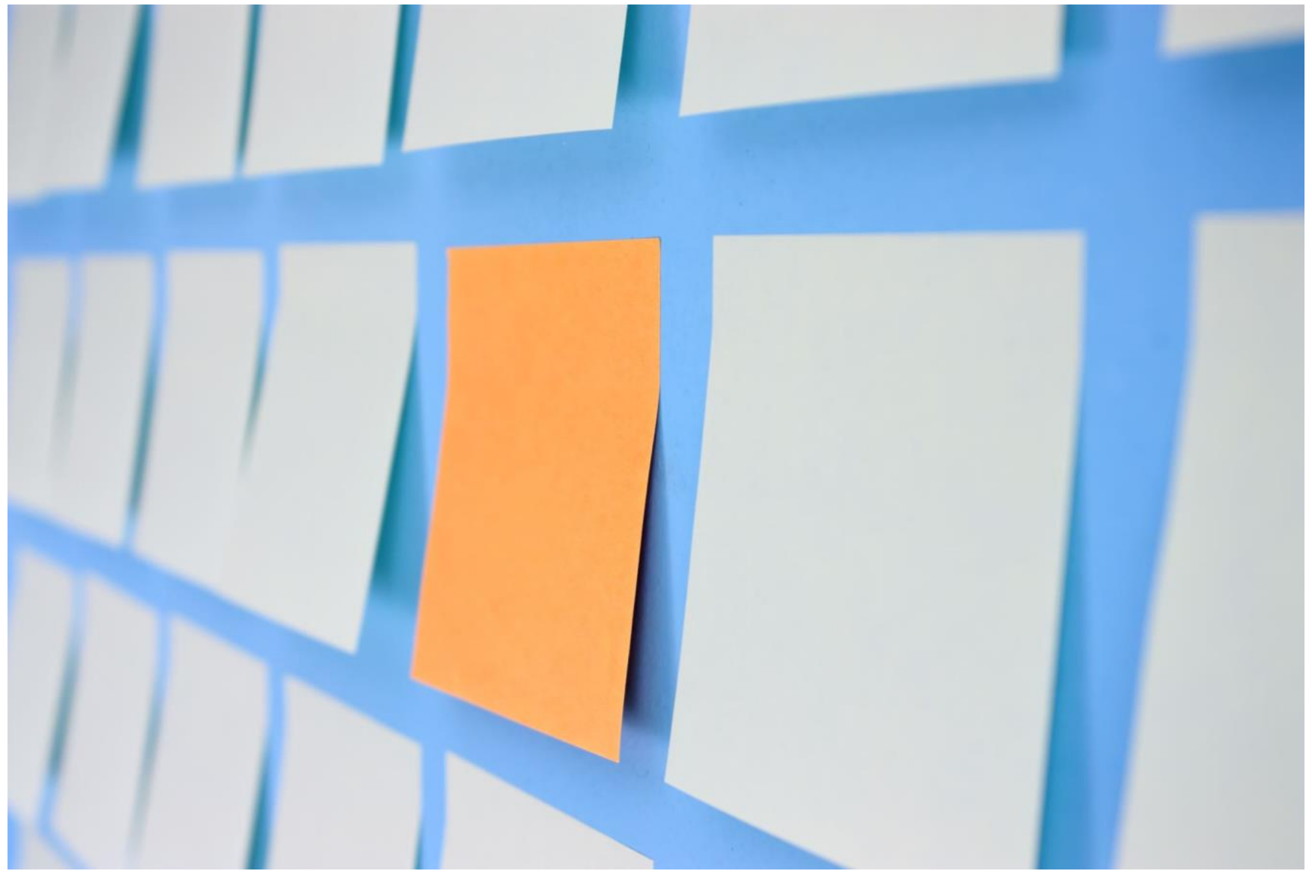

Sticking to the Plan- Tools

BB-HEALTH

Managing Calories

- Tracking- learning about food and your diet
- Choosing healthier swaps

Moving More

- Tipping the calorie balance
- Staying flexible and strong

Social Support

- Increasing confidence and well-being
- Opens up new possibilities in your community

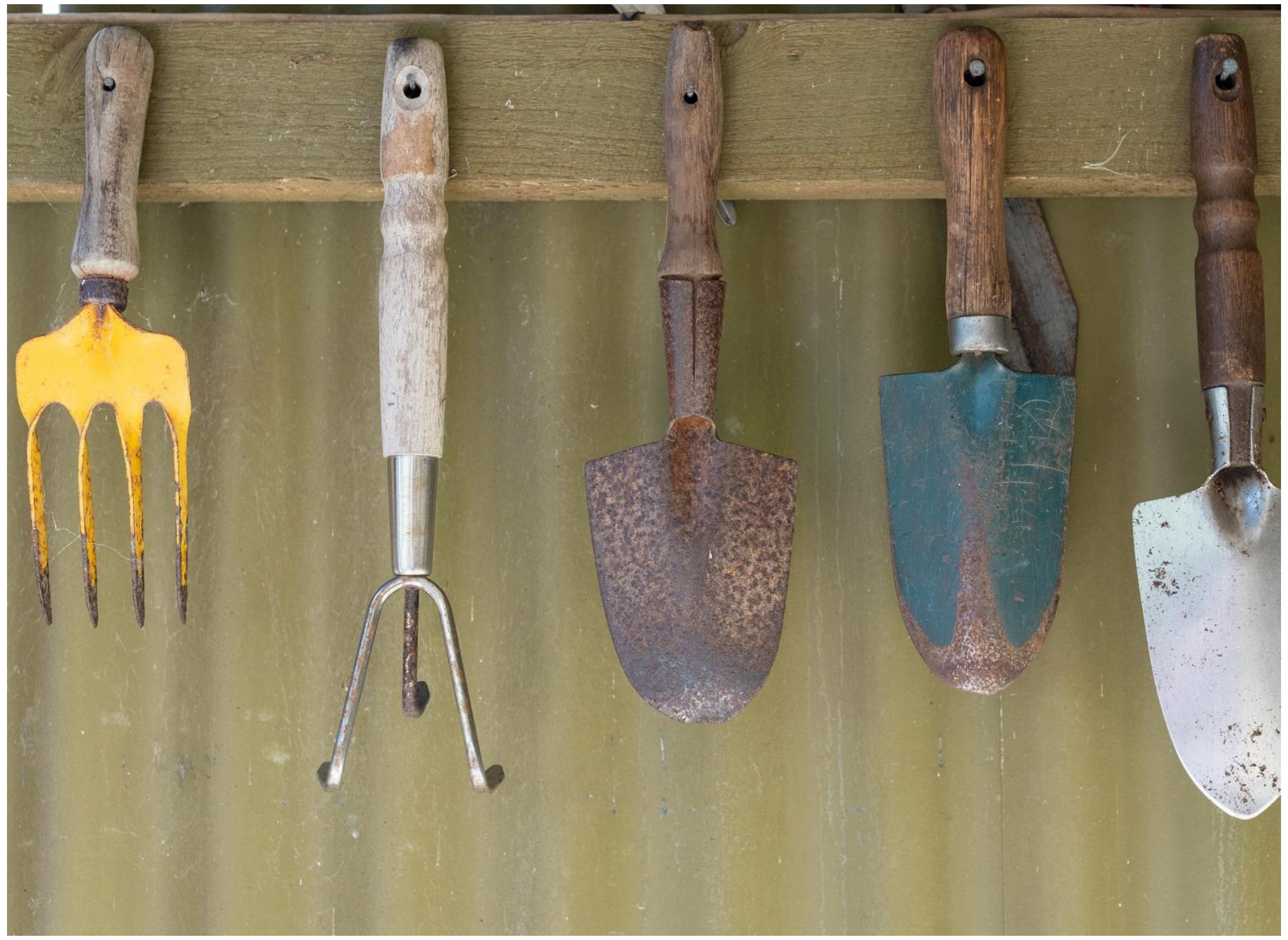

Tools take some getting used to, but eventually become second nature. Before you know it, you are cultivating health effortlessly!

Stay AWARE and IN CONTROL

These tools can help you stay aware, which means control over your health is always in your hands.

Staying on Track

BB-HEALTH

1. Keep what is working for you
   - Success isn't time to stop, it's proof to keep it up!

2. Change what isn't working
   - Good habits that don't work for you
   - Bad habits still lingering around

3. Build resilience to slips and negative thoughts
   - How do we talk back to our SLIPS?
   - How do we talk back to our FEARS?
   - **What's an example of a good response to a slip?**

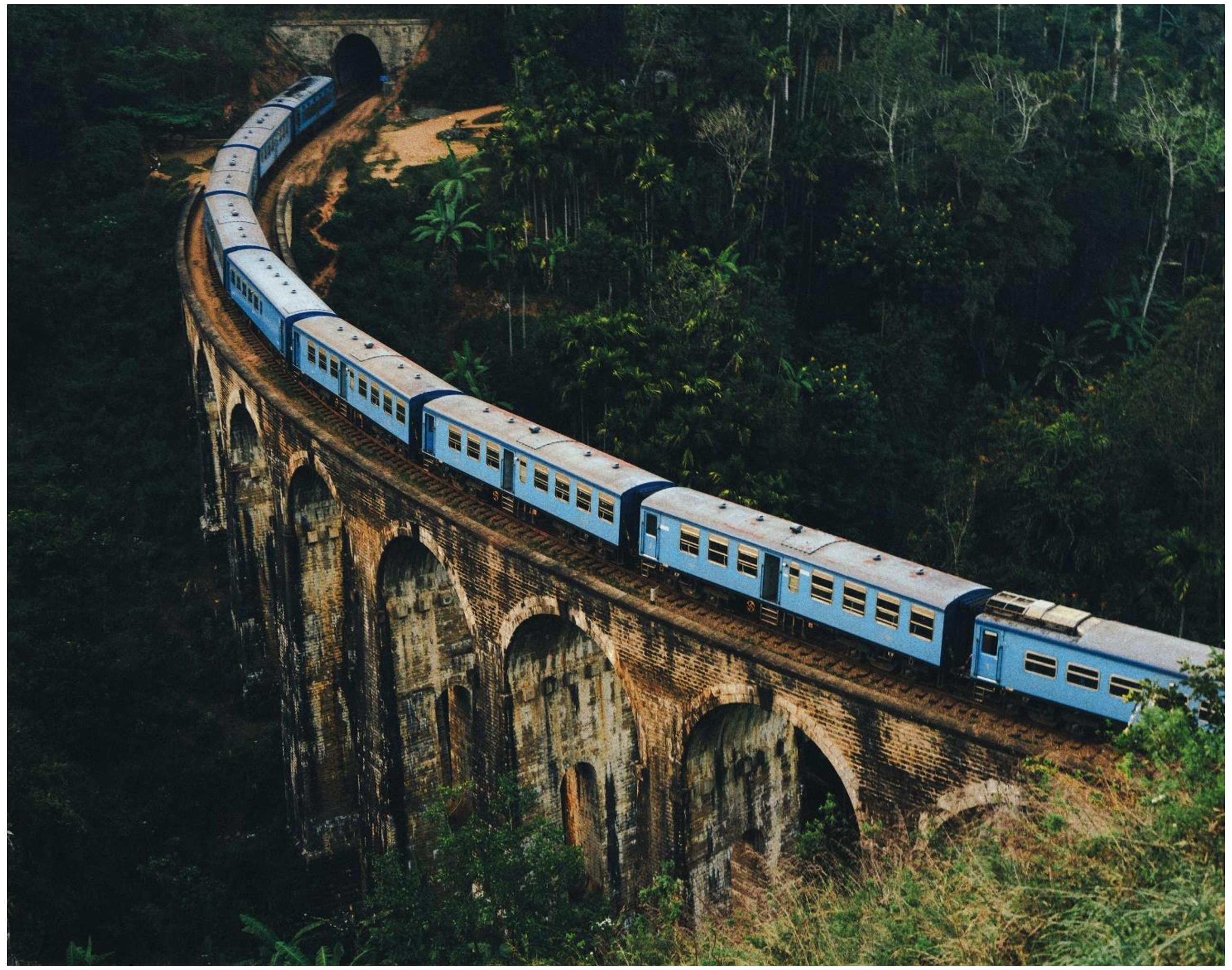

You aren't in this alone! In This group we work towards our goals together! Bring questions and ideas to the group to share, and reach out to your interventionist for private conversations.

Put it into Practice

BB-HEALTH

Eat Healthy Everywhere!

1. Track those meals and take your health EVERYDAY!
    - Monitor your progress and identify areas for improvement towards YOUR GOALS

2. Aim to get in 60 min of moderate or vigorous exercise ***a week*** by our next session!
    - Make your total active time over 200 min this week!
    - Keep up your steps, reduce sedentary time– get all 9!
    - Use your activity planner and record your 10min+ successes!

3. Eat healthy outside of your own home
    - Make a plan
    - Come with a story to tell!

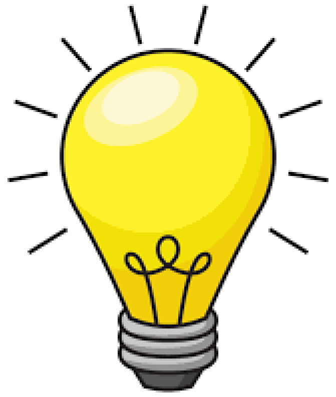

THIS WEEK'S TIP: ***Reinforce those habits***
We have practiced together every week, and now we shift to less frequent sessions. Make sure to have your plans in place to keep these habits strong in the months ahead!

Questions?

B B - H E A L T H

Over the next two weeks:

1. Increase your physical activity!

2. Track your food and exercise and **Weigh Every Day**

3. Remember your calorie, fat, and activity targets

4. Email your logs and progress to the Intervention Team and Review the response!

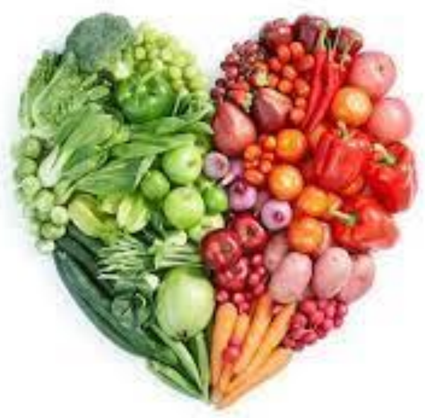

FOOD FOR THOUGHT

- Take an honest inventory of what is working and not working
- Keep some indulgences, but recognize if there are big obstacles between you and your goals
- Double down on what is working, and keep going!
