# Supplementary material for: Development and Feasibility of an eHealth Diabetes Prevention Program Adapted for Older Adults—Results from a Randomized Control Pilot Study
Source: Nutrients. 2024 Mar 23;16(7):930. doi: 10.3390/nu16070930 (PMC11154527; doi:10.3390/nu16070930)
Supplement: Supplementary file 1 [file nutrients-16-00930-s001.zip › Session14.pptx]

## Slide 1
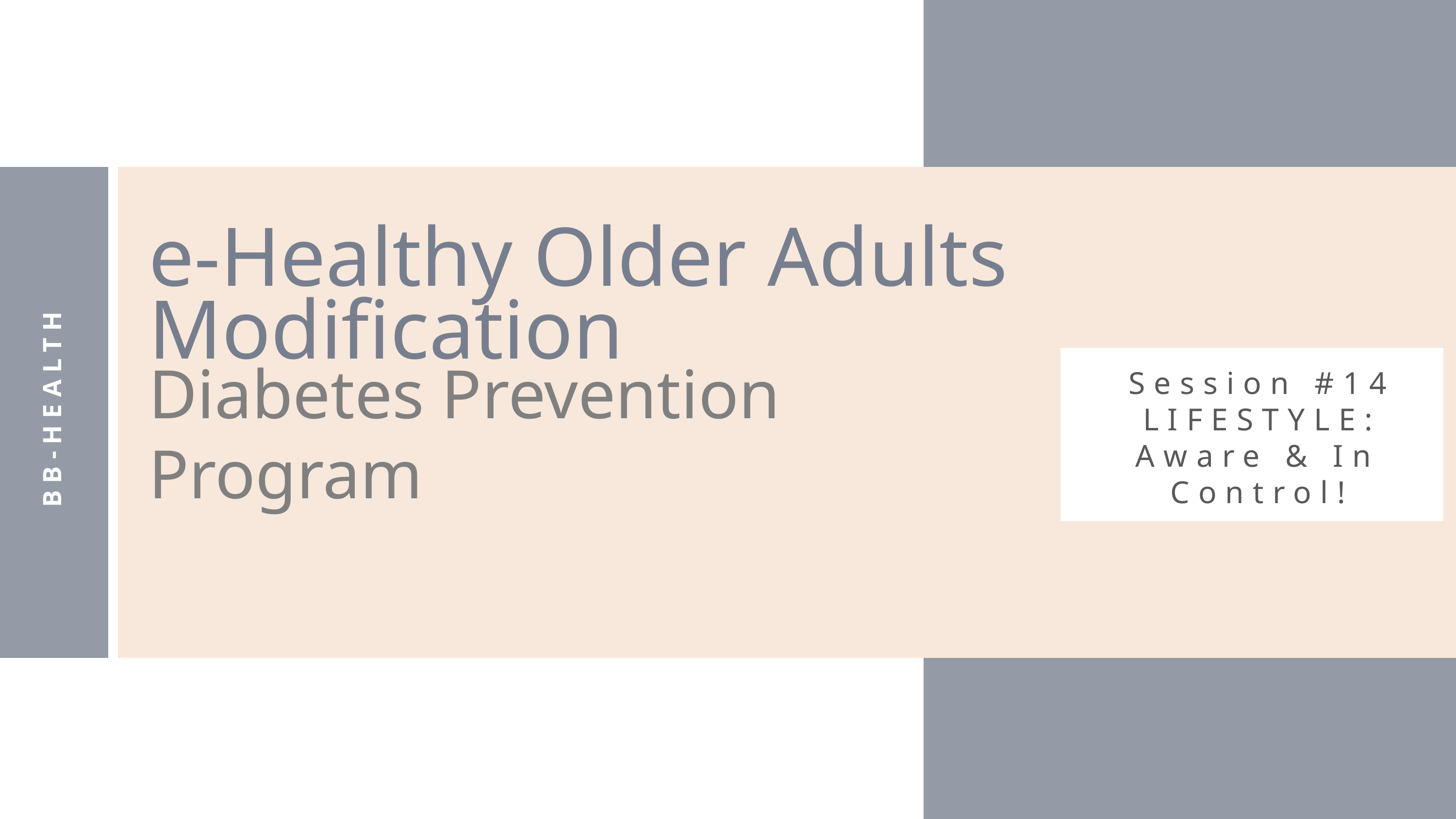

OPEN REPORTS
e-Healthy Older Adults Modification
Session #14
LIFESTYLE:
Aware & In Control!
Diabetes Prevention Program
BB-HEALTH

## Slide 2
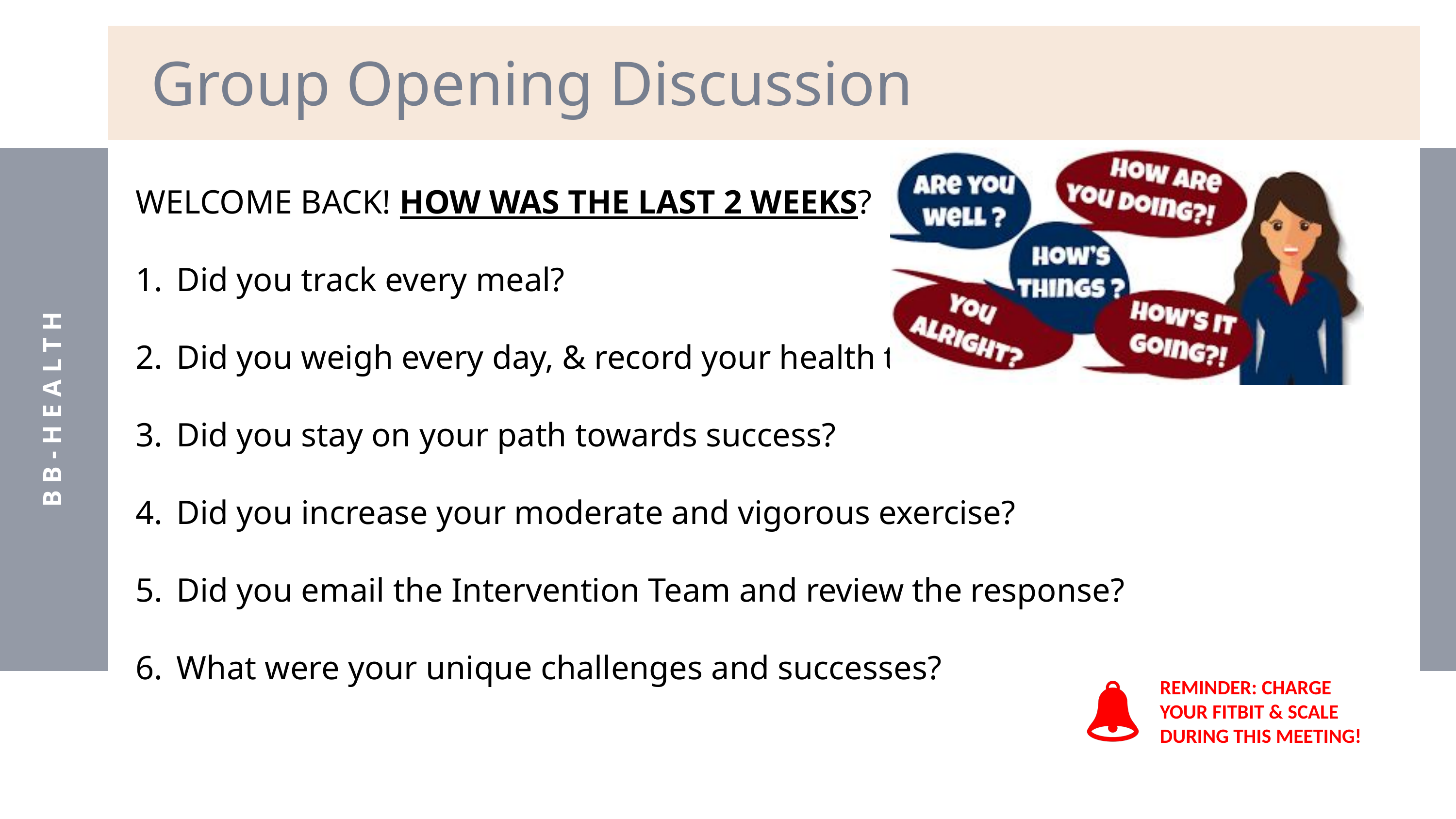

Group Opening Discussion
WELCOME BACK! HOW WAS THE LAST 2 WEEKS?
Did you track every meal?
Did you weigh every day, & record your health today?
Did you stay on your path towards success?
Did you increase your moderate and vigorous exercise?
Did you email the Intervention Team and review the response?
What were your unique challenges and successes?
BB-HEALTH
REMINDER: CHARGE YOUR FITBIT & SCALE DURING THIS MEETING!

## Slide 3
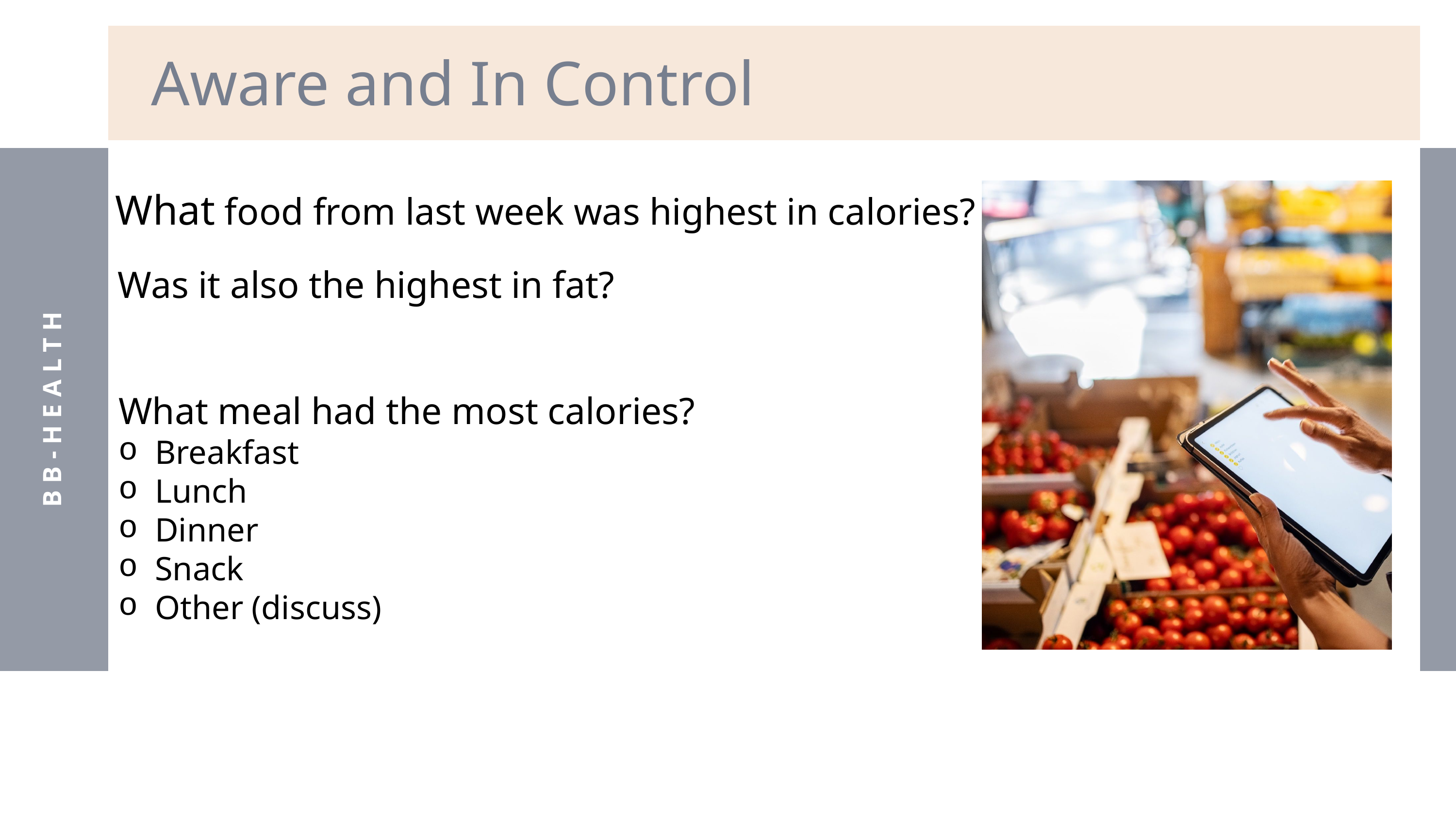

Aware and In Control
What food from last week was highest in calories?
Was it also the highest in fat?
What meal had the most calories?
Breakfast
Lunch
Dinner
Snack
Other (discuss)
BB-HEALTH

## Slide 4
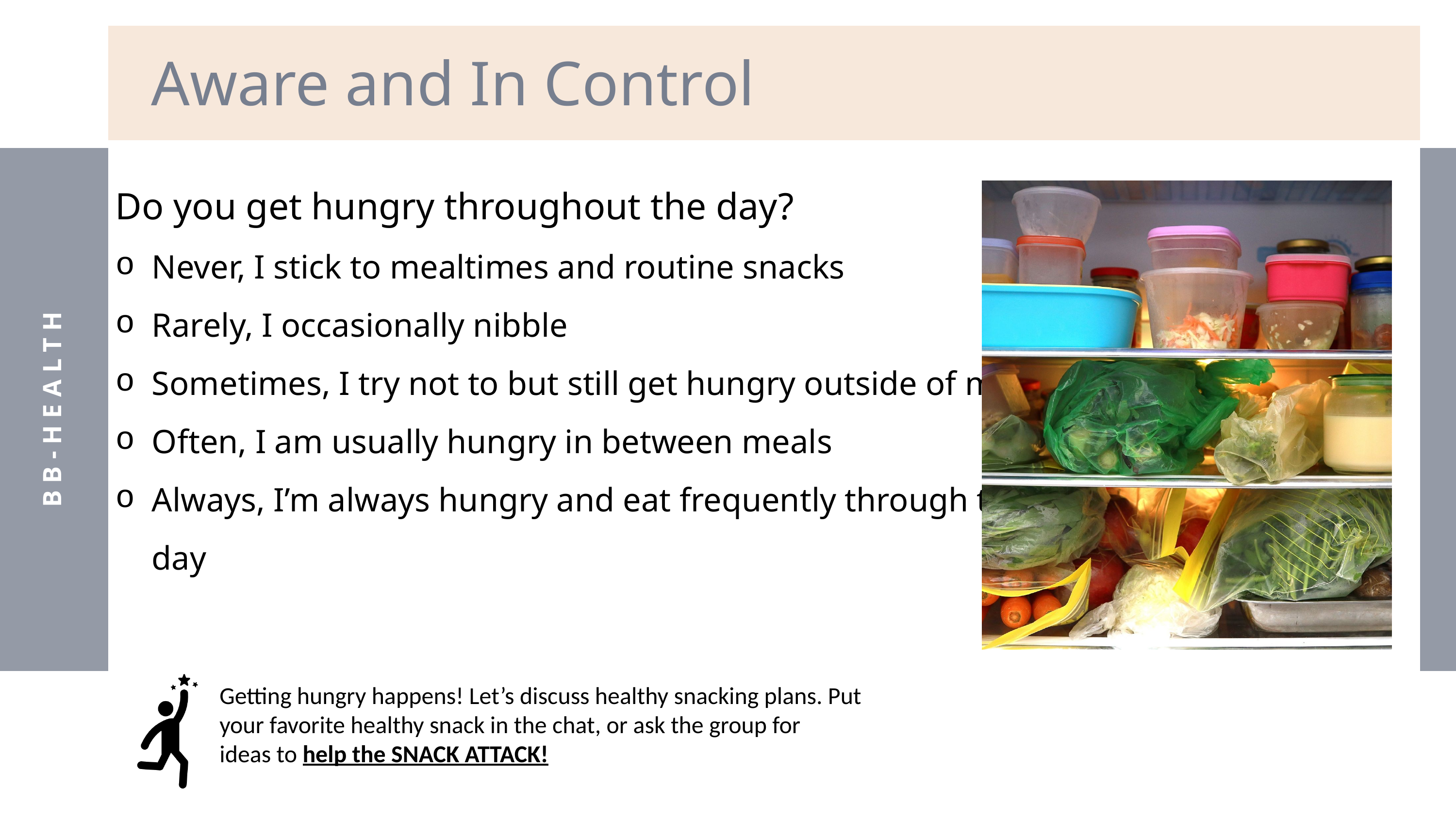

Aware and In Control
Do you get hungry throughout the day?
Never, I stick to mealtimes and routine snacks
Rarely, I occasionally nibble
Sometimes, I try not to but still get hungry outside of meals
Often, I am usually hungry in between meals
Always, I’m always hungry and eat frequently through the day
BB-HEALTH
Getting hungry happens! Let’s discuss healthy snacking plans. Put your favorite healthy snack in the chat, or ask the group for
ideas to help the SNACK ATTACK!

## Slide 5
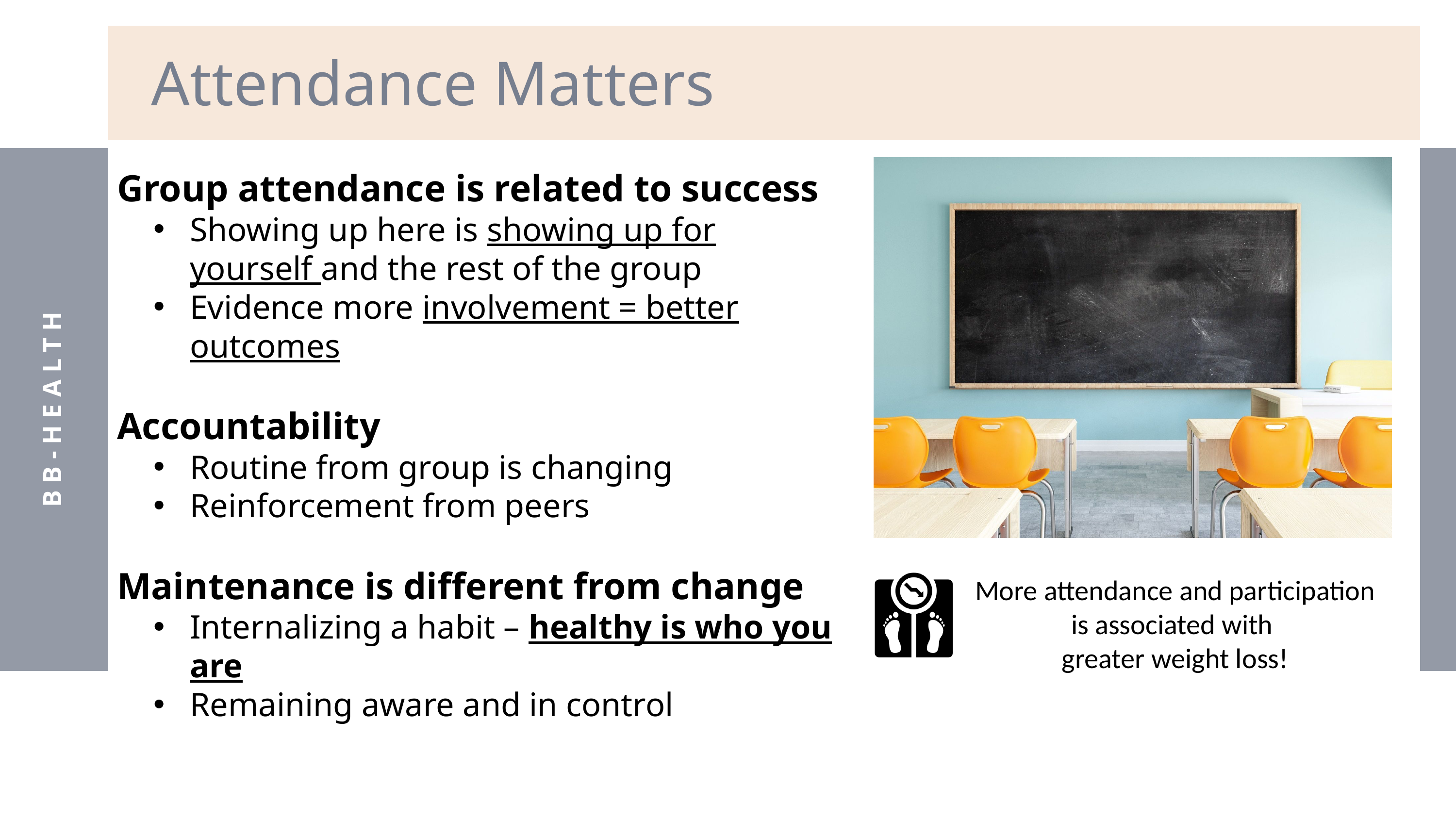

Attendance Matters
Group attendance is related to success
Showing up here is showing up for yourself and the rest of the group
Evidence more involvement = better outcomes
Accountability
Routine from group is changing
Reinforcement from peers
Maintenance is different from change
Internalizing a habit – healthy is who you are
Remaining aware and in control
BB-HEALTH
More attendance and participation is associated with
greater weight loss!

## Slide 6
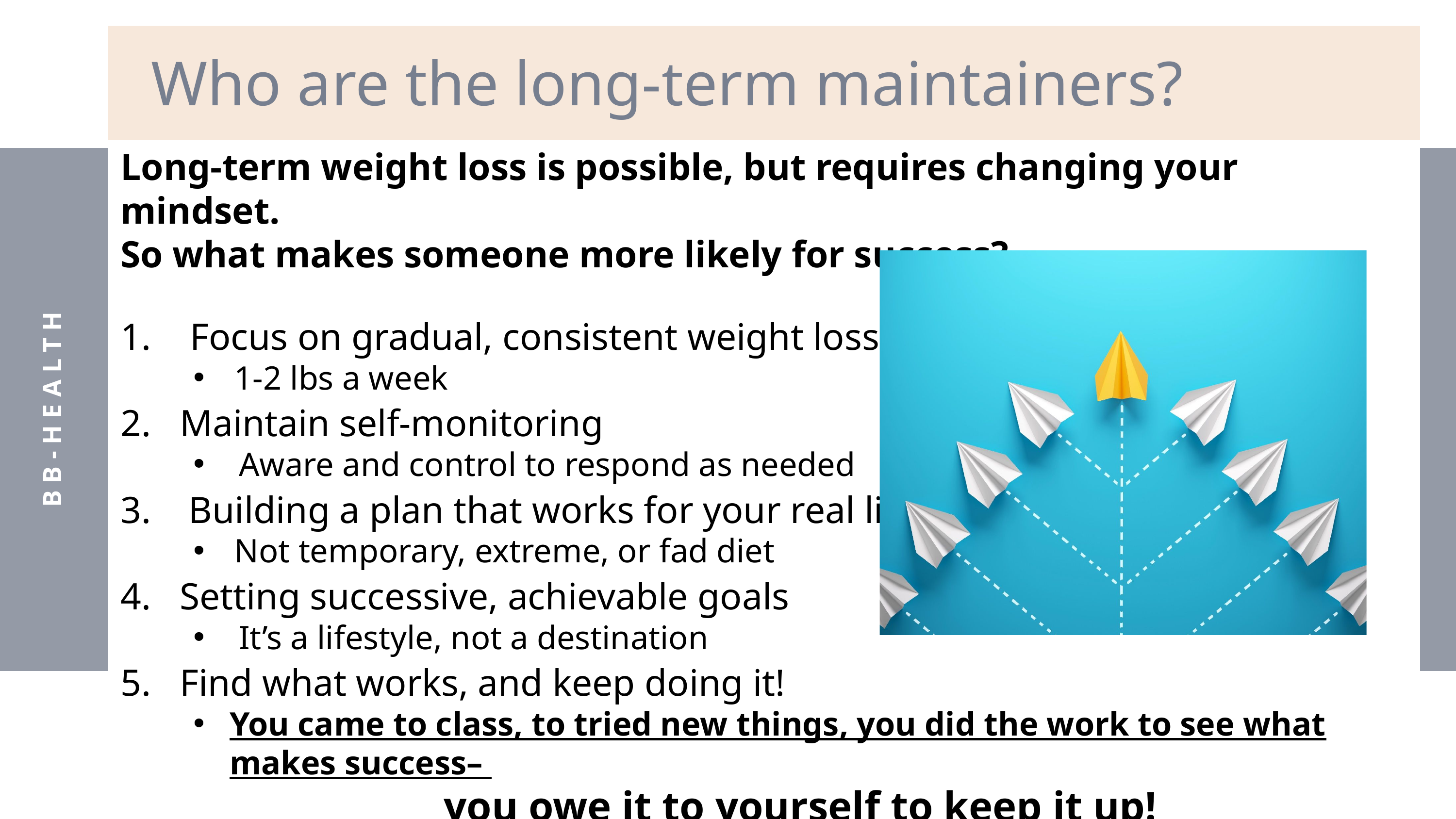

Who are the long-term maintainers?
Long-term weight loss is possible, but requires changing your mindset.
So what makes someone more likely for success?
 Focus on gradual, consistent weight loss
1-2 lbs a week
Maintain self-monitoring
Aware and control to respond as needed
3. Building a plan that works for your real life
Not temporary, extreme, or fad diet
Setting successive, achievable goals
It’s a lifestyle, not a destination
Find what works, and keep doing it!
You came to class, to tried new things, you did the work to see what makes success–
you owe it to yourself to keep it up!
BB-HEALTH

## Slide 7
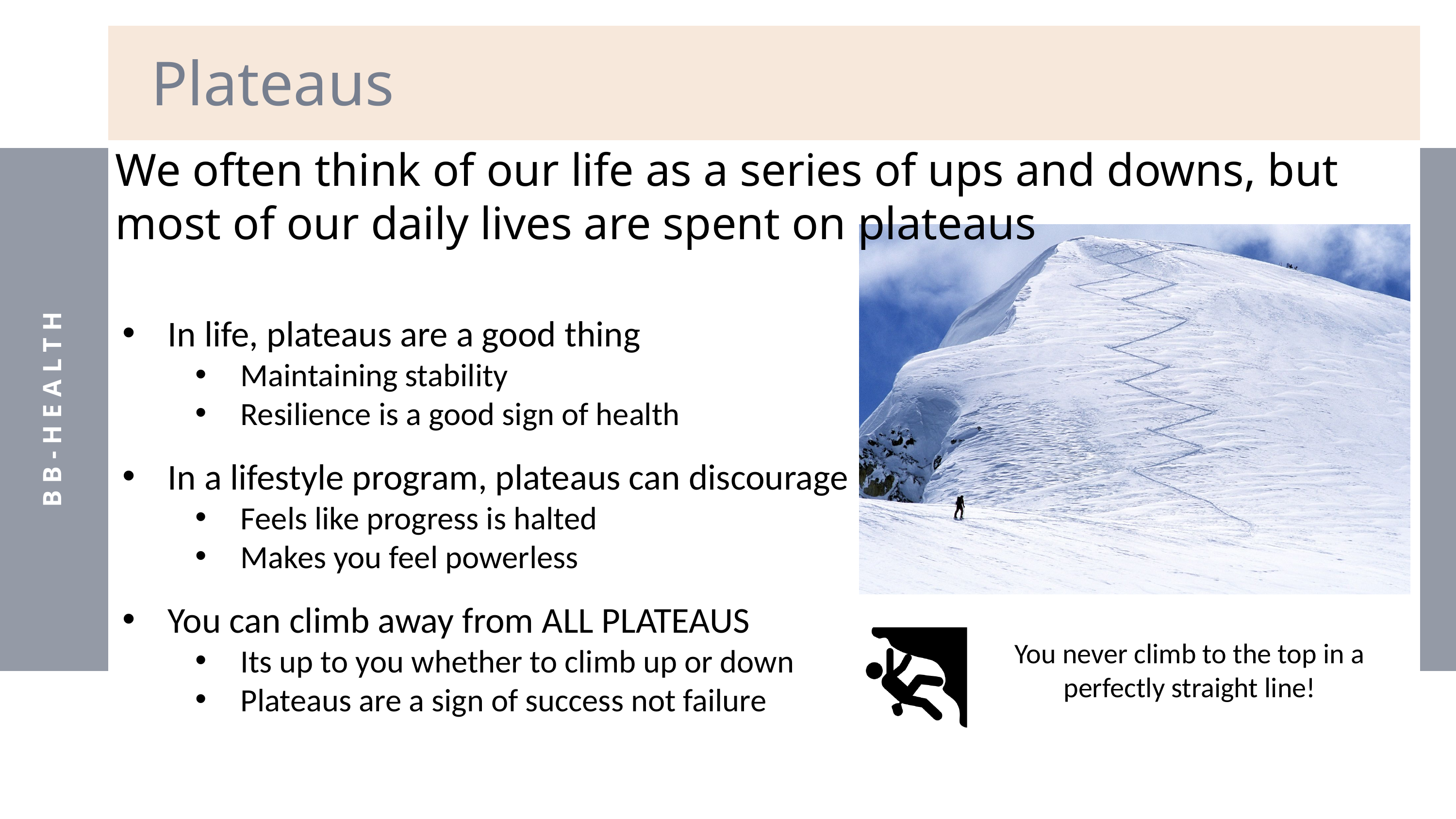

Plateaus
We often think of our life as a series of ups and downs, but most of our daily lives are spent on plateaus
In life, plateaus are a good thing
Maintaining stability
Resilience is a good sign of health
In a lifestyle program, plateaus can discourage
Feels like progress is halted
Makes you feel powerless
You can climb away from ALL PLATEAUS
Its up to you whether to climb up or down
Plateaus are a sign of success not failure
BB-HEALTH
You never climb to the top in a perfectly straight line!

## Slide 8
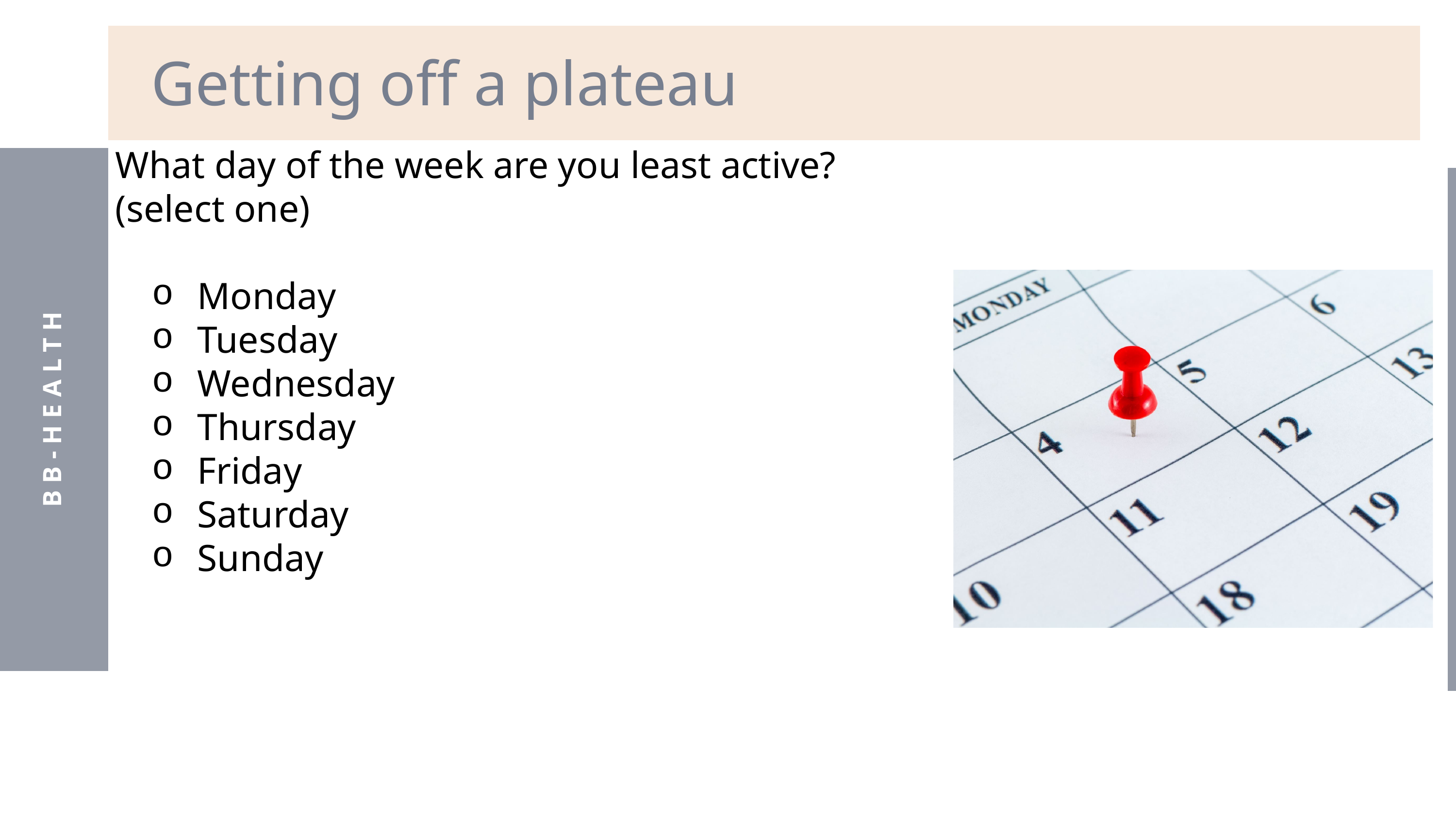

Getting off a plateau
What day of the week are you least active? (select one)
Monday
Tuesday
Wednesday
Thursday
Friday
Saturday
Sunday
BB-HEALTH

## Slide 9
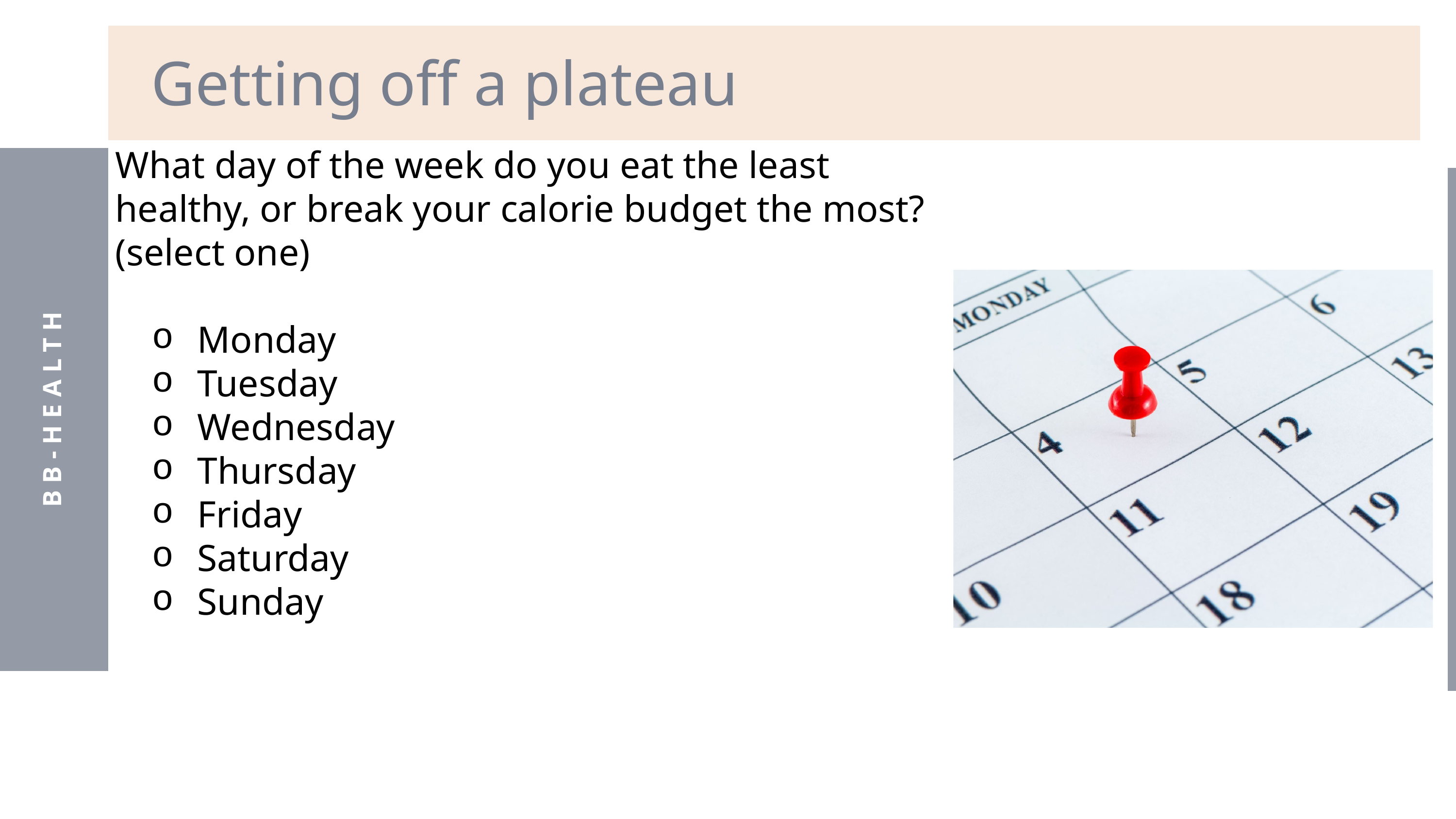

Getting off a plateau
What day of the week do you eat the least healthy, or break your calorie budget the most? (select one)
Monday
Tuesday
Wednesday
Thursday
Friday
Saturday
Sunday
BB-HEALTH

## Slide 10
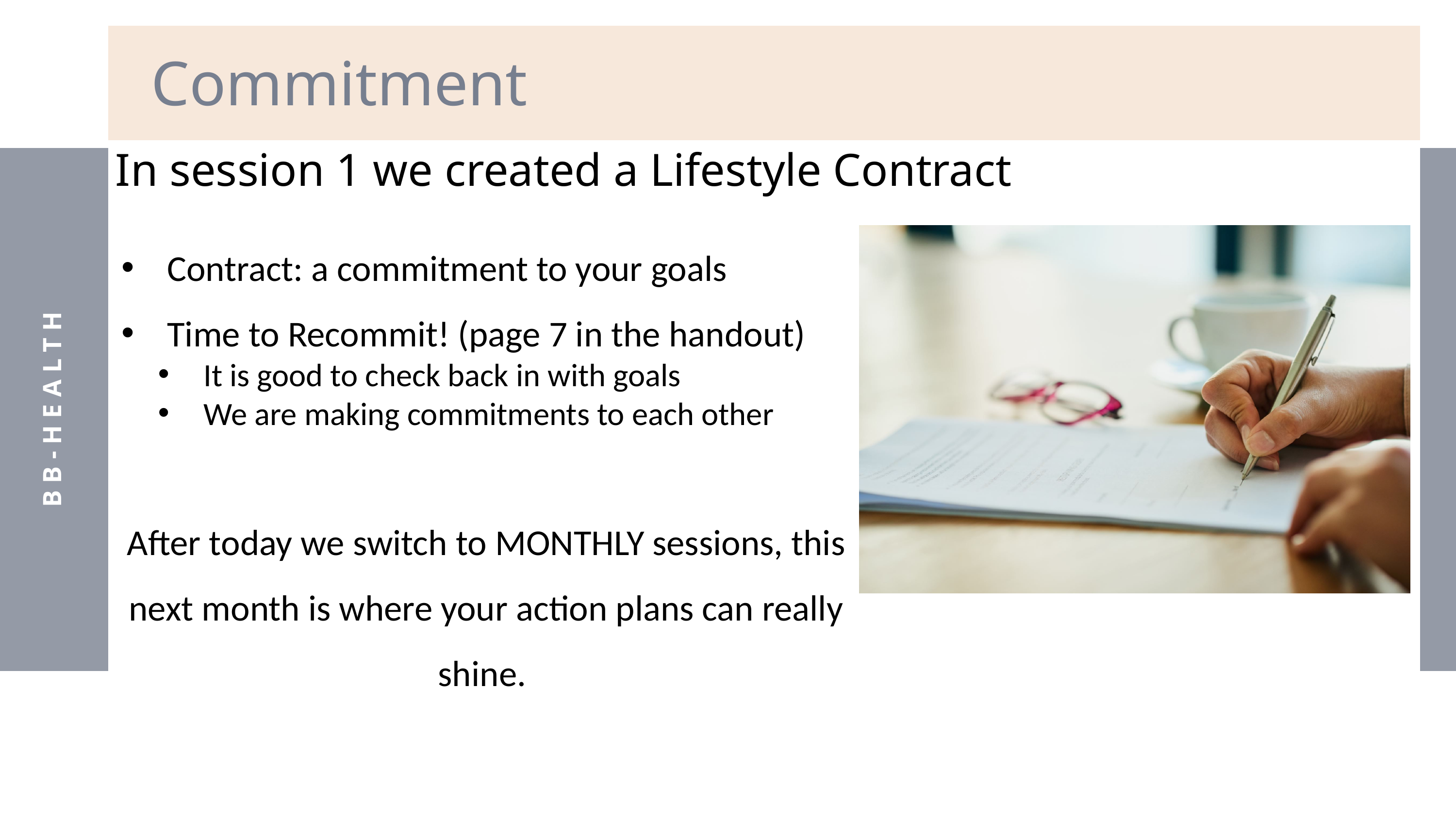

Commitment
In session 1 we created a Lifestyle Contract
Contract: a commitment to your goals
Time to Recommit! (page 7 in the handout)
It is good to check back in with goals
We are making commitments to each other
After today we switch to MONTHLY sessions, this next month is where your action plans can really shine.
BB-HEALTH

## Slide 11
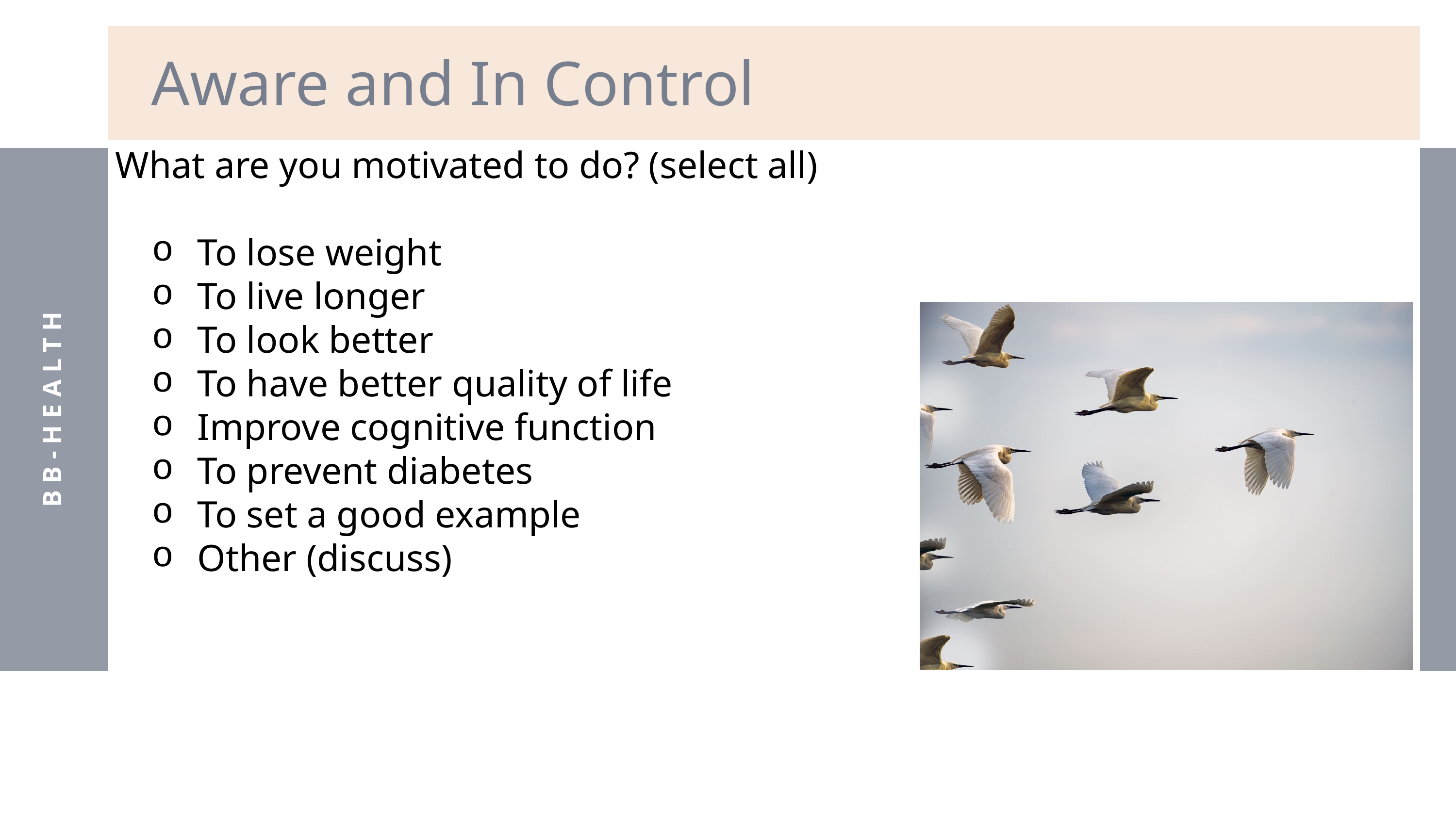

Aware and In Control
What are you motivated to do? (select all)
To lose weight
To live longer
To look better
To have better quality of life
Improve cognitive function
To prevent diabetes
To set a good example
Other (discuss)
BB-HEALTH

## Slide 12
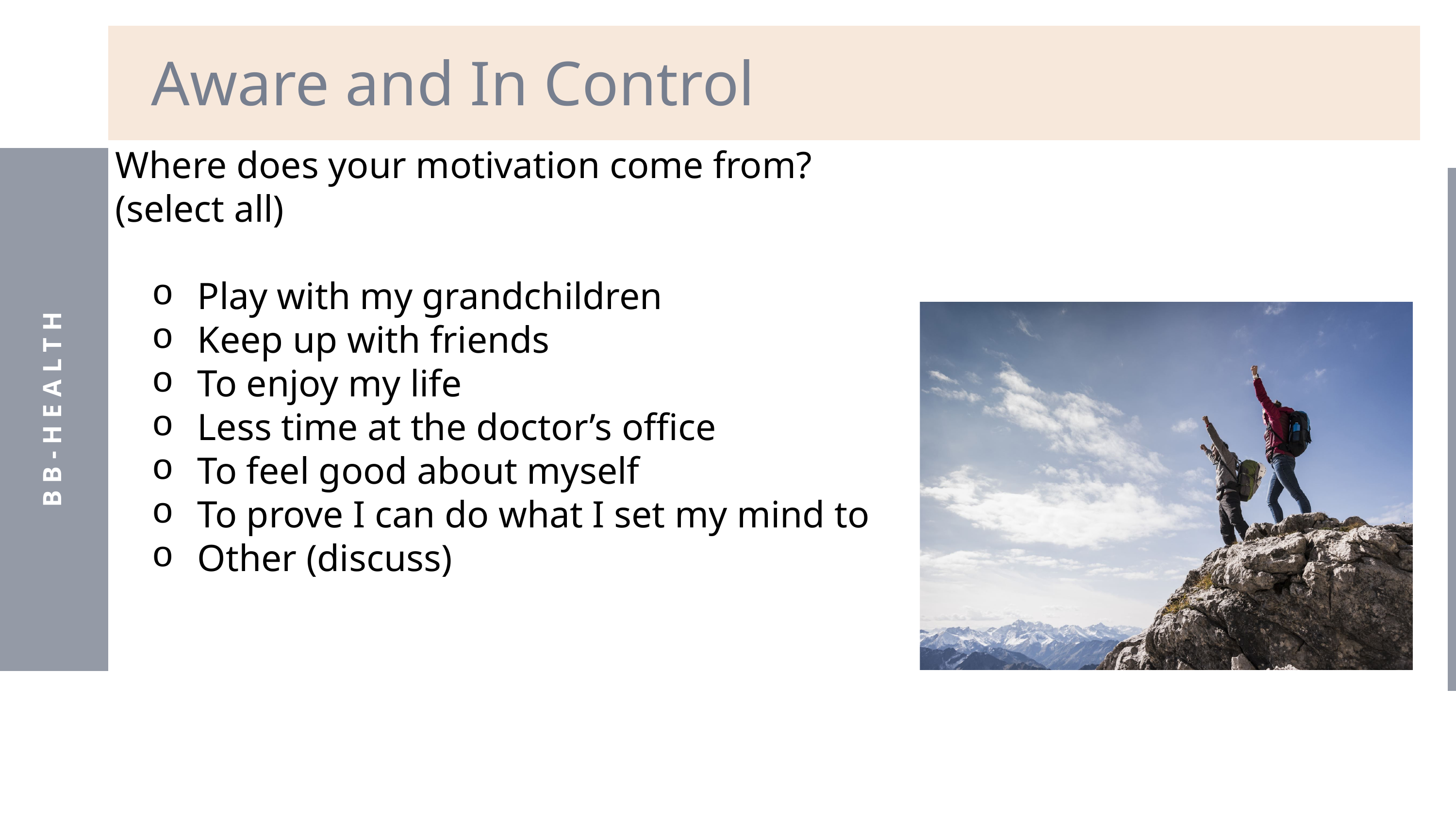

Aware and In Control
Where does your motivation come from? (select all)
Play with my grandchildren
Keep up with friends
To enjoy my life
Less time at the doctor’s office
To feel good about myself
To prove I can do what I set my mind to
Other (discuss)
BB-HEALTH

## Slide 13
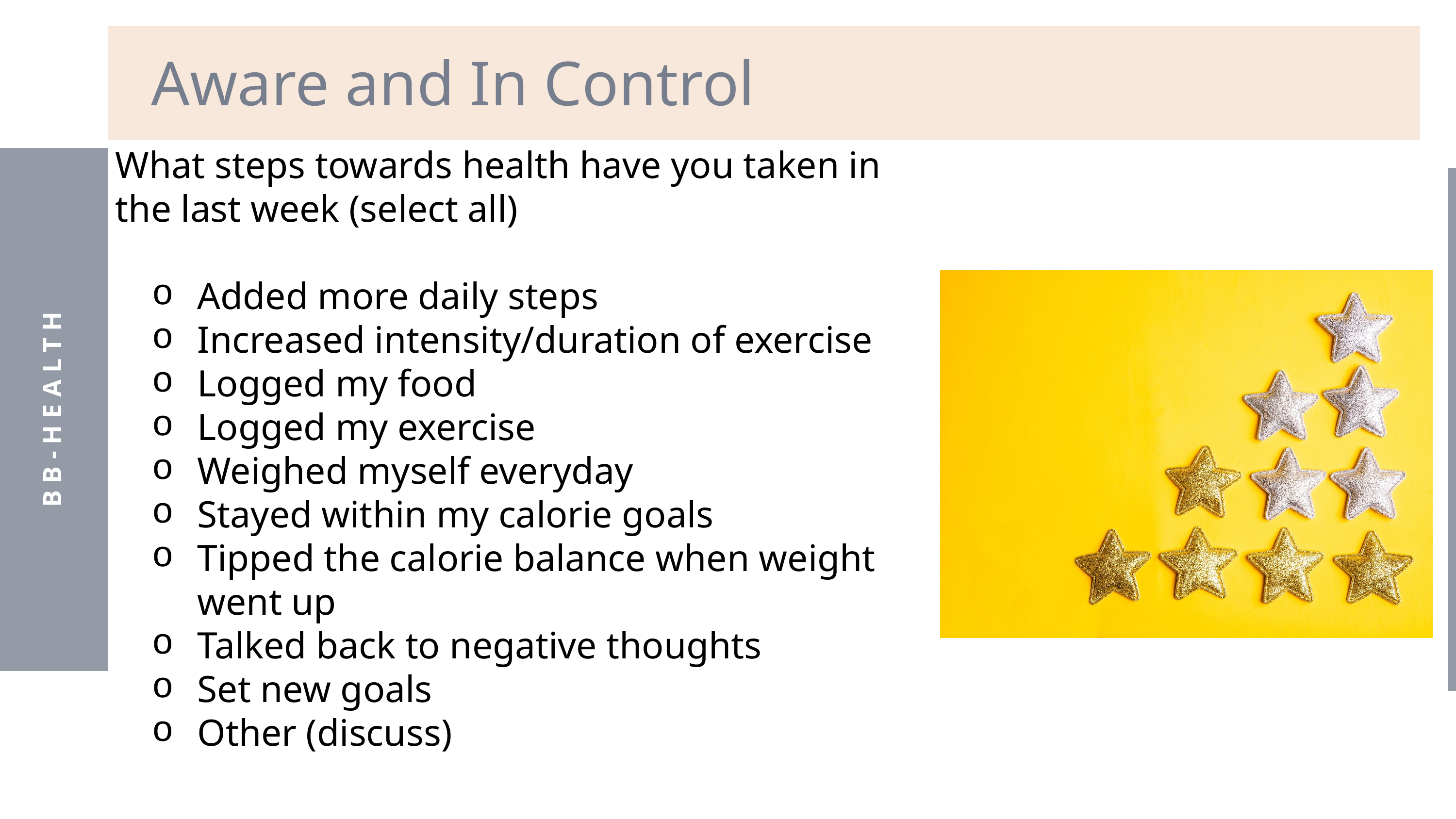

Aware and In Control
What steps towards health have you taken in the last week (select all)
Added more daily steps
Increased intensity/duration of exercise
Logged my food
Logged my exercise
Weighed myself everyday
Stayed within my calorie goals
Tipped the calorie balance when weight went up
Talked back to negative thoughts
Set new goals
Other (discuss)
BB-HEALTH

## Slide 14
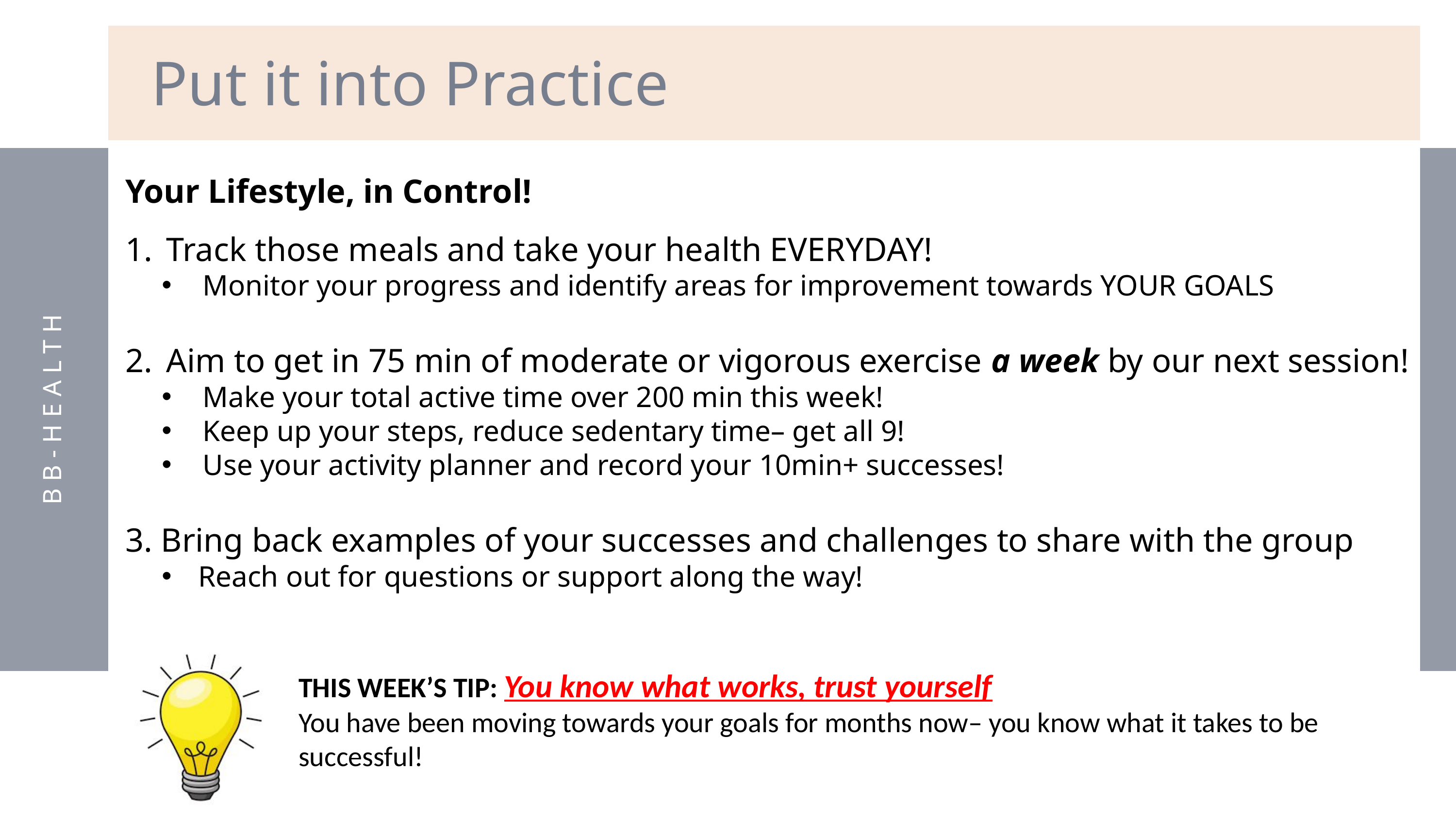

Put it into Practice
Your Lifestyle, in Control!
Track those meals and take your health EVERYDAY!
Monitor your progress and identify areas for improvement towards YOUR GOALS
Aim to get in 75 min of moderate or vigorous exercise a week by our next session!
Make your total active time over 200 min this week!
Keep up your steps, reduce sedentary time– get all 9!
Use your activity planner and record your 10min+ successes!
3. Bring back examples of your successes and challenges to share with the group
Reach out for questions or support along the way!
BB-HEALTH
THIS WEEK’S TIP: You know what works, trust yourself
You have been moving towards your goals for months now– you know what it takes to be successful!

## Slide 15
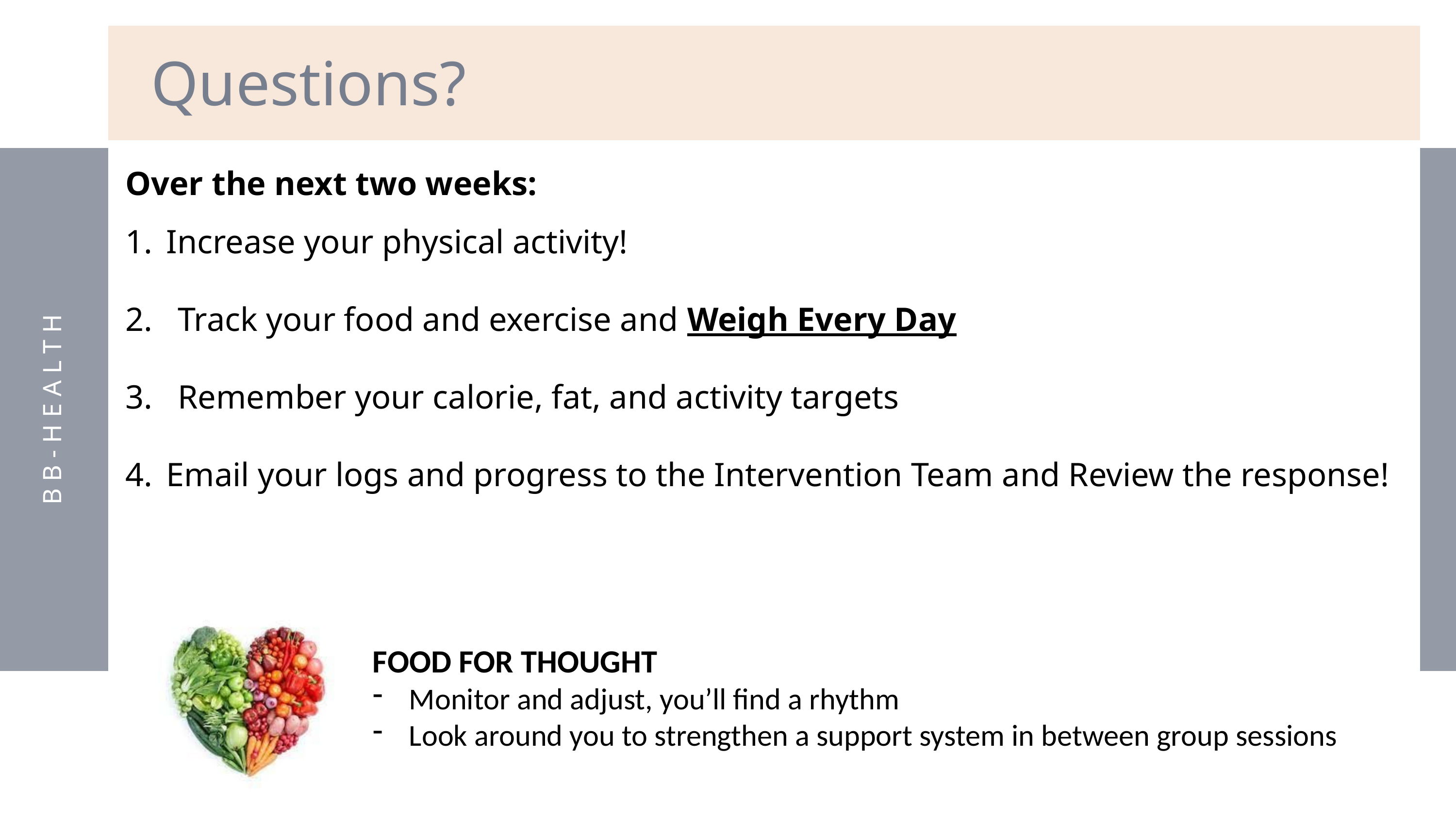

Questions?
Over the next two weeks:
Increase your physical activity!
2. Track your food and exercise and Weigh Every Day
3. Remember your calorie, fat, and activity targets
Email your logs and progress to the Intervention Team and Review the response!
BB-HEALTH
FOOD FOR THOUGHT
Monitor and adjust, you’ll find a rhythm
Look around you to strengthen a support system in between group sessions
