# Supplementary material for: Development and Feasibility of an eHealth Diabetes Prevention Program Adapted for Older Adults—Results from a Randomized Control Pilot Study
Source: Nutrients. 2024 Mar 23;16(7):930. doi: 10.3390/nu16070930 (PMC11154527; doi:10.3390/nu16070930)
Supplement: Supplementary file 1 [file nutrients-16-00930-s001.zip › Session14_SUPP.pdf]

BB-HEALTH

e-Healthy Older Adults Modification

Diabetes Prevention Program

Session #14
LIFESTYLE:
Aware & In
Control!

Group Opening Discussion

BB-HEALTH

WELCOME BACK! <u>**HOW WAS THE LAST 2 WEEKS**</u>?

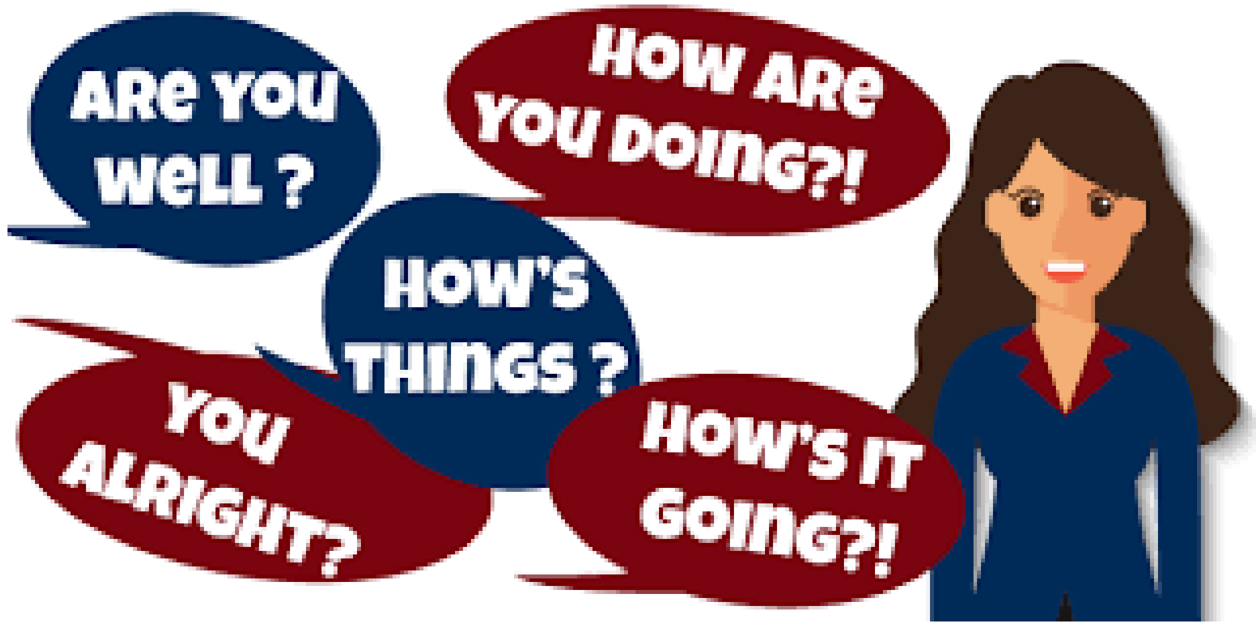

1. Did you track every meal?
2. Did you weigh every day, & record your health today?
3. Did you stay on your path towards success?
4. Did you increase your moderate and vigorous exercise?
5. Did you email the Intervention Team and review the response?
6. What were your unique challenges and successes?

REMINDER: CHARGE YOUR FITBIT & SCALE DURING THIS MEETING!

Aware and In Control

What food from last week was highest in calories?

Was it also the highest in fat?

What meal had the most calories?

- Breakfast
- Lunch
- Dinner
- Snack
- Other (discuss)

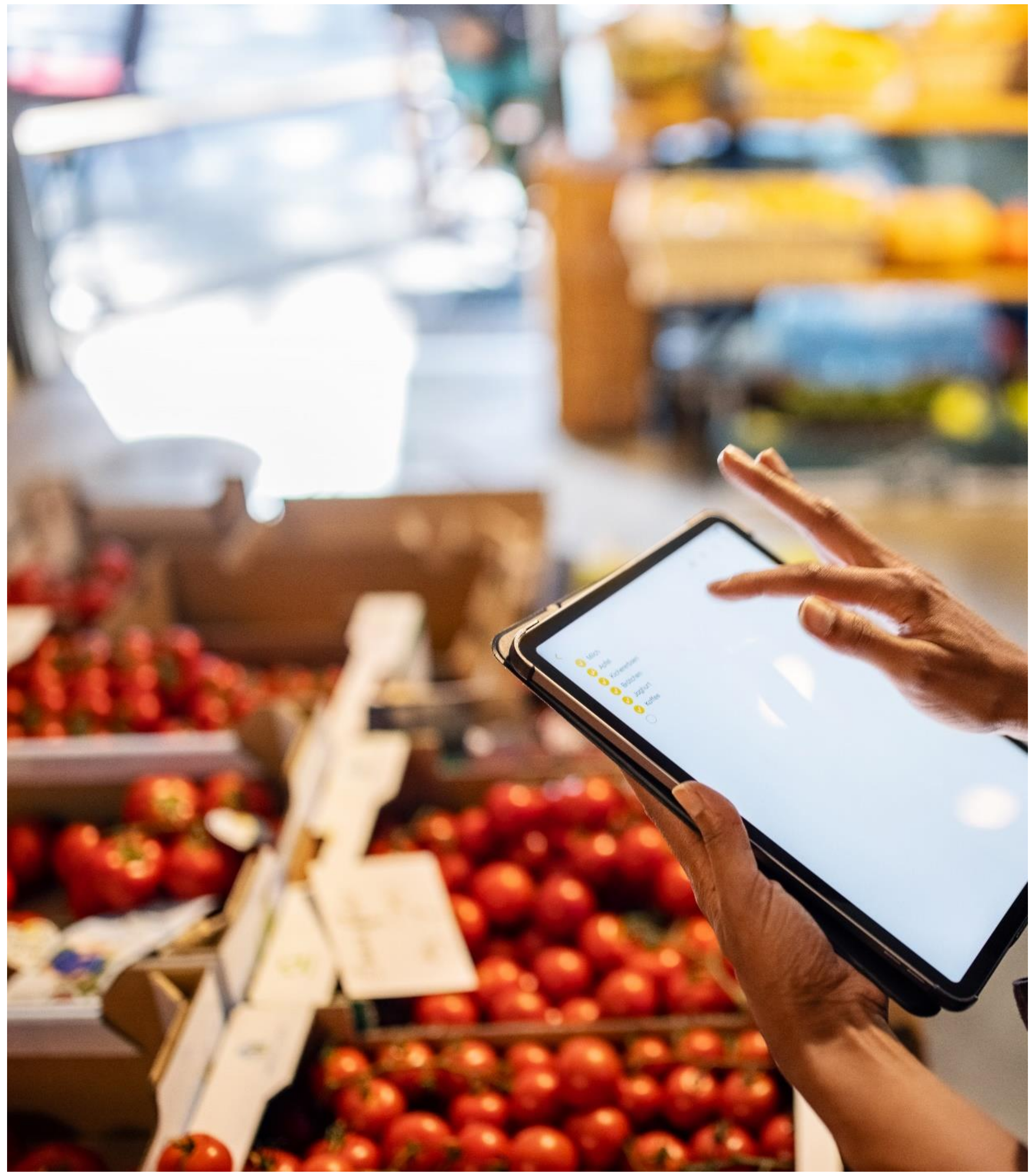

Aware and In Control

BB-HEALTH

Do you get hungry throughout the day?

- Never, I stick to mealtimes and routine snacks
- Rarely, I occasionally nibble
- Sometimes, I try not to but still get hungry outside of meals
- Often, I am usually hungry in between meals
- Always, I'm always hungry and eat frequently through the day

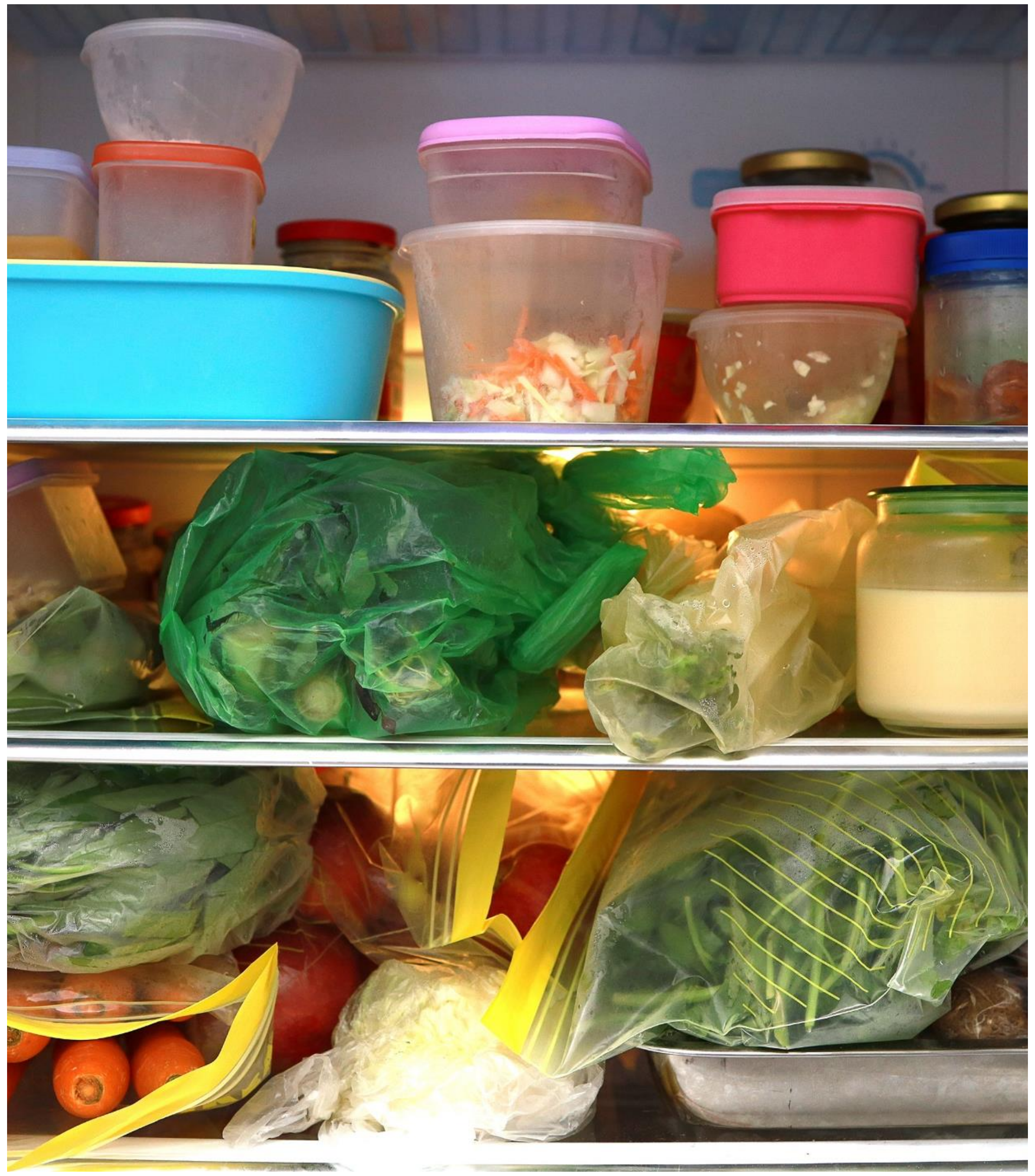

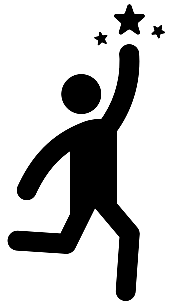

Getting hungry happens! Let's discuss healthy snacking plans. Put your favorite healthy snack in the chat, or ask the group for ideas to **help the SNACK ATTACK!**

Attendance Matters

BB-HEALTH

Group attendance is related to success

- Showing up here is showing up for yourself and the rest of the group
- Evidence more involvement = better outcomes

Accountability

- Routine from group is changing
- Reinforcement from peers

Maintenance is different from change

- Internalizing a habit – **healthy is who you are**
- Remaining aware and in control

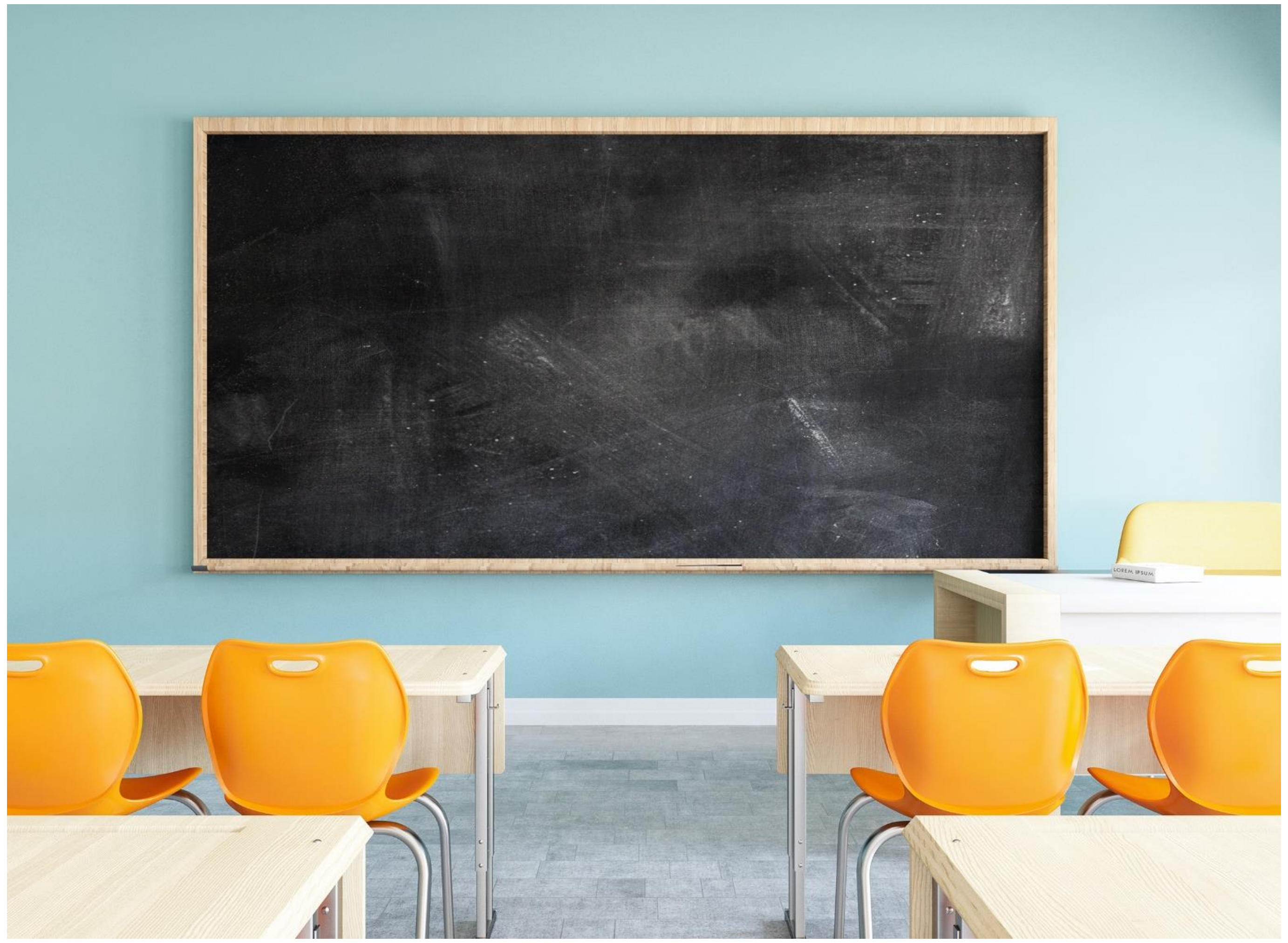

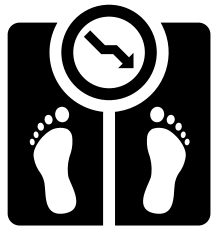

More attendance and participation is associated with greater weight loss!

Who are the long-term maintainers?

BB-HEALTH

Long-term weight loss is possible, but requires changing your mindset.
So what makes someone more likely for success?

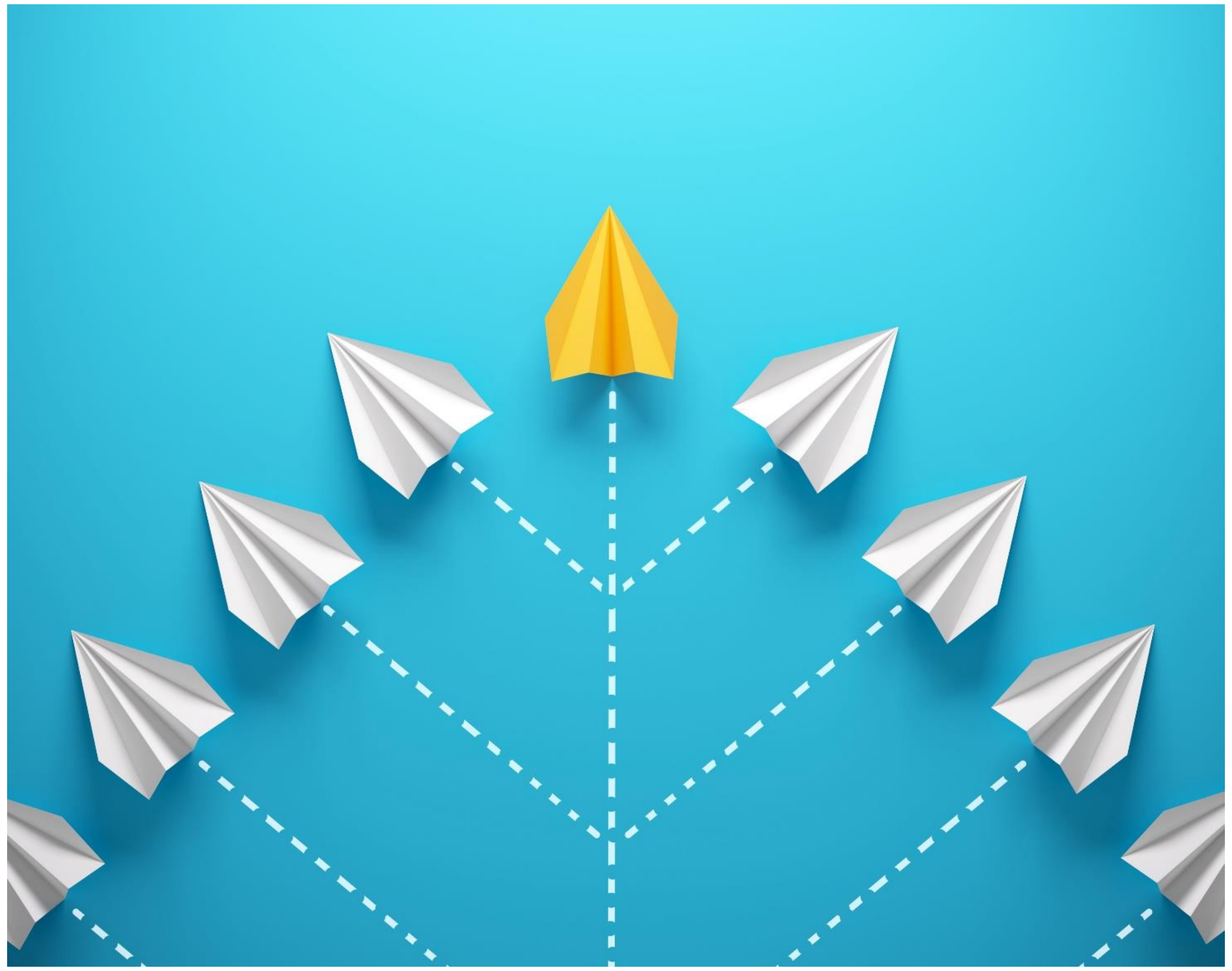

1. Focus on gradual, consistent weight loss
   - 1-2 lbs a week
2. Maintain self-monitoring
   - Aware and control to respond as needed
3. Building a plan that works for your real life
   - Not temporary, extreme, or fad diet
4. Setting successive, achievable goals
   - It's a lifestyle, not a destination
5. Find what works, and keep doing it!
   - <u>You came to class, to tried new things, you did the work to see what makes success–</u>

<u>you owe it to yourself to keep it up!</u>

Plateaus

We often think of our life as a series of ups and downs, but most of our daily lives are spent on plateaus

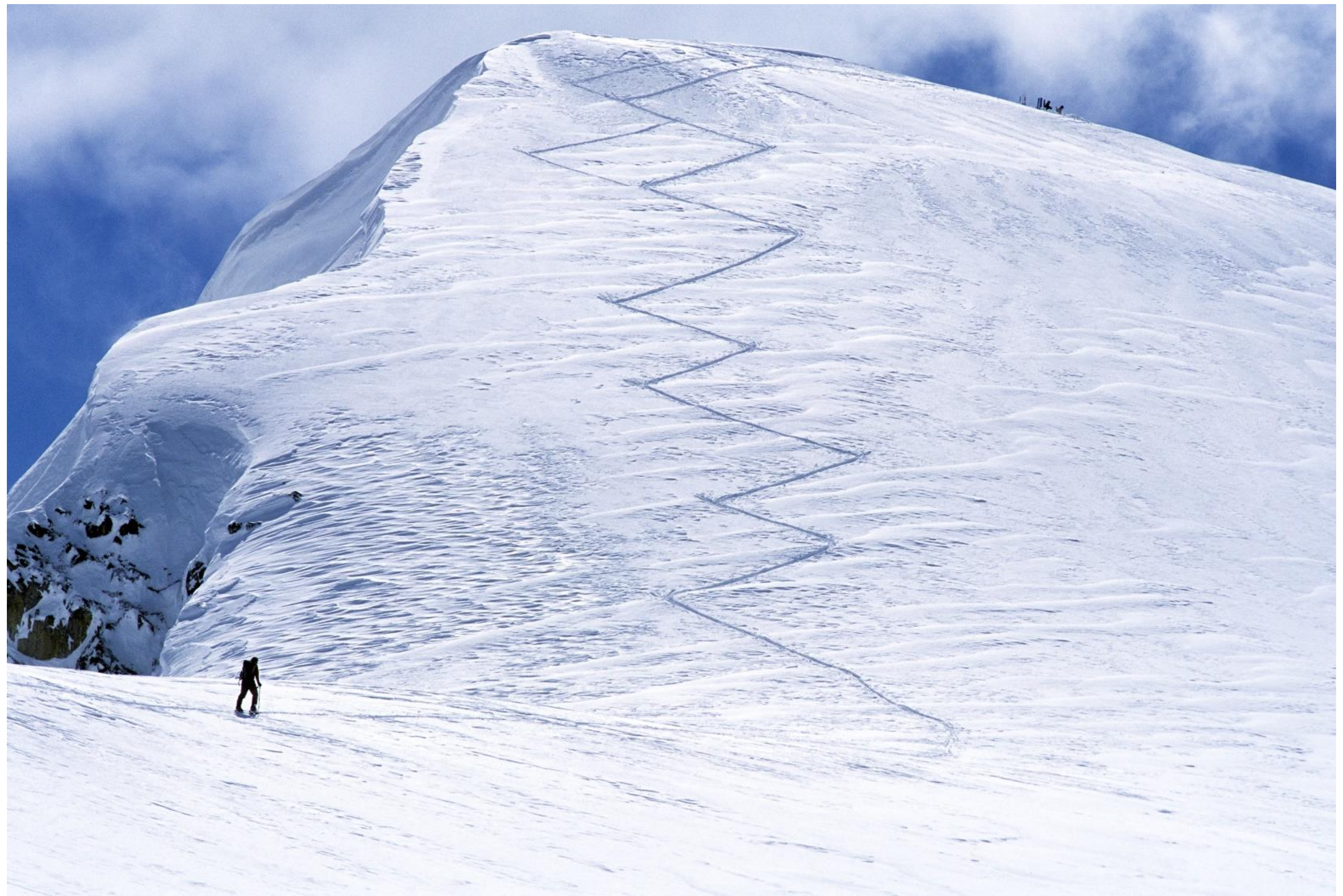

- In life, plateaus are a good thing
  - Maintaining stability
  - Resilience is a good sign of health
- In a lifestyle program, plateaus can discourage
  - Feels like progress is halted
  - Makes you feel powerless
- You can climb away from ALL PLATEAUS
  - Its up to you whether to climb up or down
  - Plateaus are a sign of success not failure

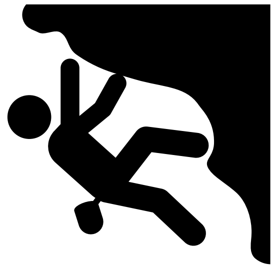

You never climb to the top in a perfectly straight line!

Getting off a plateau

What day of the week are you least active? (select one)

- Monday
- Tuesday
- Wednesday
- Thursday
- Friday
- Saturday
- Sunday

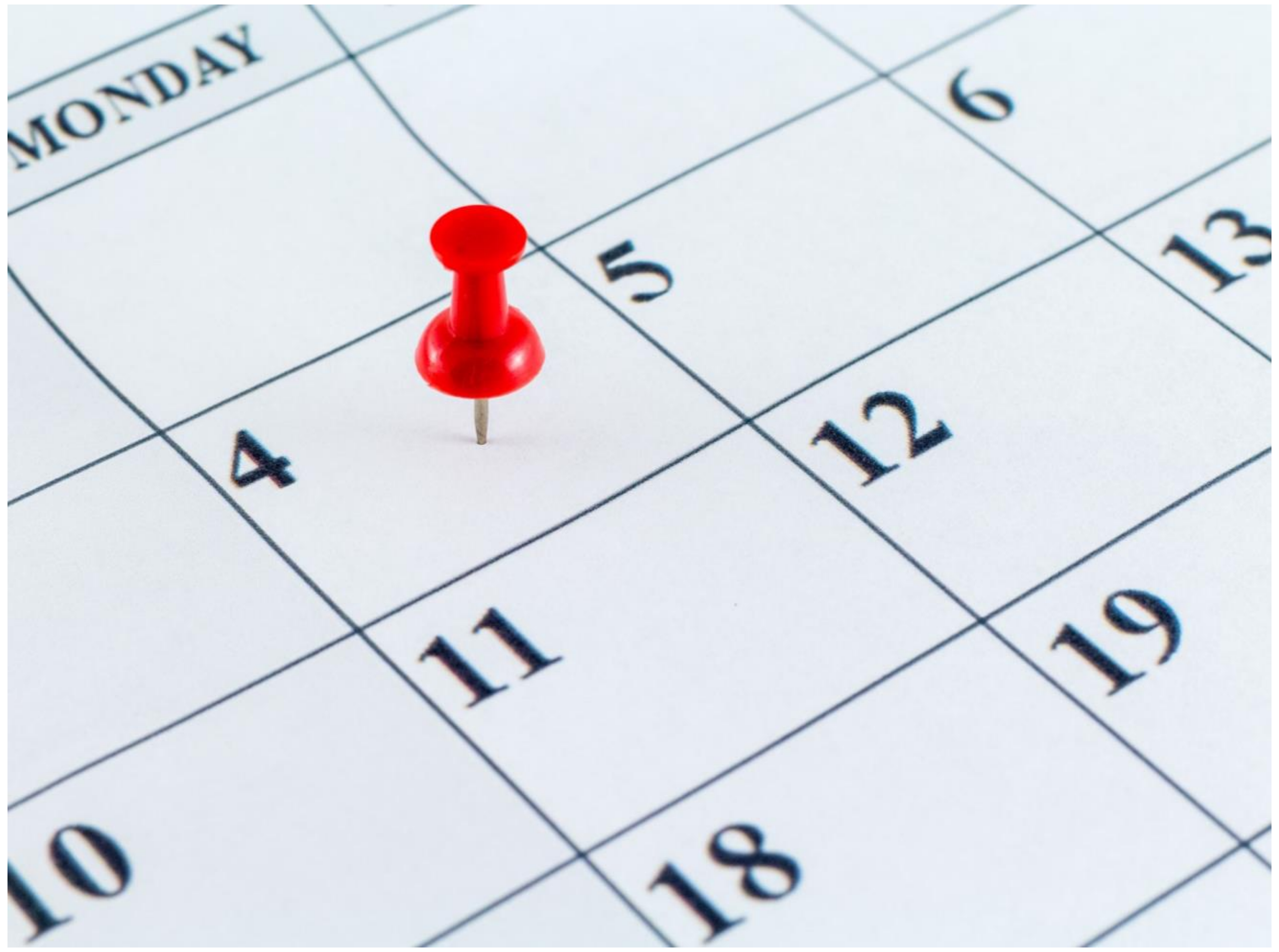

Getting off a plateau

What day of the week do you eat the least healthy, or break your calorie budget the most? (select one)

- Monday
- Tuesday
- Wednesday
- Thursday
- Friday
- Saturday
- Sunday

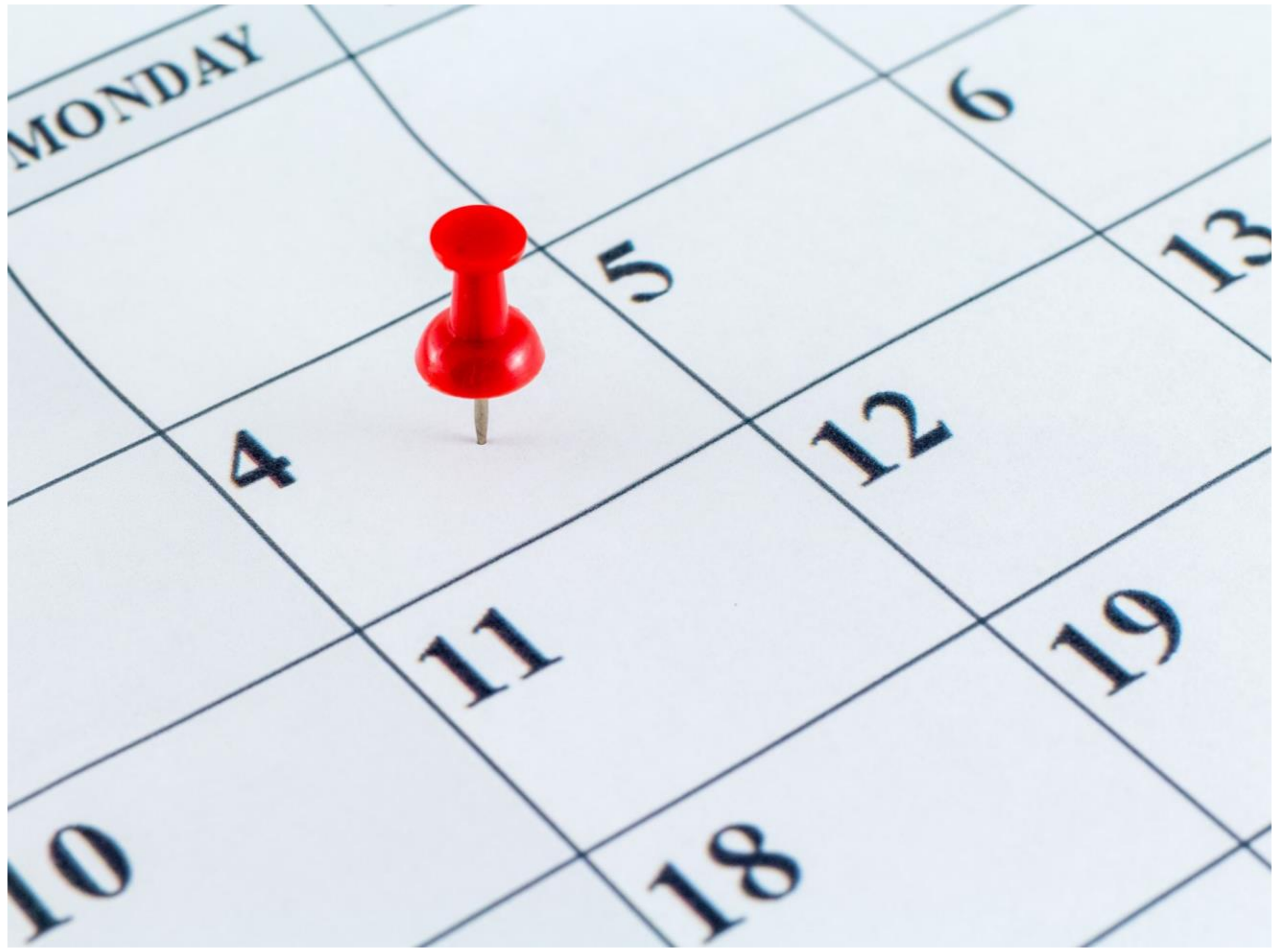

Commitment

BB-HEALTH

In session 1 we created a Lifestyle Contract

- Contract: a commitment to your goals
- Time to Recommit! (page 7 in the handout)
  - It is good to check back in with goals
  - We are making commitments to each other

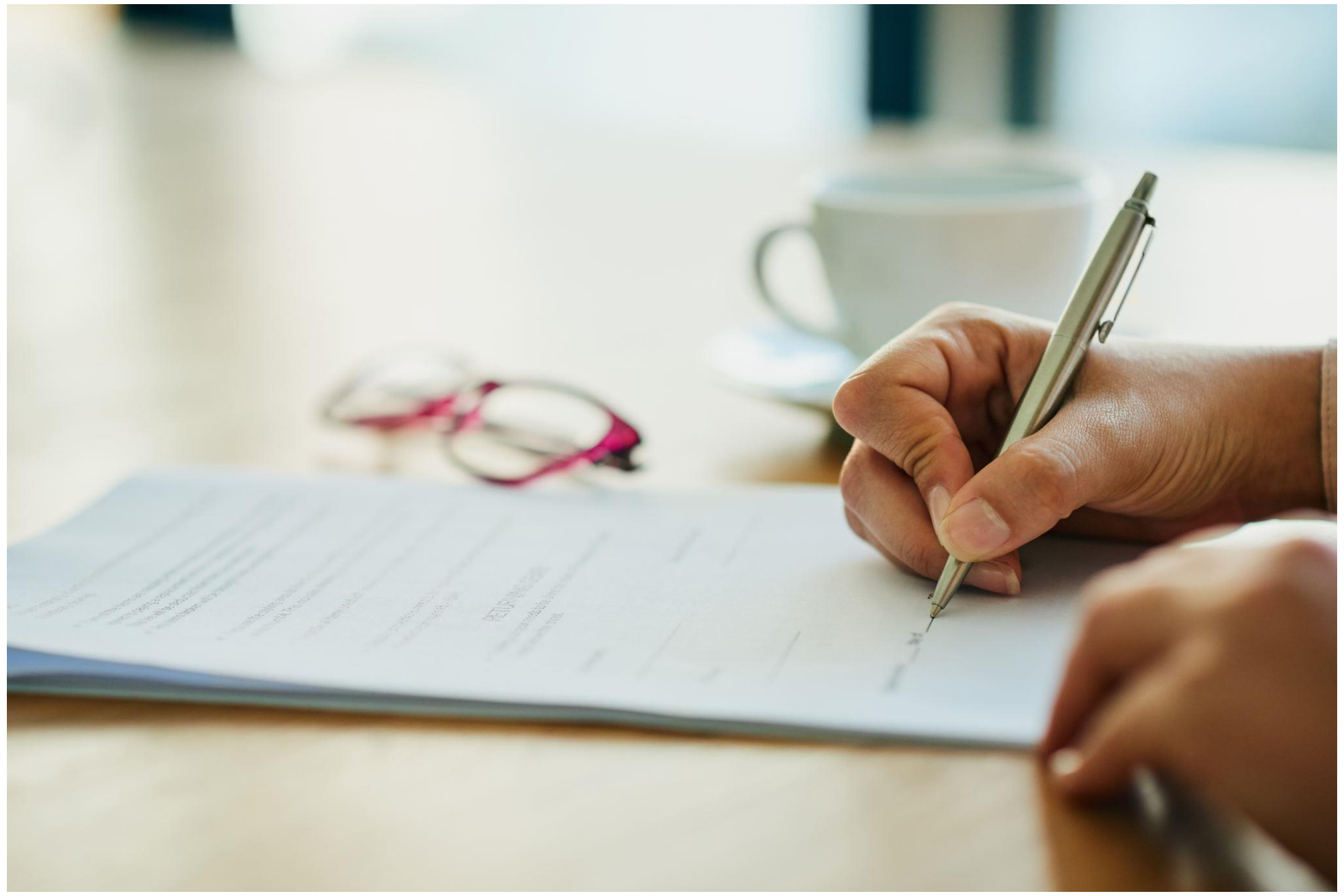

After today we switch to MONTHLY sessions, this next month is where your action plans can really shine.

Aware and In Control

BB-HEALTH

What are you motivated to do? (select all)

- To lose weight
- To live longer
- To look better
- To have better quality of life
- Improve cognitive function
- To prevent diabetes
- To set a good example
- Other (discuss)

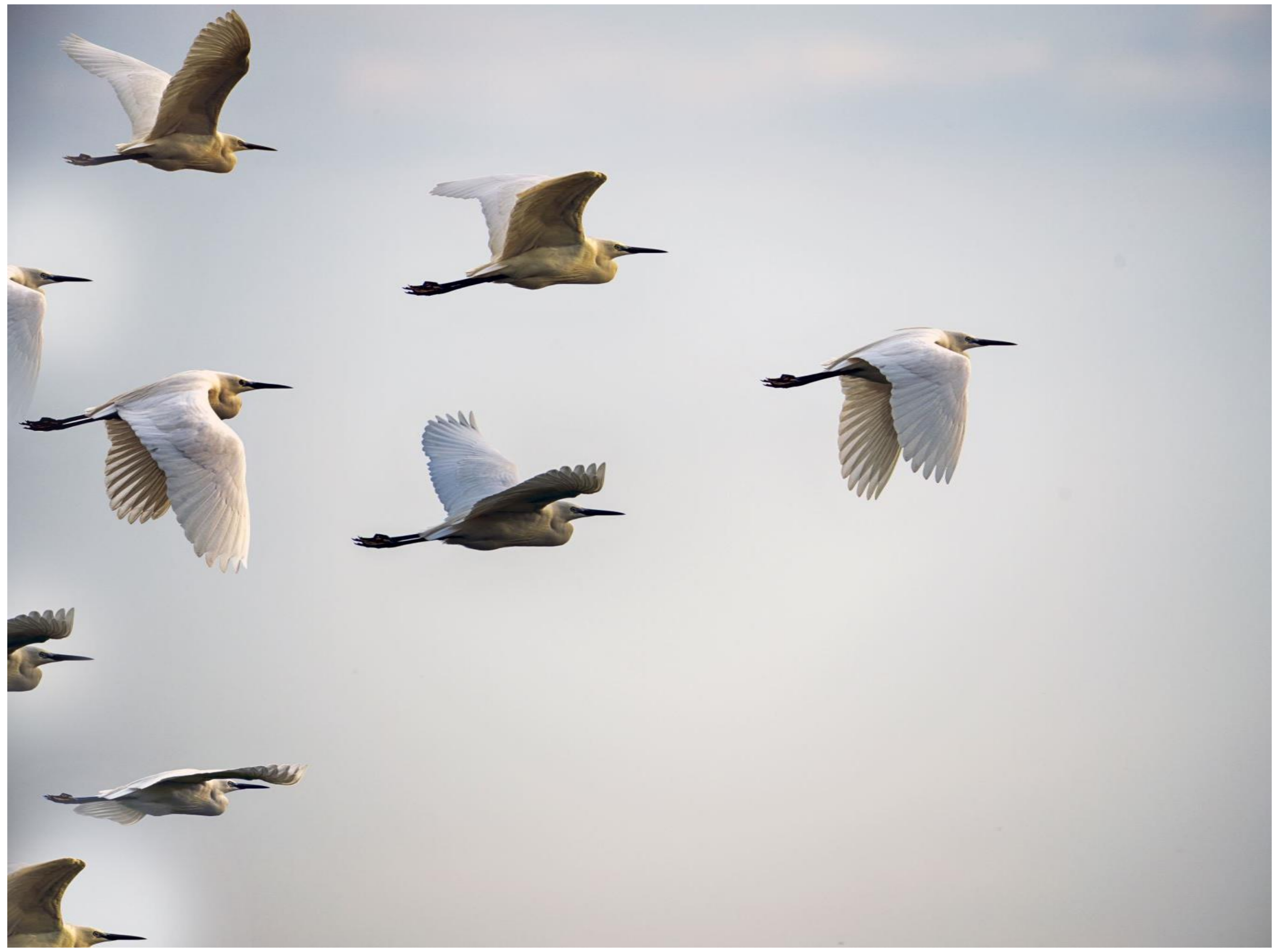

Aware and In Control

Where does your motivation come from? (select all)

- Play with my grandchildren
- Keep up with friends
- To enjoy my life
- Less time at the doctor's office
- To feel good about myself
- To prove I can do what I set my mind to
- Other (discuss)

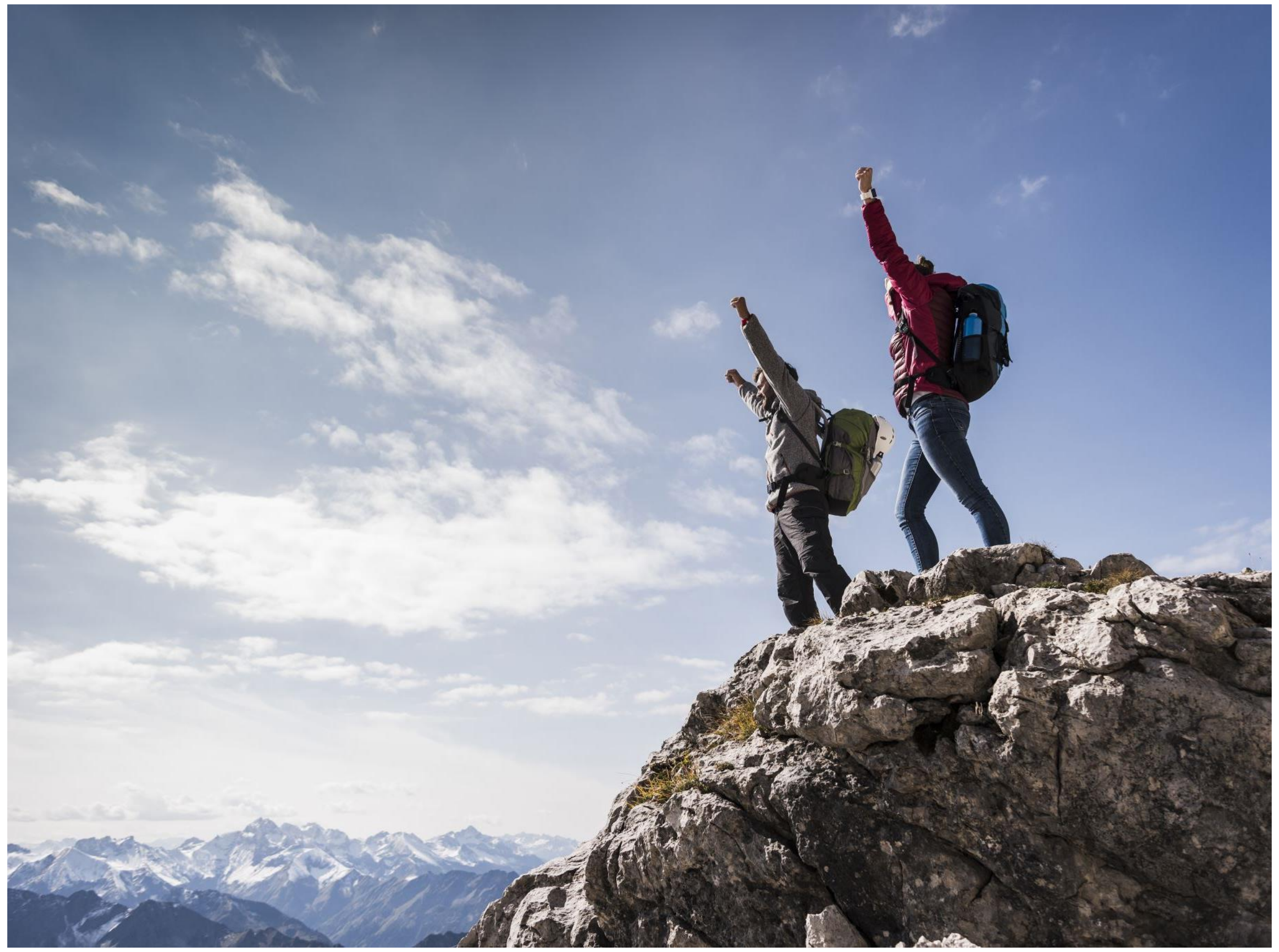

Aware and In Control

What steps towards health have you taken in the last week (select all)

- Added more daily steps
- Increased intensity/duration of exercise
- Logged my food
- Logged my exercise
- Weighed myself everyday
- Stayed within my calorie goals
- Tipped the calorie balance when weight went up
- Talked back to negative thoughts
- Set new goals
- Other (discuss)

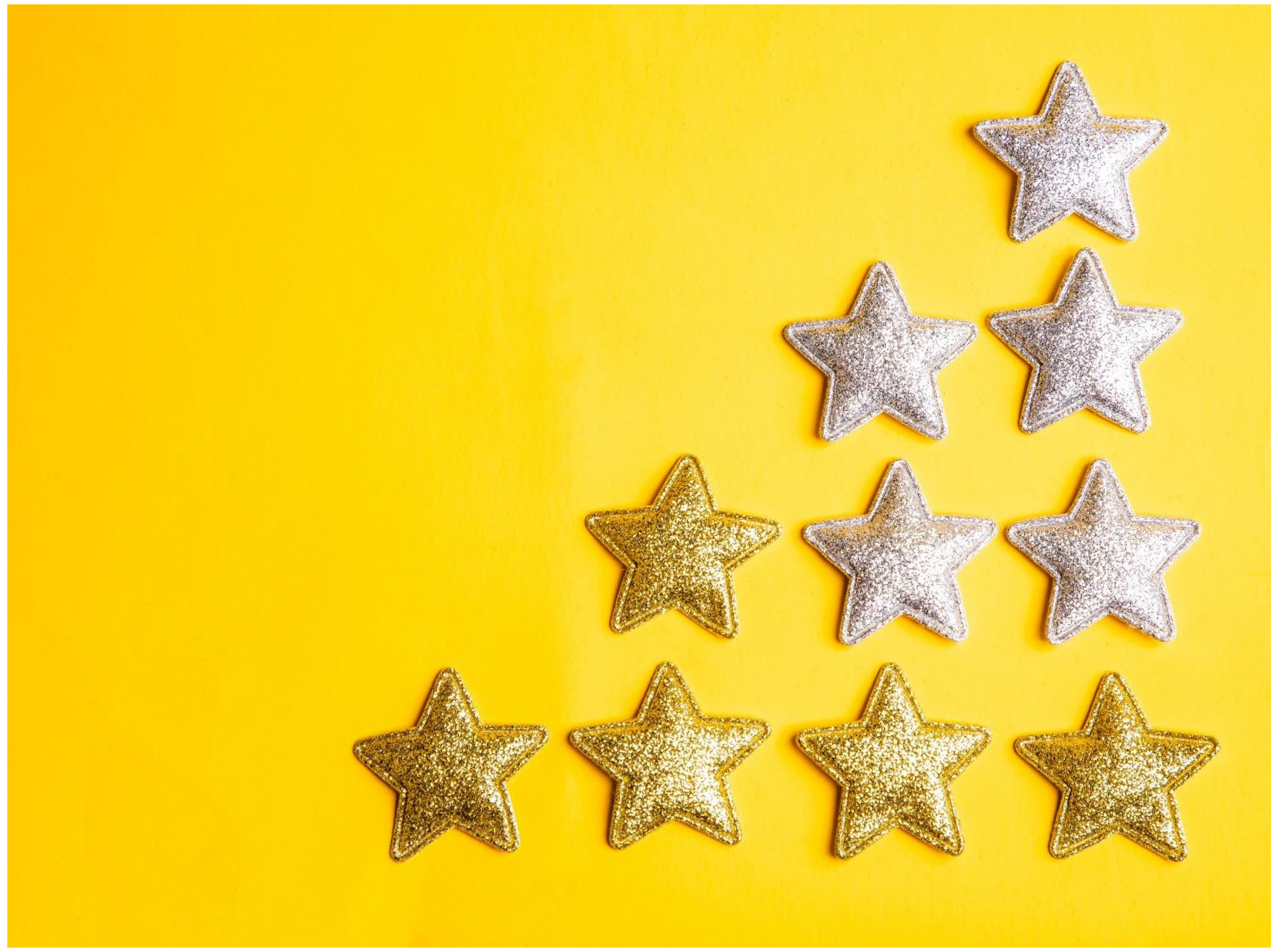

Put it into Practice

BB-HEALTH

Your Lifestyle, in Control!

1. Track those meals and take your health EVERYDAY!
   - Monitor your progress and identify areas for improvement towards YOUR GOALS

2. Aim to get in 75 min of moderate or vigorous exercise ***a week*** by our next session!
   - Make your total active time over 200 min this week!
   - Keep up your steps, reduce sedentary time– get all 9!
   - Use your activity planner and record your 10min+ successes!

3. Bring back examples of your successes and challenges to share with the group
   - Reach out for questions or support along the way!

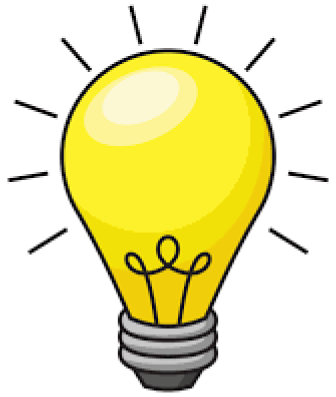

THIS WEEK'S TIP: ***You know what works, trust yourself***
You have been moving towards your goals for months now– you know what it takes to be successful!

Questions?

B B - H E A L T H

Over the next two weeks:

1. Increase your physical activity!

2. Track your food and exercise and **Weigh Every Day**

3. Remember your calorie, fat, and activity targets

4. Email your logs and progress to the Intervention Team and Review the response!

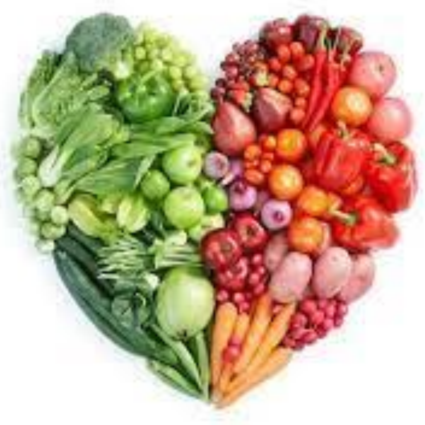

FOOD FOR THOUGHT

- Monitor and adjust, you'll find a rhythm
- Look around you to strengthen a support system in between group sessions
