# Supplementary material for: Development and Feasibility of an eHealth Diabetes Prevention Program Adapted for Older Adults—Results from a Randomized Control Pilot Study
Source: Nutrients. 2024 Mar 23;16(7):930. doi: 10.3390/nu16070930 (PMC11154527; doi:10.3390/nu16070930)
Supplement: Supplementary file 1 [file nutrients-16-00930-s001.zip › Session15.pptx]

## Slide 1
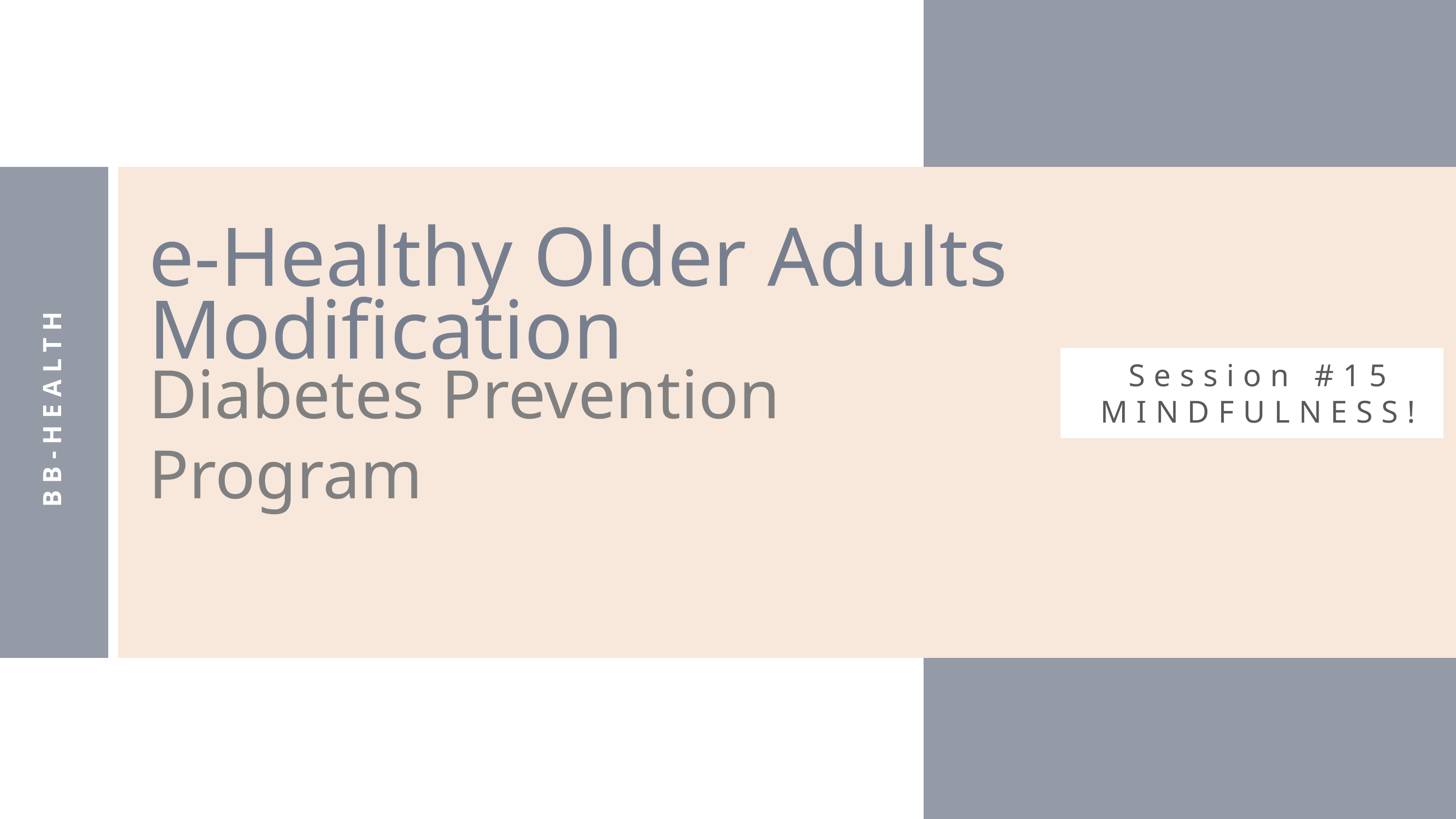

OPEN REPORTS
e-Healthy Older Adults Modification
Session #15
MINDFULNESS!
Diabetes Prevention Program
BB-HEALTH

## Slide 2
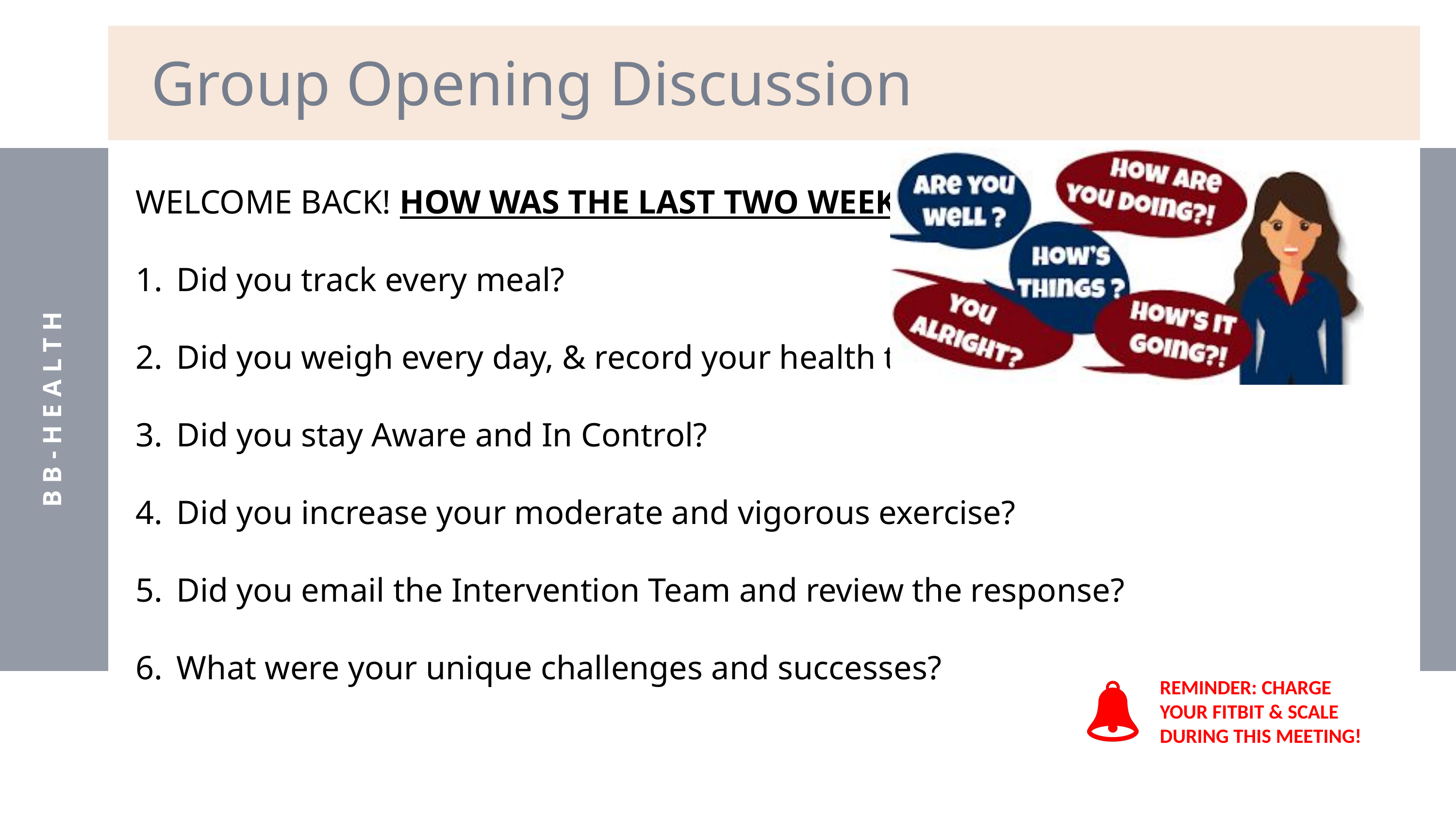

Group Opening Discussion
WELCOME BACK! HOW WAS THE LAST TWO WEEKS?
Did you track every meal?
Did you weigh every day, & record your health today?
Did you stay Aware and In Control?
Did you increase your moderate and vigorous exercise?
Did you email the Intervention Team and review the response?
What were your unique challenges and successes?
BB-HEALTH
REMINDER: CHARGE YOUR FITBIT & SCALE DURING THIS MEETING!

## Slide 3
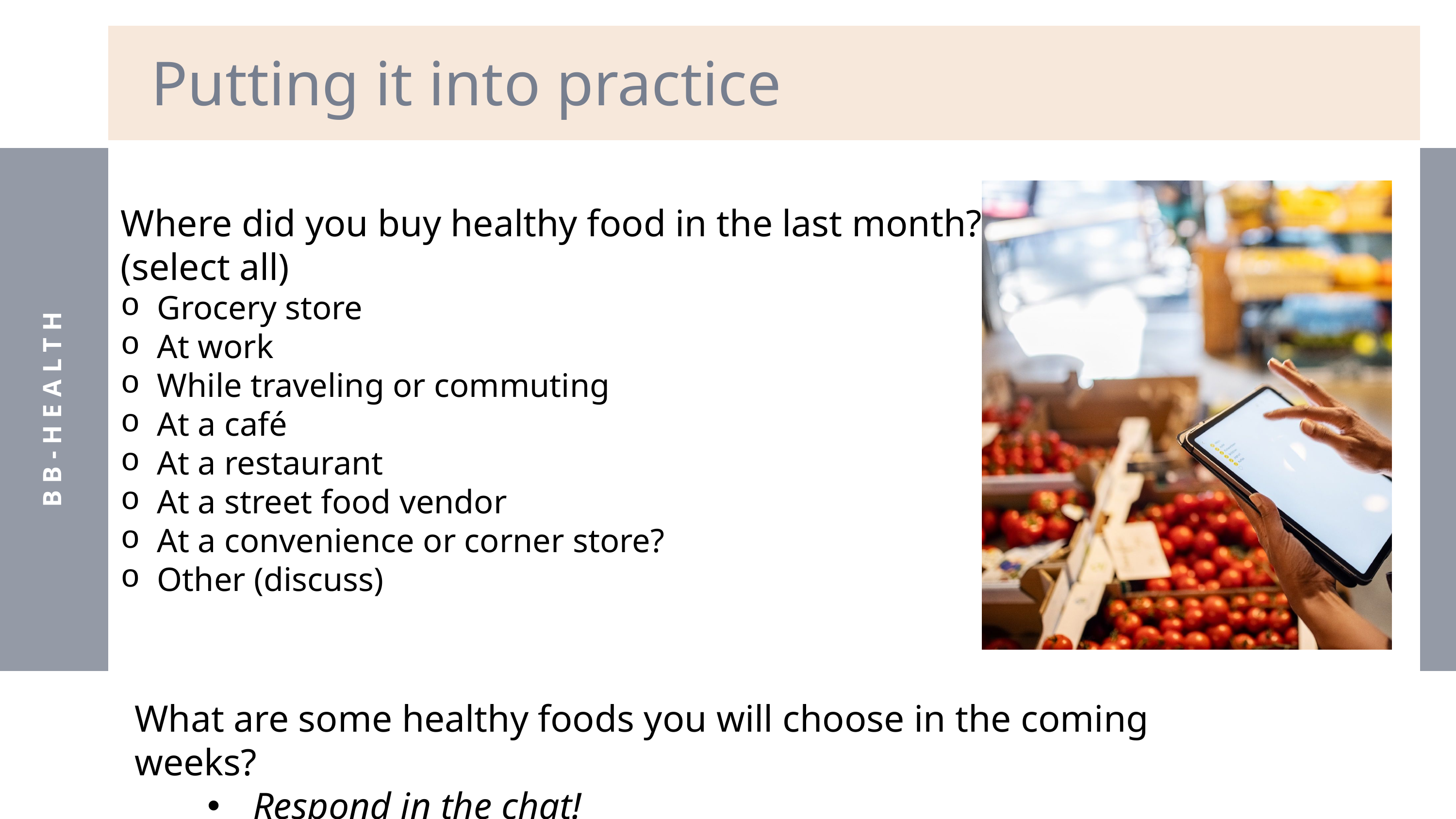

Putting it into practice
Where did you buy healthy food in the last month?
(select all)
Grocery store
At work
While traveling or commuting
At a café
At a restaurant
At a street food vendor
At a convenience or corner store?
Other (discuss)
BB-HEALTH
What are some healthy foods you will choose in the coming weeks?
Respond in the chat!

## Slide 4
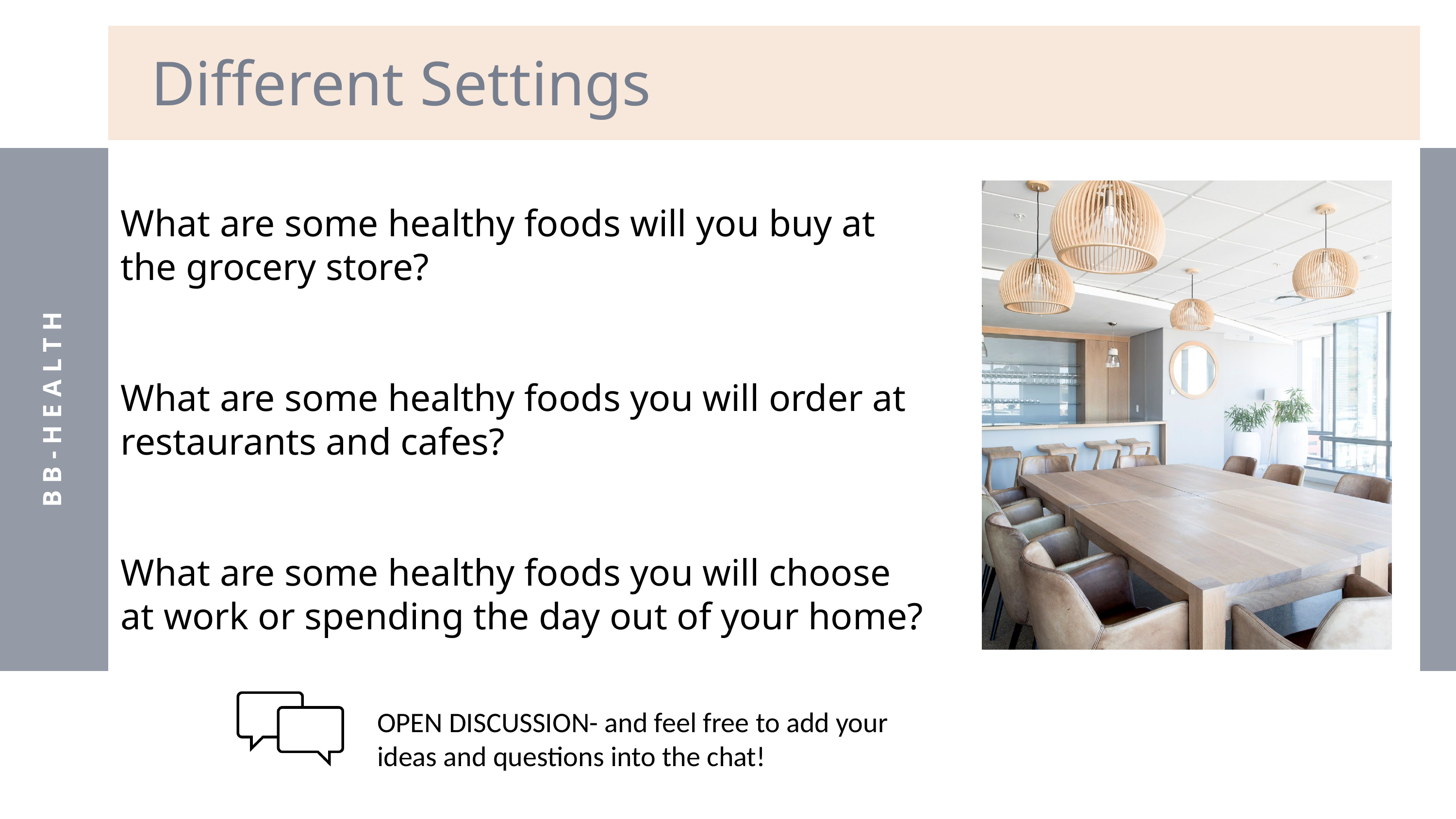

Different Settings
What are some healthy foods will you buy at the grocery store?
What are some healthy foods you will order at restaurants and cafes?
What are some healthy foods you will choose at work or spending the day out of your home?
BB-HEALTH
OPEN DISCUSSION- and feel free to add your ideas and questions into the chat!

## Slide 5
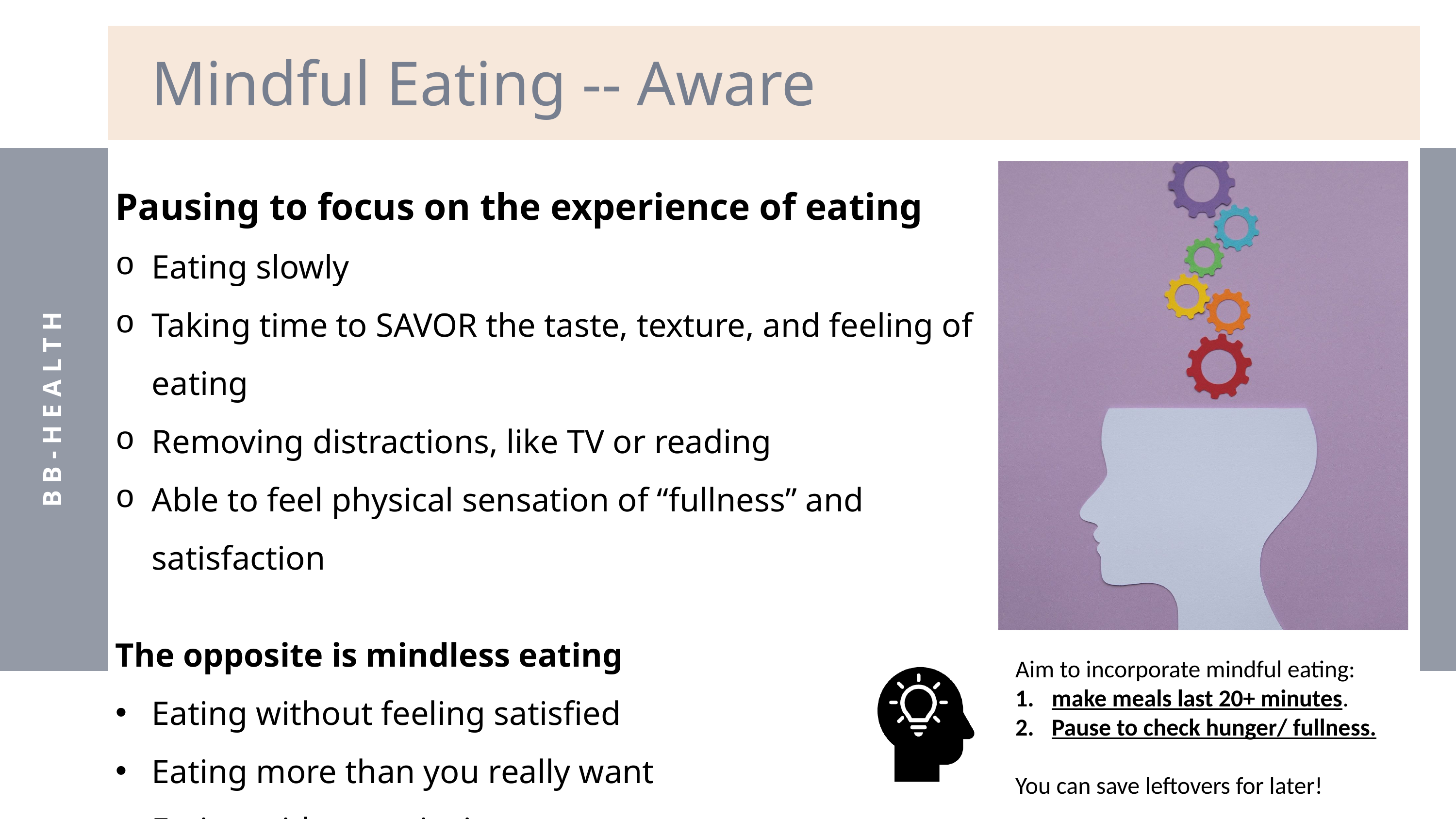

Mindful Eating -- Aware
Pausing to focus on the experience of eating
Eating slowly
Taking time to SAVOR the taste, texture, and feeling of eating
Removing distractions, like TV or reading
Able to feel physical sensation of “fullness” and satisfaction
The opposite is mindless eating
Eating without feeling satisfied
Eating more than you really want
Eating without enjoying
BB-HEALTH
Aim to incorporate mindful eating:
make meals last 20+ minutes.
Pause to check hunger/ fullness.
You can save leftovers for later!

## Slide 6
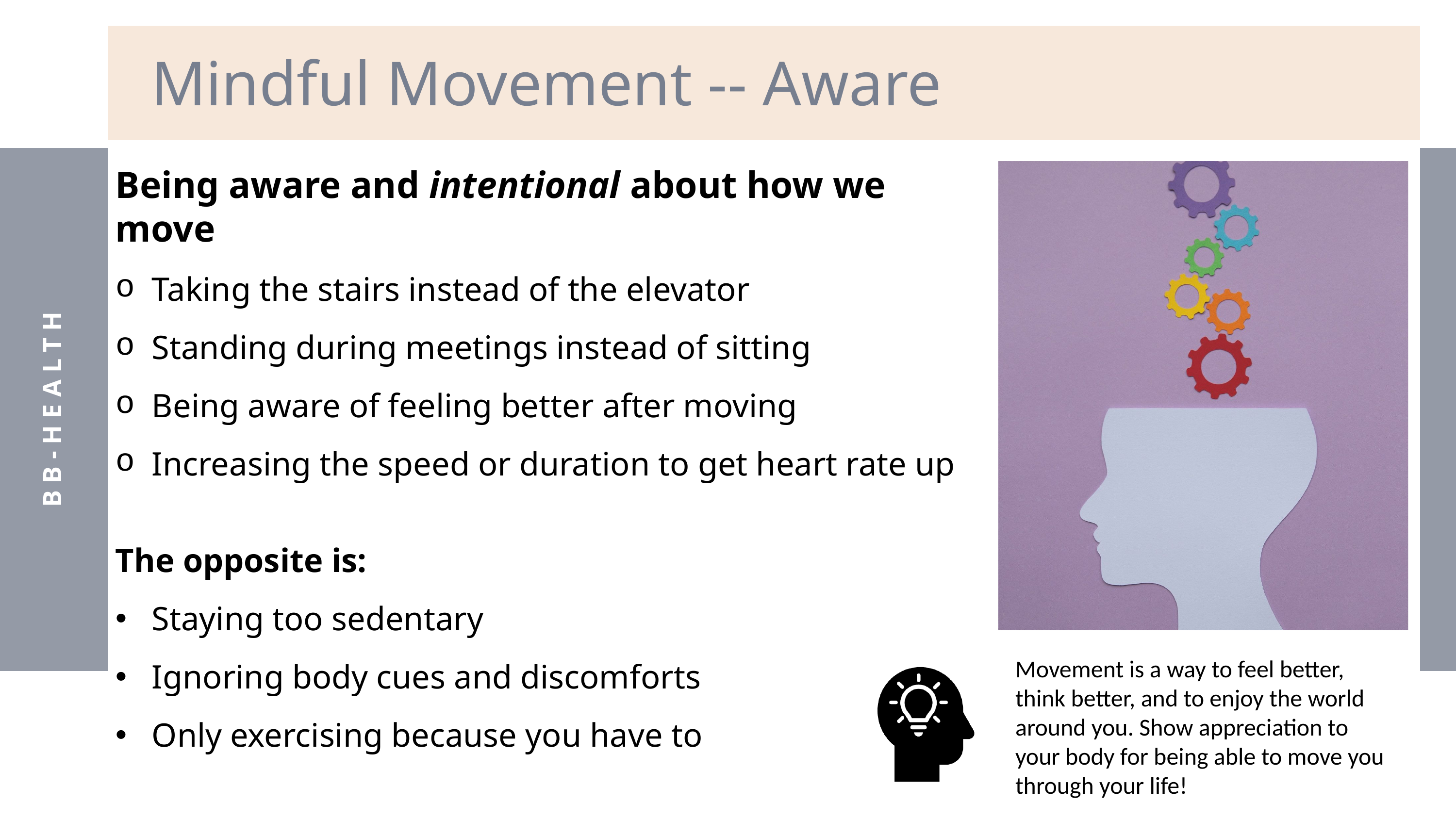

Mindful Movement -- Aware
Being aware and intentional about how we move
Taking the stairs instead of the elevator
Standing during meetings instead of sitting
Being aware of feeling better after moving
Increasing the speed or duration to get heart rate up
The opposite is:
Staying too sedentary
Ignoring body cues and discomforts
Only exercising because you have to
BB-HEALTH
Movement is a way to feel better, think better, and to enjoy the world around you. Show appreciation to your body for being able to move you through your life!

## Slide 7
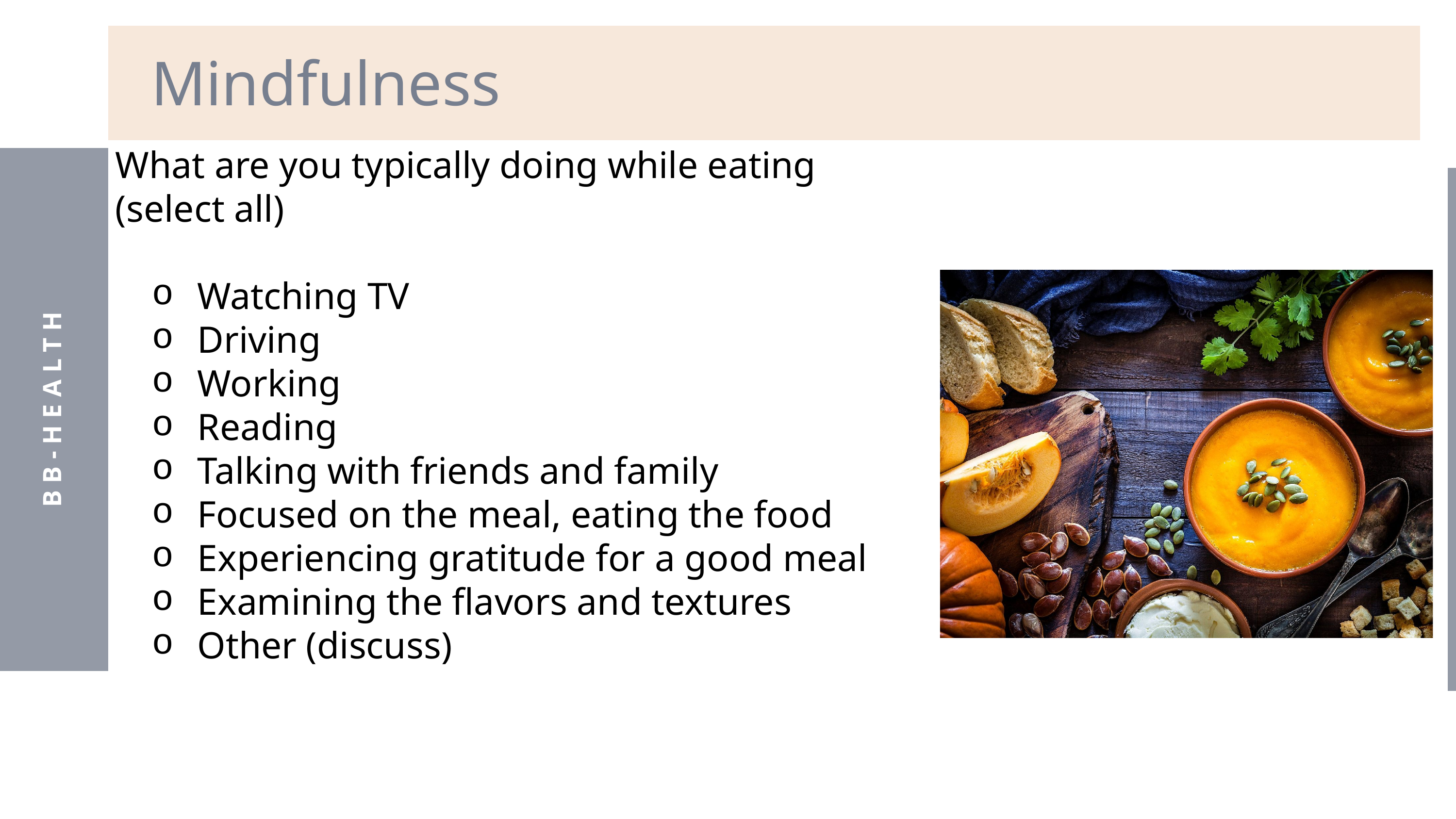

Mindfulness
What are you typically doing while eating (select all)
Watching TV
Driving
Working
Reading
Talking with friends and family
Focused on the meal, eating the food
Experiencing gratitude for a good meal
Examining the flavors and textures
Other (discuss)
BB-HEALTH

## Slide 8
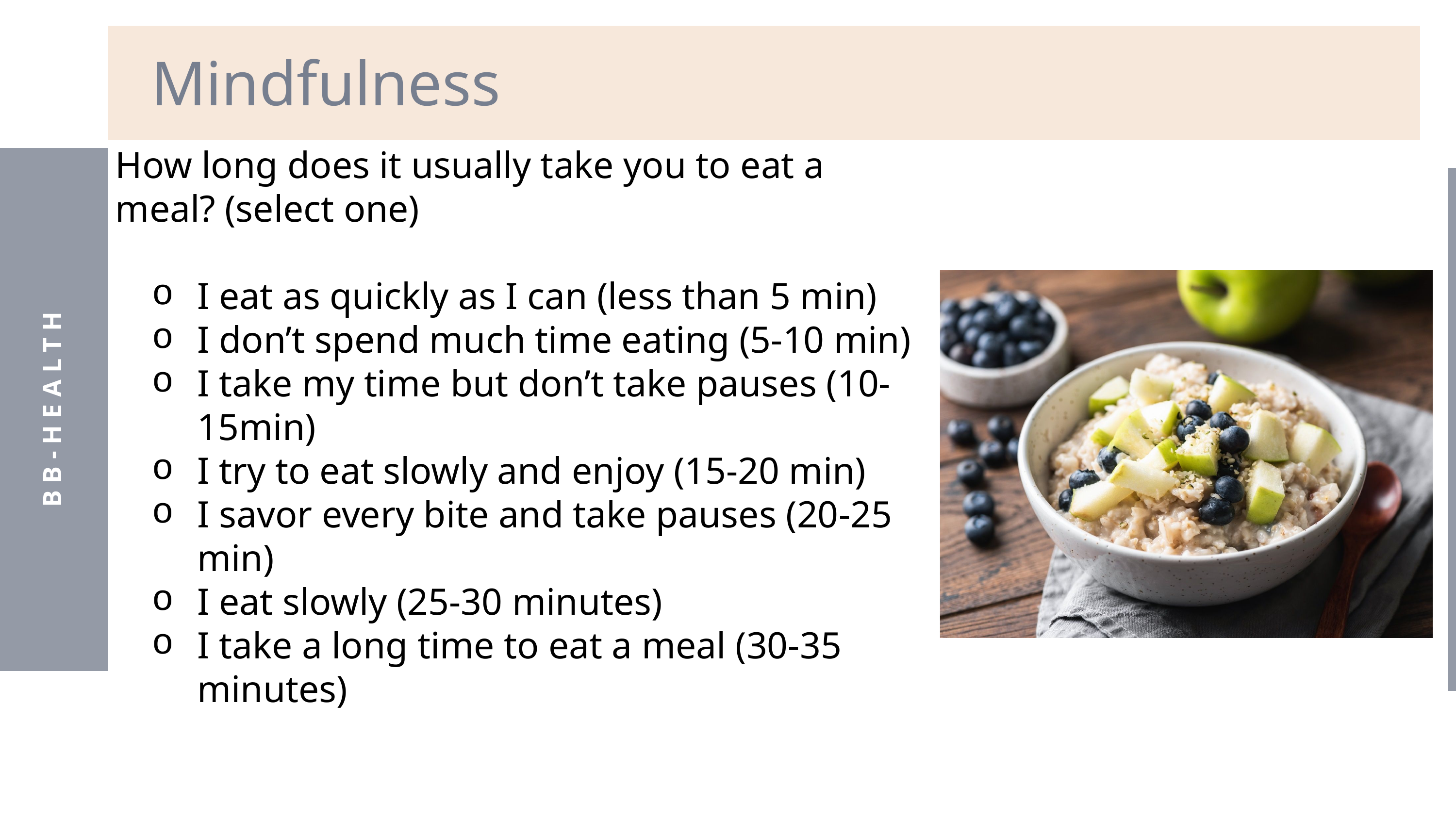

Mindfulness
How long does it usually take you to eat a meal? (select one)
I eat as quickly as I can (less than 5 min)
I don’t spend much time eating (5-10 min)
I take my time but don’t take pauses (10-15min)
I try to eat slowly and enjoy (15-20 min)
I savor every bite and take pauses (20-25 min)
I eat slowly (25-30 minutes)
I take a long time to eat a meal (30-35 minutes)
BB-HEALTH

## Slide 9
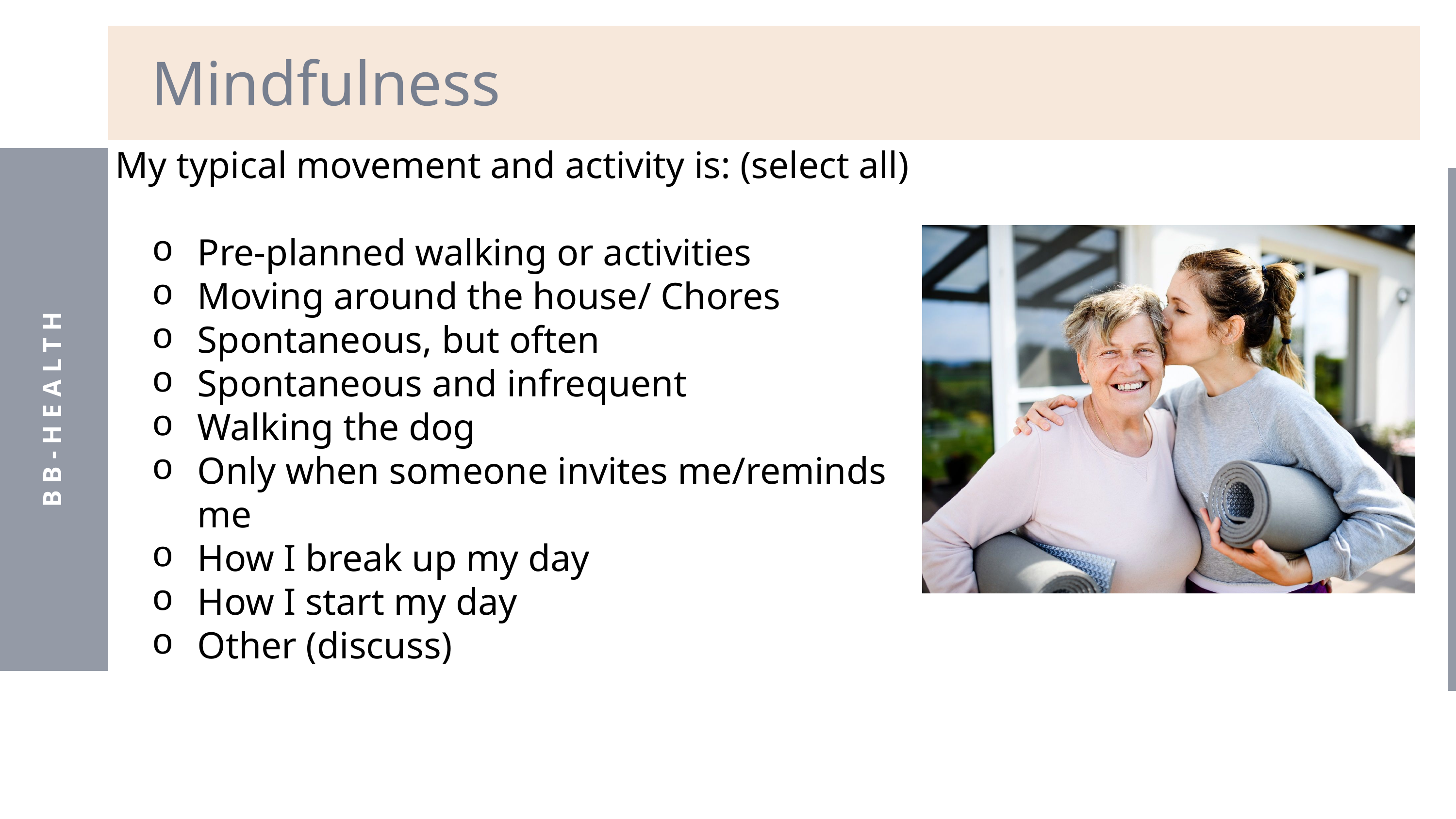

Mindfulness
My typical movement and activity is: (select all)
Pre-planned walking or activities
Moving around the house/ Chores
Spontaneous, but often
Spontaneous and infrequent
Walking the dog
Only when someone invites me/reminds me
How I break up my day
How I start my day
Other (discuss)
BB-HEALTH

## Slide 10
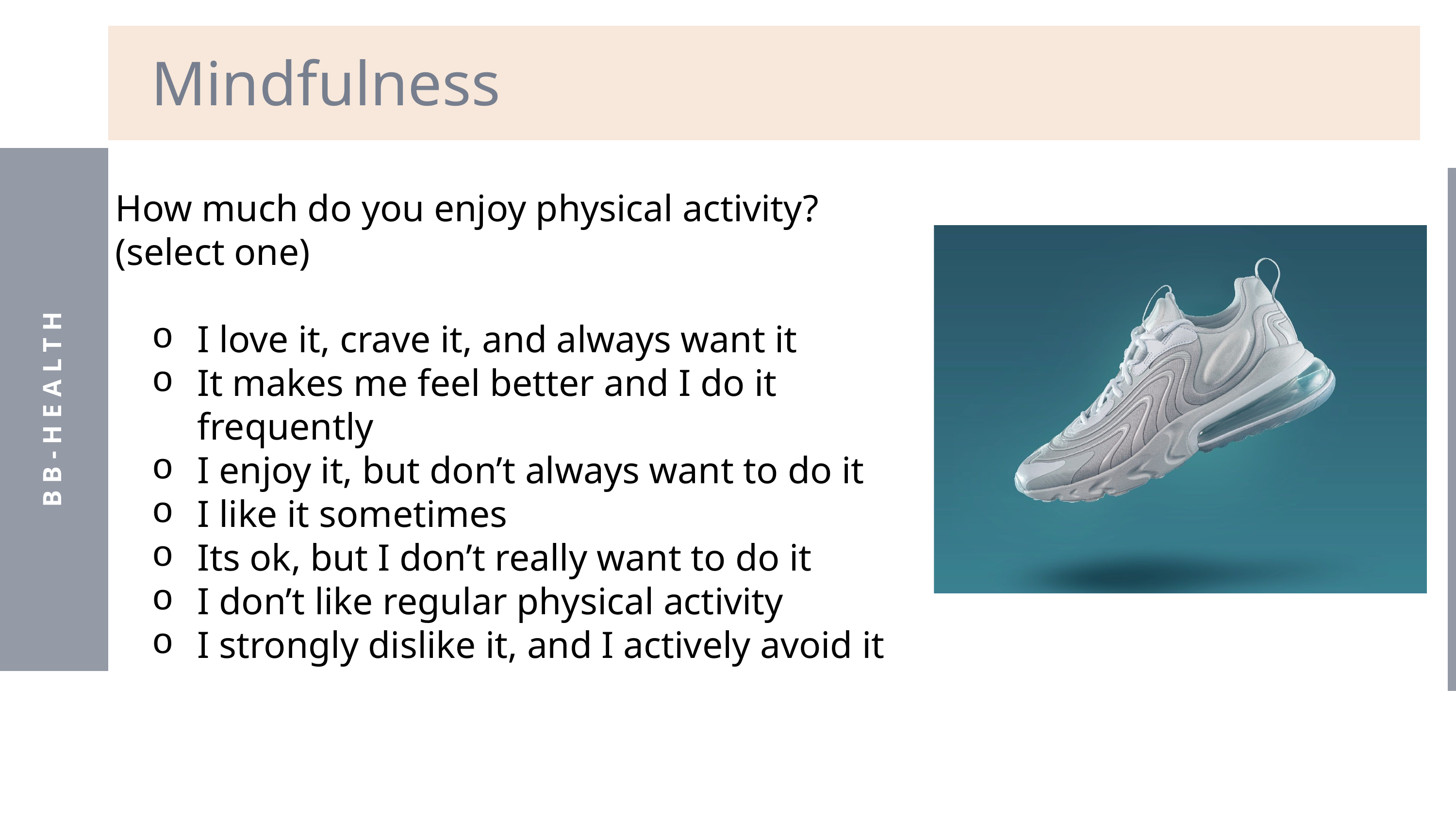

Mindfulness
How much do you enjoy physical activity? (select one)
I love it, crave it, and always want it
It makes me feel better and I do it frequently
I enjoy it, but don’t always want to do it
I like it sometimes
Its ok, but I don’t really want to do it
I don’t like regular physical activity
I strongly dislike it, and I actively avoid it
BB-HEALTH

## Slide 11
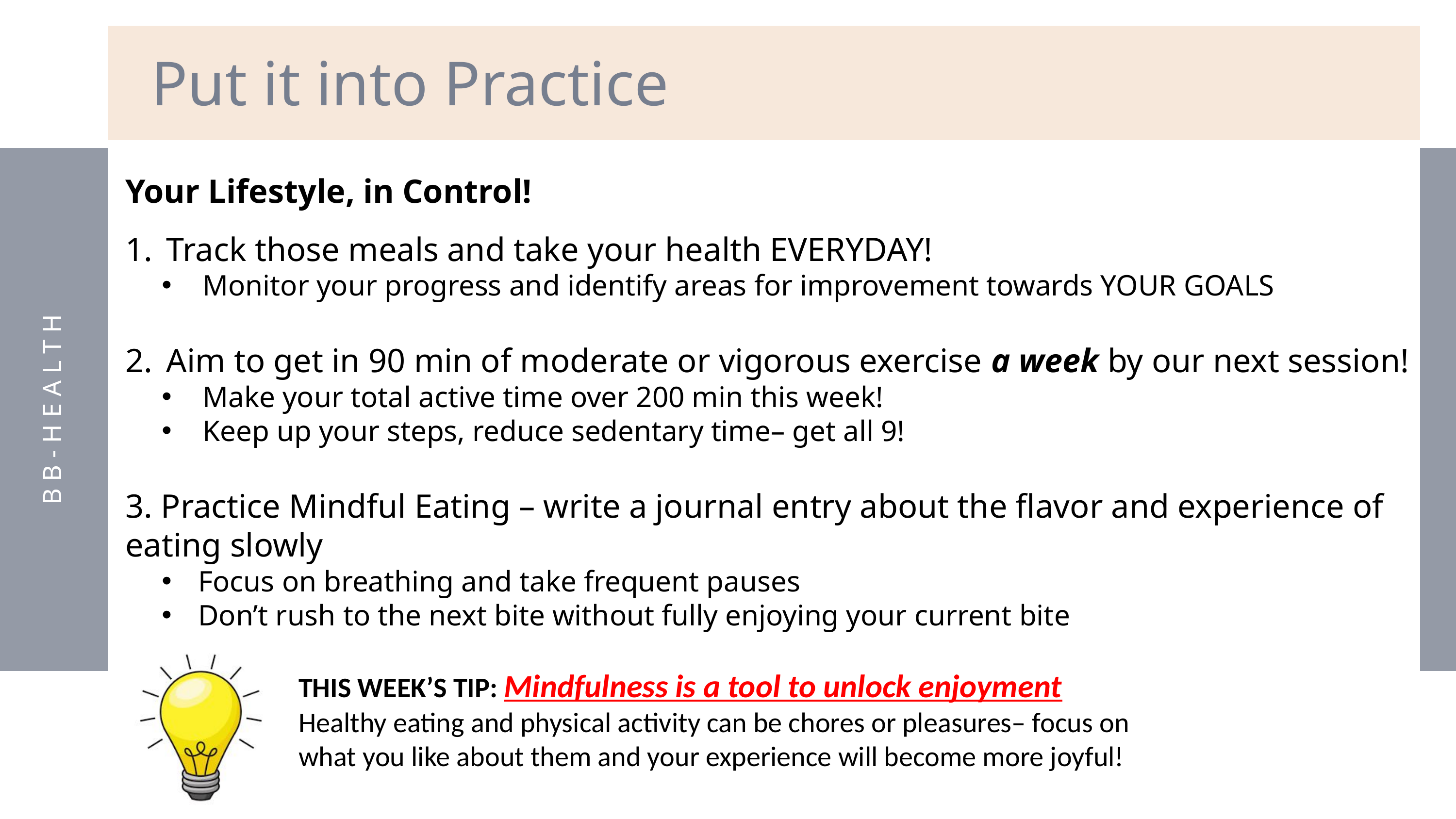

Put it into Practice
Your Lifestyle, in Control!
Track those meals and take your health EVERYDAY!
Monitor your progress and identify areas for improvement towards YOUR GOALS
Aim to get in 90 min of moderate or vigorous exercise a week by our next session!
Make your total active time over 200 min this week!
Keep up your steps, reduce sedentary time– get all 9!
3. Practice Mindful Eating – write a journal entry about the flavor and experience of eating slowly
Focus on breathing and take frequent pauses
Don’t rush to the next bite without fully enjoying your current bite
BB-HEALTH
THIS WEEK’S TIP: Mindfulness is a tool to unlock enjoyment
Healthy eating and physical activity can be chores or pleasures– focus on what you like about them and your experience will become more joyful!

## Slide 12
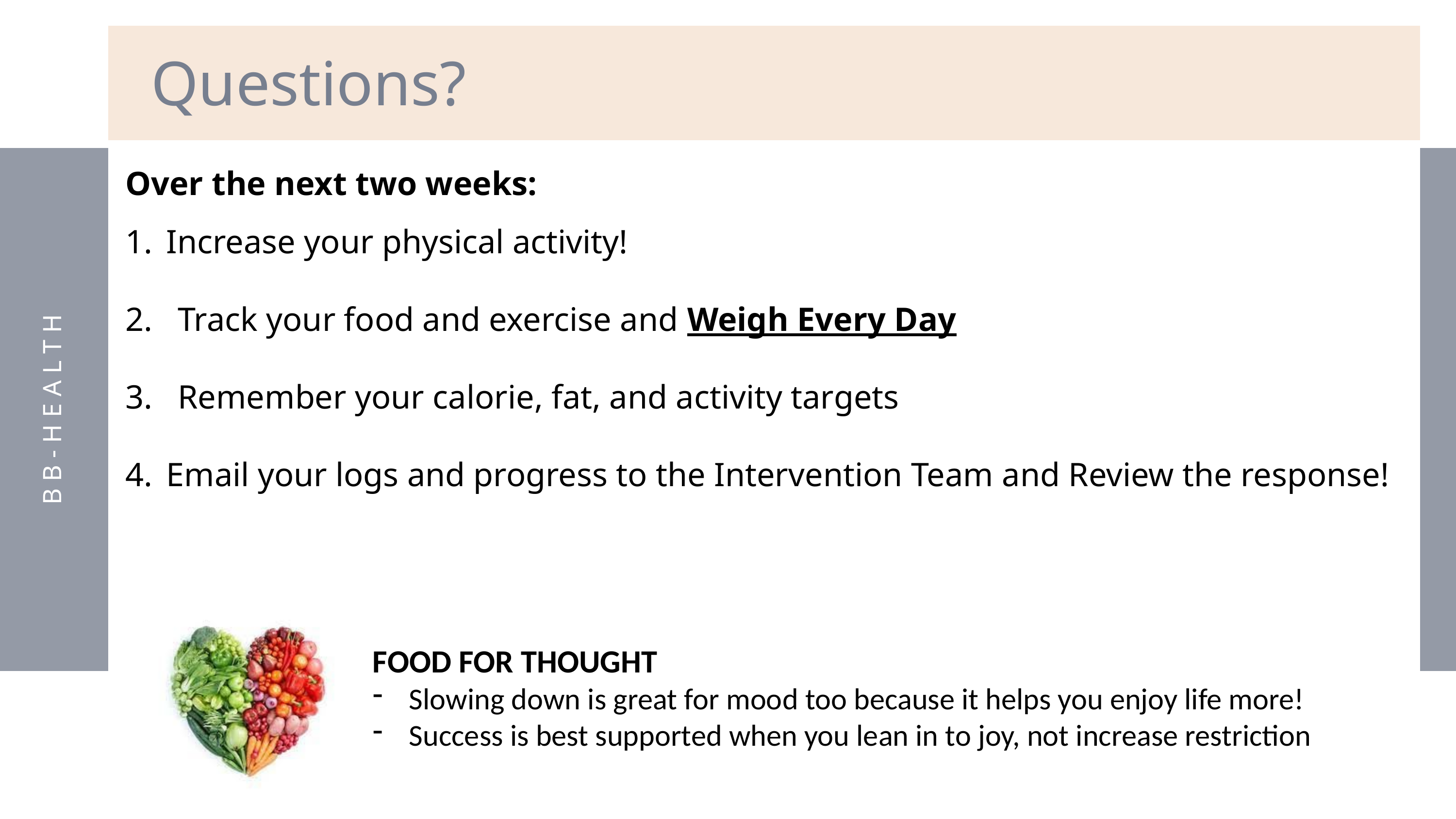

Questions?
Over the next two weeks:
Increase your physical activity!
2. Track your food and exercise and Weigh Every Day
3. Remember your calorie, fat, and activity targets
Email your logs and progress to the Intervention Team and Review the response!
BB-HEALTH
FOOD FOR THOUGHT
Slowing down is great for mood too because it helps you enjoy life more!
Success is best supported when you lean in to joy, not increase restriction
