# Supplementary material for: Development and Feasibility of an eHealth Diabetes Prevention Program Adapted for Older Adults—Results from a Randomized Control Pilot Study
Source: Nutrients. 2024 Mar 23;16(7):930. doi: 10.3390/nu16070930 (PMC11154527; doi:10.3390/nu16070930)
Supplement: Supplementary file 1 [file nutrients-16-00930-s001.zip › Session16.pptx]

## Slide 1
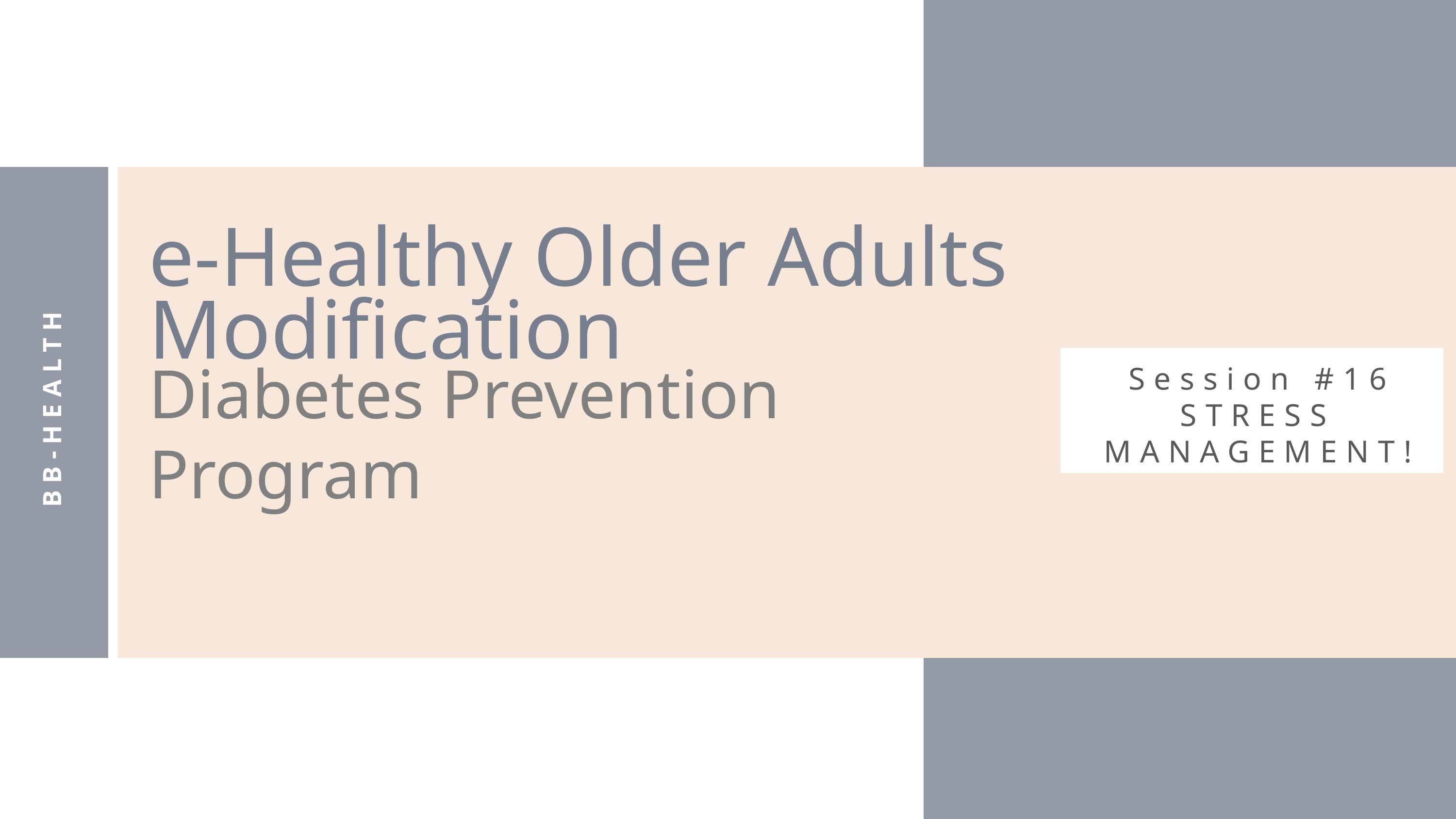

OPEN REPORTS
e-Healthy Older Adults Modification
Session #16
STRESS MANAGEMENT!
Diabetes Prevention Program
BB-HEALTH

## Slide 2
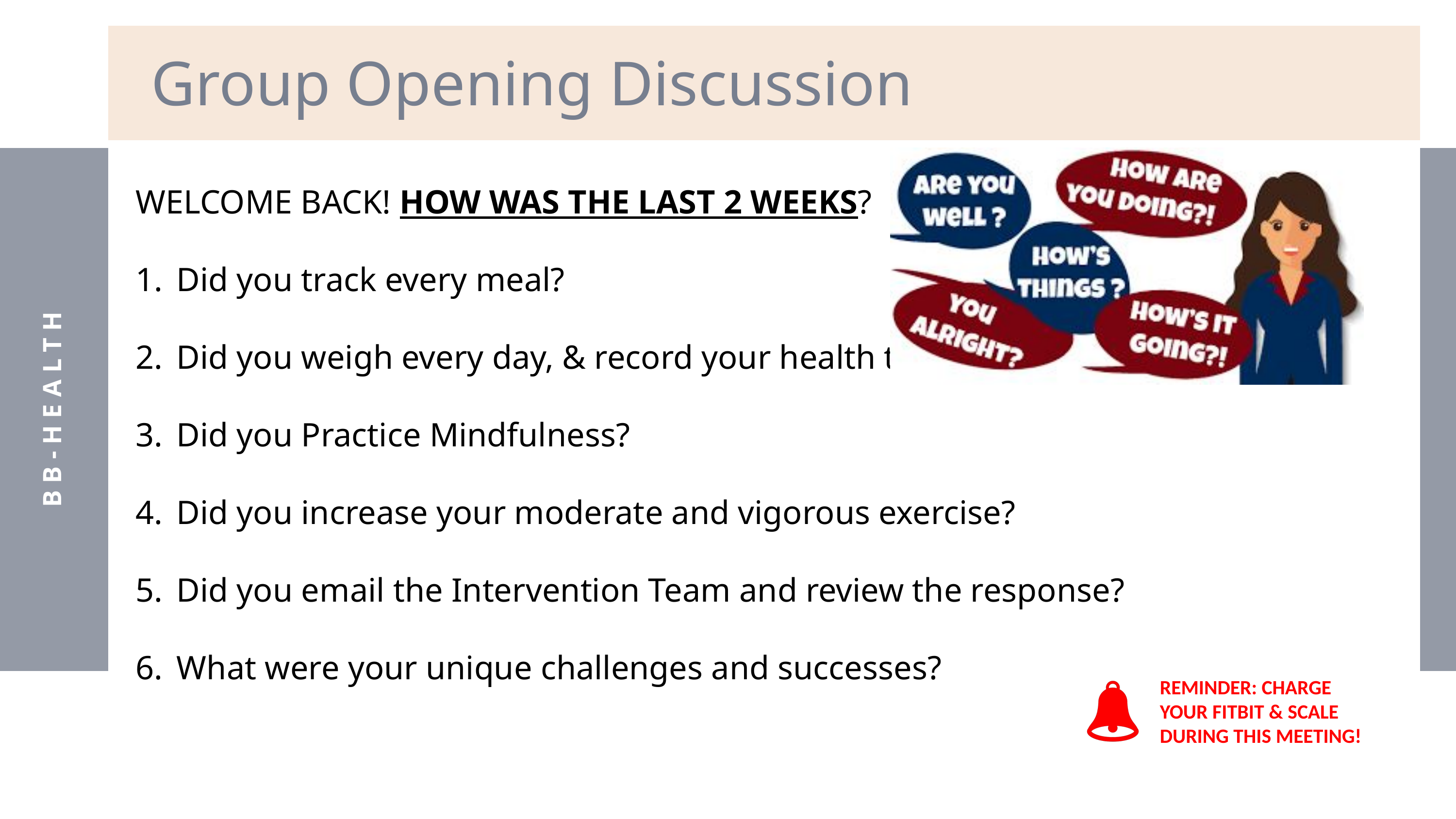

Group Opening Discussion
WELCOME BACK! HOW WAS THE LAST 2 WEEKS?
Did you track every meal?
Did you weigh every day, & record your health today?
Did you Practice Mindfulness?
Did you increase your moderate and vigorous exercise?
Did you email the Intervention Team and review the response?
What were your unique challenges and successes?
BB-HEALTH
REMINDER: CHARGE YOUR FITBIT & SCALE DURING THIS MEETING!

## Slide 3
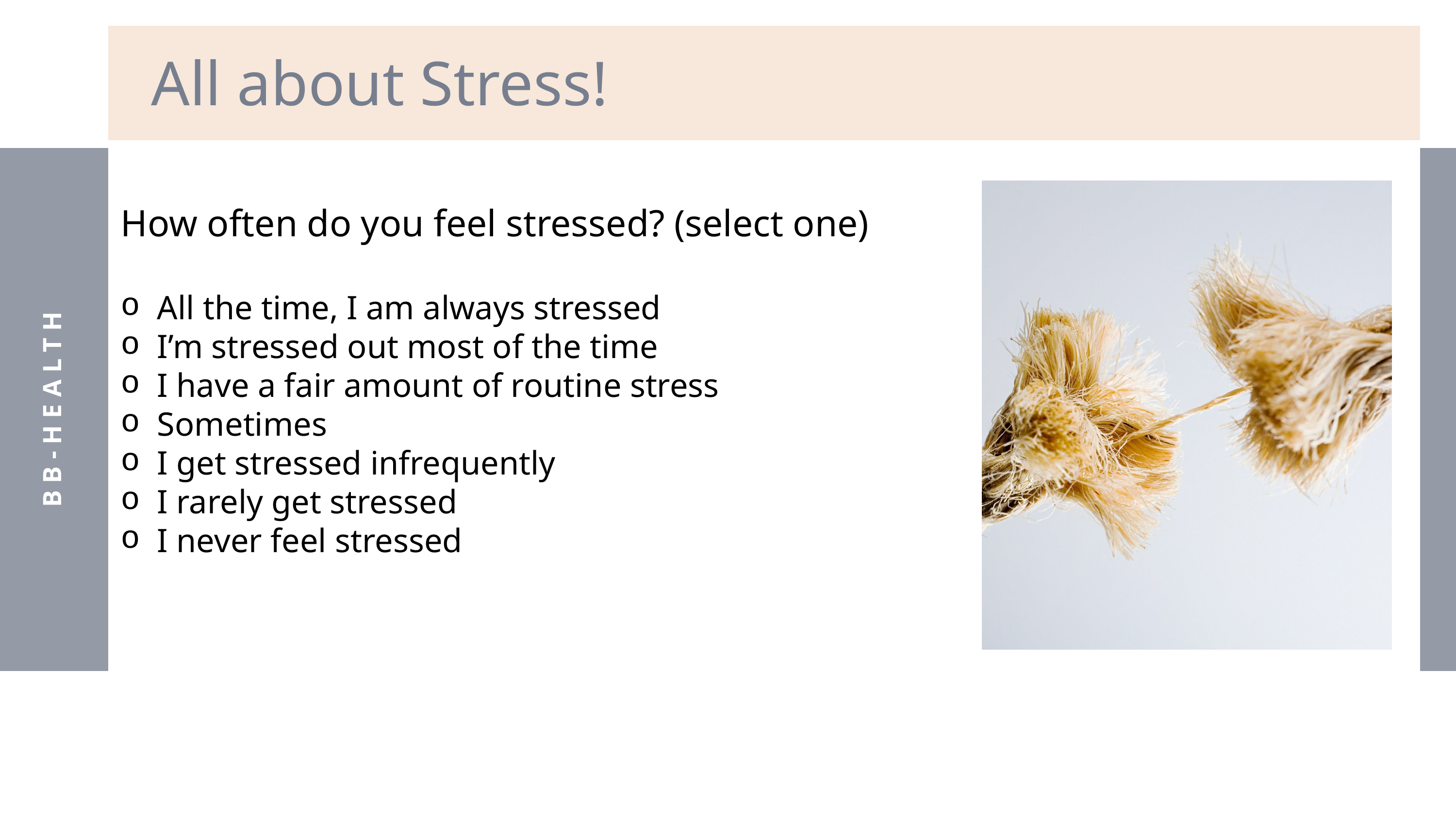

All about Stress!
How often do you feel stressed? (select one)
All the time, I am always stressed
I’m stressed out most of the time
I have a fair amount of routine stress
Sometimes
I get stressed infrequently
I rarely get stressed
I never feel stressed
BB-HEALTH

## Slide 4
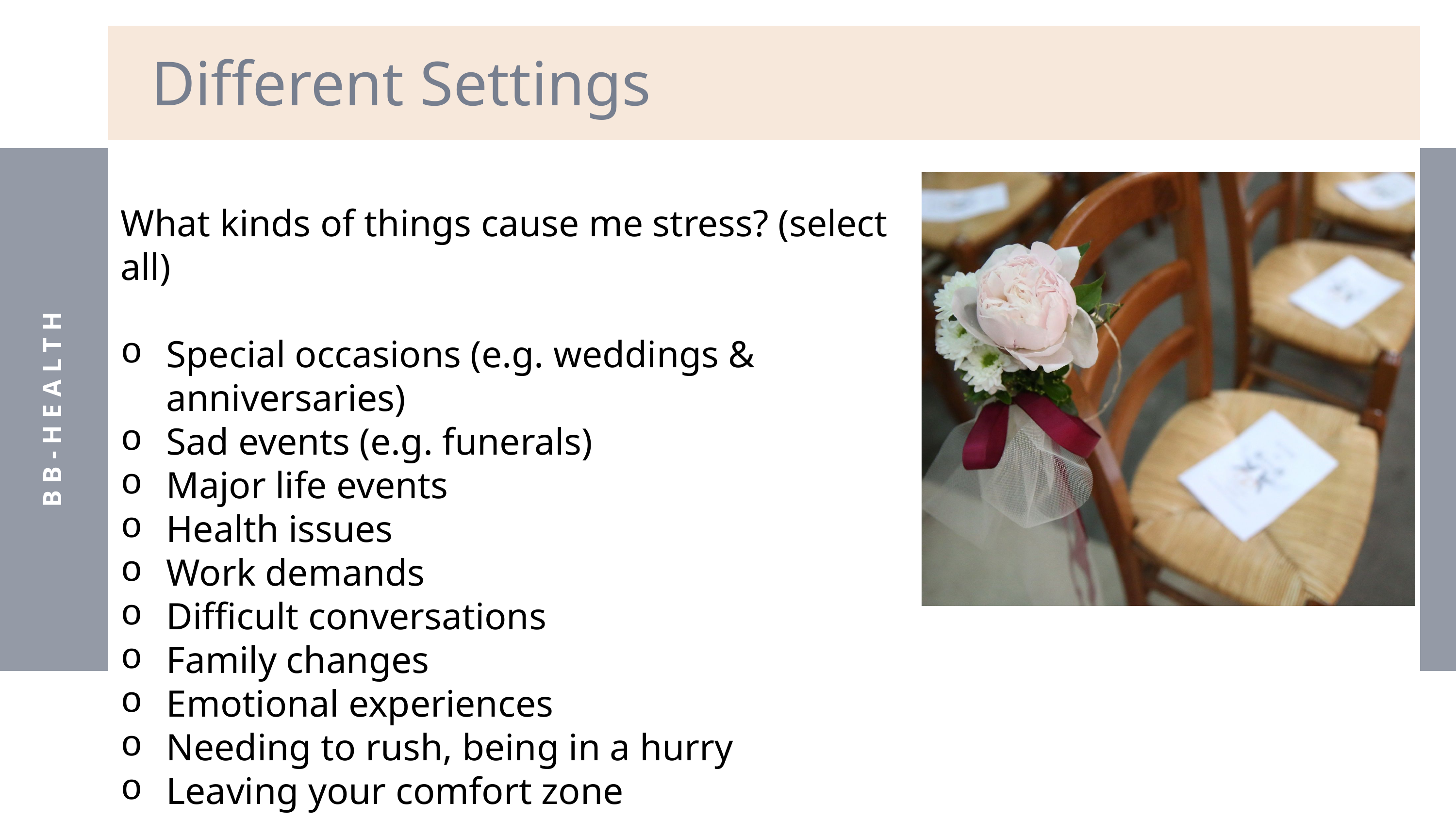

Different Settings
What kinds of things cause me stress? (select all)
Special occasions (e.g. weddings & anniversaries)
Sad events (e.g. funerals)
Major life events
Health issues
Work demands
Difficult conversations
Family changes
Emotional experiences
Needing to rush, being in a hurry
Leaving your comfort zone
Other (discuss)
BB-HEALTH

## Slide 5
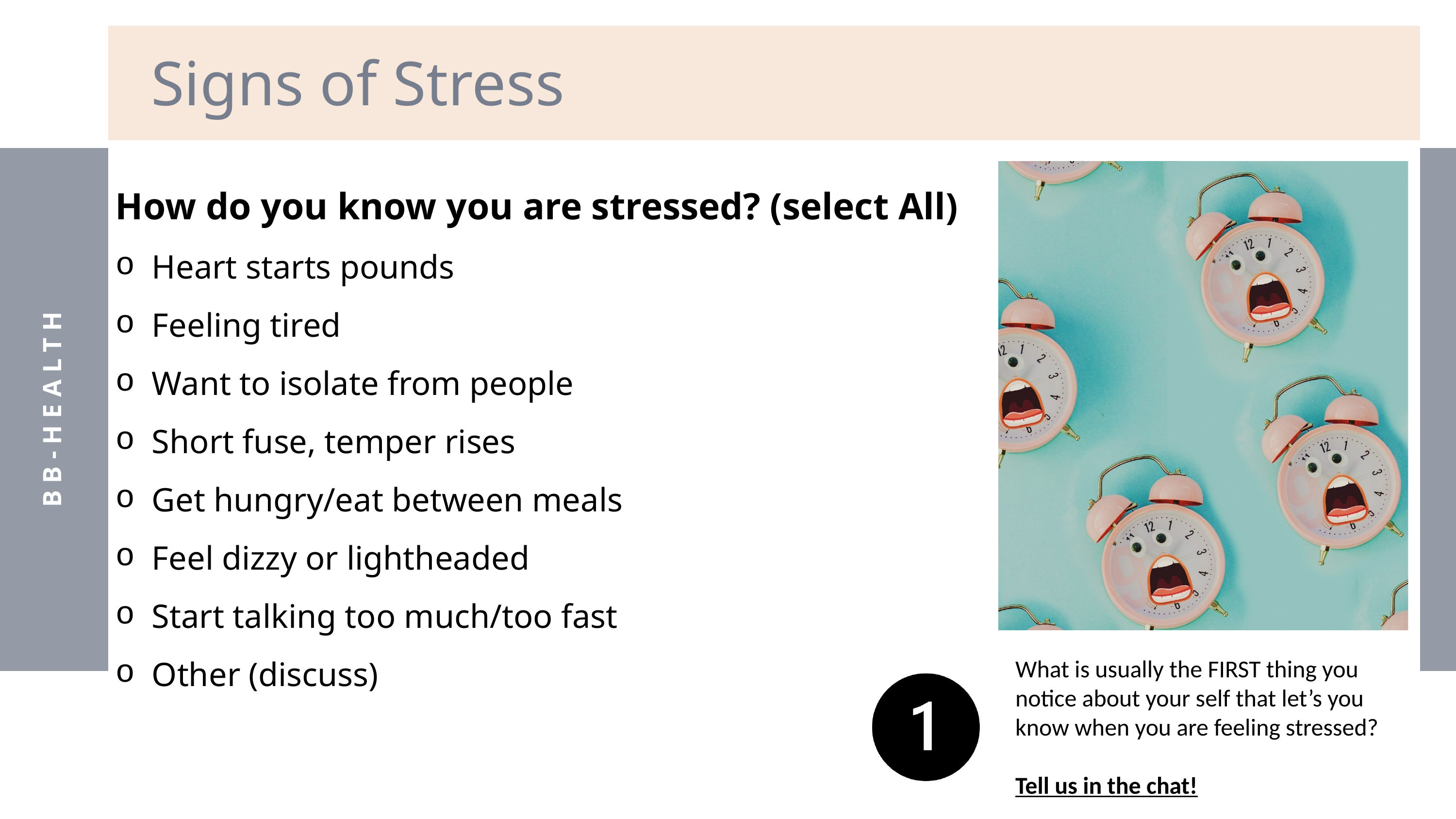

Signs of Stress
How do you know you are stressed? (select All)
Heart starts pounds
Feeling tired
Want to isolate from people
Short fuse, temper rises
Get hungry/eat between meals
Feel dizzy or lightheaded
Start talking too much/too fast
Other (discuss)
BB-HEALTH
What is usually the FIRST thing you notice about your self that let’s you know when you are feeling stressed?
Tell us in the chat!

## Slide 6
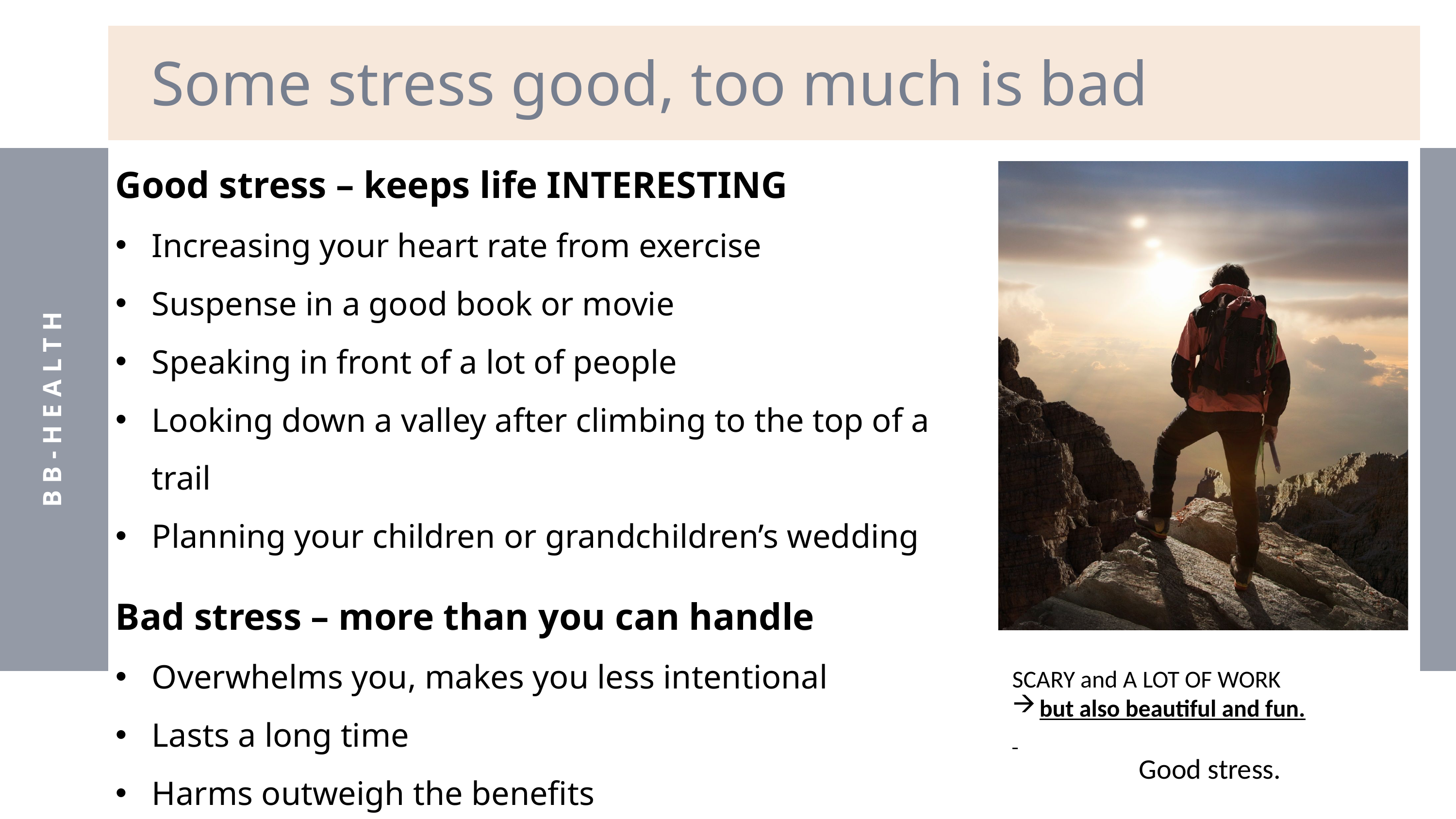

Some stress good, too much is bad
Good stress – keeps life INTERESTING
Increasing your heart rate from exercise
Suspense in a good book or movie
Speaking in front of a lot of people
Looking down a valley after climbing to the top of a trail
Planning your children or grandchildren’s wedding
Bad stress – more than you can handle
Overwhelms you, makes you less intentional
Lasts a long time
Harms outweigh the benefits
BB-HEALTH
SCARY and A LOT OF WORK
but also beautiful and fun.
Good stress.

## Slide 7
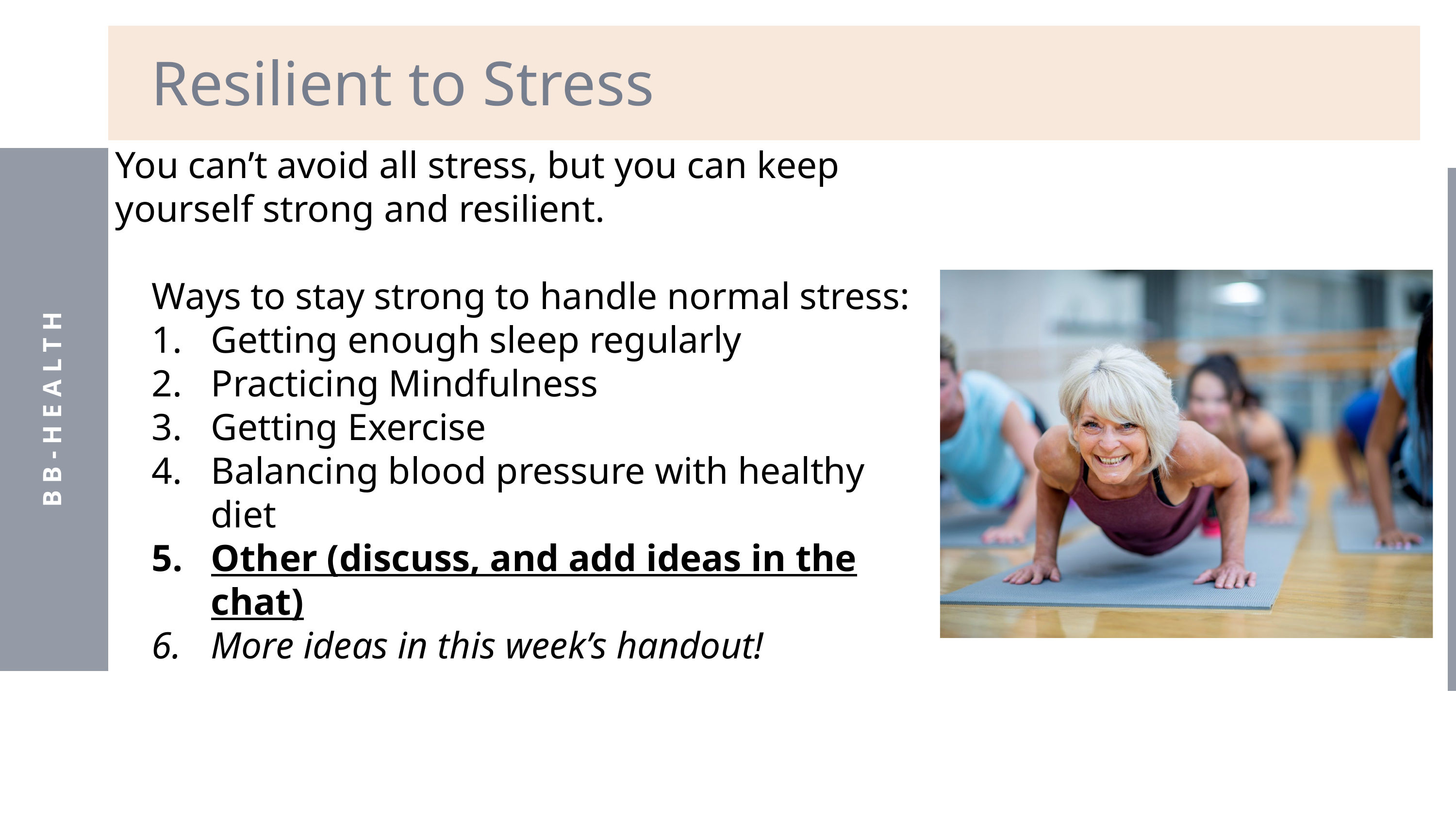

Resilient to Stress
You can’t avoid all stress, but you can keep yourself strong and resilient.
Ways to stay strong to handle normal stress:
Getting enough sleep regularly
Practicing Mindfulness
Getting Exercise
Balancing blood pressure with healthy diet
Other (discuss, and add ideas in the chat)
More ideas in this week’s handout!
BB-HEALTH

## Slide 8
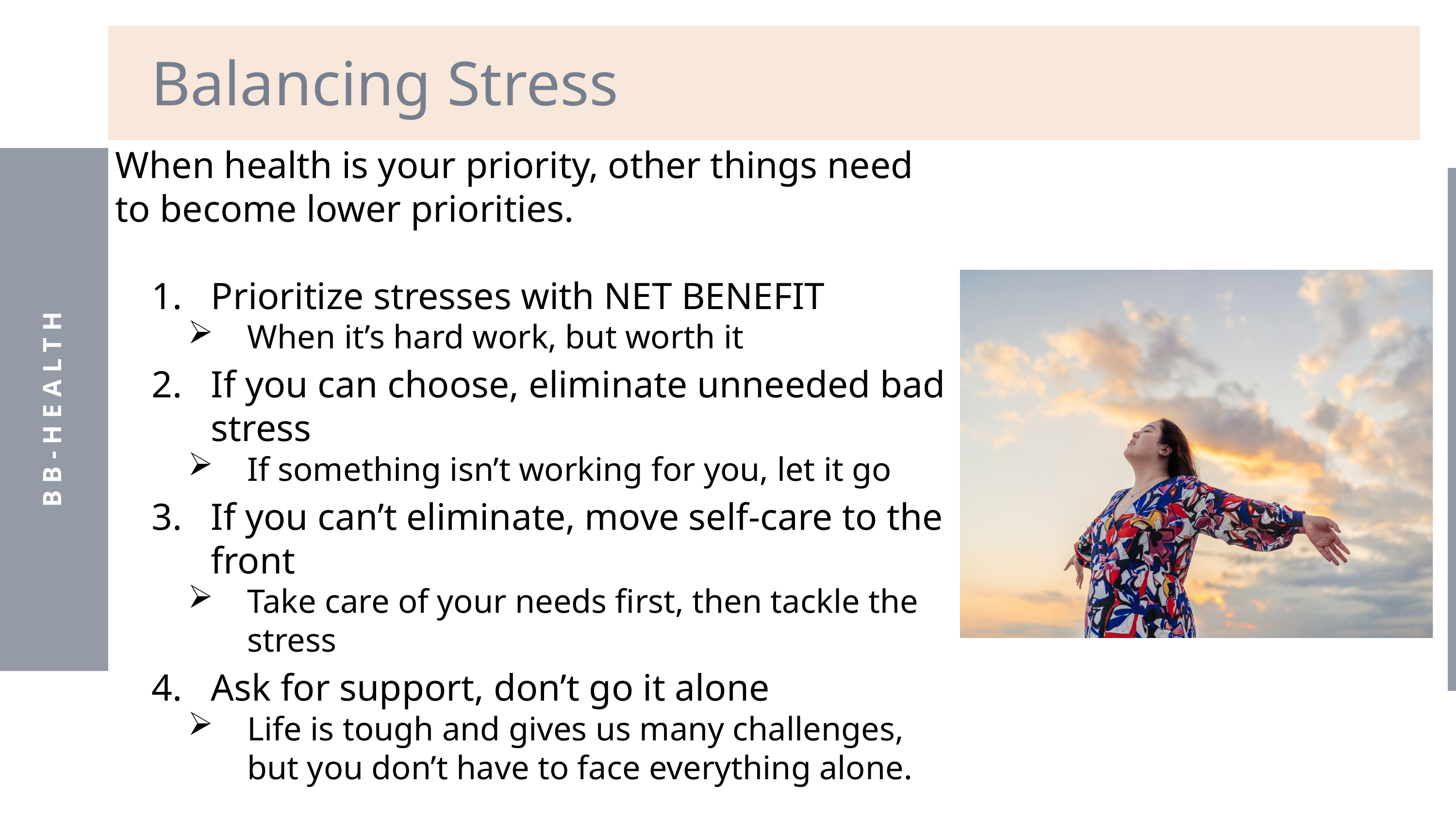

Balancing Stress
When health is your priority, other things need to become lower priorities.
Prioritize stresses with NET BENEFIT
When it’s hard work, but worth it
If you can choose, eliminate unneeded bad stress
If something isn’t working for you, let it go
If you can’t eliminate, move self-care to the front
Take care of your needs first, then tackle the stress
Ask for support, don’t go it alone
Life is tough and gives us many challenges, but you don’t have to face everything alone.
BB-HEALTH

## Slide 9
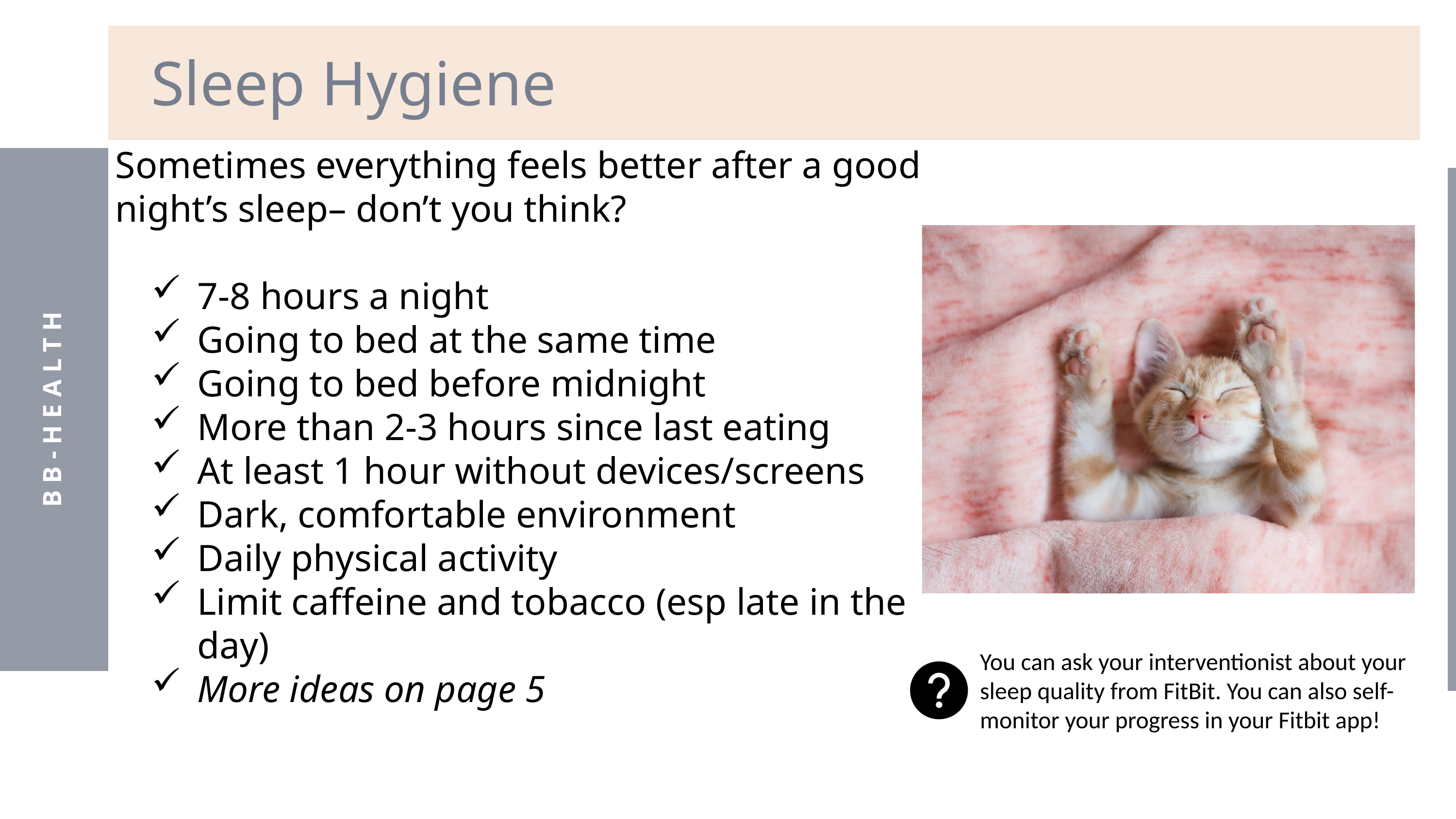

Sleep Hygiene
Sometimes everything feels better after a good night’s sleep– don’t you think?
7-8 hours a night
Going to bed at the same time
Going to bed before midnight
More than 2-3 hours since last eating
At least 1 hour without devices/screens
Dark, comfortable environment
Daily physical activity
Limit caffeine and tobacco (esp late in the day)
More ideas on page 5
BB-HEALTH
You can ask your interventionist about your sleep quality from FitBit. You can also self-monitor your progress in your Fitbit app!

## Slide 10
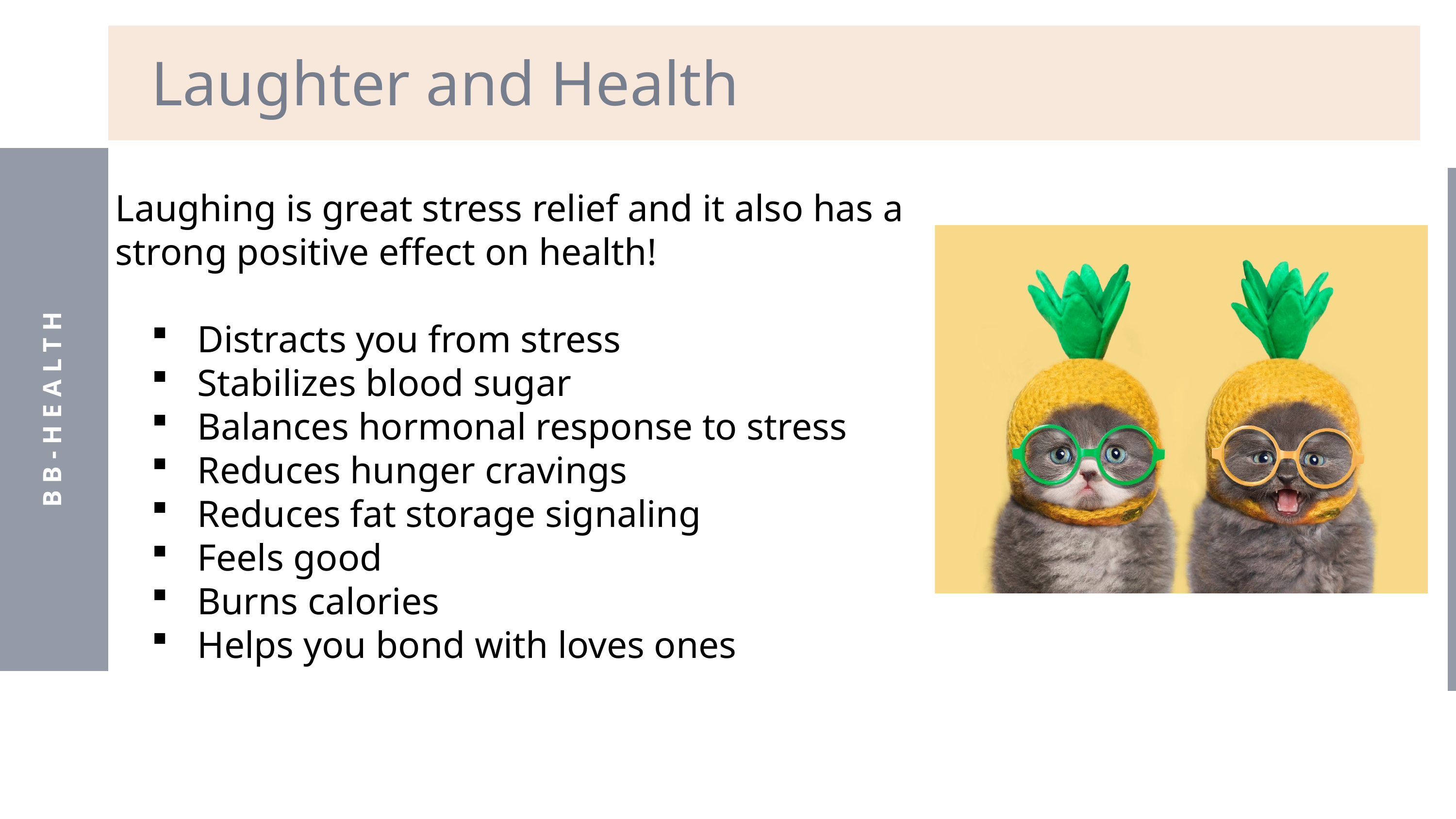

Laughter and Health
Laughing is great stress relief and it also has a strong positive effect on health!
Distracts you from stress
Stabilizes blood sugar
Balances hormonal response to stress
Reduces hunger cravings
Reduces fat storage signaling
Feels good
Burns calories
Helps you bond with loves ones
BB-HEALTH

## Slide 11
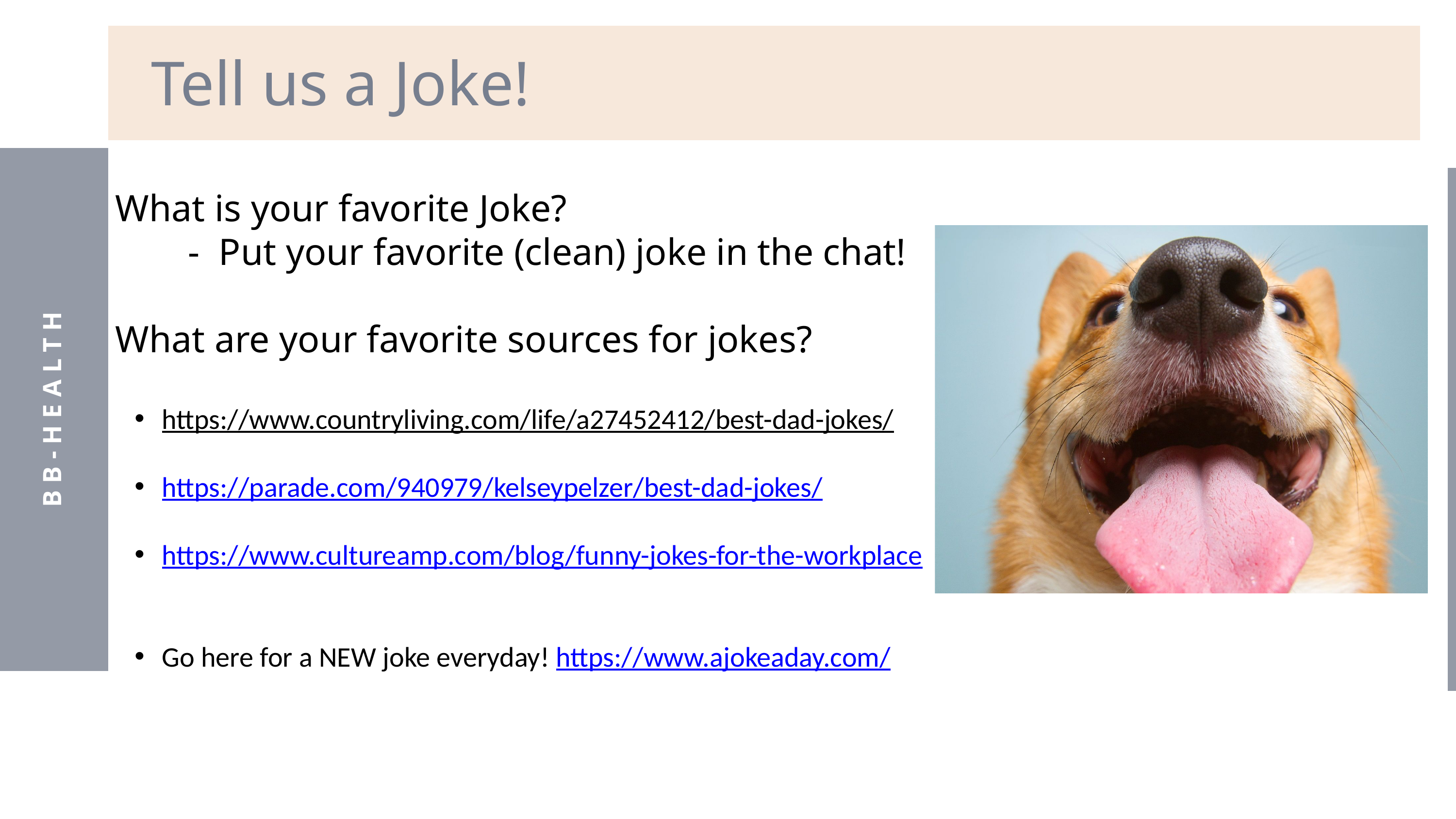

Tell us a Joke!
What is your favorite Joke?
	- Put your favorite (clean) joke in the chat!
What are your favorite sources for jokes?
BB-HEALTH
https://www.countryliving.com/life/a27452412/best-dad-jokes/
https://parade.com/940979/kelseypelzer/best-dad-jokes/
https://www.cultureamp.com/blog/funny-jokes-for-the-workplace
Go here for a NEW joke everyday! https://www.ajokeaday.com/

## Slide 12
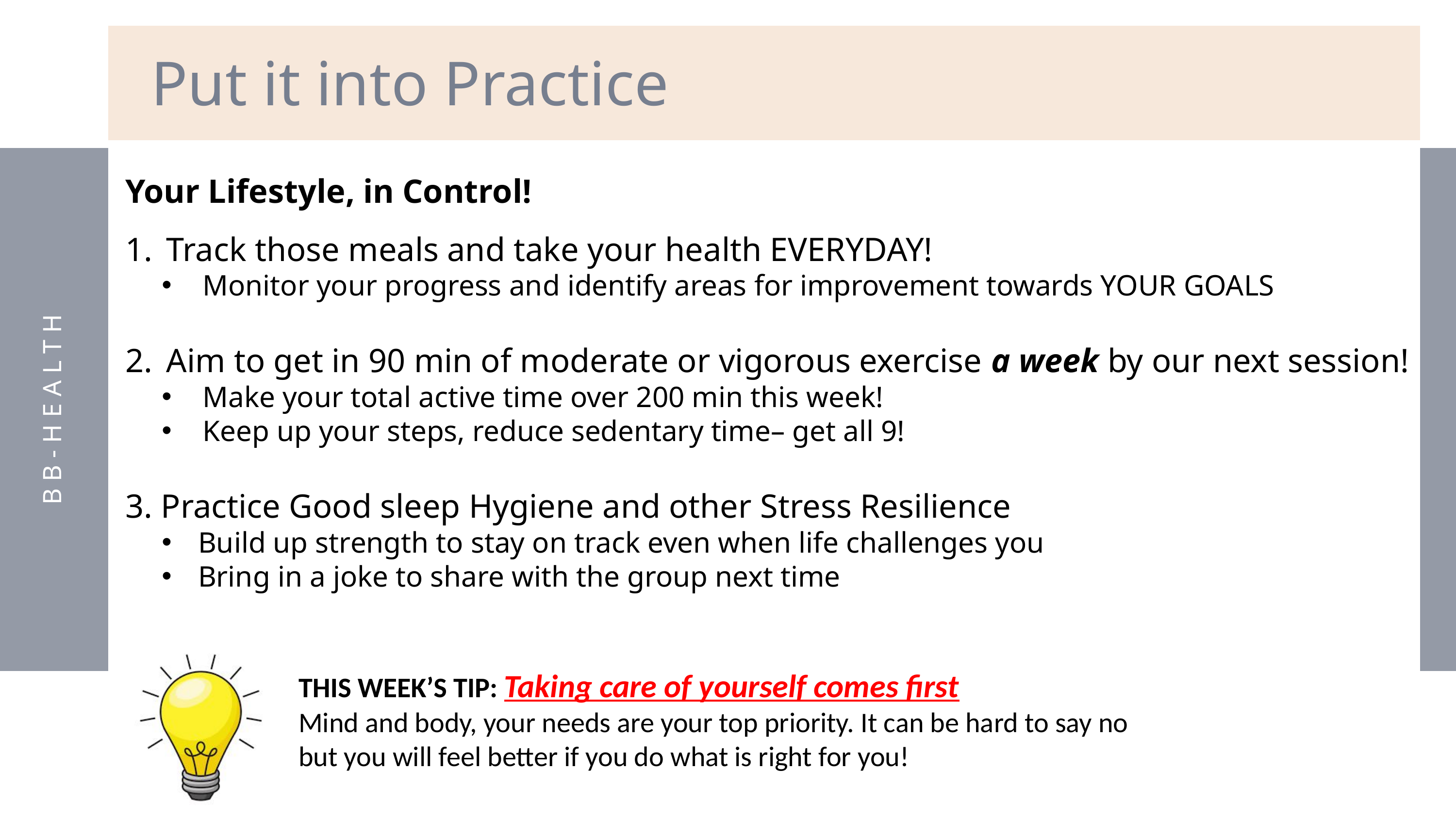

Put it into Practice
Your Lifestyle, in Control!
Track those meals and take your health EVERYDAY!
Monitor your progress and identify areas for improvement towards YOUR GOALS
Aim to get in 90 min of moderate or vigorous exercise a week by our next session!
Make your total active time over 200 min this week!
Keep up your steps, reduce sedentary time– get all 9!
3. Practice Good sleep Hygiene and other Stress Resilience
Build up strength to stay on track even when life challenges you
Bring in a joke to share with the group next time
BB-HEALTH
THIS WEEK’S TIP: Taking care of yourself comes first
Mind and body, your needs are your top priority. It can be hard to say no but you will feel better if you do what is right for you!

## Slide 13
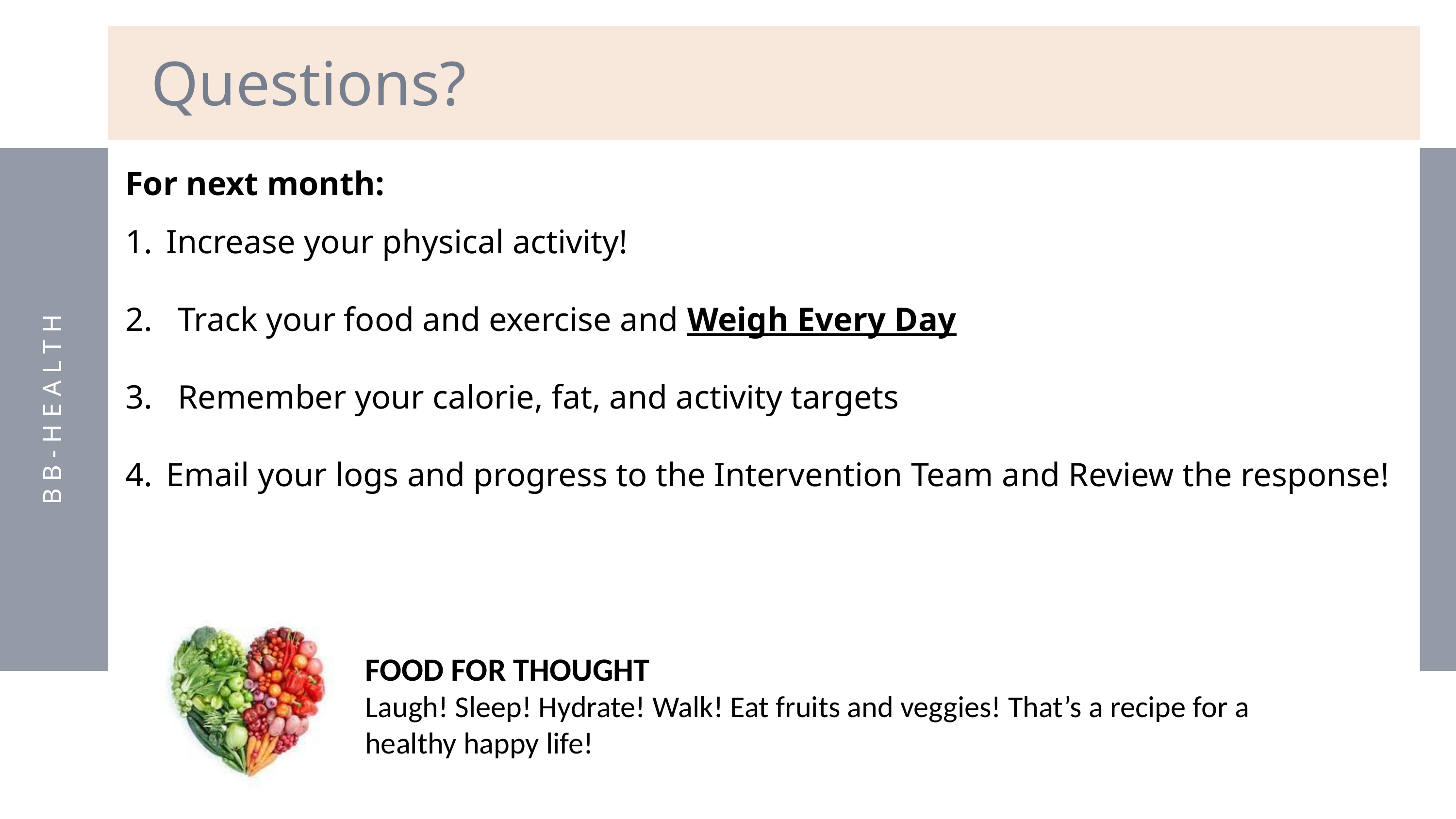

Questions?
For next month:
Increase your physical activity!
2. Track your food and exercise and Weigh Every Day
3. Remember your calorie, fat, and activity targets
Email your logs and progress to the Intervention Team and Review the response!
BB-HEALTH
FOOD FOR THOUGHT
Laugh! Sleep! Hydrate! Walk! Eat fruits and veggies! That’s a recipe for a healthy happy life!
