# Supplementary material for: Development and Feasibility of an eHealth Diabetes Prevention Program Adapted for Older Adults—Results from a Randomized Control Pilot Study
Source: Nutrients. 2024 Mar 23;16(7):930. doi: 10.3390/nu16070930 (PMC11154527; doi:10.3390/nu16070930)
Supplement: Supplementary file 1 [file nutrients-16-00930-s001.zip › Session16_supp.pdf]

BB-HEALTH

e-Healthy Older Adults Modification

Diabetes Prevention Program

Session #16
STRESS MANAGEMENT!

Group Opening Discussion

BB-HEALTH

WELCOME BACK! <u>**HOW WAS THE LAST 2 WEEKS**</u>?

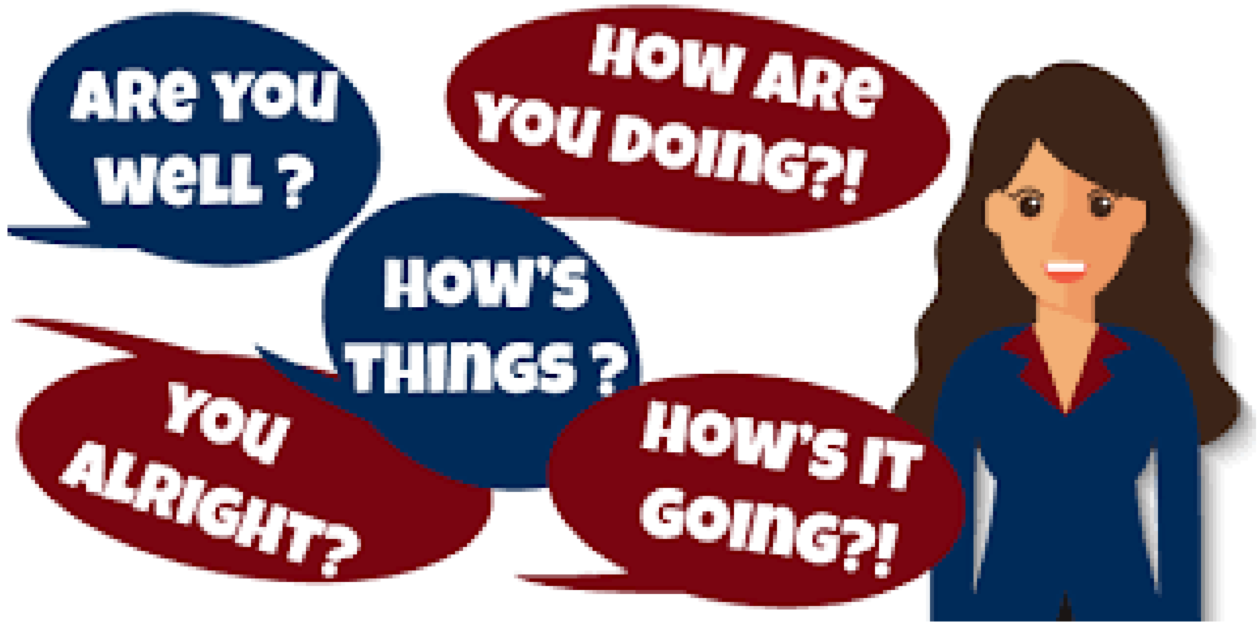

1. Did you track every meal?
2. Did you weigh every day, & record your health today?
3. Did you Practice Mindfulness?
4. Did you increase your moderate and vigorous exercise?
5. Did you email the Intervention Team and review the response?
6. What were your unique challenges and successes?

REMINDER: CHARGE YOUR FITBIT & SCALE DURING THIS MEETING!

All about Stress!

BB-HEALTH

How often do you feel stressed? (select one)

- All the time, I am always stressed
- I'm stressed out most of the time
- I have a fair amount of routine stress
- Sometimes
- I get stressed infrequently
- I rarely get stressed
- I never feel stressed

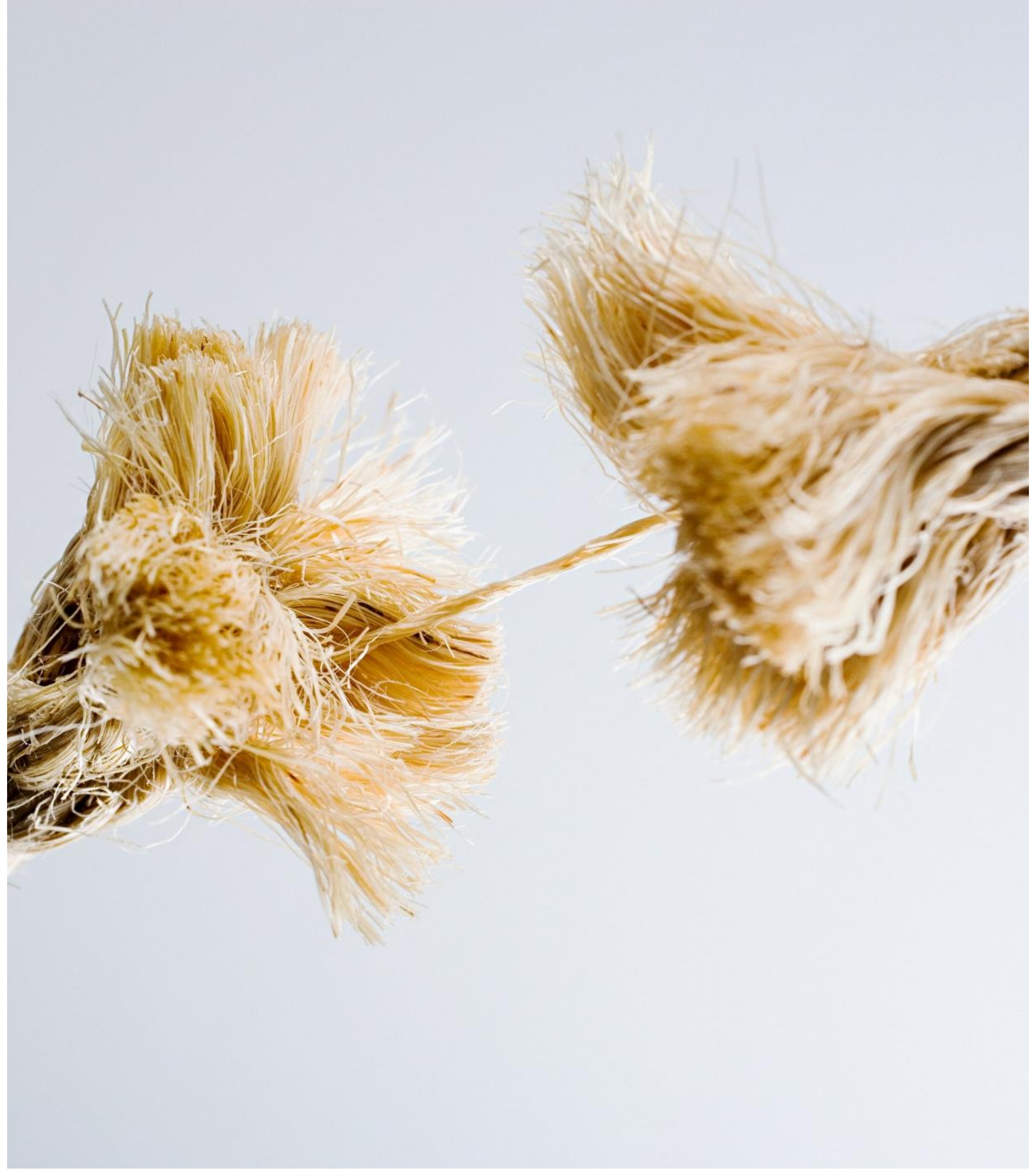

Different Settings

BB-HEALTH

What kinds of things cause me stress? (select all)

- Special occasions (e.g. weddings & anniversaries)
- Sad events (e.g. funerals)
- Major life events
- Health issues
- Work demands
- Difficult conversations
- Family changes
- Emotional experiences
- Needing to rush, being in a hurry
- Leaving your comfort zone
- Other (discuss)

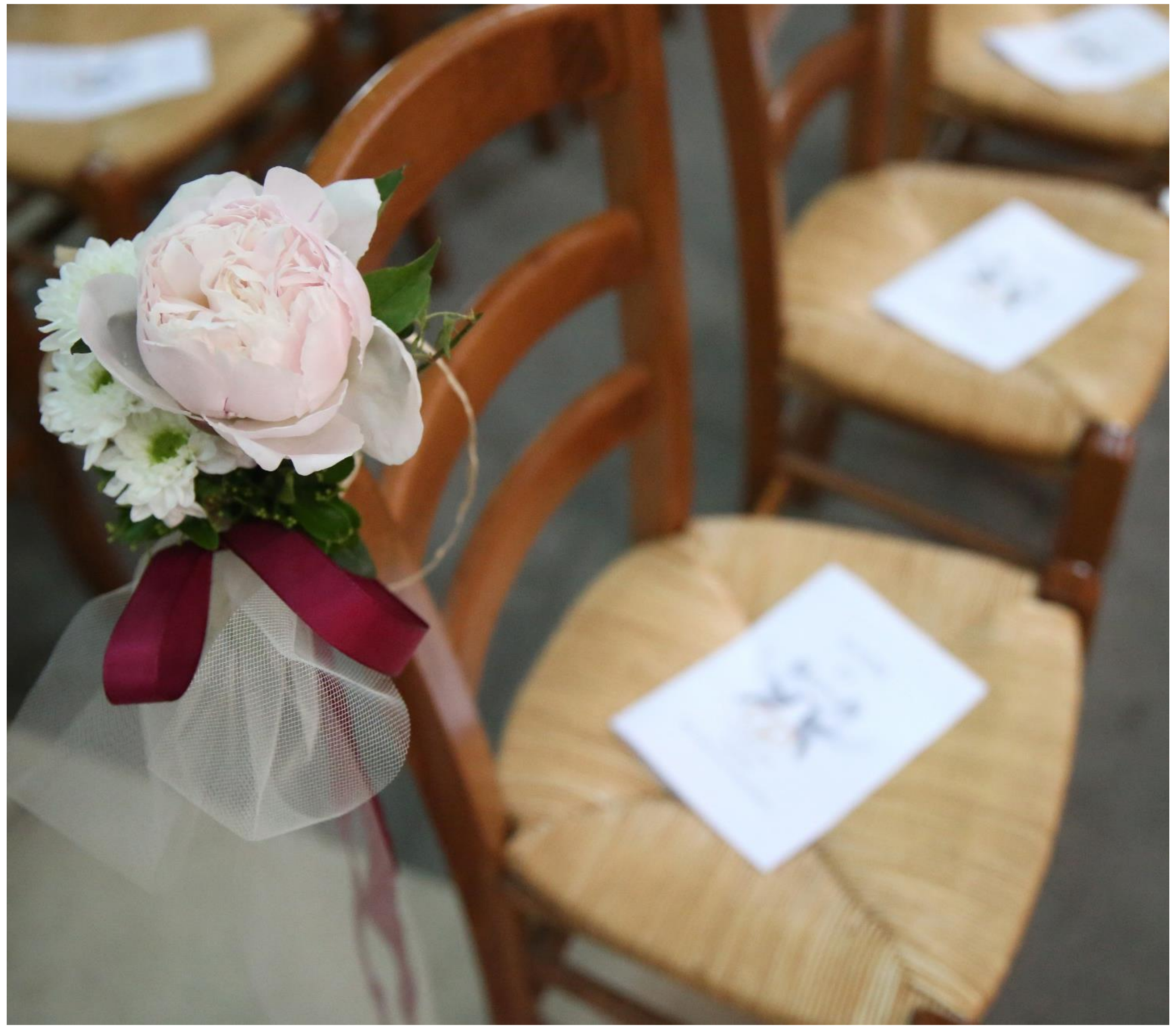

Signs of Stress

B B - H E A L T H

How do you know you are stressed? (select All)

- Heart starts pounds
- Feeling tired
- Want to isolate from people
- Short fuse, temper rises
- Get hungry/eat between meals
- Feel dizzy or lightheaded
- Start talking too much/too fast
- Other (discuss)

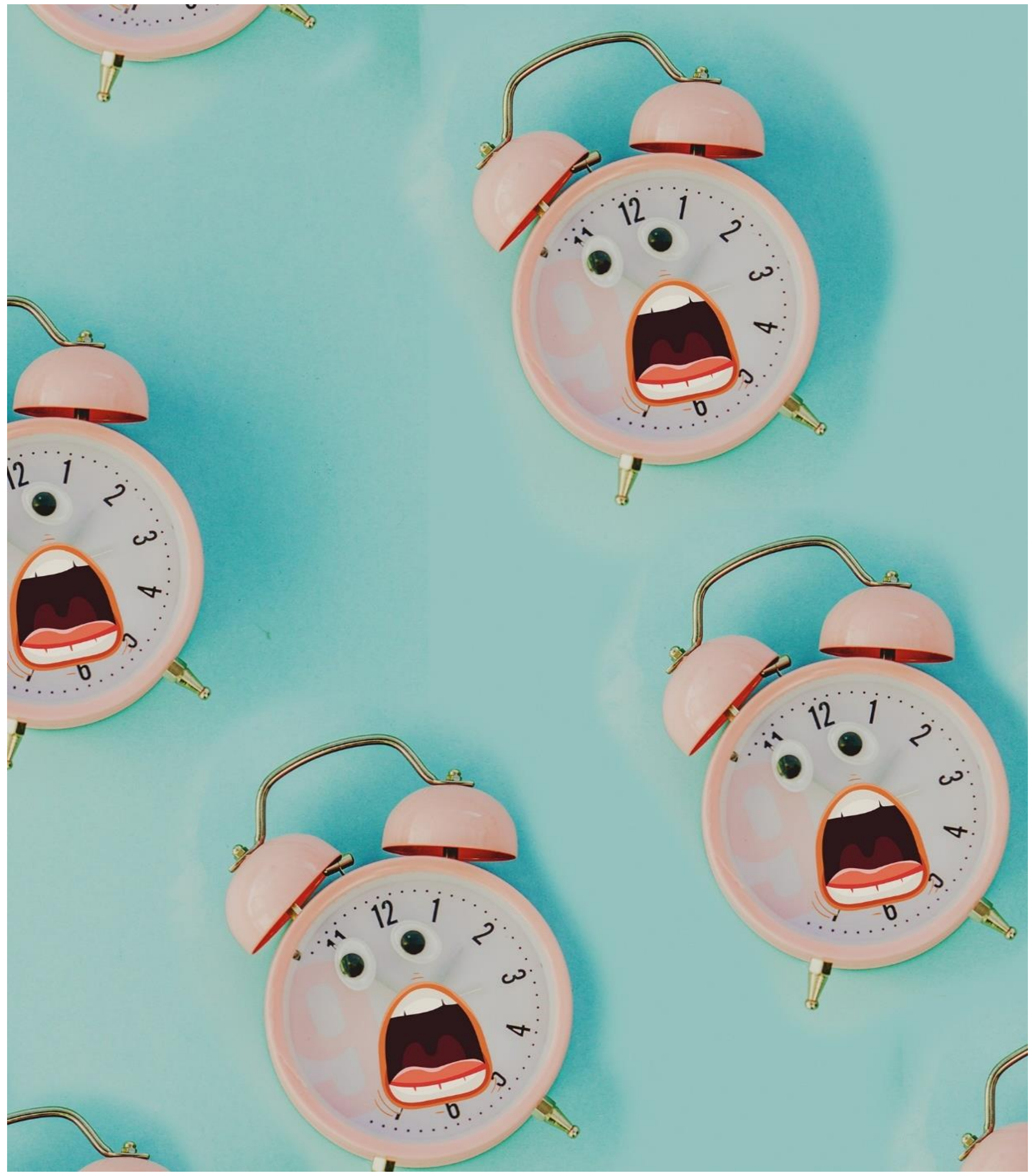

1

What is usually the FIRST thing you notice about your self that let's you know when you are feeling stressed?

Tell us in the chat!

Some stress good, too much is bad

Good stress – keeps life INTERESTING

- Increasing your heart rate from exercise
- Suspense in a good book or movie
- Speaking in front of a lot of people
- Looking down a valley after climbing to the top of a trail
- Planning your children or grandchildren’s wedding

Bad stress – more than you can handle

- Overwhelms you, makes you less intentional
- Lasts a long time
- Harms outweigh the benefits

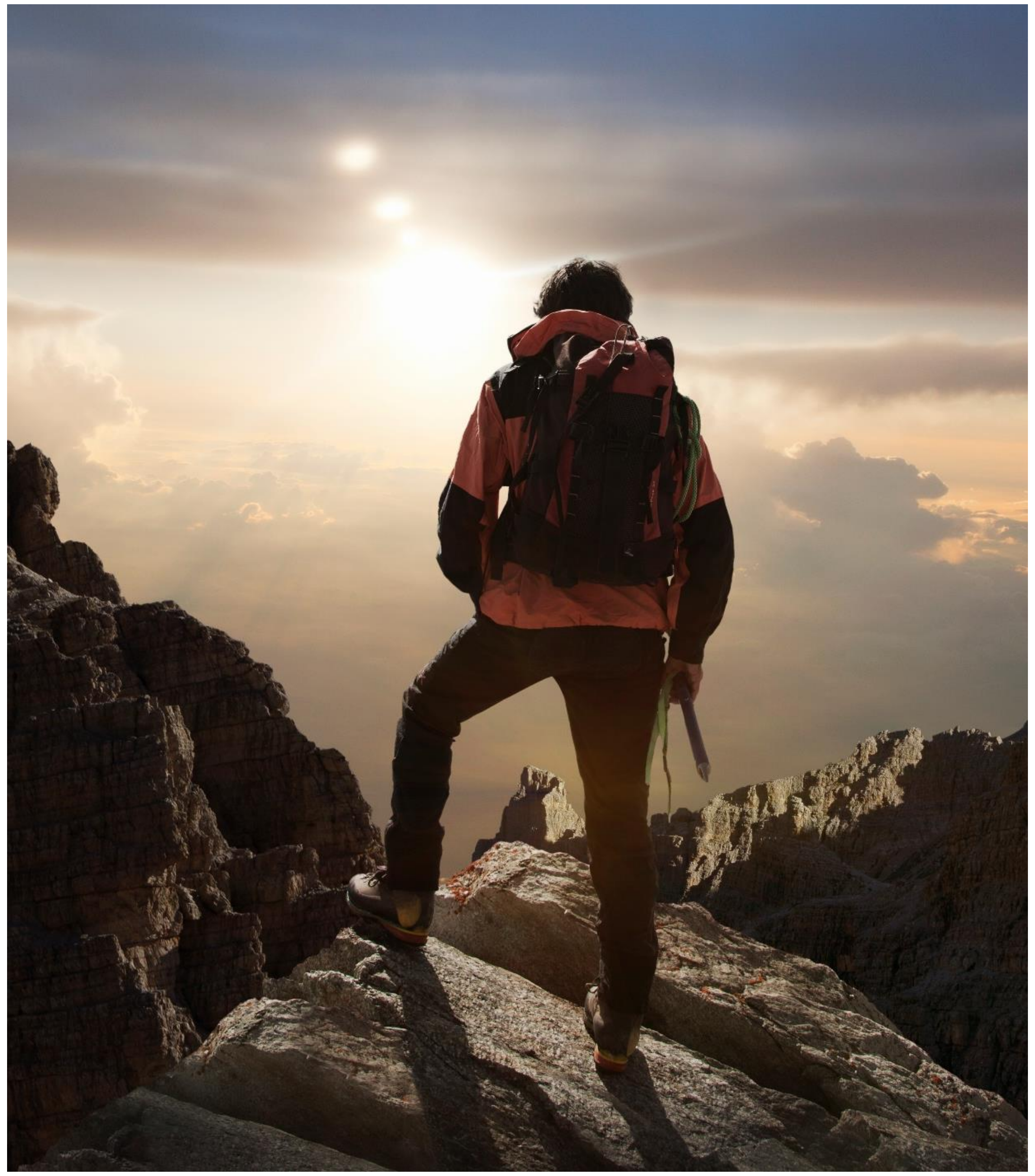

SCARY and A LOT OF WORK
→ **but also beautiful and fun.**

Good stress.

Resilient to Stress

You can't avoid all stress, but you can keep yourself strong and resilient.

Ways to stay strong to handle normal stress:

1. Getting enough sleep regularly
2. Practicing Mindfulness
3. Getting Exercise
4. Balancing blood pressure with healthy diet
5. **Other (discuss, and add ideas in the chat)**
6. *More ideas in this week's handout!*

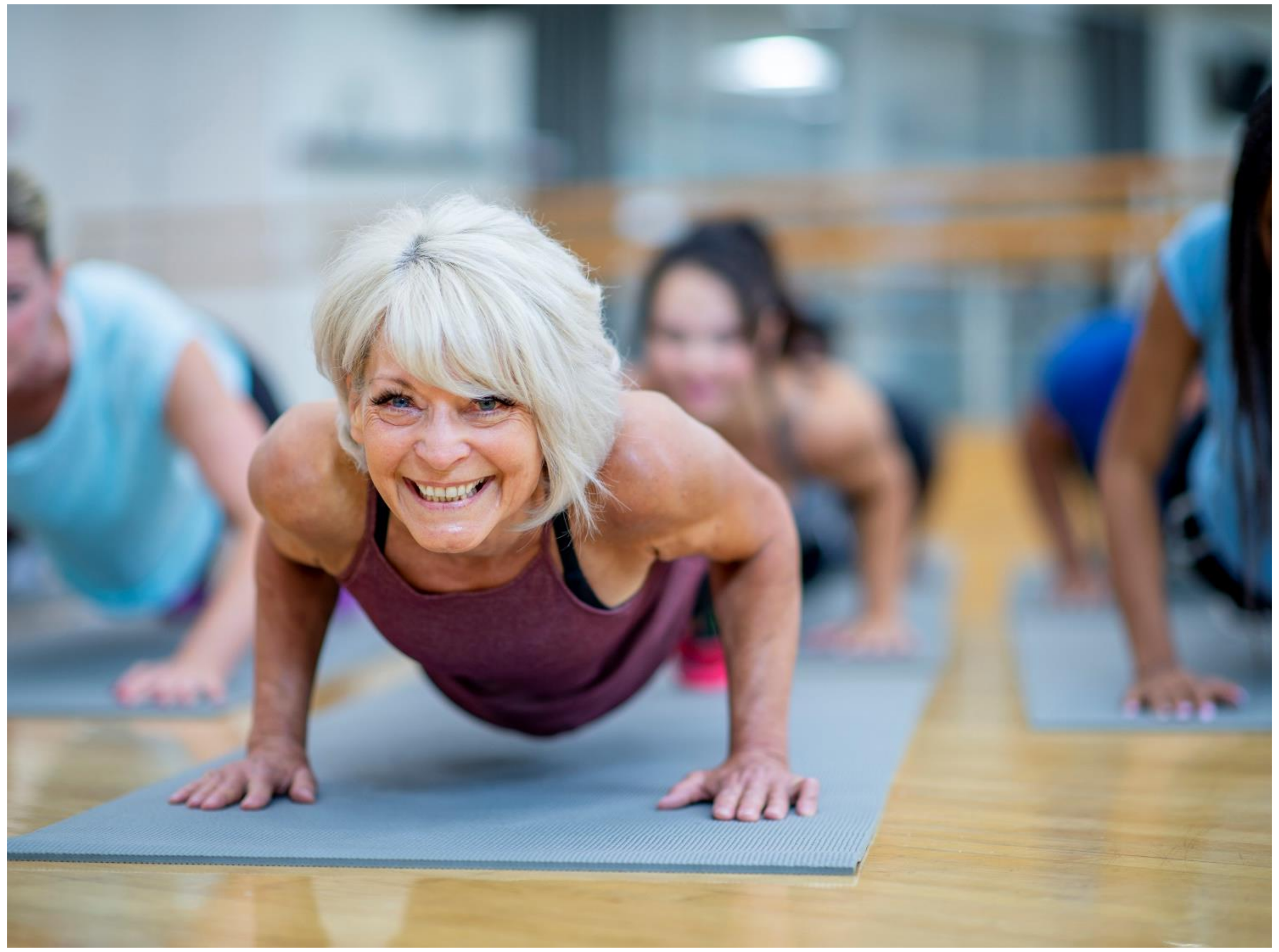

Balancing Stress

When health is your priority, other things need to become lower priorities.

1. Prioritize stresses with NET BENEFIT
   - When it's hard work, but worth it
2. If you can choose, eliminate unneeded bad stress
   - If something isn't working for you, let it go
3. If you can't eliminate, move self-care to the front
   - Take care of your needs first, then tackle the stress
4. Ask for support, don't go it alone
   - Life is tough and gives us many challenges, but you don't have to face everything alone.

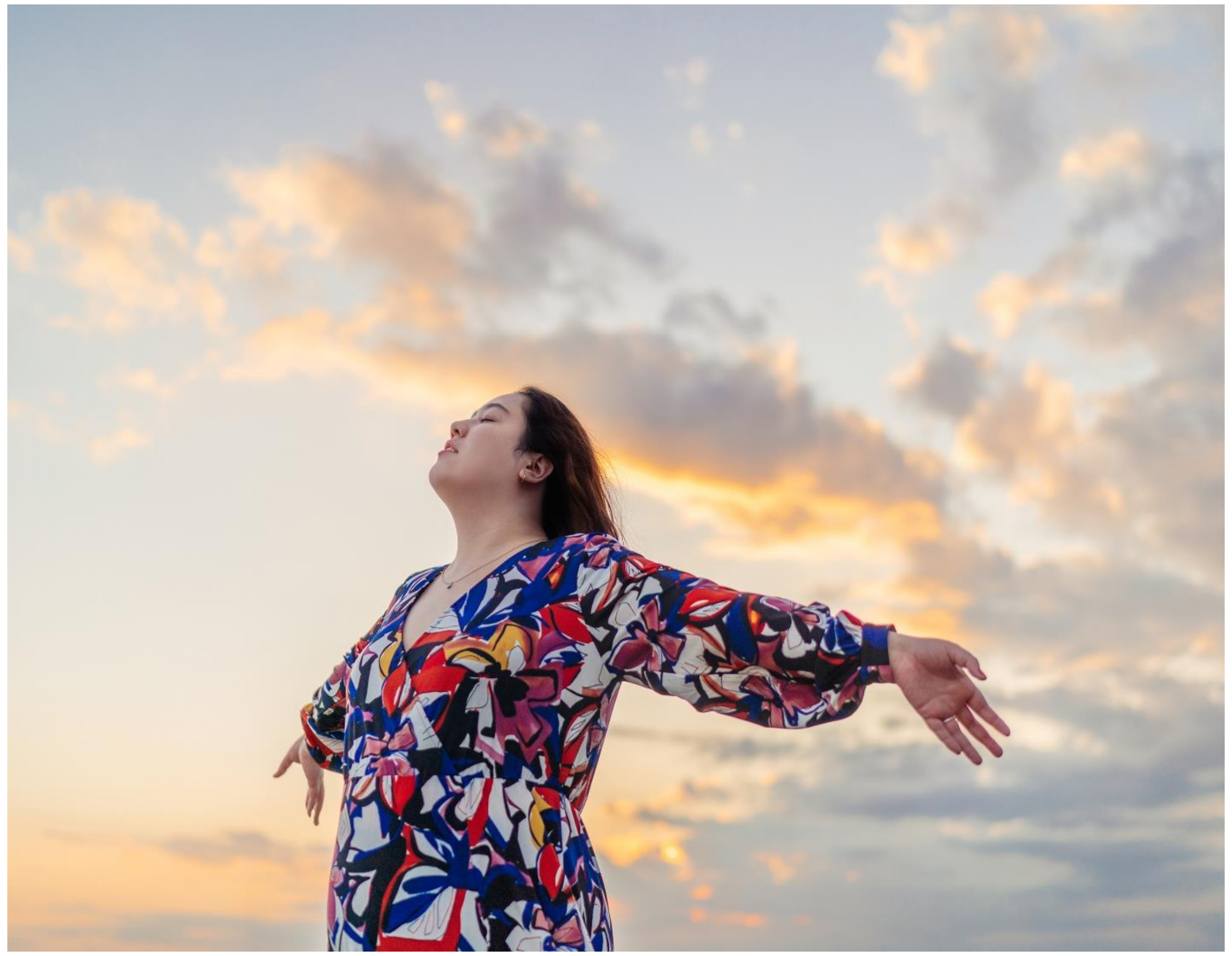

Sleep Hygiene

B B - H E A L T H

Sometimes everything feels better after a good night's sleep– don't you think?

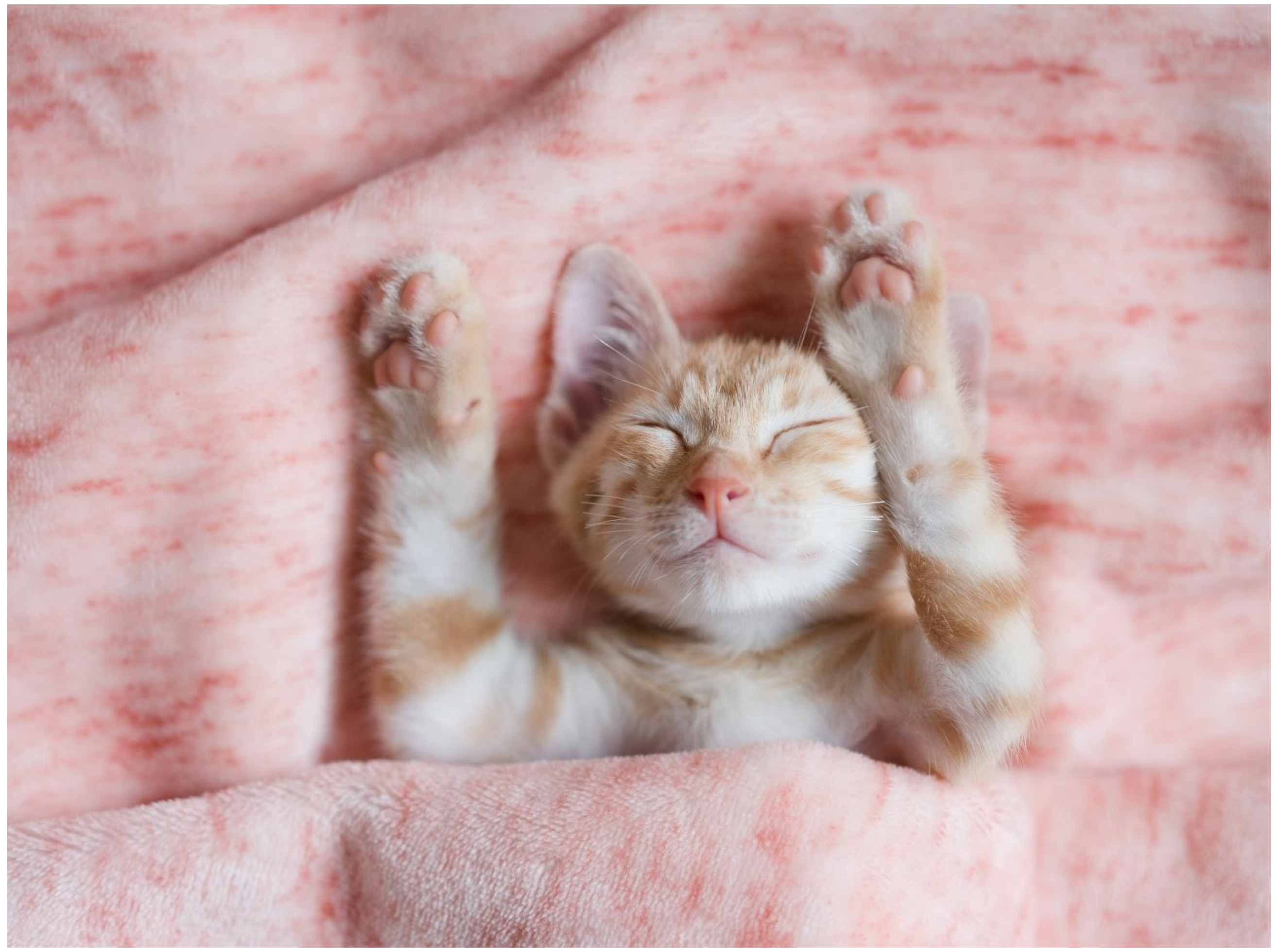

- ✓ 7-8 hours a night
- ✓ Going to bed at the same time
- ✓ Going to bed before midnight
- ✓ More than 2-3 hours since last eating
- ✓ At least 1 hour without devices/screens
- ✓ Dark, comfortable environment
- ✓ Daily physical activity
- ✓ Limit caffeine and tobacco (esp late in the day)
- ✓ *More ideas on page 5*

? You can ask your interventionist about your sleep quality from FitBit. You can also self-monitor your progress in your Fitbit app!

Laughter and Health

BB-HEALTH

Laughing is great stress relief and it also has a strong positive effect on health!

- Distracts you from stress
- Stabilizes blood sugar
- Balances hormonal response to stress
- Reduces hunger cravings
- Reduces fat storage signaling
- Feels good
- Burns calories
- Helps you bond with loves ones

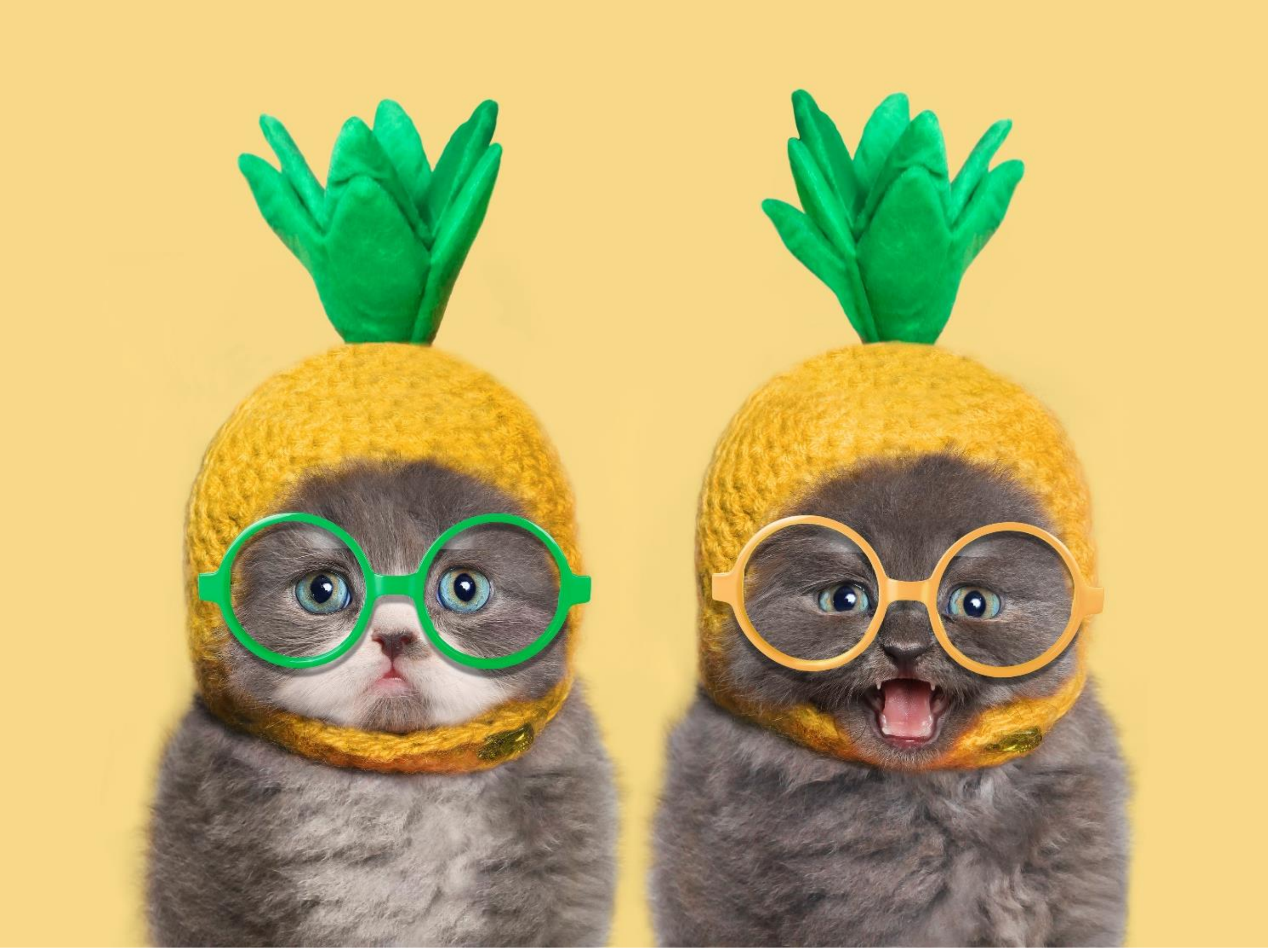

Tell us a Joke!

What is your favorite Joke?

- Put your favorite (clean) joke in the chat!

What are your favorite sources for jokes?

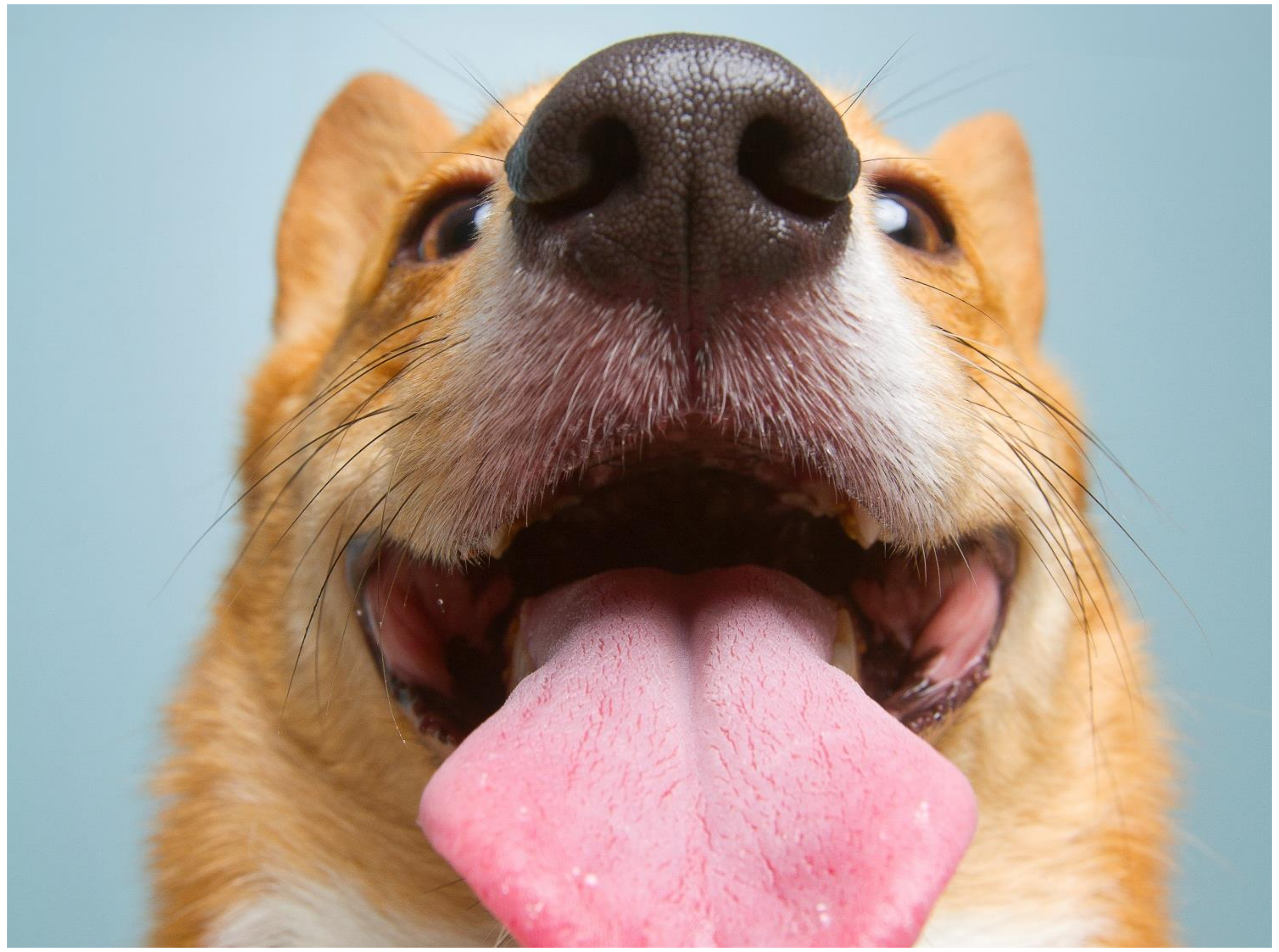

- https://www.countryliving.com/life/a27452412/best-dad-jokes/
- https://parade.com/940979/kelseypelzer/best-dad-jokes/
- https://www.cultureamp.com/blog/funny-jokes-for-the-workplace

- Go here for a NEW joke everyday! https://www.ajokeaday.com/

Put it into Practice

Your Lifestyle, in Control!

1. Track those meals and take your health EVERYDAY!
   - Monitor your progress and identify areas for improvement towards YOUR GOALS

2. Aim to get in 90 min of moderate or vigorous exercise ***a week*** by our next session!
   - Make your total active time over 200 min this week!
   - Keep up your steps, reduce sedentary time– get all 9!

3. Practice Good sleep Hygiene and other Stress Resilience
   - Build up strength to stay on track even when life challenges you
   - Bring in a joke to share with the group next time

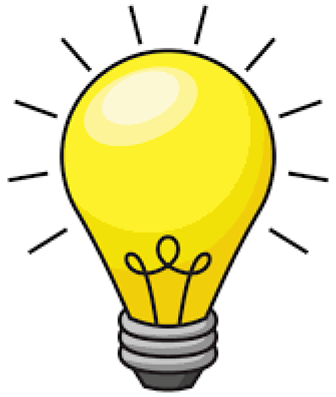

THIS WEEK'S TIP: ***Taking care of yourself comes first***
Mind and body, your needs are your top priority. It can be hard to say no but you will feel better if you do what is right for you!

Questions?

BB-HEALTH

For next month:

1. Increase your physical activity!

2. Track your food and exercise and **Weigh Every Day**

3. Remember your calorie, fat, and activity targets

4. Email your logs and progress to the Intervention Team and Review the response!

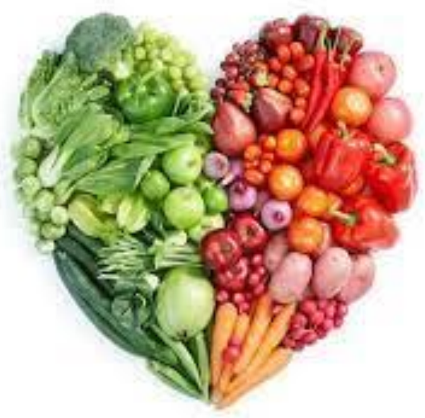

FOOD FOR THOUGHT
Laugh! Sleep! Hydrate! Walk! Eat fruits and veggies! That's a recipe for a healthy happy life!
