# Supplementary material for: Development and Feasibility of an eHealth Diabetes Prevention Program Adapted for Older Adults—Results from a Randomized Control Pilot Study
Source: Nutrients. 2024 Mar 23;16(7):930. doi: 10.3390/nu16070930 (PMC11154527; doi:10.3390/nu16070930)
Supplement: Supplementary file 1 [file nutrients-16-00930-s001.zip › session17.pptx]

## Slide 1
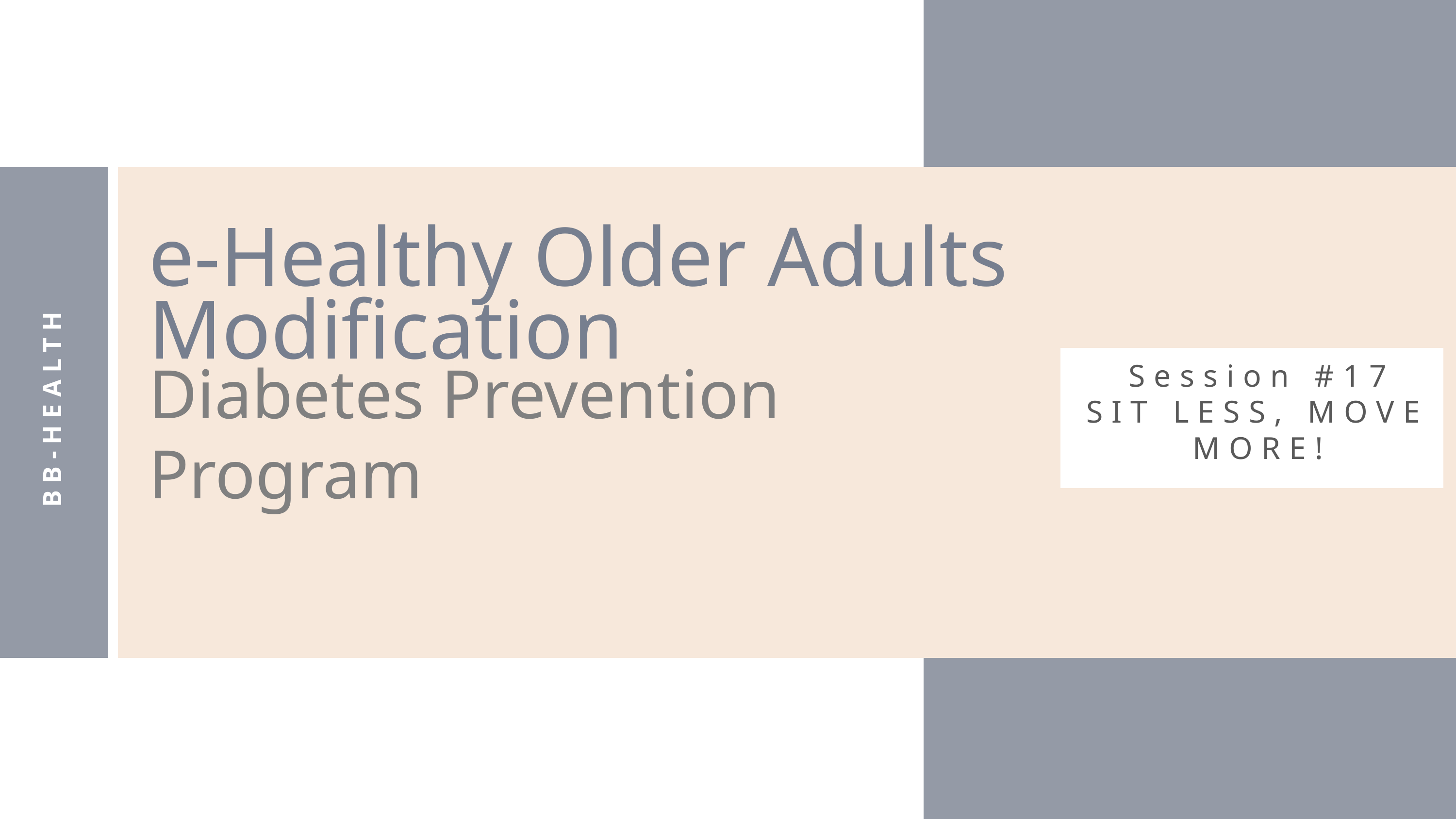

OPEN REPORTS
e-Healthy Older Adults Modification
Session #17
SIT LESS, MOVE MORE!
Diabetes Prevention Program
BB-HEALTH

## Slide 2
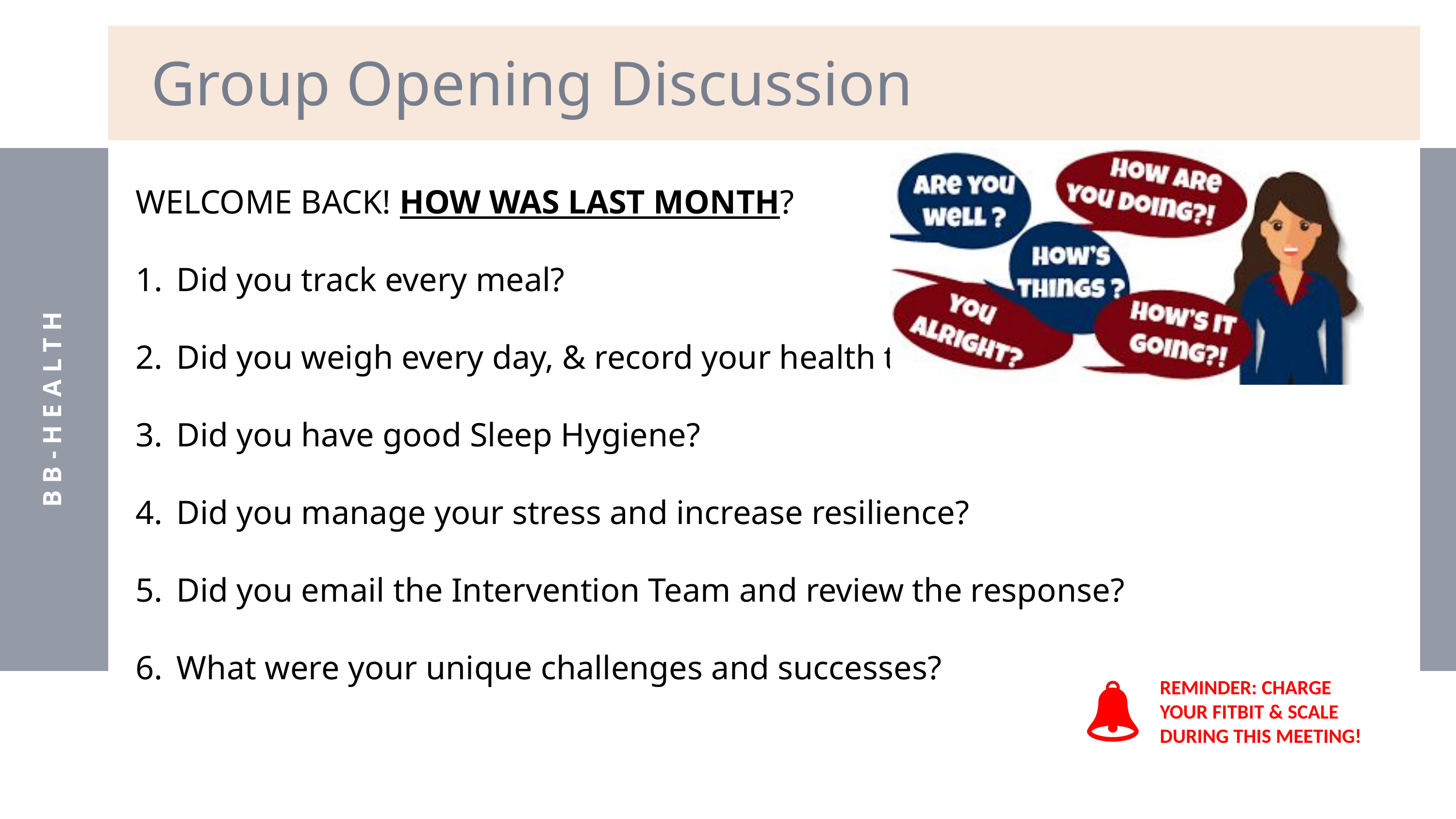

Group Opening Discussion
WELCOME BACK! HOW WAS LAST MONTH?
Did you track every meal?
Did you weigh every day, & record your health today?
Did you have good Sleep Hygiene?
Did you manage your stress and increase resilience?
Did you email the Intervention Team and review the response?
What were your unique challenges and successes?
BB-HEALTH
REMINDER: CHARGE YOUR FITBIT & SCALE DURING THIS MEETING!

## Slide 3
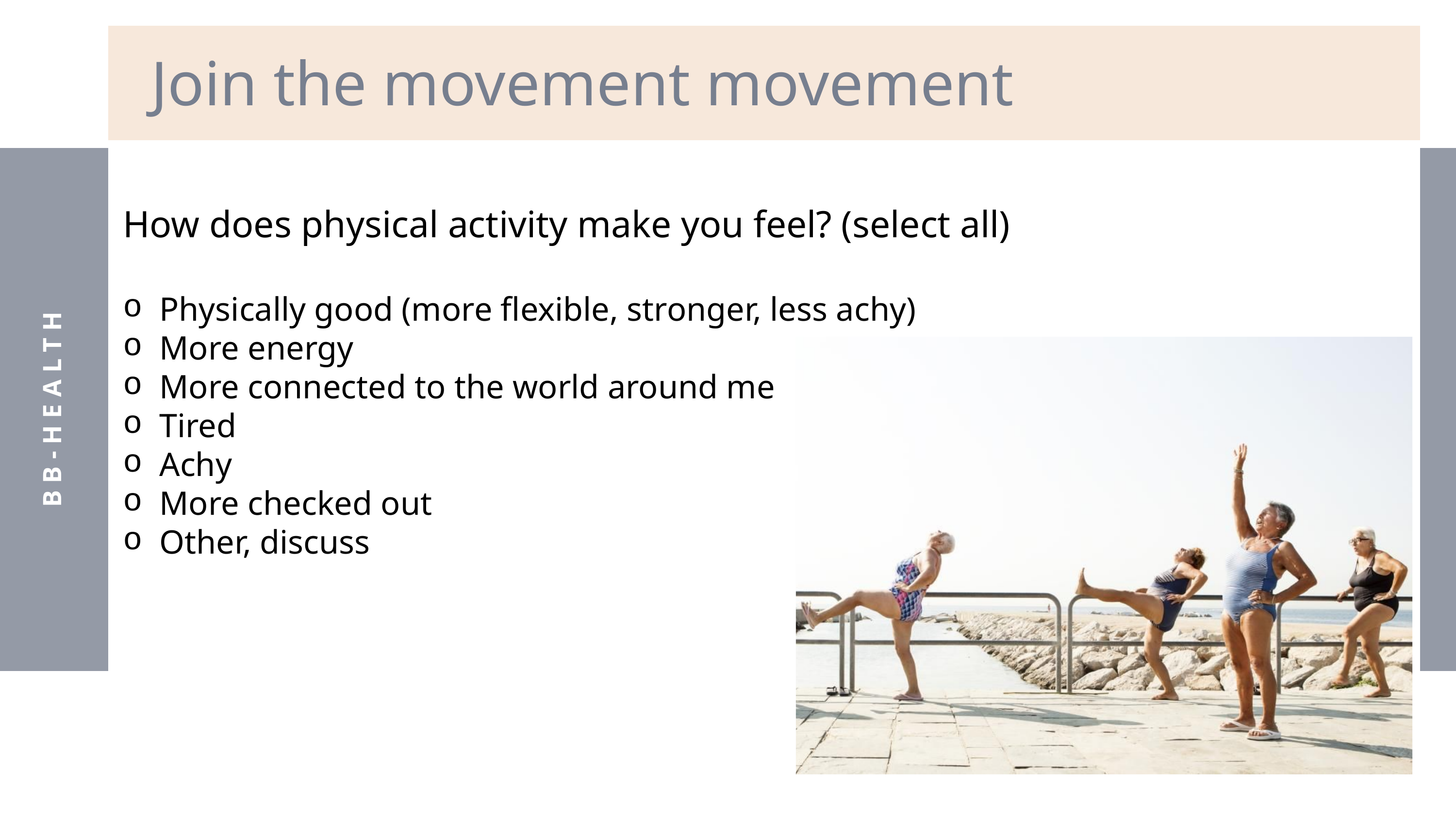

Join the movement movement
How does physical activity make you feel? (select all)
Physically good (more flexible, stronger, less achy)
More energy
More connected to the world around me
Tired
Achy
More checked out
Other, discuss
BB-HEALTH

## Slide 4
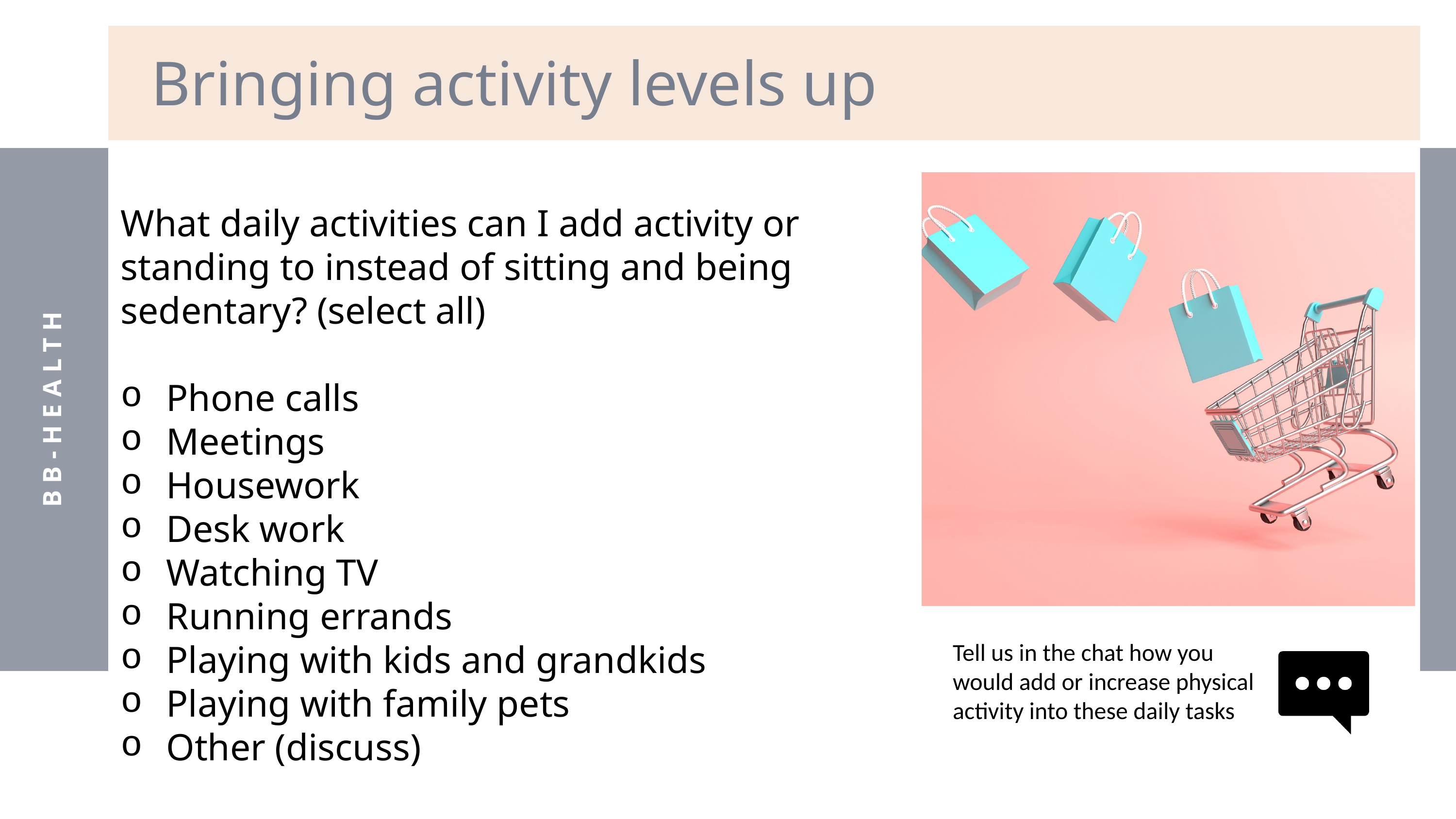

Bringing activity levels up
What daily activities can I add activity or standing to instead of sitting and being sedentary? (select all)
Phone calls
Meetings
Housework
Desk work
Watching TV
Running errands
Playing with kids and grandkids
Playing with family pets
Other (discuss)
BB-HEALTH
Tell us in the chat how you would add or increase physical activity into these daily tasks

## Slide 5
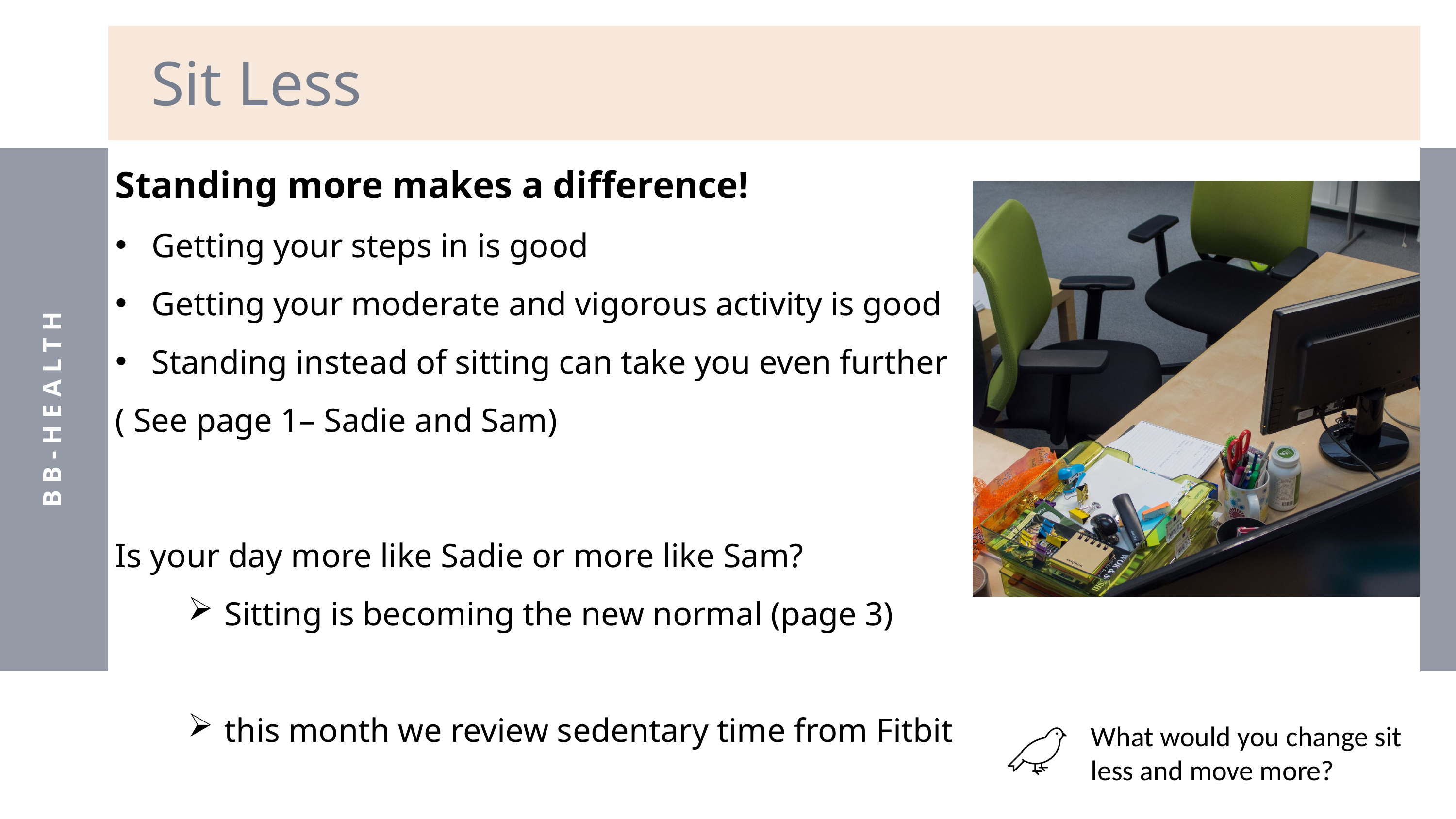

Sit Less
Standing more makes a difference!
Getting your steps in is good
Getting your moderate and vigorous activity is good
Standing instead of sitting can take you even further
( See page 1– Sadie and Sam)
Is your day more like Sadie or more like Sam?
Sitting is becoming the new normal (page 3)
this month we review sedentary time from Fitbit
BB-HEALTH
What would you change sit less and move more?

## Slide 6
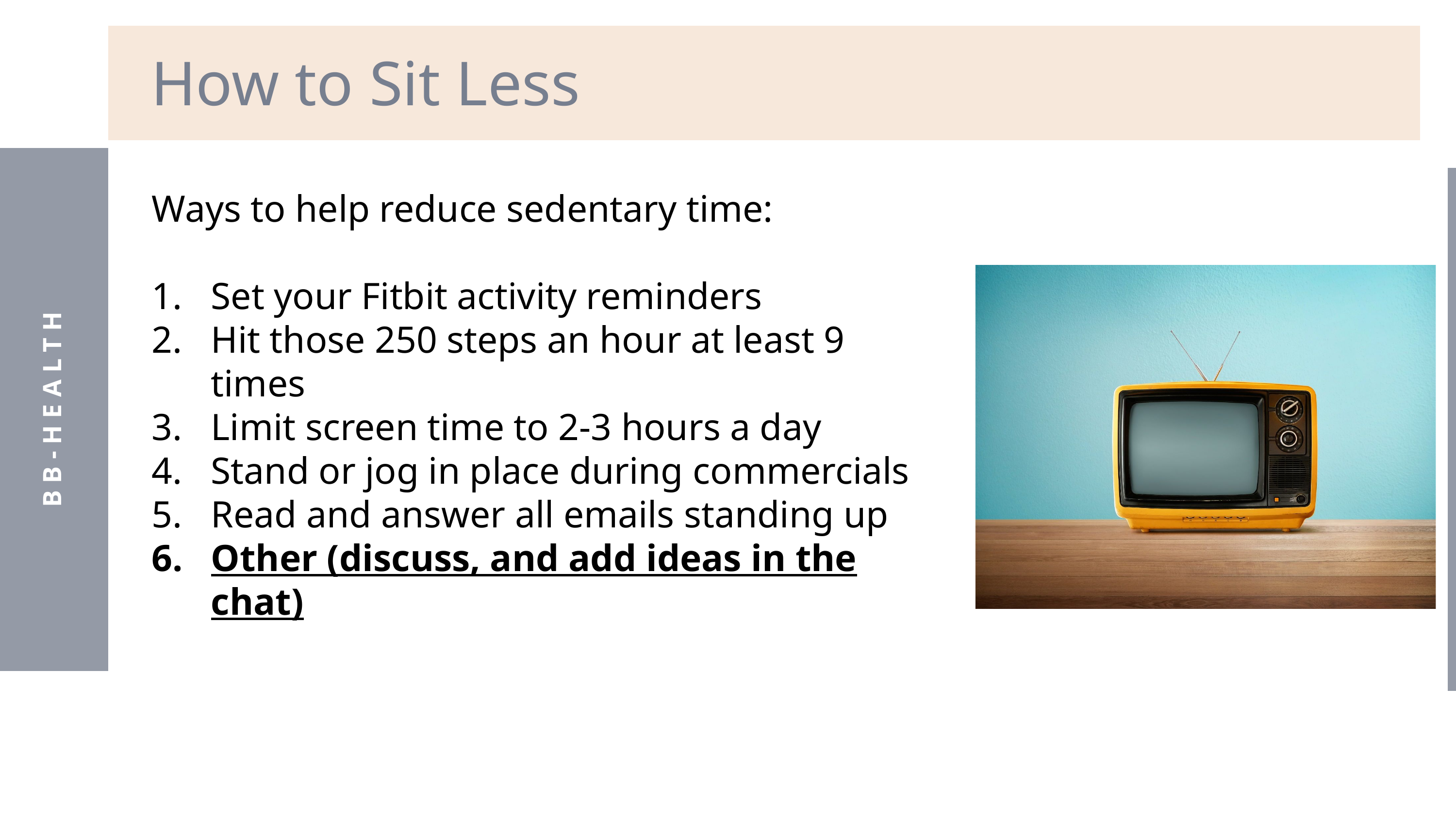

How to Sit Less
Ways to help reduce sedentary time:
Set your Fitbit activity reminders
Hit those 250 steps an hour at least 9 times
Limit screen time to 2-3 hours a day
Stand or jog in place during commercials
Read and answer all emails standing up
Other (discuss, and add ideas in the chat)
BB-HEALTH

## Slide 7
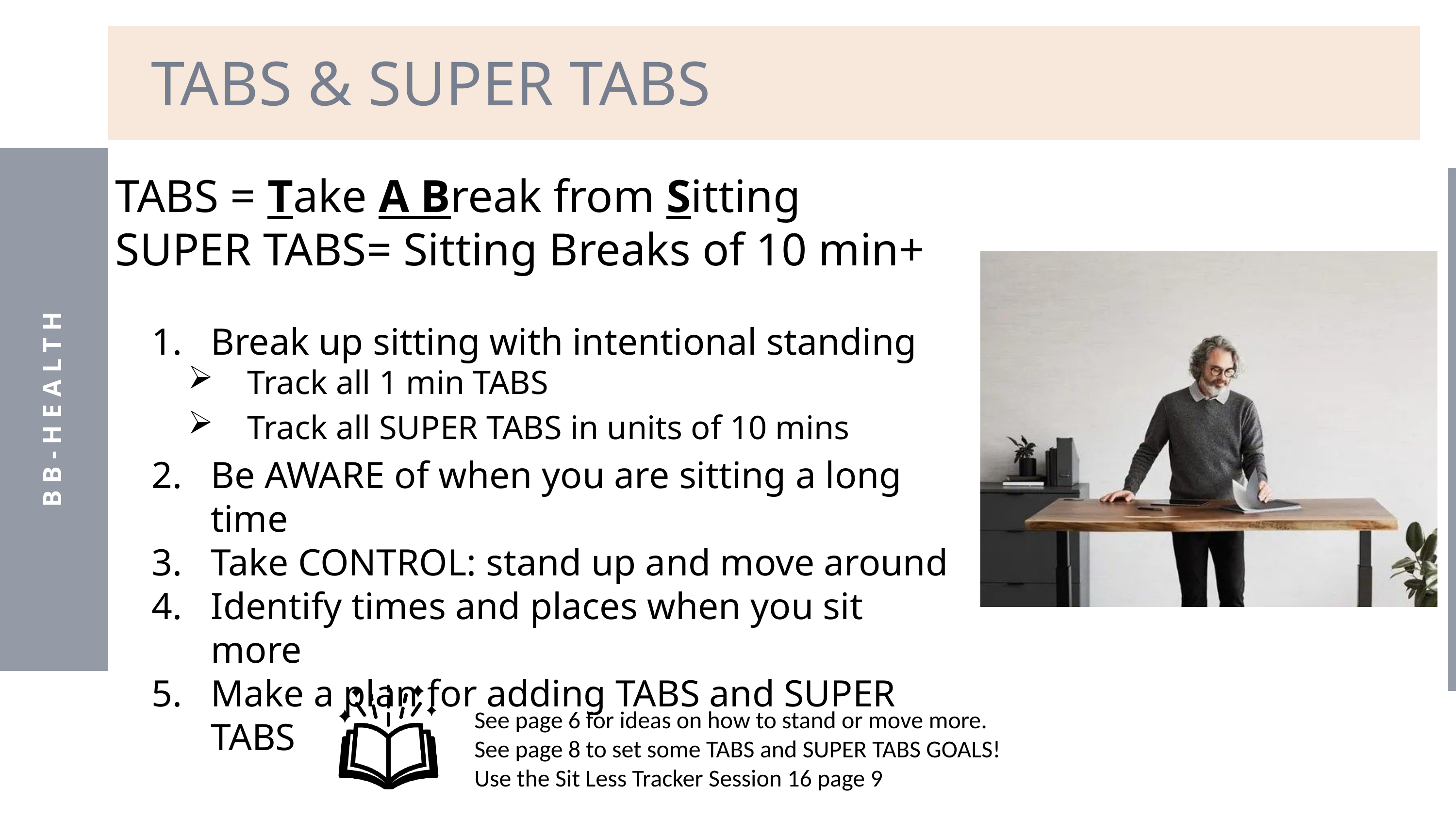

TABS & SUPER TABS
TABS = Take A Break from Sitting
SUPER TABS= Sitting Breaks of 10 min+
Break up sitting with intentional standing
Track all 1 min TABS
Track all SUPER TABS in units of 10 mins
Be AWARE of when you are sitting a long time
Take CONTROL: stand up and move around
Identify times and places when you sit more
Make a plan for adding TABS and SUPER TABS
BB-HEALTH
See page 6 for ideas on how to stand or move more.
See page 8 to set some TABS and SUPER TABS GOALS!
Use the Sit Less Tracker Session 16 page 9

## Slide 8
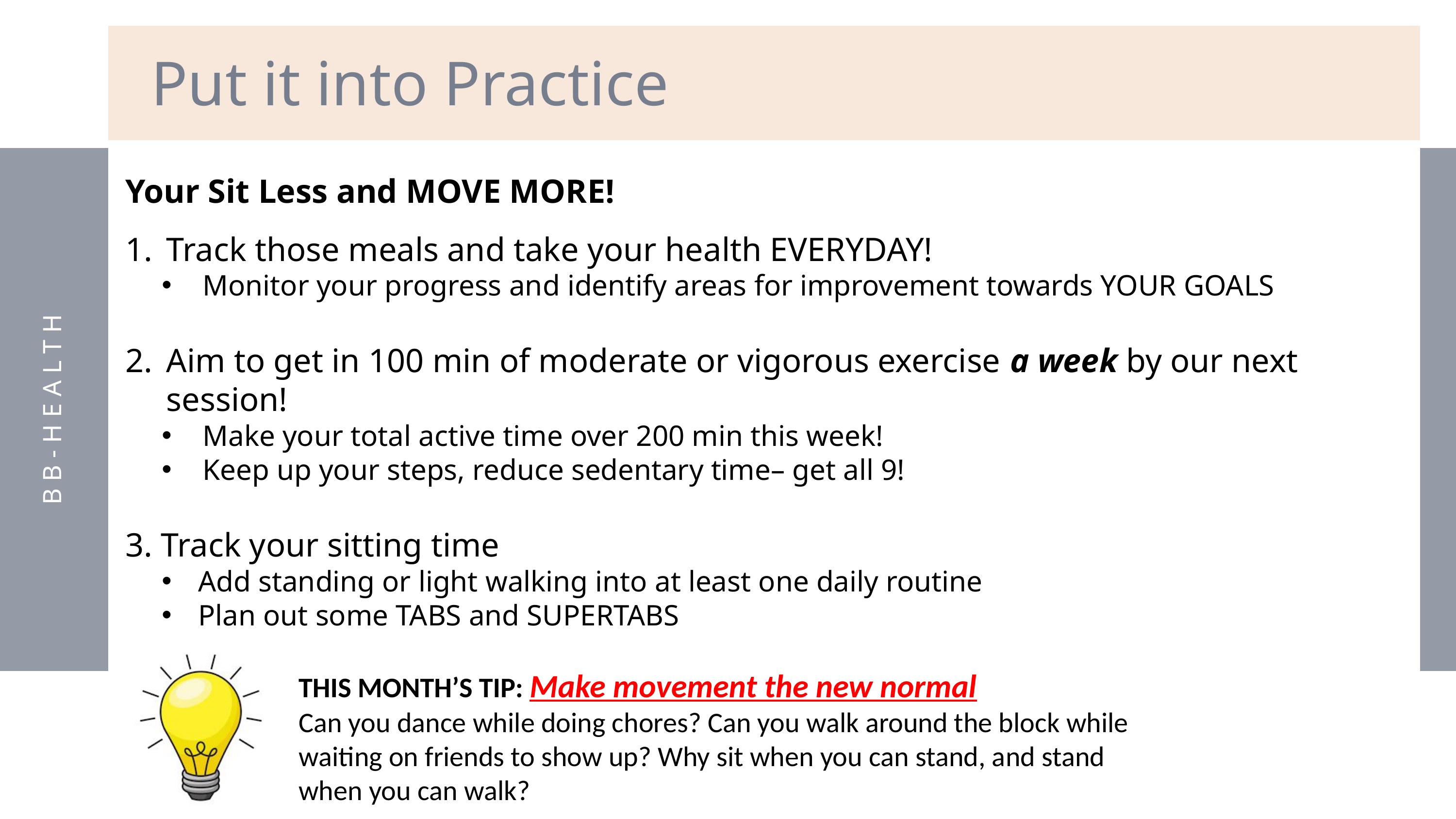

Put it into Practice
Your Sit Less and MOVE MORE!
Track those meals and take your health EVERYDAY!
Monitor your progress and identify areas for improvement towards YOUR GOALS
Aim to get in 100 min of moderate or vigorous exercise a week by our next session!
Make your total active time over 200 min this week!
Keep up your steps, reduce sedentary time– get all 9!
3. Track your sitting time
Add standing or light walking into at least one daily routine
Plan out some TABS and SUPERTABS
BB-HEALTH
THIS MONTH’S TIP: Make movement the new normal
Can you dance while doing chores? Can you walk around the block while waiting on friends to show up? Why sit when you can stand, and stand when you can walk?

## Slide 9
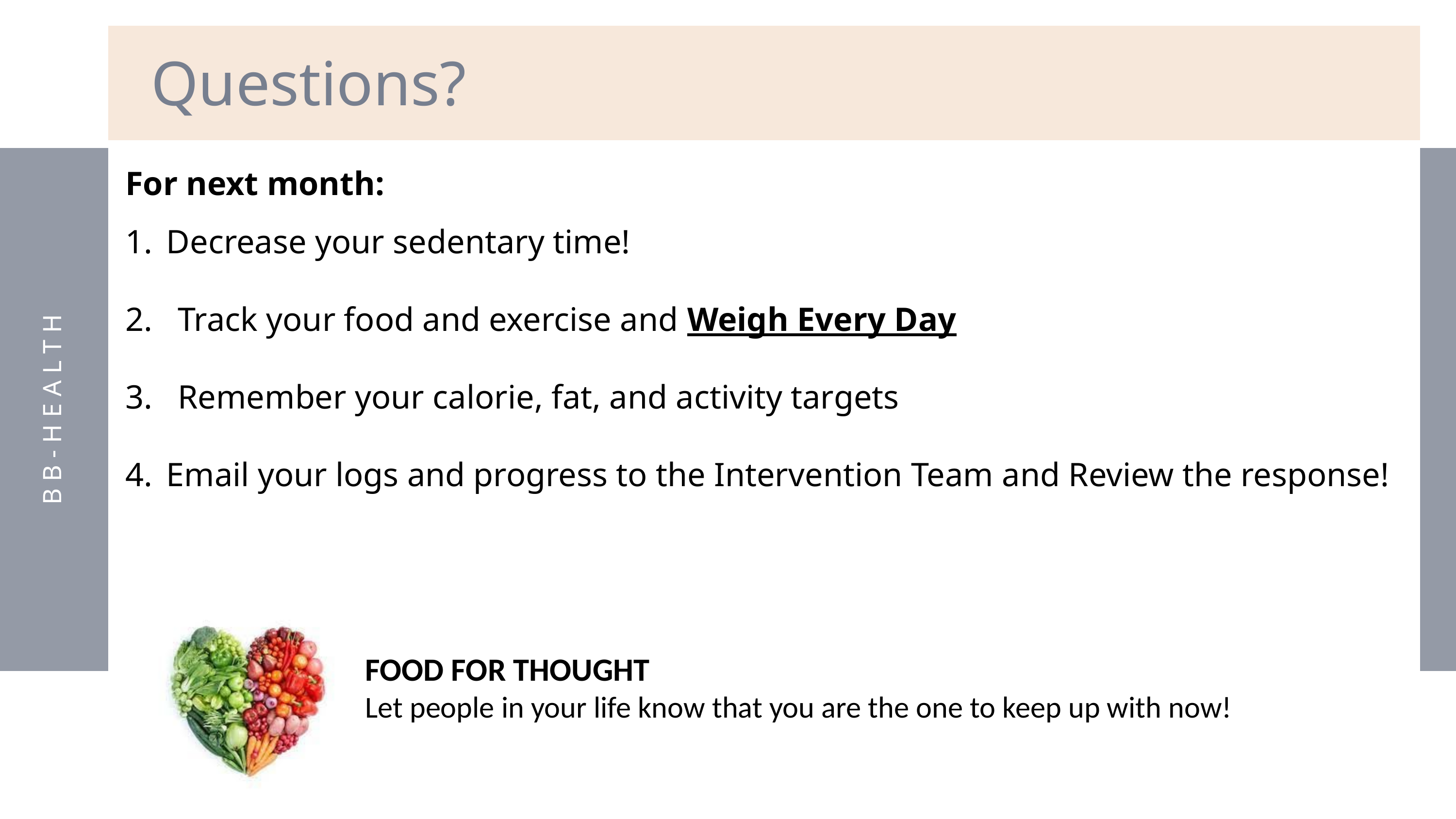

Questions?
For next month:
Decrease your sedentary time!
2. Track your food and exercise and Weigh Every Day
3. Remember your calorie, fat, and activity targets
Email your logs and progress to the Intervention Team and Review the response!
BB-HEALTH
FOOD FOR THOUGHT
Let people in your life know that you are the one to keep up with now!
