# Supplementary material for: Development and Feasibility of an eHealth Diabetes Prevention Program Adapted for Older Adults—Results from a Randomized Control Pilot Study
Source: Nutrients. 2024 Mar 23;16(7):930. doi: 10.3390/nu16070930 (PMC11154527; doi:10.3390/nu16070930)
Supplement: Supplementary file 1 [file nutrients-16-00930-s001.zip › Session18.pptx]

## Slide 1
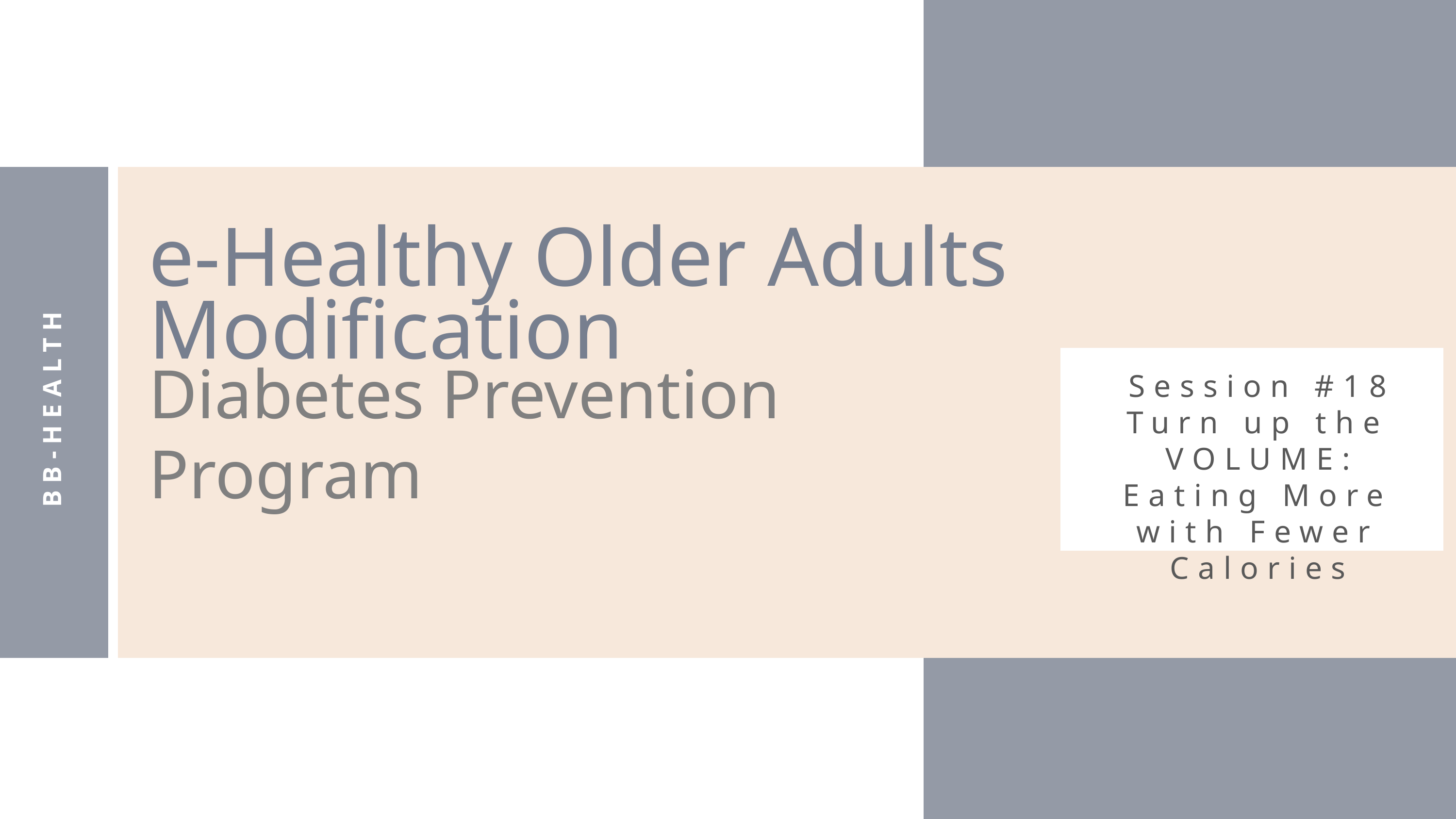

OPEN REPORTS
e-Healthy Older Adults Modification
Session #18
Turn up the VOLUME:
Eating More with Fewer Calories
Diabetes Prevention Program
BB-HEALTH

## Slide 2
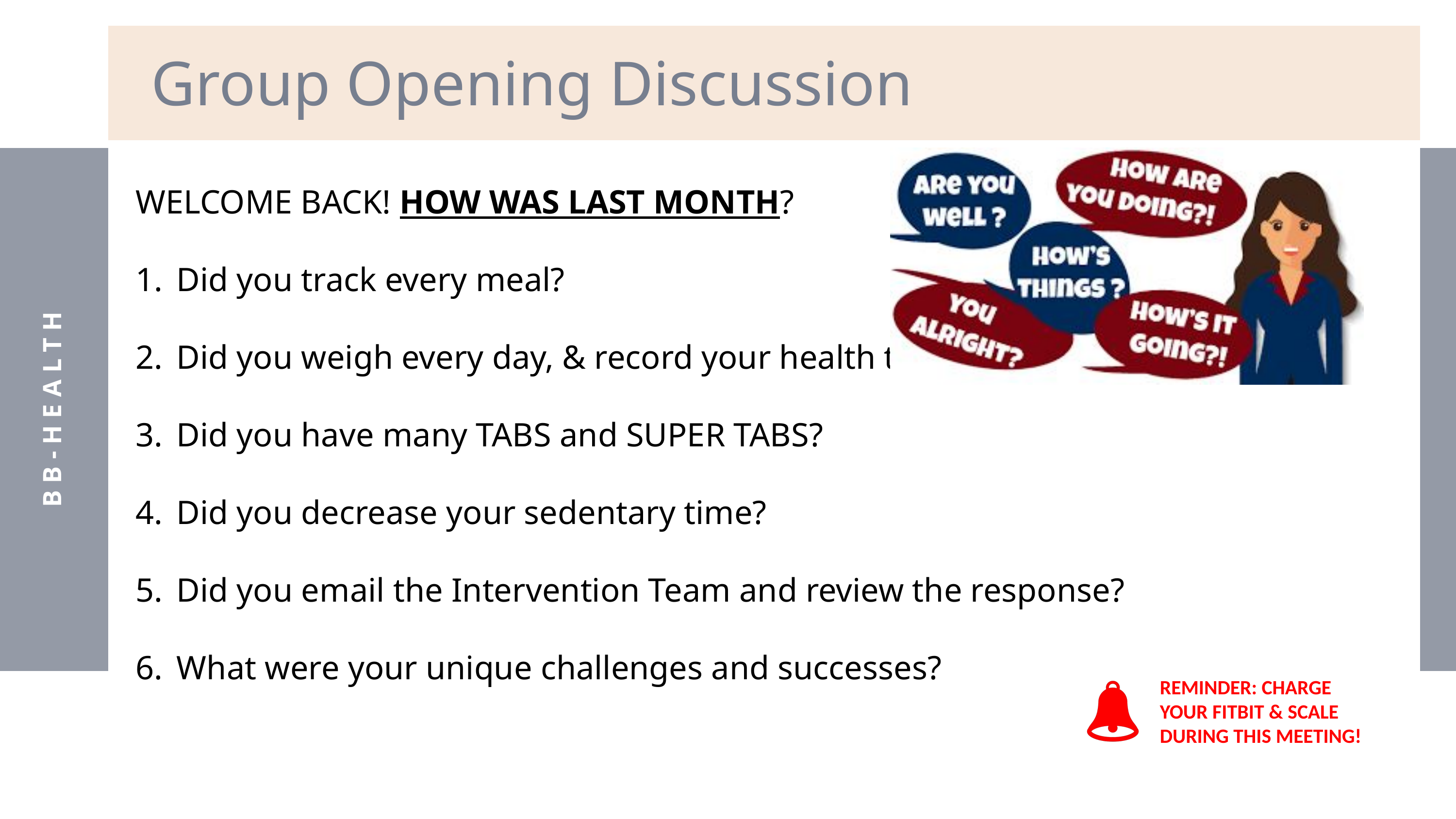

Group Opening Discussion
WELCOME BACK! HOW WAS LAST MONTH?
Did you track every meal?
Did you weigh every day, & record your health today?
Did you have many TABS and SUPER TABS?
Did you decrease your sedentary time?
Did you email the Intervention Team and review the response?
What were your unique challenges and successes?
BB-HEALTH
REMINDER: CHARGE YOUR FITBIT & SCALE DURING THIS MEETING!

## Slide 3
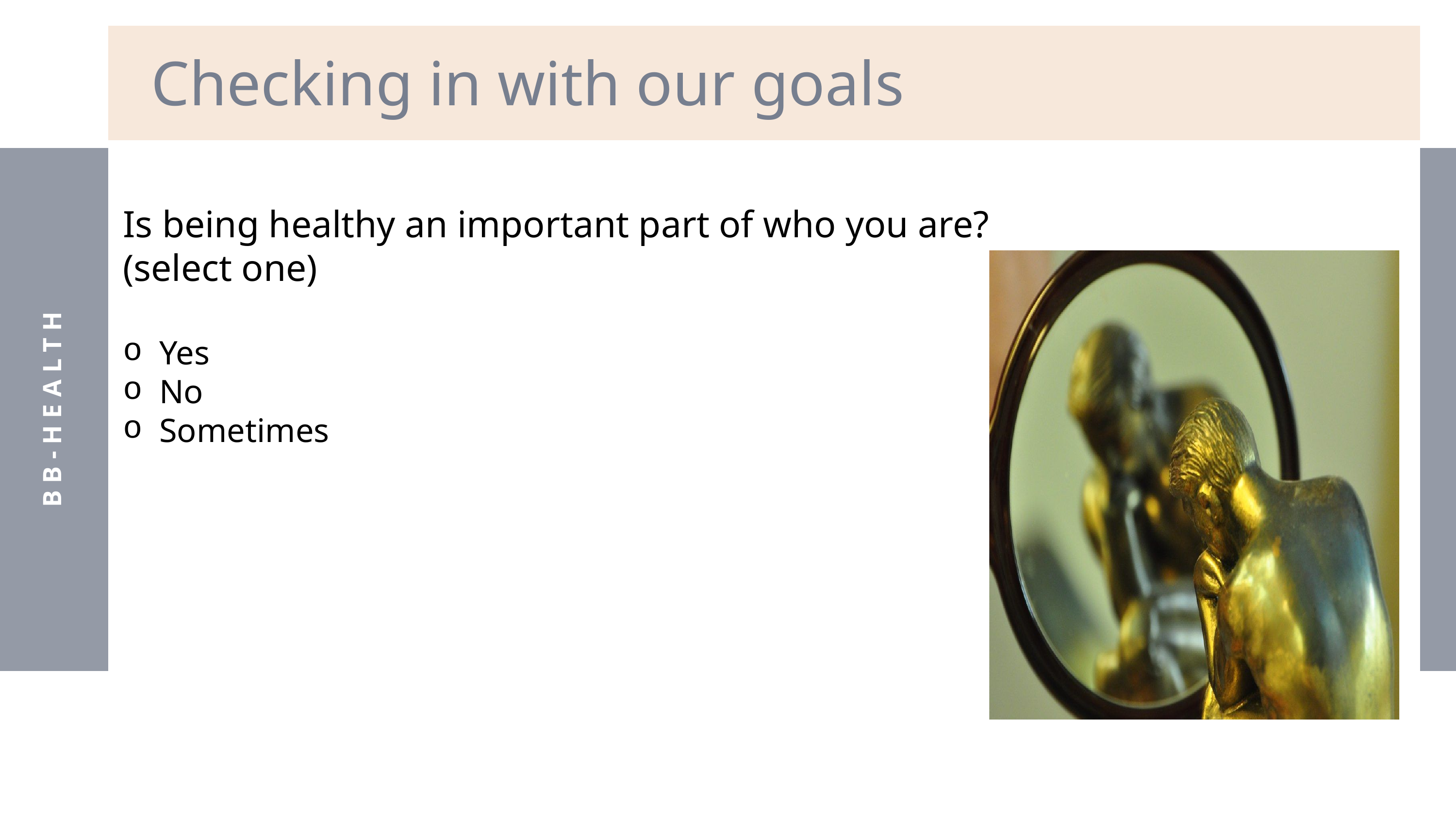

Checking in with our goals
Is being healthy an important part of who you are? (select one)
Yes
No
Sometimes
BB-HEALTH

## Slide 4
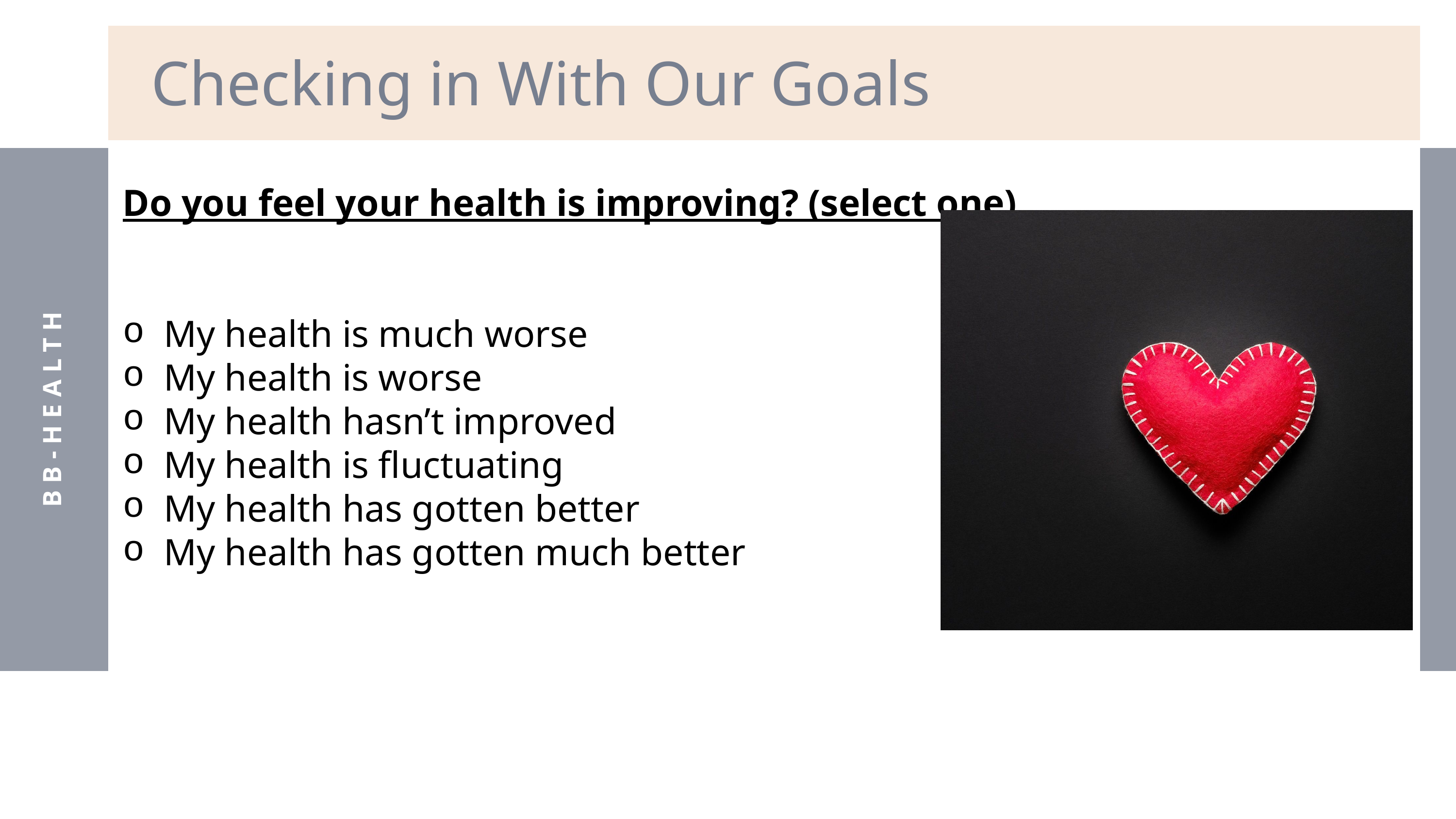

Checking in With Our Goals
Do you feel your health is improving? (select one)
My health is much worse
My health is worse
My health hasn’t improved
My health is fluctuating
My health has gotten better
My health has gotten much better
BB-HEALTH

## Slide 5
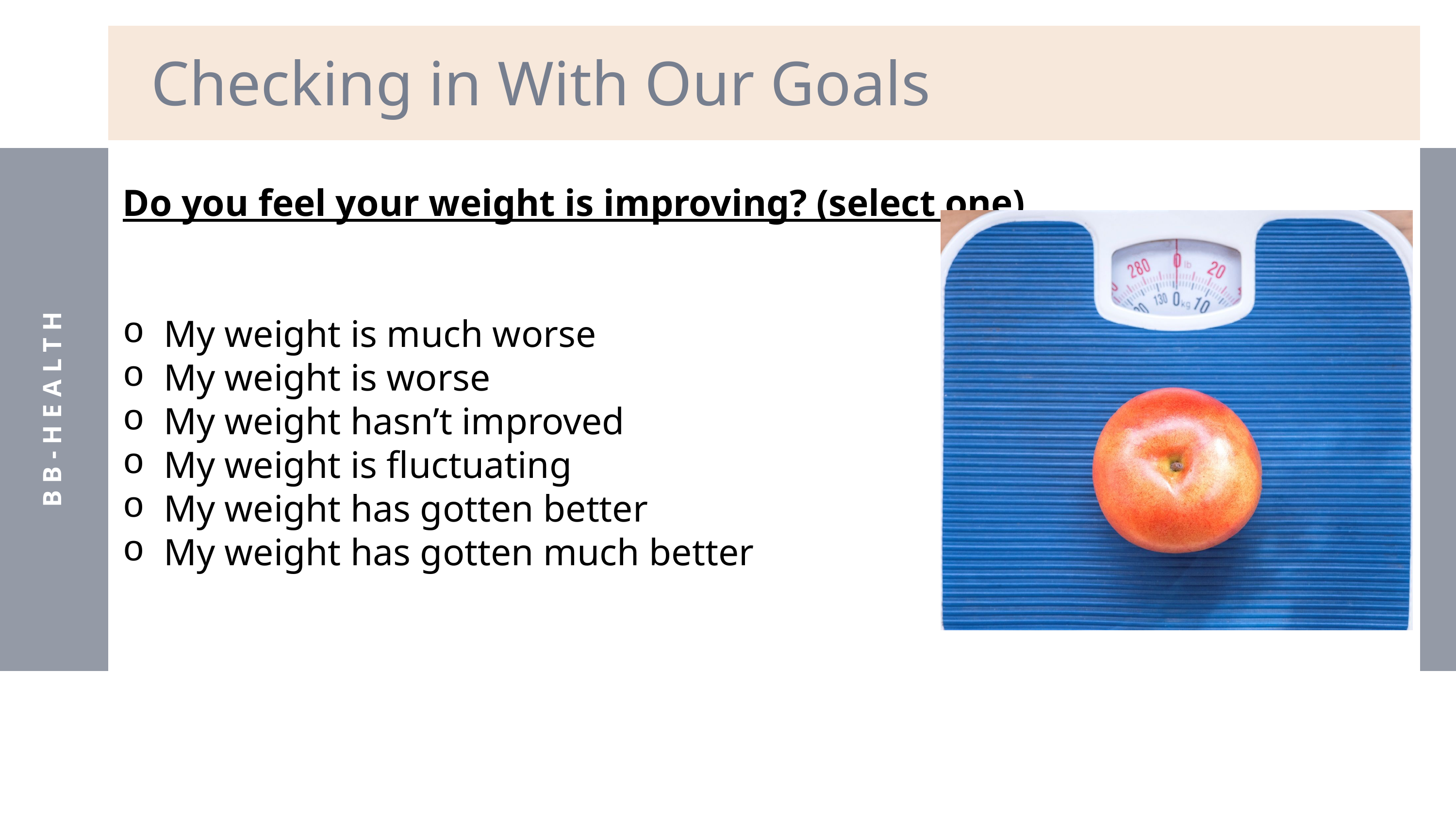

Checking in With Our Goals
Do you feel your weight is improving? (select one)
My weight is much worse
My weight is worse
My weight hasn’t improved
My weight is fluctuating
My weight has gotten better
My weight has gotten much better
BB-HEALTH

## Slide 6
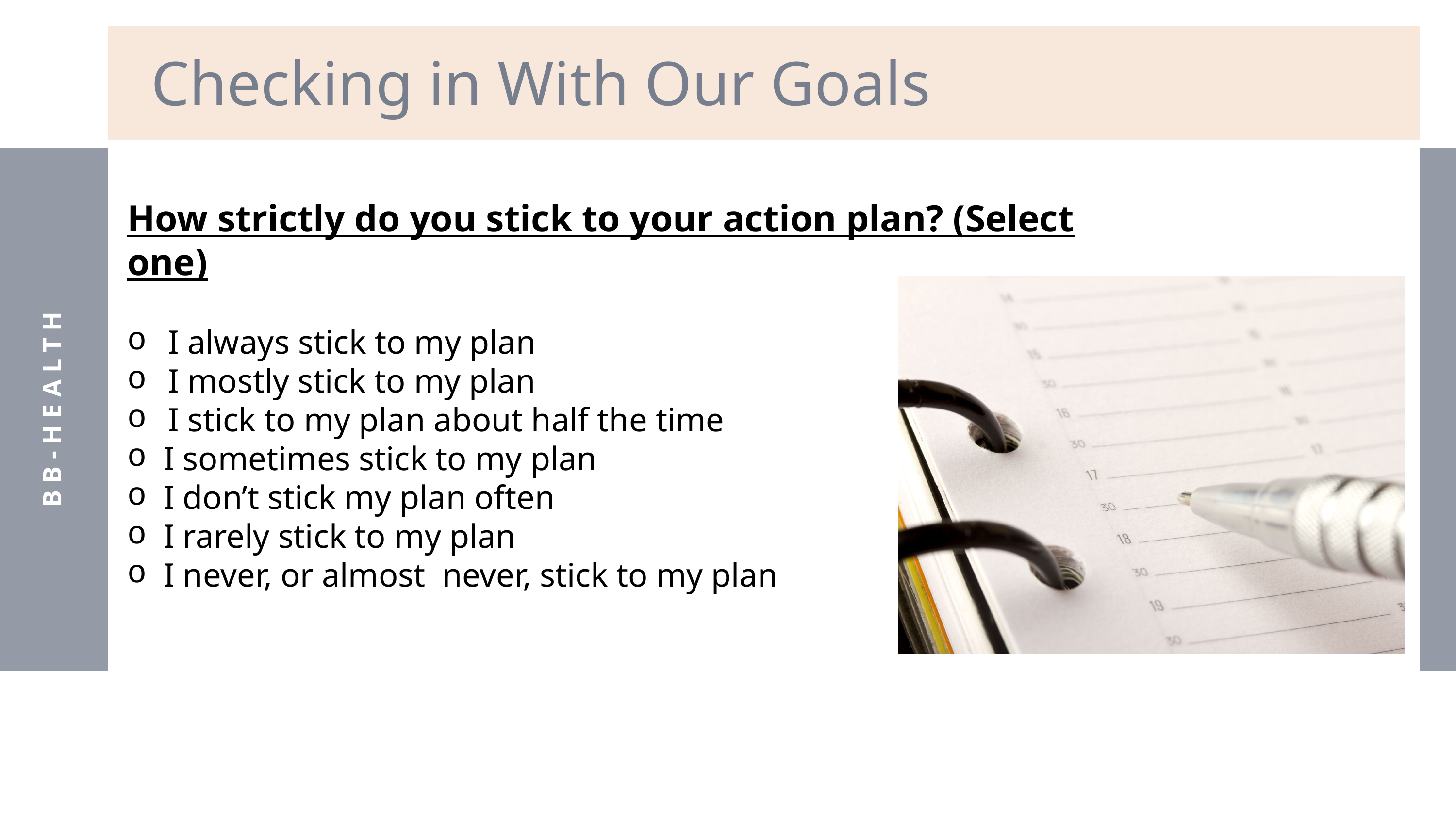

Checking in With Our Goals
How strictly do you stick to your action plan? (Select one)
I always stick to my plan
I mostly stick to my plan
I stick to my plan about half the time
I sometimes stick to my plan
I don’t stick my plan often
I rarely stick to my plan
I never, or almost never, stick to my plan
BB-HEALTH

## Slide 7
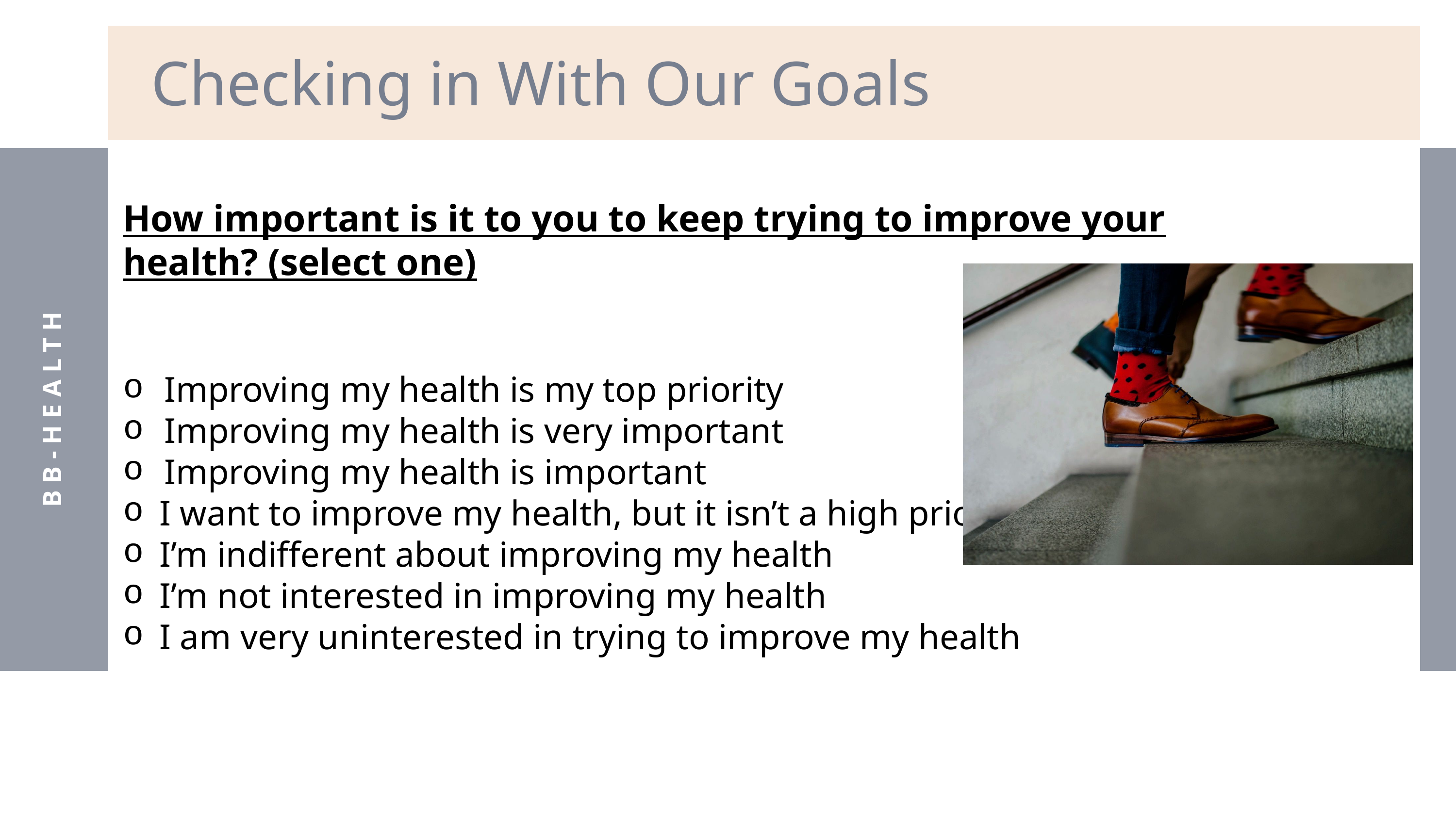

Checking in With Our Goals
How important is it to you to keep trying to improve your health? (select one)
Improving my health is my top priority
Improving my health is very important
Improving my health is important
I want to improve my health, but it isn’t a high priority
I’m indifferent about improving my health
I’m not interested in improving my health
I am very uninterested in trying to improve my health
BB-HEALTH

## Slide 8
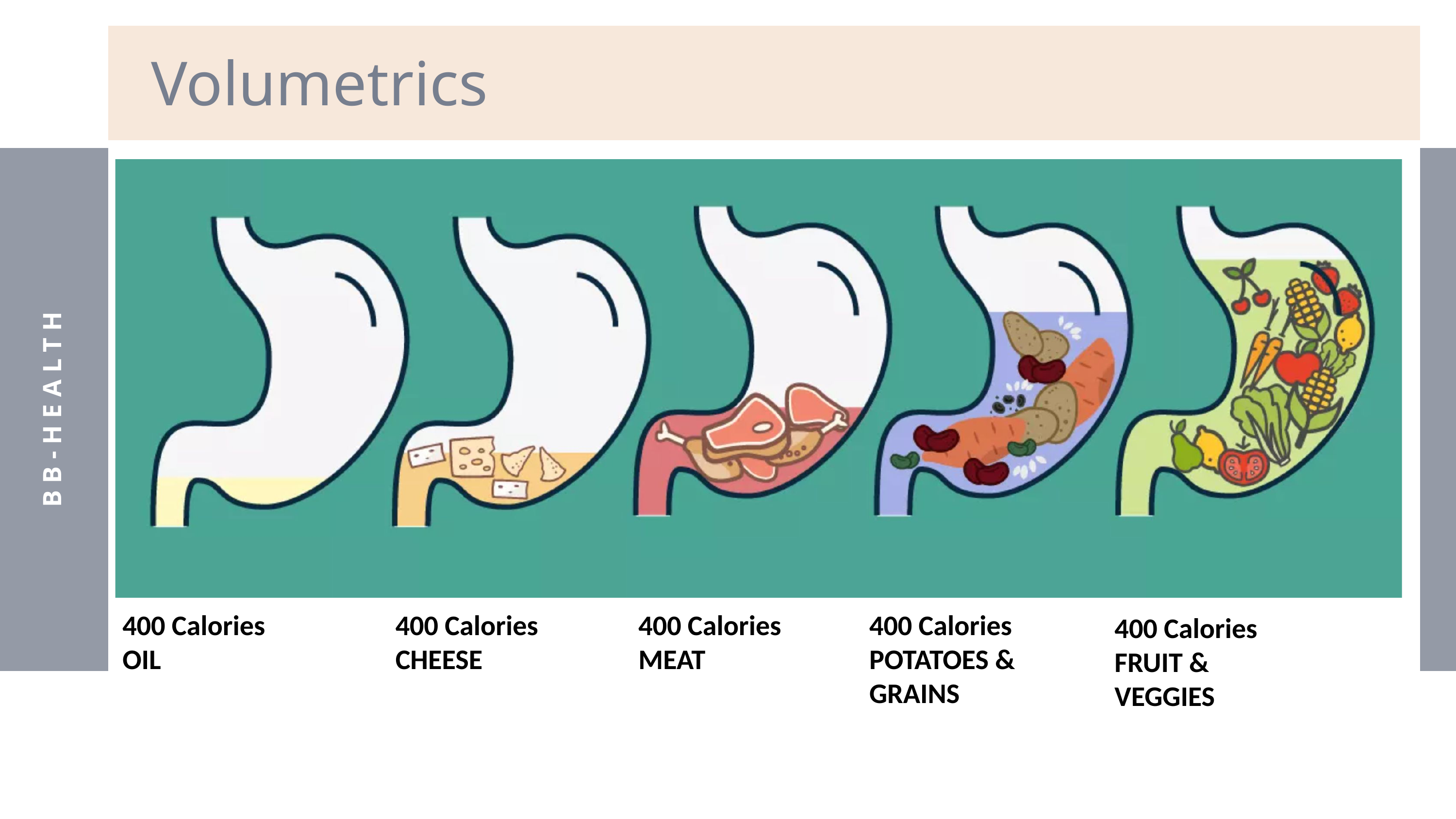

Volumetrics
BB-HEALTH
400 Calories
POTATOES &
GRAINS
400 Calories
CHEESE
400 Calories
OIL
400 Calories
MEAT
400 Calories
FRUIT & VEGGIES

## Slide 9
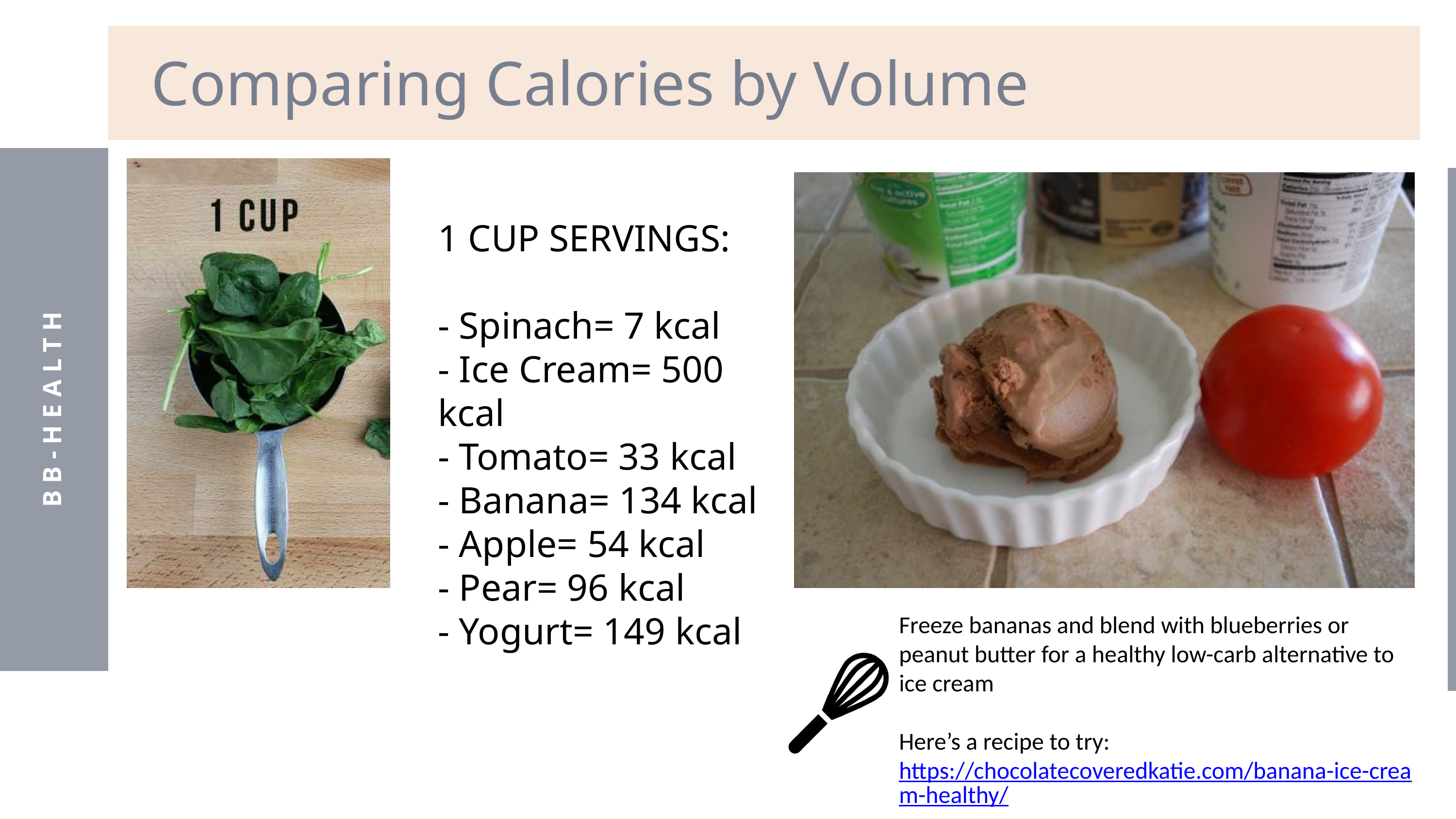

Comparing Calories by Volume
1 CUP SERVINGS:
- Spinach= 7 kcal
- Ice Cream= 500 kcal
- Tomato= 33 kcal
- Banana= 134 kcal
- Apple= 54 kcal
- Pear= 96 kcal
- Yogurt= 149 kcal
BB-HEALTH
Freeze bananas and blend with blueberries or peanut butter for a healthy low-carb alternative to ice cream
Here’s a recipe to try: https://chocolatecoveredkatie.com/banana-ice-cream-healthy/

## Slide 10
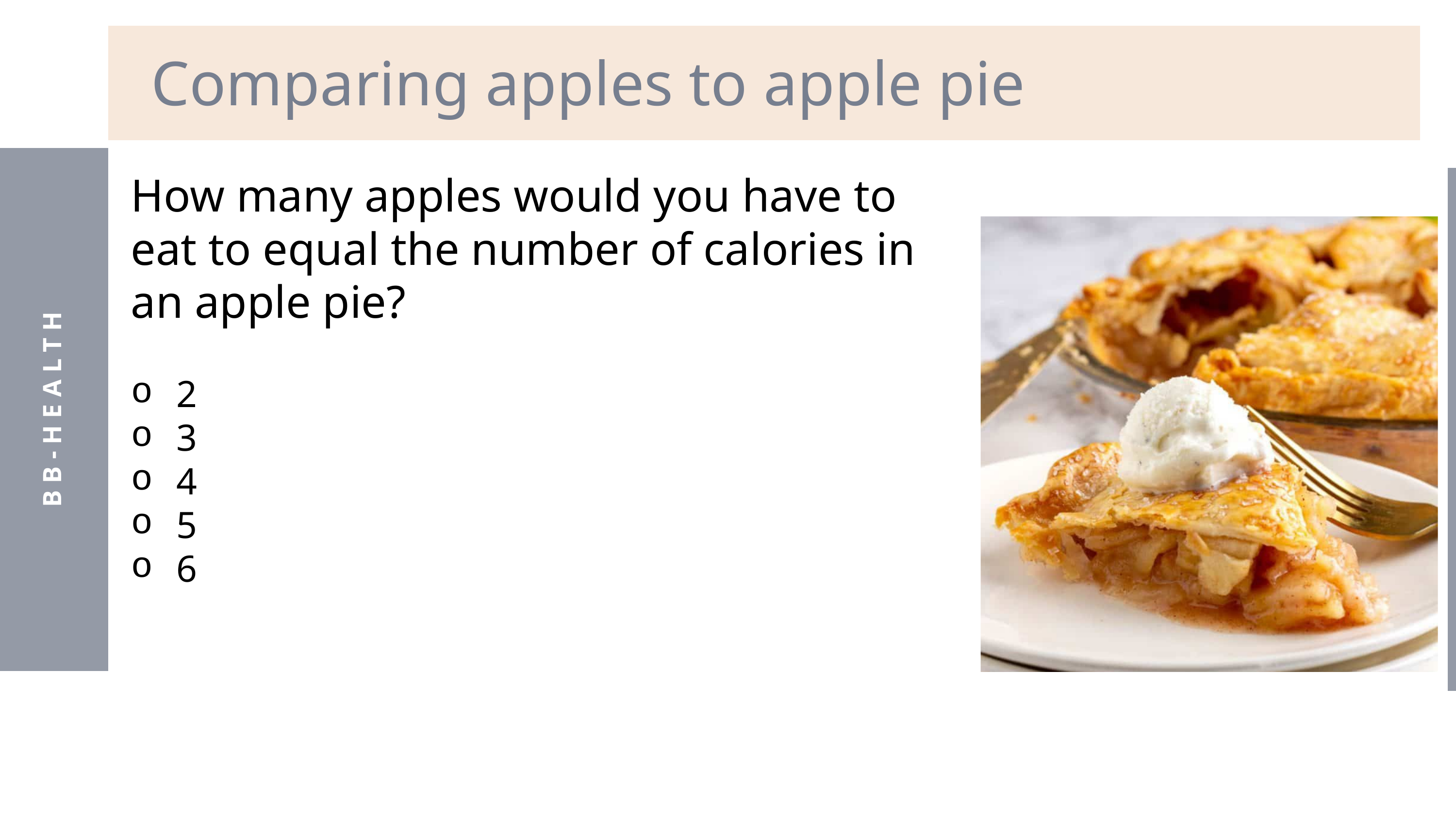

Comparing apples to apple pie
How many apples would you have to eat to equal the number of calories in an apple pie?
2
3
4
5
6
BB-HEALTH

## Slide 11
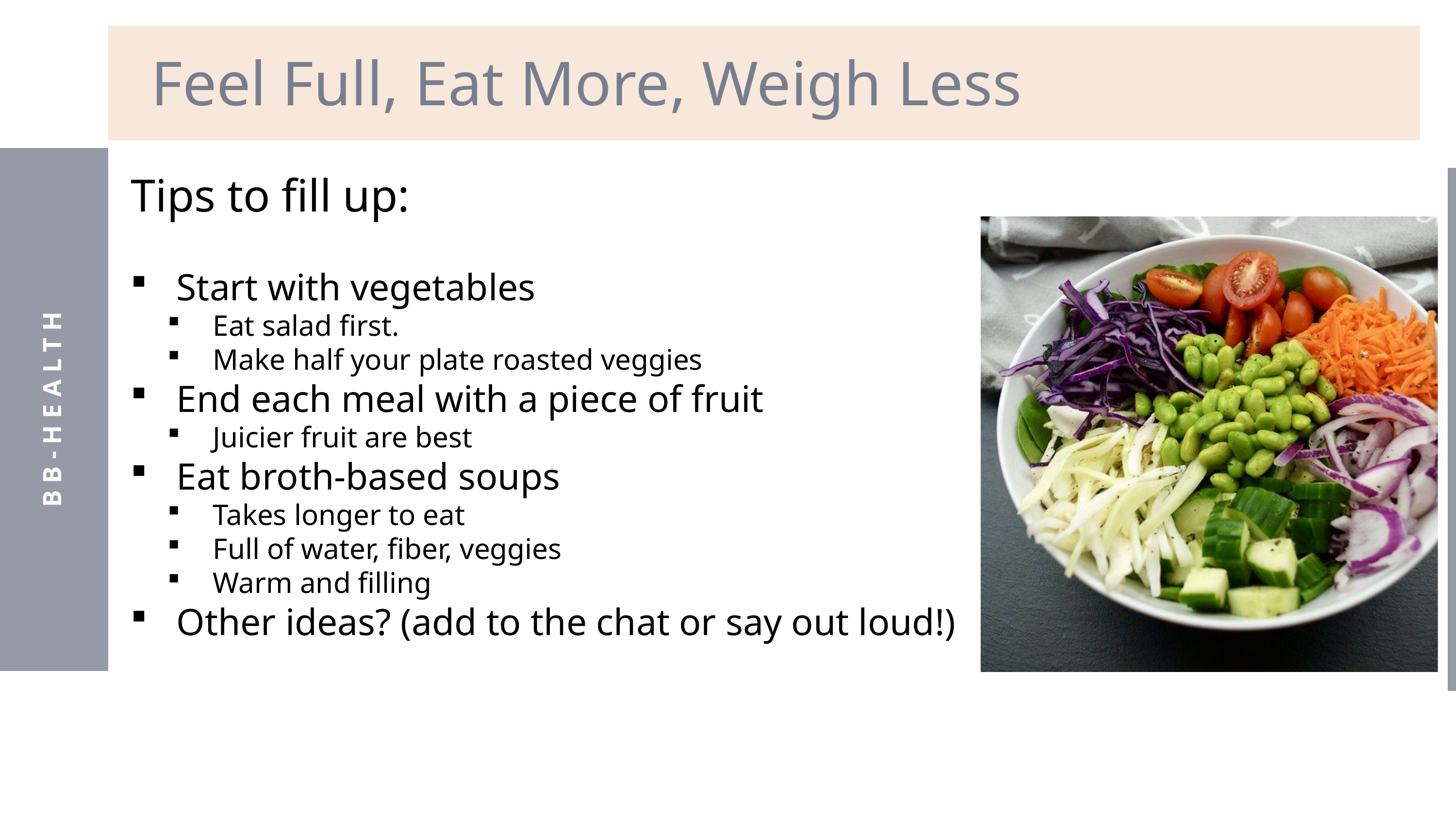

Feel Full, Eat More, Weigh Less
Tips to fill up:
Start with vegetables
Eat salad first.
Make half your plate roasted veggies
End each meal with a piece of fruit
Juicier fruit are best
Eat broth-based soups
Takes longer to eat
Full of water, fiber, veggies
Warm and filling
Other ideas? (add to the chat or say out loud!)
BB-HEALTH

## Slide 12
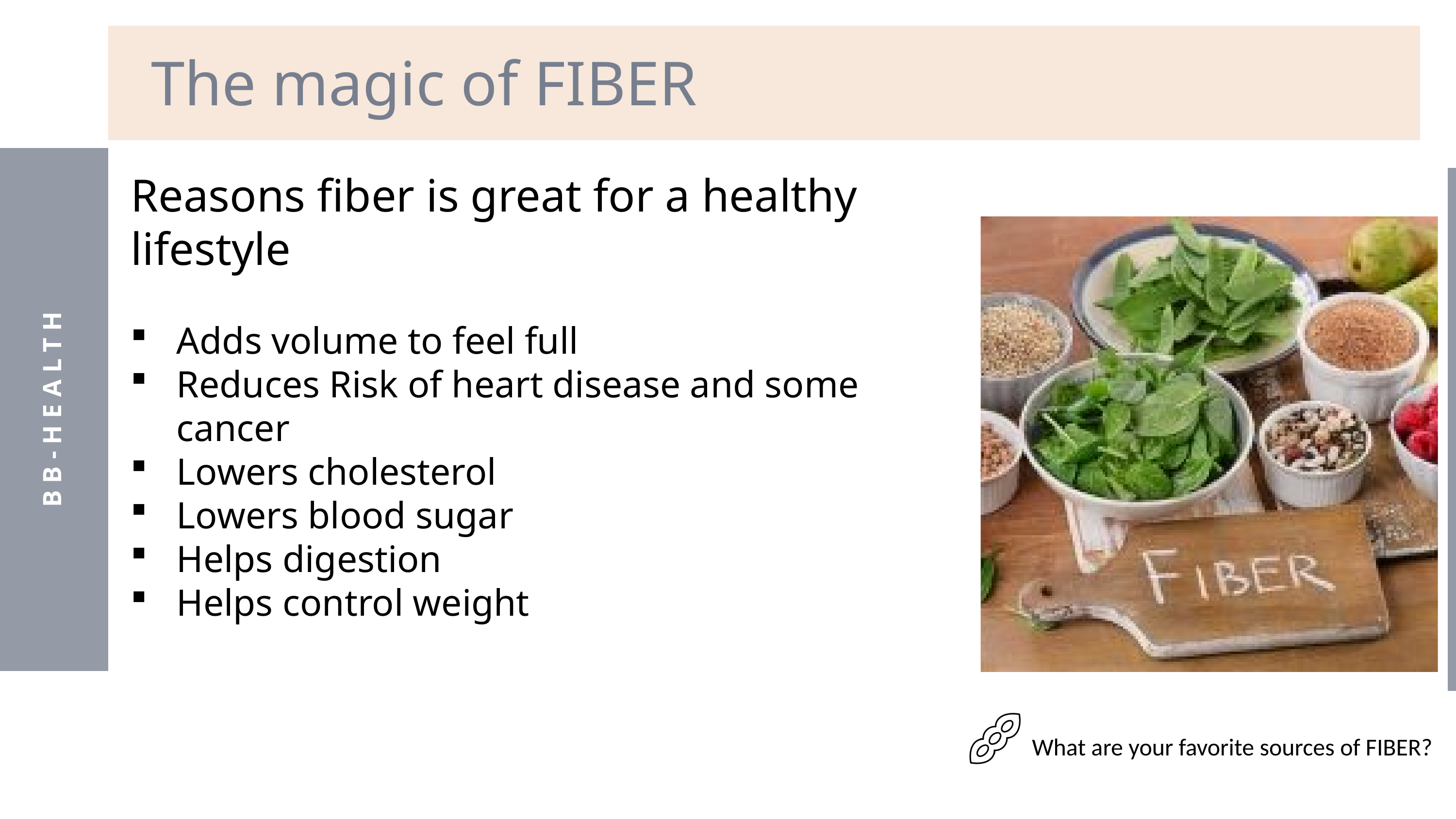

The magic of FIBER
Reasons fiber is great for a healthy lifestyle
Adds volume to feel full
Reduces Risk of heart disease and some cancer
Lowers cholesterol
Lowers blood sugar
Helps digestion
Helps control weight
BB-HEALTH
What are your favorite sources of FIBER?

## Slide 13
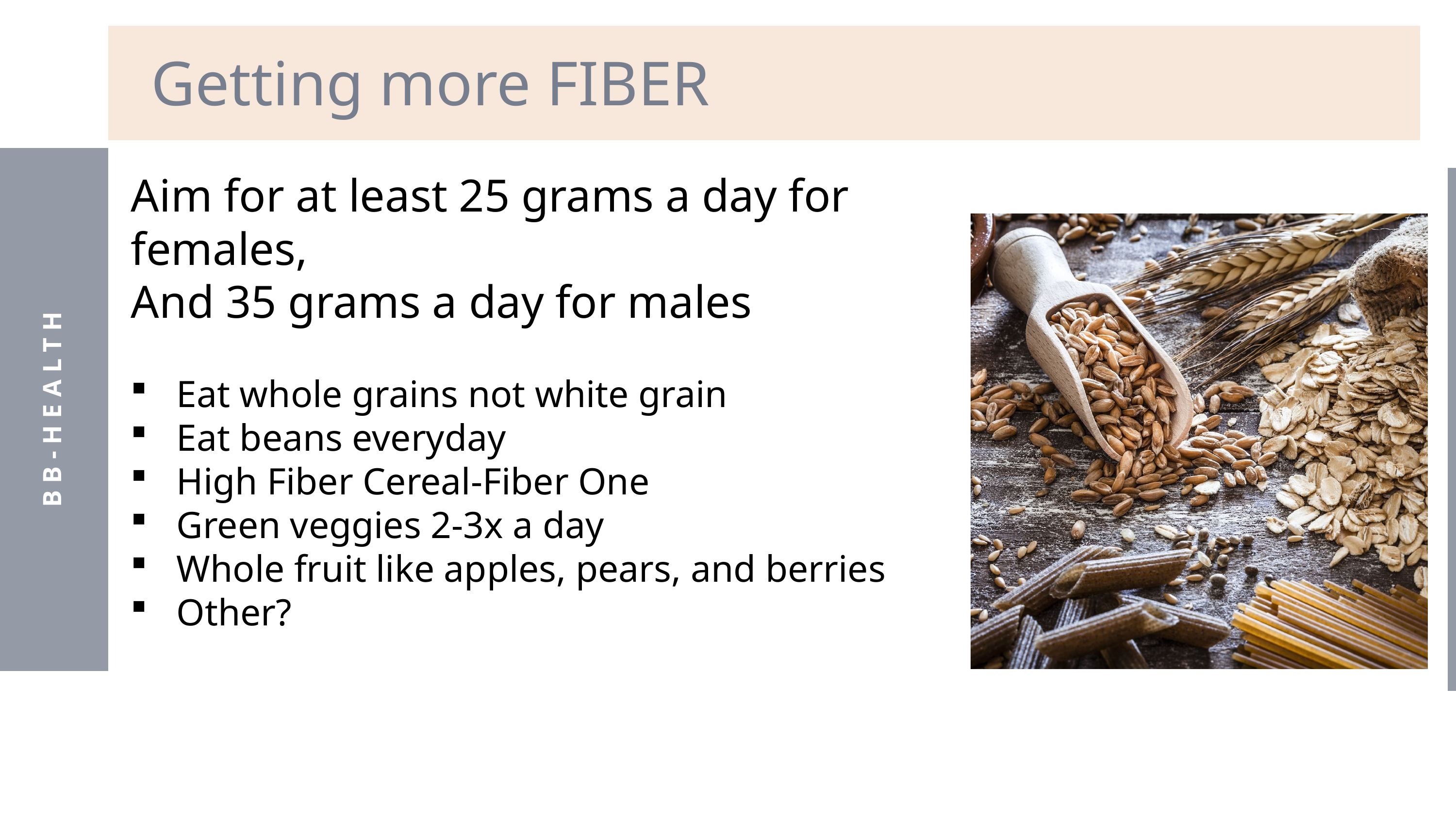

Getting more FIBER
Aim for at least 25 grams a day for females,
And 35 grams a day for males
Eat whole grains not white grain
Eat beans everyday
High Fiber Cereal-Fiber One
Green veggies 2-3x a day
Whole fruit like apples, pears, and berries
Other?
BB-HEALTH

## Slide 14
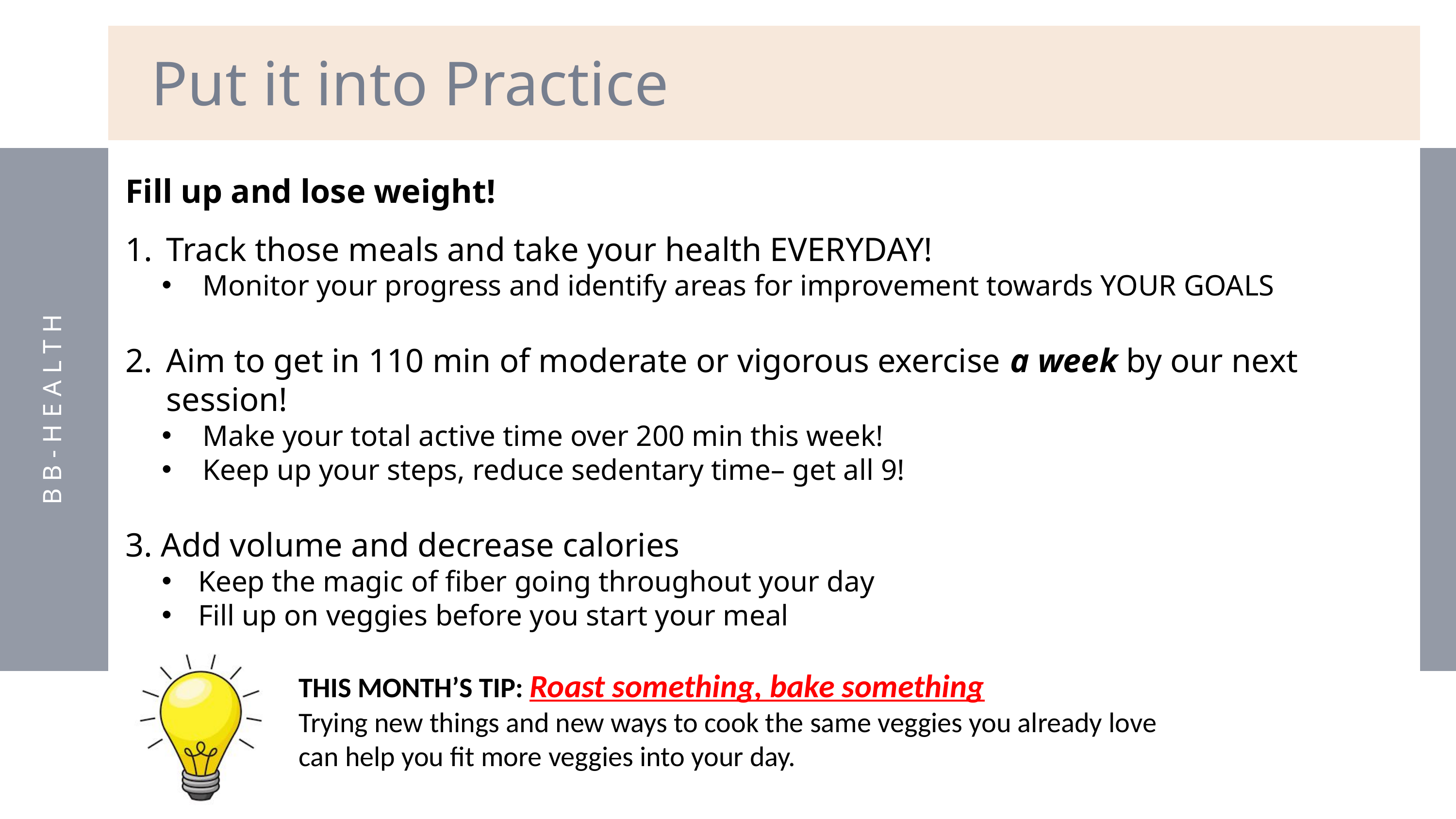

Put it into Practice
Fill up and lose weight!
Track those meals and take your health EVERYDAY!
Monitor your progress and identify areas for improvement towards YOUR GOALS
Aim to get in 110 min of moderate or vigorous exercise a week by our next session!
Make your total active time over 200 min this week!
Keep up your steps, reduce sedentary time– get all 9!
3. Add volume and decrease calories
Keep the magic of fiber going throughout your day
Fill up on veggies before you start your meal
BB-HEALTH
THIS MONTH’S TIP: Roast something, bake something
Trying new things and new ways to cook the same veggies you already love can help you fit more veggies into your day.

## Slide 15
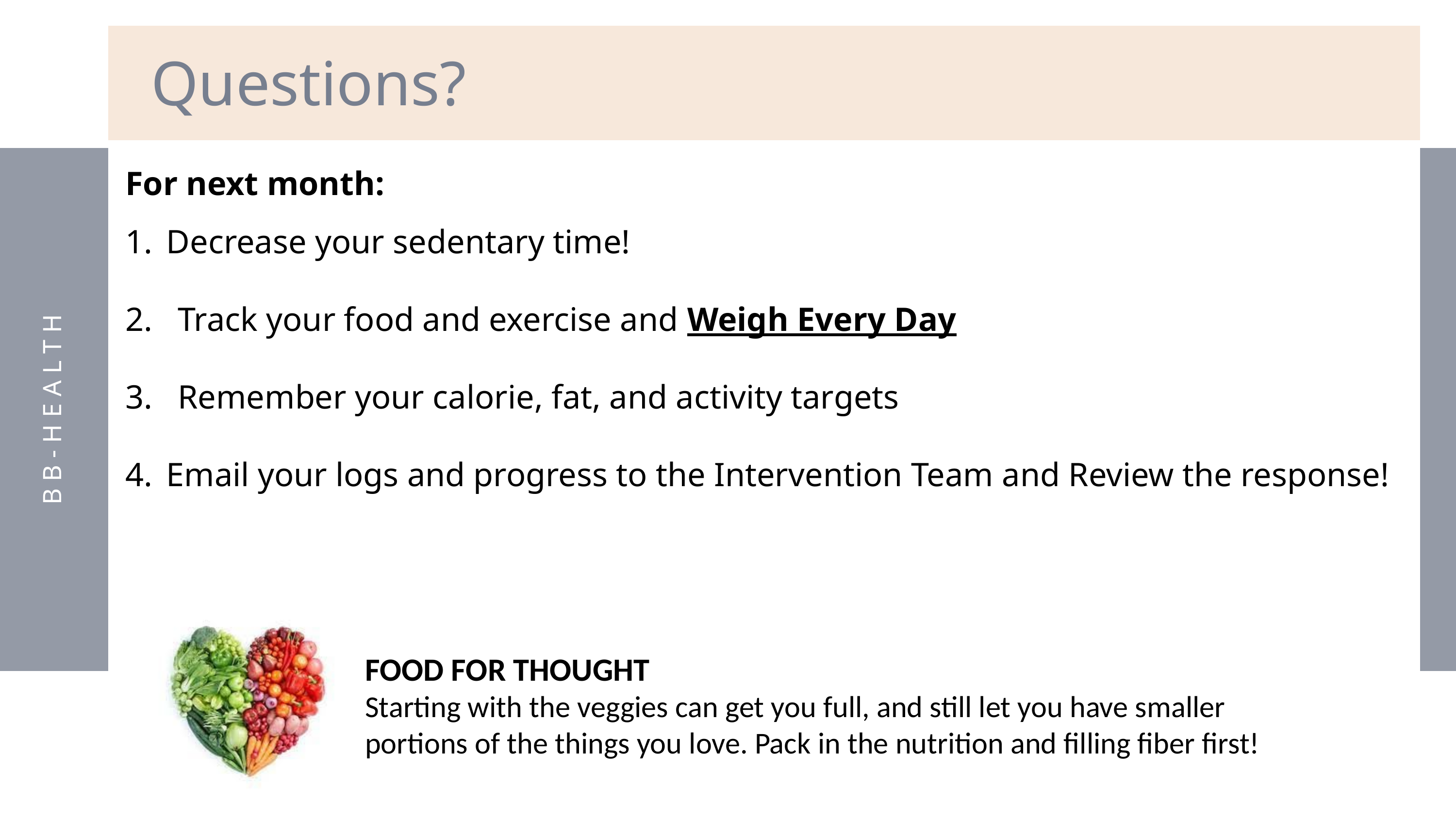

Questions?
For next month:
Decrease your sedentary time!
2. Track your food and exercise and Weigh Every Day
3. Remember your calorie, fat, and activity targets
Email your logs and progress to the Intervention Team and Review the response!
BB-HEALTH
FOOD FOR THOUGHT
Starting with the veggies can get you full, and still let you have smaller portions of the things you love. Pack in the nutrition and filling fiber first!
