# Supplementary material for: Development and Feasibility of an eHealth Diabetes Prevention Program Adapted for Older Adults—Results from a Randomized Control Pilot Study
Source: Nutrients. 2024 Mar 23;16(7):930. doi: 10.3390/nu16070930 (PMC11154527; doi:10.3390/nu16070930)
Supplement: Supplementary file 1 [file nutrients-16-00930-s001.zip › Session18_SUPP.pdf]

BB-HEALTH

e-Healthy Older Adults Modification

Diabetes Prevention Program

Session #18
Turn up the VOLUME:
Eating More with Fewer Calories

Group Opening Discussion

WELCOME BACK! <u>**HOW WAS LAST MONTH**</u>?

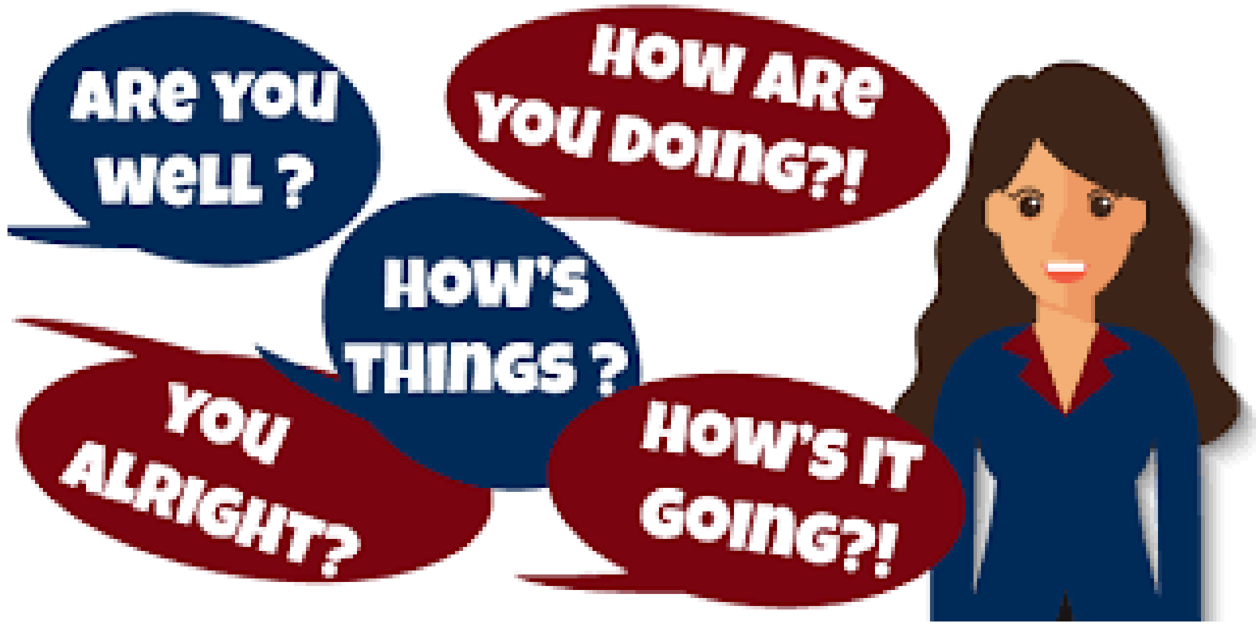

1. Did you track every meal?
2. Did you weigh every day, & record your health today?
3. Did you have many TABS and SUPER TABS?
4. Did you decrease your sedentary time?
5. Did you email the Intervention Team and review the response?
6. What were your unique challenges and successes?

REMINDER: CHARGE YOUR FITBIT & SCALE DURING THIS MEETING!

Checking in with our goals

B B - H E A L T H

Is being healthy an important part of who you are? (select one)

- Yes
- No
- Sometimes

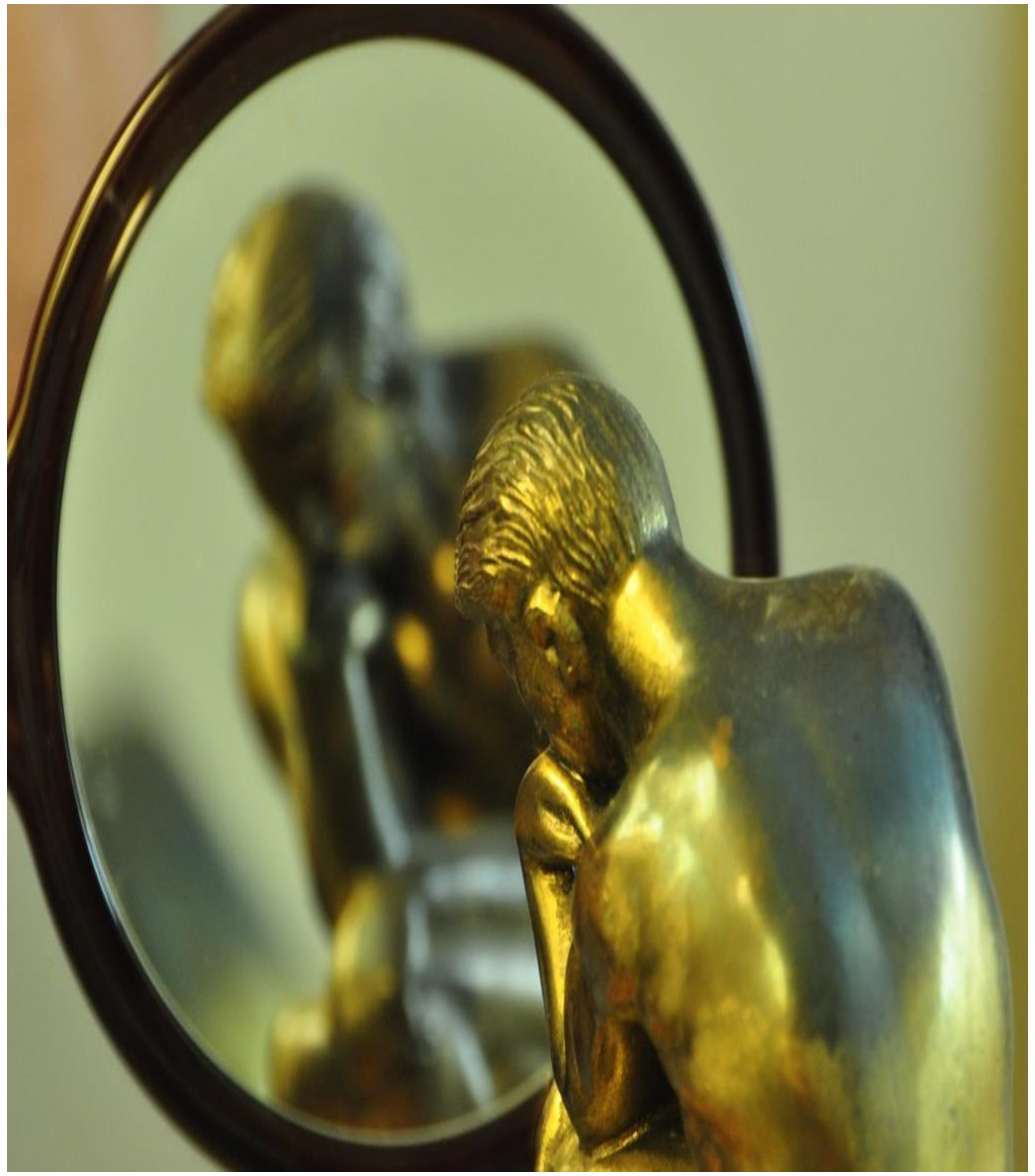

Checking in With Our Goals

BB-HEALTH

<u>Do you feel your health is improving? (select one)</u>

- My health is much worse
- My health is worse
- My health hasn’t improved
- My health is fluctuating
- My health has gotten better
- My health has gotten much better

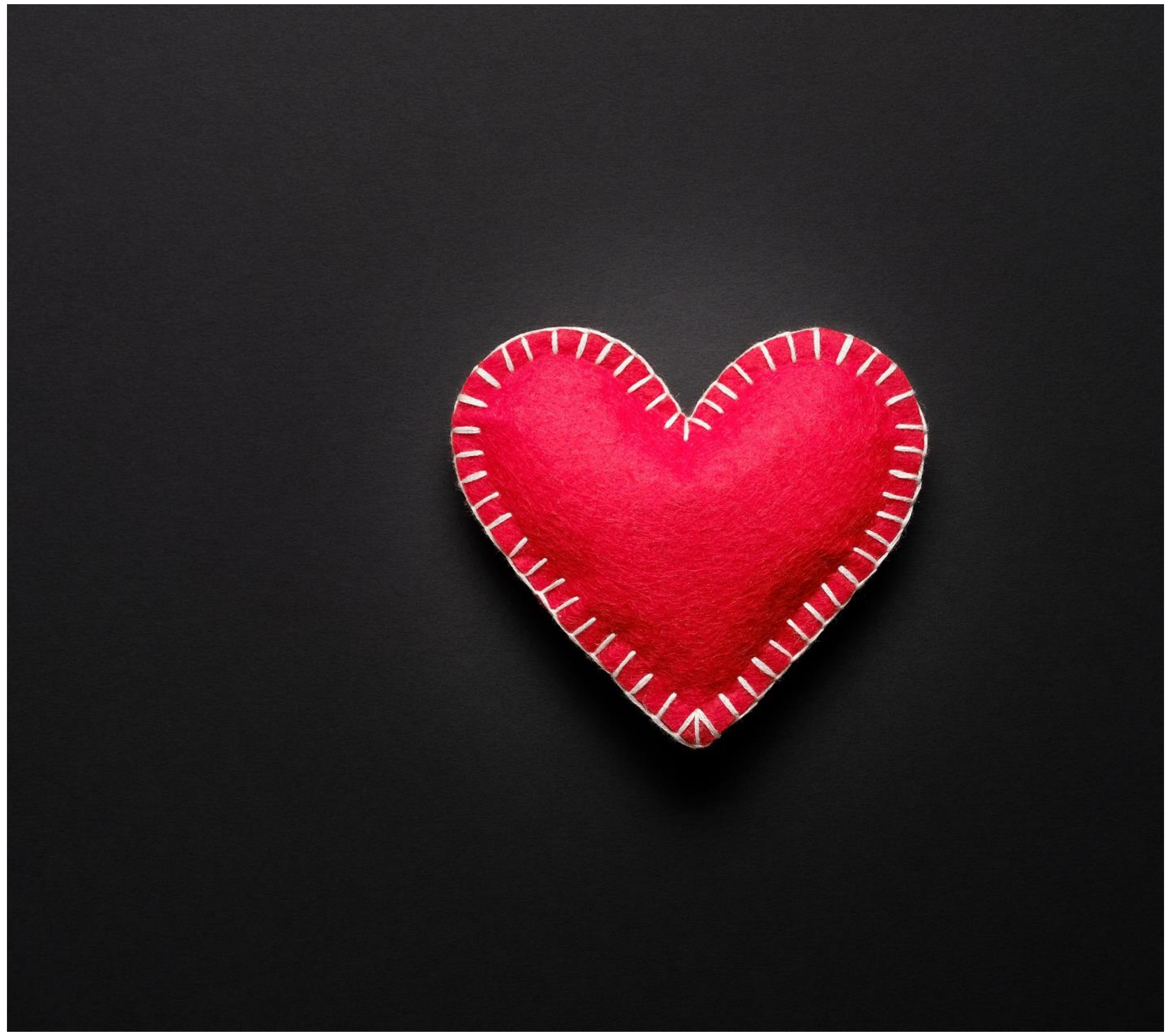

Checking in With Our Goals

BB-HEALTH

<u>Do you feel your weight is improving? (select one)</u>

- My weight is much worse
- My weight is worse
- My weight hasn’t improved
- My weight is fluctuating
- My weight has gotten better
- My weight has gotten much better

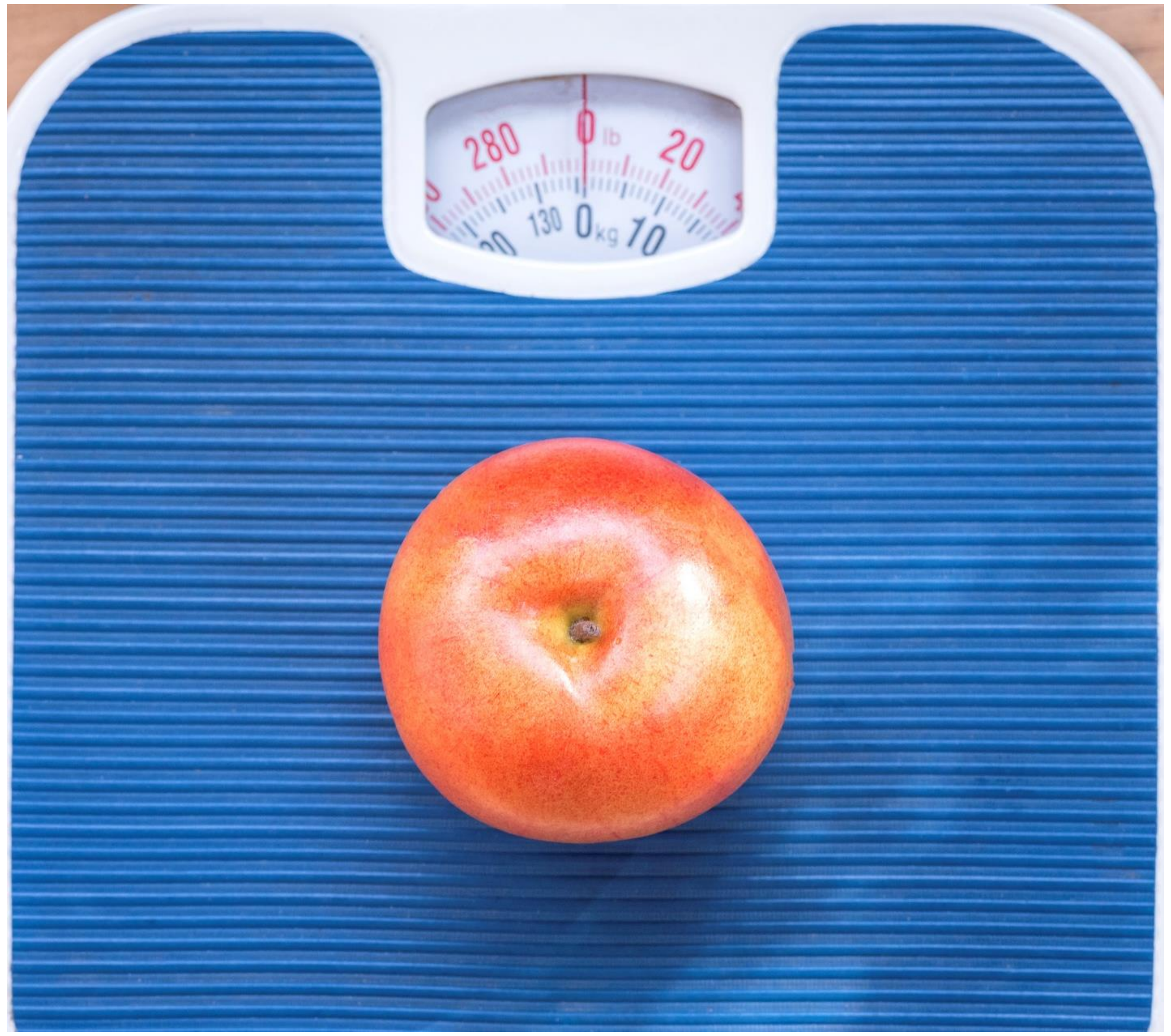

Checking in With Our Goals

<u>How strictly do you stick to your action plan? (Select one)</u>

- I always stick to my plan
- I mostly stick to my plan
- I stick to my plan about half the time
- I sometimes stick to my plan
- I don’t stick my plan often
- I rarely stick to my plan
- I never, or almost never, stick to my plan

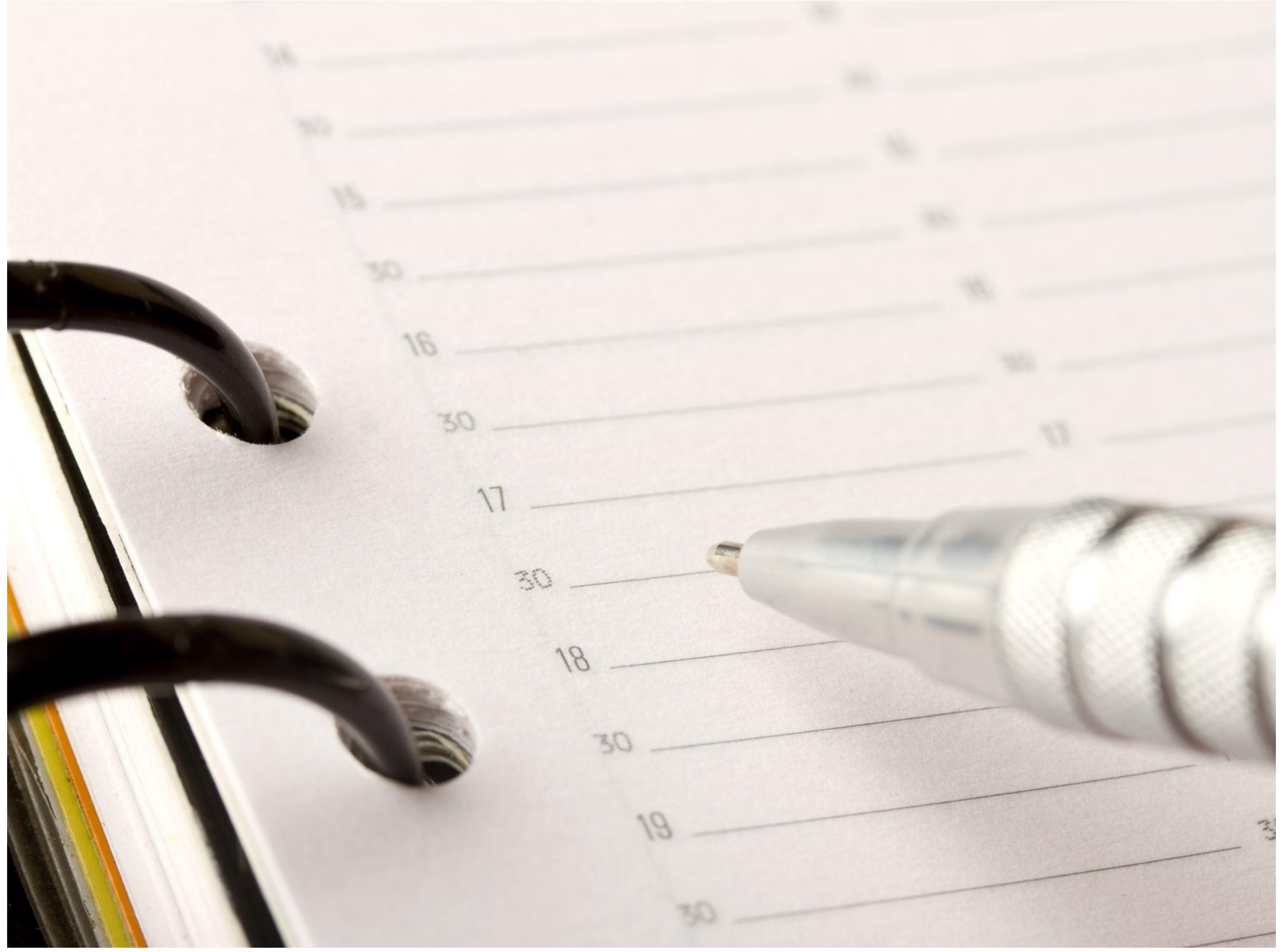

Checking in With Our Goals

<u>How important is it to you to keep trying to improve your health? (select one)</u>

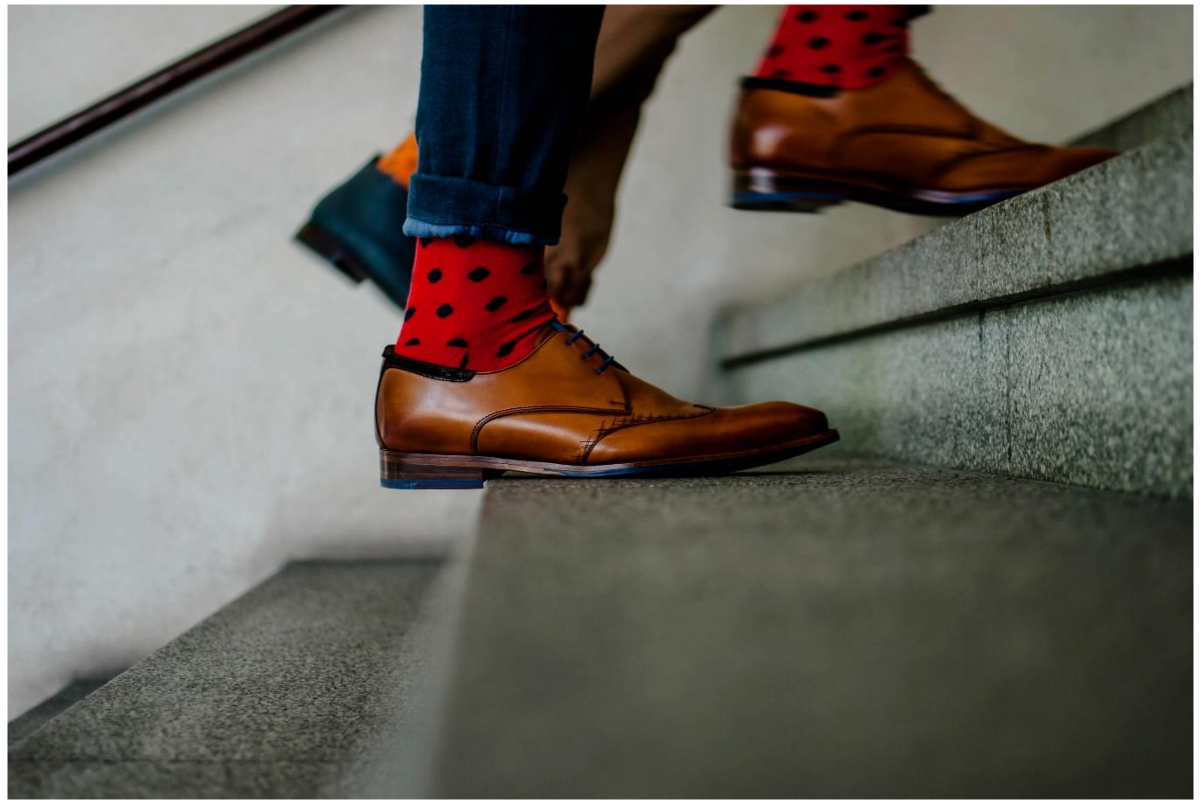

- Improving my health is my top priority
- Improving my health is very important
- Improving my health is important
- I want to improve my health, but it isn't a high priority
- I'm indifferent about improving my health
- I'm not interested in improving my health
- I am very uninterested in trying to improve my health

Volumetrics

BB-HEALTH

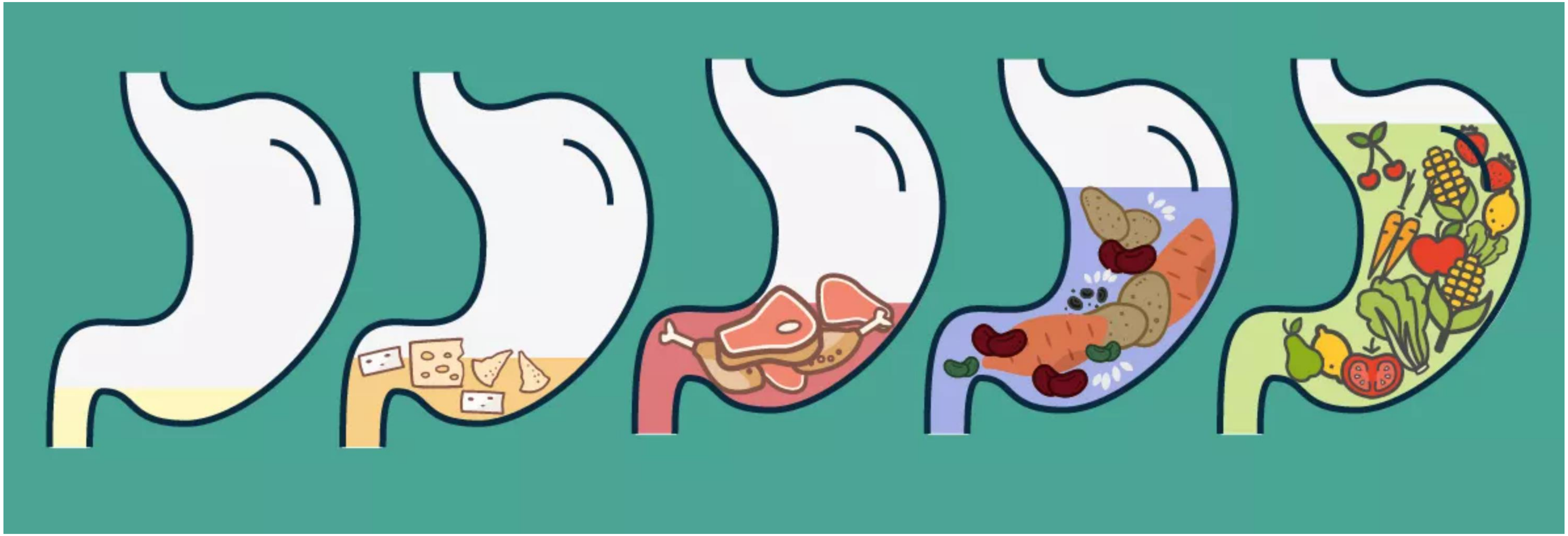

400 Calories<br>OIL	400 Calories<br>CHEESE	400 Calories<br>MEAT	400 Calories<br>POTATOES &<br>GRAINS	400 Calories<br>FRUIT &<br>VEGGIES

Comparing Calories by Volume

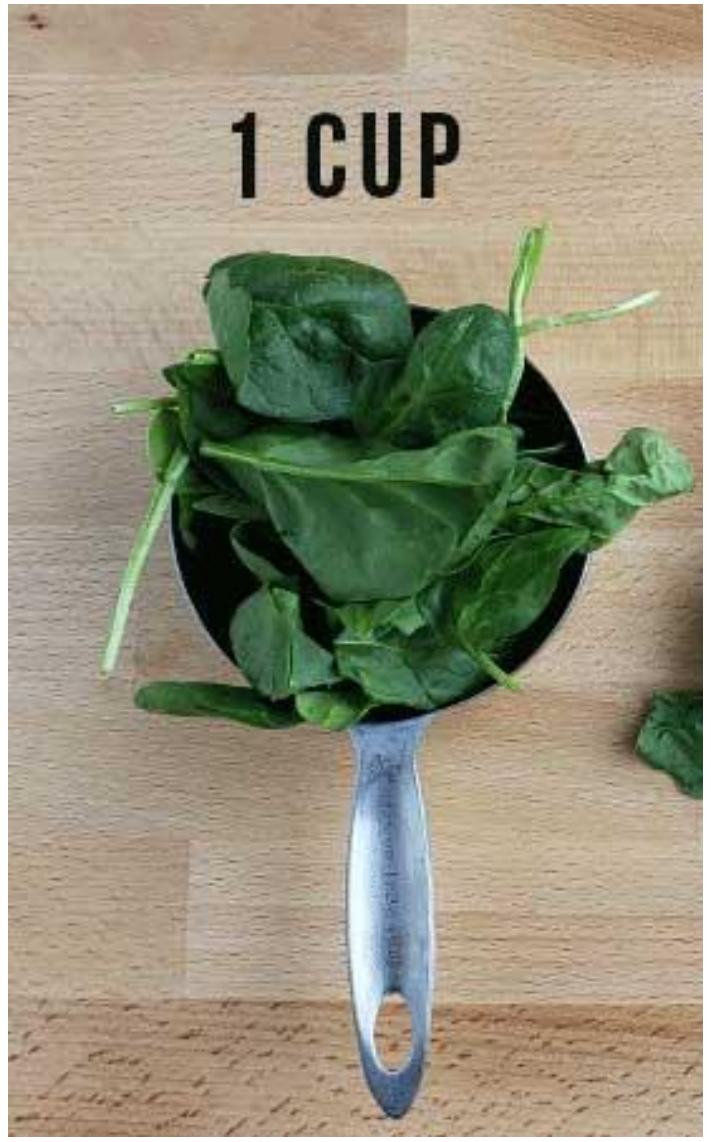

1 CUP SERVINGS:

- Spinach= 7 kcal
- Ice Cream= 500 kcal
- Tomato= 33 kcal
- Banana= 134 kcal
- Apple= 54 kcal
- Pear= 96 kcal
- Yogurt= 149 kcal

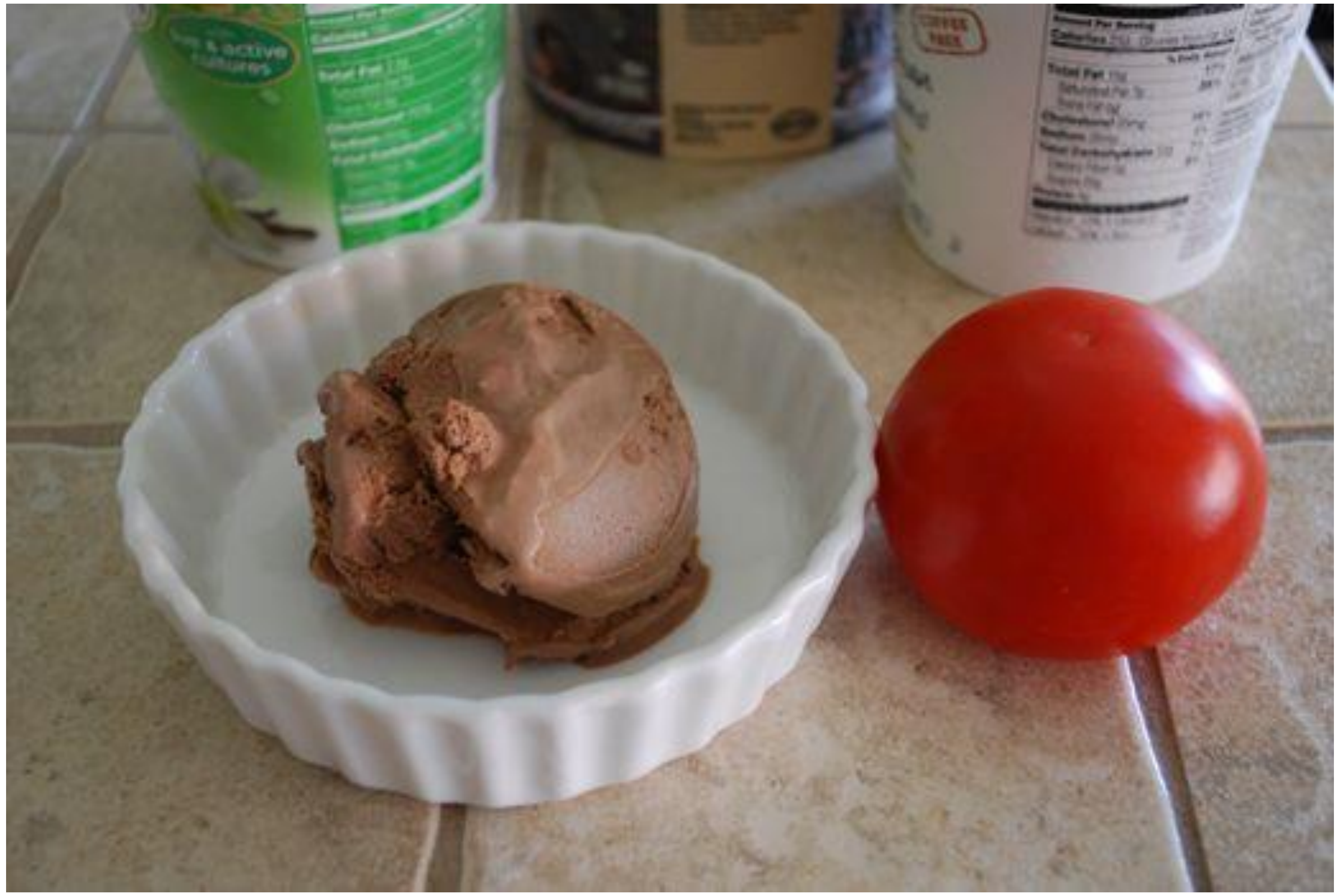

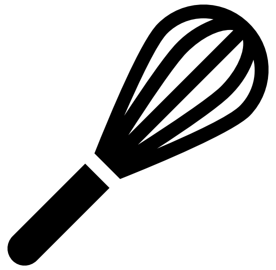

Freeze bananas and blend with blueberries or peanut butter for a healthy low-carb alternative to ice cream

Here's a recipe to try:
https://chocolatecoveredkatie.com/banana-ice-cream-healthy/

Comparing apples to apple pie

How many apples would you have to eat to equal the number of calories in an apple pie?

- ○ 2
- ○ 3
- ○ 4
- ○ 5
- ○ 6

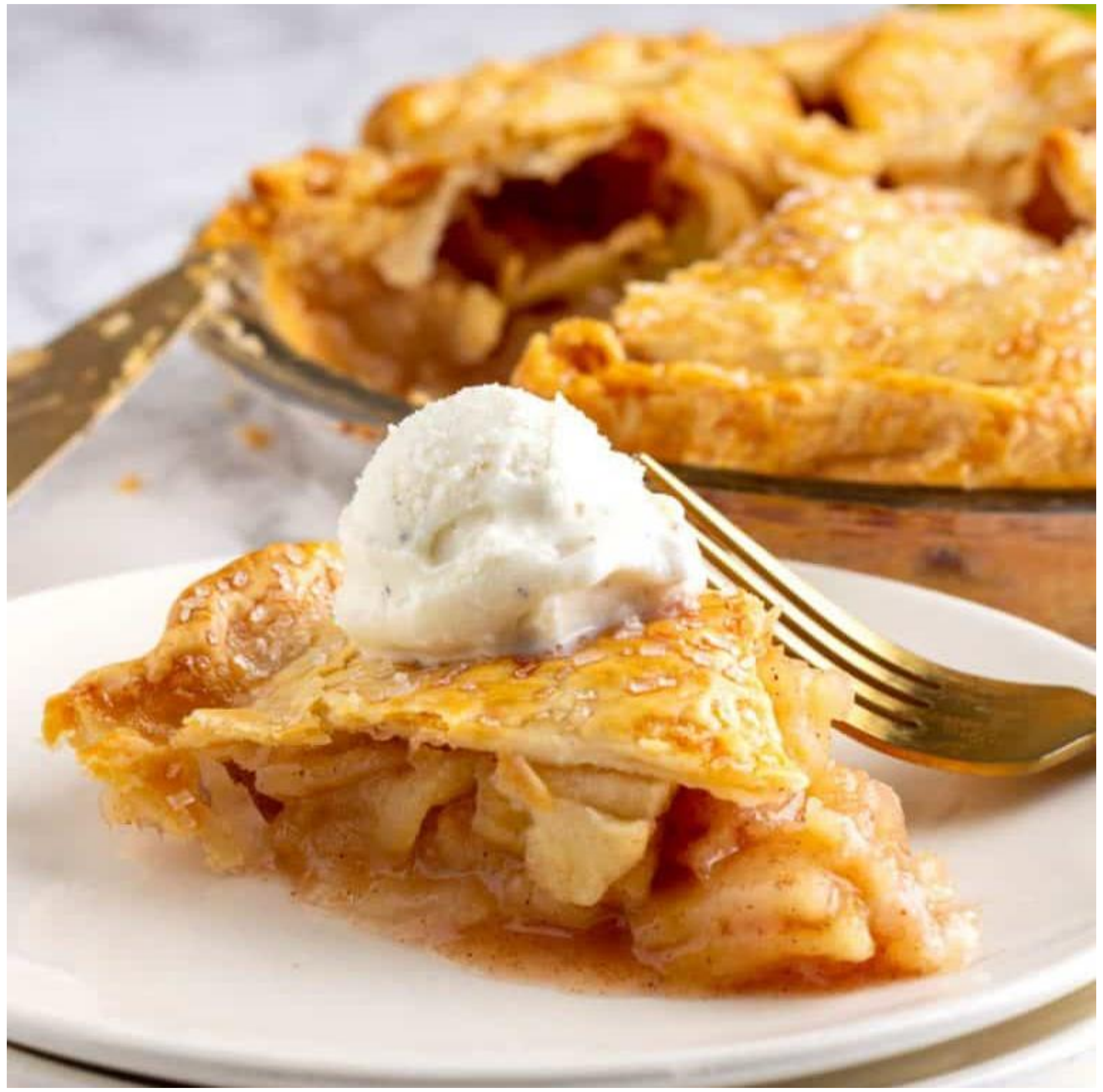

Feel Full, Eat More, Weigh Less

Tips to fill up:

- Start with vegetables
  - Eat salad first.
  - Make half your plate roasted veggies
- End each meal with a piece of fruit
  - Juicier fruit are best
- Eat broth-based soups
  - Takes longer to eat
  - Full of water, fiber, veggies
  - Warm and filling
- Other ideas? (add to the chat or say out loud!)

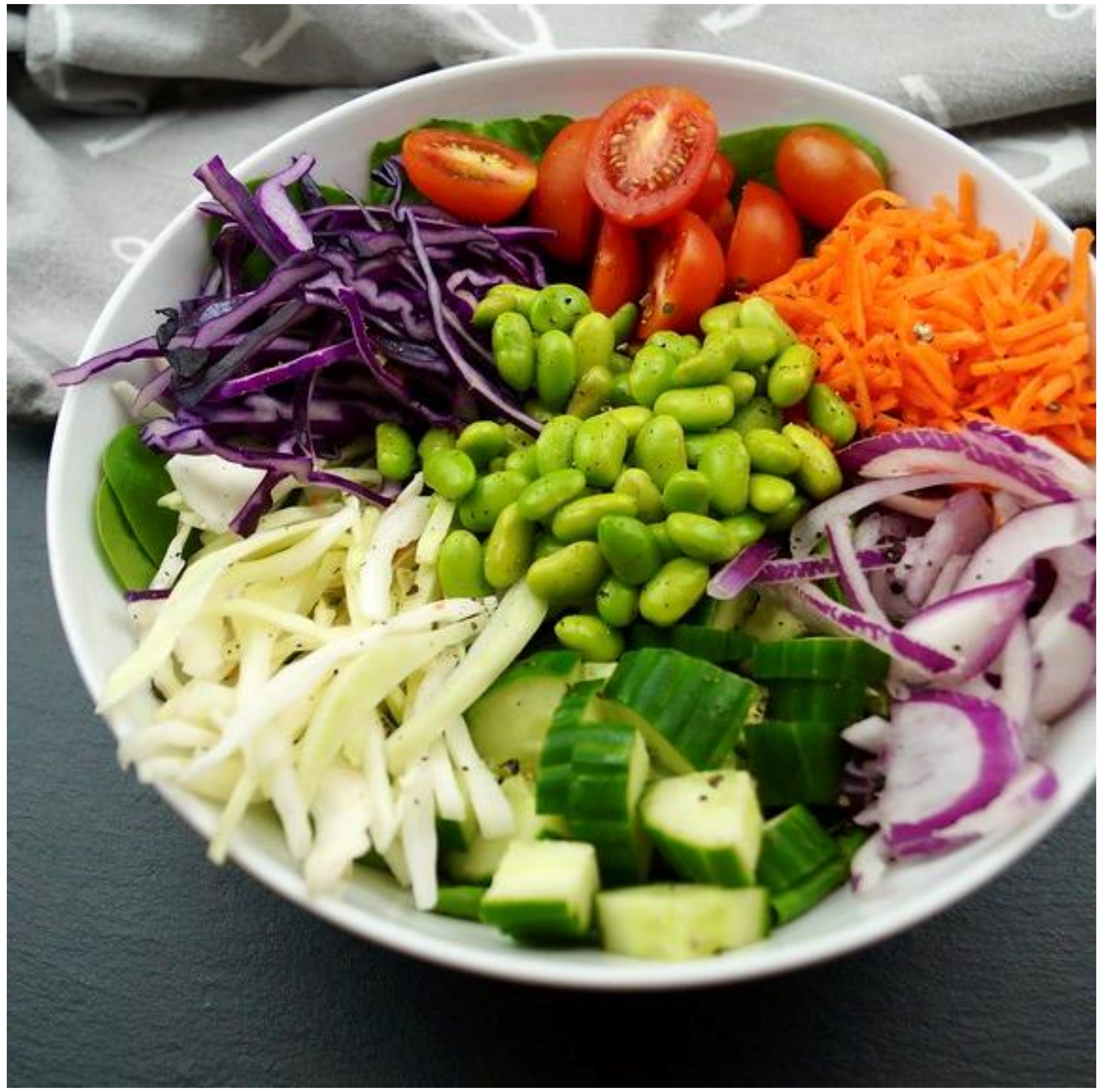

The magic of FIBER

BB-HEALTH

Reasons fiber is great for a healthy lifestyle

- Adds volume to feel full
- Reduces Risk of heart disease and some cancer
- Lowers cholesterol
- Lowers blood sugar
- Helps digestion
- Helps control weight

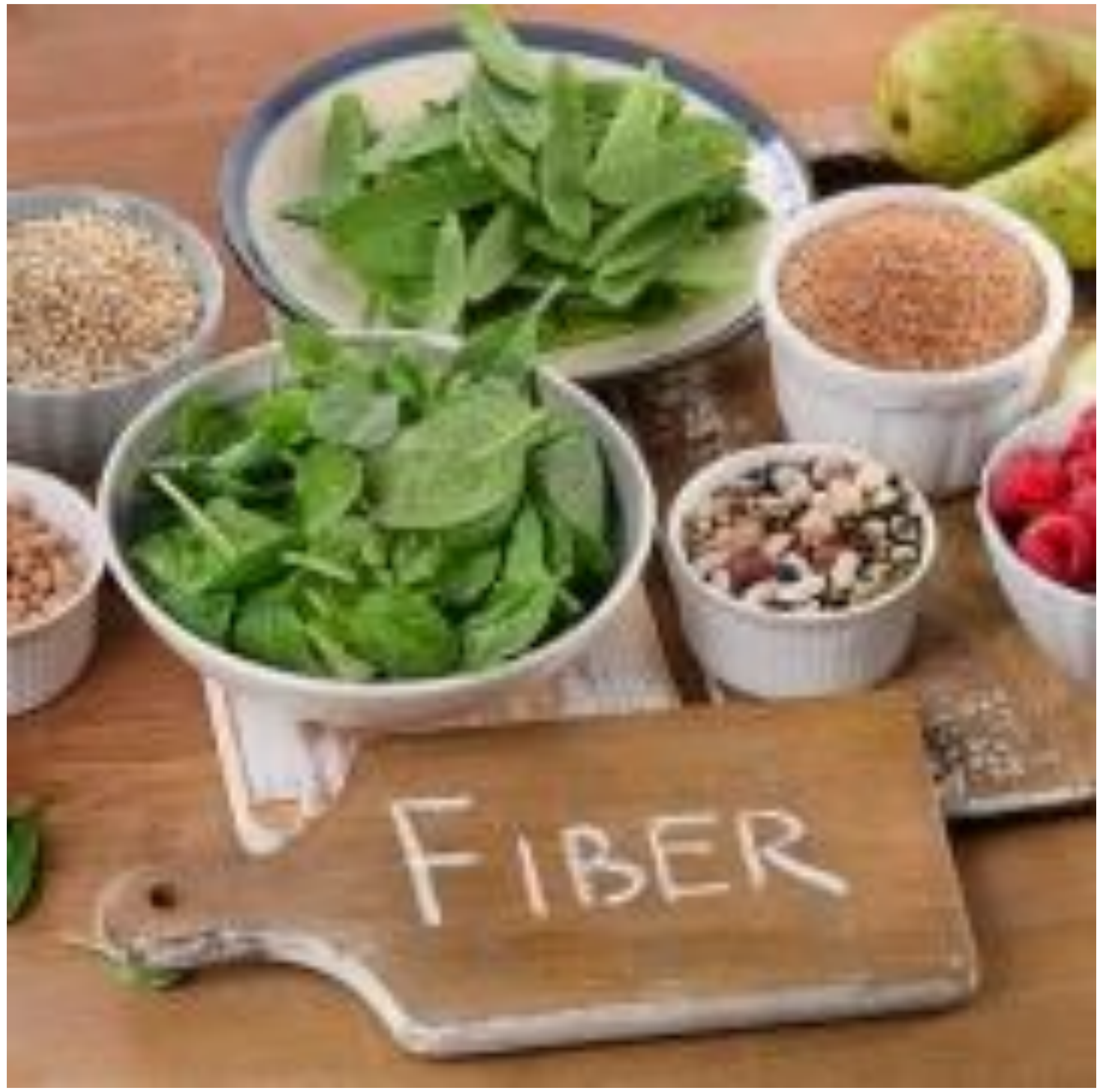

What are your favorite sources of FIBER?

Getting more FIBER

Aim for at least 25 grams a day for females,
And 35 grams a day for males

- Eat whole grains not white grain
- Eat beans everyday
- High Fiber Cereal-Fiber One
- Green veggies 2-3x a day
- Whole fruit like apples, pears, and berries
- Other?

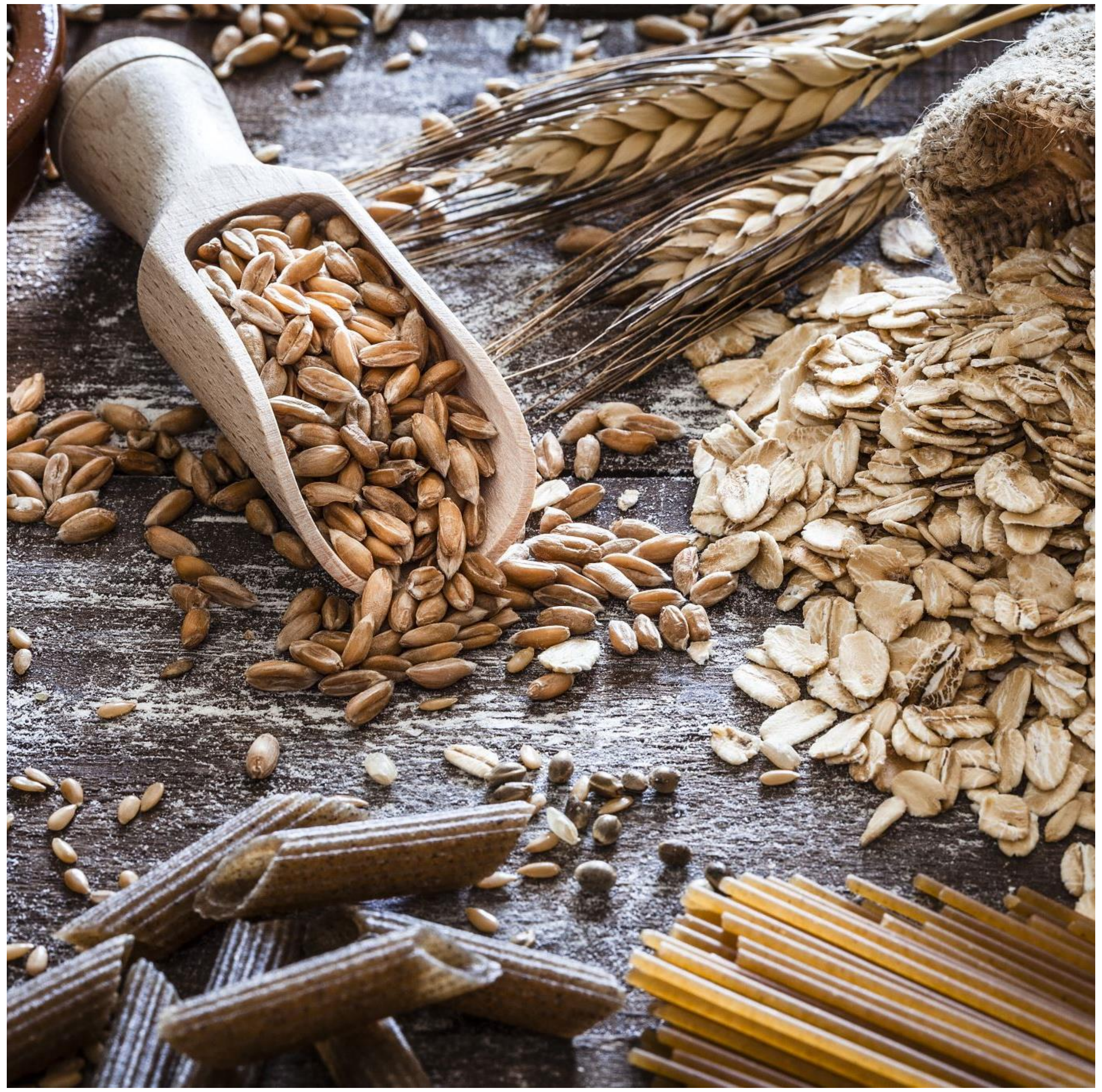

Put it into Practice

Fill up and lose weight!

1. Track those meals and take your health EVERYDAY!
    - Monitor your progress and identify areas for improvement towards YOUR GOALS

2. Aim to get in 110 min of moderate or vigorous exercise ***a week*** by our next session!
    - Make your total active time over 200 min this week!
    - Keep up your steps, reduce sedentary time– get all 9!

3. Add volume and decrease calories
    - Keep the magic of fiber going throughout your day
    - Fill up on veggies before you start your meal

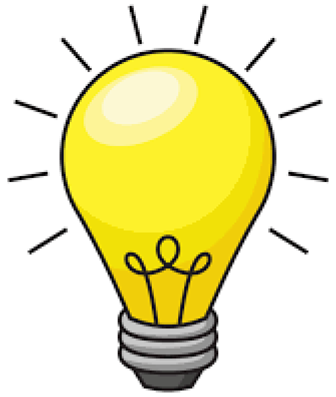

THIS MONTH'S TIP: *Roast something, bake something*
Trying new things and new ways to cook the same veggies you already love can help you fit more veggies into your day.

Questions?

For next month:

1. Decrease your sedentary time!
2. Track your food and exercise and **Weigh Every Day**
3. Remember your calorie, fat, and activity targets
4. Email your logs and progress to the Intervention Team and Review the response!

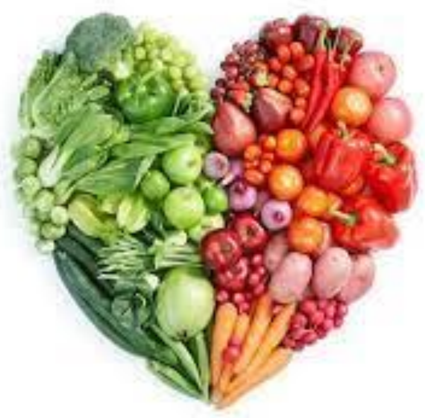

FOOD FOR THOUGHT
Starting with the veggies can get you full, and still let you have smaller portions of the things you love. Pack in the nutrition and filling fiber first!
