# Supplementary material for: Development and Feasibility of an eHealth Diabetes Prevention Program Adapted for Older Adults—Results from a Randomized Control Pilot Study
Source: Nutrients. 2024 Mar 23;16(7):930. doi: 10.3390/nu16070930 (PMC11154527; doi:10.3390/nu16070930)
Supplement: Supplementary file 1 [file nutrients-16-00930-s001.zip › Session19.pptx]

## Slide 1
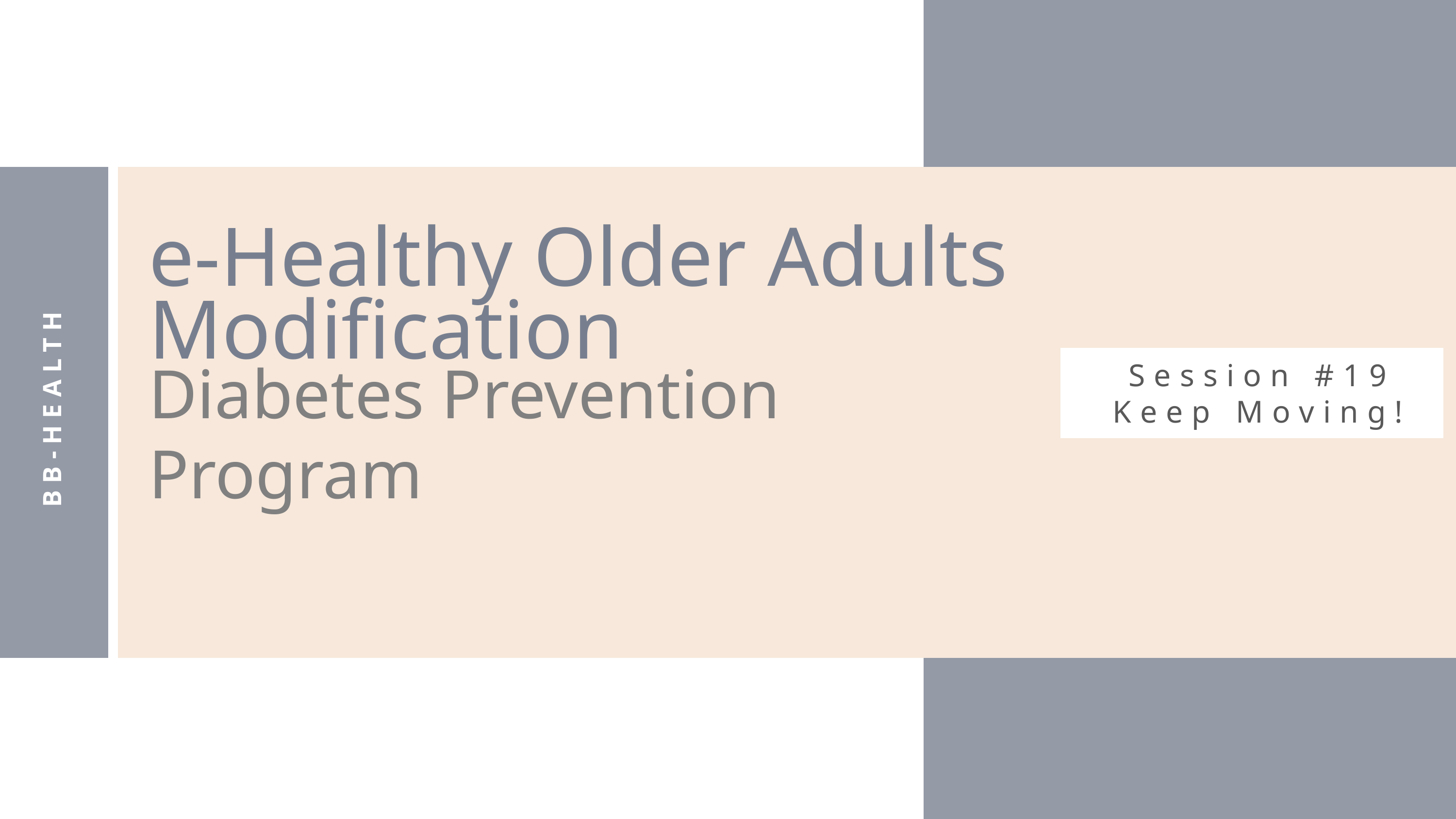

OPEN REPORTS
e-Healthy Older Adults Modification
Session #19
Keep Moving!
Diabetes Prevention Program
BB-HEALTH

## Slide 2
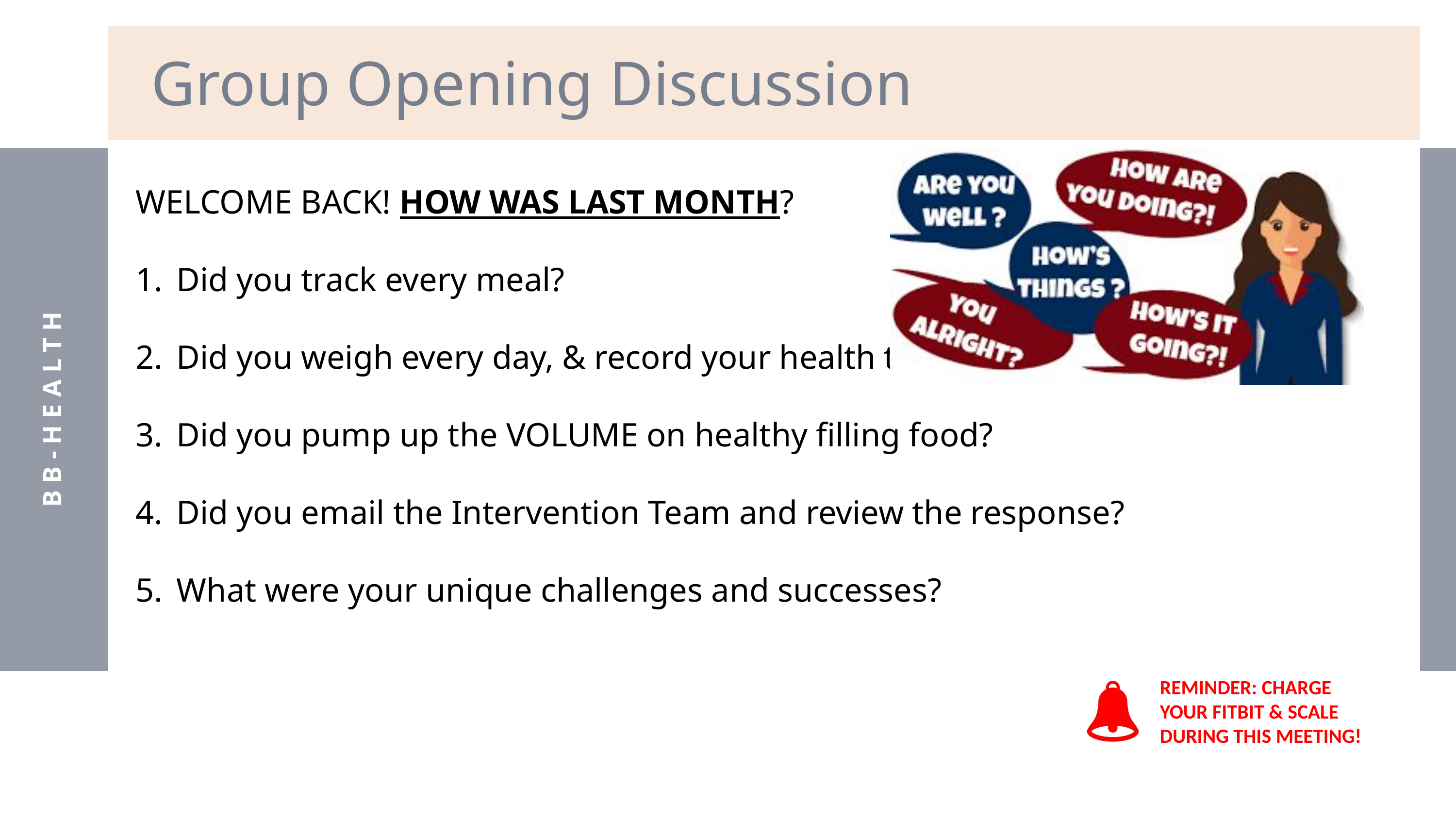

Group Opening Discussion
WELCOME BACK! HOW WAS LAST MONTH?
Did you track every meal?
Did you weigh every day, & record your health today?
Did you pump up the VOLUME on healthy filling food?
Did you email the Intervention Team and review the response?
What were your unique challenges and successes?
BB-HEALTH
REMINDER: CHARGE YOUR FITBIT & SCALE DURING THIS MEETING!

## Slide 3
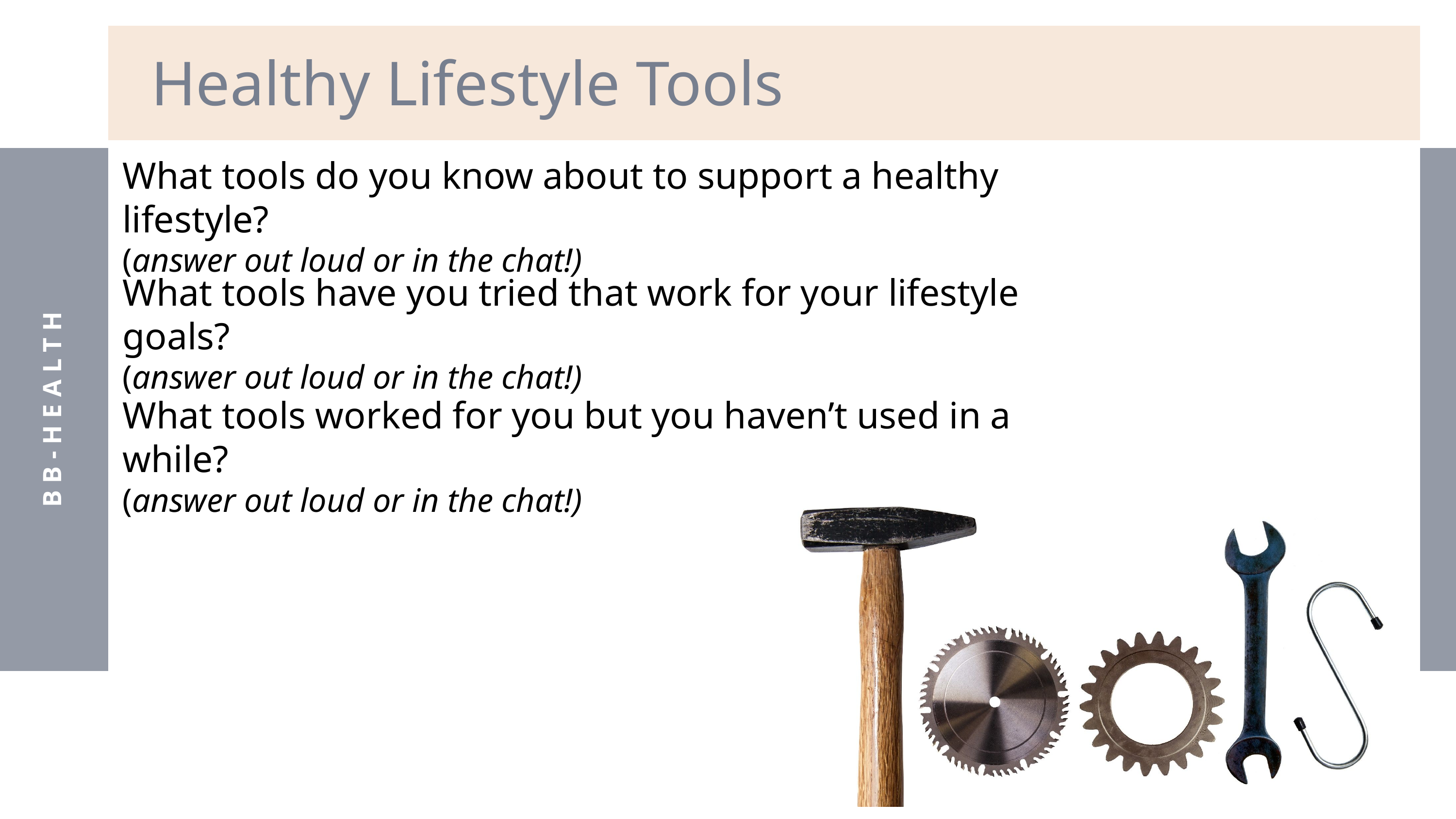

Healthy Lifestyle Tools
What tools do you know about to support a healthy lifestyle?
(answer out loud or in the chat!)
What tools have you tried that work for your lifestyle goals?
(answer out loud or in the chat!)
What tools worked for you but you haven’t used in a while?
(answer out loud or in the chat!)
BB-HEALTH

## Slide 4
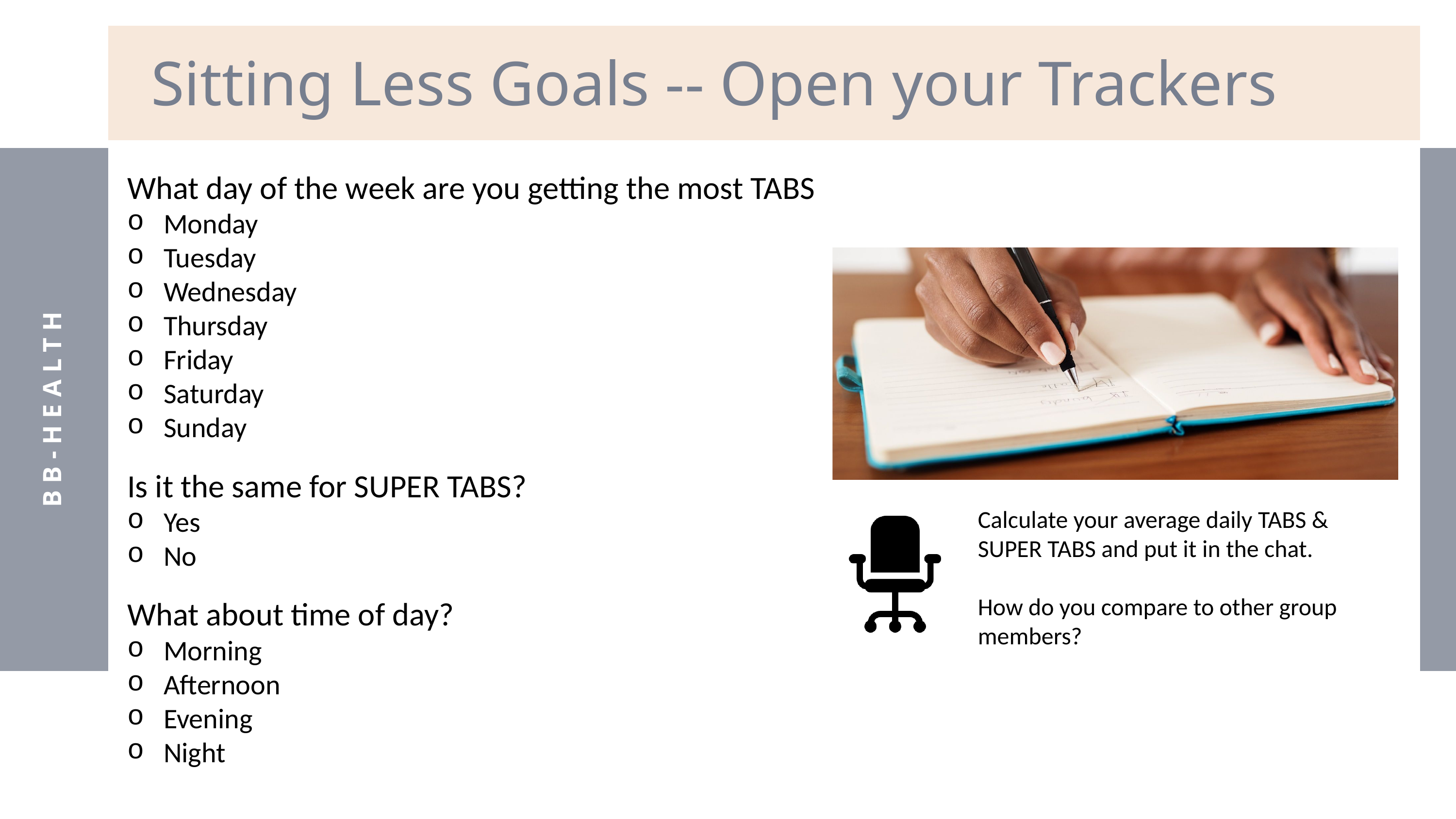

Sitting Less Goals -- Open your Trackers
What day of the week are you getting the most TABS
Monday
Tuesday
Wednesday
Thursday
Friday
Saturday
Sunday
Is it the same for SUPER TABS?
Yes
No
What about time of day?
Morning
Afternoon
Evening
Night
BB-HEALTH
Calculate your average daily TABS & SUPER TABS and put it in the chat.
How do you compare to other group members?

## Slide 5
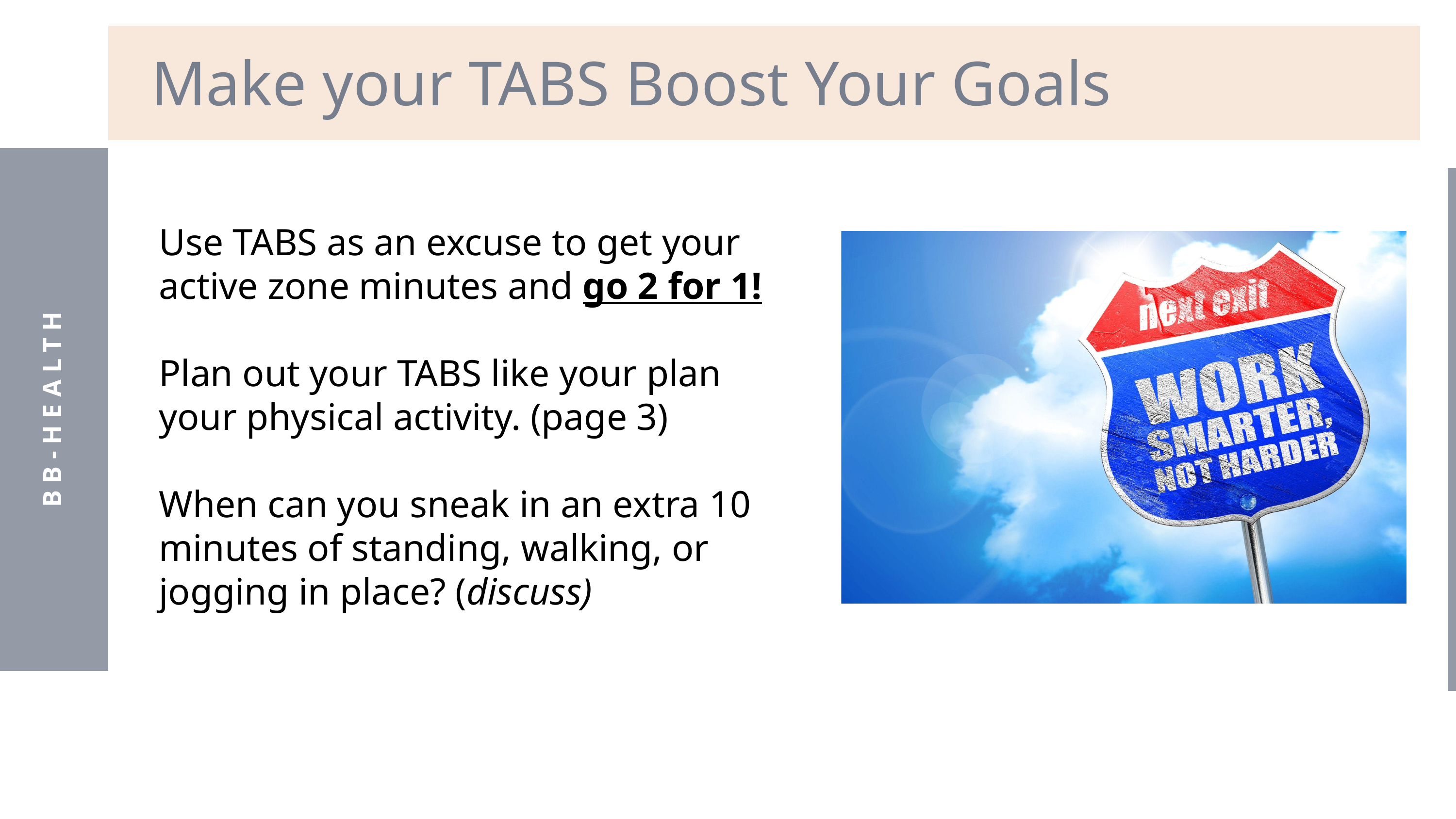

Make your TABS Boost Your Goals
Use TABS as an excuse to get your active zone minutes and go 2 for 1!
Plan out your TABS like your plan your physical activity. (page 3)
When can you sneak in an extra 10 minutes of standing, walking, or jogging in place? (discuss)
BB-HEALTH

## Slide 6
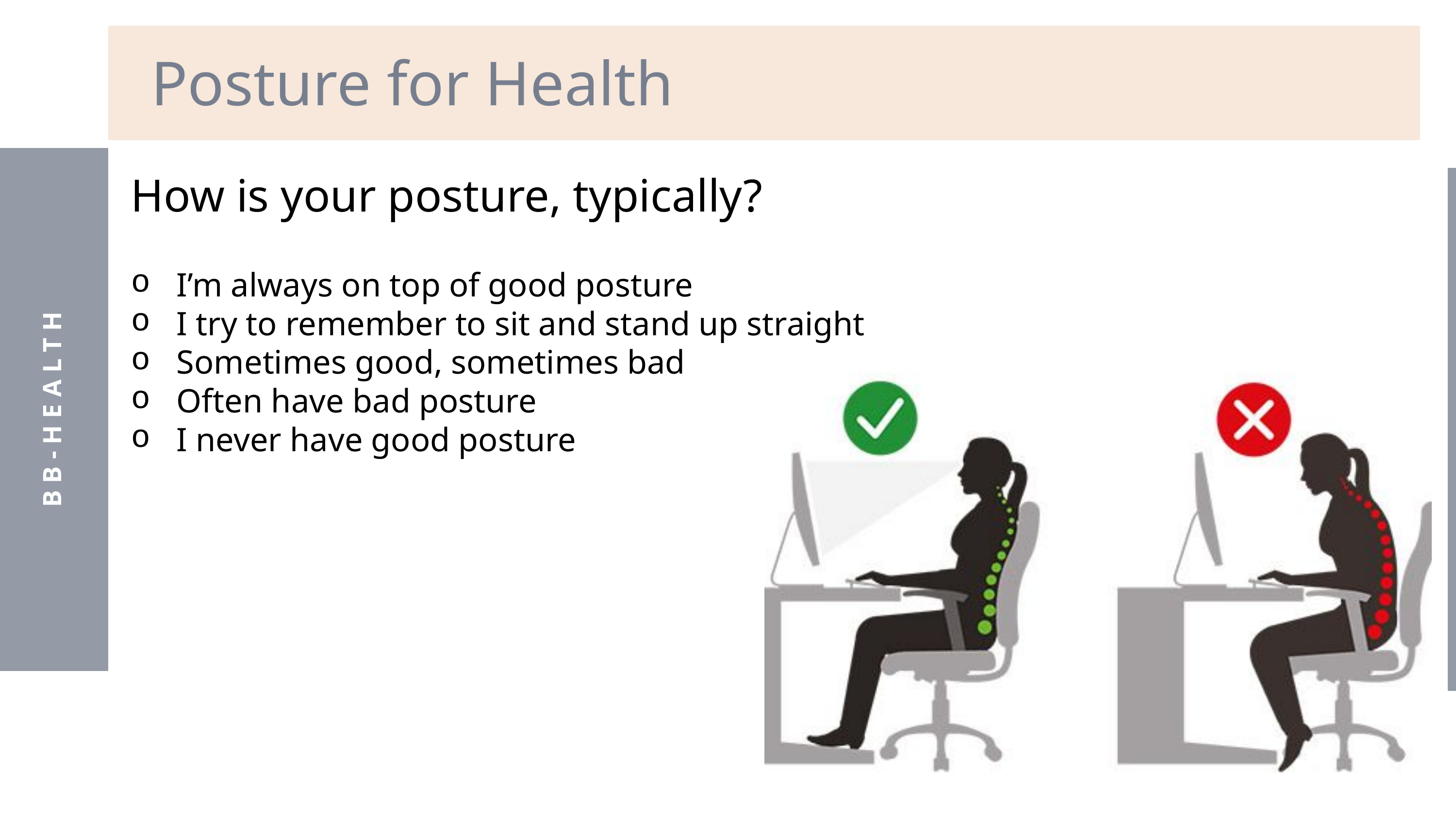

Posture for Health
How is your posture, typically?
I’m always on top of good posture
I try to remember to sit and stand up straight
Sometimes good, sometimes bad
Often have bad posture
I never have good posture
BB-HEALTH

## Slide 7
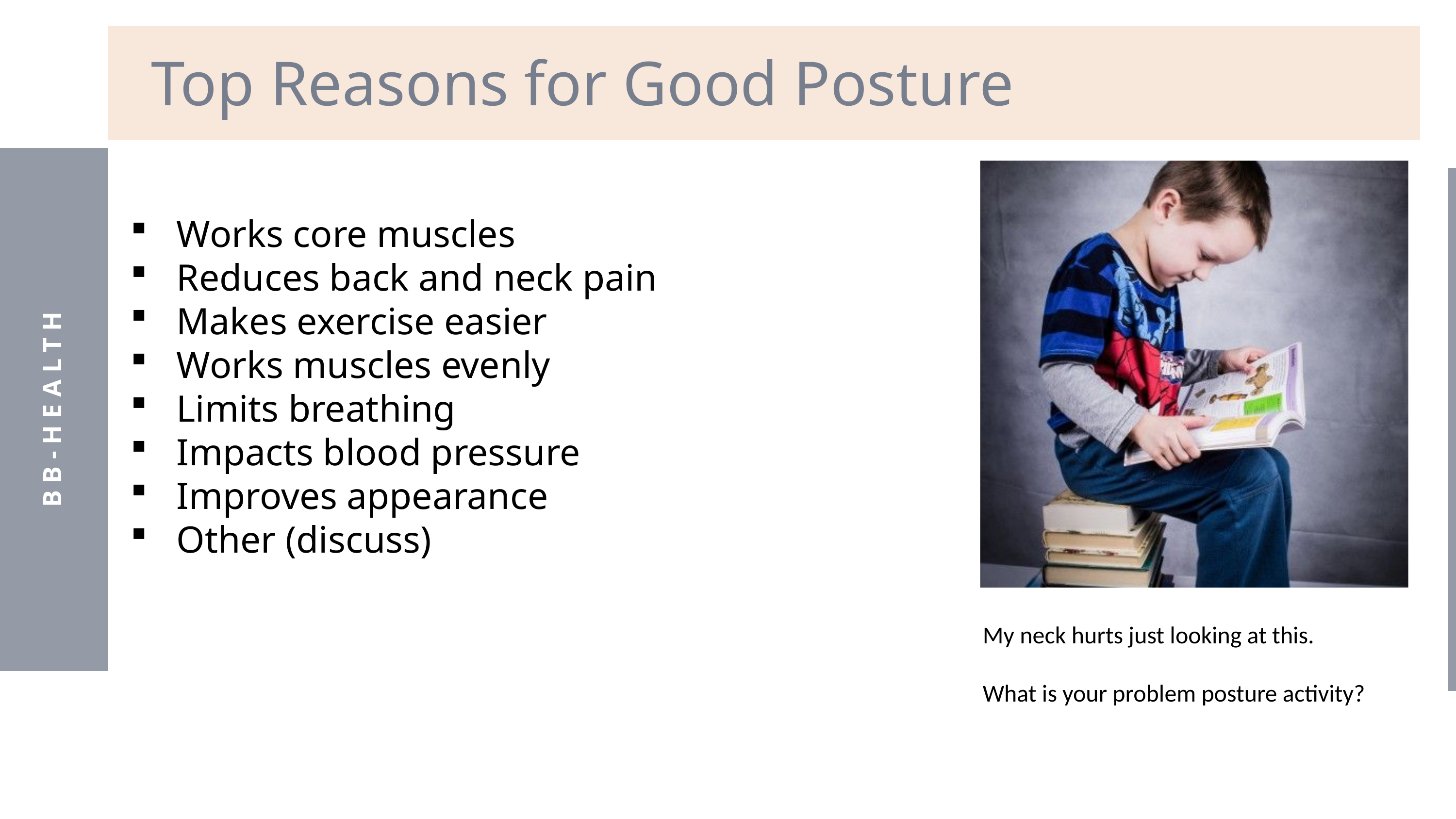

Top Reasons for Good Posture
Works core muscles
Reduces back and neck pain
Makes exercise easier
Works muscles evenly
Limits breathing
Impacts blood pressure
Improves appearance
Other (discuss)
BB-HEALTH
My neck hurts just looking at this.
What is your problem posture activity?

## Slide 8
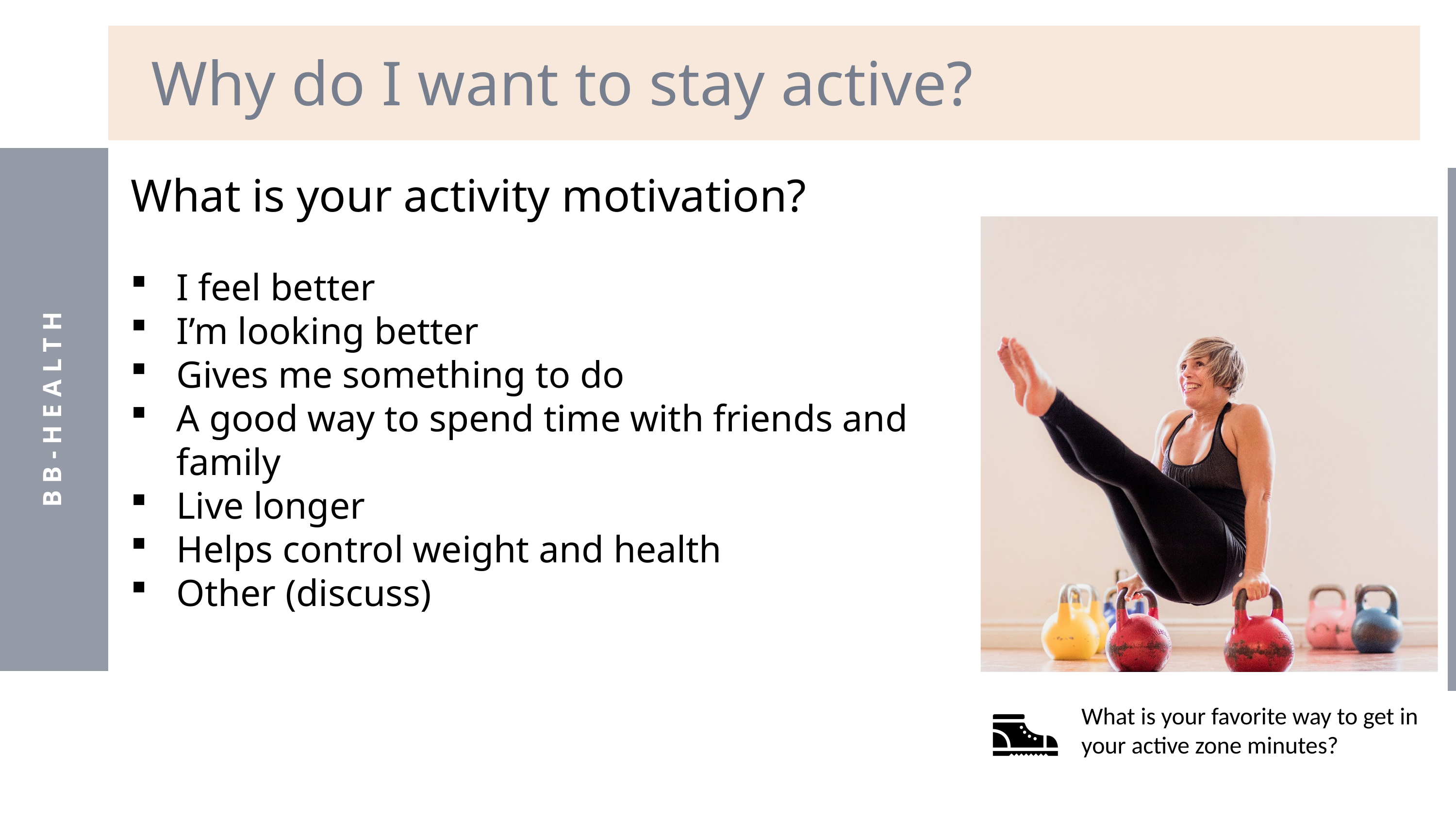

Why do I want to stay active?
What is your activity motivation?
I feel better
I’m looking better
Gives me something to do
A good way to spend time with friends and family
Live longer
Helps control weight and health
Other (discuss)
BB-HEALTH
What is your favorite way to get in your active zone minutes?

## Slide 9
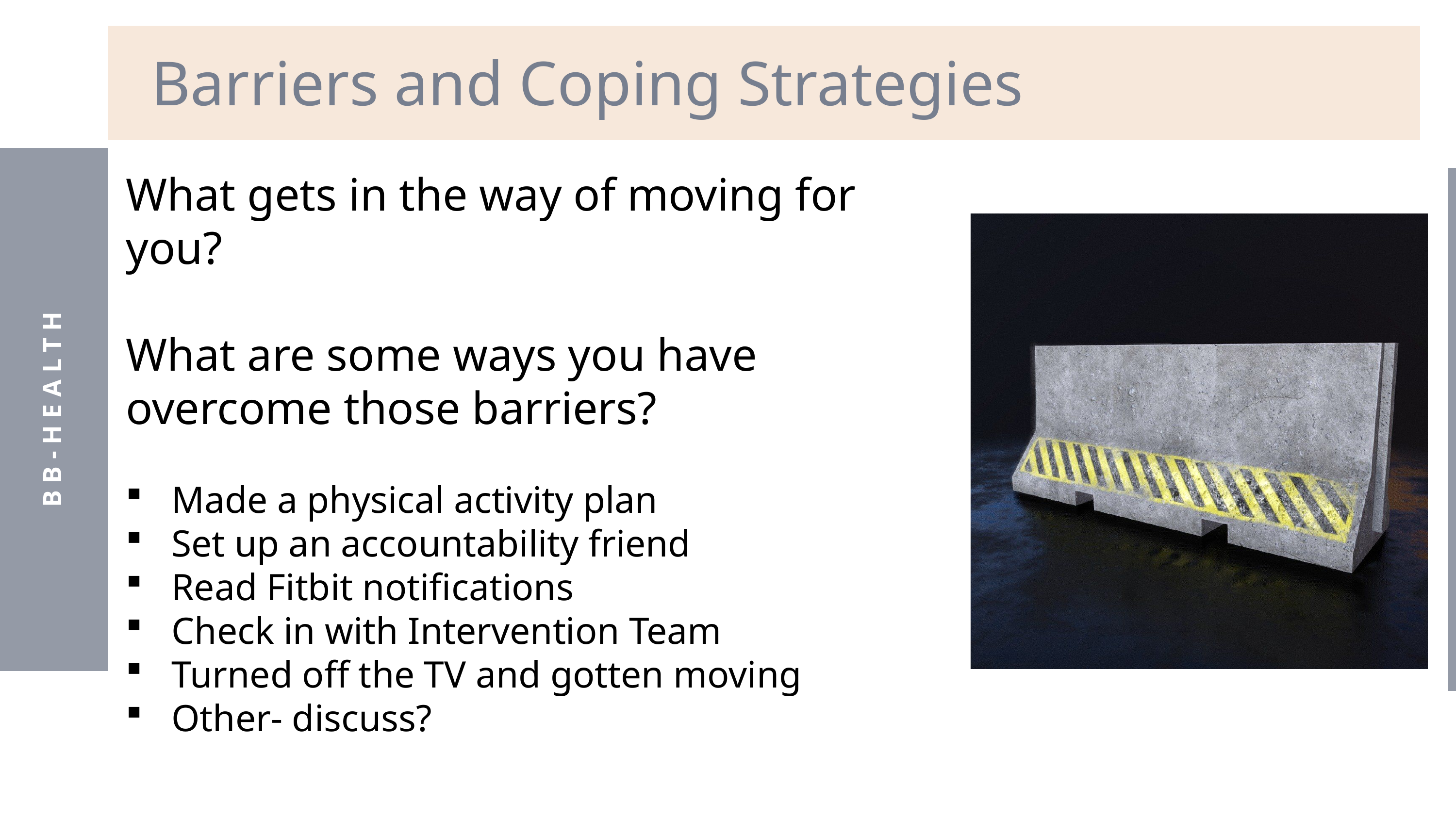

Barriers and Coping Strategies
What gets in the way of moving for you?
What are some ways you have overcome those barriers?
Made a physical activity plan
Set up an accountability friend
Read Fitbit notifications
Check in with Intervention Team
Turned off the TV and gotten moving
Other- discuss?
BB-HEALTH

## Slide 10
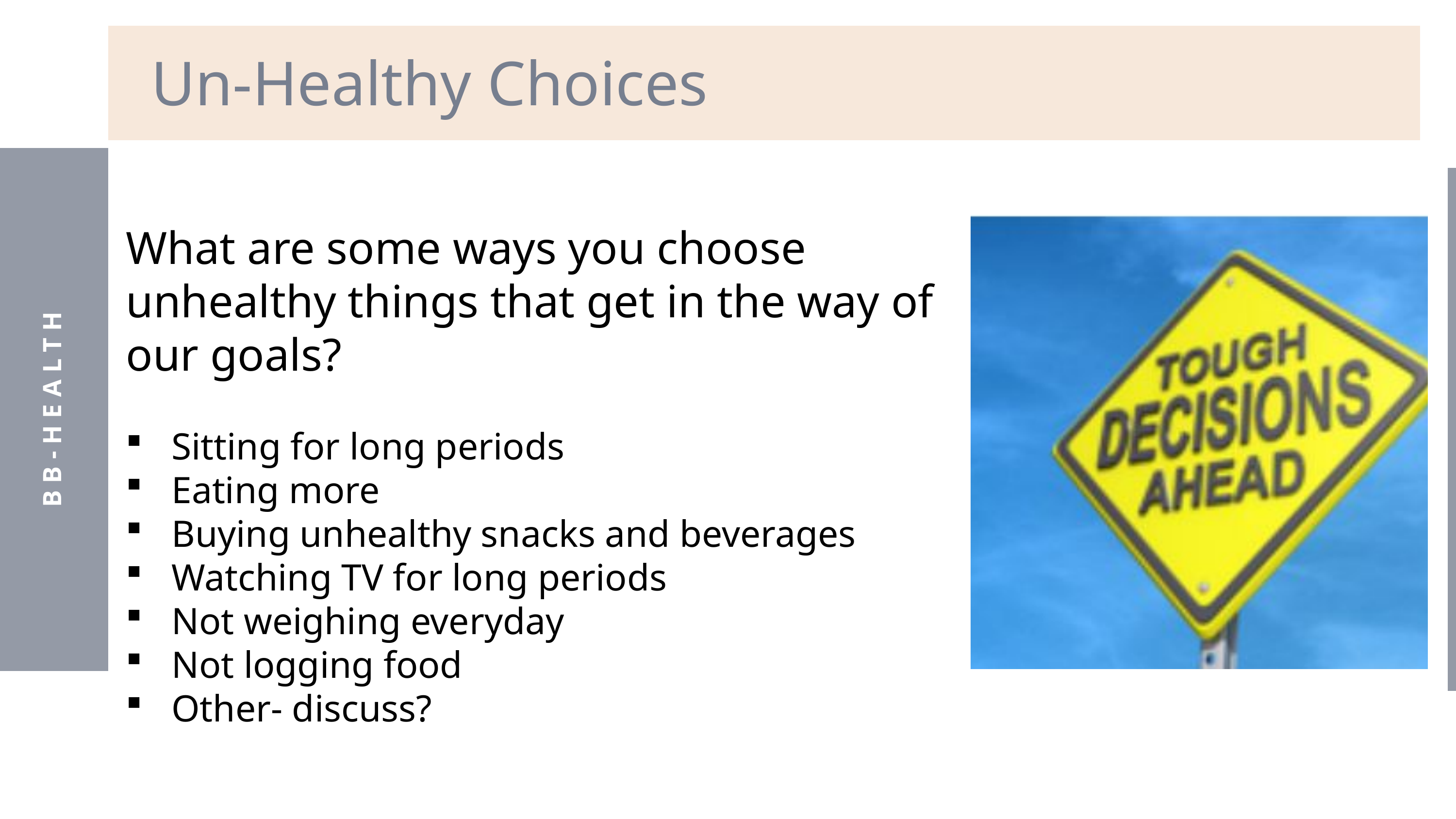

Un-Healthy Choices
What are some ways you choose unhealthy things that get in the way of our goals?
Sitting for long periods
Eating more
Buying unhealthy snacks and beverages
Watching TV for long periods
Not weighing everyday
Not logging food
Other- discuss?
BB-HEALTH

## Slide 11
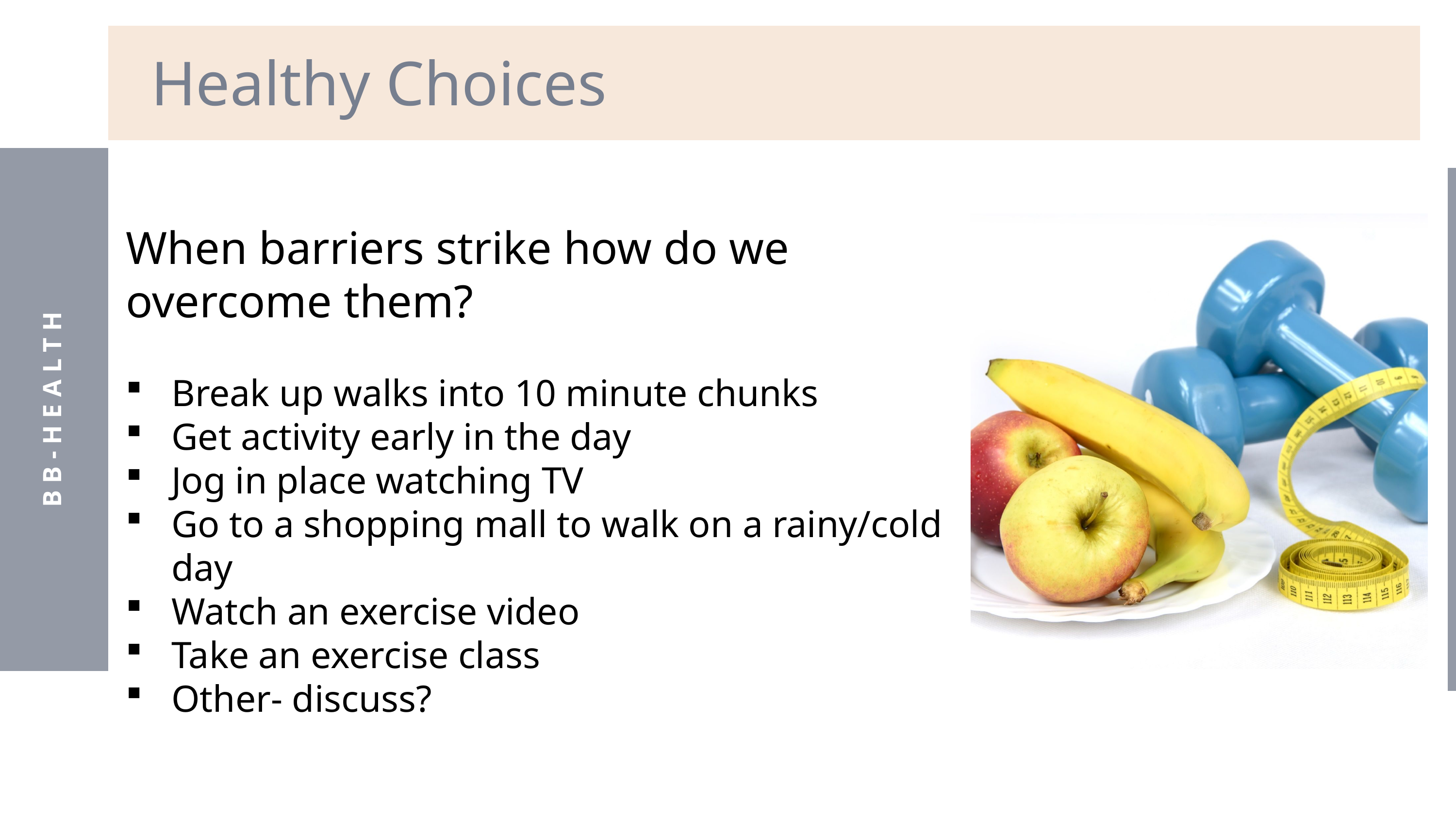

Healthy Choices
When barriers strike how do we overcome them?
Break up walks into 10 minute chunks
Get activity early in the day
Jog in place watching TV
Go to a shopping mall to walk on a rainy/cold day
Watch an exercise video
Take an exercise class
Other- discuss?
BB-HEALTH

## Slide 12
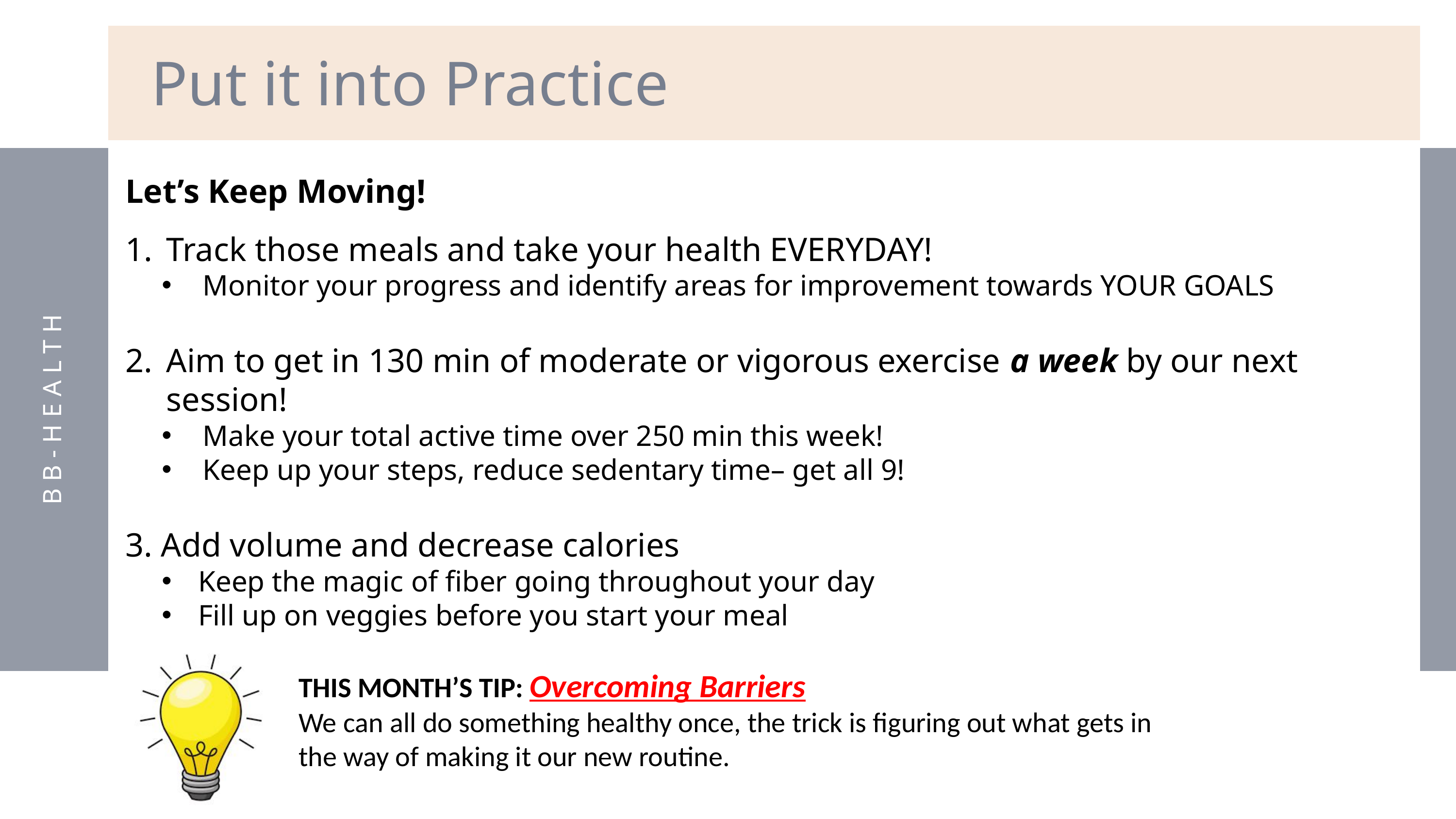

Put it into Practice
Let’s Keep Moving!
Track those meals and take your health EVERYDAY!
Monitor your progress and identify areas for improvement towards YOUR GOALS
Aim to get in 130 min of moderate or vigorous exercise a week by our next session!
Make your total active time over 250 min this week!
Keep up your steps, reduce sedentary time– get all 9!
3. Add volume and decrease calories
Keep the magic of fiber going throughout your day
Fill up on veggies before you start your meal
BB-HEALTH
THIS MONTH’S TIP: Overcoming Barriers
We can all do something healthy once, the trick is figuring out what gets in the way of making it our new routine.

## Slide 13
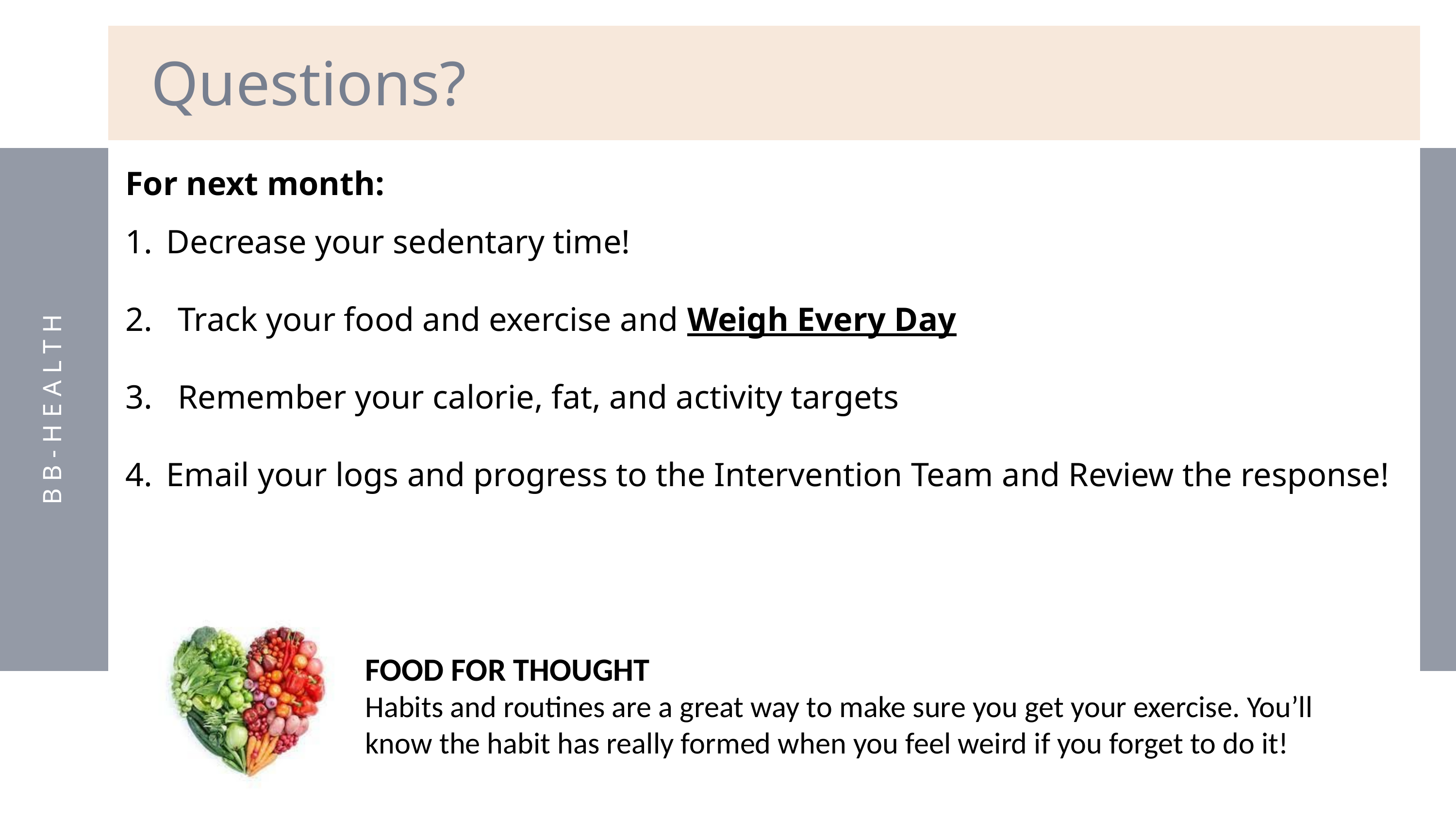

Questions?
For next month:
Decrease your sedentary time!
2. Track your food and exercise and Weigh Every Day
3. Remember your calorie, fat, and activity targets
Email your logs and progress to the Intervention Team and Review the response!
BB-HEALTH
FOOD FOR THOUGHT
Habits and routines are a great way to make sure you get your exercise. You’ll know the habit has really formed when you feel weird if you forget to do it!
