# Supplementary material for: Development and Feasibility of an eHealth Diabetes Prevention Program Adapted for Older Adults—Results from a Randomized Control Pilot Study
Source: Nutrients. 2024 Mar 23;16(7):930. doi: 10.3390/nu16070930 (PMC11154527; doi:10.3390/nu16070930)
Supplement: Supplementary file 1 [file nutrients-16-00930-s001.zip › Session19_SUPP.pdf]

BB-HEALTH

e-Healthy Older Adults Modification

Diabetes Prevention Program

Session #19
Keep Moving!

Group Opening Discussion

BB-HEALTH

WELCOME BACK! **<u>HOW WAS LAST MONTH</u>**?

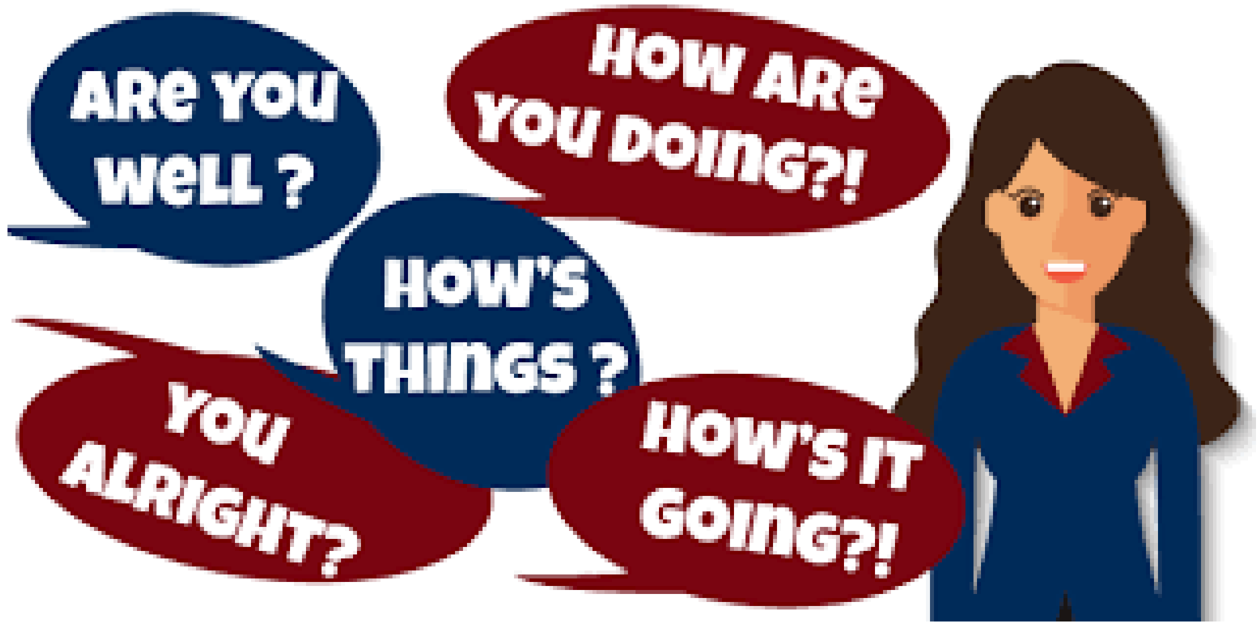

1. Did you track every meal?

2. Did you weigh every day, & record your health today?

3. Did you pump up the VOLUME on healthy filling food?

4. Did you email the Intervention Team and review the response?

5. What were your unique challenges and successes?

REMINDER: CHARGE YOUR FITBIT & SCALE DURING THIS MEETING!

Healthy Lifestyle Tools

BB-HEALTH

What tools do you know about to support a healthy lifestyle?
(*answer out loud or in the chat!)*

What tools have you tried that work for your lifestyle goals?
(*answer out loud or in the chat!)*

What tools worked for you but you haven't used in a while?
(*answer out loud or in the chat!)*

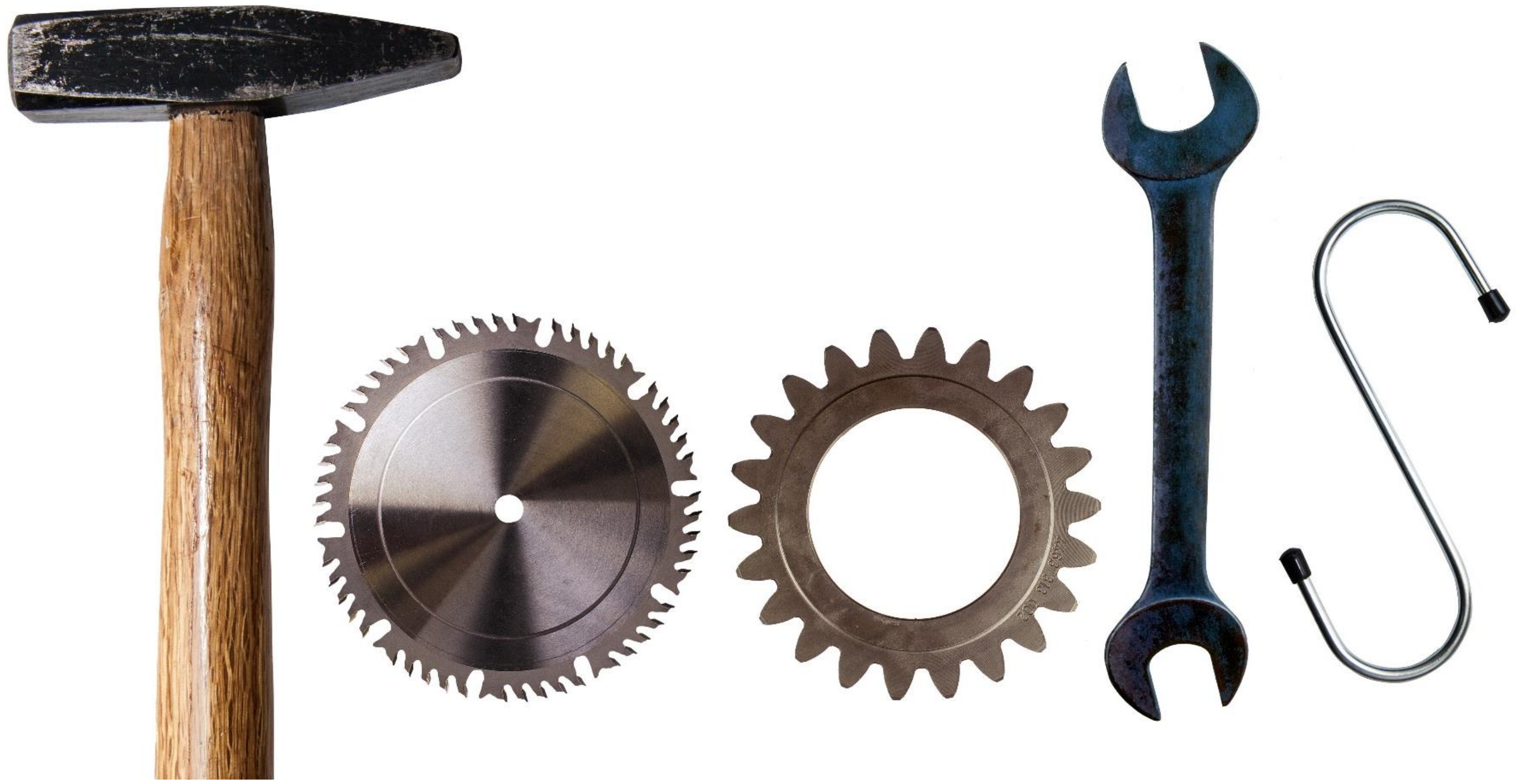

Sitting Less Goals -- Open your Trackers

BB-HEALTH

What day of the week are you getting the most TABS

- Monday
- Tuesday
- Wednesday
- Thursday
- Friday
- Saturday
- Sunday

Is it the same for SUPER TABS?

- Yes
- No

What about time of day?

- Morning
- Afternoon
- Evening
- Night

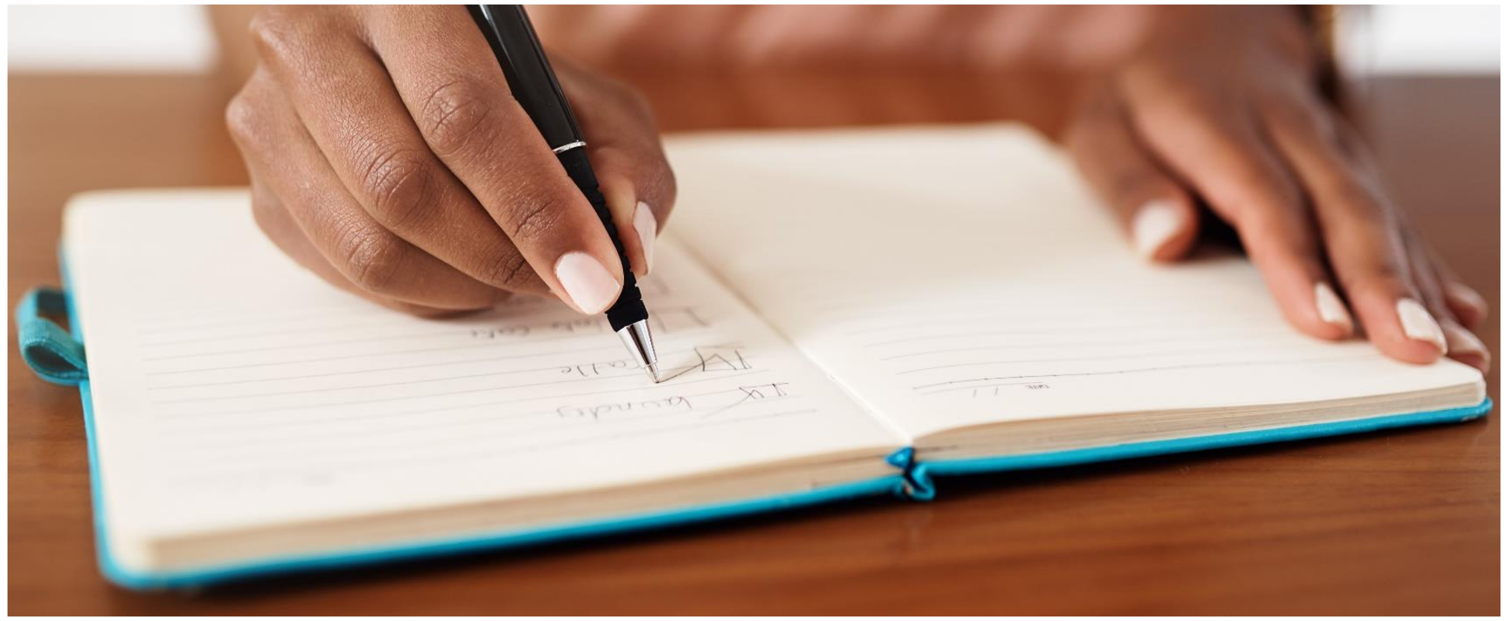

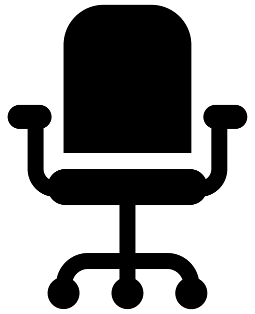

Calculate your average daily TABS & SUPER TABS and put it in the chat.

How do you compare to other group members?

Make your TABS Boost Your Goals

Use TABS as an excuse to get your active zone minutes and **go 2 for 1!**

Plan out your TABS like your plan your physical activity. (page 3)

When can you sneak in an extra 10 minutes of standing, walking, or jogging in place? (*discuss)*

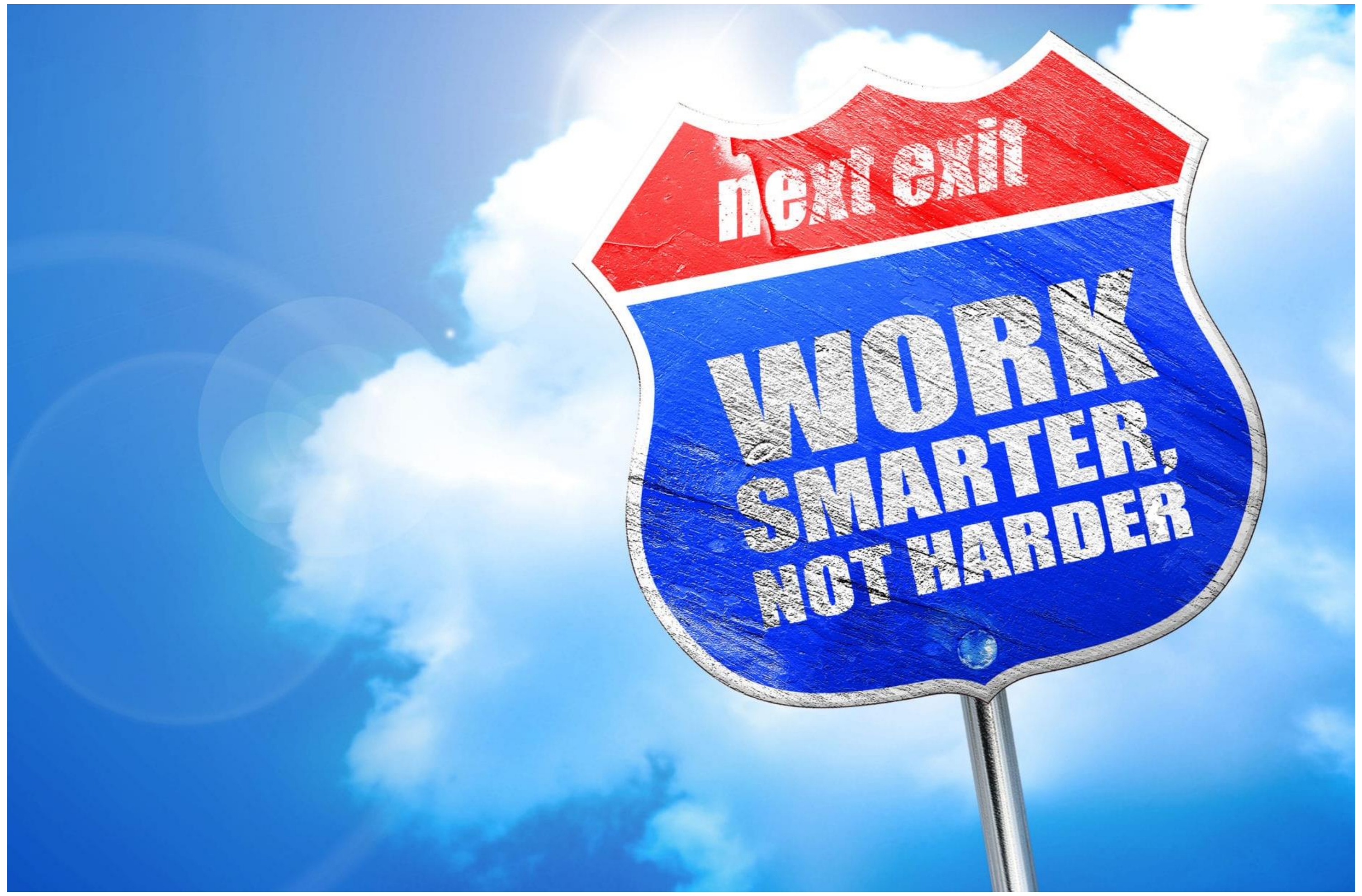

Posture for Health

How is your posture, typically?

- I'm always on top of good posture
- I try to remember to sit and stand up straight
- Sometimes good, sometimes bad
- Often have bad posture
- I never have good posture

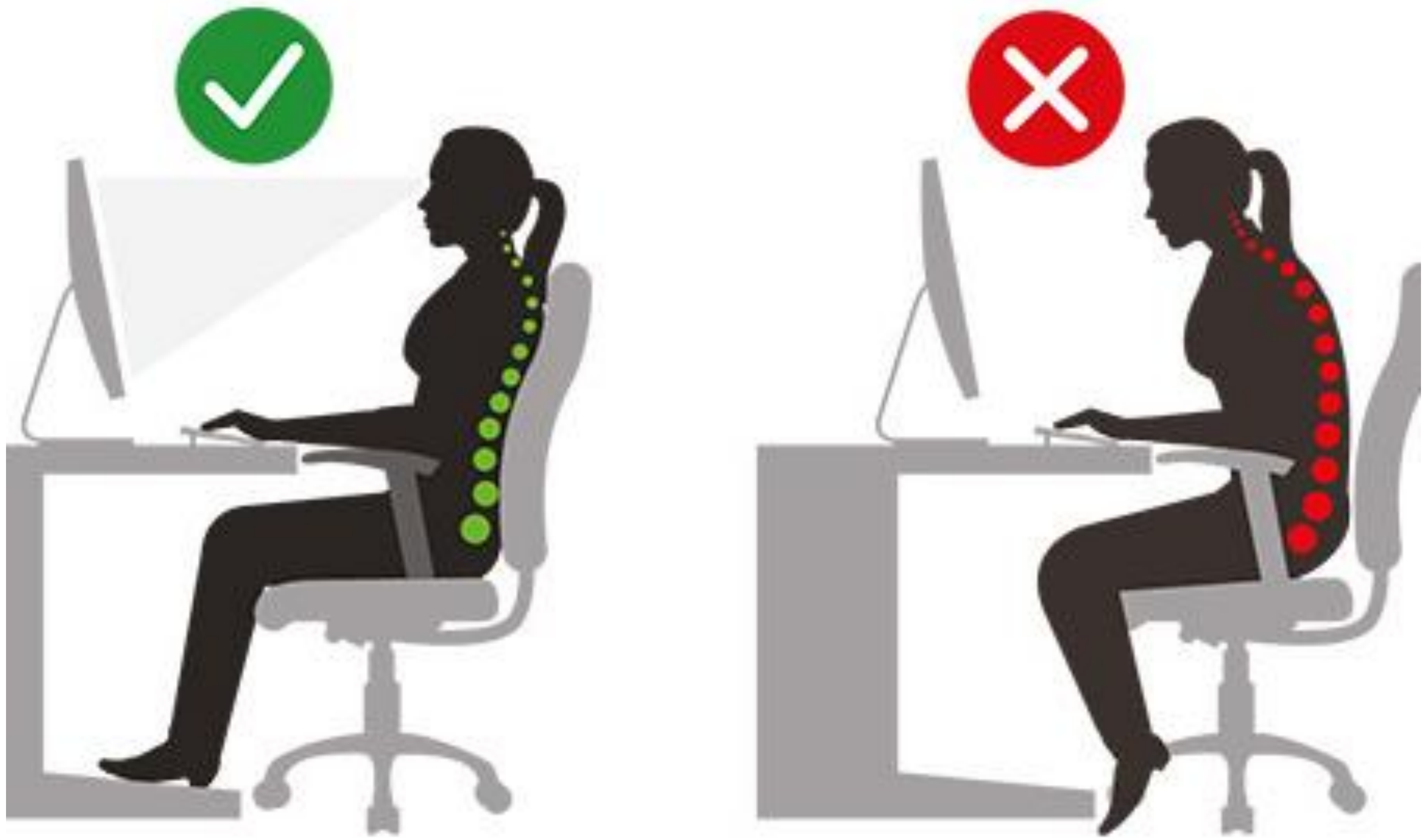

Top Reasons for Good Posture

- Works core muscles
- Reduces back and neck pain
- Makes exercise easier
- Works muscles evenly
- Limits breathing
- Impacts blood pressure
- Improves appearance
- Other (discuss)

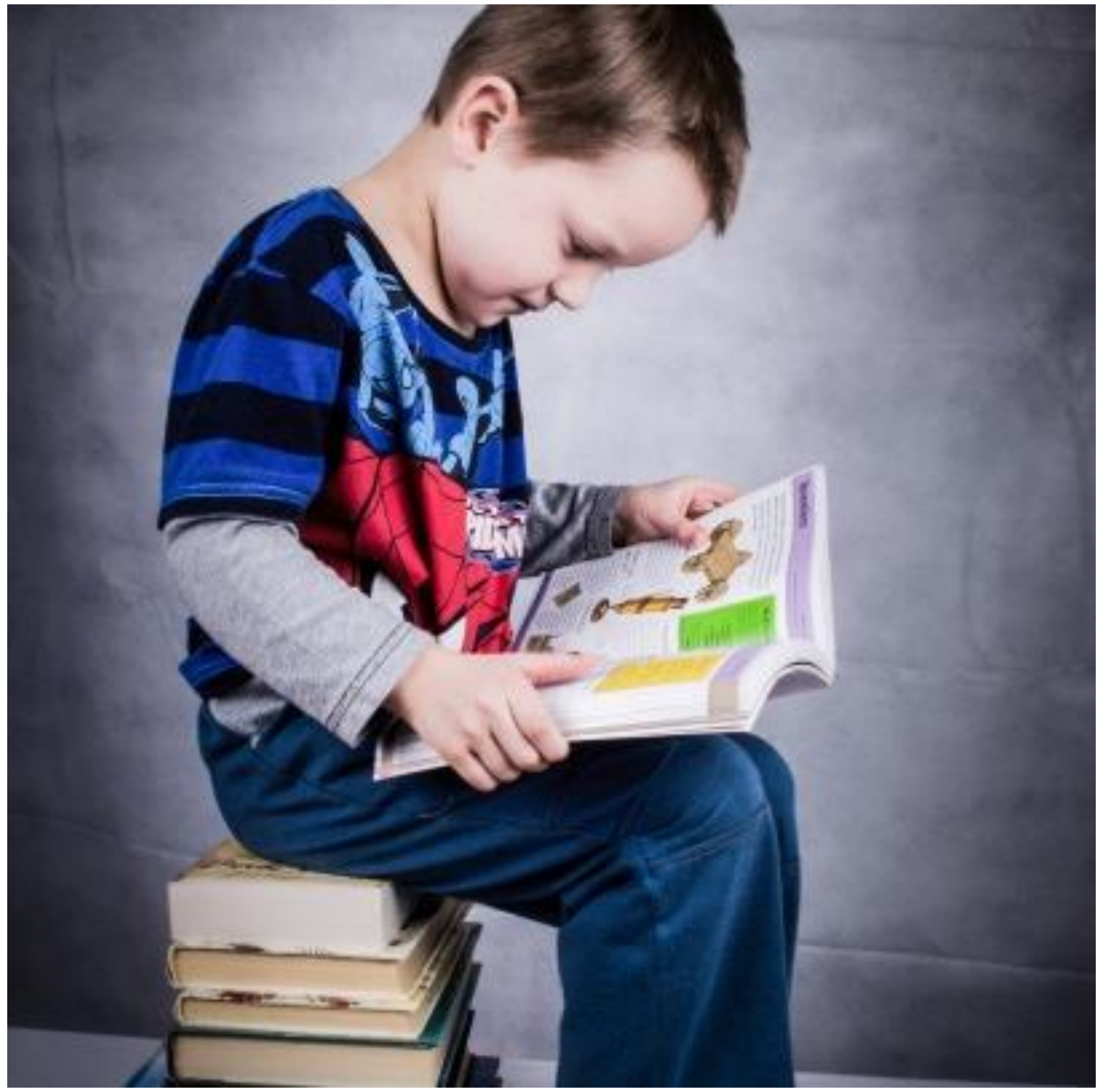

My neck hurts just looking at this.

What is your problem posture activity?

Why do I want to stay active?

BB-HEALTH

What is your activity motivation?

- I feel better
- I’m looking better
- Gives me something to do
- A good way to spend time with friends and family
- Live longer
- Helps control weight and health
- Other (discuss)

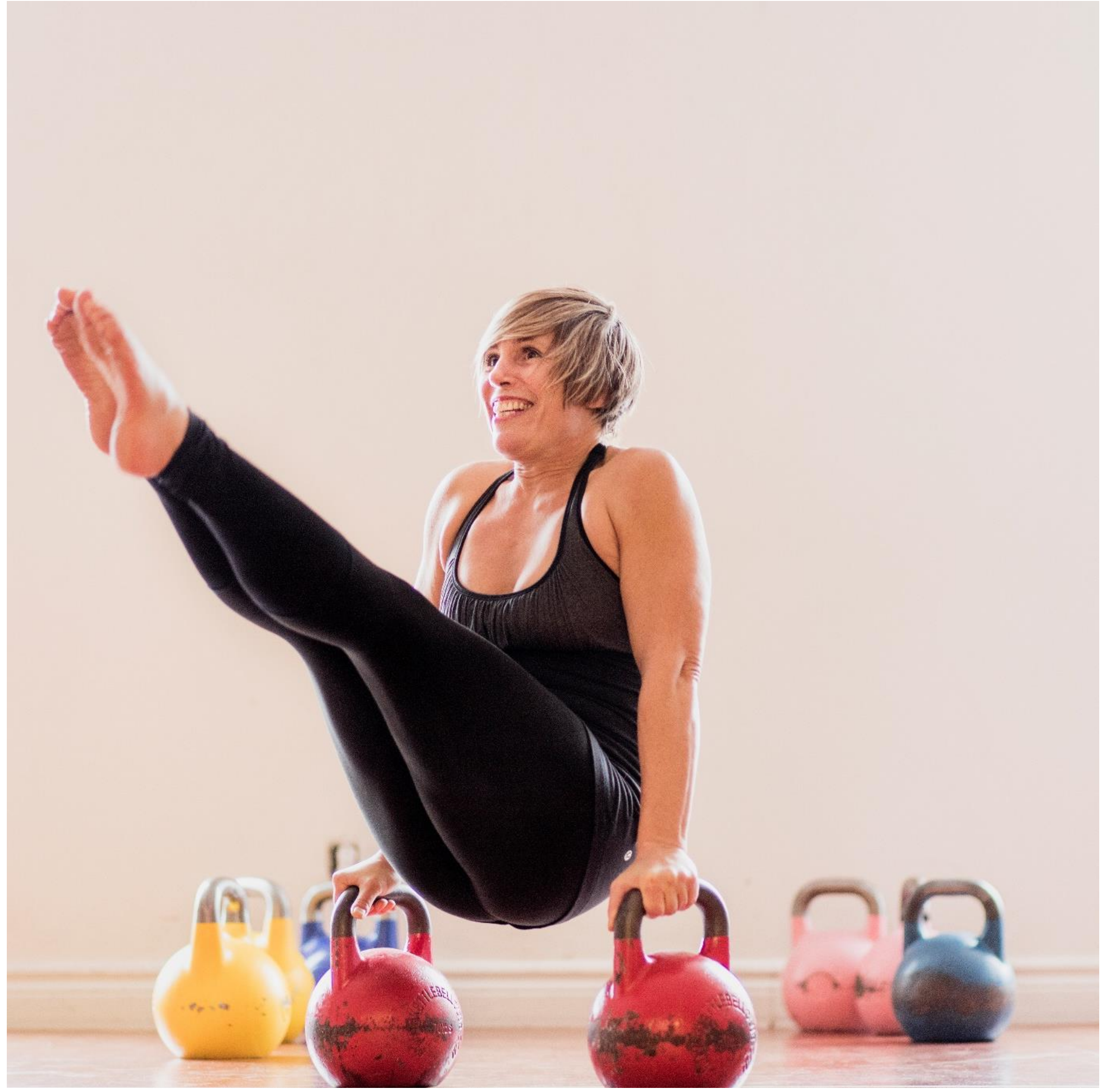

What is your favorite way to get in your active zone minutes?

Barriers and Coping Strategies

BB-HEALTH

What gets in the way of moving for you?

What are some ways you have overcome those barriers?

- Made a physical activity plan
- Set up an accountability friend
- Read Fitbit notifications
- Check in with Intervention Team
- Turned off the TV and gotten moving
- Other- discuss?

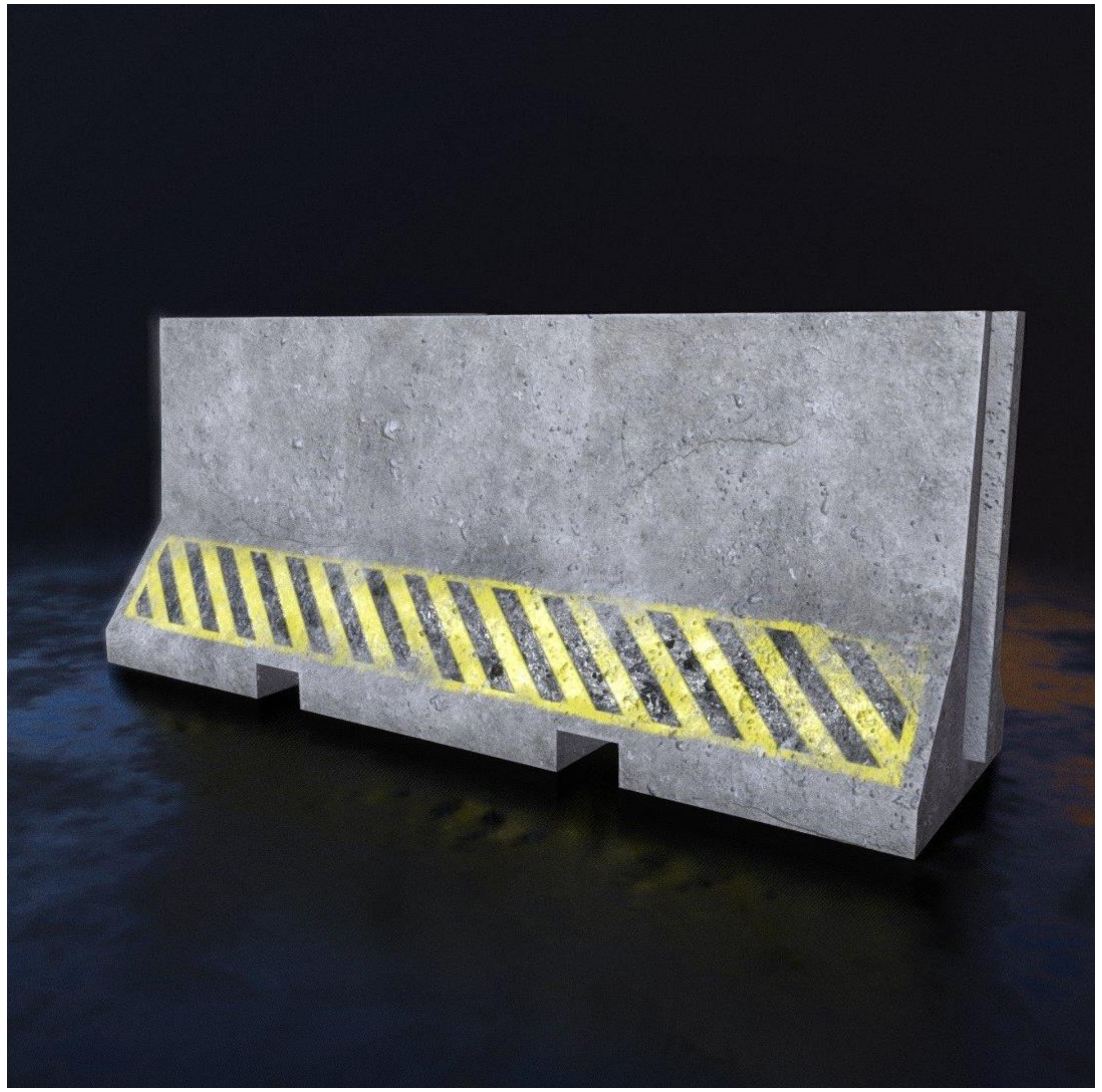

Un-Healthy Choices

What are some ways you choose unhealthy things that get in the way of our goals?

- Sitting for long periods
- Eating more
- Buying unhealthy snacks and beverages
- Watching TV for long periods
- Not weighing everyday
- Not logging food
- Other- discuss?

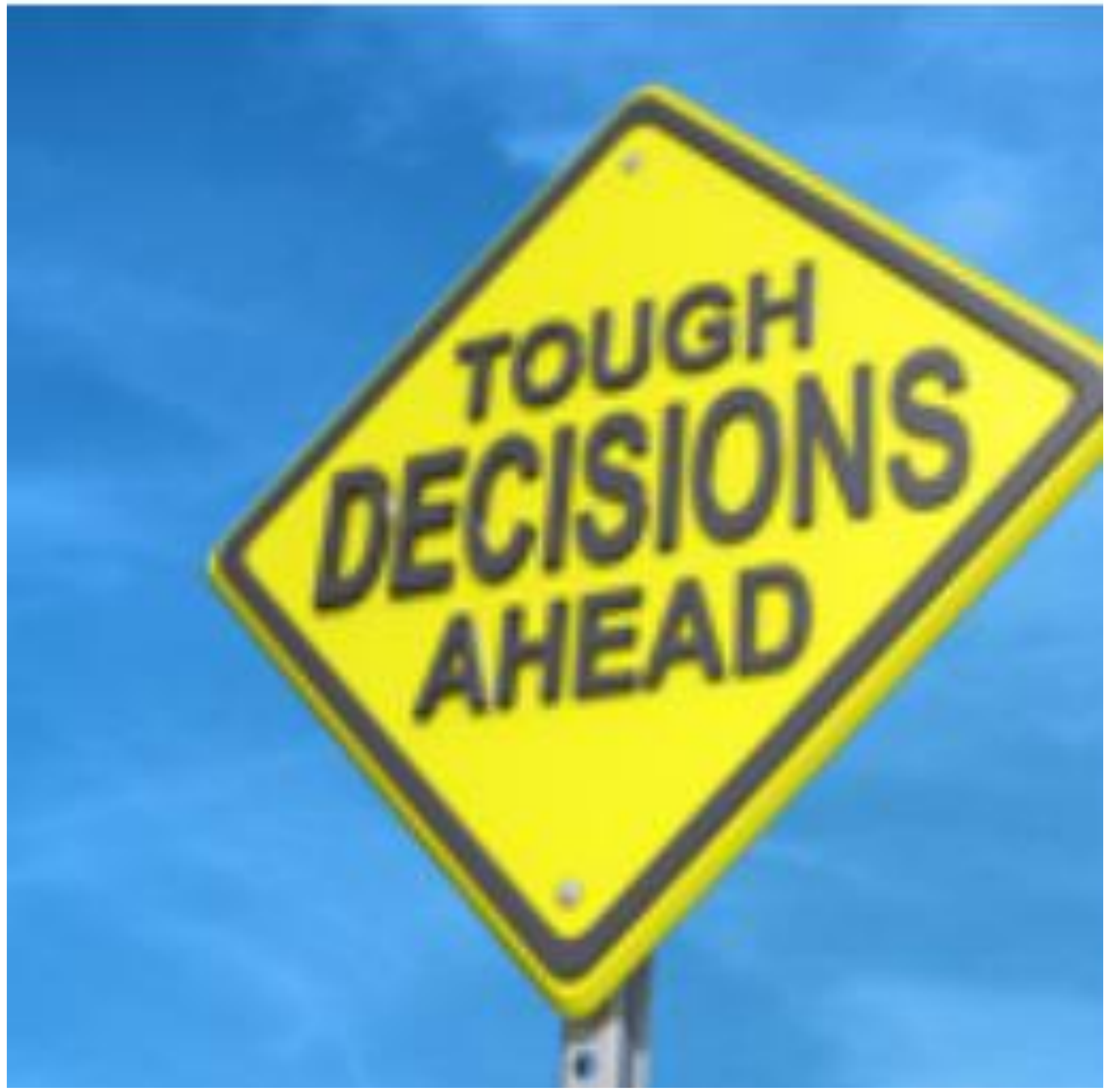

B B - H E A L T H

Healthy Choices

When barriers strike how do we overcome them?

- Break up walks into 10 minute chunks
- Get activity early in the day
- Jog in place watching TV
- Go to a shopping mall to walk on a rainy/cold day
- Watch an exercise video
- Take an exercise class
- Other- discuss?

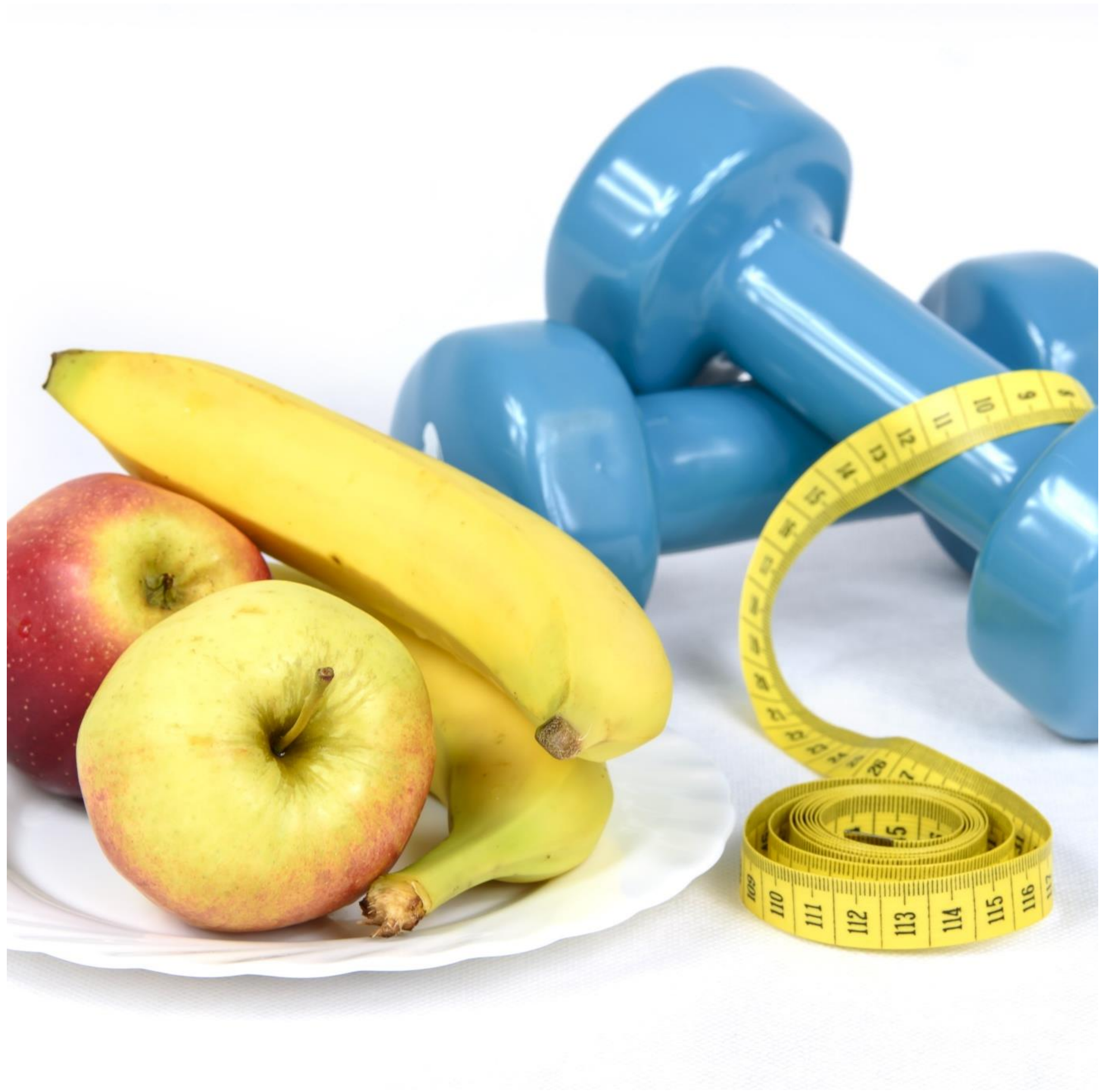

Put it into Practice

BB-HEALTH

Let's Keep Moving!

1. Track those meals and take your health EVERYDAY!
   - Monitor your progress and identify areas for improvement towards YOUR GOALS

2. Aim to get in 130 min of moderate or vigorous exercise ***a week*** by our next session!
   - Make your total active time over 250 min this week!
   - Keep up your steps, reduce sedentary time– get all 9!

3. Add volume and decrease calories
   - Keep the magic of fiber going throughout your day
   - Fill up on veggies before you start your meal

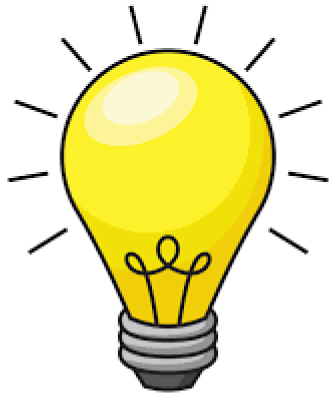

THIS MONTH'S TIP: *Overcoming Barriers*
We can all do something healthy once, the trick is figuring out what gets in the way of making it our new routine.

Questions?

B B - H E A L T H

For next month:

1. Decrease your sedentary time!

2. Track your food and exercise and **<u>Weigh Every Day</u>**

3. Remember your calorie, fat, and activity targets

4. Email your logs and progress to the Intervention Team and Review the response!

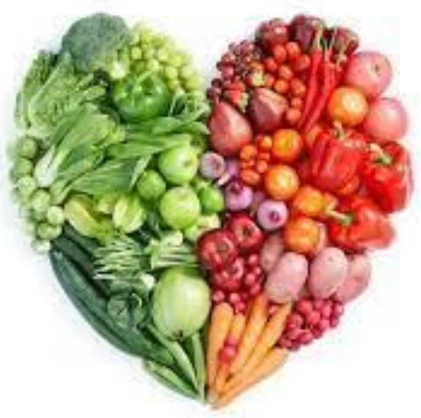

FOOD FOR THOUGHT

Habits and routines are a great way to make sure you get your exercise. You'll know the habit has really formed when you feel weird if you forget to do it!
