# Supplementary material for: Development and Feasibility of an eHealth Diabetes Prevention Program Adapted for Older Adults—Results from a Randomized Control Pilot Study
Source: Nutrients. 2024 Mar 23;16(7):930. doi: 10.3390/nu16070930 (PMC11154527; doi:10.3390/nu16070930)
Supplement: Supplementary file 1 [file nutrients-16-00930-s001.zip › Session20.pptx]

## Slide 1
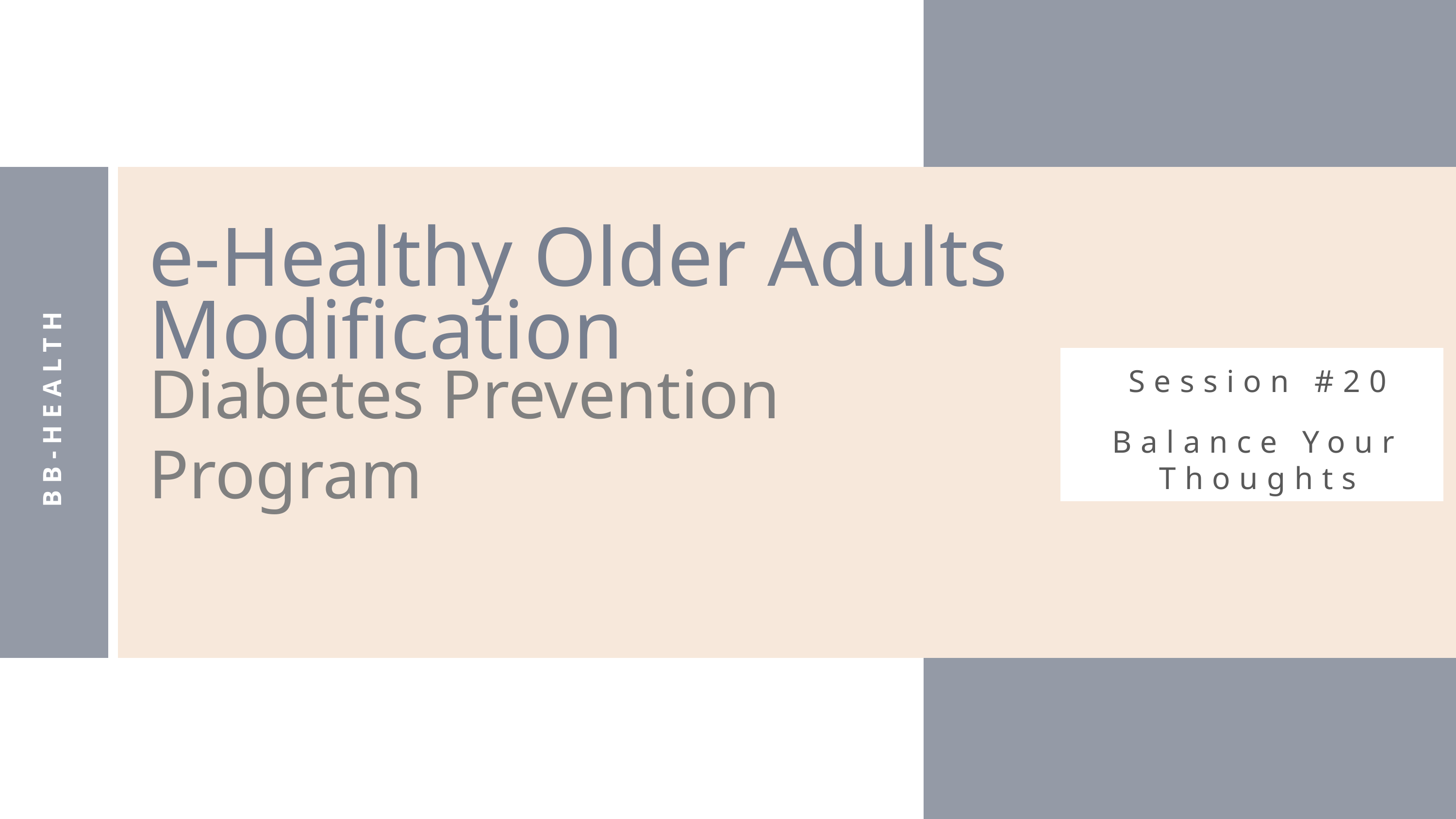

OPEN REPORTS
e-Healthy Older Adults Modification
Session #20
Balance Your Thoughts
Diabetes Prevention Program
BB-HEALTH

## Slide 2
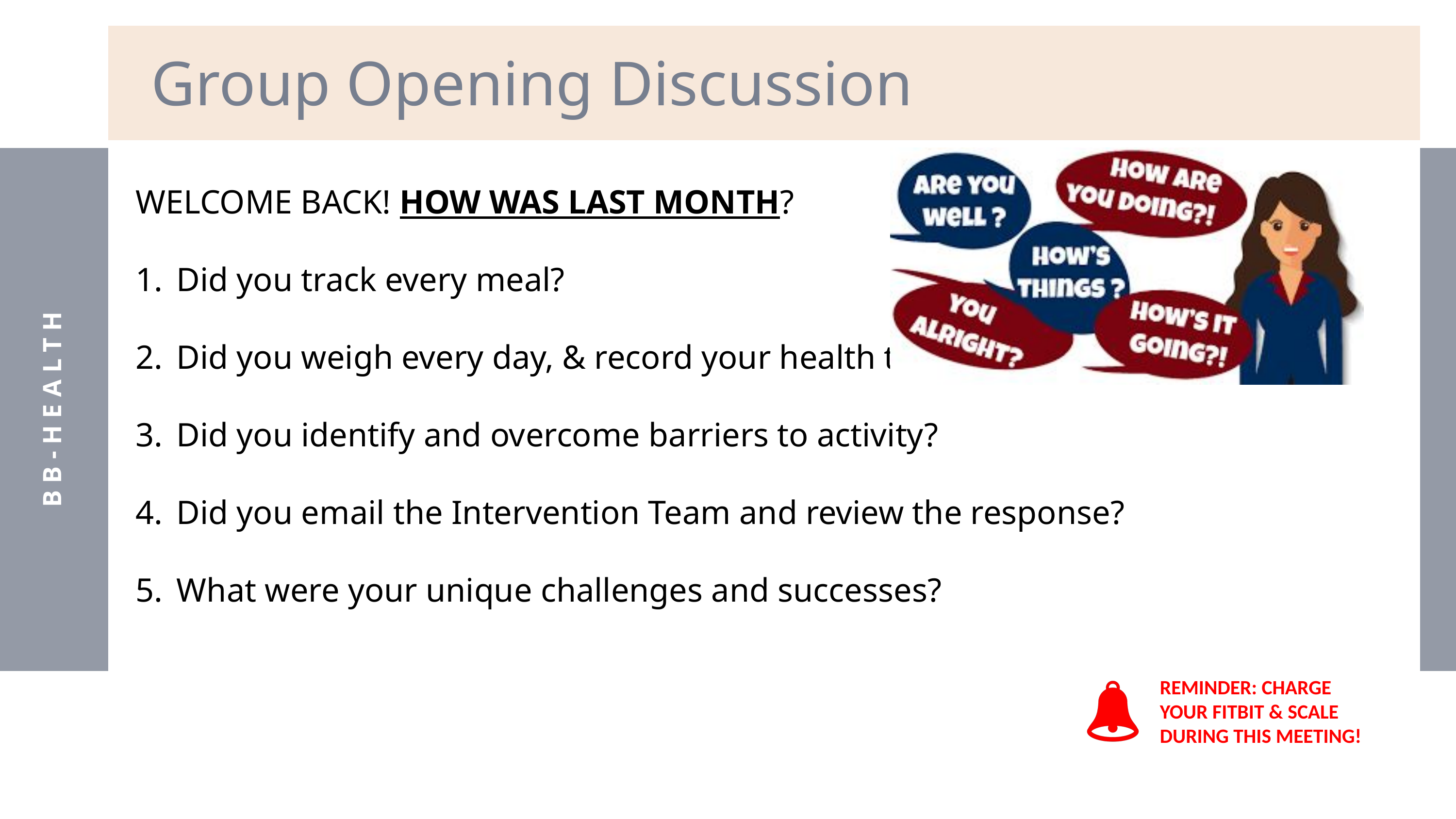

Group Opening Discussion
WELCOME BACK! HOW WAS LAST MONTH?
Did you track every meal?
Did you weigh every day, & record your health today?
Did you identify and overcome barriers to activity?
Did you email the Intervention Team and review the response?
What were your unique challenges and successes?
BB-HEALTH
REMINDER: CHARGE YOUR FITBIT & SCALE DURING THIS MEETING!

## Slide 3
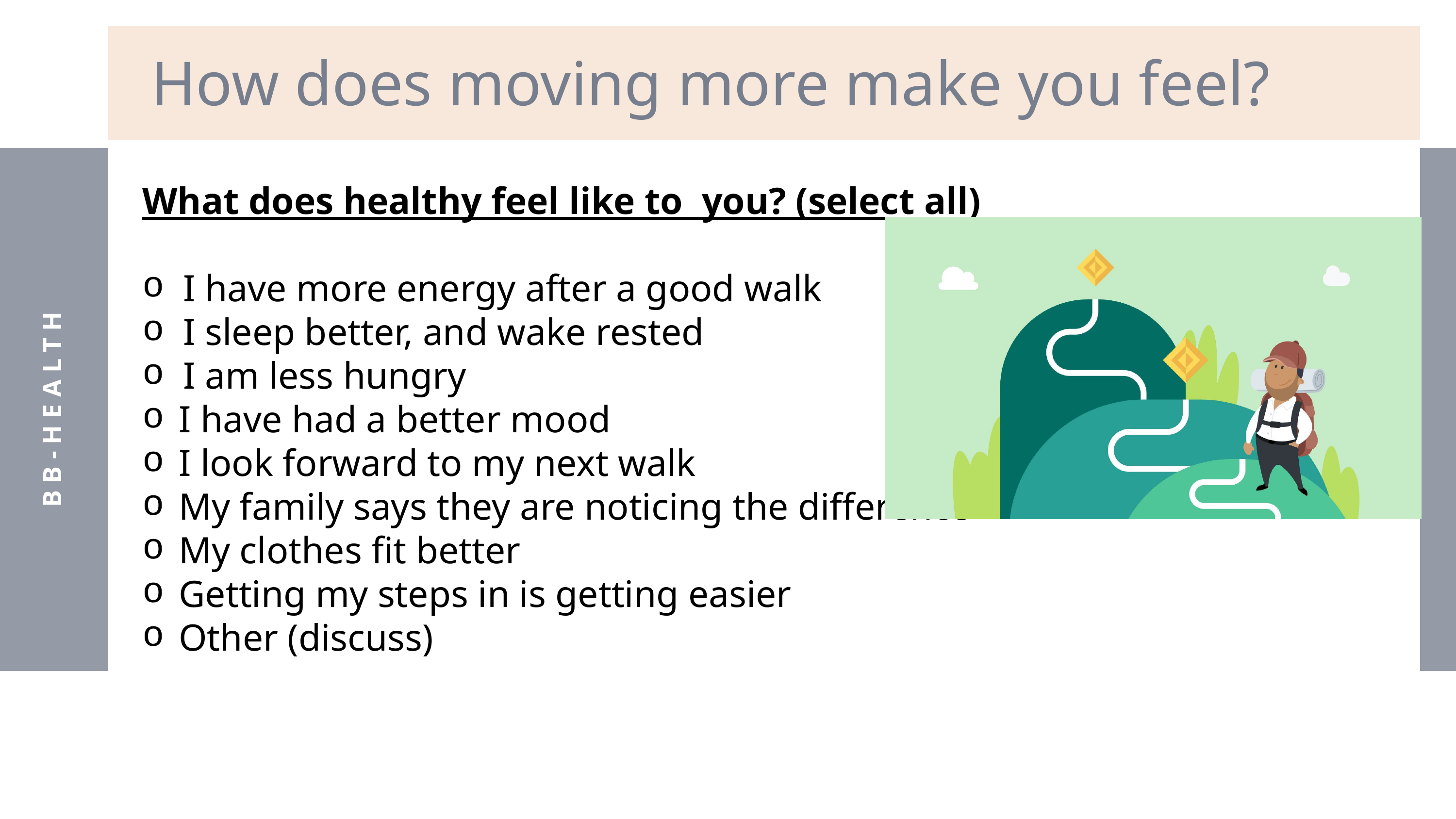

How does moving more make you feel?
What does healthy feel like to you? (select all)
I have more energy after a good walk
I sleep better, and wake rested
I am less hungry
I have had a better mood
I look forward to my next walk
My family says they are noticing the difference
My clothes fit better
Getting my steps in is getting easier
Other (discuss)
BB-HEALTH

## Slide 4
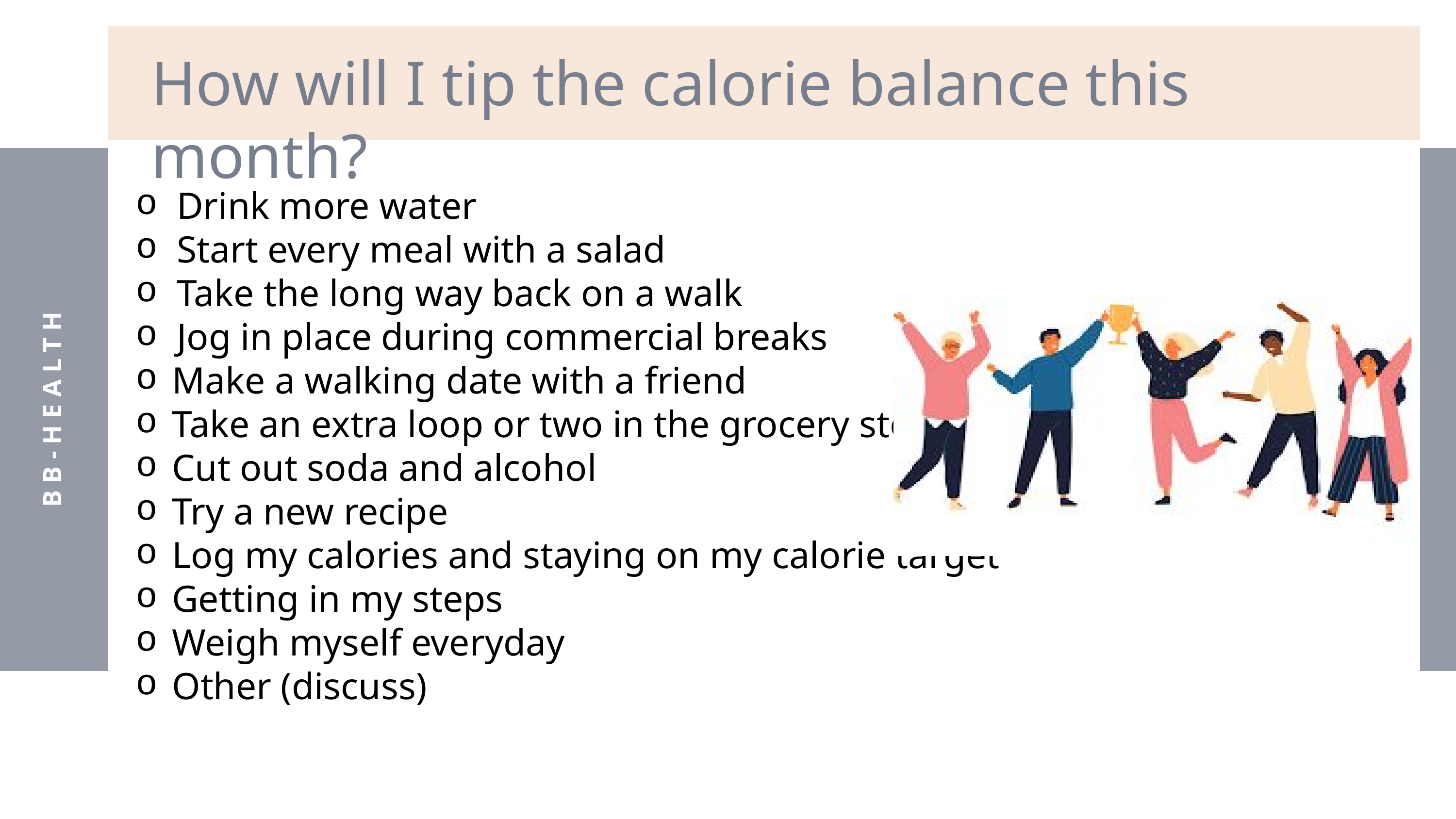

How will I tip the calorie balance this month?
Drink more water
Start every meal with a salad
Take the long way back on a walk
Jog in place during commercial breaks
Make a walking date with a friend
Take an extra loop or two in the grocery store
Cut out soda and alcohol
Try a new recipe
Log my calories and staying on my calorie target
Getting in my steps
Weigh myself everyday
Other (discuss)
BB-HEALTH

## Slide 5
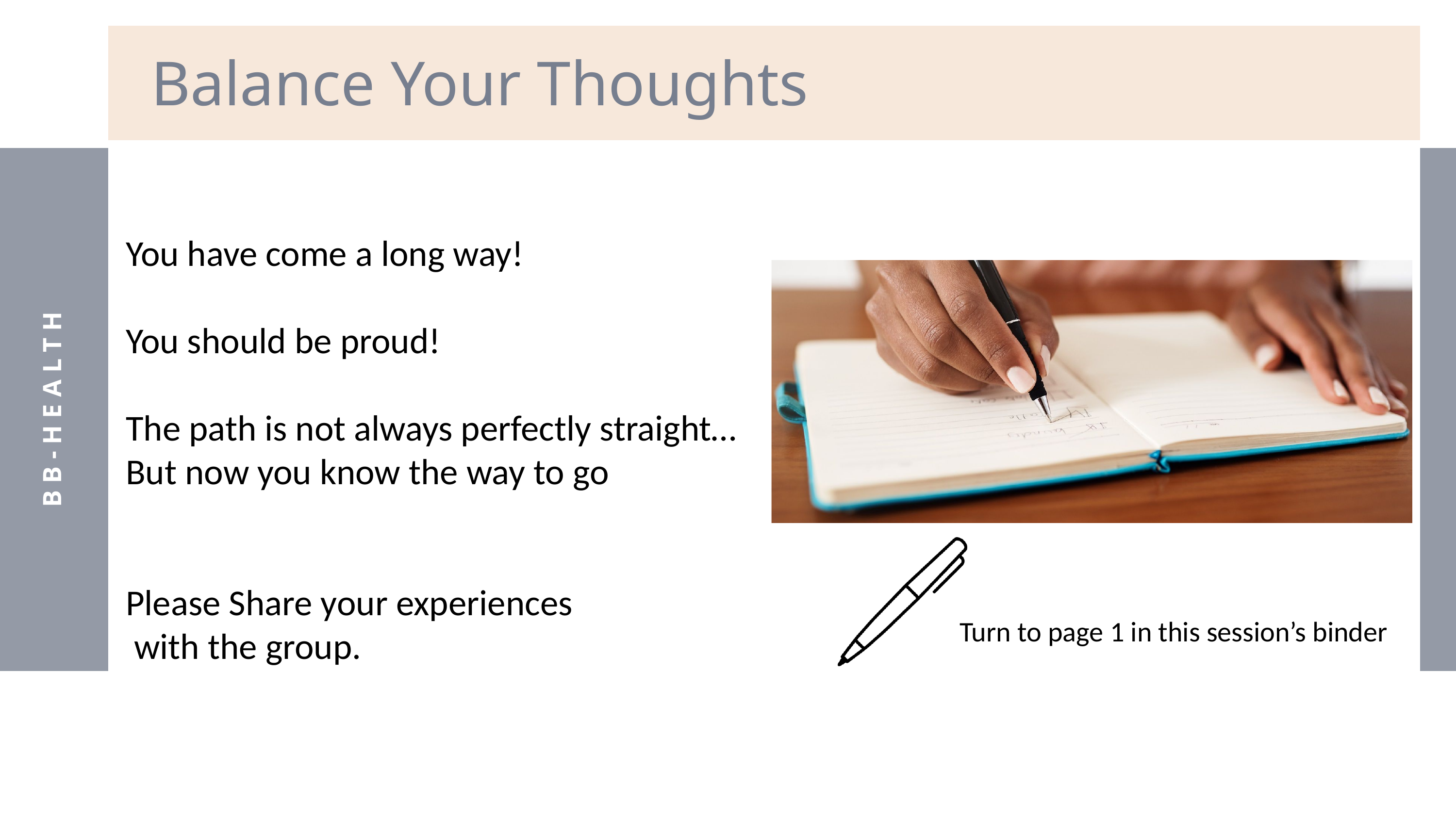

Balance Your Thoughts
You have come a long way!
You should be proud!
The path is not always perfectly straight…
But now you know the way to go
Please Share your experiences
 with the group.
BB-HEALTH
Turn to page 1 in this session’s binder

## Slide 6
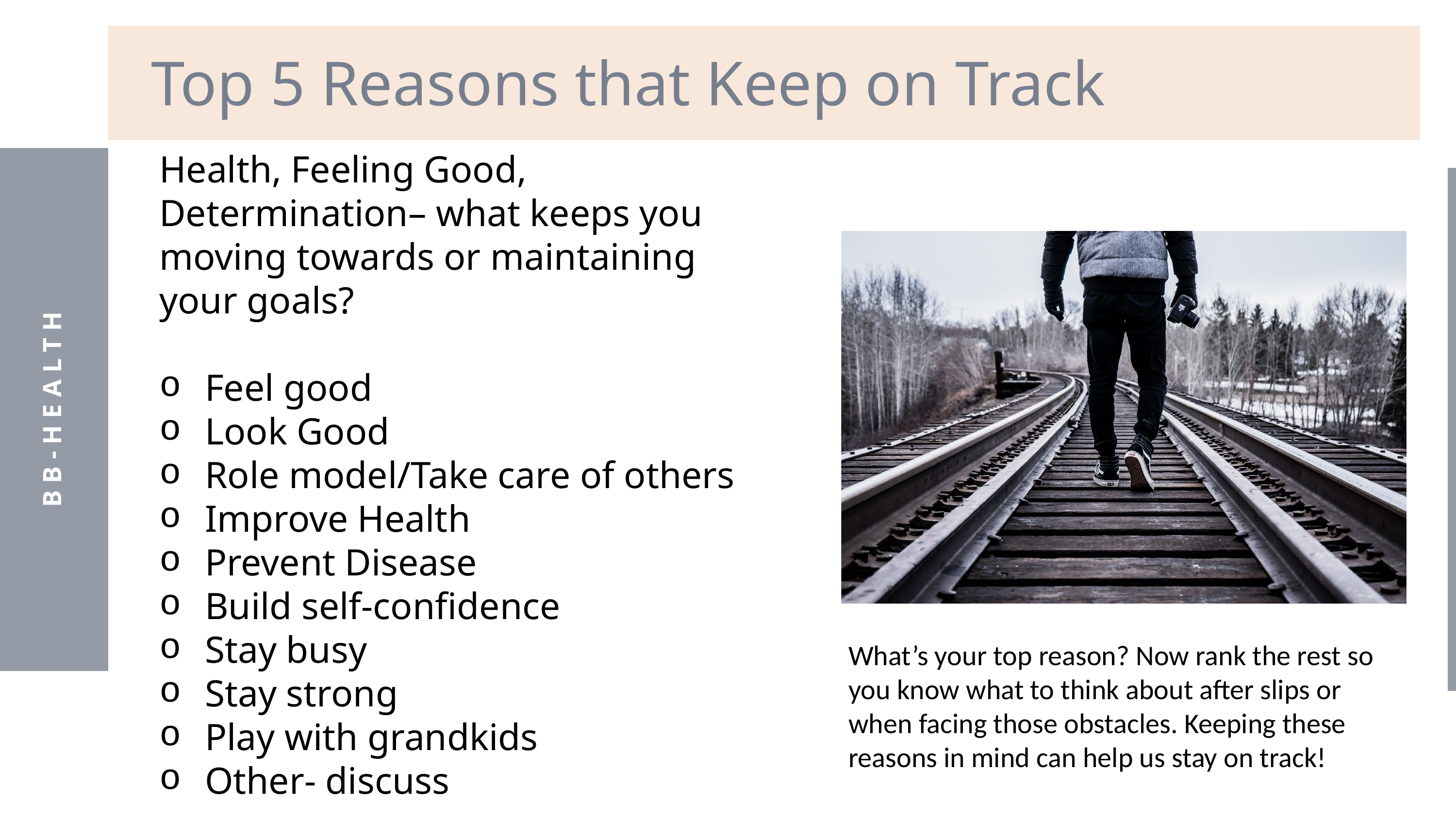

Top 5 Reasons that Keep on Track
Health, Feeling Good, Determination– what keeps you moving towards or maintaining your goals?
Feel good
Look Good
Role model/Take care of others
Improve Health
Prevent Disease
Build self-confidence
Stay busy
Stay strong
Play with grandkids
Other- discuss
BB-HEALTH
What’s your top reason? Now rank the rest so you know what to think about after slips or when facing those obstacles. Keeping these reasons in mind can help us stay on track!

## Slide 7
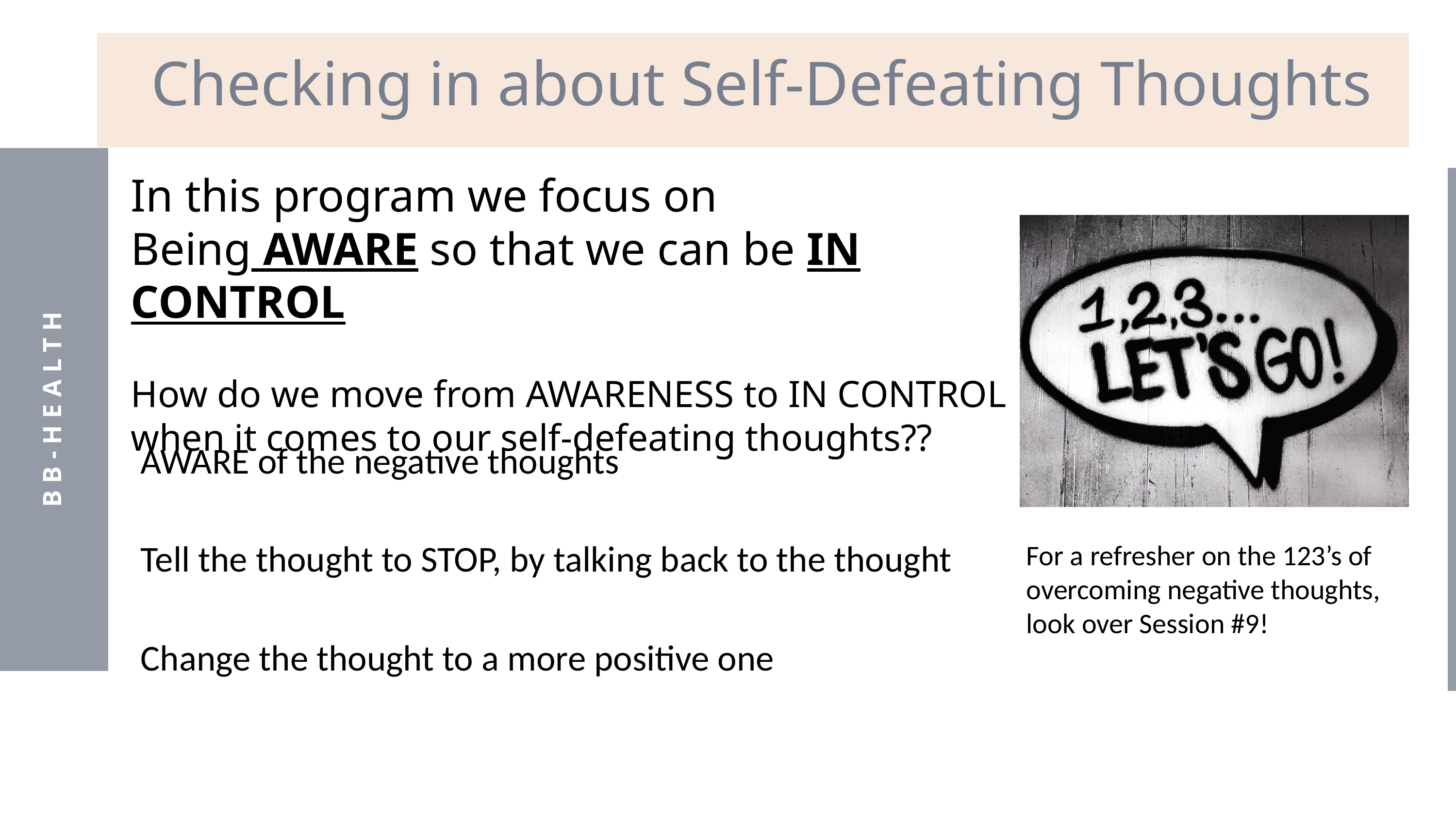

Checking in about Self-Defeating Thoughts
In this program we focus on
Being AWARE so that we can be IN CONTROL
How do we move from AWARENESS to IN CONTROL
when it comes to our self-defeating thoughts??
BB-HEALTH
AWARE of the negative thoughts
Tell the thought to STOP, by talking back to the thought
For a refresher on the 123’s of overcoming negative thoughts, look over Session #9!
Change the thought to a more positive one

## Slide 8
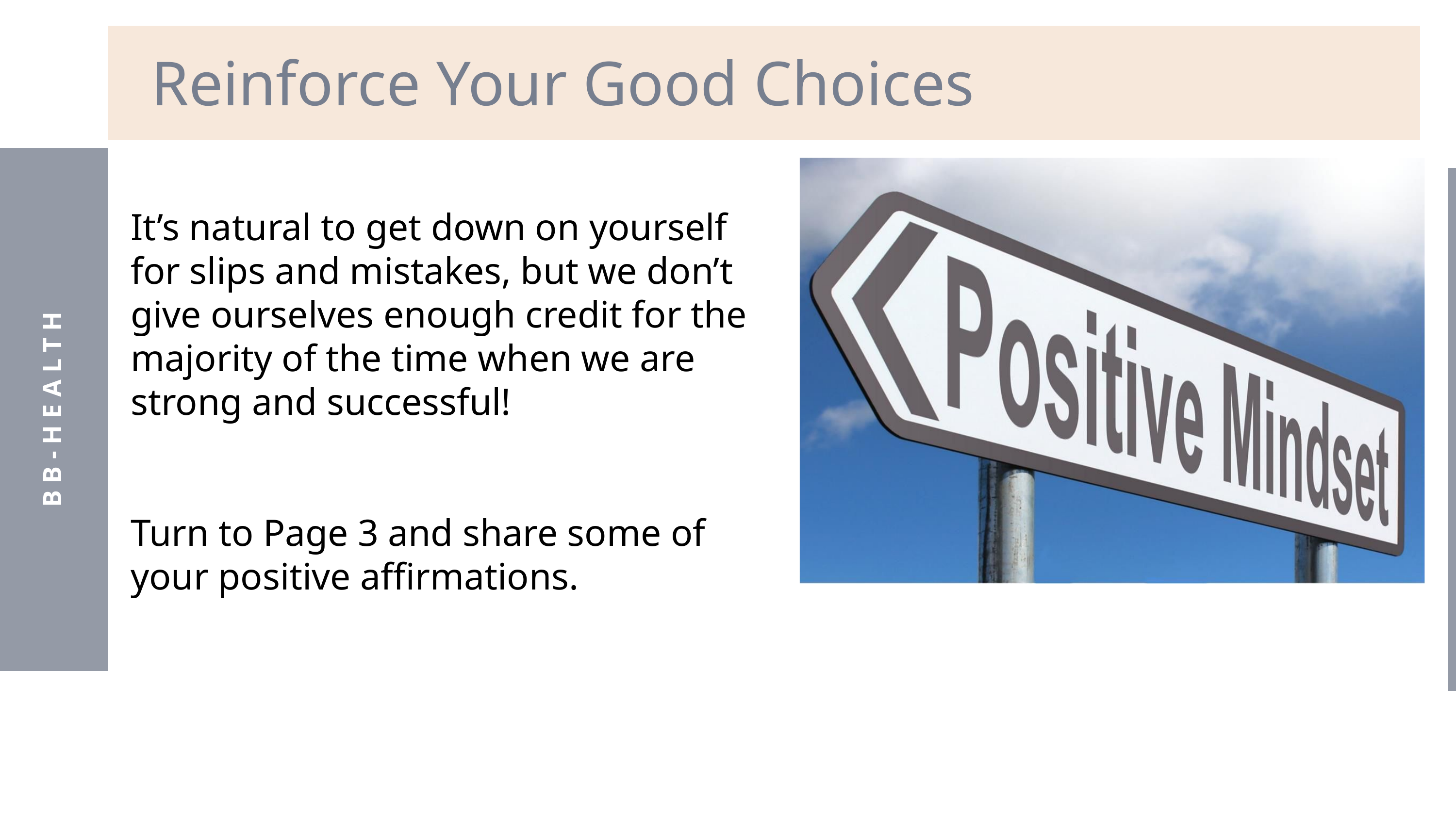

Reinforce Your Good Choices
It’s natural to get down on yourself for slips and mistakes, but we don’t give ourselves enough credit for the majority of the time when we are strong and successful!
Turn to Page 3 and share some of your positive affirmations.
BB-HEALTH

## Slide 9
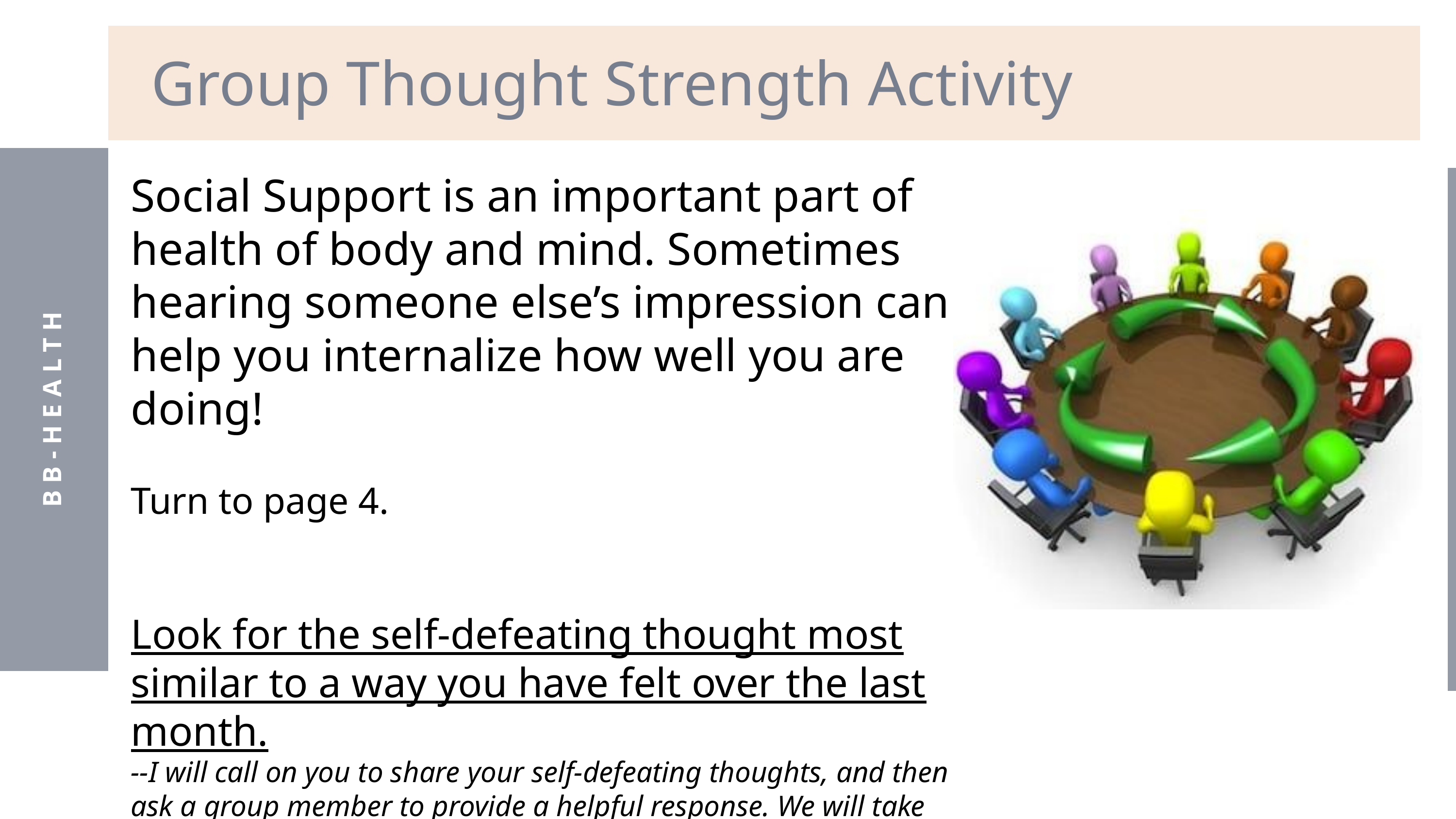

Group Thought Strength Activity
Social Support is an important part of health of body and mind. Sometimes hearing someone else’s impression can help you internalize how well you are doing!
Turn to page 4.
Look for the self-defeating thought most similar to a way you have felt over the last month.
--I will call on you to share your self-defeating thoughts, and then ask a group member to provide a helpful response. We will take turns.
BB-HEALTH

## Slide 10
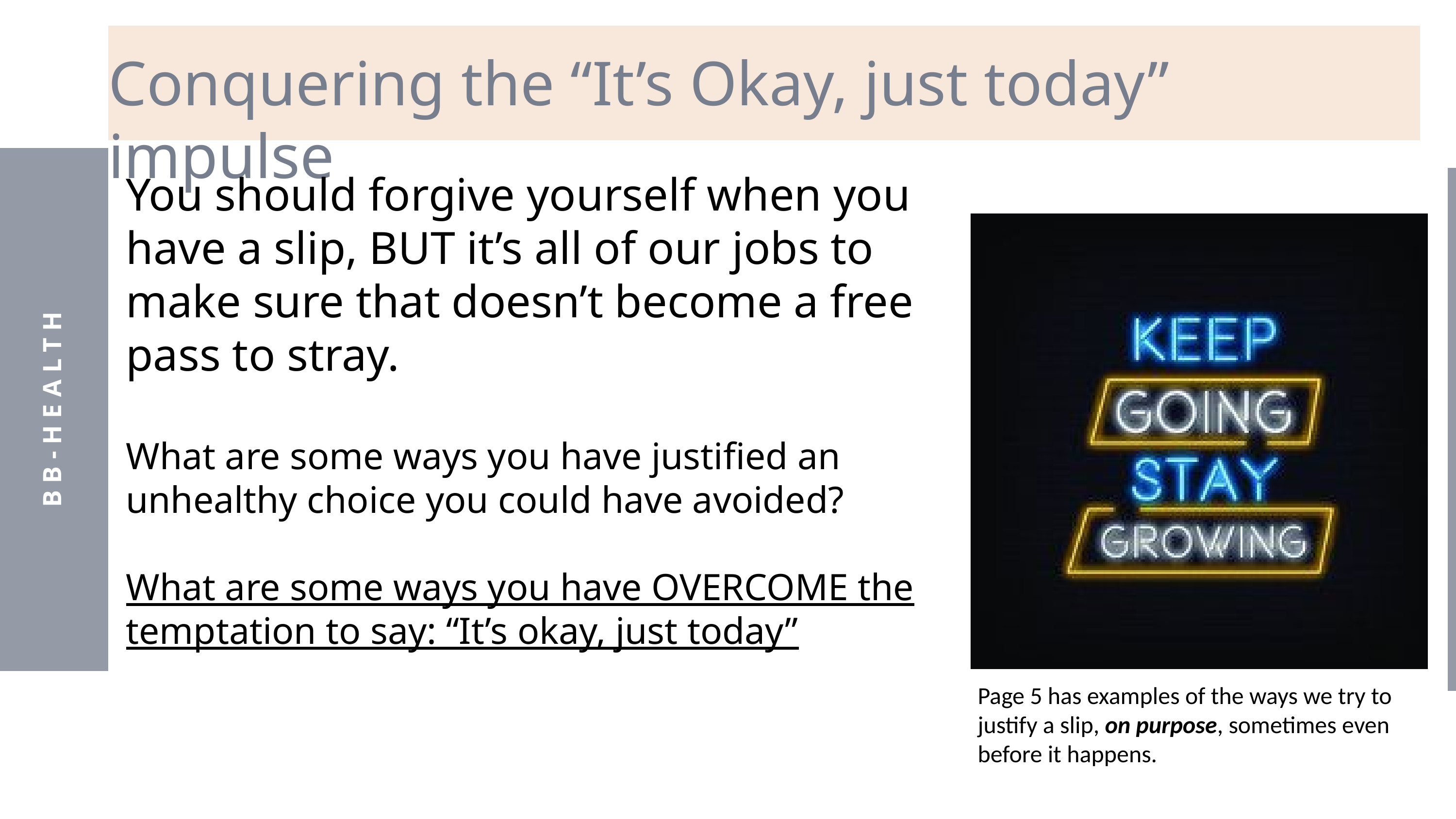

Conquering the “It’s Okay, just today” impulse
You should forgive yourself when you have a slip, BUT it’s all of our jobs to make sure that doesn’t become a free pass to stray.
What are some ways you have justified an unhealthy choice you could have avoided?
What are some ways you have OVERCOME the temptation to say: “It’s okay, just today”
BB-HEALTH
Page 5 has examples of the ways we try to justify a slip, on purpose, sometimes even before it happens.

## Slide 11
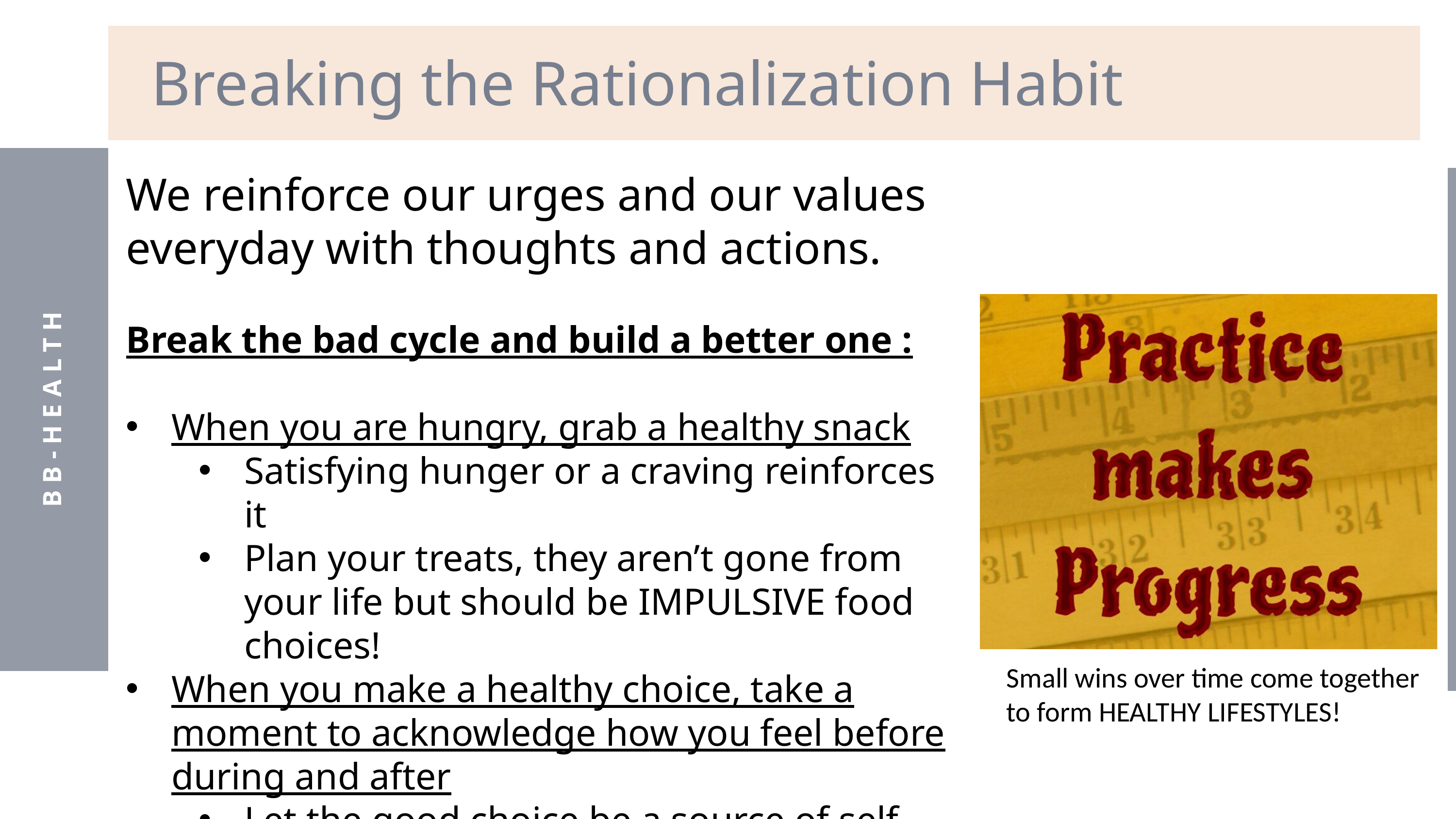

Breaking the Rationalization Habit
We reinforce our urges and our values everyday with thoughts and actions.
Break the bad cycle and build a better one :
When you are hungry, grab a healthy snack
Satisfying hunger or a craving reinforces it
Plan your treats, they aren’t gone from your life but should be IMPULSIVE food choices!
When you make a healthy choice, take a moment to acknowledge how you feel before during and after
Let the good choice be a source of self-praise
Let the good feelings guide future good habits
BB-HEALTH
Small wins over time come together to form HEALTHY LIFESTYLES!

## Slide 12
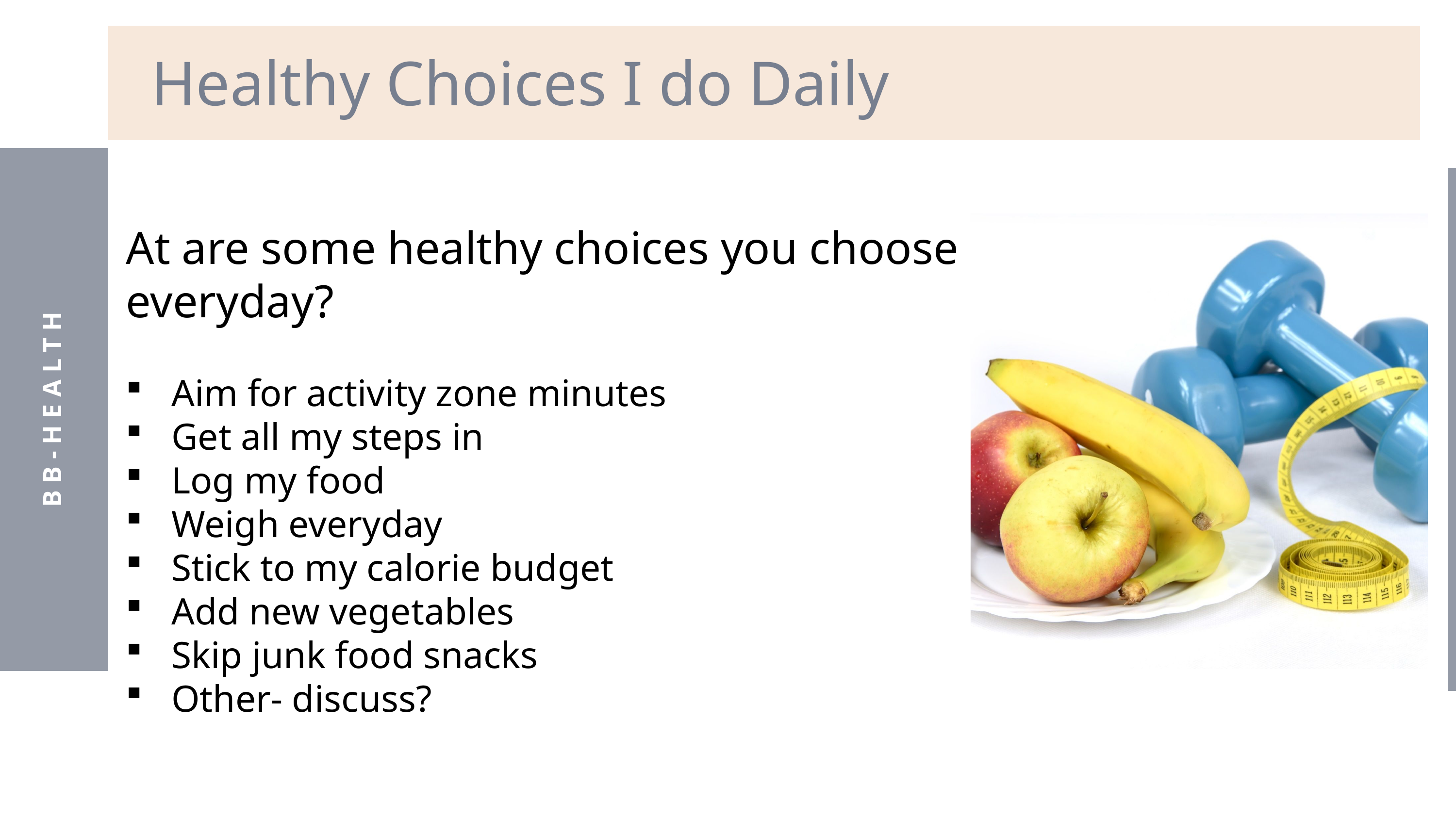

Healthy Choices I do Daily
At are some healthy choices you choose everyday?
Aim for activity zone minutes
Get all my steps in
Log my food
Weigh everyday
Stick to my calorie budget
Add new vegetables
Skip junk food snacks
Other- discuss?
BB-HEALTH

## Slide 13
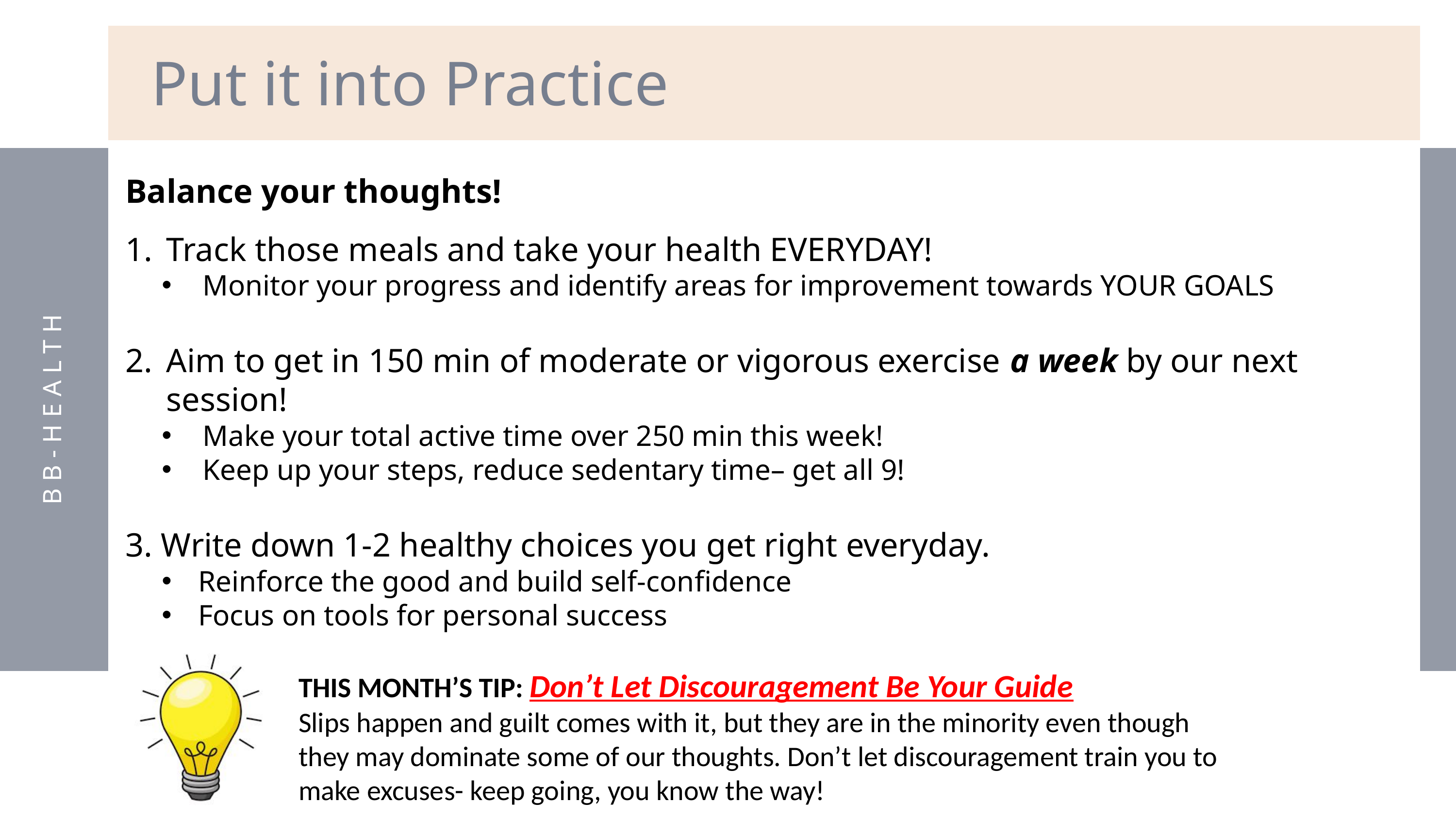

Put it into Practice
Balance your thoughts!
Track those meals and take your health EVERYDAY!
Monitor your progress and identify areas for improvement towards YOUR GOALS
Aim to get in 150 min of moderate or vigorous exercise a week by our next session!
Make your total active time over 250 min this week!
Keep up your steps, reduce sedentary time– get all 9!
3. Write down 1-2 healthy choices you get right everyday.
Reinforce the good and build self-confidence
Focus on tools for personal success
BB-HEALTH
THIS MONTH’S TIP: Don’t Let Discouragement Be Your Guide
Slips happen and guilt comes with it, but they are in the minority even though they may dominate some of our thoughts. Don’t let discouragement train you to make excuses- keep going, you know the way!

## Slide 14
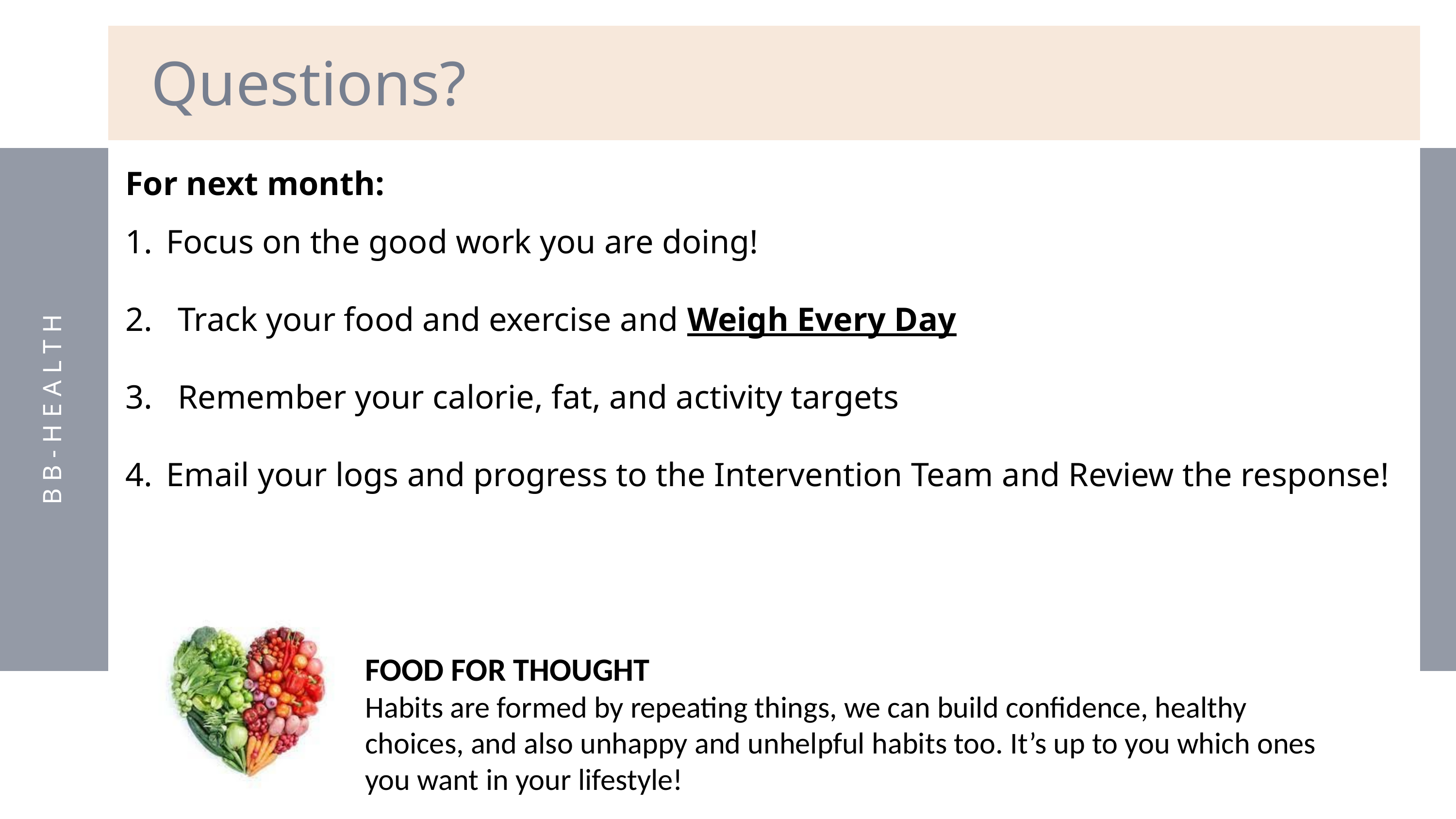

Questions?
For next month:
Focus on the good work you are doing!
2. Track your food and exercise and Weigh Every Day
3. Remember your calorie, fat, and activity targets
Email your logs and progress to the Intervention Team and Review the response!
BB-HEALTH
FOOD FOR THOUGHT
Habits are formed by repeating things, we can build confidence, healthy choices, and also unhappy and unhelpful habits too. It’s up to you which ones you want in your lifestyle!
