# Supplementary material for: Development and Feasibility of an eHealth Diabetes Prevention Program Adapted for Older Adults—Results from a Randomized Control Pilot Study
Source: Nutrients. 2024 Mar 23;16(7):930. doi: 10.3390/nu16070930 (PMC11154527; doi:10.3390/nu16070930)
Supplement: Supplementary file 1 [file nutrients-16-00930-s001.zip › Session20_SUPP.pdf]

BB-HEALTH

e-Healthy Older Adults Modification

Diabetes Prevention Program

Session #20

Balance Your Thoughts

Group Opening Discussion

BB-HEALTH

WELCOME BACK! <u>HOW WAS LAST MONTH</u>?

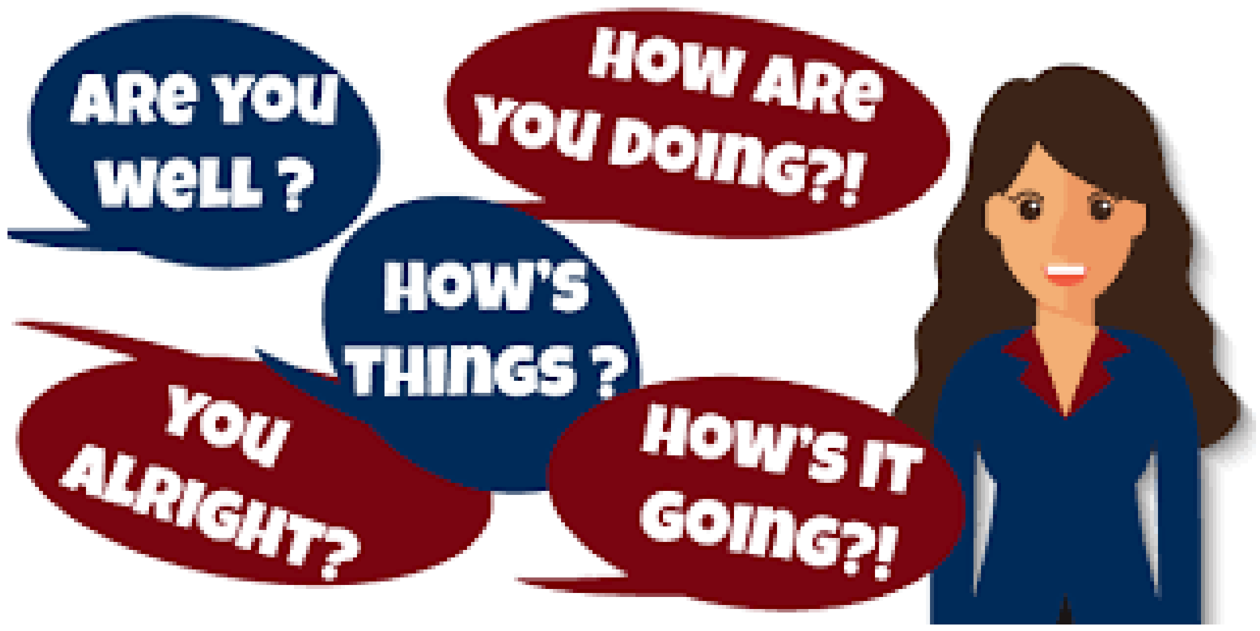

1. Did you track every meal?

2. Did you weigh every day, & record your health today?

3. Did you identify and overcome barriers to activity?

4. Did you email the Intervention Team and review the response?

5. What were your unique challenges and successes?

REMINDER: CHARGE YOUR FITBIT & SCALE DURING THIS MEETING!

How does moving more make you feel?

<u>What does healthy feel like to you? (select all)</u>

- I have more energy after a good walk
- I sleep better, and wake rested
- I am less hungry
- I have had a better mood
- I look forward to my next walk
- My family says they are noticing the difference
- My clothes fit better
- Getting my steps in is getting easier
- Other (discuss)

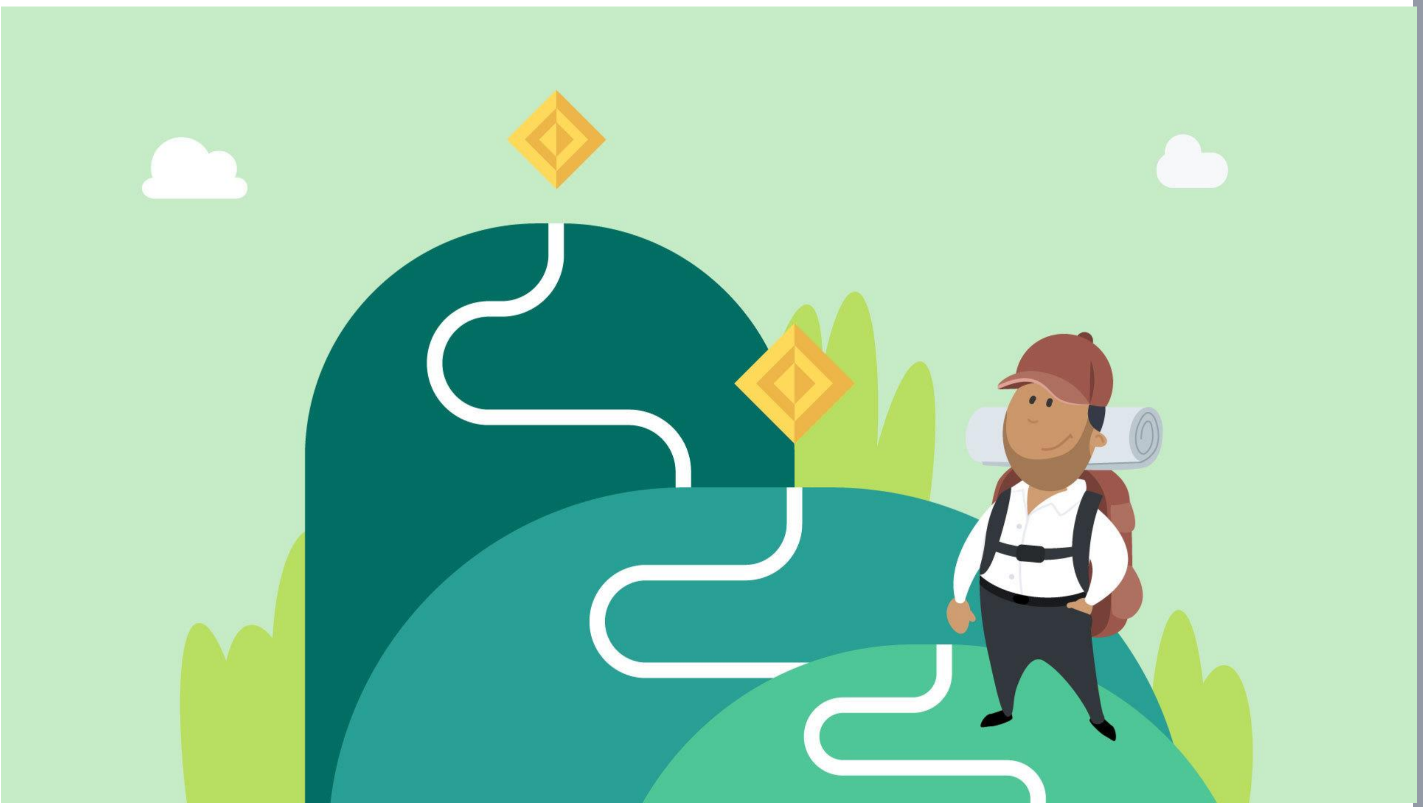

How will I tip the calorie balance this month?

BB-HEALTH

- Drink more water
- Start every meal with a salad
- Take the long way back on a walk
- Jog in place during commercial breaks
- Make a walking date with a friend
- Take an extra loop or two in the grocery store
- Cut out soda and alcohol
- Try a new recipe
- Log my calories and staying on my calorie target
- Getting in my steps
- Weigh myself everyday
- Other (discuss)

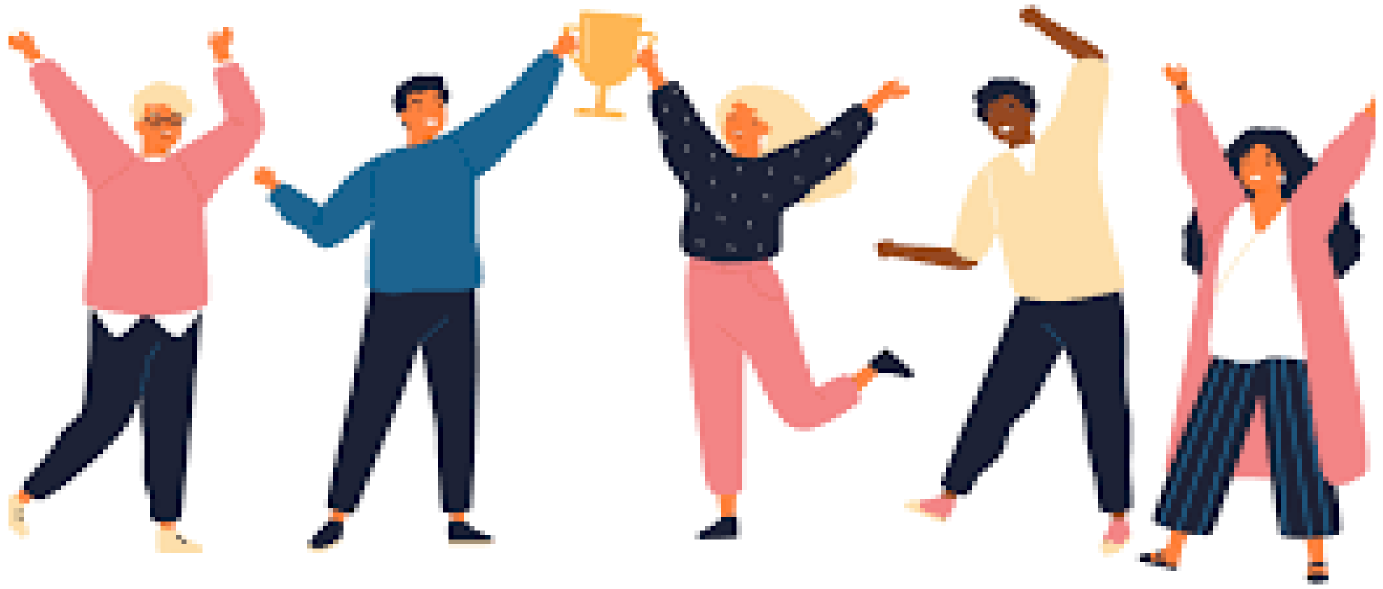

Balance Your Thoughts

BB-HEALTH

You have come a long way!

You should be proud!

The path is not always perfectly straight…
But now you know the way to go

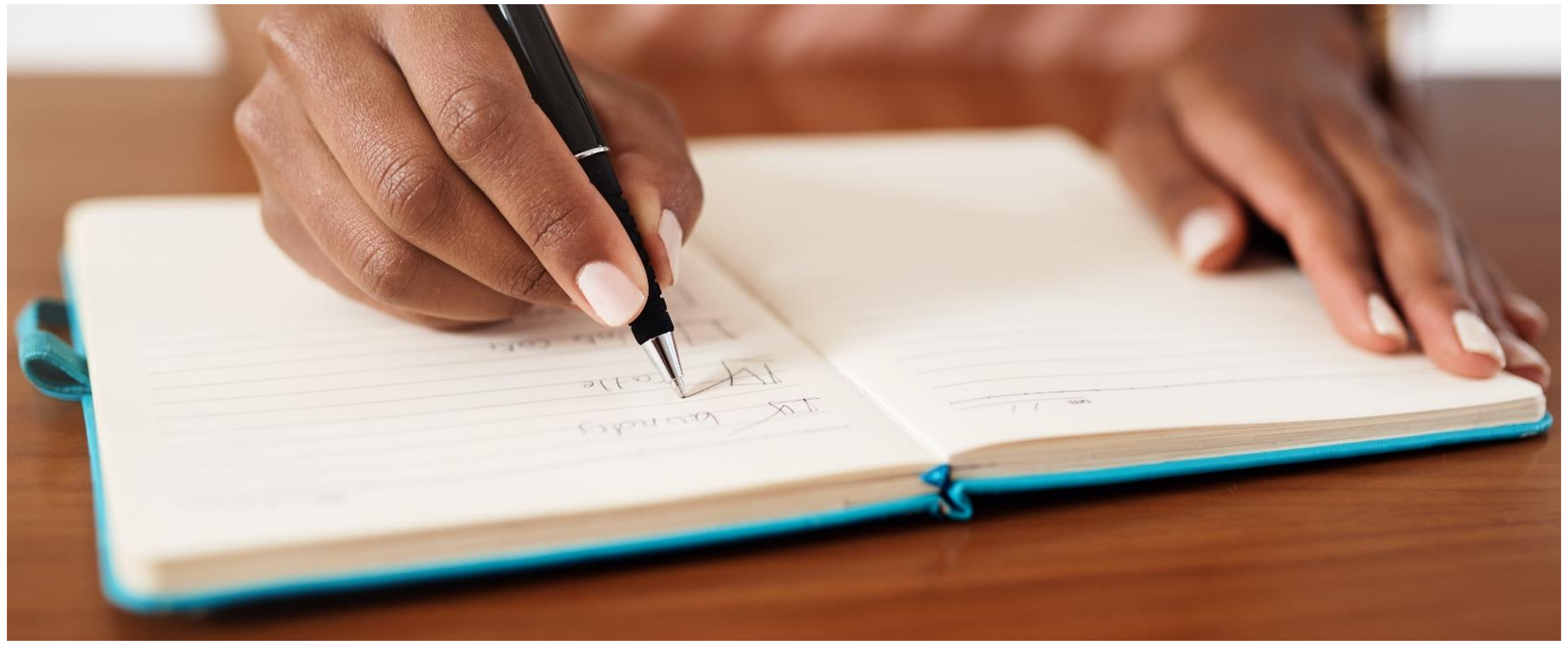

Please Share your experiences
with the group.

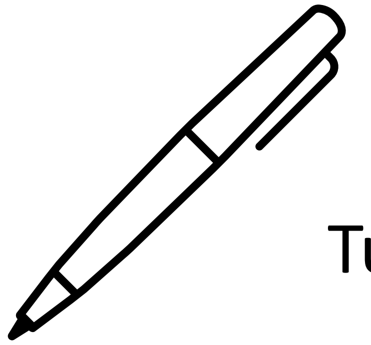

Turn to page 1 in this session's binder

Top 5 Reasons that Keep on Track

BB-HEALTH

Health, Feeling Good, Determination– what keeps you moving towards or maintaining your goals?

- Feel good
- Look Good
- Role model/Take care of others
- Improve Health
- Prevent Disease
- Build self-confidence
- Stay busy
- Stay strong
- Play with grandkids
- Other- discuss

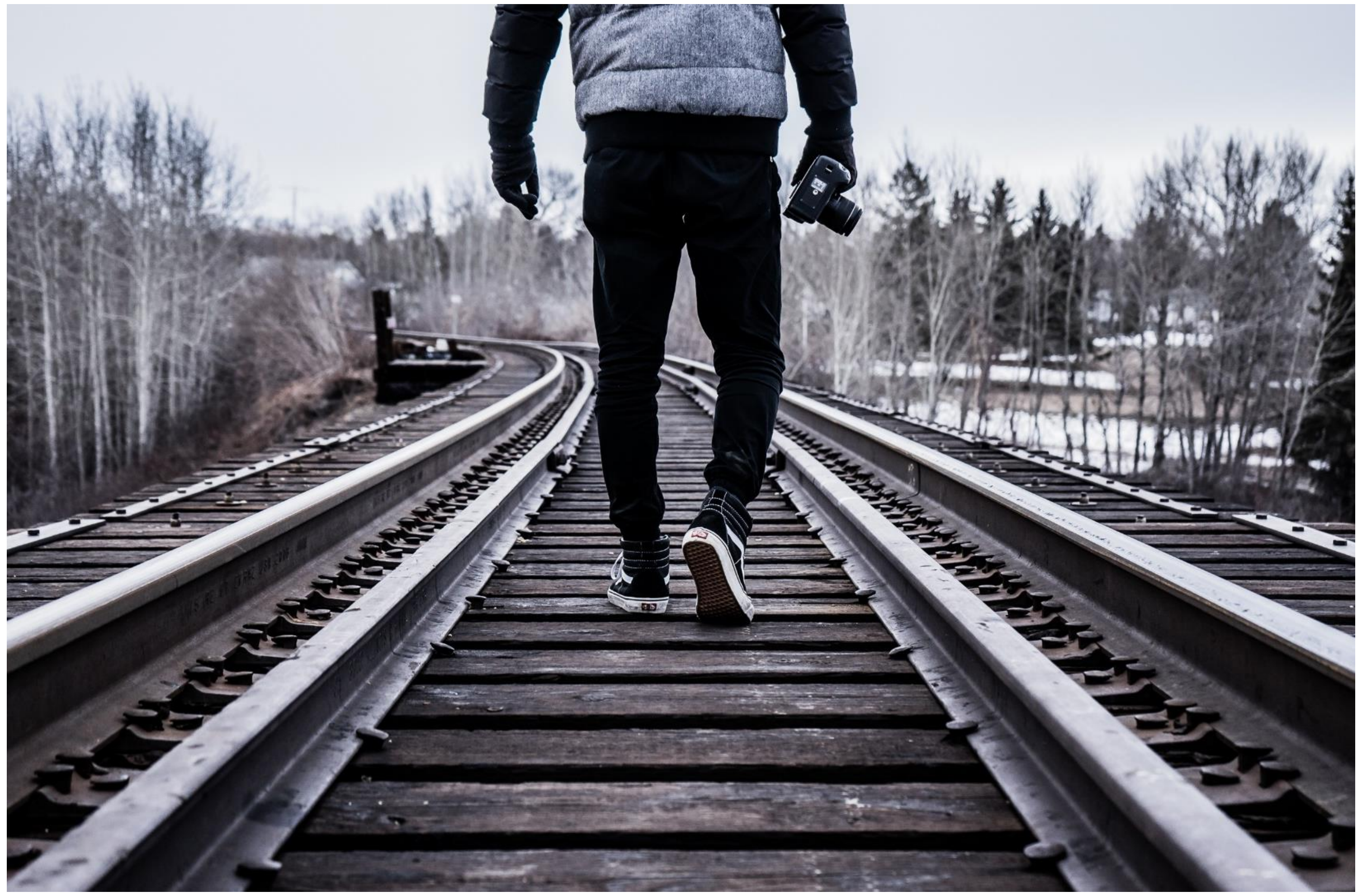

What's your top reason? Now rank the rest so you know what to think about after slips or when facing those obstacles. Keeping these reasons in mind can help us stay on track!

Checking in about Self-Defeating Thoughts

BB-HEALTH

In this program we focus on
Being **AWARE** so that we can be **IN CONTROL**

How do we move from AWARENESS to IN CONTROL when it comes to our self-defeating thoughts??

AWARE of the negative thoughts

Tell the thought to STOP, by talking back to the thought

Change the thought to a more positive one

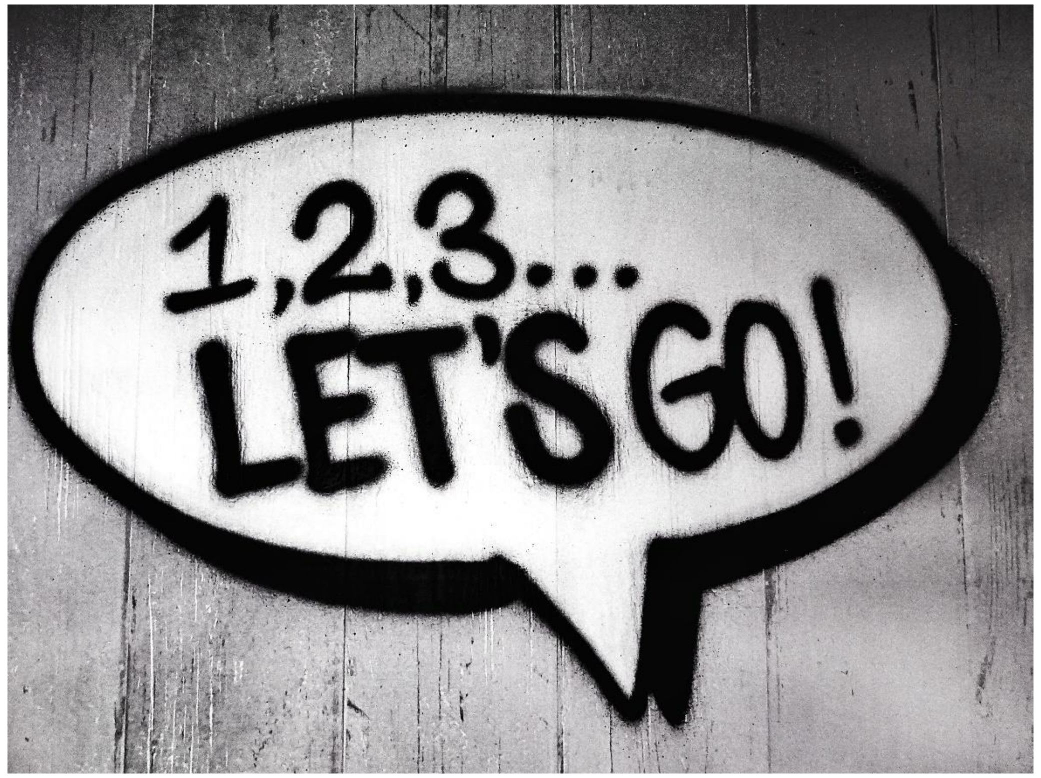

For a refresher on the 123's of overcoming negative thoughts, look over Session #9!

Reinforce Your Good Choices

It’s natural to get down on yourself for slips and mistakes, but we don’t give ourselves enough credit for the majority of the time when we are strong and successful!

Turn to Page 3 and share some of your positive affirmations.

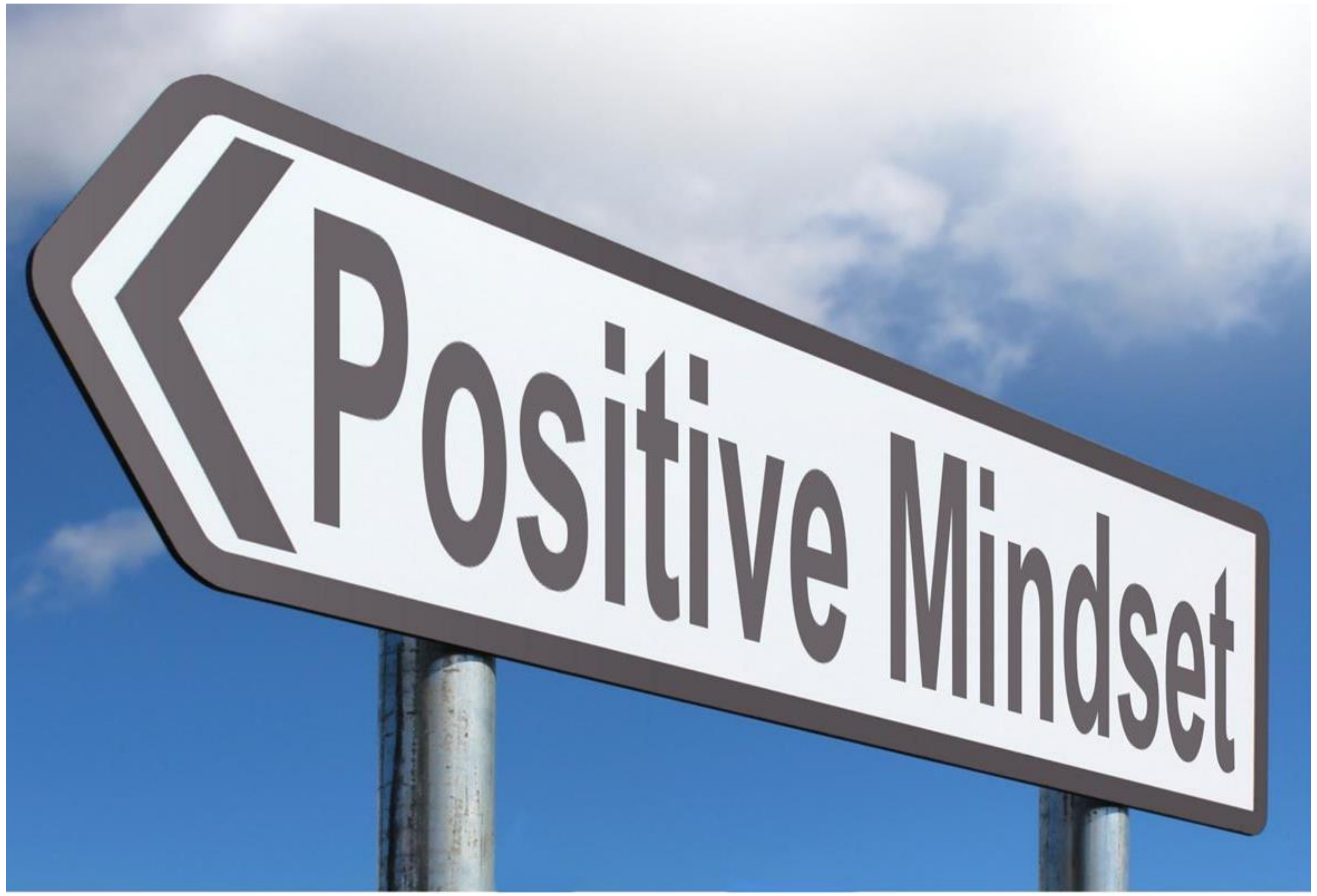

Group Thought Strength Activity

BB-HEALTH

Social Support is an important part of health of body and mind. Sometimes hearing someone else's impression can help you internalize how well you are doing!

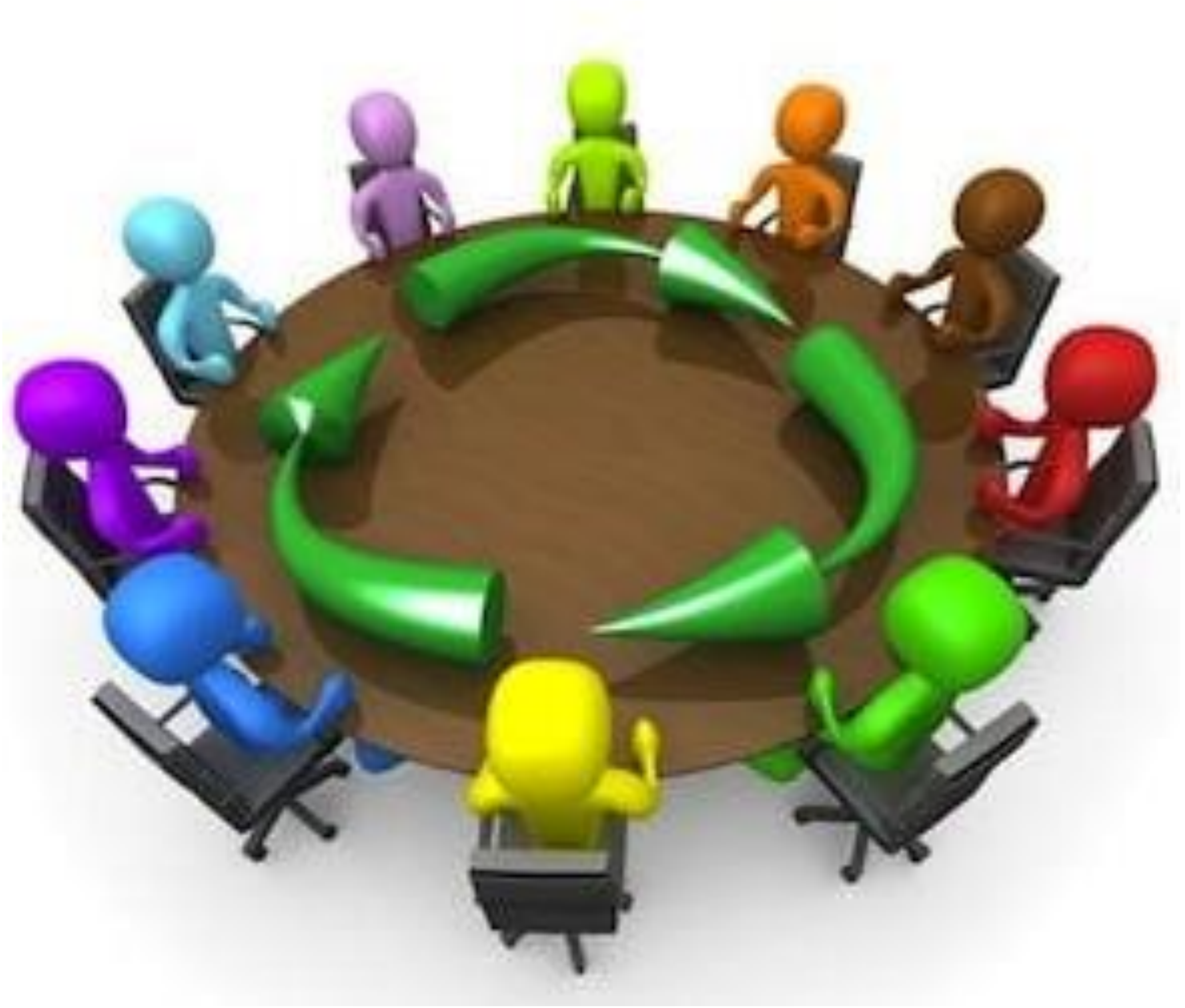

Turn to page 4.

Look for the self-defeating thought most similar to a way you have felt over the last month.

--I will call on you to share your self-defeating thoughts, and then ask a group member to provide a helpful response. We will take turns.

Conquering the "It's Okay, just today" impulse

You should forgive yourself when you have a slip, BUT it's all of our jobs to make sure that doesn't become a free pass to stray.

What are some ways you have justified an unhealthy choice you could have avoided?

<u>What are some ways you have OVERCOME the temptation to say: "It's okay, just today"</u>

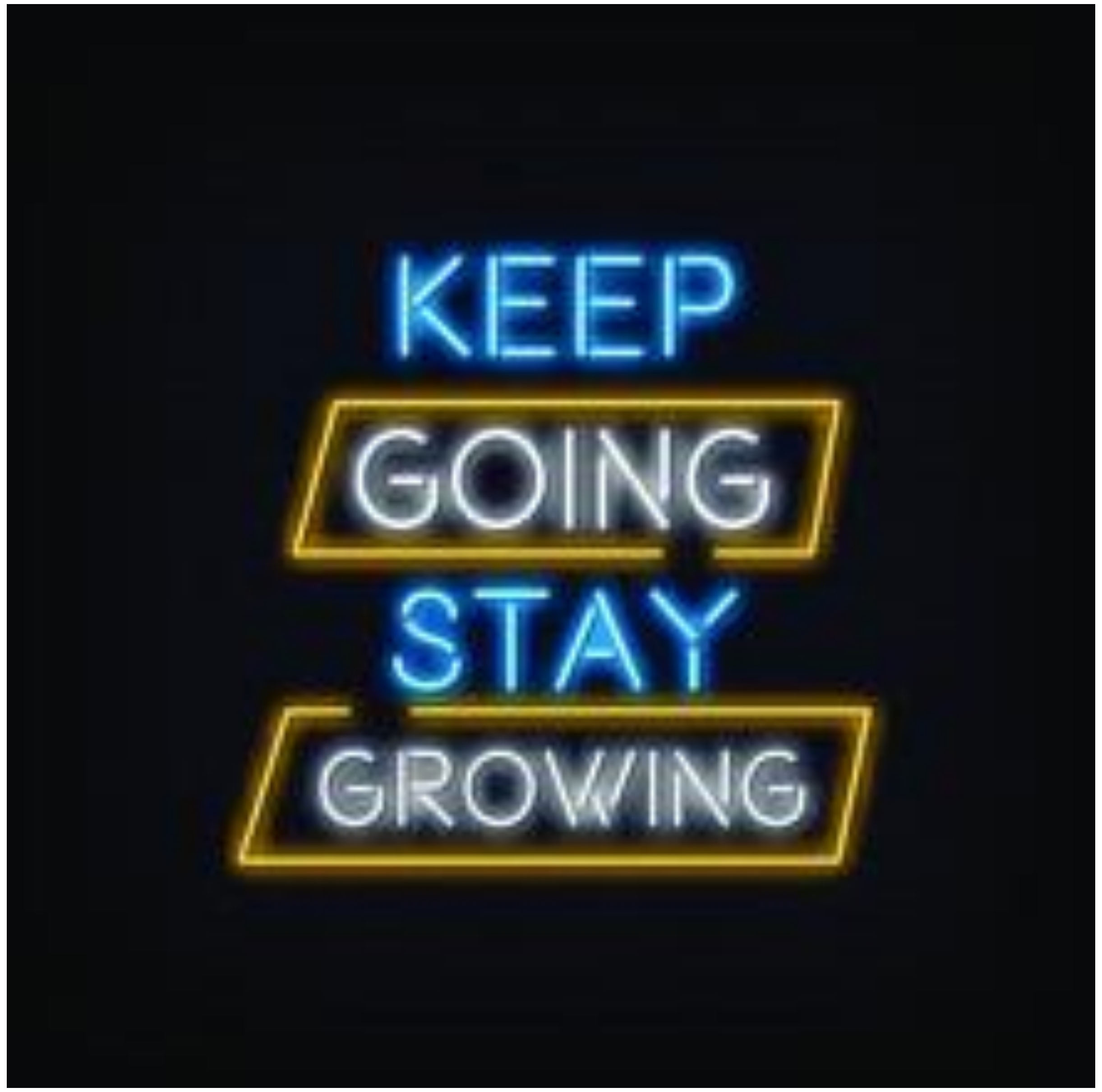

Page 5 has examples of the ways we try to justify a slip, ***on purpose***, sometimes even before it happens.

Breaking the Rationalization Habit

We reinforce our urges and our values everyday with thoughts and actions.

Break the bad cycle and build a better one :

- When you are hungry, grab a healthy snack
  - Satisfying hunger or a craving reinforces it
  - Plan your treats, they aren't gone from your life but should be IMPULSIVE food choices!
- When you make a healthy choice, take a moment to acknowledge how you feel before during and after
  - Let the good choice be a source of self-praise
  - Let the good feelings guide future good habits

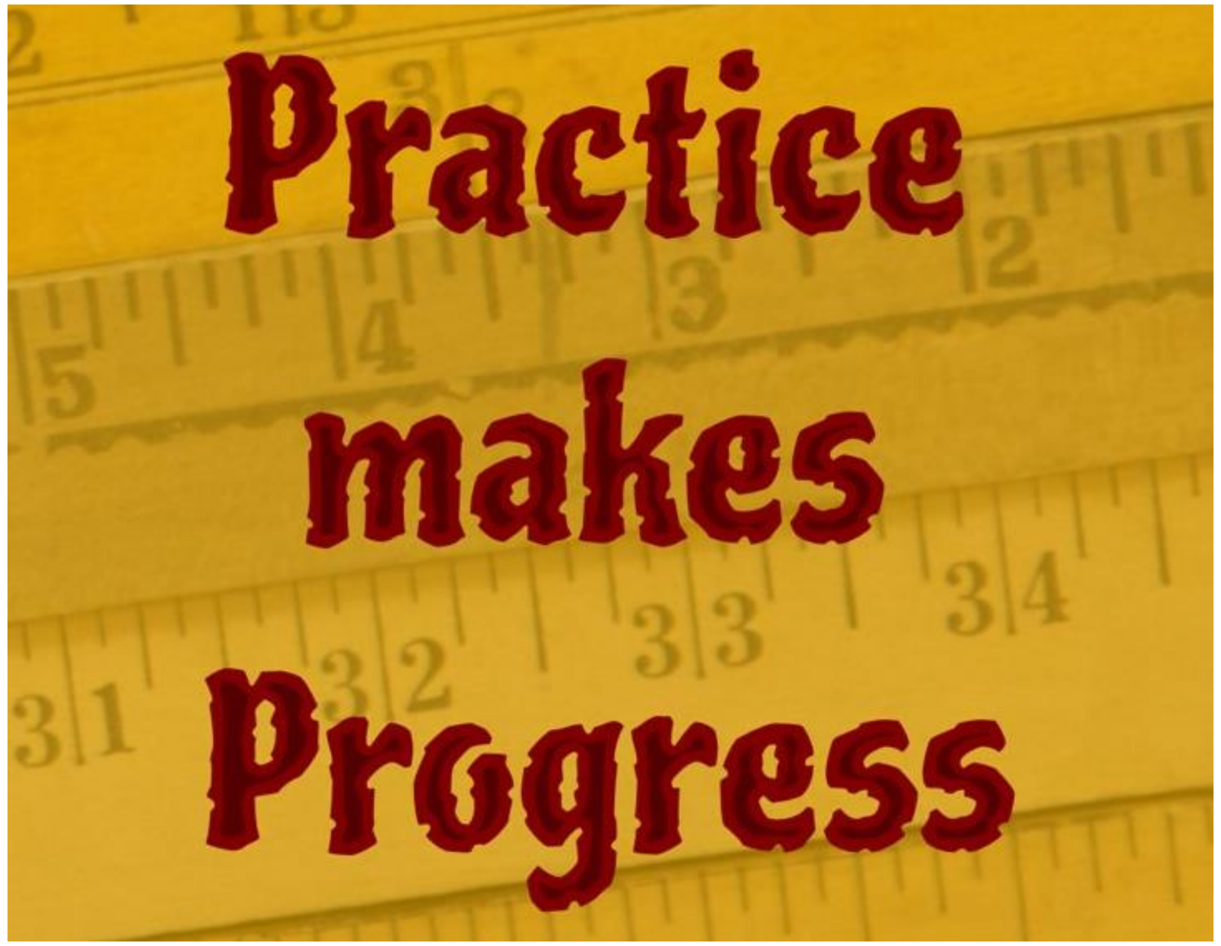

Small wins over time come together to form HEALTHY LIFESTYLES!

Healthy Choices I do Daily

BB-HEALTH

At are some healthy choices you choose everyday?

- Aim for activity zone minutes
- Get all my steps in
- Log my food
- Weigh everyday
- Stick to my calorie budget
- Add new vegetables
- Skip junk food snacks
- Other- discuss?

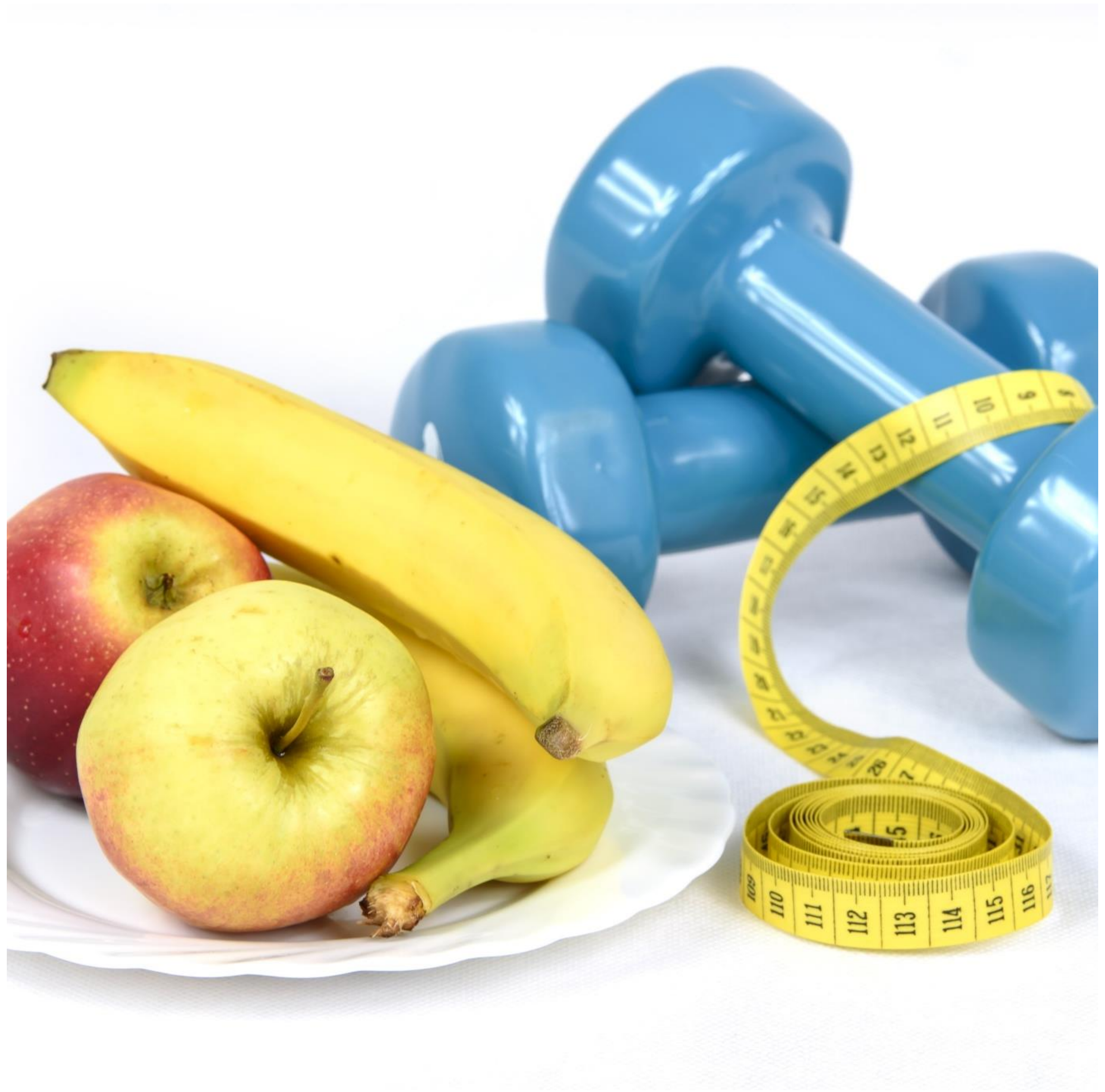

Put it into Practice

BB-HEALTH

Balance your thoughts!

1. Track those meals and take your health EVERYDAY!
   - Monitor your progress and identify areas for improvement towards YOUR GOALS

2. Aim to get in 150 min of moderate or vigorous exercise ***a week*** by our next session!
   - Make your total active time over 250 min this week!
   - Keep up your steps, reduce sedentary time– get all 9!

3. Write down 1-2 healthy choices you get right everyday.
   - Reinforce the good and build self-confidence
   - Focus on tools for personal success

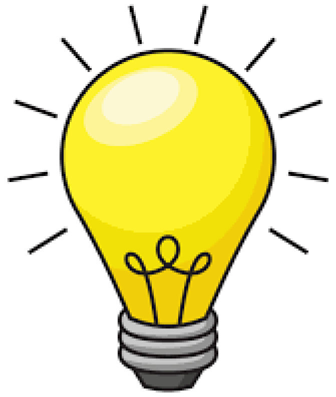

THIS MONTH'S TIP: ***Don't Let Discouragement Be Your Guide***
Slips happen and guilt comes with it, but they are in the minority even though they may dominate some of our thoughts. Don't let discouragement train you to make excuses- keep going, you know the way!

Questions?

B B - H E A L T H

For next month:

1. Focus on the good work you are doing!

2. Track your food and exercise and **<u>Weigh Every Day</u>**

3. Remember your calorie, fat, and activity targets

4. Email your logs and progress to the Intervention Team and Review the response!

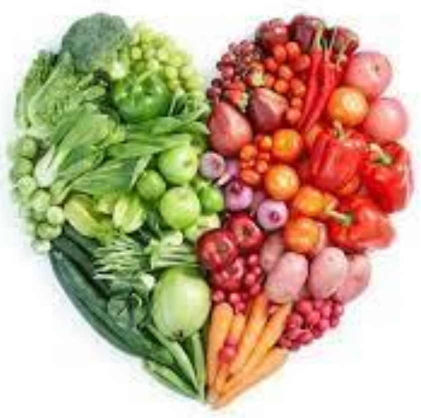

FOOD FOR THOUGHT

Habits are formed by repeating things, we can build confidence, healthy choices, and also unhappy and unhelpful habits too. It’s up to you which ones you want in your lifestyle!
