# Supplementary material for: Development and Feasibility of an eHealth Diabetes Prevention Program Adapted for Older Adults—Results from a Randomized Control Pilot Study
Source: Nutrients. 2024 Mar 23;16(7):930. doi: 10.3390/nu16070930 (PMC11154527; doi:10.3390/nu16070930)
Supplement: Supplementary file 1 [file nutrients-16-00930-s001.zip › Week1.pptx]

## Slide 1
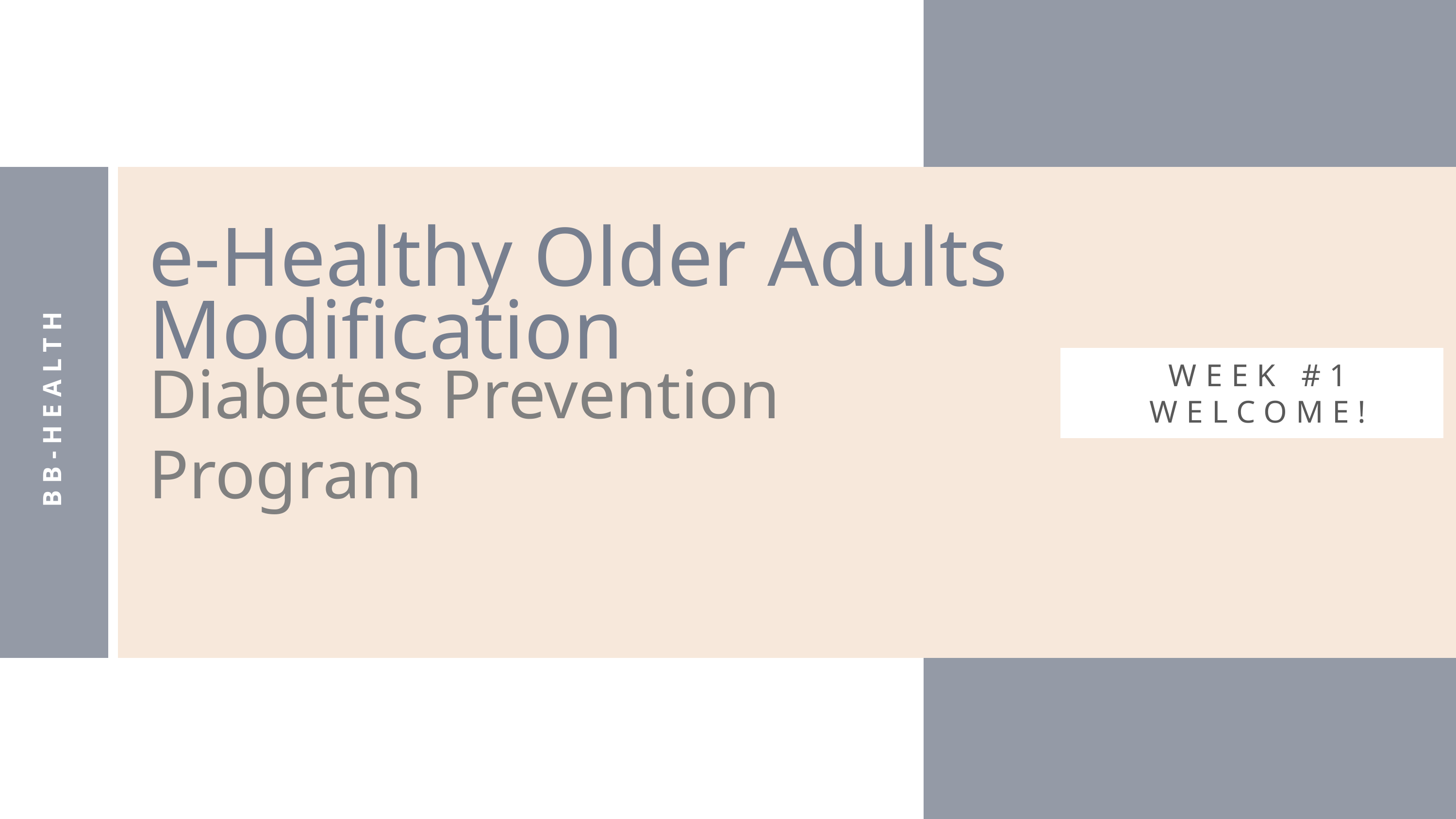

OPEN REPORTS
e-Healthy Older Adults Modification
WEEK #1
WELCOME!
Diabetes Prevention Program
BB-HEALTH

## Slide 2
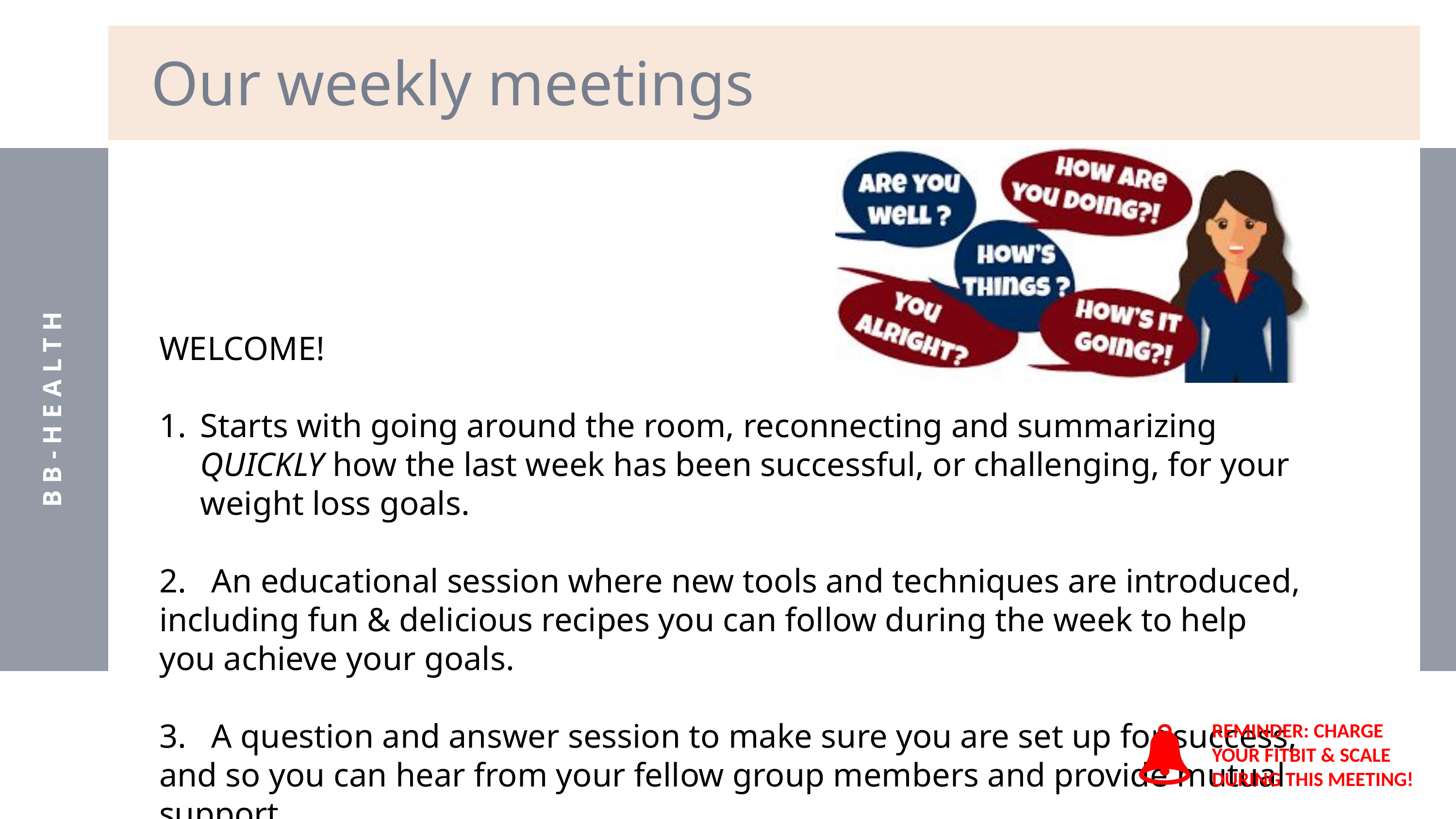

Our weekly meetings
WELCOME!
Starts with going around the room, reconnecting and summarizing QUICKLY how the last week has been successful, or challenging, for your weight loss goals.
2. An educational session where new tools and techniques are introduced, including fun & delicious recipes you can follow during the week to help you achieve your goals.
3. A question and answer session to make sure you are set up for success, and so you can hear from your fellow group members and provide mutual support.
BB-HEALTH
REMINDER: CHARGE YOUR FITBIT & SCALE DURING THIS MEETING!

## Slide 3
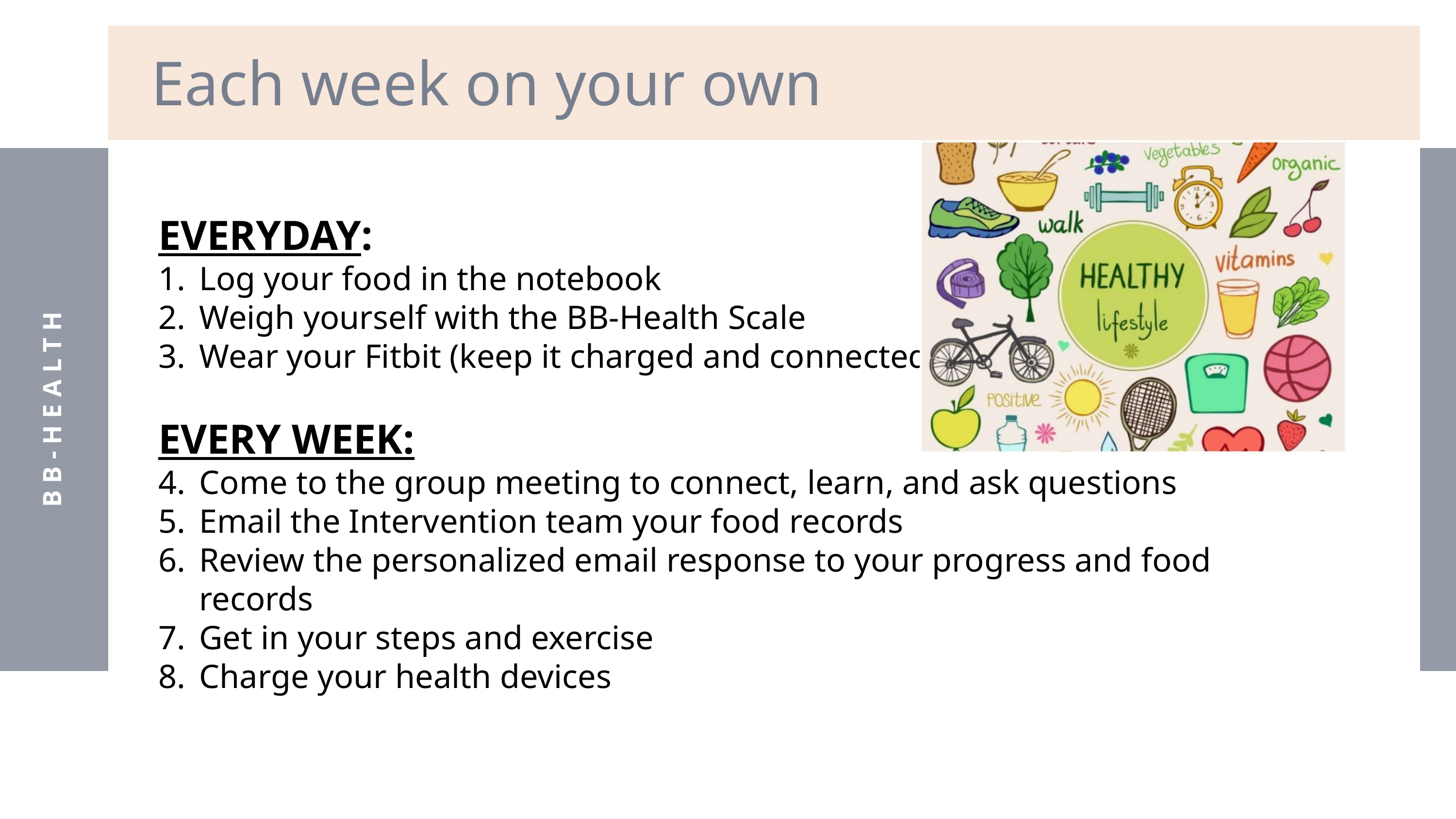

Each week on your own
EVERYDAY:
Log your food in the notebook
Weigh yourself with the BB-Health Scale
Wear your Fitbit (keep it charged and connected)
EVERY WEEK:
Come to the group meeting to connect, learn, and ask questions
Email the Intervention team your food records
Review the personalized email response to your progress and food records
Get in your steps and exercise
Charge your health devices
BB-HEALTH

## Slide 4
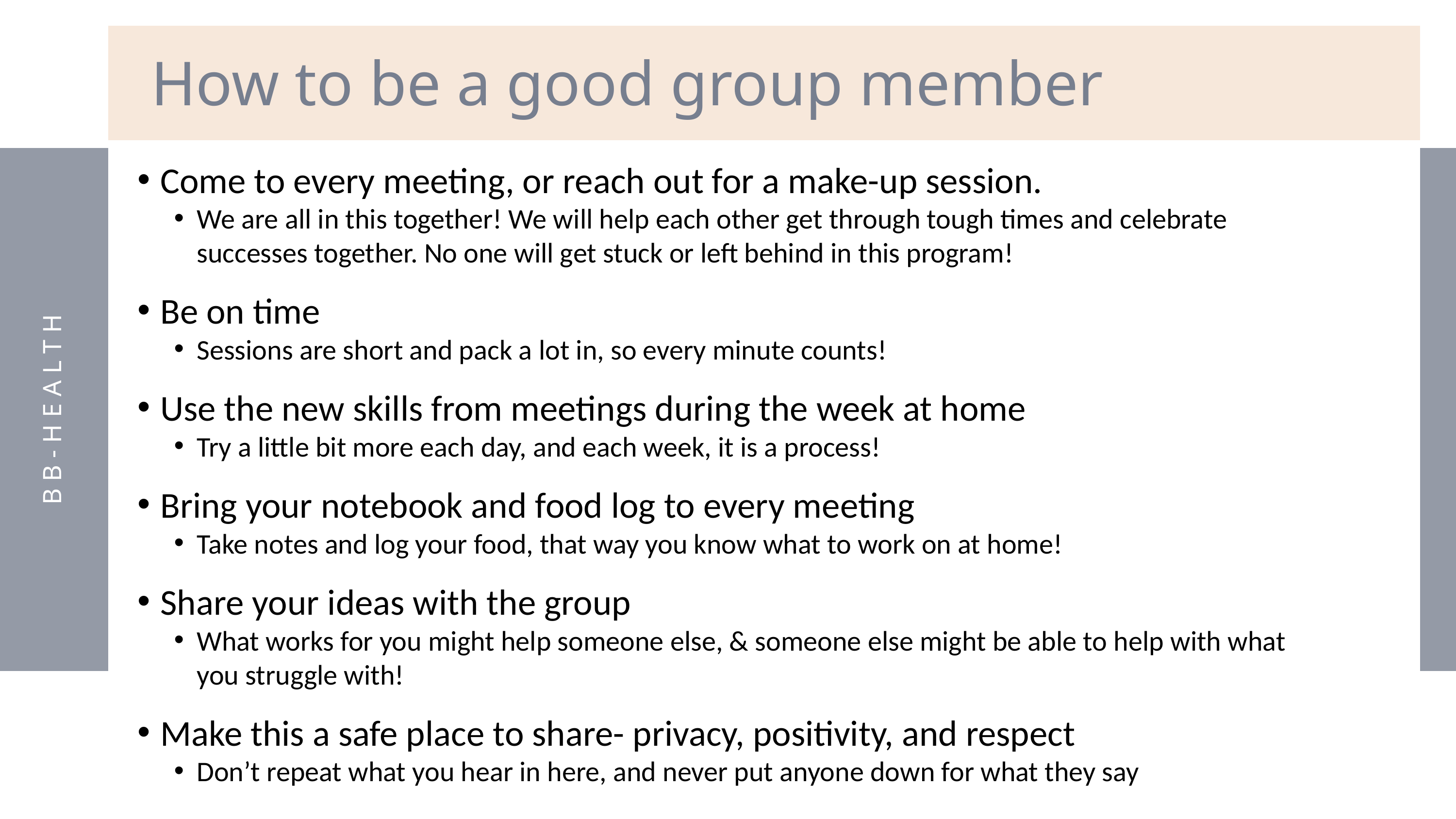

How to be a good group member
Come to every meeting, or reach out for a make-up session.
We are all in this together! We will help each other get through tough times and celebrate successes together. No one will get stuck or left behind in this program!
Be on time
Sessions are short and pack a lot in, so every minute counts!
Use the new skills from meetings during the week at home
Try a little bit more each day, and each week, it is a process!
Bring your notebook and food log to every meeting
Take notes and log your food, that way you know what to work on at home!
Share your ideas with the group
What works for you might help someone else, & someone else might be able to help with what you struggle with!
Make this a safe place to share- privacy, positivity, and respect
Don’t repeat what you hear in here, and never put anyone down for what they say
BB-HEALTH

## Slide 5
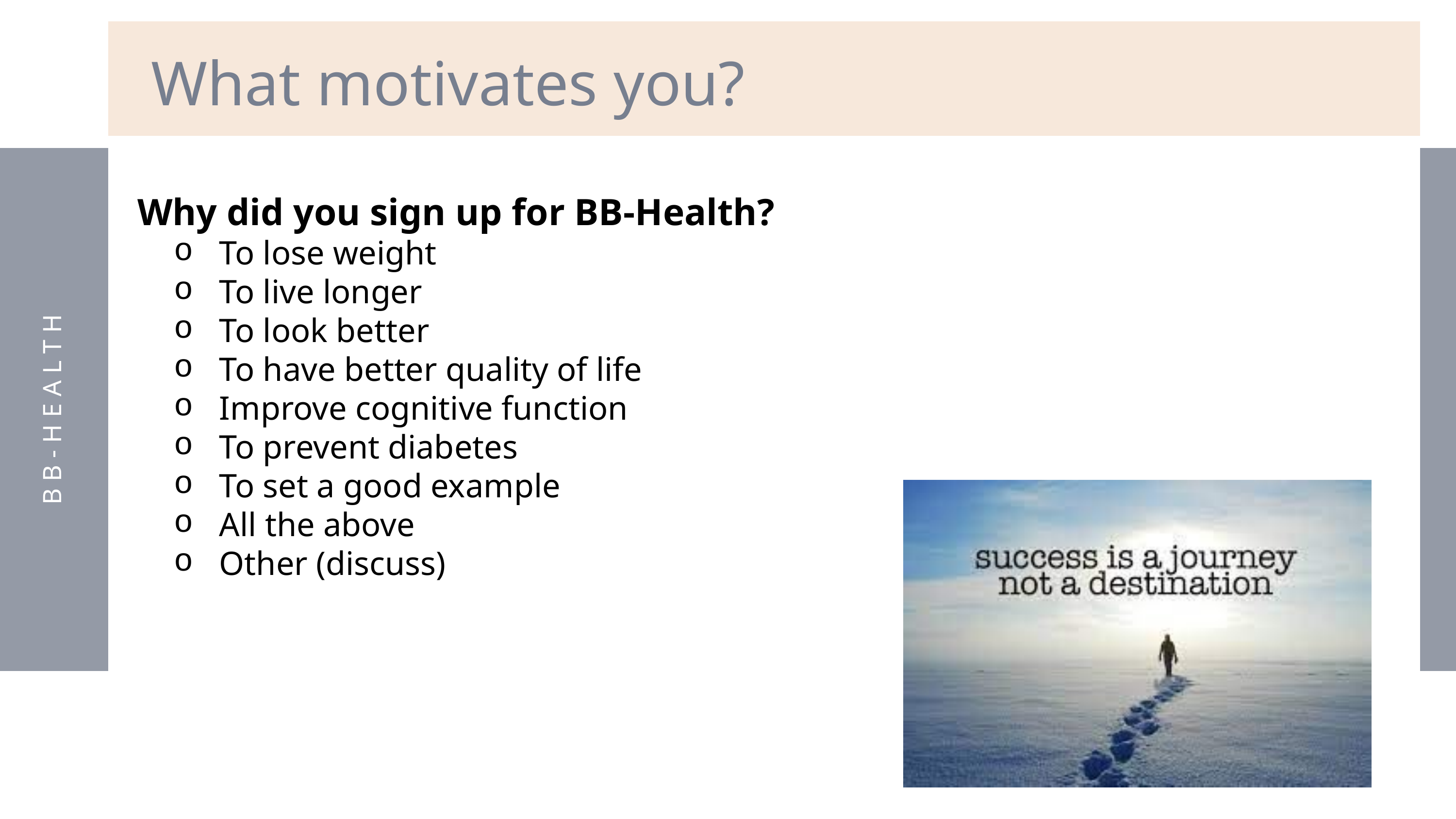

What motivates you?
Why did you sign up for BB-Health?
To lose weight
To live longer
To look better
To have better quality of life
Improve cognitive function
To prevent diabetes
To set a good example
All the above
Other (discuss)
BB-HEALTH

## Slide 6
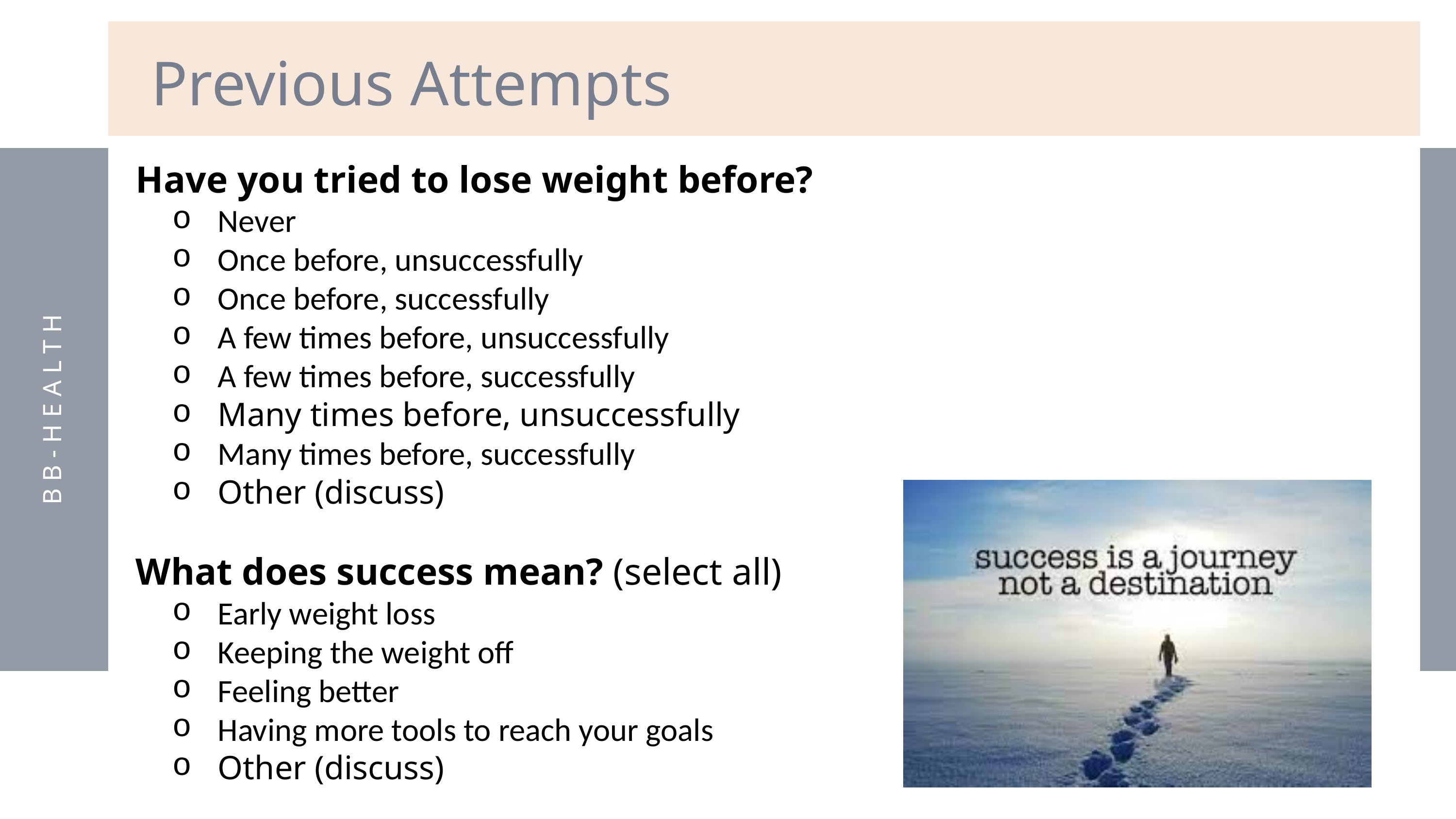

Previous Attempts
Have you tried to lose weight before?
Never
Once before, unsuccessfully
Once before, successfully
A few times before, unsuccessfully
A few times before, successfully
Many times before, unsuccessfully
Many times before, successfully
Other (discuss)
BB-HEALTH
What does success mean? (select all)
Early weight loss
Keeping the weight off
Feeling better
Having more tools to reach your goals
Other (discuss)

## Slide 7
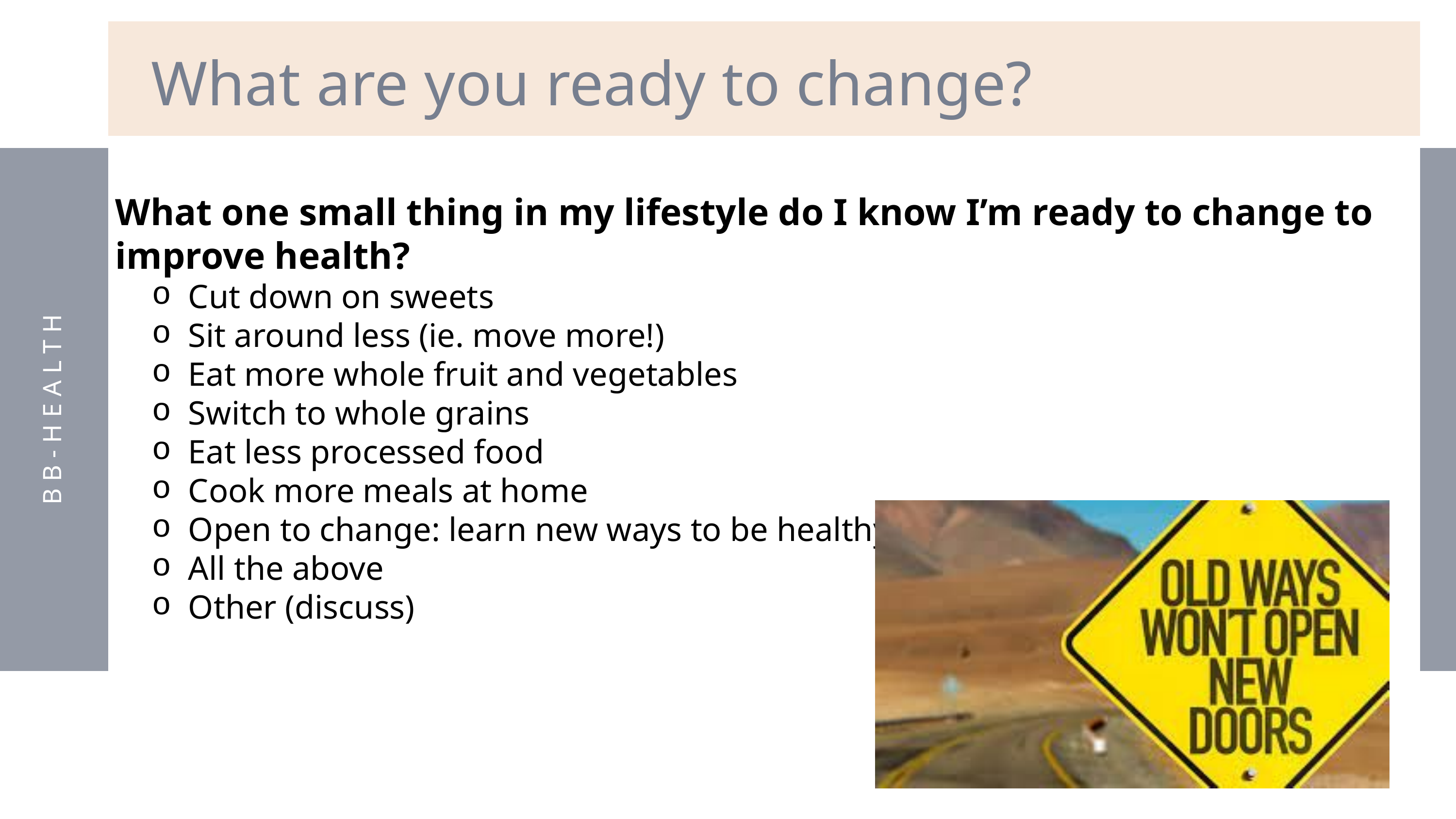

What are you ready to change?
What one small thing in my lifestyle do I know I’m ready to change to improve health?
Cut down on sweets
Sit around less (ie. move more!)
Eat more whole fruit and vegetables
Switch to whole grains
Eat less processed food
Cook more meals at home
Open to change: learn new ways to be healthy
All the above
Other (discuss)
BB-HEALTH

## Slide 8
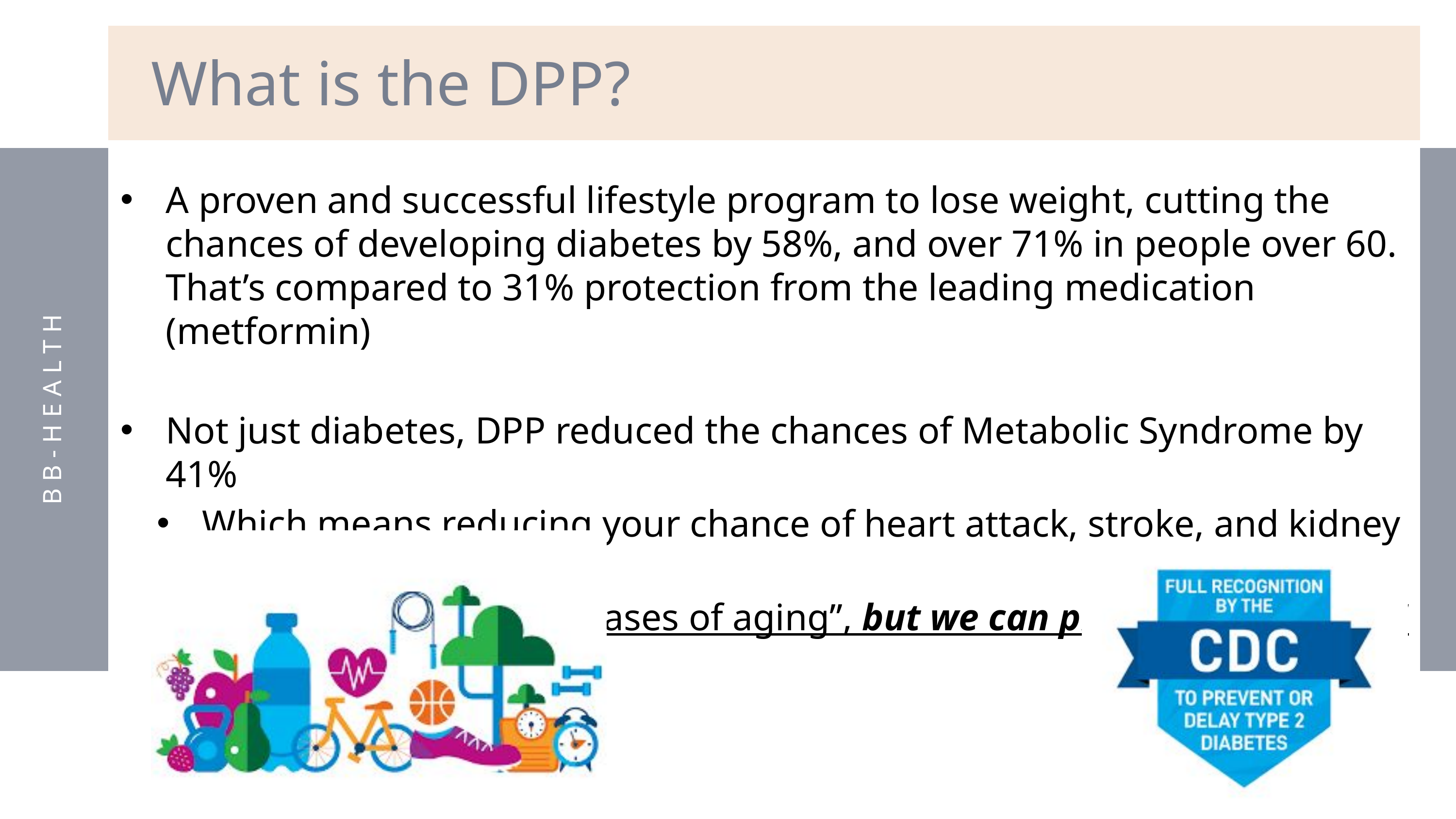

STUDY PROTOCOL: JUSTIFICATION
What is the DPP?
A proven and successful lifestyle program to lose weight, cutting the chances of developing diabetes by 58%, and over 71% in people over 60. That’s compared to 31% protection from the leading medication (metformin)
Not just diabetes, DPP reduced the chances of Metabolic Syndrome by 41%
Which means reducing your chance of heart attack, stroke, and kidney disease
These are called “diseases of aging”, but we can prove them WRONG!
BB-HEALTH
BB-HEALTH

## Slide 9
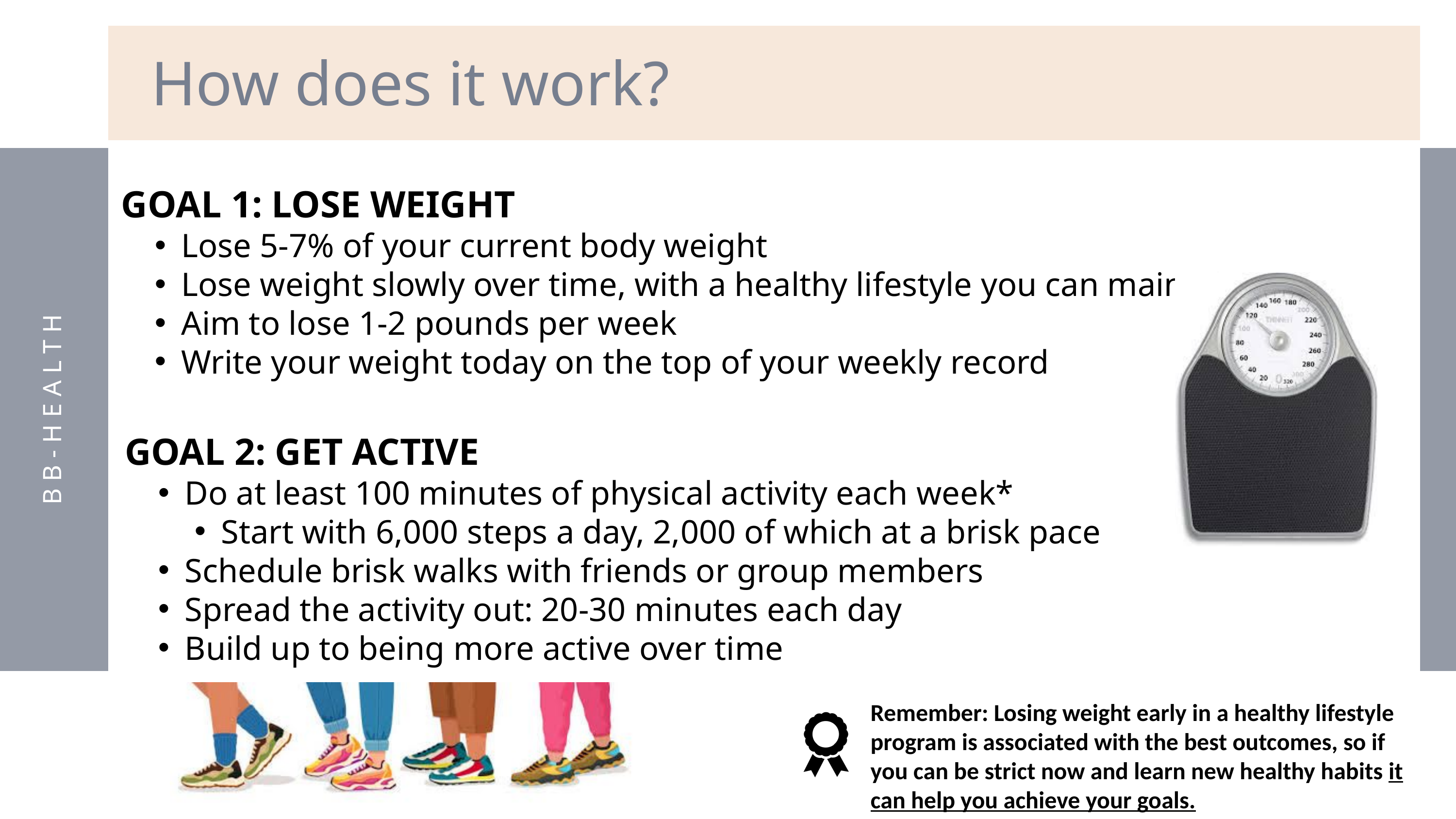

How does it work?
GOAL 1: LOSE WEIGHT
Lose 5-7% of your current body weight
Lose weight slowly over time, with a healthy lifestyle you can maintain
Aim to lose 1-2 pounds per week
Write your weight today on the top of your weekly record
BB-HEALTH
BB-HEALTH
GOAL 2: GET ACTIVE
Do at least 100 minutes of physical activity each week*
Start with 6,000 steps a day, 2,000 of which at a brisk pace
Schedule brisk walks with friends or group members
Spread the activity out: 20-30 minutes each day
Build up to being more active over time
Remember: Losing weight early in a healthy lifestyle program is associated with the best outcomes, so if you can be strict now and learn new healthy habits it can help you achieve your goals.

## Slide 10
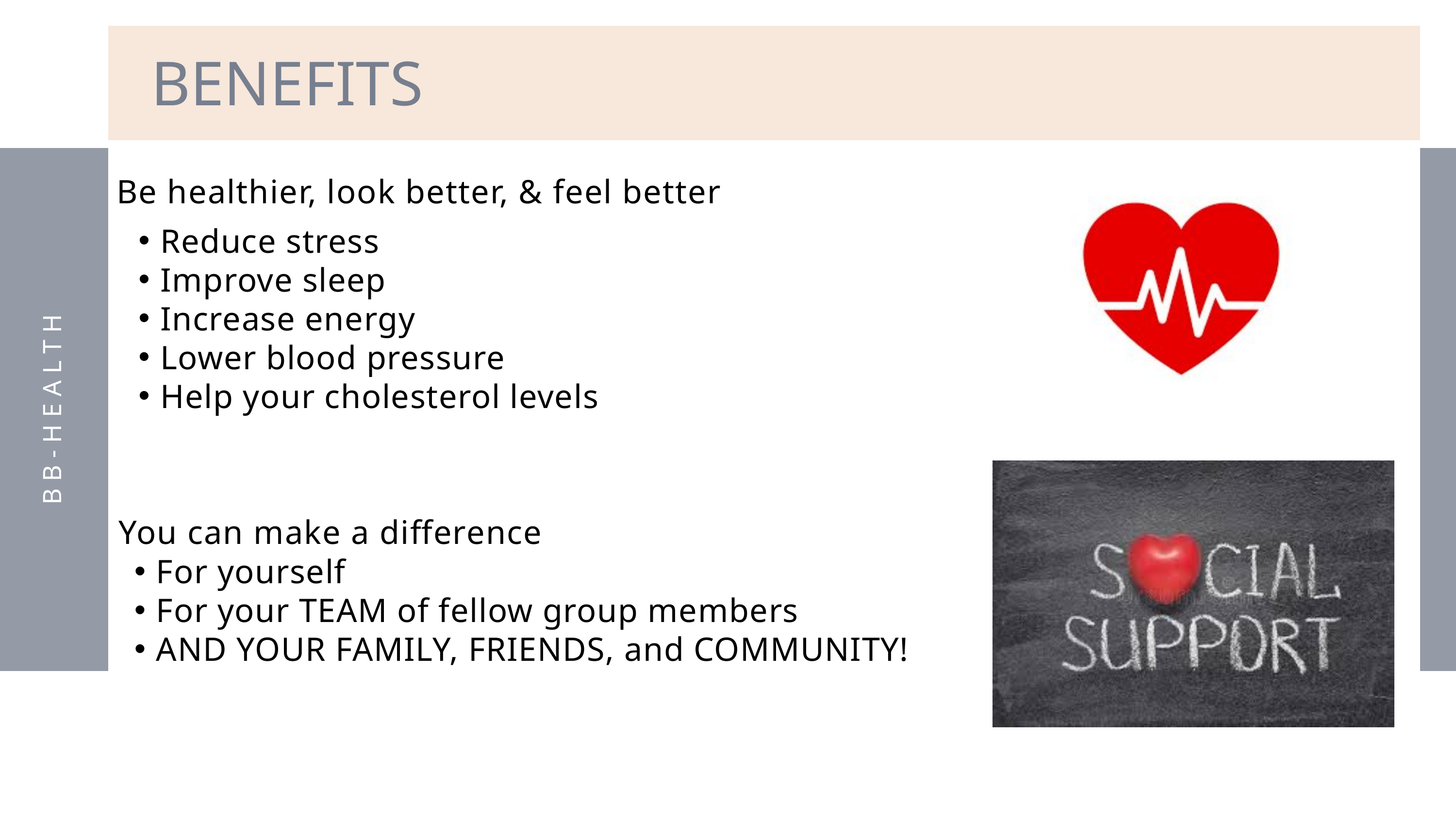

BENEFITS
Be healthier, look better, & feel better
Reduce stress
Improve sleep
Increase energy
Lower blood pressure
Help your cholesterol levels
BB-HEALTH
You can make a difference
For yourself
For your TEAM of fellow group members
AND YOUR FAMILY, FRIENDS, and COMMUNITY!

## Slide 11
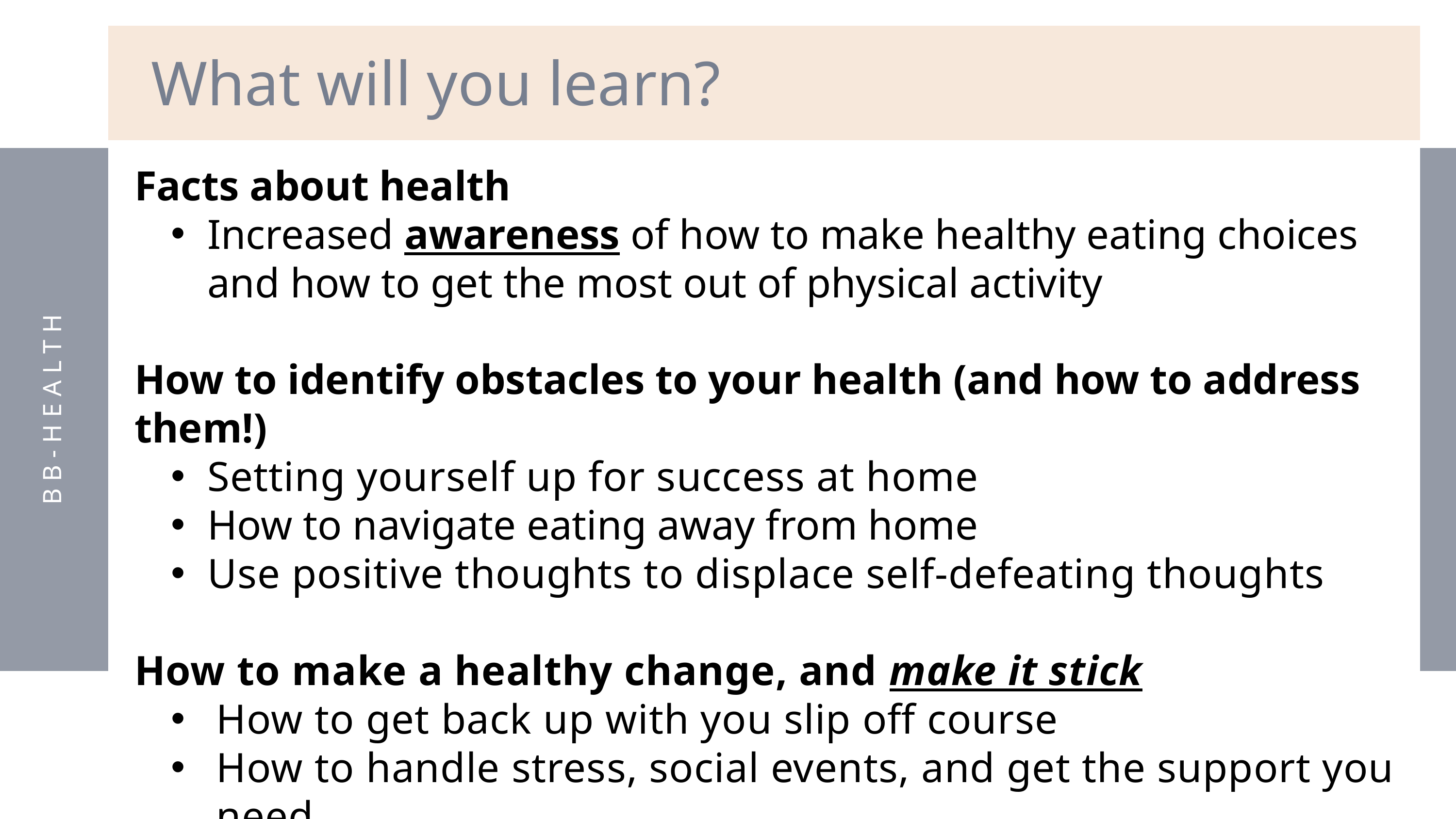

What will you learn?
Facts about health
Increased awareness of how to make healthy eating choices and how to get the most out of physical activity
How to identify obstacles to your health (and how to address them!)
Setting yourself up for success at home
How to navigate eating away from home
Use positive thoughts to displace self-defeating thoughts
How to make a healthy change, and make it stick
How to get back up with you slip off course
How to handle stress, social events, and get the support you need
BB-HEALTH

## Slide 12
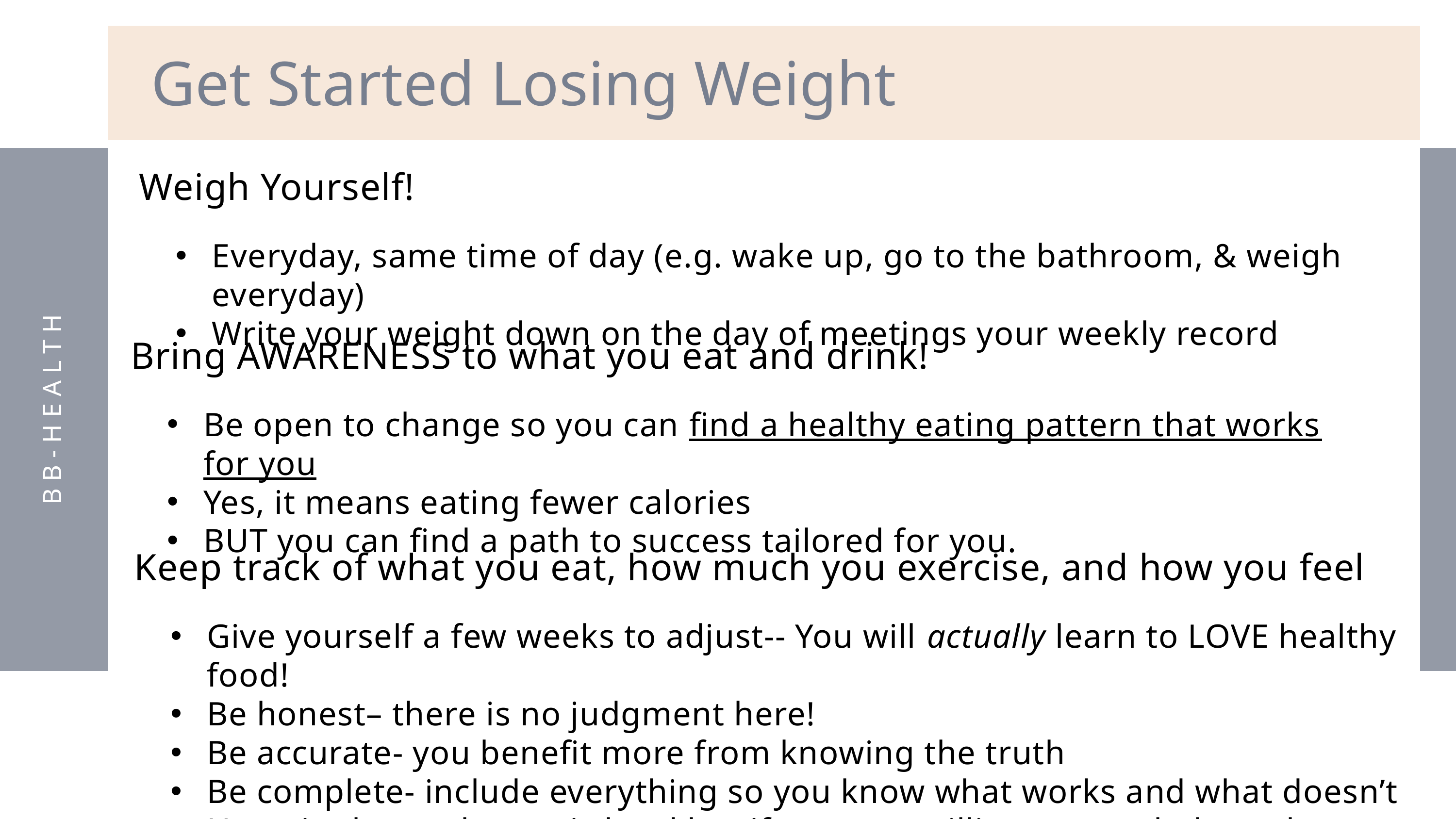

Get Started Losing Weight
Weigh Yourself!
Everyday, same time of day (e.g. wake up, go to the bathroom, & weigh everyday)
Write your weight down on the day of meetings your weekly record
Bring AWARENESS to what you eat and drink!
Be open to change so you can find a healthy eating pattern that works for you
Yes, it means eating fewer calories
BUT you can find a path to success tailored for you.
BB-HEALTH
Keep track of what you eat, how much you exercise, and how you feel
Give yourself a few weeks to adjust-- You will actually learn to LOVE healthy food!
Be honest– there is no judgment here!
Be accurate- you benefit more from knowing the truth
Be complete- include everything so you know what works and what doesn’t
Hang in there- change is hard but if you stay willing we can help each other!

## Slide 13
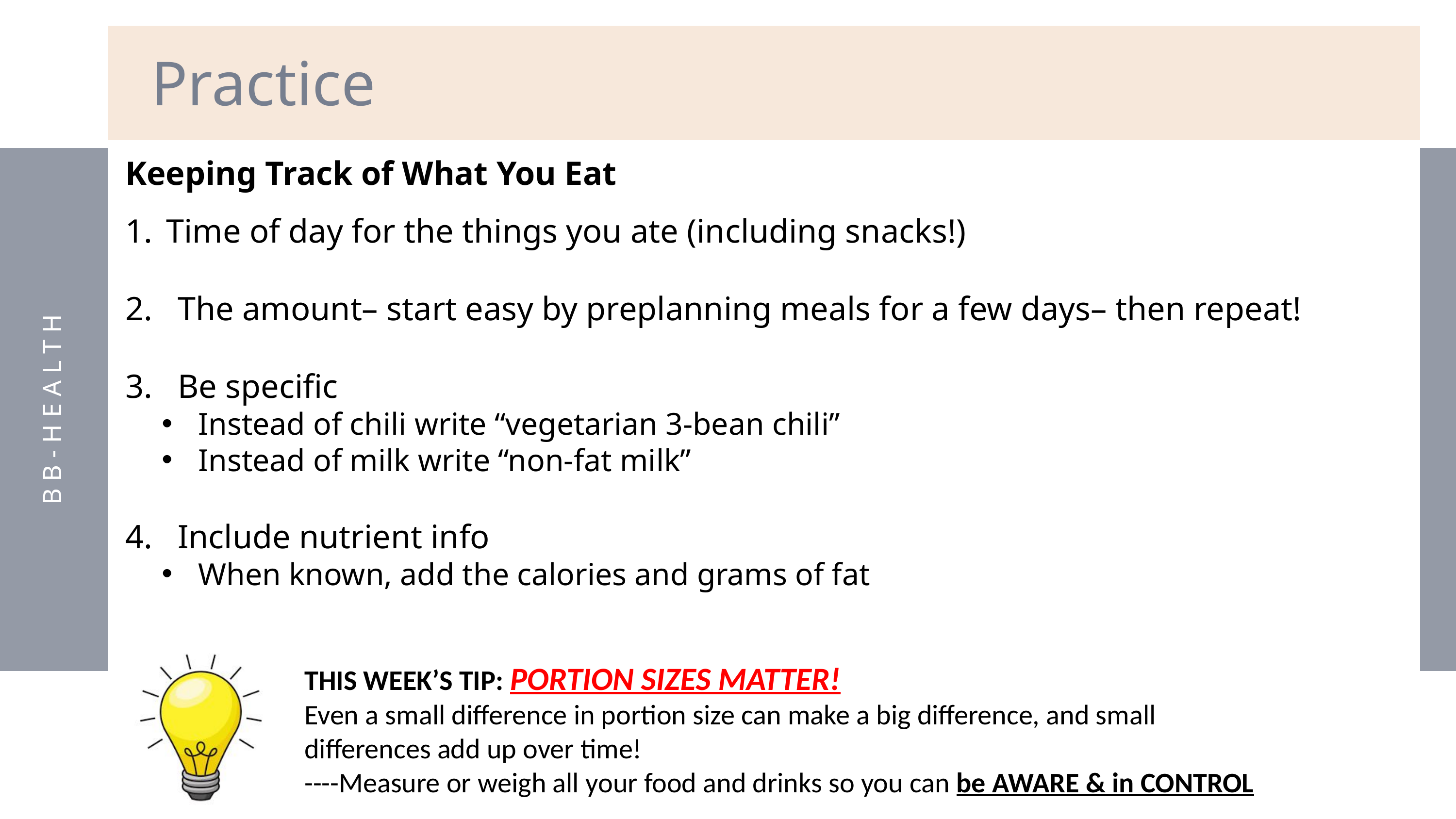

Practice
Keeping Track of What You Eat
Time of day for the things you ate (including snacks!)
2. The amount– start easy by preplanning meals for a few days– then repeat!
3. Be specific
Instead of chili write “vegetarian 3-bean chili”
Instead of milk write “non-fat milk”
4. Include nutrient info
When known, add the calories and grams of fat
BB-HEALTH
THIS WEEK’S TIP: PORTION SIZES MATTER!
Even a small difference in portion size can make a big difference, and small differences add up over time!
----Measure or weigh all your food and drinks so you can be AWARE & in CONTROL

## Slide 14
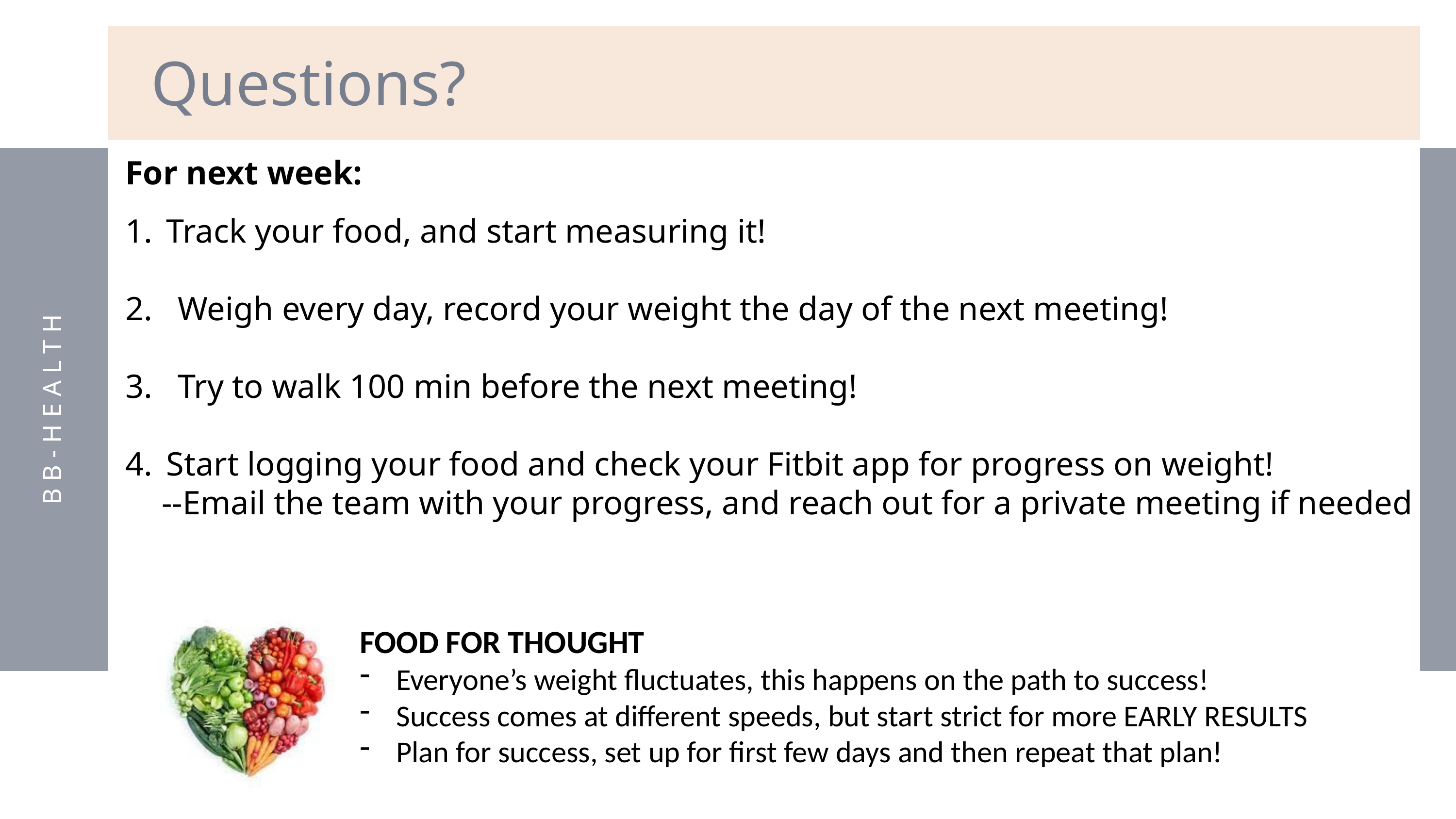

Questions?
For next week:
Track your food, and start measuring it!
2. Weigh every day, record your weight the day of the next meeting!
3. Try to walk 100 min before the next meeting!
Start logging your food and check your Fitbit app for progress on weight!
--Email the team with your progress, and reach out for a private meeting if needed
BB-HEALTH
FOOD FOR THOUGHT
Everyone’s weight fluctuates, this happens on the path to success!
Success comes at different speeds, but start strict for more EARLY RESULTS
Plan for success, set up for first few days and then repeat that plan!
