# Supplementary material for: Development and Feasibility of an eHealth Diabetes Prevention Program Adapted for Older Adults—Results from a Randomized Control Pilot Study
Source: Nutrients. 2024 Mar 23;16(7):930. doi: 10.3390/nu16070930 (PMC11154527; doi:10.3390/nu16070930)
Supplement: Supplementary file 1 [file nutrients-16-00930-s001.zip › Week10.pptx]

## Slide 1
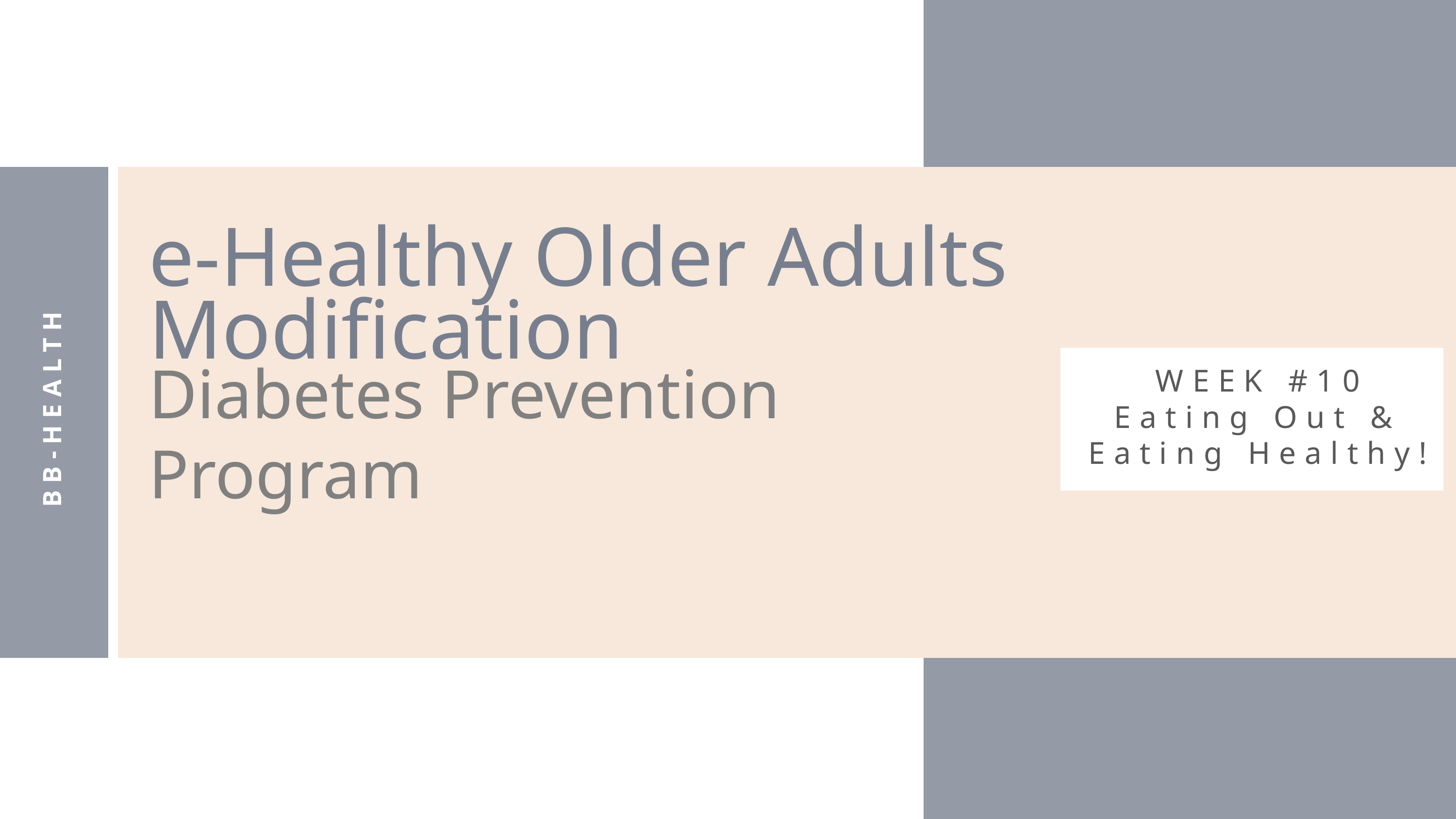

OPEN REPORTS
e-Healthy Older Adults Modification
WEEK #10
Eating Out & Eating Healthy!
Diabetes Prevention Program
BB-HEALTH

## Slide 2
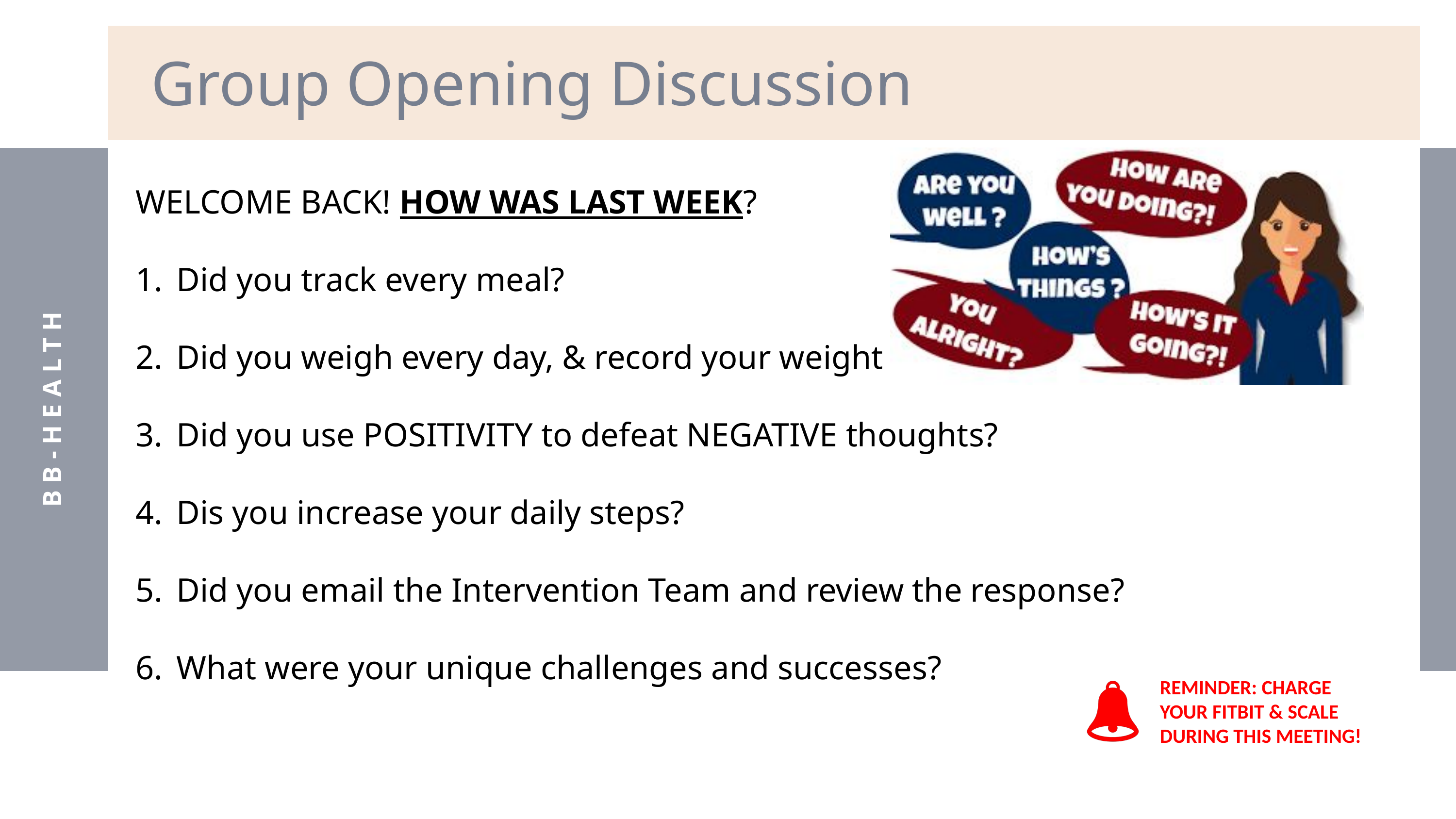

Group Opening Discussion
WELCOME BACK! HOW WAS LAST WEEK?
Did you track every meal?
Did you weigh every day, & record your weight today?
Did you use POSITIVITY to defeat NEGATIVE thoughts?
Dis you increase your daily steps?
Did you email the Intervention Team and review the response?
What were your unique challenges and successes?
BB-HEALTH
REMINDER: CHARGE YOUR FITBIT & SCALE DURING THIS MEETING!

## Slide 3
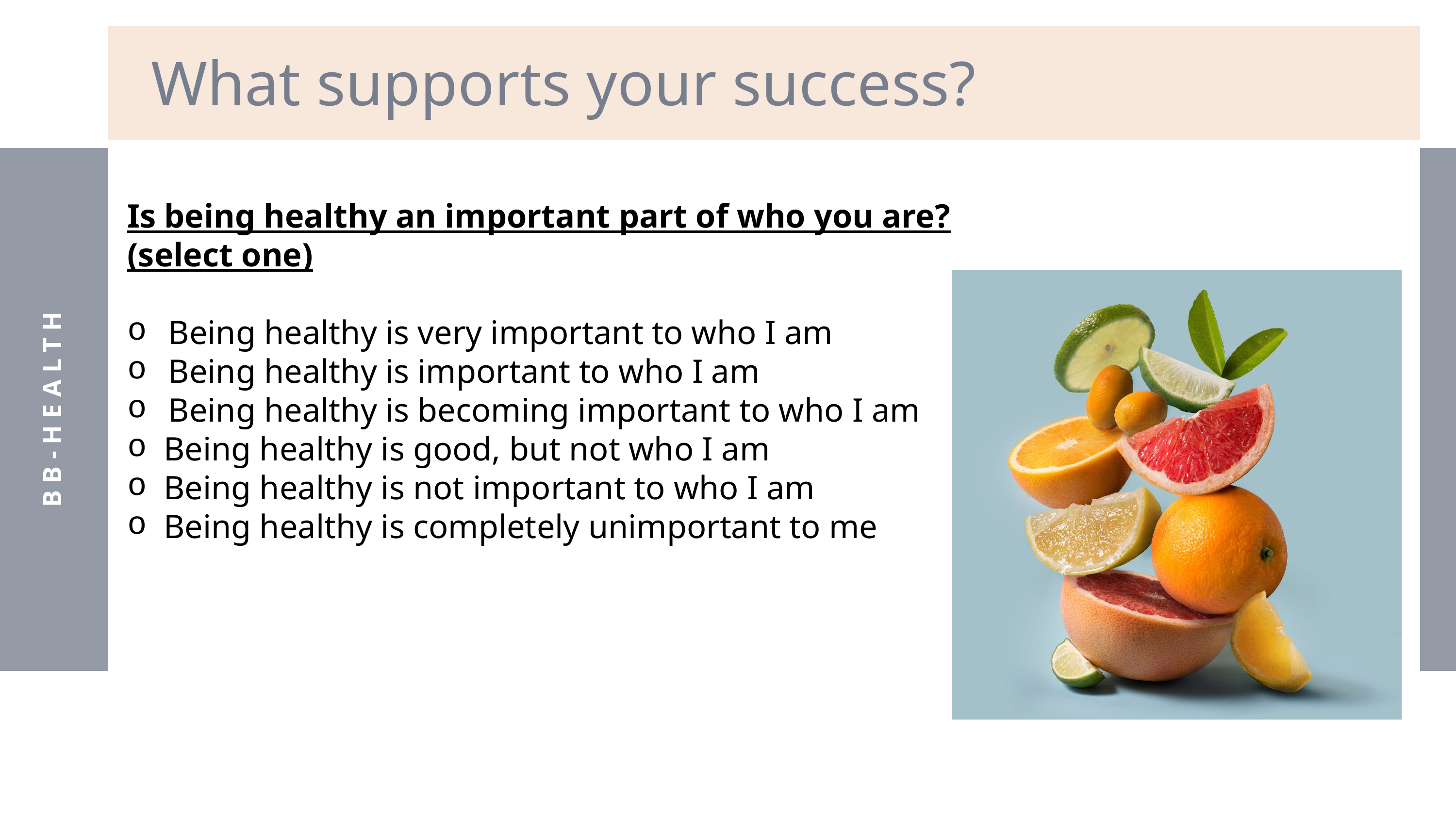

What supports your success?
Is being healthy an important part of who you are? (select one)
Being healthy is very important to who I am
Being healthy is important to who I am
Being healthy is becoming important to who I am
Being healthy is good, but not who I am
Being healthy is not important to who I am
Being healthy is completely unimportant to me
BB-HEALTH

## Slide 4
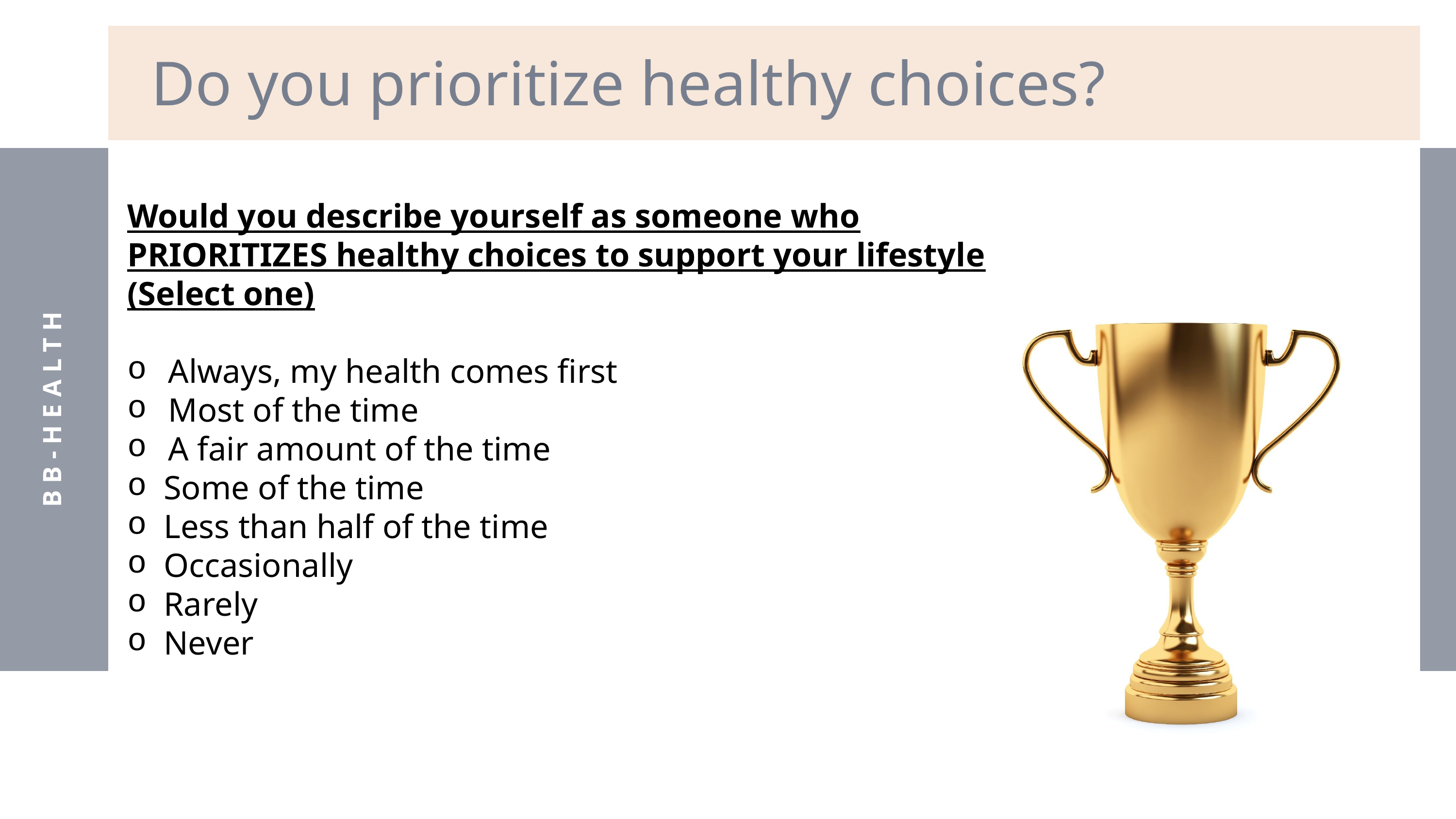

Do you prioritize healthy choices?
Would you describe yourself as someone who PRIORITIZES healthy choices to support your lifestyle (Select one)
Always, my health comes first
Most of the time
A fair amount of the time
Some of the time
Less than half of the time
Occasionally
Rarely
Never
BB-HEALTH

## Slide 5
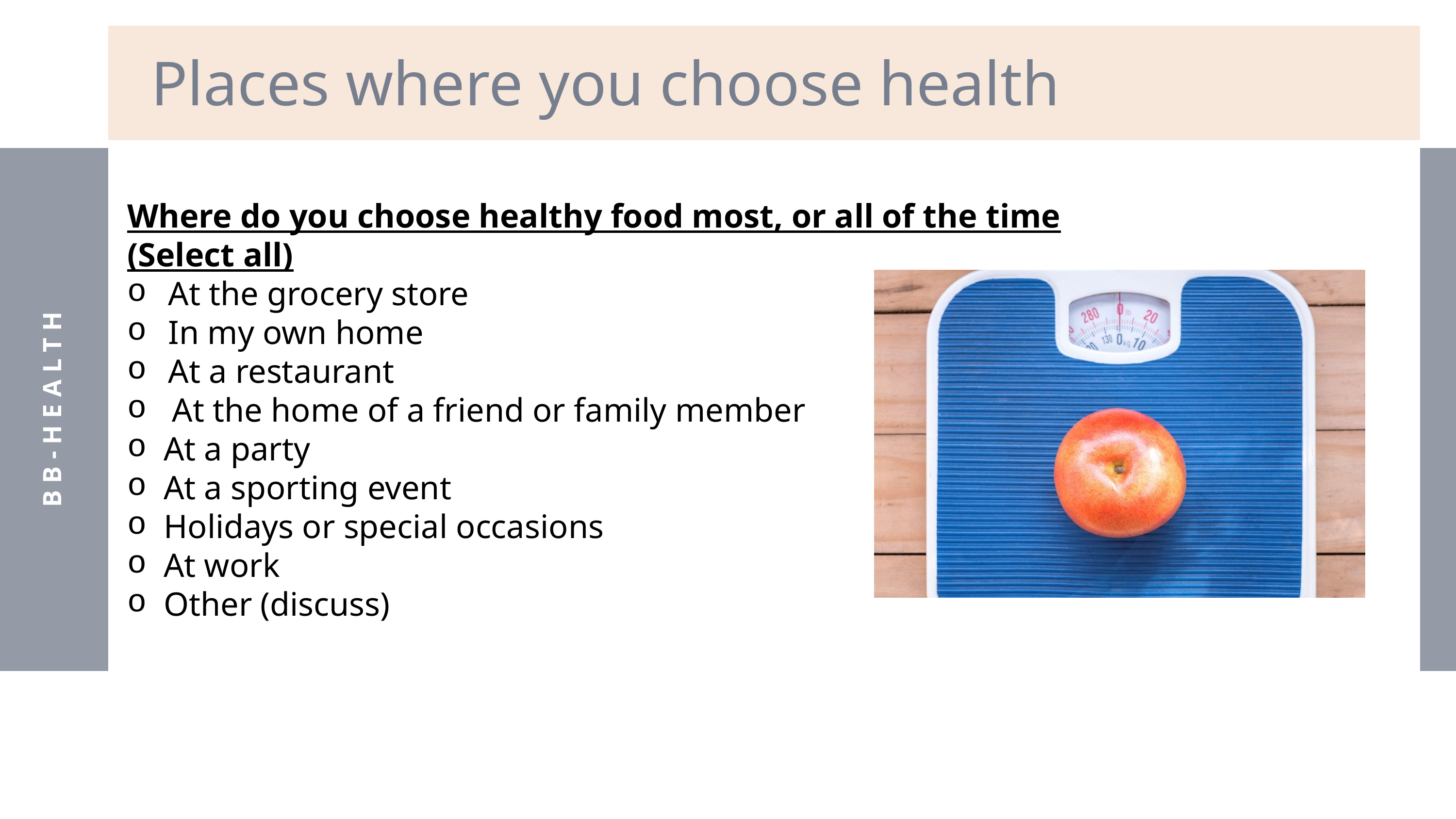

Places where you choose health
Where do you choose healthy food most, or all of the time (Select all)
At the grocery store
In my own home
At a restaurant
 At the home of a friend or family member
At a party
At a sporting event
Holidays or special occasions
At work
Other (discuss)
BB-HEALTH

## Slide 6
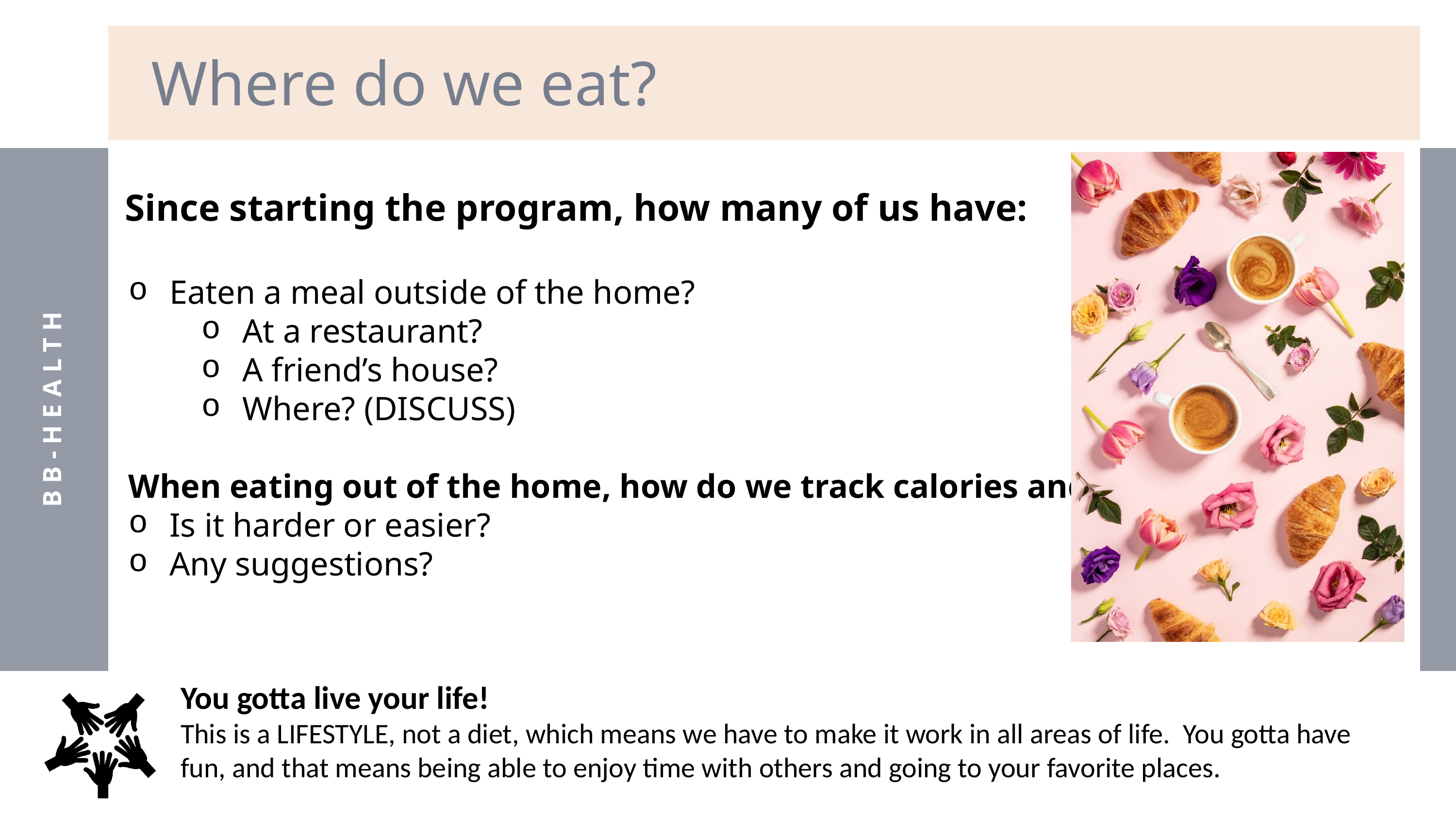

Where do we eat?
Since starting the program, how many of us have:
Eaten a meal outside of the home?
At a restaurant?
A friend’s house?
Where? (DISCUSS)
When eating out of the home, how do we track calories and fat grams?
Is it harder or easier?
Any suggestions?
BB-HEALTH
You gotta live your life!
This is a LIFESTYLE, not a diet, which means we have to make it work in all areas of life. You gotta have fun, and that means being able to enjoy time with others and going to your favorite places.

## Slide 7
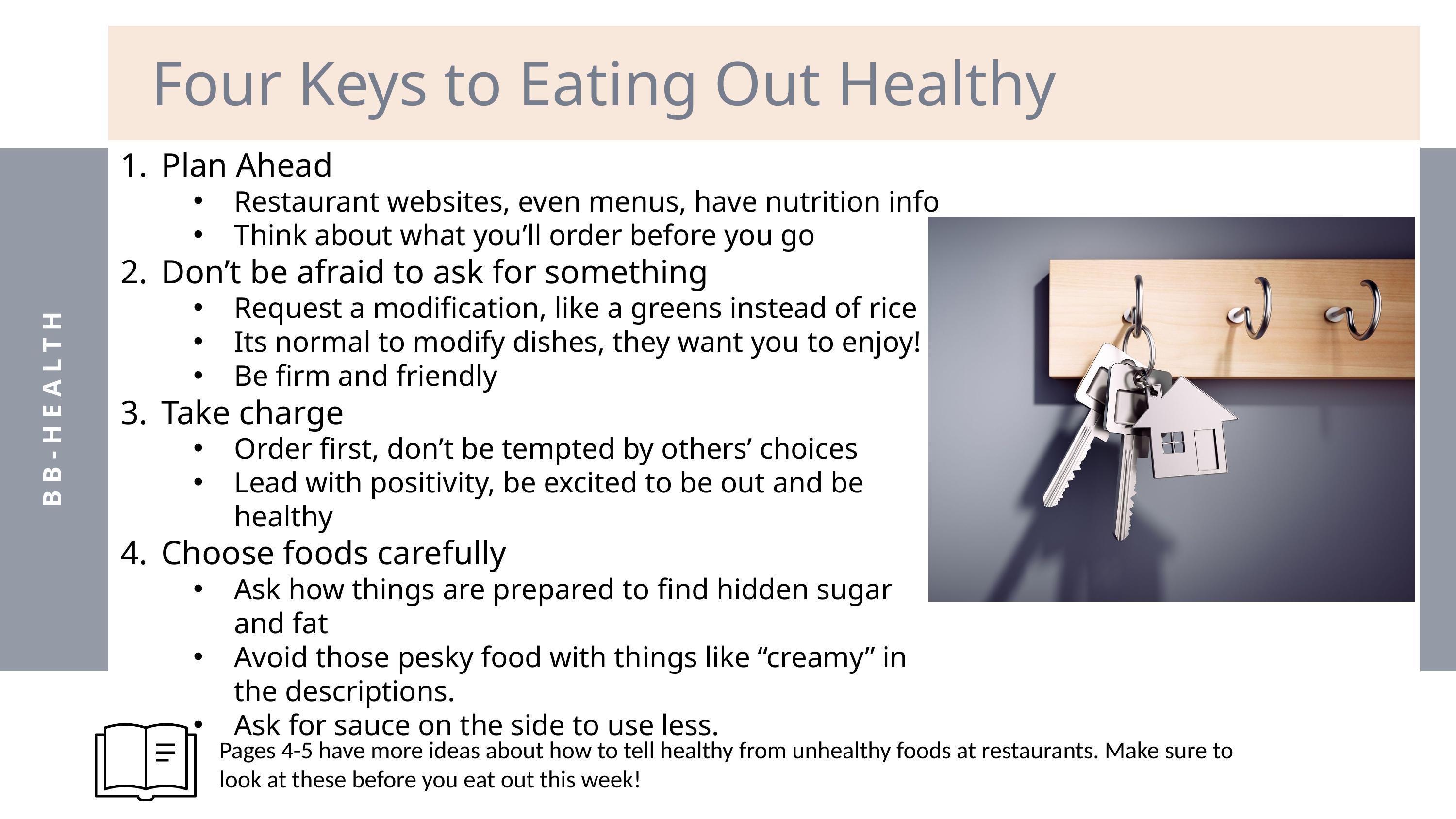

Four Keys to Eating Out Healthy
Plan Ahead
Restaurant websites, even menus, have nutrition info
Think about what you’ll order before you go
Don’t be afraid to ask for something
Request a modification, like a greens instead of rice
Its normal to modify dishes, they want you to enjoy!
Be firm and friendly
Take charge
Order first, don’t be tempted by others’ choices
Lead with positivity, be excited to be out and be healthy
Choose foods carefully
Ask how things are prepared to find hidden sugar and fat
Avoid those pesky food with things like “creamy” in the descriptions.
Ask for sauce on the side to use less.
BB-HEALTH
Pages 4-5 have more ideas about how to tell healthy from unhealthy foods at restaurants. Make sure to look at these before you eat out this week!

## Slide 8
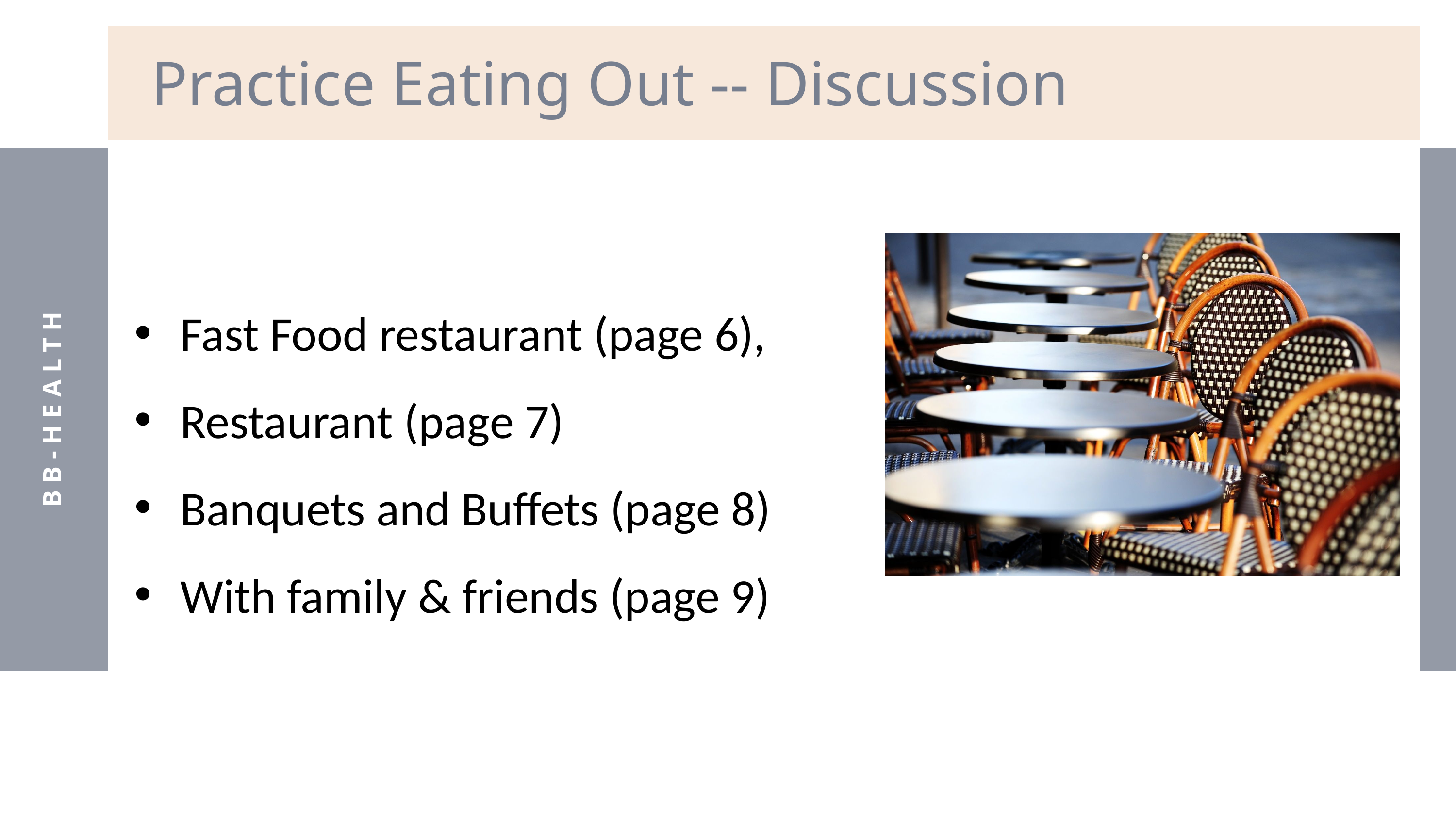

Practice Eating Out -- Discussion
Fast Food restaurant (page 6),
Restaurant (page 7)
Banquets and Buffets (page 8)
With family & friends (page 9)
BB-HEALTH

## Slide 9
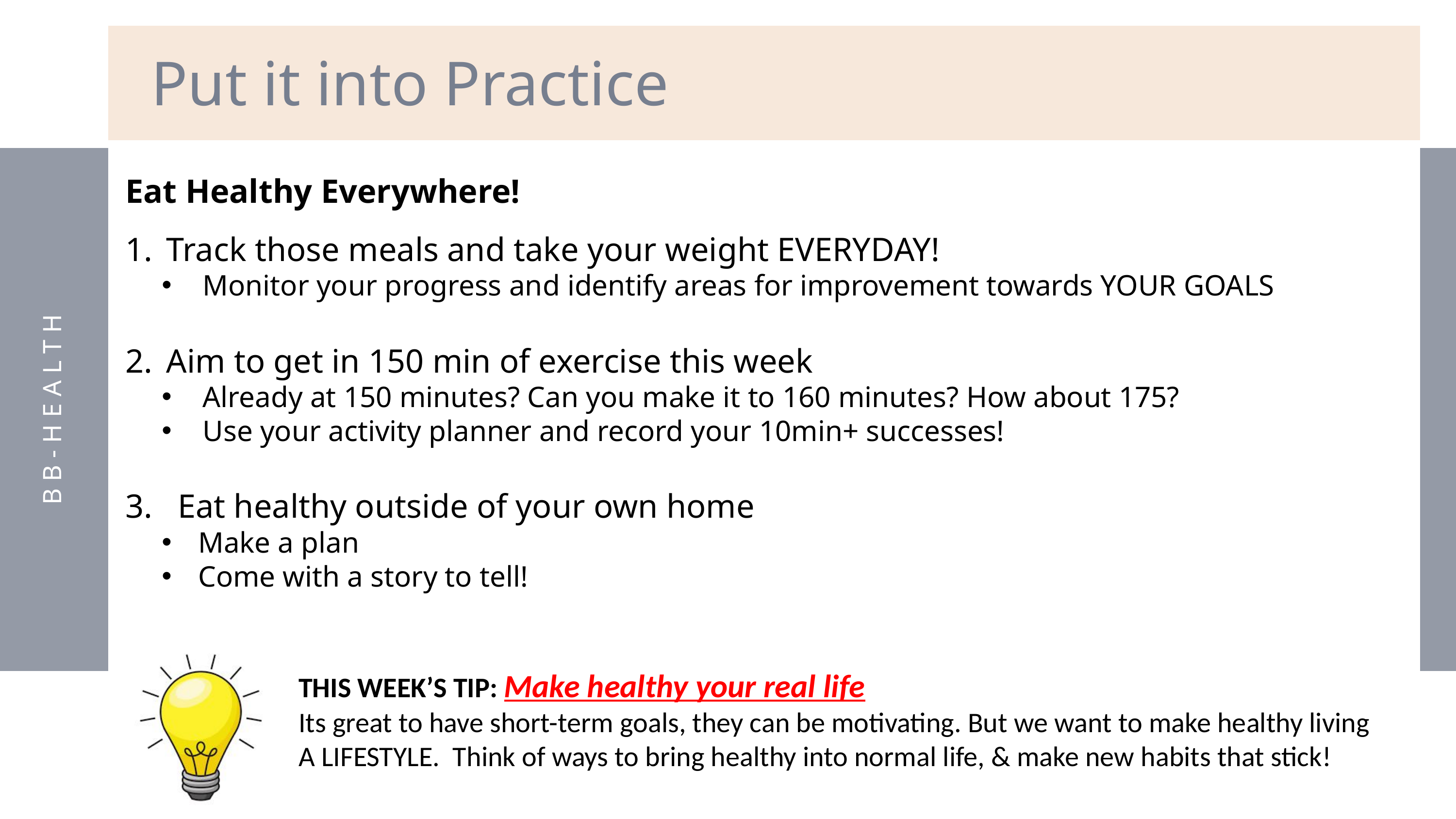

Put it into Practice
Eat Healthy Everywhere!
Track those meals and take your weight EVERYDAY!
Monitor your progress and identify areas for improvement towards YOUR GOALS
Aim to get in 150 min of exercise this week
Already at 150 minutes? Can you make it to 160 minutes? How about 175?
Use your activity planner and record your 10min+ successes!
3. Eat healthy outside of your own home
Make a plan
Come with a story to tell!
BB-HEALTH
THIS WEEK’S TIP: Make healthy your real life
Its great to have short-term goals, they can be motivating. But we want to make healthy living A LIFESTYLE. Think of ways to bring healthy into normal life, & make new habits that stick!

## Slide 10
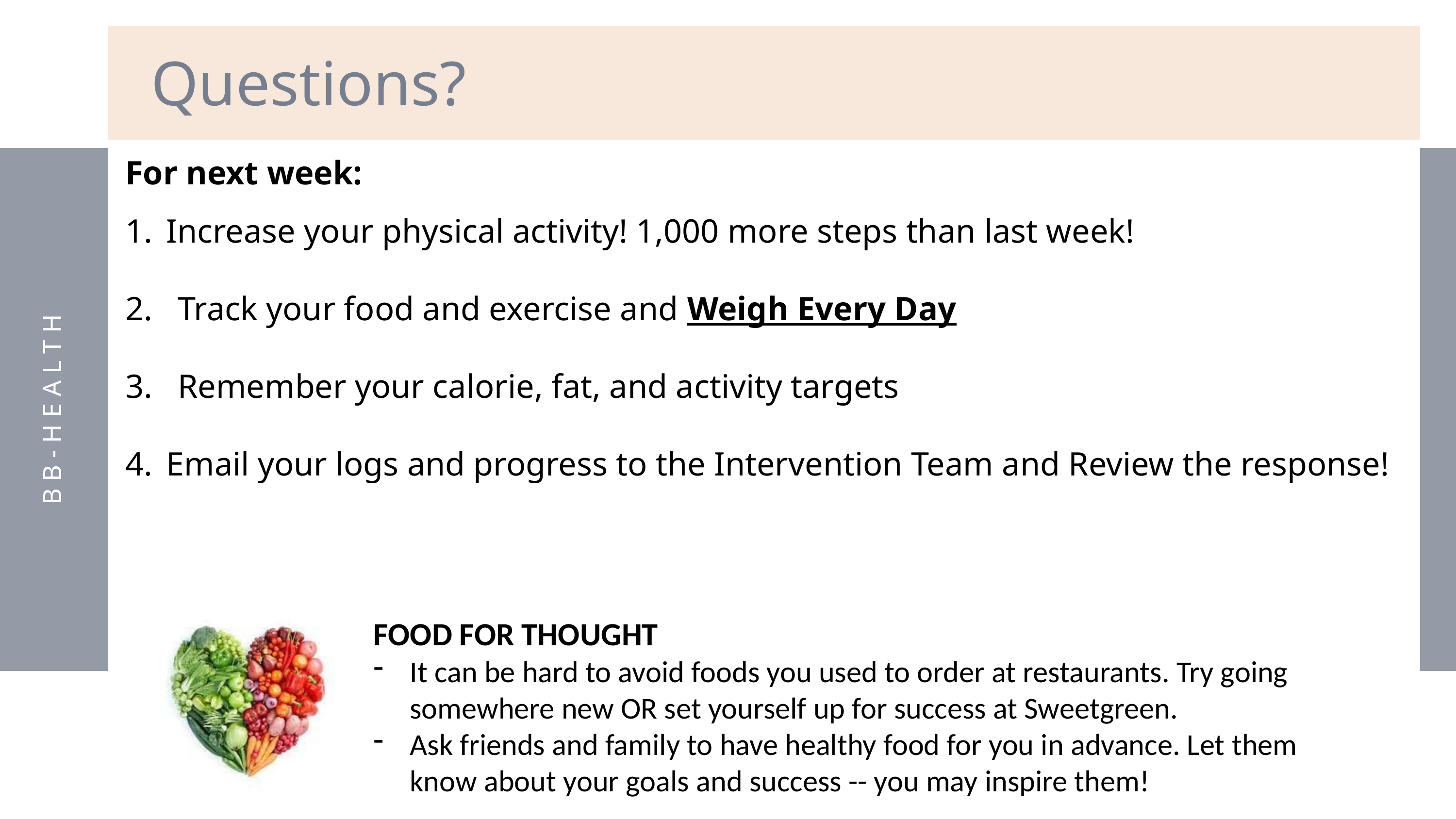

Questions?
For next week:
Increase your physical activity! 1,000 more steps than last week!
2. Track your food and exercise and Weigh Every Day
3. Remember your calorie, fat, and activity targets
Email your logs and progress to the Intervention Team and Review the response!
BB-HEALTH
FOOD FOR THOUGHT
It can be hard to avoid foods you used to order at restaurants. Try going somewhere new OR set yourself up for success at Sweetgreen.
Ask friends and family to have healthy food for you in advance. Let them know about your goals and success -- you may inspire them!
