# Supplementary material for: Development and Feasibility of an eHealth Diabetes Prevention Program Adapted for Older Adults—Results from a Randomized Control Pilot Study
Source: Nutrients. 2024 Mar 23;16(7):930. doi: 10.3390/nu16070930 (PMC11154527; doi:10.3390/nu16070930)
Supplement: Supplementary file 1 [file nutrients-16-00930-s001.zip › Week10_SUPP.pdf]

BB-HEALTH

e-Healthy Older Adults Modification

Diabetes Prevention Program

WEEK #10
Eating Out &
Eating Healthy!

Group Opening Discussion

WELCOME BACK! <u>**HOW WAS LAST WEEK**</u>?

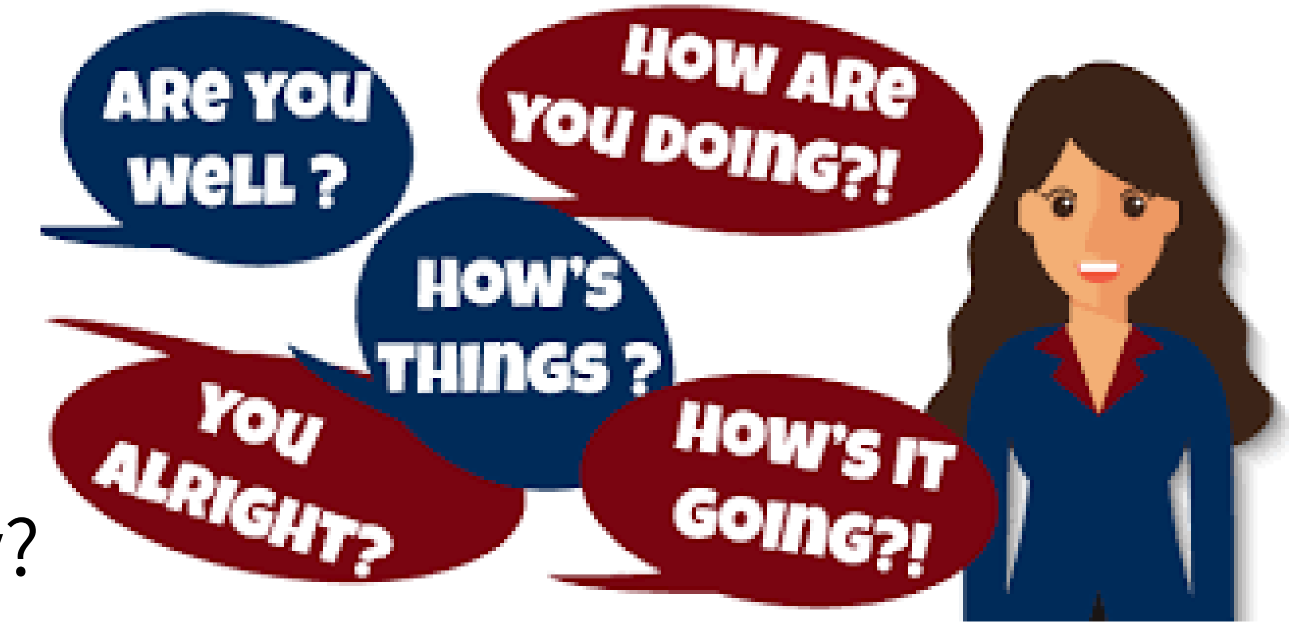

1. Did you track every meal?
2. Did you weigh every day, & record your weight today?
3. Did you use POSITIVITY to defeat NEGATIVE thoughts?
4. Dis you increase your daily steps?
5. Did you email the Intervention Team and review the response?
6. What were your unique challenges and successes?

BB-HEALTH

REMINDER: CHARGE YOUR FITBIT & SCALE DURING THIS MEETING!

What supports your success?

BB-HEALTH

<u>Is being healthy an important part of who you are? (select one)</u>

- ○ Being healthy is very important to who I am
- ○ Being healthy is important to who I am
- ○ Being healthy is becoming important to who I am
- ○ Being healthy is good, but not who I am
- ○ Being healthy is not important to who I am
- ○ Being healthy is completely unimportant to me

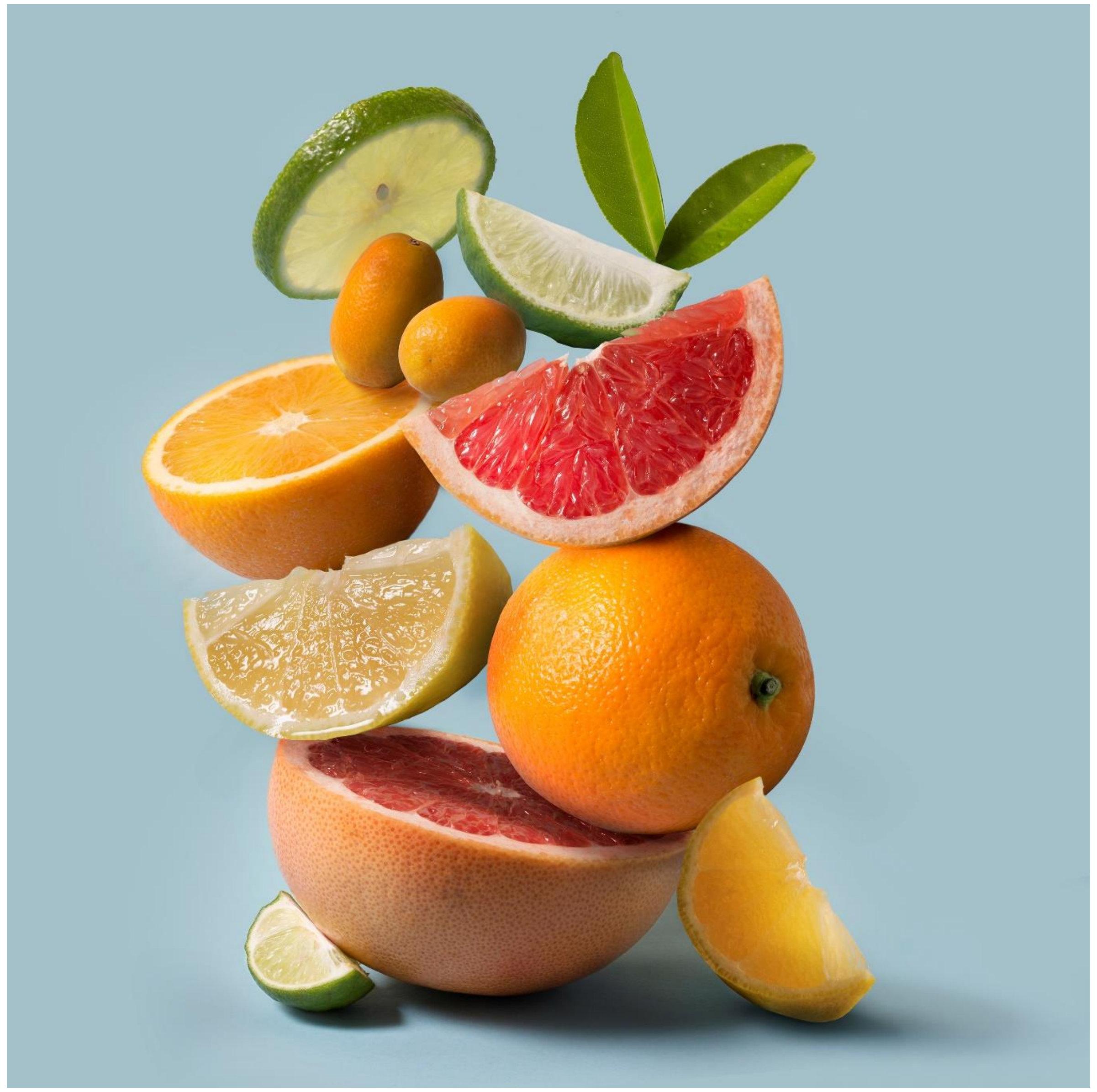

Do you prioritize healthy choices?

<u>**Would you describe yourself as someone who PRIORITIZES healthy choices to support your lifestyle (Select one)**</u>

- Always, my health comes first
- Most of the time
- A fair amount of the time
- Some of the time
- Less than half of the time
- Occasionally
- Rarely
- Never

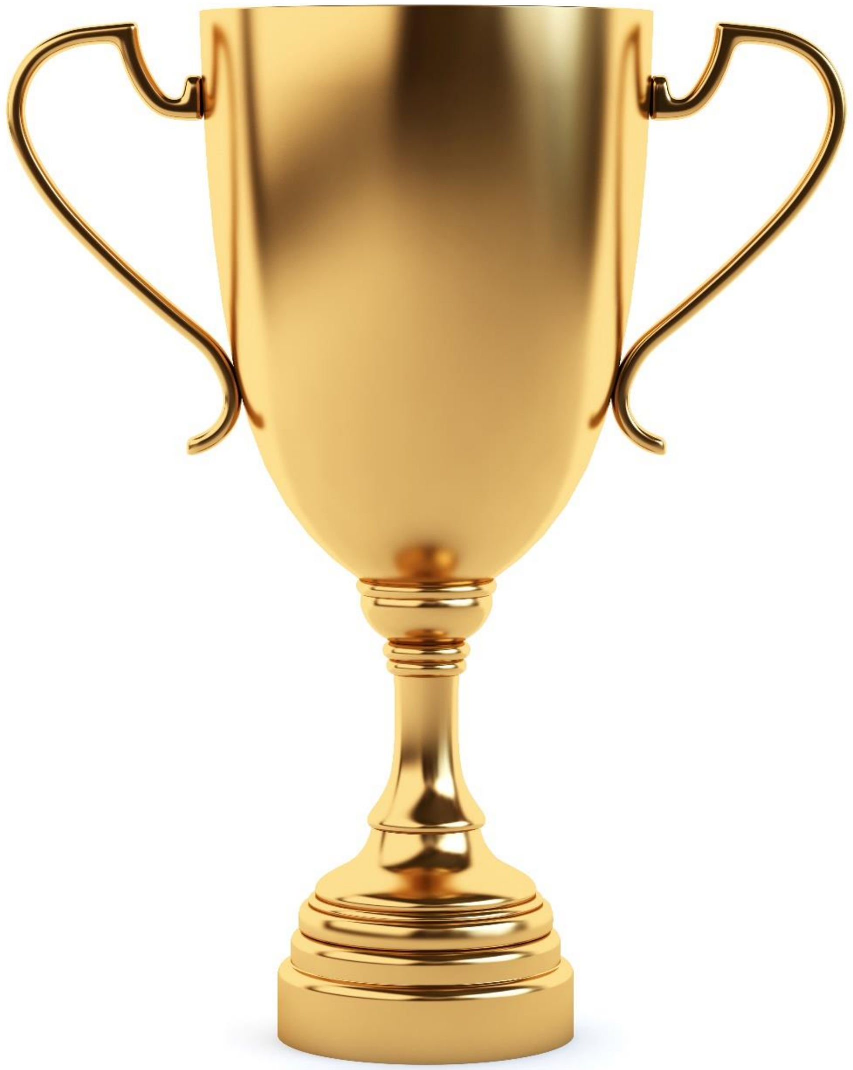

BB-HEALTH

Places where you choose health

<u>**Where do you choose healthy food most, or all of the time (Select all)**</u>

- At the grocery store
- In my own home
- At a restaurant
- At the home of a friend or family member
- At a party
- At a sporting event
- Holidays or special occasions
- At work
- Other (discuss)

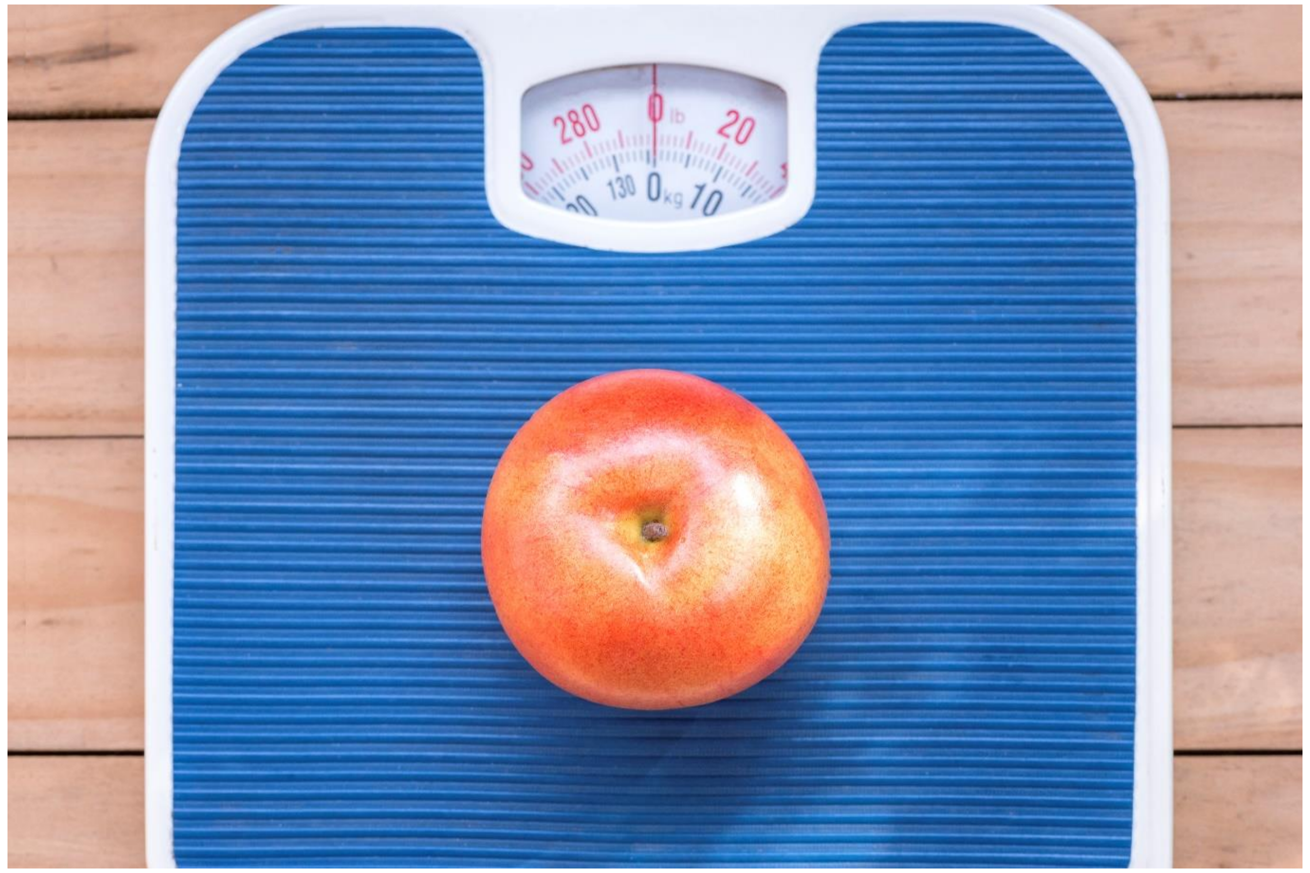

Where do we eat?

BB-HEALTH

Since starting the program, how many of us have:

- Eaten a meal outside of the home?
  - At a restaurant?
  - A friend's house?
  - Where? (DISCUSS)

When eating out of the home, how do we track calories and fat grams?

- Is it harder or easier?
- Any suggestions?

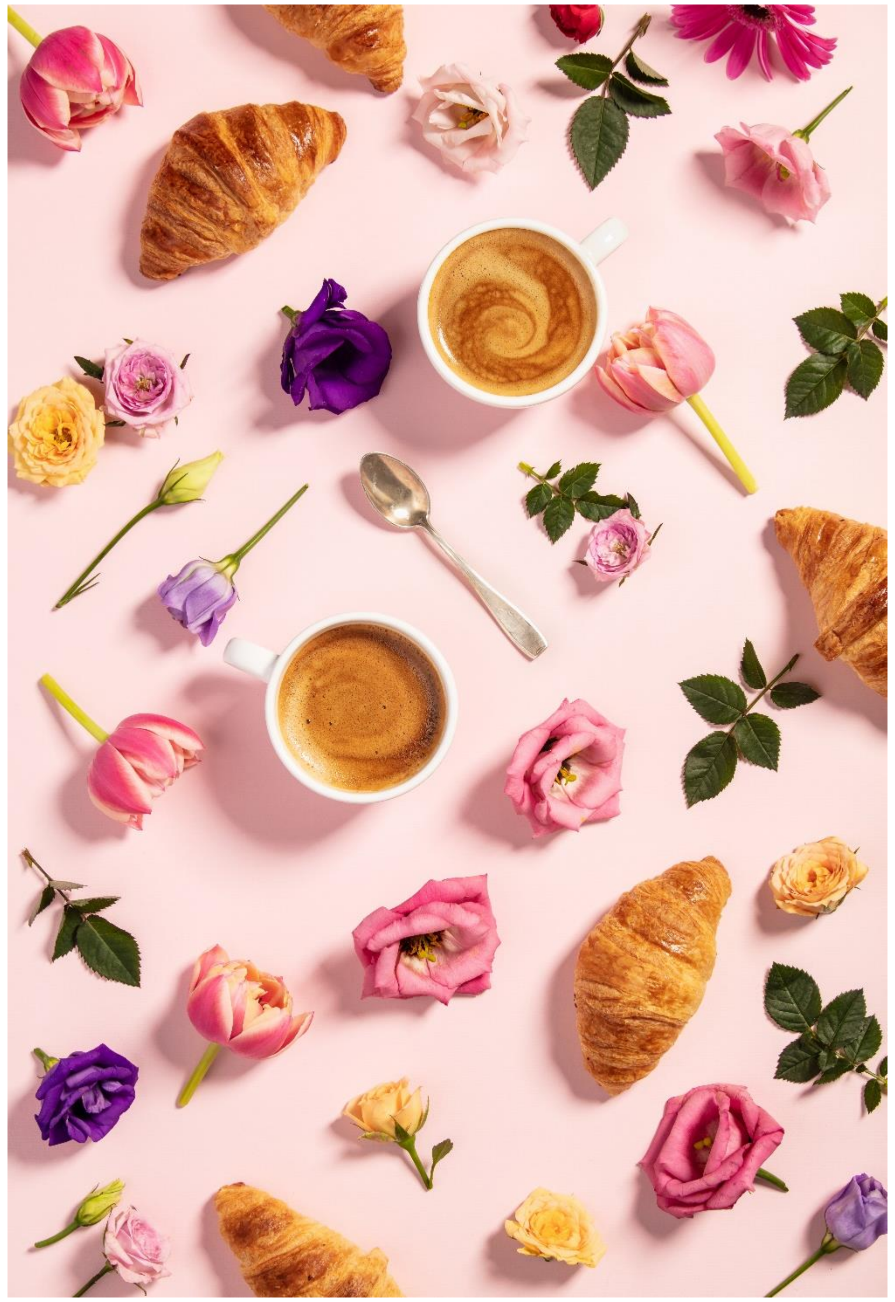

You gotta live your life!

This is a LIFESTYLE, not a diet, which means we have to make it work in all areas of life. You gotta have fun, and that means being able to enjoy time with others and going to your favorite places.

Four Keys to Eating Out Healthy

B B - H E A L T H

1. Plan Ahead
    - Restaurant websites, even menus, have nutrition info
    - Think about what you'll order before you go
2. Don't be afraid to ask for something
    - Request a modification, like a greens instead of rice
    - Its normal to modify dishes, they want you to enjoy!
    - Be firm and friendly
3. Take charge
    - Order first, don't be tempted by others' choices
    - Lead with positivity, be excited to be out and be healthy
4. Choose foods carefully
    - Ask how things are prepared to find hidden sugar and fat
    - Avoid those pesky food with things like "creamy" in the descriptions.
    - Ask for sauce on the side to use less.

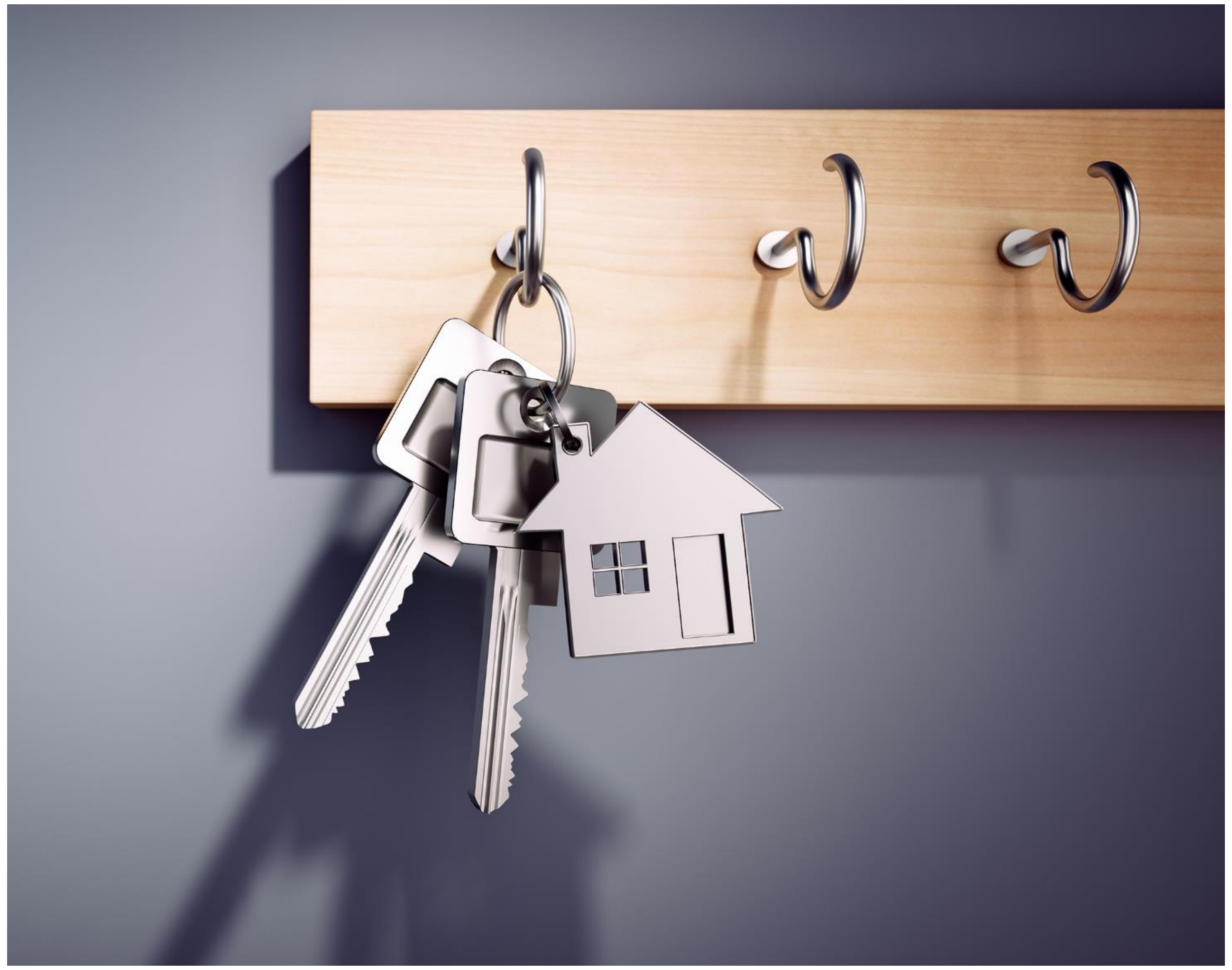

Pages 4-5 have more ideas about how to tell healthy from unhealthy foods at restaurants. Make sure to look at these before you eat out this week!

Practice Eating Out -- Discussion

BB-HEALTH

- Fast Food restaurant (page 6),
- Restaurant (page 7)
- Banquets and Buffets (page 8)
- With family & friends (page 9)

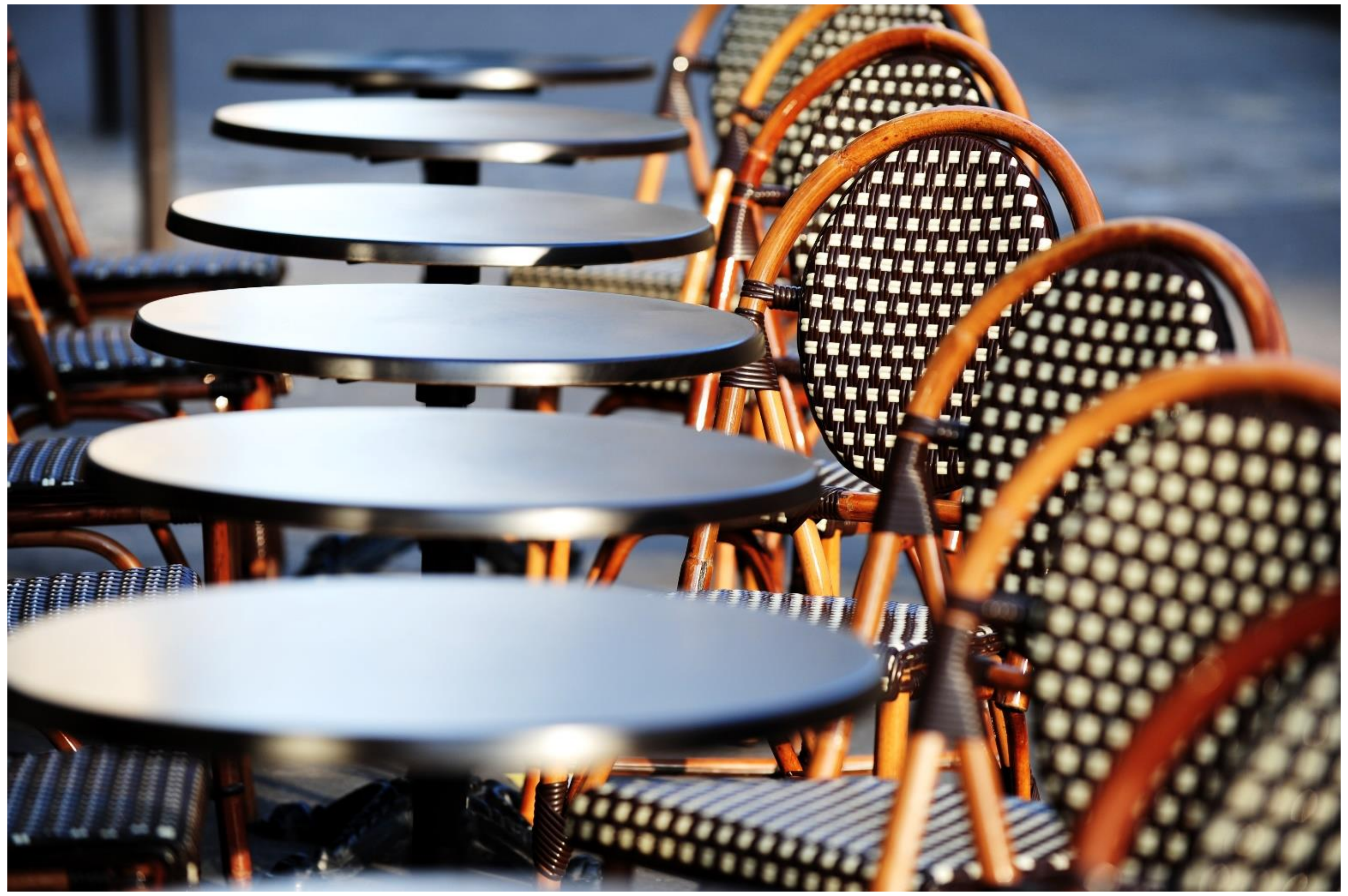

Put it into Practice

Eat Healthy Everywhere!

1. Track those meals and take your weight EVERYDAY!
   - Monitor your progress and identify areas for improvement towards YOUR GOALS

2. Aim to get in 150 min of exercise this week
   - Already at 150 minutes? Can you make it to 160 minutes? How about 175?
   - Use your activity planner and record your 10min+ successes!

3. Eat healthy outside of your own home
   - Make a plan
   - Come with a story to tell!

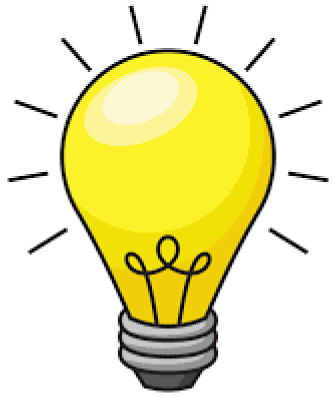

THIS WEEK'S TIP: ***Make healthy your real life***
Its great to have short-term goals, they can be motivating. But we want to make healthy living A LIFESTYLE. Think of ways to bring healthy into normal life, & make new habits that stick!

Questions?

B B - H E A L T H

For next week:

1. Increase your physical activity! 1,000 more steps than last week!

2. Track your food and exercise and **<u>Weigh Every Day</u>**

3. Remember your calorie, fat, and activity targets

4. Email your logs and progress to the Intervention Team and Review the response!

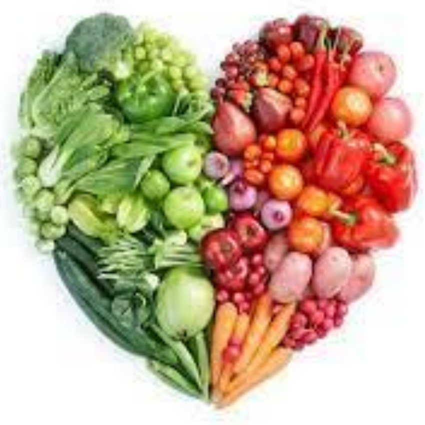

FOOD FOR THOUGHT

- It can be hard to avoid foods you used to order at restaurants. Try going somewhere new OR set yourself up for success at Sweetgreen.
- Ask friends and family to have healthy food for you in advance. Let them know about your goals and success -- you may inspire them!
