# Supplementary material for: Development and Feasibility of an eHealth Diabetes Prevention Program Adapted for Older Adults—Results from a Randomized Control Pilot Study
Source: Nutrients. 2024 Mar 23;16(7):930. doi: 10.3390/nu16070930 (PMC11154527; doi:10.3390/nu16070930)
Supplement: Supplementary file 1 [file nutrients-16-00930-s001.zip › Week11.pptx]

## Slide 1
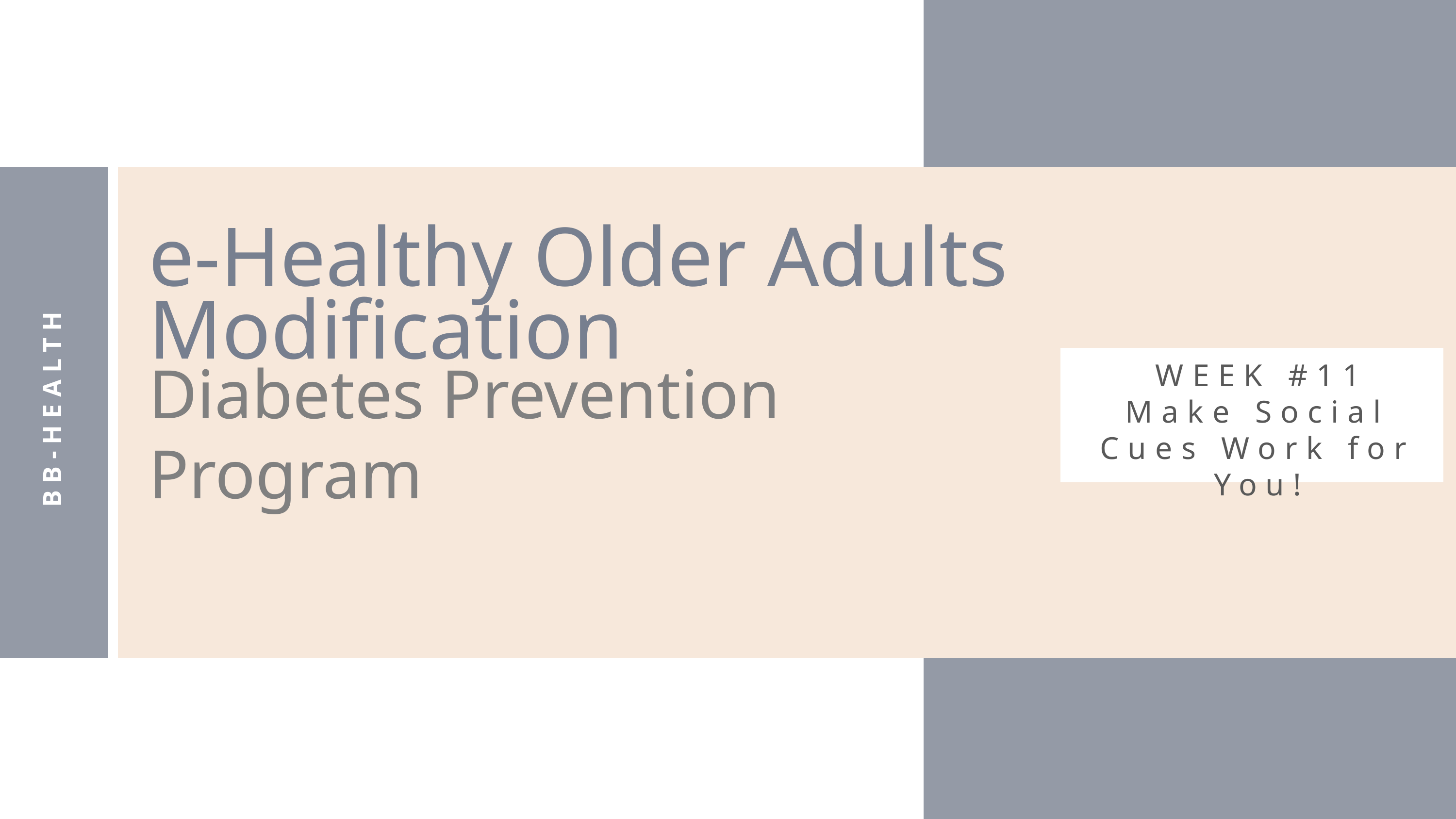

OPEN REPORTS
e-Healthy Older Adults Modification
WEEK #11
Make Social Cues Work for You!
Diabetes Prevention Program
BB-HEALTH

## Slide 2
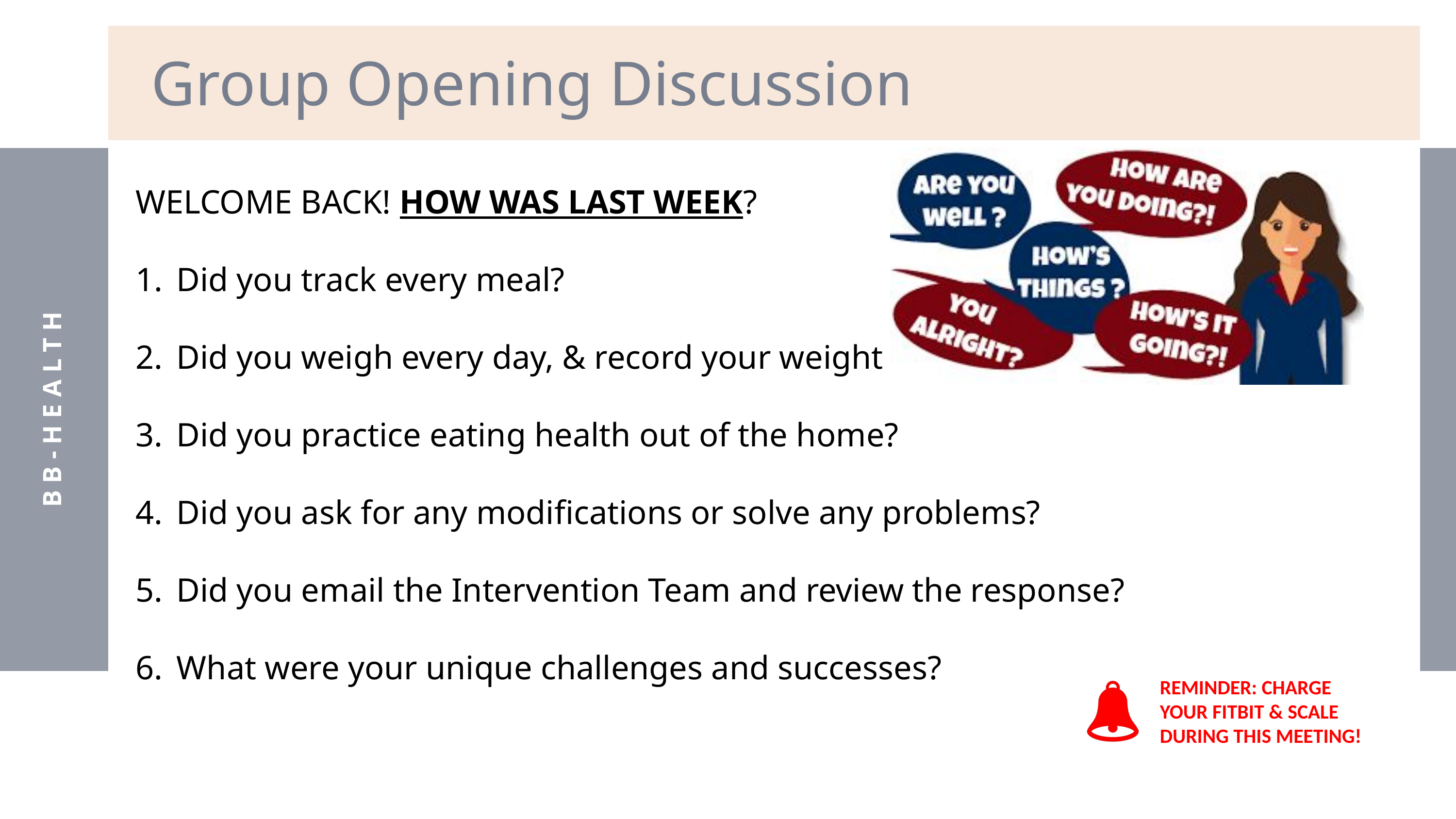

Group Opening Discussion
WELCOME BACK! HOW WAS LAST WEEK?
Did you track every meal?
Did you weigh every day, & record your weight today?
Did you practice eating health out of the home?
Did you ask for any modifications or solve any problems?
Did you email the Intervention Team and review the response?
What were your unique challenges and successes?
BB-HEALTH
REMINDER: CHARGE YOUR FITBIT & SCALE DURING THIS MEETING!

## Slide 3
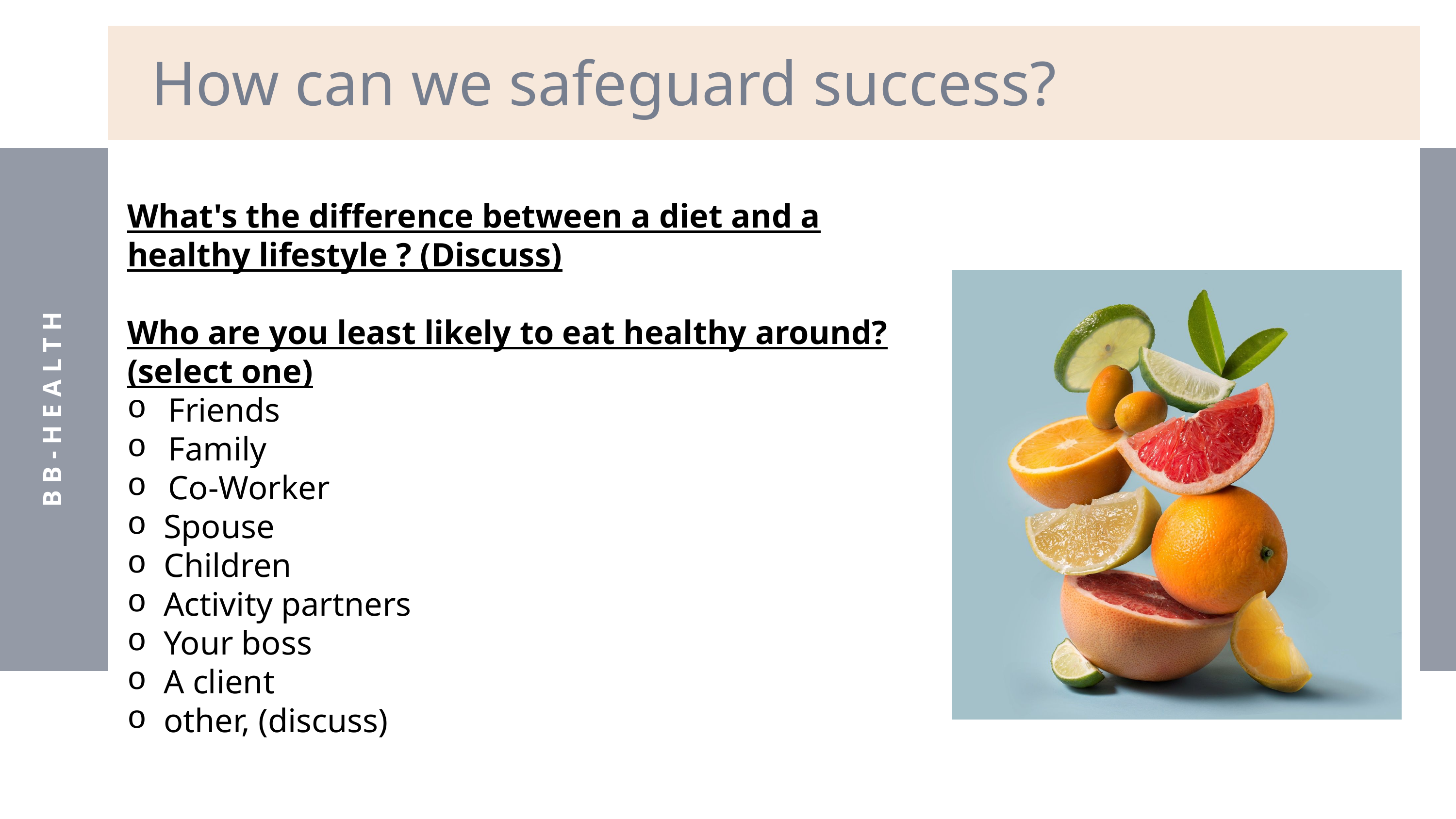

How can we safeguard success?
What's the difference between a diet and a healthy lifestyle ? (Discuss)
Who are you least likely to eat healthy around? (select one)
Friends
Family
Co-Worker
Spouse
Children
Activity partners
Your boss
A client
other, (discuss)
BB-HEALTH

## Slide 4
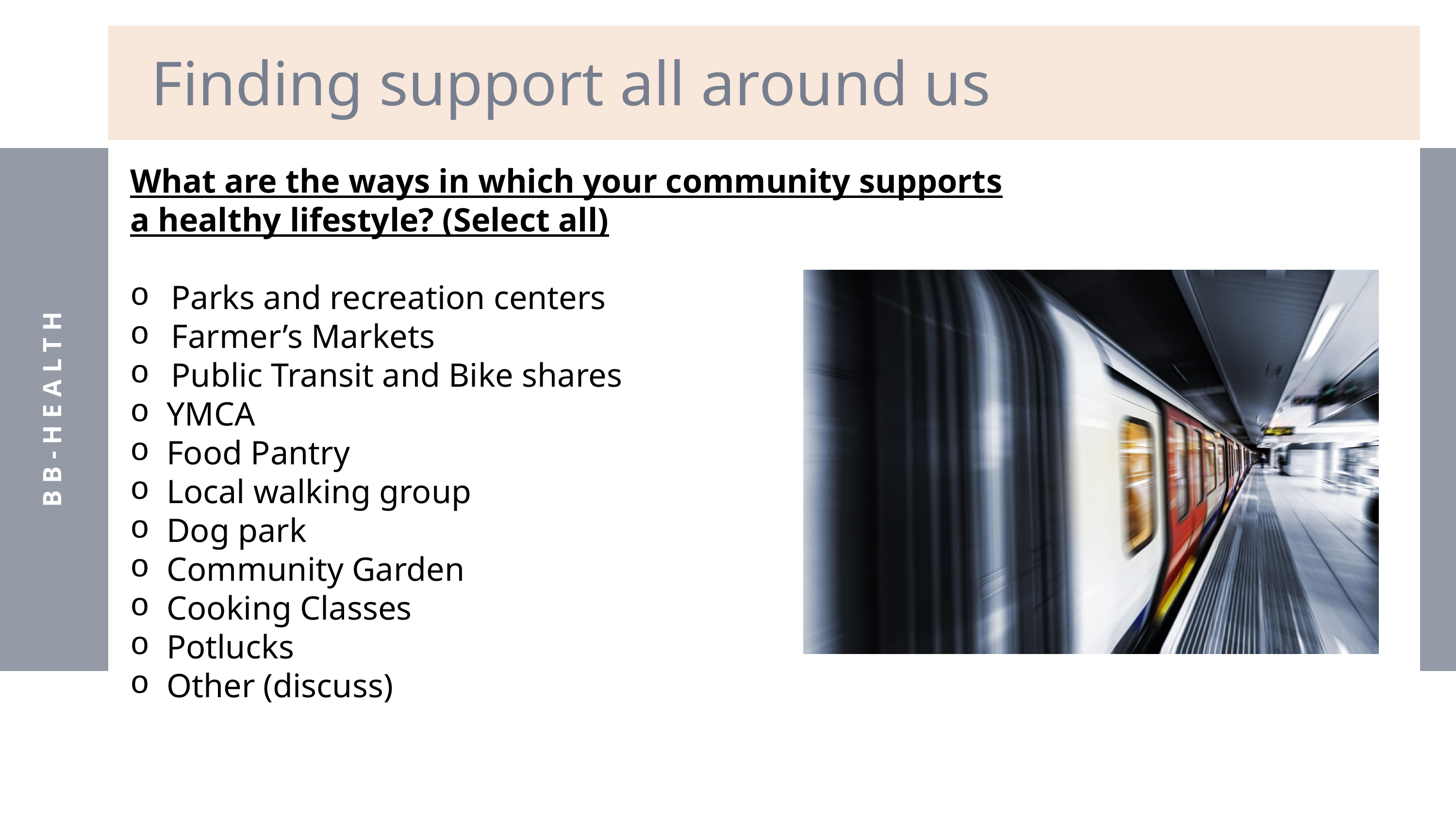

Finding support all around us
What are the ways in which your community supports a healthy lifestyle? (Select all)
Parks and recreation centers
Farmer’s Markets
Public Transit and Bike shares
YMCA
Food Pantry
Local walking group
Dog park
Community Garden
Cooking Classes
Potlucks
Other (discuss)
BB-HEALTH

## Slide 5
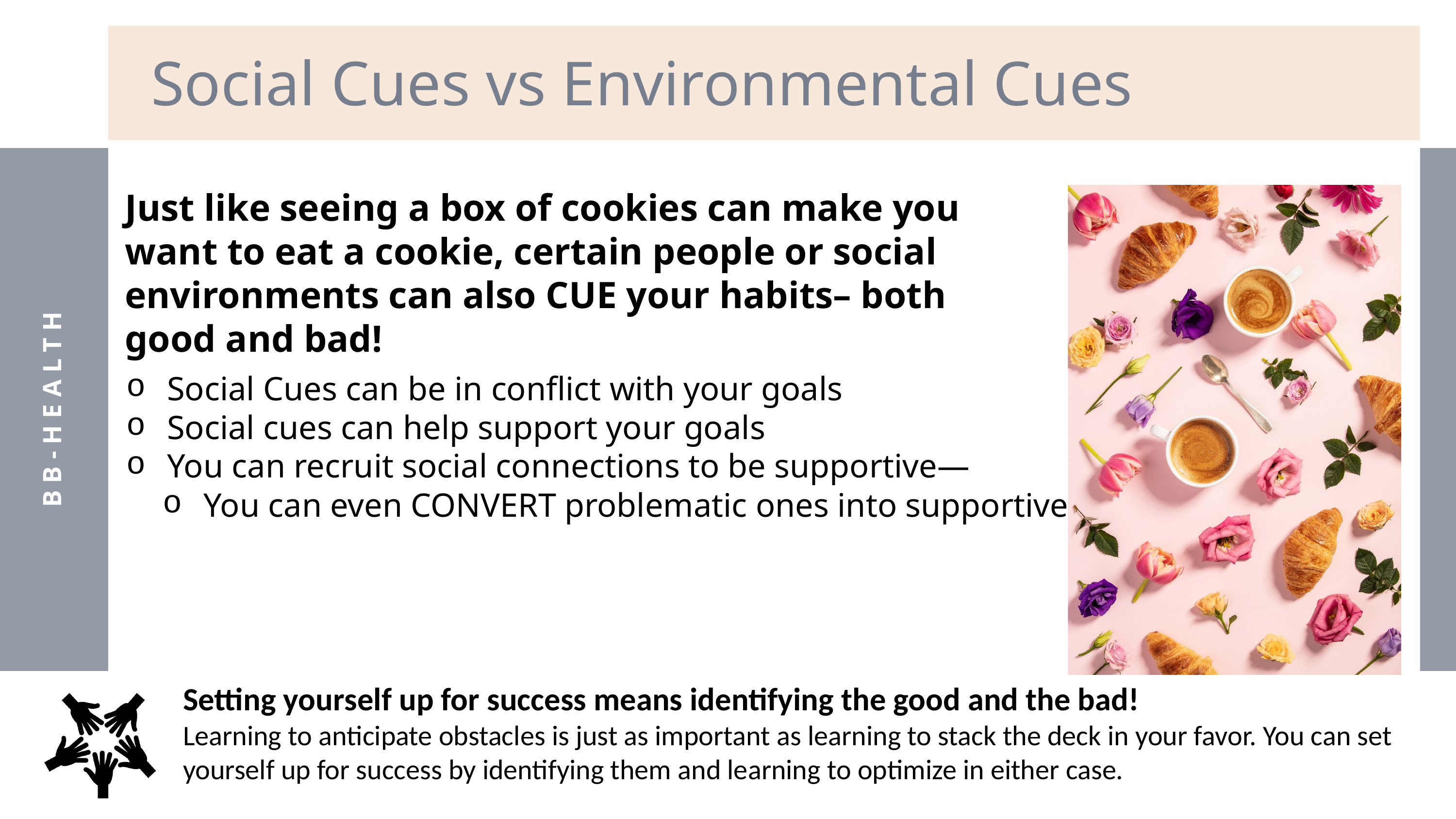

Social Cues vs Environmental Cues
Just like seeing a box of cookies can make you want to eat a cookie, certain people or social environments can also CUE your habits– both good and bad!
Social Cues can be in conflict with your goals
Social cues can help support your goals
You can recruit social connections to be supportive—
You can even CONVERT problematic ones into supportive ones!
BB-HEALTH
Setting yourself up for success means identifying the good and the bad!
Learning to anticipate obstacles is just as important as learning to stack the deck in your favor. You can set yourself up for success by identifying them and learning to optimize in either case.

## Slide 6
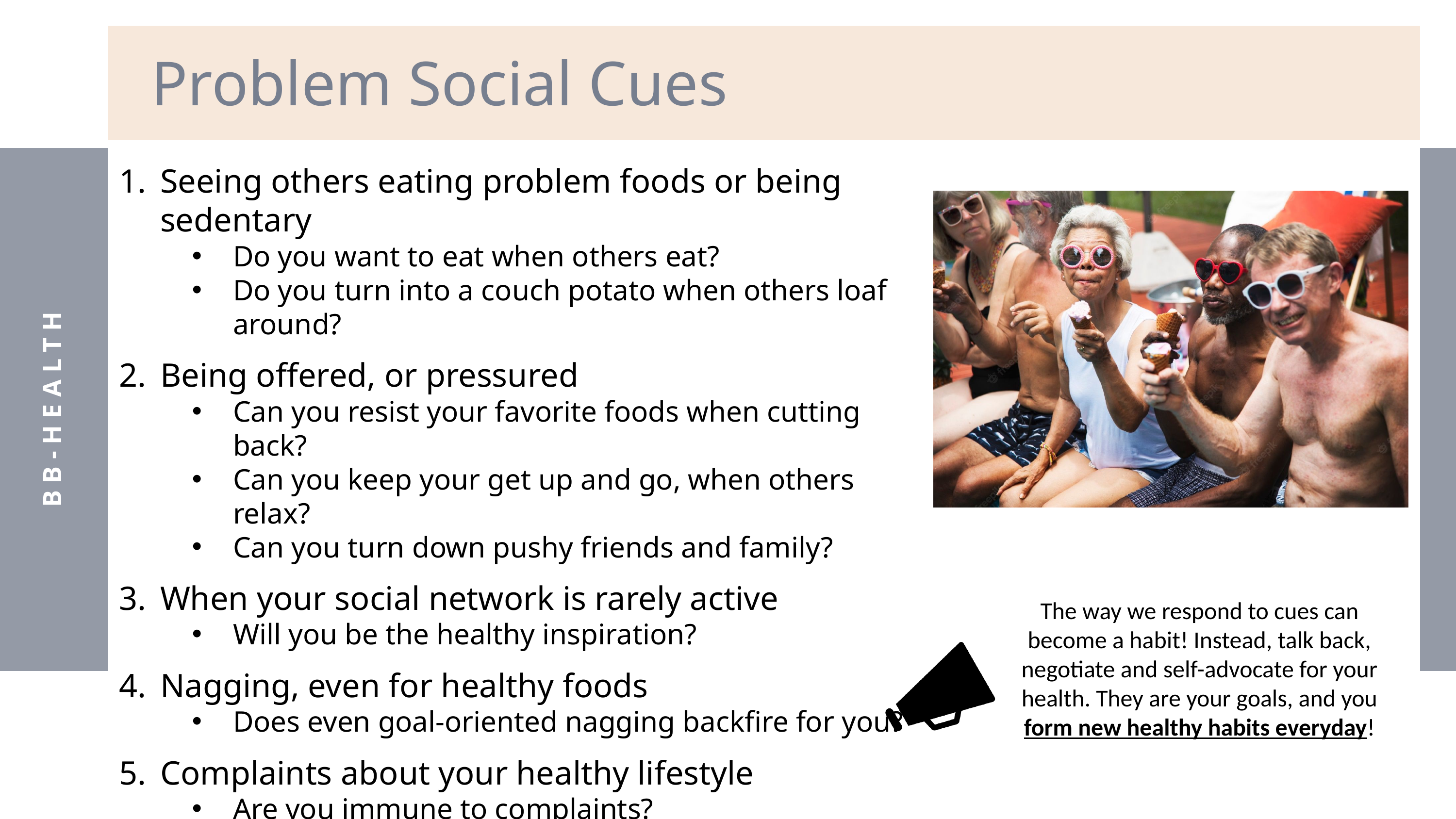

Problem Social Cues
Seeing others eating problem foods or being sedentary
Do you want to eat when others eat?
Do you turn into a couch potato when others loaf around?
Being offered, or pressured
Can you resist your favorite foods when cutting back?
Can you keep your get up and go, when others relax?
Can you turn down pushy friends and family?
When your social network is rarely active
Will you be the healthy inspiration?
Nagging, even for healthy foods
Does even goal-oriented nagging backfire for you?
Complaints about your healthy lifestyle
Are you immune to complaints?
BB-HEALTH
The way we respond to cues can become a habit! Instead, talk back, negotiate and self-advocate for your health. They are your goals, and you form new healthy habits everyday!

## Slide 7
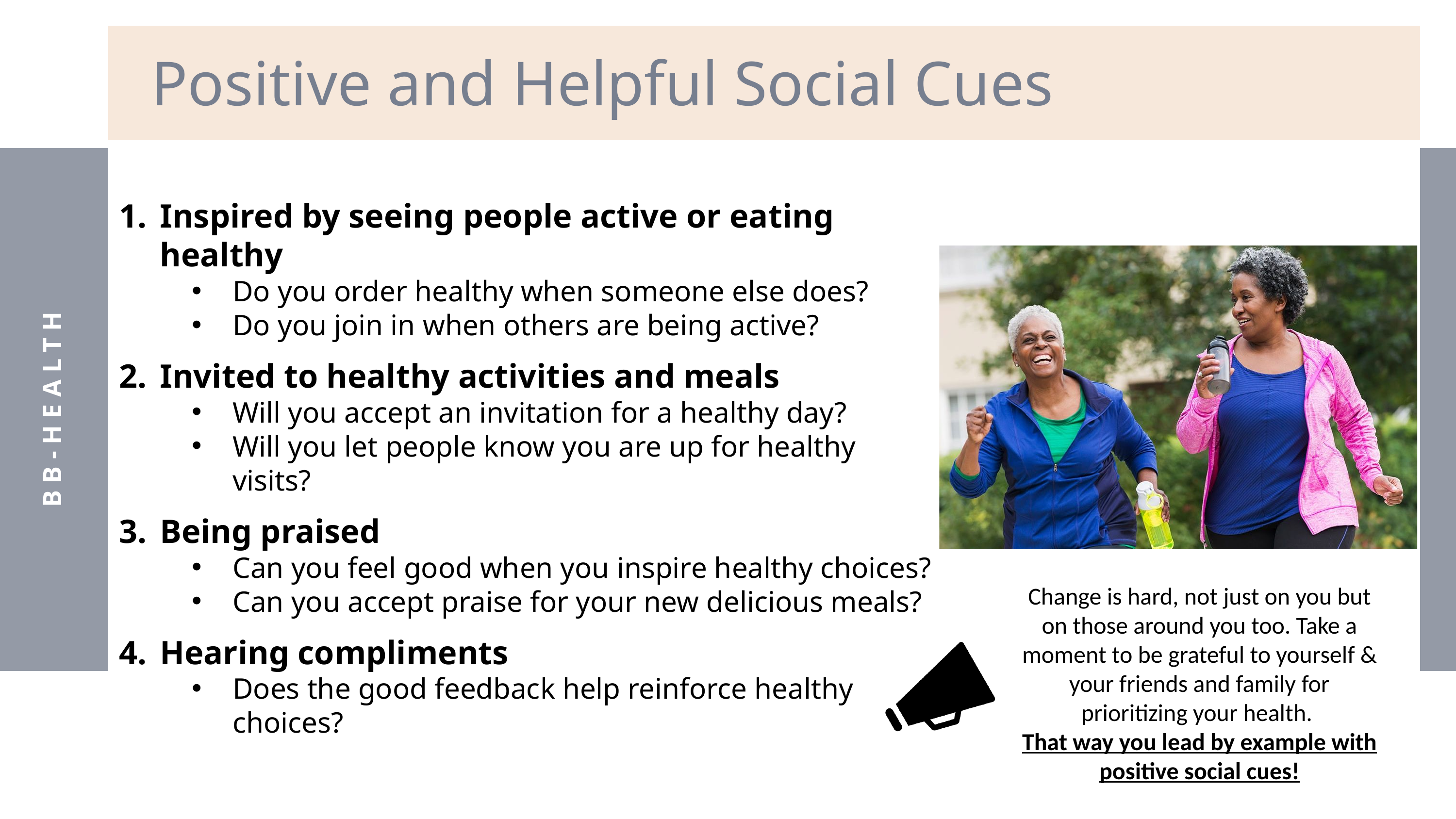

Positive and Helpful Social Cues
Inspired by seeing people active or eating healthy
Do you order healthy when someone else does?
Do you join in when others are being active?
Invited to healthy activities and meals
Will you accept an invitation for a healthy day?
Will you let people know you are up for healthy visits?
Being praised
Can you feel good when you inspire healthy choices?
Can you accept praise for your new delicious meals?
Hearing compliments
Does the good feedback help reinforce healthy choices?
BB-HEALTH
Change is hard, not just on you but on those around you too. Take a moment to be grateful to yourself & your friends and family for prioritizing your health.
That way you lead by example with positive social cues!

## Slide 8
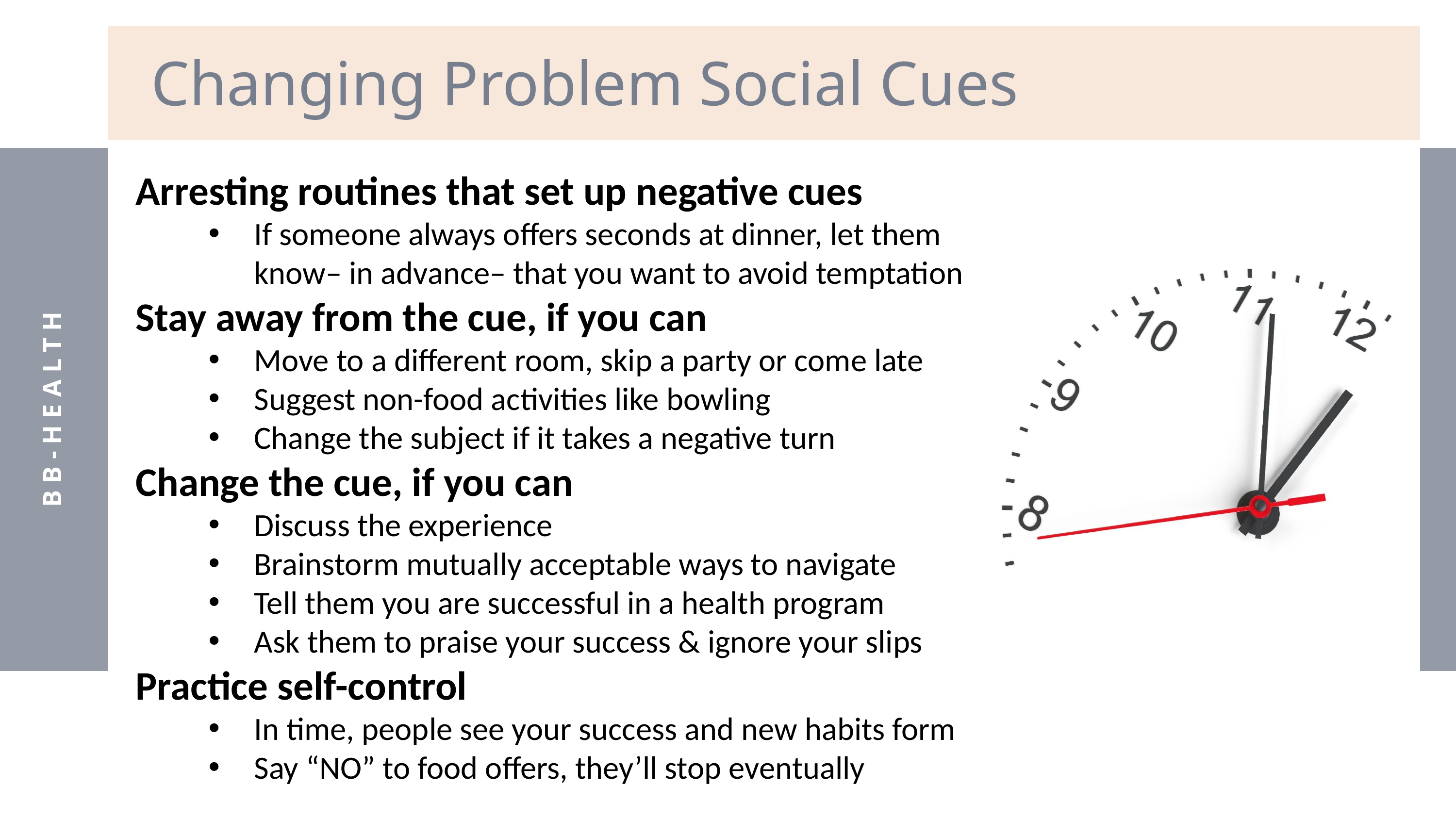

Changing Problem Social Cues
Arresting routines that set up negative cues
If someone always offers seconds at dinner, let them know– in advance– that you want to avoid temptation
Stay away from the cue, if you can
Move to a different room, skip a party or come late
Suggest non-food activities like bowling
Change the subject if it takes a negative turn
Change the cue, if you can
Discuss the experience
Brainstorm mutually acceptable ways to navigate
Tell them you are successful in a health program
Ask them to praise your success & ignore your slips
Practice self-control
In time, people see your success and new habits form
Say “NO” to food offers, they’ll stop eventually
BB-HEALTH

## Slide 9
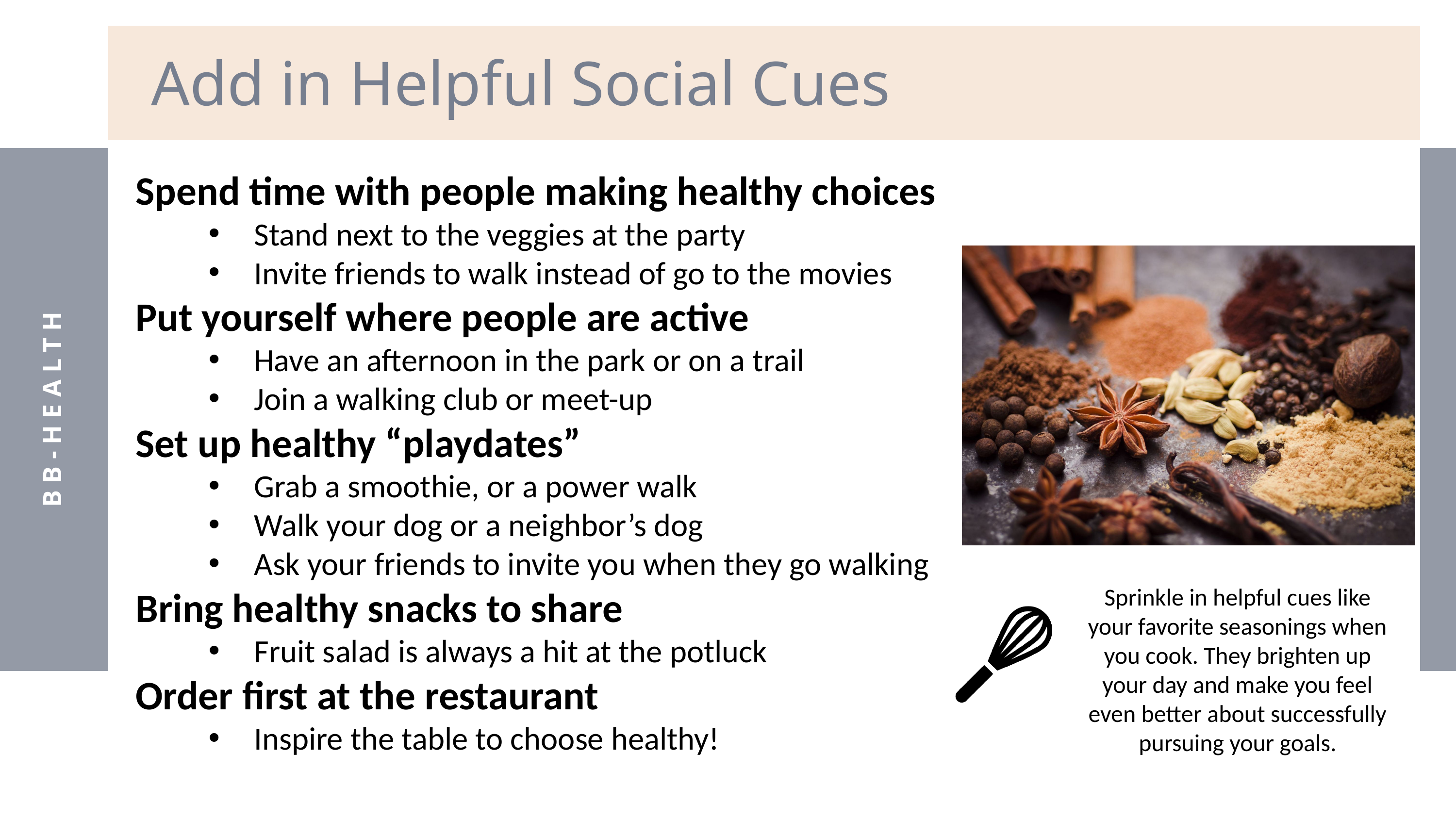

Add in Helpful Social Cues
Spend time with people making healthy choices
Stand next to the veggies at the party
Invite friends to walk instead of go to the movies
Put yourself where people are active
Have an afternoon in the park or on a trail
Join a walking club or meet-up
Set up healthy “playdates”
Grab a smoothie, or a power walk
Walk your dog or a neighbor’s dog
Ask your friends to invite you when they go walking
Bring healthy snacks to share
Fruit salad is always a hit at the potluck
Order first at the restaurant
Inspire the table to choose healthy!
BB-HEALTH
Sprinkle in helpful cues like your favorite seasonings when you cook. They brighten up your day and make you feel even better about successfully pursuing your goals.

## Slide 10
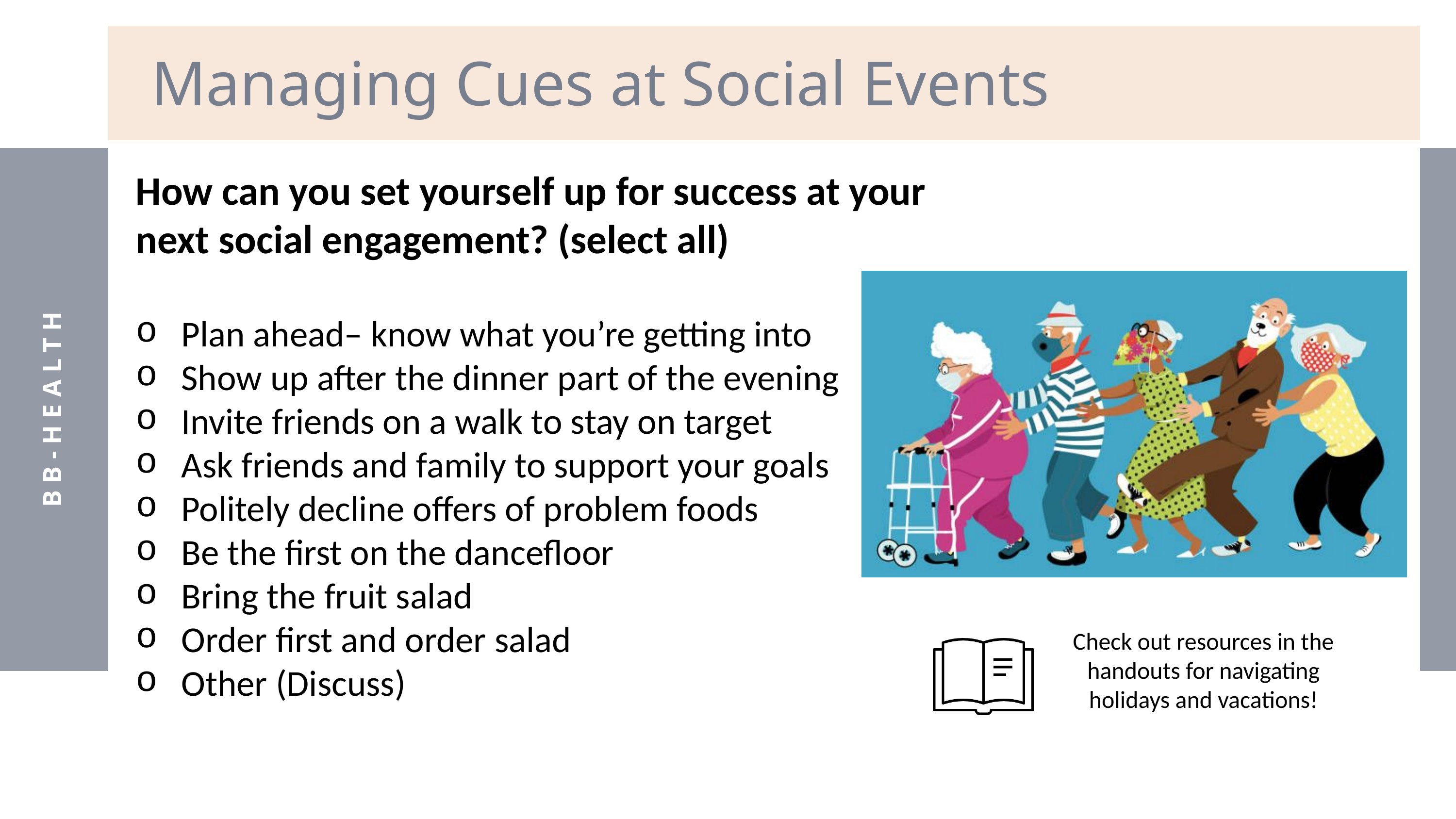

Managing Cues at Social Events
How can you set yourself up for success at your next social engagement? (select all)
Plan ahead– know what you’re getting into
Show up after the dinner part of the evening
Invite friends on a walk to stay on target
Ask friends and family to support your goals
Politely decline offers of problem foods
Be the first on the dancefloor
Bring the fruit salad
Order first and order salad
Other (Discuss)
BB-HEALTH
Check out resources in the handouts for navigating holidays and vacations!

## Slide 11
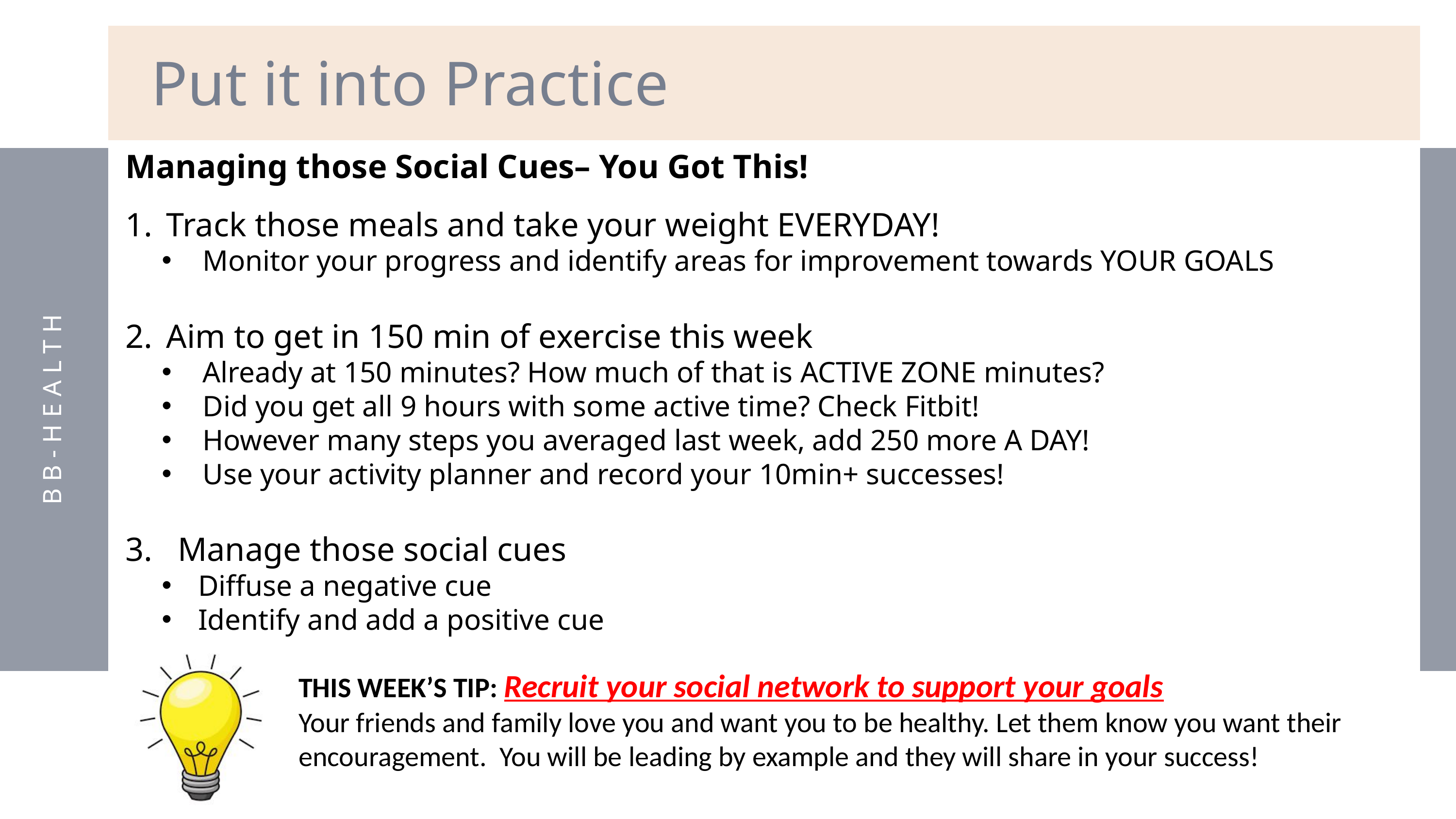

Put it into Practice
Managing those Social Cues– You Got This!
Track those meals and take your weight EVERYDAY!
Monitor your progress and identify areas for improvement towards YOUR GOALS
Aim to get in 150 min of exercise this week
Already at 150 minutes? How much of that is ACTIVE ZONE minutes?
Did you get all 9 hours with some active time? Check Fitbit!
However many steps you averaged last week, add 250 more A DAY!
Use your activity planner and record your 10min+ successes!
3. Manage those social cues
Diffuse a negative cue
Identify and add a positive cue
BB-HEALTH
THIS WEEK’S TIP: Recruit your social network to support your goals
Your friends and family love you and want you to be healthy. Let them know you want their encouragement. You will be leading by example and they will share in your success!

## Slide 12
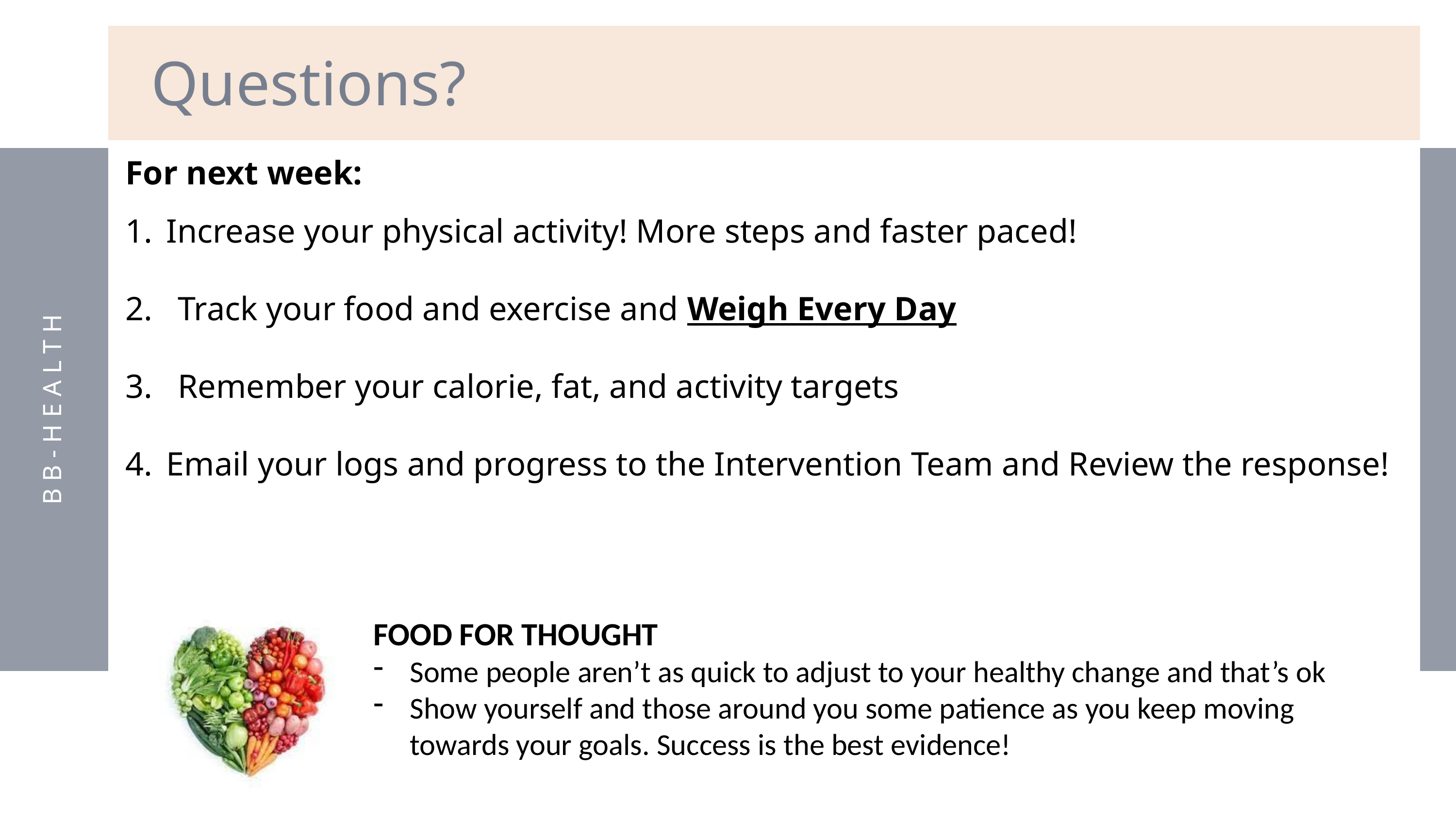

Questions?
For next week:
Increase your physical activity! More steps and faster paced!
2. Track your food and exercise and Weigh Every Day
3. Remember your calorie, fat, and activity targets
Email your logs and progress to the Intervention Team and Review the response!
BB-HEALTH
FOOD FOR THOUGHT
Some people aren’t as quick to adjust to your healthy change and that’s ok
Show yourself and those around you some patience as you keep moving towards your goals. Success is the best evidence!
