# Supplementary material for: Development and Feasibility of an eHealth Diabetes Prevention Program Adapted for Older Adults—Results from a Randomized Control Pilot Study
Source: Nutrients. 2024 Mar 23;16(7):930. doi: 10.3390/nu16070930 (PMC11154527; doi:10.3390/nu16070930)
Supplement: Supplementary file 1 [file nutrients-16-00930-s001.zip › Week11_SUPP.pdf]

BB-HEALTH

e-Healthy Older Adults Modification

Diabetes Prevention Program

WEEK #11
Make Social Cues Work for You!

Group Opening Discussion

B B - H E A L T H

WELCOME BACK! **HOW WAS LAST WEEK**?

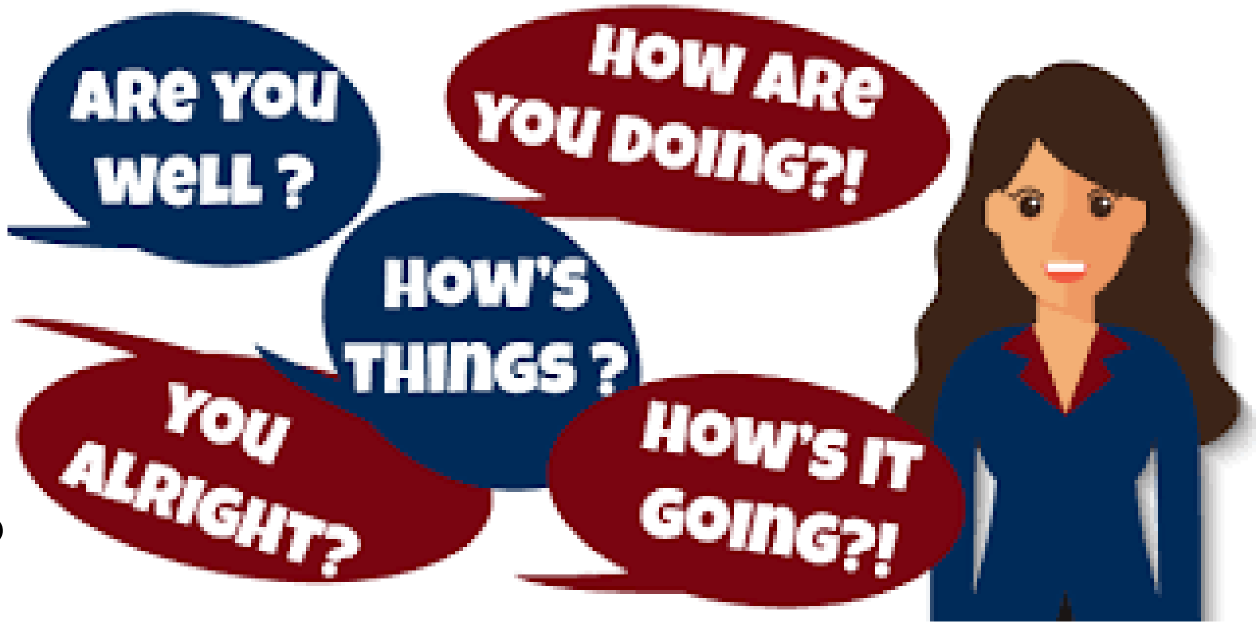

1. Did you track every meal?
2. Did you weigh every day, & record your weight today?
3. Did you practice eating health out of the home?
4. Did you ask for any modifications or solve any problems?
5. Did you email the Intervention Team and review the response?
6. What were your unique challenges and successes?

REMINDER: CHARGE YOUR FITBIT & SCALE DURING THIS MEETING!

How can we safeguard success?

<u>What's the difference between a diet and a healthy lifestyle ? (Discuss)</u>

<u>Who are you least likely to eat healthy around? (select one)</u>

- Friends
- Family
- Co-Worker
- Spouse
- Children
- Activity partners
- Your boss
- A client
- other, (discuss)

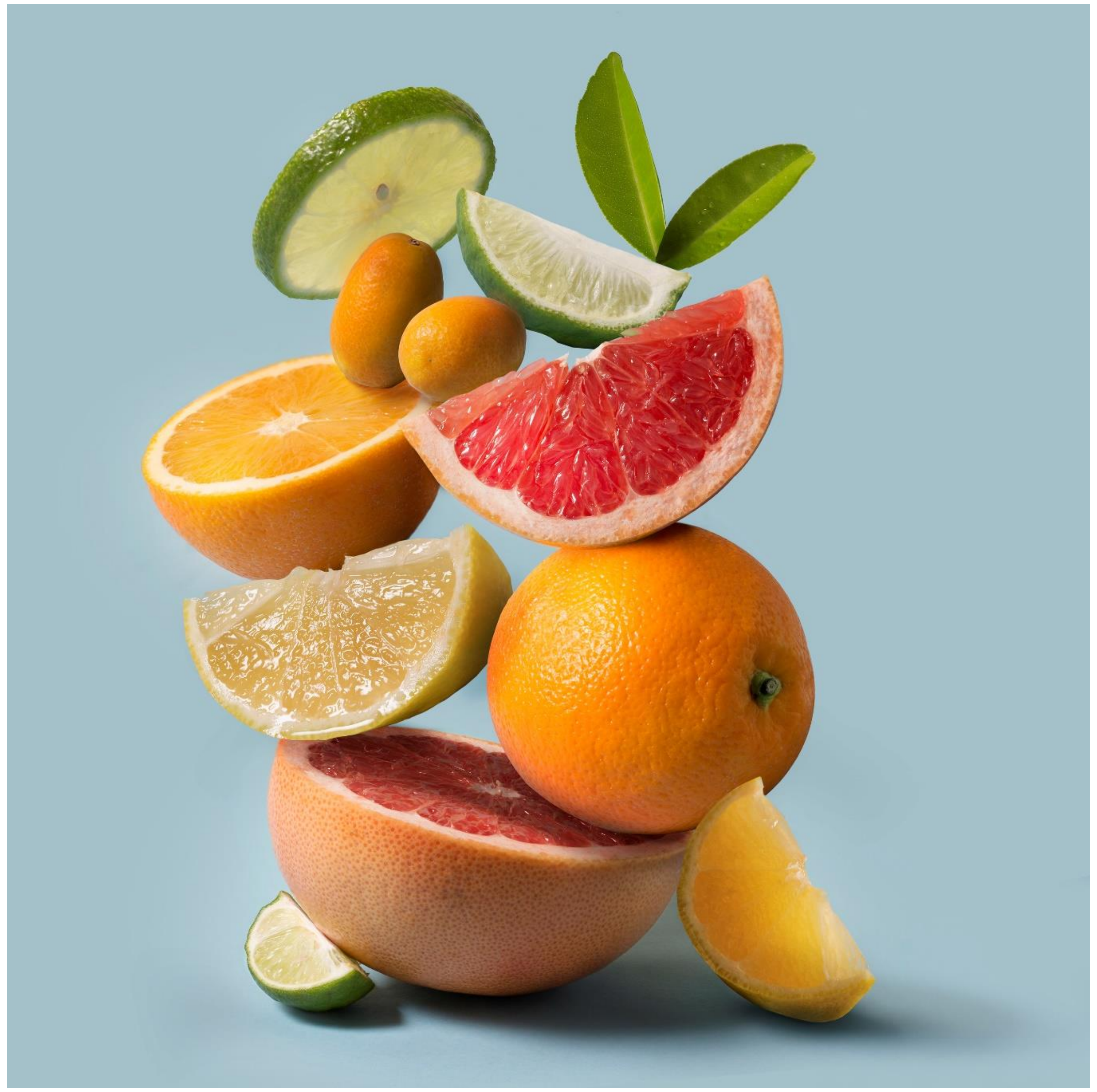

Finding support all around us

BB-HEALTH

<u>What are the ways in which your community supports a healthy lifestyle? (Select all)</u>

- Parks and recreation centers
- Farmer’s Markets
- Public Transit and Bike shares
- YMCA
- Food Pantry
- Local walking group
- Dog park
- Community Garden
- Cooking Classes
- Potlucks
- Other (discuss)

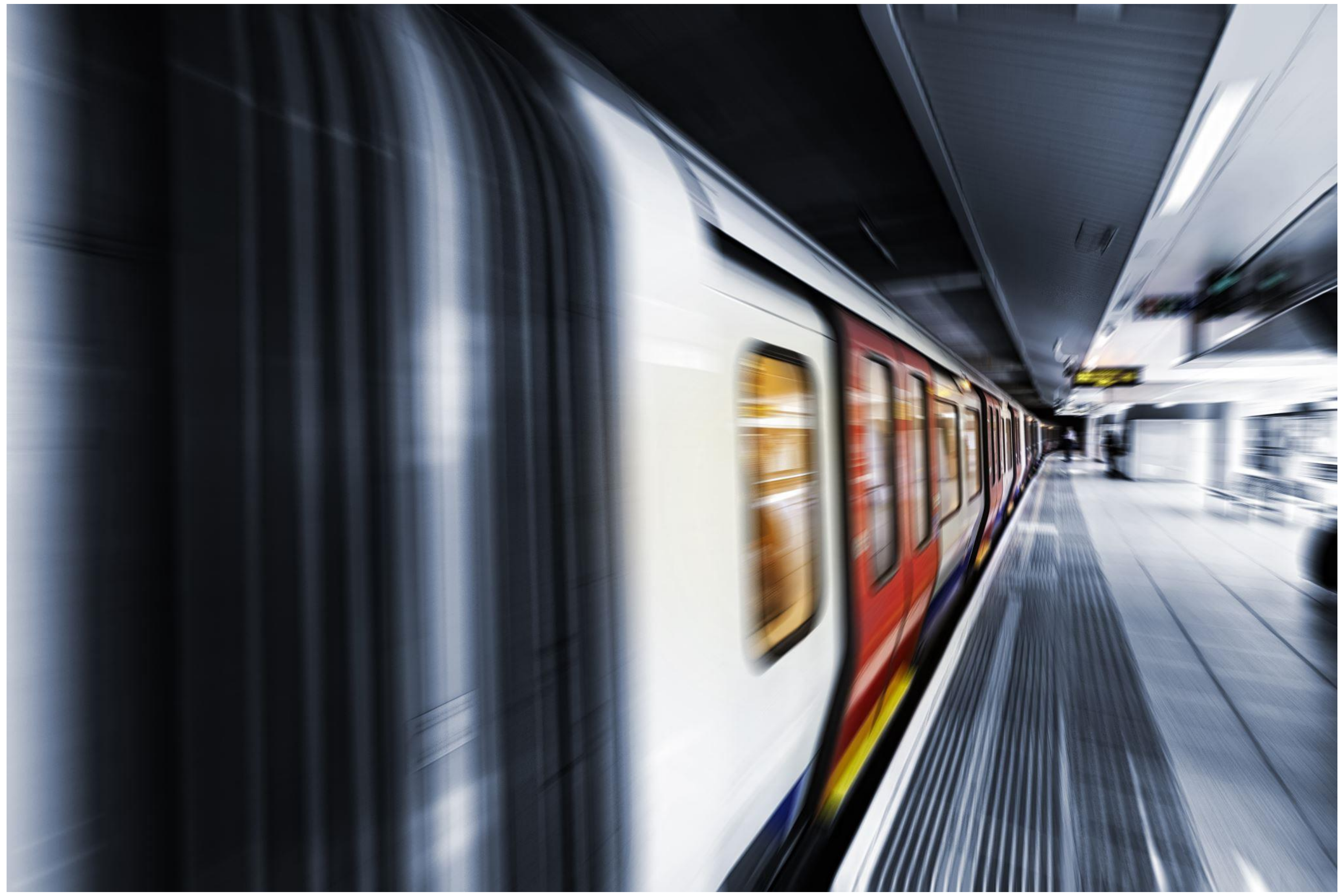

Social Cues vs Environmental Cues

B B - H E A L T H

Just like seeing a box of cookies can make you want to eat a cookie, certain people or social environments can also CUE your habits– both good and bad!

- Social Cues can be in conflict with your goals
- Social cues can help support your goals
- You can recruit social connections to be supportive—
  - You can even CONVERT problematic ones into supportive ones!

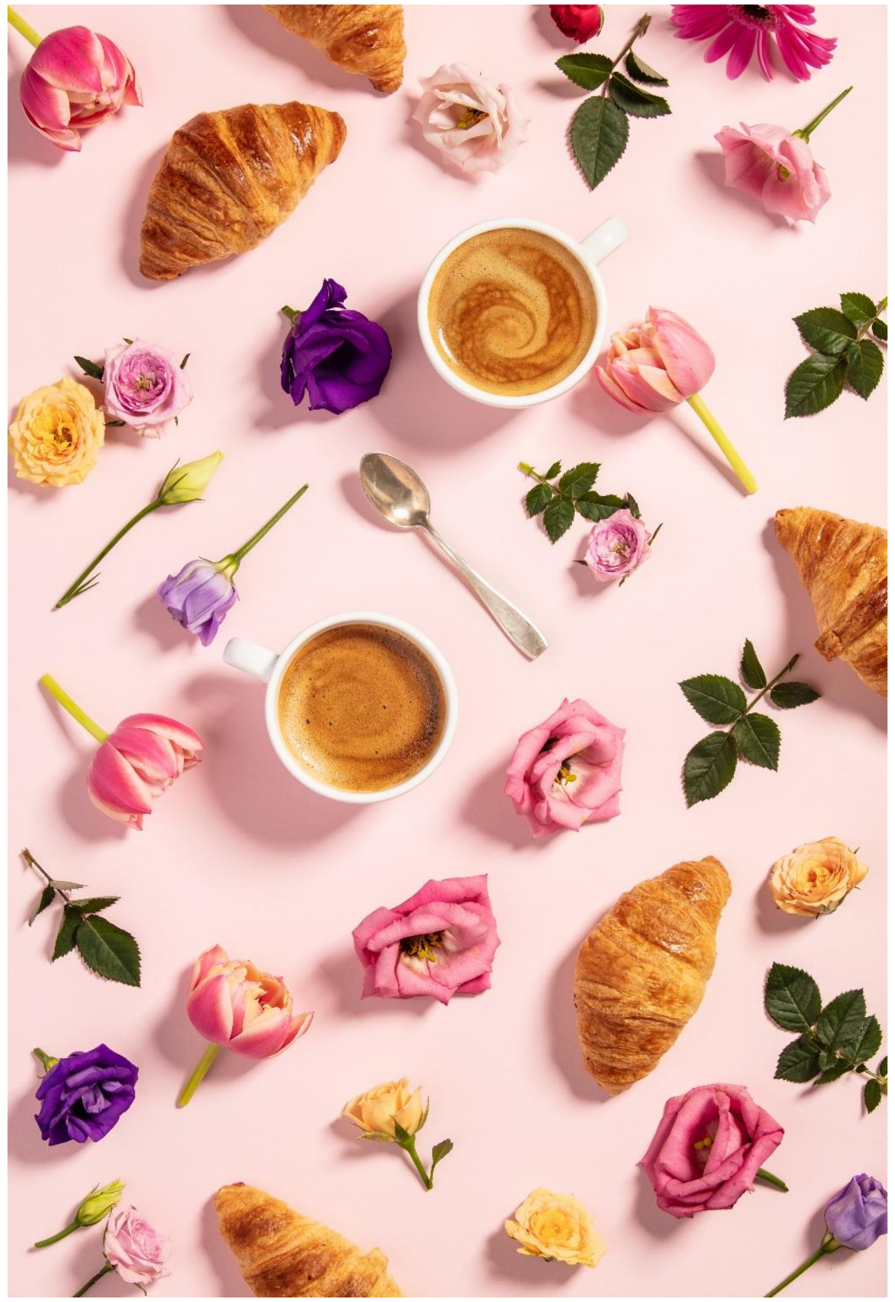

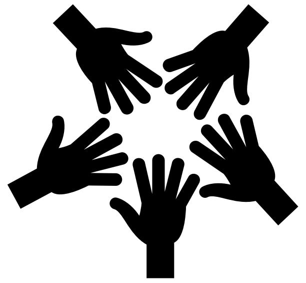

Setting yourself up for success means identifying the good and the bad!
Learning to anticipate obstacles is just as important as learning to stack the deck in your favor. You can set yourself up for success by identifying them and learning to optimize in either case.

Problem Social Cues

1. Seeing others eating problem foods or being sedentary
    - Do you want to eat when others eat?
    - Do you turn into a couch potato when others loaf around?
2. Being offered, or pressured
    - Can you resist your favorite foods when cutting back?
    - Can you keep your get up and go, when others relax?
    - Can you turn down pushy friends and family?
3. When your social network is rarely active
    - Will you be the healthy inspiration?
4. Nagging, even for healthy foods
    - Does even goal-oriented nagging backfire for you?
5. Complaints about your healthy lifestyle
    - Are you immune to complaints?

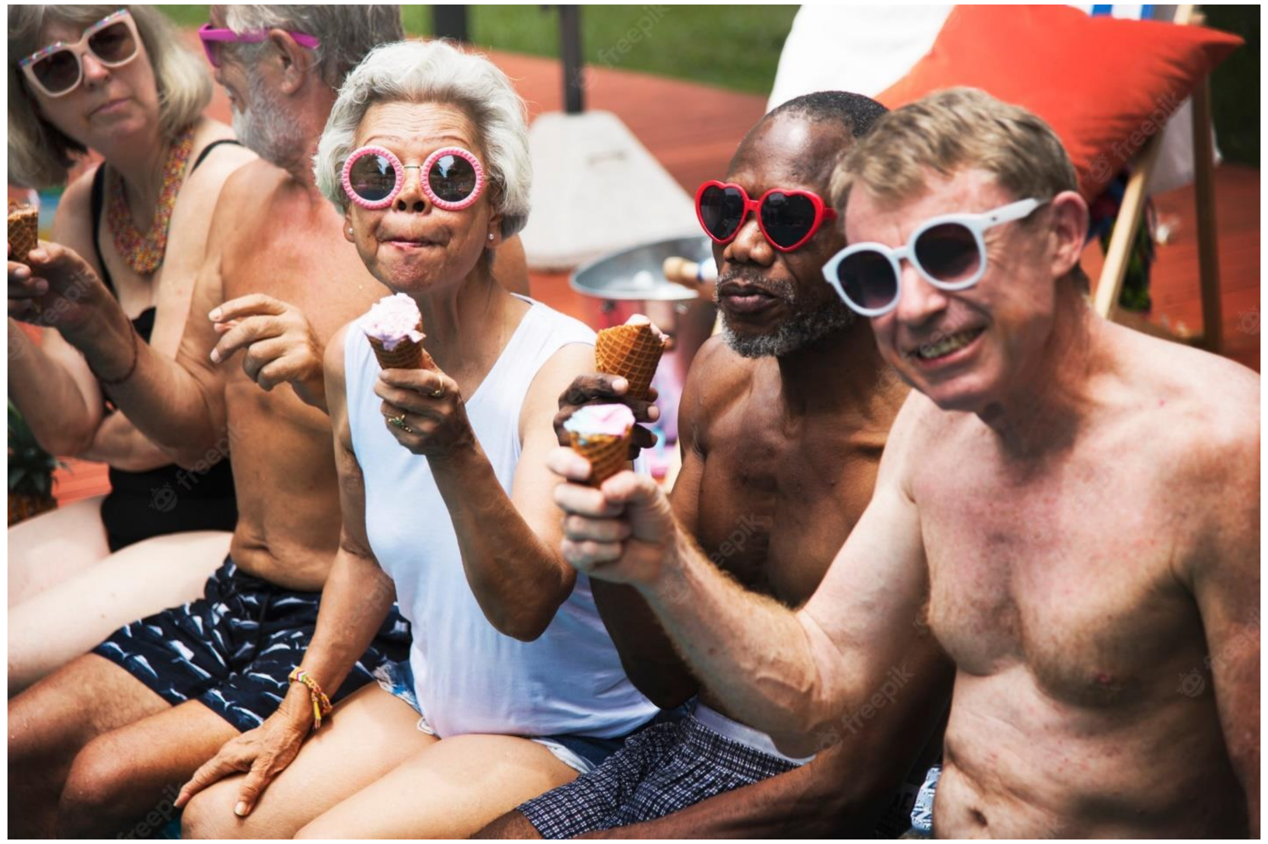

The way we respond to cues can become a habit! Instead, talk back, negotiate and self-advocate for your health. They are your goals, and you **form new healthy habits everyday**!

Positive and Helpful Social Cues

BB-HEALTH

1. **Inspired by seeing people active or eating healthy**
    - Do you order healthy when someone else does?
    - Do you join in when others are being active?
2. **Invited to healthy activities and meals**
    - Will you accept an invitation for a healthy day?
    - Will you let people know you are up for healthy visits?
3. **Being praised**
    - Can you feel good when you inspire healthy choices?
    - Can you accept praise for your new delicious meals?
4. **Hearing compliments**
    - Does the good feedback help reinforce healthy choices?

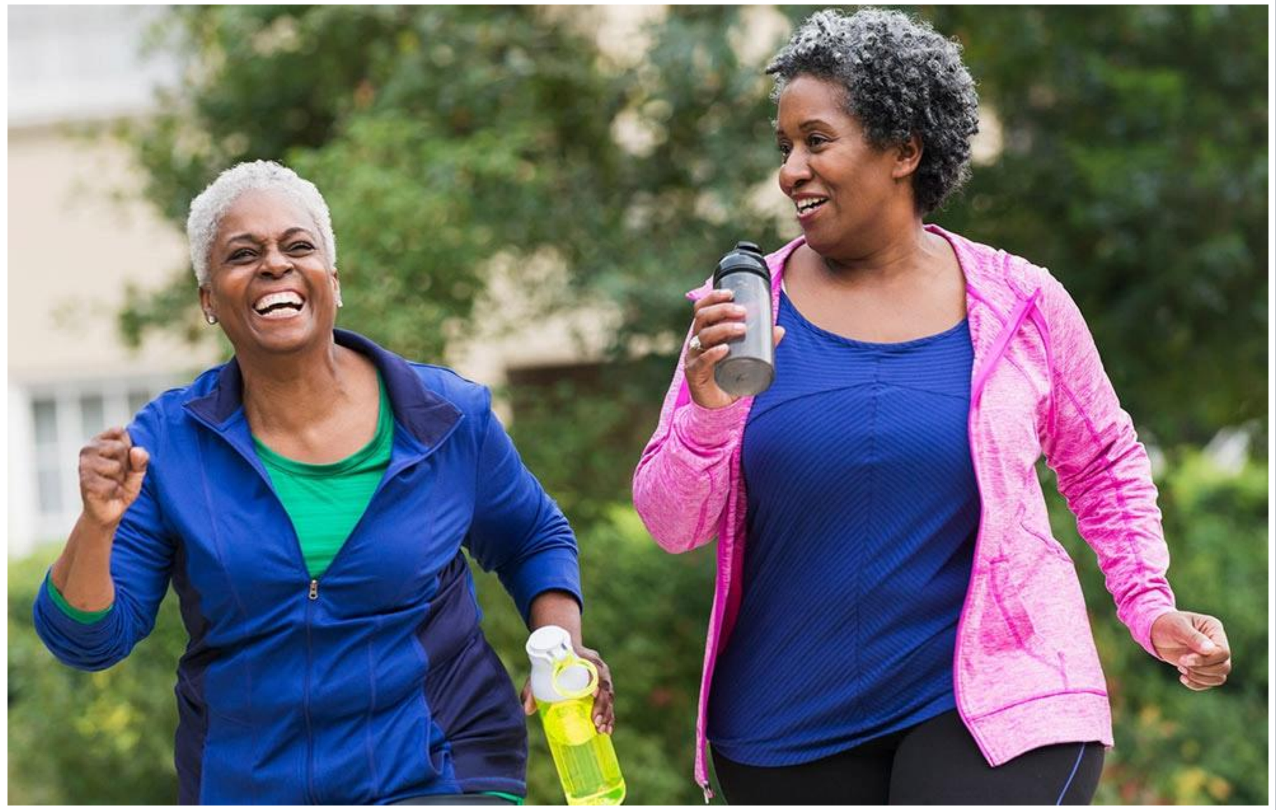

Change is hard, not just on you but on those around you too. Take a moment to be grateful to yourself & your friends and family for prioritizing your health.
That way you lead by example with positive social cues!

Changing Problem Social Cues

Arresting routines that set up negative cues

- If someone always offers seconds at dinner, let them know– in advance– that you want to avoid temptation

Stay away from the cue, if you can

- Move to a different room, skip a party or come late
- Suggest non-food activities like bowling
- Change the subject if it takes a negative turn

Change the cue, if you can

- Discuss the experience
- Brainstorm mutually acceptable ways to navigate
- Tell them you are successful in a health program
- Ask them to praise your success & ignore your slips

Practice self-control

- In time, people see your success and new habits form
- Say “NO” to food offers, they’ll stop eventually

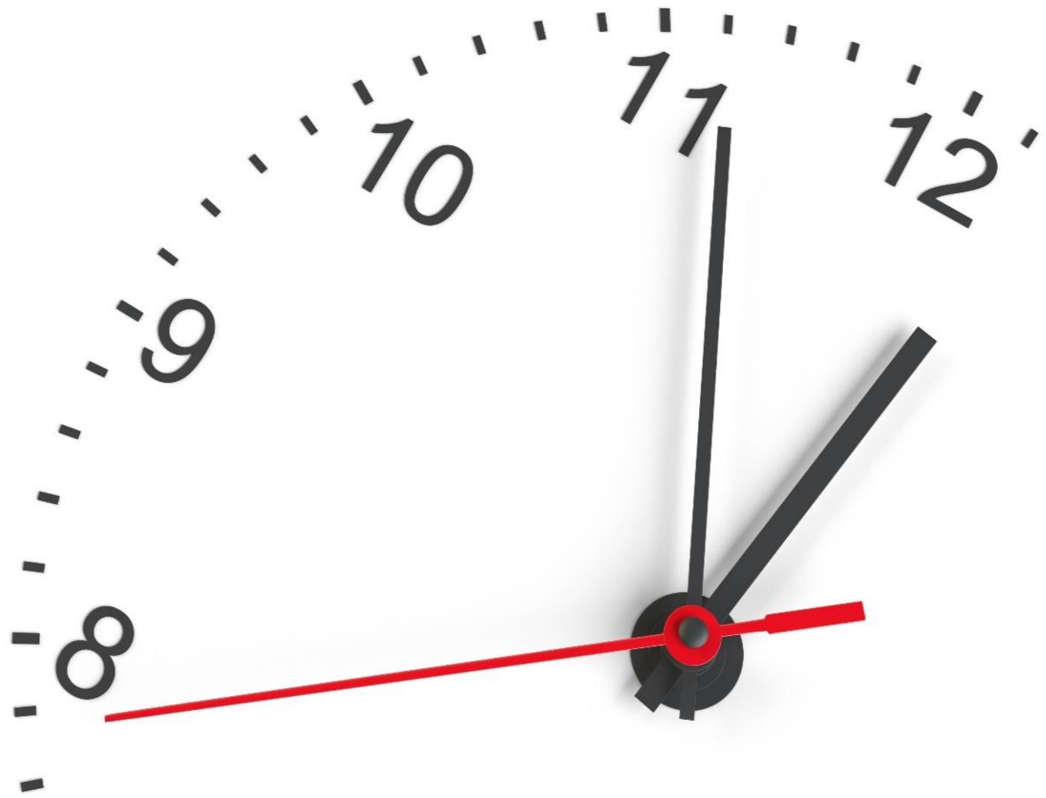

Add in Helpful Social Cues

B B - H E A L T H

Spend time with people making healthy choices

- Stand next to the veggies at the party
- Invite friends to walk instead of go to the movies

Put yourself where people are active

- Have an afternoon in the park or on a trail
- Join a walking club or meet-up

Set up healthy "playdates"

- Grab a smoothie, or a power walk
- Walk your dog or a neighbor's dog
- Ask your friends to invite you when they go walking

Bring healthy snacks to share

- Fruit salad is always a hit at the potluck

Order first at the restaurant

- Inspire the table to choose healthy!

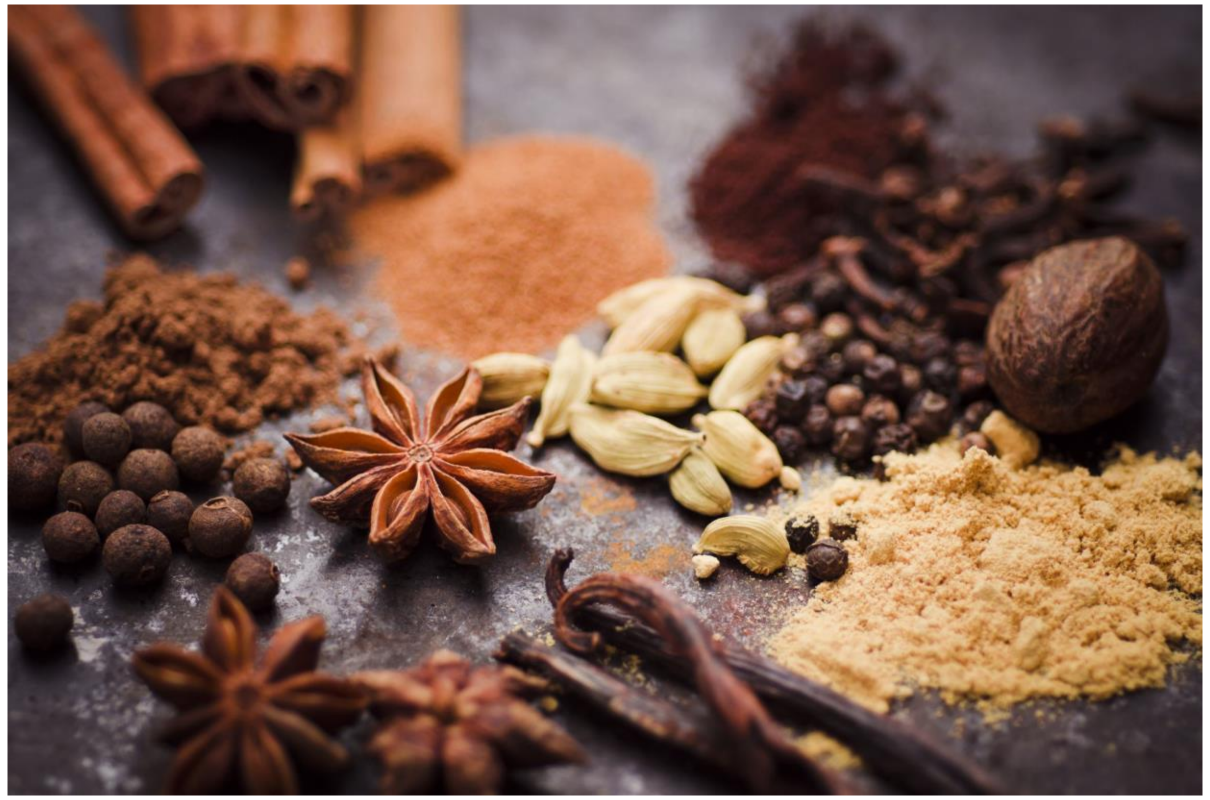

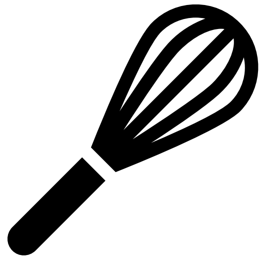

Sprinkle in helpful cues like your favorite seasonings when you cook. They brighten up your day and make you feel even better about successfully pursuing your goals.

Managing Cues at Social Events

How can you set yourself up for success at your next social engagement? (select all)

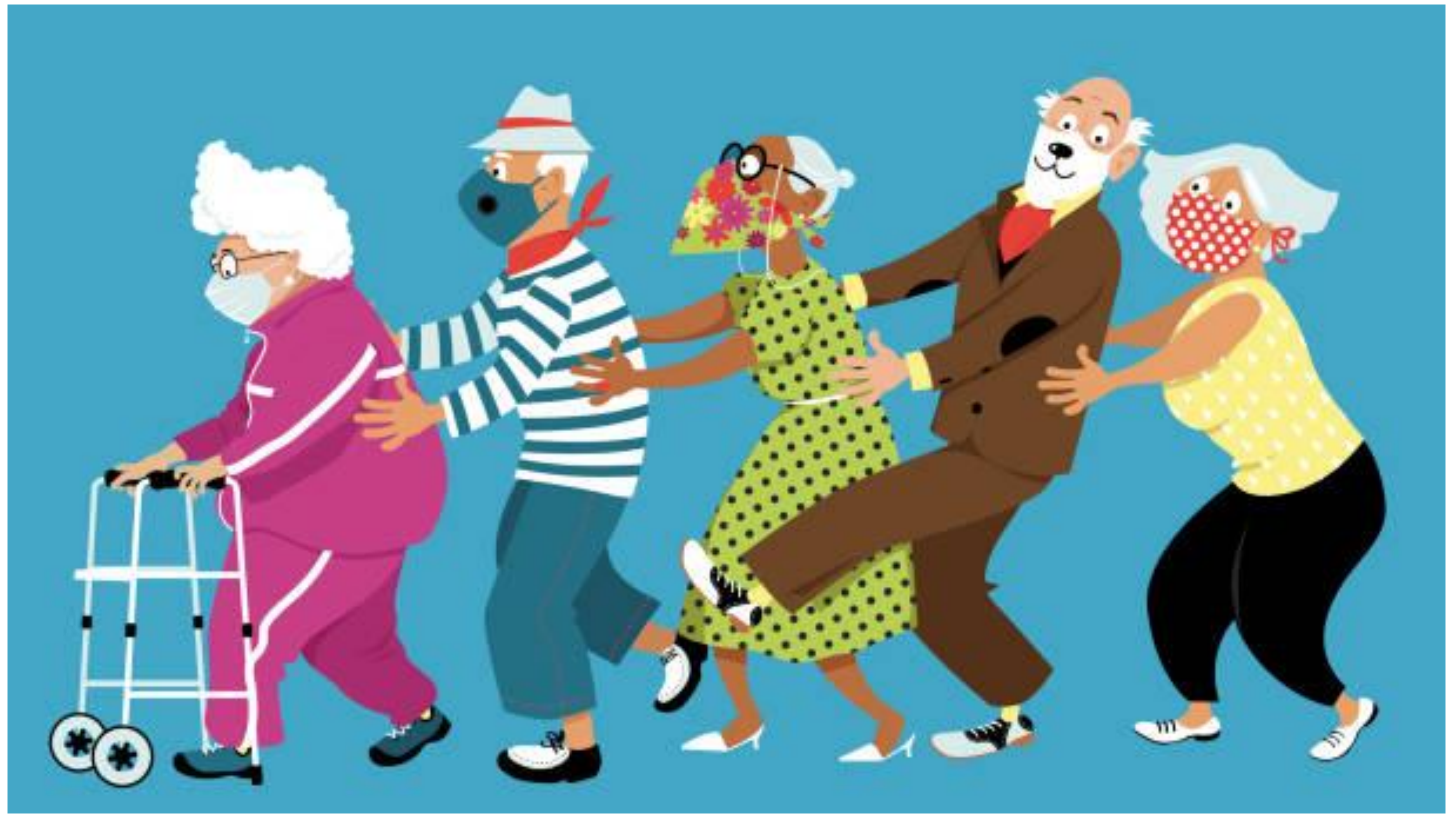

- Plan ahead– know what you're getting into
- Show up after the dinner part of the evening
- Invite friends on a walk to stay on target
- Ask friends and family to support your goals
- Politely decline offers of problem foods
- Be the first on the dancefloor
- Bring the fruit salad
- Order first and order salad
- Other (Discuss)

Check out resources in the handouts for navigating holidays and vacations!

Put it into Practice

Managing those Social Cues– You Got This!

1. Track those meals and take your weight EVERYDAY!
   - Monitor your progress and identify areas for improvement towards YOUR GOALS

2. Aim to get in 150 min of exercise this week
   - Already at 150 minutes? How much of that is ACTIVE ZONE minutes?
   - Did you get all 9 hours with some active time? Check Fitbit!
   - However many steps you averaged last week, add 250 more A DAY!
   - Use your activity planner and record your 10min+ successes!

3. Manage those social cues
   - Diffuse a negative cue
   - Identify and add a positive cue

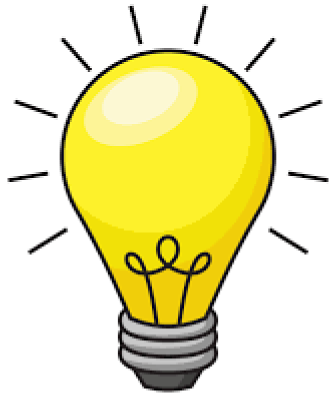

THIS WEEK'S TIP: ***Recruit your social network to support your goals***
Your friends and family love you and want you to be healthy. Let them know you want their encouragement. You will be leading by example and they will share in your success!

Questions?

B B - H E A L T H

For next week:

1. Increase your physical activity! More steps and faster paced!
2. Track your food and exercise and **Weigh Every Day**
3. Remember your calorie, fat, and activity targets
4. Email your logs and progress to the Intervention Team and Review the response!

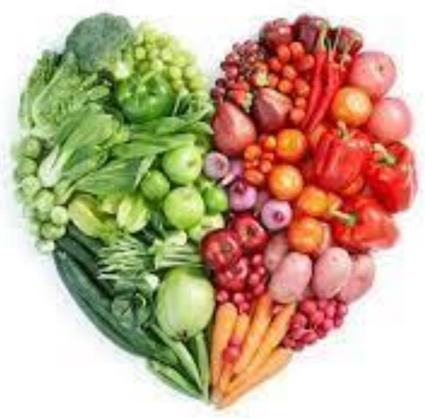

FOOD FOR THOUGHT

- Some people aren't as quick to adjust to your healthy change and that's ok
- Show yourself and those around you some patience as you keep moving towards your goals. Success is the best evidence!
