# Supplementary material for: Development and Feasibility of an eHealth Diabetes Prevention Program Adapted for Older Adults—Results from a Randomized Control Pilot Study
Source: Nutrients. 2024 Mar 23;16(7):930. doi: 10.3390/nu16070930 (PMC11154527; doi:10.3390/nu16070930)
Supplement: Supplementary file 1 [file nutrients-16-00930-s001.zip › Week12.pptx]

## Slide 1
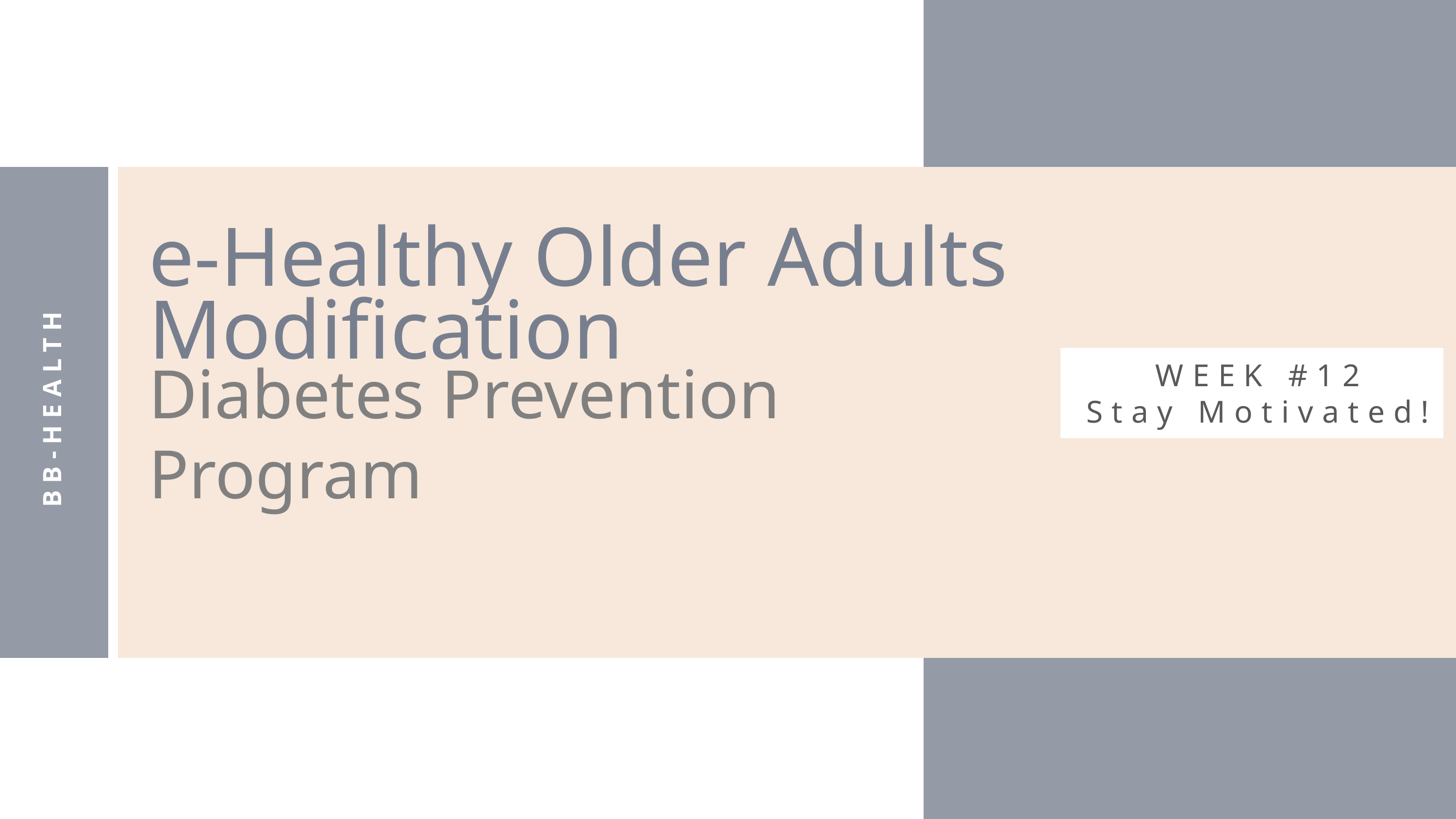

OPEN REPORTS
e-Healthy Older Adults Modification
WEEK #12
Stay Motivated!
Diabetes Prevention Program
BB-HEALTH

## Slide 2
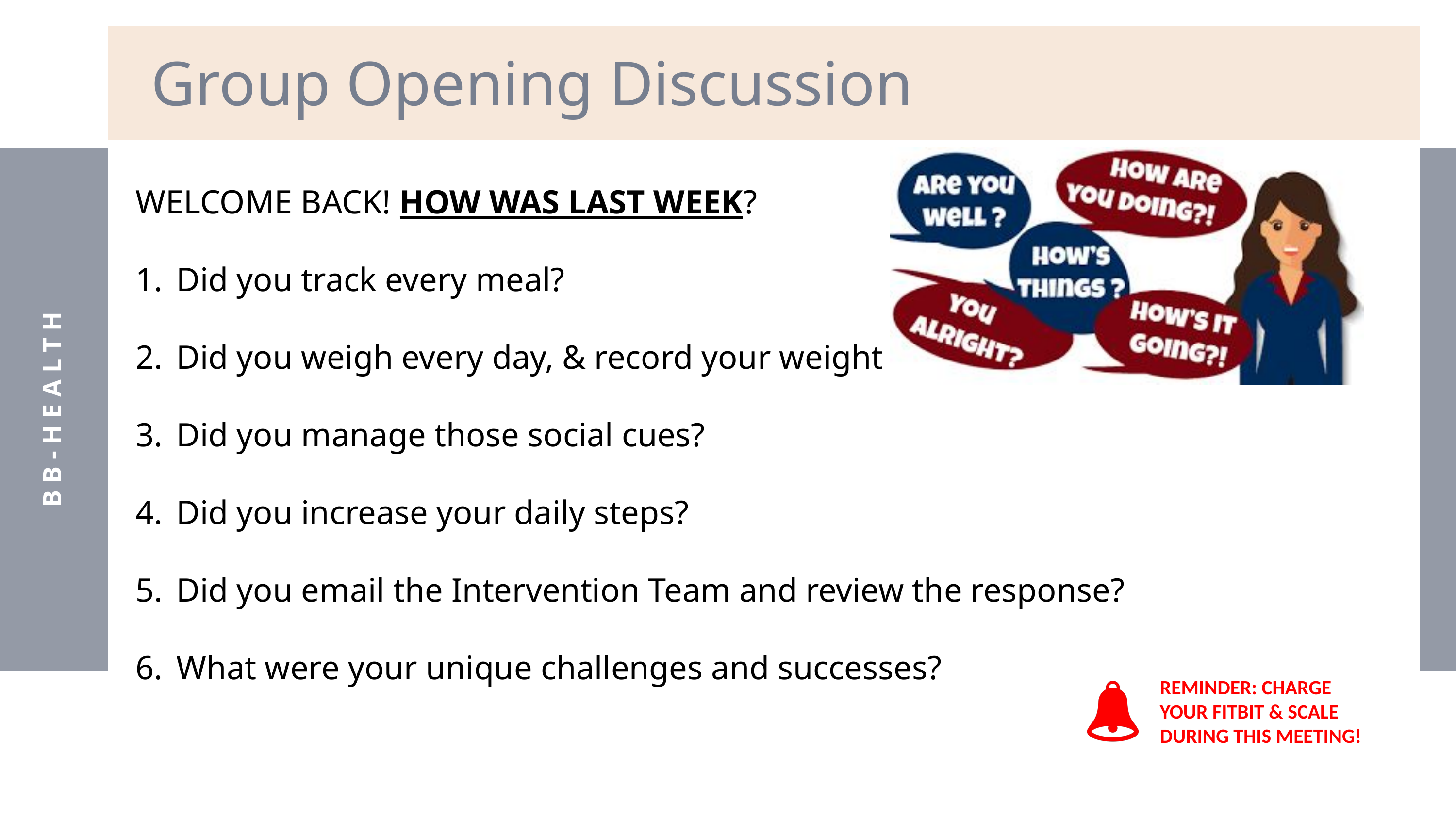

Group Opening Discussion
WELCOME BACK! HOW WAS LAST WEEK?
Did you track every meal?
Did you weigh every day, & record your weight today?
Did you manage those social cues?
Did you increase your daily steps?
Did you email the Intervention Team and review the response?
What were your unique challenges and successes?
BB-HEALTH
REMINDER: CHARGE YOUR FITBIT & SCALE DURING THIS MEETING!

## Slide 3
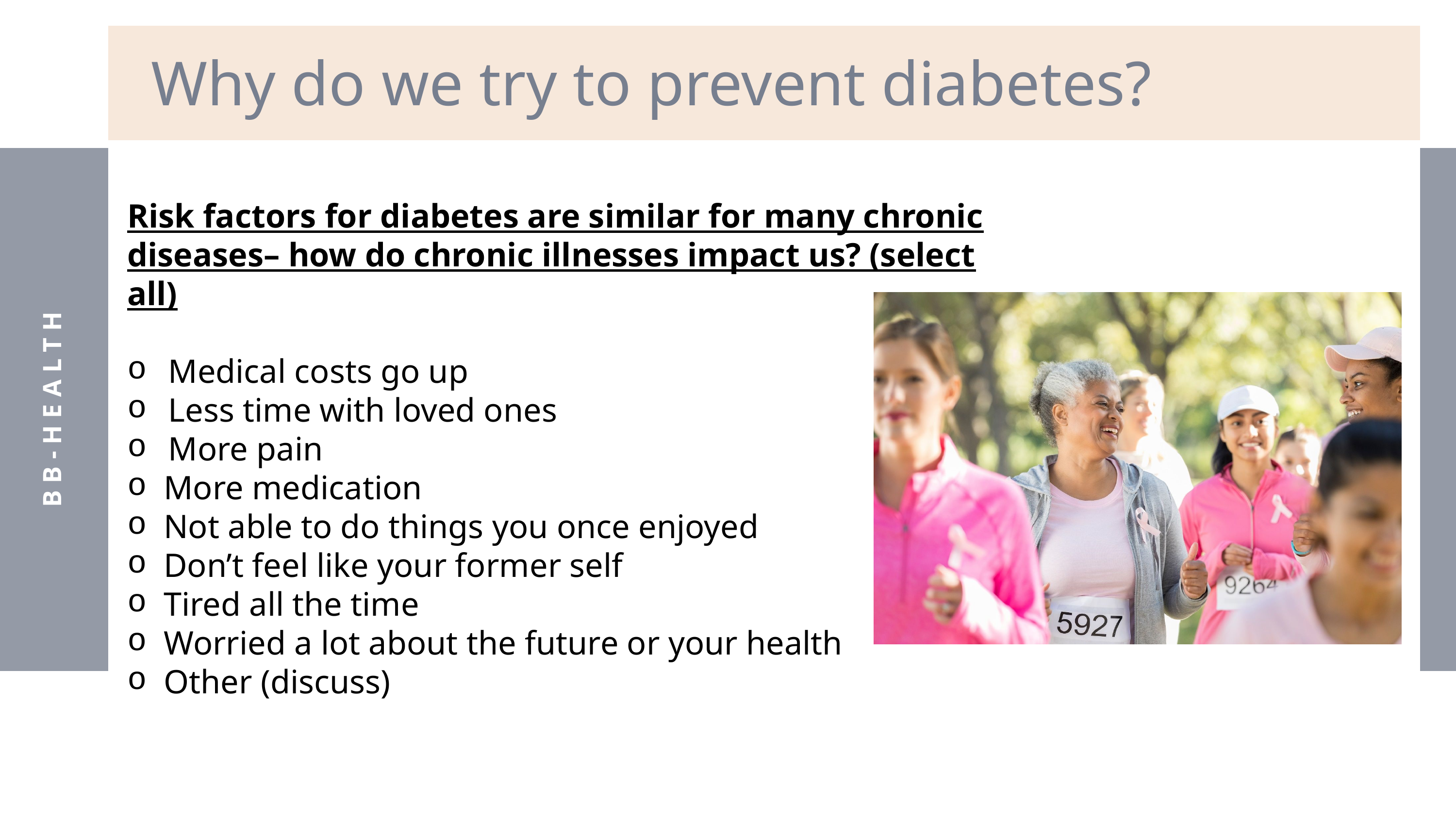

Why do we try to prevent diabetes?
Risk factors for diabetes are similar for many chronic diseases– how do chronic illnesses impact us? (select all)
Medical costs go up
Less time with loved ones
More pain
More medication
Not able to do things you once enjoyed
Don’t feel like your former self
Tired all the time
Worried a lot about the future or your health
Other (discuss)
BB-HEALTH

## Slide 4
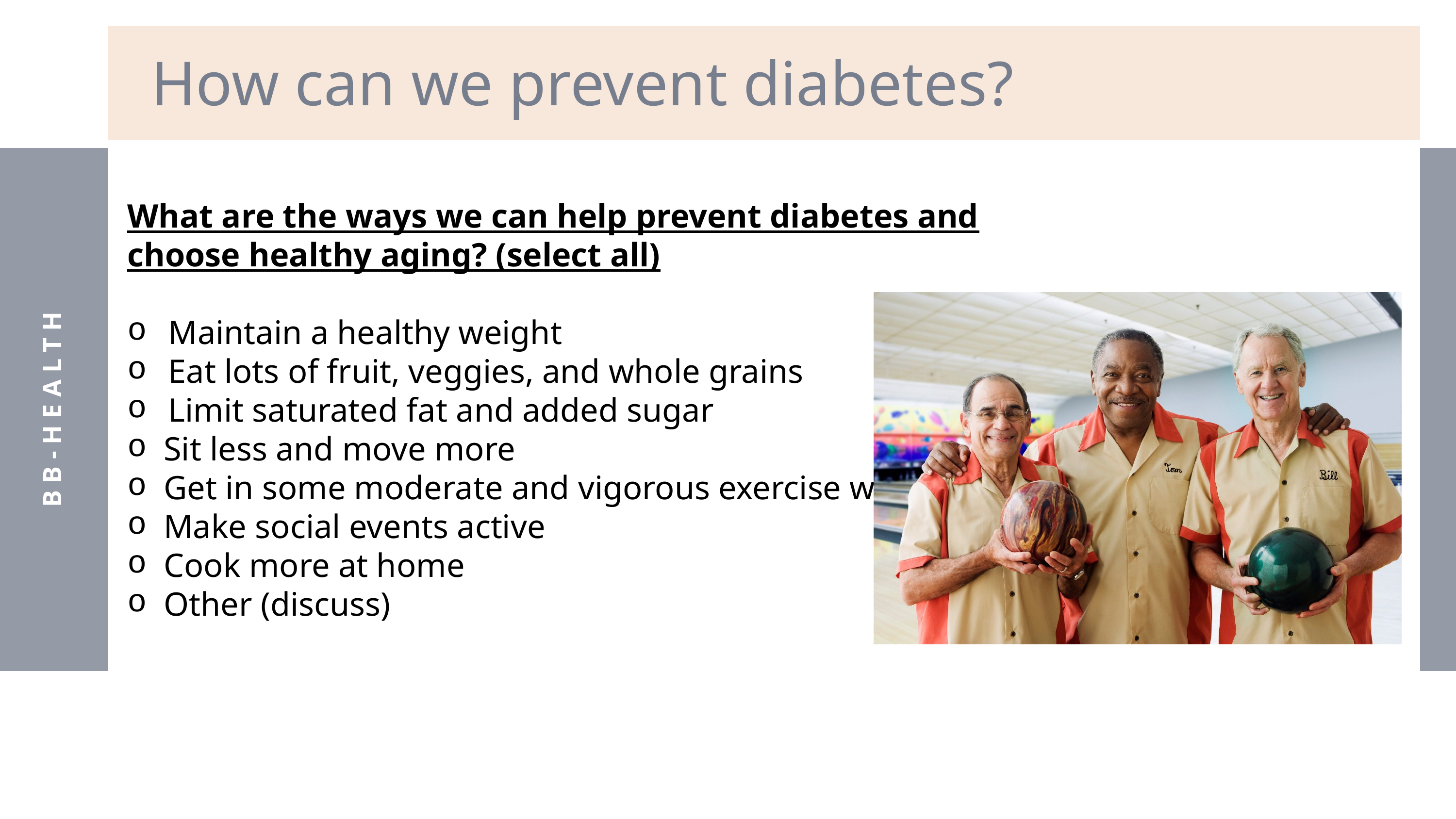

How can we prevent diabetes?
What are the ways we can help prevent diabetes and choose healthy aging? (select all)
Maintain a healthy weight
Eat lots of fruit, veggies, and whole grains
Limit saturated fat and added sugar
Sit less and move more
Get in some moderate and vigorous exercise weekly
Make social events active
Cook more at home
Other (discuss)
BB-HEALTH

## Slide 5
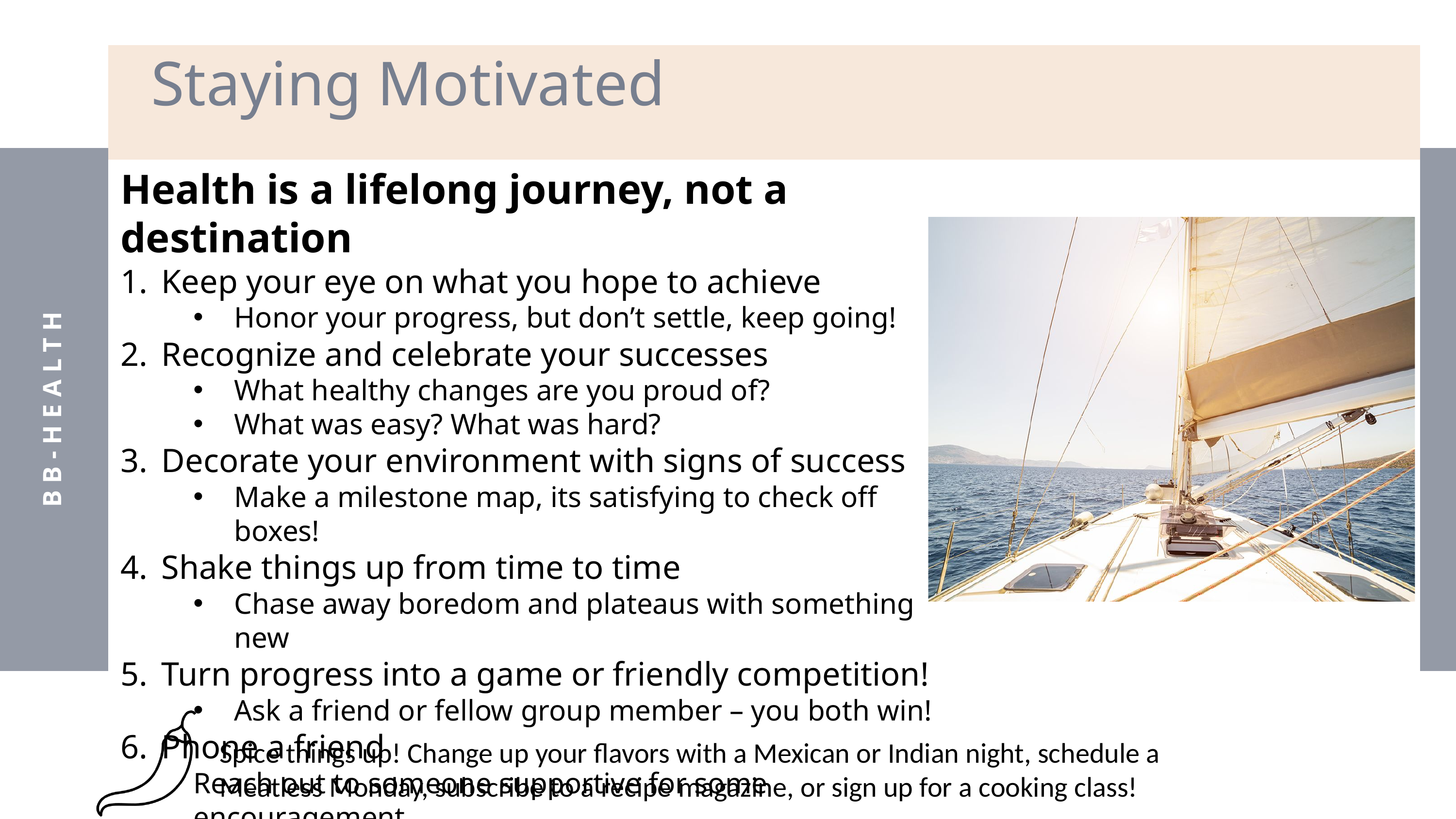

Staying Motivated
Health is a lifelong journey, not a destination
Keep your eye on what you hope to achieve
Honor your progress, but don’t settle, keep going!
Recognize and celebrate your successes
What healthy changes are you proud of?
What was easy? What was hard?
Decorate your environment with signs of success
Make a milestone map, its satisfying to check off boxes!
Shake things up from time to time
Chase away boredom and plateaus with something new
Turn progress into a game or friendly competition!
Ask a friend or fellow group member – you both win!
Phone a friend
Reach out to someone supportive for some encouragement
BB-HEALTH
Spice things up! Change up your flavors with a Mexican or Indian night, schedule a Meatless Monday, subscribe to a recipe magazine, or sign up for a cooking class!

## Slide 6
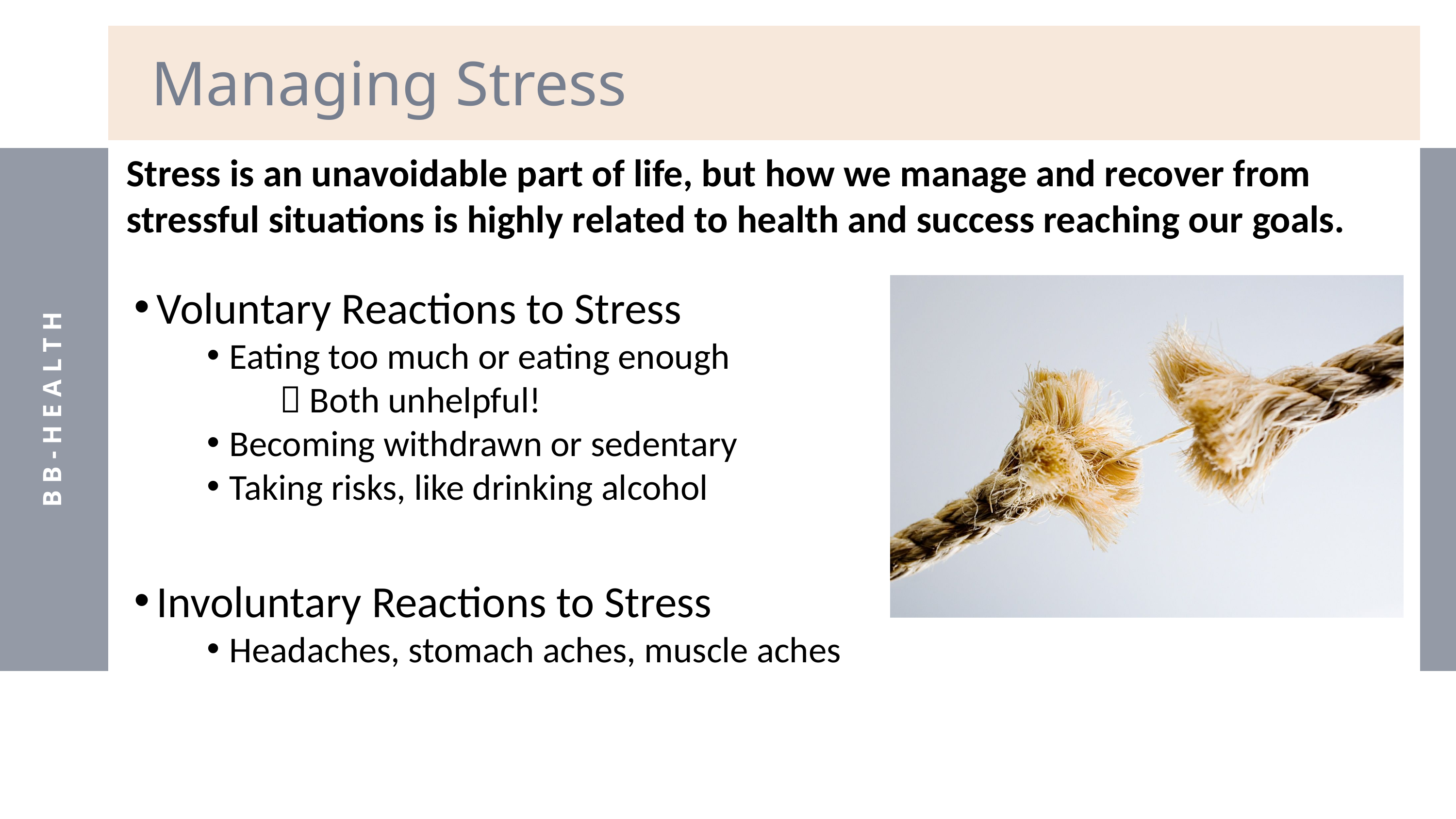

Managing Stress
Stress is an unavoidable part of life, but how we manage and recover from stressful situations is highly related to health and success reaching our goals.
Voluntary Reactions to Stress
Eating too much or eating enough
 Both unhelpful!
Becoming withdrawn or sedentary
Taking risks, like drinking alcohol
Involuntary Reactions to Stress
Headaches, stomach aches, muscle aches
BB-HEALTH

## Slide 7
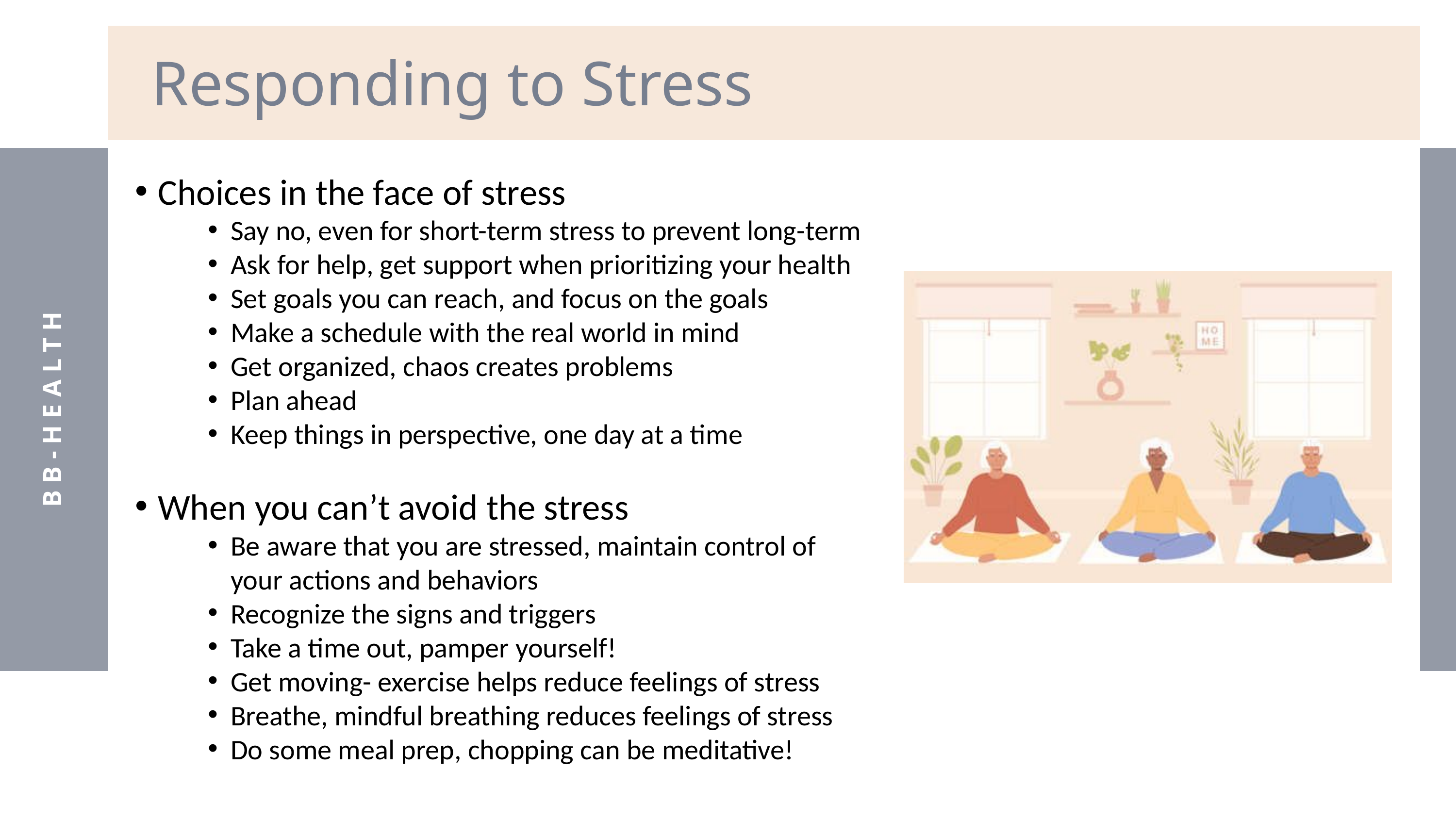

Responding to Stress
Choices in the face of stress
Say no, even for short-term stress to prevent long-term
Ask for help, get support when prioritizing your health
Set goals you can reach, and focus on the goals
Make a schedule with the real world in mind
Get organized, chaos creates problems
Plan ahead
Keep things in perspective, one day at a time
When you can’t avoid the stress
Be aware that you are stressed, maintain control of your actions and behaviors
Recognize the signs and triggers
Take a time out, pamper yourself!
Get moving- exercise helps reduce feelings of stress
Breathe, mindful breathing reduces feelings of stress
Do some meal prep, chopping can be meditative!
BB-HEALTH

## Slide 8
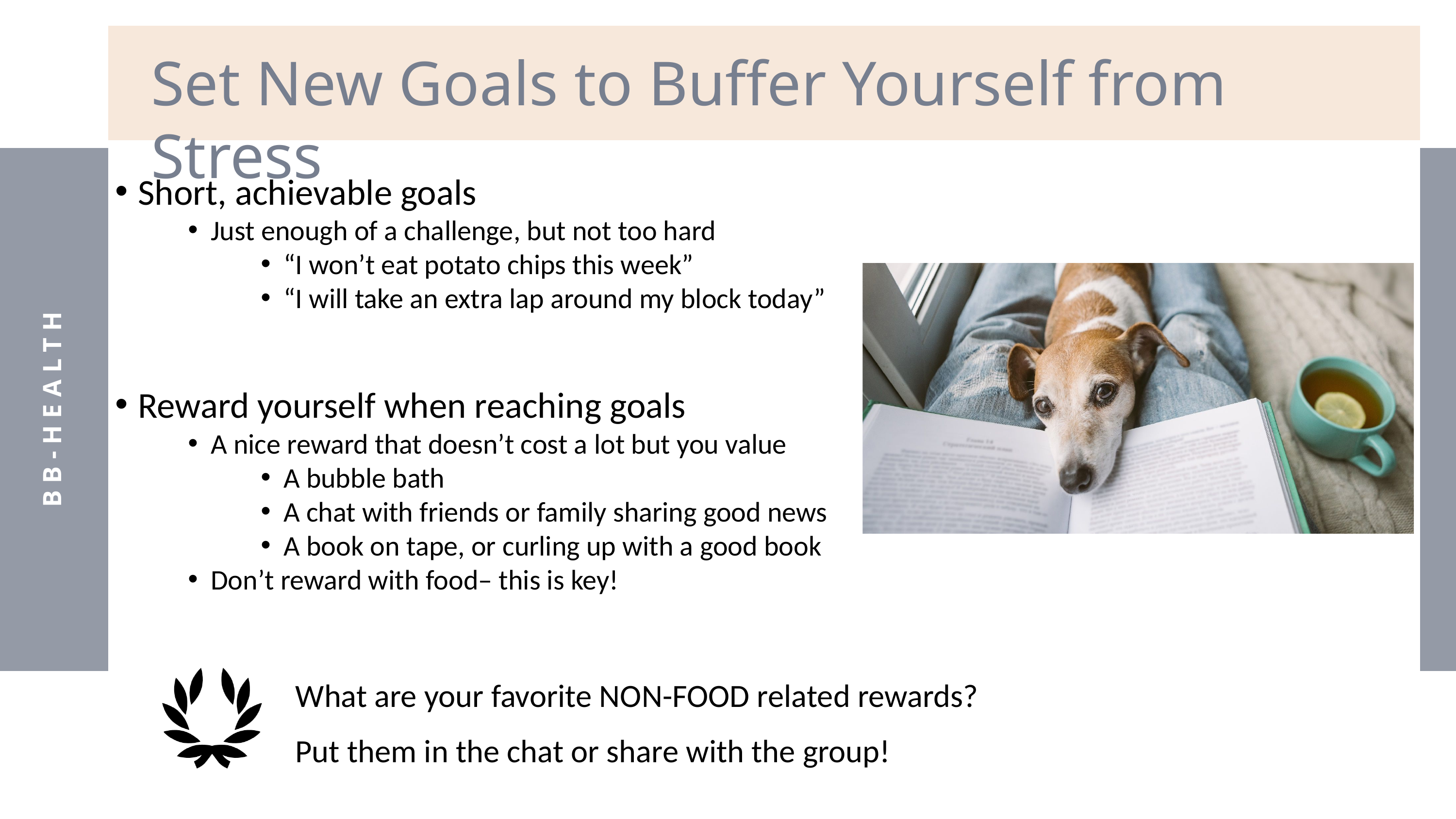

Set New Goals to Buffer Yourself from Stress
Short, achievable goals
Just enough of a challenge, but not too hard
“I won’t eat potato chips this week”
“I will take an extra lap around my block today”
Reward yourself when reaching goals
A nice reward that doesn’t cost a lot but you value
A bubble bath
A chat with friends or family sharing good news
A book on tape, or curling up with a good book
Don’t reward with food– this is key!
BB-HEALTH
What are your favorite NON-FOOD related rewards?
Put them in the chat or share with the group!

## Slide 9
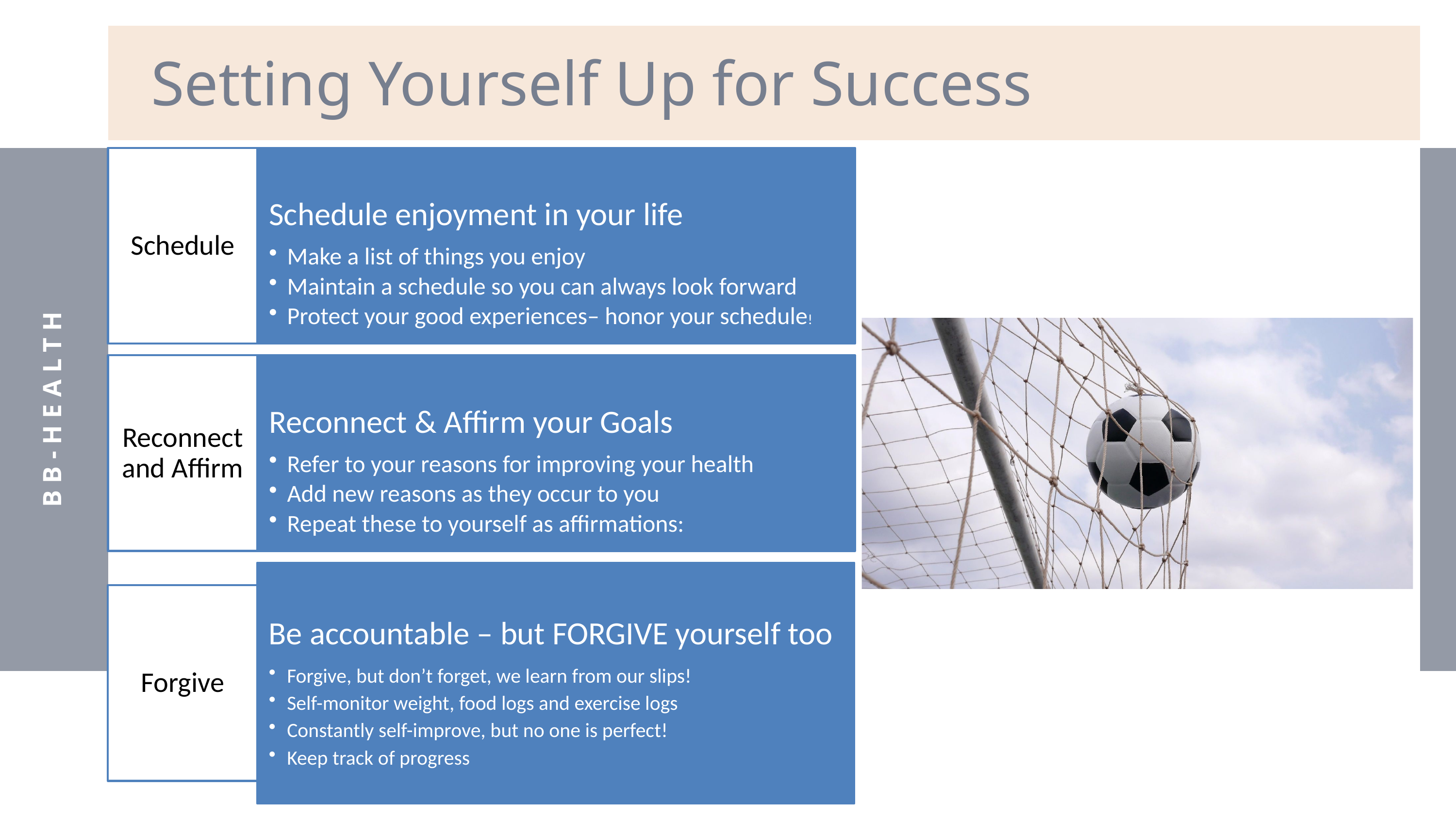

Setting Yourself Up for Success
BB-HEALTH

## Slide 10
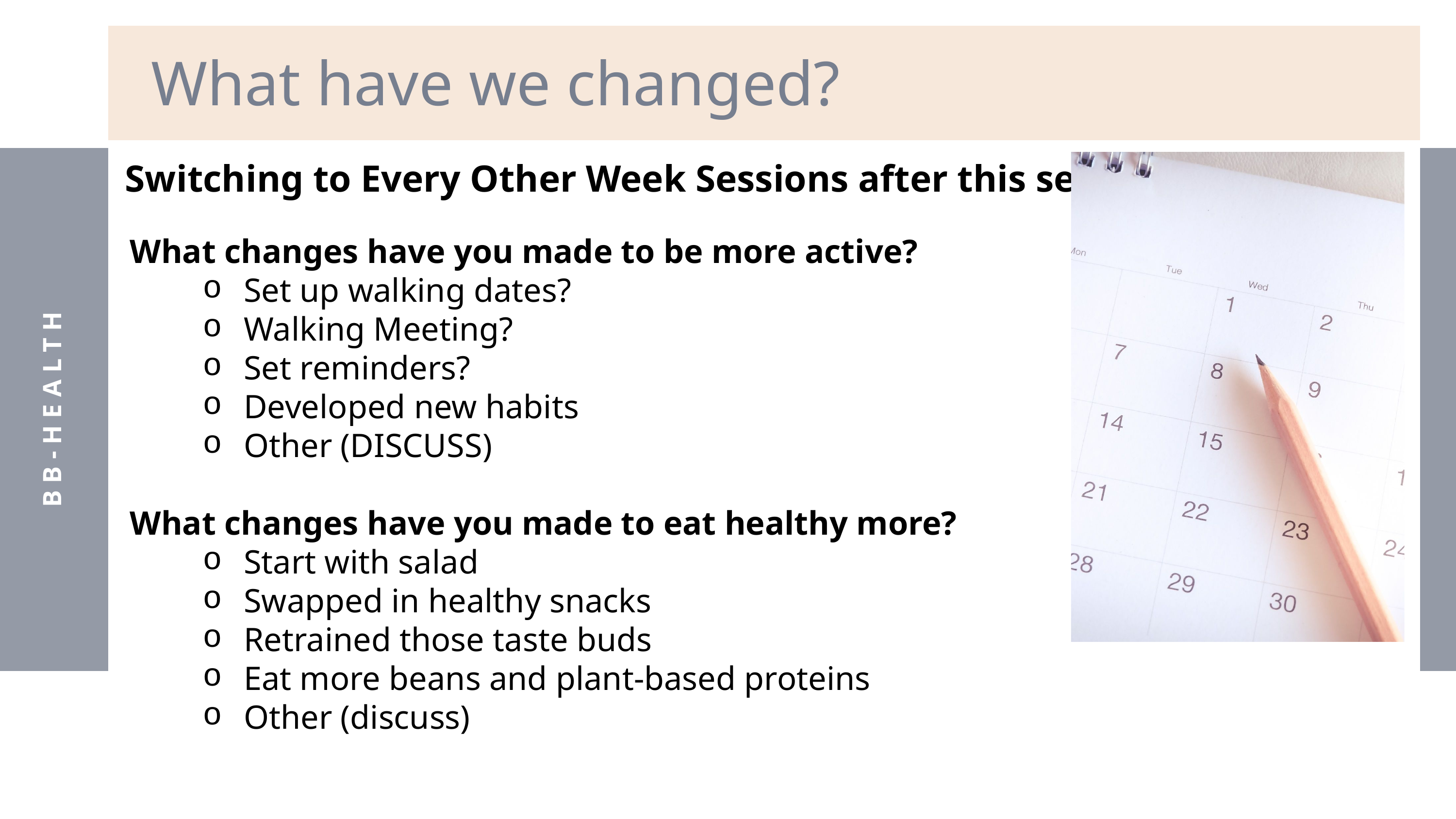

What have we changed?
Switching to Every Other Week Sessions after this session!
What changes have you made to be more active?
Set up walking dates?
Walking Meeting?
Set reminders?
Developed new habits
Other (DISCUSS)
What changes have you made to eat healthy more?
Start with salad
Swapped in healthy snacks
Retrained those taste buds
Eat more beans and plant-based proteins
Other (discuss)
BB-HEALTH

## Slide 11
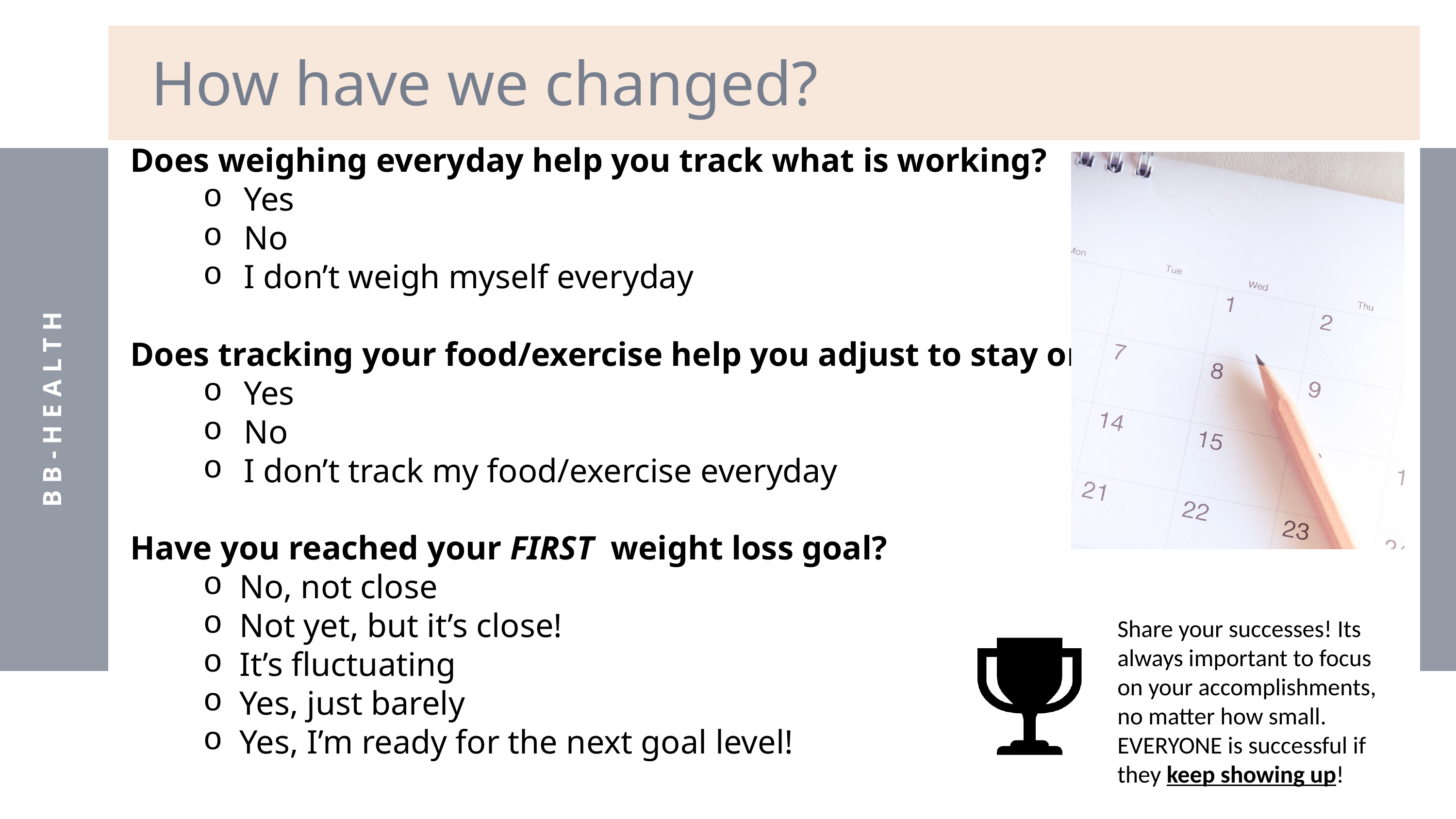

How have we changed?
Does weighing everyday help you track what is working?
Yes
No
I don’t weigh myself everyday
Does tracking your food/exercise help you adjust to stay on track?
Yes
No
I don’t track my food/exercise everyday
Have you reached your FIRST weight loss goal?
No, not close
Not yet, but it’s close!
It’s fluctuating
Yes, just barely
Yes, I’m ready for the next goal level!
BB-HEALTH
Share your successes! Its always important to focus on your accomplishments, no matter how small. EVERYONE is successful if they keep showing up!

## Slide 12
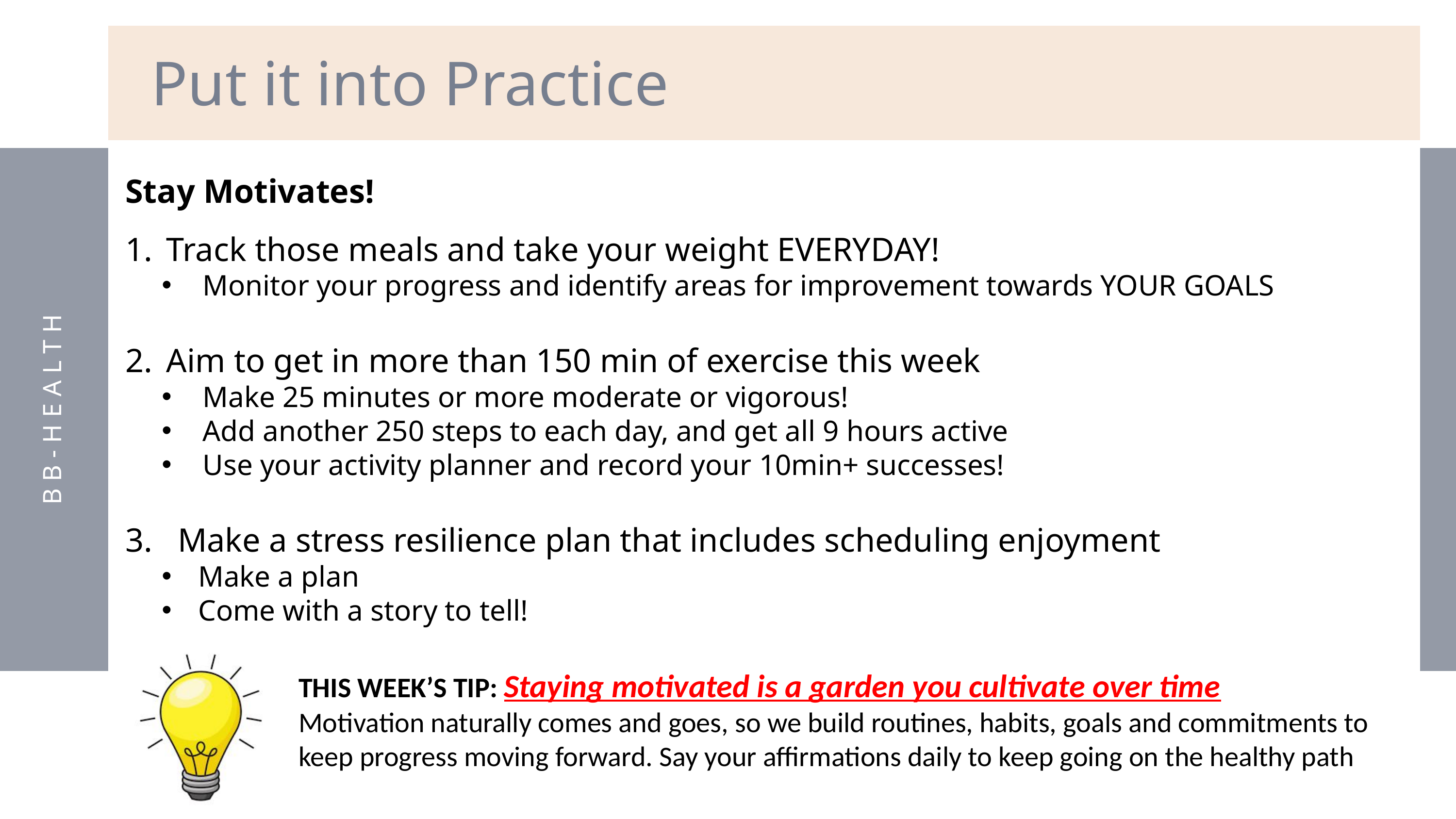

Put it into Practice
Stay Motivates!
Track those meals and take your weight EVERYDAY!
Monitor your progress and identify areas for improvement towards YOUR GOALS
Aim to get in more than 150 min of exercise this week
Make 25 minutes or more moderate or vigorous!
Add another 250 steps to each day, and get all 9 hours active
Use your activity planner and record your 10min+ successes!
3. Make a stress resilience plan that includes scheduling enjoyment
Make a plan
Come with a story to tell!
BB-HEALTH
THIS WEEK’S TIP: Staying motivated is a garden you cultivate over time
Motivation naturally comes and goes, so we build routines, habits, goals and commitments to keep progress moving forward. Say your affirmations daily to keep going on the healthy path

## Slide 13
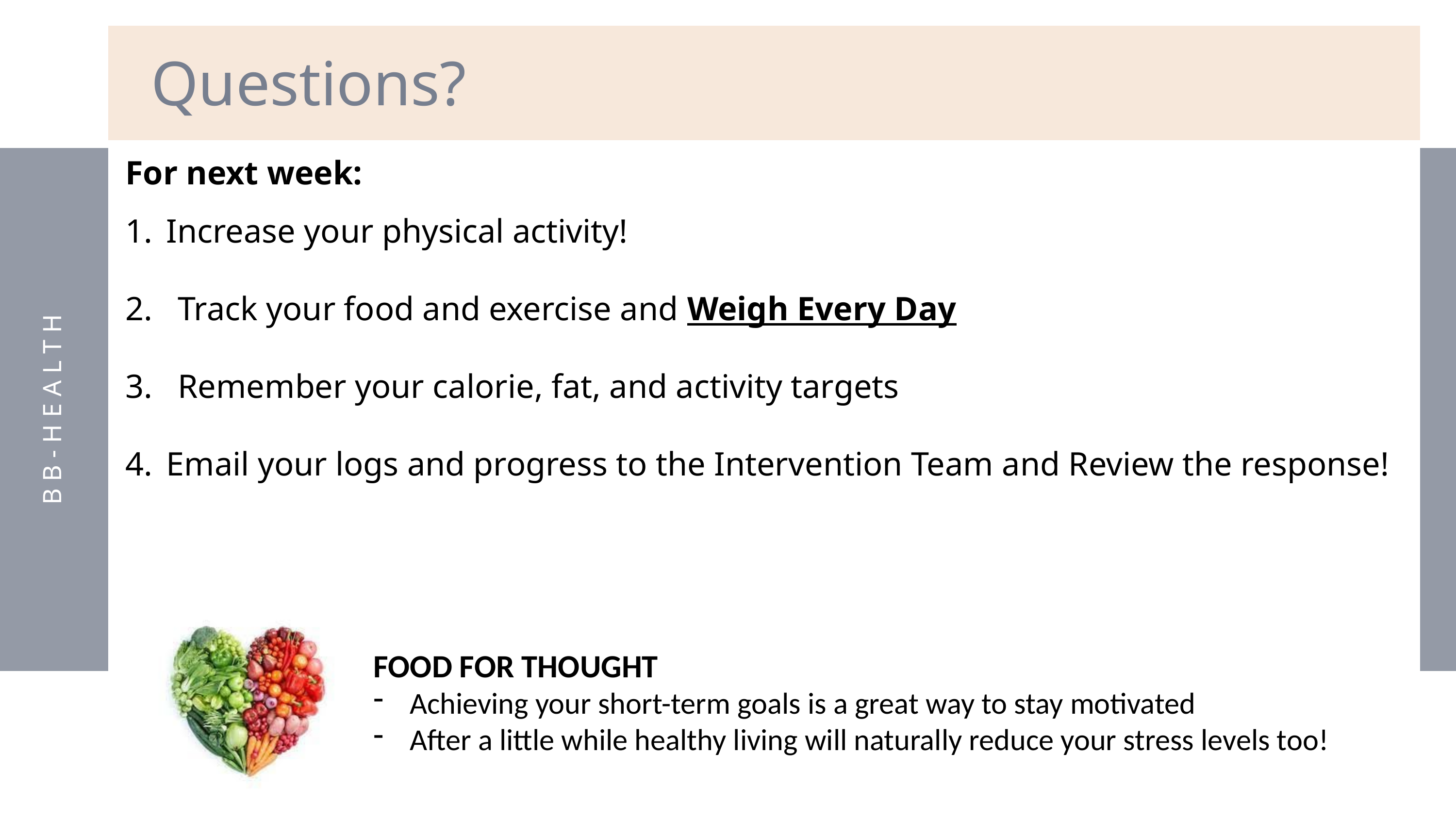

Questions?
For next week:
Increase your physical activity!
2. Track your food and exercise and Weigh Every Day
3. Remember your calorie, fat, and activity targets
Email your logs and progress to the Intervention Team and Review the response!
BB-HEALTH
FOOD FOR THOUGHT
Achieving your short-term goals is a great way to stay motivated
After a little while healthy living will naturally reduce your stress levels too!
