# Supplementary material for: Development and Feasibility of an eHealth Diabetes Prevention Program Adapted for Older Adults—Results from a Randomized Control Pilot Study
Source: Nutrients. 2024 Mar 23;16(7):930. doi: 10.3390/nu16070930 (PMC11154527; doi:10.3390/nu16070930)
Supplement: Supplementary file 1 [file nutrients-16-00930-s001.zip › Week12_SUPP.pdf]

BB-HEALTH

# e-Healthy Older Adults Modification

## Diabetes Prevention Program

**WEEK #12**
Stay Motivated!

# Group Opening Discussion

BB-HEALTH

WELCOME BACK! <u>HOW WAS LAST WEEK</u>?

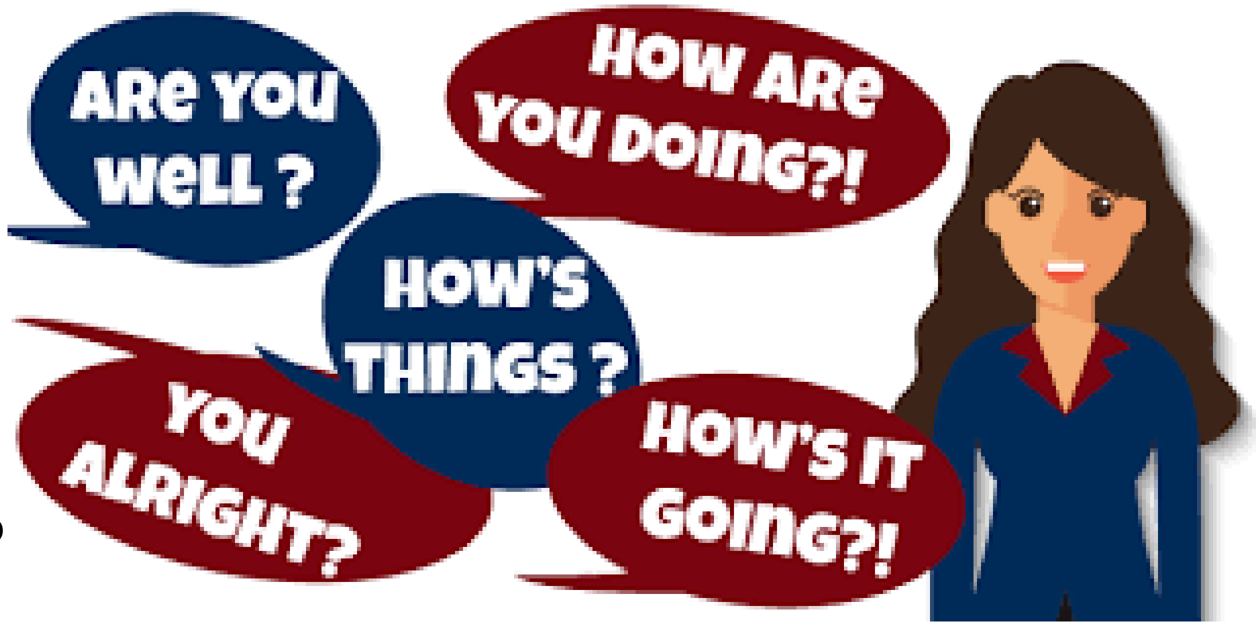


1. Did you track every meal?
2. Did you weigh every day, & record your weight today?
3. Did you manage those social cues?
4. Did you increase your daily steps?
5. Did you email the Intervention Team and review the response?
6. What were your unique challenges and successes?

**REMINDER: CHARGE YOUR FITBIT & SCALE DURING THIS MEETING!**

# Why do we try to prevent diabetes?

BB-HEALTH

<u>Risk factors for diabetes are similar for many chronic diseases– how do chronic illnesses impact us? (select all)</u>

- Medical costs go up
- Less time with loved ones
- More pain
- More medication
- Not able to do things you once enjoyed
- Don’t feel like your former self
- Tired all the time
- Worried a lot about the future or your health
- Other (discuss)

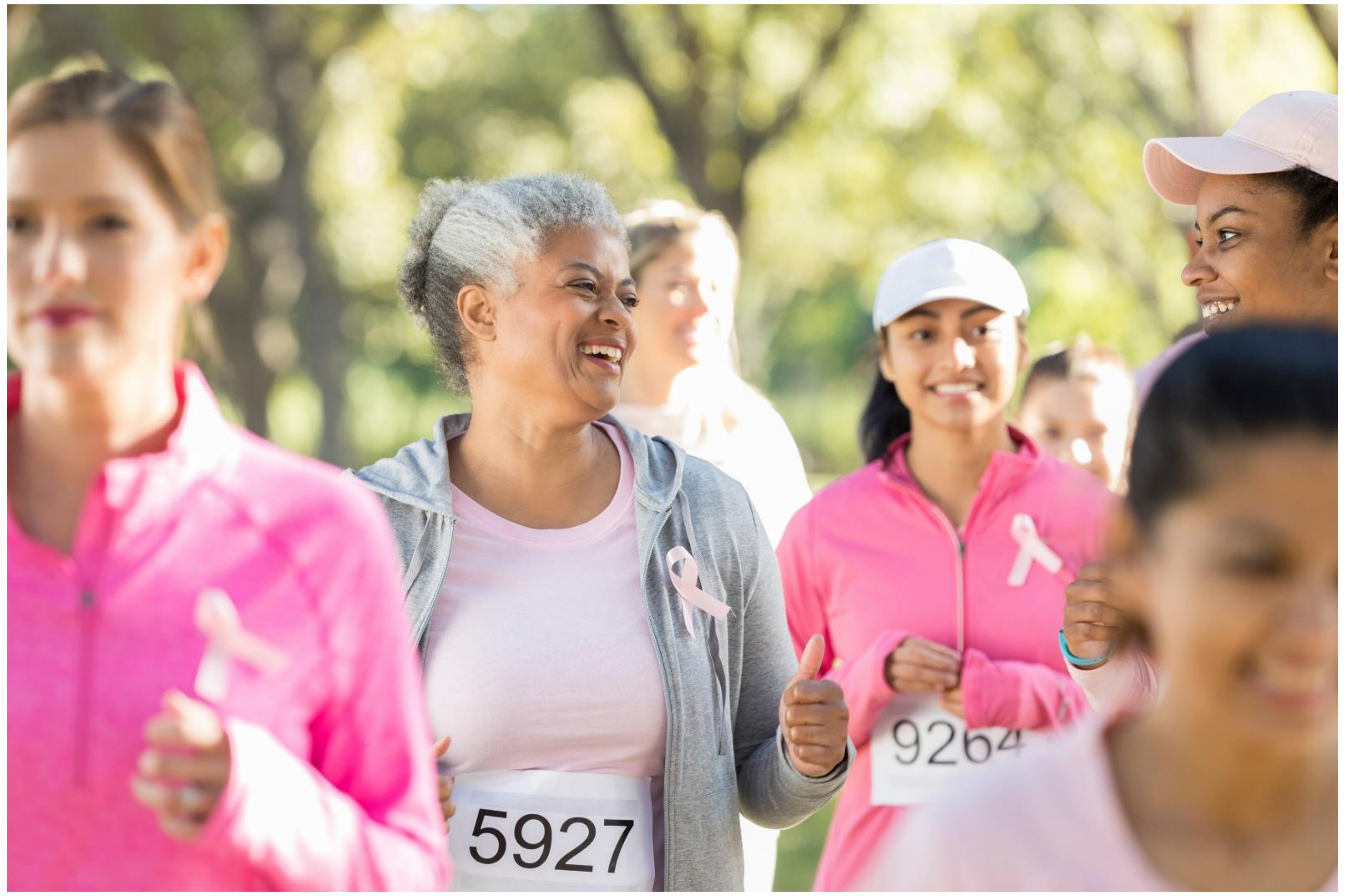

# How can we prevent diabetes?

BB-HEALTH

**<u>What are the ways we can help prevent diabetes and choose healthy aging? (select all)</u>**

- ○ Maintain a healthy weight
- ○ Eat lots of fruit, veggies, and whole grains
- ○ Limit saturated fat and added sugar
- ○ Sit less and move more
- ○ Get in some moderate and vigorous exercise weekly
- ○ Make social events active
- ○ Cook more at home
- ○ Other (discuss)

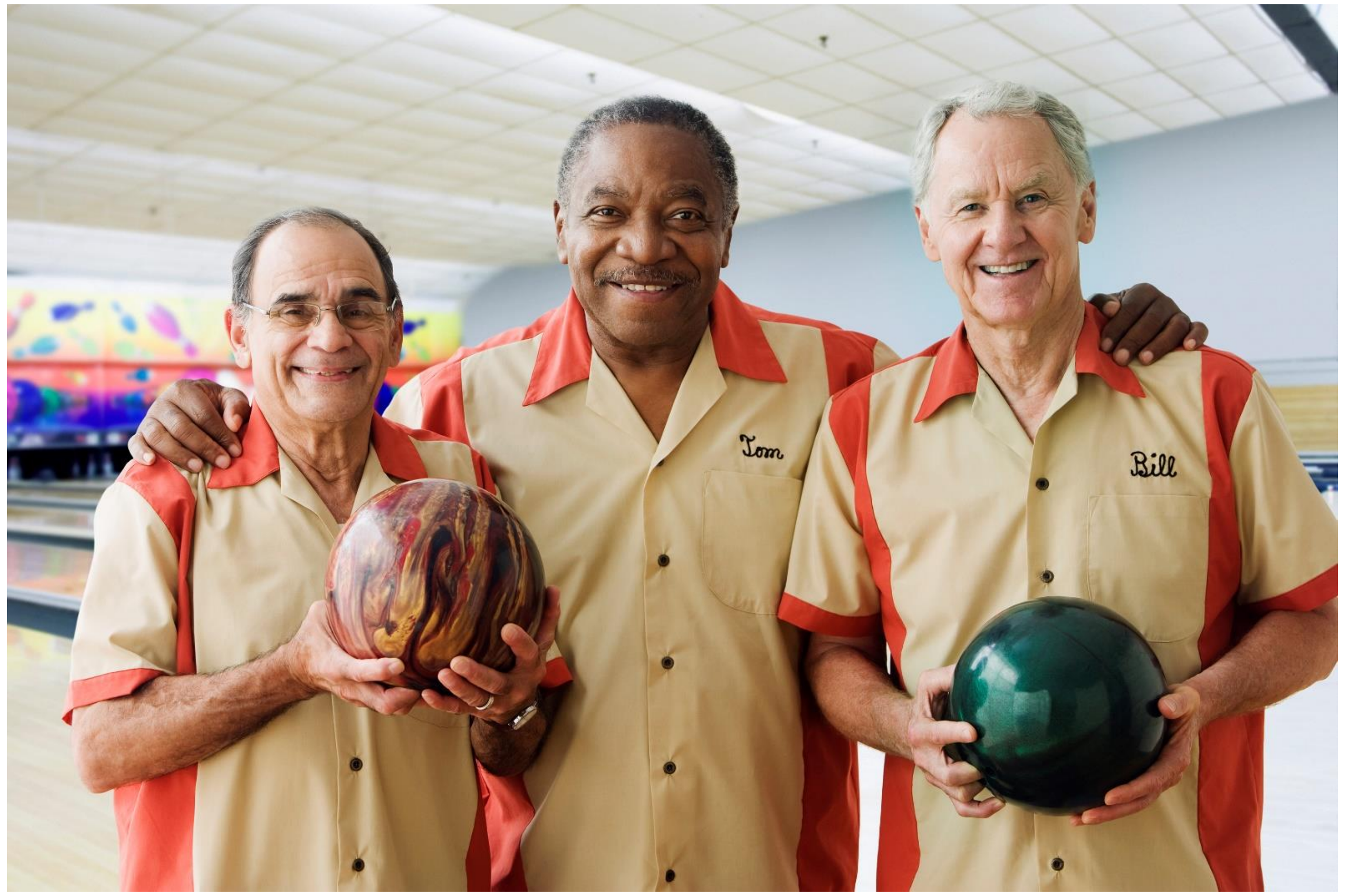

# Staying Motivated



## Health is a lifelong journey, not a destination

1. Keep your eye on what you hope to achieve
    - Honor your progress, but don't settle, keep going!
2. Recognize and celebrate your successes
    - What healthy changes are you proud of?
    - What was easy? What was hard?
3. Decorate your environment with signs of success
    - Make a milestone map, its satisfying to check off boxes!
4. Shake things up from time to time
    - Chase away boredom and plateaus with something new
5. Turn progress into a game or friendly competition!
    - Ask a friend or fellow group member – you both win!
6. Phone a friend

    Reach out to someone supportive for some encouragement

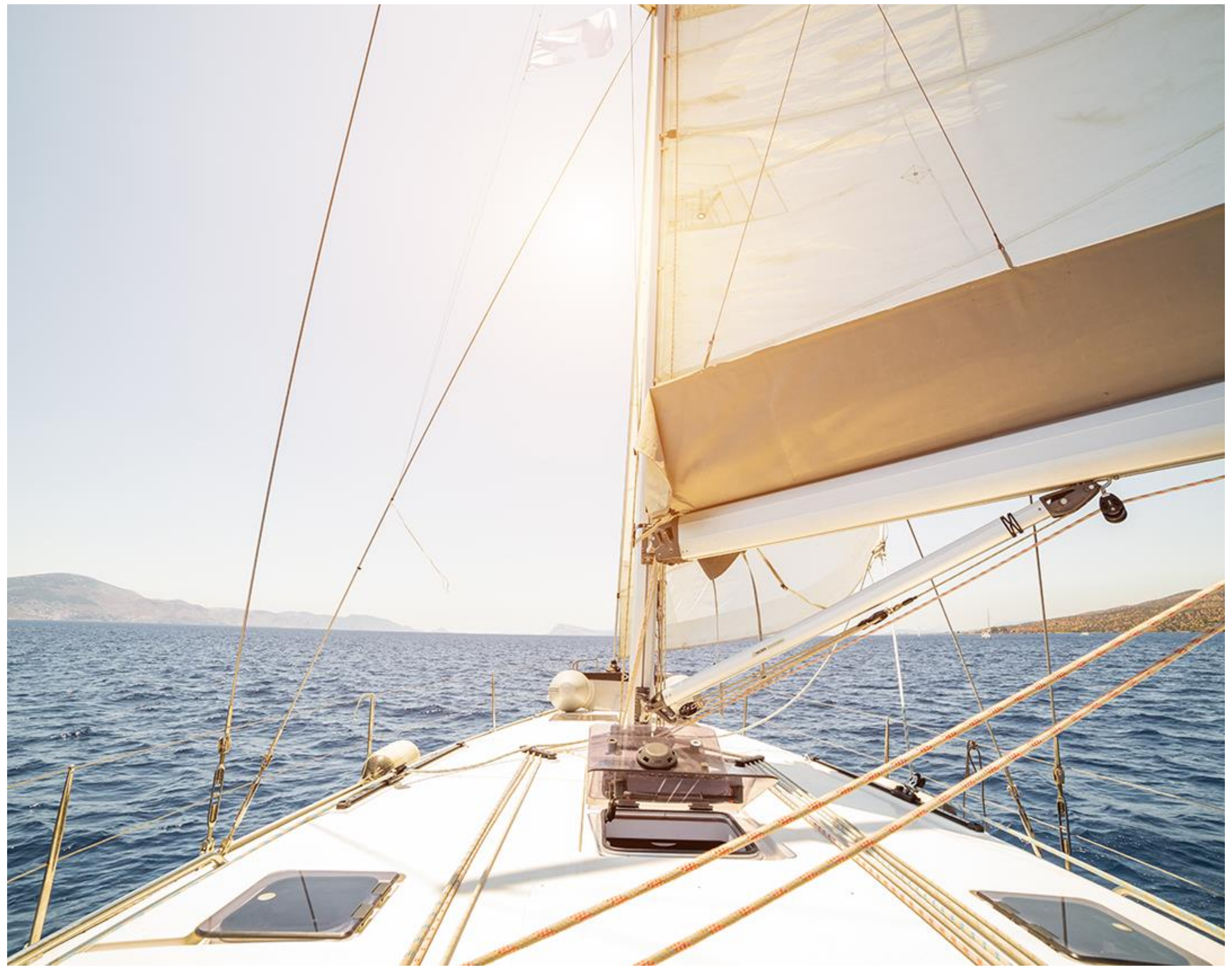

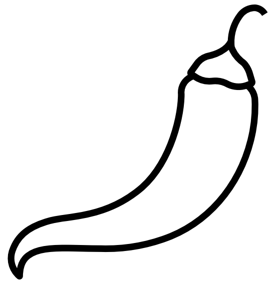

Spice things up! Change up your flavors with a Mexican or Indian night, schedule a Meatless Monday, subscribe to a recipe magazine, or sign up for a cooking class!

# Managing Stress

**Stress is an unavoidable part of life, but how we manage and recover from stressful situations is highly related to health and success reaching our goals.**

- Voluntary Reactions to Stress
  - Eating too much or eating enough
    → Both unhelpful!
  - Becoming withdrawn or sedentary
  - Taking risks, like drinking alcohol

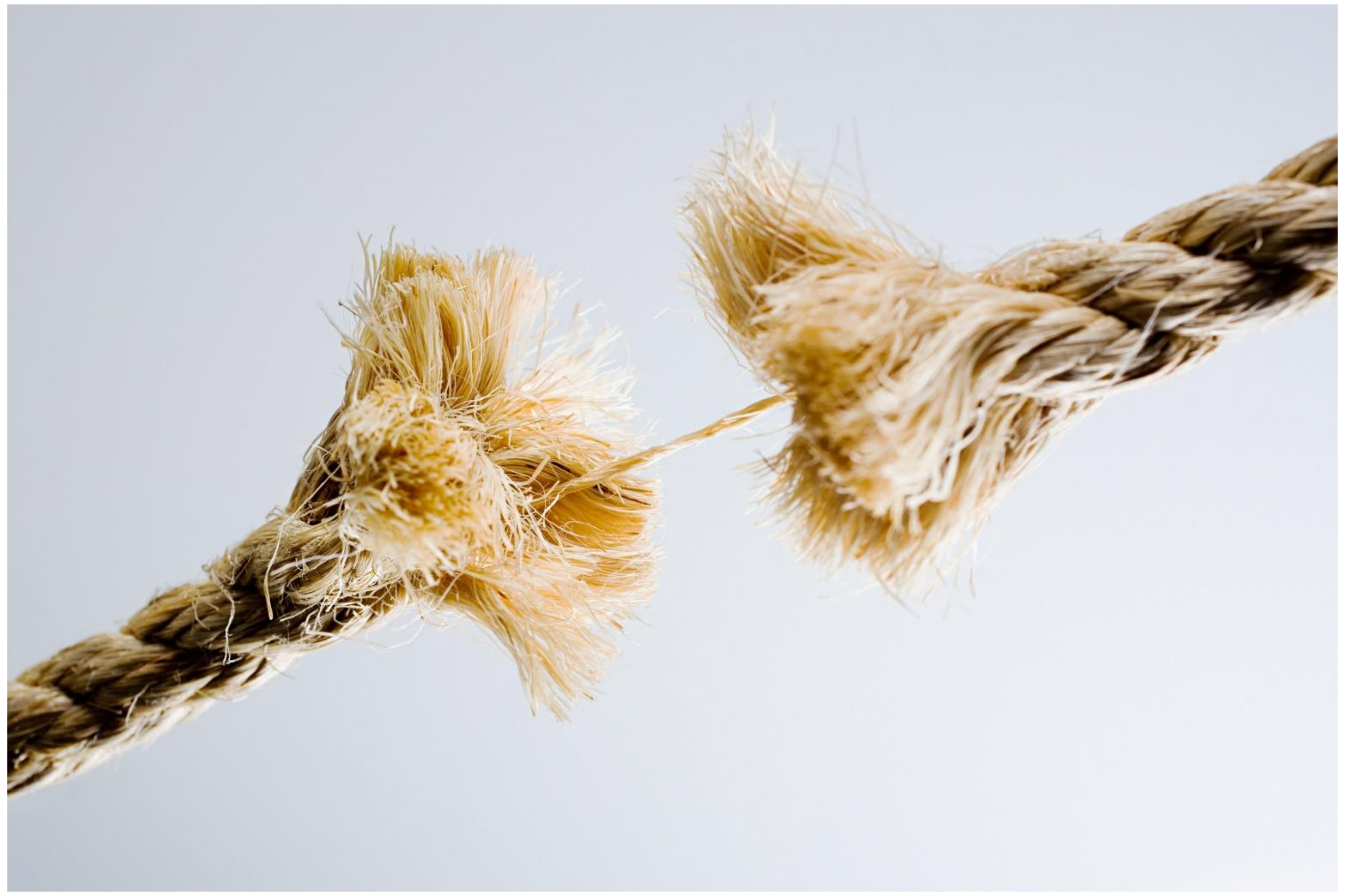

- Involuntary Reactions to Stress
  - Headaches, stomach aches, muscle aches

# Responding to Stress

B B - H E A L T H

- Choices in the face of stress
  - Say no, even for short-term stress to prevent long-term
  - Ask for help, get support when prioritizing your health
  - Set goals you can reach, and focus on the goals
  - Make a schedule with the real world in mind
  - Get organized, chaos creates problems
  - Plan ahead
  - Keep things in perspective, one day at a time
- When you can’t avoid the stress
  - Be aware that you are stressed, maintain control of your actions and behaviors
  - Recognize the signs and triggers
  - Take a time out, pamper yourself!
  - Get moving- exercise helps reduce feelings of stress
  - Breathe, mindful breathing reduces feelings of stress
  - Do some meal prep, chopping can be meditative!

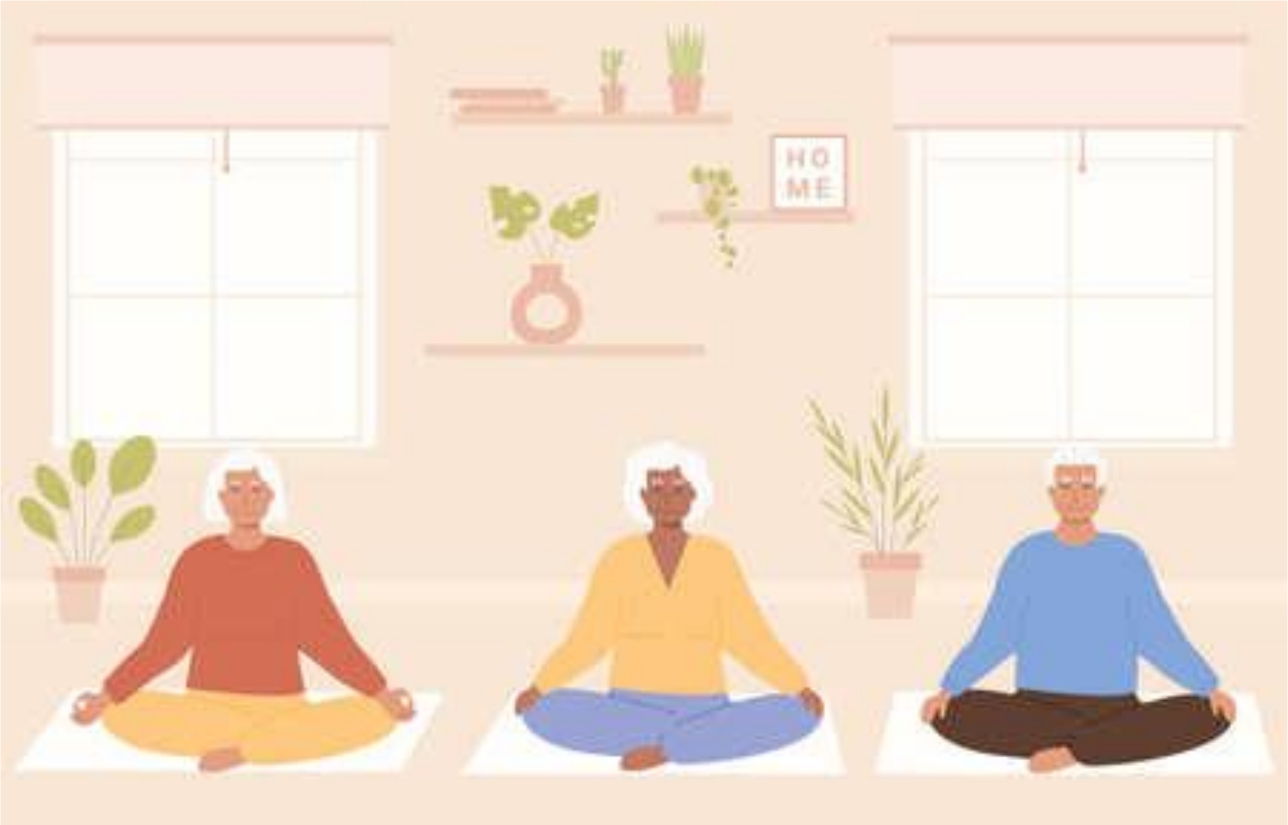

# Set New Goals to Buffer Yourself from Stress

B B - H E A L T H

- Short, achievable goals
  - Just enough of a challenge, but not too hard
    - "I won't eat potato chips this week"
    - "I will take an extra lap around my block today"

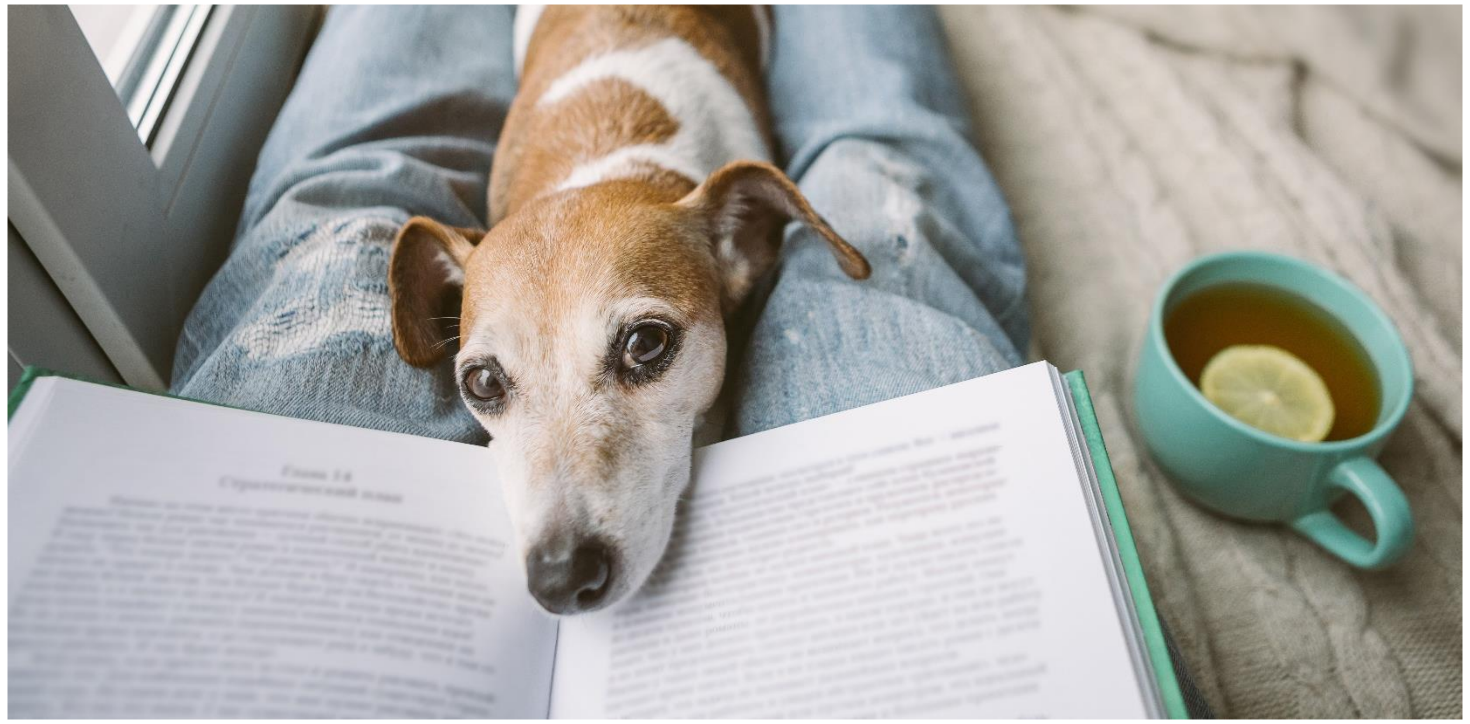

- Reward yourself when reaching goals
  - A nice reward that doesn't cost a lot but you value
    - A bubble bath
    - A chat with friends or family sharing good news
    - A book on tape, or curling up with a good book
  - Don't reward with food– this is key!

What are your favorite NON-FOOD related rewards?

Put them in the chat or share with the group!

# Setting Yourself Up for Success

BB-HEALTH

**Schedule**

## Schedule enjoyment in your life

- Make a list of things you enjoy
- Maintain a schedule so you can always look forward
- Protect your good experiences– honor your schedule!

**Reconnect and Affirm**

## Reconnect & Affirm your Goals

- Refer to your reasons for improving your health
- Add new reasons as they occur to you
- Repeat these to yourself as affirmations:

**Forgive**

## Be accountable – but FORGIVE yourself too

- Forgive, but don't forget, we learn from our slips!
- Self-monitor weight, food logs and exercise logs
- Constantly self-improve, but no one is perfect!
- Keep track of progress

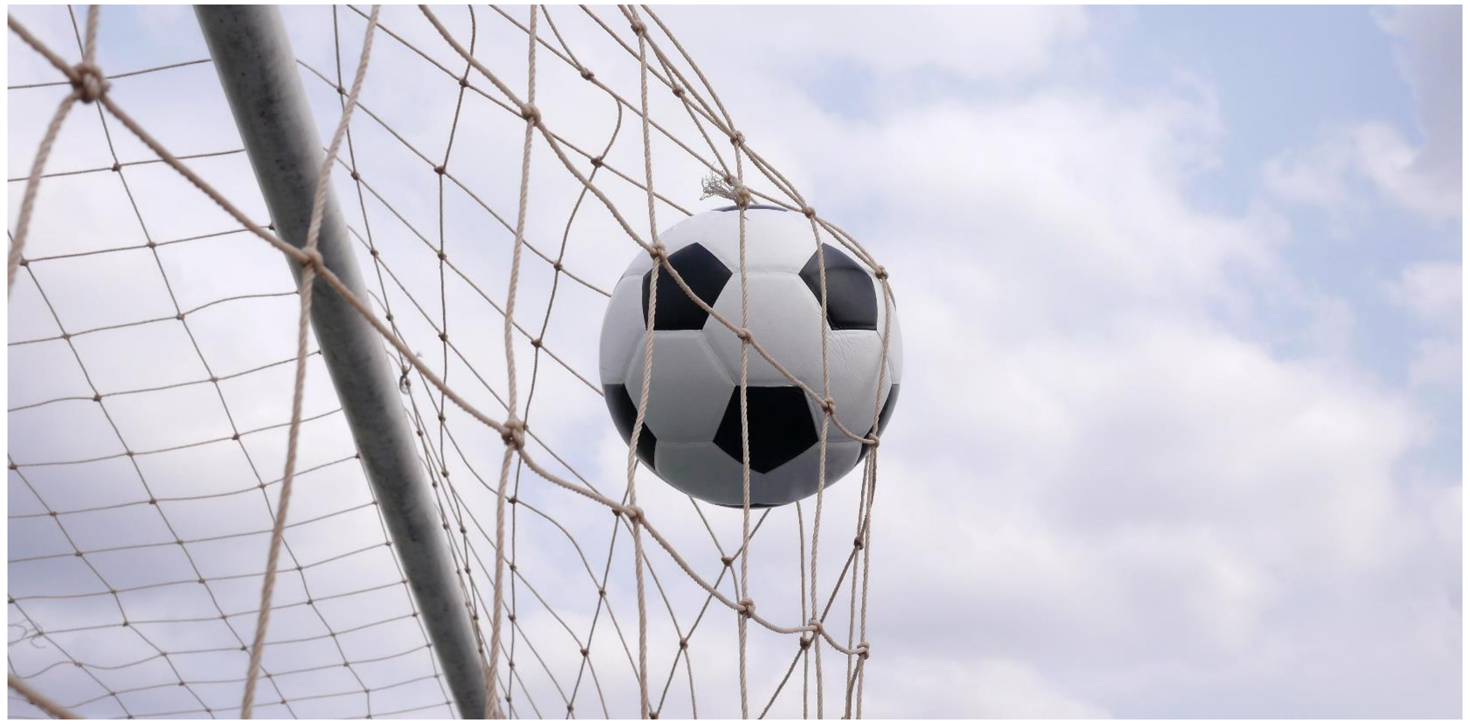

# What have we changed?



Switching to Every Other Week Sessions after this session!

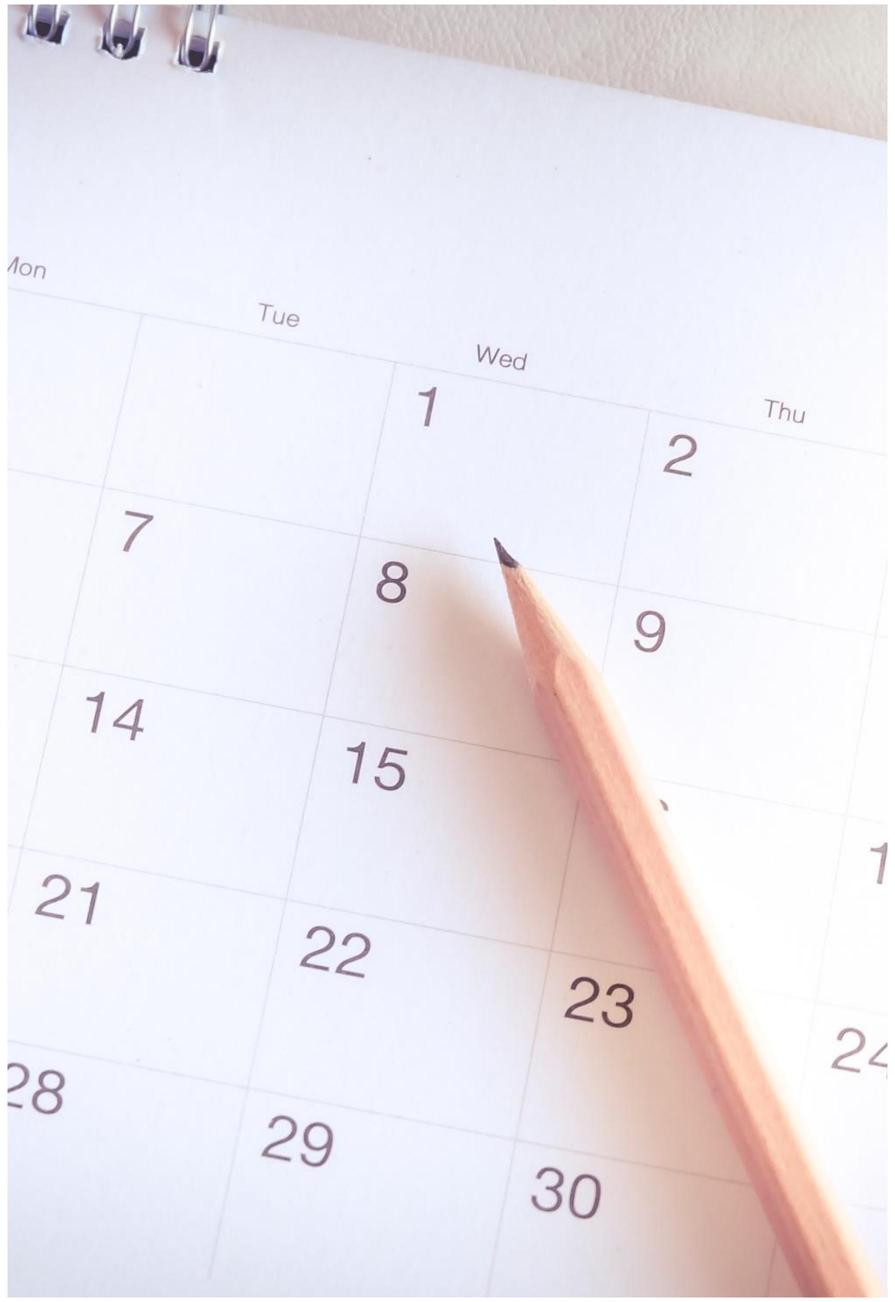


**What changes have you made to be more active?**

- Set up walking dates?
- Walking Meeting?
- Set reminders?
- Developed new habits
- Other (DISCUSS)

**What changes have you made to eat healthy more?**

- Start with salad
- Swapped in healthy snacks
- Retrained those taste buds
- Eat more beans and plant-based proteins
- Other (discuss)

# How have we changed?

BB-HEALTH

**Does weighing everyday help you track what is working?**

- Yes
- No
- I don’t weigh myself everyday

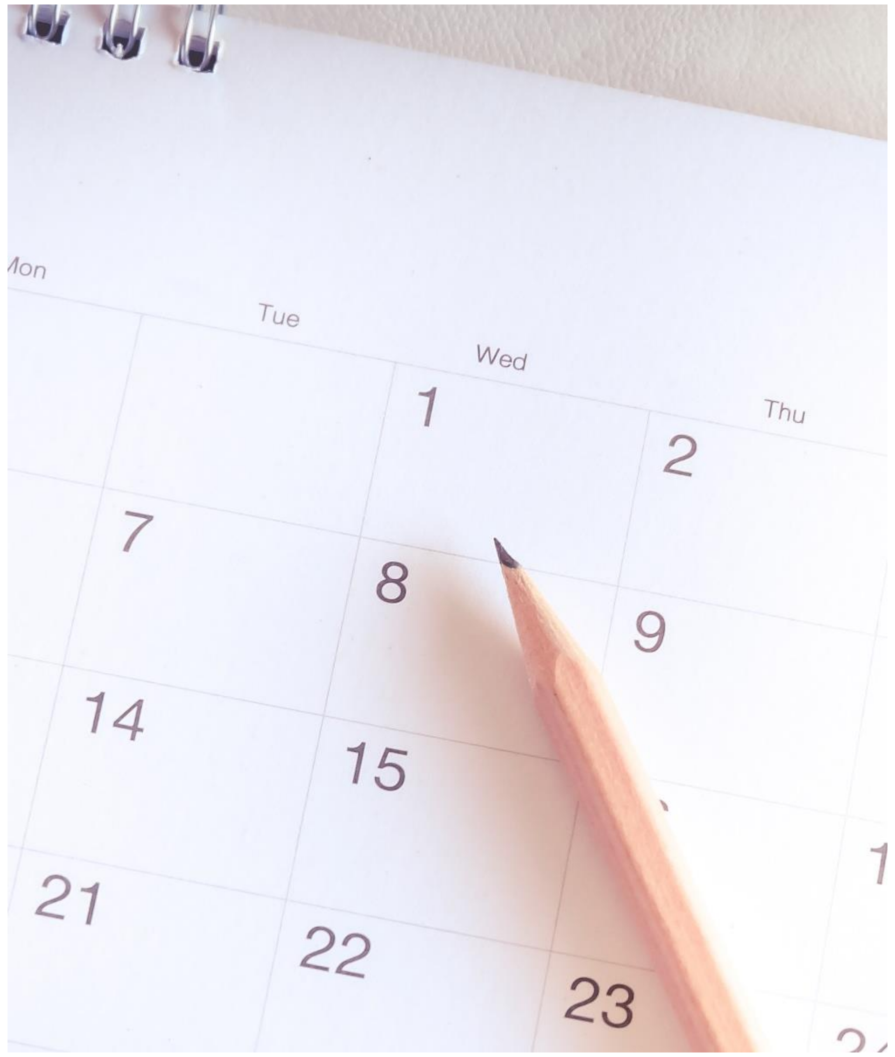


**Does tracking your food/exercise help you adjust to stay on track?**

- Yes
- No
- I don’t track my food/exercise everyday

**Have you reached your *FIRST* weight loss goal?**

- No, not close
- Not yet, but it’s close!
- It’s fluctuating
- Yes, just barely
- Yes, I’m ready for the next goal level!

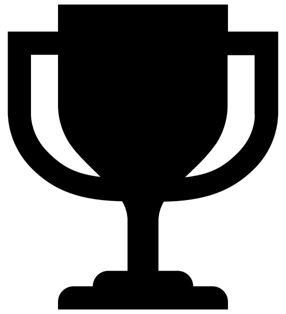

Share your successes! Its always important to focus on your accomplishments, no matter how small. EVERYONE is successful if they **<u>keep showing up</u>**!

# Put it into Practice

B B - H E A L T H

**Stay Motivates!**

1. Track those meals and take your weight EVERYDAY!
    - Monitor your progress and identify areas for improvement towards YOUR GOALS

2. Aim to get in more than 150 min of exercise this week
    - Make 25 minutes or more moderate or vigorous!
    - Add another 250 steps to each day, and get all 9 hours active
    - Use your activity planner and record your 10min+ successes!

3. Make a stress resilience plan that includes scheduling enjoyment
    - Make a plan
    - Come with a story to tell!

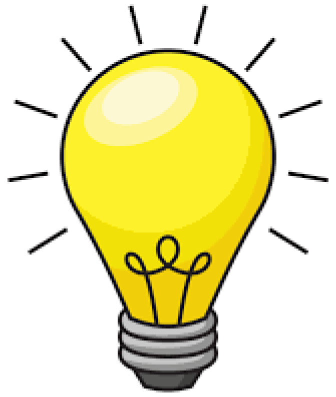

**THIS WEEK'S TIP:** ***<u>Staying motivated is a garden you cultivate over time</u>***
Motivation naturally comes and goes, so we build routines, habits, goals and commitments to keep progress moving forward. Say your affirmations daily to keep going on the healthy path

# Questions?

B B - H E A L T H

**For next week:**

1. Increase your physical activity!

2. Track your food and exercise and **<u>Weigh Every Day</u>**

3. Remember your calorie, fat, and activity targets

4. Email your logs and progress to the Intervention Team and Review the response!

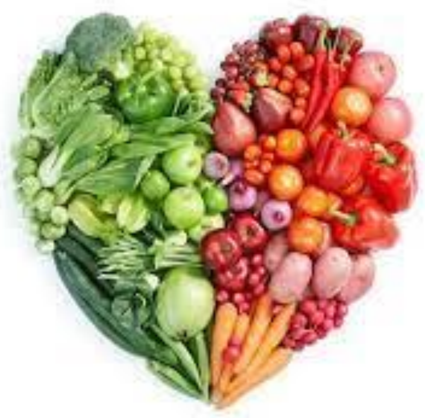

**FOOD FOR THOUGHT**

- Achieving your short-term goals is a great way to stay motivated
- After a little while healthy living will naturally reduce your stress levels too!
