# Supplementary material for: Development and Feasibility of an eHealth Diabetes Prevention Program Adapted for Older Adults—Results from a Randomized Control Pilot Study
Source: Nutrients. 2024 Mar 23;16(7):930. doi: 10.3390/nu16070930 (PMC11154527; doi:10.3390/nu16070930)
Supplement: Supplementary file 1 [file nutrients-16-00930-s001.zip › Week1_SUPP.pdf]

BB-HEALTH

e-Healthy Older Adults Modification

Diabetes Prevention Program

WEEK #1
WELCOME!

Our weekly meetings

BB-HEALTH

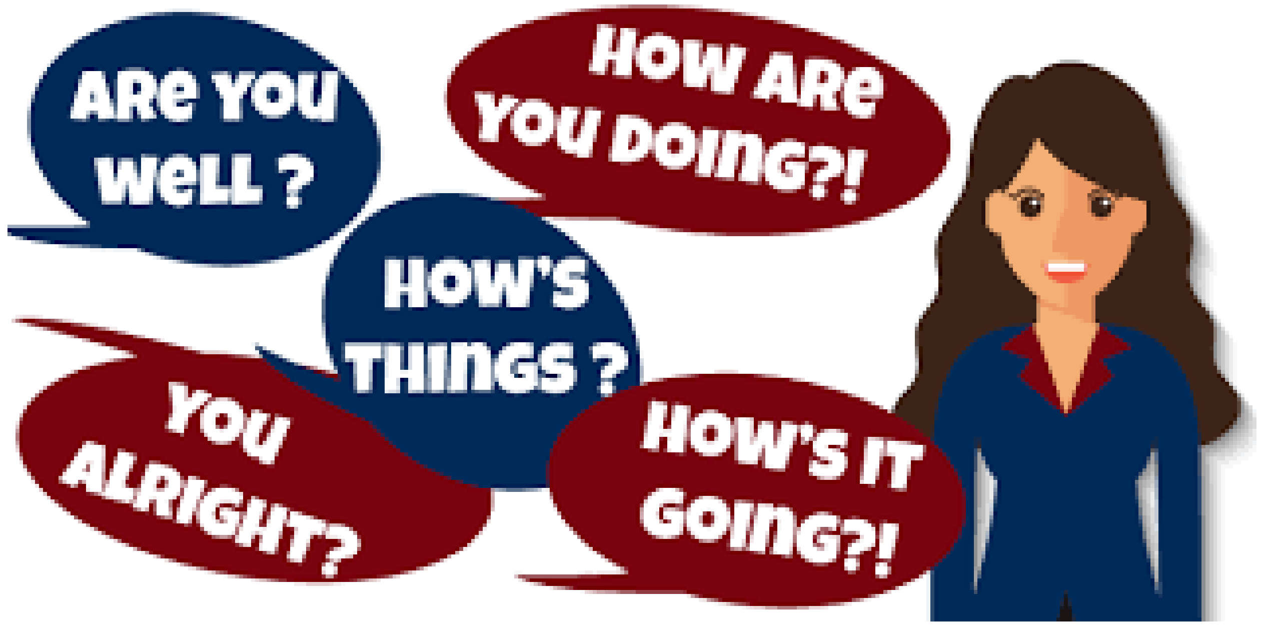

WELCOME!

1. Starts with going around the room, reconnecting and summarizing *QUICKLY* how the last week has been successful, or challenging, for your weight loss goals.

2. An educational session where new tools and techniques are introduced, including fun & delicious recipes you can follow during the week to help you achieve your goals.

3. A question and answer session to make sure you are set up for success, and so you can hear from your fellow group members and provide mutual support.

REMINDER: CHARGE YOUR FITBIT & SCALE DURING THIS MEETING!

Each week on your own

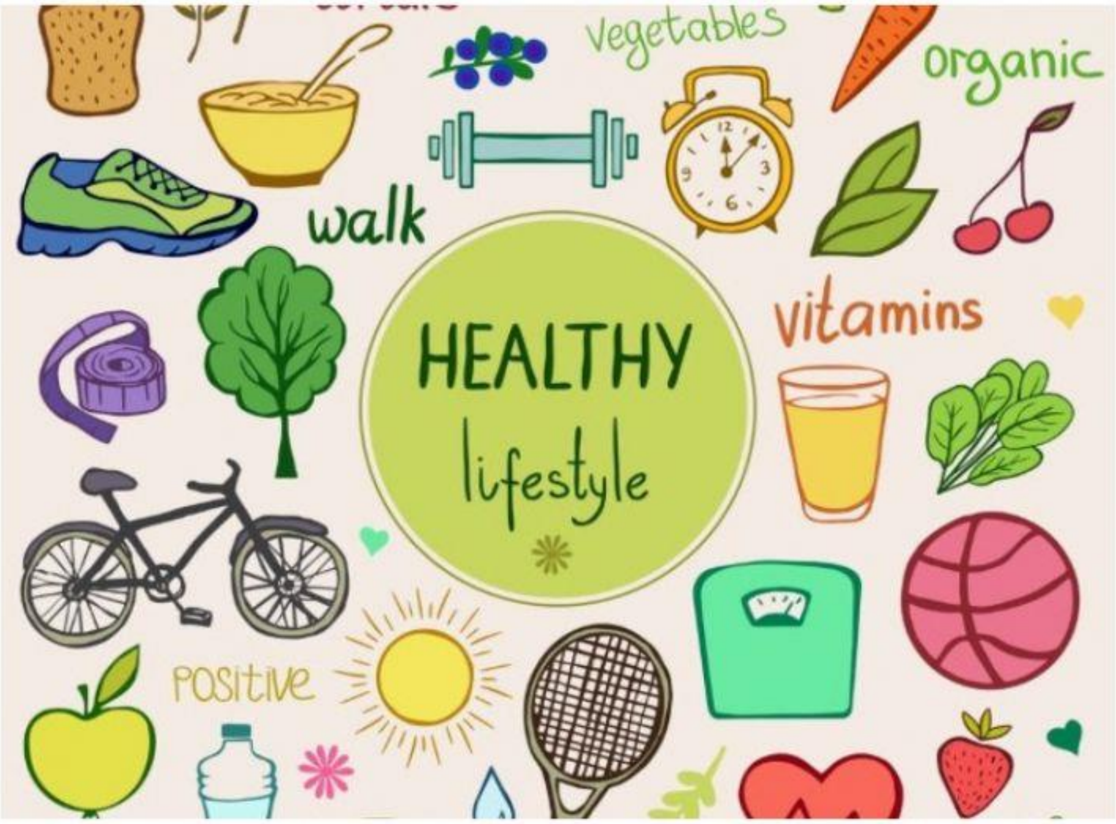

EVERYDAY:

1. Log your food in the notebook
2. Weigh yourself with the BB-Health Scale
3. Wear your Fitbit (keep it charged and connected)

EVERY WEEK:

1. Come to the group meeting to connect, learn, and ask questions
2. Email the Intervention team your food records
3. Review the personalized email response to your progress and food records
4. Get in your steps and exercise
5. Charge your health devices

How to be a good group member

B B - H E A L T H

- Come to every meeting, or reach out for a make-up session.
  - We are all in this together! We will help each other get through tough times and celebrate successes together. No one will get stuck or left behind in this program!
- Be on time
  - Sessions are short and pack a lot in, so every minute counts!
- Use the new skills from meetings during the week at home
  - Try a little bit more each day, and each week, it is a process!
- Bring your notebook and food log to every meeting
  - Take notes and log your food, that way you know what to work on at home!
- Share your ideas with the group
  - What works for you might help someone else, & someone else might be able to help with what you struggle with!
- Make this a safe place to share- privacy, positivity, and respect
  - Don’t repeat what you hear in here, and never put anyone down for what they say

What motivates you?

Why did you sign up for BB-Health?

- To lose weight
- To live longer
- To look better
- To have better quality of life
- Improve cognitive function
- To prevent diabetes
- To set a good example
- All the above
- Other (discuss)

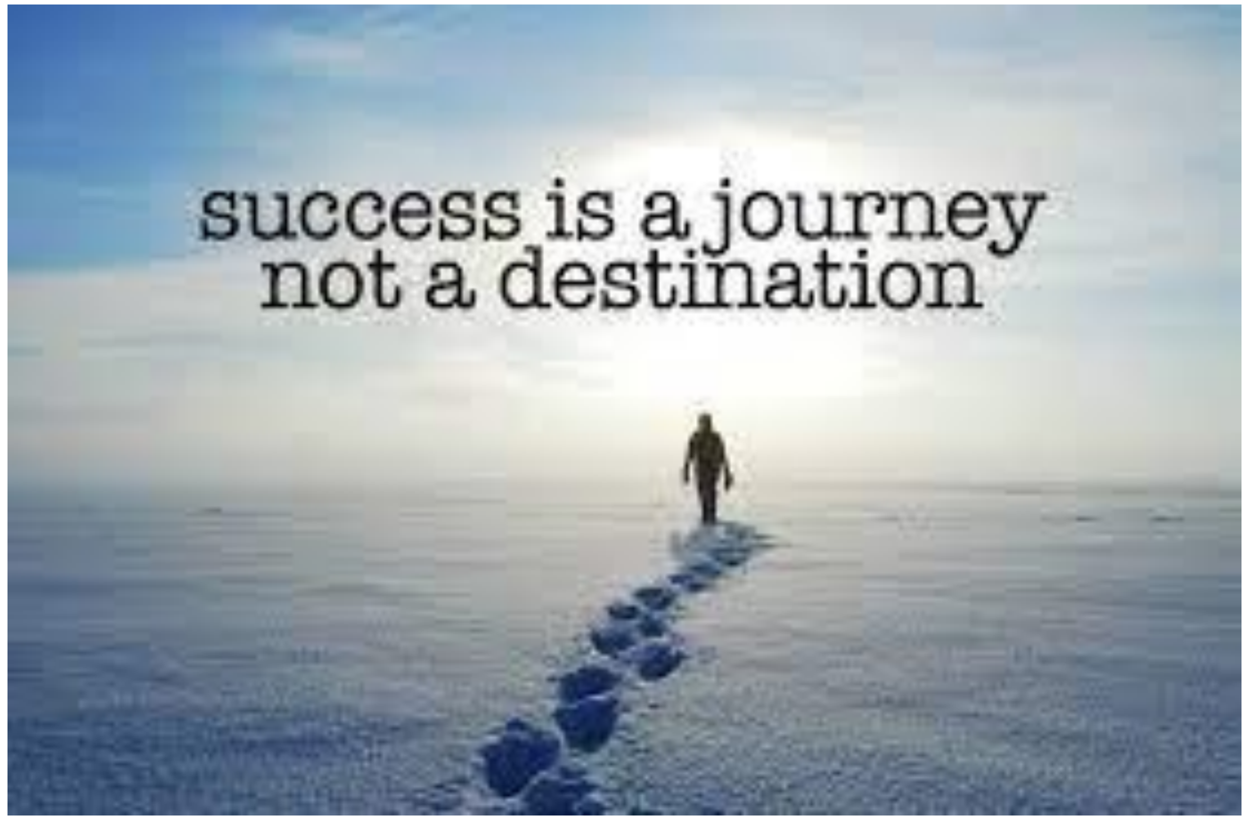

Previous Attempts

Have you tried to lose weight before?

- Never
- Once before, unsuccessfully
- Once before, successfully
- A few times before, unsuccessfully
- A few times before, successfully
- Many times before, unsuccessfully
- Many times before, successfully
- Other (discuss)

What does success mean? (select all)

- Early weight loss
- Keeping the weight off
- Feeling better
- Having more tools to reach your goals
- Other (discuss)

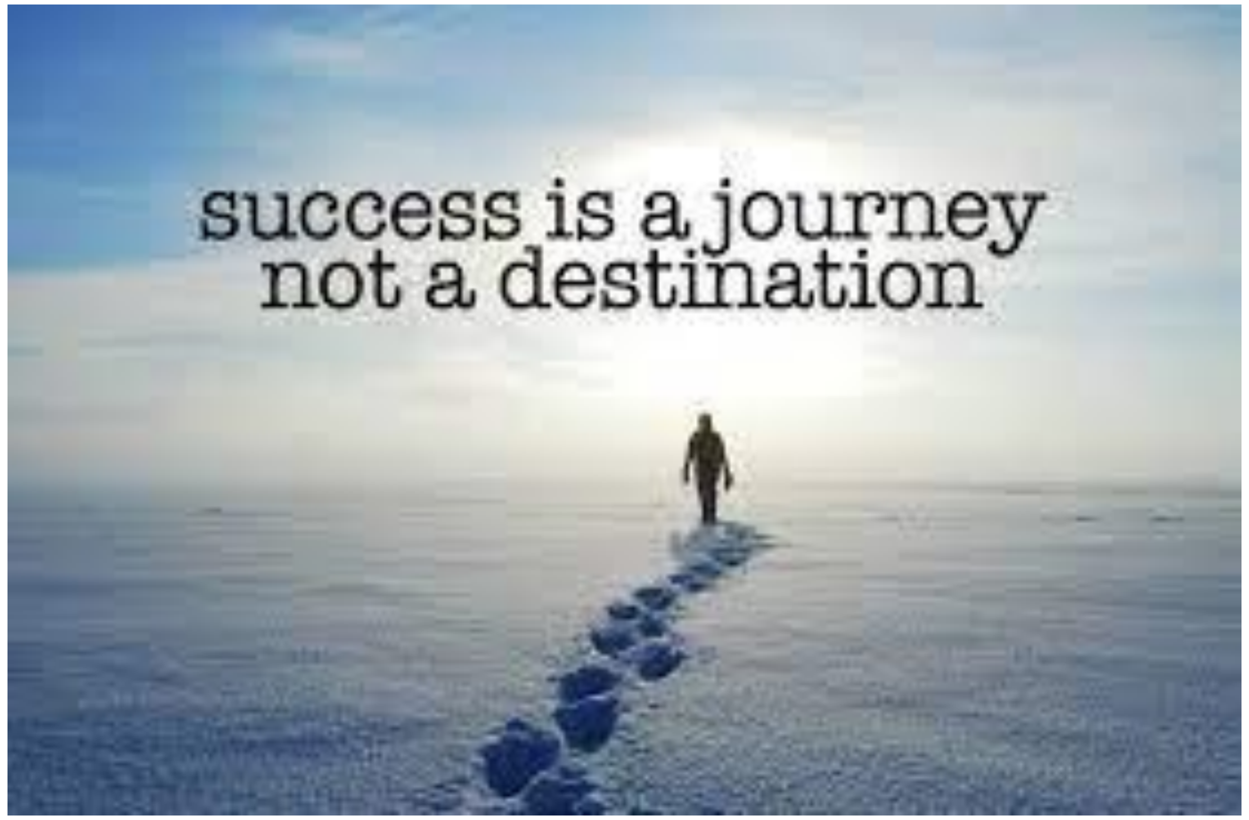

What are you ready to change?

What one small thing in my lifestyle do I know I'm ready to change to improve health?

- Cut down on sweets
- Sit around less (ie. move more!)
- Eat more whole fruit and vegetables
- Switch to whole grains
- Eat less processed food
- Cook more meals at home
- Open to change: learn new ways to be healthy
- All the above
- Other (discuss)

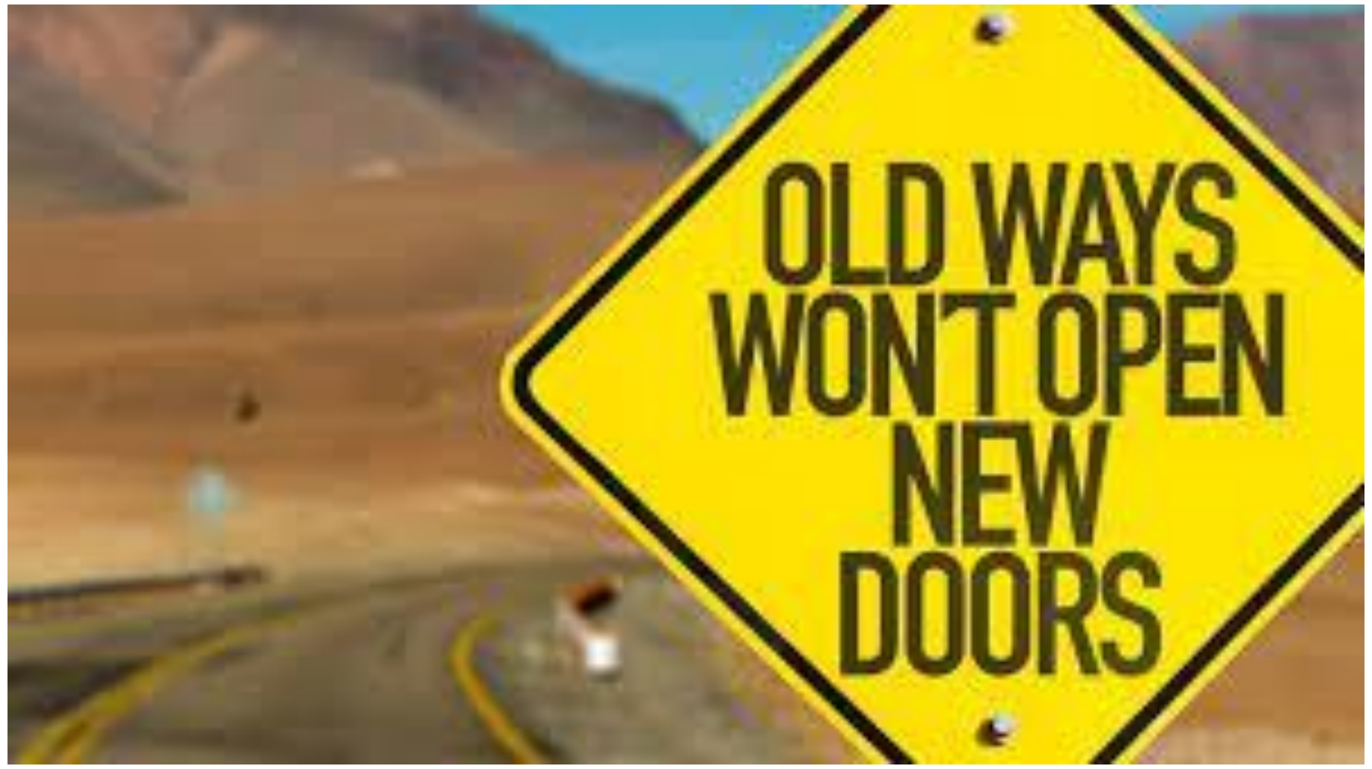

What is the DPP?

BB-HEALTH

- A proven and successful lifestyle program to lose weight, cutting the chances of developing diabetes by 58%, and over 71% in people over 60. That's compared to 31% protection from the leading medication (metformin)

- Not just diabetes, DPP reduced the chances of Metabolic Syndrome by 41%
  - Which means reducing your chance of heart attack, stroke, and kidney disease
    - These are called "diseases of aging", ***but we can prove them WRONG!***

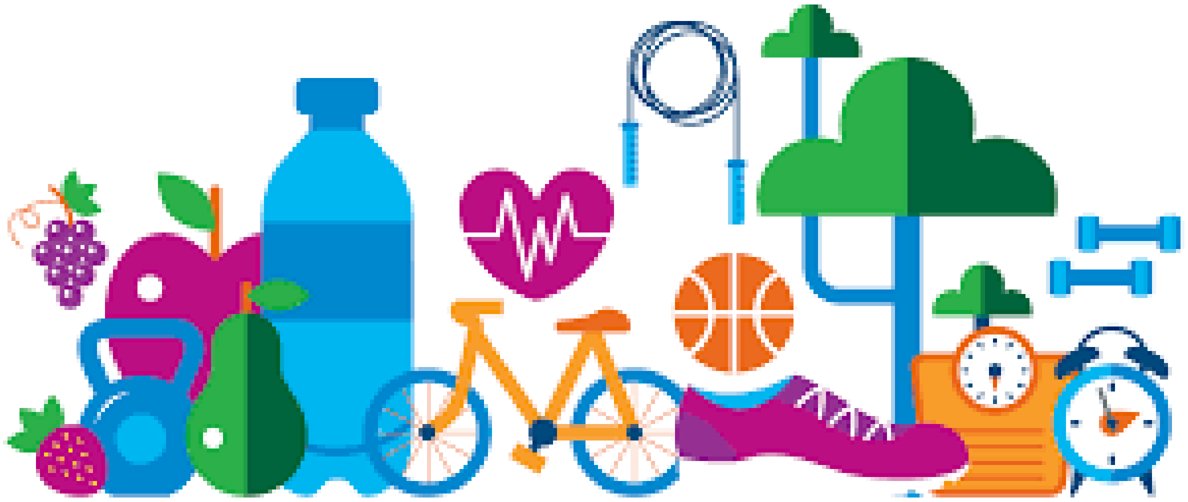

FULL RECOGNITION BY THE CDC TO PREVENT OR DELAY TYPE 2 DIABETES

How does it work?

GOAL 1: LOSE WEIGHT

- Lose 5-7% of your current body weight
- Lose weight slowly over time, with a healthy lifestyle you can maintain
- Aim to lose 1-2 pounds per week
- Write your weight today on the top of your weekly record

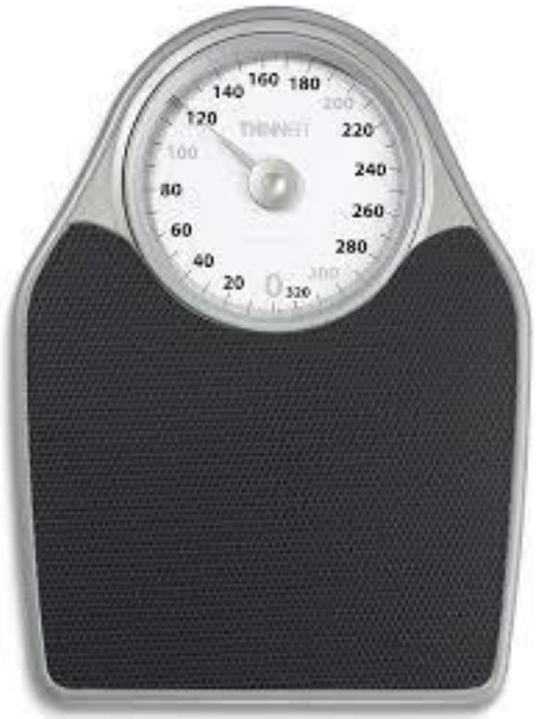

GOAL 2: GET ACTIVE

- Do at least 100 minutes of physical activity each week*
    - Start with 6,000 steps a day, 2,000 of which at a brisk pace
- Schedule brisk walks with friends or group members
- Spread the activity out: 20-30 minutes each day
- Build up to being more active over time

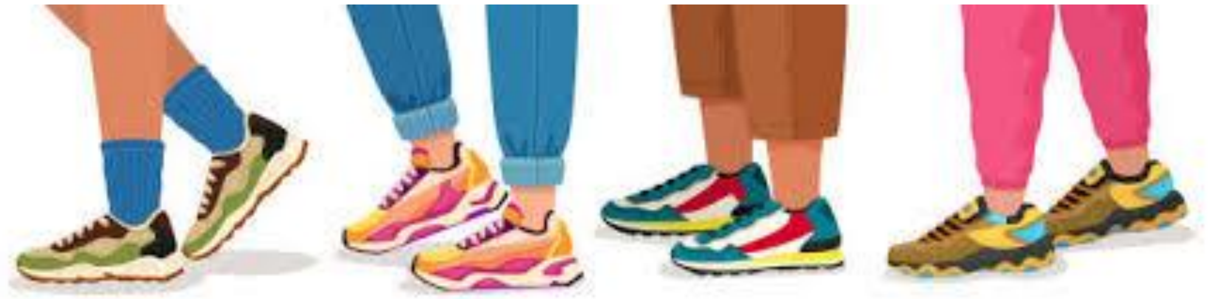

Remember: Losing weight early in a healthy lifestyle program is associated with the best outcomes, so if you can be strict now and learn new healthy habits <u>it can help you achieve your goals.</u>

BENEFITS

Be healthier, look better, & feel better

- Reduce stress
- Improve sleep
- Increase energy
- Lower blood pressure
- Help your cholesterol levels

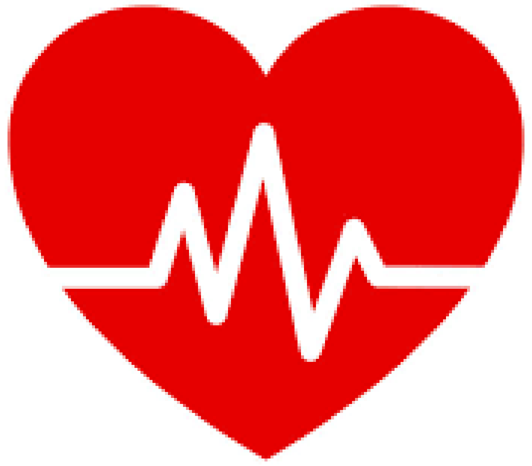

You can make a difference

- For yourself
- For your TEAM of fellow group members
- AND YOUR FAMILY, FRIENDS, and COMMUNITY!

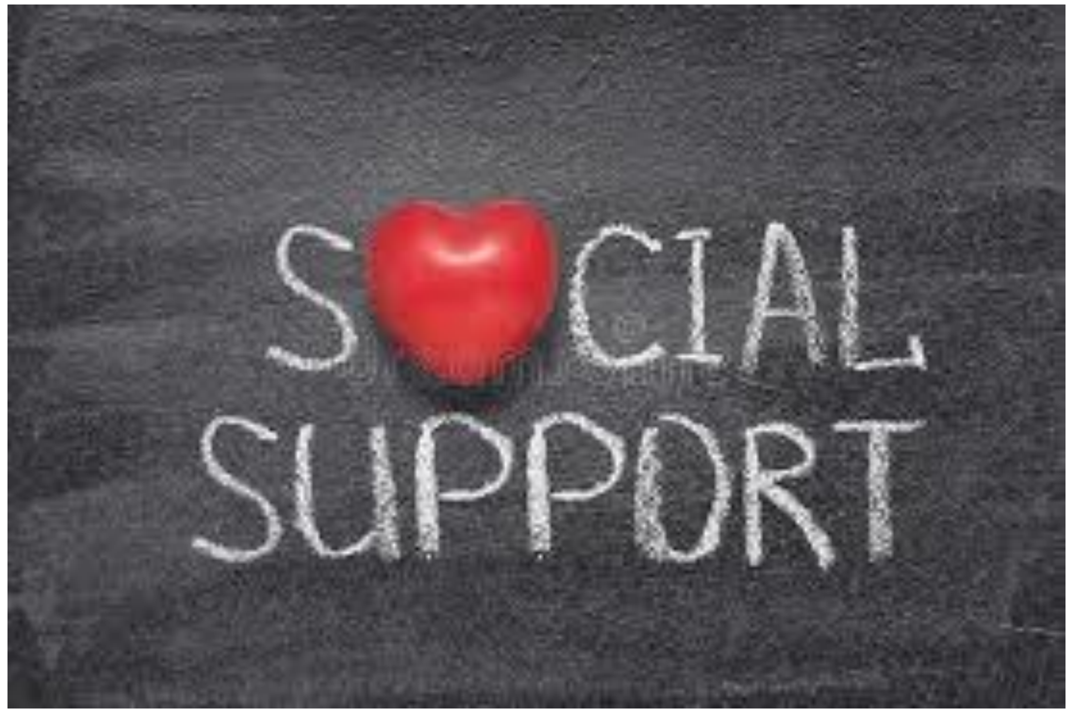

What will you learn?

Facts about health

- Increased **<u>awareness</u>** of how to make healthy eating choices and how to get the most out of physical activity

How to identify obstacles to your health (and how to address them!)

- Setting yourself up for success at home
- How to navigate eating away from home
- Use positive thoughts to displace self-defeating thoughts

How to make a healthy change, and *<u>make it stick</u>*

- How to get back up with you slip off course
- How to handle stress, social events, and get the support you need

Get Started Losing Weight

Weigh Yourself!

- Everyday, same time of day (e.g. wake up, go to the bathroom, & weigh everyday)
- Write your weight down on the day of meetings your weekly record

Bring AWARENESS to what you eat and drink!

- Be open to change so you can find a healthy eating pattern that works for you
- Yes, it means eating fewer calories
- BUT you can find a path to success tailored for you.

Keep track of what you eat, how much you exercise, and how you feel

- Give yourself a few weeks to adjust-- You will *actually* learn to LOVE healthy food!
- Be honest– there is no judgment here!
- Be accurate- you benefit more from knowing the truth
- Be complete- include everything so you know what works and what doesn't
- Hang in there- change is hard but if you stay willing we can help each other!

Practice

Keeping Track of What You Eat

1. Time of day for the things you ate (including snacks!)

2. The amount– start easy by preplanning meals for a few days– then repeat!

3. Be specific
   - Instead of chili write “vegetarian 3-bean chili”
   - Instead of milk write “non-fat milk”

4. Include nutrient info
   - When known, add the calories and grams of fat

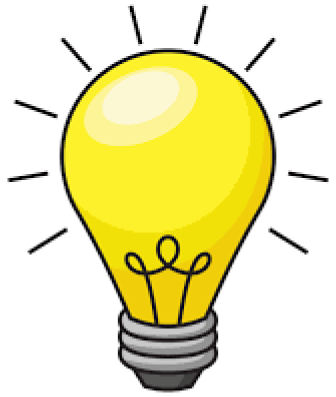

THIS WEEK’S TIP: *PORTION SIZES MATTER!*
Even a small difference in portion size can make a big difference, and small differences add up over time!
----Measure or weigh all your food and drinks so you can **be AWARE & in CONTROL**

Questions?

B B - H E A L T H

For next week:

1. Track your food, and start measuring it!

2. Weigh every day, record your weight the day of the next meeting!

3. Try to walk 100 min before the next meeting!

4. Start logging your food and check your Fitbit app for progress on weight!
   --Email the team with your progress, and reach out for a private meeting if needed

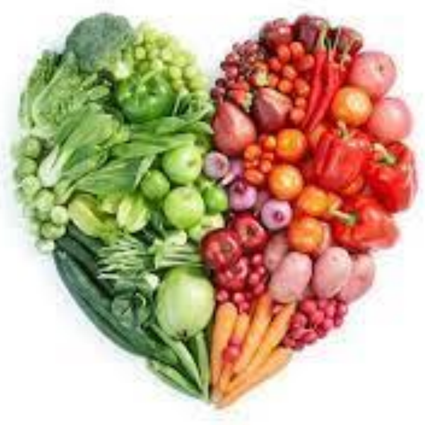

FOOD FOR THOUGHT

- Everyone's weight fluctuates, this happens on the path to success!
- Success comes at different speeds, but start strict for more EARLY RESULTS
- Plan for success, set up for first few days and then repeat that plan!
