# Supplementary material for: Development and Feasibility of an eHealth Diabetes Prevention Program Adapted for Older Adults—Results from a Randomized Control Pilot Study
Source: Nutrients. 2024 Mar 23;16(7):930. doi: 10.3390/nu16070930 (PMC11154527; doi:10.3390/nu16070930)
Supplement: Supplementary file 1 [file nutrients-16-00930-s001.zip › Week2.pptx]

## Slide 1
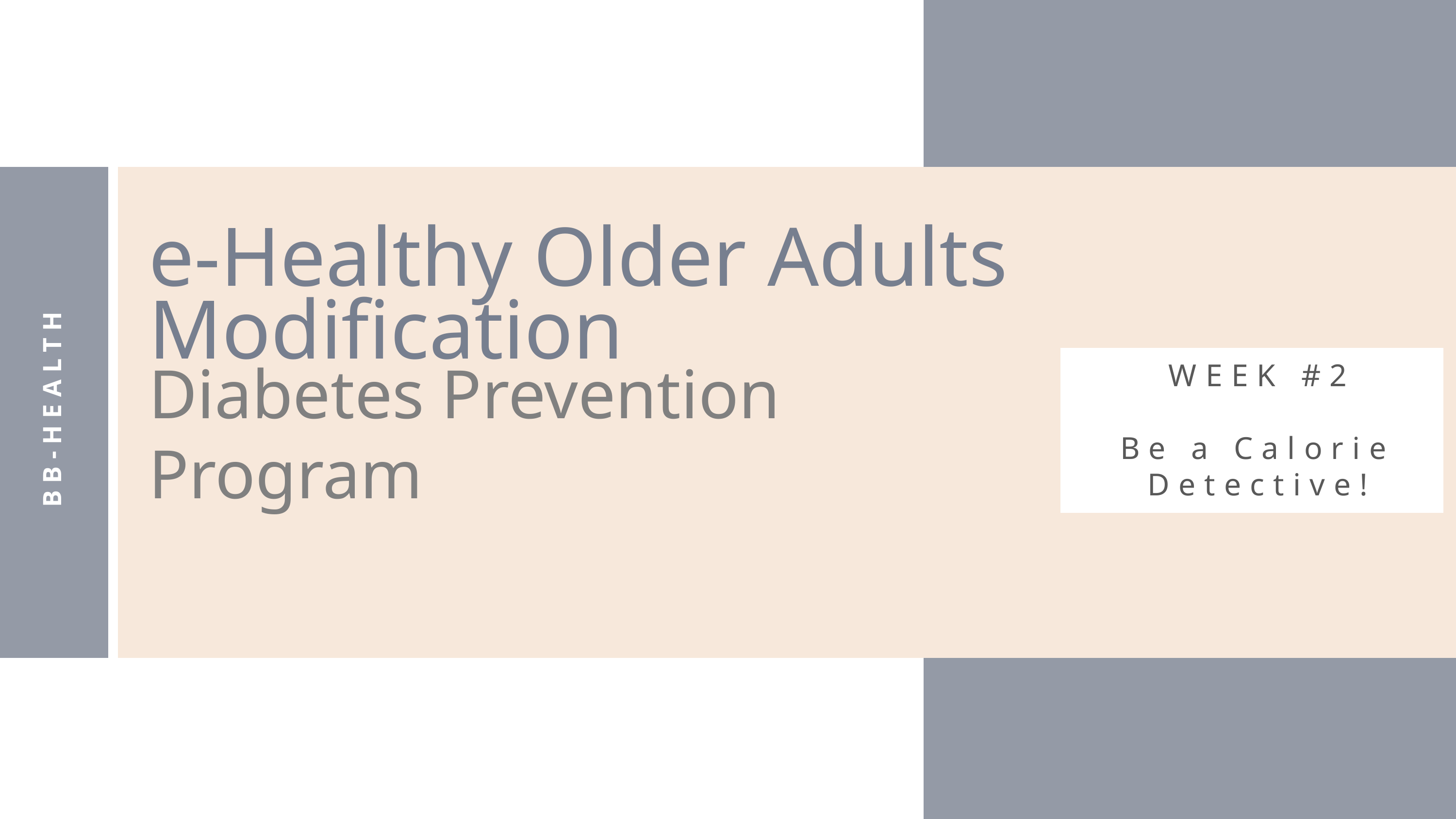

OPEN REPORTS
e-Healthy Older Adults Modification
WEEK #2
Be a Calorie Detective!
Diabetes Prevention Program
BB-HEALTH

## Slide 2
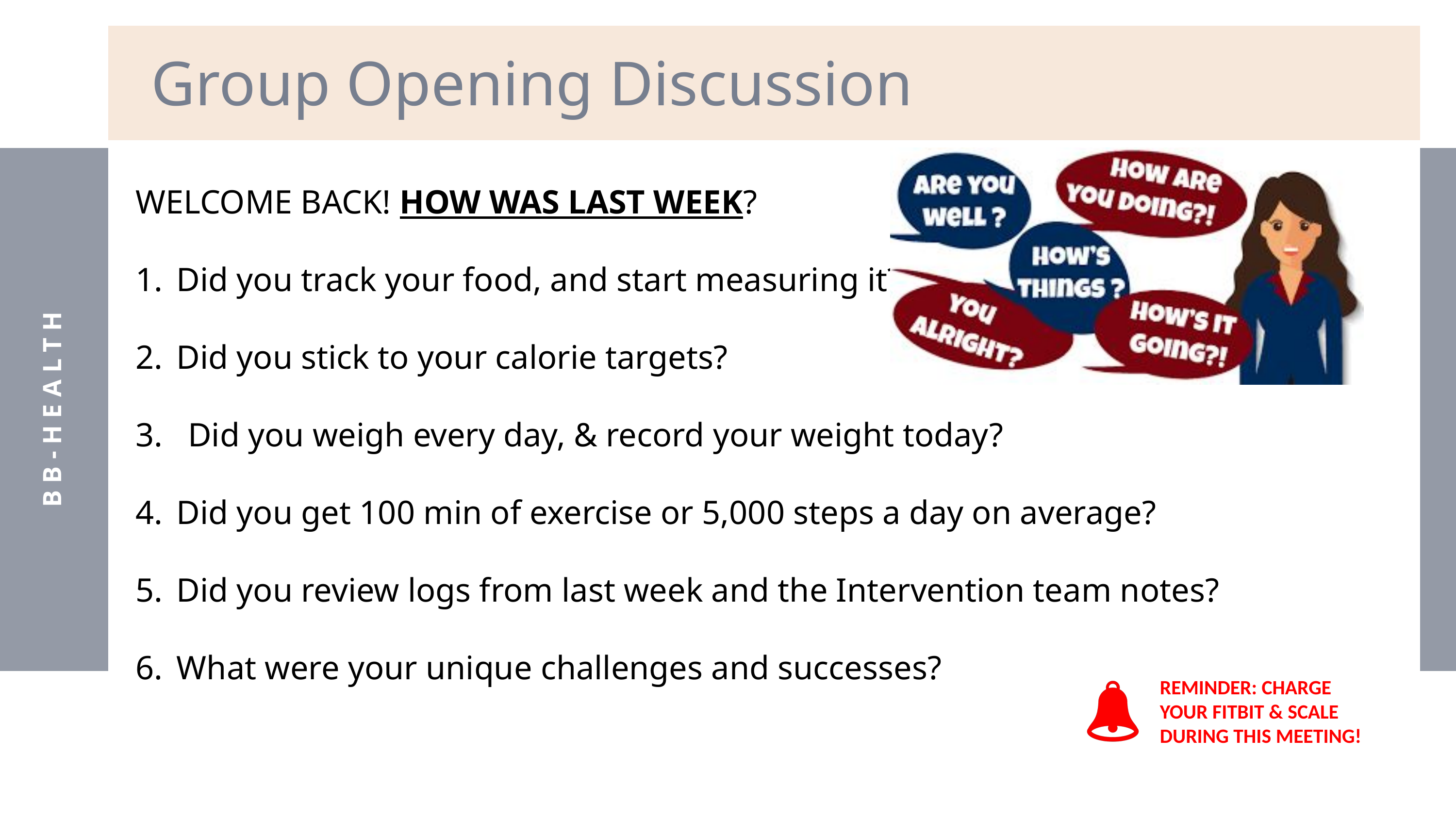

Group Opening Discussion
WELCOME BACK! HOW WAS LAST WEEK?
Did you track your food, and start measuring it?
Did you stick to your calorie targets?
3. Did you weigh every day, & record your weight today?
Did you get 100 min of exercise or 5,000 steps a day on average?
Did you review logs from last week and the Intervention team notes?
What were your unique challenges and successes?
BB-HEALTH
REMINDER: CHARGE YOUR FITBIT & SCALE DURING THIS MEETING!

## Slide 3
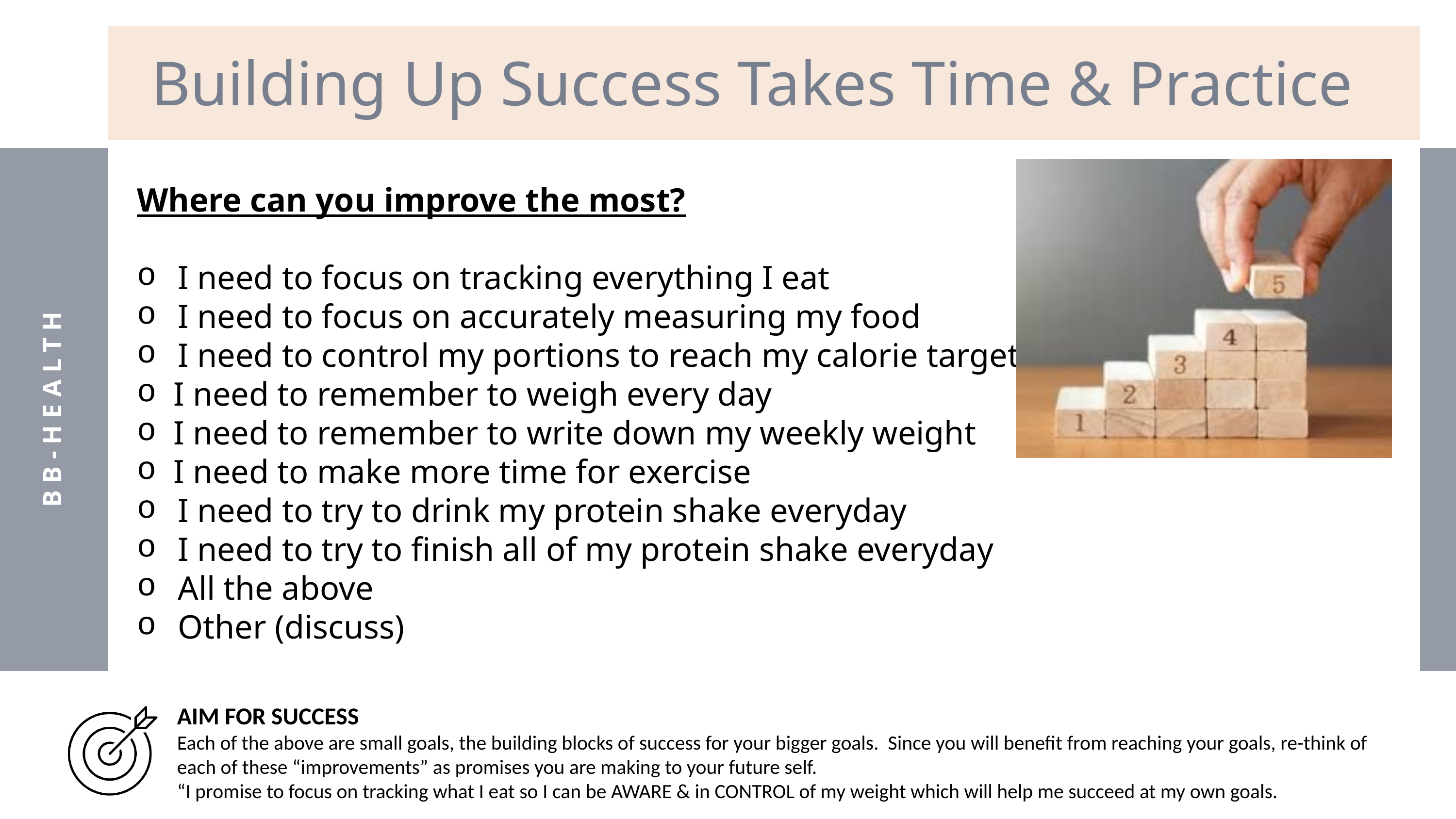

Building Up Success Takes Time & Practice
Where can you improve the most?
I need to focus on tracking everything I eat
I need to focus on accurately measuring my food
I need to control my portions to reach my calorie target
I need to remember to weigh every day
I need to remember to write down my weekly weight
I need to make more time for exercise
I need to try to drink my protein shake everyday
I need to try to finish all of my protein shake everyday
All the above
Other (discuss)
BB-HEALTH
AIM FOR SUCCESS
Each of the above are small goals, the building blocks of success for your bigger goals. Since you will benefit from reaching your goals, re-think of each of these “improvements” as promises you are making to your future self.
“I promise to focus on tracking what I eat so I can be AWARE & in CONTROL of my weight which will help me succeed at my own goals.

## Slide 4
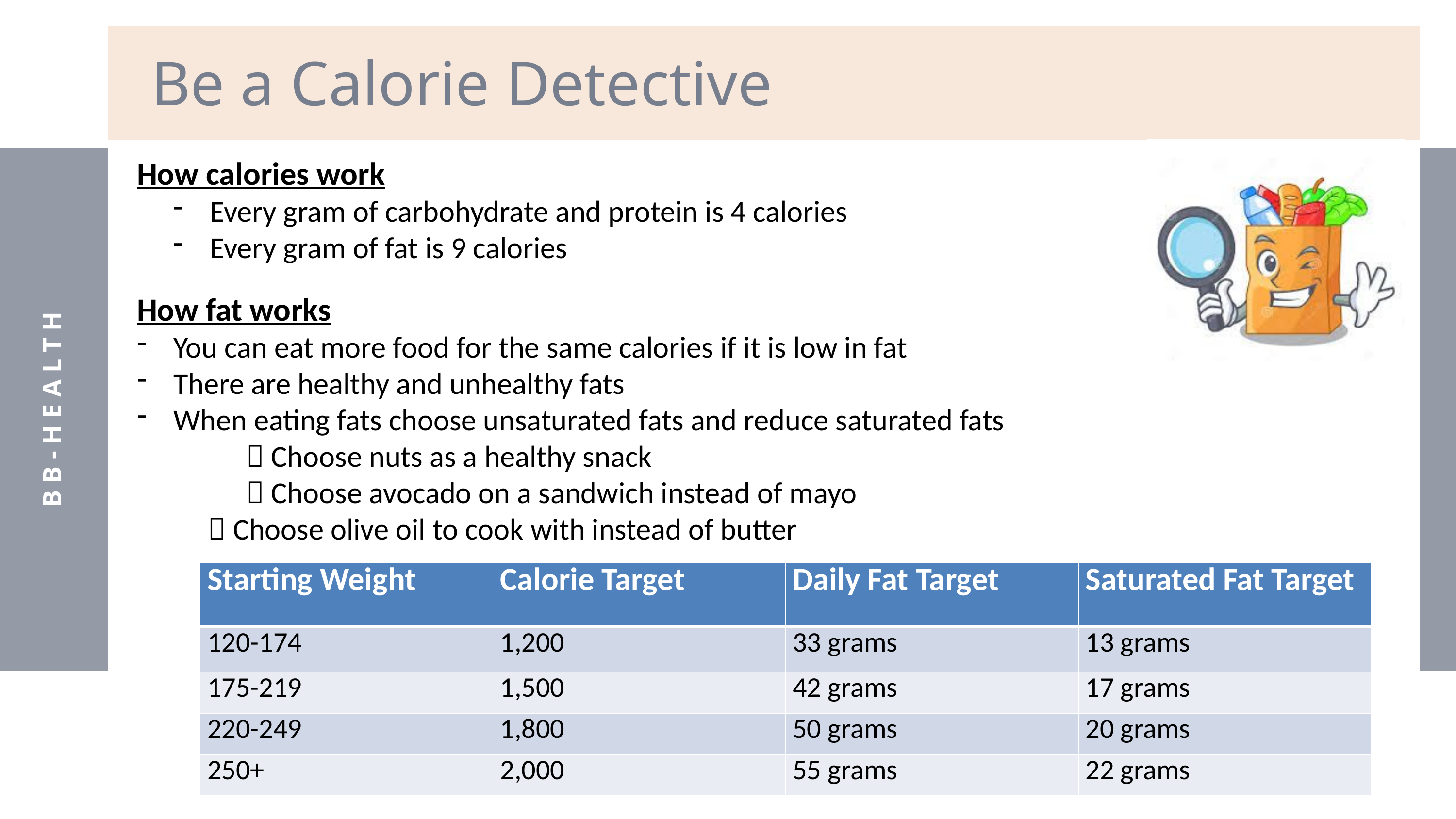

Be a Calorie Detective
How calories work
Every gram of carbohydrate and protein is 4 calories
Every gram of fat is 9 calories
How fat works
You can eat more food for the same calories if it is low in fat
There are healthy and unhealthy fats
When eating fats choose unsaturated fats and reduce saturated fats
	 Choose nuts as a healthy snack
	 Choose avocado on a sandwich instead of mayo
  Choose olive oil to cook with instead of butter
BB-HEALTH
| Starting Weight | Calorie Target | Daily Fat Target | Saturated Fat Target |
| --- | --- | --- | --- |
| 120-174 | 1,200 | 33 grams | 13 grams |
| 175-219 | 1,500 | 42 grams | 17 grams |
| 220-249 | 1,800 | 50 grams | 20 grams |
| 250+ | 2,000 | 55 grams | 22 grams |
HOW DO WE EAT A MODERATE FAT DIET?

## Slide 5
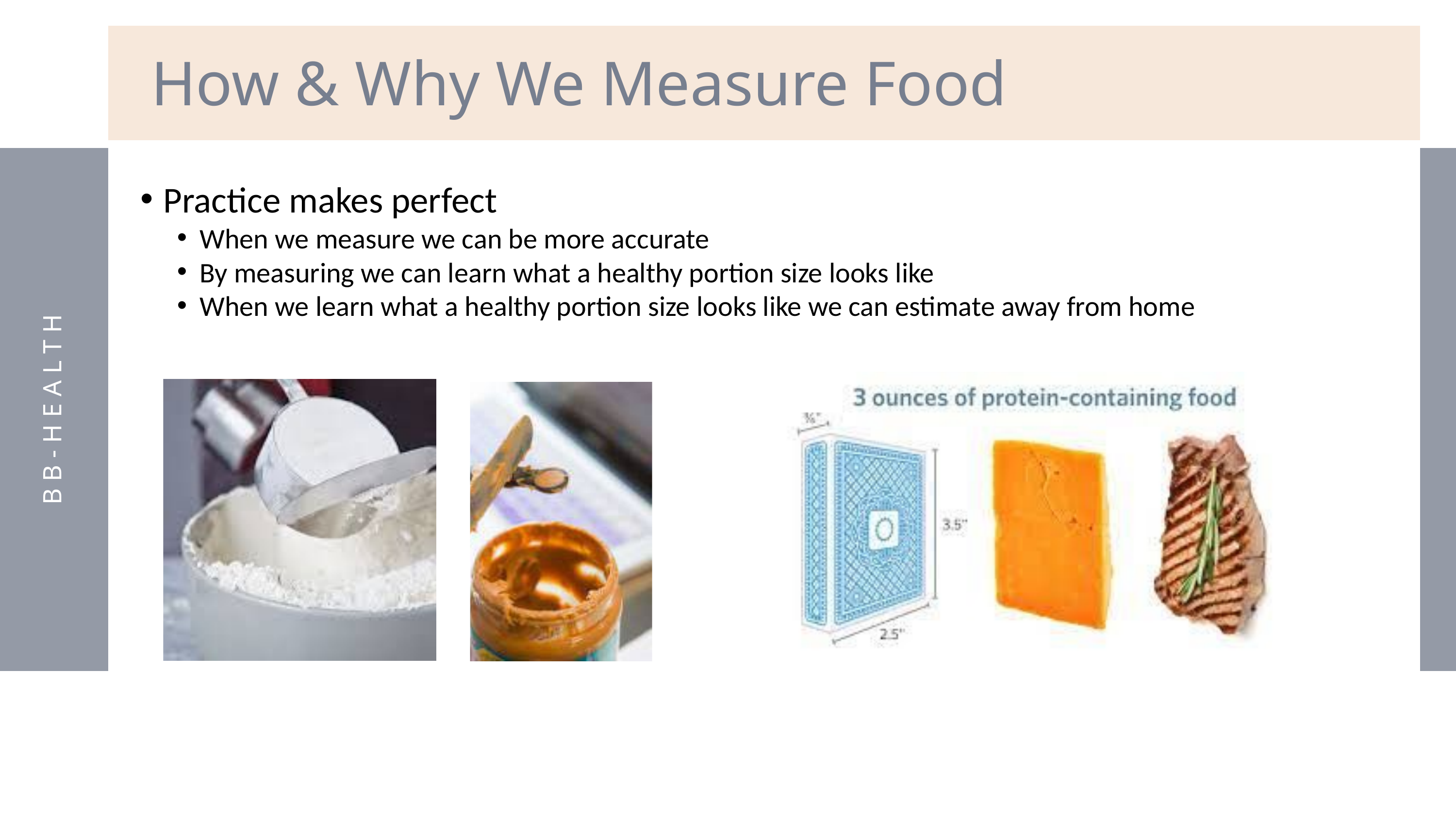

How & Why We Measure Food
Practice makes perfect
When we measure we can be more accurate
By measuring we can learn what a healthy portion size looks like
When we learn what a healthy portion size looks like we can estimate away from home
BB-HEALTH

## Slide 6
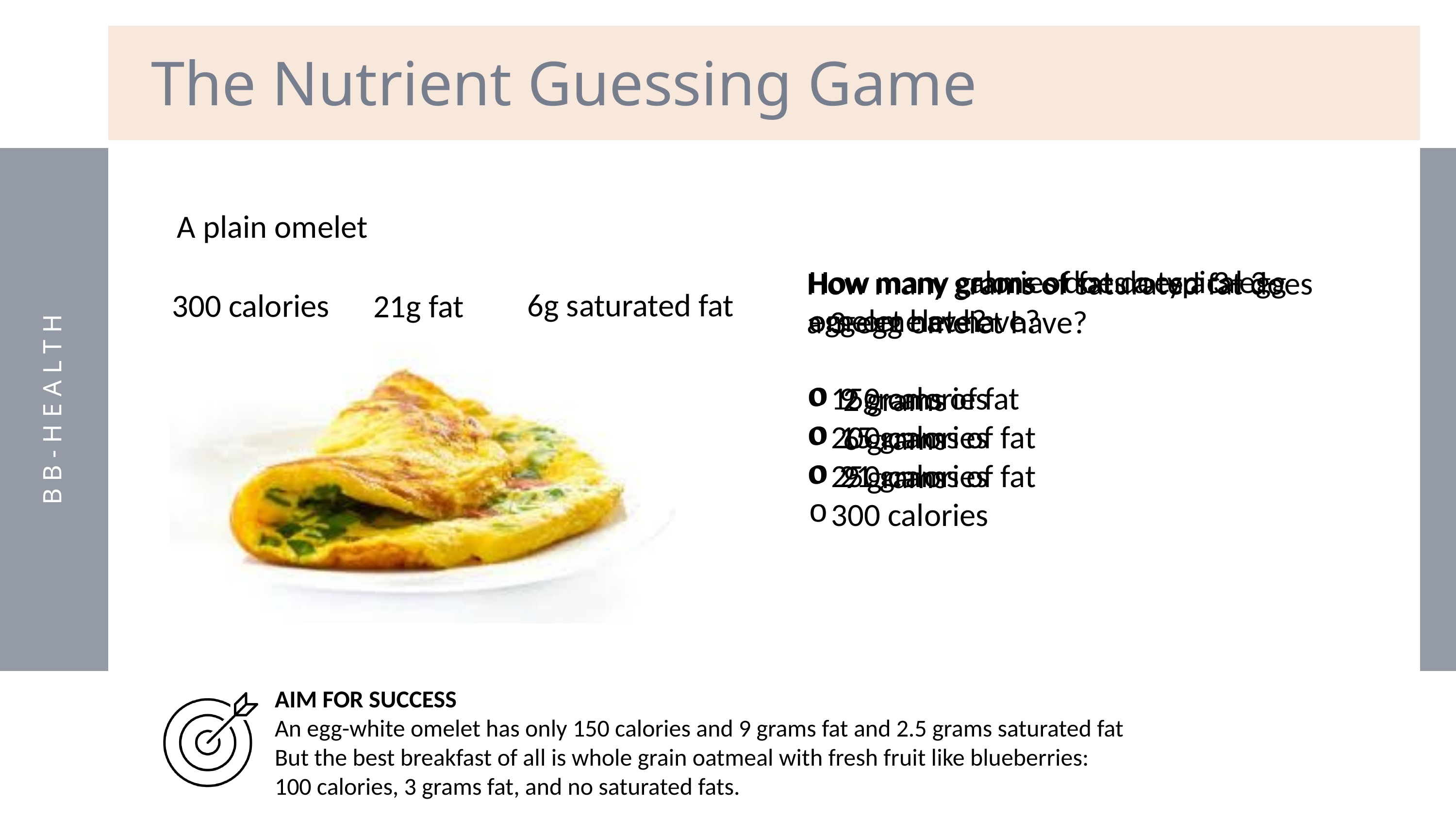

The Nutrient Guessing Game
A plain omelet
How many calories does a typical 3-egg omelet have?
150 calories
200 calories
250 calories
300 calories
How many grams of fat does a 3-egg omelet have?
 9 grams of fat
 15 grams of fat
 21 grams of fat
How many grams of saturated fat does a 3-egg omelet have?
2 grams
6 grams
9 grams
6g saturated fat
300 calories
21g fat
BB-HEALTH
AIM FOR SUCCESS
An egg-white omelet has only 150 calories and 9 grams fat and 2.5 grams saturated fat
But the best breakfast of all is whole grain oatmeal with fresh fruit like blueberries:
100 calories, 3 grams fat, and no saturated fats.

## Slide 7
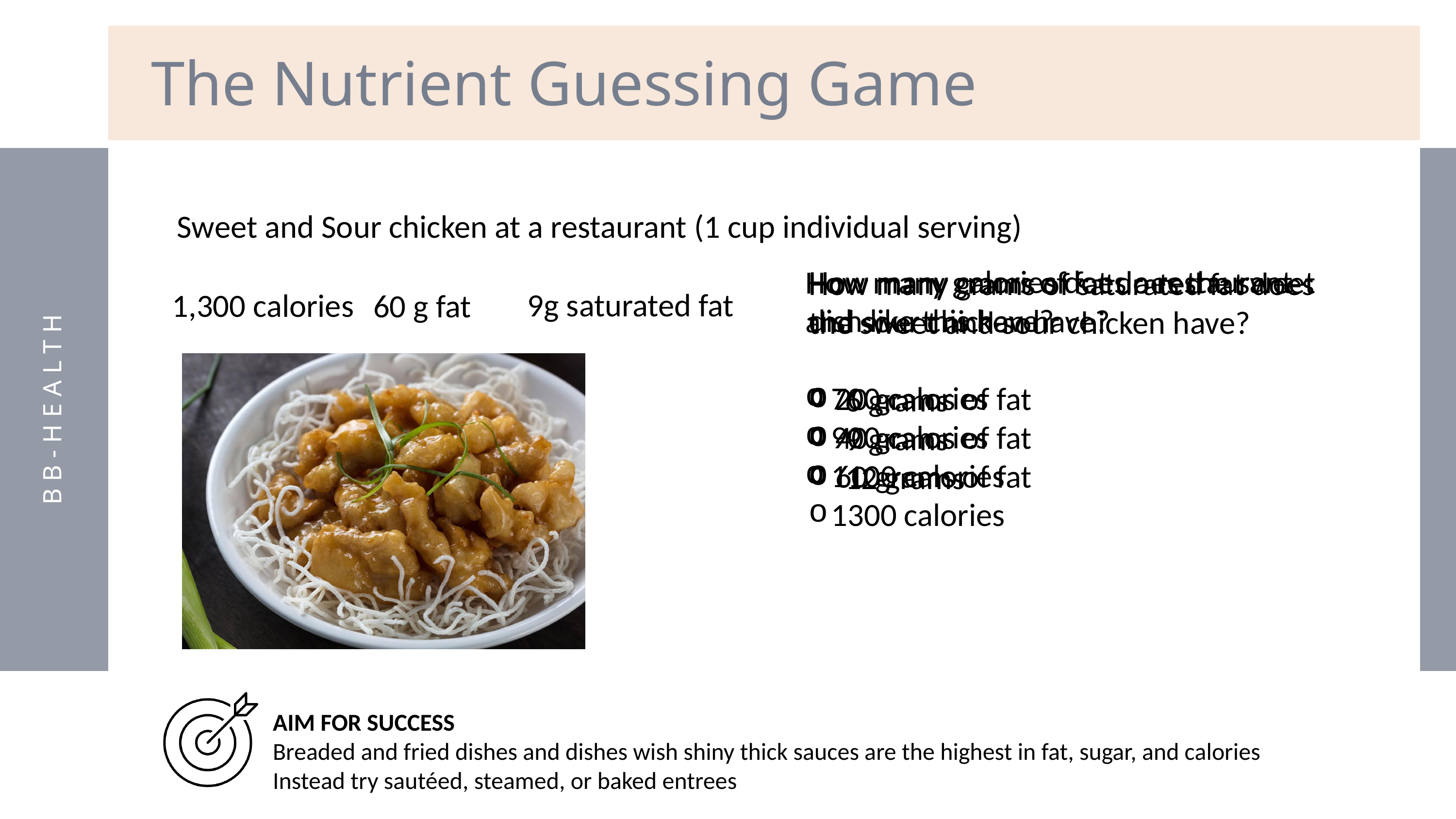

The Nutrient Guessing Game
Sweet and Sour chicken at a restaurant (1 cup individual serving)
How many calories does a restaurant dish like this have?
700 calories
900 calories
1100 calories
1300 calories
How many grams of fat does the sweet and sour chicken have?
 20 grams of fat
 40 grams of fat
 60 grams of fat
How many grams of saturated fat does the sweet and sour chicken have?
6 grams
9 grams
12 grams
9g saturated fat
1,300 calories
60 g fat
BB-HEALTH
AIM FOR SUCCESS
Breaded and fried dishes and dishes wish shiny thick sauces are the highest in fat, sugar, and calories
Instead try sautéed, steamed, or baked entrees

## Slide 8
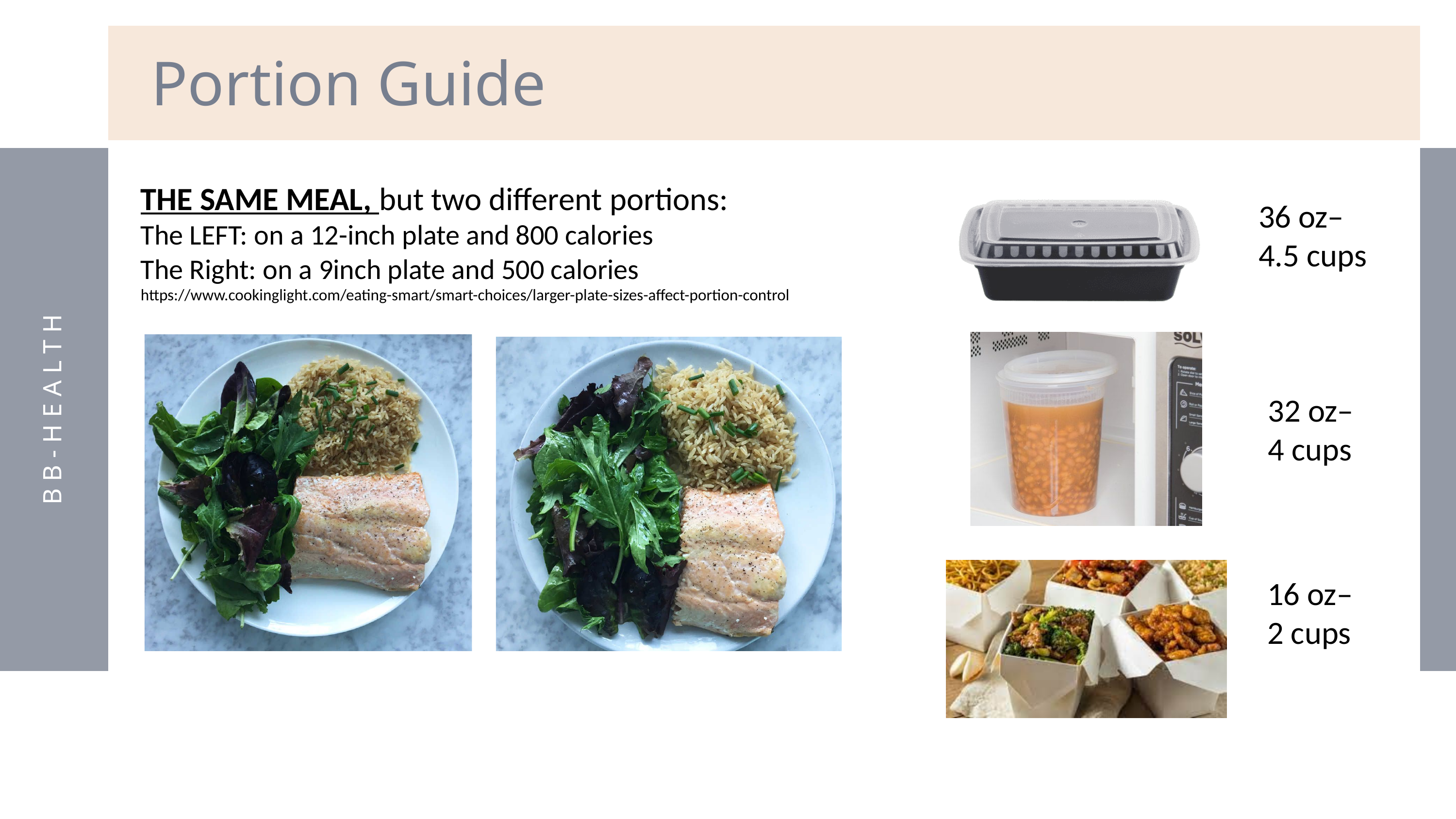

Portion Guide
THE SAME MEAL, but two different portions:
The LEFT: on a 12-inch plate and 800 calories
The Right: on a 9inch plate and 500 calories
https://www.cookinglight.com/eating-smart/smart-choices/larger-plate-sizes-affect-portion-control
36 oz– 4.5 cups
32 oz–
4 cups
BB-HEALTH
16 oz–
2 cups

## Slide 9
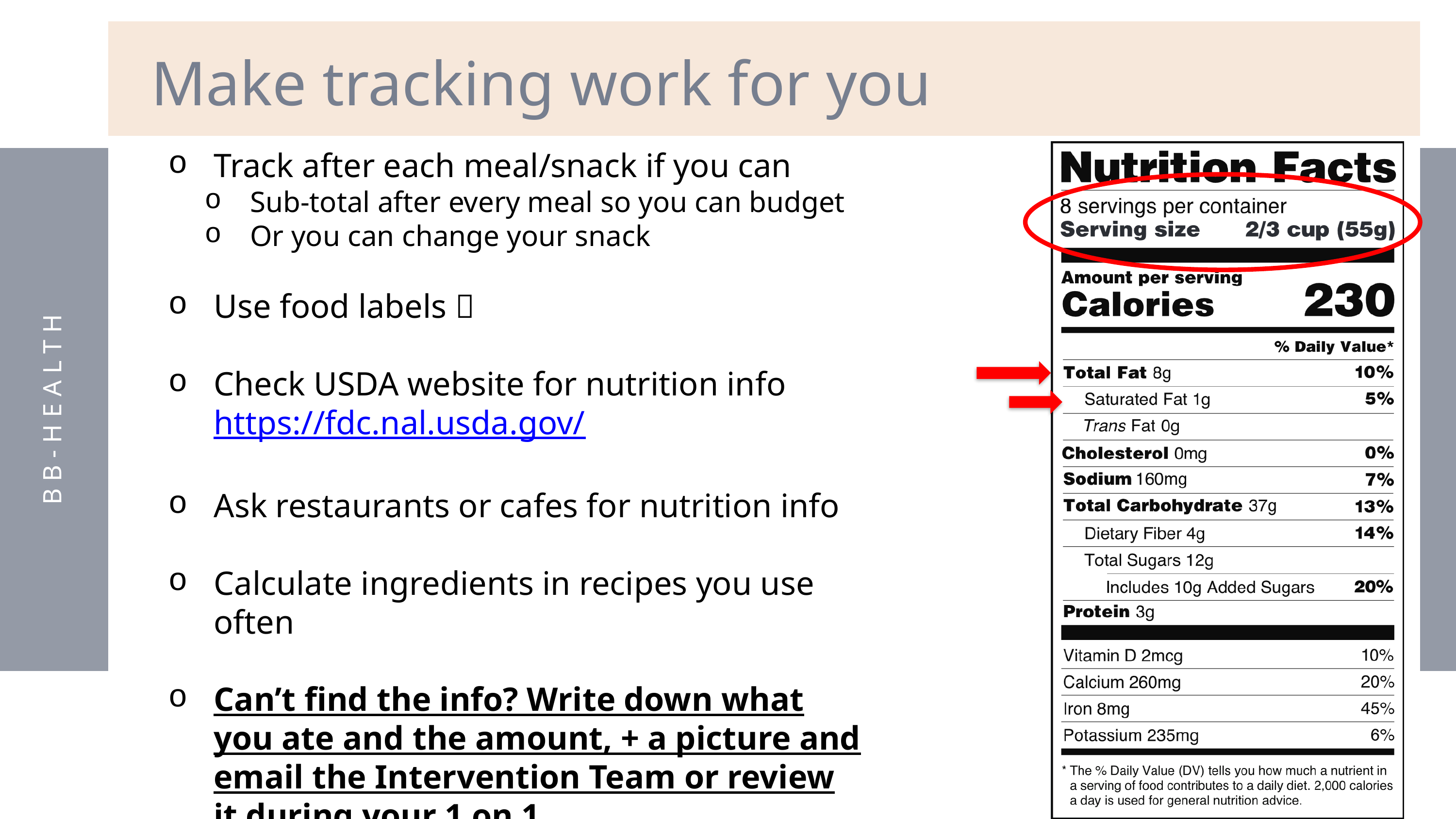

Make tracking work for you
Track after each meal/snack if you can
Sub-total after every meal so you can budget
Or you can change your snack
Use food labels 
Check USDA website for nutrition info https://fdc.nal.usda.gov/
Ask restaurants or cafes for nutrition info
Calculate ingredients in recipes you use often
Can’t find the info? Write down what you ate and the amount, + a picture and email the Intervention Team or review it during your 1 on 1
BB-HEALTH

## Slide 10
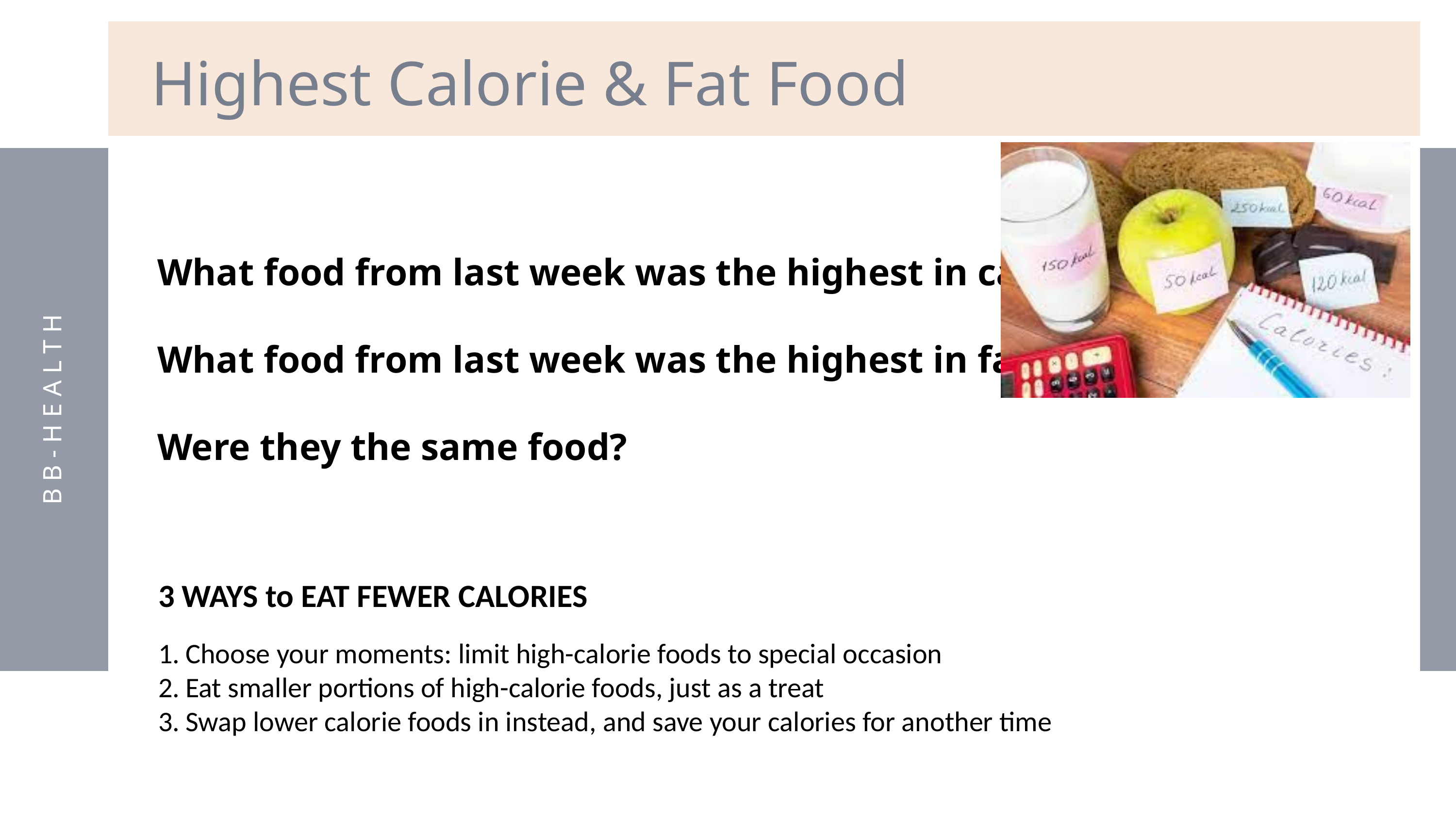

Highest Calorie & Fat Food
What food from last week was the highest in calories?
What food from last week was the highest in fat?
Were they the same food?
BB-HEALTH
3 WAYS to EAT FEWER CALORIES
Choose your moments: limit high-calorie foods to special occasion
Eat smaller portions of high-calorie foods, just as a treat
Swap lower calorie foods in instead, and save your calories for another time

## Slide 11
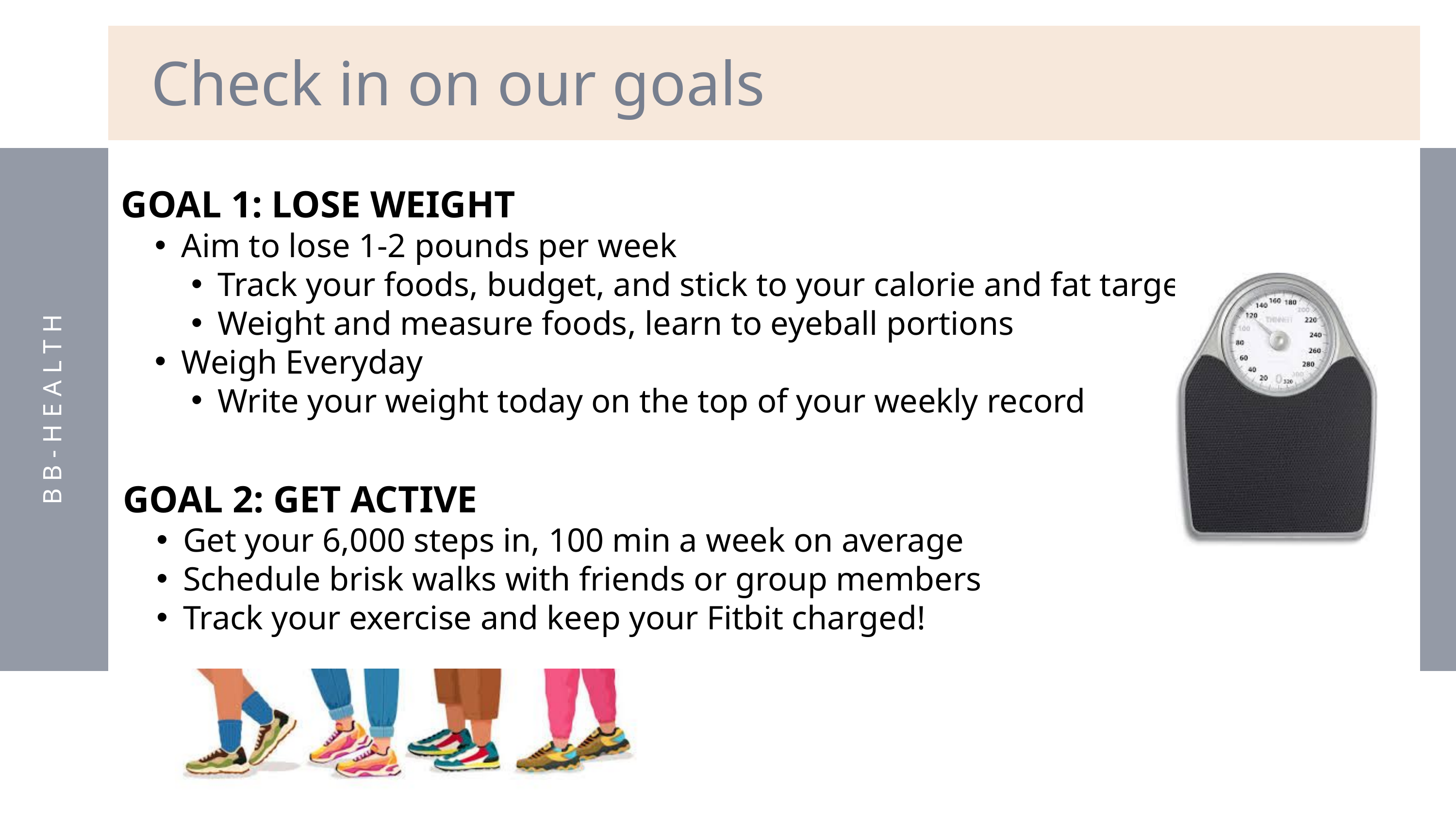

Check in on our goals
GOAL 1: LOSE WEIGHT
Aim to lose 1-2 pounds per week
Track your foods, budget, and stick to your calorie and fat targets
Weight and measure foods, learn to eyeball portions
Weigh Everyday
Write your weight today on the top of your weekly record
BB-HEALTH
BB-HEALTH
GOAL 2: GET ACTIVE
Get your 6,000 steps in, 100 min a week on average
Schedule brisk walks with friends or group members
Track your exercise and keep your Fitbit charged!

## Slide 12
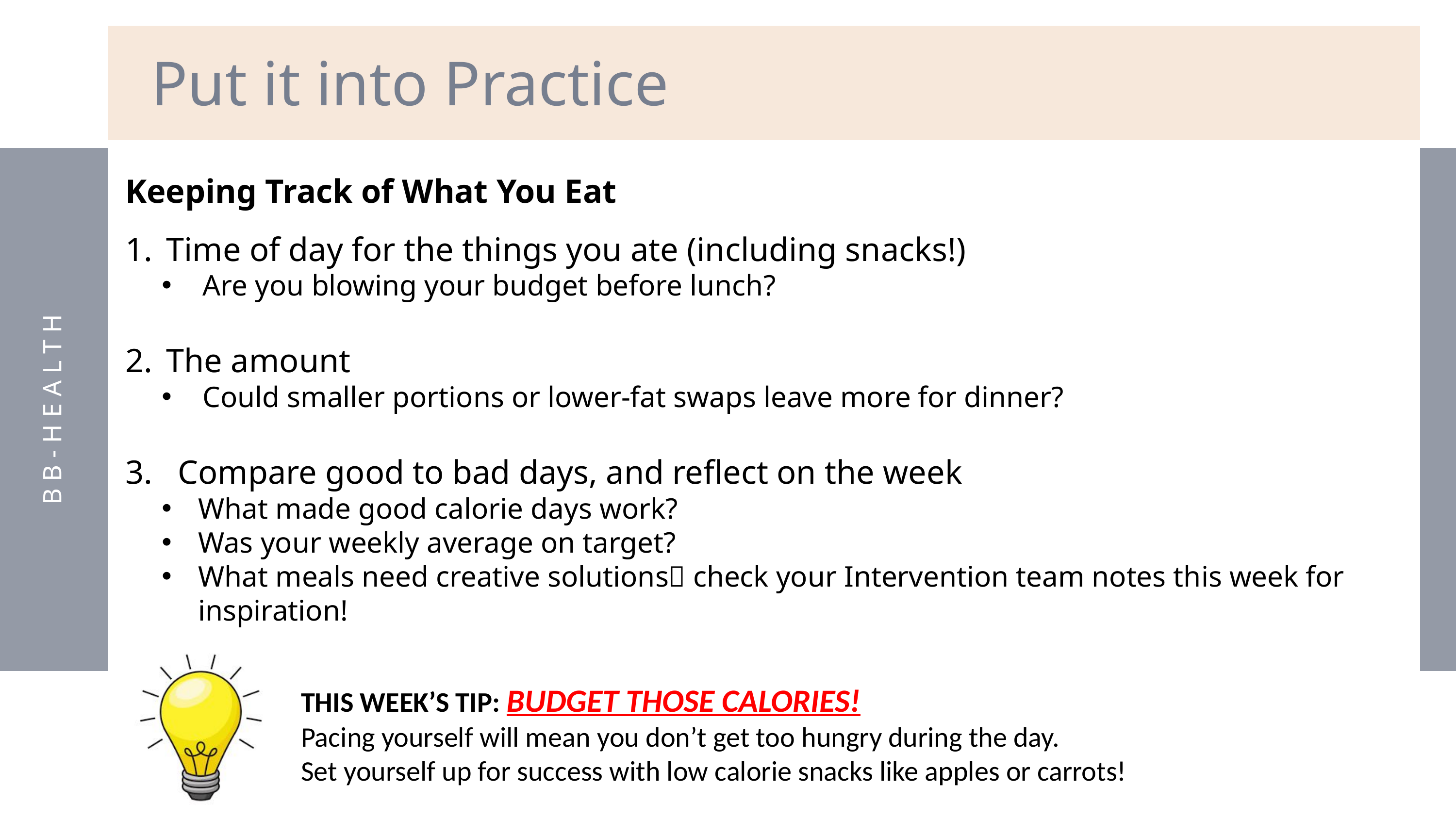

Put it into Practice
Keeping Track of What You Eat
Time of day for the things you ate (including snacks!)
Are you blowing your budget before lunch?
The amount
Could smaller portions or lower-fat swaps leave more for dinner?
3. Compare good to bad days, and reflect on the week
What made good calorie days work?
Was your weekly average on target?
What meals need creative solutions check your Intervention team notes this week for inspiration!
BB-HEALTH
THIS WEEK’S TIP: BUDGET THOSE CALORIES!
Pacing yourself will mean you don’t get too hungry during the day.
Set yourself up for success with low calorie snacks like apples or carrots!

## Slide 13
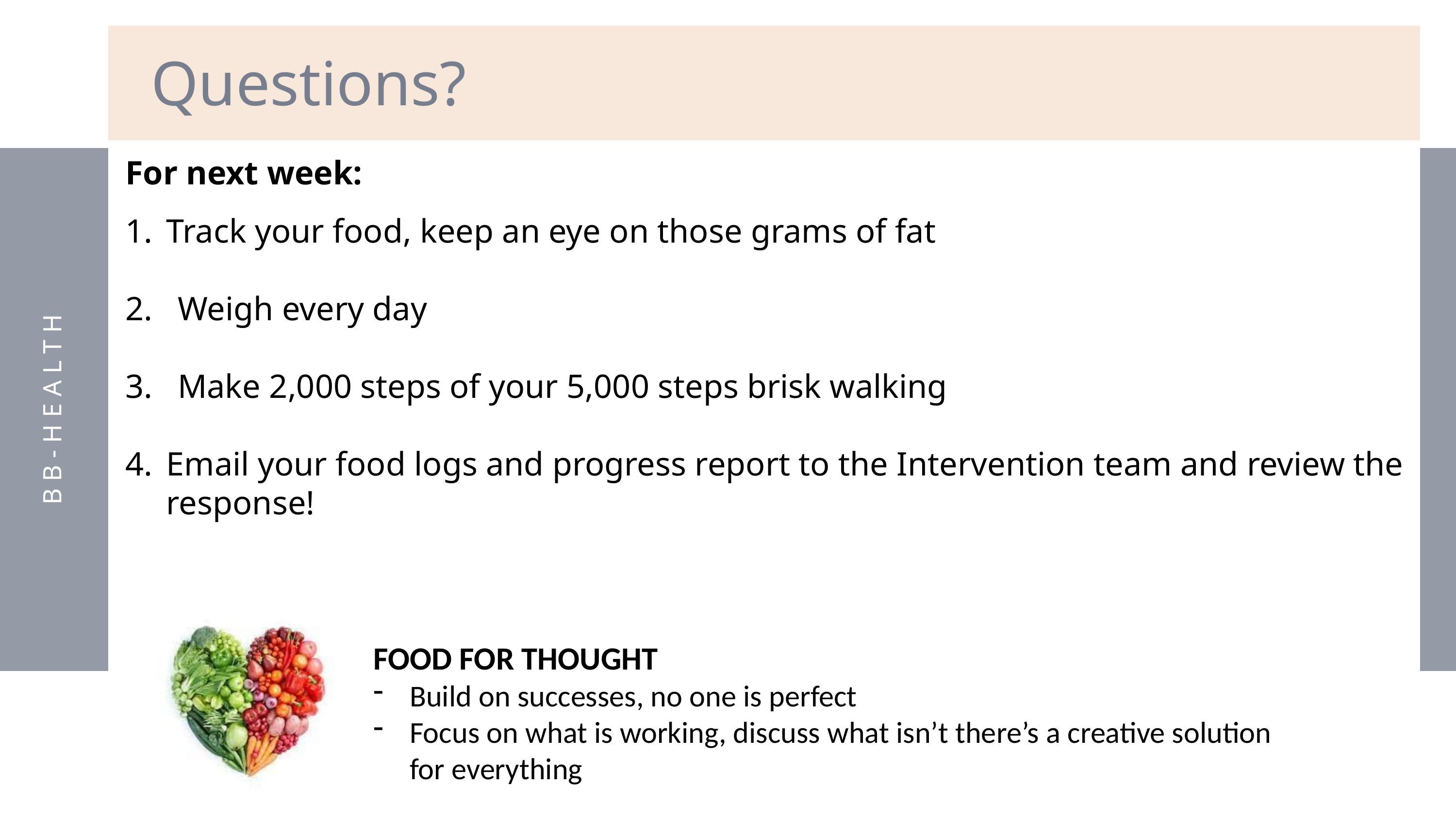

Questions?
For next week:
Track your food, keep an eye on those grams of fat
2. Weigh every day
3. Make 2,000 steps of your 5,000 steps brisk walking
Email your food logs and progress report to the Intervention team and review the response!
BB-HEALTH
FOOD FOR THOUGHT
Build on successes, no one is perfect
Focus on what is working, discuss what isn’t there’s a creative solution for everything
