# Supplementary material for: Development and Feasibility of an eHealth Diabetes Prevention Program Adapted for Older Adults—Results from a Randomized Control Pilot Study
Source: Nutrients. 2024 Mar 23;16(7):930. doi: 10.3390/nu16070930 (PMC11154527; doi:10.3390/nu16070930)
Supplement: Supplementary file 1 [file nutrients-16-00930-s001.zip › Week2_SUPP.pdf]

BB-HEALTH

e-Healthy Older Adults Modification

Diabetes Prevention Program

WEEK #2

Be a Calorie Detective!

Group Opening Discussion

WELCOME BACK! <u>**HOW WAS LAST WEEK**</u>?

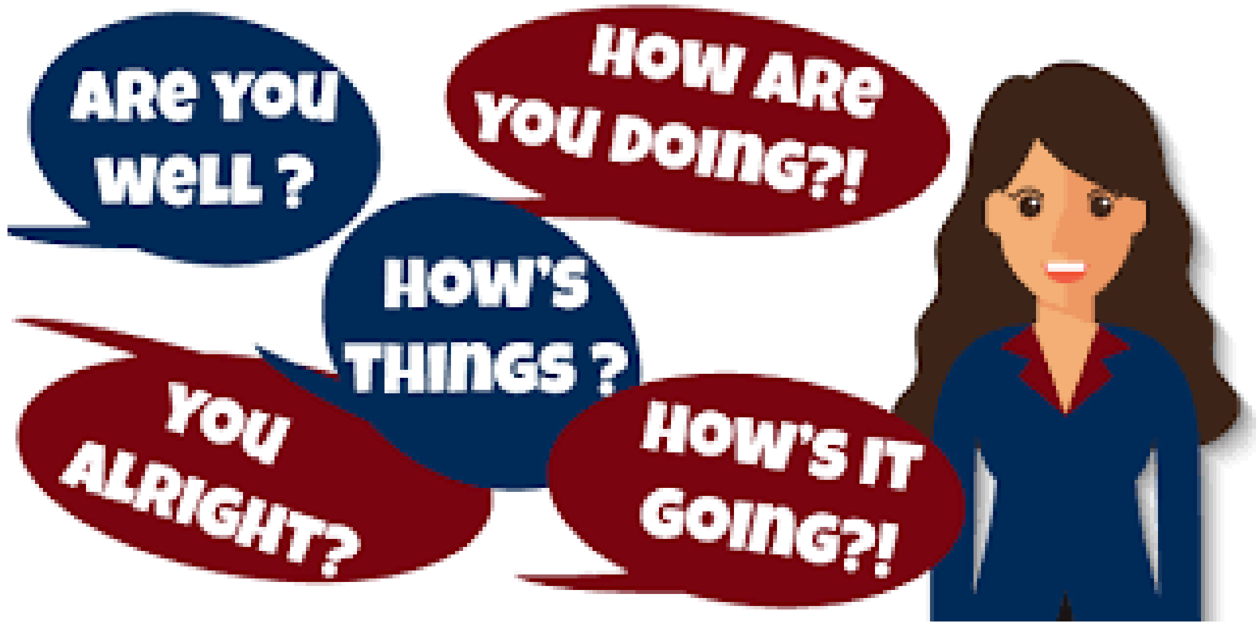

1. Did you track your food, and start measuring it?
2. Did you stick to your calorie targets?
3. Did you weigh every day, & record your weight today?
4. Did you get 100 min of exercise or 5,000 steps a day on average?
5. Did you review logs from last week and the Intervention team notes?
6. What were your unique challenges and successes?

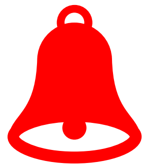

REMINDER: CHARGE YOUR FITBIT & SCALE DURING THIS MEETING!

Building Up Success Takes Time & Practice

Where can you improve the most?

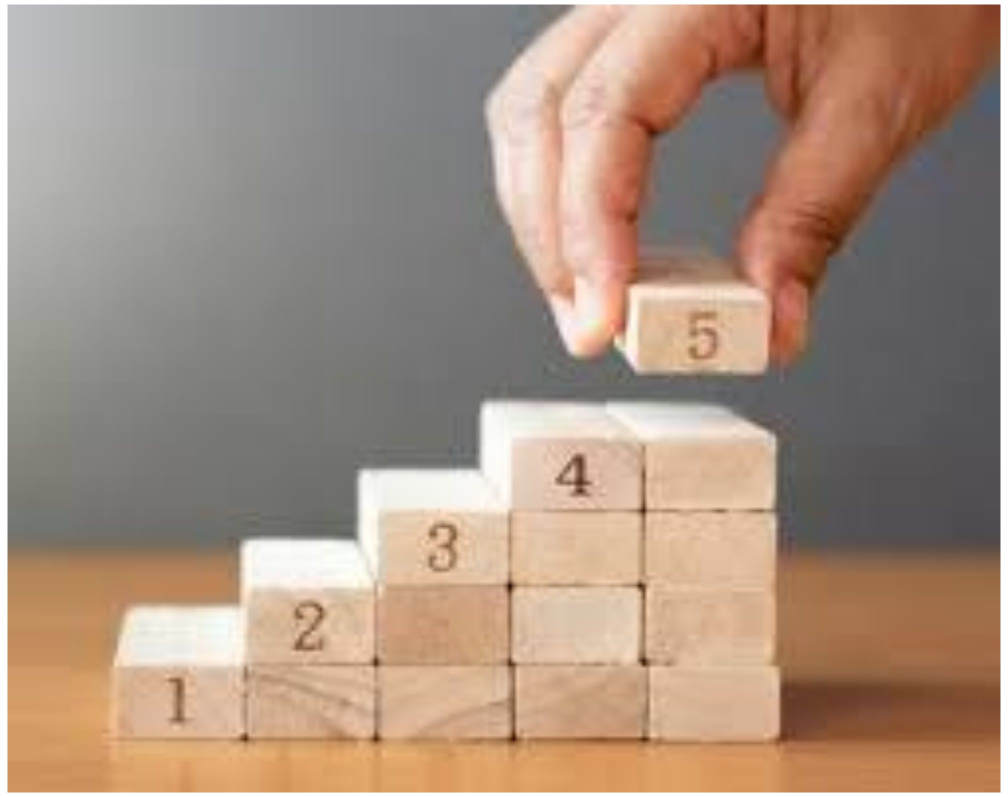

- I need to focus on tracking everything I eat
- I need to focus on accurately measuring my food
- I need to control my portions to reach my calorie target
- I need to remember to weigh every day
- I need to remember to write down my weekly weight
- I need to make more time for exercise
- I need to try to drink my protein shake everyday
- I need to try to finish all of my protein shake everyday
- All the above
- Other (discuss)

AIM FOR SUCCESS

Each of the above are small goals, the building blocks of success for your bigger goals. Since you will benefit from reaching your goals, re-think of each of these "improvements" as promises you are making to your future self.

"I promise to focus on tracking what I eat so I can be AWARE & in CONTROL of my weight which will help me succeed at my own goals.

Be a Calorie Detective

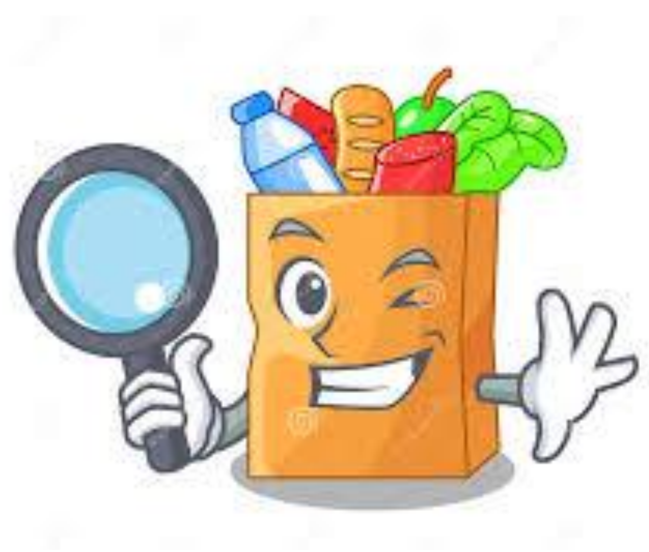

How calories work

- Every gram of carbohydrate and protein is 4 calories
- Every gram of fat is 9 calories

How fat works

- You can eat more food for the same calories if it is low in fat
- There are healthy and unhealthy fats
- When eating fats choose unsaturated fats and reduce saturated fats
  - → Choose nuts as a healthy snack
  - → Choose avocado on a sandwich instead of mayo
  - → Choose olive oil to cook with instead of butter

Starting Weight	Calorie Target	Daily Fat Target	Saturated Fat Target
120-174	1,200	33 grams	13 grams
175-219	1,500	42 grams	17 grams
220-249	1,800	50 grams	20 grams
250+	2,000	55 grams	22 grams

How & Why We Measure Food

- Practice makes perfect
  - When we measure we can be more accurate
  - By measuring we can learn what a healthy portion size looks like
  - When we learn what a healthy portion size looks like we can estimate away from home

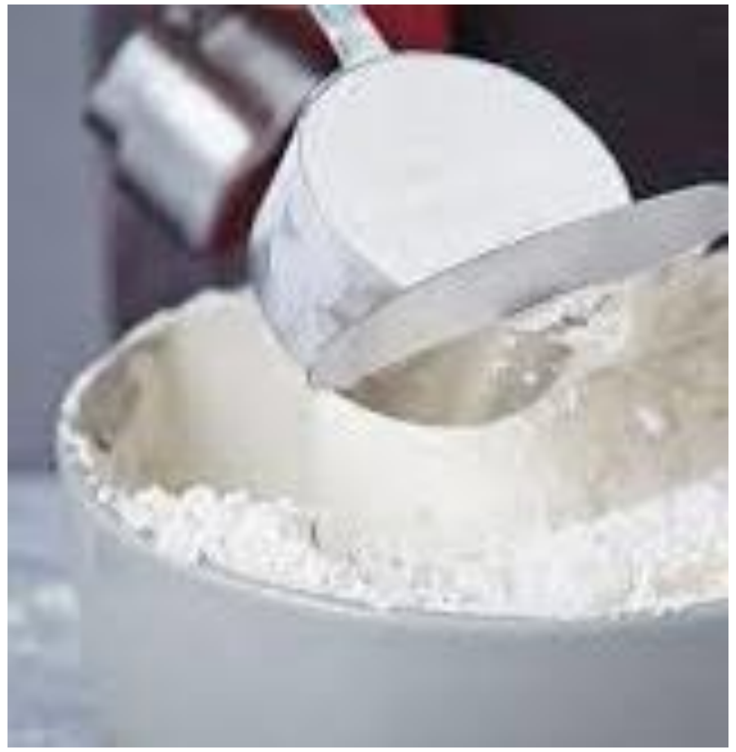

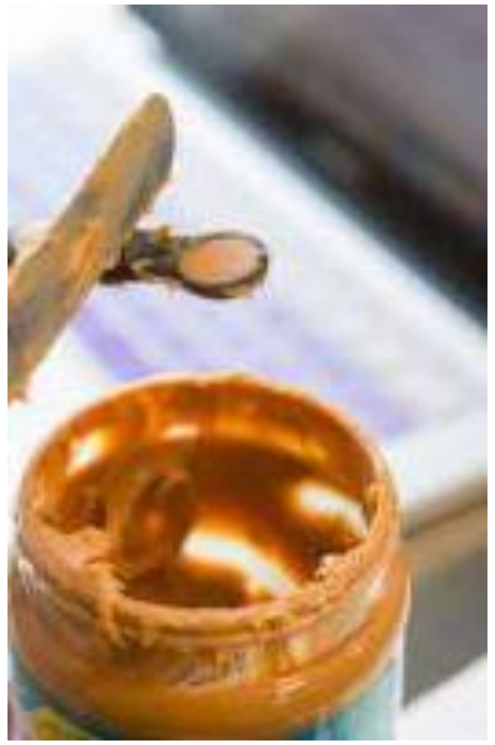

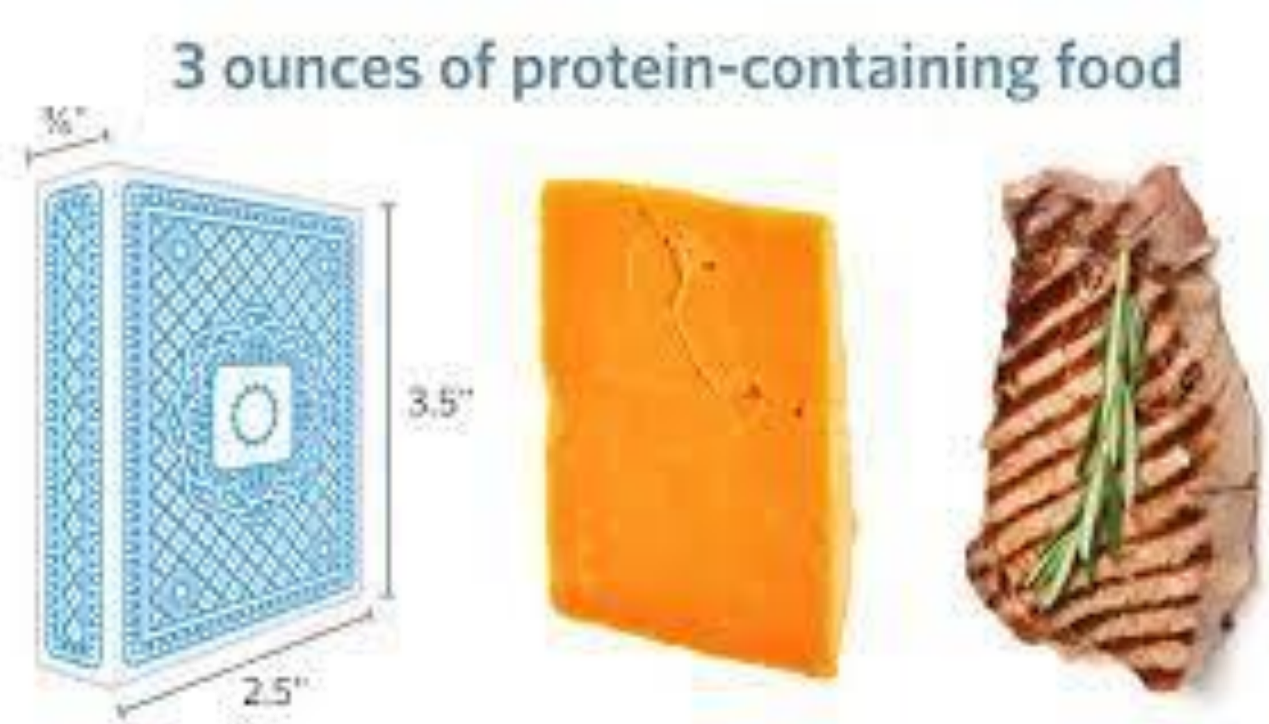

BB-HEALTH

The Nutrient Guessing Game

B B - H E A L T H

A plain omelet

300 calories 21g fat 6g saturated fat

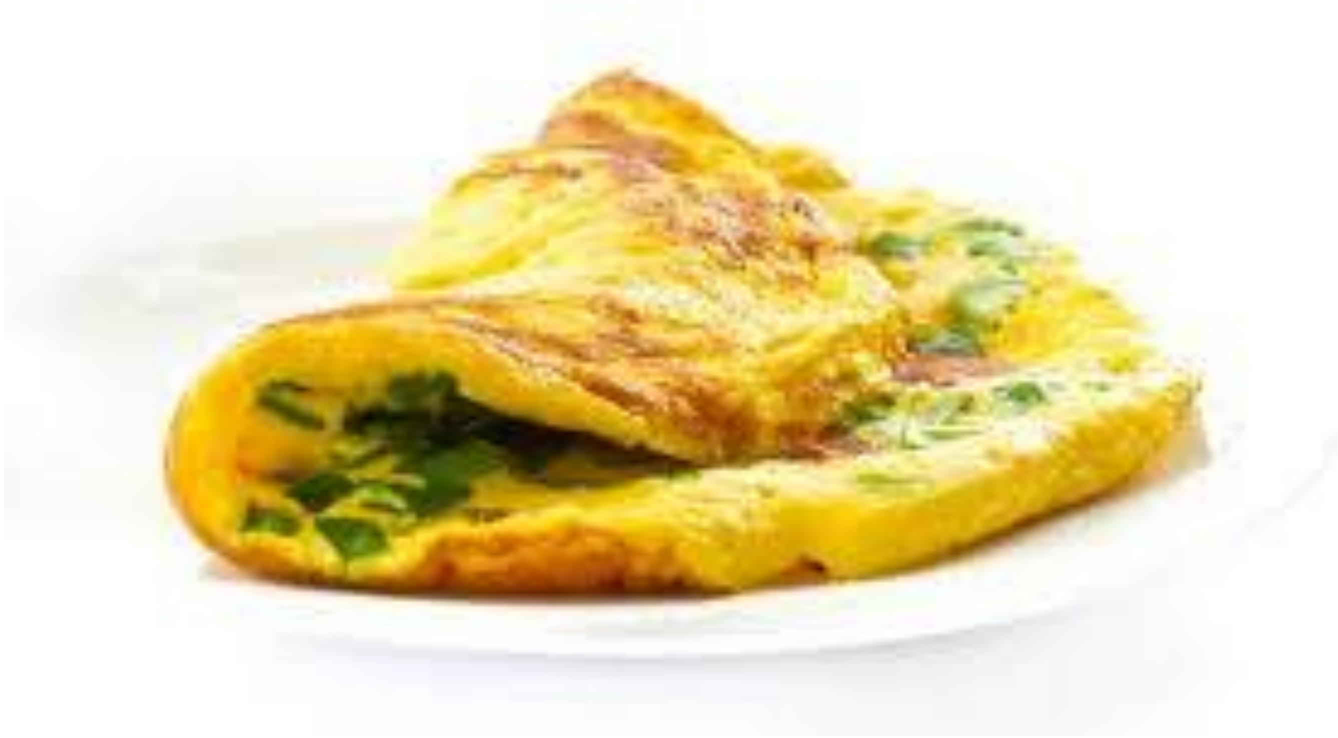

How many [illegible]

[illegible]

- [illegible]
- [illegible]
- [illegible]
- 300 calories

AIM FOR SUCCESS

An egg-white omelet has only 150 calories and 9 grams fat and 2.5 grams saturated fat
But the best breakfast of all is whole grain oatmeal with fresh fruit like blueberries:
100 calories, 3 grams fat, and no saturated fats.

The Nutrient Guessing Game

Sweet and Sour chicken at a restaurant (1 cup individual serving)

1,300 calories   60 g fat   9g saturated fat

How many grams of saturated fat does the sweet and sour chicken have?

- 200 calories of fat
- 400 calories of fat
- 600 calories of fat
- 1300 calories

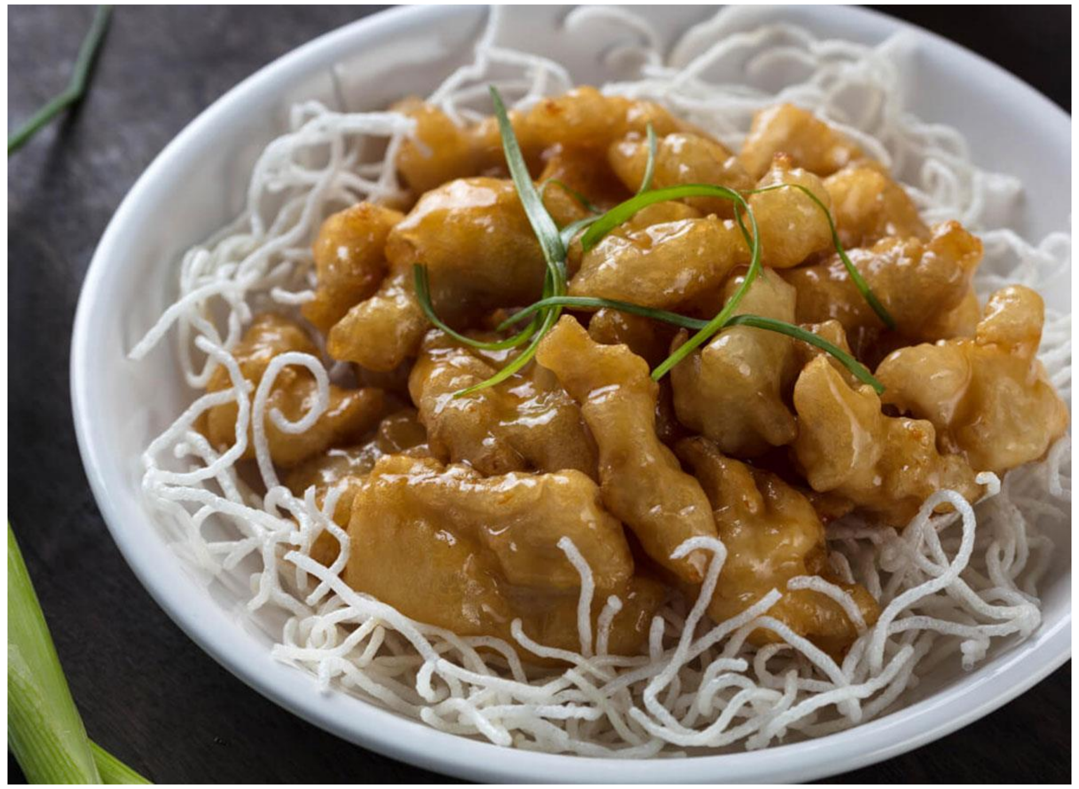

AIM FOR SUCCESS

Breaded and fried dishes and dishes wish shiny thick sauces are the highest in fat, sugar, and calories

Instead try sautéed, steamed, or baked entrees

Portion Guide

BB-HEALTH

<u>THE SAME MEAL,</u> but two different portions:

The LEFT: on a 12-inch plate and 800 calories

The Right: on a 9inch plate and 500 calories

https://www.cookinglight.com/eating-smart/smart-choices/larger-plate-sizes-affect-portion-control

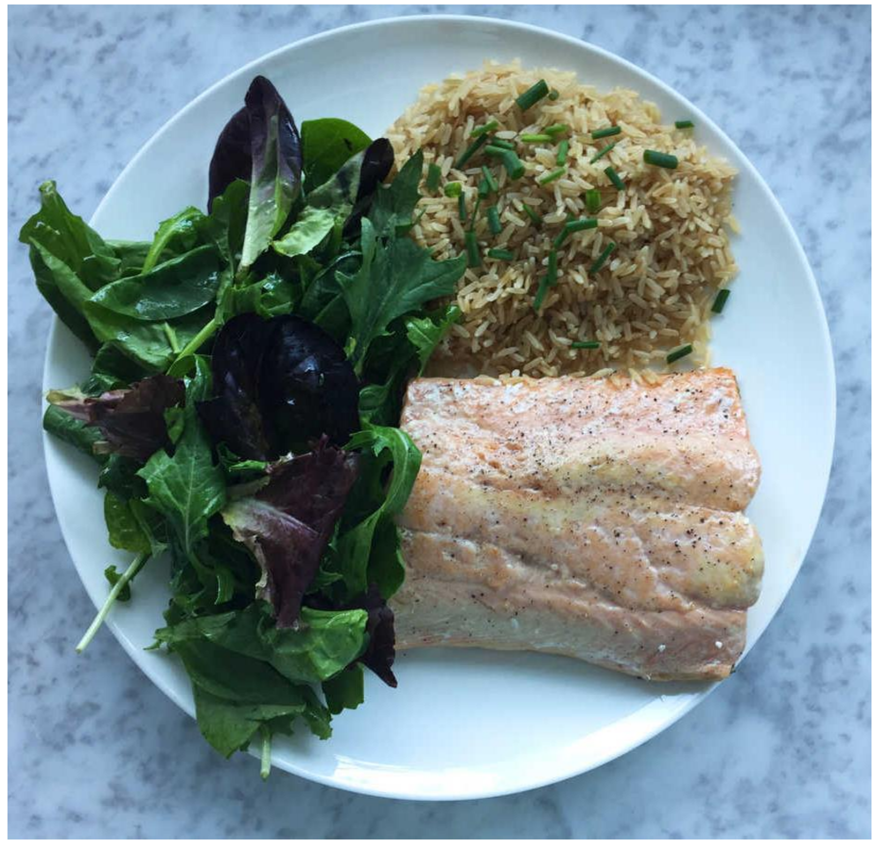

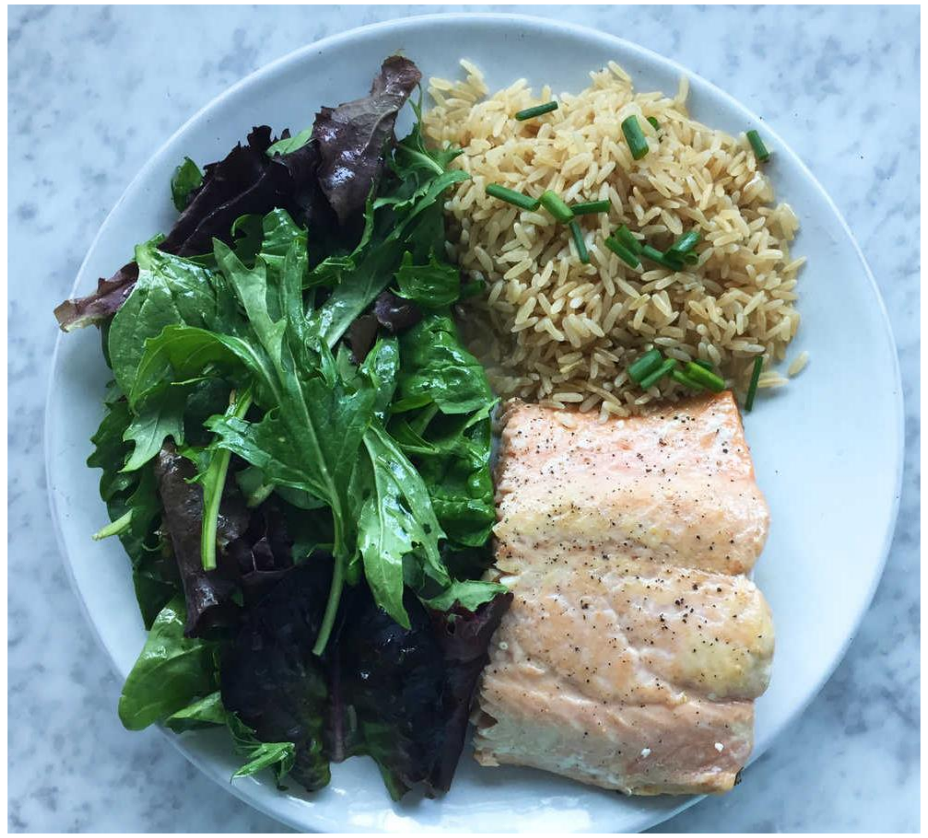

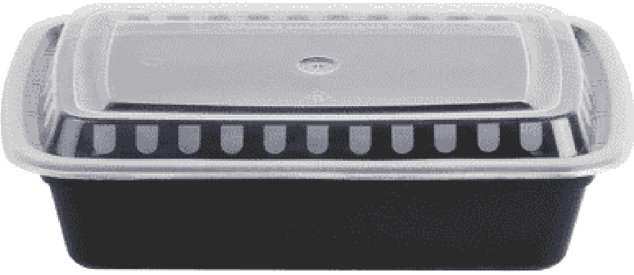

36 oz–
4.5 cups

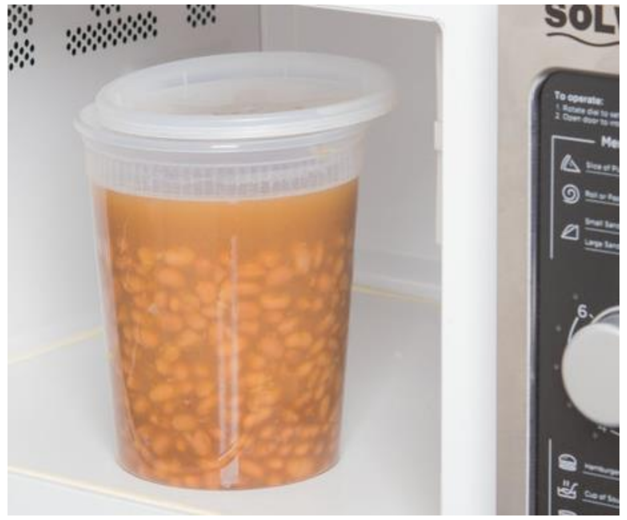

32 oz–
4 cups

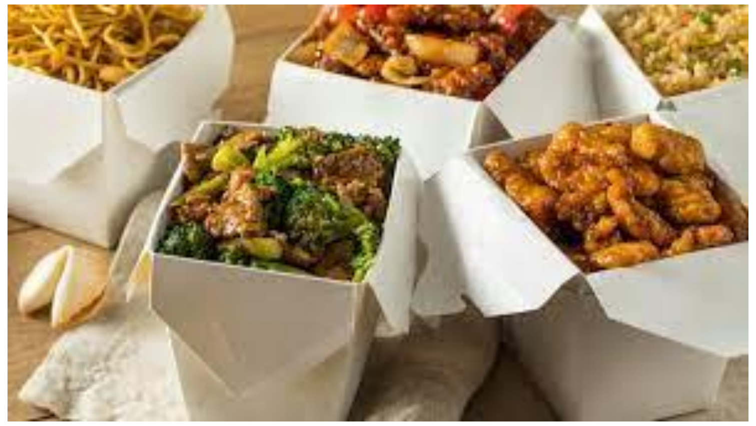

16 oz–
2 cups

Make tracking work for you

- Track after each meal/snack if you can
  - Sub-total after every meal so you can budget
  - Or you can change your snack
- Use food labels →
- Check USDA website for nutrition info https://fdc.nal.usda.gov/
- Ask restaurants or cafes for nutrition info
- Calculate ingredients in recipes you use often
- <u>Can't find the info? Write down what you ate and the amount, + a picture and email the Intervention Team or review it during your 1 on 1</u>

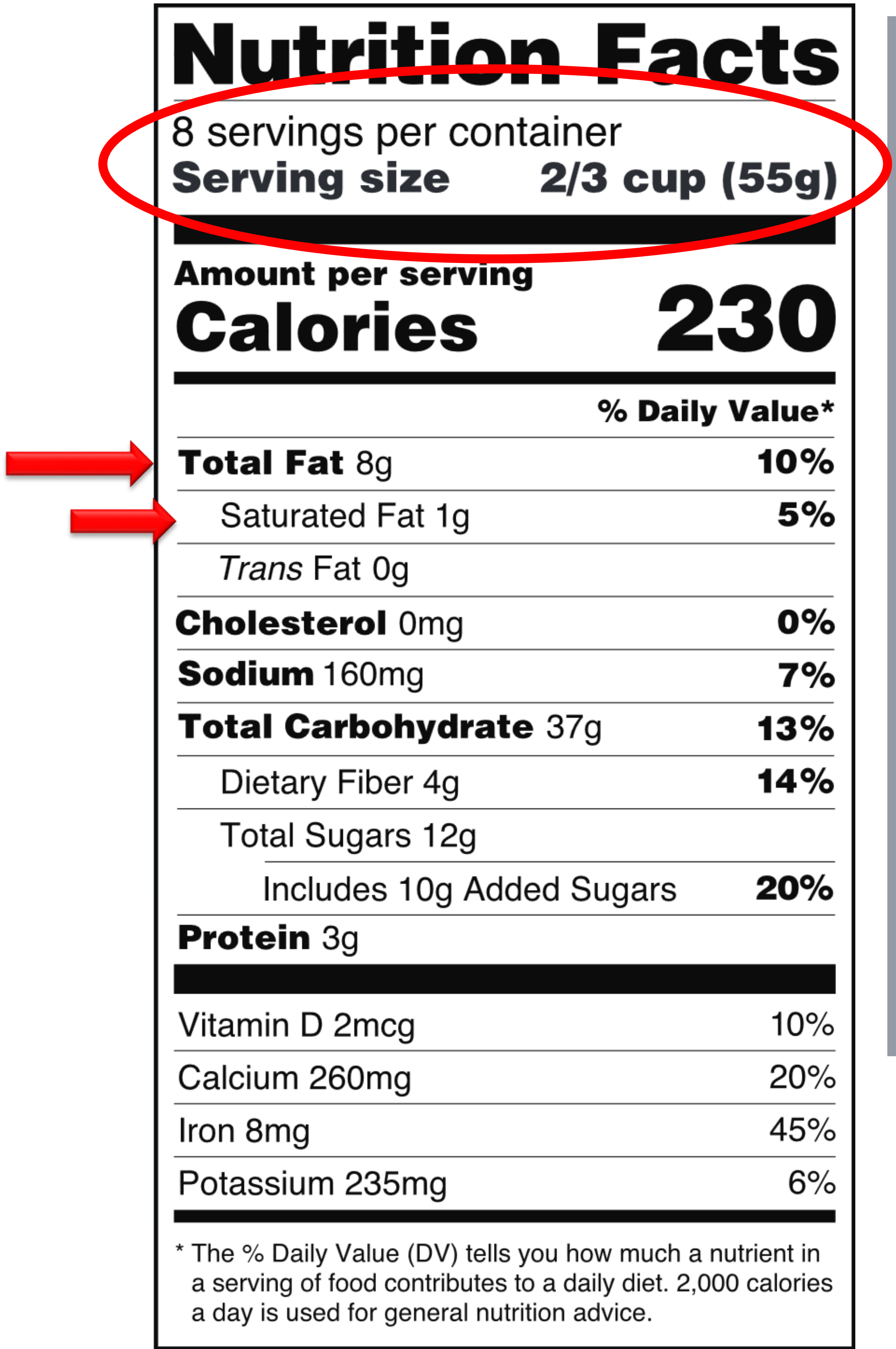

Highest Calorie & Fat Food

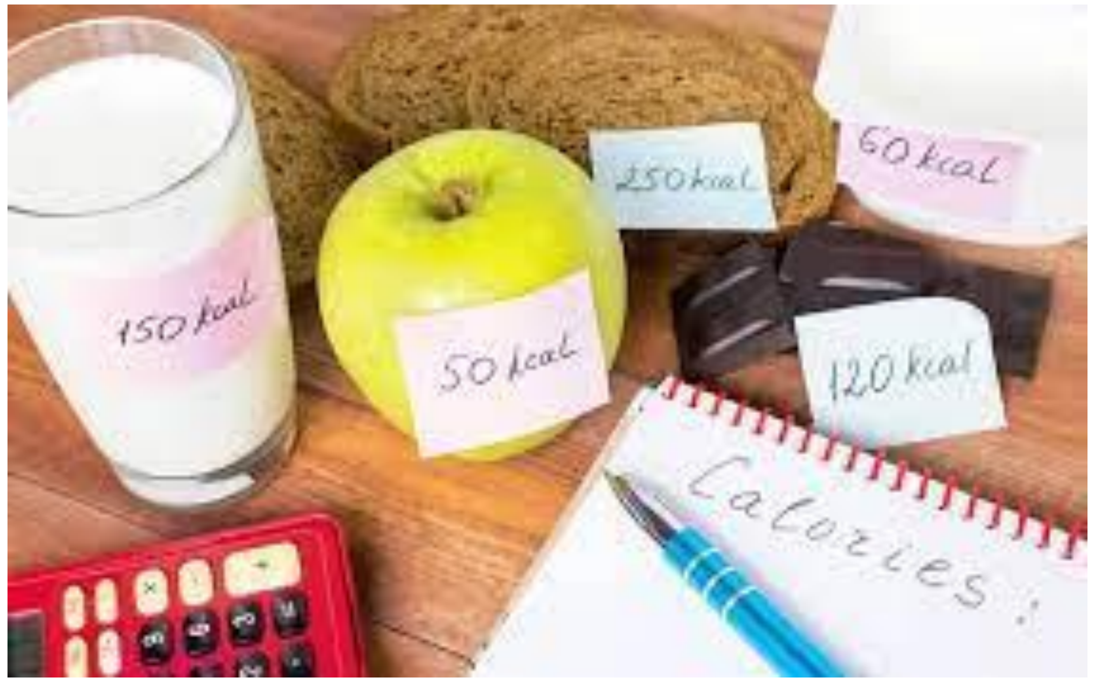

What food from last week was the highest in calories?

What food from last week was the highest in fat?

Were they the same food?

3 WAYS to EAT FEWER CALORIES

1. Choose your moments: limit high-calorie foods to special occasion
2. Eat smaller portions of high-calorie foods, just as a treat
3. Swap lower calorie foods in instead, and save your calories for another time

Check in on our goals

BB-HEALTH

GOAL 1: LOSE WEIGHT

- Aim to lose 1-2 pounds per week
  - Track your foods, budget, and stick to your calorie and fat targets
  - Weight and measure foods, learn to eyeball portions
- Weigh Everyday
  - Write your weight today on the top of your weekly record

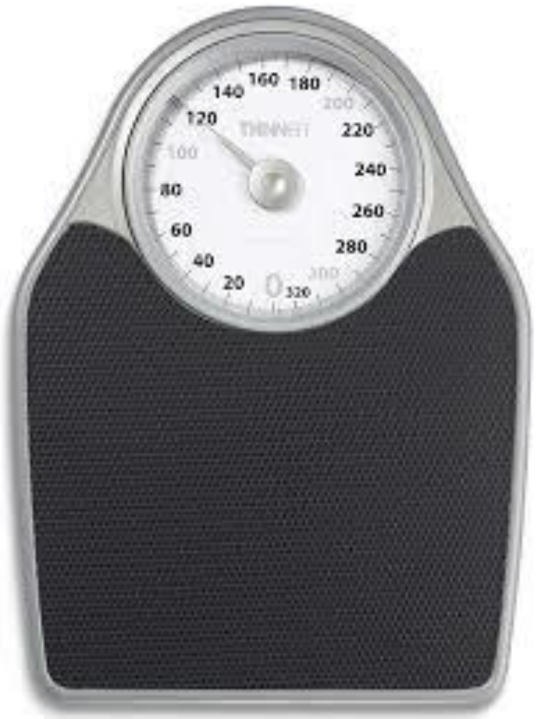

GOAL 2: GET ACTIVE

- Get your 6,000 steps in, 100 min a week on average
- Schedule brisk walks with friends or group members
- Track your exercise and keep your Fitbit charged!

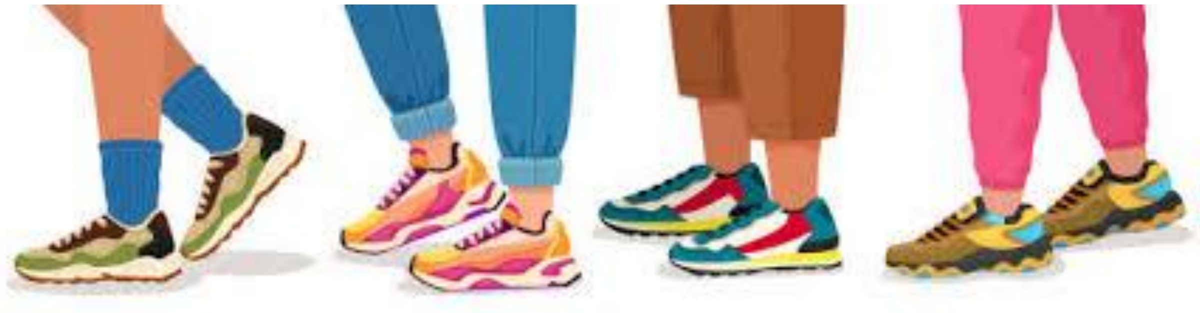

Put it into Practice

Keeping Track of What You Eat

1. Time of day for the things you ate (including snacks!)
   - Are you blowing your budget before lunch?

2. The amount
   - Could smaller portions or lower-fat swaps leave more for dinner?

3. Compare good to bad days, and reflect on the week
   - What made good calorie days work?
   - Was your weekly average on target?
   - What meals need creative solutions→ check your Intervention team notes this week for inspiration!

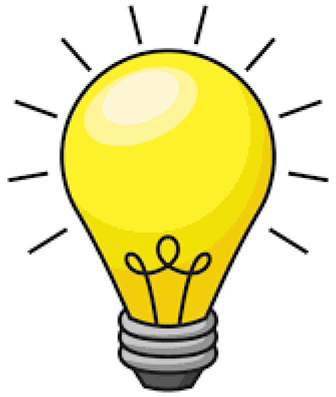

THIS WEEK'S TIP: ***BUDGET THOSE CALORIES!***
Pacing yourself will mean you don't get too hungry during the day.
Set yourself up for success with low calorie snacks like apples or carrots!

Questions?

For next week:

1. Track your food, keep an eye on those grams of fat
2. Weigh every day
3. Make 2,000 steps of your 5,000 steps brisk walking
4. Email your food logs and progress report to the Intervention team and review the response!

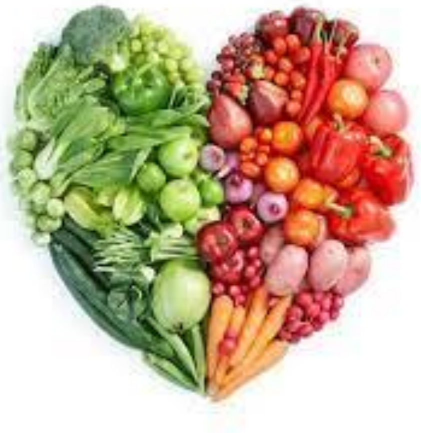

FOOD FOR THOUGHT

- Build on successes, no one is perfect
- Focus on what is working, discuss what isn’t there’s a creative solution for everything
