# Supplementary material for: Development and Feasibility of an eHealth Diabetes Prevention Program Adapted for Older Adults—Results from a Randomized Control Pilot Study
Source: Nutrients. 2024 Mar 23;16(7):930. doi: 10.3390/nu16070930 (PMC11154527; doi:10.3390/nu16070930)
Supplement: Supplementary file 1 [file nutrients-16-00930-s001.zip › Week3.pptx]

## Slide 1
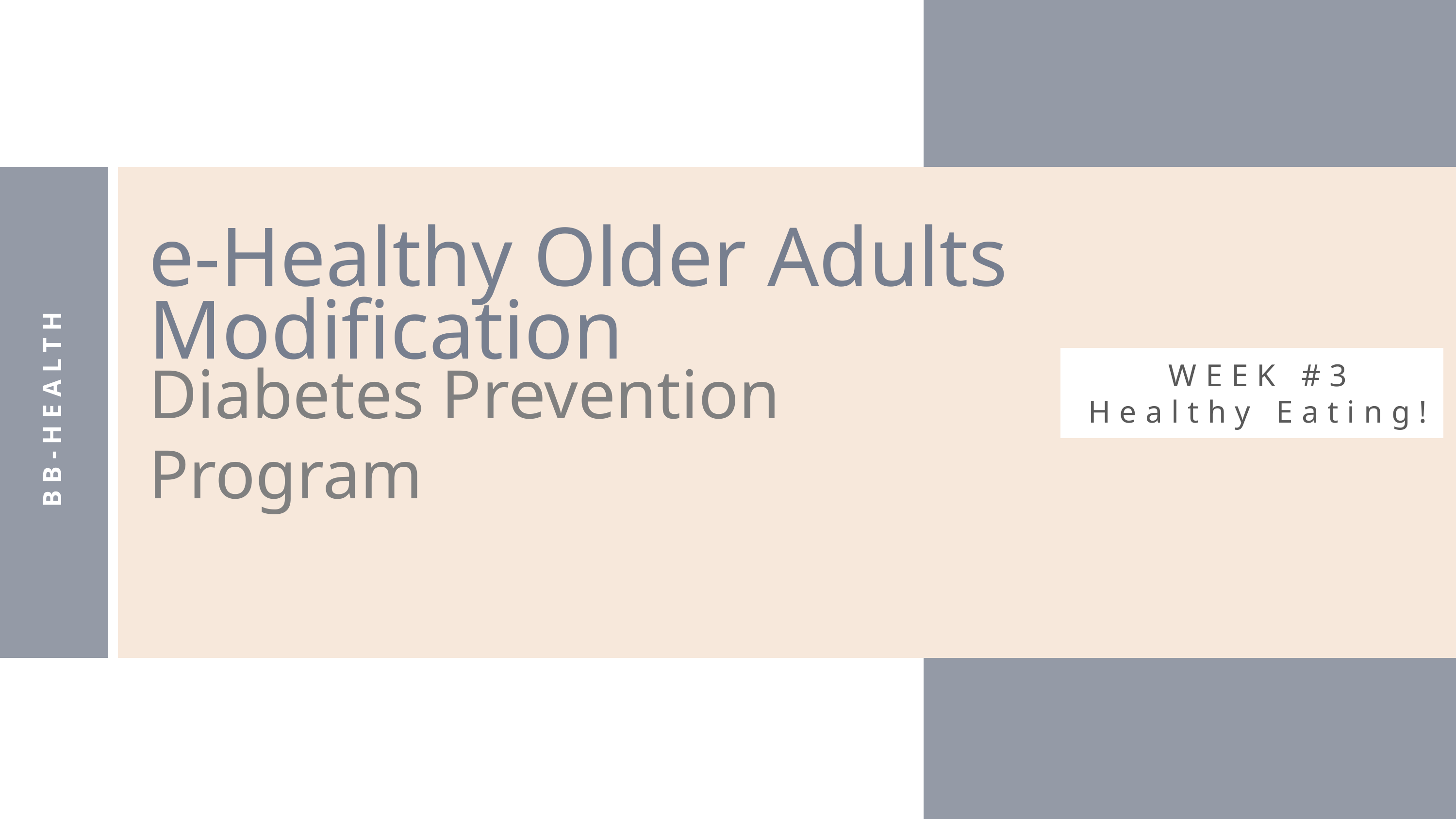

OPEN REPORTS
e-Healthy Older Adults Modification
WEEK #3
Healthy Eating!
Diabetes Prevention Program
BB-HEALTH

## Slide 2
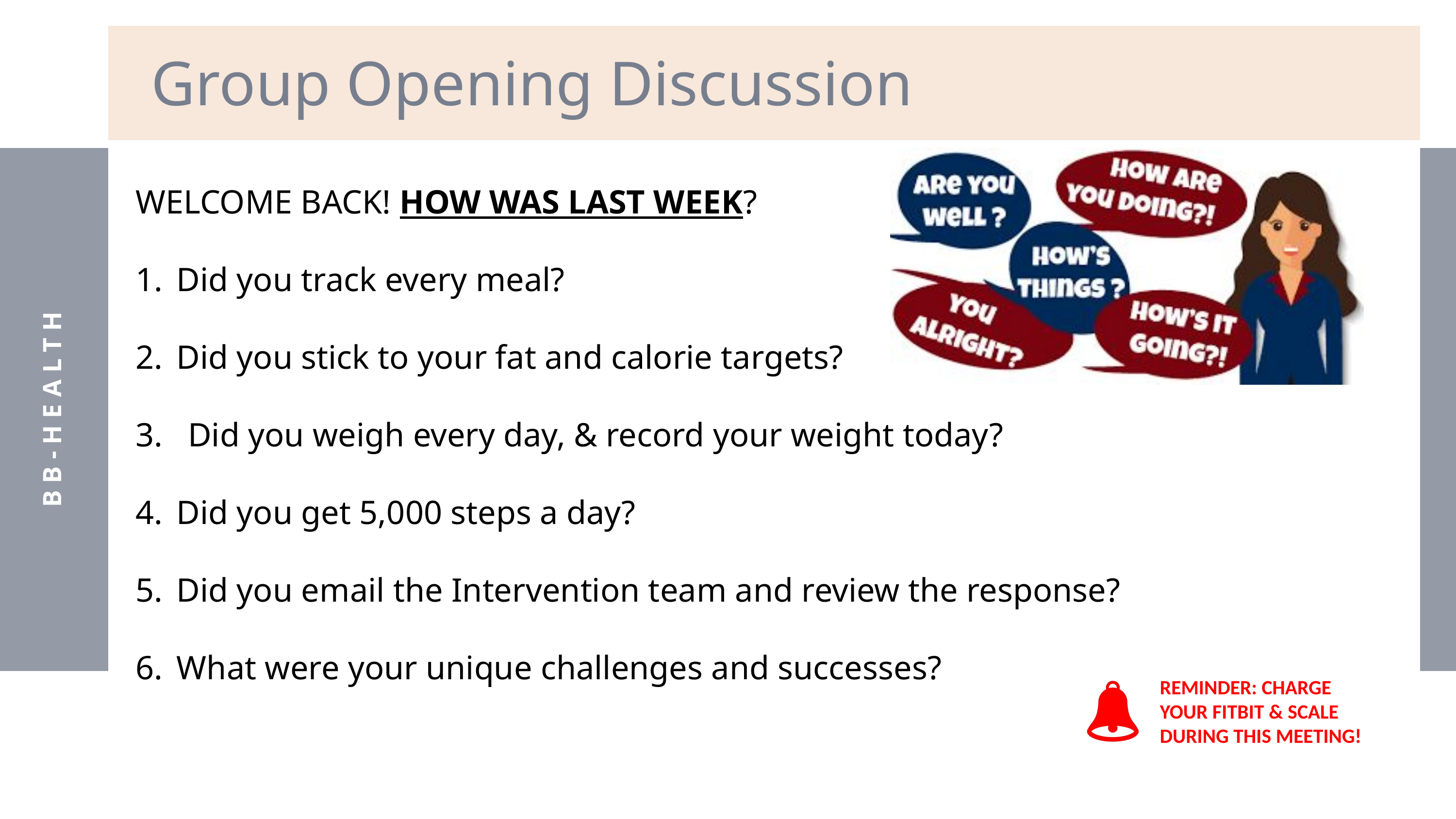

Group Opening Discussion
WELCOME BACK! HOW WAS LAST WEEK?
Did you track every meal?
Did you stick to your fat and calorie targets?
3. Did you weigh every day, & record your weight today?
Did you get 5,000 steps a day?
Did you email the Intervention team and review the response?
What were your unique challenges and successes?
BB-HEALTH
REMINDER: CHARGE YOUR FITBIT & SCALE DURING THIS MEETING!

## Slide 3
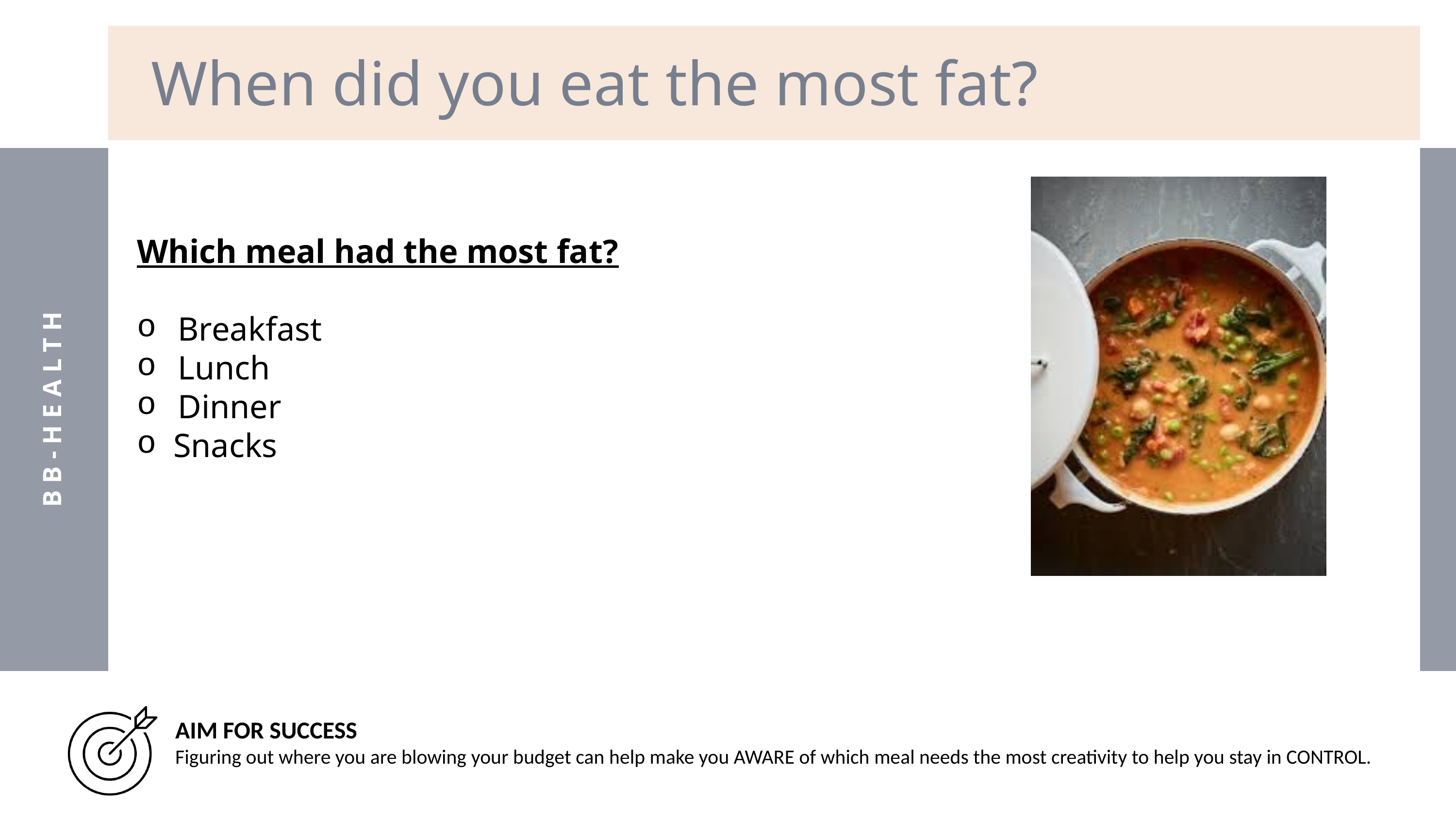

When did you eat the most fat?
Which meal had the most fat?
Breakfast
Lunch
Dinner
Snacks
BB-HEALTH
AIM FOR SUCCESS
Figuring out where you are blowing your budget can help make you AWARE of which meal needs the most creativity to help you stay in CONTROL.

## Slide 4
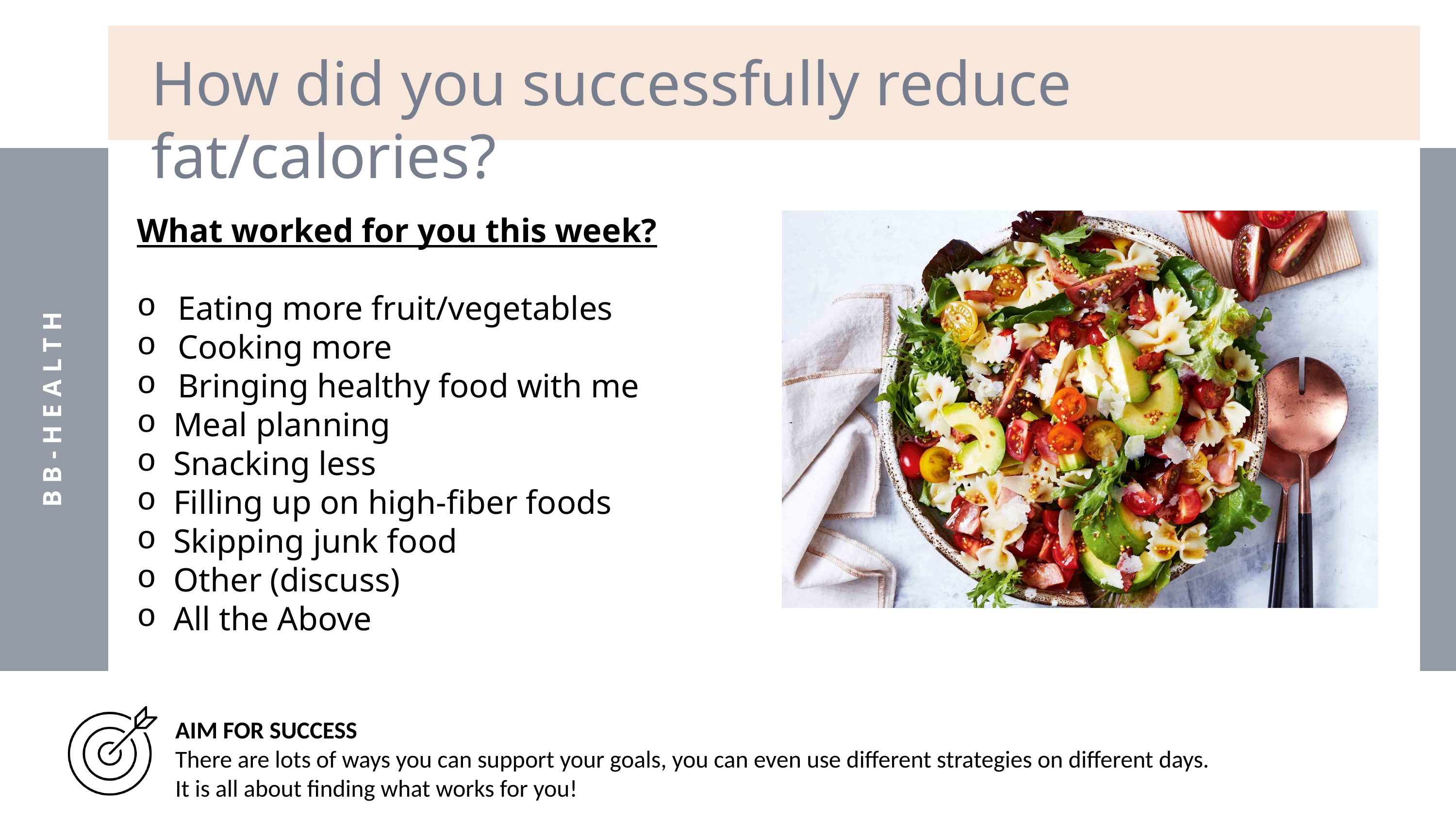

How did you successfully reduce fat/calories?
What worked for you this week?
Eating more fruit/vegetables
Cooking more
Bringing healthy food with me
Meal planning
Snacking less
Filling up on high-fiber foods
Skipping junk food
Other (discuss)
All the Above
BB-HEALTH
AIM FOR SUCCESS
There are lots of ways you can support your goals, you can even use different strategies on different days.
It is all about finding what works for you!

## Slide 5
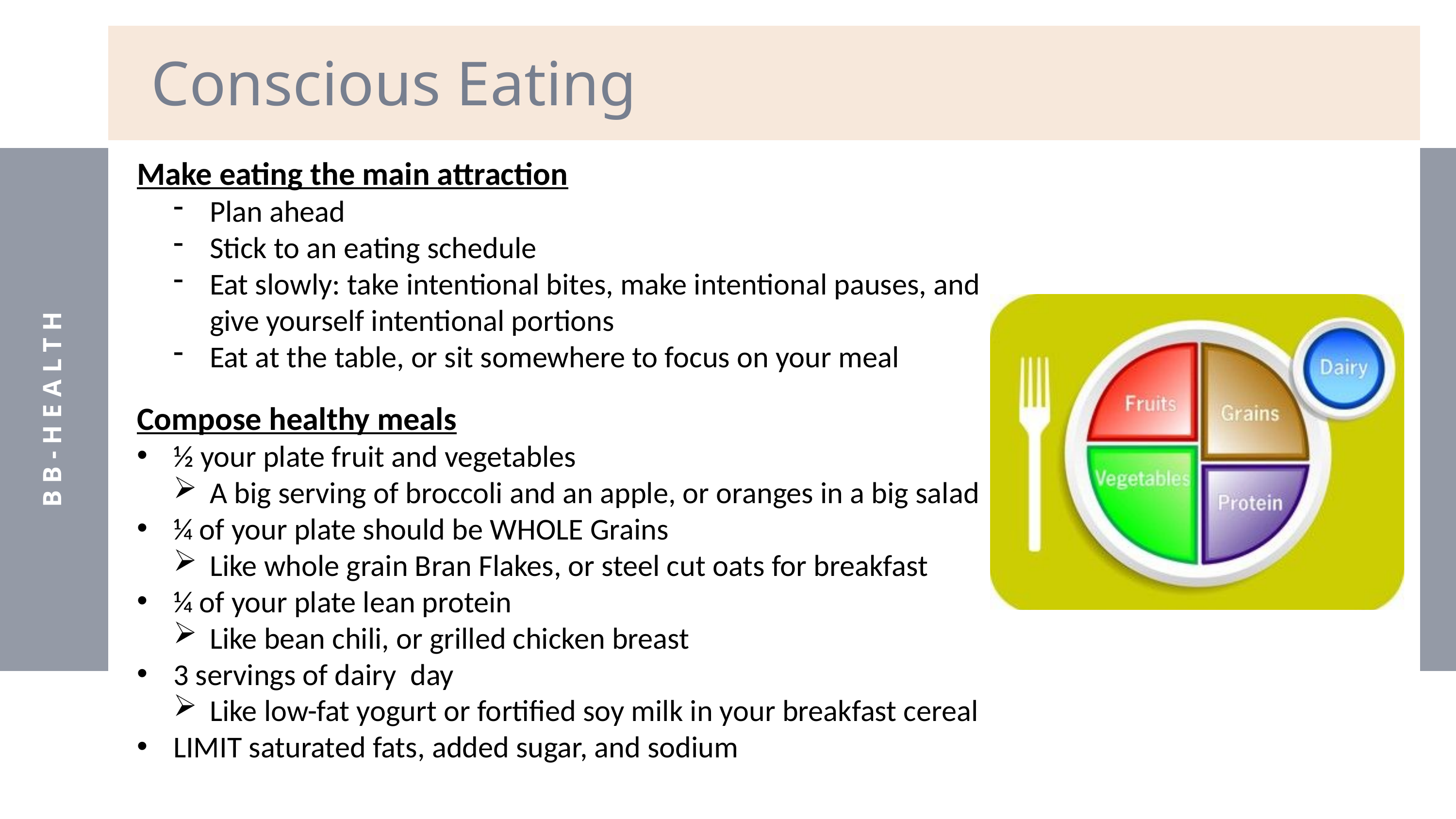

Conscious Eating
Make eating the main attraction
Plan ahead
Stick to an eating schedule
Eat slowly: take intentional bites, make intentional pauses, and give yourself intentional portions
Eat at the table, or sit somewhere to focus on your meal
Compose healthy meals
½ your plate fruit and vegetables
A big serving of broccoli and an apple, or oranges in a big salad
¼ of your plate should be WHOLE Grains
Like whole grain Bran Flakes, or steel cut oats for breakfast
¼ of your plate lean protein
Like bean chili, or grilled chicken breast
3 servings of dairy day
Like low-fat yogurt or fortified soy milk in your breakfast cereal
LIMIT saturated fats, added sugar, and sodium
BB-HEALTH

## Slide 6
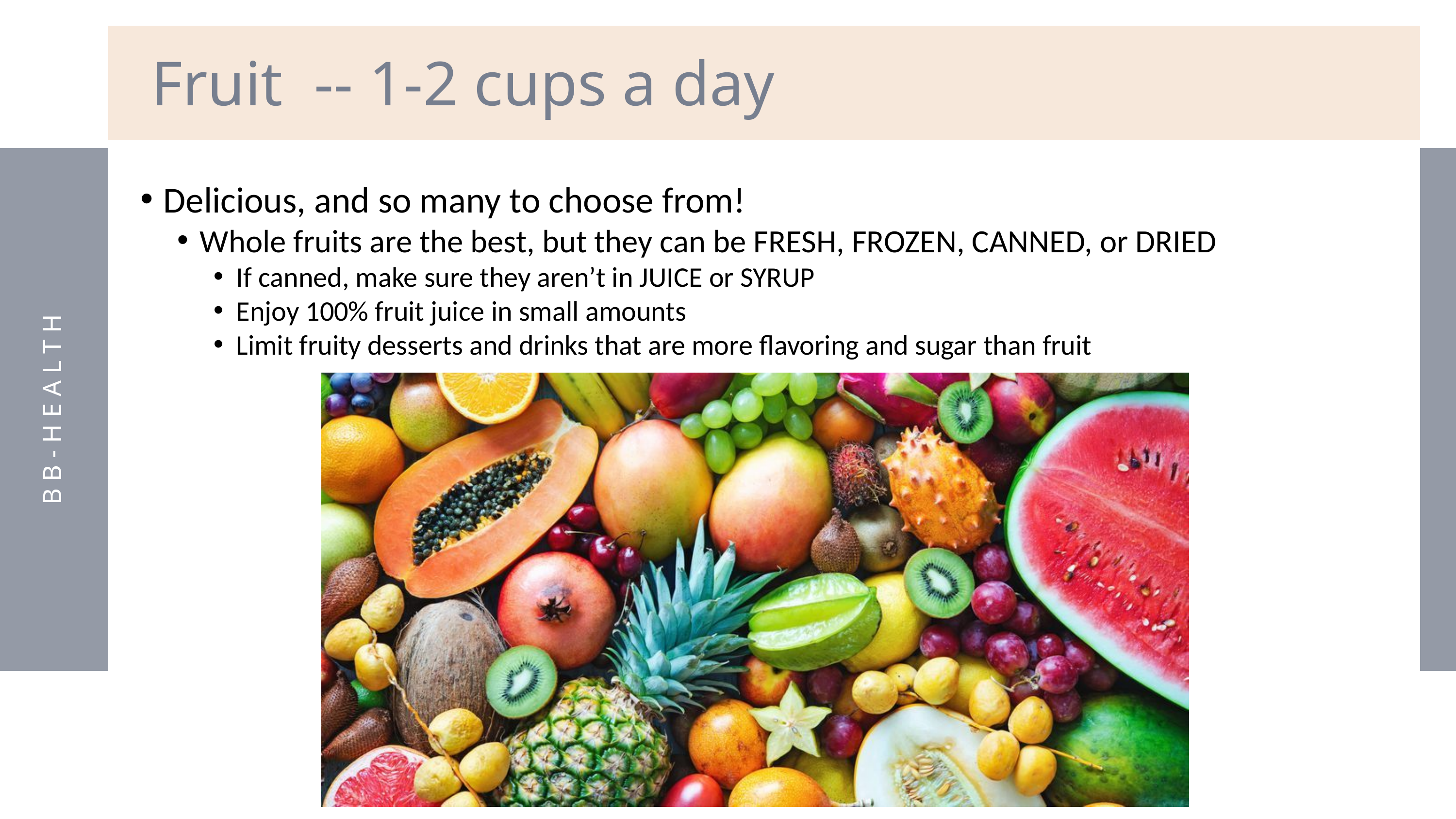

Fruit -- 1-2 cups a day
Delicious, and so many to choose from!
Whole fruits are the best, but they can be FRESH, FROZEN, CANNED, or DRIED
If canned, make sure they aren’t in JUICE or SYRUP
Enjoy 100% fruit juice in small amounts
Limit fruity desserts and drinks that are more flavoring and sugar than fruit
BB-HEALTH

## Slide 7
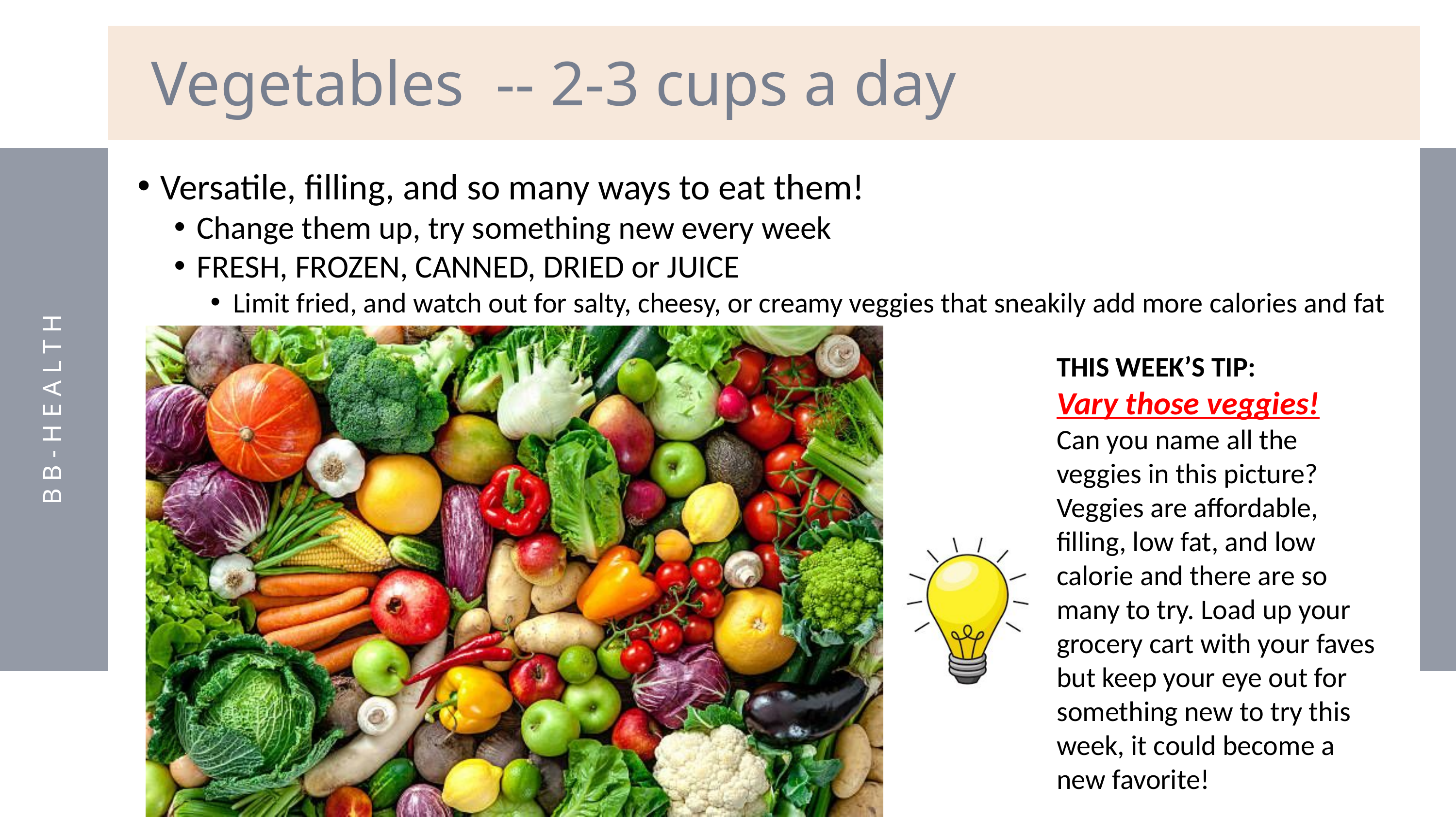

Vegetables -- 2-3 cups a day
Versatile, filling, and so many ways to eat them!
Change them up, try something new every week
FRESH, FROZEN, CANNED, DRIED or JUICE
Limit fried, and watch out for salty, cheesy, or creamy veggies that sneakily add more calories and fat
THIS WEEK’S TIP:
Vary those veggies!
Can you name all the veggies in this picture?
Veggies are affordable, filling, low fat, and low calorie and there are so many to try. Load up your grocery cart with your faves but keep your eye out for something new to try this week, it could become a new favorite!
BB-HEALTH

## Slide 8
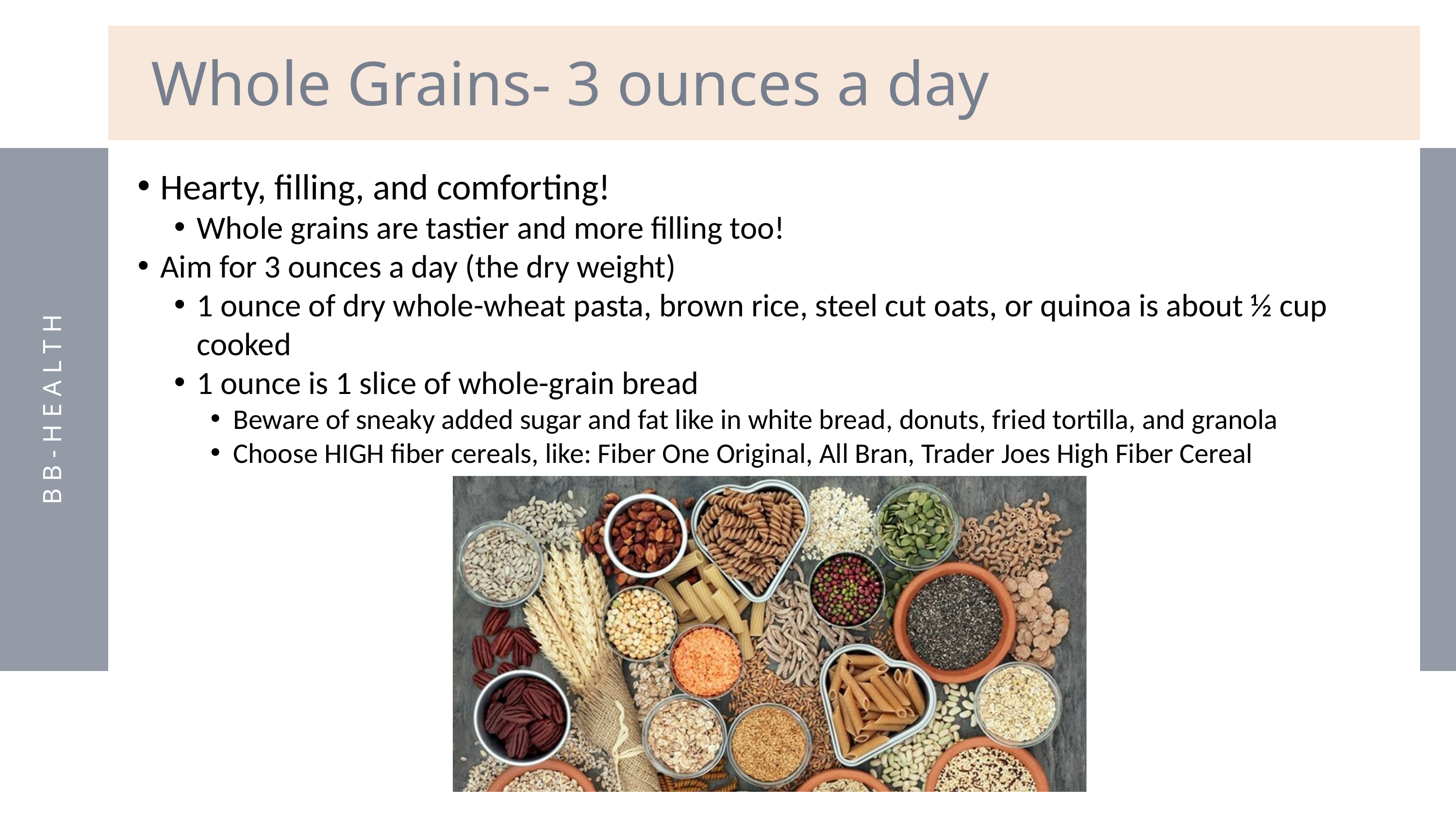

Whole Grains- 3 ounces a day
Hearty, filling, and comforting!
Whole grains are tastier and more filling too!
Aim for 3 ounces a day (the dry weight)
1 ounce of dry whole-wheat pasta, brown rice, steel cut oats, or quinoa is about ½ cup cooked
1 ounce is 1 slice of whole-grain bread
Beware of sneaky added sugar and fat like in white bread, donuts, fried tortilla, and granola
Choose HIGH fiber cereals, like: Fiber One Original, All Bran, Trader Joes High Fiber Cereal
BB-HEALTH

## Slide 9
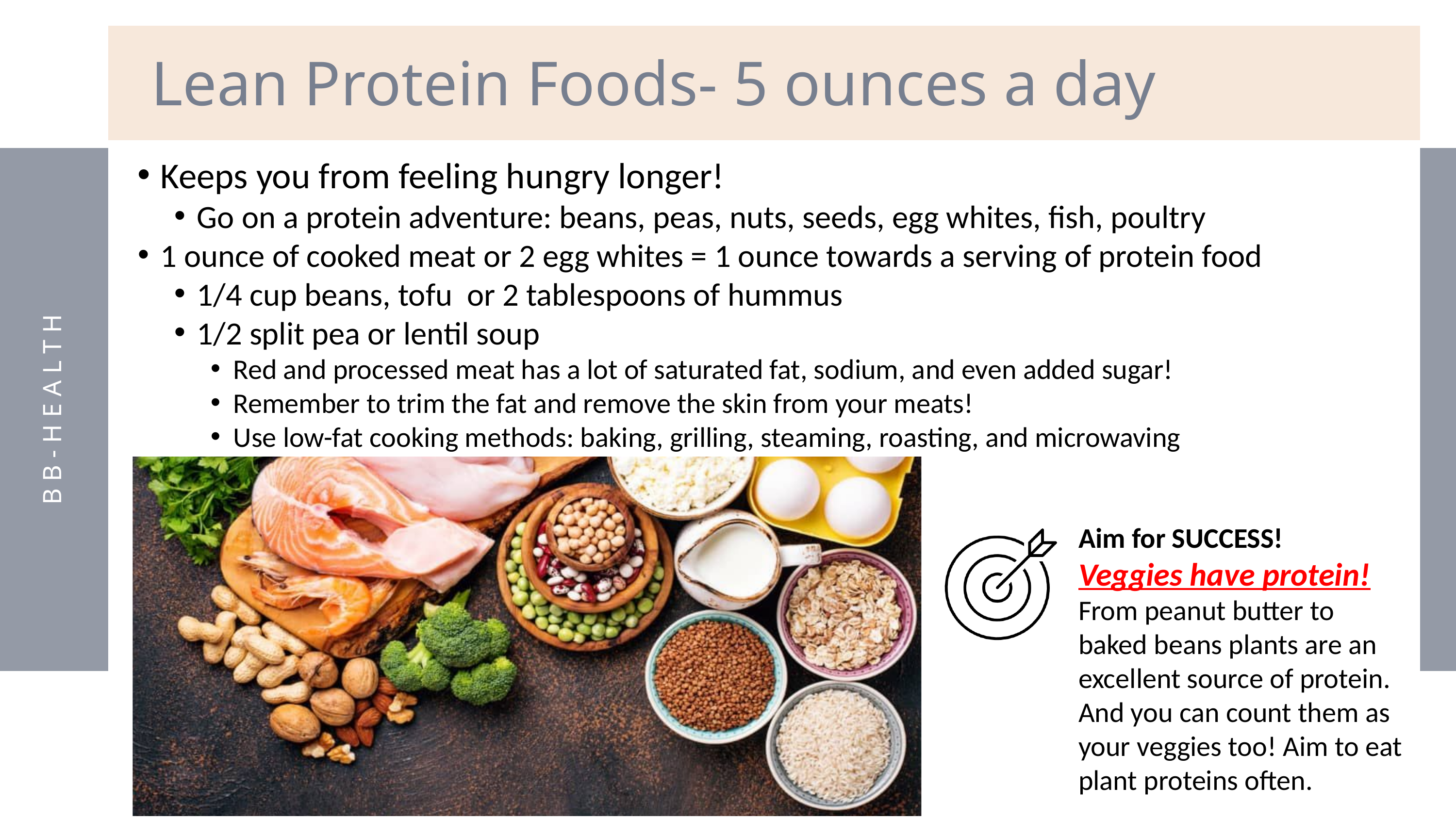

Lean Protein Foods- 5 ounces a day
Keeps you from feeling hungry longer!
Go on a protein adventure: beans, peas, nuts, seeds, egg whites, fish, poultry
1 ounce of cooked meat or 2 egg whites = 1 ounce towards a serving of protein food
1/4 cup beans, tofu or 2 tablespoons of hummus
1/2 split pea or lentil soup
Red and processed meat has a lot of saturated fat, sodium, and even added sugar!
Remember to trim the fat and remove the skin from your meats!
Use low-fat cooking methods: baking, grilling, steaming, roasting, and microwaving
BB-HEALTH
Aim for SUCCESS!
Veggies have protein!
From peanut butter to baked beans plants are an excellent source of protein. And you can count them as your veggies too! Aim to eat plant proteins often.

## Slide 10
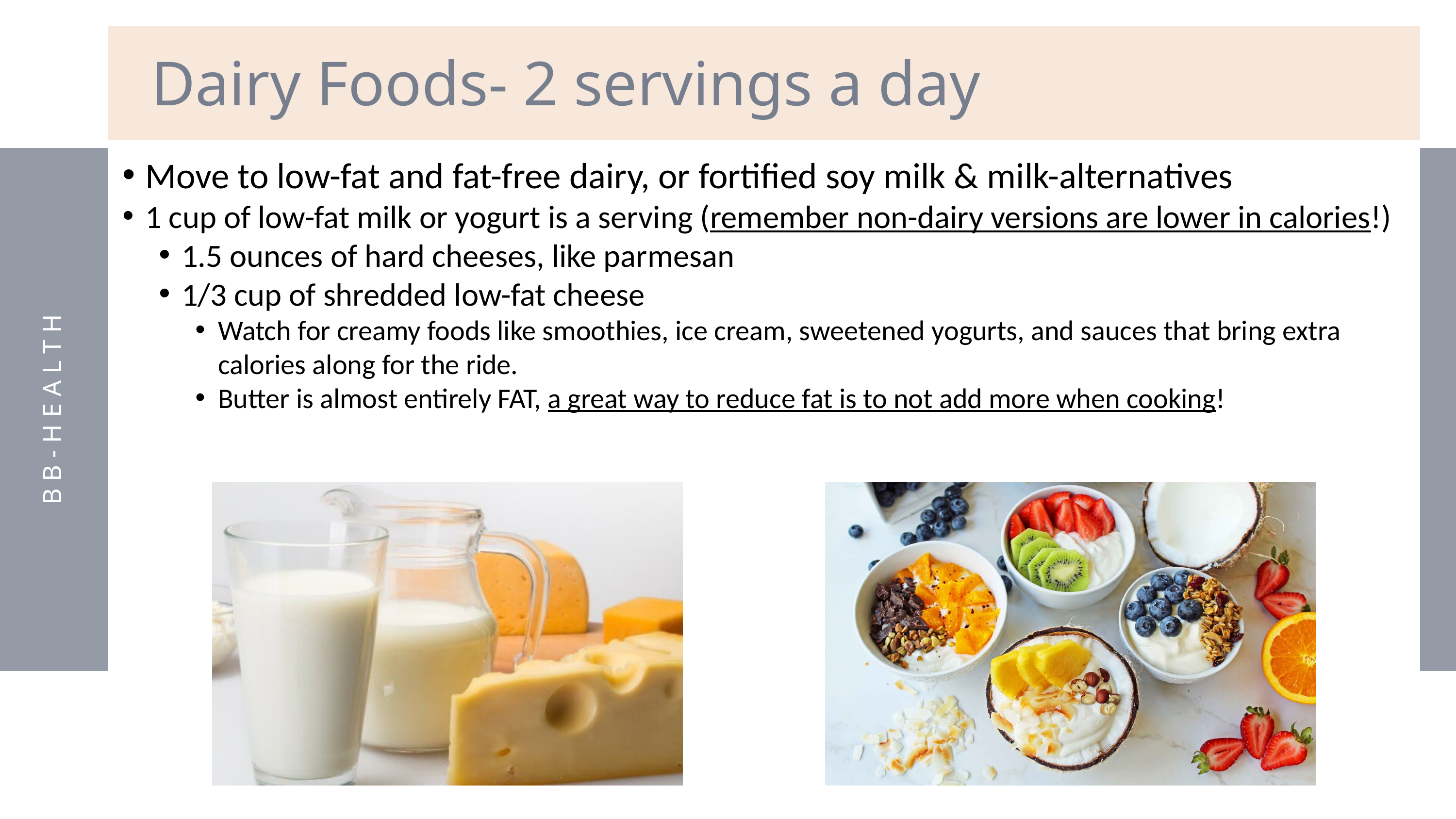

Dairy Foods- 2 servings a day
Move to low-fat and fat-free dairy, or fortified soy milk & milk-alternatives
1 cup of low-fat milk or yogurt is a serving (remember non-dairy versions are lower in calories!)
1.5 ounces of hard cheeses, like parmesan
1/3 cup of shredded low-fat cheese
Watch for creamy foods like smoothies, ice cream, sweetened yogurts, and sauces that bring extra calories along for the ride.
Butter is almost entirely FAT, a great way to reduce fat is to not add more when cooking!
BB-HEALTH

## Slide 11
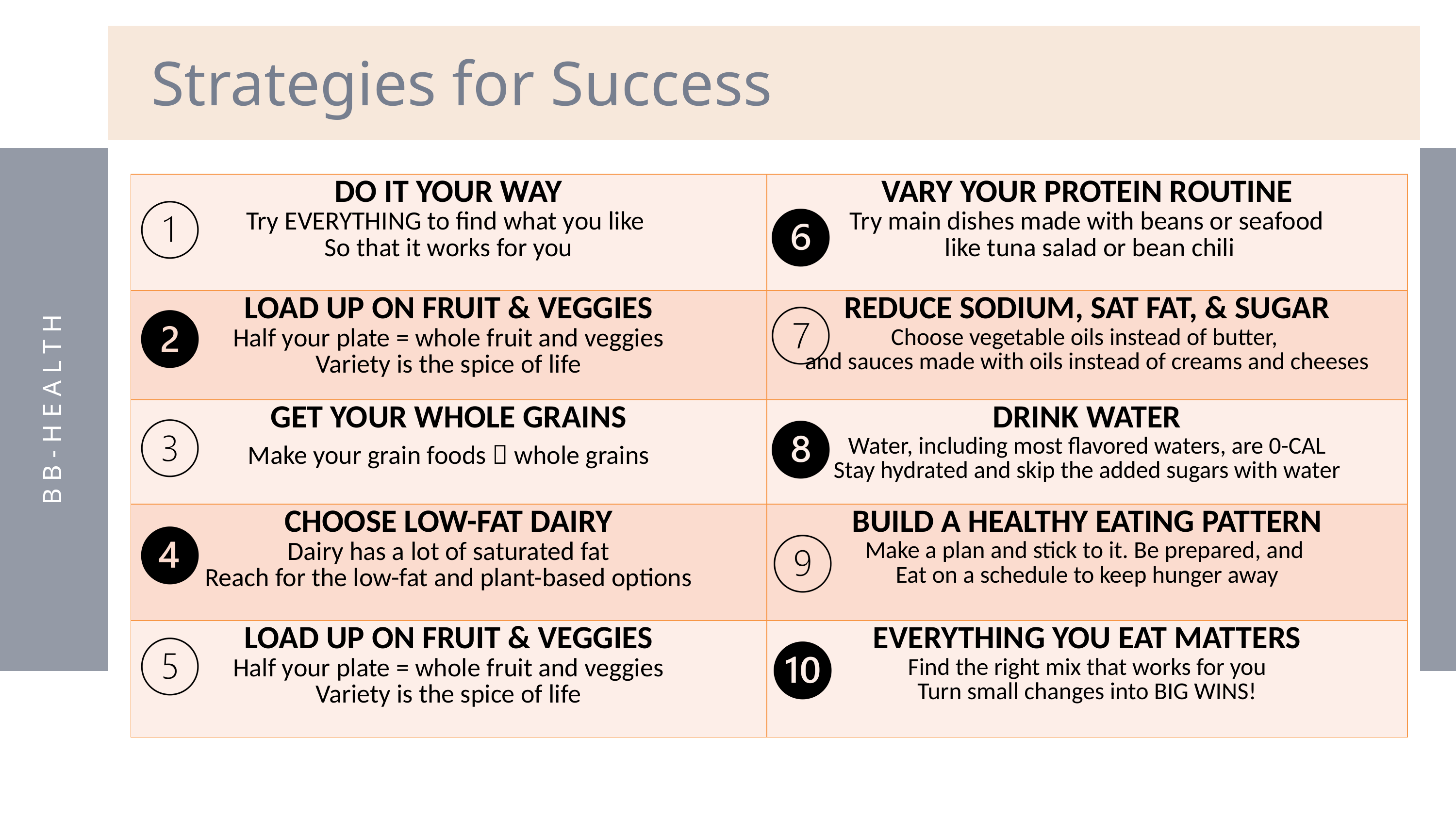

Strategies for Success
| DO IT YOUR WAY Try EVERYTHING to find what you like So that it works for you | VARY YOUR PROTEIN ROUTINE Try main dishes made with beans or seafood like tuna salad or bean chili |
| --- | --- |
| LOAD UP ON FRUIT & VEGGIES Half your plate = whole fruit and veggies Variety is the spice of life | REDUCE SODIUM, SAT FAT, & SUGAR Choose vegetable oils instead of butter, and sauces made with oils instead of creams and cheeses |
| GET YOUR WHOLE GRAINS Make your grain foods  whole grains | DRINK WATER Water, including most flavored waters, are 0-CAL Stay hydrated and skip the added sugars with water |
| CHOOSE LOW-FAT DAIRY Dairy has a lot of saturated fat Reach for the low-fat and plant-based options | BUILD A HEALTHY EATING PATTERN Make a plan and stick to it. Be prepared, and Eat on a schedule to keep hunger away |
| LOAD UP ON FRUIT & VEGGIES Half your plate = whole fruit and veggies Variety is the spice of life | EVERYTHING YOU EAT MATTERS Find the right mix that works for you Turn small changes into BIG WINS! |
BB-HEALTH

## Slide 12
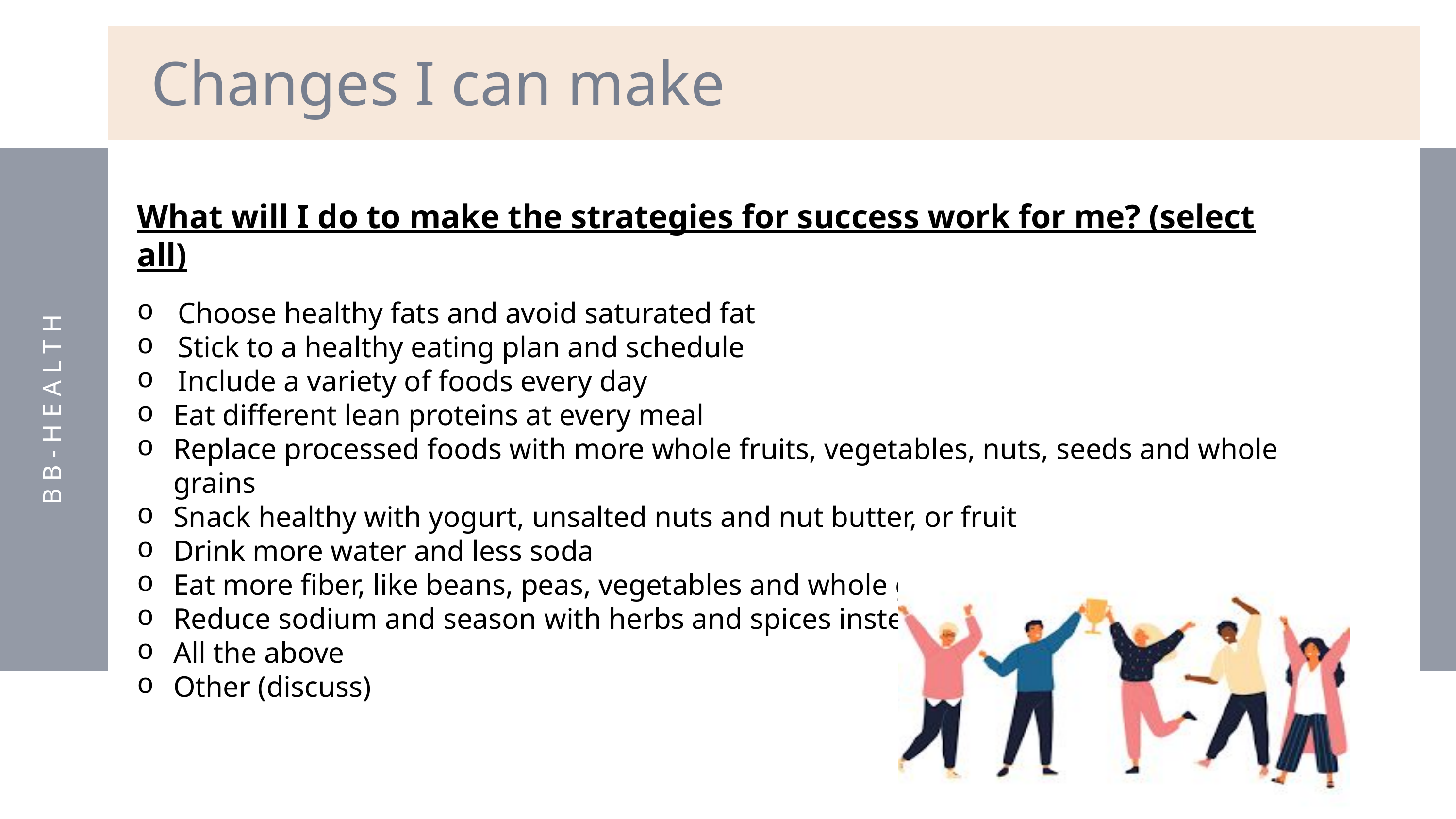

Changes I can make
What will I do to make the strategies for success work for me? (select all)
Choose healthy fats and avoid saturated fat
Stick to a healthy eating plan and schedule
Include a variety of foods every day
Eat different lean proteins at every meal
Replace processed foods with more whole fruits, vegetables, nuts, seeds and whole grains
Snack healthy with yogurt, unsalted nuts and nut butter, or fruit
Drink more water and less soda
Eat more fiber, like beans, peas, vegetables and whole grain cereal
Reduce sodium and season with herbs and spices instead of salt
All the above
Other (discuss)
BB-HEALTH

## Slide 13
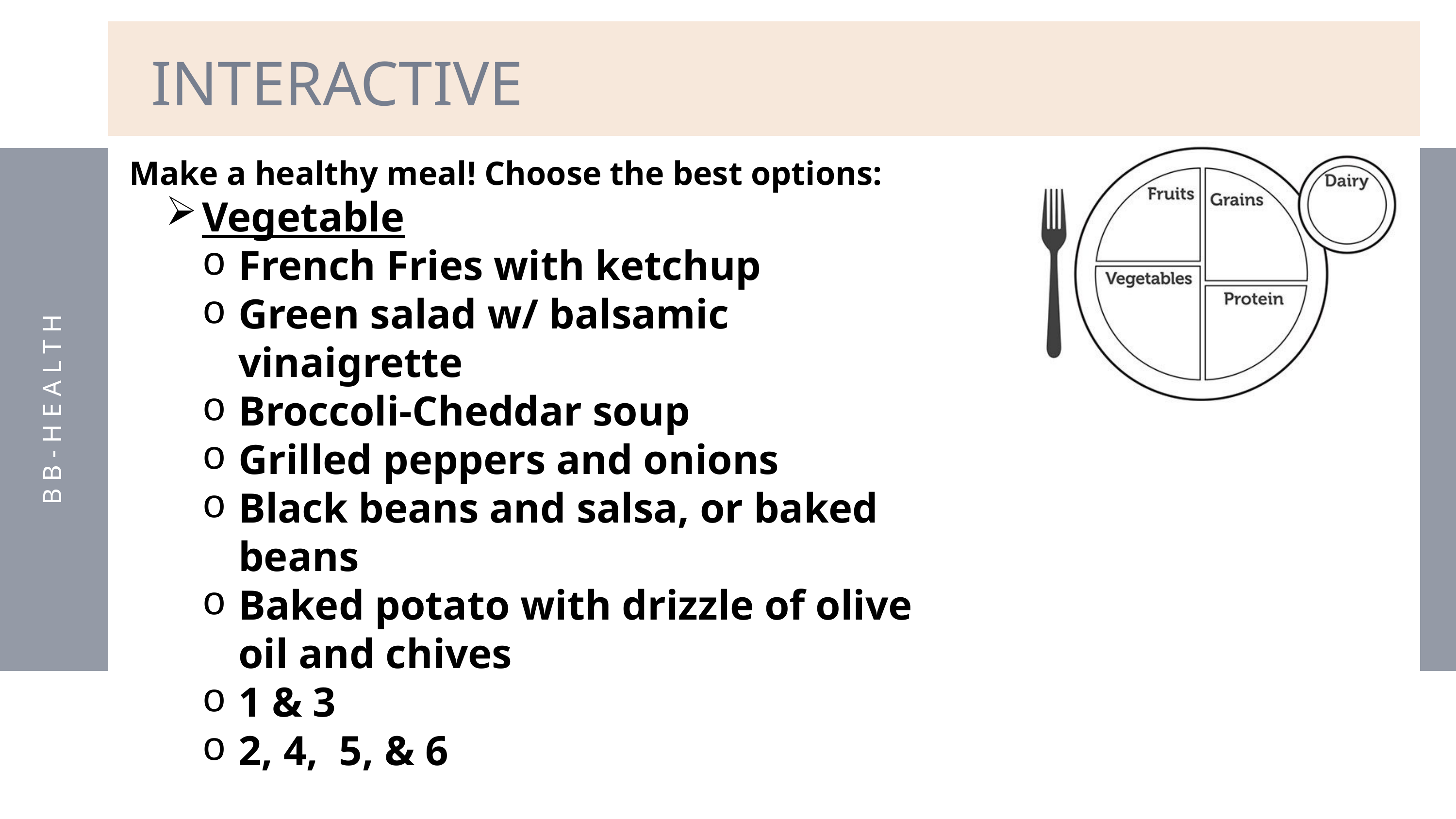

INTERACTIVE
Make a healthy meal! Choose the best options:
Vegetable
French Fries with ketchup
Green salad w/ balsamic vinaigrette
Broccoli-Cheddar soup
Grilled peppers and onions
Black beans and salsa, or baked beans
Baked potato with drizzle of olive oil and chives
1 & 3
2, 4, 5, & 6
BB-HEALTH

## Slide 14
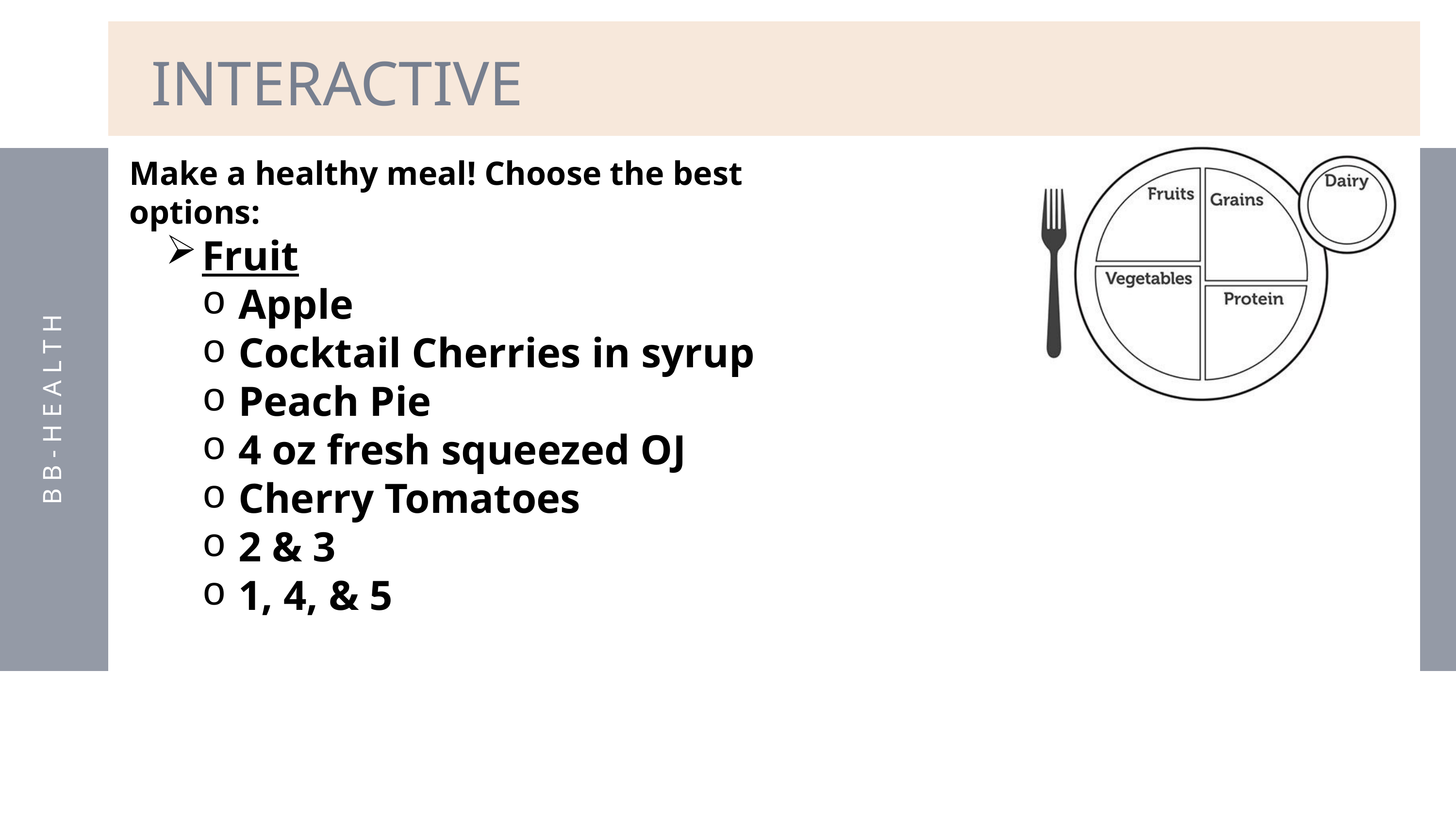

INTERACTIVE
Make a healthy meal! Choose the best options:
Fruit
Apple
Cocktail Cherries in syrup
Peach Pie
4 oz fresh squeezed OJ
Cherry Tomatoes
2 & 3
1, 4, & 5
BB-HEALTH

## Slide 15
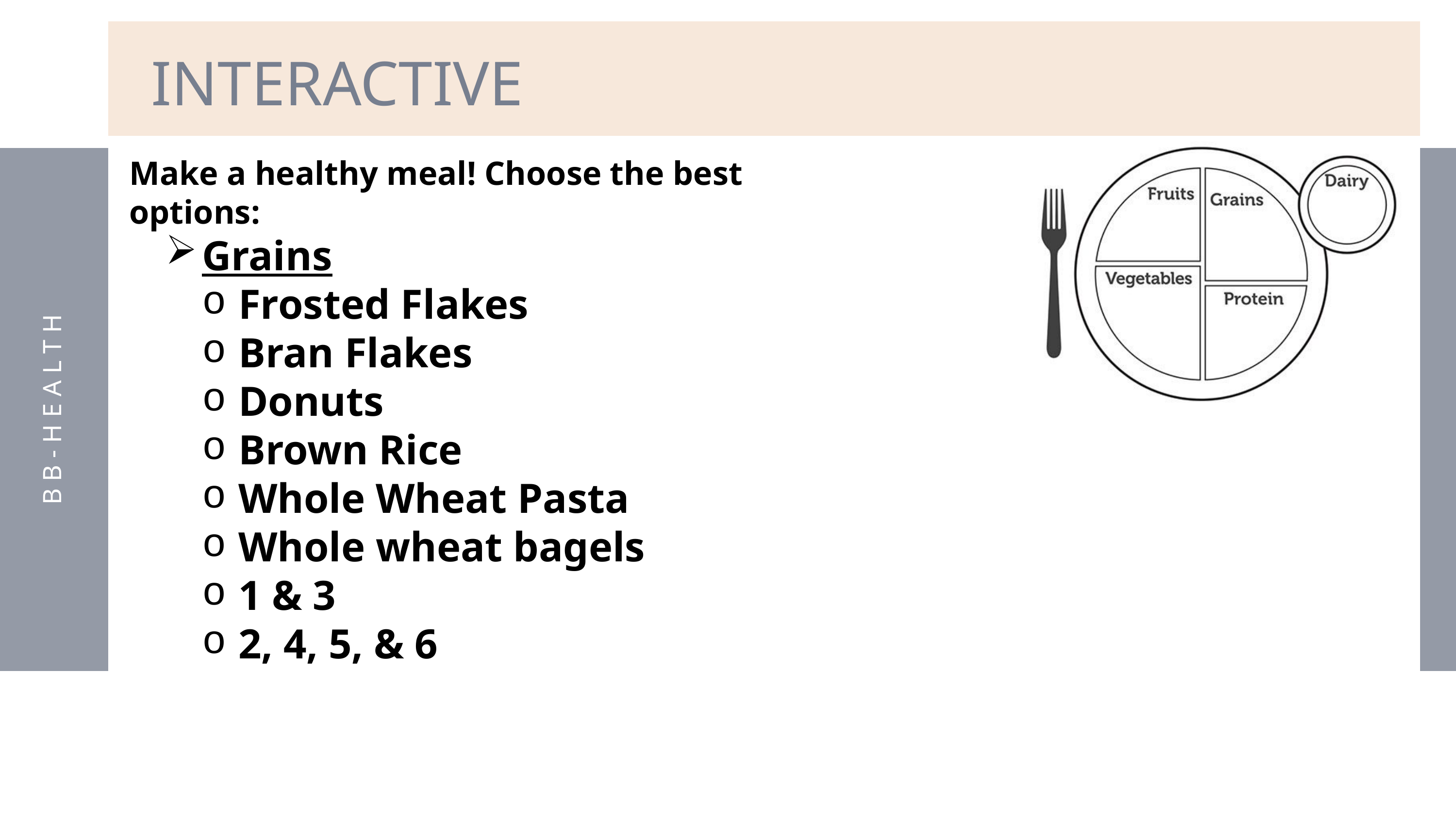

INTERACTIVE
Make a healthy meal! Choose the best options:
Grains
Frosted Flakes
Bran Flakes
Donuts
Brown Rice
Whole Wheat Pasta
Whole wheat bagels
1 & 3
2, 4, 5, & 6
BB-HEALTH

## Slide 16
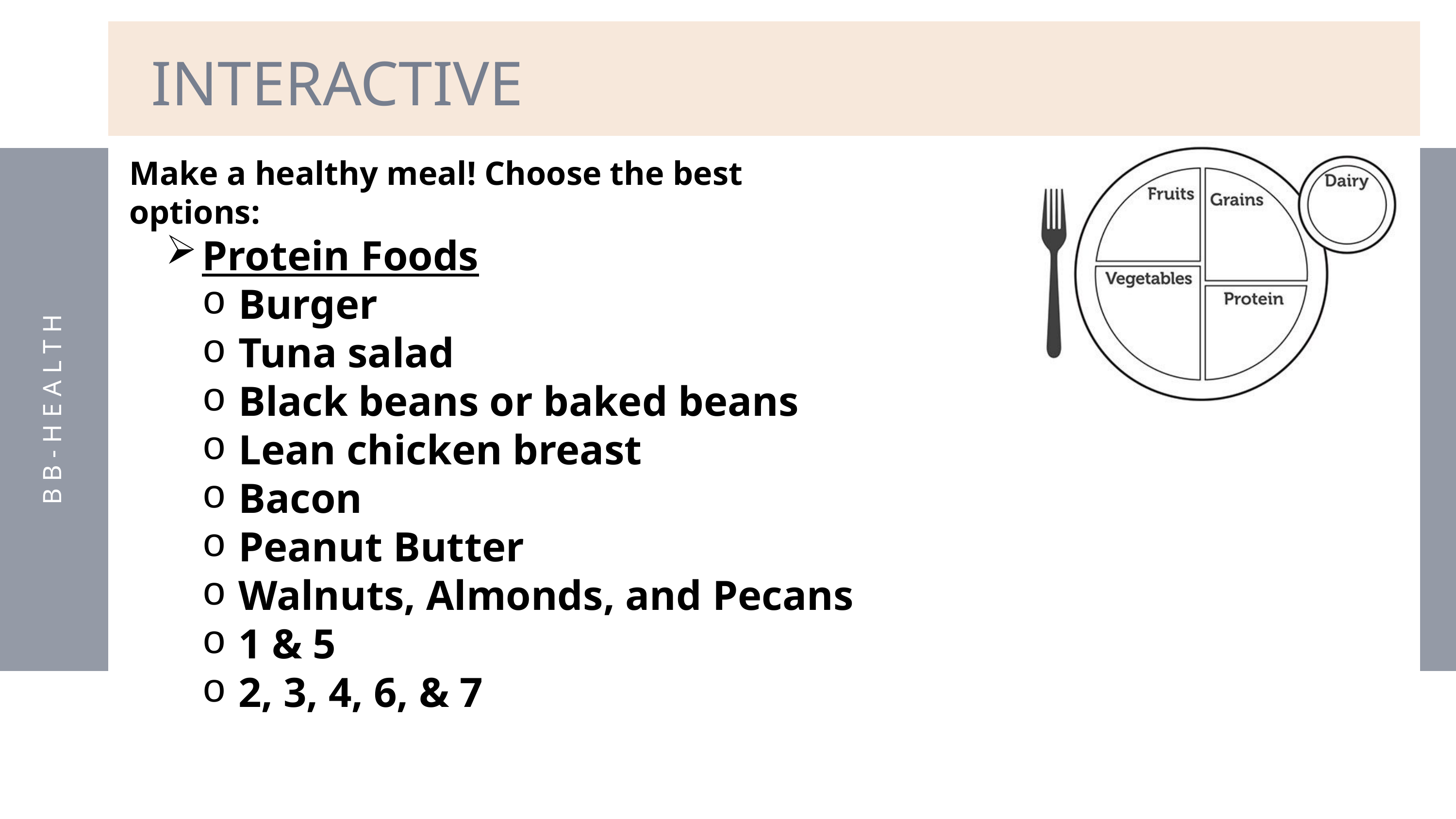

INTERACTIVE
Make a healthy meal! Choose the best options:
Protein Foods
Burger
Tuna salad
Black beans or baked beans
Lean chicken breast
Bacon
Peanut Butter
Walnuts, Almonds, and Pecans
1 & 5
2, 3, 4, 6, & 7
BB-HEALTH

## Slide 17
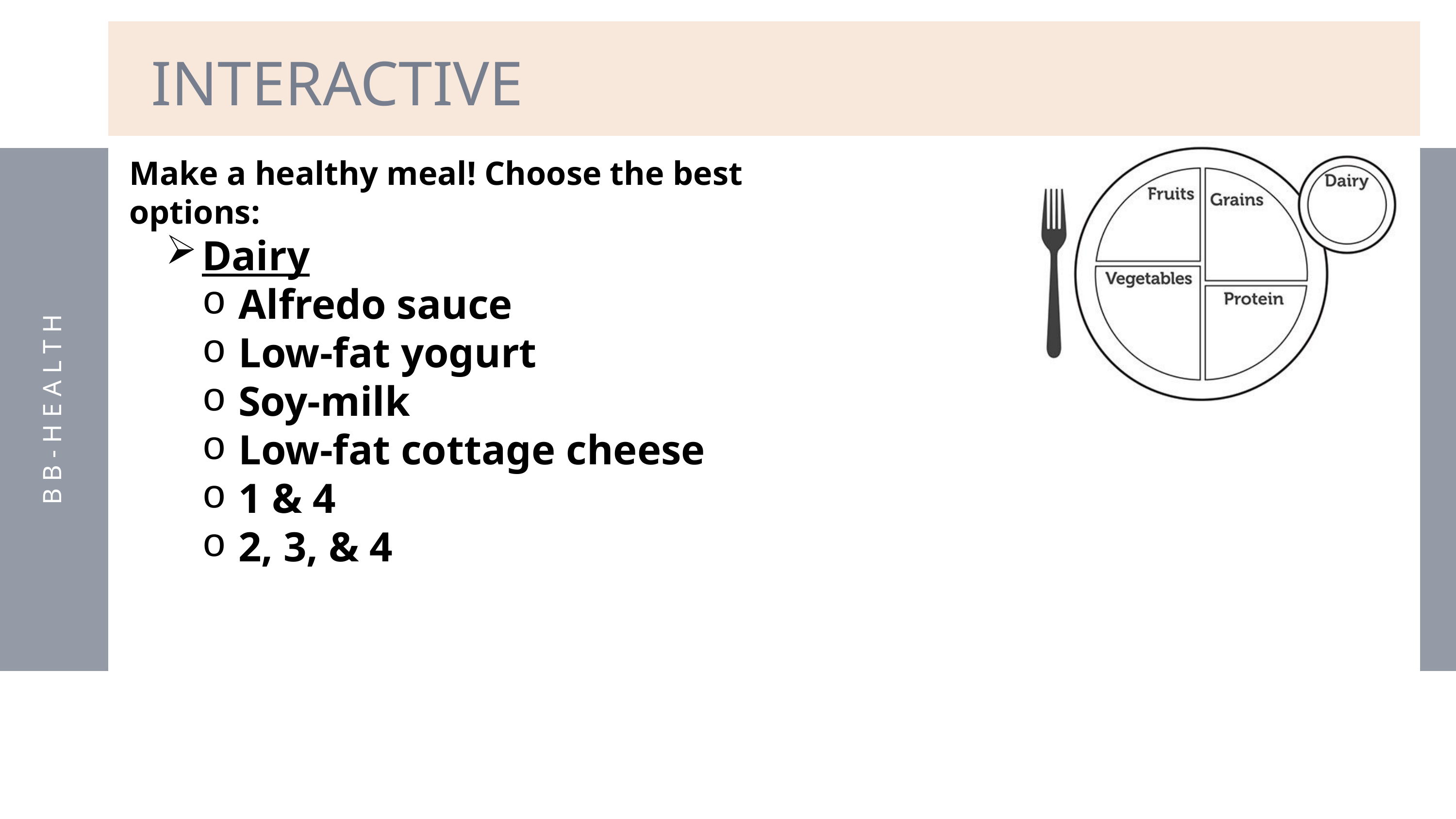

INTERACTIVE
Make a healthy meal! Choose the best options:
Dairy
Alfredo sauce
Low-fat yogurt
Soy-milk
Low-fat cottage cheese
1 & 4
2, 3, & 4
BB-HEALTH

## Slide 18
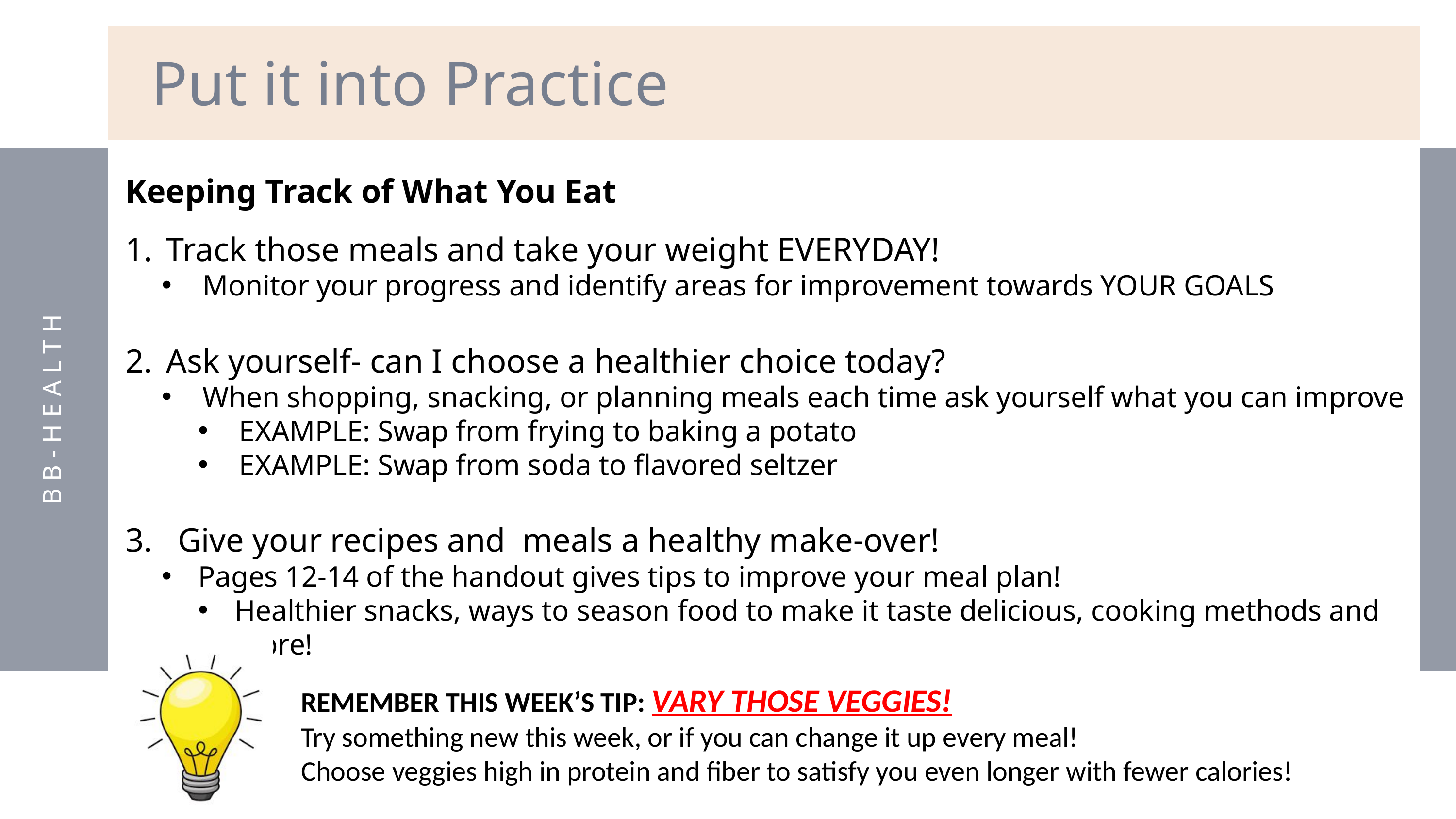

Put it into Practice
Keeping Track of What You Eat
Track those meals and take your weight EVERYDAY!
Monitor your progress and identify areas for improvement towards YOUR GOALS
Ask yourself- can I choose a healthier choice today?
When shopping, snacking, or planning meals each time ask yourself what you can improve
EXAMPLE: Swap from frying to baking a potato
EXAMPLE: Swap from soda to flavored seltzer
3. Give your recipes and meals a healthy make-over!
Pages 12-14 of the handout gives tips to improve your meal plan!
Healthier snacks, ways to season food to make it taste delicious, cooking methods and more!
BB-HEALTH
REMEMBER THIS WEEK’S TIP: VARY THOSE VEGGIES!
Try something new this week, or if you can change it up every meal!
Choose veggies high in protein and fiber to satisfy you even longer with fewer calories!

## Slide 19
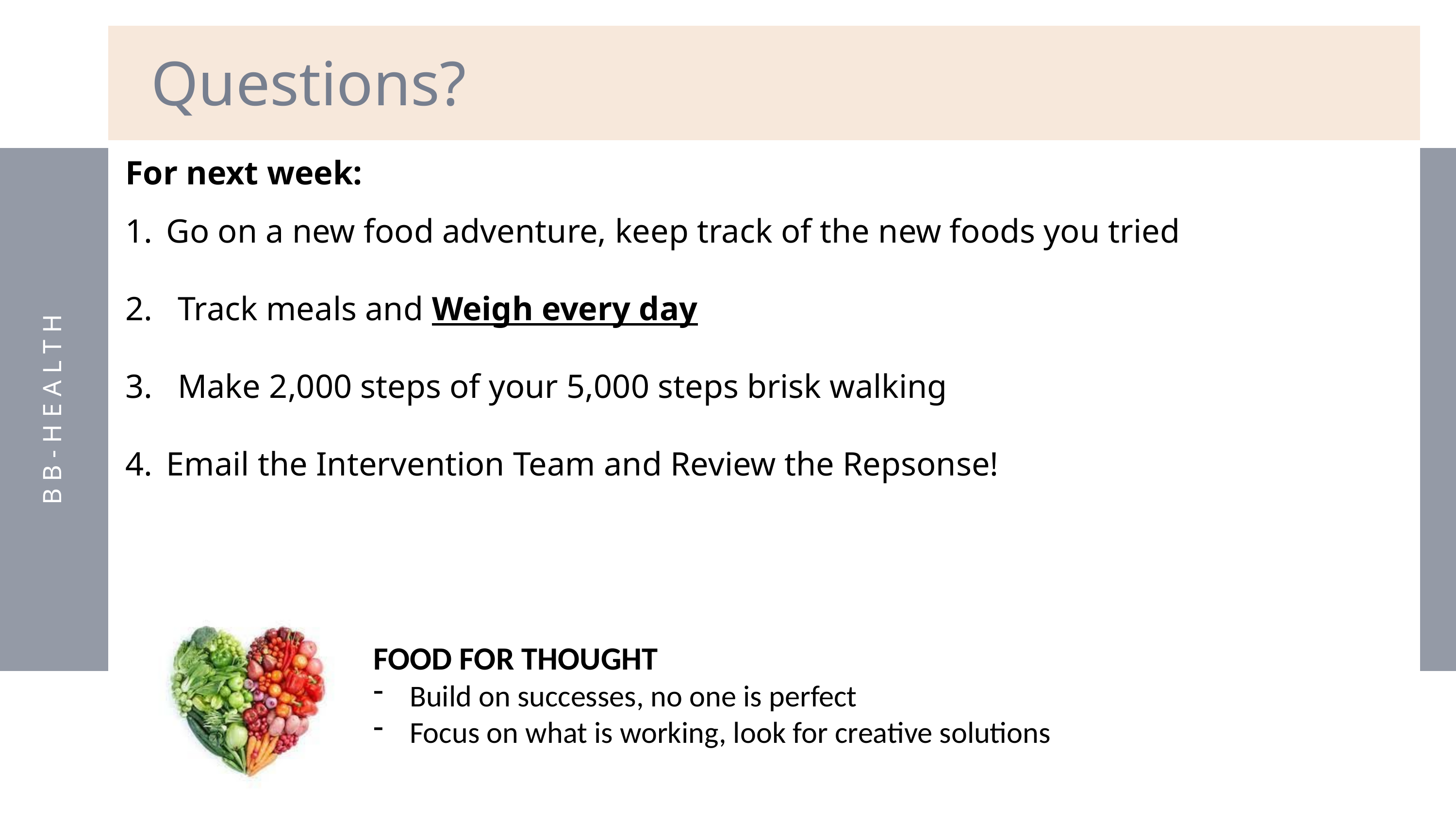

Questions?
For next week:
Go on a new food adventure, keep track of the new foods you tried
2. Track meals and Weigh every day
3. Make 2,000 steps of your 5,000 steps brisk walking
Email the Intervention Team and Review the Repsonse!
BB-HEALTH
FOOD FOR THOUGHT
Build on successes, no one is perfect
Focus on what is working, look for creative solutions
