# Supplementary material for: Development and Feasibility of an eHealth Diabetes Prevention Program Adapted for Older Adults—Results from a Randomized Control Pilot Study
Source: Nutrients. 2024 Mar 23;16(7):930. doi: 10.3390/nu16070930 (PMC11154527; doi:10.3390/nu16070930)
Supplement: Supplementary file 1 [file nutrients-16-00930-s001.zip › Week3_SUPP.pdf]

BB-HEALTH

e-Healthy Older Adults Modification

Diabetes Prevention Program

WEEK #3
Healthy Eating!

Group Opening Discussion

BB-HEALTH

WELCOME BACK! <u>**HOW WAS LAST WEEK**</u>?

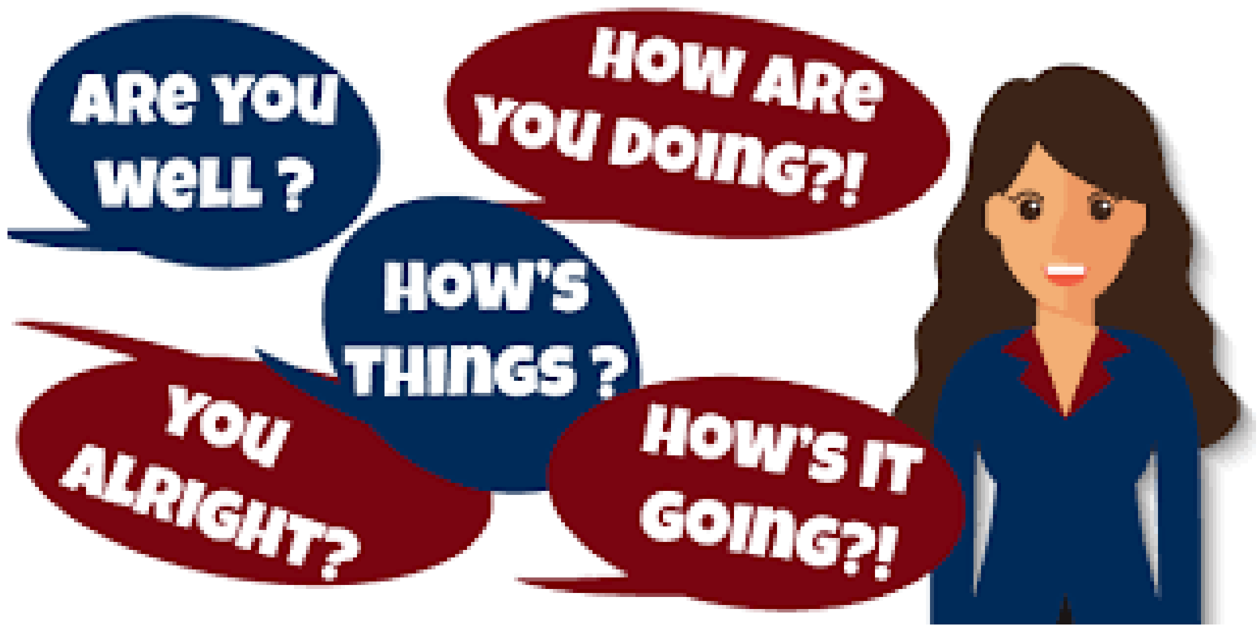

1. Did you track every meal?
2. Did you stick to your fat and calorie targets?
3. Did you weigh every day, & record your weight today?
4. Did you get 5,000 steps a day?
5. Did you email the Intervention team and review the response?
6. What were your unique challenges and successes?

REMINDER: CHARGE YOUR FITBIT & SCALE DURING THIS MEETING!

When did you eat the most fat?

BB-HEALTH

Which meal had the most fat?

- Breakfast
- Lunch
- Dinner
- Snacks

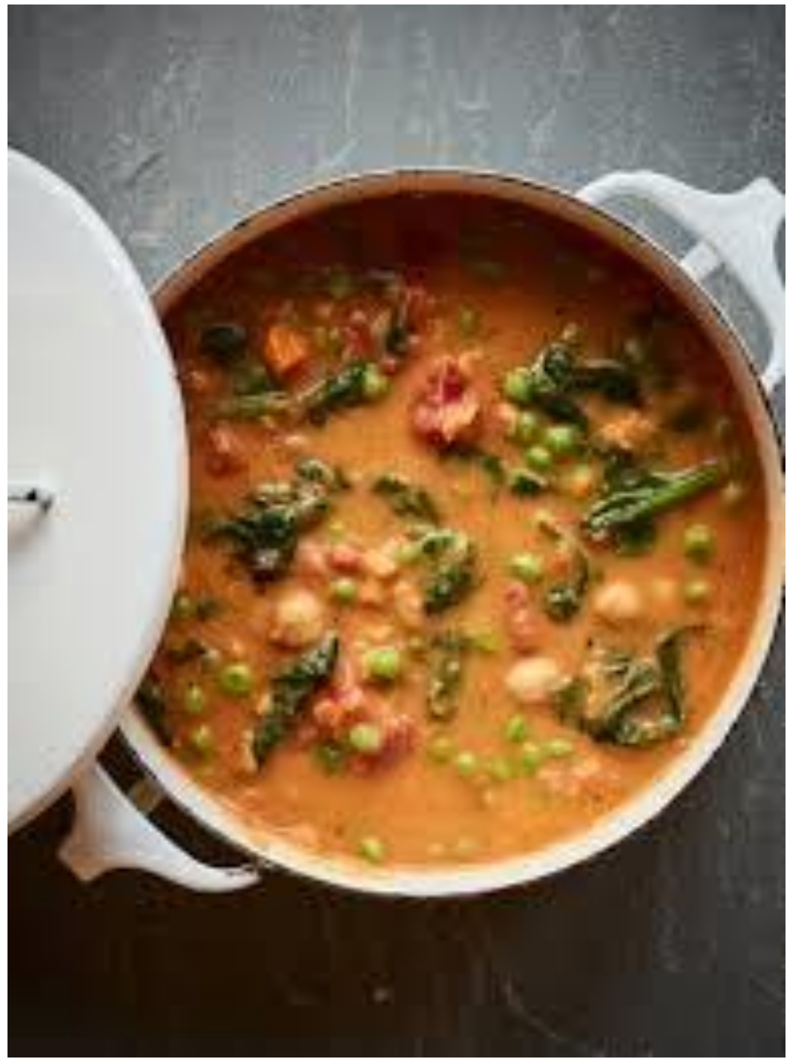

AIM FOR SUCCESS

Figuring out where you are blowing your budget can help make you AWARE of which meal needs the most creativity to help you stay in CONTROL.

How did you successfully reduce fat/calories?

BB-HEALTH

What worked for you this week?

- Eating more fruit/vegetables
- Cooking more
- Bringing healthy food with me
- Meal planning
- Snacking less
- Filling up on high-fiber foods
- Skipping junk food
- Other (discuss)
- All the Above

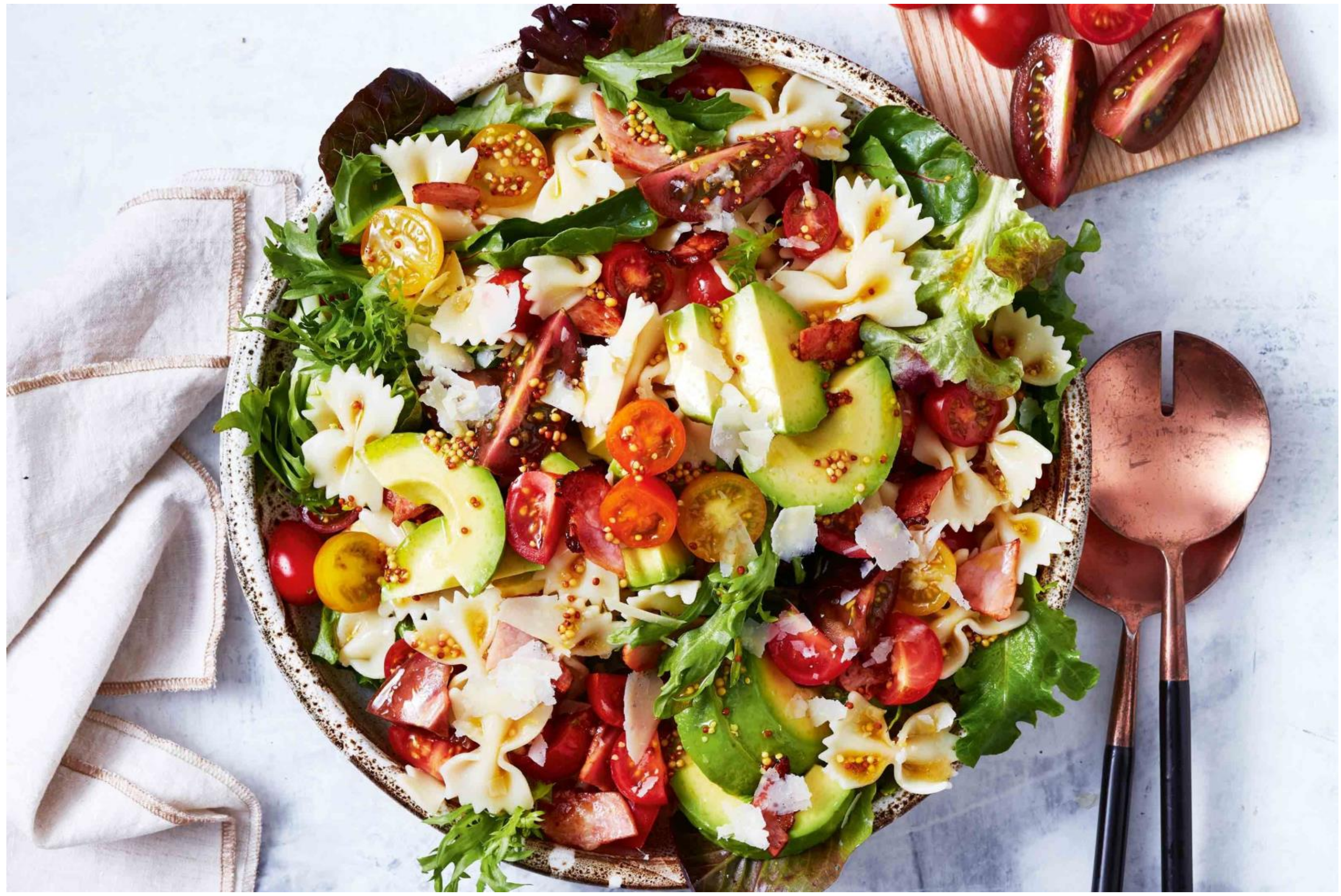

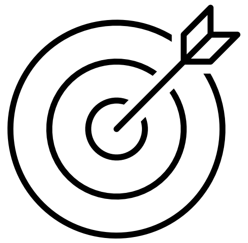

AIM FOR SUCCESS
There are lots of ways you can support your goals, you can even use different strategies on different days.
It is all about finding what works for you!

Conscious Eating

Make eating the main attraction

- Plan ahead
- Stick to an eating schedule
- Eat slowly: take intentional bites, make intentional pauses, and give yourself intentional portions
- Eat at the table, or sit somewhere to focus on your meal

Compose healthy meals

- ½ your plate fruit and vegetables
  - ➢ A big serving of broccoli and an apple, or oranges in a big salad
- ¼ of your plate should be WHOLE Grains
  - ➢ Like whole grain Bran Flakes, or steel cut oats for breakfast
- ¼ of your plate lean protein
  - ➢ Like bean chili, or grilled chicken breast
- 3 servings of dairy day
  - ➢ Like low-fat yogurt or fortified soy milk in your breakfast cereal
- LIMIT saturated fats, added sugar, and sodium

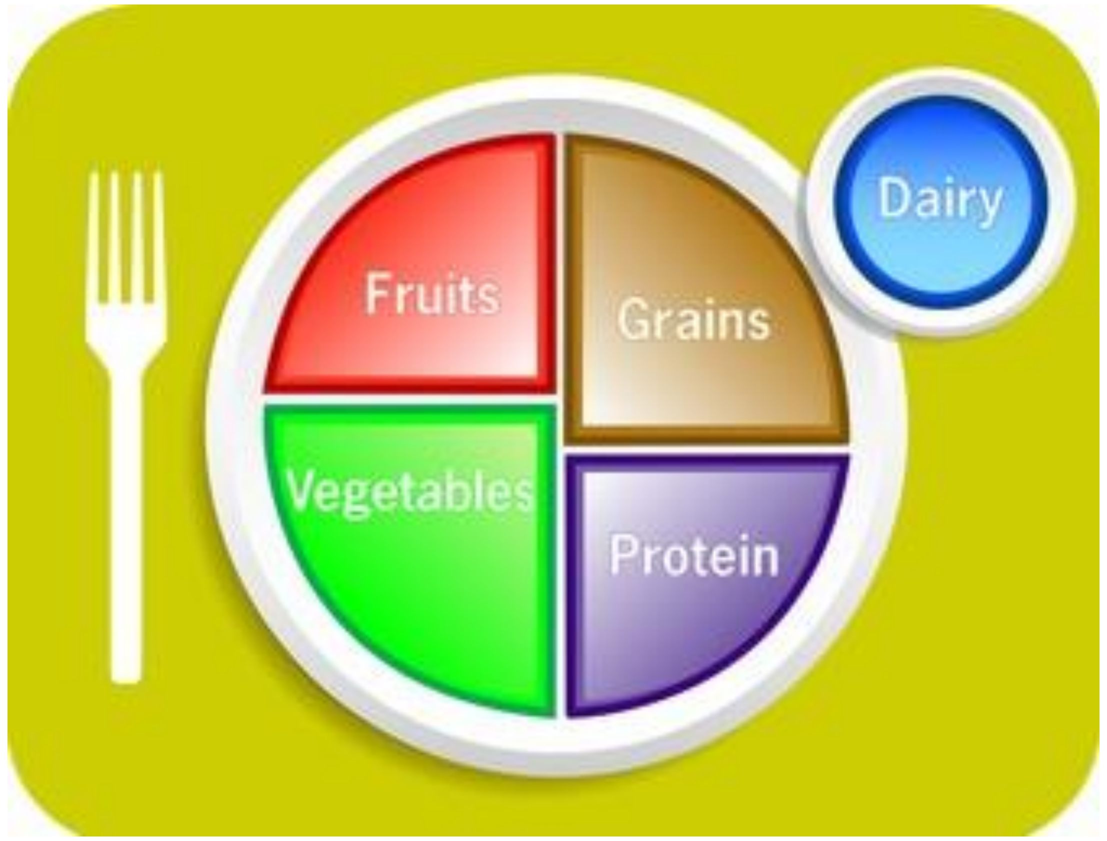

Fruit -- 1-2 cups a day

- Delicious, and so many to choose from!
  - Whole fruits are the best, but they can be FRESH, FROZEN, CANNED, or DRIED
    - If canned, make sure they aren't in JUICE or SYRUP
    - Enjoy 100% fruit juice in small amounts
    - Limit fruity desserts and drinks that are more flavoring and sugar than fruit

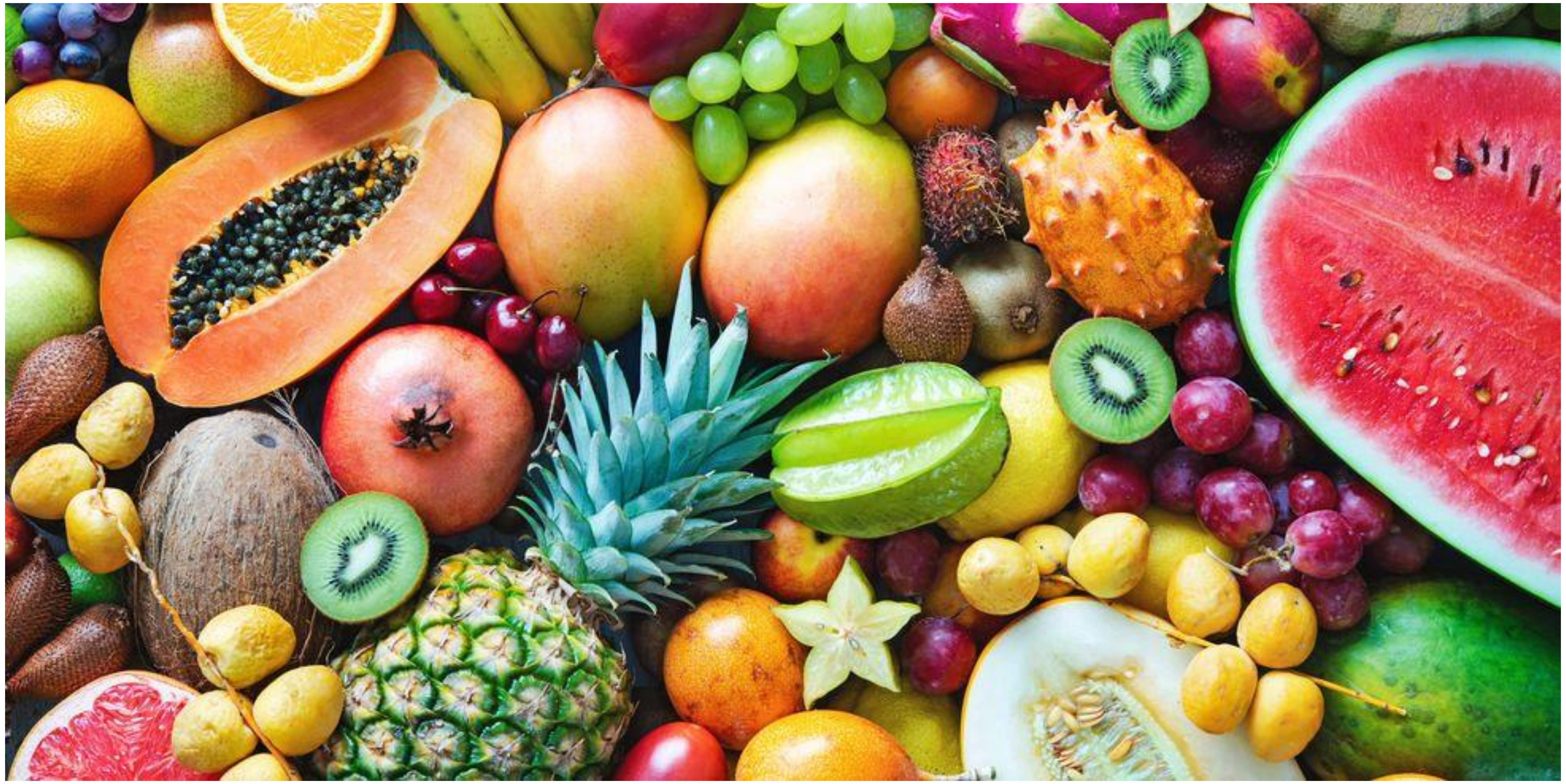

Vegetables -- 2-3 cups a day

BB-HEALTH

- Versatile, filling, and so many ways to eat them!
  - Change them up, try something new every week
  - FRESH, FROZEN, CANNED, DRIED or JUICE
    - Limit fried, and watch out for salty, cheesy, or creamy veggies that sneakily add more calories and fat

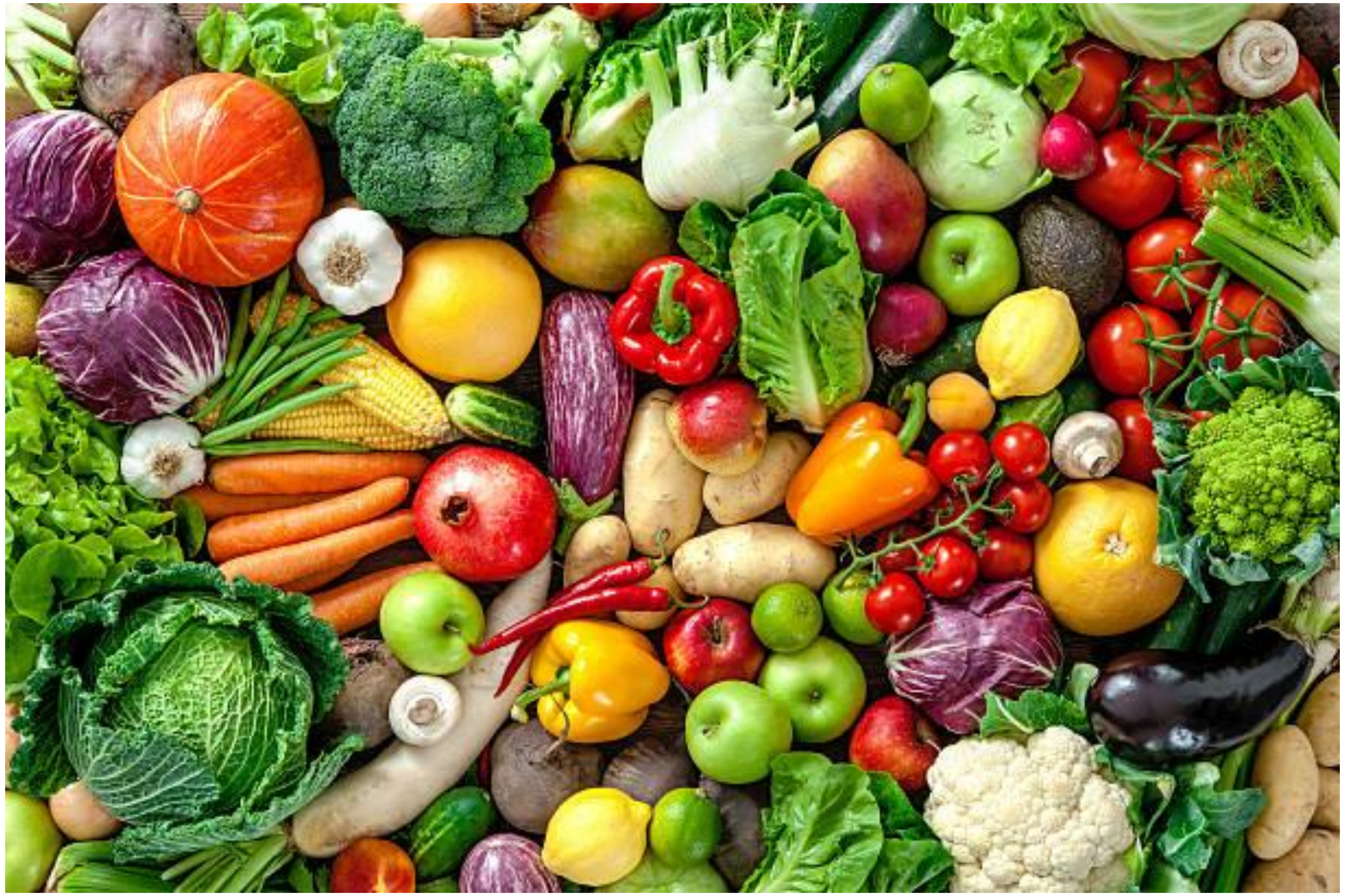

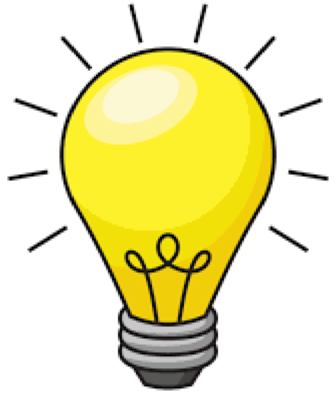

THIS WEEK'S TIP:

Vary those veggies!

Can you name all the veggies in this picture? Veggies are affordable, filling, low fat, and low calorie and there are so many to try. Load up your grocery cart with your faves but keep your eye out for something new to try this week, it could become a new favorite!

Whole Grains- 3 ounces a day

B B - H E A L T H

- Hearty, filling, and comforting!
  - Whole grains are tastier and more filling too!
- Aim for 3 ounces a day (the dry weight)
  - 1 ounce of dry whole-wheat pasta, brown rice, steel cut oats, or quinoa is about ½ cup cooked
  - 1 ounce is 1 slice of whole-grain bread
    - Beware of sneaky added sugar and fat like in white bread, donuts, fried tortilla, and granola
    - Choose HIGH fiber cereals, like: Fiber One Original, All Bran, Trader Joes High Fiber Cereal

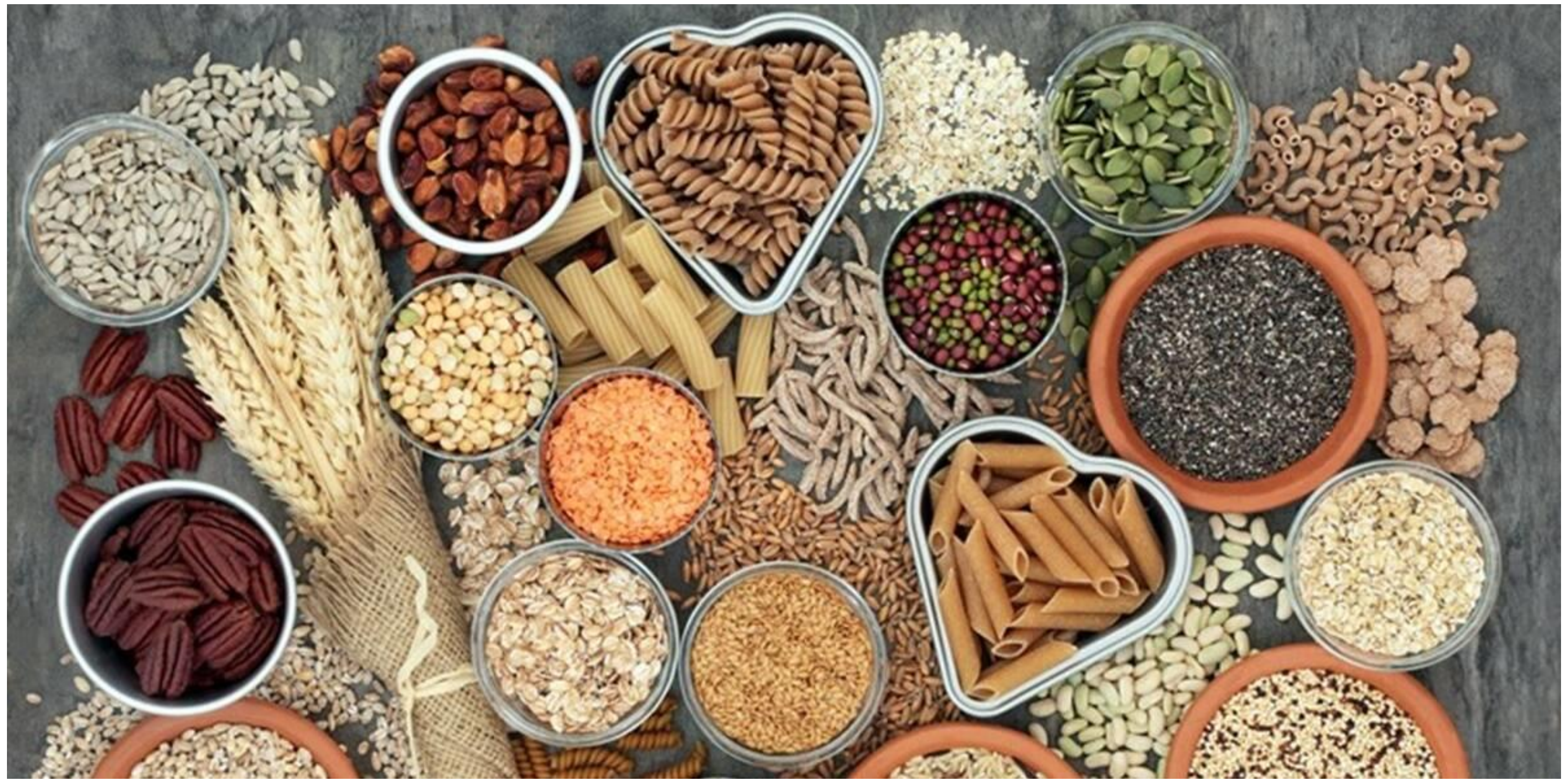

Lean Protein Foods- 5 ounces a day

- Keeps you from feeling hungry longer!
  - Go on a protein adventure: beans, peas, nuts, seeds, egg whites, fish, poultry
- 1 ounce of cooked meat or 2 egg whites = 1 ounce towards a serving of protein food
  - 1/4 cup beans, tofu or 2 tablespoons of hummus
  - 1/2 split pea or lentil soup
    - Red and processed meat has a lot of saturated fat, sodium, and even added sugar!
    - Remember to trim the fat and remove the skin from your meats!
    - Use low-fat cooking methods: baking, grilling, steaming, roasting, and microwaving

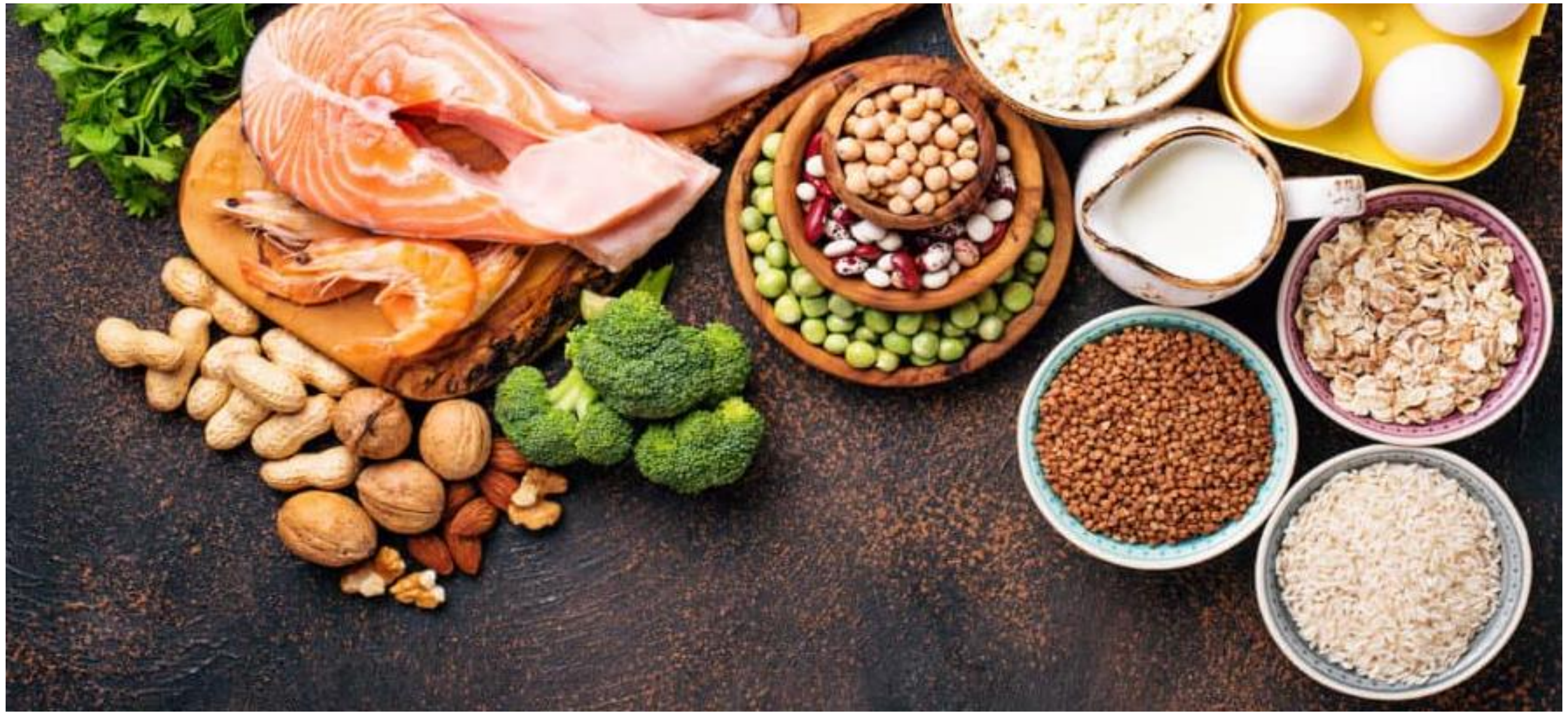

Aim for SUCCESS!

Veggies have protein!

From peanut butter to baked beans plants are an excellent source of protein. And you can count them as your veggies too! Aim to eat plant proteins often.

Dairy Foods- 2 servings a day

- Move to low-fat and fat-free dairy, or fortified soy milk & milk-alternatives
- 1 cup of low-fat milk or yogurt is a serving (remember non-dairy versions are lower in calories!)
  - 1.5 ounces of hard cheeses, like parmesan
  - 1/3 cup of shredded low-fat cheese
    - Watch for creamy foods like smoothies, ice cream, sweetened yogurts, and sauces that bring extra calories along for the ride.
    - Butter is almost entirely FAT, a great way to reduce fat is to not add more when cooking!

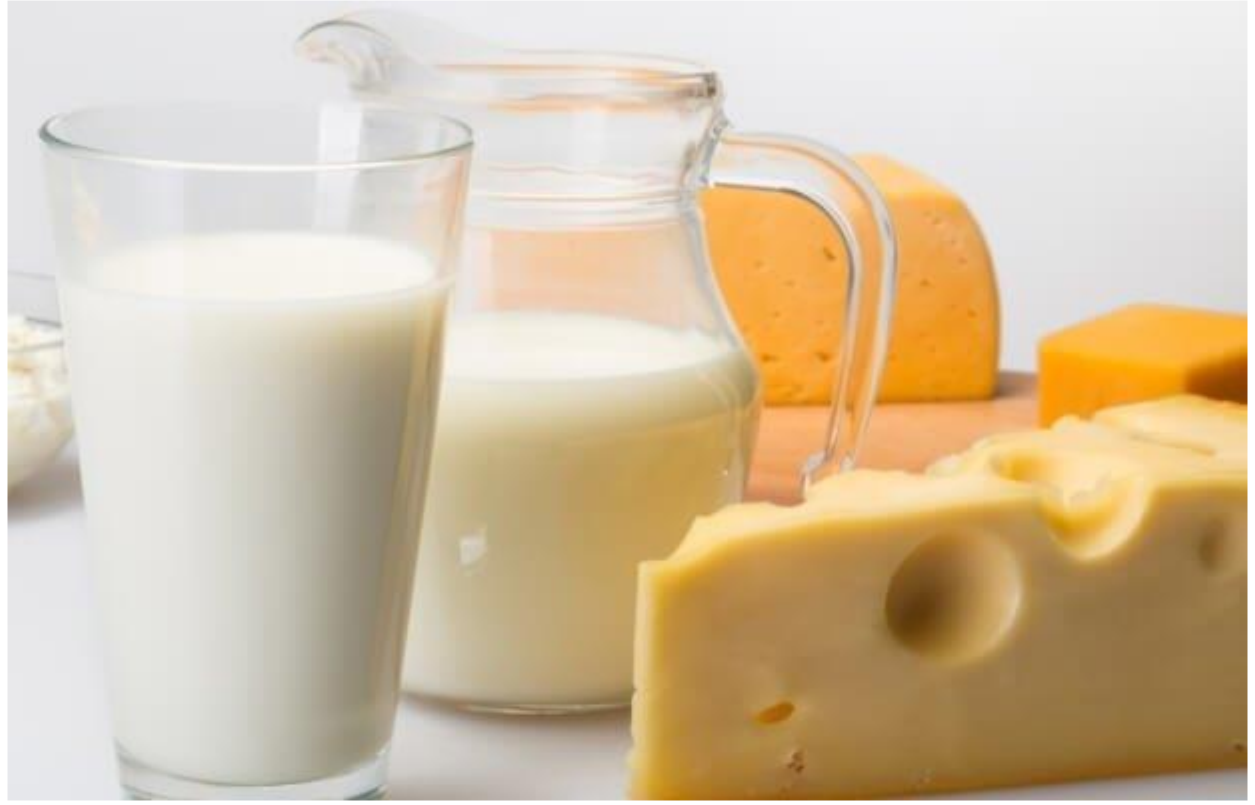

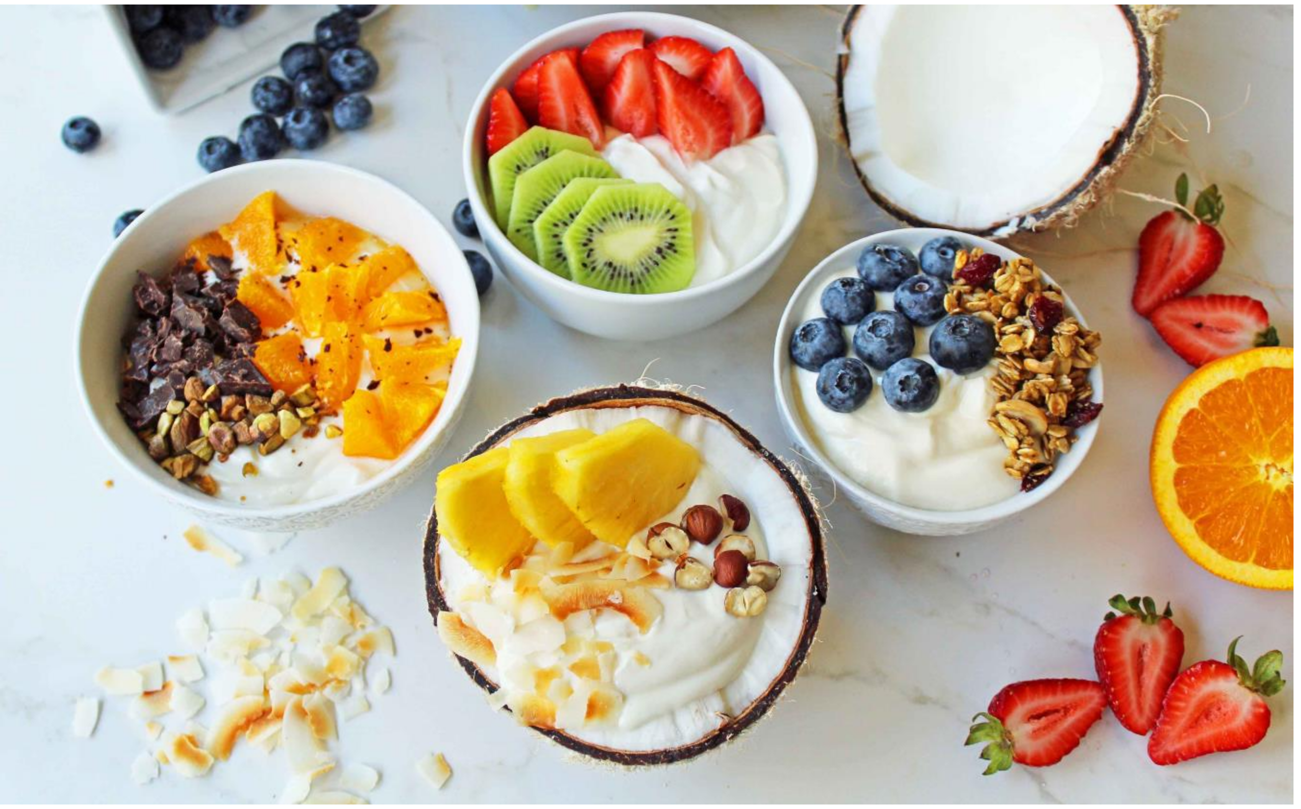

Strategies for Success

1 **DO IT YOUR WAY**<br>Try EVERYTHING to find what you like<br>So that it works for you	6 **VARY YOUR PROTEIN ROUTINE**<br>Try main dishes made with beans or seafood<br>like tuna salad or bean chili
2 **LOAD UP ON FRUIT & VEGGIES**<br>Half your plate = whole fruit and veggies<br>Variety is the spice of life	7 **REDUCE SODIUM, SAT FAT, & SUGAR**<br>Choose vegetable oils instead of butter,<br>and sauces made with oils instead of creams and cheeses
3 **GET YOUR WHOLE GRAINS**<br>Make your grain foods → whole grains	8 **DRINK WATER**<br>Water, including most flavored waters, are 0-CAL<br>Stay hydrated and skip the added sugars with water
4 **CHOOSE LOW-FAT DAIRY**<br>Dairy has a lot of saturated fat<br>Reach for the low-fat and plant-based options	9 **BUILD A HEALTHY EATING PATTERN**<br>Make a plan and stick to it. Be prepared, and<br>Eat on a schedule to keep hunger away
5 **LOAD UP ON FRUIT & VEGGIES**<br>Half your plate = whole fruit and veggies<br>Variety is the spice of life	10 **EVERYTHING YOU EAT MATTERS**<br>Find the right mix that works for you<br>Turn small changes into BIG WINS!

Changes I can make

BB-HEALTH

<u>What will I do to make the strategies for success work for me? (select all)</u>

- ○ Choose healthy fats and avoid saturated fat
- ○ Stick to a healthy eating plan and schedule
- ○ Include a variety of foods every day
- ○ Eat different lean proteins at every meal
- ○ Replace processed foods with more whole fruits, vegetables, nuts, seeds and whole grains
- ○ Snack healthy with yogurt, unsalted nuts and nut butter, or fruit
- ○ Drink more water and less soda
- ○ Eat more fiber, like beans, peas, vegetables and whole grain cereal
- ○ Reduce sodium and season with herbs and spices instead of salt
- ○ All the above
- ○ Other (discuss)

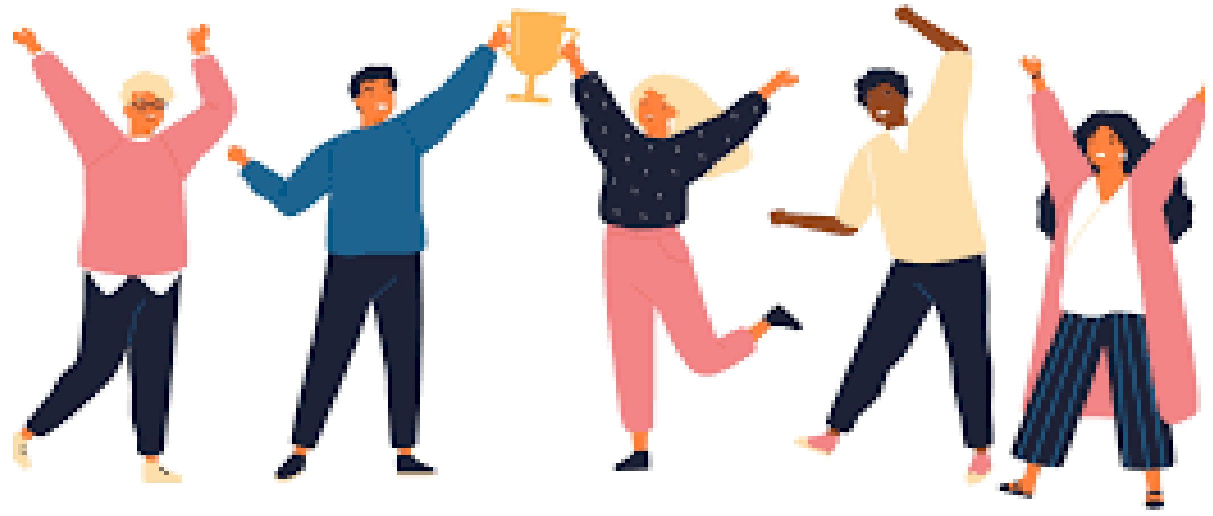

INTERACTIVE

Make a healthy meal! Choose the best options:

- <u>Vegetable</u>
  - French Fries with ketchup
  - Green salad w/ balsamic vinaigrette
  - Broccoli-Cheddar soup
  - Grilled peppers and onions
  - Black beans and salsa, or baked beans
  - Baked potato with drizzle of olive oil and chives
  - 1 & 3
  - 2, 4, 5, & 6

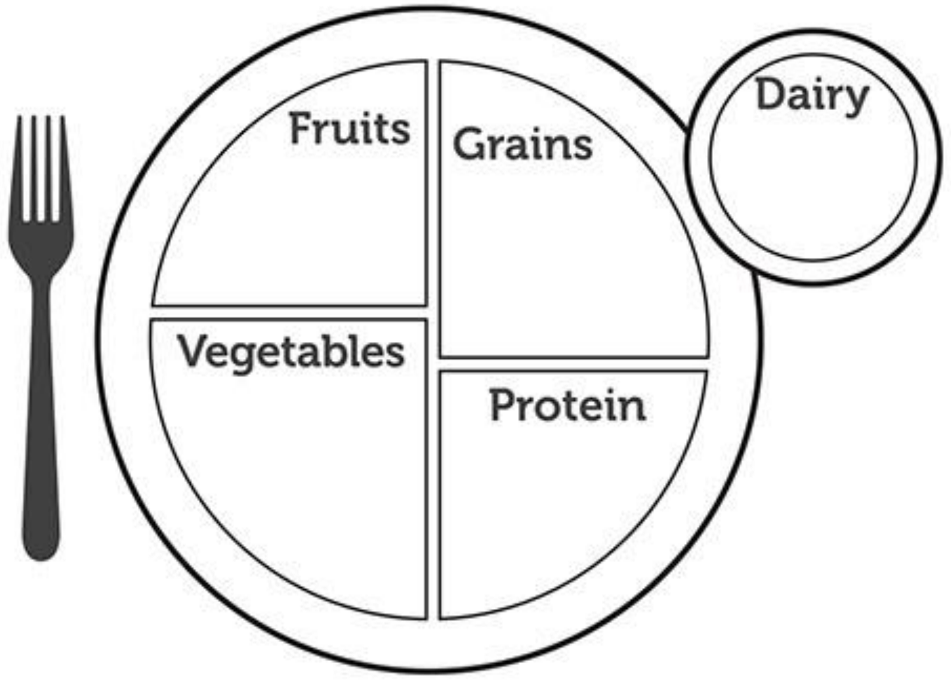

INTERACTIVE

Make a healthy meal! Choose the best options:

- **Fruit**
  - Apple
  - Cocktail Cherries in syrup
  - Peach Pie
  - 4 oz fresh squeezed OJ
  - Cherry Tomatoes
  - 2 & 3
  - 1, 4, & 5

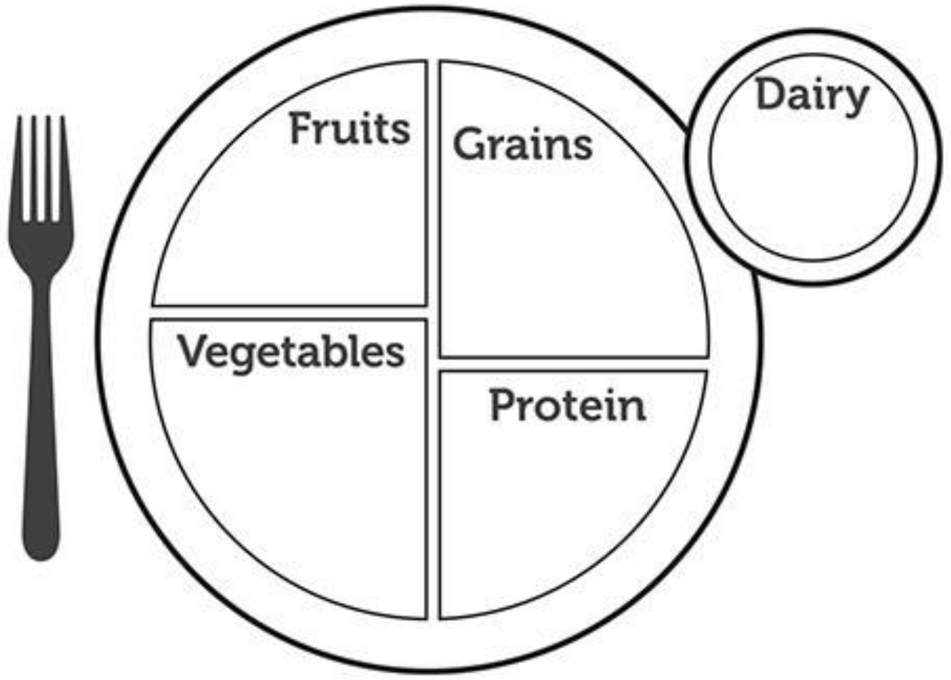

INTERACTIVE

Make a healthy meal! Choose the best options:

- **Grains**
  - Frosted Flakes
  - Bran Flakes
  - Donuts
  - Brown Rice
  - Whole Wheat Pasta
  - Whole wheat bagels
  - 1 & 3
  - 2, 4, 5, & 6

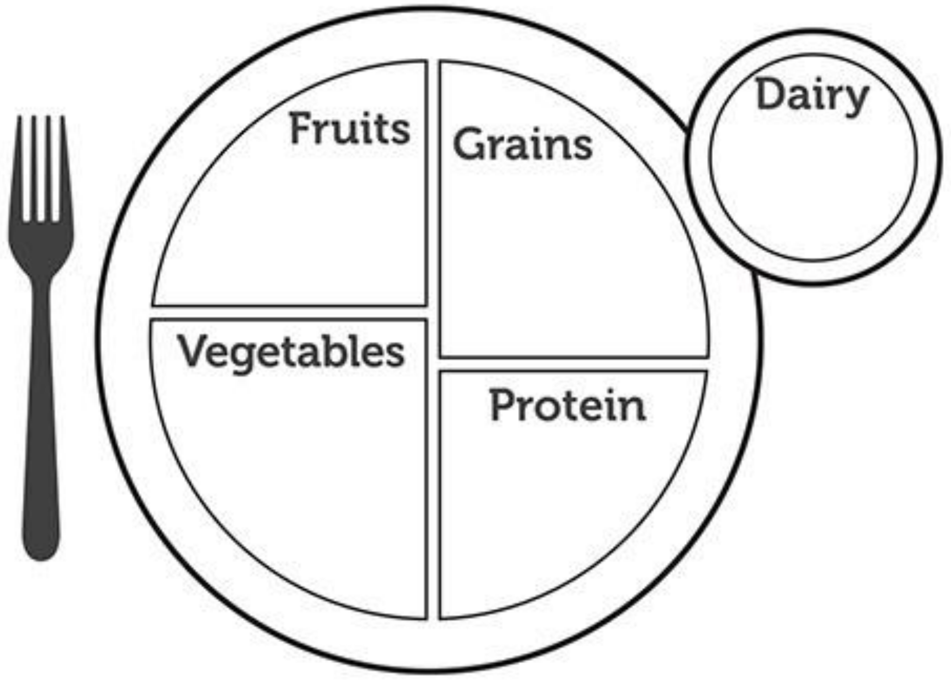

INTERACTIVE

Make a healthy meal! Choose the best options:

- <u>Protein Foods</u>
  - Burger
  - Tuna salad
  - Black beans or baked beans
  - Lean chicken breast
  - Bacon
  - Peanut Butter
  - Walnuts, Almonds, and Pecans
  - 1 & 5
  - 2, 3, 4, 6, & 7

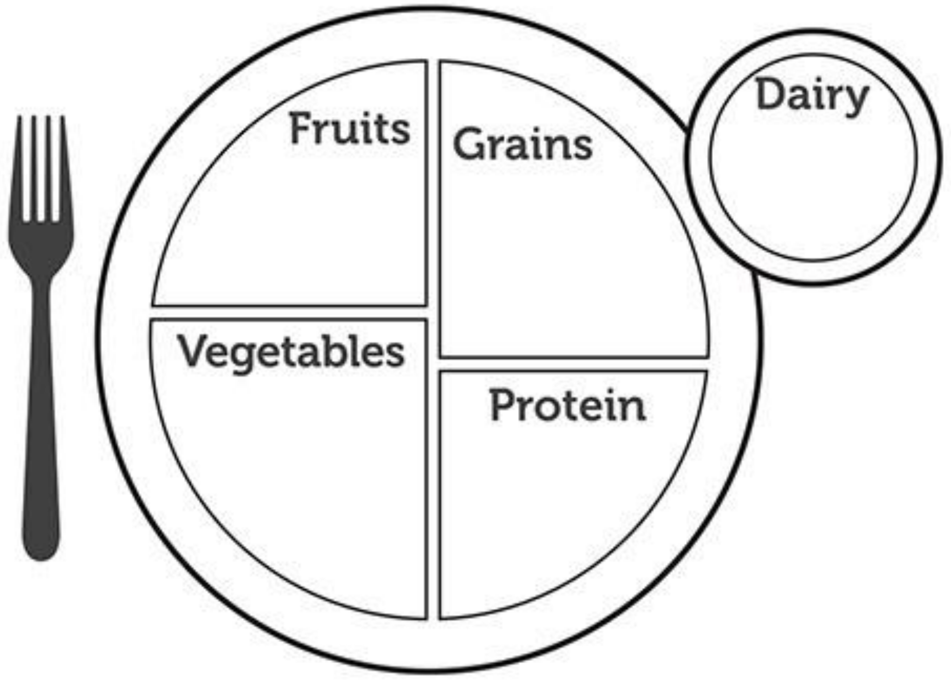

INTERACTIVE

Make a healthy meal! Choose the best options:

- Dairy
  - Alfredo sauce
  - Low-fat yogurt
  - Soy-milk
  - Low-fat cottage cheese
  - 1 & 4
  - 2, 3, & 4

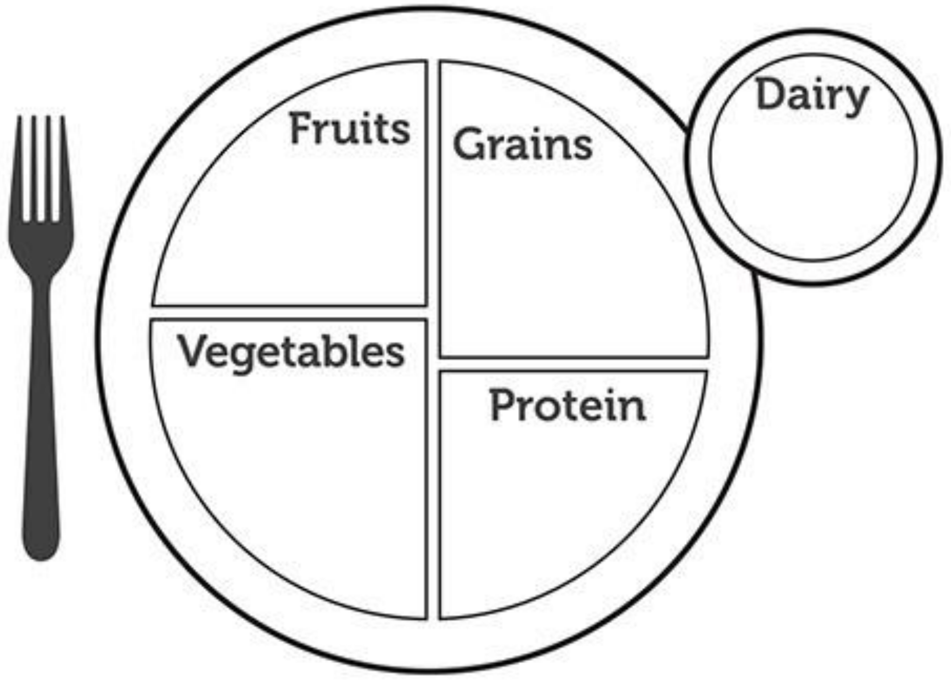

Put it into Practice

BB-HEALTH

Keeping Track of What You Eat

1. Track those meals and take your weight EVERYDAY!
   - Monitor your progress and identify areas for improvement towards YOUR GOALS

2. Ask yourself- can I choose a healthier choice today?
   - When shopping, snacking, or planning meals each time ask yourself what you can improve
     - EXAMPLE: Swap from frying to baking a potato
     - EXAMPLE: Swap from soda to flavored seltzer

3. Give your recipes and meals a healthy make-over!
   - Pages 12-14 of the handout gives tips to improve your meal plan!
     - Healthier snacks, ways to season food to make it taste delicious, cooking methods and more!

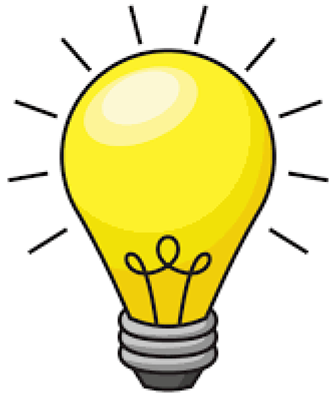

REMEMBER THIS WEEK'S TIP: ***VARY THOSE VEGGIES!***
Try something new this week, or if you can change it up every meal!
Choose veggies high in protein and fiber to satisfy you even longer with fewer calories!

Questions?

B B - H E A L T H

For next week:

1. Go on a new food adventure, keep track of the new foods you tried

2. Track meals and **<u>Weigh every day</u>**

3. Make 2,000 steps of your 5,000 steps brisk walking

4. Email the Intervention Team and Review the Repsonse!

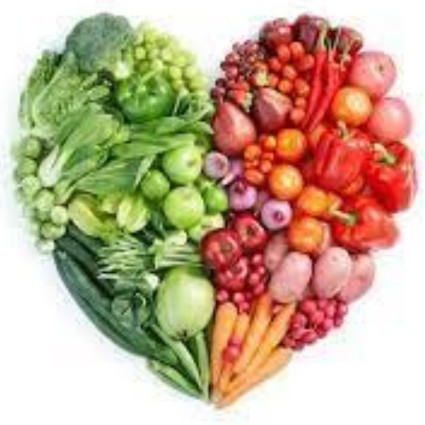

FOOD FOR THOUGHT

- Build on successes, no one is perfect
- Focus on what is working, look for creative solutions
