# Supplementary material for: Development and Feasibility of an eHealth Diabetes Prevention Program Adapted for Older Adults—Results from a Randomized Control Pilot Study
Source: Nutrients. 2024 Mar 23;16(7):930. doi: 10.3390/nu16070930 (PMC11154527; doi:10.3390/nu16070930)
Supplement: Supplementary file 1 [file nutrients-16-00930-s001.zip › Week4.pptx]

## Slide 1
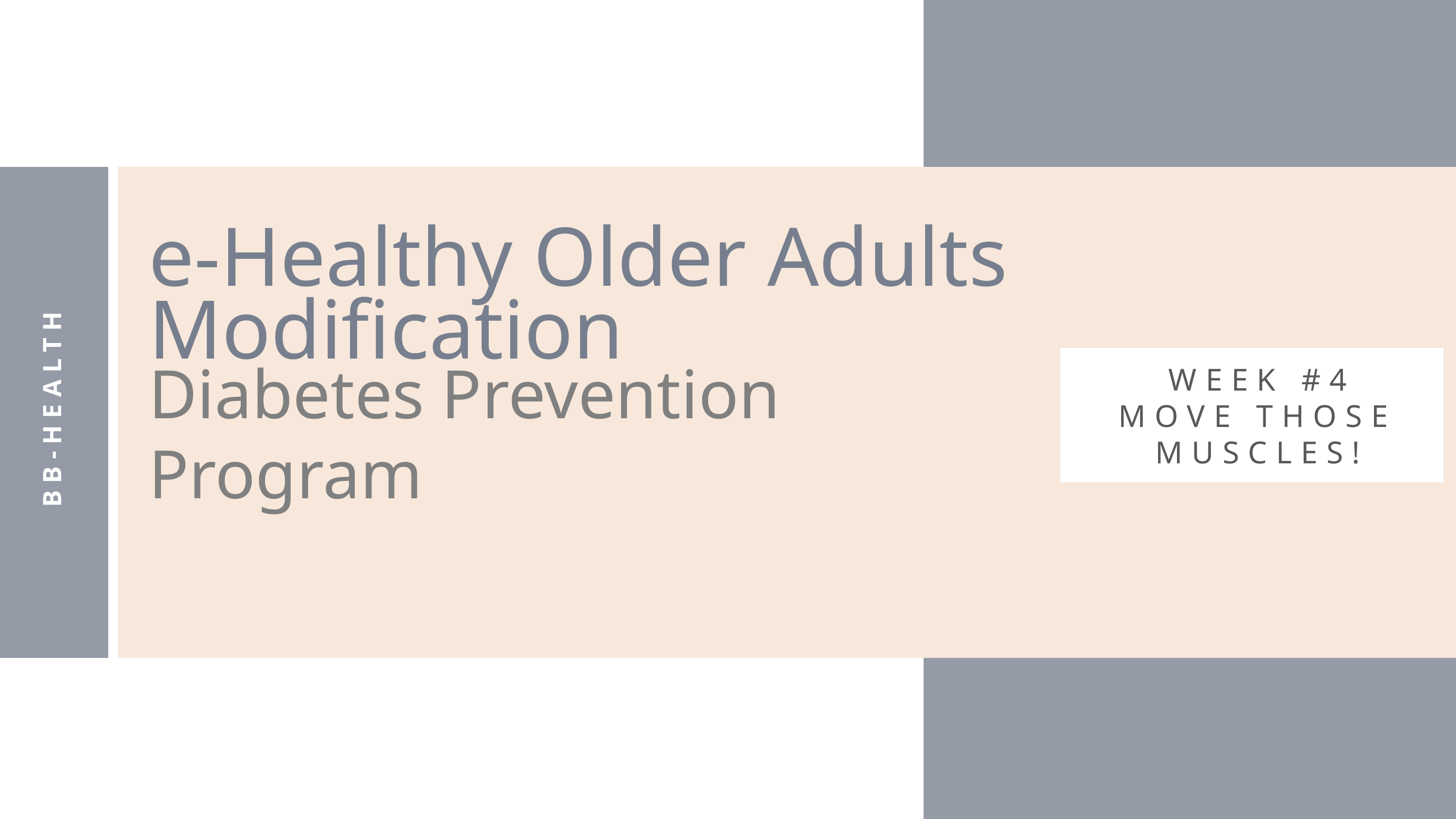

OPEN REPORTS
e-Healthy Older Adults Modification
WEEK #4
MOVE THOSE MUSCLES!
Diabetes Prevention Program
BB-HEALTH

## Slide 2
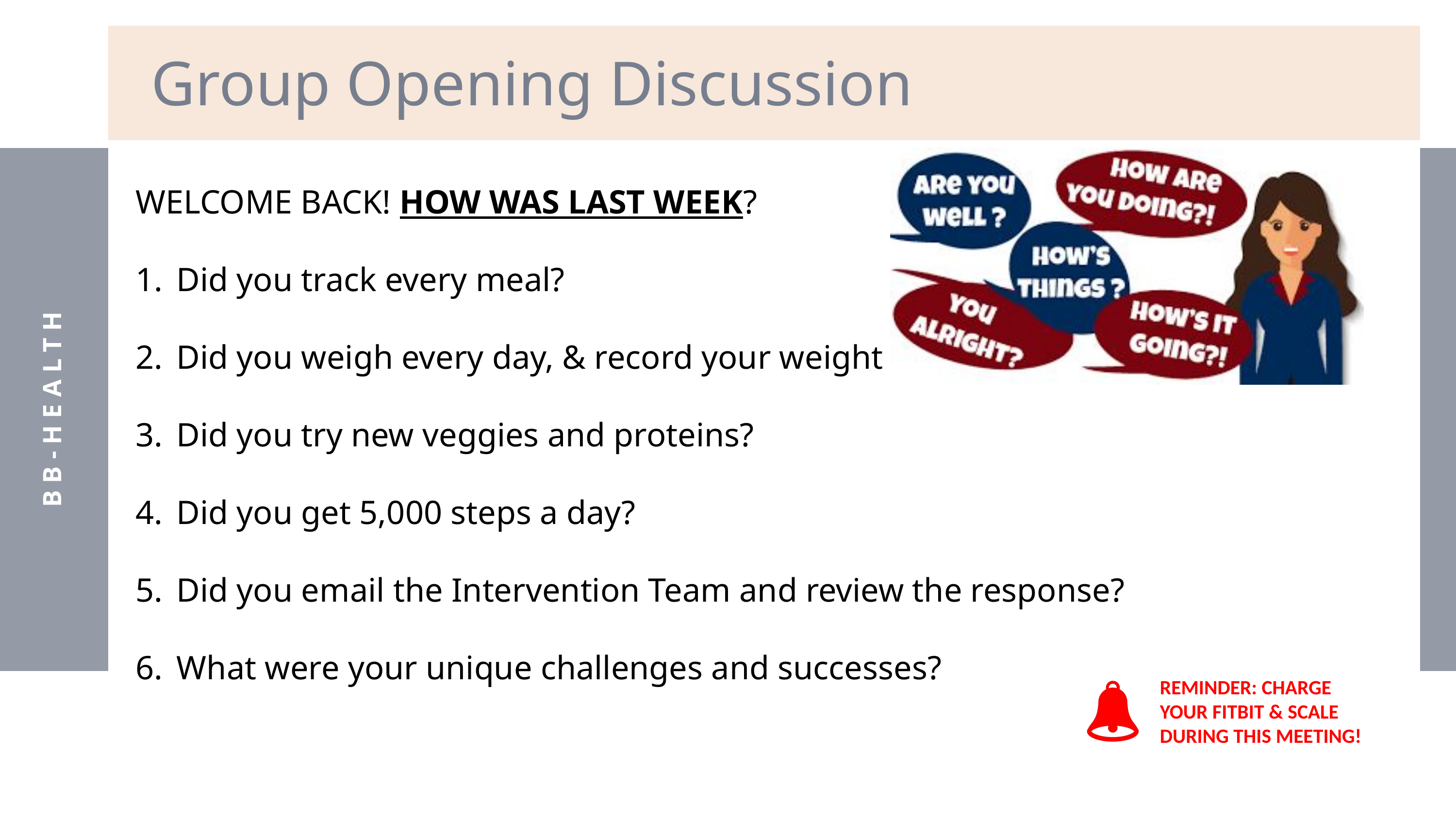

Group Opening Discussion
WELCOME BACK! HOW WAS LAST WEEK?
Did you track every meal?
Did you weigh every day, & record your weight today?
Did you try new veggies and proteins?
Did you get 5,000 steps a day?
Did you email the Intervention Team and review the response?
What were your unique challenges and successes?
BB-HEALTH
REMINDER: CHARGE YOUR FITBIT & SCALE DURING THIS MEETING!

## Slide 3
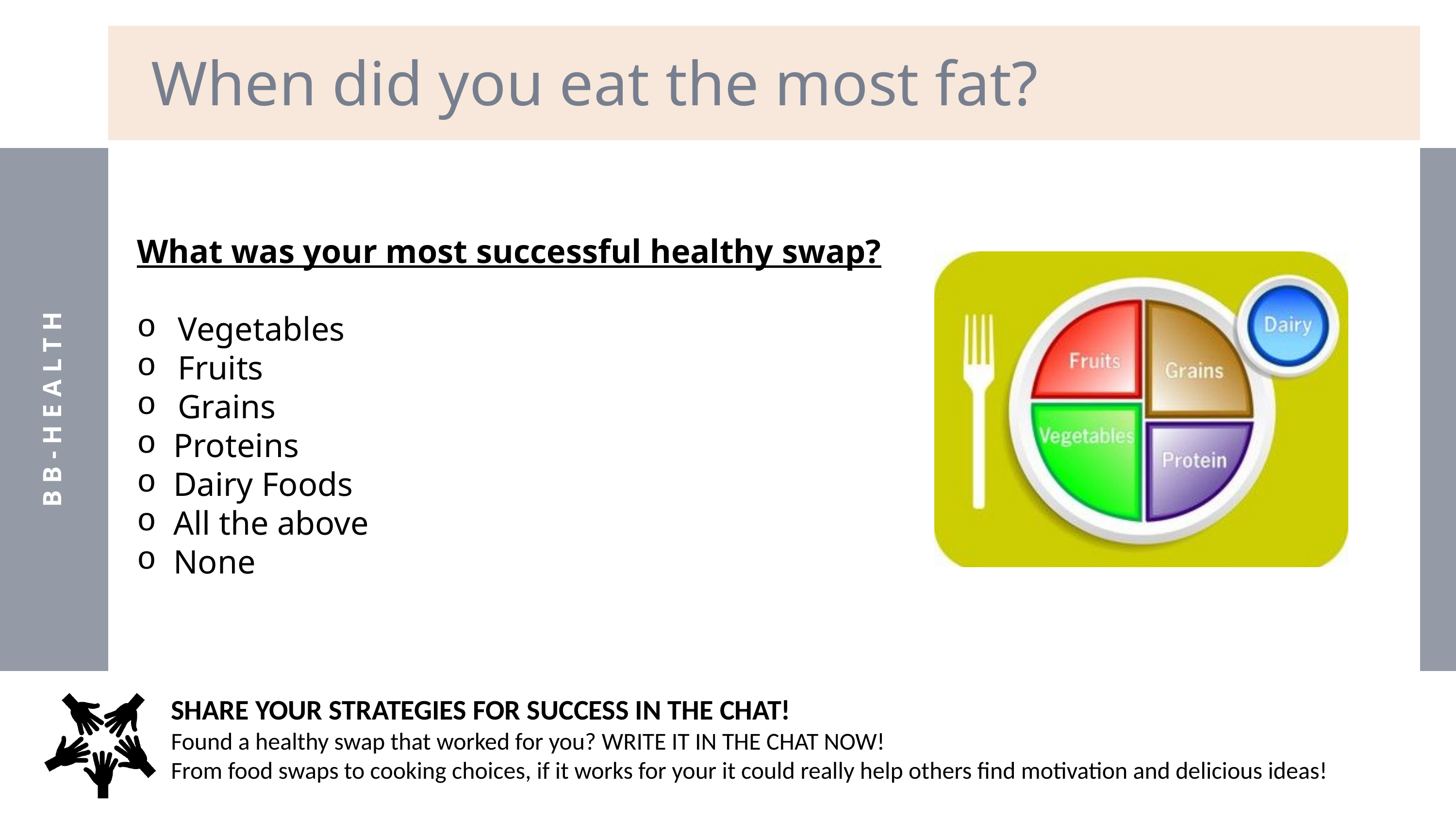

When did you eat the most fat?
What was your most successful healthy swap?
Vegetables
Fruits
Grains
Proteins
Dairy Foods
All the above
None
BB-HEALTH
SHARE YOUR STRATEGIES FOR SUCCESS IN THE CHAT!
Found a healthy swap that worked for you? WRITE IT IN THE CHAT NOW!
From food swaps to cooking choices, if it works for your it could really help others find motivation and delicious ideas!

## Slide 4
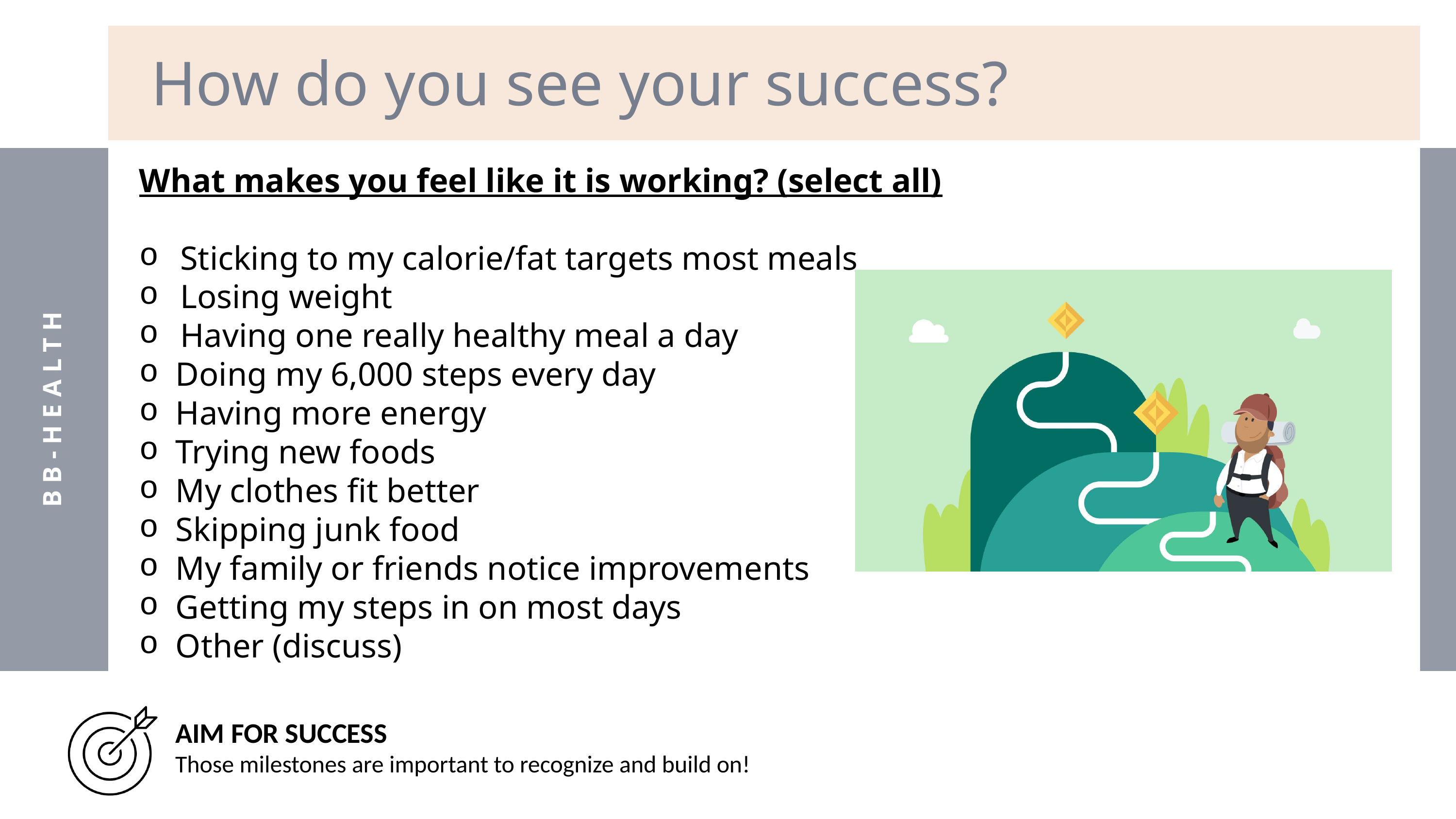

How do you see your success?
What makes you feel like it is working? (select all)
Sticking to my calorie/fat targets most meals
Losing weight
Having one really healthy meal a day
Doing my 6,000 steps every day
Having more energy
Trying new foods
My clothes fit better
Skipping junk food
My family or friends notice improvements
Getting my steps in on most days
Other (discuss)
BB-HEALTH
AIM FOR SUCCESS
Those milestones are important to recognize and build on!

## Slide 5
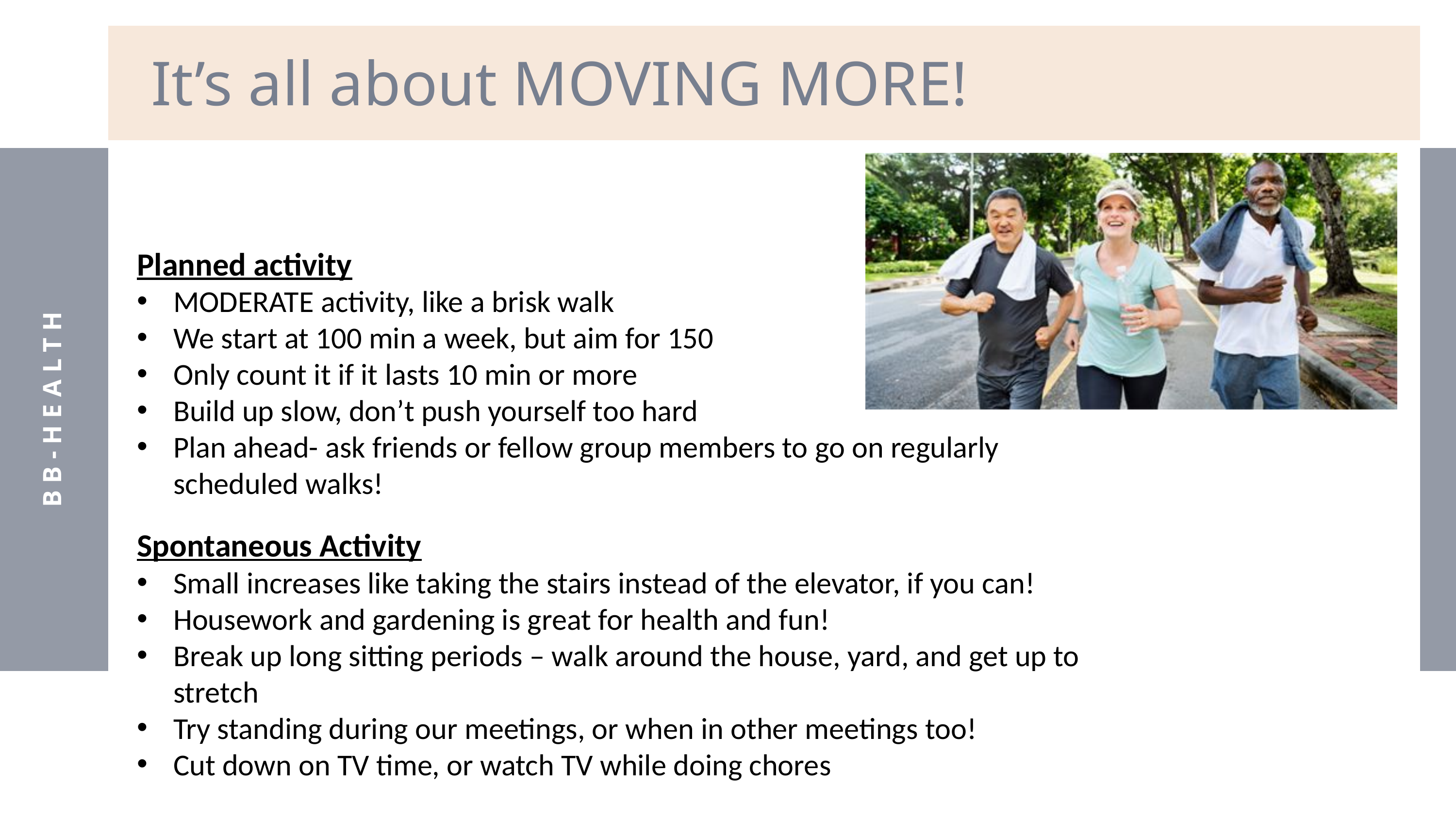

It’s all about MOVING MORE!
Planned activity
MODERATE activity, like a brisk walk
We start at 100 min a week, but aim for 150
Only count it if it lasts 10 min or more
Build up slow, don’t push yourself too hard
Plan ahead- ask friends or fellow group members to go on regularly scheduled walks!
Spontaneous Activity
Small increases like taking the stairs instead of the elevator, if you can!
Housework and gardening is great for health and fun!
Break up long sitting periods – walk around the house, yard, and get up to stretch
Try standing during our meetings, or when in other meetings too!
Cut down on TV time, or watch TV while doing chores
BB-HEALTH

## Slide 6
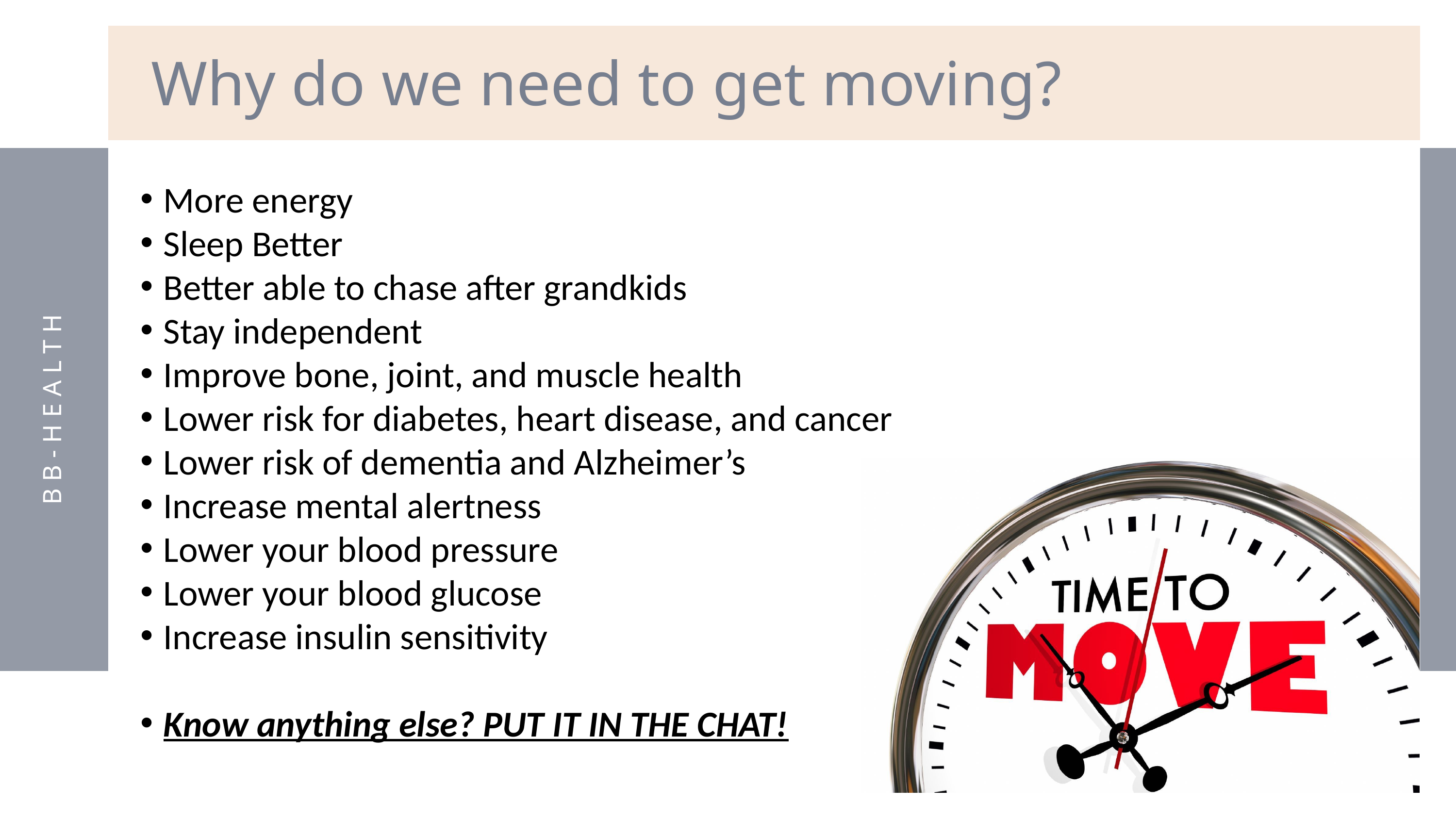

Why do we need to get moving?
More energy
Sleep Better
Better able to chase after grandkids
Stay independent
Improve bone, joint, and muscle health
Lower risk for diabetes, heart disease, and cancer
Lower risk of dementia and Alzheimer’s
Increase mental alertness
Lower your blood pressure
Lower your blood glucose
Increase insulin sensitivity
Know anything else? PUT IT IN THE CHAT!
BB-HEALTH

## Slide 7
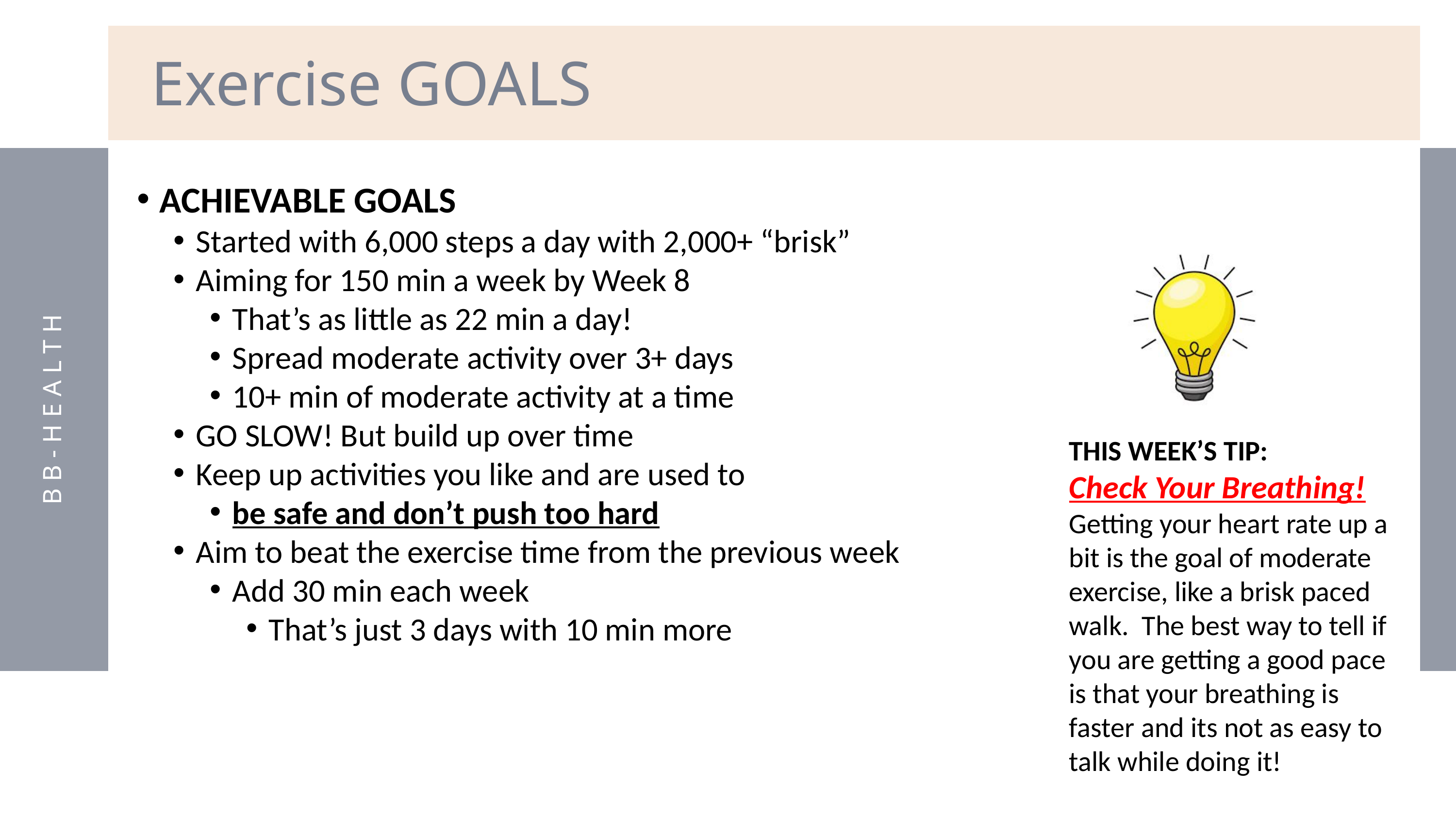

Exercise GOALS
ACHIEVABLE GOALS
Started with 6,000 steps a day with 2,000+ “brisk”
Aiming for 150 min a week by Week 8
That’s as little as 22 min a day!
Spread moderate activity over 3+ days
10+ min of moderate activity at a time
GO SLOW! But build up over time
Keep up activities you like and are used to
be safe and don’t push too hard
Aim to beat the exercise time from the previous week
Add 30 min each week
That’s just 3 days with 10 min more
BB-HEALTH
THIS WEEK’S TIP:
Check Your Breathing!
Getting your heart rate up a bit is the goal of moderate exercise, like a brisk paced walk. The best way to tell if you are getting a good pace is that your breathing is faster and its not as easy to talk while doing it!

## Slide 8
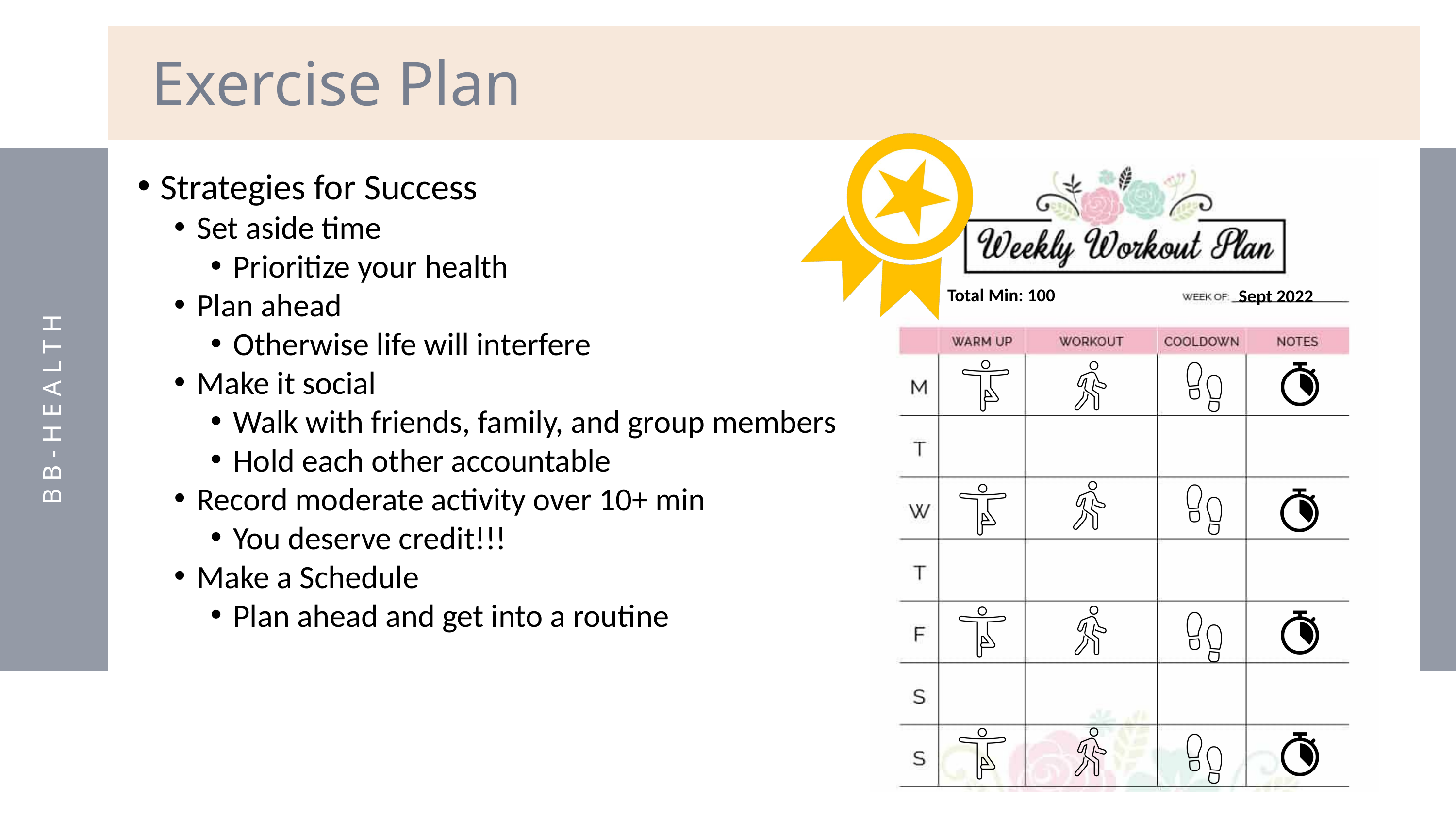

Exercise Plan
Strategies for Success
Set aside time
Prioritize your health
Plan ahead
Otherwise life will interfere
Make it social
Walk with friends, family, and group members
Hold each other accountable
Record moderate activity over 10+ min
You deserve credit!!!
Make a Schedule
Plan ahead and get into a routine
Total Min: 100
Sept 2022
BB-HEALTH

## Slide 9
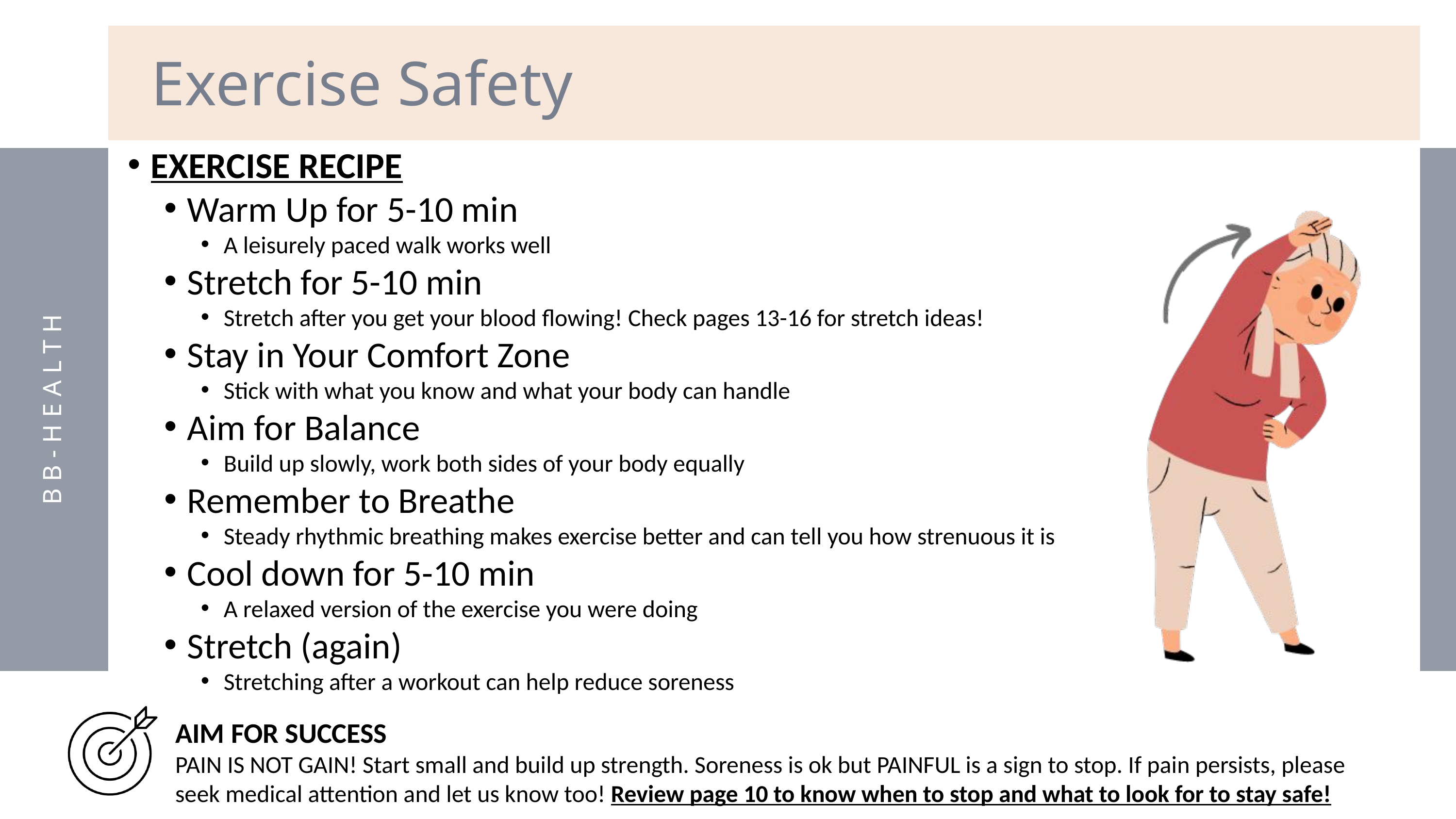

Exercise Safety
EXERCISE RECIPE
Warm Up for 5-10 min
A leisurely paced walk works well
Stretch for 5-10 min
Stretch after you get your blood flowing! Check pages 13-16 for stretch ideas!
Stay in Your Comfort Zone
Stick with what you know and what your body can handle
Aim for Balance
Build up slowly, work both sides of your body equally
Remember to Breathe
Steady rhythmic breathing makes exercise better and can tell you how strenuous it is
Cool down for 5-10 min
A relaxed version of the exercise you were doing
Stretch (again)
Stretching after a workout can help reduce soreness
BB-HEALTH
AIM FOR SUCCESS
PAIN IS NOT GAIN! Start small and build up strength. Soreness is ok but PAINFUL is a sign to stop. If pain persists, please seek medical attention and let us know too! Review page 10 to know when to stop and what to look for to stay safe!

## Slide 10
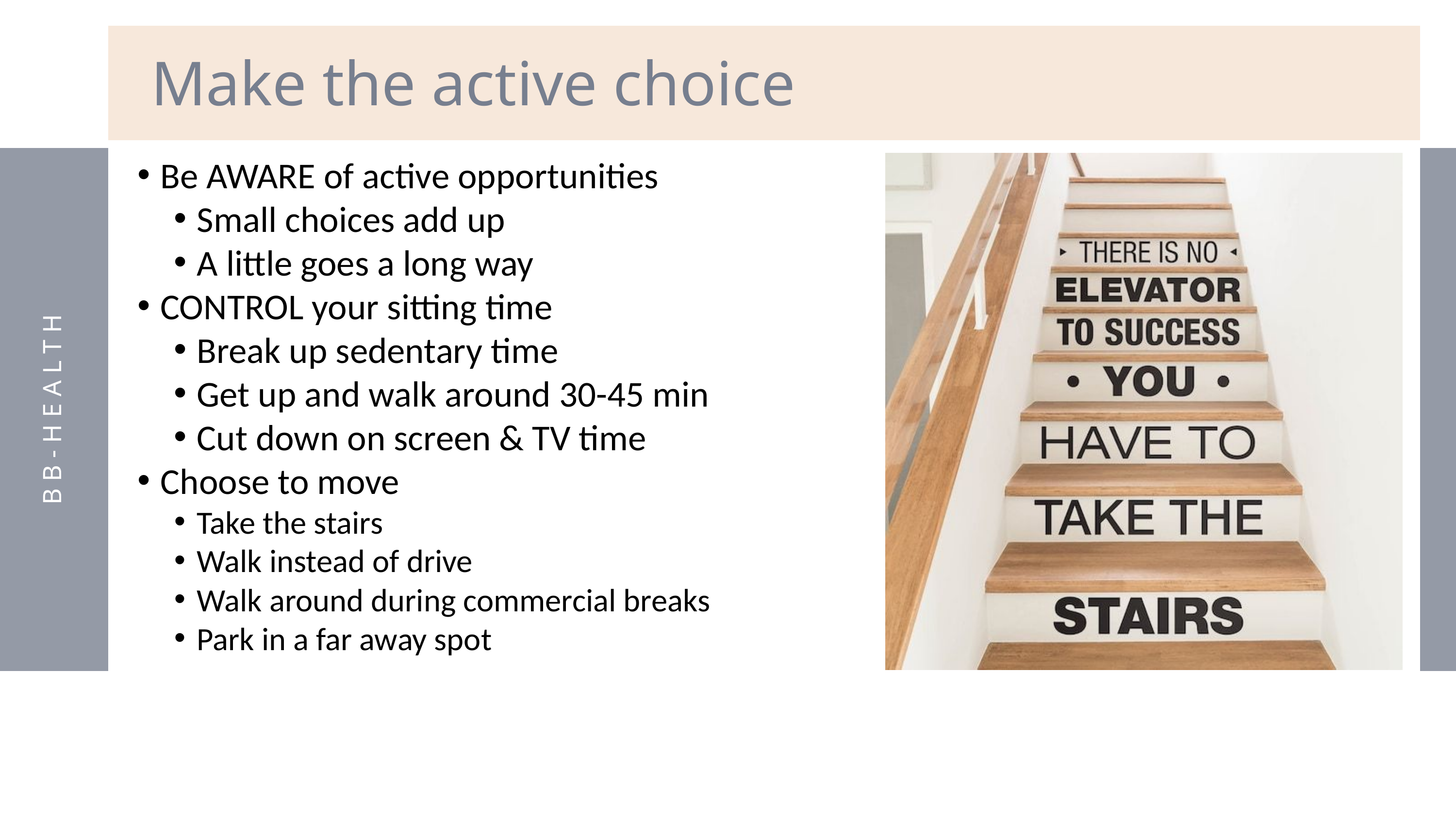

Make the active choice
Be AWARE of active opportunities
Small choices add up
A little goes a long way
CONTROL your sitting time
Break up sedentary time
Get up and walk around 30-45 min
Cut down on screen & TV time
Choose to move
Take the stairs
Walk instead of drive
Walk around during commercial breaks
Park in a far away spot
BB-HEALTH

## Slide 11
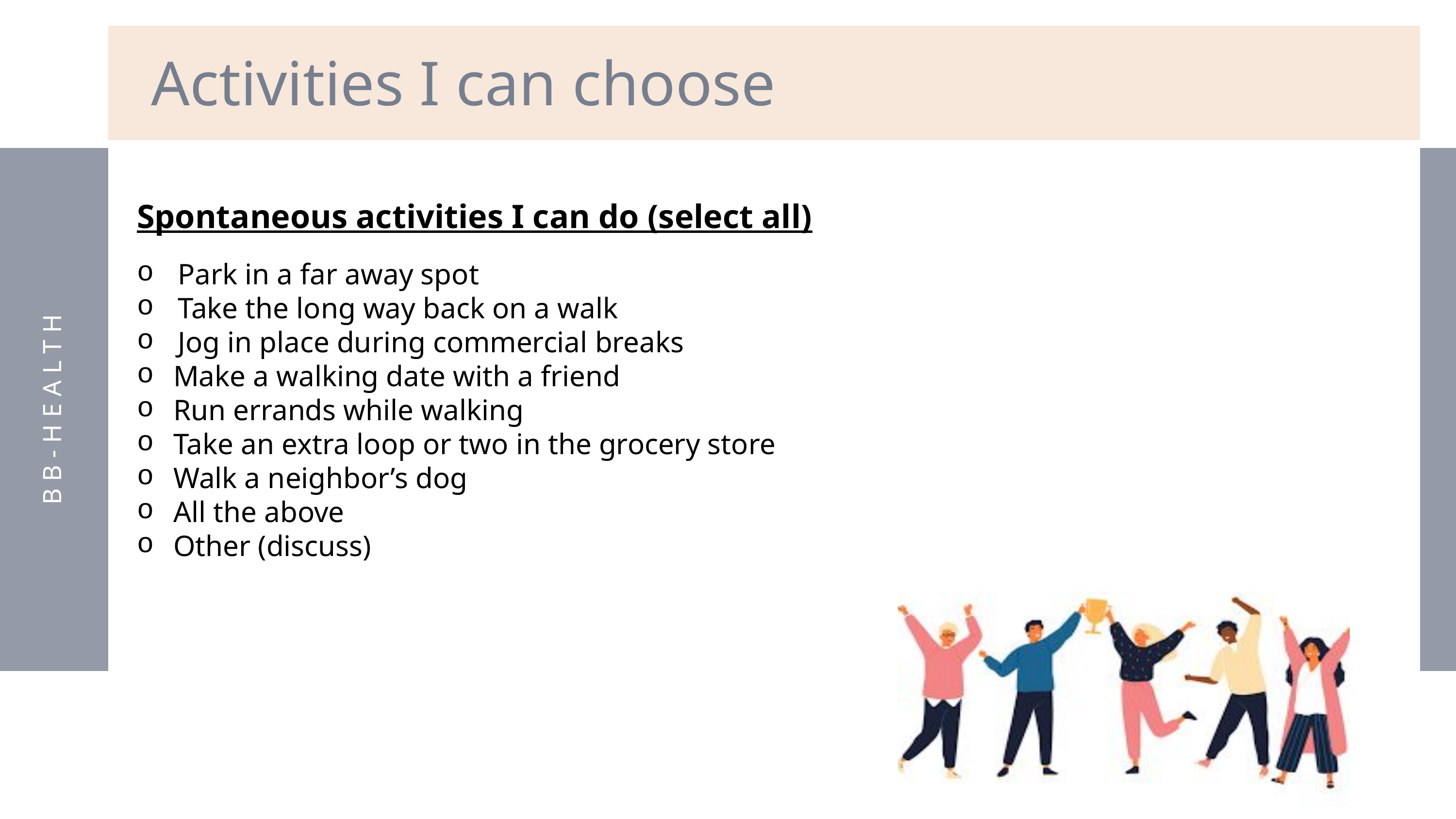

Activities I can choose
Spontaneous activities I can do (select all)
Park in a far away spot
Take the long way back on a walk
Jog in place during commercial breaks
Make a walking date with a friend
Run errands while walking
Take an extra loop or two in the grocery store
Walk a neighbor’s dog
All the above
Other (discuss)
BB-HEALTH

## Slide 12
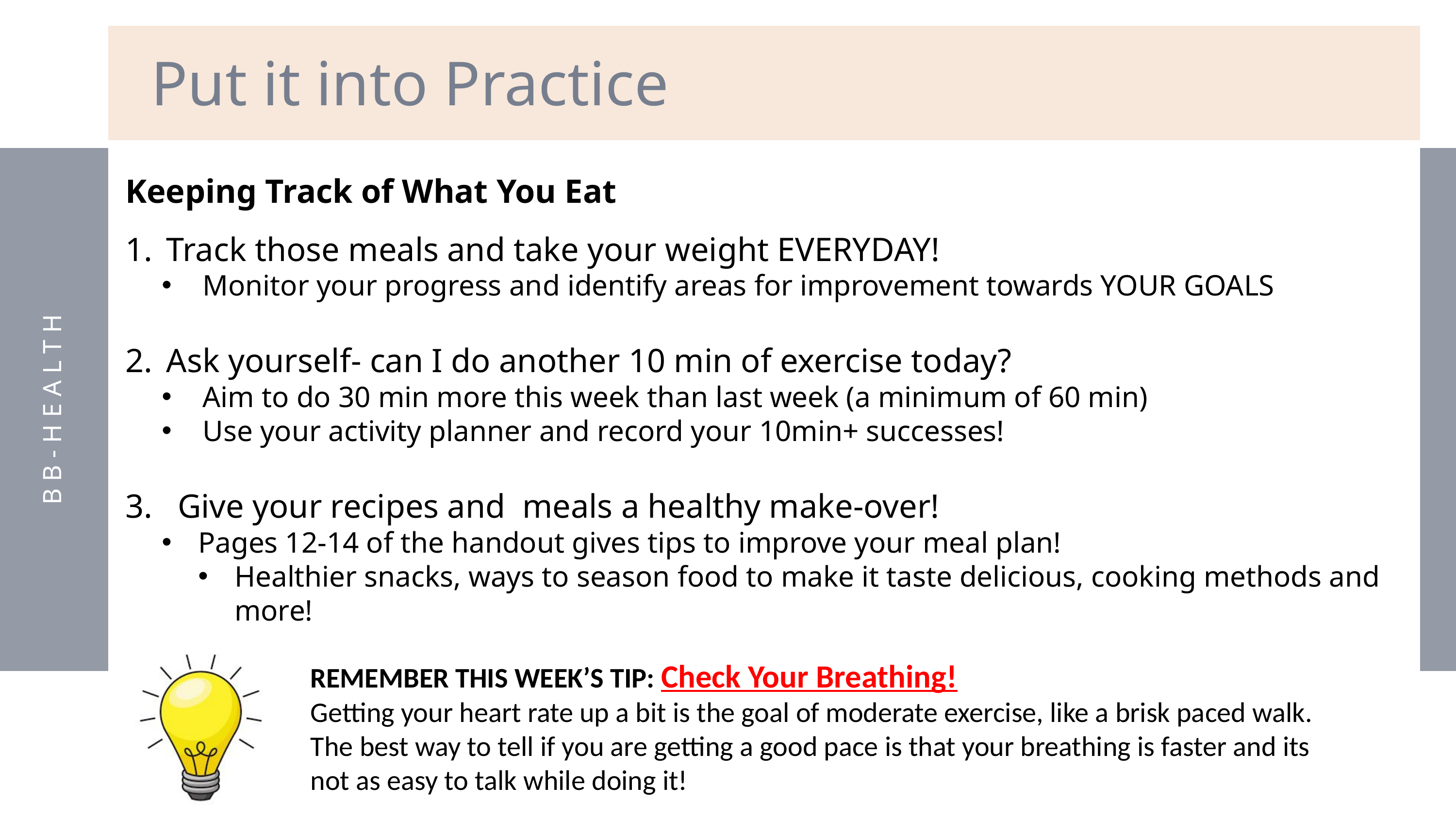

Put it into Practice
Keeping Track of What You Eat
Track those meals and take your weight EVERYDAY!
Monitor your progress and identify areas for improvement towards YOUR GOALS
Ask yourself- can I do another 10 min of exercise today?
Aim to do 30 min more this week than last week (a minimum of 60 min)
Use your activity planner and record your 10min+ successes!
3. Give your recipes and meals a healthy make-over!
Pages 12-14 of the handout gives tips to improve your meal plan!
Healthier snacks, ways to season food to make it taste delicious, cooking methods and more!
BB-HEALTH
REMEMBER THIS WEEK’S TIP: Check Your Breathing!
Getting your heart rate up a bit is the goal of moderate exercise, like a brisk paced walk. The best way to tell if you are getting a good pace is that your breathing is faster and its not as easy to talk while doing it!

## Slide 13
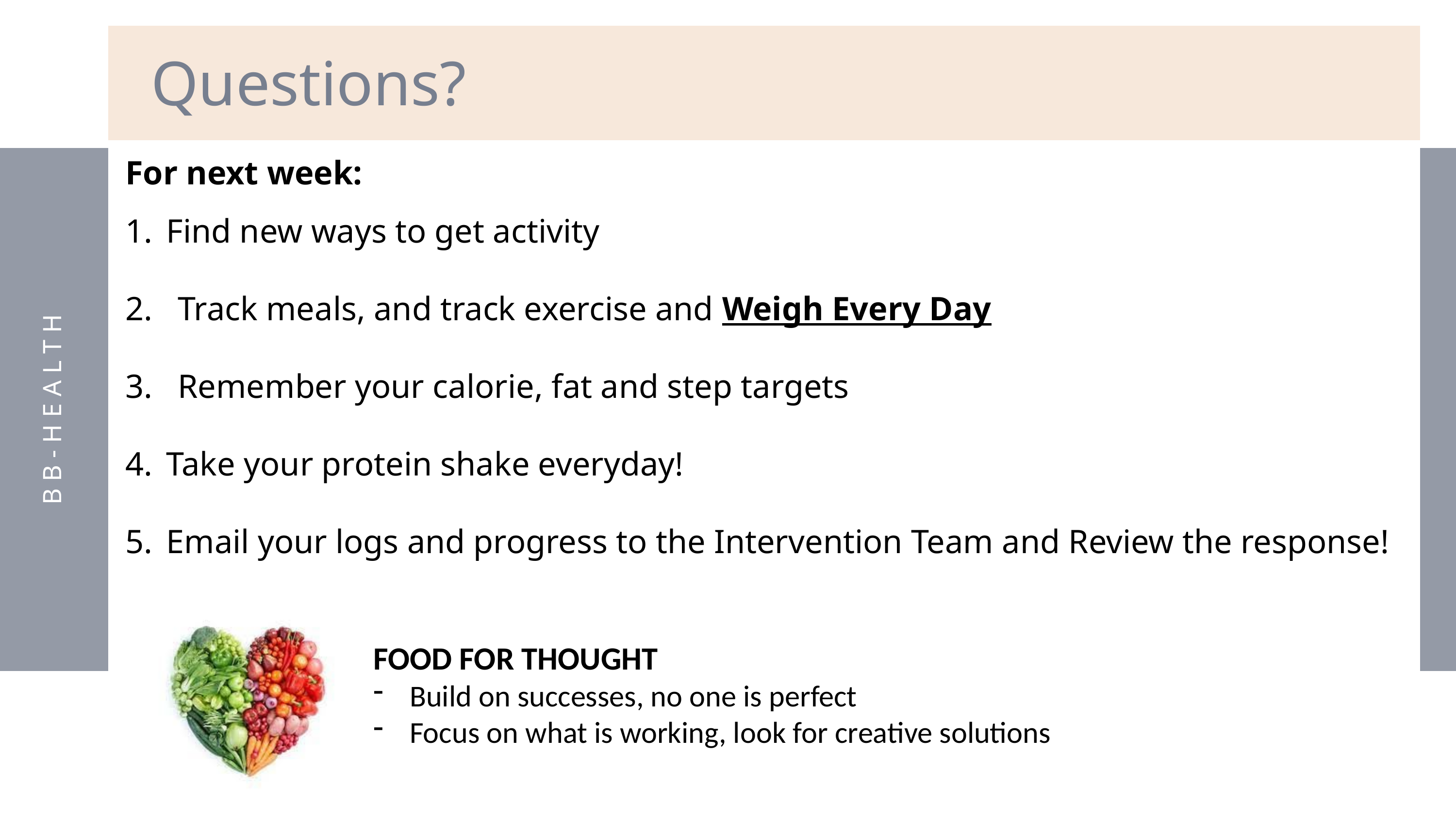

Questions?
For next week:
Find new ways to get activity
2. Track meals, and track exercise and Weigh Every Day
3. Remember your calorie, fat and step targets
Take your protein shake everyday!
Email your logs and progress to the Intervention Team and Review the response!
BB-HEALTH
FOOD FOR THOUGHT
Build on successes, no one is perfect
Focus on what is working, look for creative solutions
