# Supplementary material for: Development and Feasibility of an eHealth Diabetes Prevention Program Adapted for Older Adults—Results from a Randomized Control Pilot Study
Source: Nutrients. 2024 Mar 23;16(7):930. doi: 10.3390/nu16070930 (PMC11154527; doi:10.3390/nu16070930)
Supplement: Supplementary file 1 [file nutrients-16-00930-s001.zip › Week4_SUPP.pdf]

BB-HEALTH

e-Healthy Older Adults Modification

Diabetes Prevention Program

WEEK #4
MOVE THOSE MUSCLES!

Group Opening Discussion

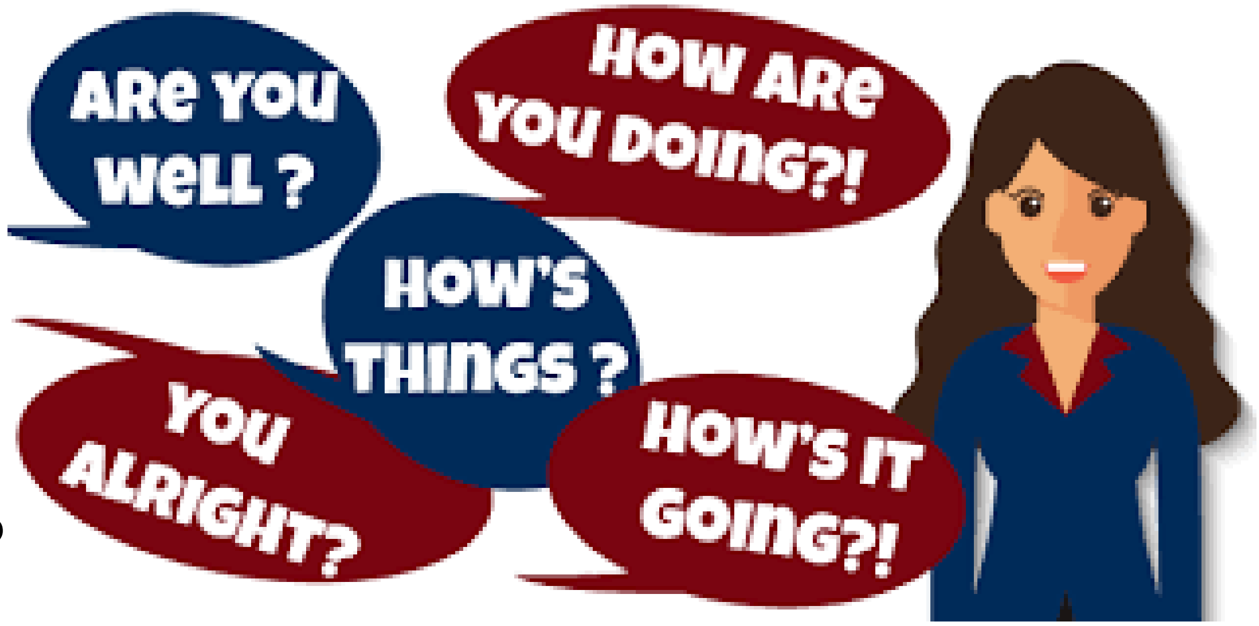

WELCOME BACK! **HOW WAS LAST WEEK**?

1. Did you track every meal?
2. Did you weigh every day, & record your weight today?
3. Did you try new veggies and proteins?
4. Did you get 5,000 steps a day?
5. Did you email the Intervention Team and review the response?
6. What were your unique challenges and successes?

REMINDER: CHARGE YOUR FITBIT & SCALE DURING THIS MEETING!

When did you eat the most fat?

<u>What was your most successful healthy swap?</u>

- Vegetables
- Fruits
- Grains
- Proteins
- Dairy Foods
- All the above
- None

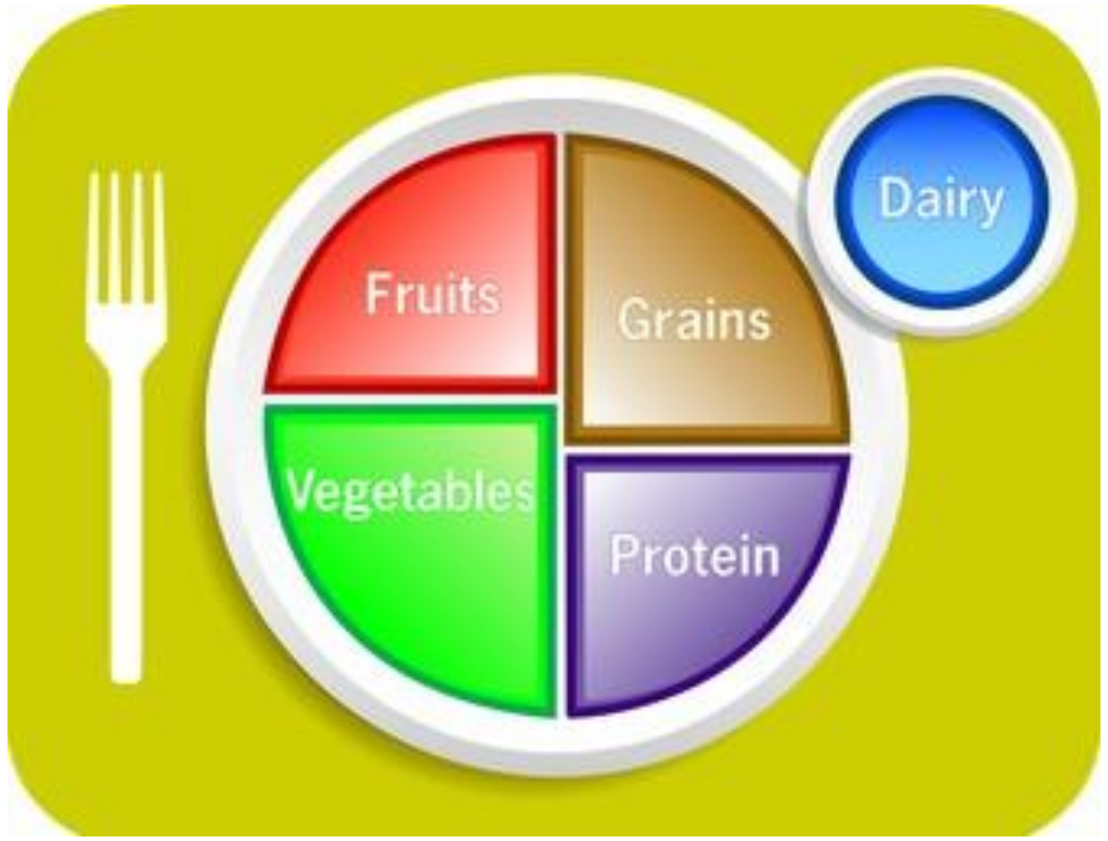

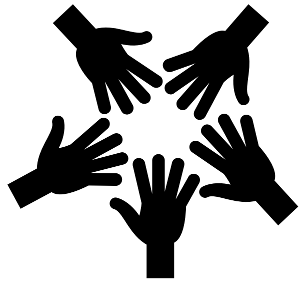

SHARE YOUR STRATEGIES FOR SUCCESS IN THE CHAT!

Found a healthy swap that worked for you? WRITE IT IN THE CHAT NOW!

From food swaps to cooking choices, if it works for your it could really help others find motivation and delicious ideas!

How do you see your success?

BB-HEALTH

What makes you feel like it is working? (select all)

- Sticking to my calorie/fat targets most meals
- Losing weight
- Having one really healthy meal a day
- Doing my 6,000 steps every day
- Having more energy
- Trying new foods
- My clothes fit better
- Skipping junk food
- My family or friends notice improvements
- Getting my steps in on most days
- Other (discuss)

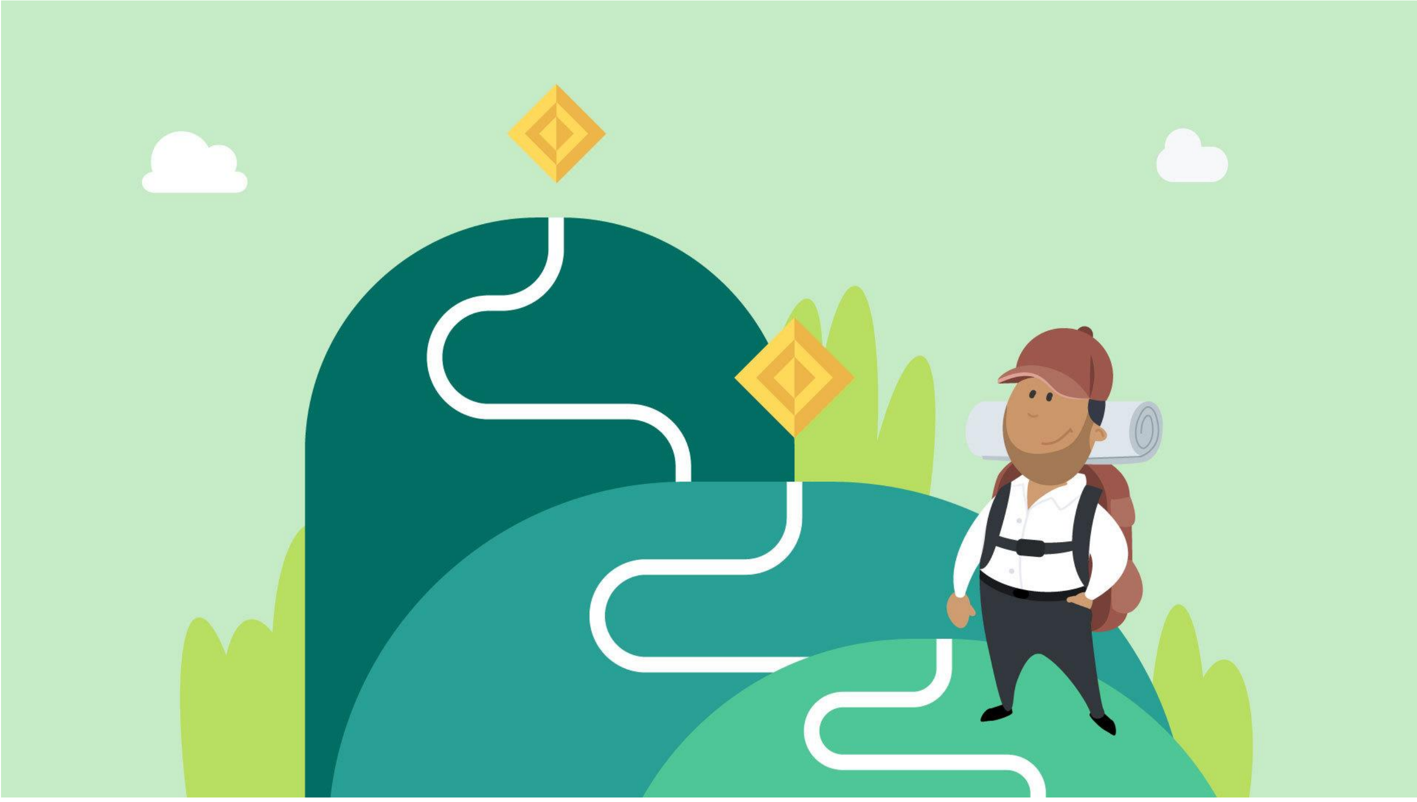

AIM FOR SUCCESS

Those milestones are important to recognize and build on!

It’s all about MOVING MORE!

BB-HEALTH

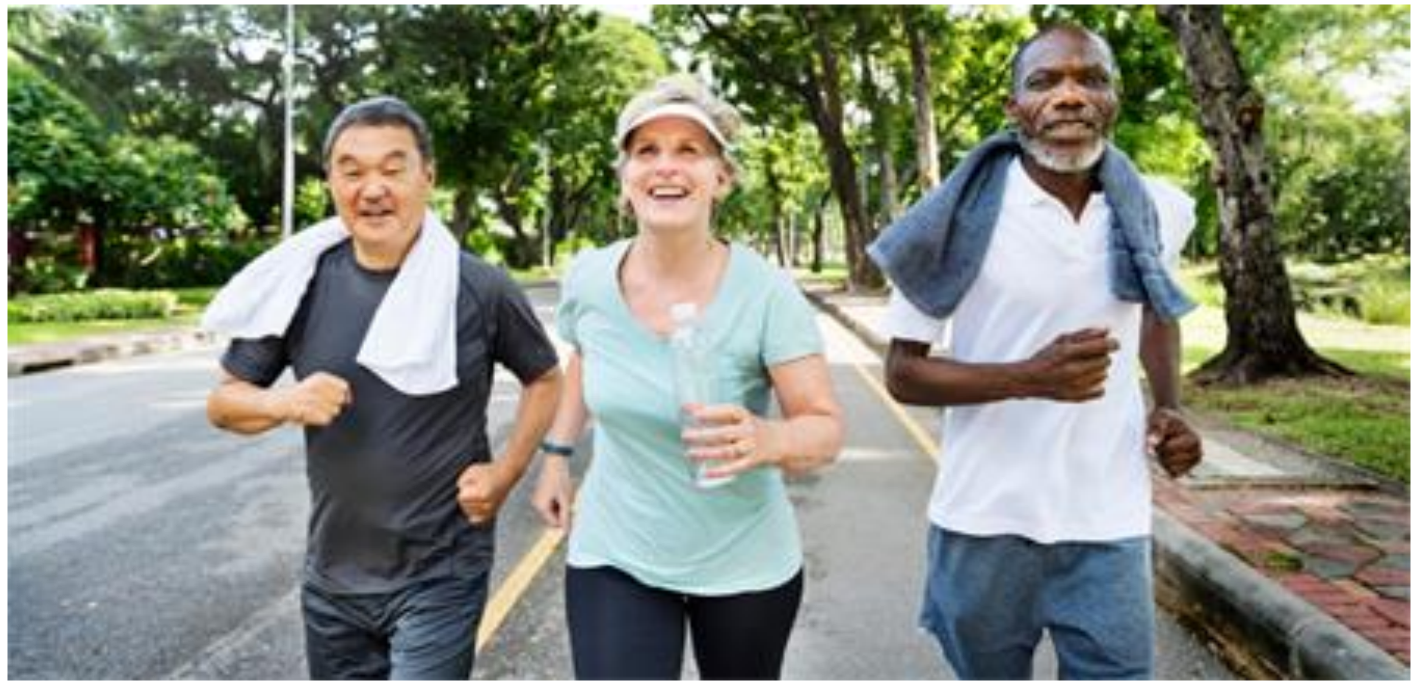

Planned activity

- MODERATE activity, like a brisk walk
- We start at 100 min a week, but aim for 150
- Only count it if it lasts 10 min or more
- Build up slow, don’t push yourself too hard
- Plan ahead- ask friends or fellow group members to go on regularly scheduled walks!

Spontaneous Activity

- Small increases like taking the stairs instead of the elevator, if you can!
- Housework and gardening is great for health and fun!
- Break up long sitting periods – walk around the house, yard, and get up to stretch
- Try standing during our meetings, or when in other meetings too!
- Cut down on TV time, or watch TV while doing chores

Why do we need to get moving?

- More energy
- Sleep Better
- Better able to chase after grandkids
- Stay independent
- Improve bone, joint, and muscle health
- Lower risk for diabetes, heart disease, and cancer
- Lower risk of dementia and Alzheimer's
- Increase mental alertness
- Lower your blood pressure
- Lower your blood glucose
- Increase insulin sensitivity

- ***Know anything else? PUT IT IN THE CHAT!***

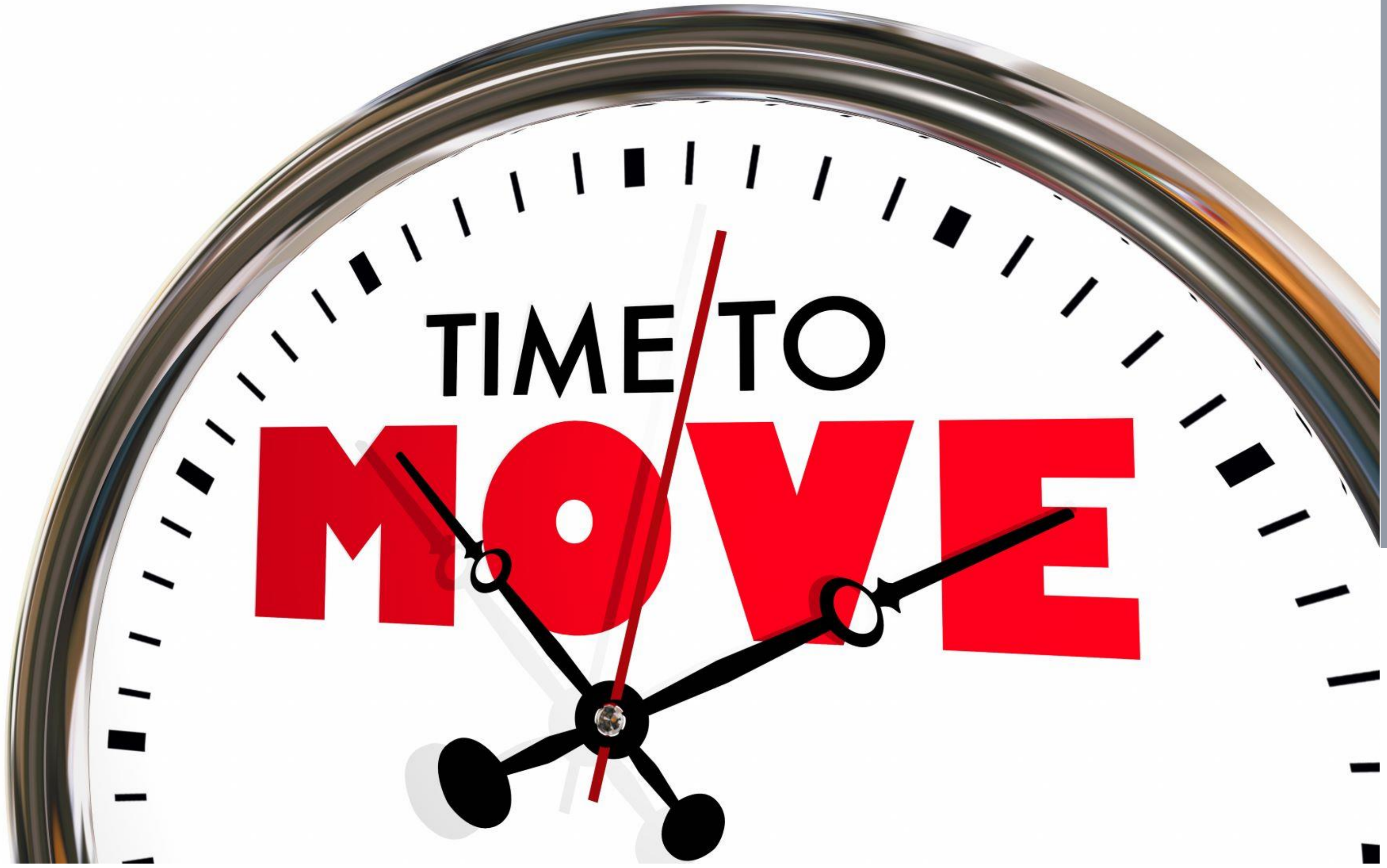

Exercise GOALS

B B - H E A L T H

- **ACHIEVABLE GOALS**
  - Started with 6,000 steps a day with 2,000+ "brisk"
  - Aiming for 150 min a week by Week 8
    - That's as little as 22 min a day!
    - Spread moderate activity over 3+ days
    - 10+ min of moderate activity at a time
  - GO SLOW! But build up over time
  - Keep up activities you like and are used to
    - **be safe and don't push too hard**
  - Aim to beat the exercise time from the previous week
    - Add 30 min each week
      - That's just 3 days with 10 min more

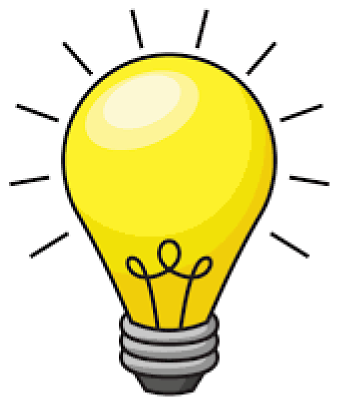

THIS WEEK'S TIP:

Check Your Breathing!

Getting your heart rate up a bit is the goal of moderate exercise, like a brisk paced walk. The best way to tell if you are getting a good pace is that your breathing is faster and its not as easy to talk while doing it!

Exercise Plan

BB-HEALTH

- Strategies for Success
  - Set aside time
    - Prioritize your health
  - Plan ahead
    - Otherwise life will interfere
  - Make it social
    - Walk with friends, family, and group members
    - Hold each other accountable
  - Record moderate activity over 10+ min
    - You deserve credit!!!
  - Make a Schedule
    - Plan ahead and get into a routine

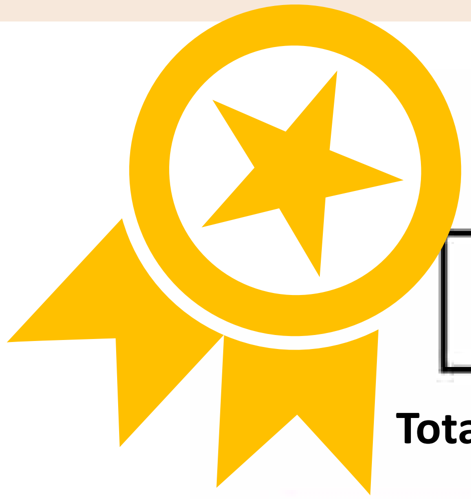

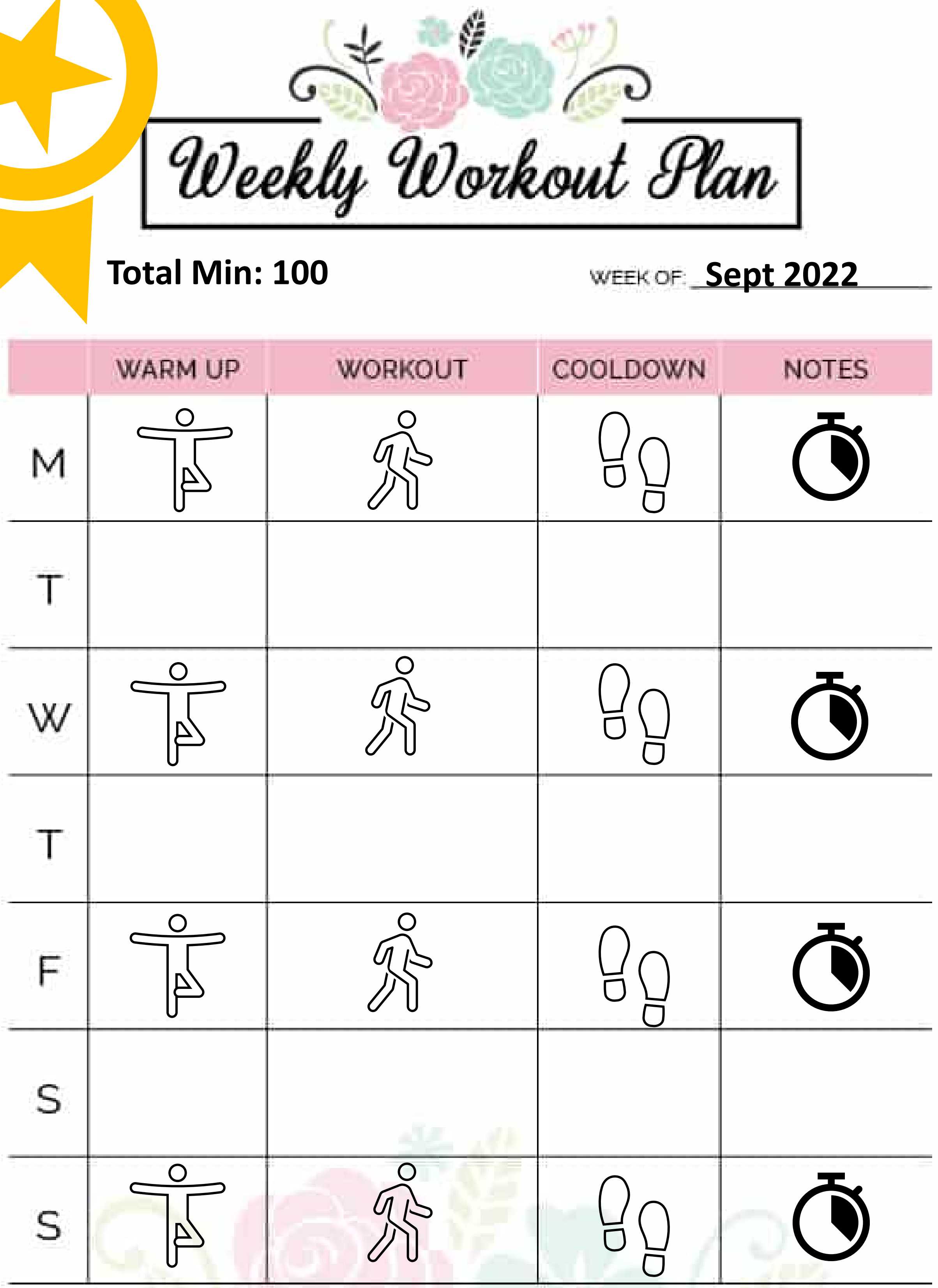
Weekly Workout Plan

Total Min: 100

WEEK OF: Sept 2022

	WARM UP	WORKOUT	COOLDOWN	NOTES
M				
T				
W				
T				
F				
S				
S				

Exercise Safety

- **EXERCISE RECIPE**
  - Warm Up for 5-10 min
    - A leisurely paced walk works well
  - Stretch for 5-10 min
    - Stretch after you get your blood flowing! Check pages 13-16 for stretch ideas!
  - Stay in Your Comfort Zone
    - Stick with what you know and what your body can handle
  - Aim for Balance
    - Build up slowly, work both sides of your body equally
  - Remember to Breathe
    - Steady rhythmic breathing makes exercise better and can tell you how strenuous it is
  - Cool down for 5-10 min
    - A relaxed version of the exercise you were doing
  - Stretch (again)
    - Stretching after a workout can help reduce soreness

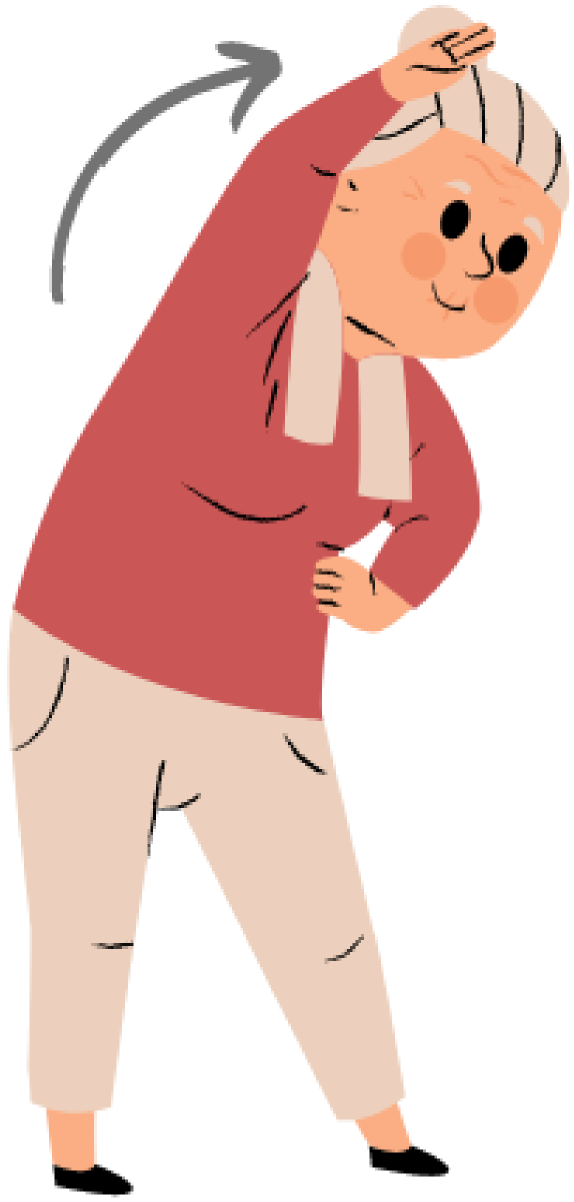

AIM FOR SUCCESS

PAIN IS NOT GAIN! Start small and build up strength. Soreness is ok but PAINFUL is a sign to stop. If pain persists, please seek medical attention and let us know too! **Review page 10 to know when to stop and what to look for to stay safe!**

Make the active choice

- Be AWARE of active opportunities
  - Small choices add up
  - A little goes a long way
- CONTROL your sitting time
  - Break up sedentary time
  - Get up and walk around 30-45 min
  - Cut down on screen & TV time
- Choose to move
  - Take the stairs
  - Walk instead of drive
  - Walk around during commercial breaks
  - Park in a far away spot

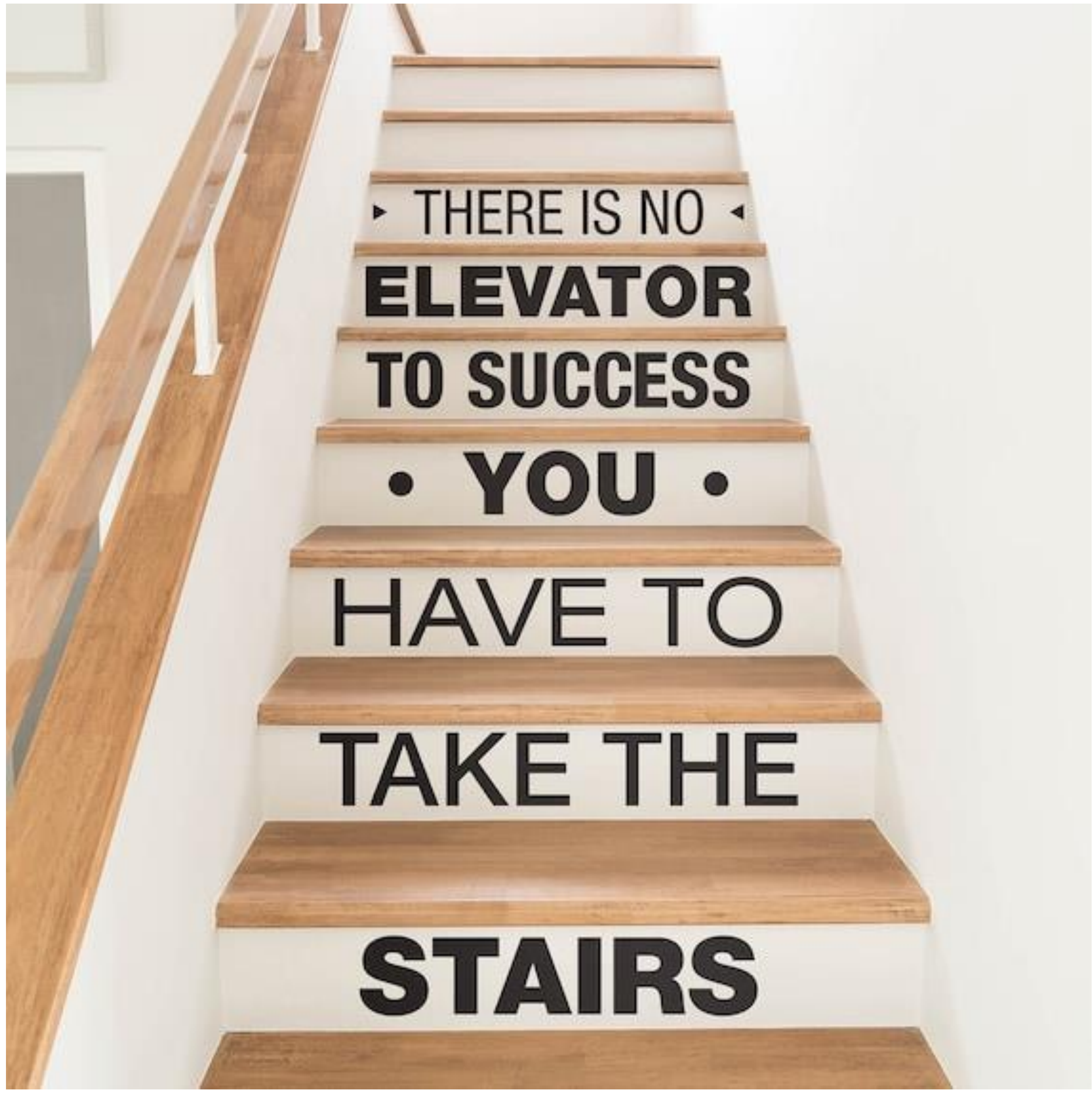

Activities I can choose

BB-HEALTH

<u>Spontaneous activities I can do (select all)</u>

- ○ Park in a far away spot
- ○ Take the long way back on a walk
- ○ Jog in place during commercial breaks
- ○ Make a walking date with a friend
- ○ Run errands while walking
- ○ Take an extra loop or two in the grocery store
- ○ Walk a neighbor's dog
- ○ All the above
- ○ Other (discuss)

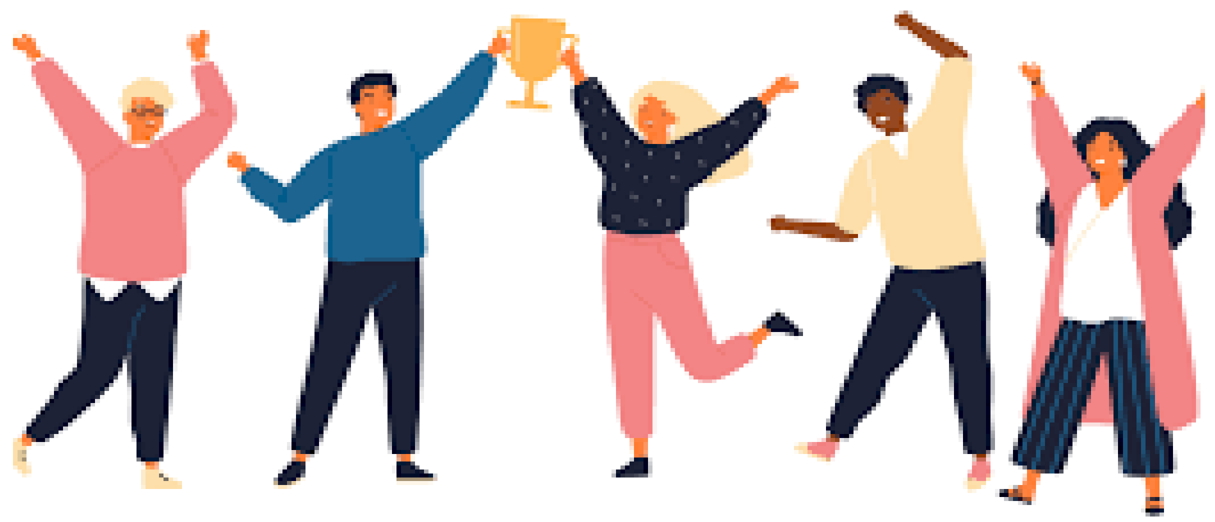

Put it into Practice

Keeping Track of What You Eat

1. Track those meals and take your weight EVERYDAY!
   - Monitor your progress and identify areas for improvement towards YOUR GOALS

2. Ask yourself- can I do another 10 min of exercise today?
   - Aim to do 30 min more this week than last week (a minimum of 60 min)
   - Use your activity planner and record your 10min+ successes!

3. Give your recipes and meals a healthy make-over!
   - Pages 12-14 of the handout gives tips to improve your meal plan!
     - Healthier snacks, ways to season food to make it taste delicious, cooking methods and more!

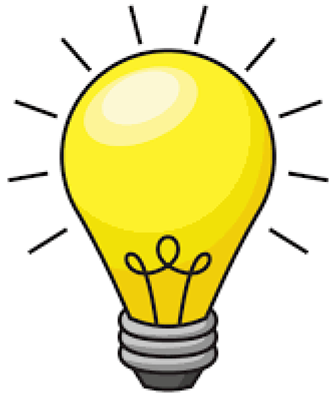

REMEMBER THIS WEEK'S TIP: <u>Check Your Breathing!</u>
Getting your heart rate up a bit is the goal of moderate exercise, like a brisk paced walk. The best way to tell if you are getting a good pace is that your breathing is faster and its not as easy to talk while doing it!

Questions?

B B - H E A L T H

For next week:

1. Find new ways to get activity

2. Track meals, and track exercise and **Weigh Every Day**

3. Remember your calorie, fat and step targets

4. Take your protein shake everyday!

5. Email your logs and progress to the Intervention Team and Review the response!

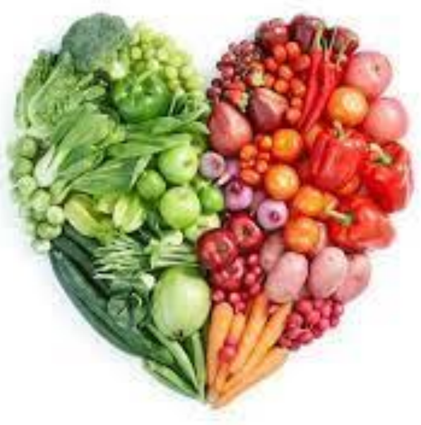

FOOD FOR THOUGHT

- Build on successes, no one is perfect
- Focus on what is working, look for creative solutions
