# Supplementary material for: Development and Feasibility of an eHealth Diabetes Prevention Program Adapted for Older Adults—Results from a Randomized Control Pilot Study
Source: Nutrients. 2024 Mar 23;16(7):930. doi: 10.3390/nu16070930 (PMC11154527; doi:10.3390/nu16070930)
Supplement: Supplementary file 1 [file nutrients-16-00930-s001.zip › Week5.pptx]

## Slide 1
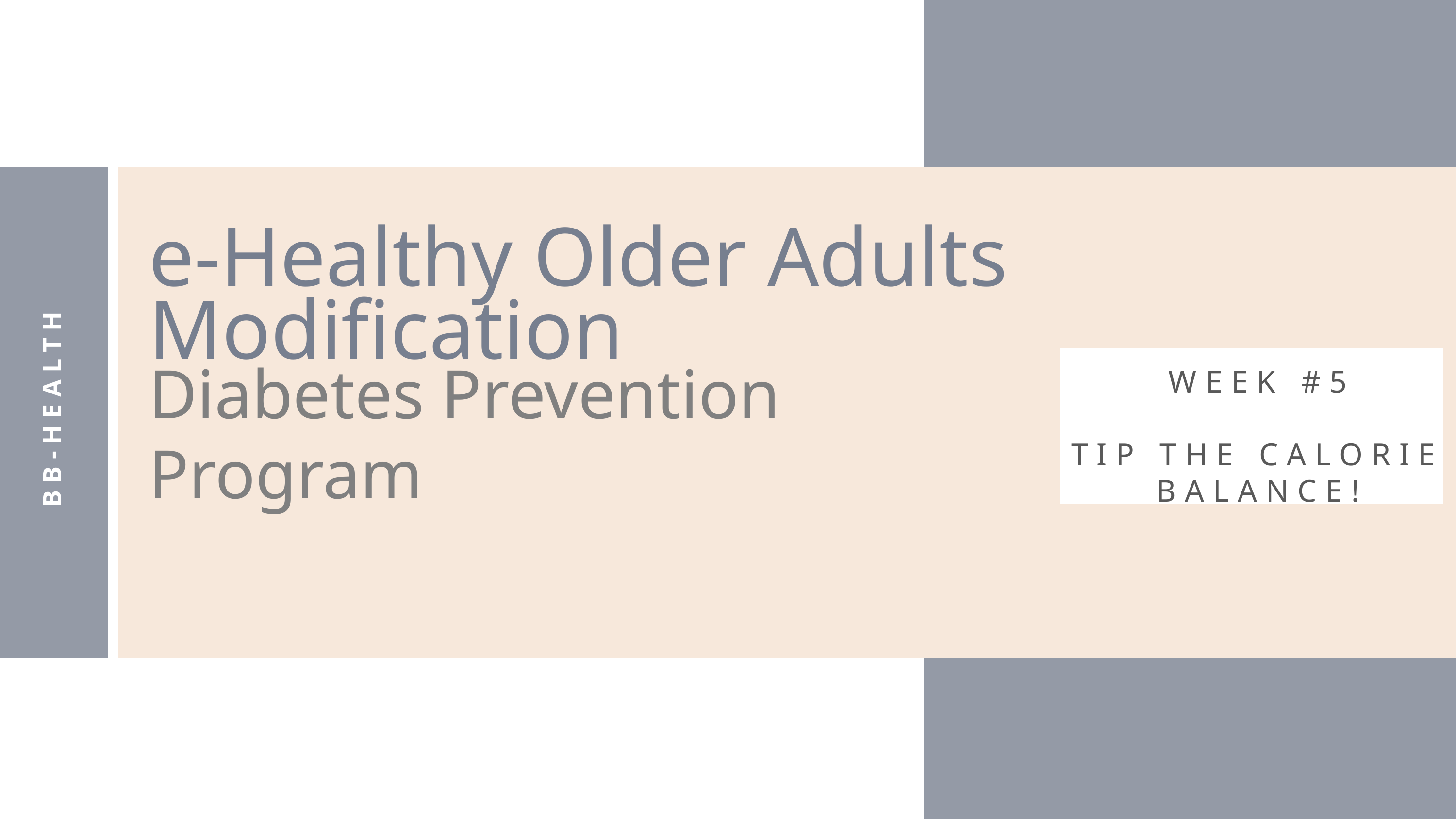

OPEN REPORTS
e-Healthy Older Adults Modification
WEEK #5
TIP THE CALORIE BALANCE!
Diabetes Prevention Program
BB-HEALTH

## Slide 2
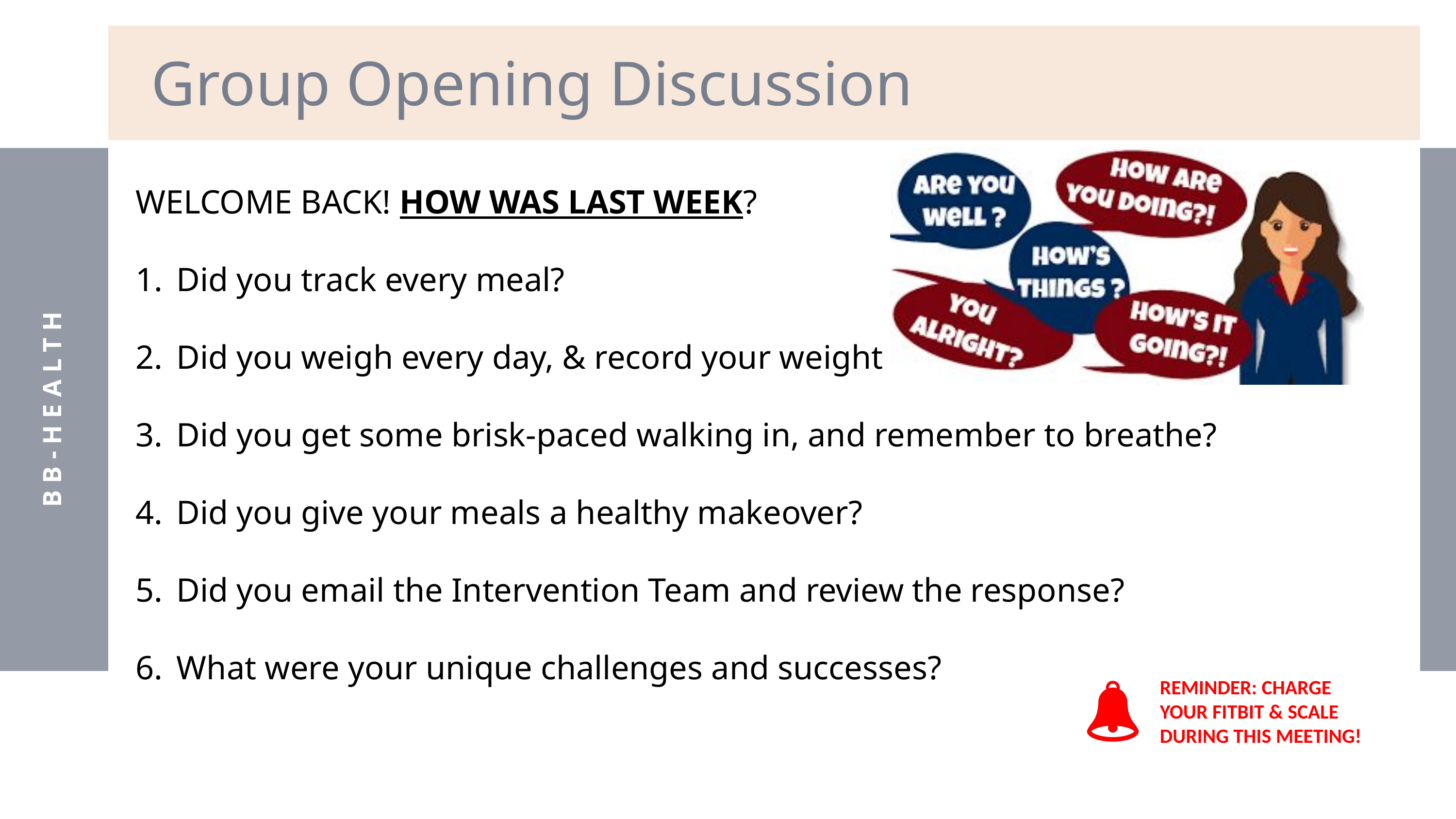

Group Opening Discussion
WELCOME BACK! HOW WAS LAST WEEK?
Did you track every meal?
Did you weigh every day, & record your weight today?
Did you get some brisk-paced walking in, and remember to breathe?
Did you give your meals a healthy makeover?
Did you email the Intervention Team and review the response?
What were your unique challenges and successes?
BB-HEALTH
REMINDER: CHARGE YOUR FITBIT & SCALE DURING THIS MEETING!

## Slide 3
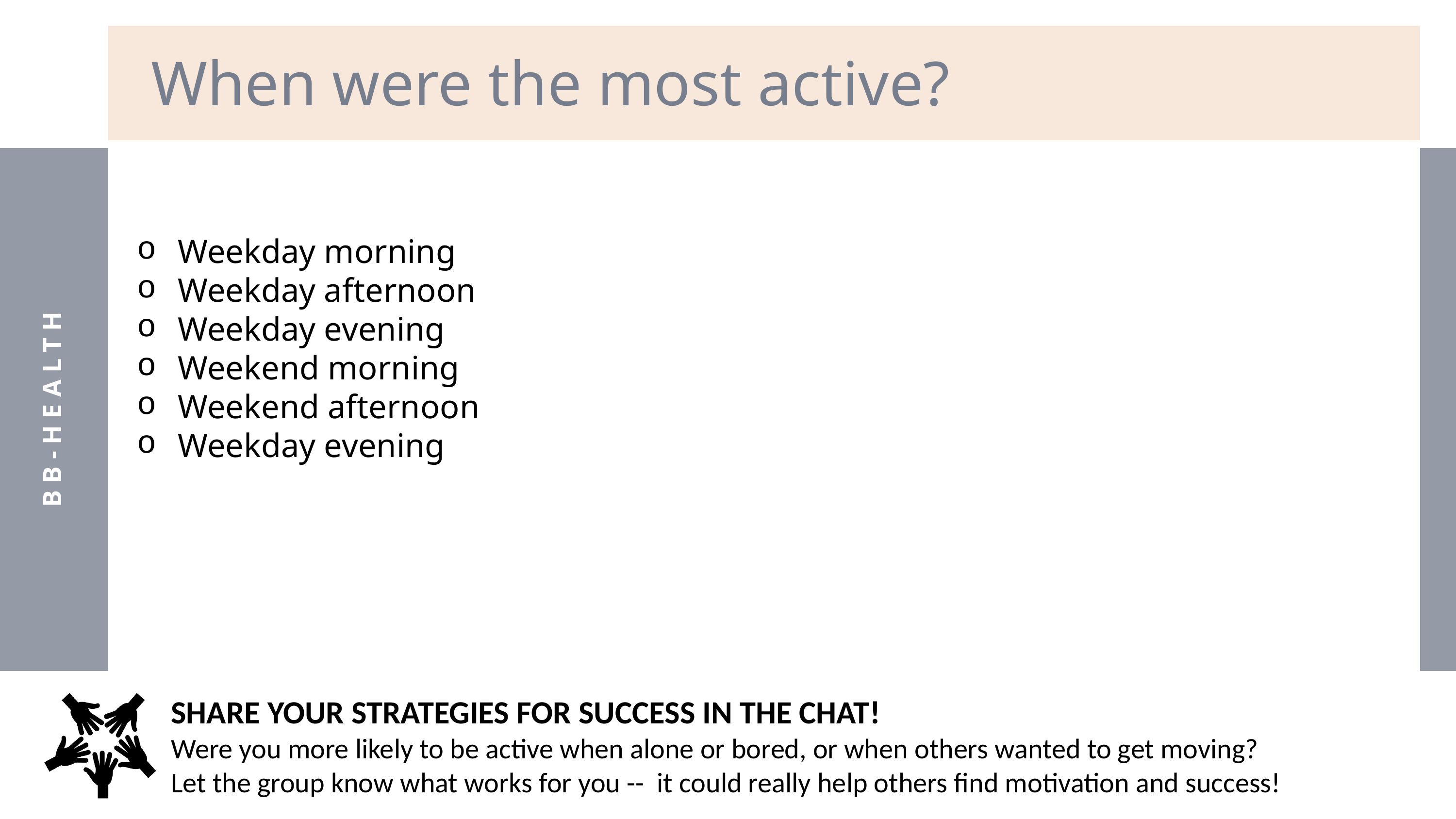

When were the most active?
Weekday morning
Weekday afternoon
Weekday evening
Weekend morning
Weekend afternoon
Weekday evening
BB-HEALTH
SHARE YOUR STRATEGIES FOR SUCCESS IN THE CHAT!
Were you more likely to be active when alone or bored, or when others wanted to get moving?
Let the group know what works for you -- it could really help others find motivation and success!

## Slide 4
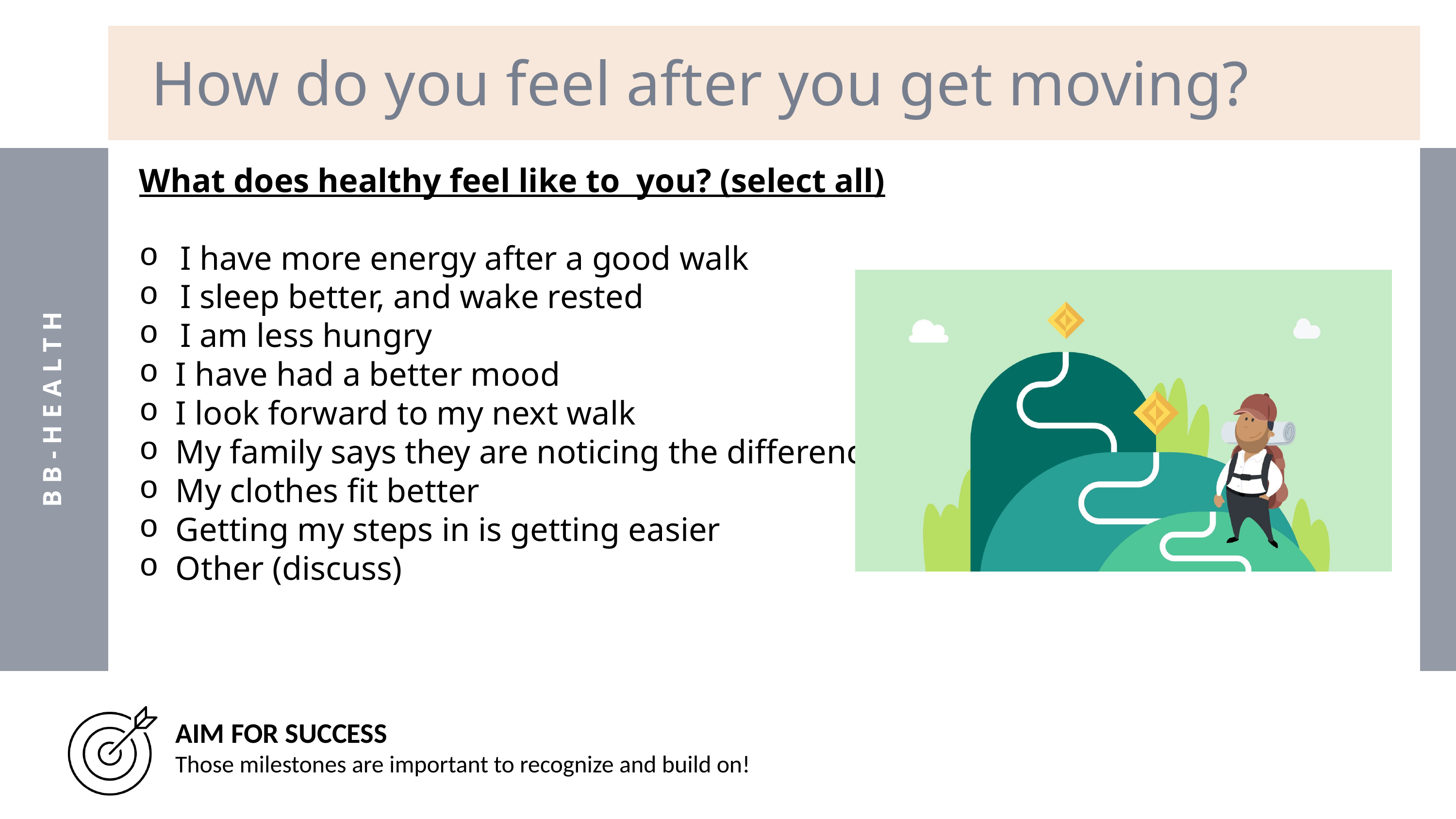

How do you feel after you get moving?
What does healthy feel like to you? (select all)
I have more energy after a good walk
I sleep better, and wake rested
I am less hungry
I have had a better mood
I look forward to my next walk
My family says they are noticing the difference
My clothes fit better
Getting my steps in is getting easier
Other (discuss)
BB-HEALTH
AIM FOR SUCCESS
Those milestones are important to recognize and build on!

## Slide 5
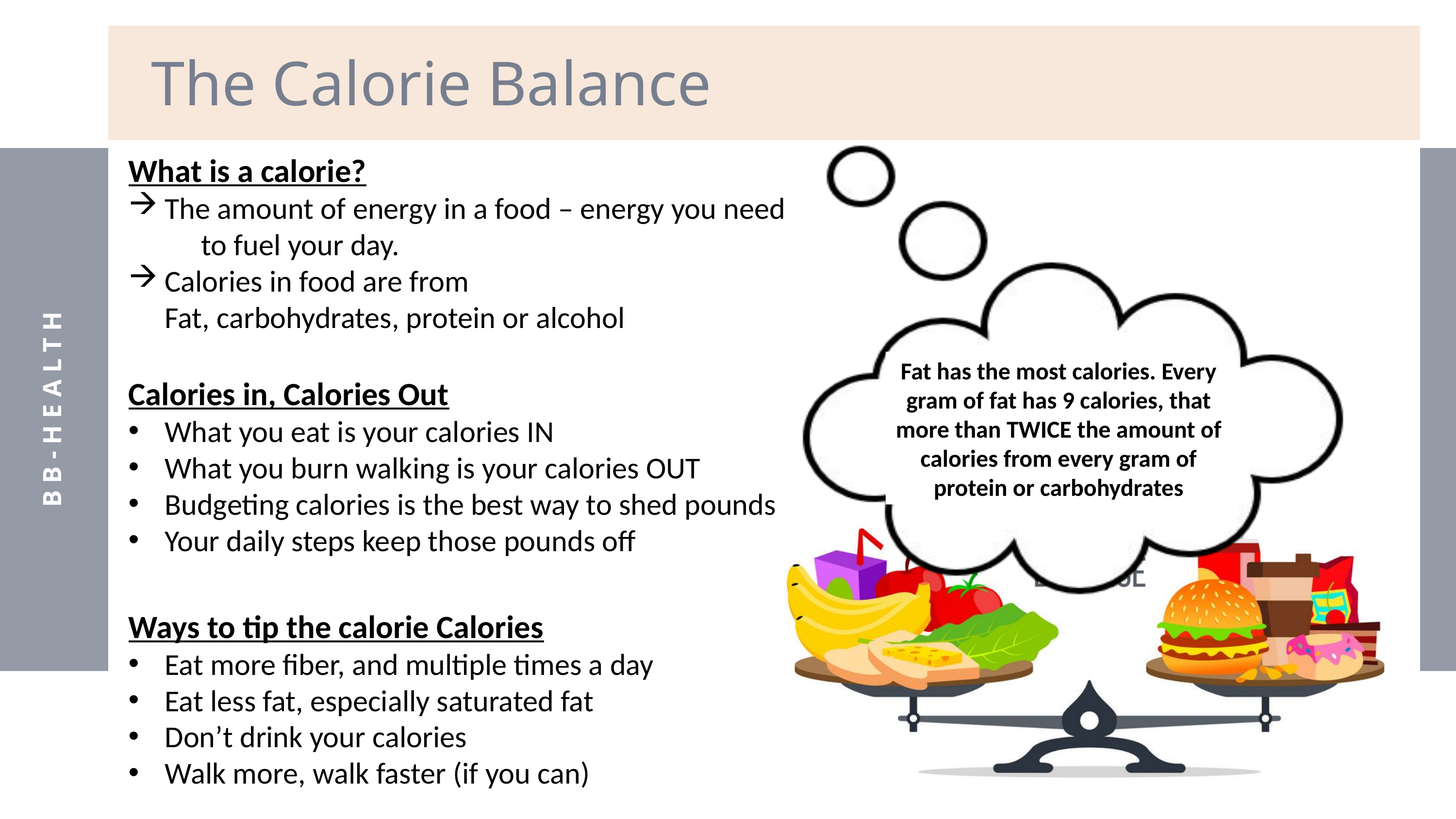

The Calorie Balance
What is a calorie?
The amount of energy in a food – energy you need
	to fuel your day.
Calories in food are from
Fat, carbohydrates, protein or alcohol
Calories in, Calories Out
What you eat is your calories IN
What you burn walking is your calories OUT
Budgeting calories is the best way to shed pounds
Your daily steps keep those pounds off
Ways to tip the calorie Calories
Eat more fiber, and multiple times a day
Eat less fat, especially saturated fat
Don’t drink your calories
Walk more, walk faster (if you can)
Fat has the most calories. Every gram of fat has 9 calories, that more than TWICE the amount of calories from every gram of protein or carbohydrates
BB-HEALTH

## Slide 6
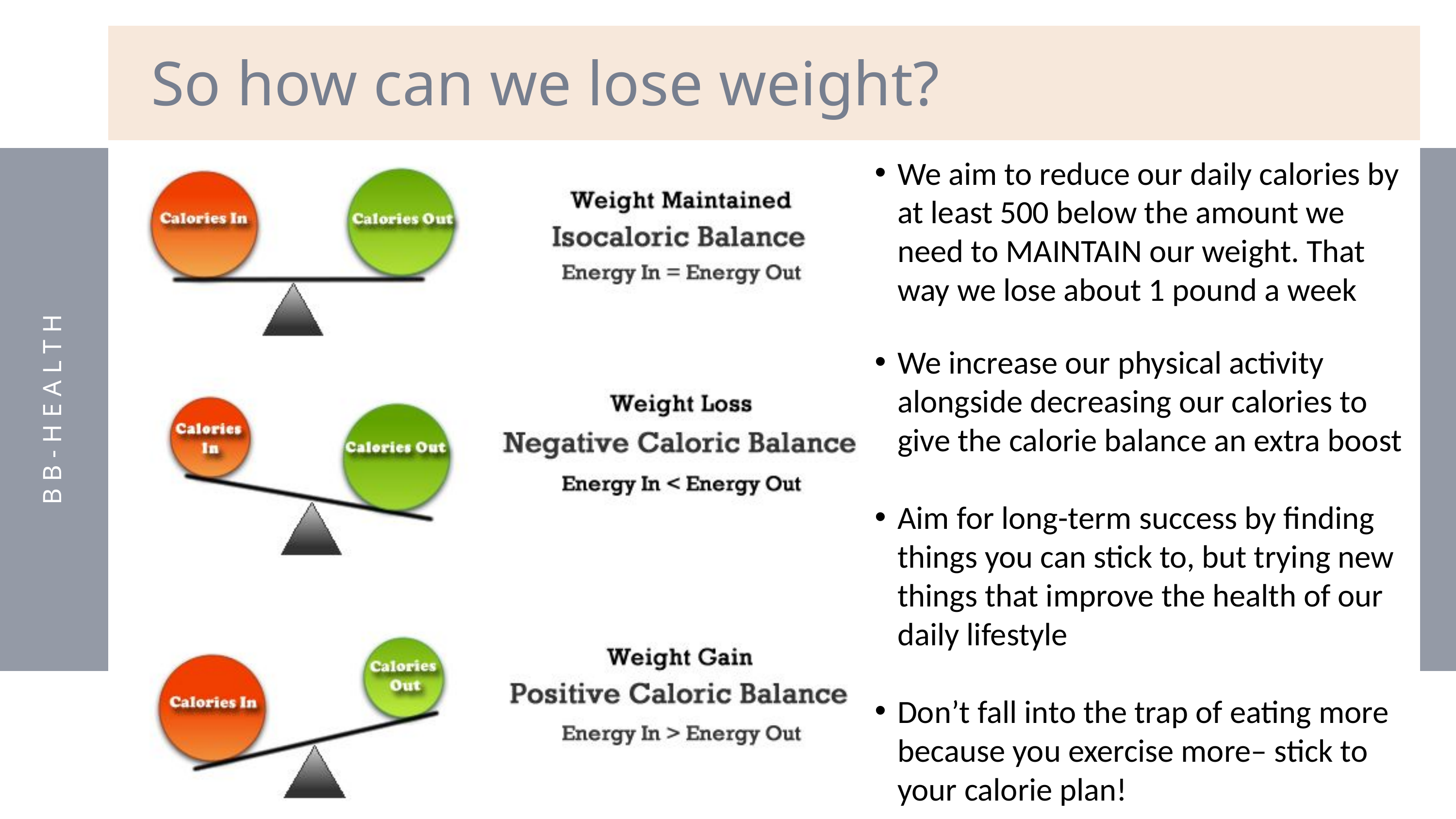

So how can we lose weight?
We aim to reduce our daily calories by at least 500 below the amount we need to MAINTAIN our weight. That way we lose about 1 pound a week
We increase our physical activity alongside decreasing our calories to give the calorie balance an extra boost
Aim for long-term success by finding things you can stick to, but trying new things that improve the health of our daily lifestyle
Don’t fall into the trap of eating more because you exercise more– stick to your calorie plan!
BB-HEALTH

## Slide 7
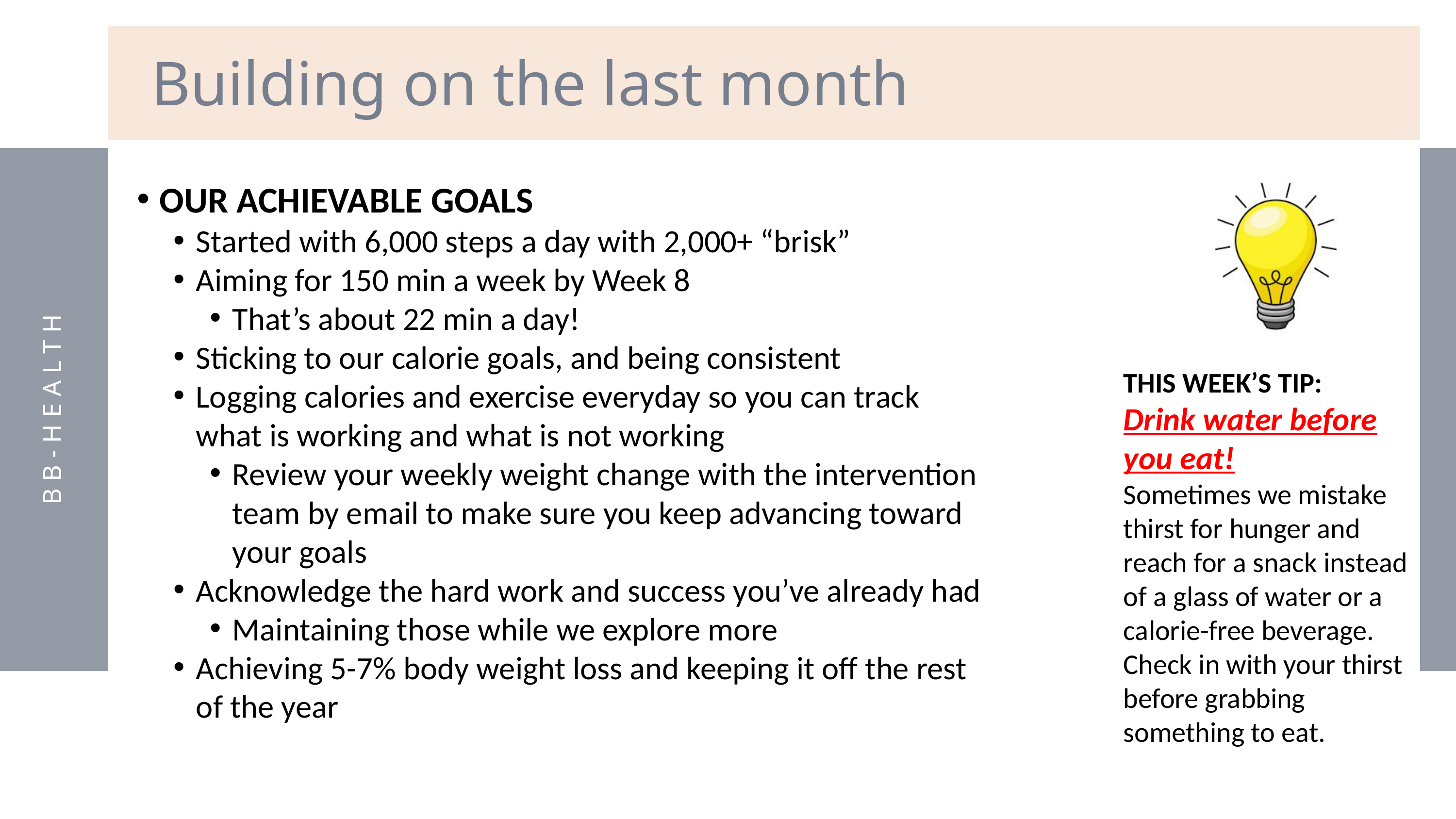

Building on the last month
OUR ACHIEVABLE GOALS
Started with 6,000 steps a day with 2,000+ “brisk”
Aiming for 150 min a week by Week 8
That’s about 22 min a day!
Sticking to our calorie goals, and being consistent
Logging calories and exercise everyday so you can track what is working and what is not working
Review your weekly weight change with the intervention team by email to make sure you keep advancing toward your goals
Acknowledge the hard work and success you’ve already had
Maintaining those while we explore more
Achieving 5-7% body weight loss and keeping it off the rest of the year
THIS WEEK’S TIP:
Drink water before you eat!
Sometimes we mistake thirst for hunger and reach for a snack instead of a glass of water or a calorie-free beverage. Check in with your thirst before grabbing something to eat.
BB-HEALTH

## Slide 8
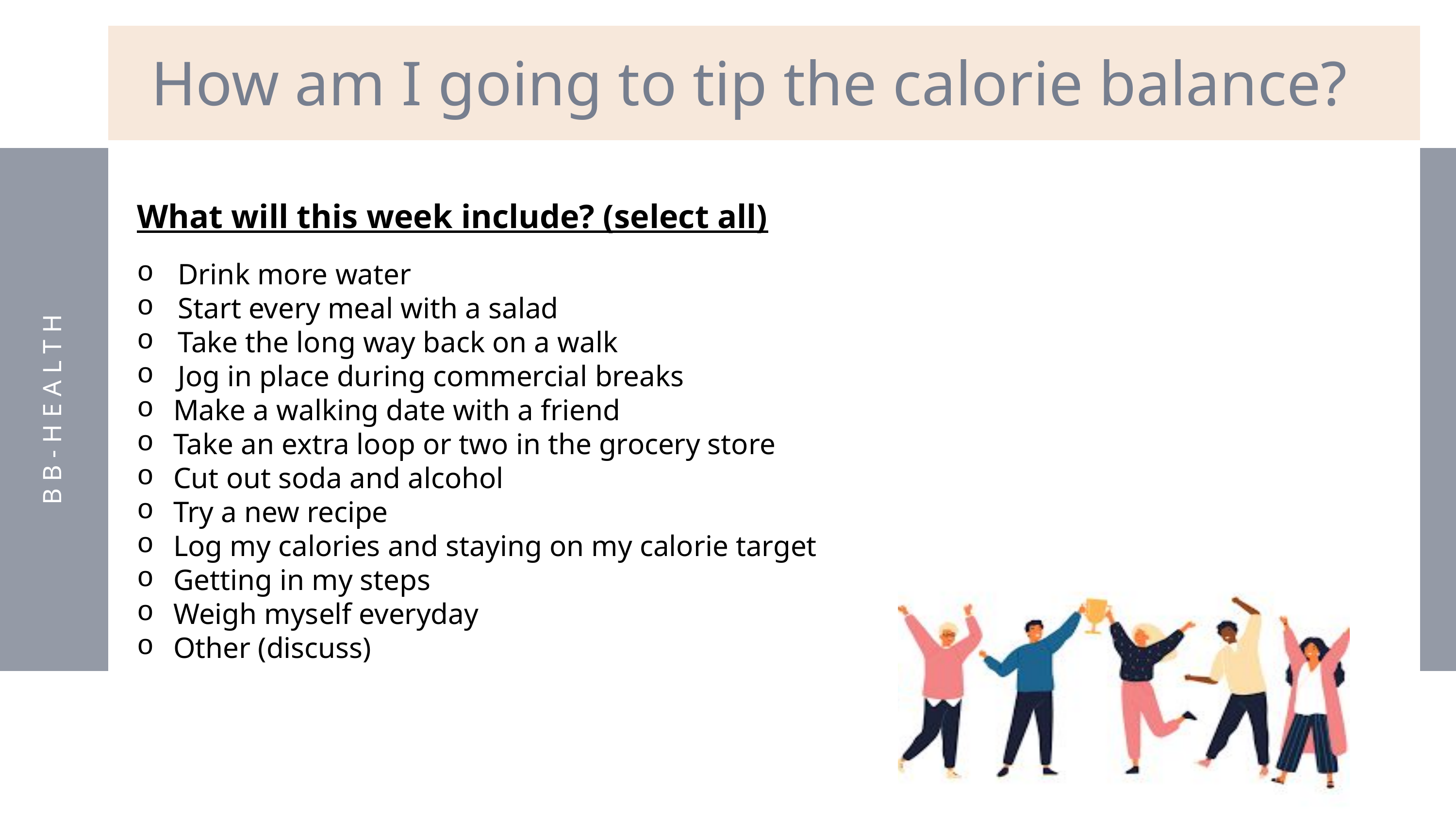

How am I going to tip the calorie balance?
What will this week include? (select all)
Drink more water
Start every meal with a salad
Take the long way back on a walk
Jog in place during commercial breaks
Make a walking date with a friend
Take an extra loop or two in the grocery store
Cut out soda and alcohol
Try a new recipe
Log my calories and staying on my calorie target
Getting in my steps
Weigh myself everyday
Other (discuss)
BB-HEALTH

## Slide 9
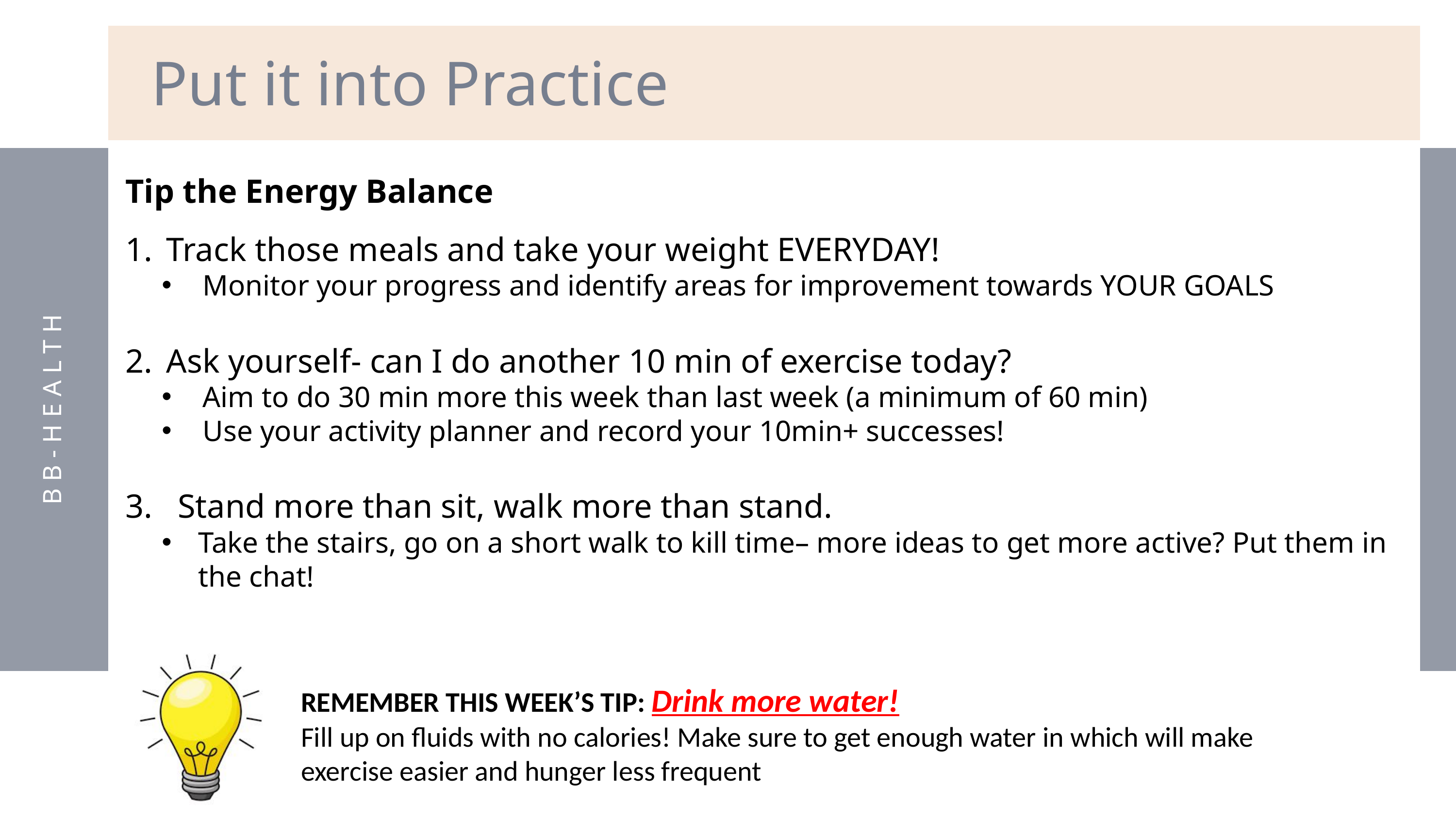

Put it into Practice
Tip the Energy Balance
Track those meals and take your weight EVERYDAY!
Monitor your progress and identify areas for improvement towards YOUR GOALS
Ask yourself- can I do another 10 min of exercise today?
Aim to do 30 min more this week than last week (a minimum of 60 min)
Use your activity planner and record your 10min+ successes!
3. Stand more than sit, walk more than stand.
Take the stairs, go on a short walk to kill time– more ideas to get more active? Put them in the chat!
BB-HEALTH
REMEMBER THIS WEEK’S TIP: Drink more water!
Fill up on fluids with no calories! Make sure to get enough water in which will make exercise easier and hunger less frequent

## Slide 10
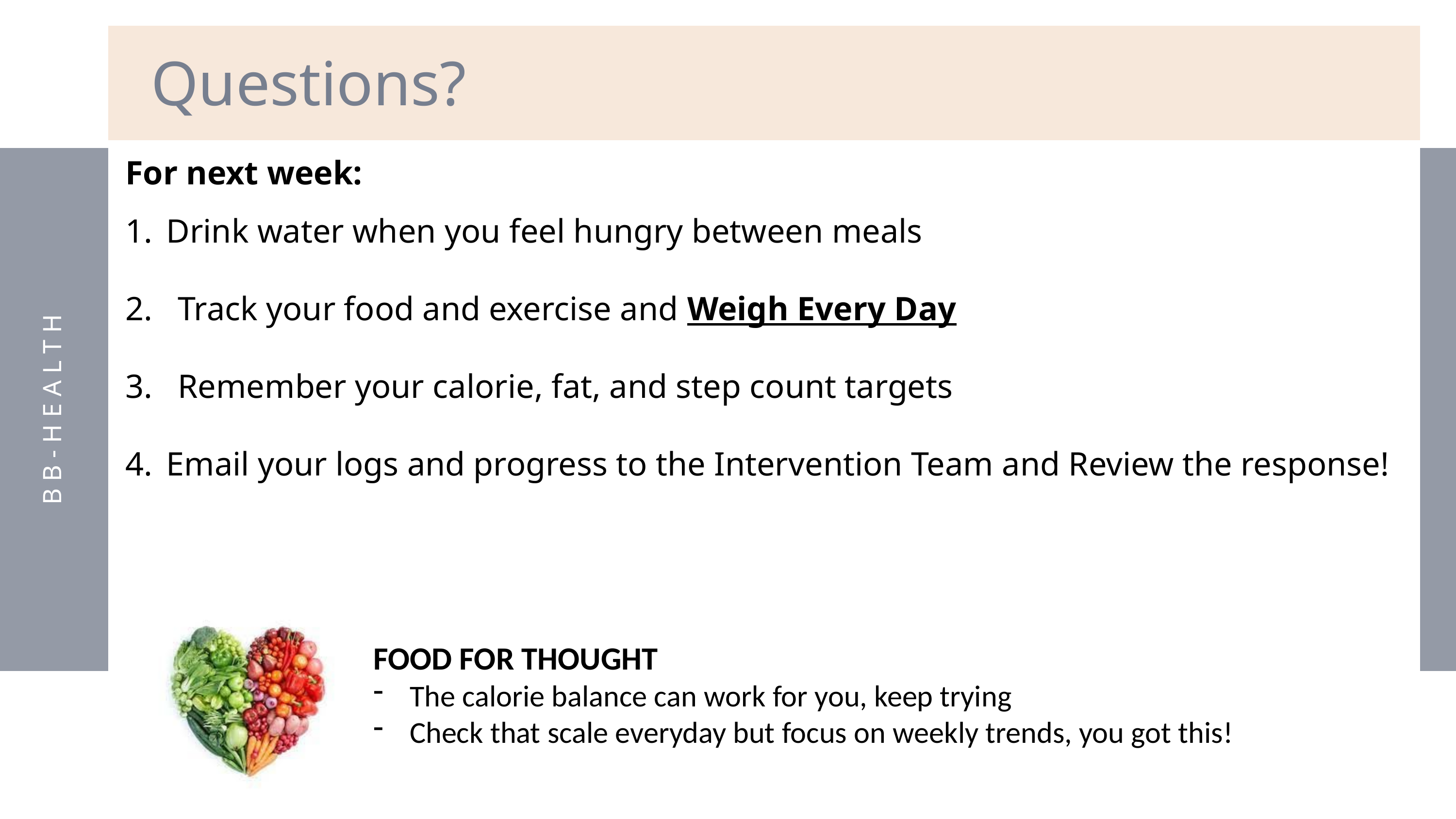

Questions?
For next week:
Drink water when you feel hungry between meals
2. Track your food and exercise and Weigh Every Day
3. Remember your calorie, fat, and step count targets
Email your logs and progress to the Intervention Team and Review the response!
BB-HEALTH
FOOD FOR THOUGHT
The calorie balance can work for you, keep trying
Check that scale everyday but focus on weekly trends, you got this!
