# Supplementary material for: Development and Feasibility of an eHealth Diabetes Prevention Program Adapted for Older Adults—Results from a Randomized Control Pilot Study
Source: Nutrients. 2024 Mar 23;16(7):930. doi: 10.3390/nu16070930 (PMC11154527; doi:10.3390/nu16070930)
Supplement: Supplementary file 1 [file nutrients-16-00930-s001.zip › Week5_SUPP.pdf]

e-Healthy Older Adults Modification

Diabetes Prevention Program

WEEK #5

TIP THE CALORIE BALANCE!

Group Opening Discussion

BB-HEALTH

WELCOME BACK! **HOW WAS LAST WEEK**?

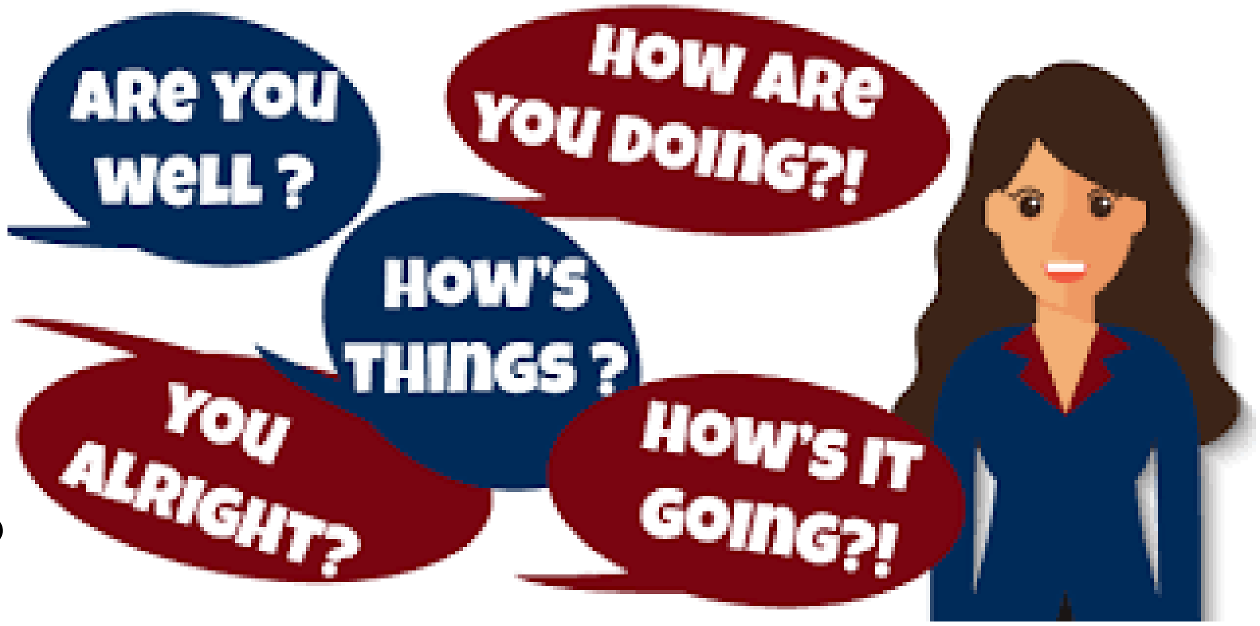

1. Did you track every meal?
2. Did you weigh every day, & record your weight today?
3. Did you get some brisk-paced walking in, and remember to breathe?
4. Did you give your meals a healthy makeover?
5. Did you email the Intervention Team and review the response?
6. What were your unique challenges and successes?

REMINDER: CHARGE YOUR FITBIT & SCALE DURING THIS MEETING!

When were the most active?

B B - H E A L T H

- Weekday morning
- Weekday afternoon
- Weekday evening
- Weekend morning
- Weekend afternoon
- Weekday evening

SHARE YOUR STRATEGIES FOR SUCCESS IN THE CHAT!

Were you more likely to be active when alone or bored, or when others wanted to get moving?
Let the group know what works for you -- it could really help others find motivation and success!

How do you feel after you get moving?

<u>What does healthy feel like to you? (select all)</u>

- I have more energy after a good walk
- I sleep better, and wake rested
- I am less hungry
- I have had a better mood
- I look forward to my next walk
- My family says they are noticing the difference
- My clothes fit better
- Getting my steps in is getting easier
- Other (discuss)

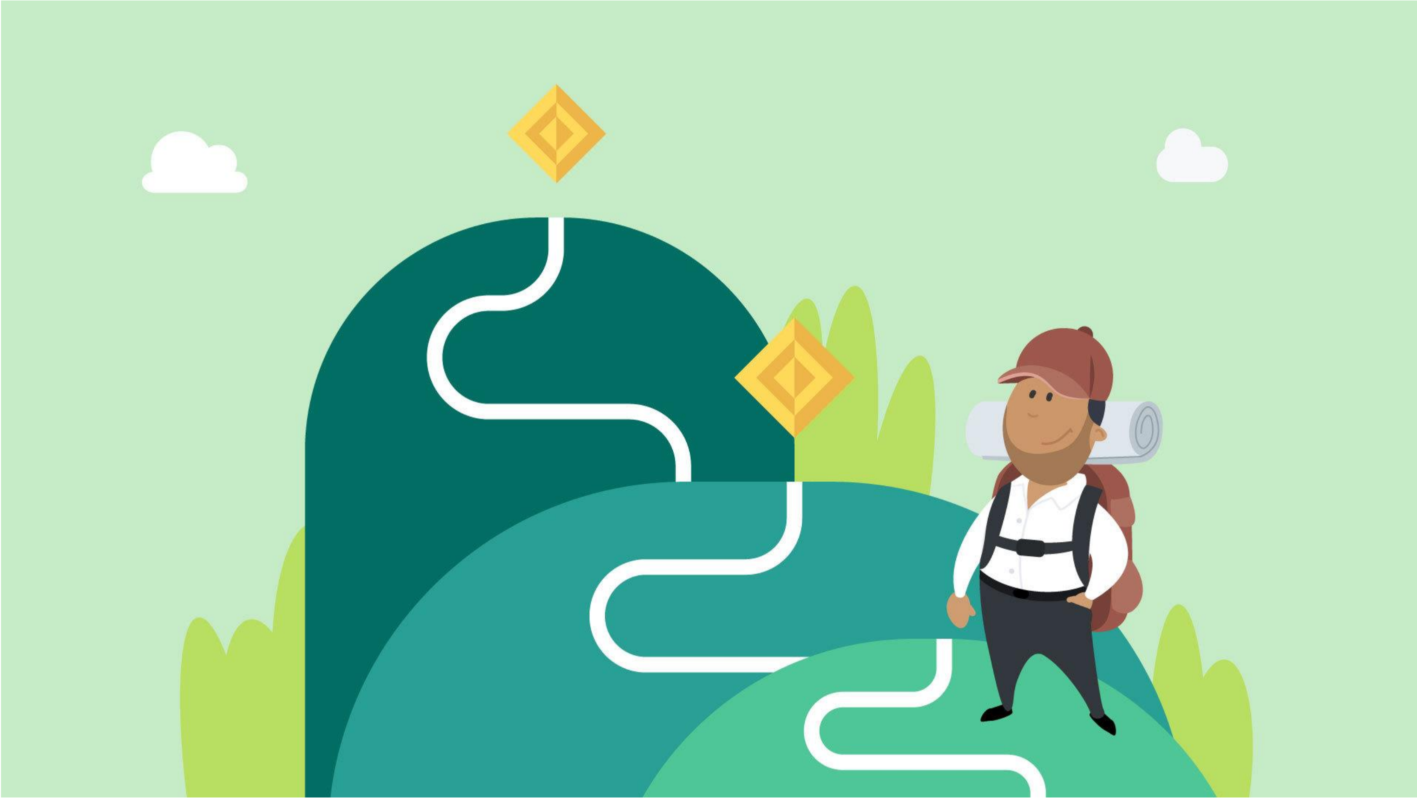

AIM FOR SUCCESS

Those milestones are important to recognize and build on!

The Calorie Balance

BB-HEALTH

What is a calorie?

→ The amount of energy in a food – energy you need to fuel your day.

→ Calories in food are from Fat, carbohydrates, protein or alcohol

Calories in, Calories Out

- What you eat is your calories IN
- What you burn walking is your calories OUT
- Budgeting calories is the best way to shed pounds
- Your daily steps keep those pounds off

Ways to tip the calorie Calories

- Eat more fiber, and multiple times a day
- Eat less fat, especially saturated fat
- Don’t drink your calories
- Walk more, walk faster (if you can)

Fat has the most calories. Every gram of fat has 9 calories, that more than TWICE the amount of calories from every gram of protein or carbohydrates

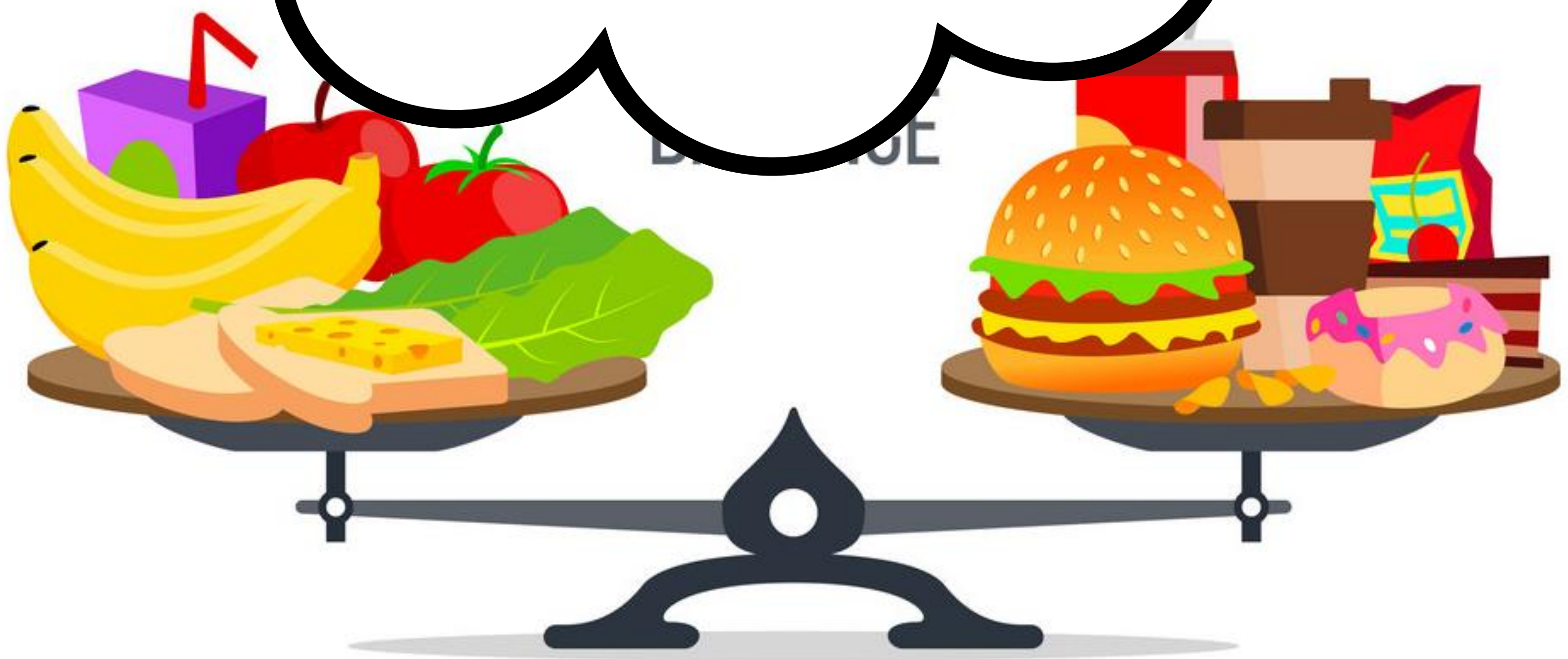

So how can we lose weight?

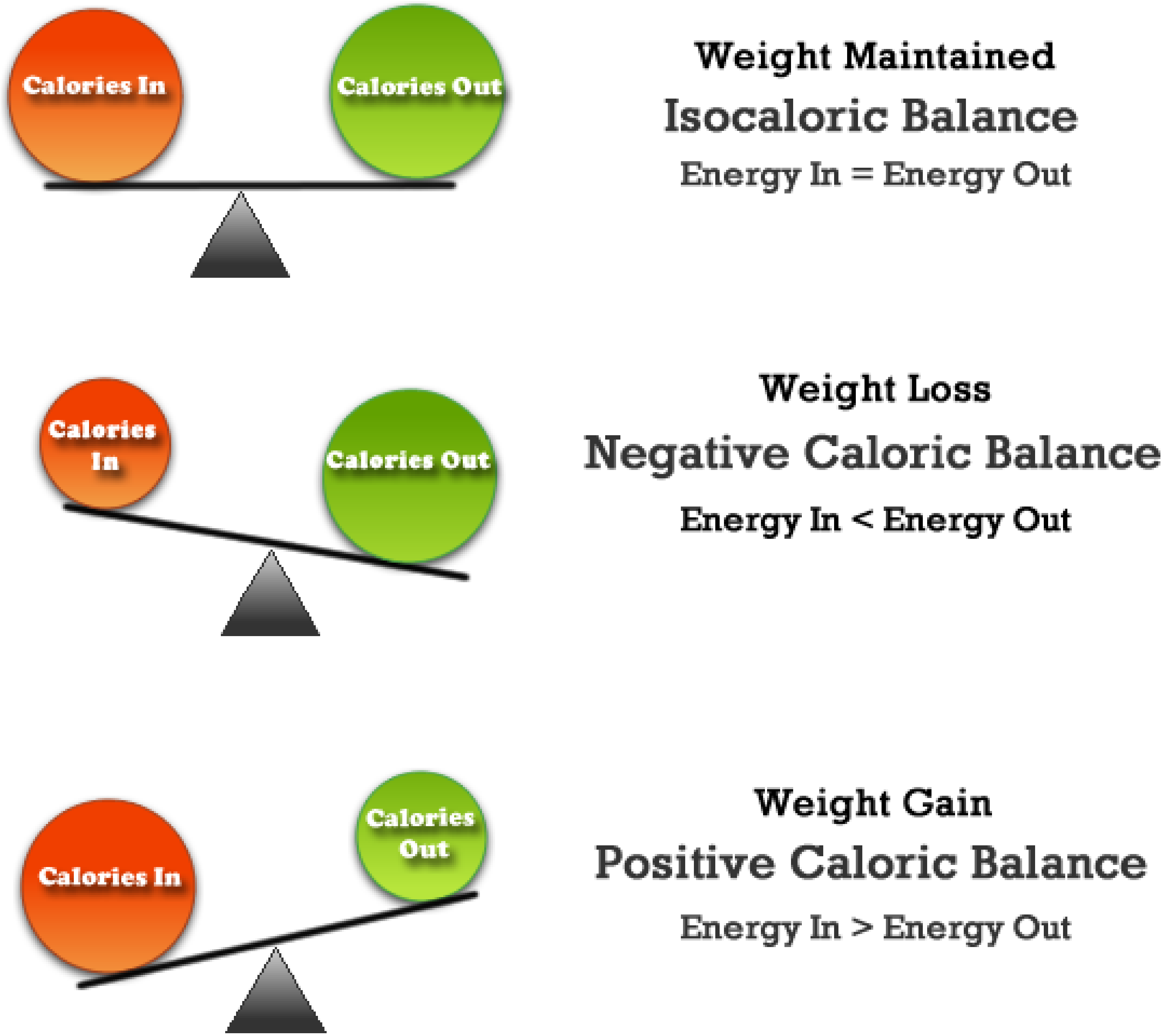

- We aim to reduce our daily calories by at least 500 below the amount we need to MAINTAIN our weight. That way we lose about 1 pound a week
- We increase our physical activity alongside decreasing our calories to give the calorie balance an extra boost
- Aim for long-term success by finding things you can stick to, but trying new things that improve the health of our daily lifestyle
- Don’t fall into the trap of eating more because you exercise more– stick to your calorie plan!

Building on the last month

- **OUR ACHIEVABLE GOALS**
  - Started with 6,000 steps a day with 2,000+ "brisk"
  - Aiming for 150 min a week by Week 8
    - That's about 22 min a day!
  - Sticking to our calorie goals, and being consistent
  - Logging calories and exercise everyday so you can track what is working and what is not working
    - Review your weekly weight change with the intervention team by email to make sure you keep advancing toward your goals
  - Acknowledge the hard work and success you've already had
    - Maintaining those while we explore more
  - Achieving 5-7% body weight loss and keeping it off the rest of the year

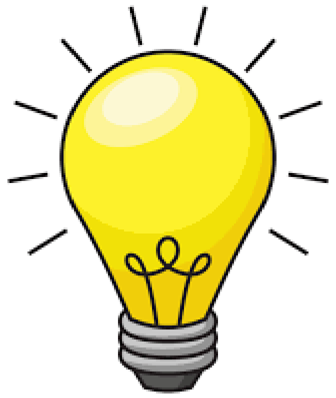

THIS WEEK'S TIP:
Drink water before you eat!
Sometimes we mistake thirst for hunger and reach for a snack instead of a glass of water or a calorie-free beverage. Check in with your thirst before grabbing something to eat.

How am I going to tip the calorie balance?

<u>What will this week include? (select all)</u>

- ○ Drink more water
- ○ Start every meal with a salad
- ○ Take the long way back on a walk
- ○ Jog in place during commercial breaks
- ○ Make a walking date with a friend
- ○ Take an extra loop or two in the grocery store
- ○ Cut out soda and alcohol
- ○ Try a new recipe
- ○ Log my calories and staying on my calorie target
- ○ Getting in my steps
- ○ Weigh myself everyday
- ○ Other (discuss)

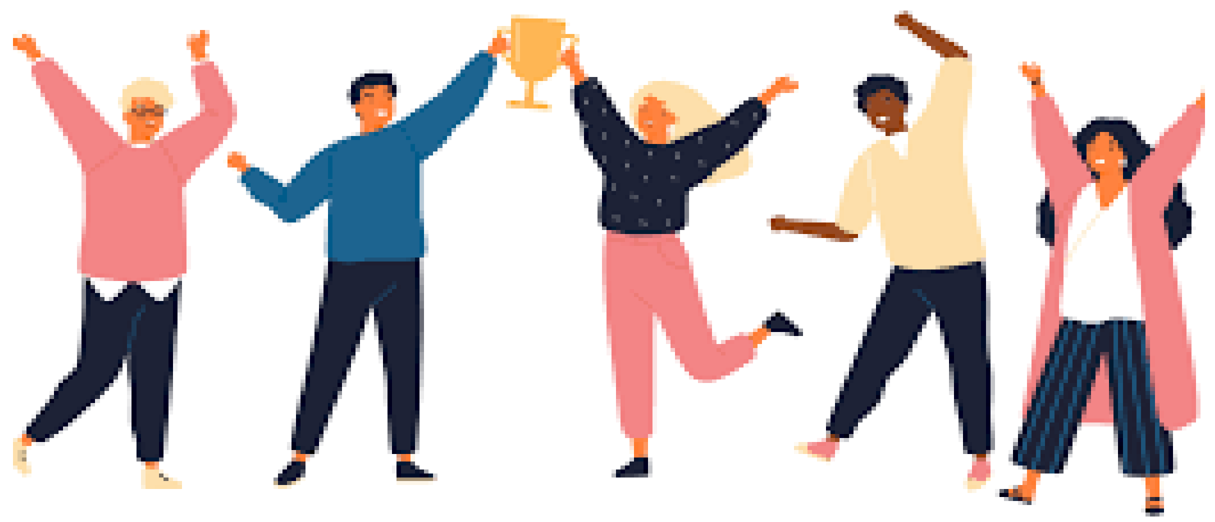

Put it into Practice

BB-HEALTH

Tip the Energy Balance

1. Track those meals and take your weight EVERYDAY!
   - Monitor your progress and identify areas for improvement towards YOUR GOALS

2. Ask yourself- can I do another 10 min of exercise today?
   - Aim to do 30 min more this week than last week (a minimum of 60 min)
   - Use your activity planner and record your 10min+ successes!

3. Stand more than sit, walk more than stand.
   - Take the stairs, go on a short walk to kill time– more ideas to get more active? Put them in the chat!

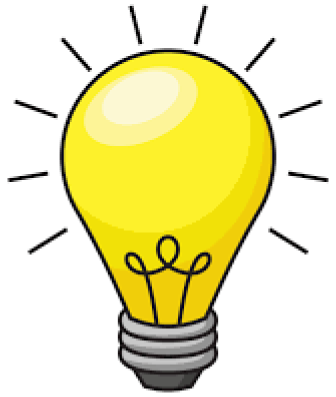

REMEMBER THIS WEEK'S TIP: ***Drink more water!***
Fill up on fluids with no calories! Make sure to get enough water in which will make exercise easier and hunger less frequent

Questions?

B B - H E A L T H

For next week:

1. Drink water when you feel hungry between meals

2. Track your food and exercise and **<u>Weigh Every Day</u>**

3. Remember your calorie, fat, and step count targets

4. Email your logs and progress to the Intervention Team and Review the response!

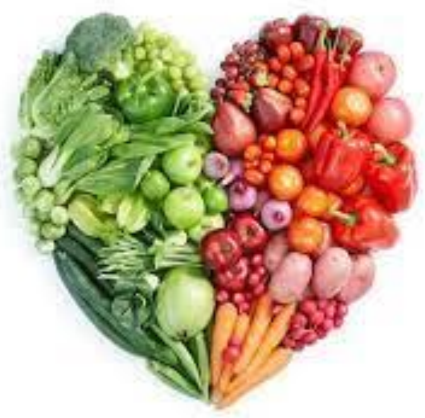

FOOD FOR THOUGHT

- The calorie balance can work for you, keep trying
- Check that scale everyday but focus on weekly trends, you got this!
