# Supplementary material for: Development and Feasibility of an eHealth Diabetes Prevention Program Adapted for Older Adults—Results from a Randomized Control Pilot Study
Source: Nutrients. 2024 Mar 23;16(7):930. doi: 10.3390/nu16070930 (PMC11154527; doi:10.3390/nu16070930)
Supplement: Supplementary file 1 [file nutrients-16-00930-s001.zip › Week6.pptx]

## Slide 1
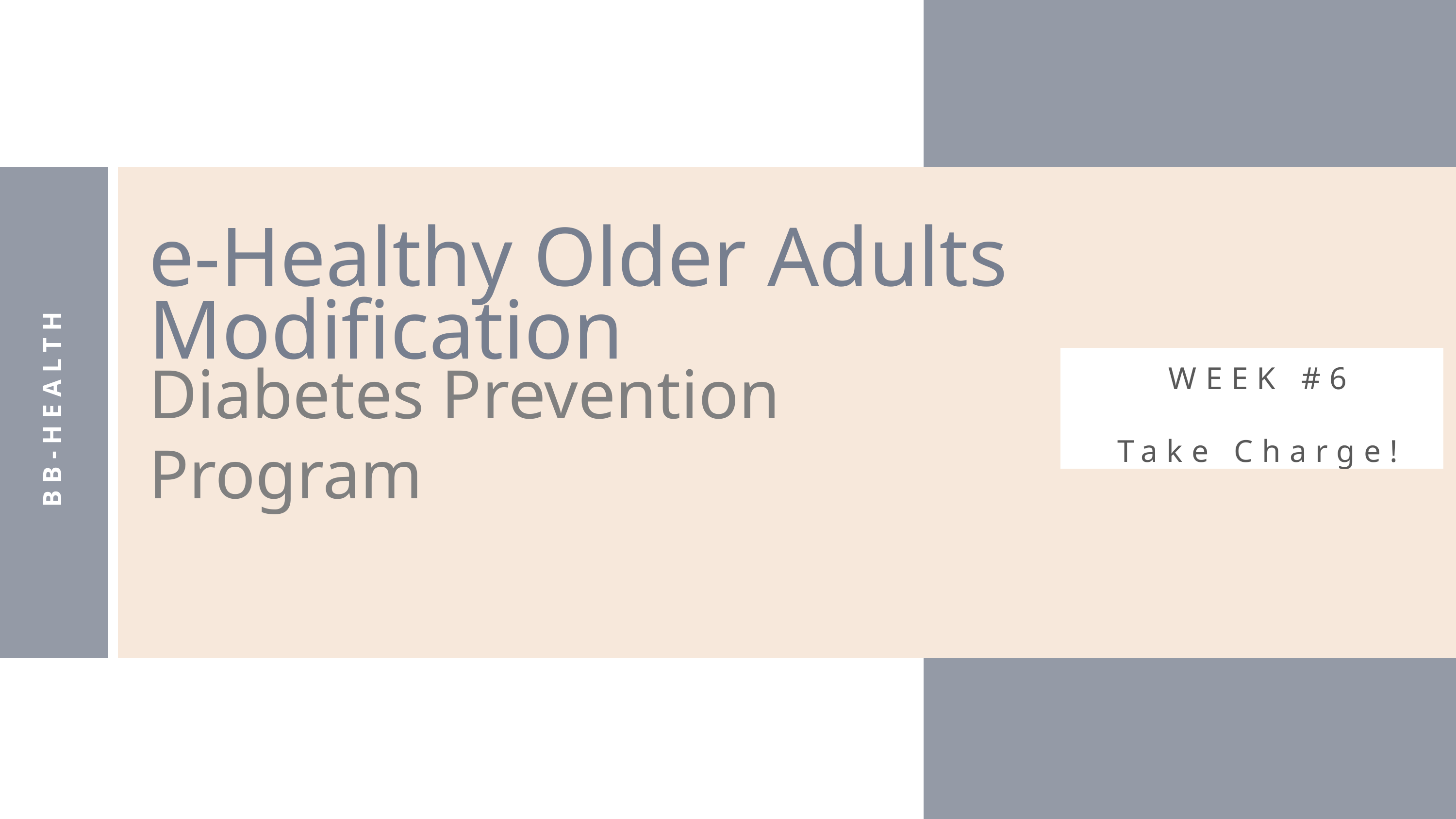

OPEN REPORTS
e-Healthy Older Adults Modification
WEEK #6
Take Charge!
Diabetes Prevention Program
BB-HEALTH

## Slide 2
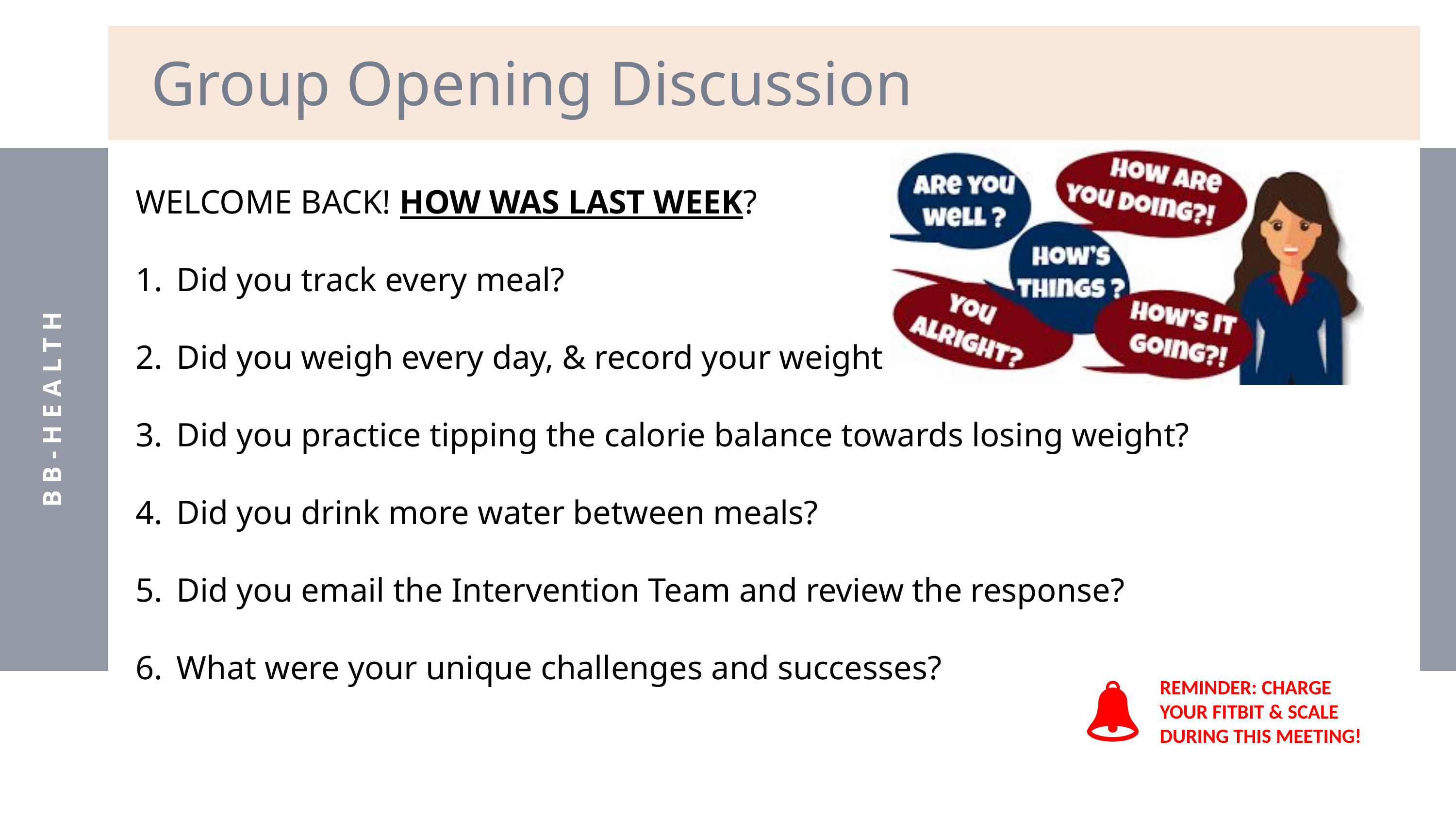

Group Opening Discussion
WELCOME BACK! HOW WAS LAST WEEK?
Did you track every meal?
Did you weigh every day, & record your weight today?
Did you practice tipping the calorie balance towards losing weight?
Did you drink more water between meals?
Did you email the Intervention Team and review the response?
What were your unique challenges and successes?
BB-HEALTH
REMINDER: CHARGE YOUR FITBIT & SCALE DURING THIS MEETING!

## Slide 3
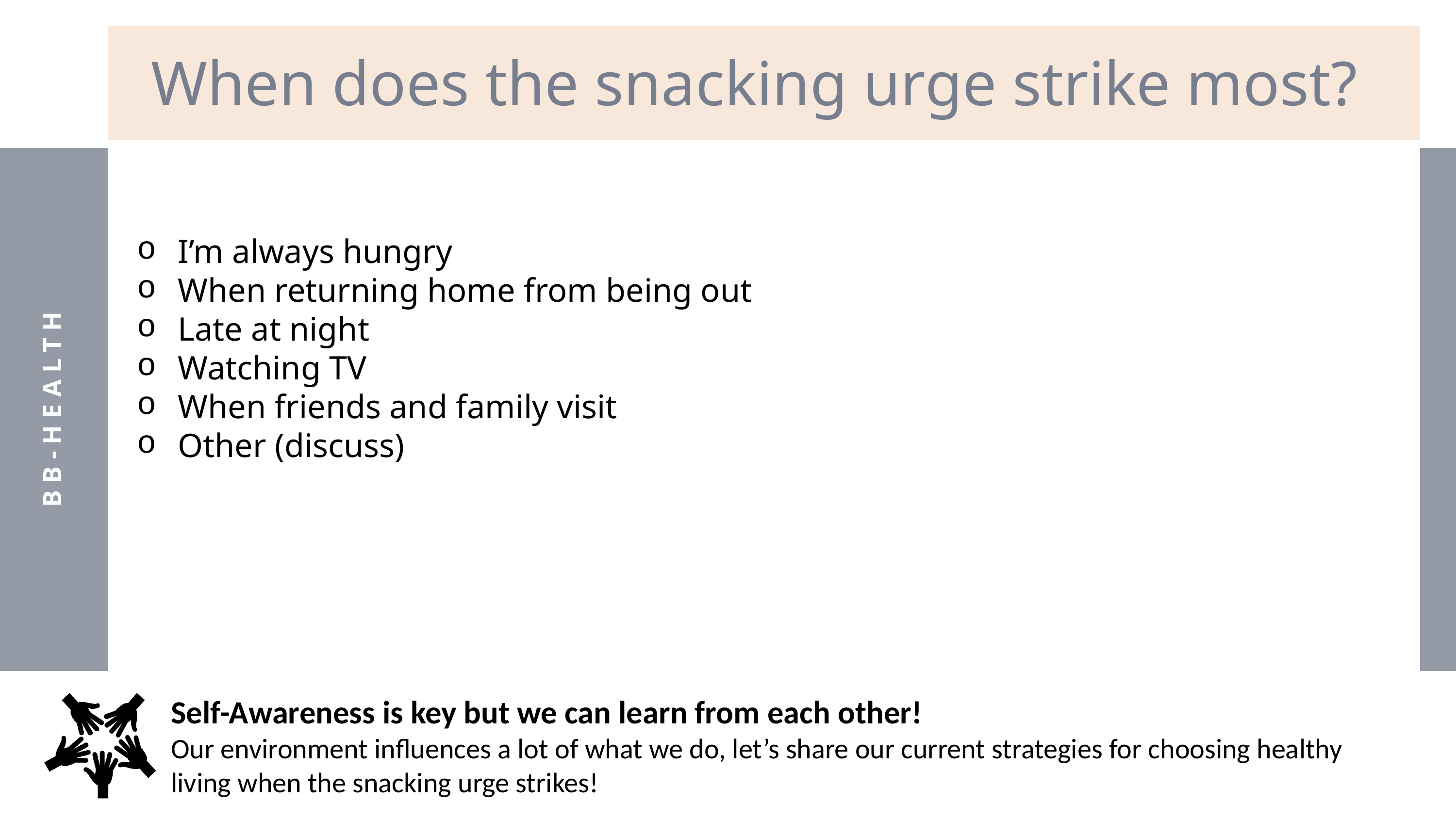

When does the snacking urge strike most?
I’m always hungry
When returning home from being out
Late at night
Watching TV
When friends and family visit
Other (discuss)
BB-HEALTH
Self-Awareness is key but we can learn from each other!
Our environment influences a lot of what we do, let’s share our current strategies for choosing healthy living when the snacking urge strikes!

## Slide 4
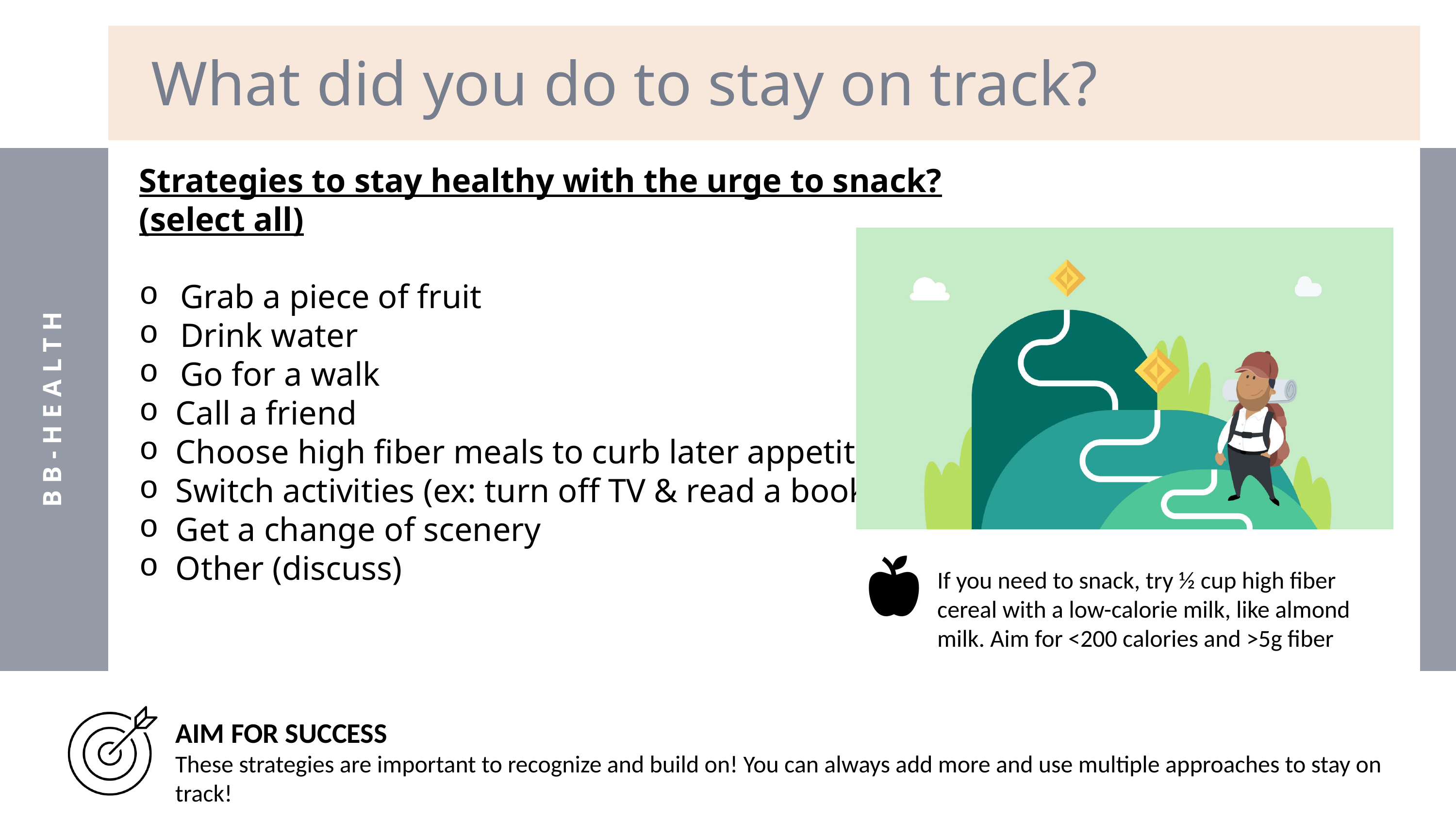

What did you do to stay on track?
Strategies to stay healthy with the urge to snack? (select all)
Grab a piece of fruit
Drink water
Go for a walk
Call a friend
Choose high fiber meals to curb later appetite
Switch activities (ex: turn off TV & read a book)
Get a change of scenery
Other (discuss)
BB-HEALTH
If you need to snack, try ½ cup high fiber cereal with a low-calorie milk, like almond milk. Aim for <200 calories and >5g fiber
AIM FOR SUCCESS
These strategies are important to recognize and build on! You can always add more and use multiple approaches to stay on track!

## Slide 5
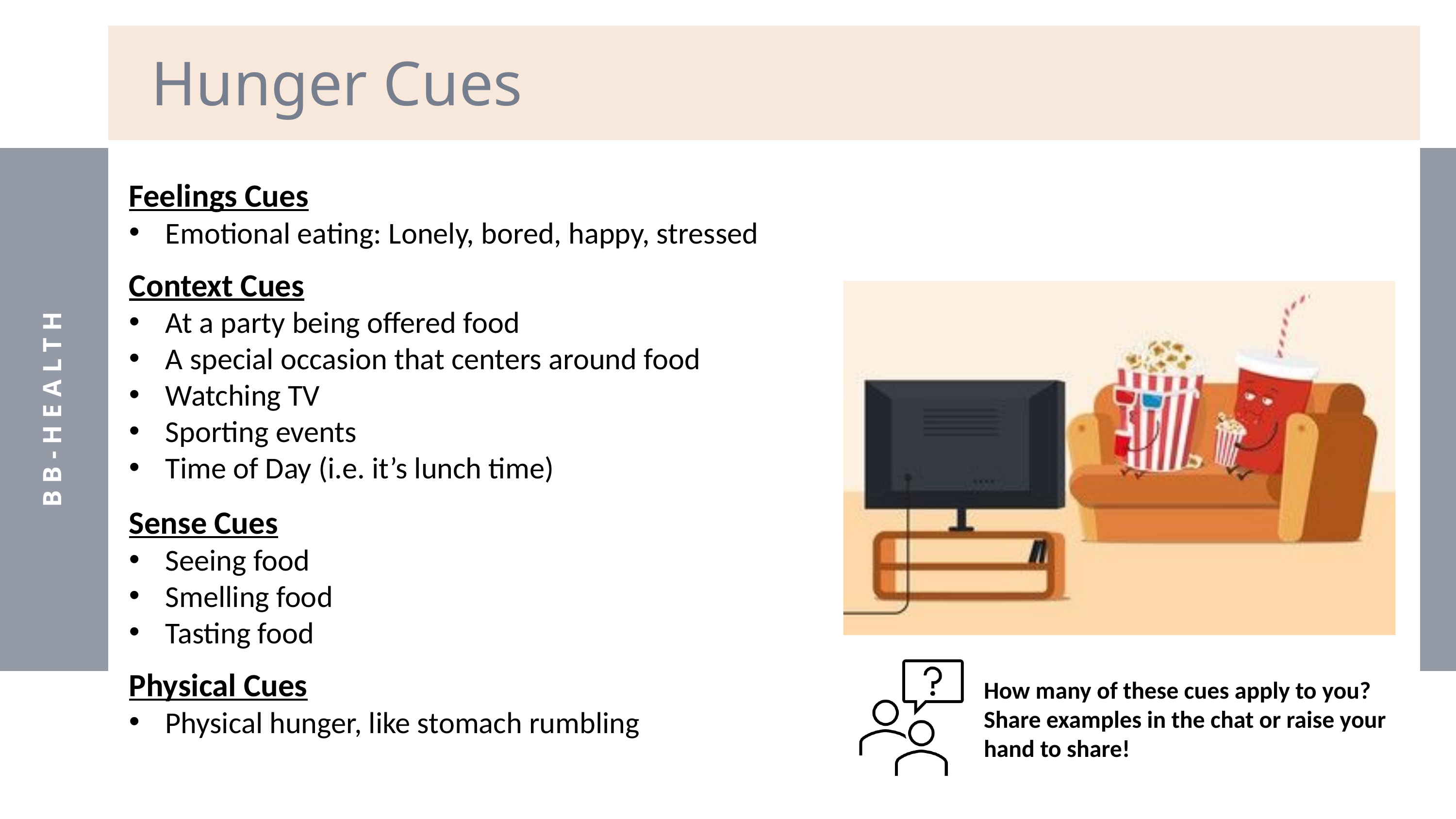

Hunger Cues
Feelings Cues
Emotional eating: Lonely, bored, happy, stressed
Context Cues
At a party being offered food
A special occasion that centers around food
Watching TV
Sporting events
Time of Day (i.e. it’s lunch time)
Sense Cues
Seeing food
Smelling food
Tasting food
Physical Cues
Physical hunger, like stomach rumbling
BB-HEALTH
How many of these cues apply to you?
Share examples in the chat or raise your hand to share!

## Slide 6
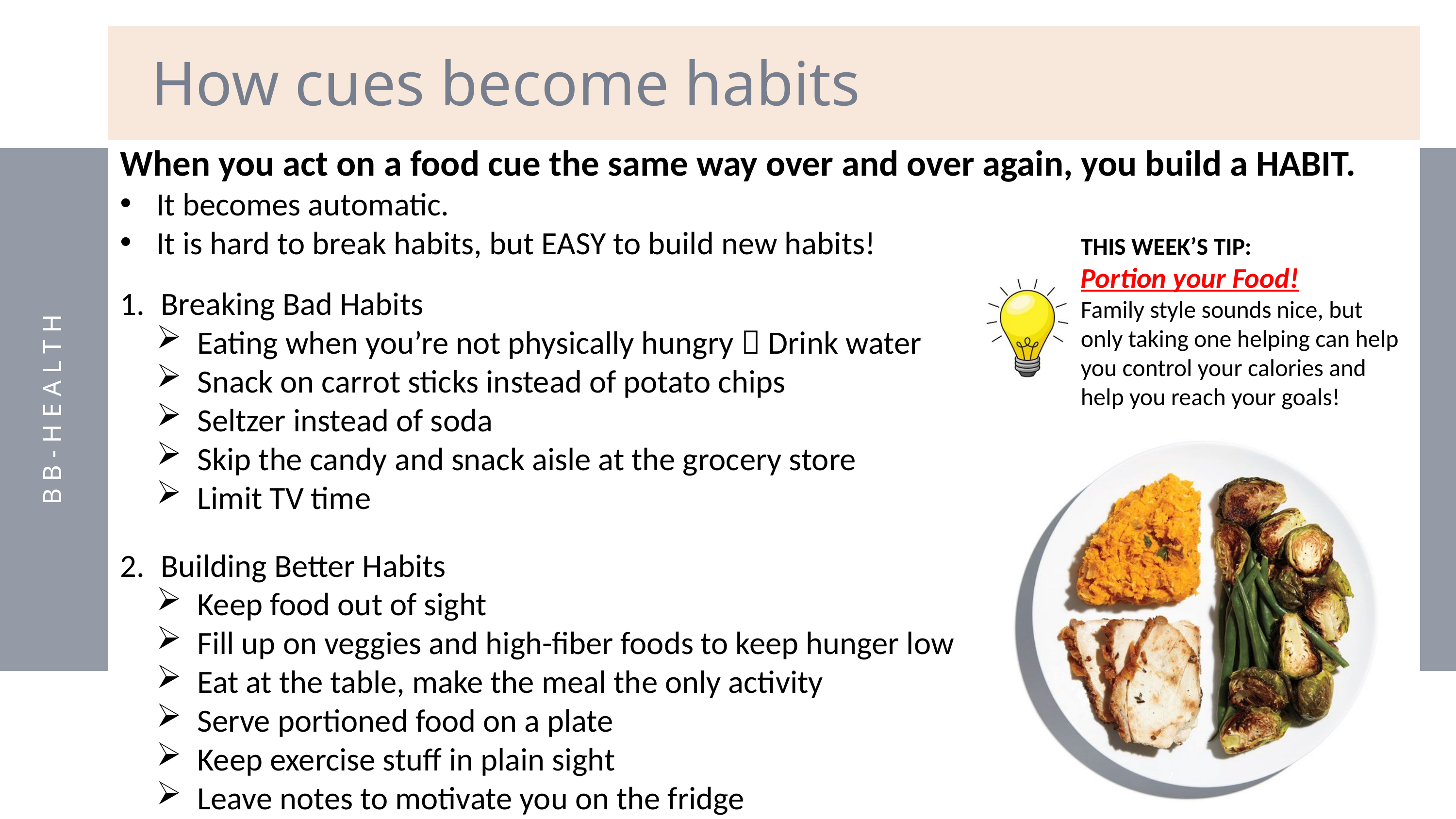

How cues become habits
When you act on a food cue the same way over and over again, you build a HABIT.
It becomes automatic.
It is hard to break habits, but EASY to build new habits!
Breaking Bad Habits
Eating when you’re not physically hungry  Drink water
Snack on carrot sticks instead of potato chips
Seltzer instead of soda
Skip the candy and snack aisle at the grocery store
Limit TV time
Building Better Habits
Keep food out of sight
Fill up on veggies and high-fiber foods to keep hunger low
Eat at the table, make the meal the only activity
Serve portioned food on a plate
Keep exercise stuff in plain sight
Leave notes to motivate you on the fridge
THIS WEEK’S TIP:
Portion your Food!
Family style sounds nice, but only taking one helping can help you control your calories and help you reach your goals!
BB-HEALTH

## Slide 7
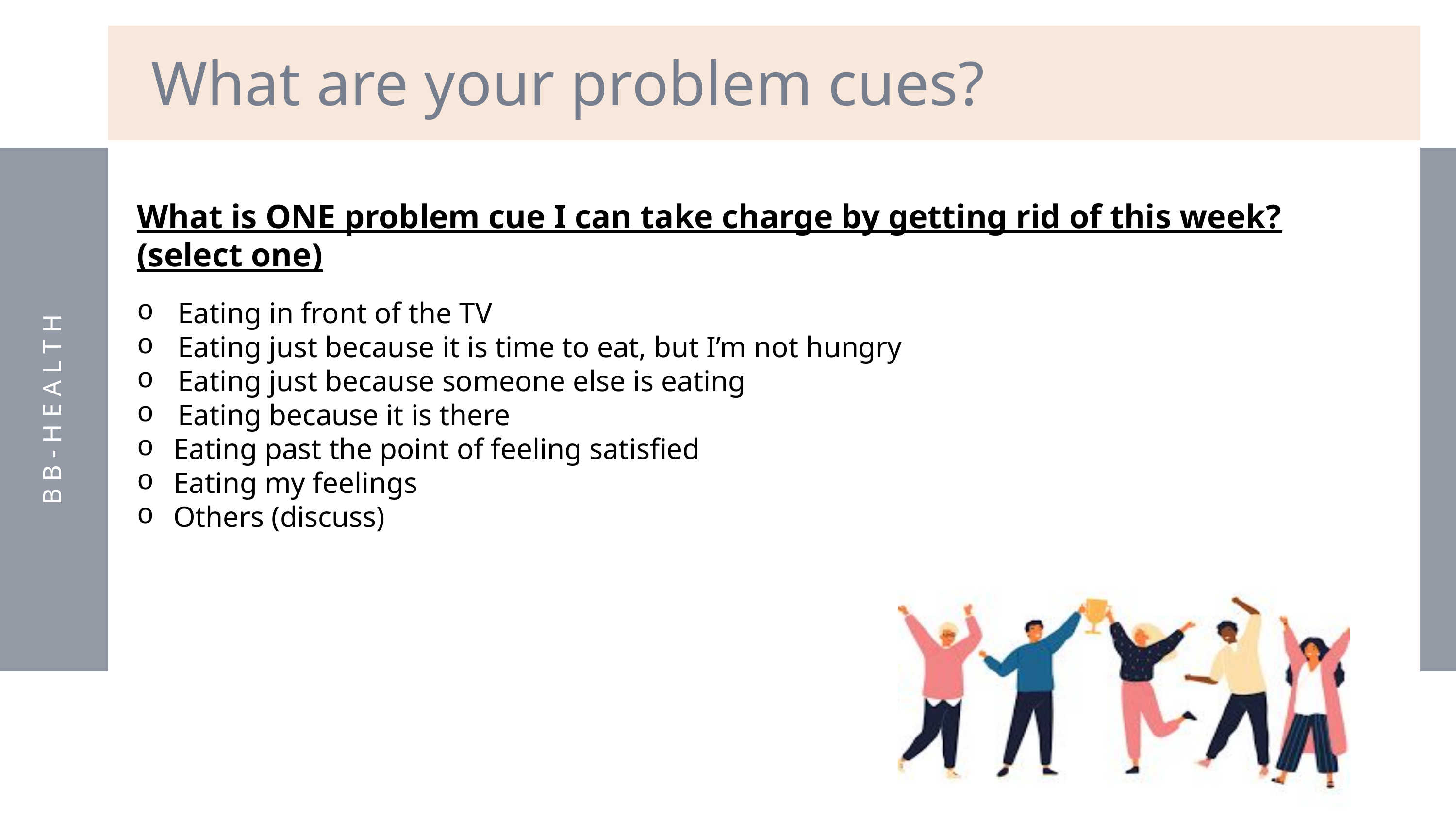

What are your problem cues?
What is ONE problem cue I can take charge by getting rid of this week? (select one)
Eating in front of the TV
Eating just because it is time to eat, but I’m not hungry
Eating just because someone else is eating
Eating because it is there
Eating past the point of feeling satisfied
Eating my feelings
Others (discuss)
BB-HEALTH

## Slide 8
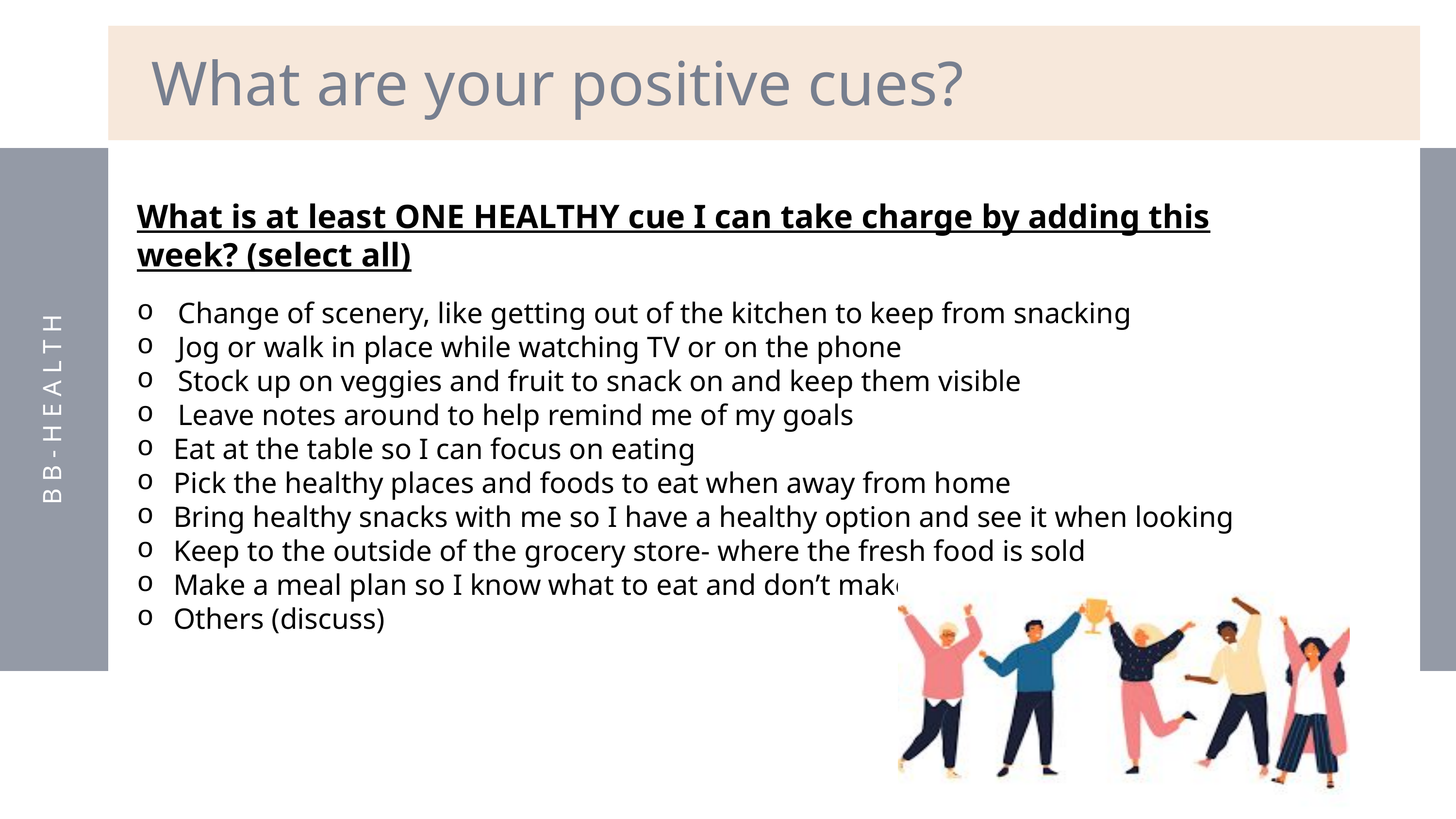

What are your positive cues?
What is at least ONE HEALTHY cue I can take charge by adding this week? (select all)
Change of scenery, like getting out of the kitchen to keep from snacking
Jog or walk in place while watching TV or on the phone
Stock up on veggies and fruit to snack on and keep them visible
Leave notes around to help remind me of my goals
Eat at the table so I can focus on eating
Pick the healthy places and foods to eat when away from home
Bring healthy snacks with me so I have a healthy option and see it when looking
Keep to the outside of the grocery store- where the fresh food is sold
Make a meal plan so I know what to eat and don’t make last minute decisions
Others (discuss)
BB-HEALTH

## Slide 9
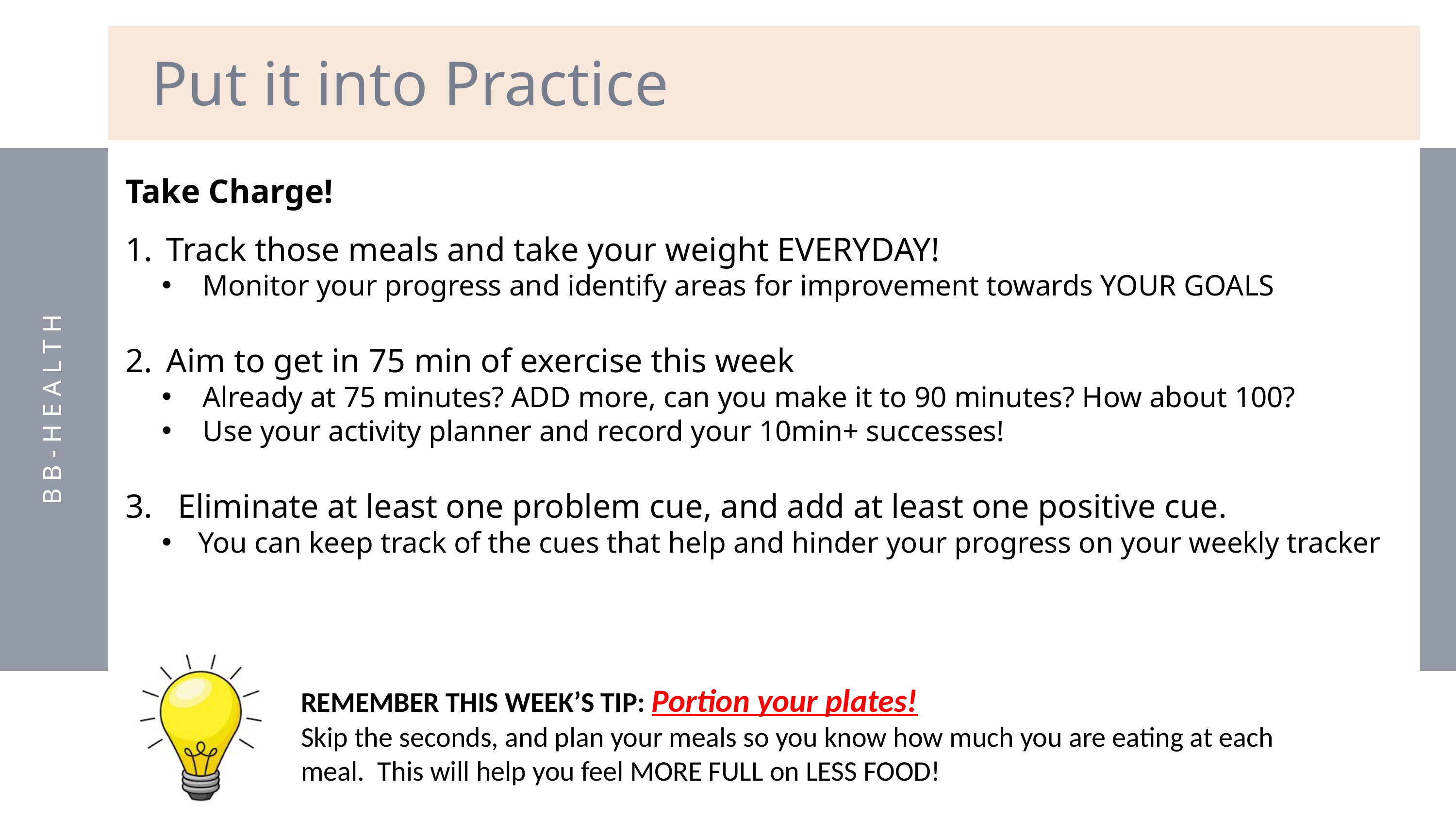

Put it into Practice
Take Charge!
Track those meals and take your weight EVERYDAY!
Monitor your progress and identify areas for improvement towards YOUR GOALS
Aim to get in 75 min of exercise this week
Already at 75 minutes? ADD more, can you make it to 90 minutes? How about 100?
Use your activity planner and record your 10min+ successes!
3. Eliminate at least one problem cue, and add at least one positive cue.
You can keep track of the cues that help and hinder your progress on your weekly tracker
BB-HEALTH
REMEMBER THIS WEEK’S TIP: Portion your plates!
Skip the seconds, and plan your meals so you know how much you are eating at each meal. This will help you feel MORE FULL on LESS FOOD!

## Slide 10
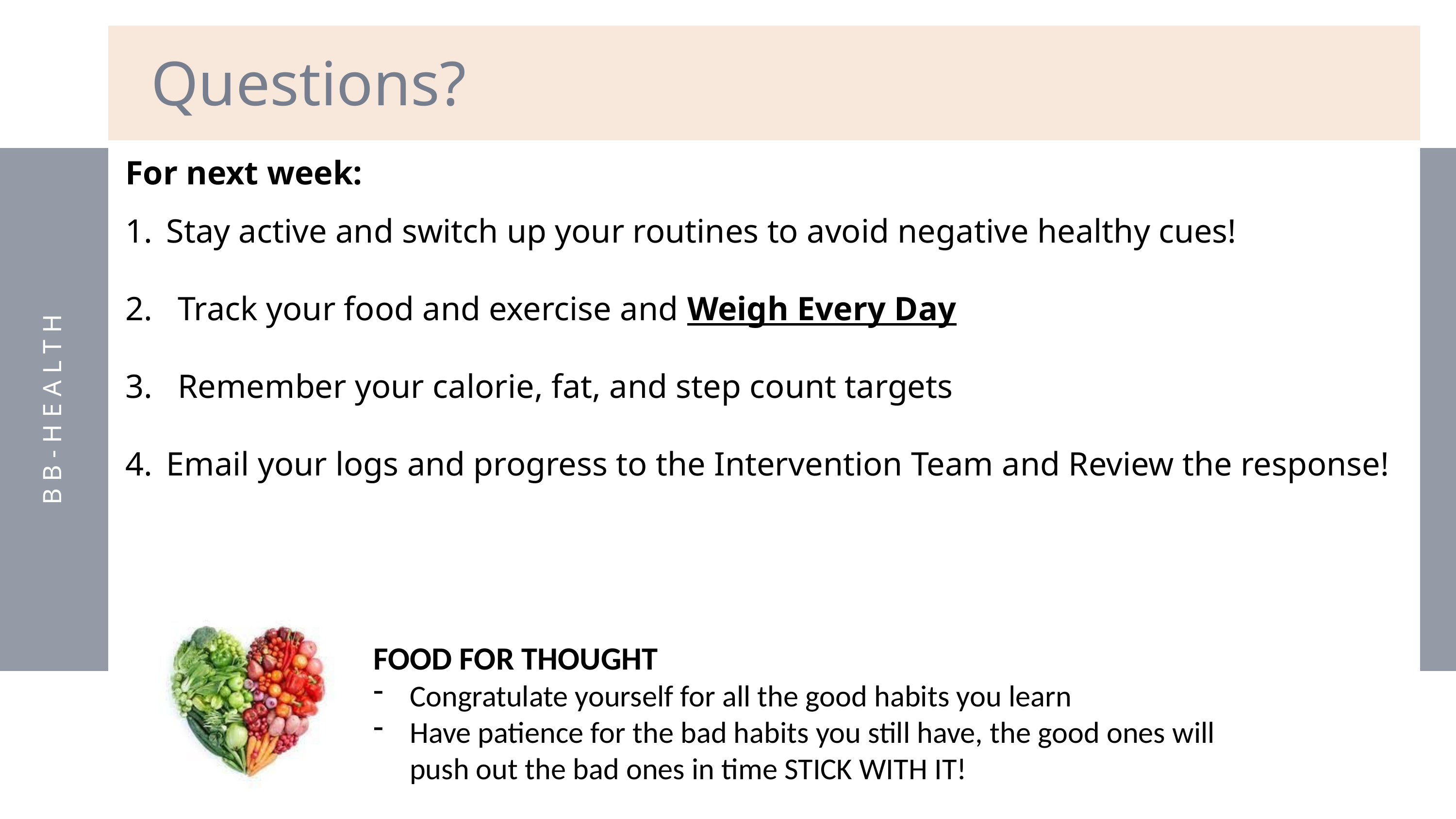

Questions?
For next week:
Stay active and switch up your routines to avoid negative healthy cues!
2. Track your food and exercise and Weigh Every Day
3. Remember your calorie, fat, and step count targets
Email your logs and progress to the Intervention Team and Review the response!
BB-HEALTH
FOOD FOR THOUGHT
Congratulate yourself for all the good habits you learn
Have patience for the bad habits you still have, the good ones will push out the bad ones in time STICK WITH IT!
