# Supplementary material for: Development and Feasibility of an eHealth Diabetes Prevention Program Adapted for Older Adults—Results from a Randomized Control Pilot Study
Source: Nutrients. 2024 Mar 23;16(7):930. doi: 10.3390/nu16070930 (PMC11154527; doi:10.3390/nu16070930)
Supplement: Supplementary file 1 [file nutrients-16-00930-s001.zip › Week6_SUPP.pdf]

BB-HEALTH

e-Healthy Older Adults Modification

Diabetes Prevention Program

WEEK #6

Take Charge!

Group Opening Discussion

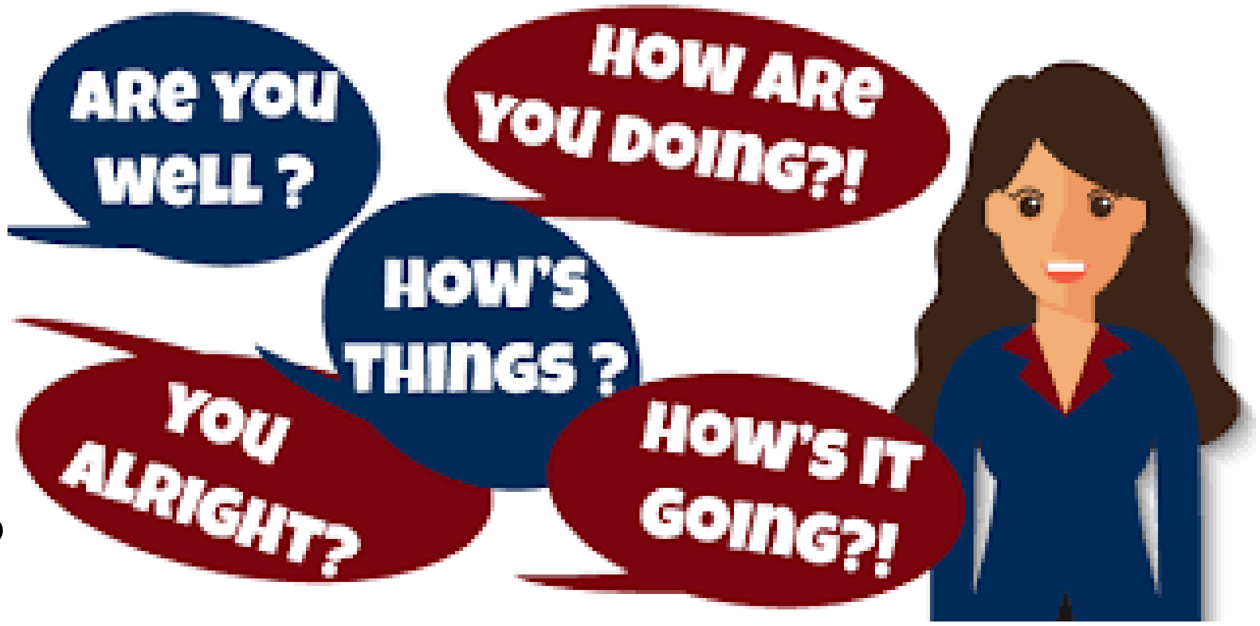

WELCOME BACK! **HOW WAS LAST WEEK**?

1. Did you track every meal?
2. Did you weigh every day, & record your weight today?
3. Did you practice tipping the calorie balance towards losing weight?
4. Did you drink more water between meals?
5. Did you email the Intervention Team and review the response?
6. What were your unique challenges and successes?

REMINDER: CHARGE YOUR FITBIT & SCALE DURING THIS MEETING!

BB-HEALTH

When does the snacking urge strike most?

BB-HEALTH

- I’m always hungry
- When returning home from being out
- Late at night
- Watching TV
- When friends and family visit
- Other (discuss)

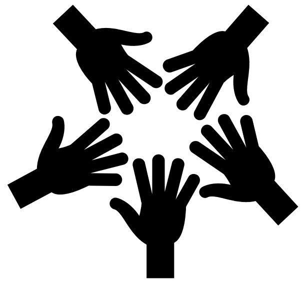

Self-Awareness is key but we can learn from each other!

Our environment influences a lot of what we do, let’s share our current strategies for choosing healthy living when the snacking urge strikes!

B B - H E A L T H

What did you do to stay on track?

<u>Strategies to stay healthy with the urge to snack? (select all)</u>

- Grab a piece of fruit
- Drink water
- Go for a walk
- Call a friend
- Choose high fiber meals to curb later appetite
- Switch activities (ex: turn off TV & read a book)
- Get a change of scenery
- Other (discuss)

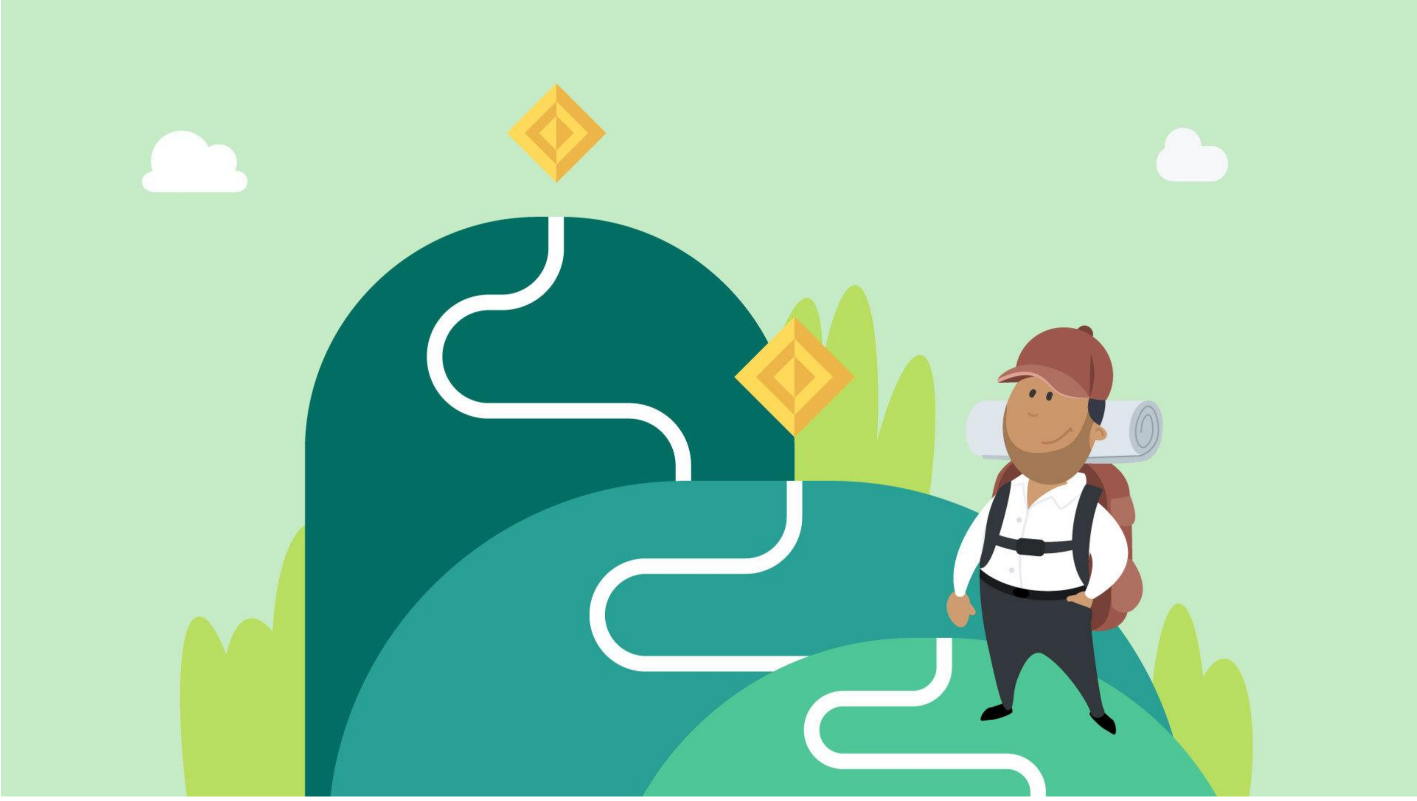

If you need to snack, try ½ cup high fiber cereal with a low-calorie milk, like almond milk. Aim for <200 calories and >5g fiber

AIM FOR SUCCESS

These strategies are important to recognize and build on! You can always add more and use multiple approaches to stay on track!

Hunger Cues

Feelings Cues

- Emotional eating: Lonely, bored, happy, stressed

Context Cues

- At a party being offered food
- A special occasion that centers around food
- Watching TV
- Sporting events
- Time of Day (i.e. it's lunch time)

Sense Cues

- Seeing food
- Smelling food
- Tasting food

Physical Cues

- Physical hunger, like stomach rumbling

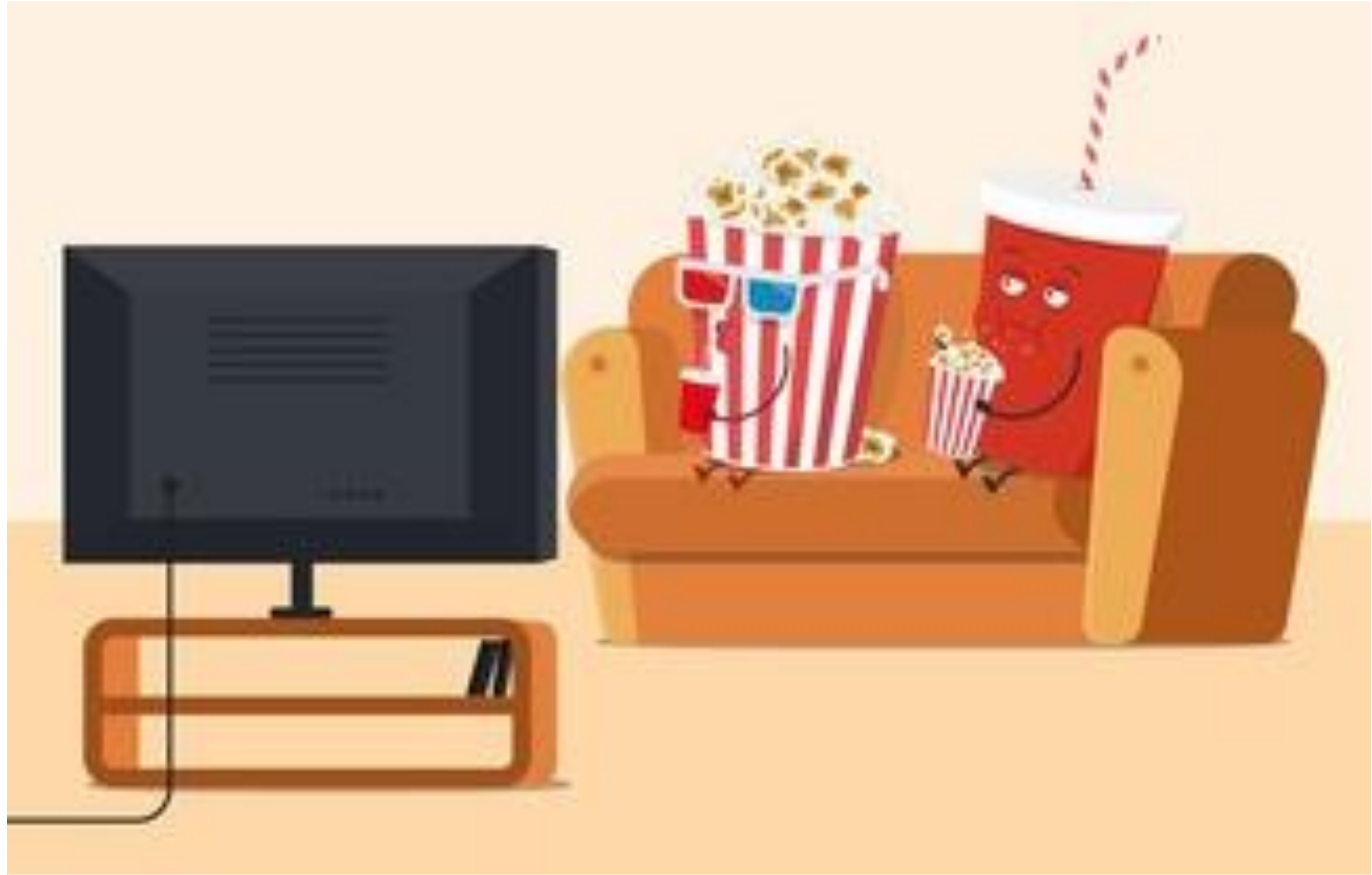

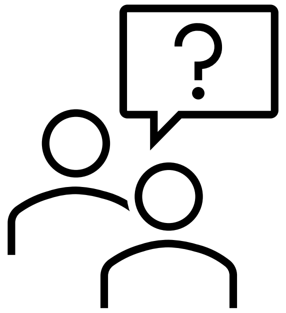

How many of these cues apply to you? Share examples in the chat or raise your hand to share!

How cues become habits

When you act on a food cue the same way over and over again, you build a HABIT.

- It becomes automatic.
- It is hard to break habits, but EASY to build new habits!

1. Breaking Bad Habits
   - ➢ Eating when you're not physically hungry → Drink water
   - ➢ Snack on carrot sticks instead of potato chips
   - ➢ Seltzer instead of soda
   - ➢ Skip the candy and snack aisle at the grocery store
   - ➢ Limit TV time

2. Building Better Habits
   - ➢ Keep food out of sight
   - ➢ Fill up on veggies and high-fiber foods to keep hunger low
   - ➢ Eat at the table, make the meal the only activity
   - ➢ Serve portioned food on a plate
   - ➢ Keep exercise stuff in plain sight
   - ➢ Leave notes to motivate you on the fridge

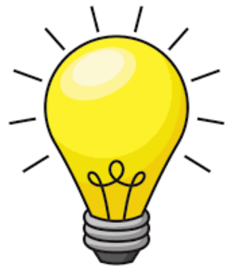

THIS WEEK'S TIP:

Portion your Food!

Family style sounds nice, but only taking one helping can help you control your calories and help you reach your goals!

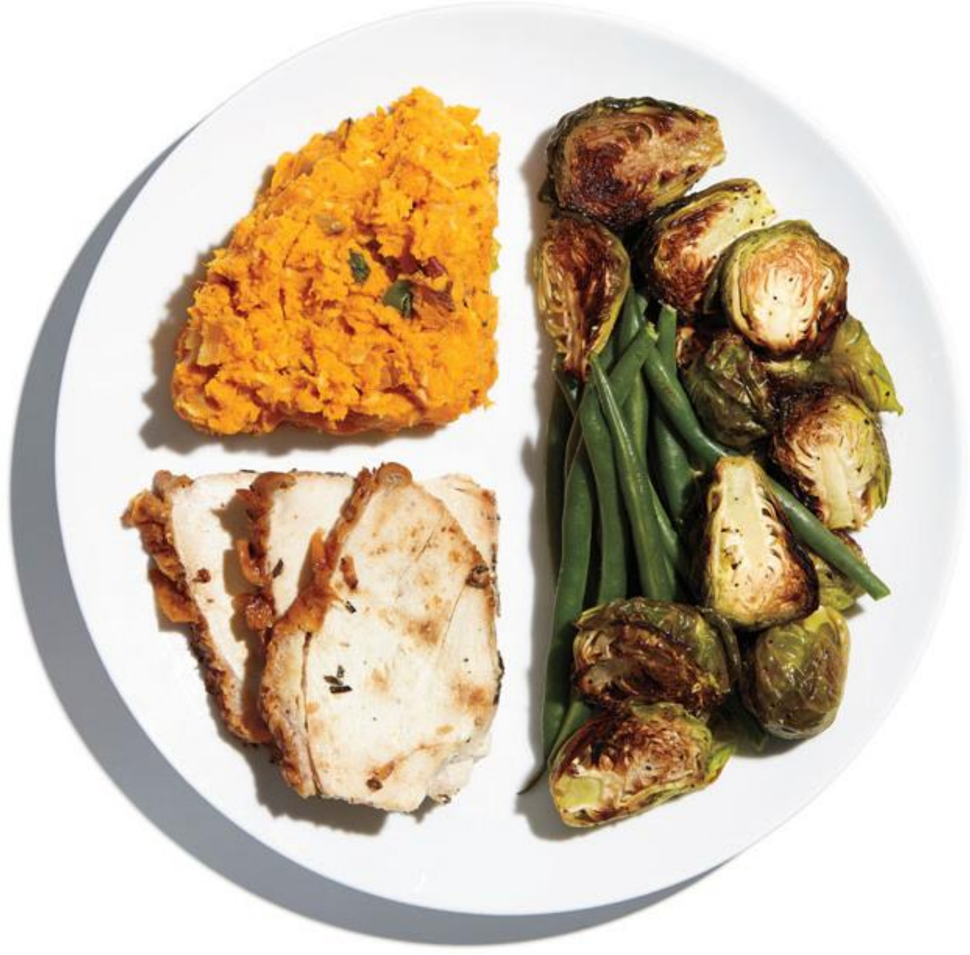

BB-HEALTH

What are your problem cues?

B B - H E A L T H

What is ONE problem cue I can take charge by getting rid of this week? (select one)

- ○ Eating in front of the TV
- ○ Eating just because it is time to eat, but I'm not hungry
- ○ Eating just because someone else is eating
- ○ Eating because it is there
- ○ Eating past the point of feeling satisfied
- ○ Eating my feelings
- ○ Others (discuss)

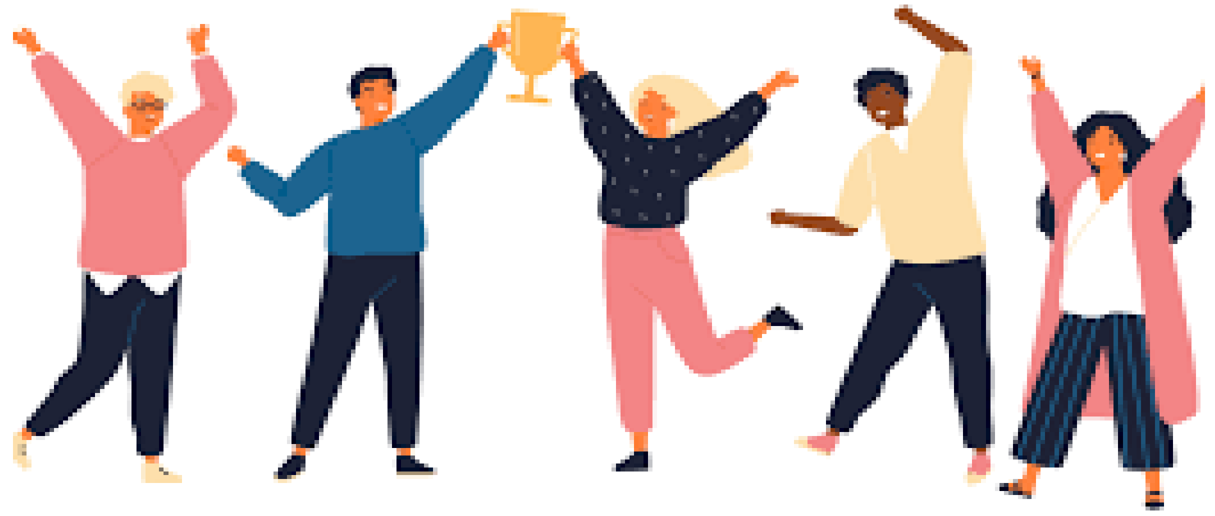

What are your positive cues?

B B - H E A L T H

What is at least ONE HEALTHY cue I can take charge by adding this week? (select all)

- Change of scenery, like getting out of the kitchen to keep from snacking
- Jog or walk in place while watching TV or on the phone
- Stock up on veggies and fruit to snack on and keep them visible
- Leave notes around to help remind me of my goals
- Eat at the table so I can focus on eating
- Pick the healthy places and foods to eat when away from home
- Bring healthy snacks with me so I have a healthy option and see it when looking
- Keep to the outside of the grocery store- where the fresh food is sold
- Make a meal plan so I know what to eat and don’t make last minute decisions
- Others (discuss)

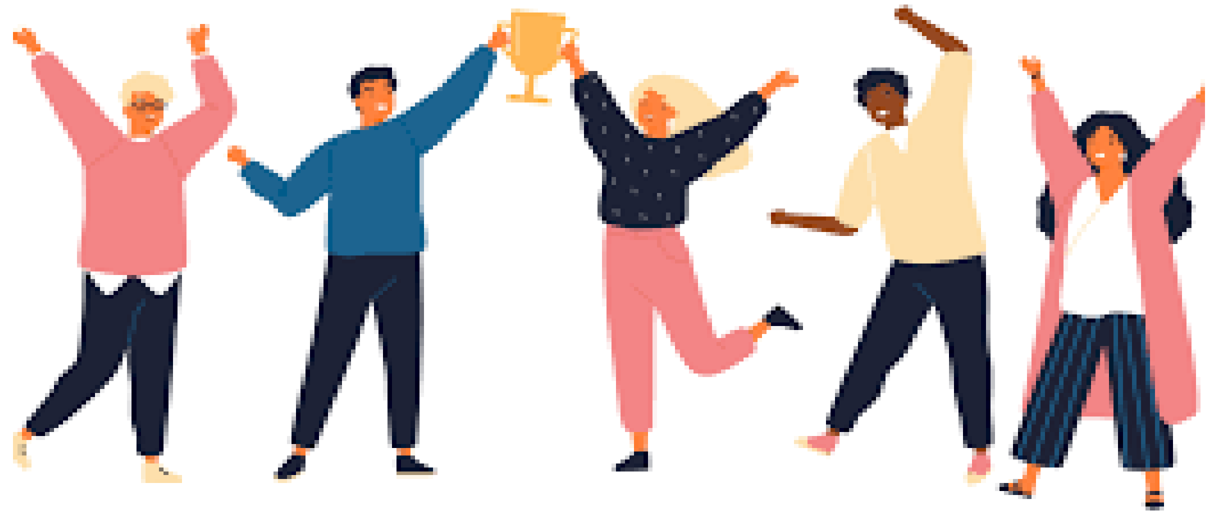

Put it into Practice

B B - H E A L T H

Take Charge!

1. Track those meals and take your weight EVERYDAY!
   - Monitor your progress and identify areas for improvement towards YOUR GOALS

2. Aim to get in 75 min of exercise this week
   - Already at 75 minutes? ADD more, can you make it to 90 minutes? How about 100?
   - Use your activity planner and record your 10min+ successes!

3. Eliminate at least one problem cue, and add at least one positive cue.
   - You can keep track of the cues that help and hinder your progress on your weekly tracker

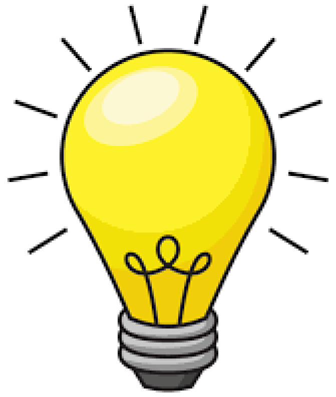

REMEMBER THIS WEEK'S TIP: ***Portion your plates!***
Skip the seconds, and plan your meals so you know how much you are eating at each meal. This will help you feel MORE FULL on LESS FOOD!

Questions?

For next week:

1. Stay active and switch up your routines to avoid negative healthy cues!

2. Track your food and exercise and **Weigh Every Day**

3. Remember your calorie, fat, and step count targets

4. Email your logs and progress to the Intervention Team and Review the response!

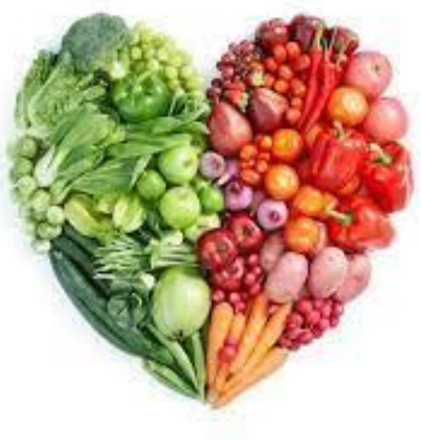

FOOD FOR THOUGHT

- Congratulate yourself for all the good habits you learn
- Have patience for the bad habits you still have, the good ones will push out the bad ones in time STICK WITH IT!
