# Supplementary material for: Development and Feasibility of an eHealth Diabetes Prevention Program Adapted for Older Adults—Results from a Randomized Control Pilot Study
Source: Nutrients. 2024 Mar 23;16(7):930. doi: 10.3390/nu16070930 (PMC11154527; doi:10.3390/nu16070930)
Supplement: Supplementary file 1 [file nutrients-16-00930-s001.zip › Week7.pptx]

## Slide 1
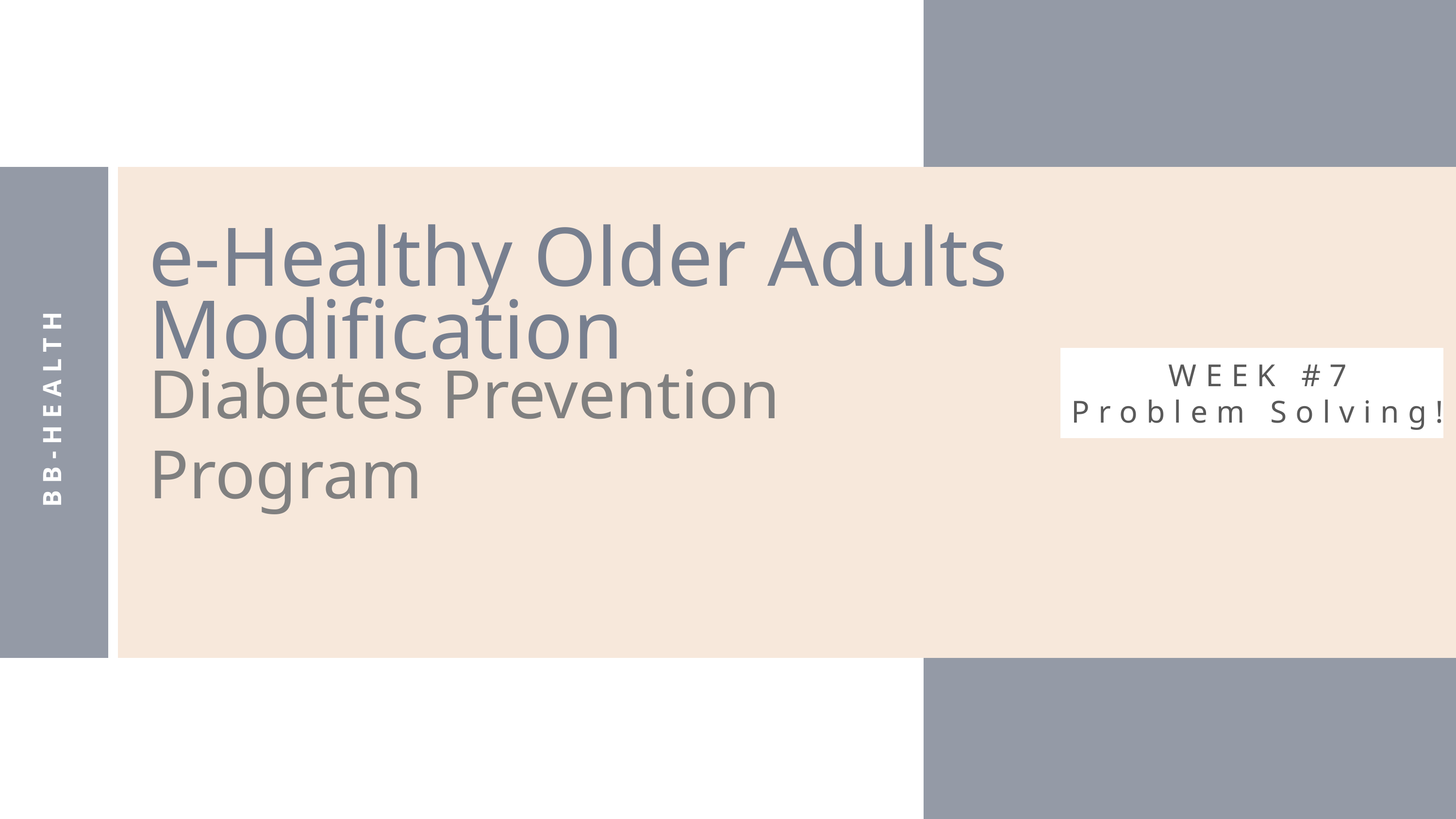

OPEN REPORTS
e-Healthy Older Adults Modification
WEEK #7
Problem Solving!
Diabetes Prevention Program
BB-HEALTH

## Slide 2
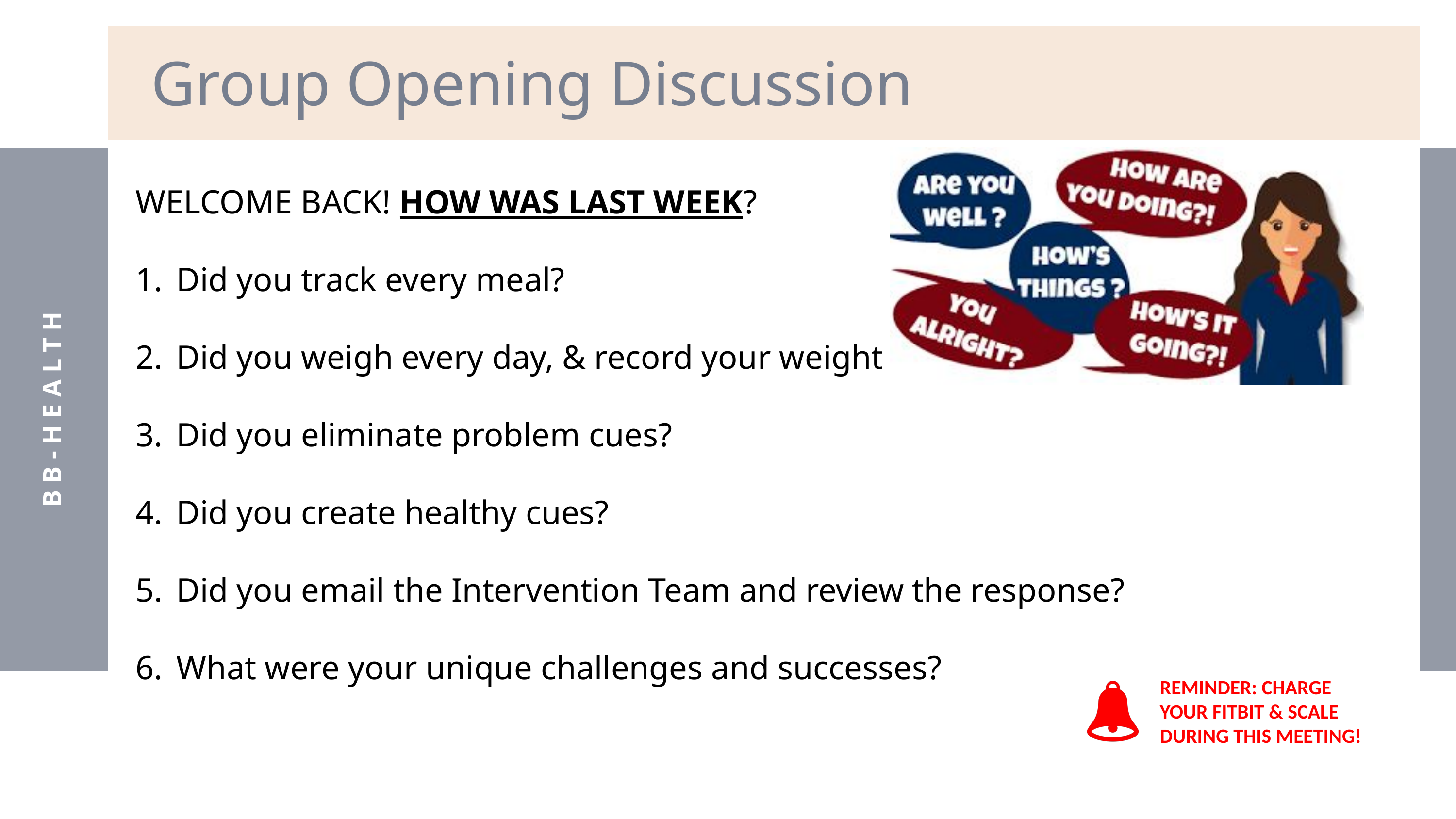

Group Opening Discussion
WELCOME BACK! HOW WAS LAST WEEK?
Did you track every meal?
Did you weigh every day, & record your weight today?
Did you eliminate problem cues?
Did you create healthy cues?
Did you email the Intervention Team and review the response?
What were your unique challenges and successes?
BB-HEALTH
REMINDER: CHARGE YOUR FITBIT & SCALE DURING THIS MEETING!

## Slide 3
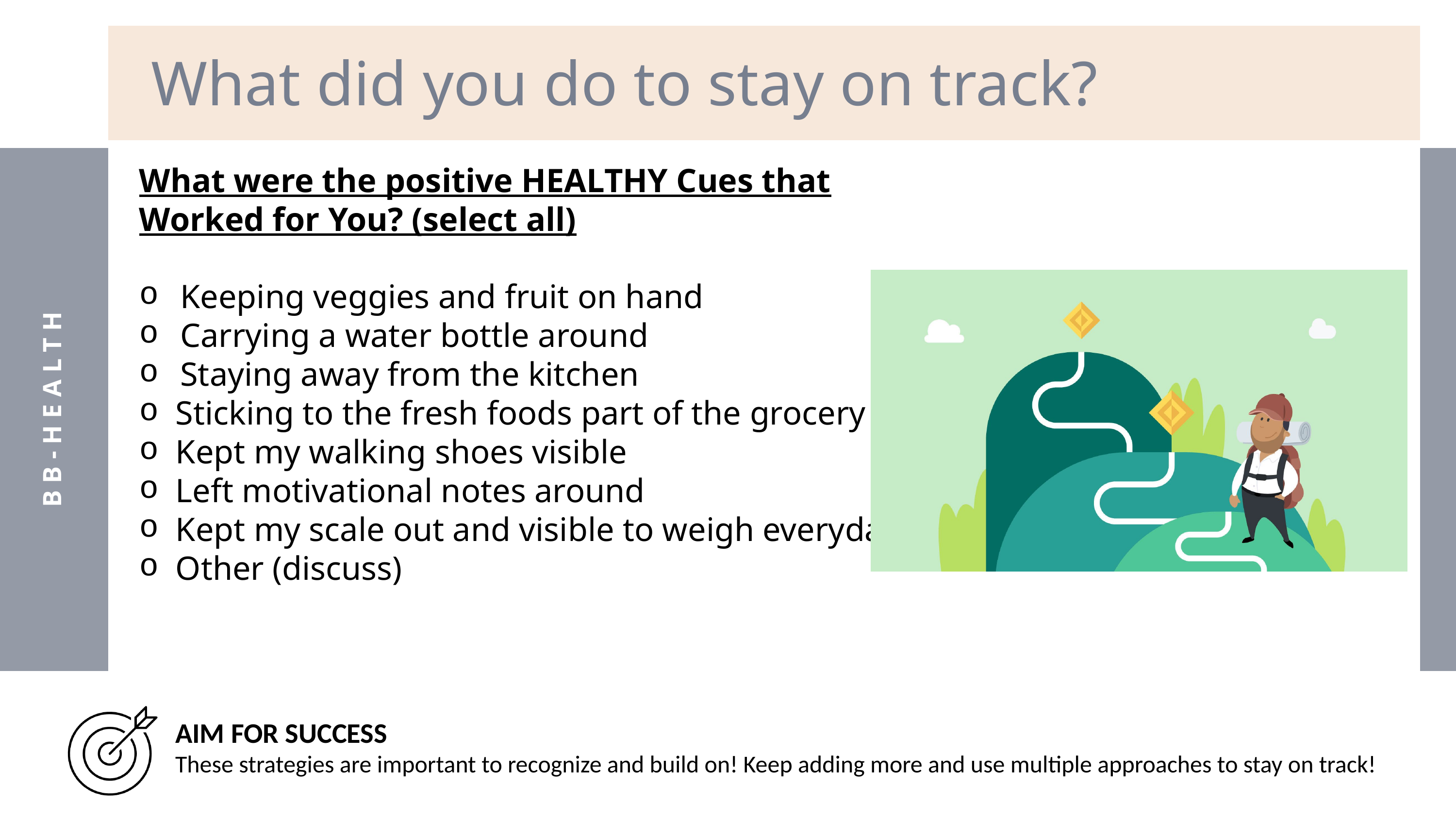

What did you do to stay on track?
What were the positive HEALTHY Cues that Worked for You? (select all)
Keeping veggies and fruit on hand
Carrying a water bottle around
Staying away from the kitchen
Sticking to the fresh foods part of the grocery store
Kept my walking shoes visible
Left motivational notes around
Kept my scale out and visible to weigh everyday
Other (discuss)
BB-HEALTH
AIM FOR SUCCESS
These strategies are important to recognize and build on! Keep adding more and use multiple approaches to stay on track!

## Slide 4
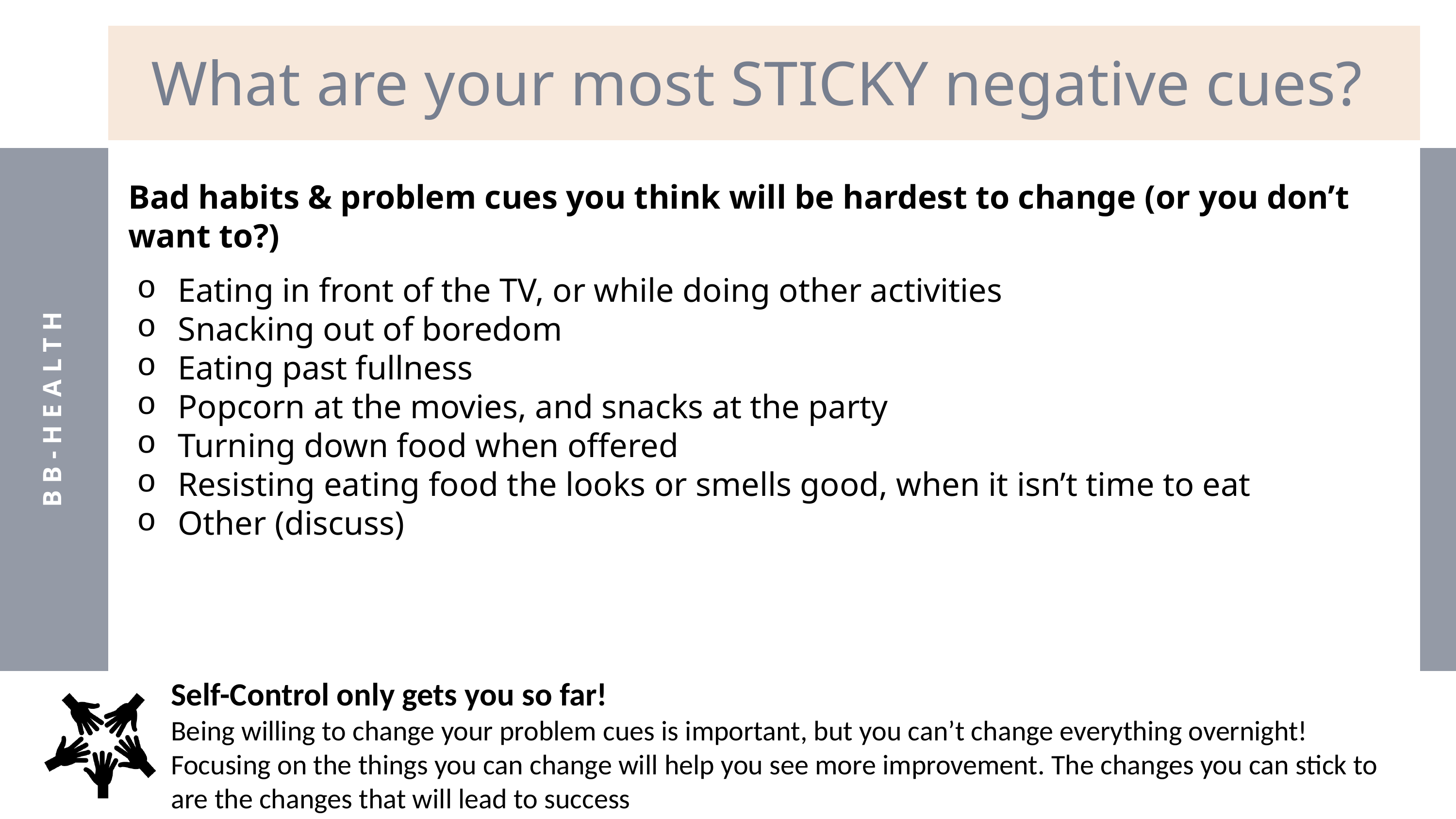

What are your most STICKY negative cues?
Bad habits & problem cues you think will be hardest to change (or you don’t want to?)
Eating in front of the TV, or while doing other activities
Snacking out of boredom
Eating past fullness
Popcorn at the movies, and snacks at the party
Turning down food when offered
Resisting eating food the looks or smells good, when it isn’t time to eat
Other (discuss)
BB-HEALTH
Self-Control only gets you so far!
Being willing to change your problem cues is important, but you can’t change everything overnight! Focusing on the things you can change will help you see more improvement. The changes you can stick to are the changes that will lead to success

## Slide 5
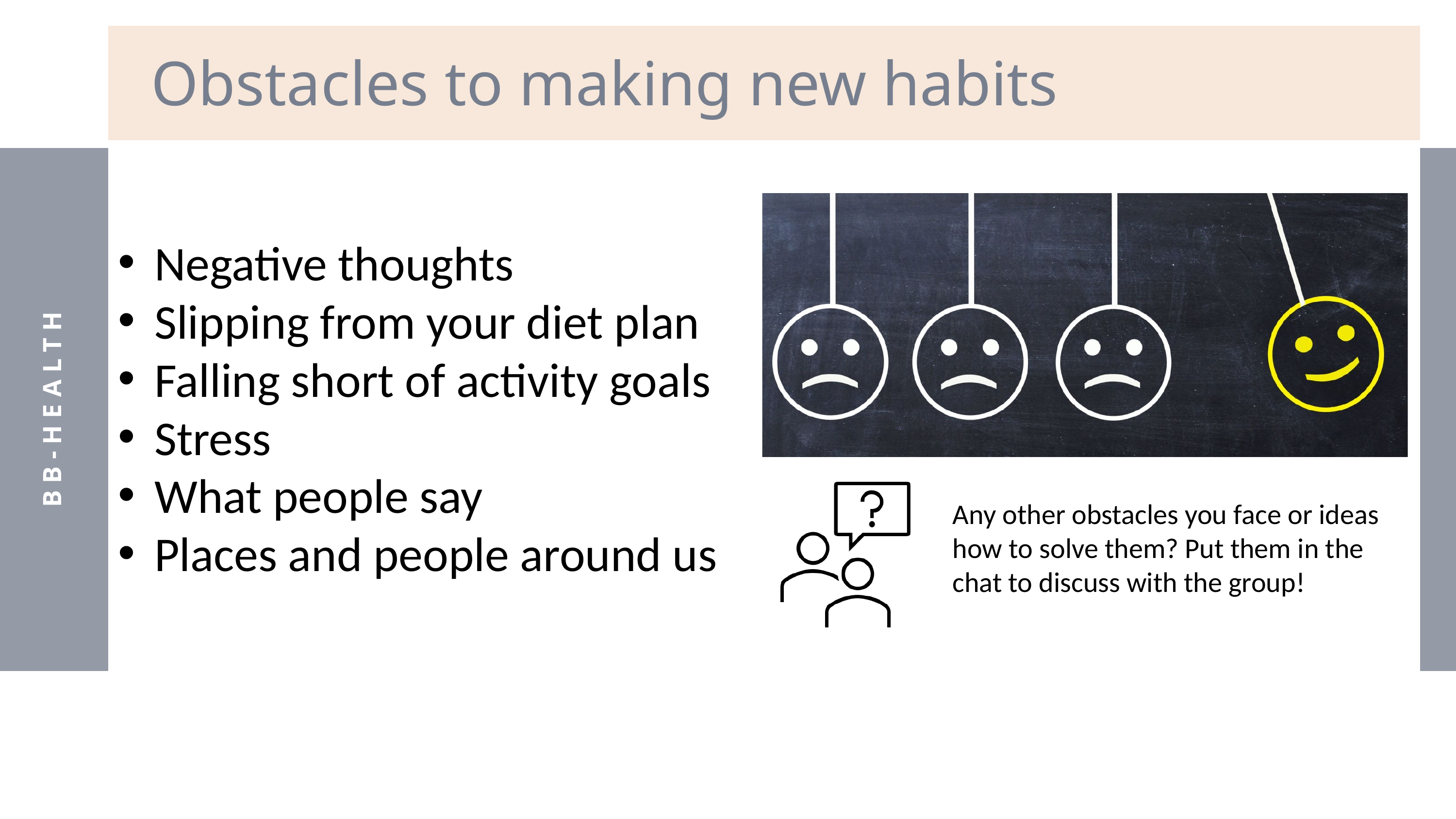

Obstacles to making new habits
Negative thoughts
Slipping from your diet plan
Falling short of activity goals
Stress
What people say
Places and people around us
BB-HEALTH
Any other obstacles you face or ideas how to solve them? Put them in the chat to discuss with the group!

## Slide 6
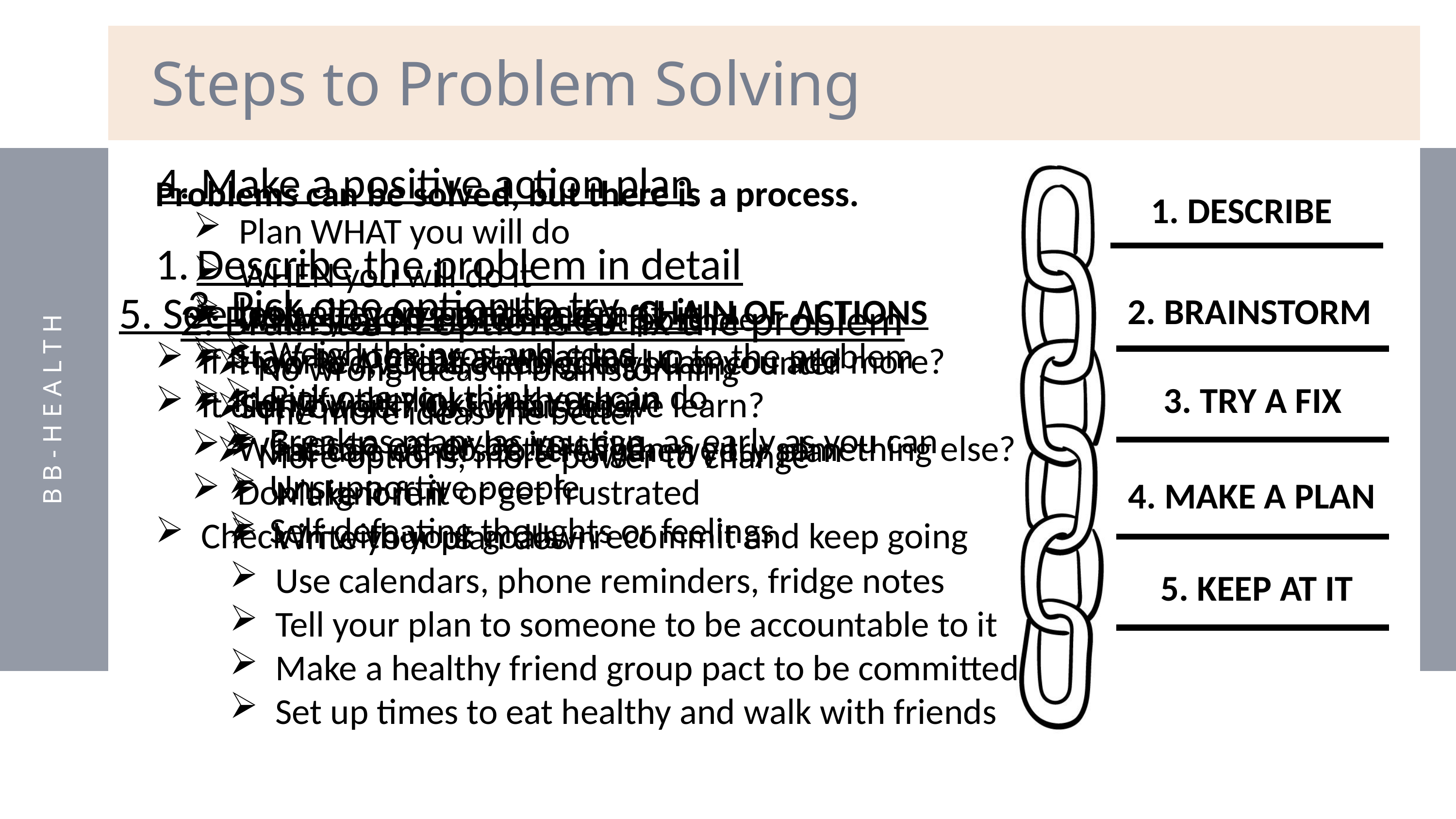

Steps to Problem Solving
4. Make a positive action plan
Plan WHAT you will do
WHEN you will do it
What you NEED to make it possible
How to AVOID roadblocks you encounter
Set yourself up for success:
Include others to strengthen your plan
Make it fun
Write your plan down
Use calendars, phone reminders, fridge notes
Tell your plan to someone to be accountable to it
Make a healthy friend group pact to be committed
Set up times to eat healthy and walk with friends
Problems can be solved, but there is a process.
Describe the problem in detail
Look at every problem as a CHAIN OF ACTIONS
Start by looking at what led up to the problem
Identify the links in the chain
Cues to eat or be inactive
Unsupportive people
Self-defeating thoughts or feelings
1. DESCRIBE
2. Brainstorm options to fix the problem
No wrong ideas in brainstorming
The more ideas the better
More options, more power to change
3. Pick one option to try
Weigh the pros and cons
Pick one you think you can do
Break as many as you can, as early as you can
5. See how it goes and keep at it
If it worked, great, keep going! Can you add more?
It didn’t work? Ok, what did we learn?
What can we do better when we try something else?
Don’t ignore it or get frustrated
Check in with your goals – recommit and keep going
2. BRAINSTORM
3. TRY A FIX
BB-HEALTH
4. MAKE A PLAN
5. KEEP AT IT

## Slide 7
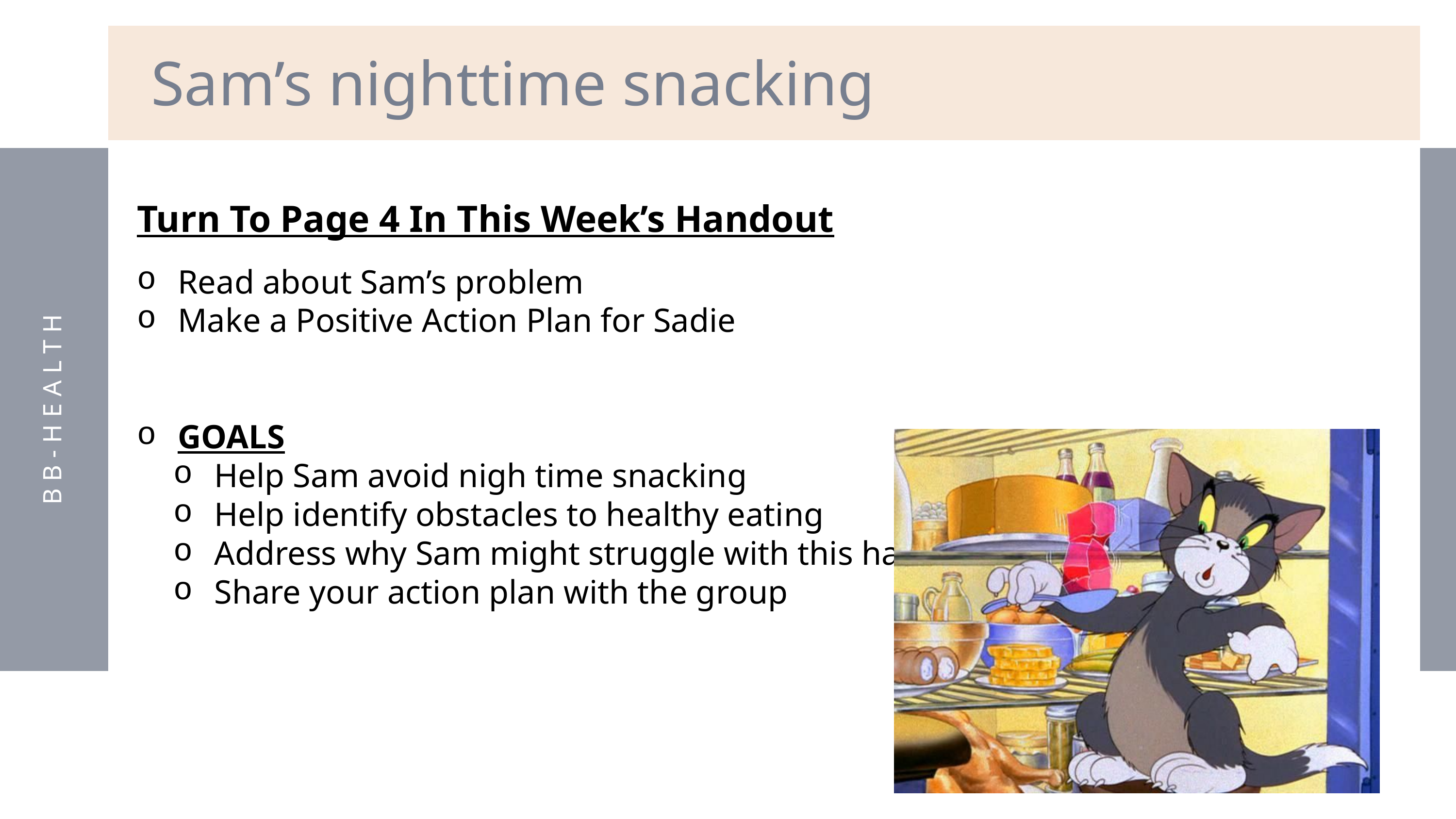

Sam’s nighttime snacking
Turn To Page 4 In This Week’s Handout
Read about Sam’s problem
Make a Positive Action Plan for Sadie
GOALS
Help Sam avoid nigh time snacking
Help identify obstacles to healthy eating
Address why Sam might struggle with this habit
Share your action plan with the group
BB-HEALTH

## Slide 8
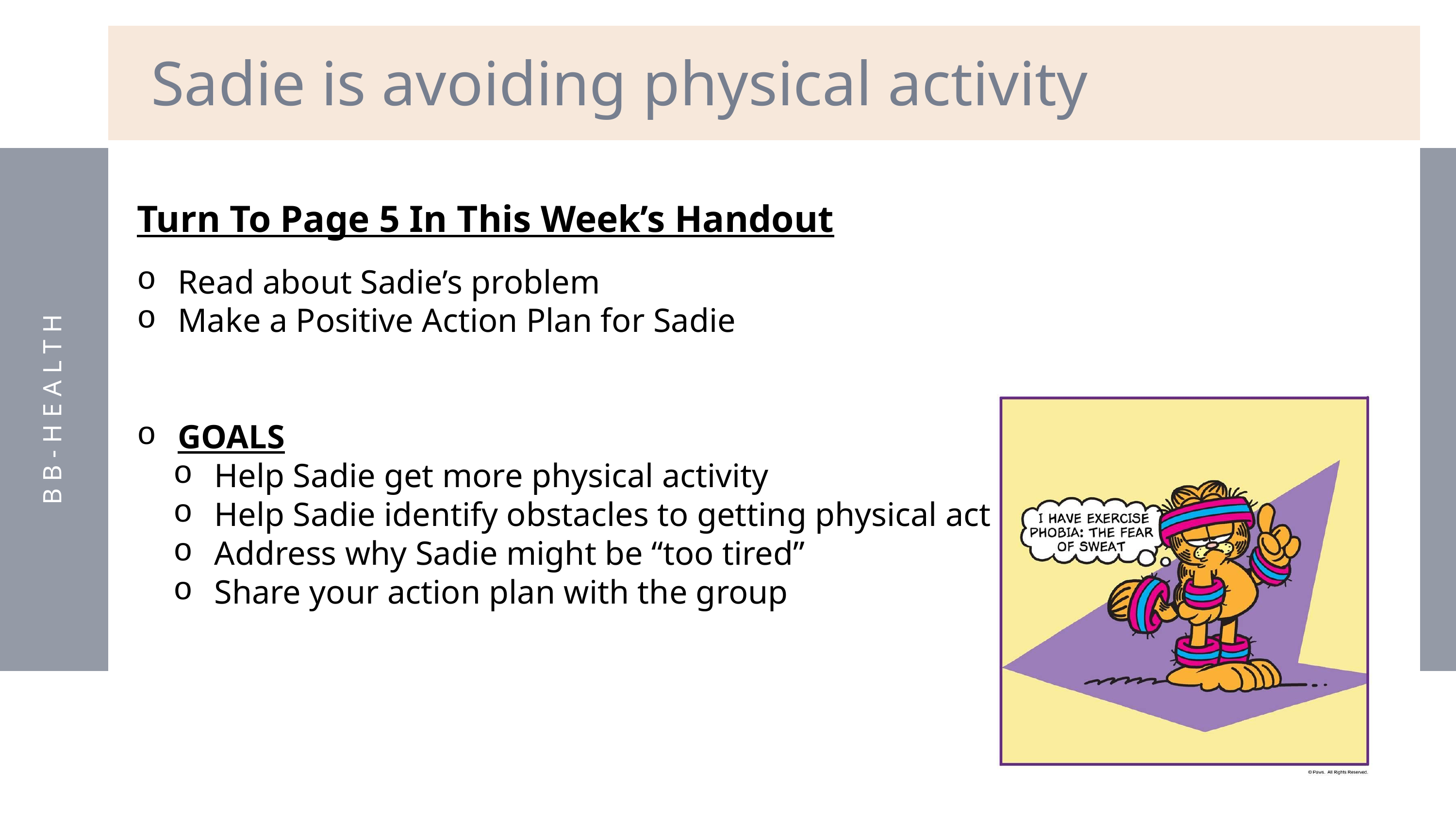

Sadie is avoiding physical activity
Turn To Page 5 In This Week’s Handout
Read about Sadie’s problem
Make a Positive Action Plan for Sadie
GOALS
Help Sadie get more physical activity
Help Sadie identify obstacles to getting physical activity
Address why Sadie might be “too tired”
Share your action plan with the group
BB-HEALTH

## Slide 9
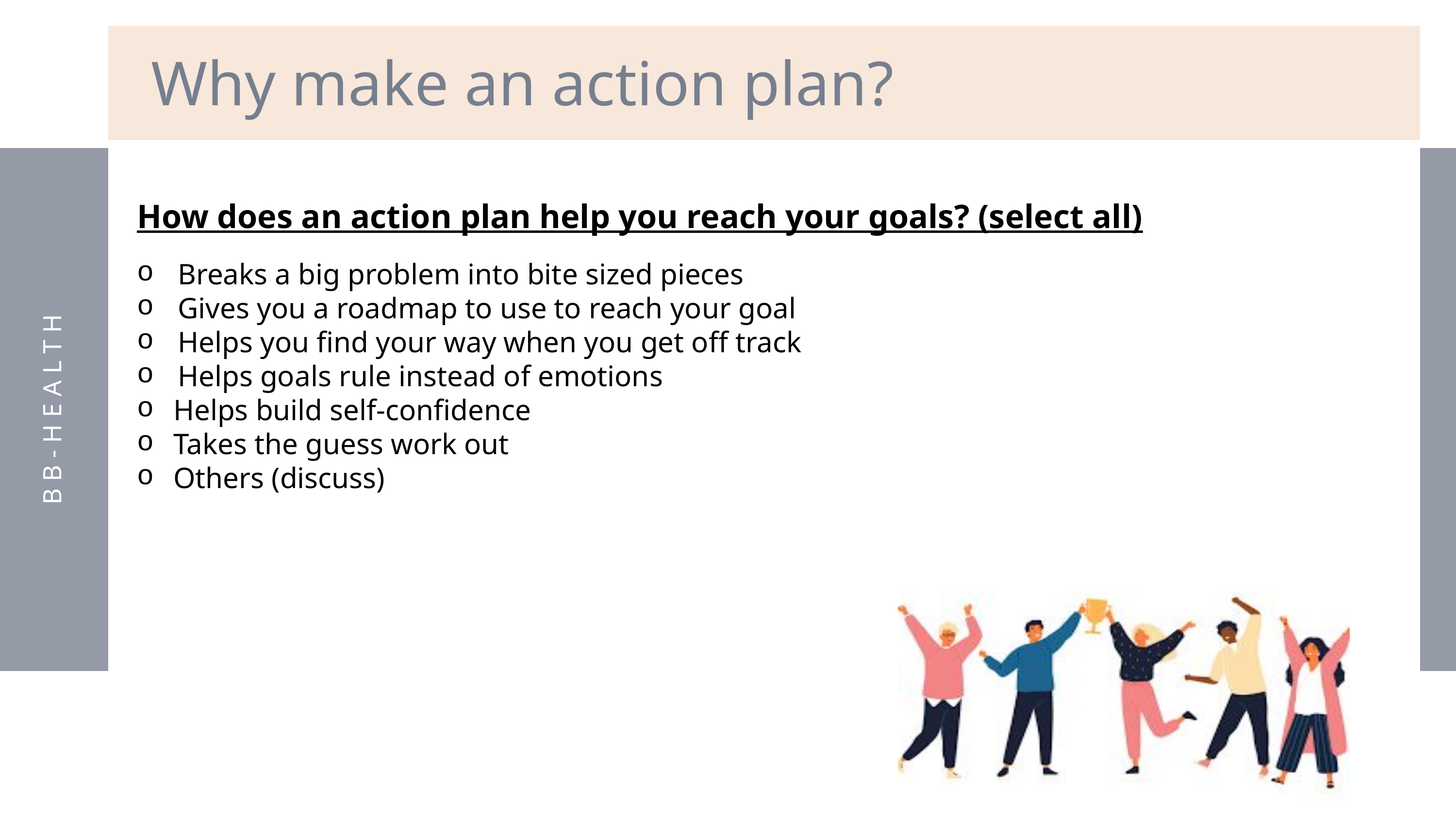

Why make an action plan?
How does an action plan help you reach your goals? (select all)
Breaks a big problem into bite sized pieces
Gives you a roadmap to use to reach your goal
Helps you find your way when you get off track
Helps goals rule instead of emotions
Helps build self-confidence
Takes the guess work out
Others (discuss)
BB-HEALTH

## Slide 10
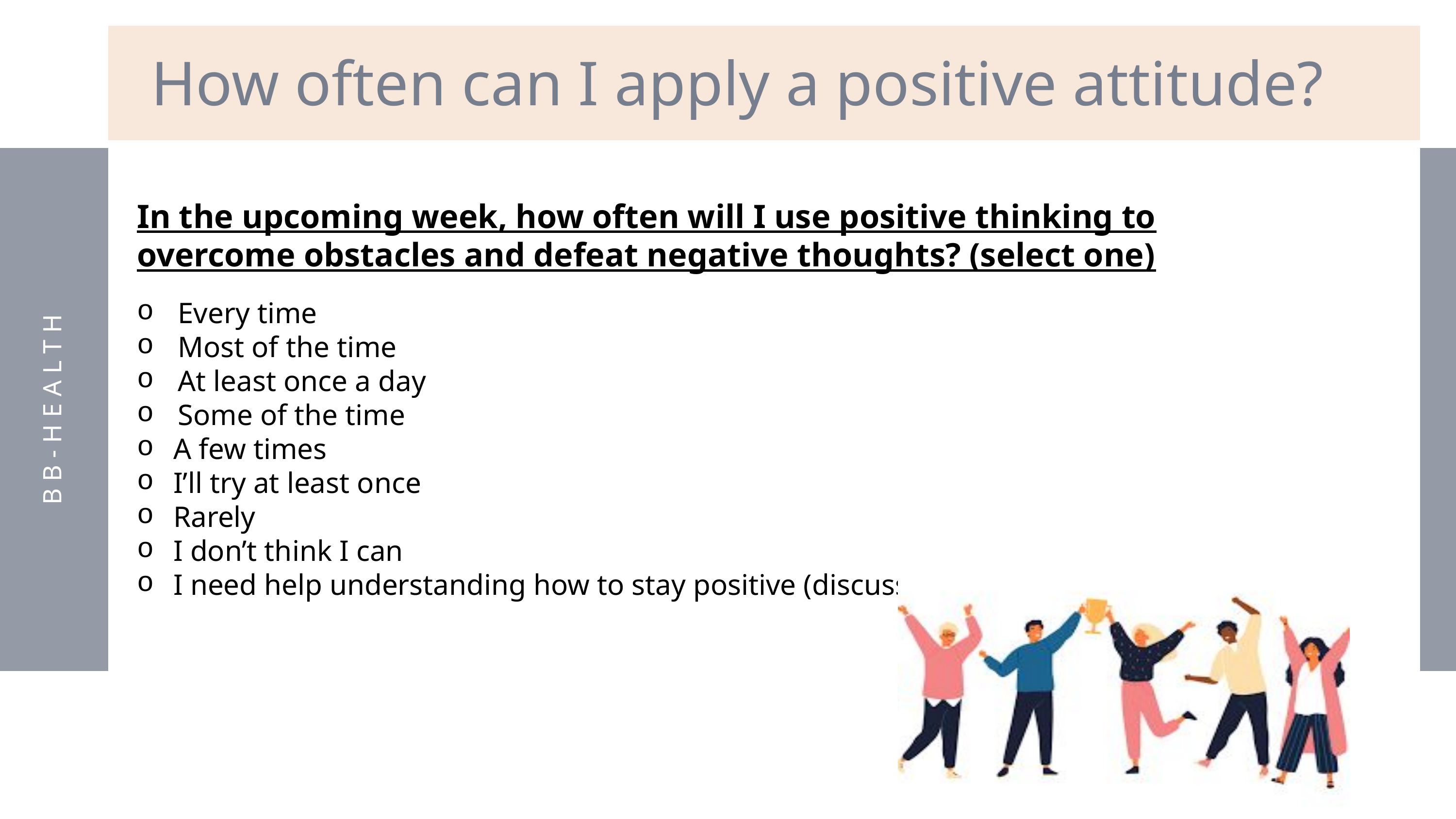

How often can I apply a positive attitude?
In the upcoming week, how often will I use positive thinking to overcome obstacles and defeat negative thoughts? (select one)
Every time
Most of the time
At least once a day
Some of the time
A few times
I’ll try at least once
Rarely
I don’t think I can
I need help understanding how to stay positive (discuss)
BB-HEALTH

## Slide 11
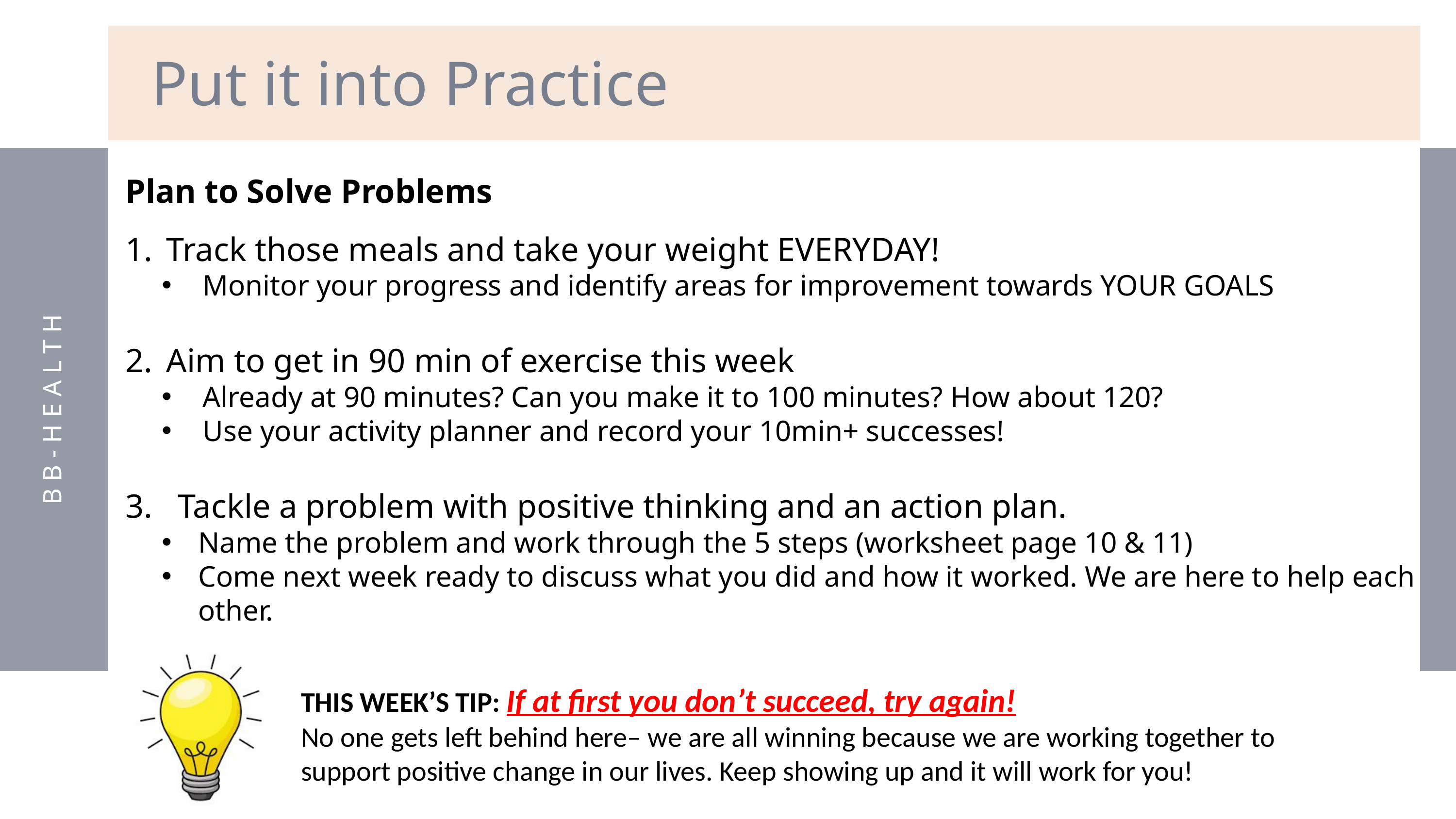

Put it into Practice
Plan to Solve Problems
Track those meals and take your weight EVERYDAY!
Monitor your progress and identify areas for improvement towards YOUR GOALS
Aim to get in 90 min of exercise this week
Already at 90 minutes? Can you make it to 100 minutes? How about 120?
Use your activity planner and record your 10min+ successes!
3. Tackle a problem with positive thinking and an action plan.
Name the problem and work through the 5 steps (worksheet page 10 & 11)
Come next week ready to discuss what you did and how it worked. We are here to help each other.
BB-HEALTH
THIS WEEK’S TIP: If at first you don’t succeed, try again!
No one gets left behind here– we are all winning because we are working together to support positive change in our lives. Keep showing up and it will work for you!

## Slide 12
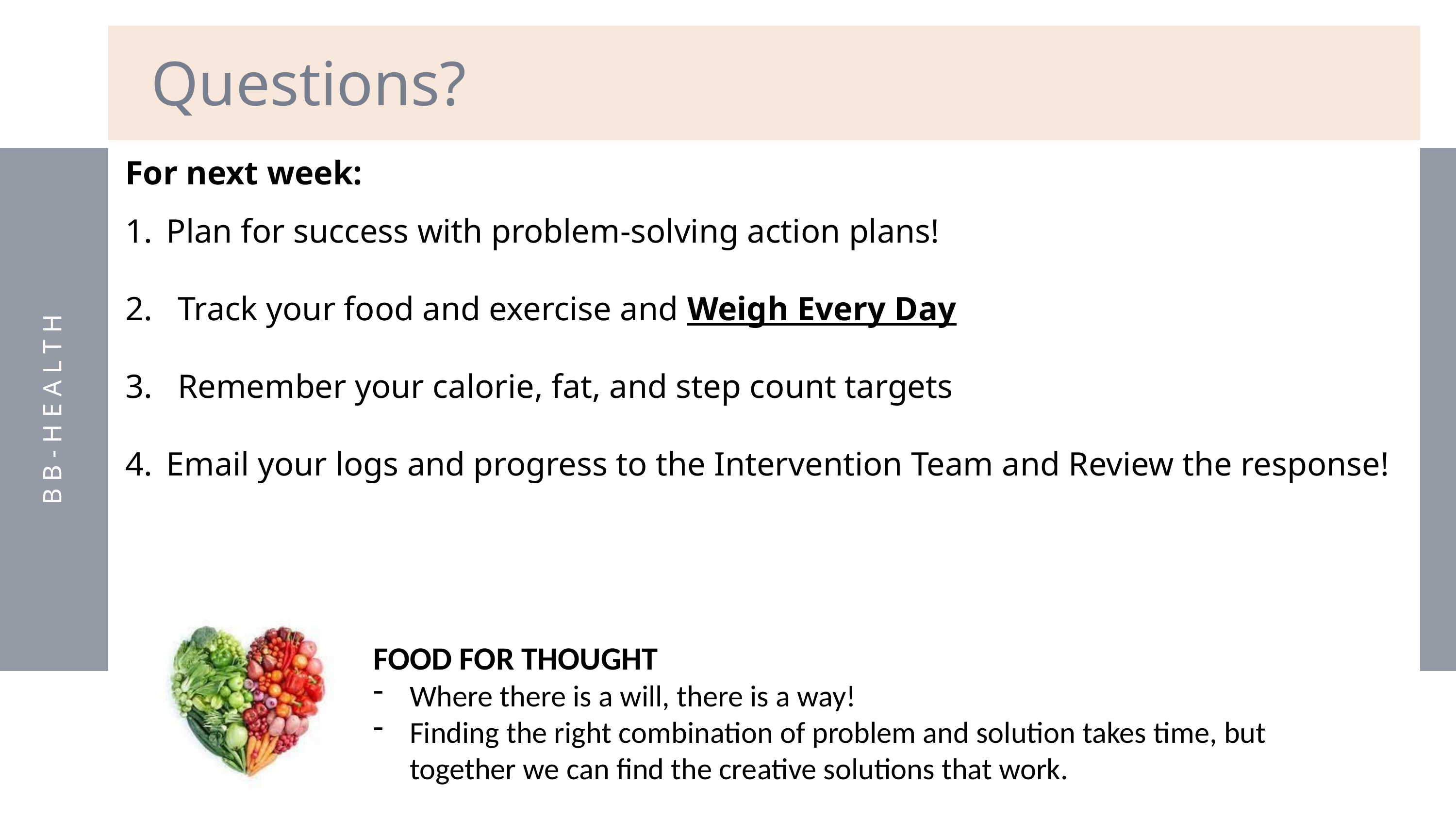

Questions?
For next week:
Plan for success with problem-solving action plans!
2. Track your food and exercise and Weigh Every Day
3. Remember your calorie, fat, and step count targets
Email your logs and progress to the Intervention Team and Review the response!
BB-HEALTH
FOOD FOR THOUGHT
Where there is a will, there is a way!
Finding the right combination of problem and solution takes time, but together we can find the creative solutions that work.
