# Supplementary material for: Development and Feasibility of an eHealth Diabetes Prevention Program Adapted for Older Adults—Results from a Randomized Control Pilot Study
Source: Nutrients. 2024 Mar 23;16(7):930. doi: 10.3390/nu16070930 (PMC11154527; doi:10.3390/nu16070930)
Supplement: Supplementary file 1 [file nutrients-16-00930-s001.zip › Week7_SUPP.pdf]

BB-HEALTH

e-Healthy Older Adults Modification

Diabetes Prevention Program

WEEK #7
Problem Solving!

Group Opening Discussion

WELCOME BACK! **HOW WAS LAST WEEK**?

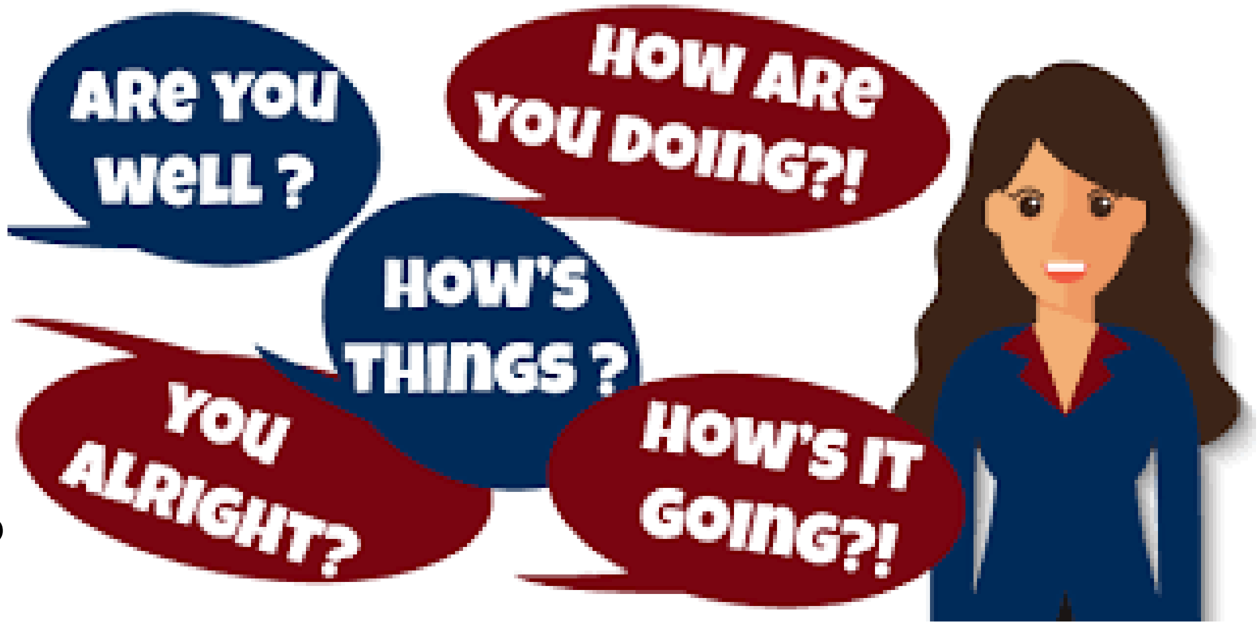

1. Did you track every meal?
2. Did you weigh every day, & record your weight today?
3. Did you eliminate problem cues?
4. Did you create healthy cues?
5. Did you email the Intervention Team and review the response?
6. What were your unique challenges and successes?

REMINDER: CHARGE YOUR FITBIT & SCALE DURING THIS MEETING!

What did you do to stay on track?

<u>What were the positive HEALTHY Cues that Worked for You? (select all)</u>

- ○ Keeping veggies and fruit on hand
- ○ Carrying a water bottle around
- ○ Staying away from the kitchen
- ○ Sticking to the fresh foods part of the grocery store
- ○ Kept my walking shoes visible
- ○ Left motivational notes around
- ○ Kept my scale out and visible to weigh everyday
- ○ Other (discuss)

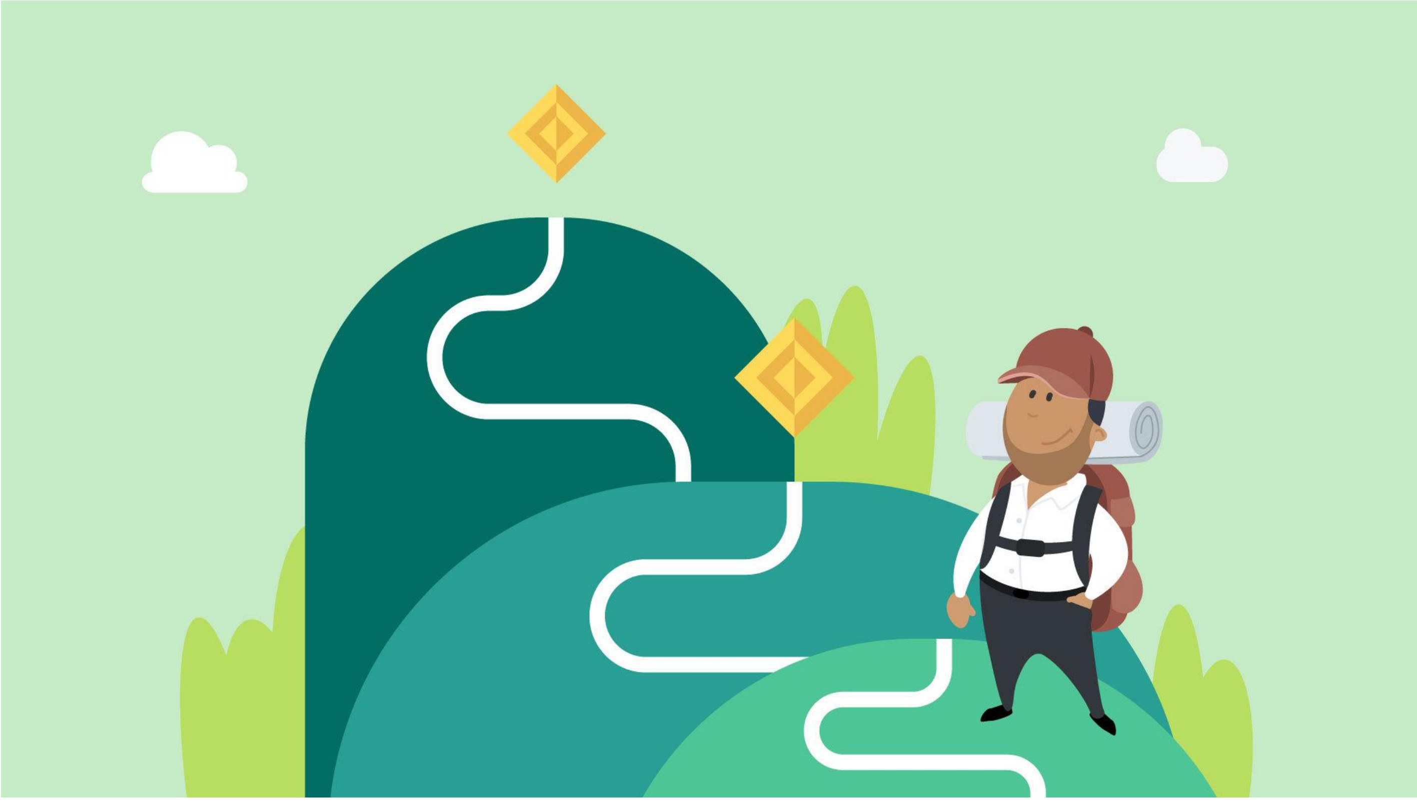

AIM FOR SUCCESS

These strategies are important to recognize and build on! Keep adding more and use multiple approaches to stay on track!

What are your most STICKY negative cues?

Bad habits & problem cues you think will be hardest to change (or you don't want to?)

- Eating in front of the TV, or while doing other activities
- Snacking out of boredom
- Eating past fullness
- Popcorn at the movies, and snacks at the party
- Turning down food when offered
- Resisting eating food the looks or smells good, when it isn't time to eat
- Other (discuss)

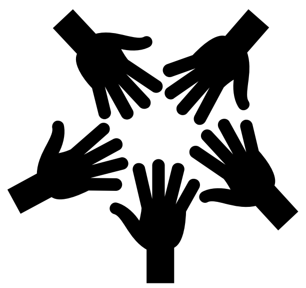

Self-Control only gets you so far!

Being willing to change your problem cues is important, but you can't change everything overnight! Focusing on the things you can change will help you see more improvement. The changes you can stick to are the changes that will lead to success

Obstacles to making new habits

- Negative thoughts
- Slipping from your diet plan
- Falling short of activity goals
- Stress
- What people say
- Places and people around us

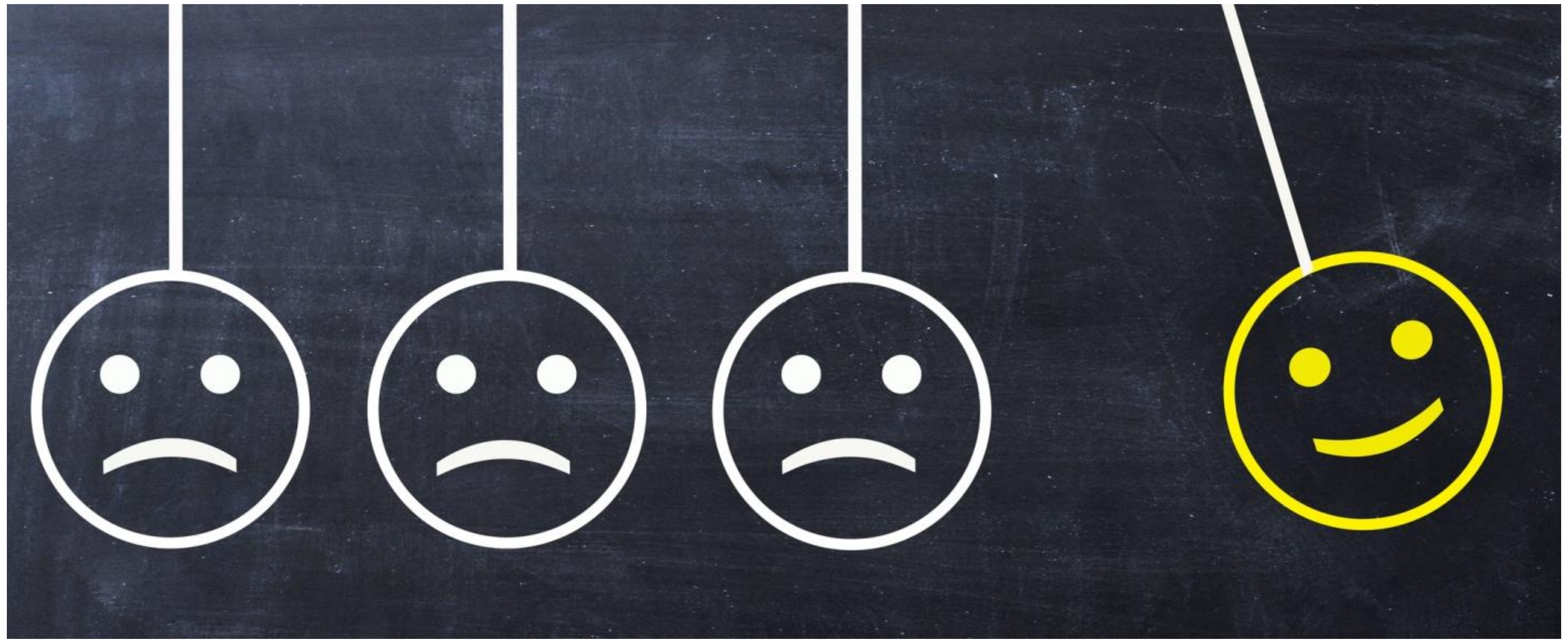

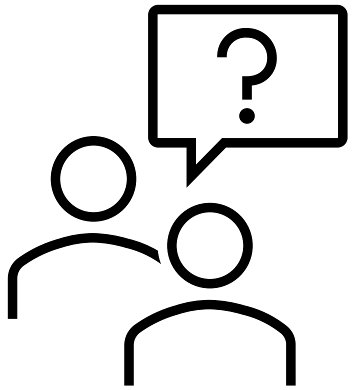

Any other obstacles you face or ideas how to solve them? Put them in the chat to discuss with the group!

Steps to Problem Solving

Problems can be solved, but there is a process.

1. Describe the problem in detail

2. Brainstorm options to fix the problem

3. Pick one option to try

4. Make a positive action plan

5. Se[illegible]

CHAIN OF ACTIONS

- Plan WHAT you will do
- WHEN you will do it
- Weigh the pros and cons
- No wrong ideas in brainstorming
- Pick one you think you can do
- The more ideas the better
- More options, more power to change
- Unsupportive people
- Self-defeating thoughts or feelings
- [illegible]
- If it worked, [illegible] more?
- If it didn't work, [illegible] learn?
- What can [illegible] something else?
- Don't give in or get frustrated
- Check [illegible] recommit and keep going
- Write your plan down
  - Use calendars, phone reminders, fridge notes
  - Tell your plan to someone to be accountable to it
  - Make a healthy friend group pact to be committed
  - Set up times to eat healthy and walk with friends

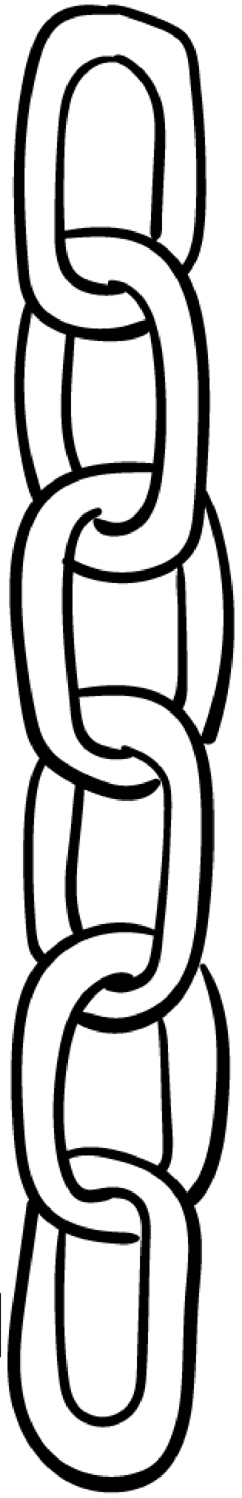

1. DESCRIBE

2. BRAINSTORM

3. TRY A FIX

4. MAKE A PLAN

5. KEEP AT IT

Sam's nighttime snacking

B B - H E A L T H

<u>Turn To Page 4 In This Week's Handout</u>

- Read about Sam's problem
- Make a Positive Action Plan for Sadie

- **<u>GOALS</u>**
  - Help Sam avoid nigh time snacking
  - Help identify obstacles to healthy eating
  - Address why Sam might struggle with this habit
  - Share your action plan with the group

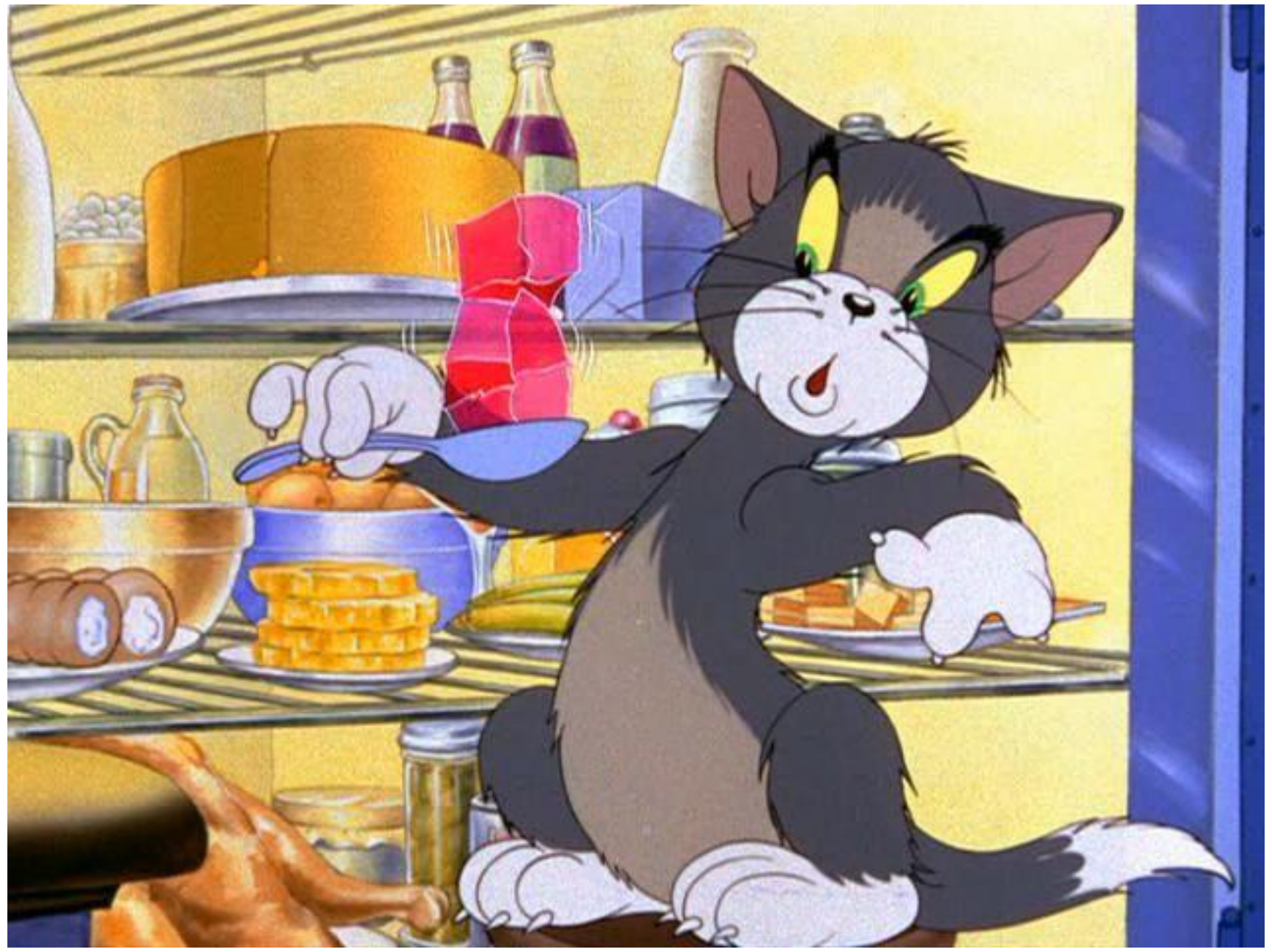

Sadie is avoiding physical activity

B B - H E A L T H

Turn To Page 5 In This Week's Handout

- Read about Sadie's problem
- Make a Positive Action Plan for Sadie

- **GOALS**
  - Help Sadie get more physical activity
  - Help Sadie identify obstacles to getting physical activity
  - Address why Sadie might be "too tired"
  - Share your action plan with the group

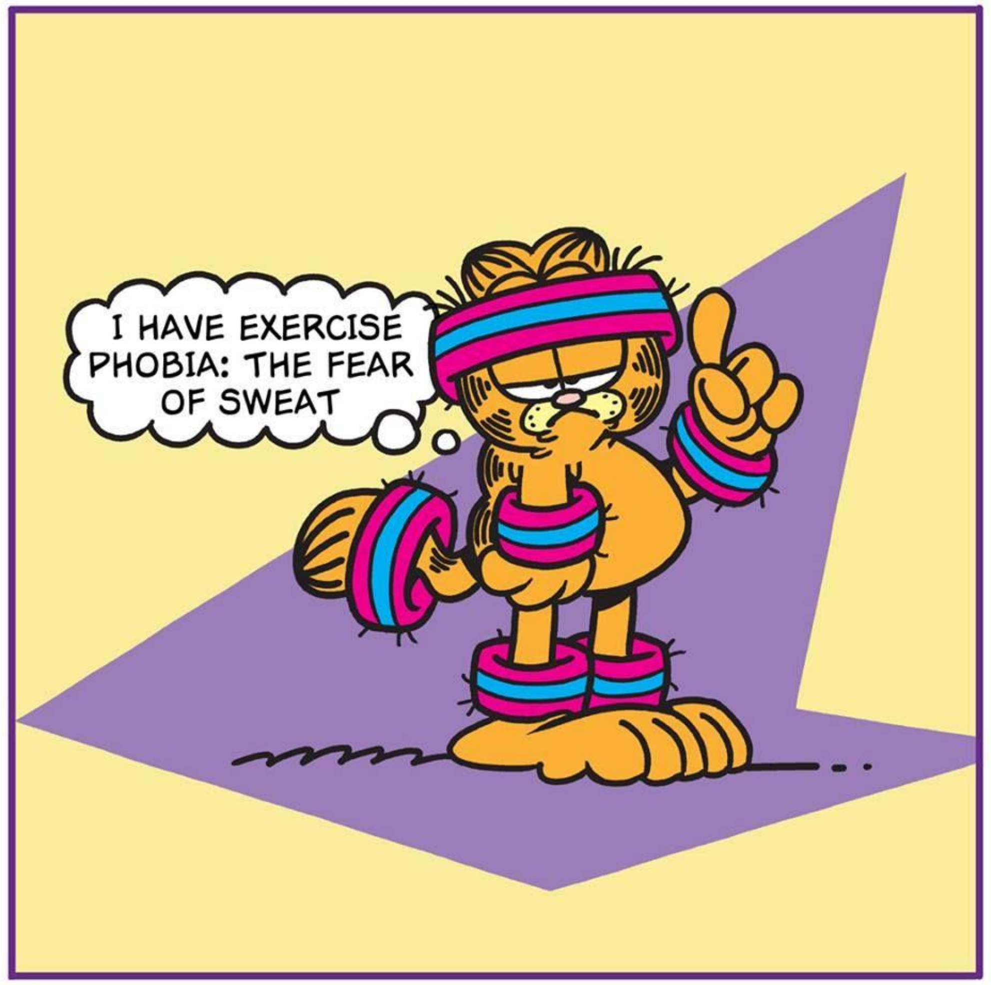

© Paws. All Rights Reserved.

Why make an action plan?

BB-HEALTH

How does an action plan help you reach your goals? (select all)

- Breaks a big problem into bite sized pieces
- Gives you a roadmap to use to reach your goal
- Helps you find your way when you get off track
- Helps goals rule instead of emotions
- Helps build self-confidence
- Takes the guess work out
- Others (discuss)

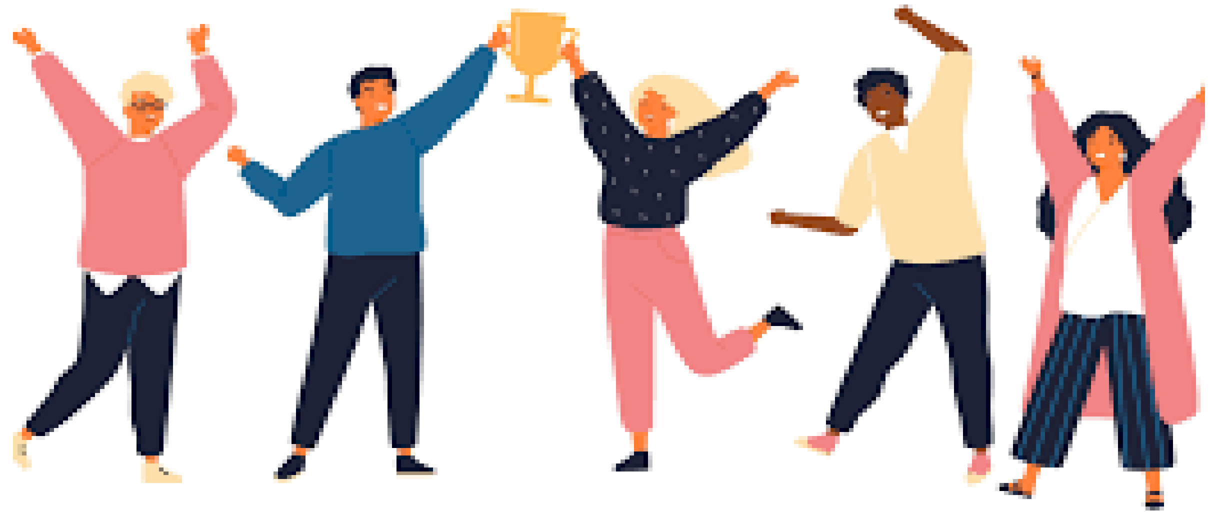

How often can I apply a positive attitude?

B B - H E A L T H

<u>In the upcoming week, how often will I use positive thinking to overcome obstacles and defeat negative thoughts? (select one)</u>

- ○ Every time
- ○ Most of the time
- ○ At least once a day
- ○ Some of the time
- ○ A few times
- ○ I’ll try at least once
- ○ Rarely
- ○ I don’t think I can
- ○ I need help understanding how to stay positive (discuss)

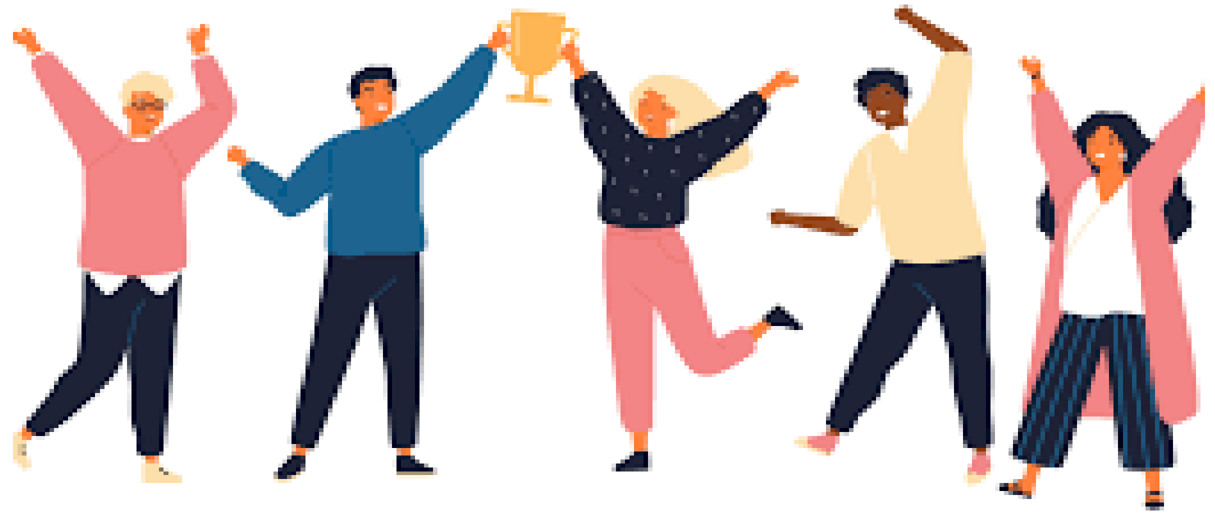

Put it into Practice

B B - H E A L T H

Plan to Solve Problems

1. Track those meals and take your weight EVERYDAY!
   - Monitor your progress and identify areas for improvement towards YOUR GOALS

2. Aim to get in 90 min of exercise this week
   - Already at 90 minutes? Can you make it to 100 minutes? How about 120?
   - Use your activity planner and record your 10min+ successes!

3. Tackle a problem with positive thinking and an action plan.
   - Name the problem and work through the 5 steps (worksheet page 10 & 11)
   - Come next week ready to discuss what you did and how it worked. We are here to help each other.

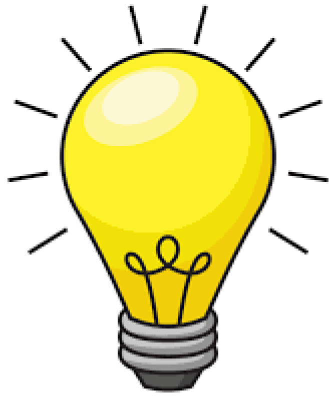

THIS WEEK'S TIP: ***If at first you don't succeed, try again!***
No one gets left behind here– we are all winning because we are working together to support positive change in our lives. Keep showing up and it will work for you!

Questions?

B B - H E A L T H

For next week:

1. Plan for success with problem-solving action plans!

2. Track your food and exercise and **<u>Weigh Every Day</u>**

3. Remember your calorie, fat, and step count targets

4. Email your logs and progress to the Intervention Team and Review the response!

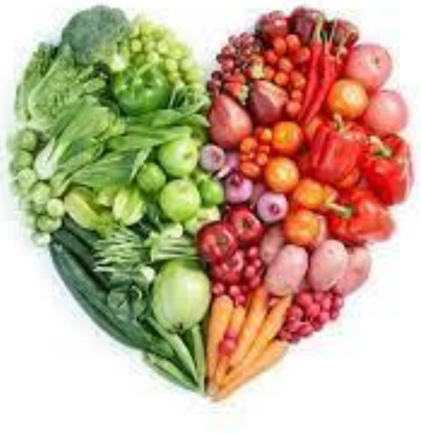

FOOD FOR THOUGHT

- Where there is a will, there is a way!
- Finding the right combination of problem and solution takes time, but together we can find the creative solutions that work.
