# Supplementary material for: Development and Feasibility of an eHealth Diabetes Prevention Program Adapted for Older Adults—Results from a Randomized Control Pilot Study
Source: Nutrients. 2024 Mar 23;16(7):930. doi: 10.3390/nu16070930 (PMC11154527; doi:10.3390/nu16070930)
Supplement: Supplementary file 1 [file nutrients-16-00930-s001.zip › Week8.pptx]

## Slide 1
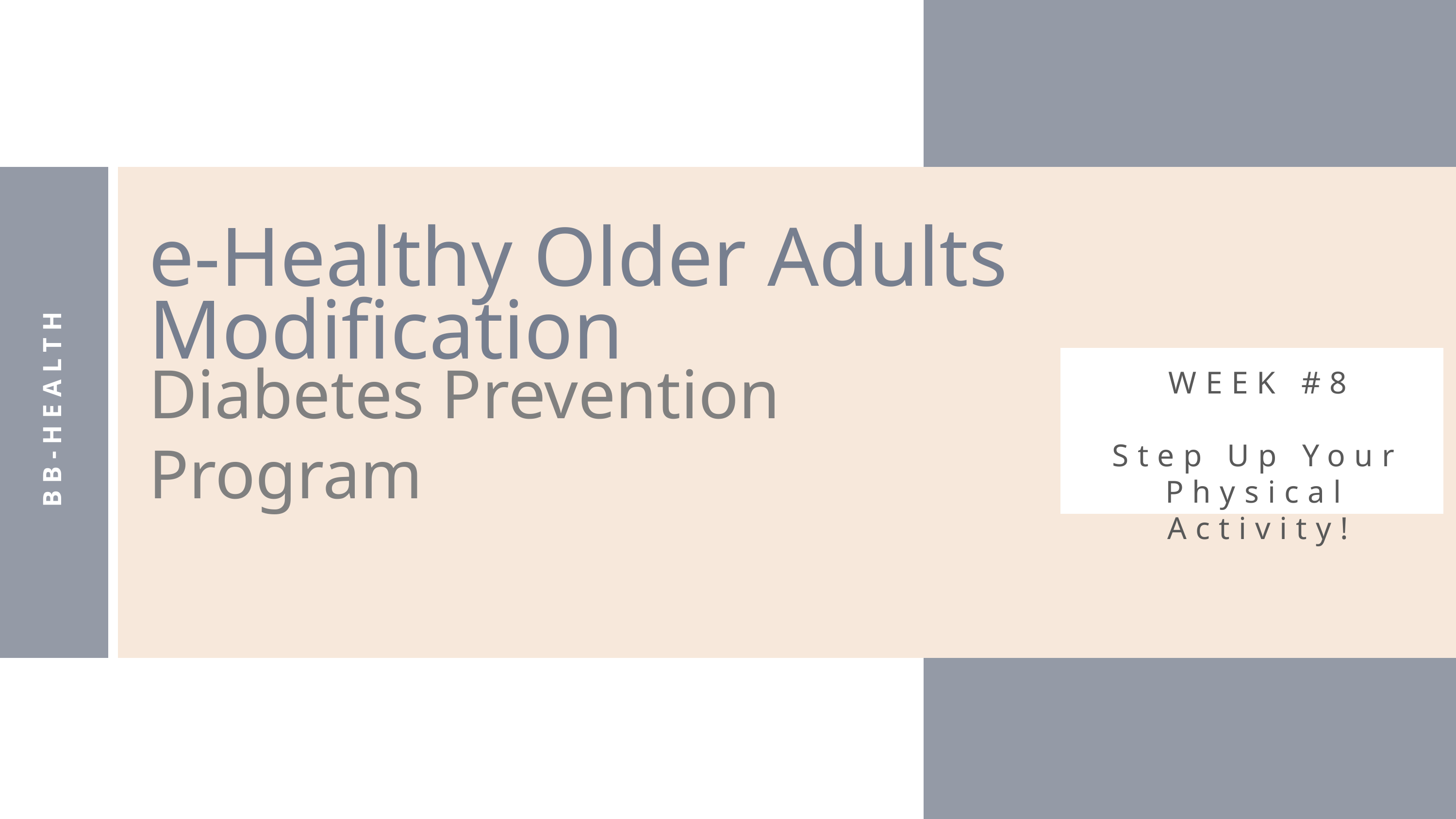

OPEN REPORTS
e-Healthy Older Adults Modification
WEEK #8
Step Up Your Physical Activity!
Diabetes Prevention Program
BB-HEALTH

## Slide 2
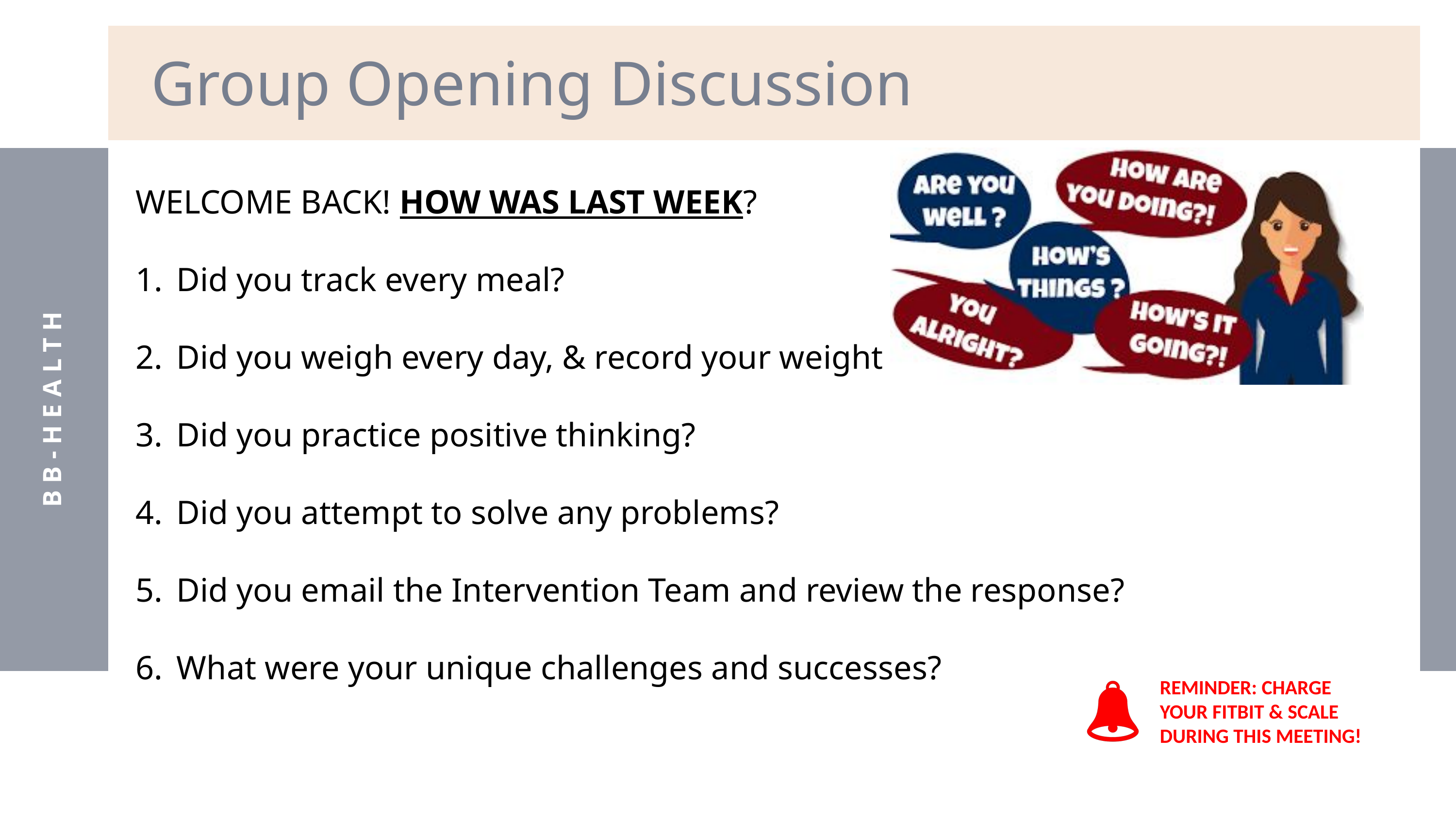

Group Opening Discussion
WELCOME BACK! HOW WAS LAST WEEK?
Did you track every meal?
Did you weigh every day, & record your weight today?
Did you practice positive thinking?
Did you attempt to solve any problems?
Did you email the Intervention Team and review the response?
What were your unique challenges and successes?
BB-HEALTH
REMINDER: CHARGE YOUR FITBIT & SCALE DURING THIS MEETING!

## Slide 3
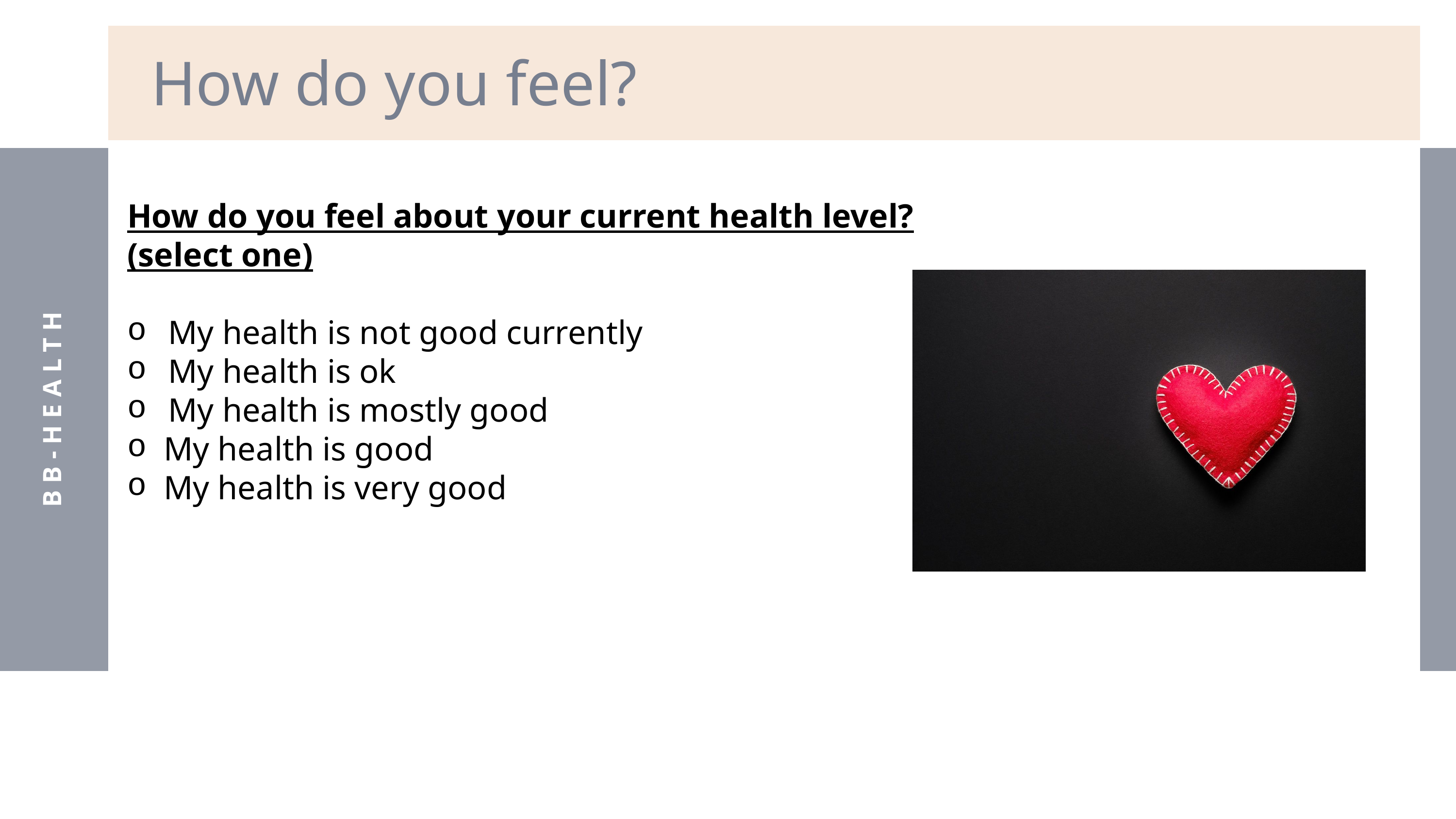

How do you feel?
How do you feel about your current health level? (select one)
My health is not good currently
My health is ok
My health is mostly good
My health is good
My health is very good
BB-HEALTH

## Slide 4
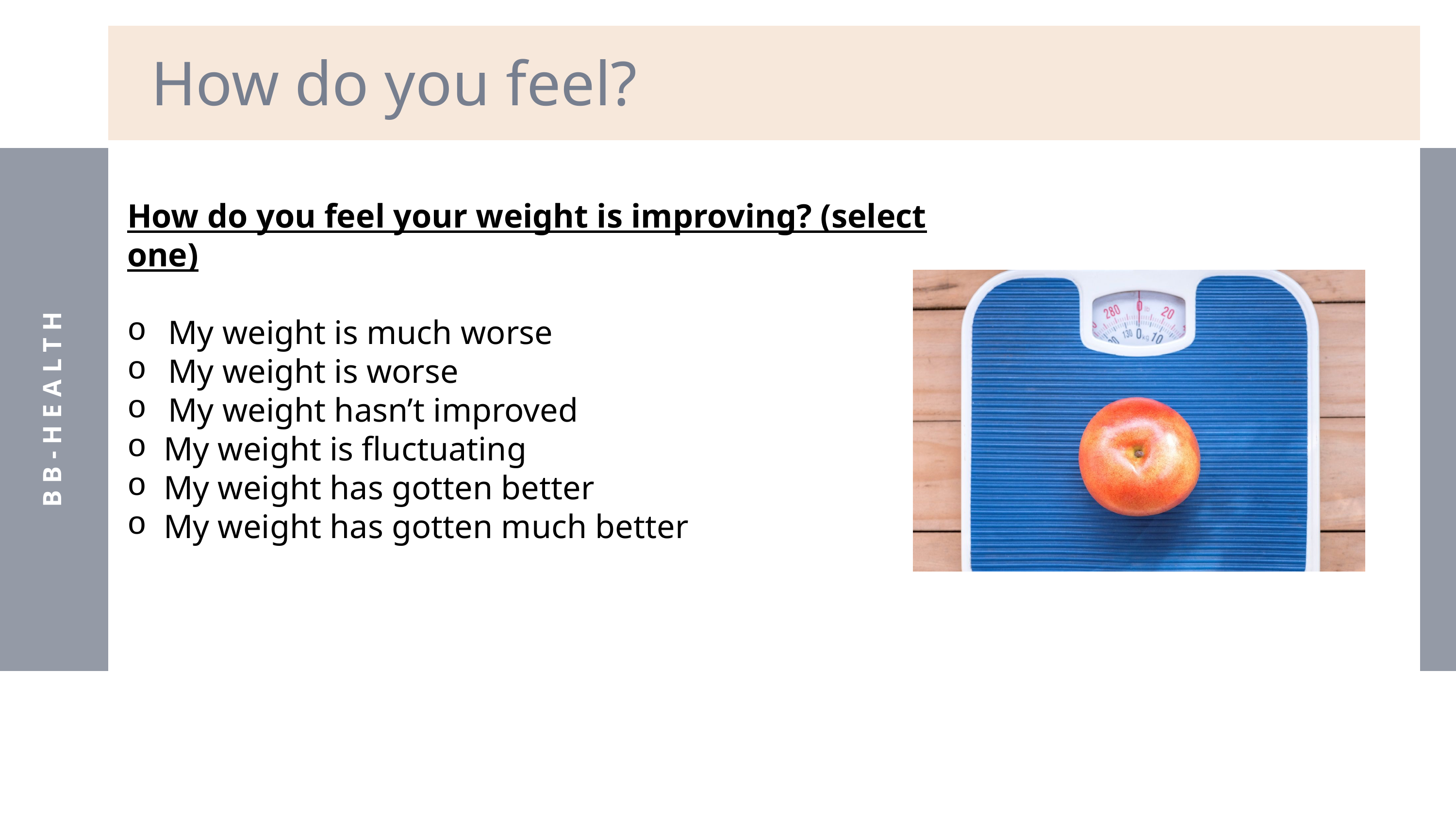

How do you feel?
How do you feel your weight is improving? (select one)
My weight is much worse
My weight is worse
My weight hasn’t improved
My weight is fluctuating
My weight has gotten better
My weight has gotten much better
BB-HEALTH

## Slide 5
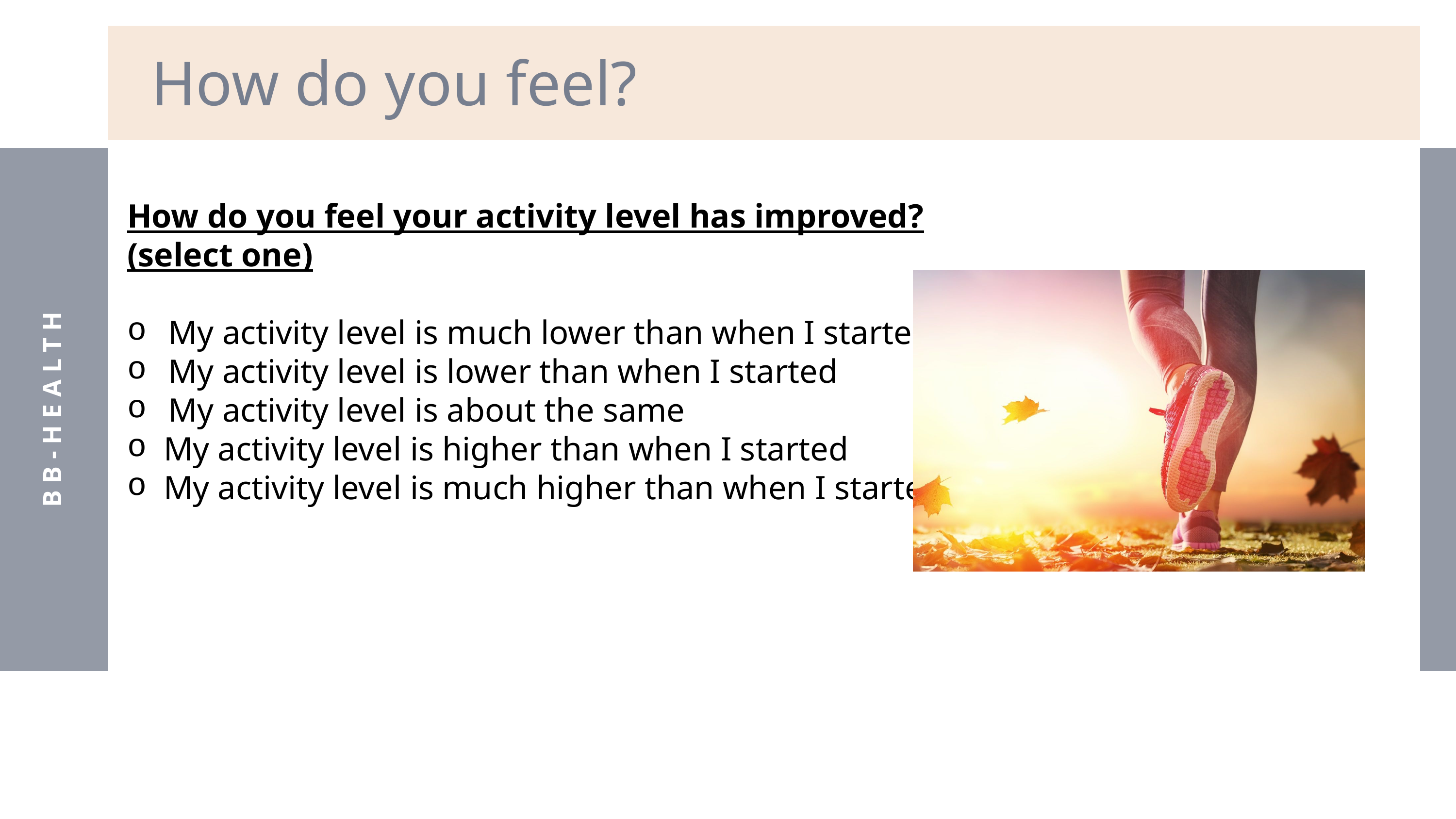

How do you feel?
How do you feel your activity level has improved? (select one)
My activity level is much lower than when I started
My activity level is lower than when I started
My activity level is about the same
My activity level is higher than when I started
My activity level is much higher than when I started
BB-HEALTH

## Slide 6
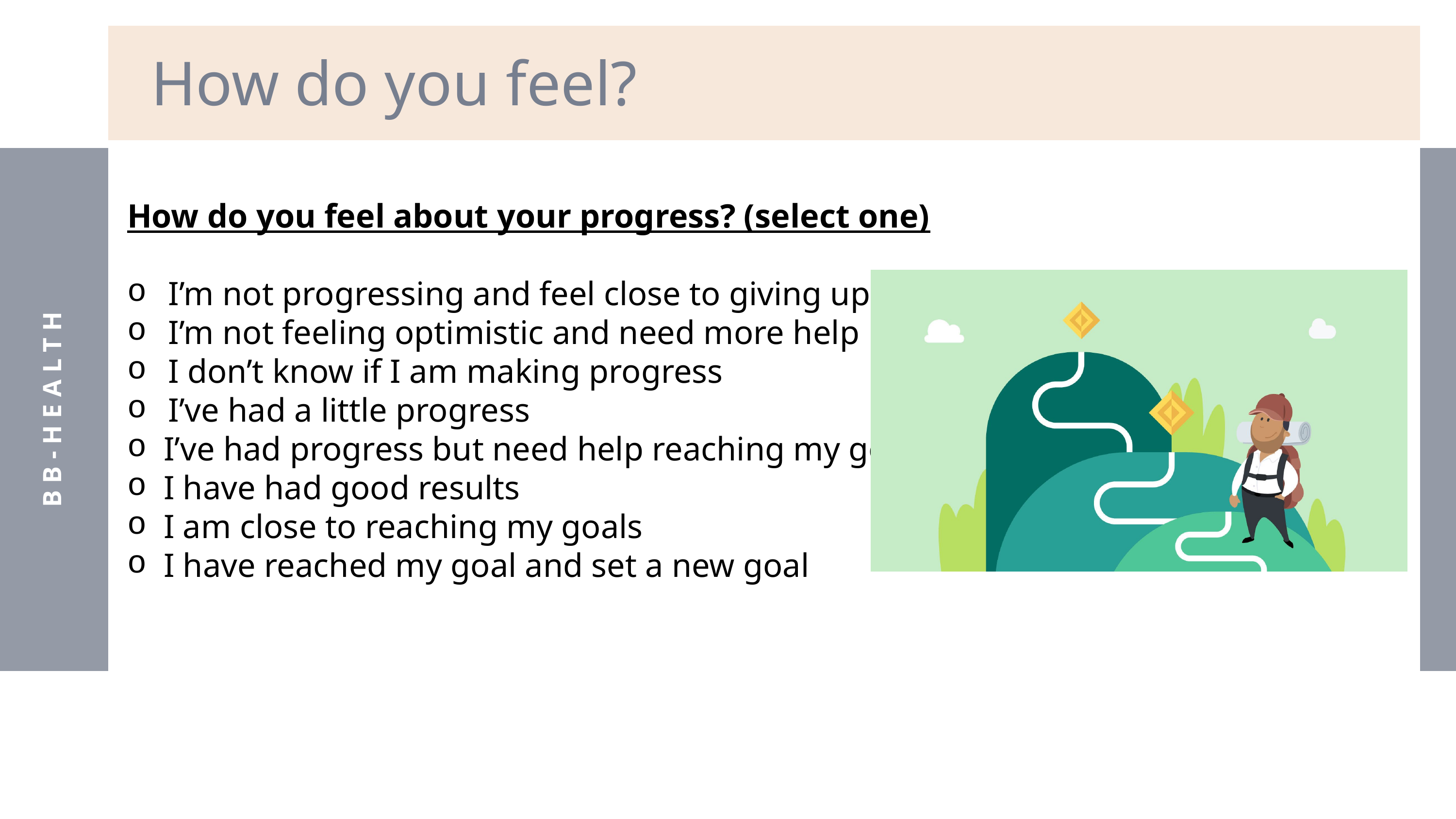

How do you feel?
How do you feel about your progress? (select one)
I’m not progressing and feel close to giving up
I’m not feeling optimistic and need more help
I don’t know if I am making progress
I’ve had a little progress
I’ve had progress but need help reaching my goals
I have had good results
I am close to reaching my goals
I have reached my goal and set a new goal
BB-HEALTH

## Slide 7
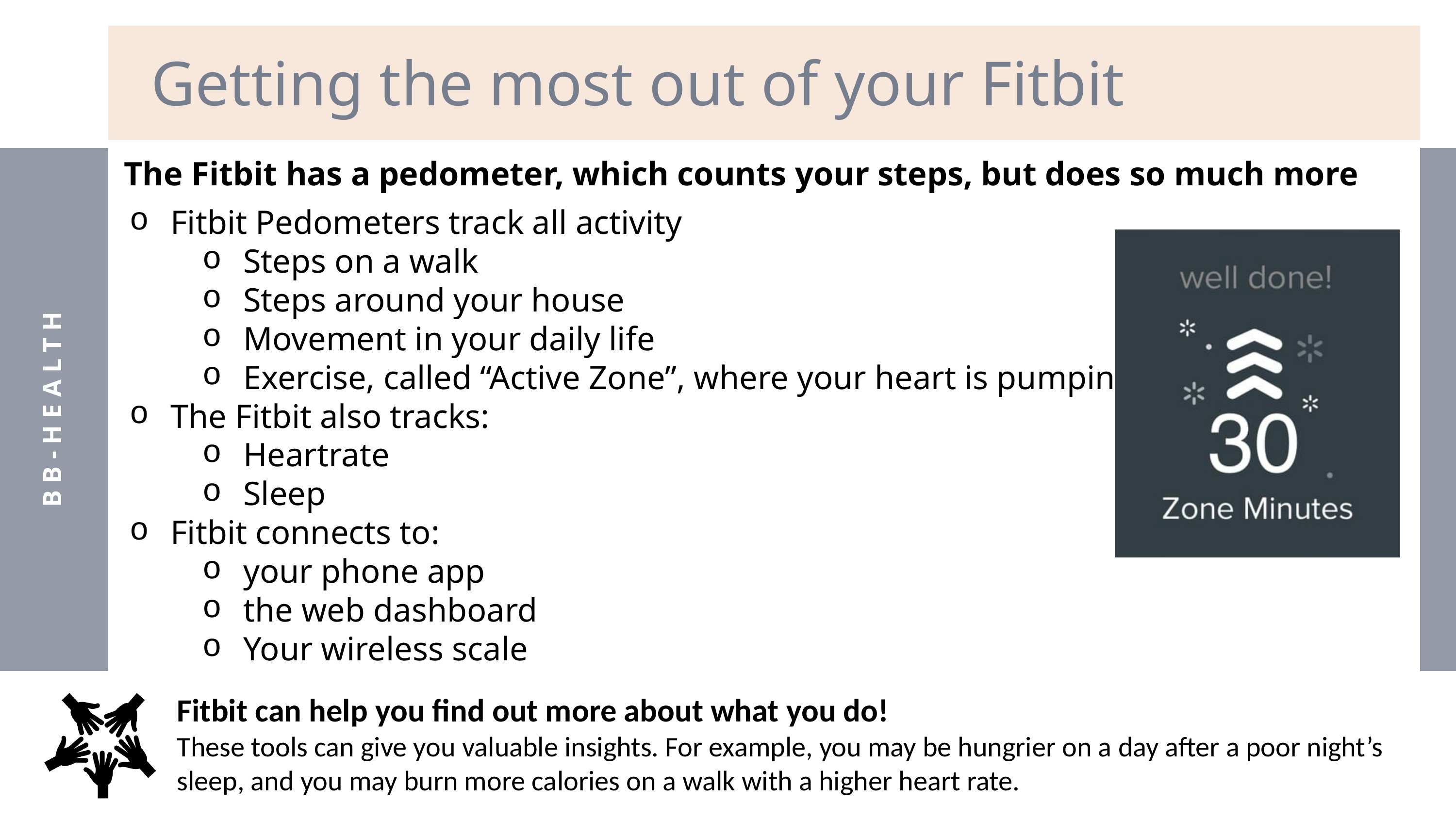

Getting the most out of your Fitbit
The Fitbit has a pedometer, which counts your steps, but does so much more
Fitbit Pedometers track all activity
Steps on a walk
Steps around your house
Movement in your daily life
Exercise, called “Active Zone”, where your heart is pumping 
The Fitbit also tracks:
Heartrate
Sleep
Fitbit connects to:
your phone app
the web dashboard
Your wireless scale
BB-HEALTH
Fitbit can help you find out more about what you do!
These tools can give you valuable insights. For example, you may be hungrier on a day after a poor night’s sleep, and you may burn more calories on a walk with a higher heart rate.

## Slide 8
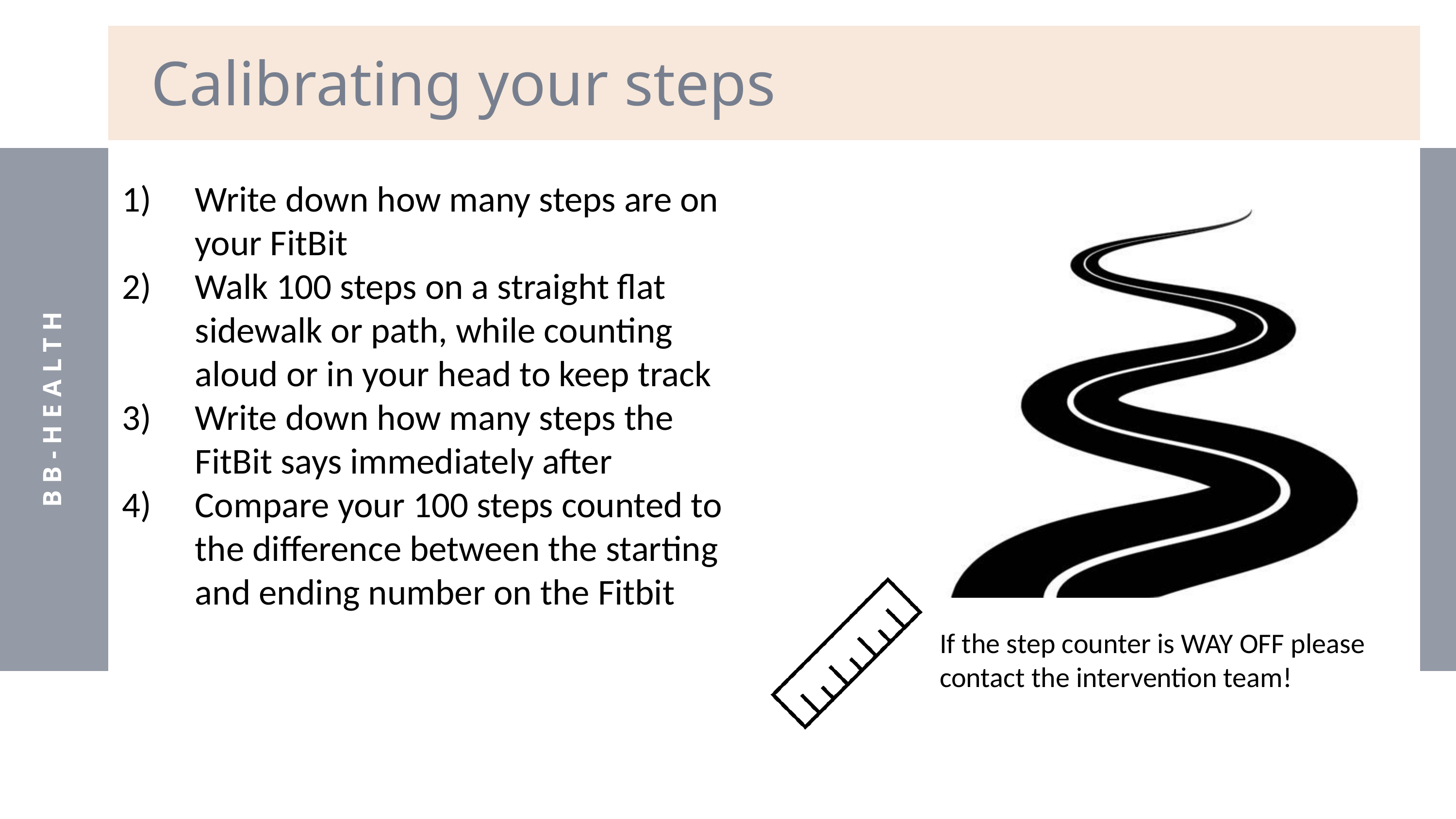

Calibrating your steps
Write down how many steps are on your FitBit
Walk 100 steps on a straight flat sidewalk or path, while counting aloud or in your head to keep track
Write down how many steps the FitBit says immediately after
Compare your 100 steps counted to the difference between the starting and ending number on the Fitbit
BB-HEALTH
If the step counter is WAY OFF please contact the intervention team!

## Slide 9
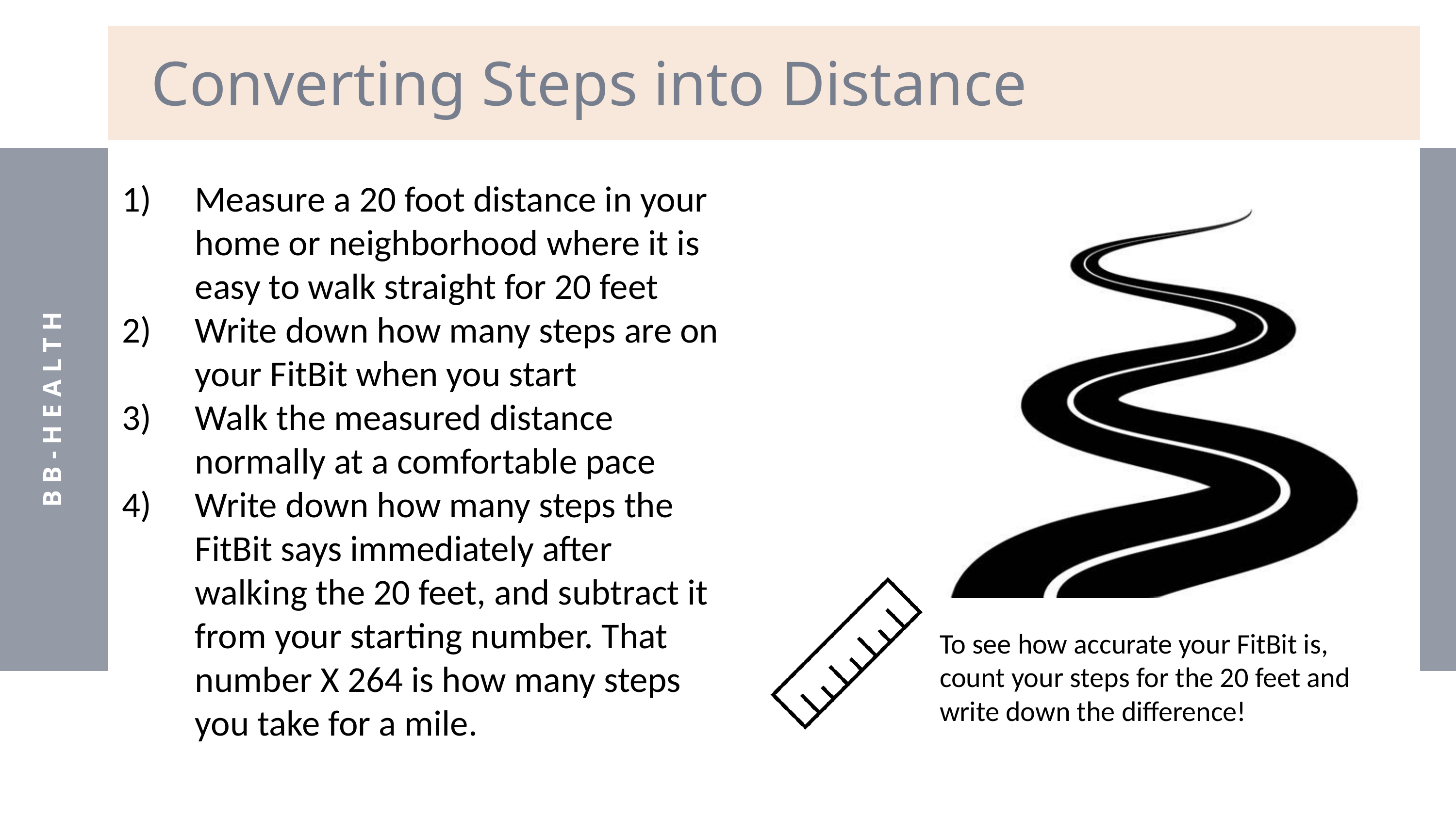

Converting Steps into Distance
Measure a 20 foot distance in your home or neighborhood where it is easy to walk straight for 20 feet
Write down how many steps are on your FitBit when you start
Walk the measured distance normally at a comfortable pace
Write down how many steps the FitBit says immediately after walking the 20 feet, and subtract it from your starting number. That number X 264 is how many steps you take for a mile.
BB-HEALTH
To see how accurate your FitBit is, count your steps for the 20 feet and write down the difference!

## Slide 10
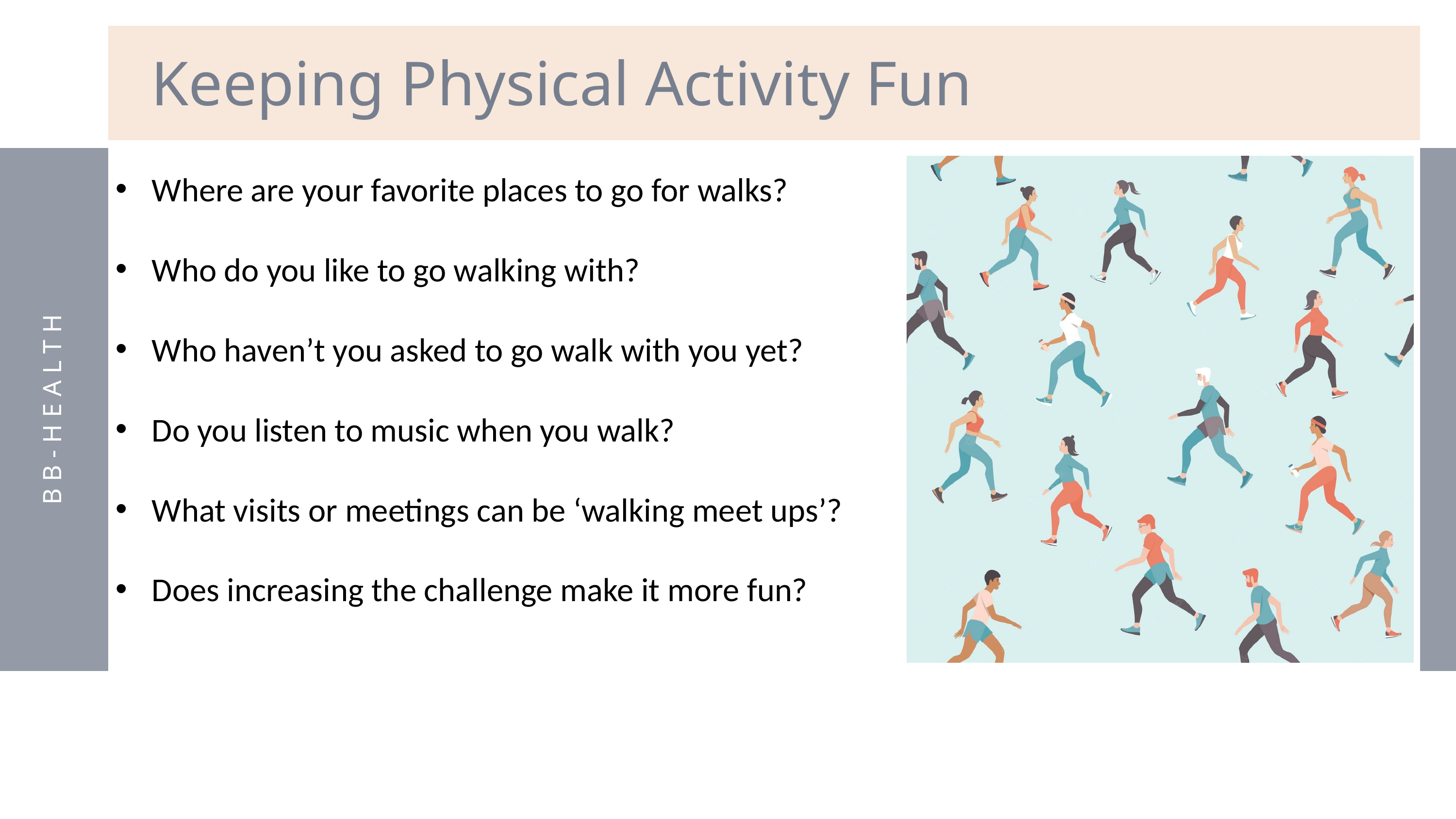

Keeping Physical Activity Fun
Where are your favorite places to go for walks?
Who do you like to go walking with?
Who haven’t you asked to go walk with you yet?
Do you listen to music when you walk?
What visits or meetings can be ‘walking meet ups’?
Does increasing the challenge make it more fun?
BB-HEALTH

## Slide 11
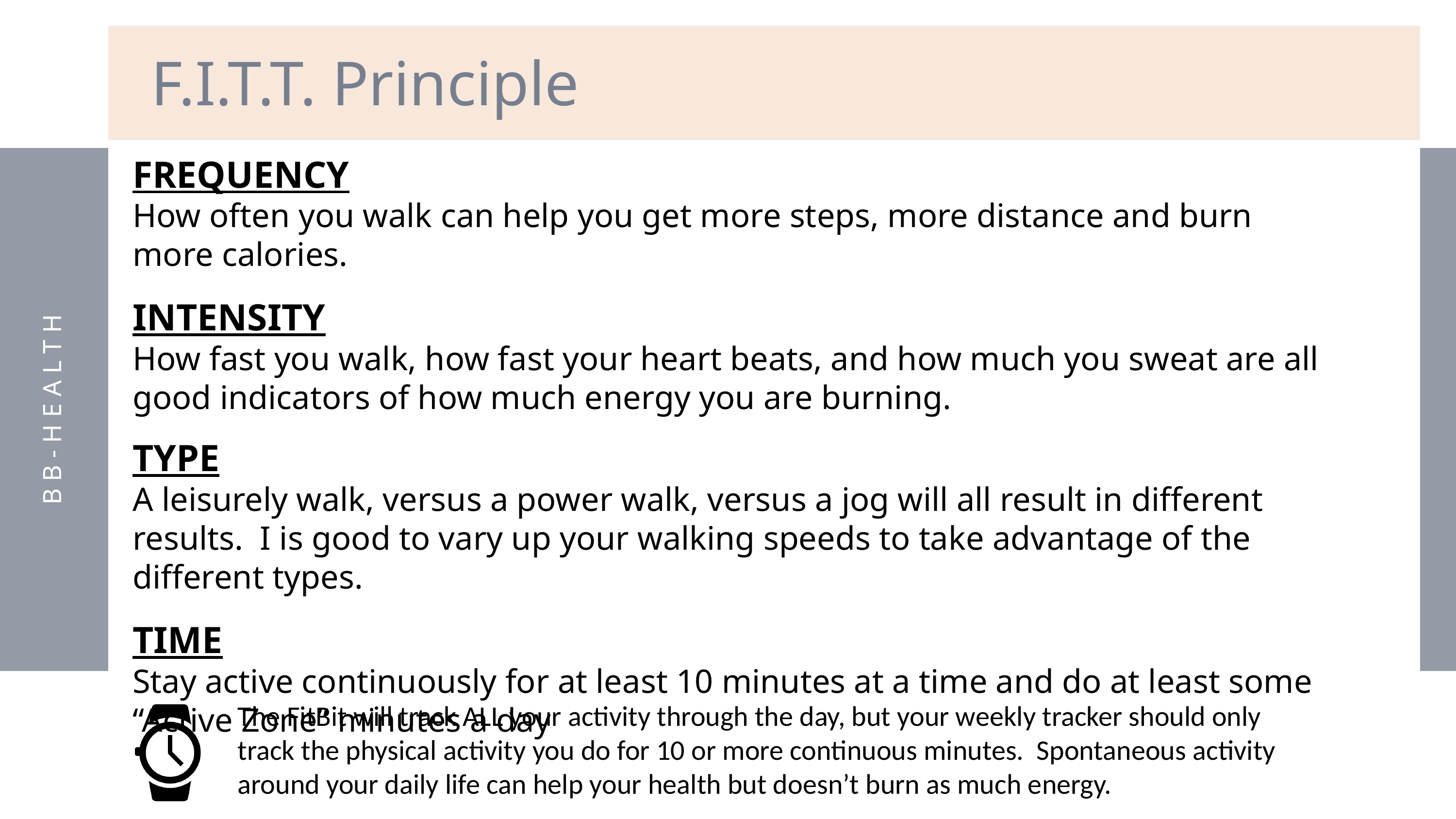

F.I.T.T. Principle
FREQUENCY
How often you walk can help you get more steps, more distance and burn more calories.
INTENSITY
How fast you walk, how fast your heart beats, and how much you sweat are all good indicators of how much energy you are burning.
TYPE
A leisurely walk, versus a power walk, versus a jog will all result in different results. I is good to vary up your walking speeds to take advantage of the different types.
TIME
Stay active continuously for at least 10 minutes at a time and do at least some “Active Zone” minutes a day
BB-HEALTH
The FitBit will track ALL your activity through the day, but your weekly tracker should only track the physical activity you do for 10 or more continuous minutes. Spontaneous activity around your daily life can help your health but doesn’t burn as much energy.

## Slide 12
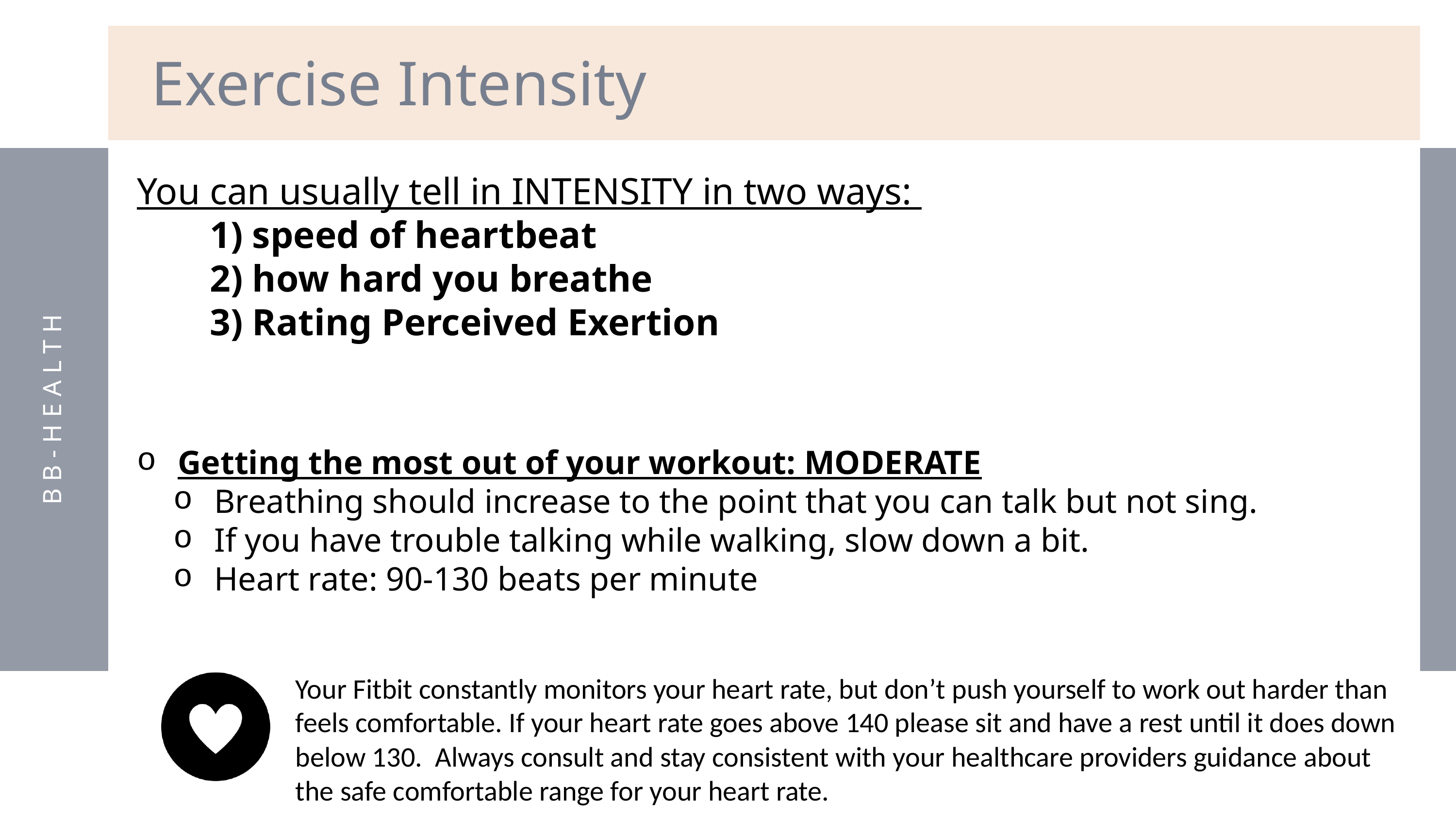

Exercise Intensity
You can usually tell in INTENSITY in two ways:
	1) speed of heartbeat
	2) how hard you breathe
	3) Rating Perceived Exertion
Getting the most out of your workout: MODERATE
Breathing should increase to the point that you can talk but not sing.
If you have trouble talking while walking, slow down a bit.
Heart rate: 90-130 beats per minute
BB-HEALTH
Your Fitbit constantly monitors your heart rate, but don’t push yourself to work out harder than feels comfortable. If your heart rate goes above 140 please sit and have a rest until it does down below 130. Always consult and stay consistent with your healthcare providers guidance about the safe comfortable range for your heart rate.

## Slide 13
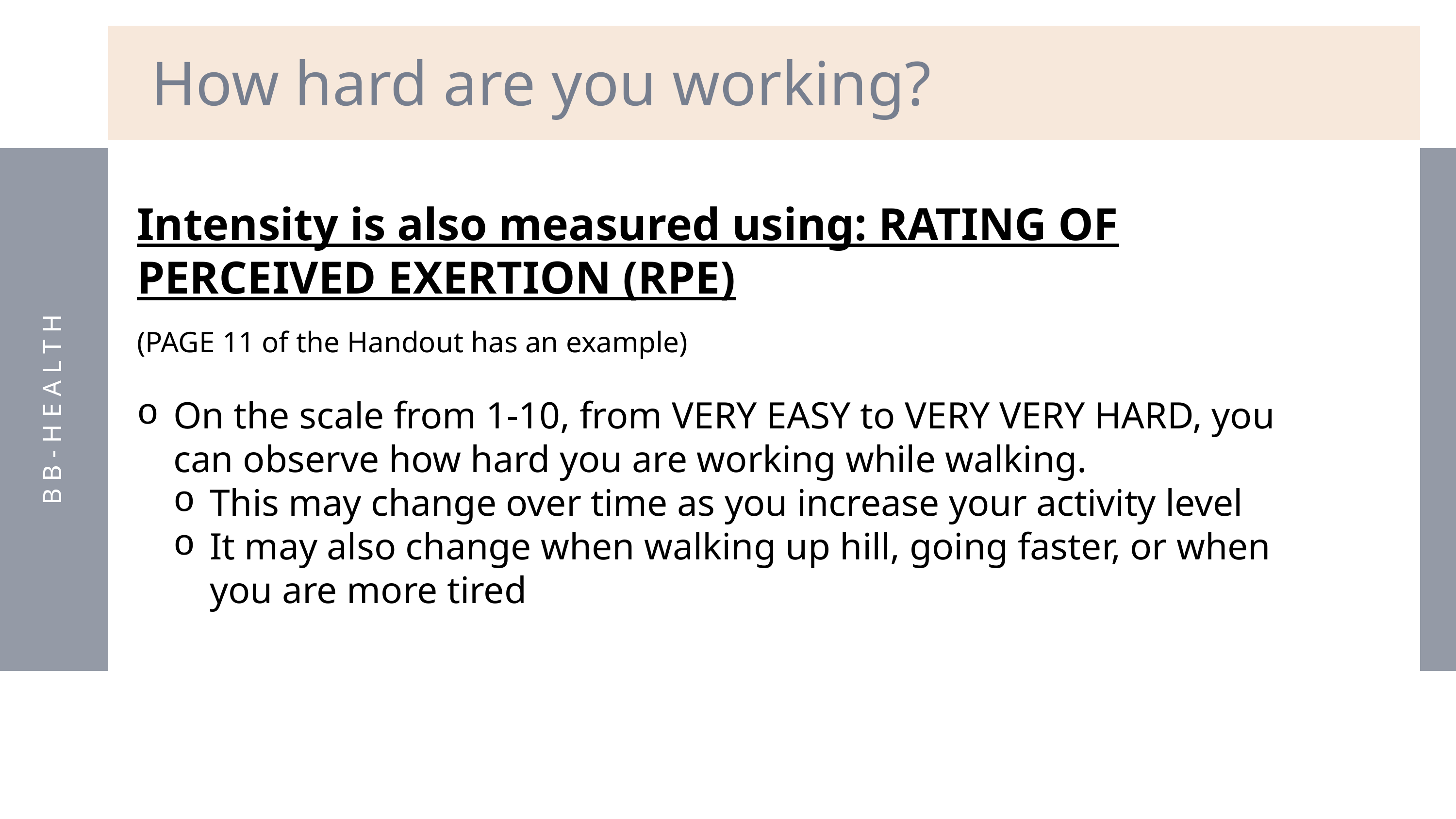

How hard are you working?
Intensity is also measured using: RATING OF PERCEIVED EXERTION (RPE)
(PAGE 11 of the Handout has an example)
On the scale from 1-10, from VERY EASY to VERY VERY HARD, you can observe how hard you are working while walking.
This may change over time as you increase your activity level
It may also change when walking up hill, going faster, or when you are more tired
BB-HEALTH

## Slide 14
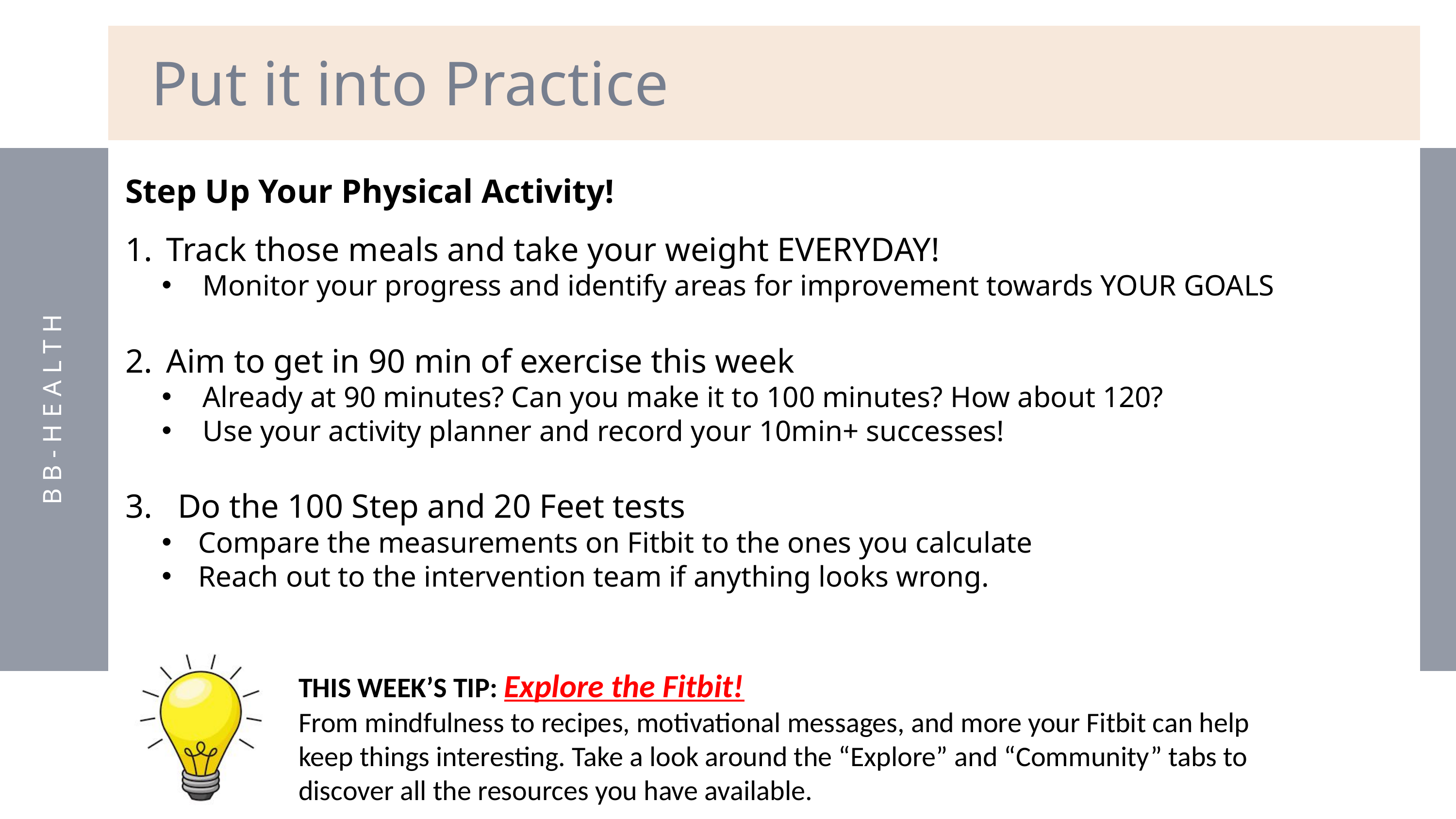

Put it into Practice
Step Up Your Physical Activity!
Track those meals and take your weight EVERYDAY!
Monitor your progress and identify areas for improvement towards YOUR GOALS
Aim to get in 90 min of exercise this week
Already at 90 minutes? Can you make it to 100 minutes? How about 120?
Use your activity planner and record your 10min+ successes!
3. Do the 100 Step and 20 Feet tests
Compare the measurements on Fitbit to the ones you calculate
Reach out to the intervention team if anything looks wrong.
BB-HEALTH
THIS WEEK’S TIP: Explore the Fitbit!
From mindfulness to recipes, motivational messages, and more your Fitbit can help keep things interesting. Take a look around the “Explore” and “Community” tabs to discover all the resources you have available.

## Slide 15
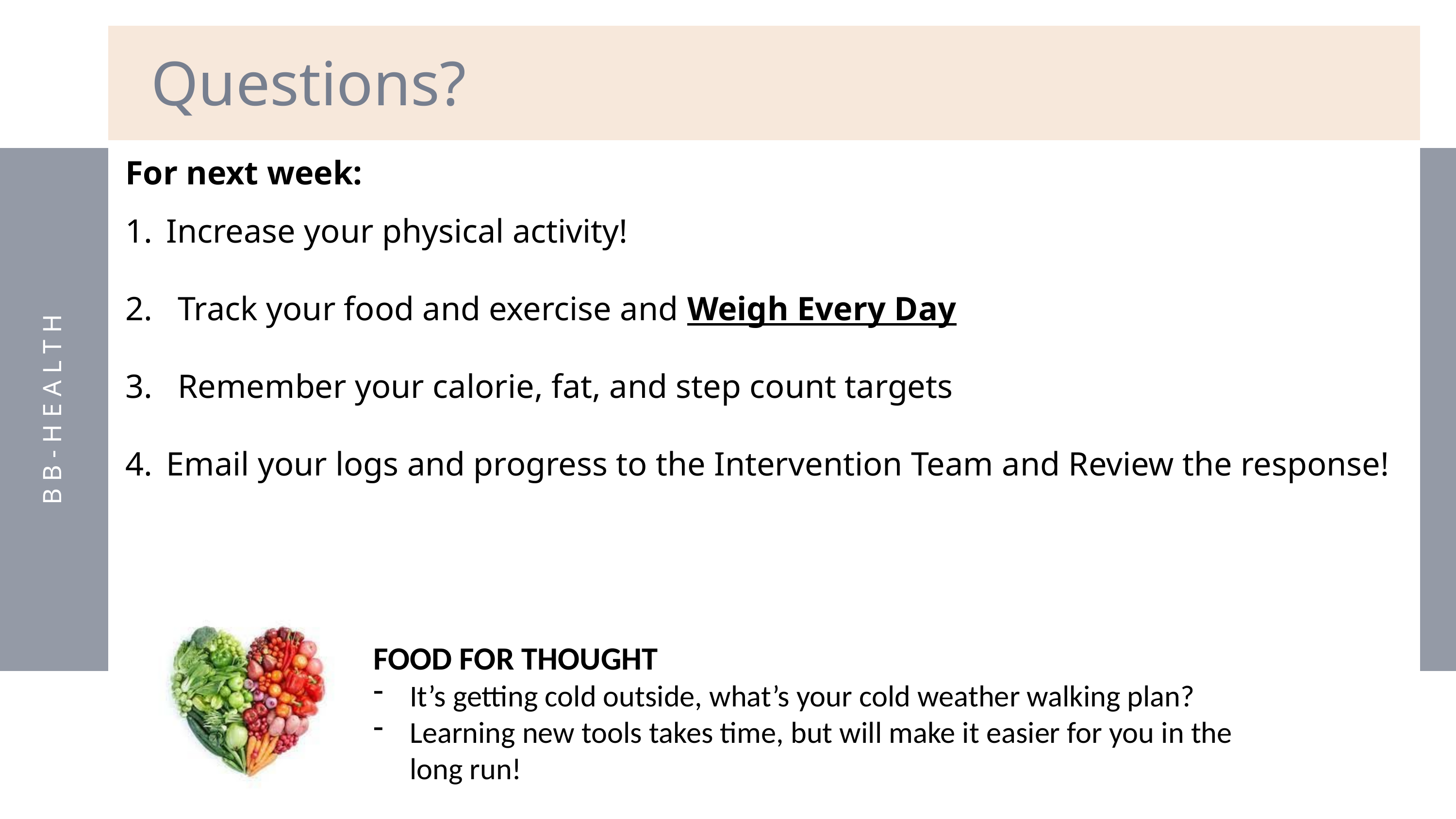

Questions?
For next week:
Increase your physical activity!
2. Track your food and exercise and Weigh Every Day
3. Remember your calorie, fat, and step count targets
Email your logs and progress to the Intervention Team and Review the response!
BB-HEALTH
FOOD FOR THOUGHT
It’s getting cold outside, what’s your cold weather walking plan?
Learning new tools takes time, but will make it easier for you in the long run!
