# Supplementary material for: Development and Feasibility of an eHealth Diabetes Prevention Program Adapted for Older Adults—Results from a Randomized Control Pilot Study
Source: Nutrients. 2024 Mar 23;16(7):930. doi: 10.3390/nu16070930 (PMC11154527; doi:10.3390/nu16070930)
Supplement: Supplementary file 1 [file nutrients-16-00930-s001.zip › Week9.pptx]

## Slide 1
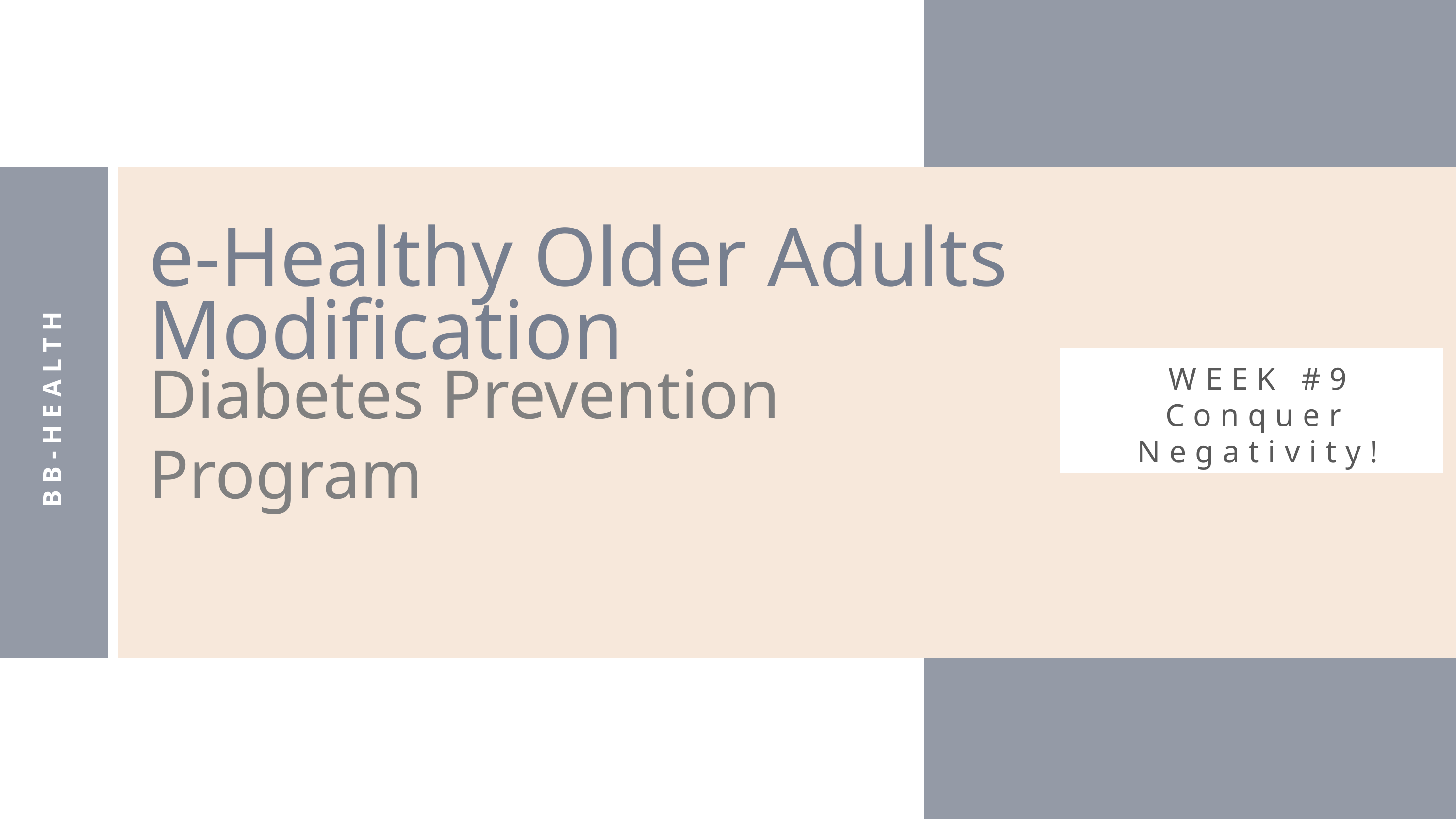

OPEN REPORTS
e-Healthy Older Adults Modification
WEEK #9
Conquer Negativity!
Diabetes Prevention Program
BB-HEALTH

## Slide 2
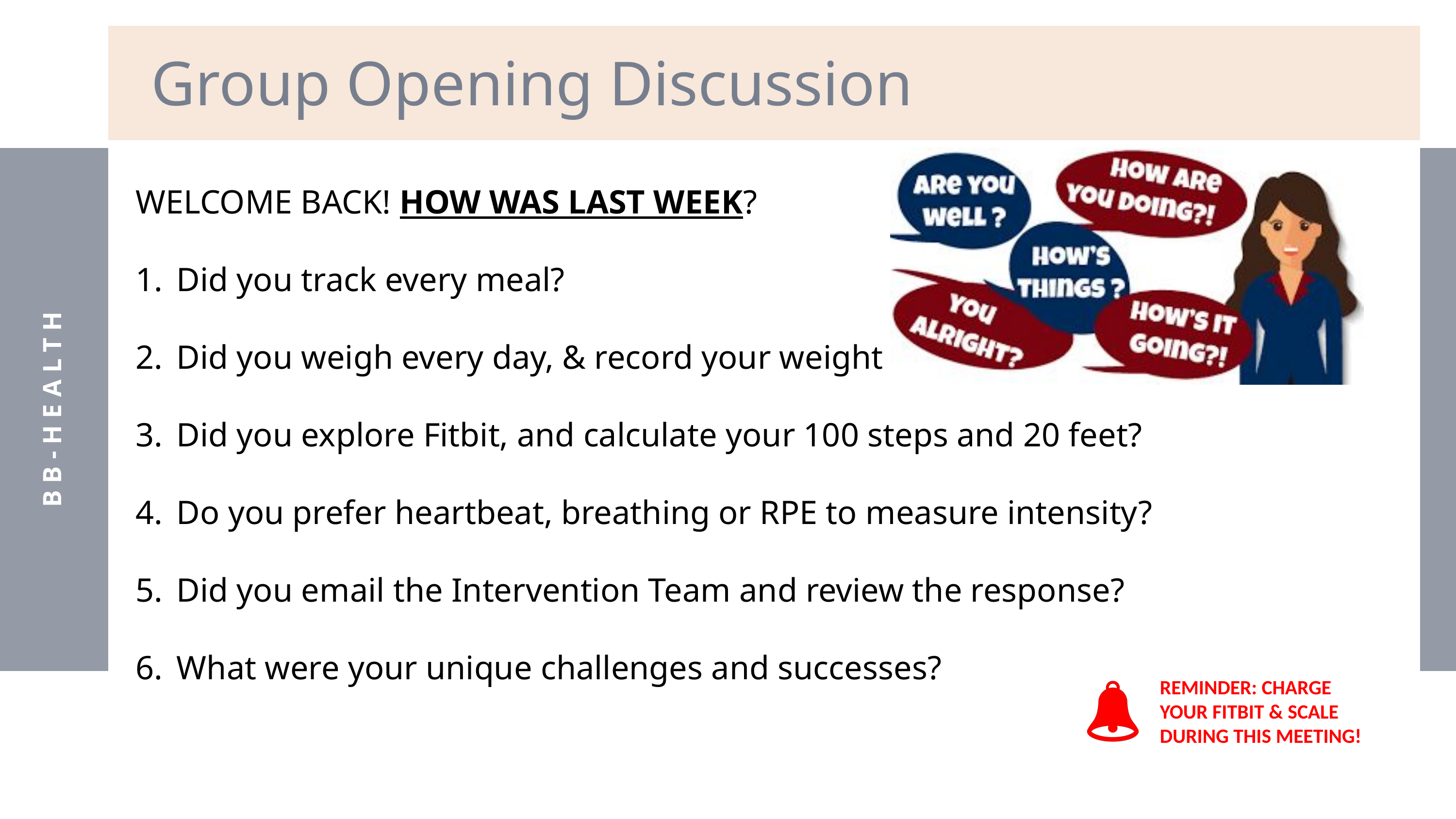

Group Opening Discussion
WELCOME BACK! HOW WAS LAST WEEK?
Did you track every meal?
Did you weigh every day, & record your weight today?
Did you explore Fitbit, and calculate your 100 steps and 20 feet?
Do you prefer heartbeat, breathing or RPE to measure intensity?
Did you email the Intervention Team and review the response?
What were your unique challenges and successes?
BB-HEALTH
REMINDER: CHARGE YOUR FITBIT & SCALE DURING THIS MEETING!

## Slide 3
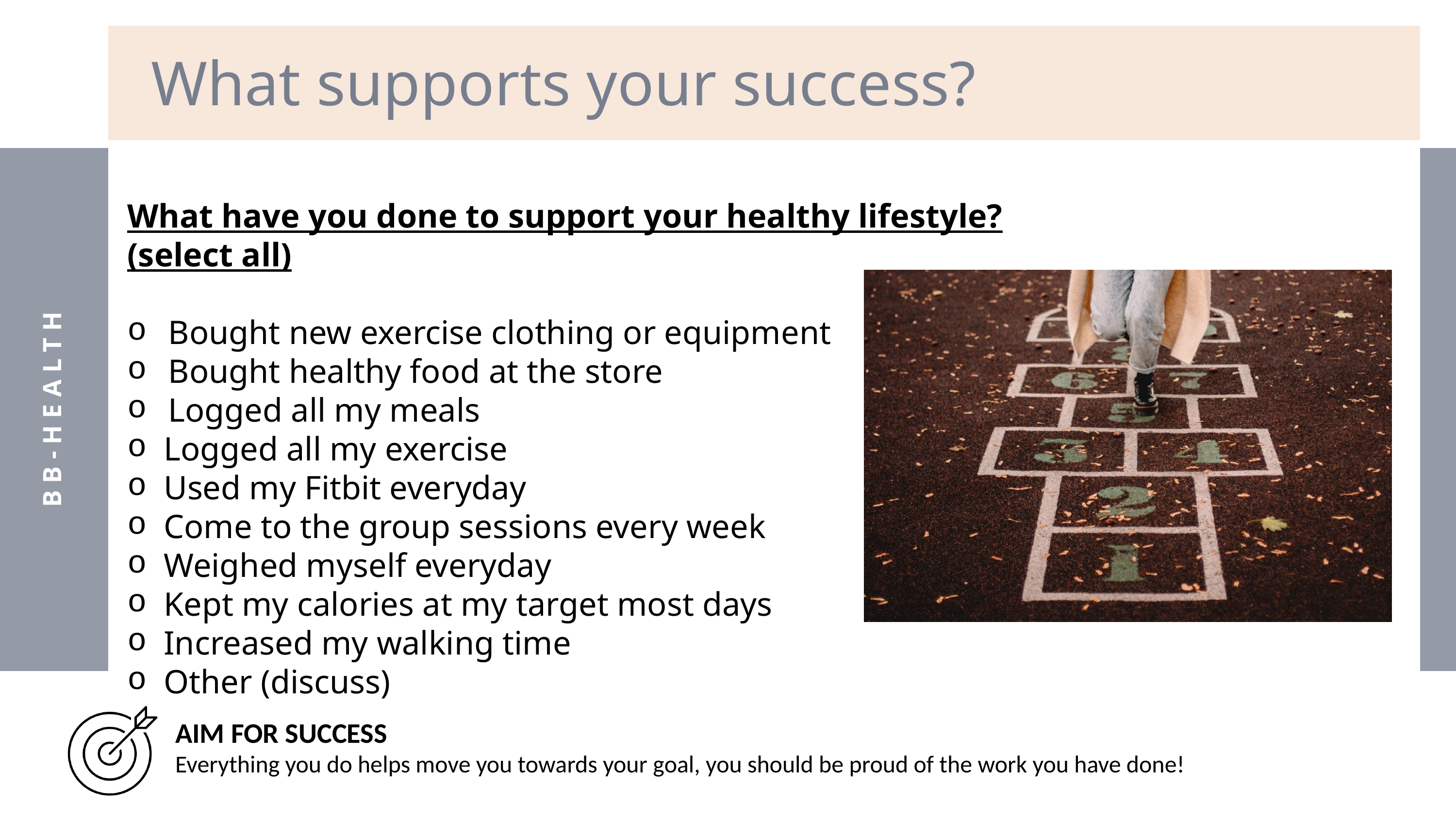

What supports your success?
What have you done to support your healthy lifestyle? (select all)
Bought new exercise clothing or equipment
Bought healthy food at the store
Logged all my meals
Logged all my exercise
Used my Fitbit everyday
Come to the group sessions every week
Weighed myself everyday
Kept my calories at my target most days
Increased my walking time
Other (discuss)
BB-HEALTH
AIM FOR SUCCESS
Everything you do helps move you towards your goal, you should be proud of the work you have done!

## Slide 4
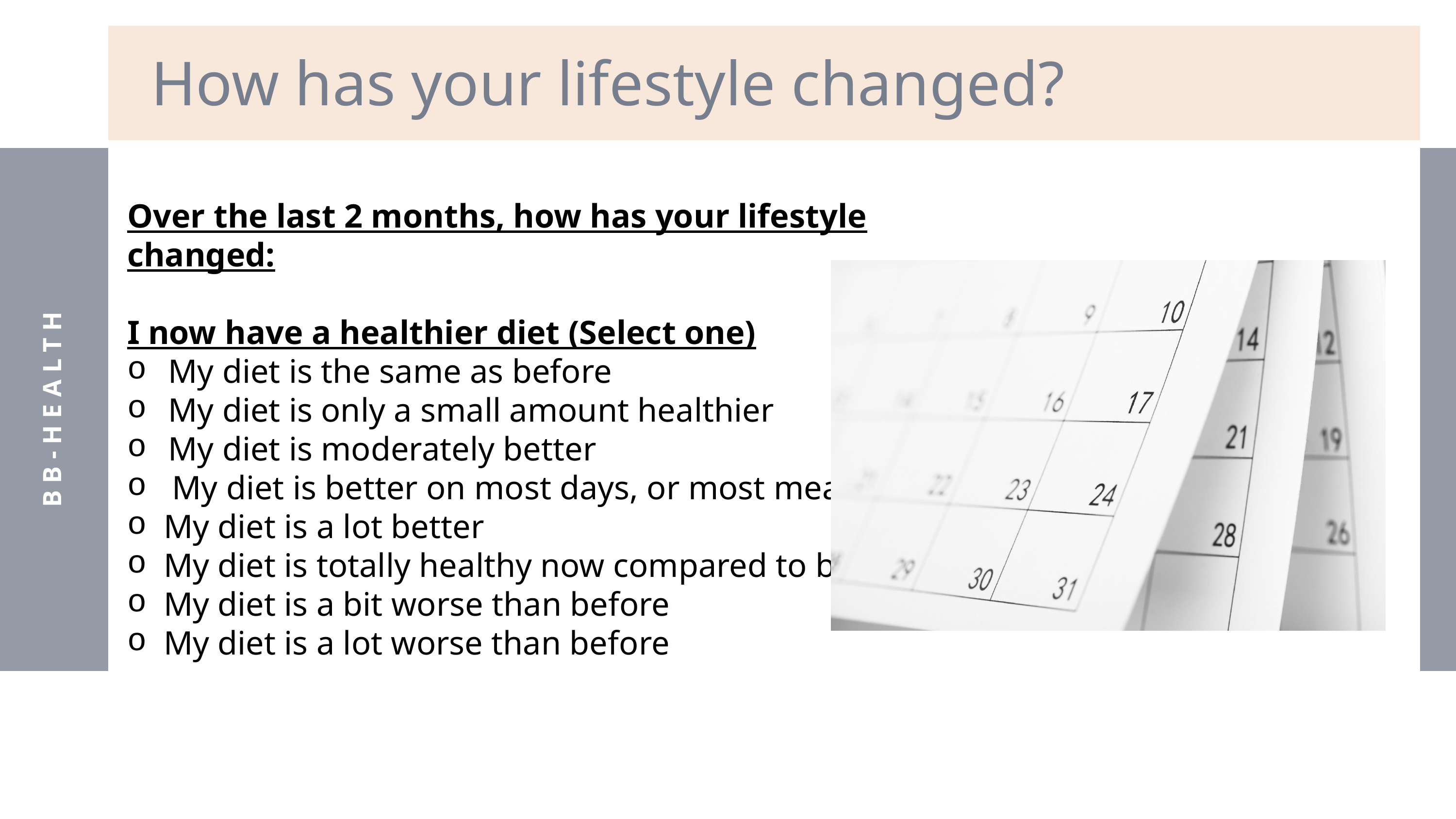

How has your lifestyle changed?
Over the last 2 months, how has your lifestyle changed:
I now have a healthier diet (Select one)
My diet is the same as before
My diet is only a small amount healthier
My diet is moderately better
 My diet is better on most days, or most meals
My diet is a lot better
My diet is totally healthy now compared to before
My diet is a bit worse than before
My diet is a lot worse than before
BB-HEALTH

## Slide 5
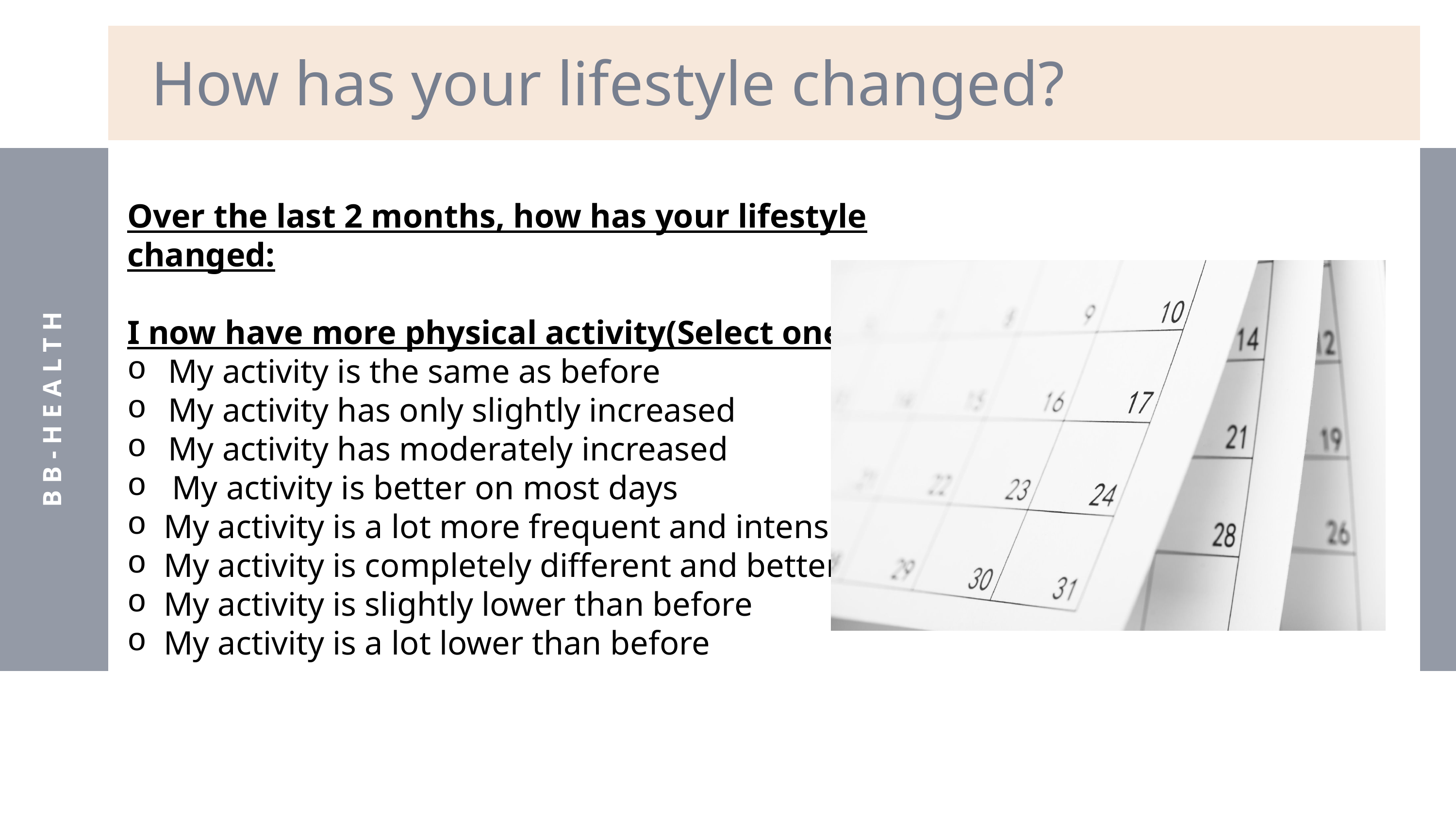

How has your lifestyle changed?
Over the last 2 months, how has your lifestyle changed:
I now have more physical activity(Select one)
My activity is the same as before
My activity has only slightly increased
My activity has moderately increased
 My activity is better on most days
My activity is a lot more frequent and intensive
My activity is completely different and better
My activity is slightly lower than before
My activity is a lot lower than before
BB-HEALTH

## Slide 6
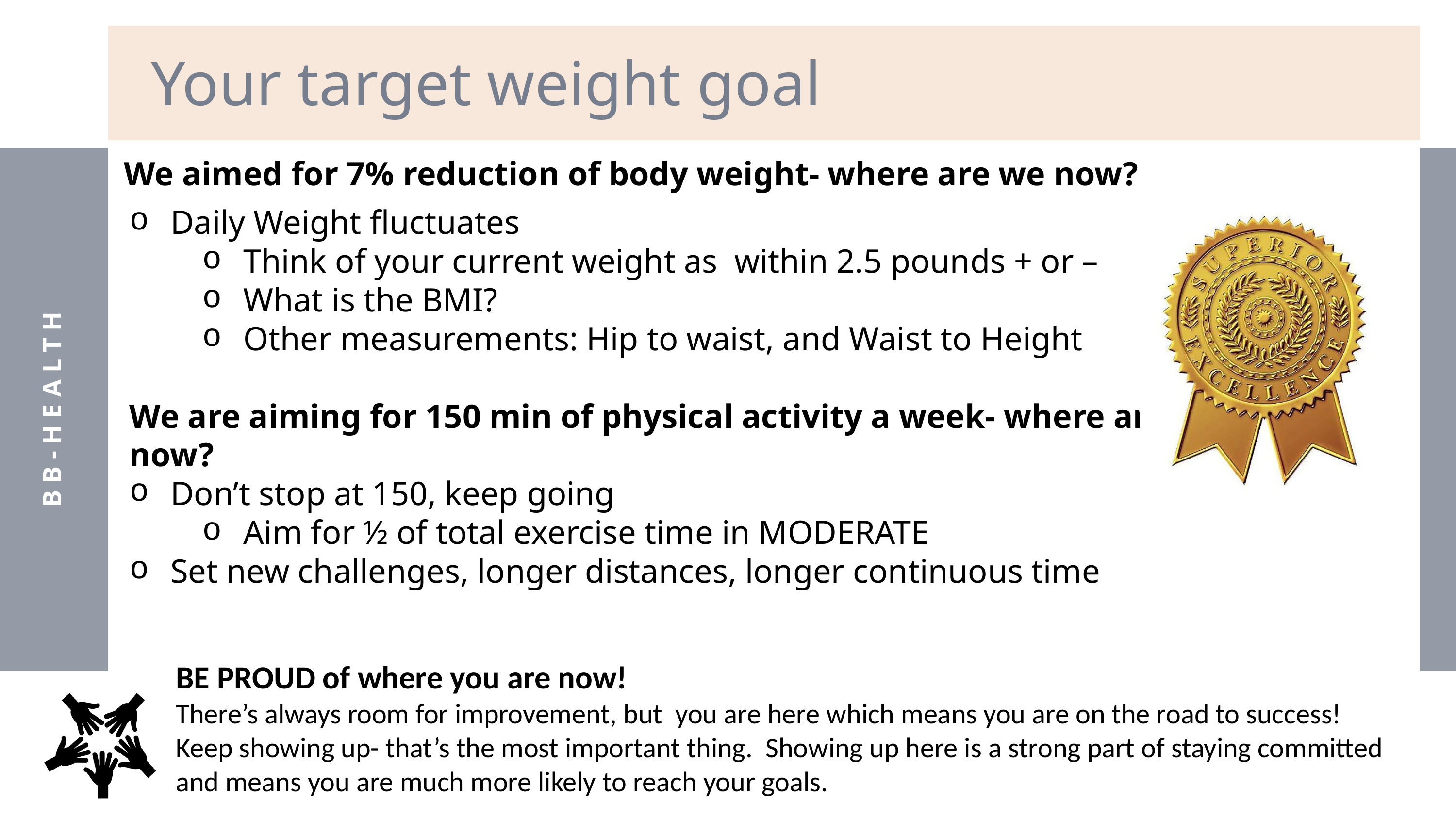

Your target weight goal
We aimed for 7% reduction of body weight- where are we now?
Daily Weight fluctuates
Think of your current weight as within 2.5 pounds + or –
What is the BMI?
Other measurements: Hip to waist, and Waist to Height
We are aiming for 150 min of physical activity a week- where are we now?
Don’t stop at 150, keep going
Aim for ½ of total exercise time in MODERATE
Set new challenges, longer distances, longer continuous time
BB-HEALTH
BE PROUD of where you are now!
There’s always room for improvement, but you are here which means you are on the road to success! Keep showing up- that’s the most important thing. Showing up here is a strong part of staying committed and means you are much more likely to reach your goals.

## Slide 7
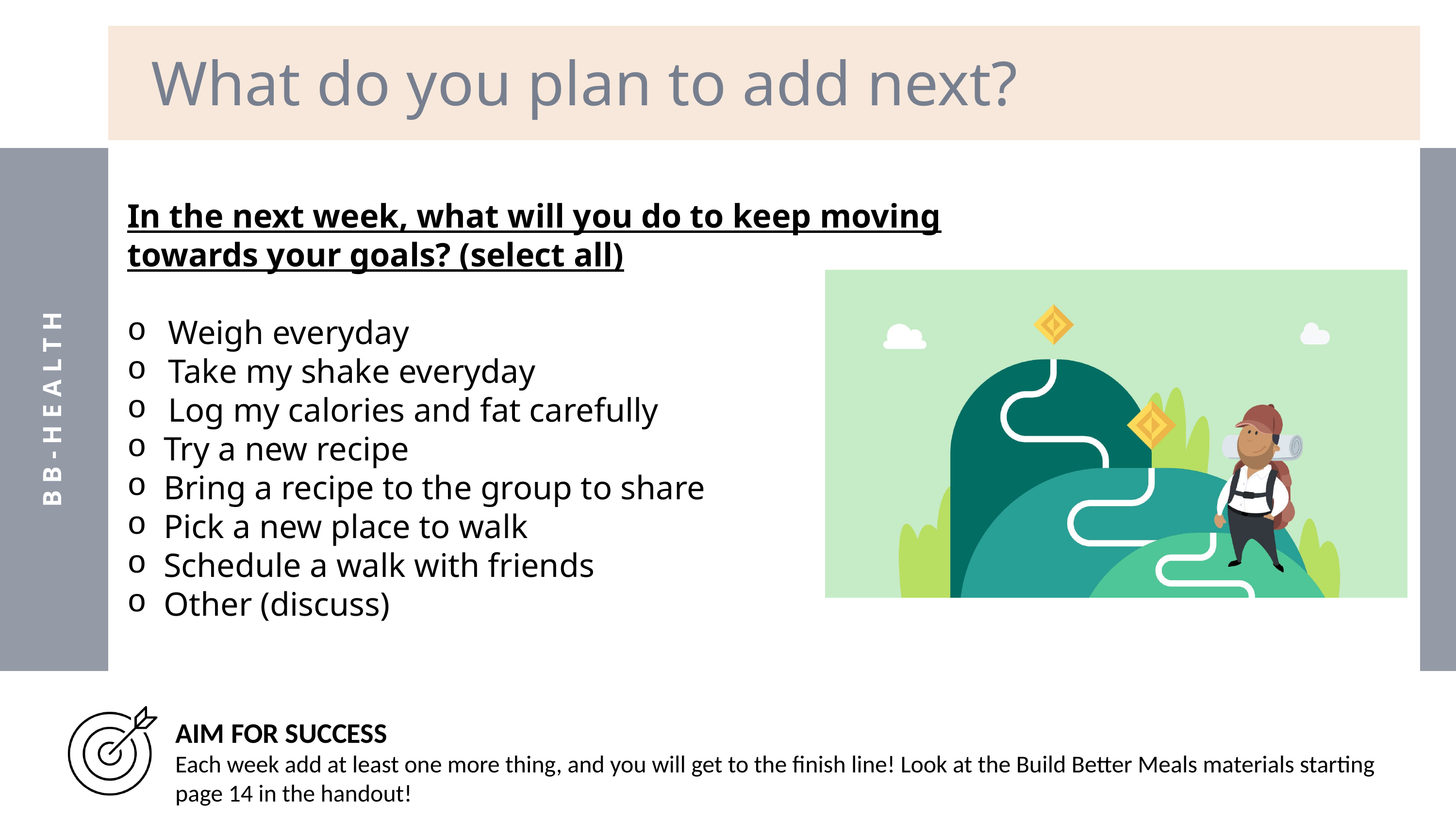

What do you plan to add next?
In the next week, what will you do to keep moving towards your goals? (select all)
Weigh everyday
Take my shake everyday
Log my calories and fat carefully
Try a new recipe
Bring a recipe to the group to share
Pick a new place to walk
Schedule a walk with friends
Other (discuss)
BB-HEALTH
AIM FOR SUCCESS
Each week add at least one more thing, and you will get to the finish line! Look at the Build Better Meals materials starting page 14 in the handout!

## Slide 8
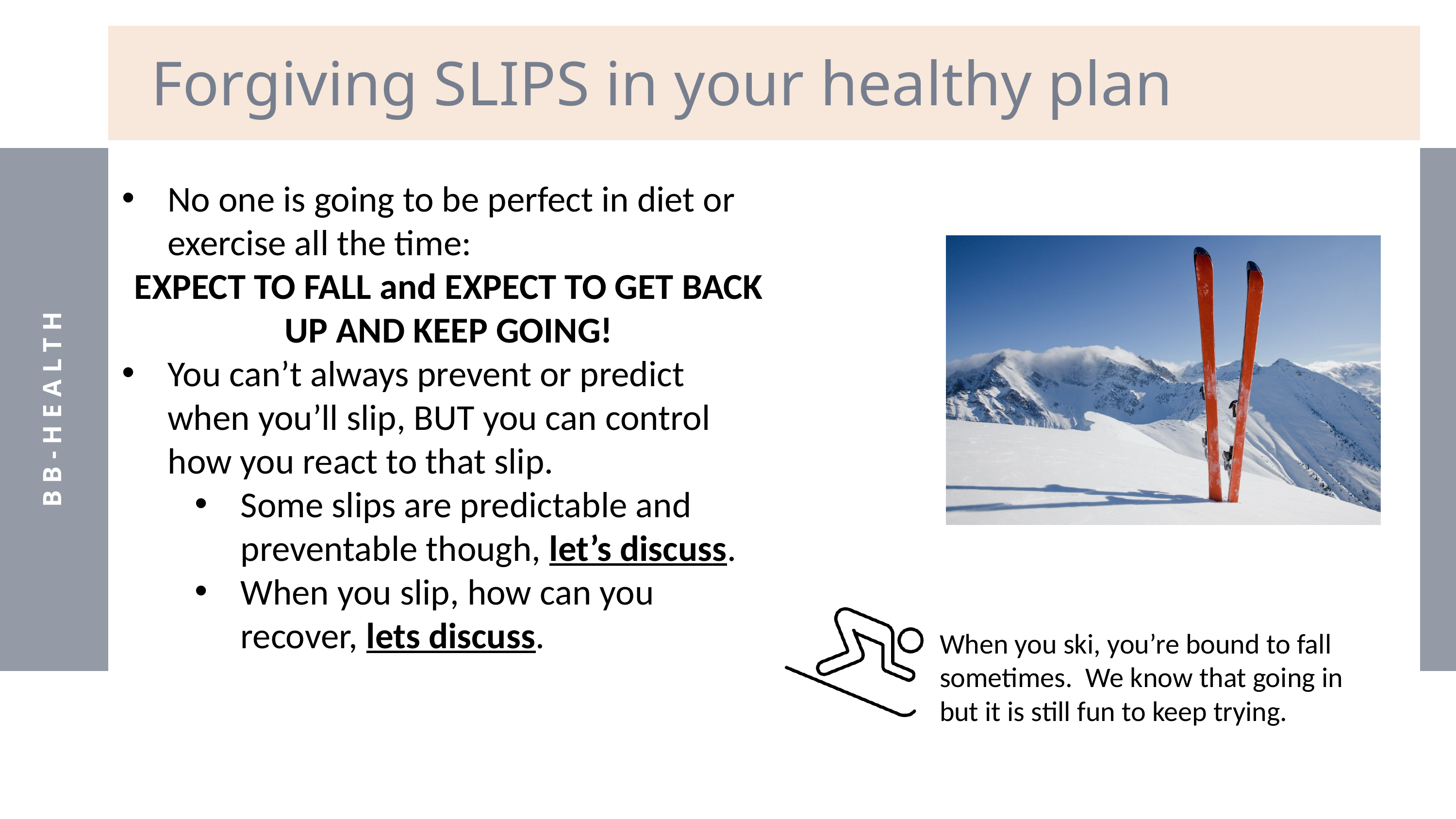

Forgiving SLIPS in your healthy plan
No one is going to be perfect in diet or exercise all the time:
EXPECT TO FALL and EXPECT TO GET BACK UP AND KEEP GOING!
You can’t always prevent or predict when you’ll slip, BUT you can control how you react to that slip.
Some slips are predictable and preventable though, let’s discuss.
When you slip, how can you recover, lets discuss.
BB-HEALTH
When you ski, you’re bound to fall sometimes. We know that going in but it is still fun to keep trying.

## Slide 9
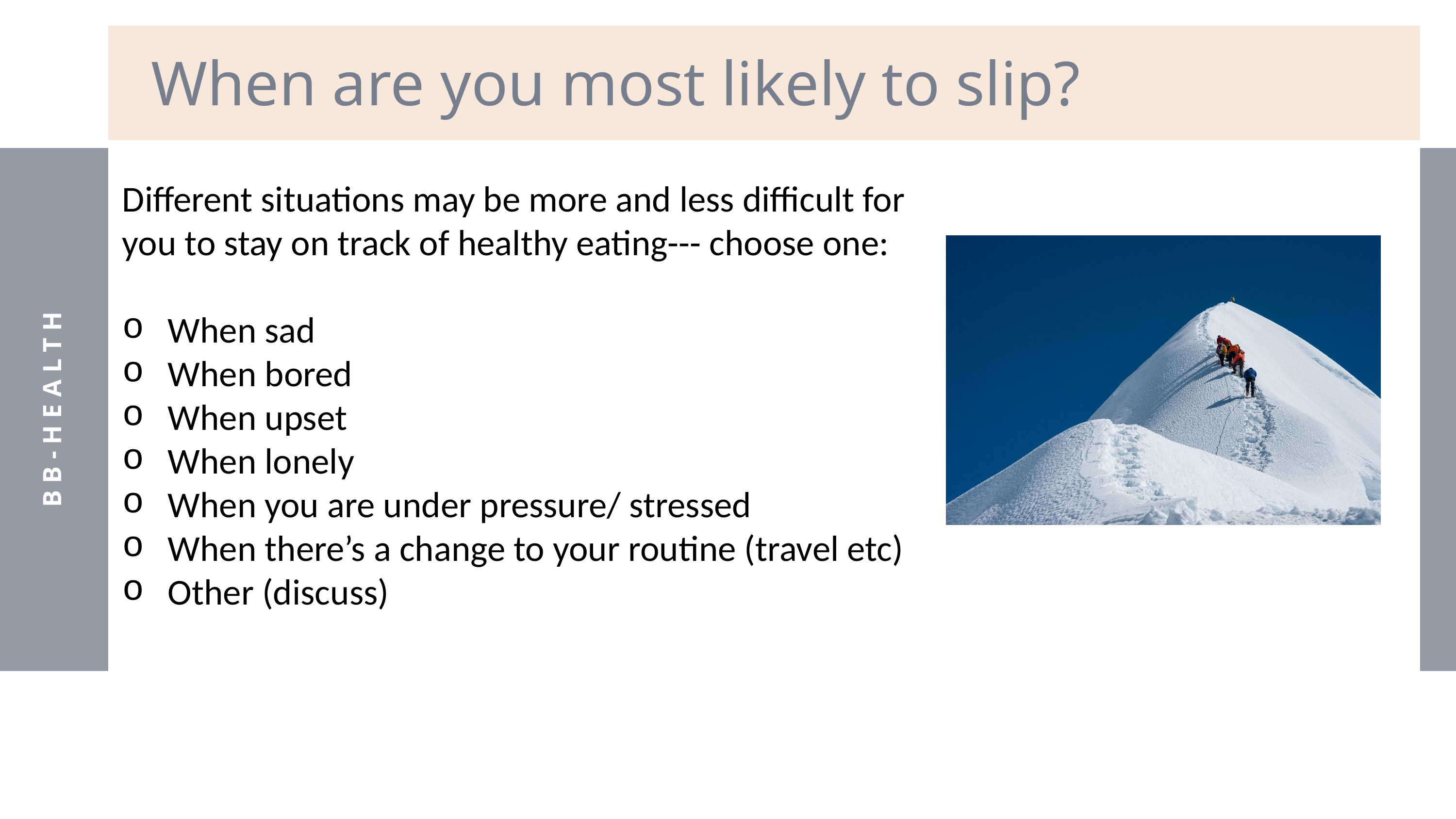

When are you most likely to slip?
Different situations may be more and less difficult for you to stay on track of healthy eating--- choose one:
When sad
When bored
When upset
When lonely
When you are under pressure/ stressed
When there’s a change to your routine (travel etc)
Other (discuss)
BB-HEALTH

## Slide 10
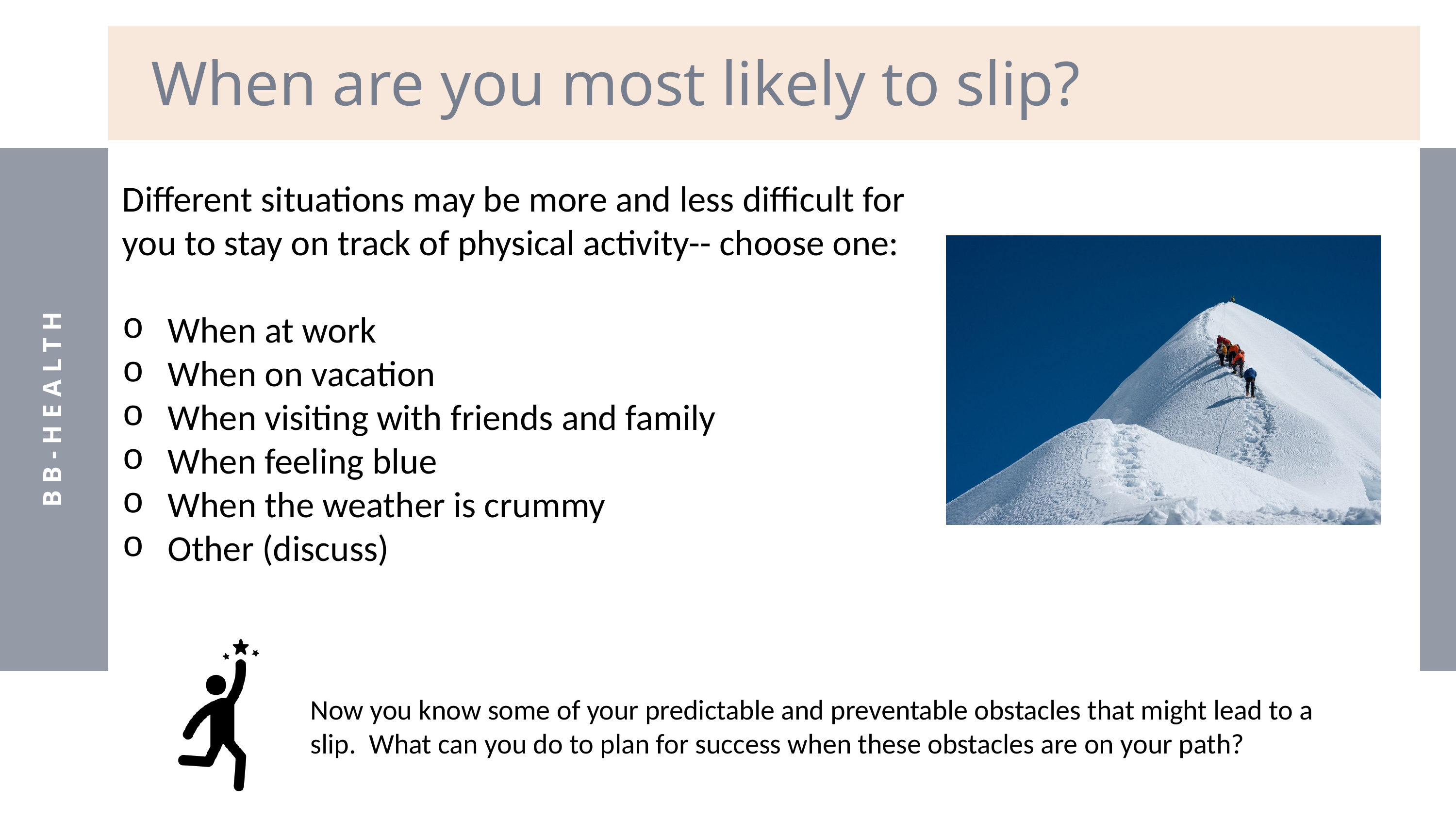

When are you most likely to slip?
Different situations may be more and less difficult for you to stay on track of physical activity-- choose one:
When at work
When on vacation
When visiting with friends and family
When feeling blue
When the weather is crummy
Other (discuss)
BB-HEALTH
Now you know some of your predictable and preventable obstacles that might lead to a slip. What can you do to plan for success when these obstacles are on your path?

## Slide 11
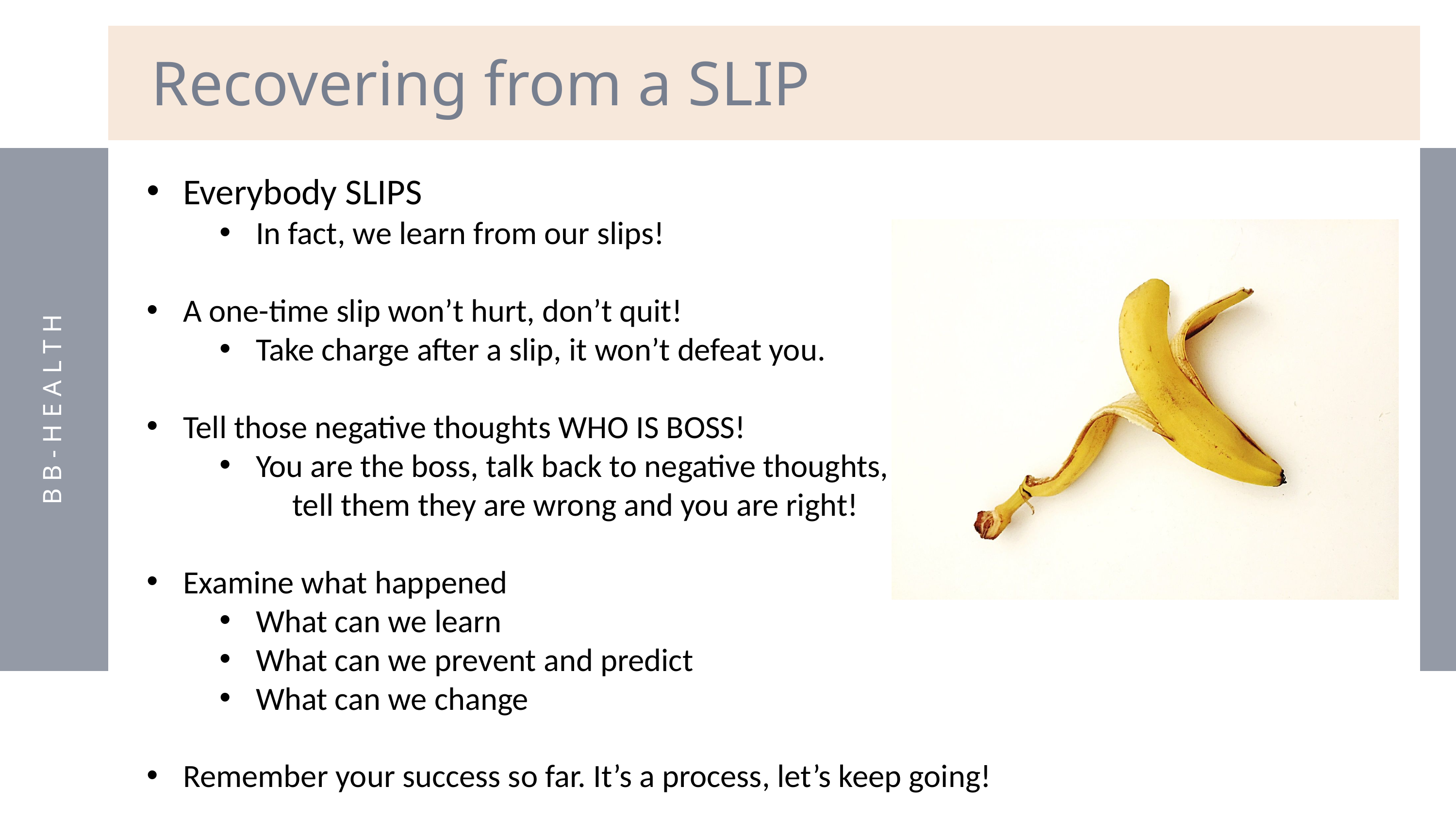

Recovering from a SLIP
Everybody SLIPS
In fact, we learn from our slips!
A one-time slip won’t hurt, don’t quit!
Take charge after a slip, it won’t defeat you.
Tell those negative thoughts WHO IS BOSS!
You are the boss, talk back to negative thoughts,
	tell them they are wrong and you are right!
Examine what happened
What can we learn
What can we prevent and predict
What can we change
Remember your success so far. It’s a process, let’s keep going!
BB-HEALTH

## Slide 12
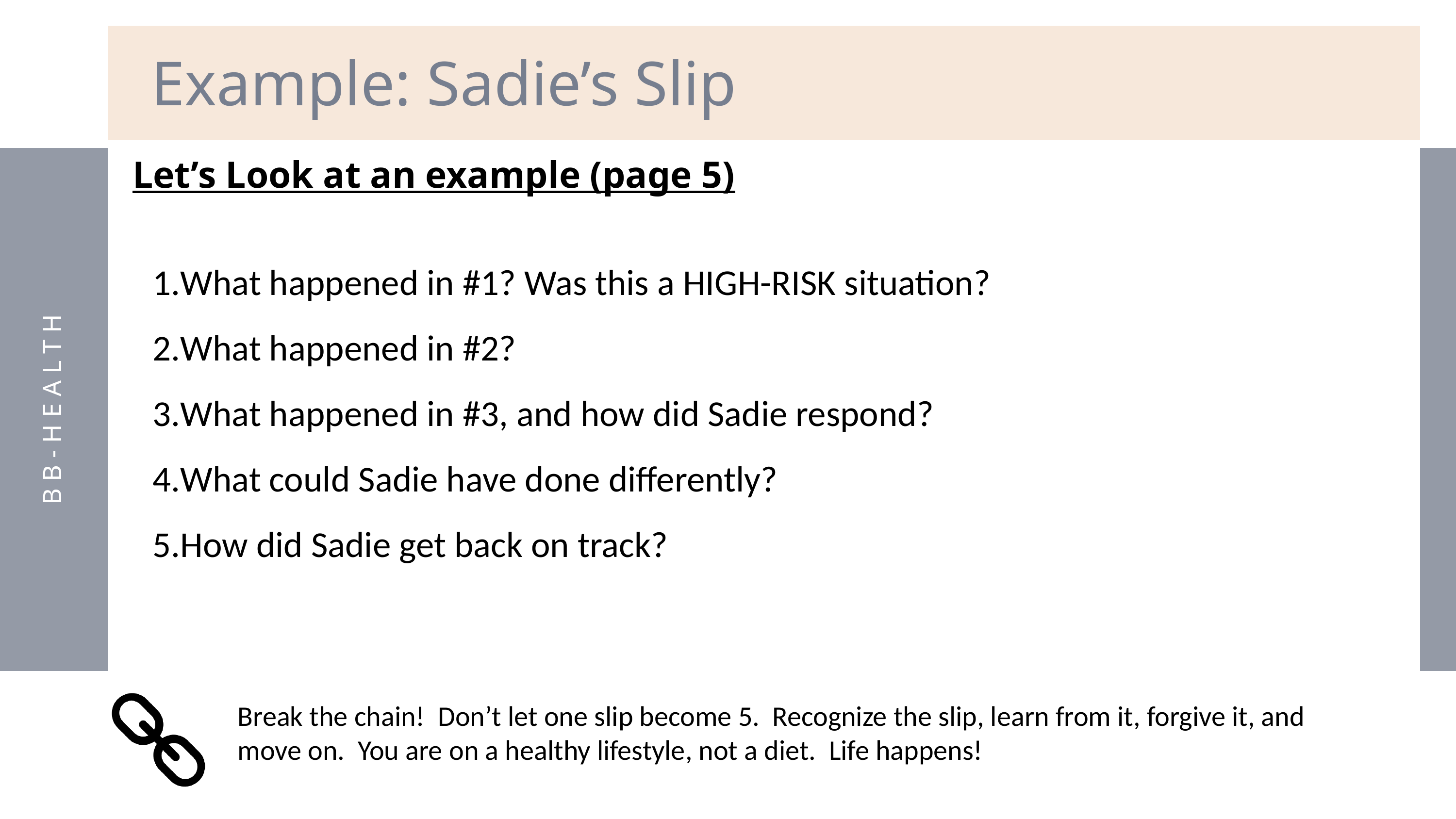

Example: Sadie’s Slip
Let’s Look at an example (page 5)
What happened in #1? Was this a HIGH-RISK situation?
What happened in #2?
What happened in #3, and how did Sadie respond?
What could Sadie have done differently?
How did Sadie get back on track?
BB-HEALTH
Break the chain! Don’t let one slip become 5. Recognize the slip, learn from it, forgive it, and move on. You are on a healthy lifestyle, not a diet. Life happens!

## Slide 13
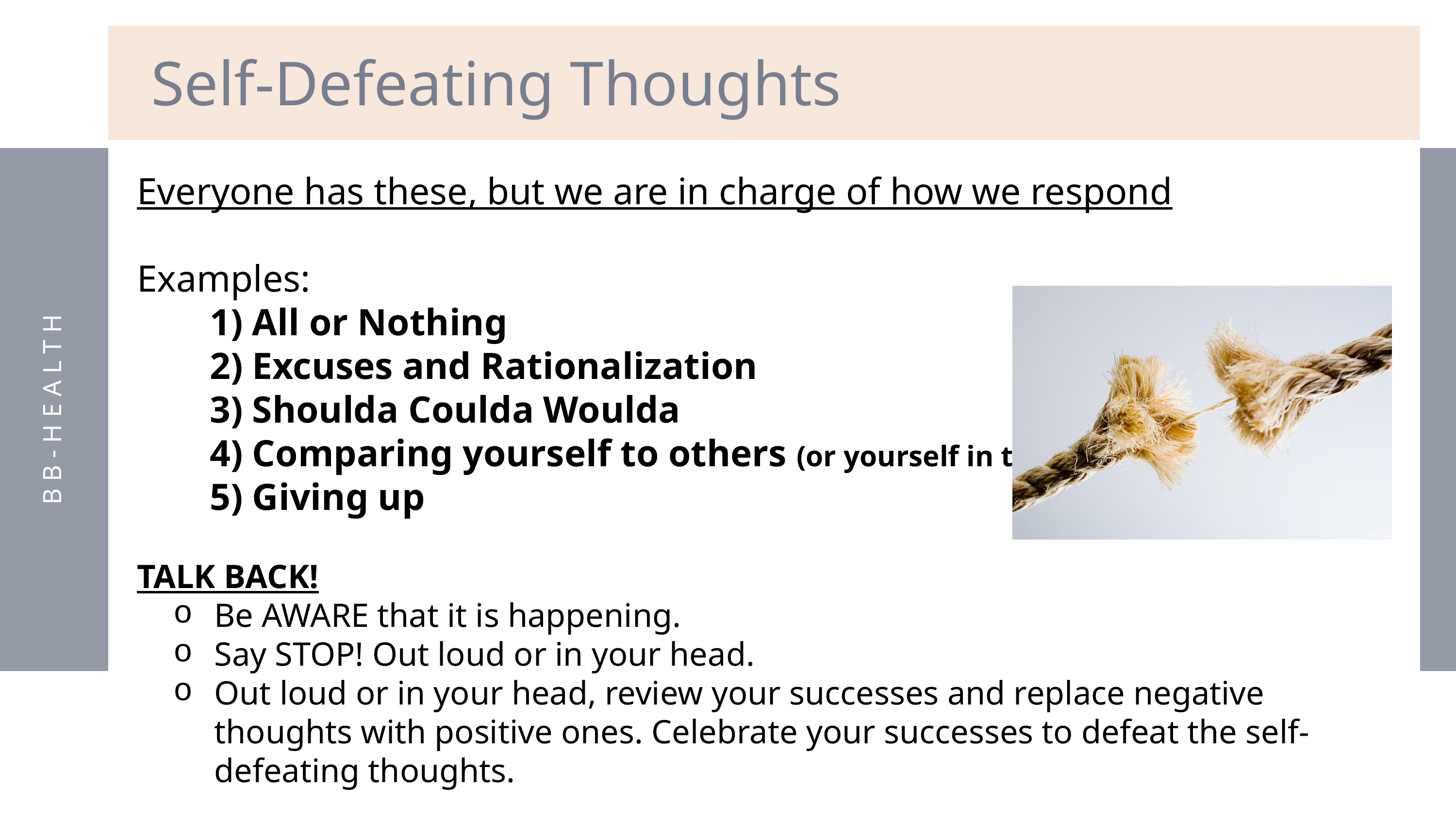

Self-Defeating Thoughts
Everyone has these, but we are in charge of how we respond
Examples:
	1) All or Nothing
	2) Excuses and Rationalization
	3) Shoulda Coulda Woulda
	4) Comparing yourself to others (or yourself in the past!)
	5) Giving up
TALK BACK!
Be AWARE that it is happening.
Say STOP! Out loud or in your head.
Out loud or in your head, review your successes and replace negative thoughts with positive ones. Celebrate your successes to defeat the self-defeating thoughts.
BB-HEALTH

## Slide 14
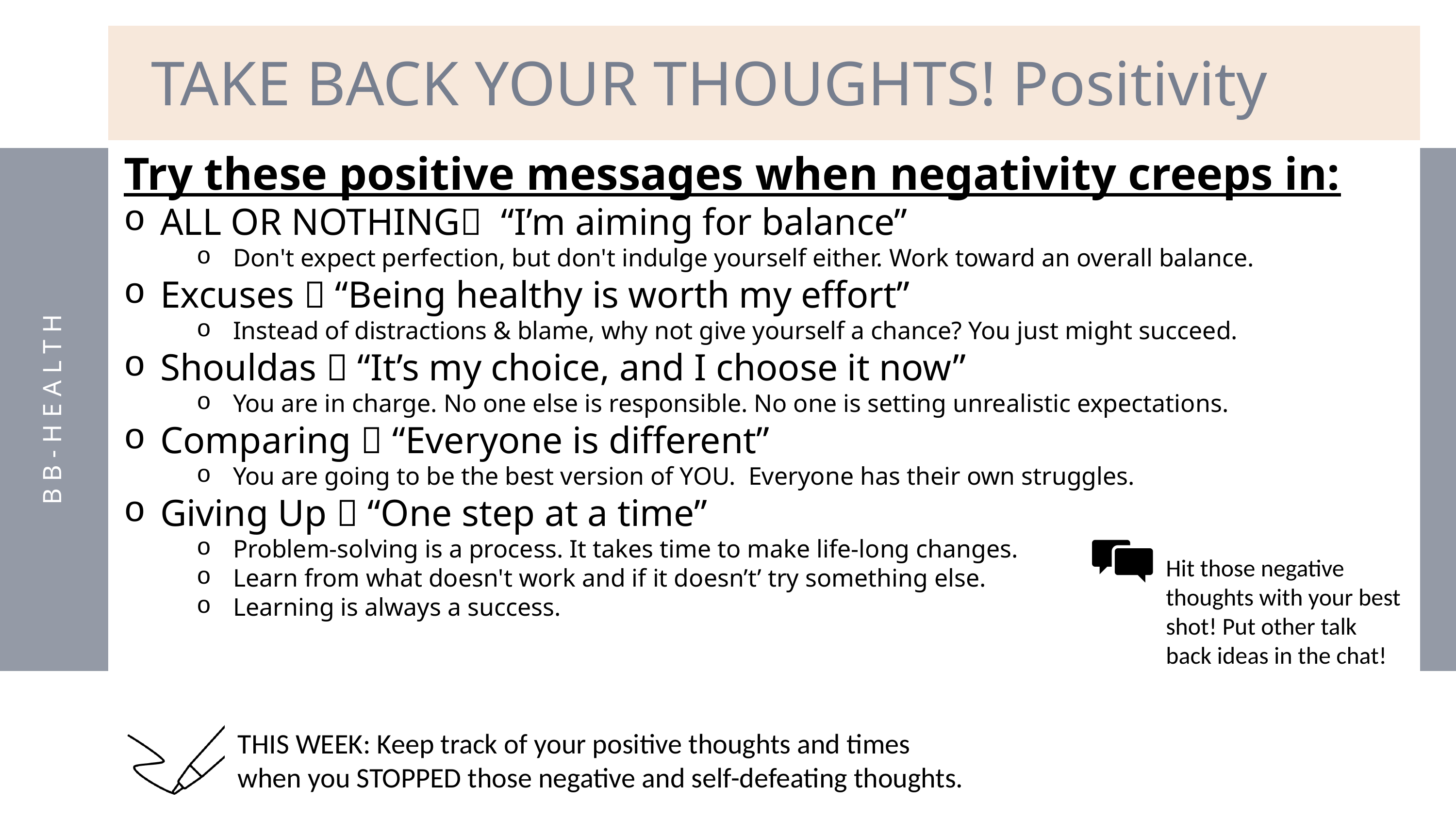

TAKE BACK YOUR THOUGHTS! Positivity
Try these positive messages when negativity creeps in:
ALL OR NOTHING “I’m aiming for balance”
Don't expect perfection, but don't indulge yourself either. Work toward an overall balance.
Excuses  “Being healthy is worth my effort”
Instead of distractions & blame, why not give yourself a chance? You just might succeed.
Shouldas  “It’s my choice, and I choose it now”
You are in charge. No one else is responsible. No one is setting unrealistic expectations.
Comparing  “Everyone is different”
You are going to be the best version of YOU. Everyone has their own struggles.
Giving Up  “One step at a time”
Problem-solving is a process. It takes time to make life-long changes.
Learn from what doesn't work and if it doesn’t’ try something else.
Learning is always a success.
BB-HEALTH
Hit those negative thoughts with your best shot! Put other talk back ideas in the chat!
THIS WEEK: Keep track of your positive thoughts and times when you STOPPED those negative and self-defeating thoughts.

## Slide 15
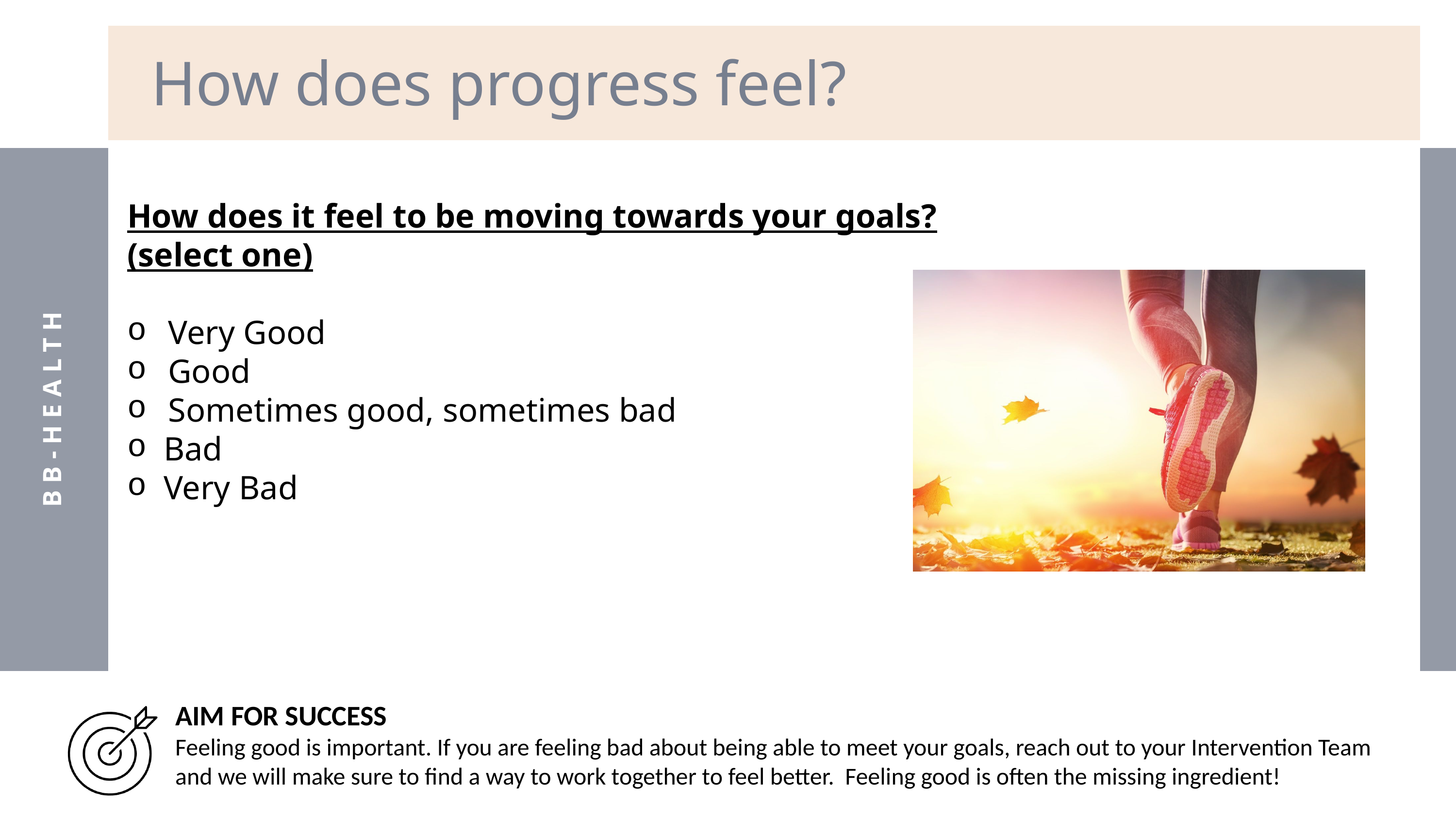

How does progress feel?
How does it feel to be moving towards your goals? (select one)
Very Good
Good
Sometimes good, sometimes bad
Bad
Very Bad
BB-HEALTH
AIM FOR SUCCESS
Feeling good is important. If you are feeling bad about being able to meet your goals, reach out to your Intervention Team and we will make sure to find a way to work together to feel better. Feeling good is often the missing ingredient!

## Slide 16
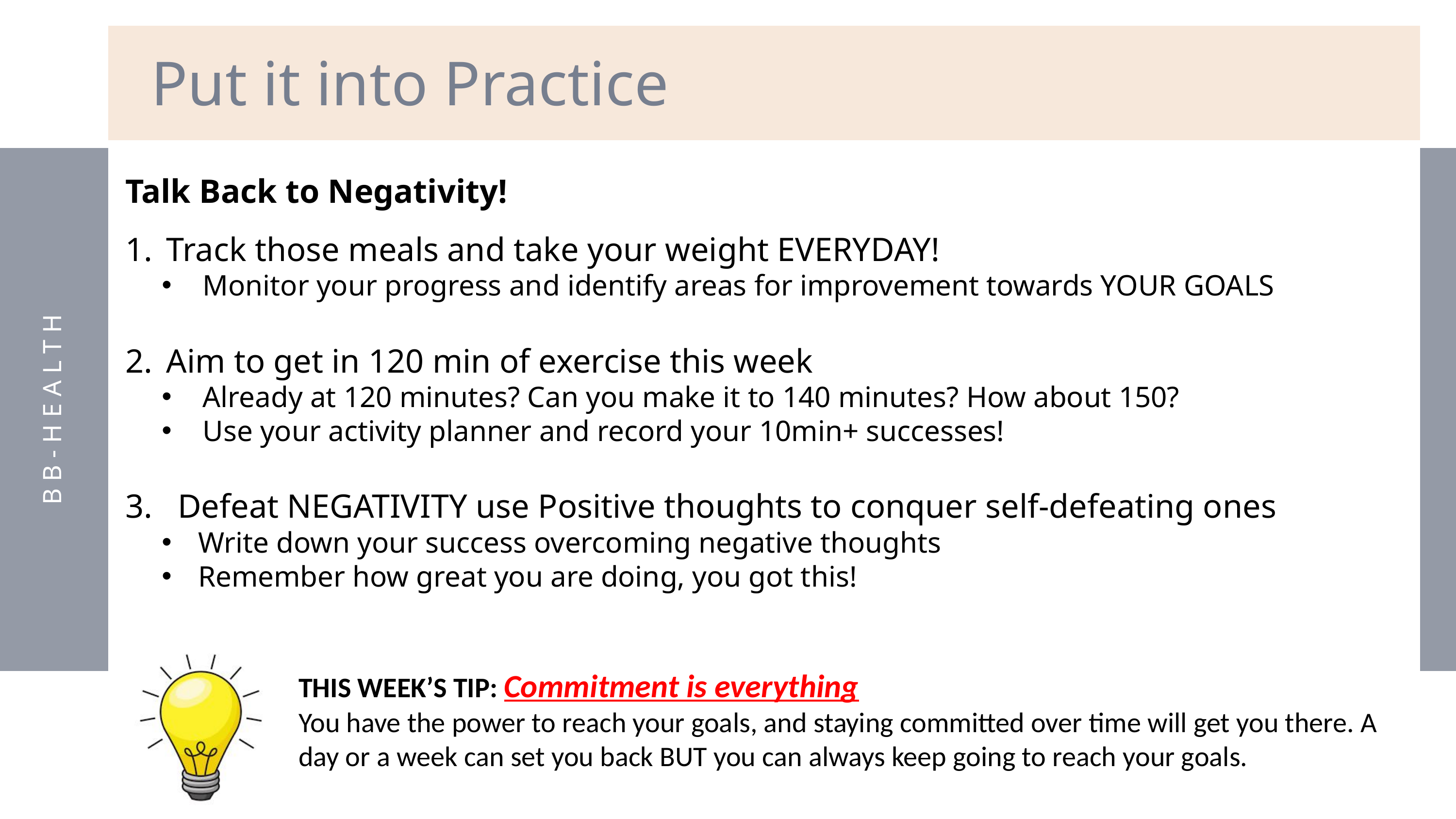

Put it into Practice
Talk Back to Negativity!
Track those meals and take your weight EVERYDAY!
Monitor your progress and identify areas for improvement towards YOUR GOALS
Aim to get in 120 min of exercise this week
Already at 120 minutes? Can you make it to 140 minutes? How about 150?
Use your activity planner and record your 10min+ successes!
3. Defeat NEGATIVITY use Positive thoughts to conquer self-defeating ones
Write down your success overcoming negative thoughts
Remember how great you are doing, you got this!
BB-HEALTH
THIS WEEK’S TIP: Commitment is everything
You have the power to reach your goals, and staying committed over time will get you there. A day or a week can set you back BUT you can always keep going to reach your goals.

## Slide 17
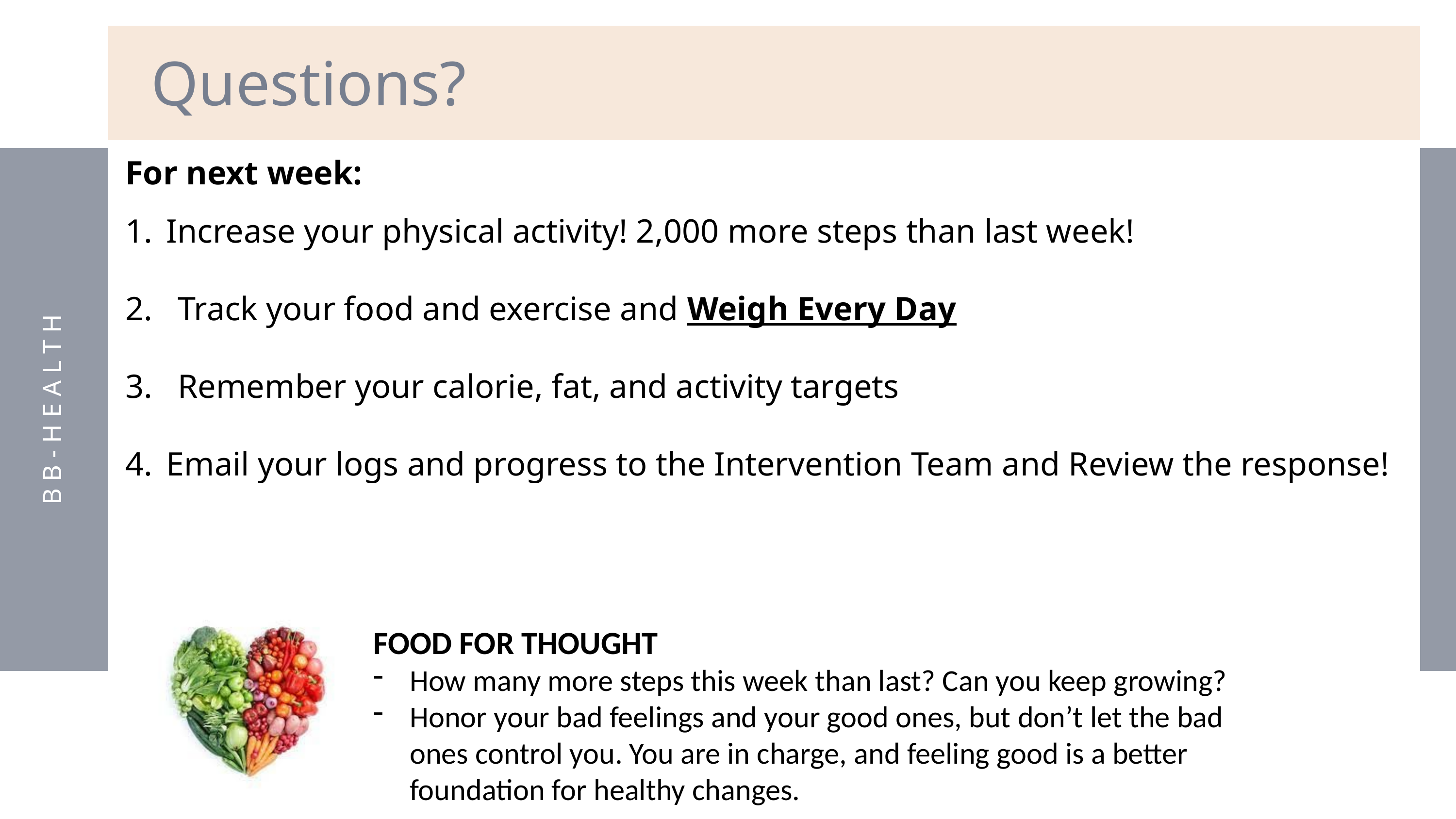

Questions?
For next week:
Increase your physical activity! 2,000 more steps than last week!
2. Track your food and exercise and Weigh Every Day
3. Remember your calorie, fat, and activity targets
Email your logs and progress to the Intervention Team and Review the response!
BB-HEALTH
FOOD FOR THOUGHT
How many more steps this week than last? Can you keep growing?
Honor your bad feelings and your good ones, but don’t let the bad ones control you. You are in charge, and feeling good is a better foundation for healthy changes.
