# Supplementary material for: Development and Feasibility of an eHealth Diabetes Prevention Program Adapted for Older Adults—Results from a Randomized Control Pilot Study
Source: Nutrients. 2024 Mar 23;16(7):930. doi: 10.3390/nu16070930 (PMC11154527; doi:10.3390/nu16070930)
Supplement: Supplementary file 1 [file nutrients-16-00930-s001.zip › Week9_SUPP.pdf]

BB-HEALTH

e-Healthy Older Adults Modification

Diabetes Prevention Program

WEEK #9
Conquer Negativity!

Group Opening Discussion

WELCOME BACK! **<u>HOW WAS LAST WEEK</u>**?

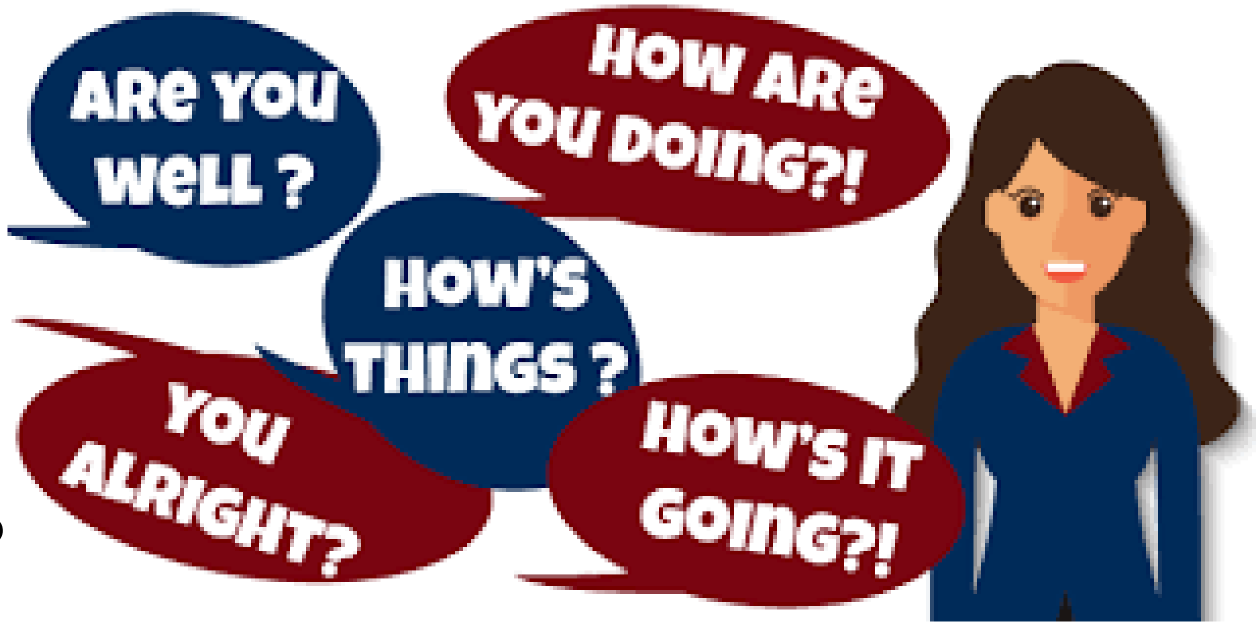

1. Did you track every meal?
2. Did you weigh every day, & record your weight today?
3. Did you explore Fitbit, and calculate your 100 steps and 20 feet?
4. Do you prefer heartbeat, breathing or RPE to measure intensity?
5. Did you email the Intervention Team and review the response?
6. What were your unique challenges and successes?

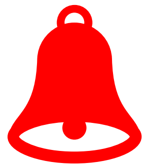

REMINDER: CHARGE YOUR FITBIT & SCALE DURING THIS MEETING!

What supports your success?

BB-HEALTH

<u>What have you done to support your healthy lifestyle? (select all)</u>

- Bought new exercise clothing or equipment
- Bought healthy food at the store
- Logged all my meals
- Logged all my exercise
- Used my Fitbit everyday
- Come to the group sessions every week
- Weighed myself everyday
- Kept my calories at my target most days
- Increased my walking time
- Other (discuss)

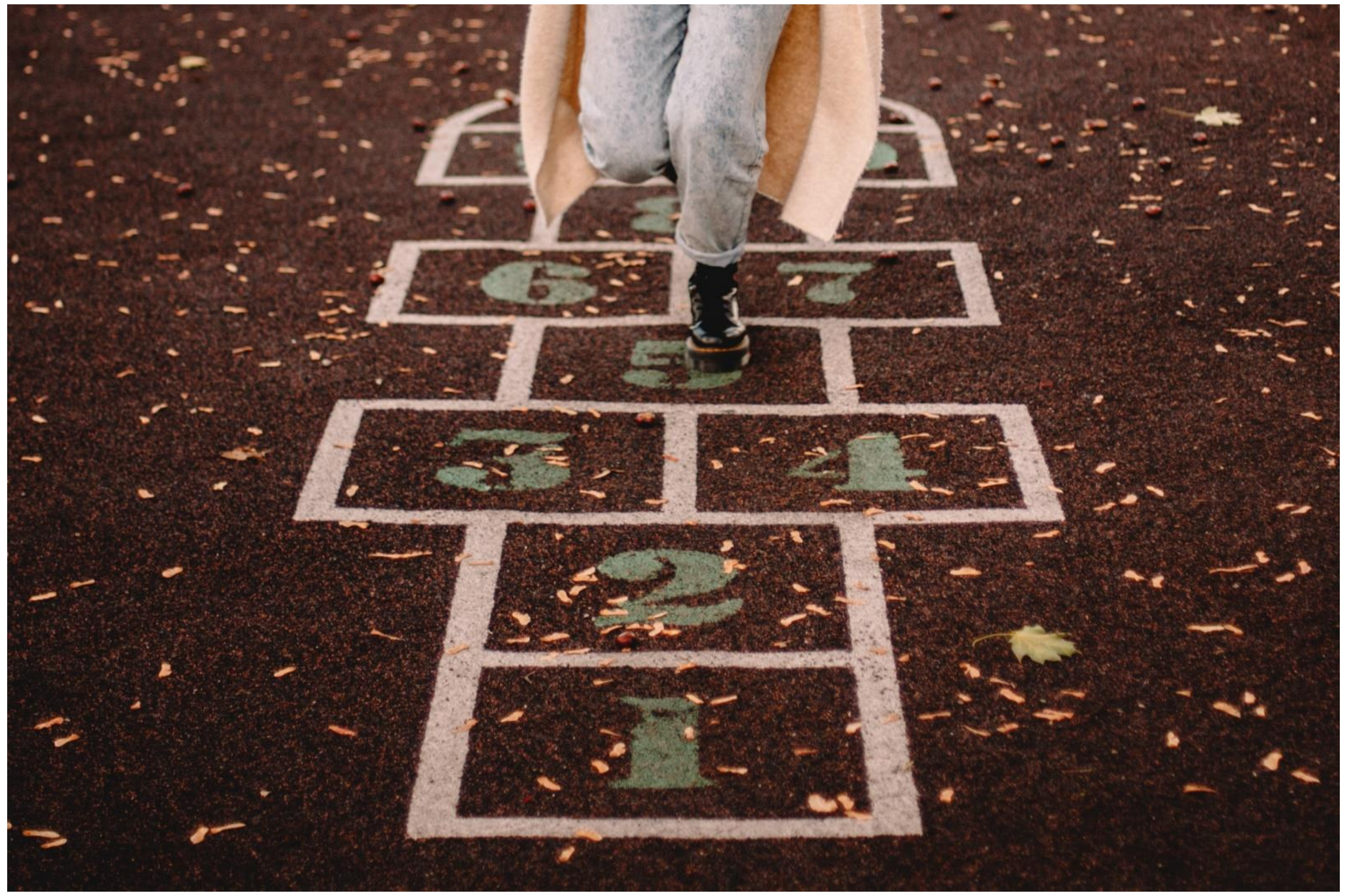

AIM FOR SUCCESS

Everything you do helps move you towards your goal, you should be proud of the work you have done!

How has your lifestyle changed?

BB-HEALTH

Over the last 2 months, how has your lifestyle changed:

I now have a healthier diet (Select one)

- o My diet is the same as before
- o My diet is only a small amount healthier
- o My diet is moderately better
- o My diet is better on most days, or most meals
- o My diet is a lot better
- o My diet is totally healthy now compared to before
- o My diet is a bit worse than before
- o My diet is a lot worse than before

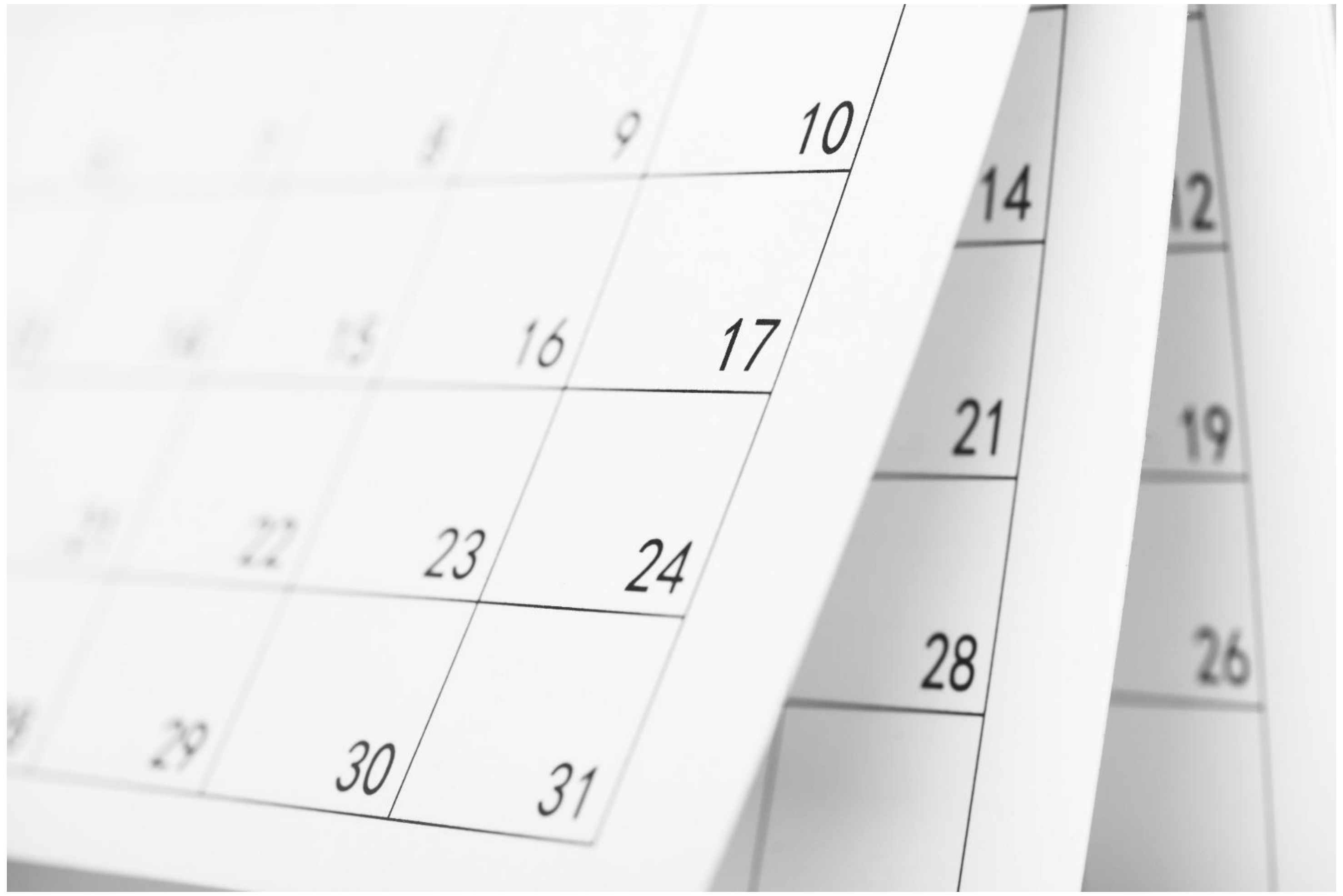

How has your lifestyle changed?

BB-HEALTH

Over the last 2 months, how has your lifestyle changed:

I now have more physical activity(Select one)

- My activity is the same as before
- My activity has only slightly increased
- My activity has moderately increased
- My activity is better on most days
- My activity is a lot more frequent and intensive
- My activity is completely different and better
- My activity is slightly lower than before
- My activity is a lot lower than before

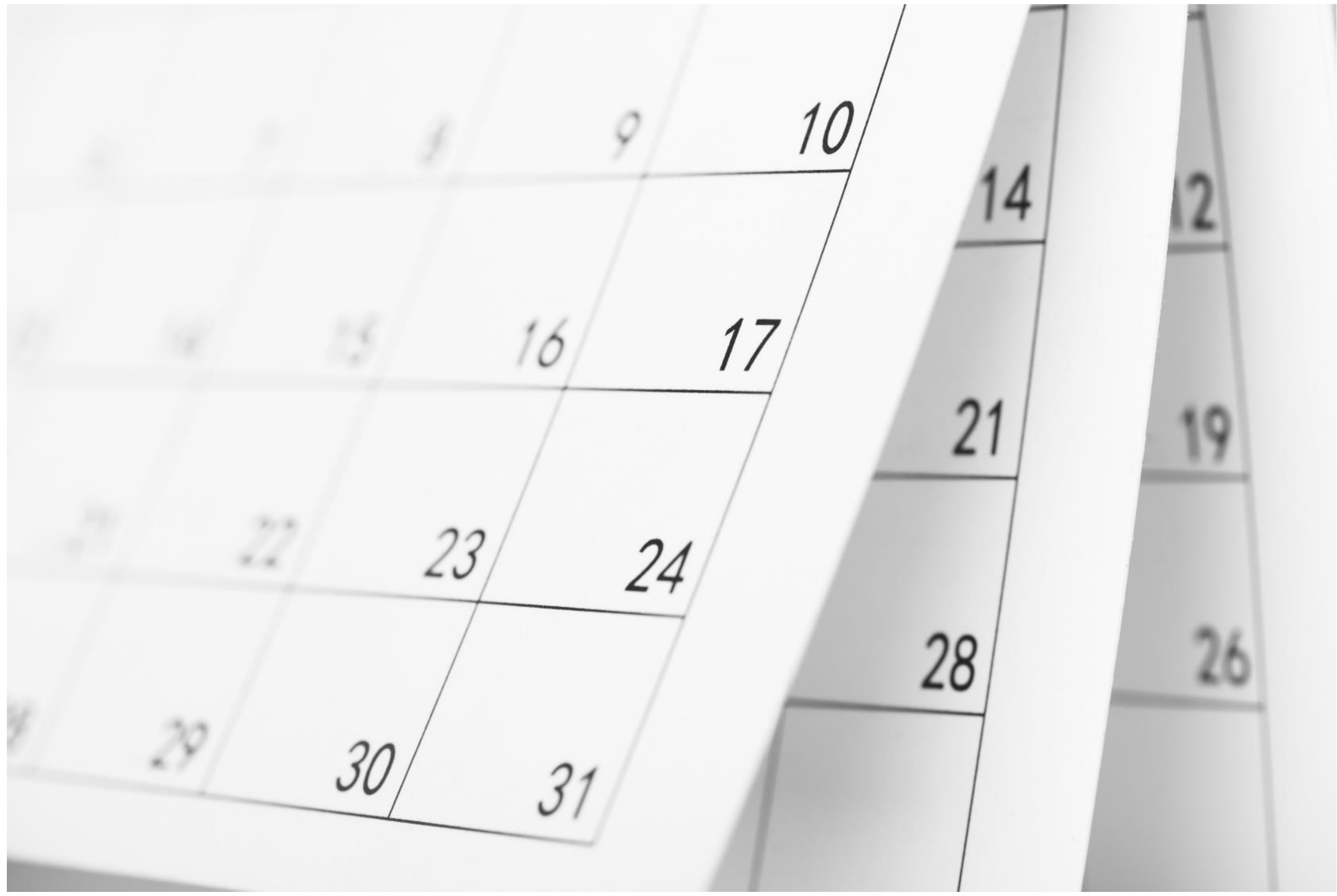

Your target weight goal

BB-HEALTH

We aimed for 7% reduction of body weight- where are we now?

- Daily Weight fluctuates
  - Think of your current weight as within 2.5 pounds + or –
  - What is the BMI?
  - Other measurements: Hip to waist, and Waist to Height

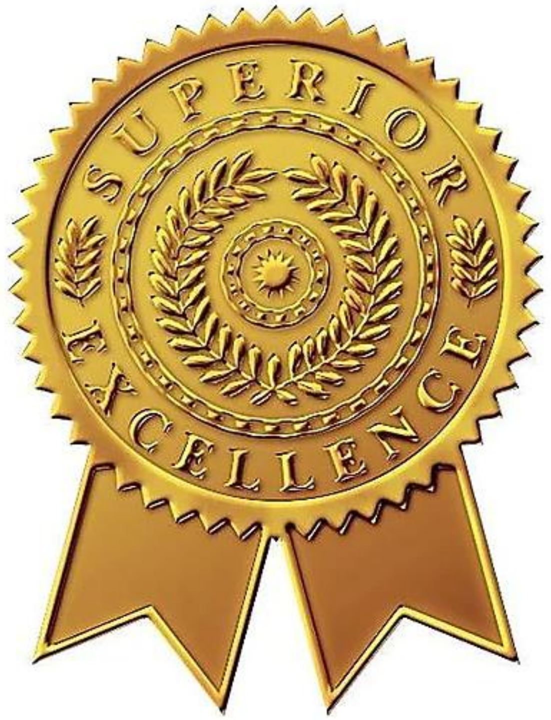

We are aiming for 150 min of physical activity a week- where are we now?

- Don't stop at 150, keep going
  - Aim for ½ of total exercise time in MODERATE
- Set new challenges, longer distances, longer continuous time

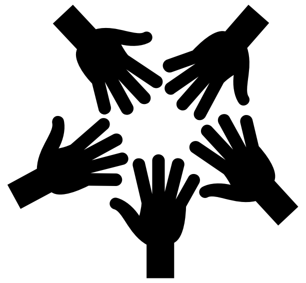

BE PROUD of where you are now!

There's always room for improvement, but you are here which means you are on the road to success! Keep showing up- that's the most important thing. Showing up here is a strong part of staying committed and means you are much more likely to reach your goals.

What do you plan to add next?

BB-HEALTH

<u>In the next week, what will you do to keep moving towards your goals? (select all)</u>

- Weigh everyday
- Take my shake everyday
- Log my calories and fat carefully
- Try a new recipe
- Bring a recipe to the group to share
- Pick a new place to walk
- Schedule a walk with friends
- Other (discuss)

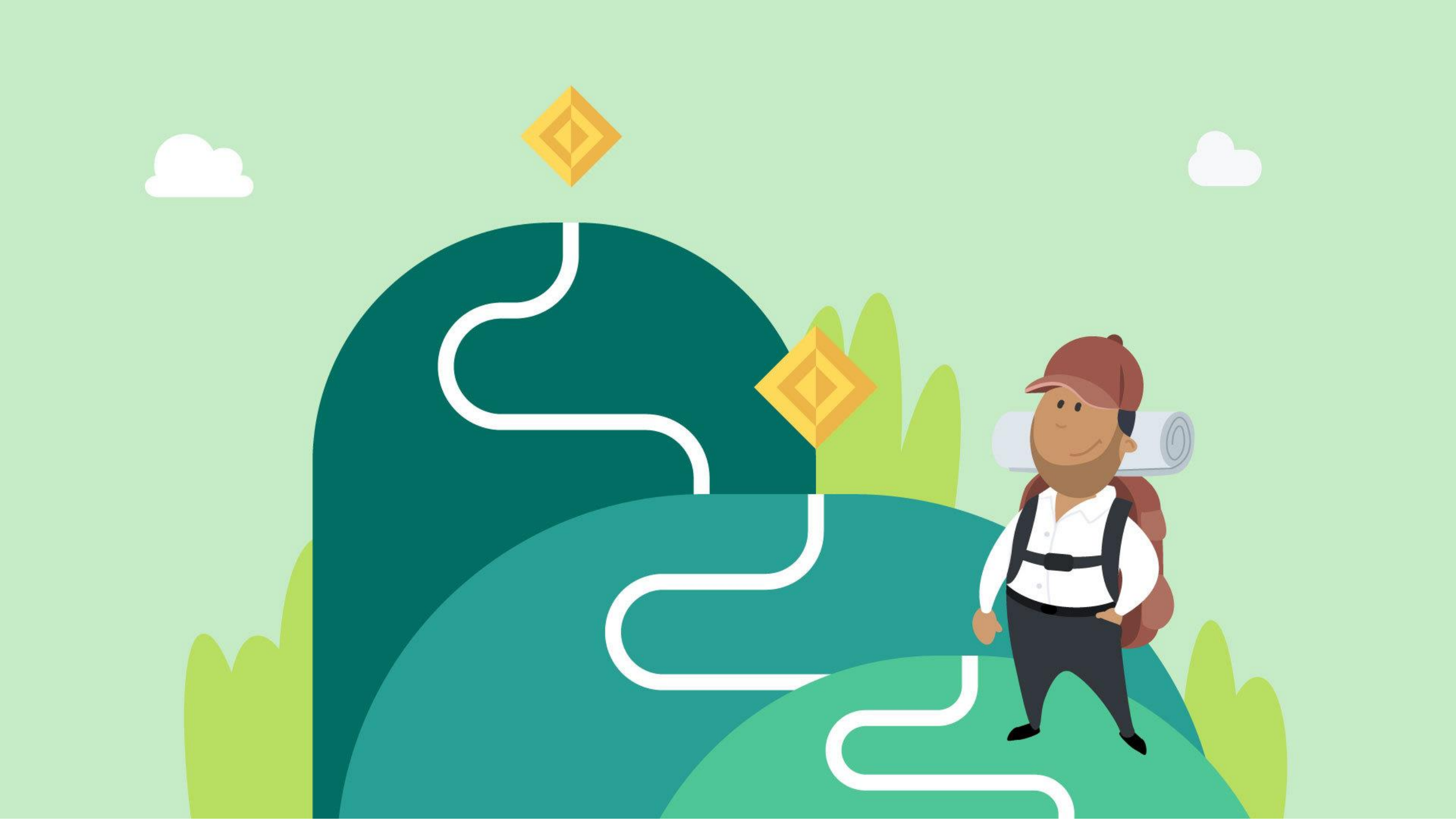

AIM FOR SUCCESS

Each week add at least one more thing, and you will get to the finish line! Look at the Build Better Meals materials starting page 14 in the handout!

Forgiving SLIPS in your healthy plan

- No one is going to be perfect in diet or exercise all the time:

EXPECT TO FALL and EXPECT TO GET BACK UP AND KEEP GOING!

- You can't always prevent or predict when you'll slip, BUT you can control how you react to that slip.
  - Some slips are predictable and preventable though, **let's discuss**.
  - When you slip, how can you recover, **lets discuss**.

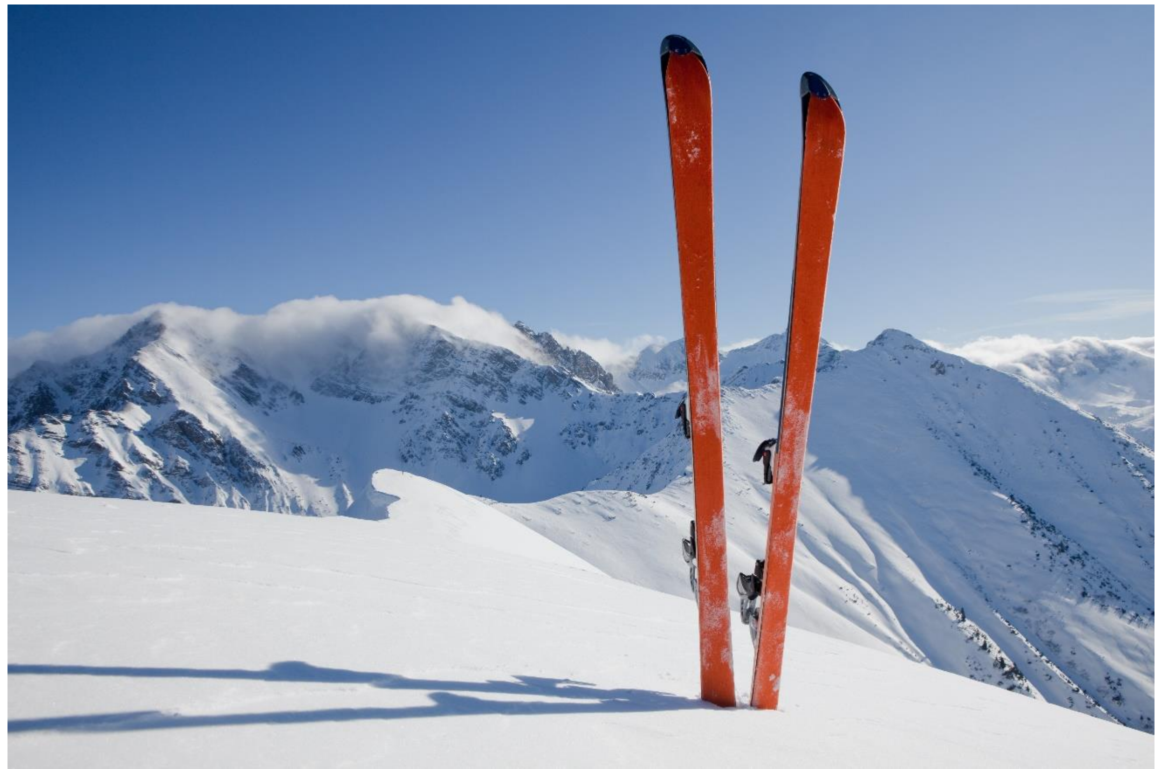

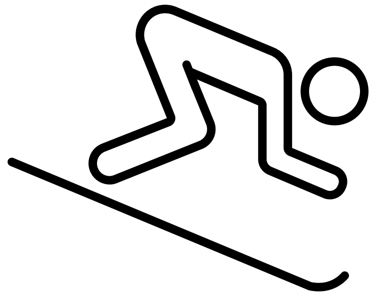

When you ski, you're bound to fall sometimes. We know that going in but it is still fun to keep trying.

When are you most likely to slip?

B B - H E A L T H

Different situations may be more and less difficult for you to stay on track of healthy eating--- choose one:

- When sad
- When bored
- When upset
- When lonely
- When you are under pressure/ stressed
- When there's a change to your routine (travel etc)
- Other (discuss)

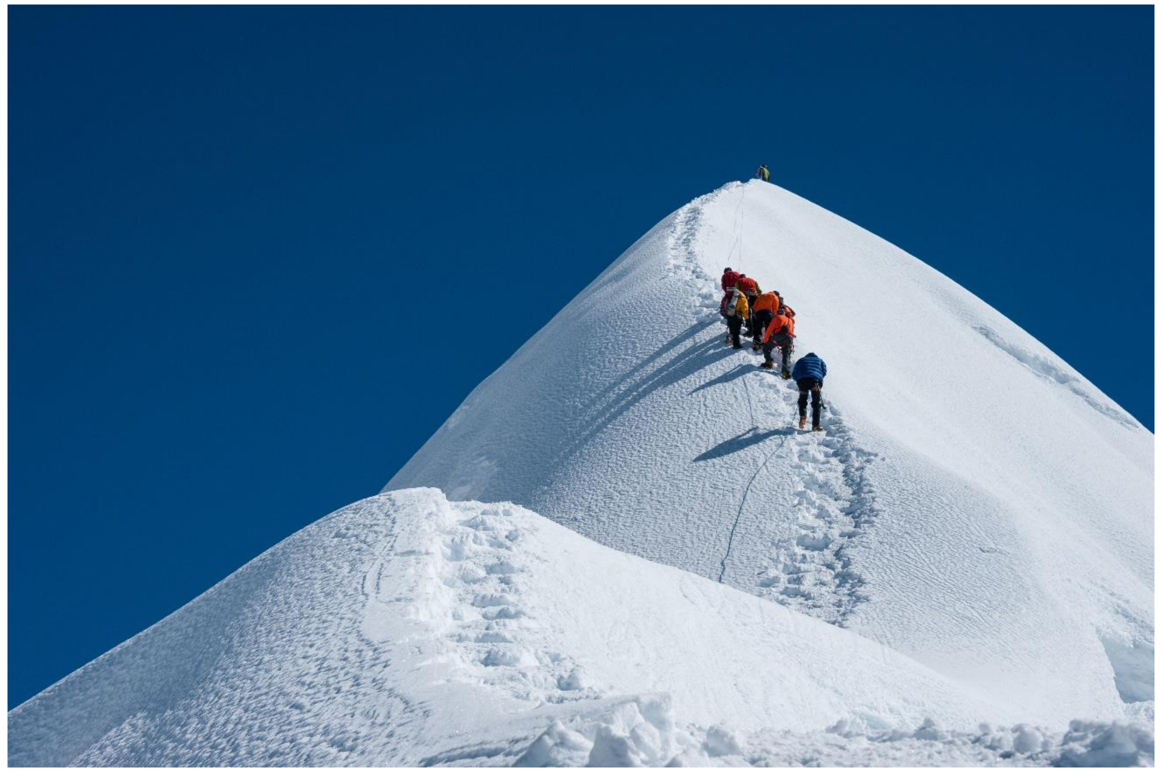

When are you most likely to slip?

Different situations may be more and less difficult for you to stay on track of physical activity-- choose one:

- When at work
- When on vacation
- When visiting with friends and family
- When feeling blue
- When the weather is crummy
- Other (discuss)

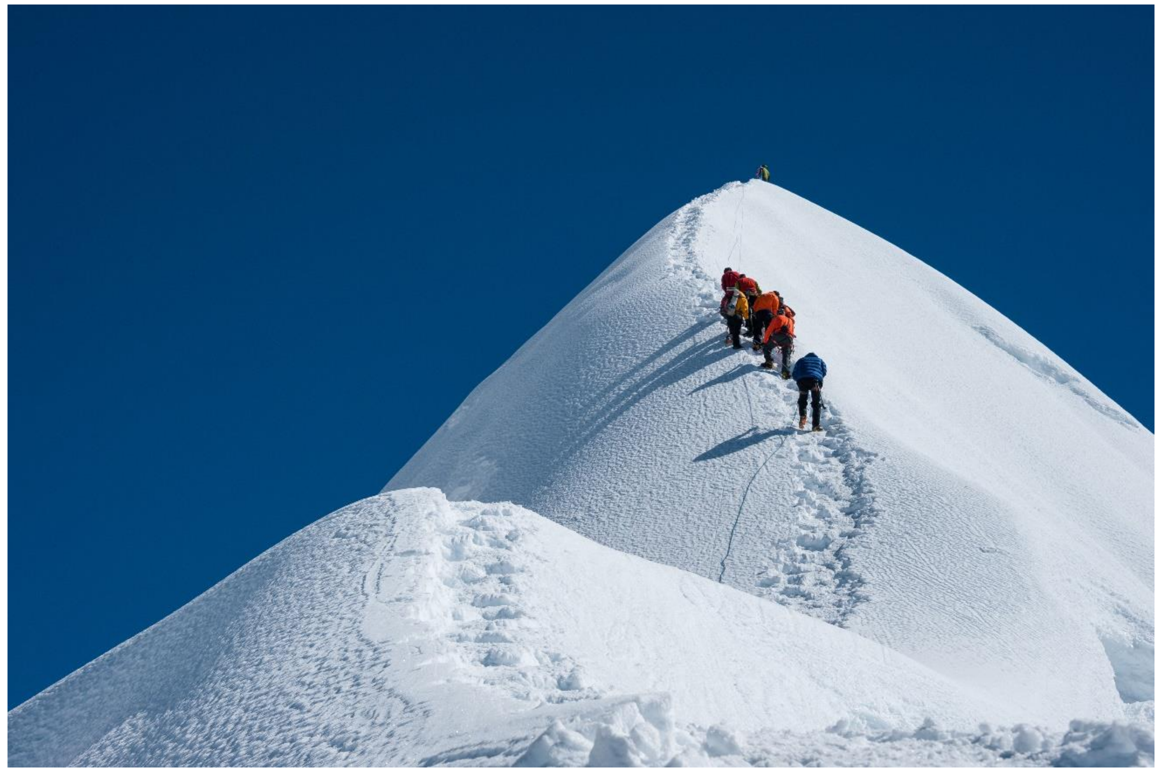

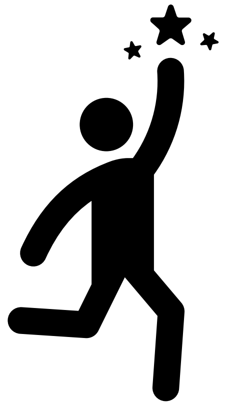

Now you know some of your predictable and preventable obstacles that might lead to a slip. What can you do to plan for success when these obstacles are on your path?

Recovering from a SLIP

- Everybody SLIPS
  - In fact, we learn from our slips!

- A one-time slip won't hurt, don't quit!
  - Take charge after a slip, it won't defeat you.

- Tell those negative thoughts WHO IS BOSS!
  - You are the boss, talk back to negative thoughts, tell them they are wrong and you are right!

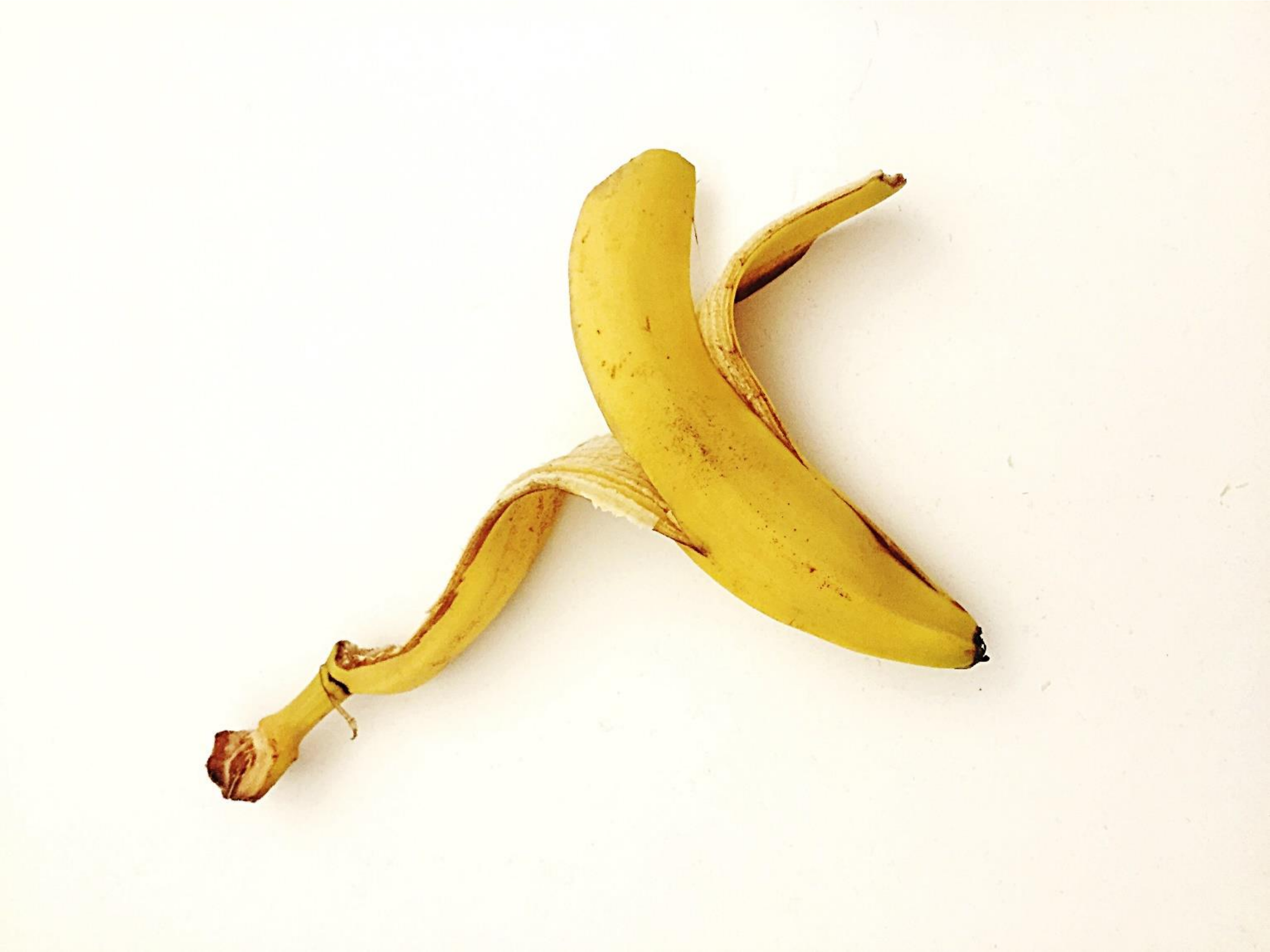

- Examine what happened
  - What can we learn
  - What can we prevent and predict
  - What can we change

- Remember your success so far. It's a process, let's keep going!

Example: Sadie’s Slip

<u>Let’s Look at an example (page 5)</u>

1. What happened in #1? Was this a HIGH-RISK situation?
2. What happened in #2?
3. What happened in #3, and how did Sadie respond?
4. What could Sadie have done differently?
5. How did Sadie get back on track?

Break the chain! Don’t let one slip become 5. Recognize the slip, learn from it, forgive it, and move on. You are on a healthy lifestyle, not a diet. Life happens!

Self-Defeating Thoughts

<u>Everyone has these, but we are in charge of how we respond</u>

Examples:

1) **All or Nothing**
2) **Excuses and Rationalization**
3) **Shoulda Coulda Woulda**
4) **Comparing yourself to others** (or yourself in the past!)
5) **Giving up**

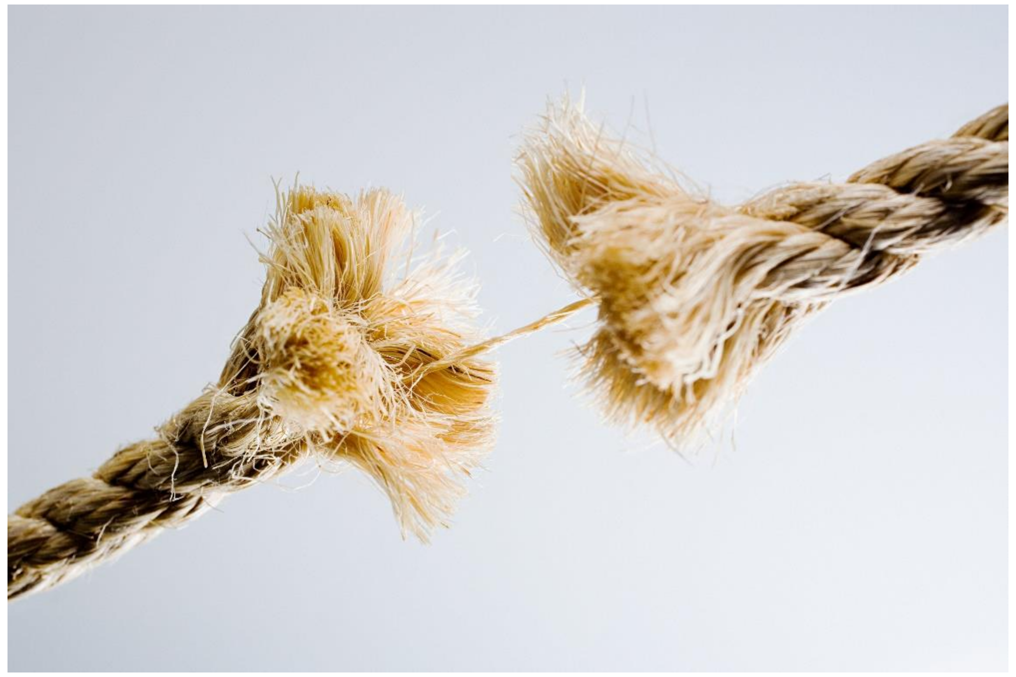

<u>TALK BACK!</u>

- Be AWARE that it is happening.
- Say STOP! Out loud or in your head.
- Out loud or in your head, review your successes and replace negative thoughts with positive ones. Celebrate your successes to defeat the self-defeating thoughts.

TAKE BACK YOUR THOUGHTS! Positivity

Try these positive messages when negativity creeps in:

- ALL OR NOTHING→ "I'm aiming for balance"
  - Don't expect perfection, but don't indulge yourself either. Work toward an overall balance.
- Excuses → "Being healthy is worth my effort"
  - Instead of distractions & blame, why not give yourself a chance? You just might succeed.
- Shouldas → "It's my choice, and I choose it now"
  - You are in charge. No one else is responsible. No one is setting unrealistic expectations.
- Comparing → "Everyone is different"
  - You are going to be the best version of YOU. Everyone has their own struggles.
- Giving Up → "One step at a time"
  - Problem-solving is a process. It takes time to make life-long changes.
  - Learn from what doesn't work and if it doesn't' try something else.
  - Learning is always a success.

Hit those negative thoughts with your best shot! Put other talk back ideas in the chat!

THIS WEEK: Keep track of your positive thoughts and times when you STOPPED those negative and self-defeating thoughts.

How does progress feel?

<u>How does it feel to be moving towards your goals? (select one)</u>

- Very Good
- Good
- Sometimes good, sometimes bad
- Bad
- Very Bad

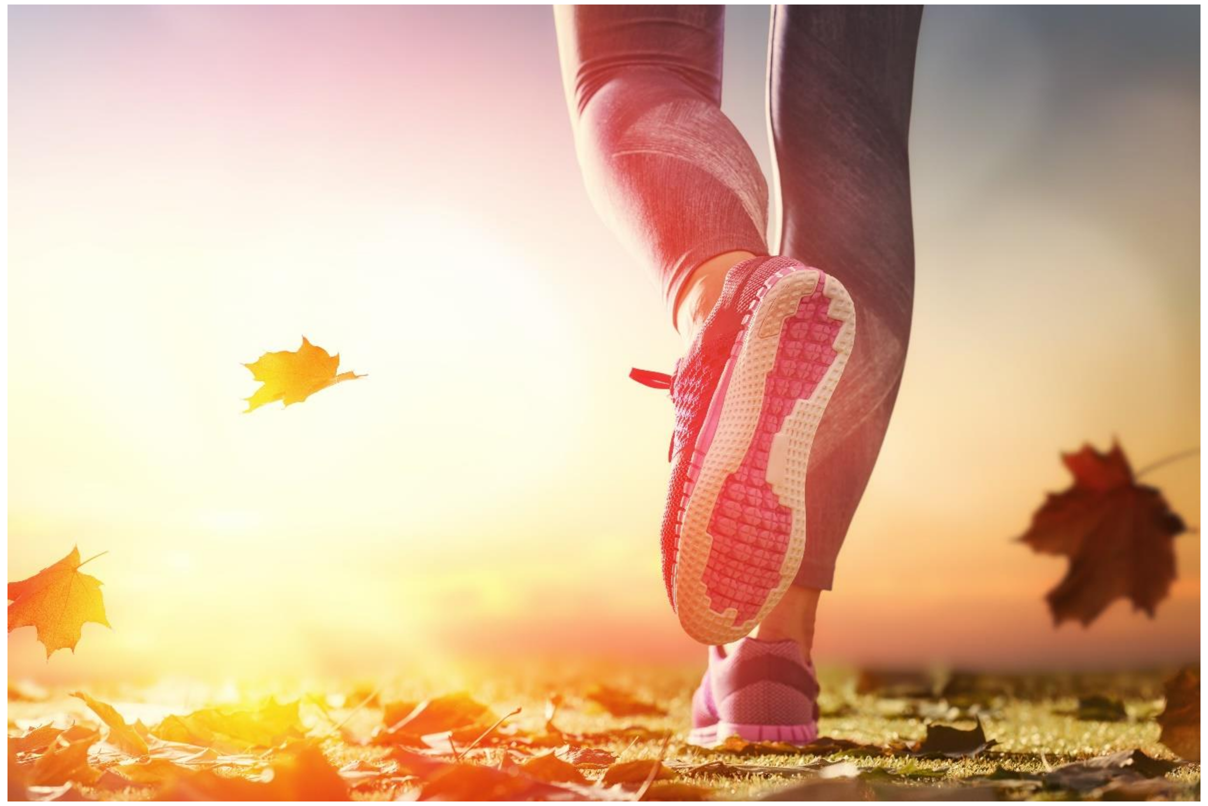

AIM FOR SUCCESS

Feeling good is important. If you are feeling bad about being able to meet your goals, reach out to your Intervention Team and we will make sure to find a way to work together to feel better. Feeling good is often the missing ingredient!

Put it into Practice

Talk Back to Negativity!

1. Track those meals and take your weight EVERYDAY!
   - Monitor your progress and identify areas for improvement towards YOUR GOALS
2. Aim to get in 120 min of exercise this week
   - Already at 120 minutes? Can you make it to 140 minutes? How about 150?
   - Use your activity planner and record your 10min+ successes!
3. Defeat NEGATIVITY use Positive thoughts to conquer self-defeating ones
   - Write down your success overcoming negative thoughts
   - Remember how great you are doing, you got this!

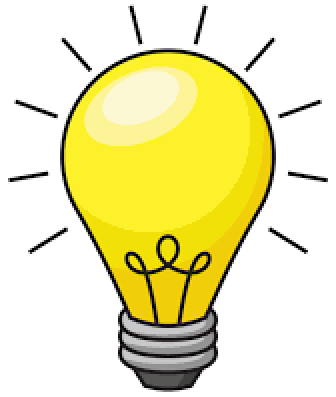

THIS WEEK'S TIP: *Commitment is everything*
You have the power to reach your goals, and staying committed over time will get you there. A day or a week can set you back BUT you can always keep going to reach your goals.

Questions?

BB-HEALTH

For next week:

1. Increase your physical activity! 2,000 more steps than last week!

2. Track your food and exercise and **Weigh Every Day**

3. Remember your calorie, fat, and activity targets

4. Email your logs and progress to the Intervention Team and Review the response!

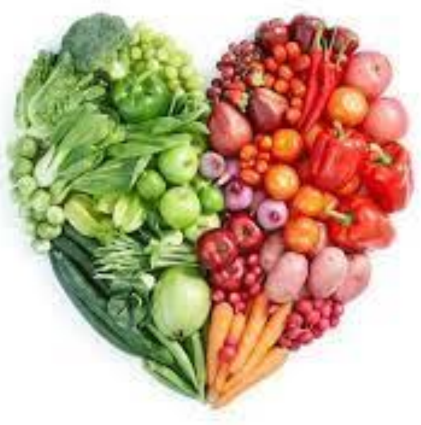

FOOD FOR THOUGHT

- How many more steps this week than last? Can you keep growing?
- Honor your bad feelings and your good ones, but don’t let the bad ones control you. You are in charge, and feeling good is a better foundation for healthy changes.
